# Supplementary material for: Can Single Cell Respiration be Measured by Scanning Electrochemical Microscopy (SECM)?
Source: ACS Meas Sci Au. 2023 Jul 10;3(5):361–70. doi: 10.1021/acsmeasuresciau.3c00019 (PMC10588932; doi:10.1021/acsmeasuresciau.3c00019)
Supplement: Supplementary file 1 — tg3c00019_si_001.pdf [file tg3c00019_si_001.pdf]

Can Single Cell Respiration be Measured by Scanning Electrochemical Microscopy (SECM)?

Kelsey Cremin[1-4†], Gabriel N. Meloni[1,2†], Dimitrios Valavanis[2], Orkun S. Soyer[1, 4], Patrick R. Unwin[1,2*]*

[1]Bio-Electrical Engineering Innovation Hub, [2]Department of Chemistry, [3]Molecular Analytical Science Centre for Doctoral Training (MAS CDT), [4]School of Life Sciences, at the University of Warwick, Coventry CV4 7AL, United Kingdom

[†]These authors contributed equally

*Corresponding author(s):

p.r.unwin@warwick.ac.uk

o.soyer@warwick.ac.uk

Contents

SI-1 HeLa cell culture and media preparation S.3

SI-2 SECM instrumentation S.3

SI-3 Cell staining and imaging S.5

SI-4 Finite Element Method (FEM) simulations.. S.6

SI-5 FEM with varied cell size……...……………………………………………………… S.8

SI-1 HeLa cell culture and media preparation

HeLa cells were obtained from Public Health England, supplied by the European Collection of Authenticated Cell Cultures (ECACC, catalogue number: 93021013). Cells were grown in Minimum Essential Medium Eagle (Sigma Aldrich, M2279) supplemented with L-glutamine (used in 100-fold dilution, Sigma Aldrich, G7513), penicillin and streptomycin (used in 1000-fold dilution, Sigma Aldrich, P4333), heat-inactivated fetal calf serum (10-fold dilution, HIFC, Sigma Aldrich, 12106C), and non-essential amino acids (100-fold dilution, Sigma Aldrich, M7145). Due to the sodium bicarbonate buffer used, cells were incubated at 37 °C in 5% CO_2. Cell flasks were passaged when the confluency reached approximately 80% (no more than a week), and never exceeded a passage number of 10.

Individual sample dishes of cells were prepared on 50 mm WillCo Wells (Glass thickness No. 1.5, USE, HBST-5040) the evening prior to experiments, cultures were diluted to approximately 5×10^6 cell mL^{-1}.

For the purposes of all SECM measurements, the medium was replaced with a Minimum Essential Medium (Sigma Aldrich, 56416C, referred to here as M5) buffered with HEPES (100-fold dilution, Sigma Aldrich, H0887), warmed to 37 °C.

SI-2 SECM instrumentation

Fabrication of SECM probes

Disk shaped platinum UMEs with a radius, *a*, of 5 μm were prepared as described elsewhere.[1] In short, this method involved encapsulating platinum wire (Goodfellow, 99.99 %, 10 μm diameter) in borosilicate glass capillaries (GC200-10, Clark Electromedical Instruments), which was then heated under vacuum and pulled to produce a tapered end using a PB-7 Narishige micropipette puller. The tapered end was then polished to create a smooth flat surface, and the glass sheath is receded through polishing the UME at an angle to create the desired ratio of active electrode surface (Pt) to glass (RG value) of 15.

The UME surface was platinised in a 0.1 M hexachloroplatinate solution (0.1 V/s deposition). Platinisation of the platinum probe creates a layer of amorphous platinum on the surface (often termed platinum black). This amorphous material increases the total surface area of available platinum, without significantly increasing the total radius of the UME. This increased platinum surface in turn allows greater sensitivity to detecting oxygen, without

needing to increasing the overall size of the UME. This was done, as several previous studies have reported increasing sensitivity through platinization for detection (reduction) of oxygen and peroxy species.[2, 3] The amount of platinum deposited in the platinization was limited in order to ensure that the electrode geometry remained as a disk. A platinized probe disk surface is shown in Figure S-1, where the rough-textured platinum accumulates most at the edge between the disk and the glass sheath.

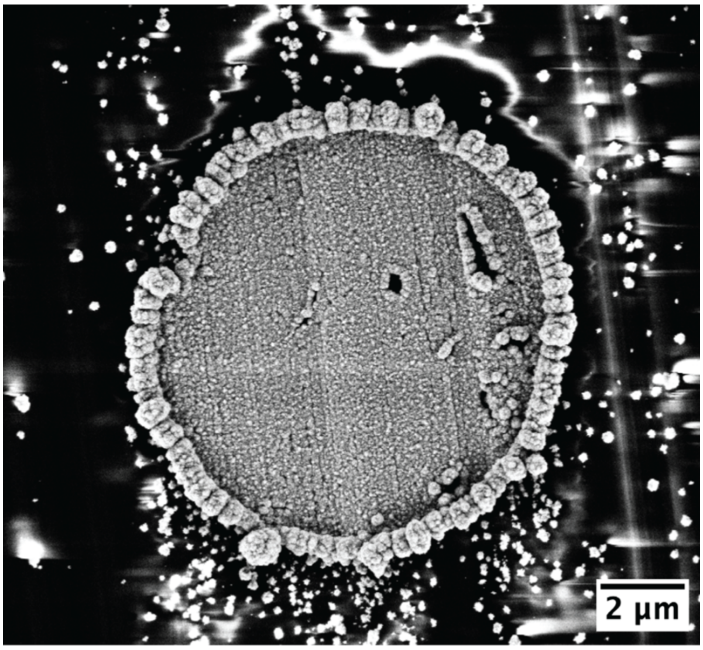

Figure S-1 SEM image of a platinised SECM probe (r = 5 μm), EHT = 6.00 kV

SECM control measurements

Figure S-2 shows the raw and normalized current response when the probe was approached to a glass slide (substrate) with the same feedback threshold as used for the cellular measurements. The normalized current fluctuates around a value of 0.85, demonstrating the extent of hindered diffusion at this tip-substrate distance (d), and demonstrating that it is highly similar to that found for the cell measurements (Figure 3).

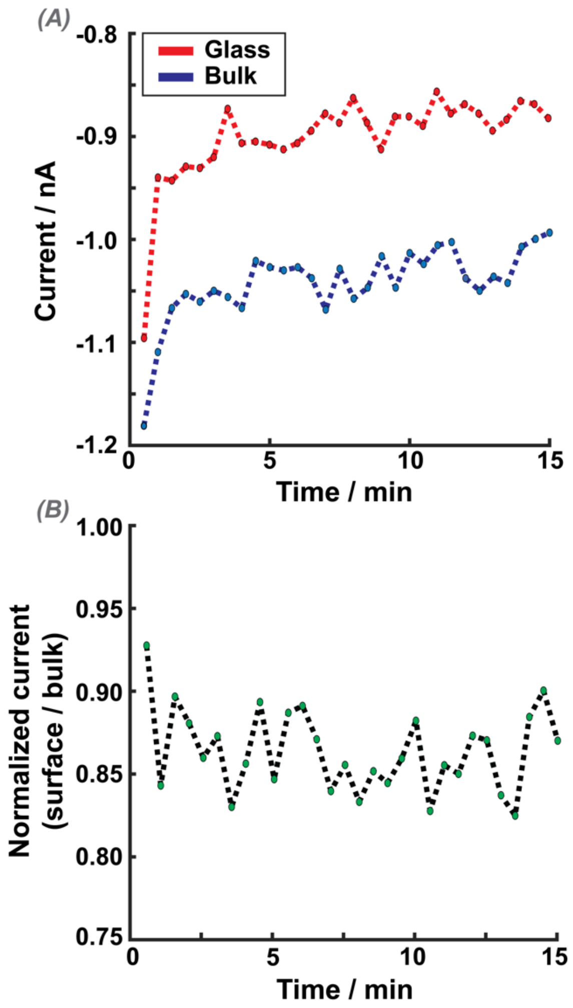

Figure S-2 *Experimental results from a self-referenced SECM experiment to a glass substrate. (A) The current recorded for each OCR measurement for each hop at the bulk (blue), and to a glass surface (red). (B) Surface current normalized to the current measured at the bulk position. 30 hops of each 1 second duration, 1 hop every 30 seconds.*

SI-3 Cell staining and imaging

Cell Staining

Some cells were stained with Tetramethylrhodamine, Methyl Ester (TMRM, Thermo Scientific, M20036), to observe the mitochondrial membrane potential using the confocal microscope. For staining, the media was removed and replaced with PBS, and supplemented with 50 nM TMRM (prepared from a stock solution of 7.5 μM in DMSO). Cells were then incubated with the dye for 30 minutes at 37 °C, before being washed with 3×1 mL applications

of sterile PBS to remove of residual dye. Fresh M5 media was then added to the plate for scanning.

Confocal Laser Scanning Microscopy (CLSM) Imaging Conditions

A Leica TCS SP5 X CLSM was used for imaging HeLa cells stained with TMRM, located in the mitochondrial membrane. Cells were imaged using an Argon laser, set to a wavelength of 514 nm. Emission was detected between 590 and 605 nm. A ×40 oil immersion objective was used, with the confocal pinhole set to 67.97 μm giving a section thickness of approximately 0.967 μm. The 514 nm laser power under these conditions was recorded to be 0.05 mW at the sample plane. Cells were typically imaged at 30 second intervals for the duration of the SECM experiment.

SI-4 Finite Element Method (FEM) Simulations

FEM Simulations

All FEM simulations were performed with COMSOL Multiphysics (v 5.5.) using the Transport of Diluted Species module. A 2D axisymmetric simulation domain was used, as depicted in Figure 2 of the main manuscript. Mesh density (> than 35000 elements) and domain size (radial length and height of the domain > than 100*a*) were set so the simulation results are independent of both. All boundary/domain conditions are summarised in Table S-1, and simulated parameters values in Table S-2.

Table S-1. *Boundary/domain conditions implemented in the FEM simulations, as shown in Figure 2 of the main manuscript. Only fluxes normal to the boundary were considered, noted by "$\boldsymbol{n}$".*

Labelled environment (as seen in Figure 2)	Concentration/flux condition
B1	$\boldsymbol{n}.J_{O_2} = -k_{O2\_UME}\, C_{O_2}$
B2	$\boldsymbol{n}.J_{O_2} = 0$
B3	$\boldsymbol{n}.J_{O_2} = -k_{O2\_cell}\, C_{O_2}$
B4	*N/A*
D1	$C_{O_2} = 227\ \mu M$
D2	$C_{O_2} = 14.5\ \mu M$

Table S-2. *Parameters used for the simulations, relating to the geometry and in other parts of the simulations.*

Geometry parameters		
Symbol	***Value***	***Description***
d	Varied	UME-cell separation. Varied as described in the main text
$Cell_{width}$	9 μm	Cell width, width of B4 from origin
$Cell_{Height}$	2.5 μm	Cell height, height of B4 from origin
$Nucleus_{width}$	4 μm	'Nucleus' width, width of B3 from origin
$Nucleus_{height}$	1.2 μm	'Nucleus' height, height of B3 from origin
a	Varied	Radius of the active electrode surface. Varied as described in the main text
RG	2 or 15	Ratio of a to radius of UME glass sheath, set as 2 for $a < 1$ μm and 15 for $a > 1$ μm
**Other parameters**		
D_{O2_bulk}	2.2×10^{-9} m^2 s^{-1}	Diffusion coefficient of oxygen in bulk (D1)
D_{O2_cyto}	7×10^{-11} m^2 s^{-1}	Diffusion coefficient of oxygen in cell cytosol (D2)
C_{O2_bulk}	227 μM	Concentration of oxygen in the bulk (D1)
C_{O2_cyto}	14.5 μM	Concentration of oxygen in the cell cytosol (D2)
k_{O2_UME}	10 m s^{-1}	Rate constant for oxygen reduction at the UME
k_{O2_cell}	1.51×10^{-9} to 1.54×10^{-4} m s^{-1}	Rate constant for oxygen consumption at the cell nucleus

Simulations consider only diffusion of oxygen (no convection), and were performed as time-dependent studies, with the duration set the same as the experiments (typically 1 second). Oxygen transport in D1 and D2 was described by Fick's second law of diffusion (Equation S1) applied to the axisymmetric geometry of Figure 2 (main text).

$$\frac{\partial c_{O_2}}{\partial t} = D_{O_2}\nabla^2 C_{O_2} \qquad \text{(S1)}$$

where D_{O_2} is the diffusion coefficient of oxygen and C_{O_2} is the oxygen concentration.

Oxygen flux at the UME surface (B1, Table S-1) was close to diffusion-controlled owing to the large cathodic potential applied in the experiments and the oxygen concentration at the B1 is effectively 0. Oxygen flux (mol s^{-1}) at the cell nucleus, simulating cell respiration, was given by a rate equation (B3, Table S-2), with the oxygen consumption rate constant (interfacial 1st order kinetics), k_{O2_cell}, varying from 1.51×10^{-9} to 1.54×10^{-4} m s^{-1}. These rate constant values resulted in OCR values, calculated by integrating the oxygen molecular flux across the length of the cell wall, boundary B4 (Figure 2, main manuscript), ranging from 1.01×10^{-19} to 9.99×10^{-15} mol s^{-1}. These values cover, and exceed, the range of values reported in the literature for single HeLa cell respiration rates.[4]

Simulated ORR currents were calculated by integrating the molecular oxygen flux over the UME disc (B1) and multiplying it by Faraday's constant, F (96485.3 C mol^{-1}), and the

number of electrons, n, involved in the ORR reaction at platinum, reasonably assumed to be 4.[5]

Platinization can change the geometry of the platinum disk electrode, where these changes would need to be accounted for in FEM models to most accurately represent the UME in the system. Whilst we tested increase the geometry of the platinum surface, converting B1 from a flat plane to a hemisphere coming out of the UME surface, the sensitivity increase did not make it possible to measure oxygen at the single-cell level.

Full COMSOL simulation details can be found in the attached report, entitled 'Cremin_etal_2023_SECM_Oxygen_COMSOL_Report'.

SI-5 FEM with varied cell size

The size of the HeLa cell used in the simulations in the manuscript are based on literature values[6] and from optical microscopy measurements acquired with the SECM-CLSM set up. However, cells can vary in size which may impact the detectable OCR. To explore this, a larger (20 μm cell radius, 5 μm cell height) and a smaller cell (5 μm cell radius, 2 μm cell height) were simulated, varying the OCR values across a similarly (and generous) physiological range. This can be found in Figure S-3, where the UME-cell separation is remained at 10 μm. The figure demonstrates that whilst there is an increase in sensitivity to the cell respiration with increasing cell size, even if the cell is increased to a radius of 20 μm, the normalized currents are still too low for accurate detection by the UME within the physiological OCR region. Even at 1×10^{-16} mol s^{-1} the normalized current is above 0.995. Therefore, at the single cell level, it is not considered that increasingly the cell size will allow achievable detection with the UME.

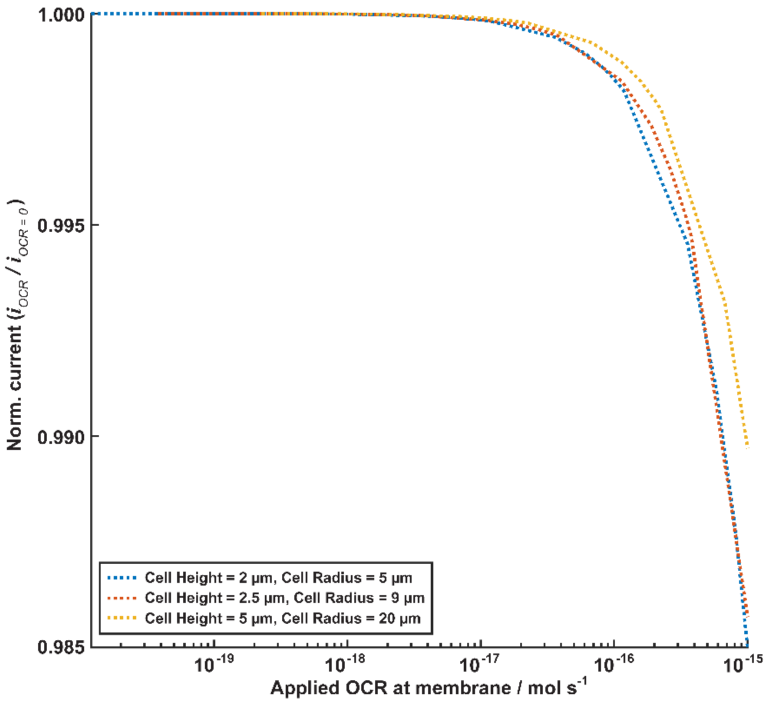

Figure S-3 *FEM simulation of the effect of cell size on the current measurements across a range of OCR values. The normalized currents are simulated for a UME performing ORR at a transport-limited rate and a cell respiring at different OCR values. Current are normalized by the UME current at the same UME-cell distance (10 µm), with a OCR set to 0 mol s^{-1}.*

References

(1) Bard, A. J.; Fan, F. R. F.; Kwak, J.; Lev, O. Scanning electrochemical microscopy. Introduction and principles. *Anal. Chem.* **1989**, *61* (2), 132-138

(2) Li, Y.; Hu, K.; Yu, Y.; Rotenberg, S. A.; Amatore, C.; Mirkin, M. V. Direct Electrochemical Measurements of Reactive Oxygen and Nitrogen Species in Nontransformed and Metastatic Human Breast Cells. *J. Am. Chem. Soc.* **2017**, *139* (37), 13055-13062

(3) Santos, C. S.; Kowaltowski, A. J.; Bertotti, M. Single Cell Oxygen Mapping (SCOM) by Scanning Electrochemical Microscopy Uncovers Heterogeneous Intracellular Oxygen Consumption. *Sci. Rep.* **2017**, *7* (1), 11428

(4) Wagner, B. A.; Venkataraman, S.; Buettner, G. R. The rate of oxygen utilization by cells. *Free. Radic. Biol. Med.* **2011**, *51* (3), 700-712

(5) Haider, R.; Yuan, X.; Bilal, M. Oxygen Reduction Reaction. In *Methods for Electrocatalysis: Advanced Materials*, Inamuddin, Boddula, R., Asiri, A. M. Eds.; Springer Nature, 2020

(6) Zhao, L.; Sukstanskii, A. L.; Kroenke, C. D.; Song, J.; Piwnica-Worms, D.; Ackerman, J. J.; Neil, J. J. Intracellular water specific MR of microbead-adherent cells: HeLa cell intracellular water diffusion. *Magn Reson Med* **2008**, *59* (1), 79-84
