# Supplementary material for: Can Single Cell Respiration be Measured by Scanning Electrochemical Microscopy (SECM)?
Source: ACS Meas Sci Au. 2023 Jul 10;3(5):361–70. doi: 10.1021/acsmeasuresciau.3c00019 (PMC10588932; doi:10.1021/acsmeasuresciau.3c00019)
Supplement: Supplementary file 2 — tg3c00019_si_002.pdf [file tg3c00019_si_002.pdf]

Cremin et al. (2023) SECM Oxygen COMSOL Reports

Report date	May 28, 2023, 6:28:10 PM

Contents

1. Global Definitions 3
1.1. Parameters 3
1.2. Functions 5
1.3. Shared Properties 8
2. Component 2 9
2.1. Definitions 9
2.2. Geometry 17
2.3. Transport of Diluted Species 2 19
2.4. Mesh 2 38
3. Oxygen Pulse 45
3.1. Parametric Sweep 45
3.2. Stationary 45
3.3. Oxygen Probe Pulse 45
3.4. Solver Configurations 46
4. Results 475
4.1. Data Sets 475
4.2. Derived Values 485
4.3. Tables 489
4.4. Plot Groups 1602

1 Global Definitions

Date	Aug 20, 2021, 4:37:57 PM

GLOBAL SETTINGS

Name	SECM Oxygen COMSOL Reports.mph
Path	D:\Kelsey_Sim4\18062020_SECM_OxygenRespiration\20210128_SECM_PaperModel\SECM_Oxygen_COMSOL_Reports.mph
Version	COMSOL Multiphysics 6.0 (Build: 405)
Unit system	SI

USED PRODUCTS

COMSOL Multiphysics

COMPUTER INFORMATION

CPU	Intel64 Family 6 Model 62 Stepping 4, 4 cores
Operating system	Windows 8.1

1.1 PARAMETERS

1.1.1 Geometry Parameters

GEOMETRY PARAMETERS

Name	Expression	Value	Description
TipRg	10	10	
RG	TipRg*Rb	1E−5 m	tip Rg
RespSurface	pi*(NucleusWidth^2)*(1 + (1 - e2)/e*(atanh(e)))[m^2]	5.9152E−11	
Rb	1[um]	1E−6 m	WElectrode radius
Ra	10 [um]	1E−5 m	Substrate radius
NucleusWidth	4[um]	4E−6 m	
NucleusHeight	1.2[um]	1.2E−6 m	
e2	1 - ((NucleusHeight^2)/(NucleusWidth^2))[m^2]	0.91 m²	
e	sqrt(e2)[m]	0.95394 m²	
Domain	2000*Rb	0.002 m	Domain
d2	d + CellHeight	3.9462E−5 m	
d	36.962[um]	3.6962E−5 m	tip-substrate separation
CellWidth	9[um]	9E−6 m	
CellHeight	2.5[um]	2.5E−6 m	
Beta	7[deg]	0.12217 rad	semi angle tip

1.1.2 ET reaction Parameters

ET REACTION PARAMETERS

Name	Expression	Value	Description
n	4	4	No of electrons
Rconst	R_const	8.3145 J/(mol·K)	Gas Constant
F	F_const	96485 C/mol	Faraday's Constant
T	298 [K]	298 K	Absolute Temp
alpha	1	1	Transfer Coefficent
k0	10 [m/s]	10 m/s	Kinetics
k0Sub	10 [m/s]	10 m/s	Substrate Kinetics
D	2.2e-5 [cm^2/s]	2.2E−9 m²/s	Diffusion coefficient for Oxygen
E0	-0.25 [V]	−0.25 V	Formal Potential for 02 to H20, Sawyer 1960's paper for pH 7
COx	227 [umol/L]	0.227 mol/m³	Dissolved oxygen conc. in serum at standard pressure, at 25 oC (184 uM at 30oC)
DCytosol	70[um^2/s]	7E−11 m²/s	Diffusion coefficient for the cytosol
COxCytosol	14.6[umol/L]	0.0146 mol/m³	Dissolved oxygen conc. in cytosol
RespRate	10[m/s]	10 m/s	Rate of respiration from within cell
k0Pause	0 [m/s]	0 m/s	

1.1.3 CV Parameters

CV PARAMETERS

Name	Expression	Value	Description
Es	-0.2	−0.2	Starting Potential
Ef	-0.8	−0.8	Final Potential
scanrate	0.02	0.02	scanrate
t_total	abs((Ef - Es)/scanrate)	30	Total Time
timestep	t_total/50	0.6	Timestep

1.1.4 PAC Parameters

PAC PARAMETERS

Name	Expression	Value	Description
Epac	-0.5 [V]	−0.5 V	Tip potential during PAC
dStart	30	30	initial height for approach
dStop	0.05	0.05	final height for approach
dSteps	150	150	steps in a PAC

1.1.5 Oxygen Pulse Parameters

OXYGEN PULSE PARAMETERS

Name	Expression	Value	Description

Name	Expression	Value	Description
Epulse	-0.65[V]	−0.65 V	Tip potential during pulse
Pause_period1	Pause_step1*50	0.25 s	
Pause_step1	Pause_t/200	0.005 s	
Pause_step2	Pause_t/50	0.02 s	
Pause_t	1[s]	1 s	
Pulse_period1	Pulse_step1*50	0.25 s	
Pulse_step1	Pulse_t/200	0.005 s	
Pulse_step2	Pulse_t/50	0.02 s	
Pulse_t	1[s]	1 s	Pulse time
Tots_period1	Tots_step1*50	0.5 s	
Tots_step1	Tots_t/200	0.01 s	
Tots_step2	Tots_t/50	0.04 s	
Tots_t	Pause_t + Pulse_t	2 s	

1.1.6 Set Current

SET CURRENT

Name	Expression	Value	Description
Iapp	1[nA]	1E−9 A	
O2flux	(Iapp/(n*F))/(pi*(Rb*Rb))	8.2476E−4 mol/(m²·s)	

1.2 FUNCTIONS

1.2.1 Interpolation 1

Function names	Potential
Function type	Interpolation

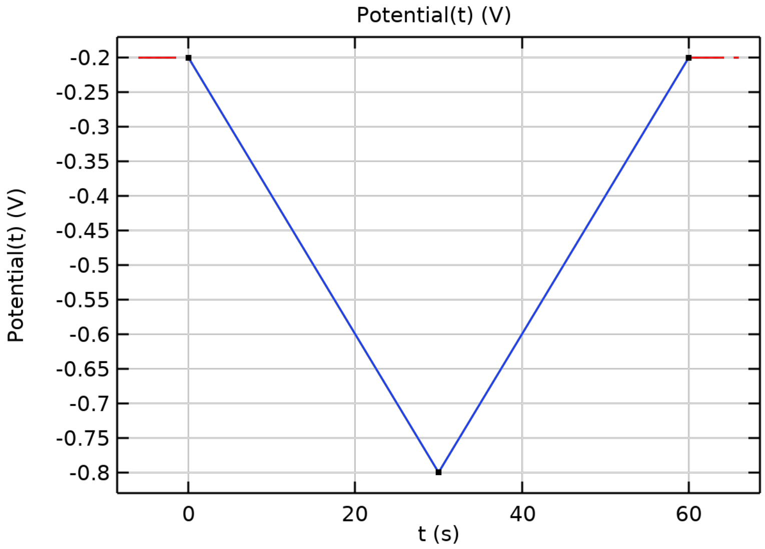

Interpolation 1

UNITS

Function	Unit
Potential	V

UNITS

Argument	Unit
t	s

1.2.2 Piecewise 1

Function name	pw1
Function type	Piecewise

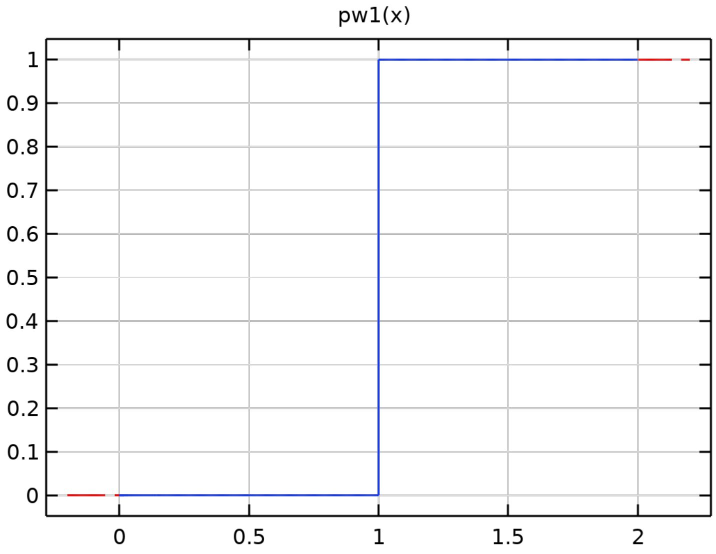

Piecewise 1

DEFINITION

Description	Value
Argument	x
Extrapolation	Constant
Smoothing	No smoothing

DEFINITION

Start	End	Function
0	Pause_t	0
Pause_t	Pause_t+Pulse_t	1

1.2.3 Step 1

Function name	step1
Function type	Step

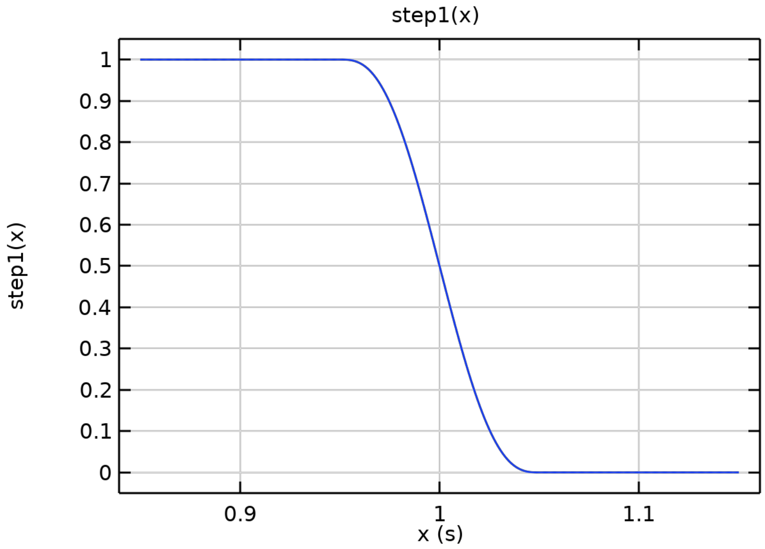

Step 1

PARAMETERS

Description	Value
Location	Pause_t
From	1
To	0

1.3 SHARED PROPERTIES

1.3.1 Default Model Inputs

Tag	cminpt

2 Component 2

Date	Apr 8, 2020, 11:50:48 AM

SETTINGS

Description	Value
Unit system	Same as global system (SI)
Geometry shape function	Automatic
Avoid inverted elements by curving interior domain elements	Off

SPATIAL FRAME COORDINATES

First	Second	Third
r	phi	z

MATERIAL FRAME COORDINATES

First	Second	Third
R	PHI	Z

GEOMETRY FRAME COORDINATES

First	Second	Third
Rg	PHIg	Zg

MESH FRAME COORDINATES

First	Second	Third
Rm	PHIm	Zm

2.1 DEFINITIONS

2.1.1 Variables

Variables 2

SELECTION

Geometric entity level	Entire model

Name	Expression	Unit	Description
kox	k0*exp((1 - alpha)*n*F*(Potential(t) - E0)/(Rconst*T))	m/s	
kred	k0*exp((-alpha)*n*F*(Potential(t) - E0)/(Rconst*T))	m/s	
koxPac	k0*exp((1 - alpha)*n*F*(Epac - E0)/(Rconst*T))	m/s	
kredPac	k0*exp((-alpha)*n*F*(Epac - E0)/(Rconst*T))	m/s	
koxSub	k0Sub*exp((1 - alpha)*n*F*(Epac - E0)/(Rconst*T))	m/s	
kredSub	k0Sub*exp((-alpha)*n*F*(Epac - E0)/(Rconst*T))	m/s	
koxPulse	k0*exp((1 - alpha)*n*F*(Epulse-E0)/(Rconst*T))	m/s	
kredPulse	k0*exp((-alpha)*n*F*(Epulse-E0)/(Rconst*T))	m/s	

2.1.2 Probes

UME

Probe type	Boundary probe

SELECTION

Geometric entity level	Boundary
Selection	Geometry geom2: Dimension 1: Boundary 3

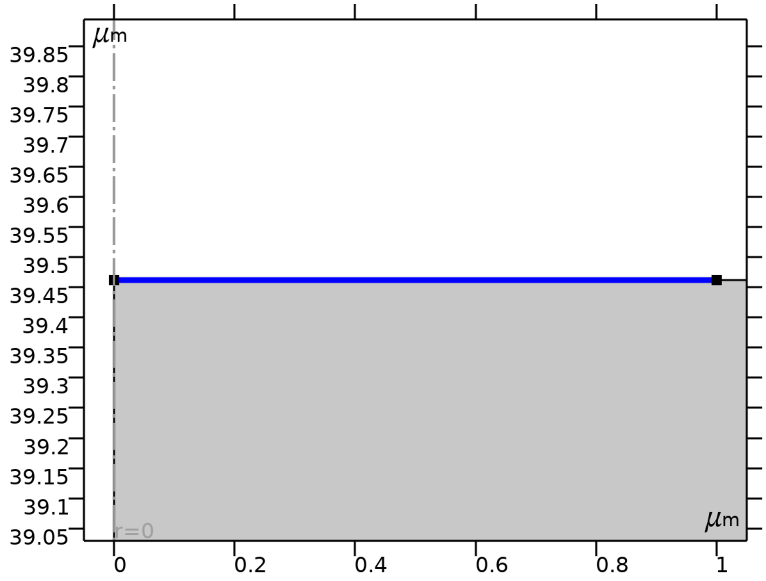

Selection

PROBE TYPE

Description	Value
Type	Integral

EXPRESSION

Description	Value
Expression	-(tds2.ntflux_Oxi)*n*F
Table and plot unit	A
Description	-(tds2.ntflux_Oxi)*n*F

TABLE AND WINDOW SETTINGS

Description	Value
Output table	Probe Table 1
Plot window	Probe Plot 1

OxiMem

Probe type	Boundary probe

SELECTION

Geometric entity level	Boundary
Selection	Geometry geom2: Dimension 1: Boundary 11

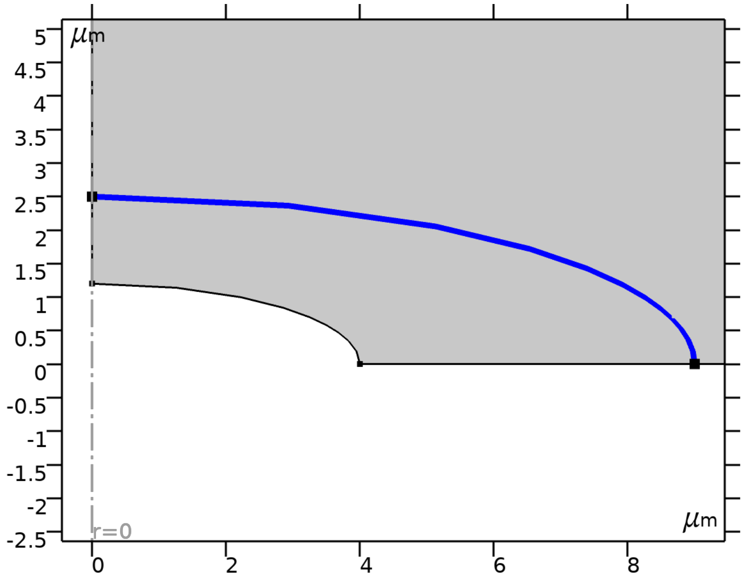

Selection

EXPRESSION

Description	Value
Expression	Oxi
Table and plot unit	M
Description	SurfaceConcOxi

TABLE AND WINDOW SETTINGS

Description	Value
Output table	Probe Table 1
Plot window	Probe Plot 3

OxiNuc

Probe type	Boundary probe

SELECTION

Geometric entity level	Boundary
Selection	Geometry geom2: Dimension 1: Boundary 10

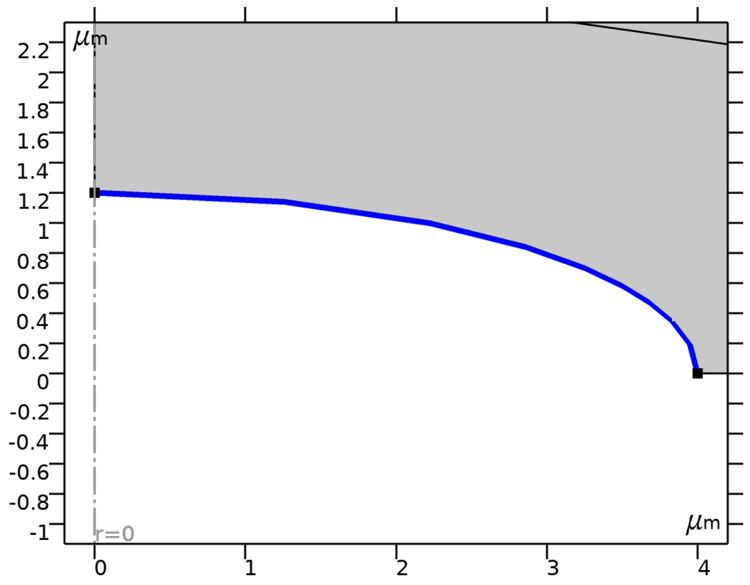

Selection

EXPRESSION

Description	Value
Expression	Oxi
Table and plot unit	M
Description	SurfaceConcOxi

TABLE AND WINDOW SETTINGS

Description	Value
Output table	Probe Table 1
Plot window	Probe Plot 4

MemFlux

Probe type	Boundary probe

SELECTION

Geometric entity level	Boundary
Selection	Geometry geom2: Dimension 1: Boundary 11

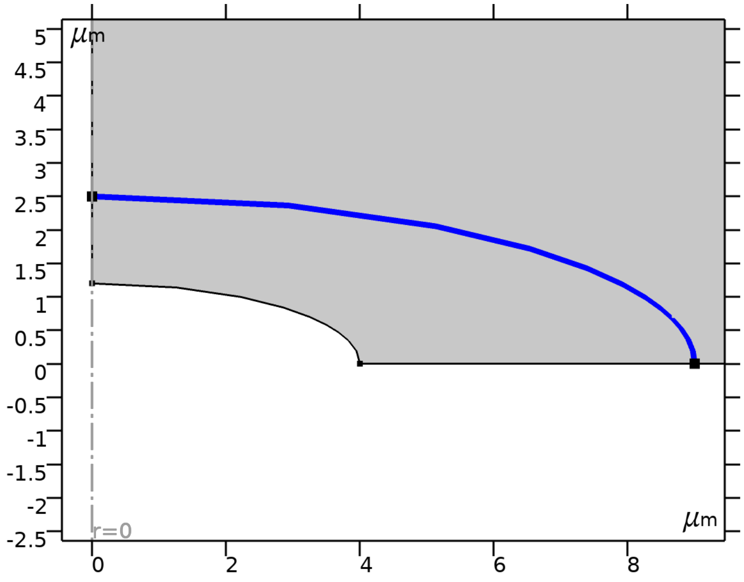

Selection

PROBE TYPE

Description	Value
Type	Integral

EXPRESSION

Description	Value
Expression	k0Sub*Oxi
Table and plot unit	mol/s
Description	k0Sub*Oxi

TABLE AND WINDOW SETTINGS

Description	Value
Output table	Probe Table 1
Plot window	Probe Plot 3

NucFlux

Probe type	Boundary probe

SELECTION

Geometric entity level	Boundary
Selection	Geometry geom2: Dimension 1: Boundary 10

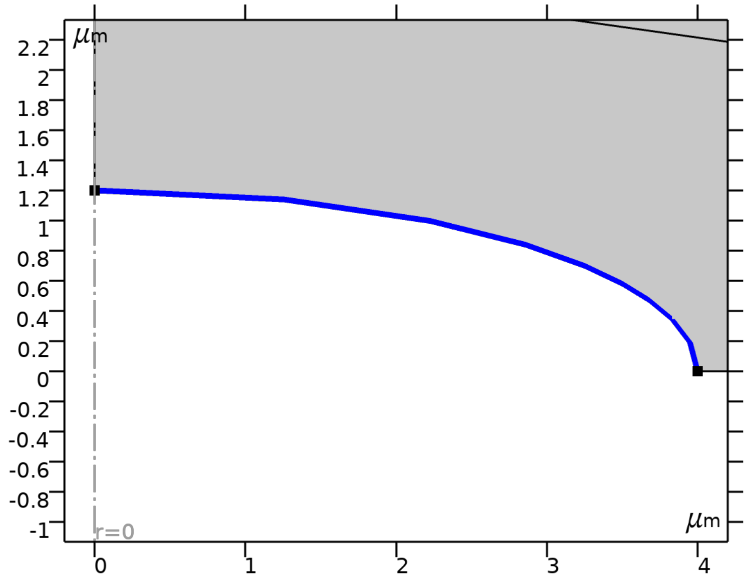

Selection

PROBE TYPE

Description	Value
Type	Integral

EXPRESSION

Description	Value
Expression	k0Sub*Oxi
Table and plot unit	mol/s
Description	k0Sub*Oxi

TABLE AND WINDOW SETTINGS

Description	Value
Output table	Probe Table 1
Plot window	Probe Plot 3

k0Sub probe

Probe type	Global variable probe

EXPRESSION

Description	Value
Expression	k0Sub
Table and plot unit	m/s
Description	Substrate Kinetics

TABLE AND WINDOW SETTINGS

Description	Value
Output table	Probe Table 1

Description	Value
Plot window	Probe Plot 3

UMEOCRmembrane

Probe type	Boundary probe

SELECTION

Geometric entity level	Boundary
Selection	Geometry geom2: Dimension 1: Boundary 11

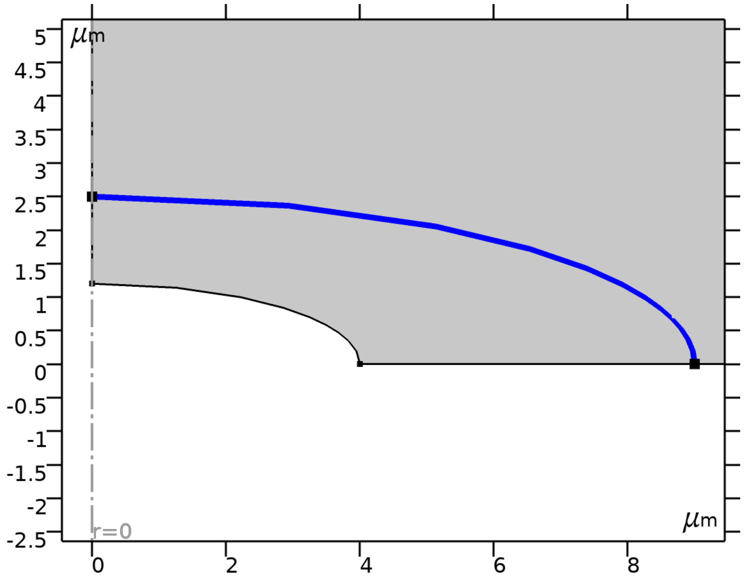

Selection

PROBE TYPE

Description	Value
Type	Integral

EXPRESSION

Description	Value
Expression	tds2.ntflux_Oxi
Table and plot unit	mol/s
Description	Normal total flux

TABLE AND WINDOW SETTINGS

Description	Value
Output table	Probe Table 1
Plot window	Probe Plot 1

UMEOCRnucleus

Probe type	Boundary probe

SELECTION

Geometric entity level	Boundary
Selection	Geometry geom2: Dimension 1: Boundary 10

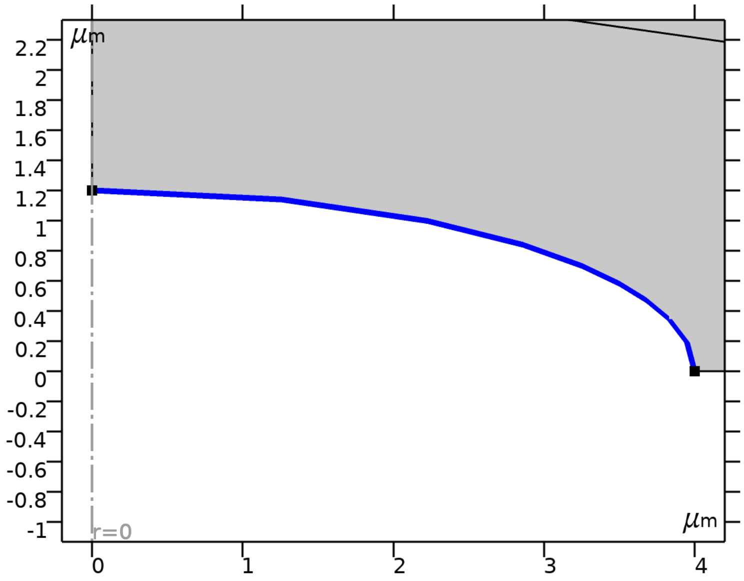

Selection

PROBE TYPE

Description	Value
Type	Integral

EXPRESSION

Description	Value
Expression	tds2.tflux_Oxiz
Description	tds2.tflux_Oxiz

TABLE AND WINDOW SETTINGS

Description	Value
Output table	Probe Table 1
Plot window	Probe Plot 1

Domain Point Probe 1

Probe type	Domain point probe

UMERadius

Probe type	Global variable probe

EXPRESSION

Description	Value
Expression	Rb

Description	Value
Table and plot unit	µm
Description	WElectrode radius

TABLE AND WINDOW SETTINGS

Description	Value
Output table	Probe Table 1
Plot window	Probe Plot 3

2.1.3 Coordinate Systems

Boundary System 2

Coordinate system type	Boundary system
Tag	sys2

COORDINATE NAMES

First	Second	Third
t1	to	n

2.2 GEOMETRY

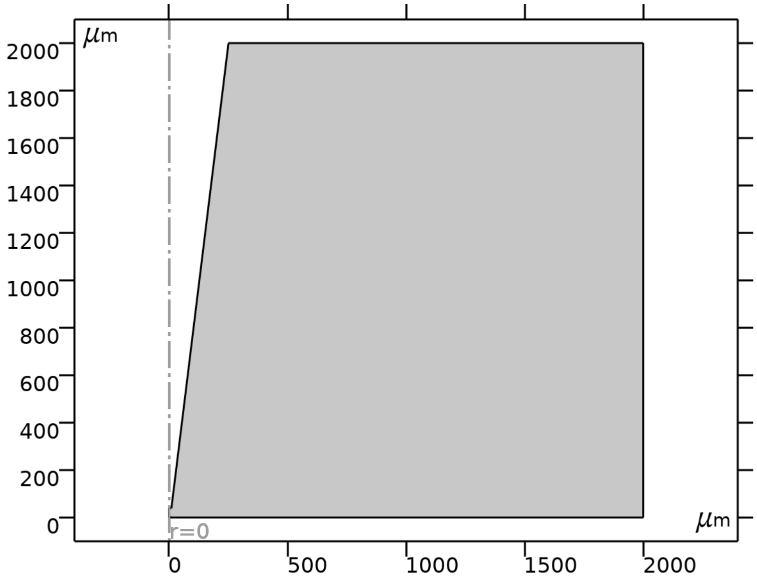

Geometry

UNITS

Length unit	µm
Angular unit	deg

GEOMETRY STATISTICS

Description	Value
Space dimension	2

Description	Value
Number of domains	2
Number of boundaries	11
Number of vertices	10

2.2.1 Bézier Polygon 1 (b1)

POLYGON SEGMENTS

Description	Value
Control points	{{0, Domain, Domain, tan(Beta)*(Domain - d2) + RG, RG, Rb, 0, 0}, {0, 0, Domain, Domain, d2, d2, d2, 0}}
Degree	{1, 1, 1, 1, 1, 1, 1}
Weights	{1, 1, 1, 1, 1, 1, 1, 1, 1, 1, 1, 1, 1, 1}
Type	Solid

2.2.2 Point 1 (pt1)

POINT

Description	Value
Point coordinate	{9, 0}

2.2.3 Ellipse 1 (e1)

POSITION

Description	Value
Position	{0, 0}

SIZE AND SHAPE

Description	Value
a-semiaxis	CellWidth
b-semiaxis	CellHeight
Sector angle	90

2.2.4 Ellipse 2 (e2)

POSITION

Description	Value
Position	{0, 0}

SIZE AND SHAPE

Description	Value
a-semiaxis	NucleusWidth
b-semiaxis	NucleusHeight
Sector angle	90

2.3 TRANSPORT OF DILUTED SPECIES 2

USED PRODUCTS

COMSOL Multiphysics

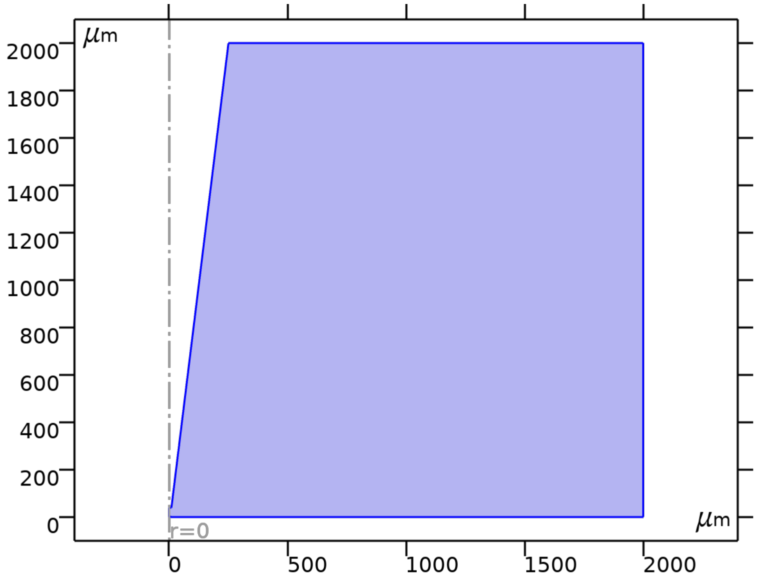

Transport of Diluted Species 2

SELECTION

Geometric entity level	Domain
Selection	Geometry geom2: Dimension 2: All domains

EQUATIONS

$$\nabla \cdot \mathbf{J}_i = R_i$$
$$\mathbf{J}_i = -D_i \nabla c_i$$

2.3.1 Interface Settings

Discretization

SETTINGS

**Description**	**Value**
Concentration	Linear

SETTINGS

**Description**	**Value**
Equation form	Study controlled

Transport Mechanisms

SETTINGS

**Description**	**Value**
Convection	Off

Description	Value
Migration in electric field	Off
Mass transfer in porous media	Off

2.3.2 Variables

Name	Expression	Unit	Description	Selection	Details
tds2.d	1	1	Out-of-plane geometry extension	Global	
tds2.nr	nr	1	Normal vector, r component	Boundary 11	
tds2.nphi	0	1	Normal vector, phi component	Boundary 11	
tds2.nz	nz	1	Normal vector, z component	Boundary 11	
tds2.nr	dnr	1	Normal vector, r component	Boundaries 1–10	
tds2.nphi	0	1	Normal vector, phi component	Boundaries 1–10	
tds2.nz	dnz	1	Normal vector, z component	Boundaries 1–10	
tds2.nrmesh	nrmesh	1	Normal vector (mesh), r component	Boundary 11	
tds2.nphimesh	0	1	Normal vector (mesh), phi component	Boundary 11	
tds2.nzmesh	nzmesh	1	Normal vector (mesh), z component	Boundary 11	
tds2.nrmesh	dnrmesh	1	Normal vector (mesh), r component	Boundaries 1–10	
tds2.nphimesh	0	1	Normal vector (mesh), phi component	Boundaries 1–10	
tds2.nzmesh	dnzmesh	1	Normal vector (mesh), z component	Boundaries 1–10	
tds2.nrc	root.nrc/tds2.ncLen	1	Normal vector, r component	Boundaries 1–11	
tds2.nphic	0	1	Normal vector, phi component	Boundaries 1–11	
tds2.nzc	root.nzc/tds2.ncLen	1	Normal vector, z component	Boundaries 1–11	
tds2.ncLen	sqrt(root.nrc^2+root.nzc^2+eps)	1	Help variable	Boundaries 1–11	
tds2.R_Oxi	0	mol/(m³·s)	Total rate	Domains 1–2	+ operation

Name	Expression	Unit	Description	Selection	Details
			expression		
tds2.cP_Oxi	0	mol/kg	Concentration species adsorbed to the solid	Domains 1–2	+ operation
tds2.cP_Oxi	0	mol/kg	Concentration species adsorbed to the solid	Boundaries 1–11	+ operation
tds2.KP_Oxi	0	m³/kg	Adsorption isotherm, first concentration derivative	Domains 1–2	+ operation
tds2.KP_Oxi	0	m³/kg	Adsorption isotherm, first concentration derivative	Boundaries 1–11	+ operation
tds2.Rads_Oxi	0	mol/(m³·s)	Total adsorption rate	Domains 1–2	+ operation
tds2.DiT_Oxi	0	m²/s	Turbulent diffusivity	Domains 1–2	
tds2.cVar_Oxi	Oxi	mol/m³	Species	Boundaries 1–11	
tds2.poro	1	1	Porosity	Domains 1–2	
tds2.theta_g	0	1	Gas volume fraction	Domains 1–2	
tds2.theta_l	1	1	Liquid volume fraction	Domains 1–2	
tds2.theta	tds2.poro	1	Mobile fluid volume fraction	Domains 1–2	

2.3.3 Media Diffusion

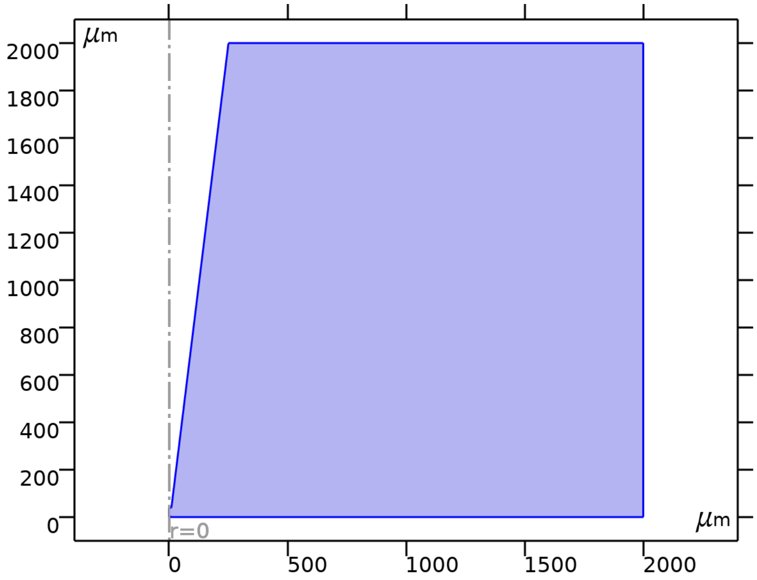

Media Diffusion

SELECTION

Geometric entity level	Domain
Selection	Geometry geom2: Dimension 2: All domains

EQUATIONS

$$\nabla \cdot \mathbf{J}_i = R_i$$
$$\mathbf{J}_i = -D_i \nabla c_i$$

Diffusion

SETTINGS

**Description**	**Value**
Source	Material
Material	None
Diffusion coefficient	User defined
Diffusion coefficient	{{D, 0, 0}, {0, D, 0}, {0, 0, D}}

Coordinate System Selection

SETTINGS

**Description**	**Value**
Coordinate system	Global coordinate system

Model Input

SETTINGS

**Description**	**Value**
Temperature	Common model input

Variables

Name	Expression	Unit	Description	Selection	Details
domflux.Oxir	2*tds2.dflux_Oxir*pi*r*tds2.d	mol/(m·s)	Domain flux, r component	Domains 1–2	
domflux.Oxiz	2*tds2.dflux_Oxiz*pi*r*tds2.d	mol/(m·s)	Domain flux, z component	Domains 1–2	
tds2.ndflux_Oxi	tds2.bndFlux_Oxi	mol/(m²·s)	Normal diffusive flux	Boundaries 3–11	
tds2.ntflux_Oxi	tds2.bndFlux_Oxi	mol/(m²·s)	Normal total flux	Boundaries 3–11	
tds2.DF_Oxirr	D	m²/s	Fluid diffusion coefficient, rr component	Domains 1–2	
tds2.DF_Oxiphir	0	m²/s	Fluid diffusion coefficient, phir component	Domains 1–2	
tds2.DF_Oxizr	0	m²/s	Fluid diffusion coefficient, zr component	Domains 1–2	
tds2.DF_Oxirphi	0	m²/s	Fluid diffusion coefficient, rphi component	Domains 1–2	
tds2.DF_Oxiphiphi	D	m²/s	Fluid diffusion coefficient, phiphi component	Domains 1–2	
tds2.DF_Oxizphi	0	m²/s	Fluid diffusion coefficient, zphi component	Domains 1–2	
tds2.DF_Oxirz	0	m²/s	Fluid diffusion coefficient, rz component	Domains 1–2	
tds2.DF_Oxiphiz	0	m²/s	Fluid diffusion coefficient, phiz component	Domains 1–2	
tds2.DF_Oxizz	D	m²/s	Fluid diffusion coefficient, zz component	Domains 1–2	
tds2.D_Oxirr	tds2.DF_Oxirr+tds2.DiT_Oxi	m²/s	Diffusion coefficient, rr component	Domains 1–2	
tds2.D_Oxiphir	tds2.DF_Oxiphir	m²/s	Diffusion coefficient, phir component	Domains 1–2	
tds2.D_Oxizr	tds2.DF_Oxizr	m²/s	Diffusion coefficient, zr component	Domains 1–2	
tds2.D_Oxirphi	tds2.DF_Oxirphi	m²/s	Diffusion coefficient, rphi component	Domains 1–2	

Name	Expression	Unit	Description	Selection	Details
tds2.D_Oxiphiphi	tds2.DF_Oxiphiphi+ tds2.DiT_Oxi	m²/s	Diffusion coefficient, phiphi component	Domains 1–2	
tds2.D_Oxizphi	tds2.DF_Oxizphi	m²/s	Diffusion coefficient, zphi component	Domains 1–2	
tds2.D_Oxirz	tds2.DF_Oxirz	m²/s	Diffusion coefficient, rz component	Domains 1–2	
tds2.D_Oxiphiz	tds2.DF_Oxiphiz	m²/s	Diffusion coefficient, phiz component	Domains 1–2	
tds2.D_Oxizz	tds2.DF_Oxizz+tds 2.DiT_Oxi	m²/s	Diffusion coefficient, zz component	Domains 1–2	
tds2.Dav_Oxi	0.5*(tds2.D_Oxirr+t ds2.D_Oxizz)	m²/s	Average diffusion coefficient	Domains 1–2	
tds2.tflux_Oxir	tds2.dflux_Oxir	mol/(m²·s)	Total flux, r component	Domains 1–2	+ operation
tds2.tflux_Oxiphi	tds2.dflux_Oxiphi	mol/(m²·s)	Total flux, phi component	Domains 1–2	+ operation
tds2.tflux_Oxiz	tds2.dflux_Oxiz	mol/(m²·s)	Total flux, z component	Domains 1–2	+ operation
tds2.dfluxMag_O xi	sqrt(tds2.dflux_Oxir ^2+tds2.dflux_Oxip hi^2+tds2.dflux_Oxi z^2)	mol/(m²·s)	Diffusive flux magnitude	Domains 1–2	
tds2.tfluxMag_Ox i	sqrt(tds2.tflux_Oxir^ 2+tds2.tflux_Oxiphi ^2+tds2.tflux_Oxiz^ 2)	mol/(m²·s)	Total flux magnitude	Domains 1–2	
tds2.dpflux_Oxir	0	mol/(m²·s)	Dispersive flux, r component	Domains 1–2	
tds2.dpflux_Oxip hi	0	mol/(m²·s)	Dispersive flux, phi component	Domains 1–2	
tds2.dpflux_Oxiz	0	mol/(m²·s)	Dispersive flux, z component	Domains 1–2	
tds2.dflux_Oxir	-tds2.D_Oxirr*Oxir- tds2.D_Oxirz*Oxiz	mol/(m²·s)	Diffusive flux, r component	Domains 1–2	+ operation
tds2.dflux_Oxiphi	- tds2.D_Oxiphir*Oxir - tds2.D_Oxiphiz*Oxi z	mol/(m²·s)	Diffusive flux, phi component	Domains 1–2	+ operation
tds2.dflux_Oxiz	-tds2.D_Oxizr*Oxir- tds2.D_Oxizz*Oxiz	mol/(m²·s)	Diffusive flux, z component	Domains 1–2	+ operation
tds2.grad_Oxir	Oxir	mol/m⁴	Concentration gradient, r component	Domains 1–2	

Name	Expression	Unit	Description	Selection	Details
tds2.grad_Oxiphi	0	mol/m⁴	Concentration gradient, phi component	Domains 1–2	
tds2.grad_Oxiz	Oxiz	mol/m⁴	Concentration gradient, z component	Domains 1–2	
tds2.bndFlux_Oxi	0.25*(uflux_spatial(Oxi)-dflux_spatial(Oxi))/(pi*r*tds2.d)	mol/(m²·s)	Boundary flux	Boundary 11	Meta
tds2.bndFlux_Oxi	-dflux_spatial(Oxi)/tds2.d	mol/(m²·s)	Boundary flux	Boundaries 1–2	
tds2.bndFlux_Oxi	-0.5*dflux_spatial(Oxi)/(pi*r*tds2.d)	mol/(m²·s)	Boundary flux	Boundaries 3–10	Meta
tds2.Res_Oxi	-tds2.R_Oxi	mol/(m³·s)	Equation residual	Domains 1–2	
tds2.Rlin_Oxi	0		Linear source term coefficient	Domains 1–2	

Shape functions

Name	Shape function	Unit	Description	Shape frame	Selection
Oxi	Lagrange (Linear)	mol/m³	Concentration	Spatial	Domains 1–2

Weak Expressions

Weak expression	Integration order	Integration frame	Selection
2*(tds2.dflux_Oxir*test(Oxir)+tds2.dflux_Oxiz*test(Oxiz))*tds2.d*pi*r	2	Spatial	Domains 1–2
2*tds2.streamline*(isScalingSystemDomain==0)*tds2.d*pi*r	2	Spatial	Domains 1–2
2*tds2.crosswind*(isScalingSystemDomain==0)*tds2.d*pi*r	4	Spatial	Domains 1–2

2.3.4 Axial Symmetry 1

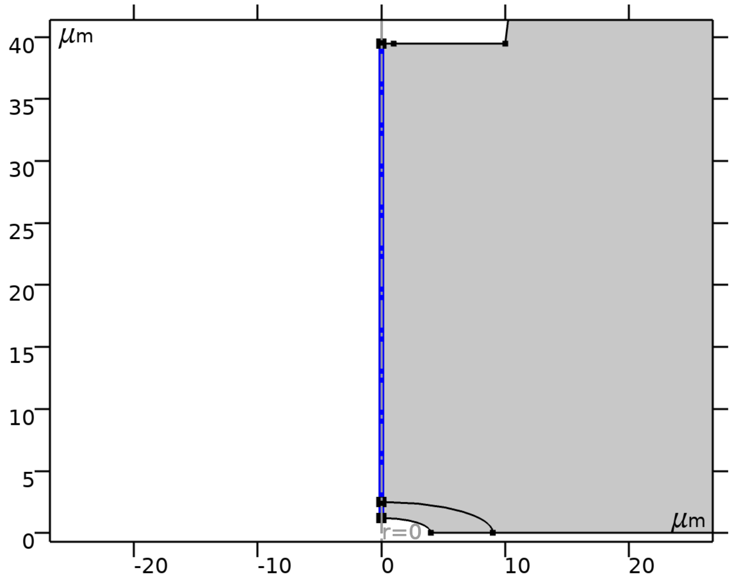

Axial Symmetry 1

SELECTION

Geometric entity level	Boundary
Selection	Geometry geom2: Dimension 1: All boundaries

2.3.5 No Flux 1

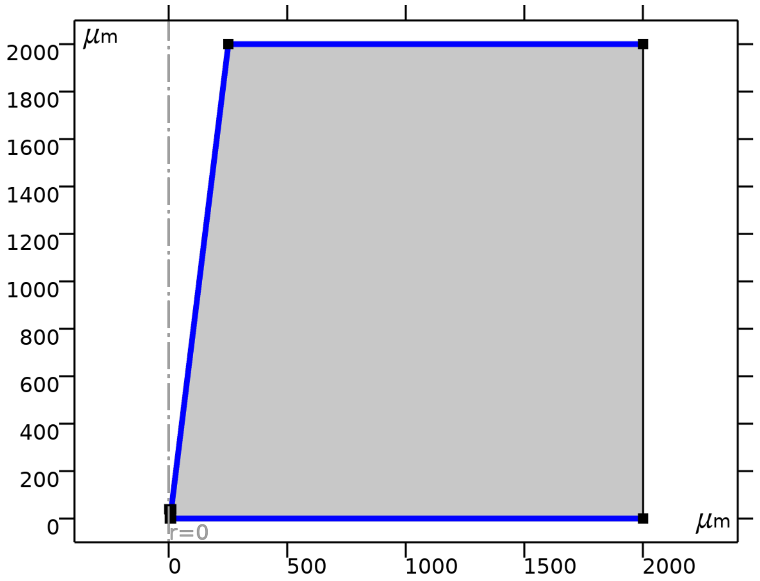

No Flux 1

SELECTION

Geometric entity level	Boundary
Selection	Geometry geom2: Dimension 1: All boundaries

EQUATIONS

$$-\mathbf{n}\cdot\mathbf{J}_i = 0$$

2.3.6 Initial Values Media

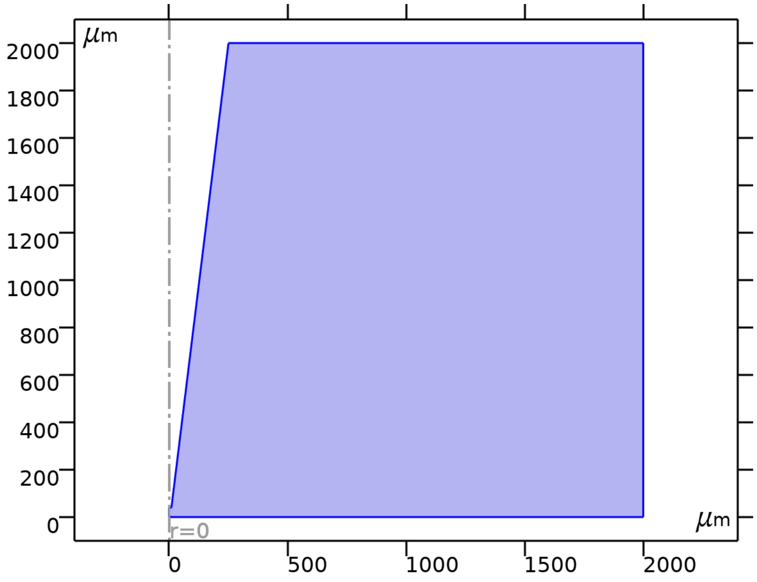

Initial Values Media

SELECTION

Geometric entity level	Domain
Selection	Geometry geom2: Dimension 2: All domains

Initial Values

SETTINGS

Description	Value
Concentration	COx

Variables

Name	Expression	Unit	Description	Selection	Details
tds2.c0_Oxi	COx	mol/m³	Concentration	Domains 1–2	+ operation

2.3.7 UME

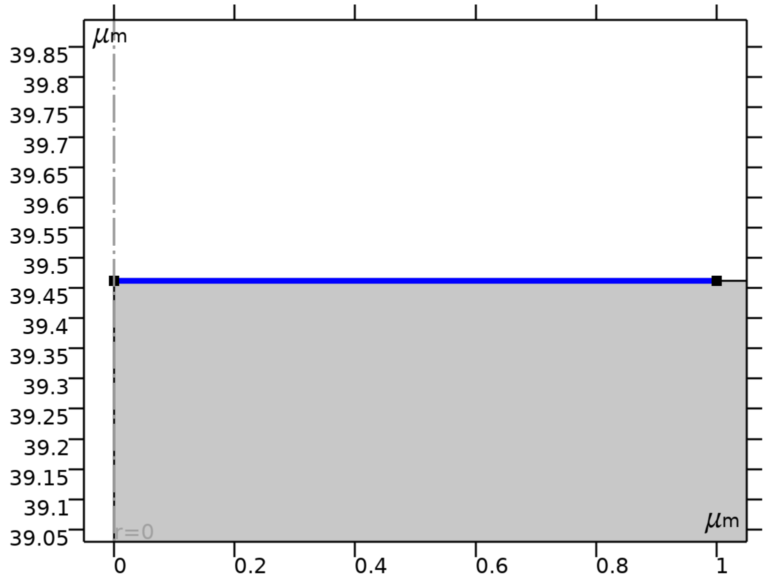

UME

SELECTION

Geometric entity level	Boundary
Selection	Geometry geom2: Dimension 1: Boundary 3

EQUATIONS

$$-\mathbf{n}\cdot\mathbf{J}_i = J_{0,i}$$

Inward Flux

SETTINGS

Description	Value
Flux type	General inward flux
Species Oxi	On
	-k0*Oxi

Variables

Name	Expression	Unit	Description	Selection
tds2.fl1.nmflow_Oxi	tds2.fl1.int(2*tds2.ntflux_Oxi*pi *r)*tds2.d	mol/s	Normal molar flow rate	Global

Weak Expressions

Weak expression	Integration order	Integration frame	Selection
-2*k0*Oxi*test(Oxi)*tds2.d*pi*r	2	Spatial	Boundary 3

2.3.8 Bulk Concentration

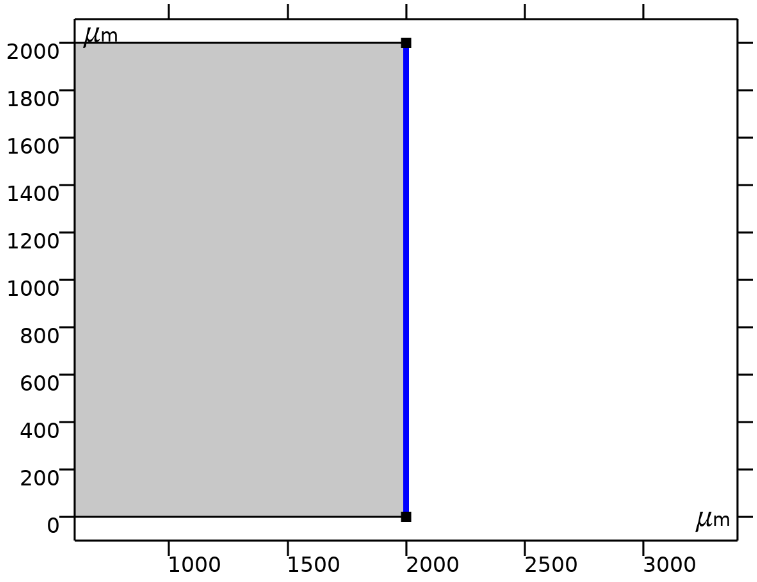

Bulk Concentration

SELECTION

Geometric entity level	Boundary
Selection	Geometry geom2: Dimension 1: Boundary 9

EQUATIONS

$$c_i = c_{0,i}$$

Concentration

SETTINGS

Description	Value
Species Oxi	On
Concentration	COx

Variables

Name	Expression	Unit	Description	Selection	Details
tds2.c0_Oxi	COx	mol/m³	Concentration	Boundary 9	+ operation
tds2.conc1.nmflow_Oxi	tds2.conc1.int(2*tds2.ntflux_Oxi*pi*r)*tds2.d	mol/s	Normal molar flow rate	Global	

Constraints

Constraint	Constraint force	Shape function	Selection	Details
-tds2.cVar_Oxi+tds2.c0_Oxi	test(-tds2.cVar_Oxi+tds2.c0_Oxi)	Lagrange (Linear)	Boundary 9	Elemental

2.3.9 UME_PAC

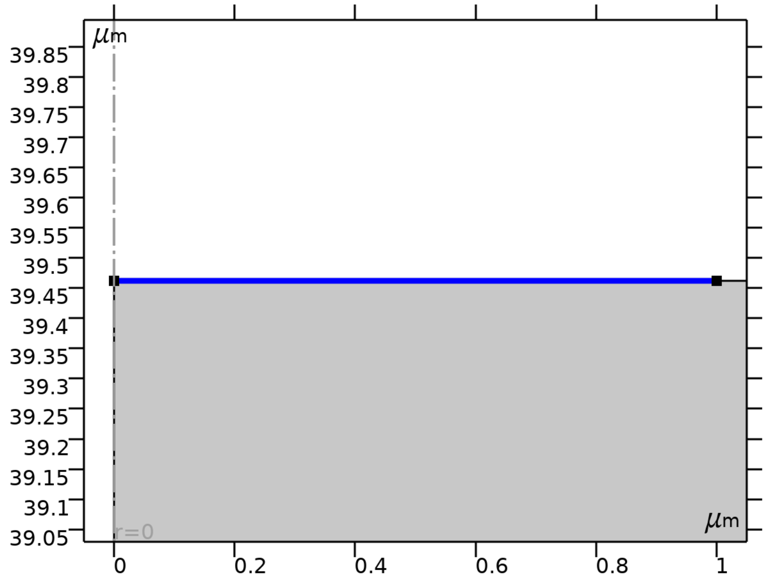

UME_PAC

SELECTION

Geometric entity level	Boundary
Selection	Geometry geom2: Dimension 1: Boundary 3

EQUATIONS

$$-\mathbf{n}\cdot\mathbf{J}_i = J_{0,i}$$

Inward Flux

SETTINGS

Description	Value
Flux type	General inward flux
Species Oxi	On
	-k0*Oxi

Variables

Name	Expression	Unit	Description	Selection
tds2.fl2.nmflow_Oxi	tds2.fl2.int(2*tds2.ntflux_Oxi*pi *r)*tds2.d	mol/s	Normal molar flow rate	Global

Weak Expressions

Weak expression	Integration order	Integration frame	Selection
-2*k0*Oxi*test(Oxi)*tds2.d*pi*r	2	Spatial	Boundary 3

2.3.10 Cell Respiration

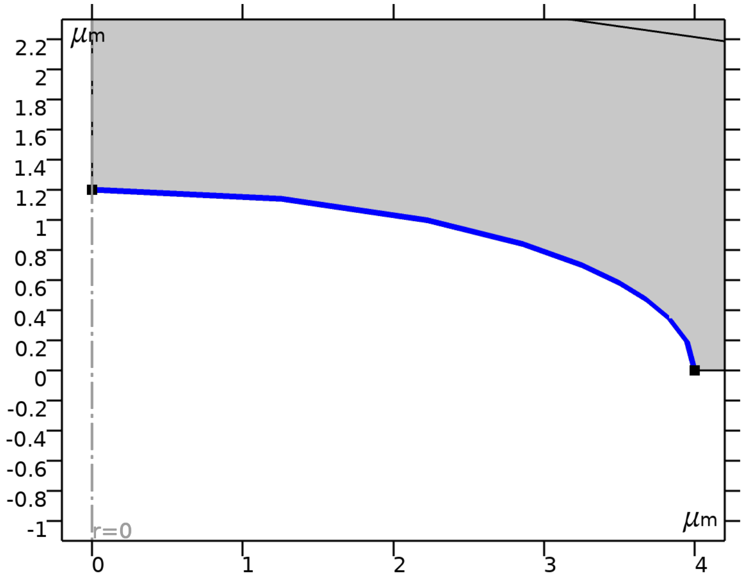

Cell Respiration

SELECTION

Geometric entity level	Boundary
Selection	Geometry geom2: Dimension 1: Boundary 10

EQUATIONS

$$-\mathbf{n}\cdot\mathbf{J}_i = J_{0,i}$$

Inward Flux

SETTINGS

Description	Value
Flux type	General inward flux
Species Oxi	On
	-k0Sub*Oxi

Variables

Name	Expression	Unit	Description	Selection
tds2.fl5.nmflow_Oxi	tds2.fl5.int(2*tds2.ntflux_Oxi*pi *r)*tds2.d	mol/s	Normal molar flow rate	Global

Weak Expressions

Weak expression	Integration order	Integration frame	Selection
-2*k0Sub*Oxi*test(Oxi)*tds2.d*pi*r	2	Spatial	Boundary 10

2.3.11 UME_Pulse

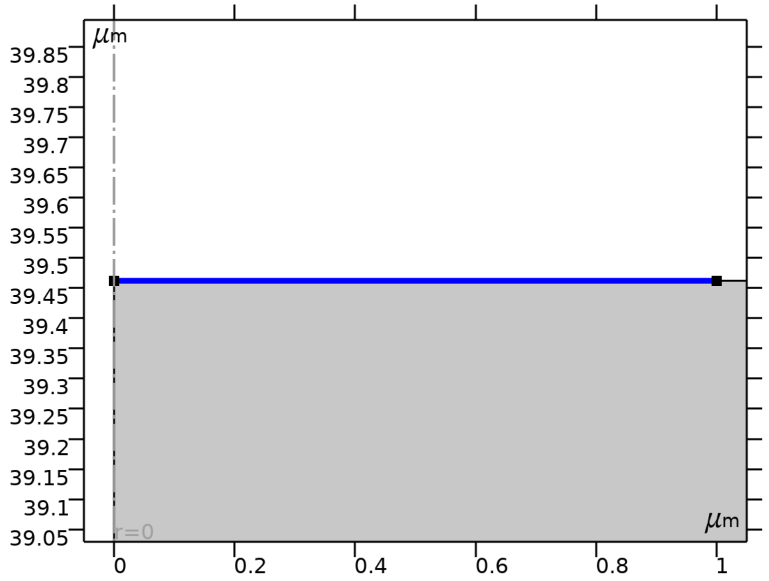

UME_Pulse

SELECTION

Geometric entity level	Boundary
Selection	Geometry geom2: Dimension 1: Boundary 3

EQUATIONS

$$-\mathbf{n}\cdot\mathbf{J}_i = J_{0,i}$$

Inward Flux

SETTINGS

Description	Value
Flux type	General inward flux
Species Oxi	On
	-k0*Oxi

Variables

Name	Expression	Unit	Description	Selection
tds2.fl4.nmflow_Oxi	tds2.fl4.int(2*tds2.ntflux_Oxi*pi *r)*tds2.d	mol/s	Normal molar flow rate	Global

Weak Expressions

Weak expression	Integration order	Integration frame	Selection
-2*k0*Oxi*test(Oxi)*tds2.d*pi*r	2	Spatial	Boundary 3

2.3.12 UME Conc at Steady State

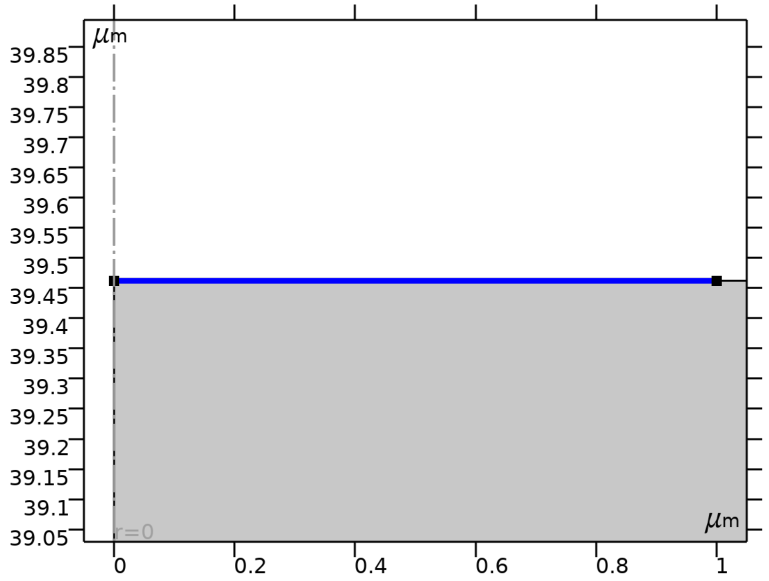

UME Conc at Steady State

SELECTION

Geometric entity level	Boundary
Selection	Geometry geom2: Dimension 1: Boundary 3

EQUATIONS

$$c_i = c_{0,j}$$

Concentration

SETTINGS

Description	Value
Species Oxi	On
Concentration	0

Variables

Name	Expression	Unit	Description	Selection	Details
tds2.c0_Oxi	0	mol/m³	Concentration	Boundary 3	+ operation
tds2.conc2.nmflow_Oxi	tds2.conc2.int(2*tds2.ntflux_Oxi*pi*r)*tds2.d	mol/s	Normal molar flow rate	Global	

Constraints

Constraint	Constraint force	Shape function	Selection	Details
-tds2.cVar_Oxi+tds2.c0_Oxi	test(-tds2.cVar_Oxi+tds2.c0_Oxi)	Lagrange (Linear)	Boundary 3	Elemental

2.3.13 UME_Pause

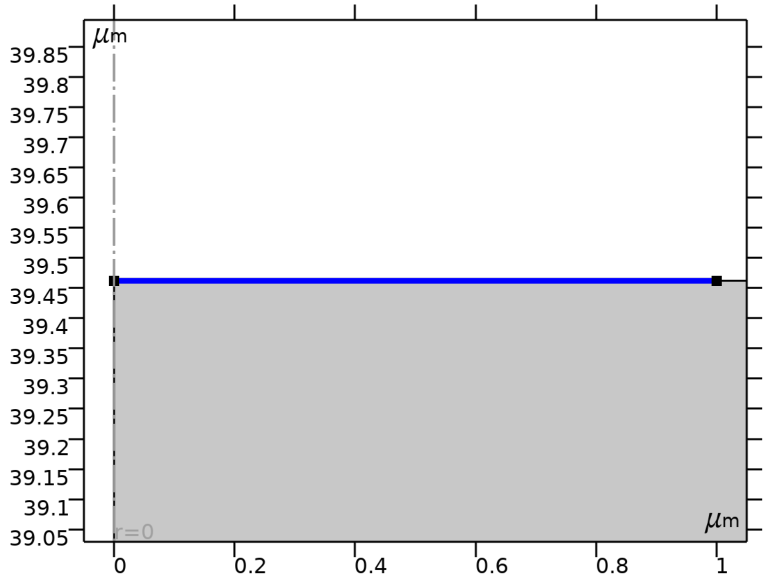

UME_Pause

SELECTION

Geometric entity level	Boundary
Selection	Geometry geom2: Dimension 1: Boundary 3

EQUATIONS

$$-\mathbf{n}\cdot\mathbf{J}_i = J_{0,i}$$

Inward Flux

SETTINGS

Description	Value
Flux type	General inward flux
Species Oxi	On
	-k0*Oxi*step1(t/1[s])

Variables

Name	Expression	Unit	Description	Selection
tds2.fl6.nmflow_Oxi	tds2.fl6.int(2*tds2.ntflux_Oxi*pi*r)*tds2.d	mol/s	Normal molar flow rate	Global

Weak Expressions

Weak expression	Integration order	Integration frame	Selection
-2*k0*Oxi*step1(t/1[s])*test(Oxi)*tds2.d*pi*r	2	Spatial	Boundary 3

2.3.14 Cytosol Diffusion

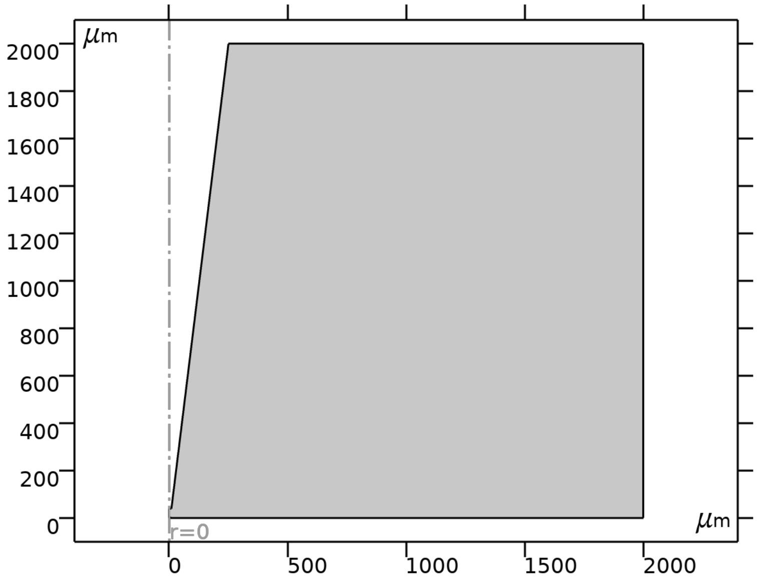

Cytosol Diffusion

SELECTION

Geometric entity level	Domain
Selection	Geometry geom2: Dimension 2: No domains

EQUATIONS

$$\nabla \cdot \mathbf{J}_i = R_i$$
$$\mathbf{J}_i = -D_i \nabla c_i$$

Diffusion

SETTINGS

Description	Value
Source	Material
Material	None
Diffusion coefficient	User defined
Diffusion coefficient	{{DCytosol, 0, 0}, {0, DCytosol, 0}, {0, 0, DCytosol}}

Coordinate System Selection

SETTINGS

Description	Value
Coordinate system	Global coordinate system

Model Input

SETTINGS

Description	Value
Temperature	Common model input

Shape functions

Name	Shape function	Unit	Description	Shape frame	Selection
Oxi	Lagrange (Linear)	mol/m³	Concentration	Spatial	No domains

2.3.15 Initial Values Cytosol

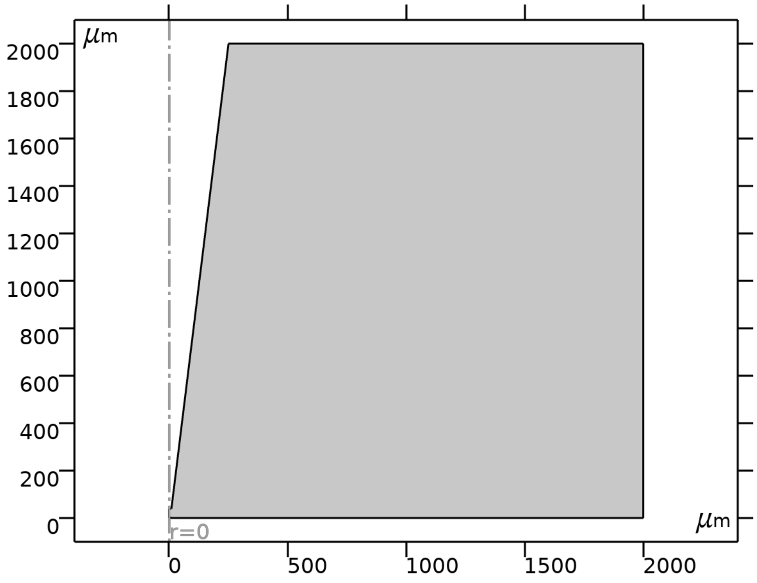

Initial Values Cytosol

SELECTION

Geometric entity level	Domain
Selection	Geometry geom2: Dimension 2: No domains

Initial Values

SETTINGS

Description	Value
Concentration	COxCytosol

2.3.16 UME_SetCurrent

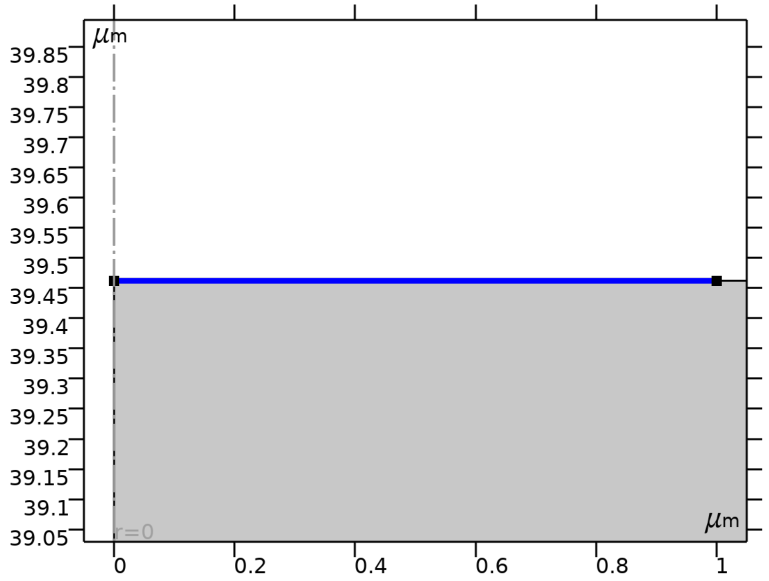

UME_SetCurrent

SELECTION

Geometric entity level	Boundary
Selection	Geometry geom2: Dimension 1: Boundary 3

EQUATIONS

$$-\mathbf{n}\cdot\mathbf{J}_i = J_{0,i}$$

Inward Flux

SETTINGS

Description	Value
Flux type	General inward flux
Species Oxi	On
	-O2flux

Variables

Name	Expression	Unit	Description	Selection
tds2.fl7.nmflow_Oxi	tds2.fl7.int(2*tds2.ntflux_Oxi*pi *r)*tds2.d	mol/s	Normal molar flow rate	Global

Weak Expressions

Weak expression	Integration order	Integration frame	Selection
-2*O2flux*test(Oxi)*tds2.d*pi*r	2	Spatial	Boundary 3

2.4 MESH 2

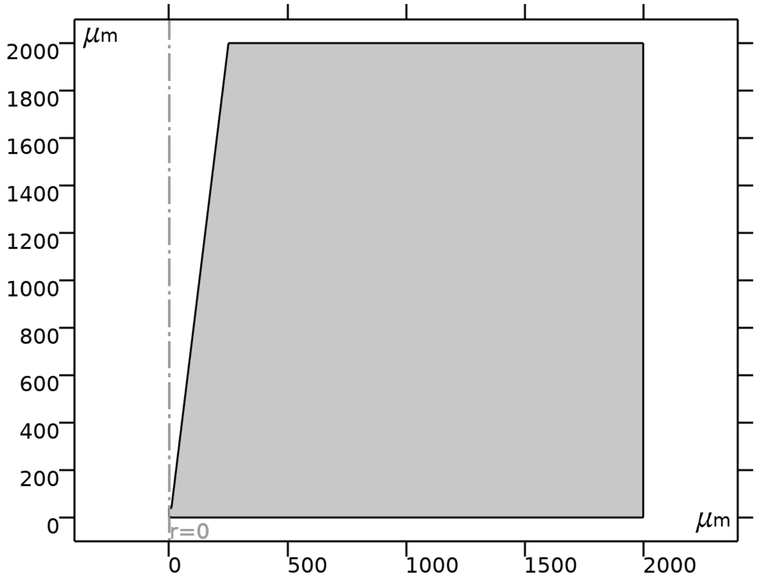

Mesh 2

MESH STATISTICS

Description	Value
Status	Empty mesh
Mesh vertices	0

2.4.1 Size (size)

SETTINGS

Description	Value
Maximum element size	106
Minimum element size	0.6
Curvature factor	0.3
Maximum element growth rate	1.3
Predefined size	Fine

2.4.2 Free Triangular 1 (ftri1)

SELECTION

Geometric entity level	Domain
Selection	Geometry geom2: Dimension 2: Domains 1–2

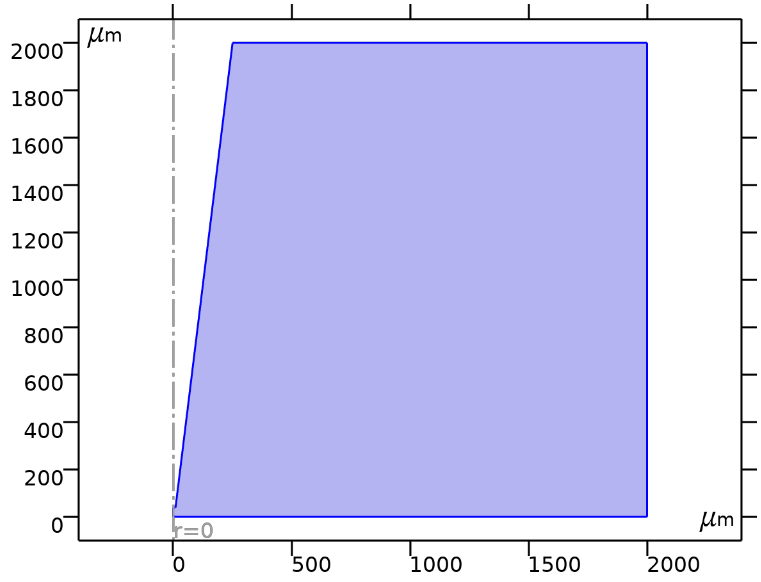

Free Triangular 1

SETTINGS

Description	Value
Last build time	1
Built with	COMSOL 6.0.0.405 (win64)|2023 - 05 - 28T16:05:53.587212600

UME (size1)

SELECTION

Geometric entity level	Boundary
Selection	Geometry geom2: Dimension 1: Boundary 3

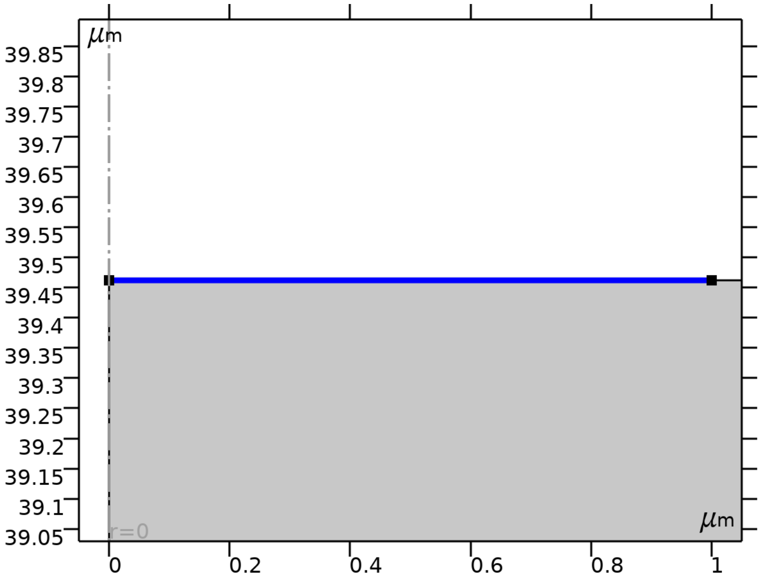

UME

SETTINGS

Description	Value
Maximum element size	Rb/200
Minimum element size	0.3
Minimum element size	Off
Curvature factor	0.3
Curvature factor	Off
Resolution of narrow regions	Off
Maximum element growth rate	1.06
Custom element size	Custom

Domain (size2)

SELECTION

Geometric entity level	Domain
Selection	Geometry geom2: Dimension 2: Domains 1–2

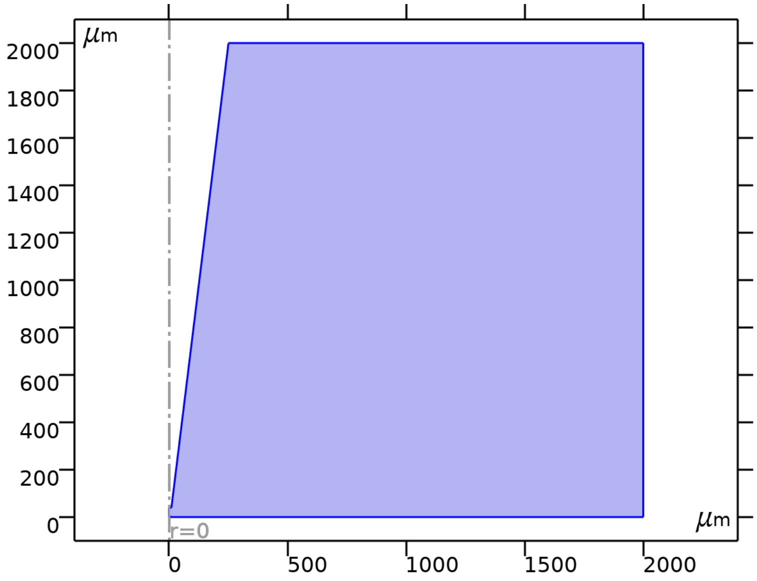

Domain

SETTINGS

Description	Value
Maximum element size	20
Minimum element size	0.04
Curvature factor	0.2
Predefined size	Extremely fine

UMEEdge (size3)

SELECTION

Geometric entity level	Point

Selection	Geometry geom2: Dimension 0: Point 4

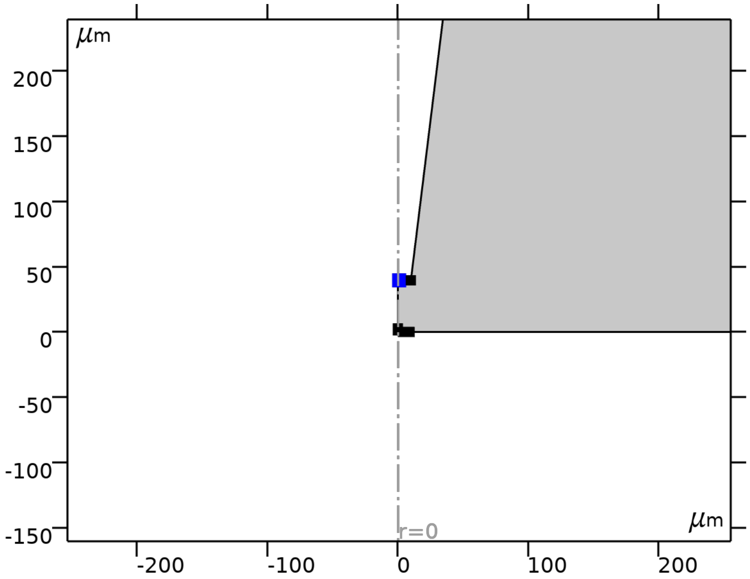

UMEEdge

SETTINGS

**Description**	**Value**
Maximum element size	Rb/300
Minimum element size	0.075
Minimum element size	Off
Curvature factor	0.3
Curvature factor	Off
Resolution of narrow regions	Off
Maximum element growth rate	1.3
Maximum element growth rate	Off
Custom element size	Custom

Substrate (size4)

SELECTION

Geometric entity level	Boundary
Selection	Geometry geom2: Dimension 1: No boundaries

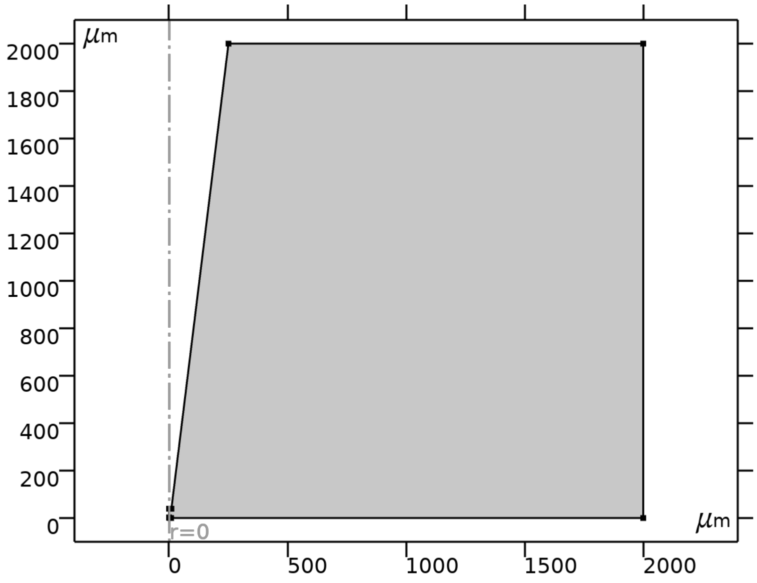

Substrate

SETTINGS

Description	Value
Maximum element size	CellWidth/250
Minimum element size	0.075
Minimum element size	Off
Curvature factor	0.3
Curvature factor	Off
Resolution of narrow regions	Off
Maximum element growth rate	1.3
Maximum element growth rate	Off
Custom element size	Custom

2.4.3 Mapped 1 (map1)

SELECTION

Geometric entity level	Remaining

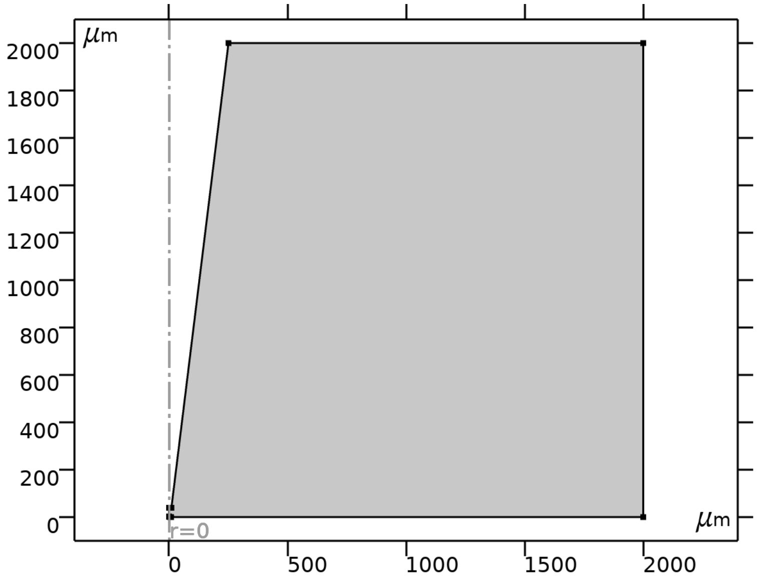

Mapped 1

SETTINGS

Description	Value
Last build time	0
Built with	COMSOL 6.0.0.405 (win64)|2023 - 05 - 28T16:28:17.341959

Distribution 1 (dis1)

SELECTION

Geometric entity level	Boundary
Selection	Geometry geom2: Dimension 1: No boundaries

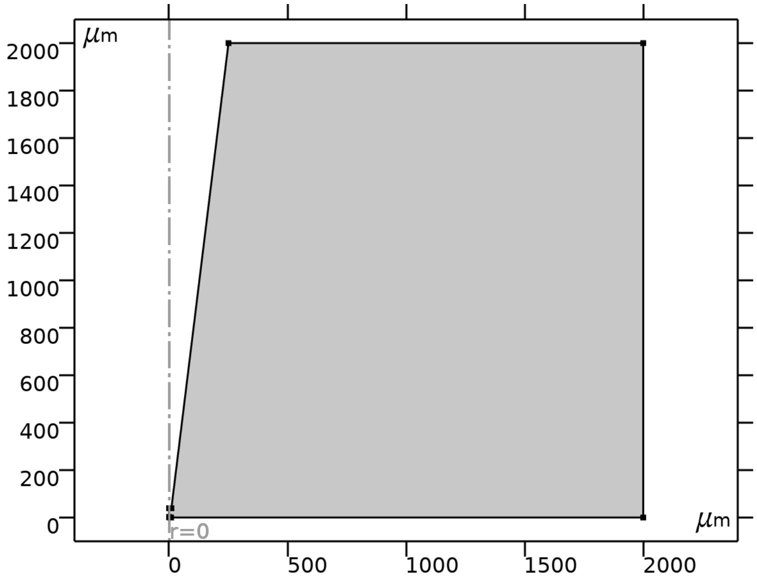

Distribution 1

SETTINGS

Description	Value
Distribution type	Predefined
Number of elements	20
Element ratio	10
Reverse direction	On

3 Oxygen Pulse

COMPUTATION INFORMATION

Computation time	26 min 29 s

3.1 PARAMETRIC SWEEP

Parameter name	Parameter value list	Parameter unit
k0Sub	0[m/s],1.51e-9[m/s], 1.51e-8[m/s], 1.51e-7[m/s], 1.51e-6[m/s], 1.51e-5[m/s], 1.51e-4[m/s]	m/s
d	0.5[um], 1[um], 1.5[um], 2[um], 2.4[um], 3[um], 4[um], 5[um], 6[um], 8[um], 12[um], 15[um]	m

STUDY SETTINGS

Description	Value
Sweep type	All combinations
Parameter name	{k0Sub, d}
Unit	{m/s, m}

PARAMETERS

Parameter name	Parameter value list	Parameter unit
k0Sub (Substrate Kinetics)	0[m/s],1.51e-9[m/s], 1.51e-8[m/s], 1.51e-7[m/s], 1.51e-6[m/s], 1.51e-5[m/s], 1.51e-4[m/s]	m/s
d (tip-substrate separation)	0.5[um], 1[um], 1.5[um], 2[um], 2.4[um], 3[um], 4[um], 5[um], 6[um], 8[um], 12[um], 15[um]	m

3.2 STATIONARY

STUDY SETTINGS

Description	Value
Include geometric nonlinearity	Off

PHYSICS AND VARIABLES SELECTION

Physics interface	Discretization
Transport of Diluted Species 2 (tds2)	physics

MESH SELECTION

Geometry	Mesh
Geometry (geom2)	mesh2

3.3 OXYGEN PROBE PULSE

Times	Unit
range(0,Pulse_step1,Pulse_period1), range(Pulse_period1,Pulse_step2,Pulse_t)	s

STUDY SETTINGS

Description	Value
Include geometric nonlinearity	Off

STUDY SETTINGS

Description	Value
Output times	{0, 0.005, 0.01, 0.015, 0.02, 0.025, 0.03, 0.035, 0.04, 0.045, 0.05, 0.055, 0.06, 0.065, 0.07, 0.075, 0.08, 0.085, 0.09, 0.095, 0.1, 0.105, 0.11, 0.115, 0.12, 0.125, 0.13, 0.135, 0.14, 0.145, 0.15, 0.155, 0.16, 0.165, 0.17, 0.17500000000000002, 0.18, 0.185, 0.19, 0.195, 0.2, 0.20500000000000002, 0.21, 0.215, 0.22, 0.225, 0.23, 0.23500000000000001, 0.24, 0.245, 0.25, 0.25, 0.27, 0.29, 0.31, 0.33, 0.35, 0.37, 0.39, 0.41000000000000003, 0.43, 0.45, 0.47, 0.49, 0.51, 0.53, 0.55, 0.5700000000000001, 0.5900000000000001, 0.61, 0.63, 0.65, 0.6699999999999999, 0.69, 0.71, 0.73, 0.75, 0.77, 0.79, 0.81, 0.83, 0.85, 0.87, 0.89, 0.91, 0.93, 0.9500000000000001, 0.97, 0.99}

PHYSICS AND VARIABLES SELECTION

Physics interface	Discretization
Transport of Diluted Species 2 (tds2)	physics

MESH SELECTION

Geometry	Mesh
Geometry (geom2)	mesh2

3.4 SOLVER CONFIGURATIONS

3.4.1 Solution 958

Compile Equations: Stationary (st1)

STUDY AND STEP

Description	Value
Use study	Oxygen Pulse
Use study step	Stationary

LOG

```
<---- Compile Equations: Stationary in Oxygen Pulse/Solution 958 (sol958) ------
Started at May 28, 2023, 4:28:17 PM.
Parameter k0Sub = 1.51E-4 (m/s).
Parameter d = 1.4999999999999999E-5 (m).
Geometry shape function: Linear Lagrange
Number of vertex elements: 10
Minimum element quality: 0.4444
Time: 1 s.
Physical memory: 1.37 GB
Virtual memory: 1.35 GB
Ended at May 28, 2023, 4:28:17 PM.
----- Compile Equations: Stationary in Oxygen Pulse/Solution 958 (sol958) ----->
```

Dependent Variables 1 (v1)

GENERAL

Description	Value
Defined by study step	Stationary

LOG

```
<---- Dependent Variables 1 in Oxygen Pulse/Solution 958 (sol958) --------------
Started at May 28, 2023, 4:28:17 PM.
Solution time: 0 s.
Physical memory: 1.37 GB
Virtual memory: 1.35 GB
Ended at May 28, 2023, 4:28:17 PM.
----- Dependent Variables 1 in Oxygen Pulse/Solution 958 (sol958) ------------->
```

Concentration (comp2.Oxi) (comp2_Oxi)

GENERAL

Description	Value
Field components	comp2.Oxi
Internal variables	{comp2.uflux.Oxi, comp2.dflux.Oxi}

Stationary Solver 1 (s1)

GENERAL

Description	Value
Defined by study step	Stationary

RESULTS WHILE SOLVING

Description	Value
Probes	None

LOG

```
<---- Stationary Solver 1 in Oxygen Pulse/Solution 958 (sol958) ----------------
Started at May 28, 2023, 4:28:17 PM.
Linear solver
Number of degrees of freedom solved for: 18918 (plus 952 internal DOFs).
Symmetric matrices found.
Scales for dependent variables:
Concentration (comp2.Oxi): 0.23
Orthonormal null-space function used.
Iter      SolEst     Damping    Stepsize #Res #Jac #Sol   LinErr   LinRes
   1       0.033   1.0000000       0.033    1    1    1  2.1e-13  3.9e-14
Solution time: 1 s.
Physical memory: 1.38 GB
Virtual memory: 1.36 GB
Ended at May 28, 2023, 4:28:18 PM.
----- Stationary Solver 1 in Oxygen Pulse/Solution 958 (sol958) --------------->
```

Fully Coupled 1 (fc1)

GENERAL

Description	Value
Linear solver	Direct 1

METHOD AND TERMINATION

Description	Value
Initial damping factor	0.01
Minimum damping factor	1.0E-6
Maximum number of iterations	50

Direct 1 (d1)

GENERAL

Description	Value
Solver	PARDISO
Pivoting perturbation	1.0E-13

Solution Store 1 (su1)

GENERAL

Description	Value
Solution	Solution Store 1

Compile Equations: Oxygen Probe Pulse (st2)

STUDY AND STEP

Description	Value
Use study	Oxygen Pulse
Use study step	Oxygen Probe Pulse

LOG

```
<---- Compile Equations: Oxygen Probe Pulse in Oxygen Pulse/Solution 958
      (sol958) -----------------------------------------------------------------
Started at May 28, 2023, 4:28:18 PM.
Geometry shape function: Linear Lagrange
Time: 0 s.
Physical memory: 1.38 GB
Virtual memory: 1.36 GB
Ended at May 28, 2023, 4:28:18 PM.
----- Compile Equations: Oxygen Probe Pulse in Oxygen Pulse/Solution 958
      (sol958) ----------------------------------------------------------------->
```

Dependent Variables 2 (v2)

GENERAL

Description	Value
Defined by study step	Oxygen Probe Pulse

INITIAL VALUES OF VARIABLES SOLVED FOR

Description	Value
Method	Solution
Solution	Solution 958

RESIDUAL SCALING

Description	Value
Method	Manual

VALUES OF VARIABLES NOT SOLVED FOR

Description	Value
Method	Solution
Solution	Solution 958

INITIAL VALUE CALCULATION CONSTANTS

Constant name	Initial value source
t	range(0,Pulse_step1,Pulse_period1), range(Pulse_period1,Pulse_step2,Pulse_t)

LOG

```
<---- Dependent Variables 2 in Oxygen Pulse/Solution 958 (sol958) --------------
Started at May 28, 2023, 4:28:18 PM.
Initial values of variables solved for: Solution 958 (sol958), Solution Store 1 (sol
985).
Values of variables not solved for: Solution 958 (sol958), Solution Store 1 (sol985)
.
Solution time: 0 s.
Physical memory: 1.38 GB
Virtual memory: 1.36 GB
Ended at May 28, 2023, 4:28:18 PM.
----- Dependent Variables 2 in Oxygen Pulse/Solution 958 (sol958) ------------->
```

Concentration (comp2.Oxi) (comp2_Oxi)

GENERAL

Description	Value
Field components	comp2.Oxi
Internal variables	{comp2.uflux.Oxi, comp2.dflux.Oxi, comp2.tds2.dt2Inv_Oxi}

Time-Dependent Solver 1 (t1)

GENERAL

Description	Value
Defined by study step	Oxygen Probe Pulse
Output times	{0, 0.005, 0.01, 0.015, 0.02, 0.025, 0.03, 0.035, 0.04, 0.045, 0.05, 0.055, 0.06, 0.065, 0.07, 0.075, 0.08, 0.085, 0.09, 0.095, 0.1, 0.105, 0.11, 0.115, 0.12, 0.125, 0.13, 0.135, 0.14, 0.145, 0.15, 0.155, 0.16, 0.165, 0.17, 0.17500000000000002, 0.18, 0.185, 0.19, 0.195, 0.2, 0.20500000000000002, 0.21, 0.215, 0.22, 0.225, 0.23, 0.23500000000000001, 0.24, 0.245, 0.25, 0.25, 0.27, 0.29, 0.31, 0.33, 0.35, 0.37, 0.39, 0.41000000000000003, 0.43, 0.45, 0.47, 0.49, 0.51, 0.53, 0.55, 0.5700000000000001, 0.5900000000000001, 0.61, 0.63, 0.65, 0.6699999999999999, 0.69, 0.71, 0.73, 0.75, 0.77, 0.79, 0.81, 0.83, 0.85, 0.87, 0.89, 0.91, 0.93, 0.9500000000000001, 0.97, 0.99}
Relative tolerance	0.005

TIME STEPPING

Description	Value

Description	Value
Maximum BDF order	2

RESULTS WHILE SOLVING

Description	Value
Plot	On
Plot group	Probe Plot Group 3

LOG

```
<---- Time-Dependent Solver 1 in Oxygen Pulse/Solution 958 (sol958) ------------
Started at May 28, 2023, 4:28:18 PM.
Time-dependent solver (BDF)
Number of degrees of freedom solved for: 18918 (plus 37906 internal DOFs).
Symmetric matrices found.
Scales for dependent variables:
Concentration (comp2.Oxi): 0.23
Step        Time    Stepsize      Res   Jac   Sol Order Tfail NLfail   LinErr   LinRes
   0           0           - out    5     3     5                 0  7.5e-16  1.4e-15
   1  2.7154e-08  2.7154e-08        7     4     7     1     0     0  3.2e-16    1e-15
   2  5.4308e-08  2.7154e-08        9     5     9     1     0     0  3.4e-16  1.2e-15
   3  1.0862e-07  5.4308e-08       11     6    11     2     0     0  3.6e-16  1.3e-15
   4  2.1723e-07  1.0862e-07       13     7    13     2     0     0  4.6e-16  1.8e-15
   5  4.3446e-07  2.1723e-07       15     8    15     2     0     0  6.8e-16  3.6e-15
   6  6.5169e-07  2.1723e-07       17     9    17     2     0     0  5.7e-16  3.8e-15
   7  8.6893e-07  2.1723e-07       19    10    19     2     0     0  5.5e-16    4e-15
   8  1.0862e-06  2.1723e-07       21    11    21     2     0     0  6.1e-16    4e-15
   9  1.5206e-06  4.3446e-07       23    12    23     2     0     0    1e-15  6.4e-15
  10  1.9551e-06  4.3446e-07       25    13    25     2     0     0    8e-16    6e-15
  11  2.3895e-06  4.3446e-07       27    14    27     2     0     0  8.7e-16    6e-15
  12   2.824e-06  4.3446e-07       29    15    29     2     0     0  7.7e-16  5.7e-15
  13  3.6929e-06  8.6893e-07       31    16    31     2     0     0    9e-16  8.5e-15
  14  4.5619e-06  8.6893e-07       33    17    33     2     0     0    1e-15  7.5e-15
  15  5.4308e-06  8.6893e-07       35    18    35     2     0     0  1.3e-15  7.3e-15
  16  6.2997e-06  8.6893e-07       37    19    37     2     0     0    1e-15  6.6e-15
  17  8.0376e-06  1.7379e-06       39    20    39     2     0     0  1.5e-15  9.5e-15
  18  9.7754e-06  1.7379e-06       41    21    41     2     0     0  1.8e-15  8.2e-15
  19  1.1513e-05  1.7379e-06       43    22    43     2     0     0  1.6e-15  8.2e-15
  20  1.3251e-05  1.7379e-06       45    23    45     2     0     0  1.3e-15  7.4e-15
  21  1.4989e-05  1.7379e-06       47    24    47     2     0     0  1.7e-15  6.6e-15
  22  1.8465e-05  3.4757e-06       49    25    49     2     0     0  1.7e-15  9.9e-15
  23   2.194e-05  3.4757e-06       51    26    51     2     0     0  1.7e-15  8.5e-15
  24  2.5416e-05  3.4757e-06       53    27    53     2     0     0  1.5e-15  8.6e-15
  25  2.8892e-05  3.4757e-06       55    28    55     2     0     0  1.6e-15    8e-15
  26  3.5843e-05  6.9514e-06       57    29    57     2     0     0  1.5e-15  1.2e-14
  27  4.2795e-05  6.9514e-06       59    30    59     2     0     0  1.8e-15  9.8e-15
  28  4.9746e-05  6.9514e-06       61    31    61     2     0     0  1.4e-15  9.9e-15
  29  5.6697e-05  6.9514e-06       63    32    63     2     0     0  1.6e-15  9.2e-15
  30    7.06e-05  1.3903e-05       65    33    65     2     0     0  1.7e-15  1.3e-14
  31  8.4503e-05  1.3903e-05       67    34    67     2     0     0  1.7e-15  1.1e-14
  32  9.8406e-05  1.3903e-05       69    35    69     2     0     0  1.7e-15  1.1e-14
  33  0.00011231  1.3903e-05       71    36    71     2     0     0  1.9e-15    1e-14
  34  0.00014011  2.7806e-05       73    37    73     2     0     0  2.4e-15  1.5e-14
  35  0.00016792  2.7806e-05       75    38    75     2     0     0  2.2e-15  1.2e-14
  36  0.00019573  2.7806e-05       77    39    77     2     0     0  2.1e-15  1.2e-14
  37  0.00022353  2.7806e-05       79    40    79     2     0     0  1.9e-15  1.1e-14
  38  0.00027914  5.5611e-05       81    41    81     2     0     0  2.4e-15  1.6e-14
  39  0.00033475  5.5611e-05       83    42    83     2     0     0  2.5e-15  1.4e-14
  40  0.00039036  5.5611e-05       85    43    85     2     0     0  1.2e-15  1.4e-14
  41  0.00050159  0.00011122       87    44    87     2     0     0  2.7e-15  2.1e-14
  42  0.00061281  0.00011122       89    45    89     2     0     0  2.9e-15  1.7e-14
  43  0.00072403  0.00011122       91    46    91     2     0     0  1.4e-15  1.7e-14
  44  0.00083525  0.00011122       93    47    93     2     0     0  2.3e-15  1.5e-14
  45   0.0010577  0.00022245       95    48    95     2     0     0  2.1e-15  2.1e-14
  46   0.0012801  0.00022245       97    49    97     2     0     0  4.5e-15  1.6e-14
  47    0.001725  0.00044489       99    50    99     2     0     0  2.1e-15  2.7e-14
```

48	0.0021699	0.00044489		101	51	101	2	0	0	4.9e-15	2.4e-14
49	0.0026148	0.00044489		103	52	103	2	0	0	2.1e-15	2.3e-14
50	0.0030597	0.00044489		105	53	105	2	0	0	3.5e-15	2e-14
51	0.0039495	0.00088978		107	54	107	2	0	0	1.4e-15	2.3e-14
52	0.0048393	0.00088978		109	55	109	2	0	0	1.9e-15	1.7e-14
-	0.005	-	out								
53	0.0066188	0.0017796		111	56	111	2	0	0	2.8e-15	3e-14
54	0.0083984	0.0017796		113	57	113	2	0	0	2.5e-15	2.6e-14
-	0.01	-	out								
55	0.011958	0.0035591		115	58	115	2	0	0	8.9e-15	3.7e-14
-	0.015	-	out								
56	0.015517	0.0035591		117	59	117	1	0	0	6.6e-15	4.1e-14
57	0.019076	0.0035591		119	60	119	1	0	0	2.9e-15	2.4e-14
-	0.02	-	out								
-	0.025	-	out								
58	0.026194	0.0071182		121	61	121	1	0	0	6.7e-15	3.6e-14
-	0.03	-	out								
-	0.035	-	out								
-	0.04	-	out								
59	0.04043	0.014236		123	62	123	1	0	0	3.7e-15	5.2e-14
-	0.045	-	out								
-	0.05	-	out								
60	0.054667	0.014236		125	63	125	1	0	0	5.5e-15	5e-14
-	0.055	-	out								
-	0.06	-	out								
-	0.065	-	out								
-	0.07	-	out								
-	0.075	-	out								
-	0.08	-	out								
61	0.08314	0.028473		127	64	127	1	0	0	3.9e-15	7e-14
-	0.085	-	out								
-	0.09	-	out								
-	0.095	-	out								
-	0.1	-	out								
-	0.105	-	out								
-	0.11	-	out								
62	0.11161	0.028473		129	65	129	1	0	0	8e-15	6.3e-14
-	0.115	-	out								
-	0.12	-	out								
-	0.125	-	out								
-	0.13	-	out								
-	0.135	-	out								
-	0.14	-	out								
-	0.145	-	out								
-	0.15	-	out								
-	0.155	-	out								
-	0.16	-	out								
-	0.165	-	out								
63	0.16856	0.056946		131	66	131	1	0	0	2.9e-15	8.4e-14
-	0.17	-	out								
-	0.175	-	out								
-	0.18	-	out								
-	0.185	-	out								
-	0.19	-	out								
-	0.195	-	out								
-	0.2	-	out								

-	0.205	- out								
-	0.21	- out								
-	0.215	- out								
-	0.22	- out								
-	0.225	- out								
-	0.23	- out								
-	0.235	- out								
-	0.24	- out								
-	0.245	- out								
-	0.25	- out								
-	0.25	- out								
64	0.26756	0.099	133	67	133	1	0	0	7e-15	9.3e-14
-	0.27	- out								
-	0.29	- out								
-	0.31	- out								
-	0.33	- out								
-	0.35	- out								
65	0.36656	0.099	135	68	135	1	0	0	1.1e-14	7.1e-14
-	0.37	- out								
-	0.39	- out								
-	0.41	- out								
-	0.43	- out								
-	0.45	- out								
66	0.46556	0.099	137	69	137	1	0	0	3.9e-15	5.6e-14
-	0.47	- out								
-	0.49	- out								
-	0.51	- out								
-	0.53	- out								
-	0.55	- out								
67	0.56456	0.099	139	70	139	1	0	0	5.9e-15	4.3e-14
-	0.57	- out								
-	0.59	- out								
-	0.61	- out								
-	0.63	- out								
-	0.65	- out								
68	0.66356	0.099	141	71	141	1	0	0	1.1e-14	3.5e-14
-	0.67	- out								
-	0.69	- out								
-	0.71	- out								
-	0.73	- out								
-	0.75	- out								
69	0.76256	0.099	142	72	142	1	0	0	1.1e-14	2.7e-14
-	0.77	- out								
-	0.79	- out								
-	0.81	- out								
-	0.83	- out								
-	0.85	- out								
70	0.86156	0.099	143	73	143	1	0	0	6.9e-15	2.5e-14
-	0.87	- out								
-	0.89	- out								
-	0.91	- out								
-	0.93	- out								
-	0.95	- out								
71	0.96056	0.099	144	74	144	2	0	0	5.7e-15	2e-14
-	0.97	- out								
-	0.99	- out								

```
  72       1.0596       0.099      145    75   145     2      0       0    1e-14  1.2e-14
Time-stepping completed.
Solution time: 15 s.
Physical memory: 1.4 GB
Virtual memory: 1.38 GB
Ended at May 28, 2023, 4:28:33 PM.
----- Time-Dependent Solver 1 in Oxygen Pulse/Solution 958 (sol958) ----------->
```

Fully Coupled 1 (fc1)

GENERAL

Description	Value
Linear solver	Direct 1

METHOD AND TERMINATION

Description	Value
Damping factor	0.9
Jacobian update	Once per time step
Maximum number of iterations	8
Stabilization and acceleration	Anderson acceleration
Dimension of iteration space	5
Mixing parameter	0.9
Iteration delay	1

Direct 1 (d1)

GENERAL

Description	Value
Solver	PARDISO
Pivoting perturbation	1.0E-13

3.4.2 Parametric Solutions 4

k0Sub=0, d=5E-7 (su1)

GENERAL

Description	Value
Solution	k0Sub=0, d=5E-7

LOG

```
<---- Compile Equations: Stationary in Oxygen Pulse/Solution 958 (sol958) ------
Started at May 28, 2023, 4:02:07 PM.
Geometry shape function: Linear Lagrange
Running on Intel64 Family 6 Model 62 Stepping 4, GenuineIntel.
Using 1 socket with 4 cores in total on comsolsim4.
Available memory: 65.48 GB.
Parameter k0Sub = 0.0 (m/s).
Parameter d = 5.0E-7 (m).
Time: 1 s.
Physical memory: 917 MB
Virtual memory: 902 MB
Ended at May 28, 2023, 4:02:08 PM.
----- Compile Equations: Stationary in Oxygen Pulse/Solution 958 (sol958) ----->

<---- Dependent Variables 1 in Oxygen Pulse/Solution 958 (sol958) --------------
Started at May 28, 2023, 4:02:08 PM.
Solution time: 0 s.
Physical memory: 913 MB
Virtual memory: 898 MB
Ended at May 28, 2023, 4:02:08 PM.
----- Dependent Variables 1 in Oxygen Pulse/Solution 958 (sol958) ------------->

<---- Stationary Solver 1 in Oxygen Pulse/Solution 958 (sol958) ----------------
Started at May 28, 2023, 4:02:08 PM.
Linear solver
Number of degrees of freedom solved for: 17710 (plus 1007 internal DOFs).
Symmetric matrices found.
Scales for dependent variables:
Concentration (comp2.Oxi): 0.23
Orthonormal null-space function used.
Iter      SolEst     Damping    Stepsize #Res #Jac #Sol   LinErr   LinRes
   1     2.1e-16   1.0000000     2.1e-16    1    1    1  3.7e-14  2.9e-16
Solution time: 0 s.
Physical memory: 916 MB
Virtual memory: 901 MB
Ended at May 28, 2023, 4:02:09 PM.
----- Stationary Solver 1 in Oxygen Pulse/Solution 958 (sol958) --------------->

<---- Compile Equations: Oxygen Probe Pulse in Oxygen Pulse/Solution 958
      (sol958) ----------------------------------------------------------------
Started at May 28, 2023, 4:02:09 PM.
Geometry shape function: Linear Lagrange
Time: 1 s.
Physical memory: 914 MB
Virtual memory: 897 MB
Ended at May 28, 2023, 4:02:10 PM.
----- Compile Equations: Oxygen Probe Pulse in Oxygen Pulse/Solution 958
      (sol958) --------------------------------------------------------------->

<---- Dependent Variables 2 in Oxygen Pulse/Solution 958 (sol958) --------------
Started at May 28, 2023, 4:02:10 PM.
Initial values of variables solved for: Solution 958 (sol958), Solution Store 1 (sol
985).
Values of variables not solved for: Solution 958 (sol958), Solution Store 1 (sol985)
.
Solution time: 0 s.
```

Physical memory: 920 MB
Virtual memory: 903 MB
Ended at May 28, 2023, 4:02:10 PM.
----- Dependent Variables 2 in Oxygen Pulse/Solution 958 (sol958) ------------->

<---- Time-Dependent Solver 1 in Oxygen Pulse/Solution 958 (sol958) ------------
Started at May 28, 2023, 4:02:10 PM.
Time-dependent solver (BDF)
Number of degrees of freedom solved for: 17710 (plus 35580 internal DOFs).
Symmetric matrices found.
Scales for dependent variables:
Concentration (comp2.Oxi): 0.23

Step	Time	Stepsize	Res	Jac	Sol	Order	Tfail	NLfail	LinErr	LinRes
0	0	- out	5	3	5			0	9.7e-16	1.4e-15
1	2.5603e-08	2.5603e-08	7	4	7	1	0	0	3.7e-16	1.1e-15
2	5.1207e-08	2.5603e-08	9	5	9	1	0	0	3.4e-16	1.2e-15
3	1.0241e-07	5.1207e-08	11	6	11	2	0	0	3.9e-16	1.3e-15
4	2.0483e-07	1.0241e-07	13	7	13	2	0	0	4.3e-16	1.7e-15
5	4.0965e-07	2.0483e-07	15	8	15	2	0	0	5.6e-16	3.3e-15
6	6.1448e-07	2.0483e-07	17	9	17	2	0	0	6.3e-16	3.8e-15
7	8.1931e-07	2.0483e-07	19	10	19	2	0	0	7.5e-16	3.9e-15
8	1.0241e-06	2.0483e-07	21	11	21	2	0	0	6.7e-16	3.8e-15
9	1.4338e-06	4.0965e-07	23	12	23	2	0	0	8.9e-16	5.8e-15
10	1.8434e-06	4.0965e-07	25	13	25	2	0	0	7.9e-16	5.5e-15
11	2.2531e-06	4.0965e-07	27	14	27	2	0	0	7.4e-16	5.4e-15
12	2.6627e-06	4.0965e-07	29	15	29	2	0	0	7.3e-16	5.2e-15
13	3.482e-06	8.1931e-07	31	16	31	2	0	0	1.1e-15	7.5e-15
14	4.3014e-06	8.1931e-07	33	17	33	2	0	0	8.7e-16	6.4e-15
15	5.1207e-06	8.1931e-07	35	18	35	2	0	0	1.2e-15	6.4e-15
16	5.94e-06	8.1931e-07	37	19	37	2	0	0	1.1e-15	6e-15
17	7.5786e-06	1.6386e-06	39	20	39	2	0	0	1.4e-15	9e-15
18	9.2172e-06	1.6386e-06	41	21	41	2	0	0	1.2e-15	7.6e-15
19	1.0856e-05	1.6386e-06	43	22	43	2	0	0	1.2e-15	7.8e-15
20	1.2494e-05	1.6386e-06	45	23	45	2	0	0	1.1e-15	7.1e-15
21	1.4133e-05	1.6386e-06	47	24	47	2	0	0	8.9e-16	6.4e-15
22	1.741e-05	3.2772e-06	49	25	49	2	0	0	1.5e-15	9.3e-15
23	2.0687e-05	3.2772e-06	51	26	51	2	0	0	1.4e-15	8.5e-15
24	2.3965e-05	3.2772e-06	53	27	53	2	0	0	1.3e-15	8.4e-15
25	2.7242e-05	3.2772e-06	55	28	55	2	0	0	1.2e-15	7.6e-15
26	3.3796e-05	6.5544e-06	57	29	57	2	0	0	2.3e-15	1.2e-14
27	4.0351e-05	6.5544e-06	59	30	59	2	0	0	1.6e-15	1e-14
28	4.6905e-05	6.5544e-06	61	31	61	2	0	0	1.2e-15	9.9e-15
29	5.346e-05	6.5544e-06	63	32	63	2	0	0	1.8e-15	8.9e-15
30	6.0014e-05	6.5544e-06	65	33	65	2	0	0	1.7e-15	8.1e-15
31	7.3123e-05	1.3109e-05	67	34	67	2	0	0	1.9e-15	1.2e-14
32	8.6232e-05	1.3109e-05	69	35	69	2	0	0	1.6e-15	1.1e-14
33	9.9341e-05	1.3109e-05	71	36	71	2	0	0	1.5e-15	1.1e-14
34	0.00011245	1.3109e-05	73	37	73	2	0	0	1.8e-15	1.1e-14
35	0.00013867	2.6218e-05	75	38	75	2	0	0	2.2e-15	1.6e-14
36	0.00016489	2.6218e-05	77	39	77	2	0	0	1.8e-15	1.4e-14
37	0.0001911	2.6218e-05	79	40	79	2	0	0	2e-15	1.3e-14
38	0.00021732	2.6218e-05	81	41	81	2	0	0	2.4e-15	1.1e-14
39	0.00026976	5.2436e-05	83	42	83	2	0	0	3.3e-15	1.6e-14
40	0.00032219	5.2436e-05	85	43	85	2	0	0	2.4e-15	1.4e-14
41	0.00037463	5.2436e-05	87	44	87	2	0	0	3.5e-15	1.4e-14
42	0.0004795	0.00010487	89	45	89	2	0	0	1.5e-15	2.1e-14

43	0.00058437	0.00010487		91	46	91	2	0	0	2.1e-15	1.7e-14
44	0.00068924	0.00010487		93	47	93	2	0	0	1.7e-15	1.6e-14
45	0.00089898	0.00020974		95	48	95	2	0	0	2.3e-15	2.3e-14
46	0.0011087	0.00020974		97	49	97	2	0	0	5.6e-15	2e-14
47	0.0013185	0.00020974		99	50	99	2	0	0	1.8e-15	2.1e-14
48	0.0015282	0.00020974		101	51	101	2	0	0	3e-15	2.1e-14
49	0.0019477	0.00041948		103	52	103	2	0	0	5e-15	3e-14
50	0.0023672	0.00041948		105	53	105	2	0	0	1.4e-15	2.7e-14
51	0.0027867	0.00041948		107	54	107	2	0	0	2.4e-15	2.6e-14
52	0.0036256	0.00083897		109	55	109	2	0	0	4.5e-15	3.8e-14
53	0.0044646	0.00083897		111	56	111	2	0	0	3.3e-15	3.4e-14
-	0.005	-	out								
54	0.0053036	0.00083897		113	57	113	2	0	0	2.7e-15	3.6e-14
55	0.0069815	0.0016779		115	58	115	2	0	0	6.9e-15	5.1e-14
56	0.0086594	0.0016779		117	59	117	2	0	0	3.1e-15	4.3e-14
-	0.01	-	out								
57	0.012015	0.0033559		119	60	119	2	0	0	3.9e-15	6.8e-14
-	0.015	-	out								
58	0.015371	0.0033559		121	61	121	2	0	0	4.7e-15	4.9e-14
59	0.018727	0.0033559		123	62	123	2	0	0	3.9e-15	5e-14
-	0.02	-	out								
60	0.022083	0.0033559		125	63	125	2	0	0	1.6e-15	4.4e-14
-	0.025	-	out								
61	0.028795	0.0067118		127	64	127	2	0	0	5.4e-15	6.2e-14
-	0.03	-	out								
-	0.035	-	out								
62	0.035506	0.0067118		129	65	129	2	0	0	4e-15	5.3e-14
-	0.04	-	out								
-	0.045	-	out								
63	0.04893	0.013424		131	66	131	2	0	0	8.3e-15	8.2e-14
-	0.05	-	out								
-	0.055	-	out								
-	0.06	-	out								
64	0.062353	0.013424		133	67	133	1	0	0	8e-15	7.4e-14
-	0.065	-	out								
-	0.07	-	out								
-	0.075	-	out								
65	0.075777	0.013424		135	68	135	1	0	0	5.7e-15	2.8e-14
-	0.08	-	out								
-	0.085	-	out								
-	0.09	-	out								
-	0.095	-	out								
-	0.1	-	out								
66	0.10262	0.026847		137	69	137	1	0	0	4.8e-15	4e-14
-	0.105	-	out								
-	0.11	-	out								
-	0.115	-	out								
-	0.12	-	out								
-	0.125	-	out								
-	0.13	-	out								
-	0.135	-	out								
-	0.14	-	out								
-	0.145	-	out								
-	0.15	-	out								
-	0.155	-	out								
67	0.15632	0.053694		139	70	139	1	0	0	1.2e-14	5.2e-14

-	0.16	-	out							
-	0.165	-	out							
-	0.17	-	out							
-	0.175	-	out							
-	0.18	-	out							
-	0.185	-	out							
-	0.19	-	out							
-	0.195	-	out							
-	0.2	-	out							
-	0.205	-	out							
-	0.21	-	out							
-	0.215	-	out							
-	0.22	-	out							
-	0.225	-	out							
-	0.23	-	out							
-	0.235	-	out							
-	0.24	-	out							
-	0.245	-	out							
-	0.25	-	out							
-	0.25	-	out							
68	0.25532	0.099	141	71	141	1	0	0	4.3e-15	5.6e-14
-	0.27	-	out							
-	0.29	-	out							
-	0.31	-	out							
-	0.33	-	out							
-	0.35	-	out							
69	0.35432	0.099	143	72	143	1	0	0	8.9e-15	4.6e-14
-	0.37	-	out							
-	0.39	-	out							
-	0.41	-	out							
-	0.43	-	out							
-	0.45	-	out							
70	0.45332	0.099	145	73	145	1	0	0	2.3e-14	3.4e-14
-	0.47	-	out							
-	0.49	-	out							
-	0.51	-	out							
-	0.53	-	out							
-	0.55	-	out							
71	0.55232	0.099	147	74	147	1	0	0	1.7e-14	2.8e-14
-	0.57	-	out							
-	0.59	-	out							
-	0.61	-	out							
-	0.63	-	out							
-	0.65	-	out							
72	0.65132	0.099	148	75	148	1	0	0	7.1e-15	2.3e-14
-	0.67	-	out							
-	0.69	-	out							
-	0.71	-	out							
-	0.73	-	out							
-	0.75	-	out							
73	0.75032	0.099	149	76	149	1	0	0	9.3e-15	2e-14
-	0.77	-	out							
-	0.79	-	out							
-	0.81	-	out							
-	0.83	-	out							
74	0.84932	0.099	150	77	150	2	0	0	2.2e-15	1.5e-14

```
   -        0.85           - out
   -        0.87           - out
   -        0.89           - out
   -        0.91           - out
   -        0.93           - out
  75     0.94832        0.099      151    78  151      2      0       0  3.3e-15  1.1e-14
   -        0.95           - out
   -        0.97           - out
   -        0.99           - out
  76      1.0473        0.099      152    79  152      2      0       0  2.7e-15    1e-14
Time-stepping completed.
Solution time: 14 s.
Physical memory: 967 MB
Virtual memory: 954 MB
Ended at May 28, 2023, 4:02:24 PM.
----- Time-Dependent Solver 1 in Oxygen Pulse/Solution 958 (sol958) ----------->
```

k0Sub=0, d=1E-6 (su2)

GENERAL

Description	Value
Solution	k0Sub=0, d=1E-6

LOG

```
<---- Compile Equations: Stationary in Oxygen Pulse/Solution 958 (sol958) ------
Started at May 28, 2023, 4:02:27 PM.
Parameter k0Sub = 0.0 (m/s).
Parameter d = 1.0E-6 (m).
Geometry shape function: Linear Lagrange
Number of vertex elements: 10
Number of boundary elements: 899
Number of elements: 34599
Minimum element quality: 0.4361
Number of vertex elements: 10
Minimum element quality: 0.4361
Time: 2 s.
Physical memory: 1 GB
Virtual memory: 0.98 GB
Ended at May 28, 2023, 4:02:29 PM.
----- Compile Equations: Stationary in Oxygen Pulse/Solution 958 (sol958) ----->

<---- Dependent Variables 1 in Oxygen Pulse/Solution 958 (sol958) --------------
Started at May 28, 2023, 4:02:29 PM.
Solution time: 0 s.
Physical memory: 1 GB
Virtual memory: 0.98 GB
Ended at May 28, 2023, 4:02:29 PM.
----- Dependent Variables 1 in Oxygen Pulse/Solution 958 (sol958) ------------->

<---- Stationary Solver 1 in Oxygen Pulse/Solution 958 (sol958) ----------------
Started at May 28, 2023, 4:02:30 PM.
Linear solver
Number of degrees of freedom solved for: 17724 (plus 963 internal DOFs).
Symmetric matrices found.
Scales for dependent variables:
Concentration (comp2.Oxi): 0.23
Orthonormal null-space function used.
Iter      SolEst     Damping    Stepsize #Res #Jac #Sol   LinErr   LinRes
   1     2.1e-16   1.0000000     2.1e-16    1    1    1  3.7e-14    3e-16
Solution time: 0 s.
Physical memory: 1.01 GB
Virtual memory: 0.98 GB
Ended at May 28, 2023, 4:02:30 PM.
----- Stationary Solver 1 in Oxygen Pulse/Solution 958 (sol958) --------------->

<---- Compile Equations: Oxygen Probe Pulse in Oxygen Pulse/Solution 958
      (sol958) ---------------------------------------------------------------
Started at May 28, 2023, 4:02:30 PM.
Geometry shape function: Linear Lagrange
Time: 1 s.
Physical memory: 1.01 GB
Virtual memory: 0.98 GB
Ended at May 28, 2023, 4:02:31 PM.
----- Compile Equations: Oxygen Probe Pulse in Oxygen Pulse/Solution 958
      (sol958) -------------------------------------------------------------->

<---- Dependent Variables 2 in Oxygen Pulse/Solution 958 (sol958) --------------
Started at May 28, 2023, 4:02:31 PM.
Initial values of variables solved for: Solution 958 (sol958), Solution Store 1 (sol
985).
```

Values of variables not solved for: Solution 958 (sol958), Solution Store 1 (sol985)
.
Solution time: 0 s.
Physical memory: 1.01 GB
Virtual memory: 0.98 GB
Ended at May 28, 2023, 4:02:31 PM.
----- Dependent Variables 2 in Oxygen Pulse/Solution 958 (sol958) ------------->

<---- Time-Dependent Solver 1 in Oxygen Pulse/Solution 958 (sol958) ------------
Started at May 28, 2023, 4:02:31 PM.
Time-dependent solver (BDF)
Number of degrees of freedom solved for: 17724 (plus 35562 internal DOFs).
Symmetric matrices found.
Scales for dependent variables:
Concentration (comp2.Oxi): 0.23

Step	Time	Stepsize	Res	Jac	Sol	Order	Tfail	NLfail	LinErr	LinRes
0	0	- out	5	3	5			0	6.8e-16	1.4e-15
1	2.5901e-08	2.5901e-08	7	4	7	1	0	0	3.3e-16	1.1e-15
2	5.1802e-08	2.5901e-08	9	5	9	1	0	0	3.6e-16	1.2e-15
3	1.036e-07	5.1802e-08	11	6	11	2	0	0	4.8e-16	1.3e-15
4	2.0721e-07	1.036e-07	13	7	13	2	0	0	4.7e-16	1.7e-15
5	4.1442e-07	2.0721e-07	15	8	15	2	0	0	5.3e-16	3.3e-15
6	6.2163e-07	2.0721e-07	17	9	17	2	0	0	6.4e-16	3.7e-15
7	8.2884e-07	2.0721e-07	19	10	19	2	0	0	8.4e-16	4e-15
8	1.036e-06	2.0721e-07	21	11	21	2	0	0	7.9e-16	4e-15
9	1.4505e-06	4.1442e-07	23	12	23	2	0	0	8.2e-16	6.4e-15
10	1.8649e-06	4.1442e-07	25	13	25	2	0	0	9.3e-16	6.1e-15
11	2.2793e-06	4.1442e-07	27	14	27	2	0	0	7.7e-16	5.7e-15
12	2.6937e-06	4.1442e-07	29	15	29	2	0	0	8.2e-16	5.3e-15
13	3.5226e-06	8.2884e-07	31	16	31	2	0	0	9.2e-16	8.1e-15
14	4.3514e-06	8.2884e-07	33	17	33	2	0	0	1.3e-15	7e-15
15	5.1802e-06	8.2884e-07	35	18	35	2	0	0	8.5e-16	7e-15
16	6.0091e-06	8.2884e-07	37	19	37	2	0	0	1.1e-15	6.2e-15
17	7.6668e-06	1.6577e-06	39	20	39	2	0	0	1.1e-15	9.4e-15
18	9.3244e-06	1.6577e-06	41	21	41	2	0	0	1.2e-15	7.8e-15
19	1.0982e-05	1.6577e-06	43	22	43	2	0	0	1.1e-15	7.9e-15
20	1.264e-05	1.6577e-06	45	23	45	2	0	0	9.3e-16	6.8e-15
21	1.4297e-05	1.6577e-06	47	24	47	2	0	0	1e-15	6.2e-15
22	1.7613e-05	3.3154e-06	49	25	49	2	0	0	1.7e-15	9.1e-15
23	2.0928e-05	3.3154e-06	51	26	51	2	0	0	1.2e-15	8e-15
24	2.4244e-05	3.3154e-06	53	27	53	2	0	0	1.6e-15	8e-15
25	2.7559e-05	3.3154e-06	55	28	55	2	0	0	1.4e-15	7.2e-15
26	3.419e-05	6.6307e-06	57	29	57	2	0	0	1.2e-15	1.1e-14
27	4.082e-05	6.6307e-06	59	30	59	2	0	0	1.2e-15	9e-15
28	4.7451e-05	6.6307e-06	61	31	61	2	0	0	1.6e-15	9e-15
29	5.4082e-05	6.6307e-06	63	32	63	2	0	0	1.3e-15	8.4e-15
30	6.0713e-05	6.6307e-06	65	33	65	2	0	0	2.2e-15	7.6e-15
31	7.3974e-05	1.3261e-05	67	34	67	2	0	0	2.8e-15	1.1e-14
32	8.7235e-05	1.3261e-05	69	35	69	2	0	0	2.5e-15	1e-14
33	0.0001005	1.3261e-05	71	36	71	2	0	0	2.3e-15	1e-14
34	0.00011376	1.3261e-05	73	37	73	2	0	0	1.4e-15	9.5e-15
35	0.00014028	2.6523e-05	75	38	75	2	0	0	2.1e-15	1.4e-14
36	0.0001668	2.6523e-05	77	39	77	2	0	0	2.4e-15	1.2e-14
37	0.00019333	2.6523e-05	79	40	79	2	0	0	1.9e-15	1.2e-14
38	0.00021985	2.6523e-05	81	41	81	2	0	0	1.3e-15	1.1e-14
39	0.0002729	5.3046e-05	83	42	83	2	0	0	2.6e-15	1.6e-14

40	0.00032594	5.3046e-05		85	43	85	2	0	0	3e-15	1.3e-14
41	0.00037899	5.3046e-05		87	44	87	2	0	0	2e-15	1.3e-14
42	0.00048508	0.00010609		89	45	89	2	0	0	3.1e-15	1.9e-14
43	0.00059117	0.00010609		91	46	91	2	0	0	5.5e-15	1.6e-14
44	0.00069726	0.00010609		93	47	93	2	0	0	2.3e-15	1.6e-14
45	0.00080335	0.00010609		95	48	95	2	0	0	2.7e-15	1.4e-14
46	0.0010155	0.00021218		97	49	97	2	0	0	2.7e-15	2e-14
47	0.0012277	0.00021218		99	50	99	2	0	0	3.4e-15	1.7e-14
48	0.0016521	0.00042437		101	51	101	2	0	0	2.4e-15	2.8e-14
49	0.0020765	0.00042437		103	52	103	2	0	0	3e-15	2.5e-14
50	0.0025008	0.00042437		105	53	105	2	0	0	3.1e-15	2.6e-14
51	0.0029252	0.00042437		107	54	107	2	0	0	3.8e-15	2.3e-14
52	0.0037739	0.00084873		109	55	109	2	0	0	2.9e-15	3.2e-14
53	0.0046226	0.00084873		111	56	111	2	0	0	3.1e-15	2.8e-14
-	0.005	-	out								
54	0.0063201	0.0016975		113	57	113	2	0	0	4.6e-15	4.4e-14
55	0.0080176	0.0016975		115	58	115	2	0	0	3.1e-15	4.1e-14
56	0.009715	0.0016975		117	59	117	2	0	0	3.4e-15	4.4e-14
-	0.01	-	out								
57	0.011413	0.0016975		119	60	119	2	0	0	2.5e-15	3.9e-14
58	0.014807	0.0033949		121	61	121	2	0	0	8.1e-15	4.7e-14
-	0.015	-	out								
59	0.018202	0.0033949		123	62	123	2	0	0	4.4e-15	3.6e-14
-	0.02	-	out								
60	0.024992	0.0067899		125	63	125	2	0	0	2.6e-15	6.3e-14
-	0.025	-	out								
-	0.03	-	out								
61	0.031782	0.0067899		127	64	127	2	0	0	9.8e-15	5.7e-14
-	0.035	-	out								
62	0.038572	0.0067899		129	65	129	2	0	0	7.3e-15	5.7e-14
-	0.04	-	out								
-	0.045	-	out								
63	0.045362	0.0067899		131	66	131	2	0	0	7e-15	5.3e-14
-	0.05	-	out								
-	0.055	-	out								
64	0.058942	0.01358		133	67	133	2	0	0	3.2e-15	6.6e-14
-	0.06	-	out								
-	0.065	-	out								
-	0.07	-	out								
65	0.072521	0.01358		135	68	135	2	0	0	6.3e-15	4.3e-14
-	0.075	-	out								
-	0.08	-	out								
-	0.085	-	out								
-	0.09	-	out								
-	0.095	-	out								
66	0.099681	0.027159		137	69	137	2	0	0	4.5e-15	5.9e-14
-	0.1	-	out								
-	0.105	-	out								
-	0.11	-	out								
-	0.115	-	out								
-	0.12	-	out								
-	0.125	-	out								
-	0.13	-	out								
-	0.135	-	out								
-	0.14	-	out								
-	0.145	-	out								

-	0.15	-	out							
67	0.154	0.054319	139	70	139	1	0	0	1.5e-14	5.9e-14
-	0.155	-	out							
-	0.16	-	out							
-	0.165	-	out							
-	0.17	-	out							
-	0.175	-	out							
-	0.18	-	out							
-	0.185	-	out							
-	0.19	-	out							
-	0.195	-	out							
-	0.2	-	out							
-	0.205	-	out							
68	0.20832	0.054319	141	71	141	1	0	0	3.8e-15	3e-14
-	0.21	-	out							
-	0.215	-	out							
-	0.22	-	out							
-	0.225	-	out							
-	0.23	-	out							
-	0.235	-	out							
-	0.24	-	out							
-	0.245	-	out							
-	0.25	-	out							
-	0.25	-	out							
-	0.27	-	out							
-	0.29	-	out							
69	0.30732	0.099	143	72	143	1	0	0	2.1e-14	3.7e-14
-	0.31	-	out							
-	0.33	-	out							
-	0.35	-	out							
-	0.37	-	out							
-	0.39	-	out							
70	0.40632	0.099	145	73	145	1	0	0	1e-14	3.3e-14
-	0.41	-	out							
-	0.43	-	out							
-	0.45	-	out							
-	0.47	-	out							
-	0.49	-	out							
71	0.50532	0.099	147	74	147	1	0	0	8.9e-15	2.7e-14
-	0.51	-	out							
-	0.53	-	out							
-	0.55	-	out							
-	0.57	-	out							
-	0.59	-	out							
72	0.60432	0.099	149	75	149	1	0	0	1.3e-14	2.4e-14
-	0.61	-	out							
-	0.63	-	out							
-	0.65	-	out							
-	0.67	-	out							
-	0.69	-	out							
73	0.70332	0.099	150	76	150	1	0	0	4.7e-15	2.1e-14
-	0.71	-	out							
-	0.73	-	out							
-	0.75	-	out							
-	0.77	-	out							
-	0.79	-	out							

```
  74      0.80232        0.099      151    77  151      1      0       0    9e-15  1.7e-14
   -         0.81            - out
   -         0.83            - out
   -         0.85            - out
   -         0.87            - out
   -         0.89            - out
  75      0.90132        0.099      152    78  152      2      0       0  1.8e-14  1.5e-14
   -         0.91            - out
   -         0.93            - out
   -         0.95            - out
   -         0.97            - out
   -         0.99            - out
  76       1.0003        0.099      153    79  153      2      0       0  3.9e-15  9.2e-15
Time-stepping completed.
Solution time: 13 s.
Physical memory: 1.02 GB
Virtual memory: 1 GB
Ended at May 28, 2023, 4:02:44 PM.
----- Time-Dependent Solver 1 in Oxygen Pulse/Solution 958 (sol958) ----------->
```

k0Sub=0, d=1.5E-6 (su3)

GENERAL

Description	Value
Solution	k0Sub=0, d=1.5E-6

LOG

```
<---- Compile Equations: Stationary in Oxygen Pulse/Solution 958 (sol958) ------
Started at May 28, 2023, 4:02:47 PM.
Parameter k0Sub = 0.0 (m/s).
Parameter d = 1.5E-6 (m).
Geometry shape function: Linear Lagrange
Number of vertex elements: 10
Number of boundary elements: 894
Number of elements: 34794
Minimum element quality: 0.395
Number of vertex elements: 10
Minimum element quality: 0.395
Time: 2 s.
Physical memory: 1.05 GB
Virtual memory: 1.02 GB
Ended at May 28, 2023, 4:02:49 PM.
----- Compile Equations: Stationary in Oxygen Pulse/Solution 958 (sol958) ----->

<---- Dependent Variables 1 in Oxygen Pulse/Solution 958 (sol958) --------------
Started at May 28, 2023, 4:02:49 PM.
Solution time: 0 s.
Physical memory: 1.05 GB
Virtual memory: 1.02 GB
Ended at May 28, 2023, 4:02:49 PM.
----- Dependent Variables 1 in Oxygen Pulse/Solution 958 (sol958) ------------->

<---- Stationary Solver 1 in Oxygen Pulse/Solution 958 (sol958) ----------------
Started at May 28, 2023, 4:02:49 PM.
Linear solver
Number of degrees of freedom solved for: 17823 (plus 950 internal DOFs).
Symmetric matrices found.
Scales for dependent variables:
Concentration (comp2.Oxi): 0.23
Orthonormal null-space function used.
Iter      SolEst     Damping    Stepsize #Res #Jac #Sol   LinErr   LinRes
   1     2.3e-16   1.0000000     2.3e-16    1    1    1  2.8e-14    3e-16
Solution time: 0 s.
Physical memory: 1.05 GB
Virtual memory: 1.03 GB
Ended at May 28, 2023, 4:02:50 PM.
----- Stationary Solver 1 in Oxygen Pulse/Solution 958 (sol958) --------------->

<---- Compile Equations: Oxygen Probe Pulse in Oxygen Pulse/Solution 958
      (sol958) -----------------------------------------------------------------
Started at May 28, 2023, 4:02:50 PM.
Geometry shape function: Linear Lagrange
Time: 1 s.
Physical memory: 1.06 GB
Virtual memory: 1.03 GB
Ended at May 28, 2023, 4:02:51 PM.
----- Compile Equations: Oxygen Probe Pulse in Oxygen Pulse/Solution 958
      (sol958) ---------------------------------------------------------------->

<---- Dependent Variables 2 in Oxygen Pulse/Solution 958 (sol958) --------------
Started at May 28, 2023, 4:02:51 PM.
Initial values of variables solved for: Solution 958 (sol958), Solution Store 1 (sol
985).
```

Values of variables not solved for: Solution 958 (sol958), Solution Store 1 (sol985)
.
Solution time: 0 s.
Physical memory: 1.06 GB
Virtual memory: 1.03 GB
Ended at May 28, 2023, 4:02:51 PM.
----- Dependent Variables 2 in Oxygen Pulse/Solution 958 (sol958) ------------->

<---- Time-Dependent Solver 1 in Oxygen Pulse/Solution 958 (sol958) ------------
Started at May 28, 2023, 4:02:51 PM.
Time-dependent solver (BDF)
Number of degrees of freedom solved for: 17823 (plus 35744 internal DOFs).
Symmetric matrices found.
Scales for dependent variables:
Concentration (comp2.Oxi): 0.23

Step	Time	Stepsize	Res	Jac	Sol	Order	Tfail	NLfail	LinErr	LinRes
0	0	- out	5	3	5			0	8.3e-16	1.4e-15
1	2.5748e-08	2.5748e-08	7	4	7	1	0	0	3.3e-16	1e-15
2	5.1496e-08	2.5748e-08	9	5	9	1	0	0	3.6e-16	1.1e-15
3	1.0299e-07	5.1496e-08	11	6	11	2	0	0	3.7e-16	1.3e-15
4	2.0598e-07	1.0299e-07	13	7	13	2	0	0	4.4e-16	1.7e-15
5	4.1197e-07	2.0598e-07	15	8	15	2	0	0	7.4e-16	3.4e-15
6	6.1795e-07	2.0598e-07	17	9	17	2	0	0	5.4e-16	3.7e-15
7	8.2394e-07	2.0598e-07	19	10	19	2	0	0	5.4e-16	4e-15
8	1.0299e-06	2.0598e-07	21	11	21	2	0	0	5.6e-16	3.9e-15
9	1.4419e-06	4.1197e-07	23	12	23	2	0	0	8.2e-16	6.2e-15
10	1.8539e-06	4.1197e-07	25	13	25	2	0	0	9.9e-16	6.1e-15
11	2.2658e-06	4.1197e-07	27	14	27	2	0	0	7.5e-16	6e-15
12	2.6778e-06	4.1197e-07	29	15	29	2	0	0	9.9e-16	5.6e-15
13	3.5017e-06	8.2394e-07	31	16	31	2	0	0	1.2e-15	8.1e-15
14	4.3257e-06	8.2394e-07	33	17	33	2	0	0	1.3e-15	6.9e-15
15	5.1496e-06	8.2394e-07	35	18	35	2	0	0	1.2e-15	7.1e-15
16	5.9735e-06	8.2394e-07	37	19	37	2	0	0	9.4e-16	6.2e-15
17	7.6214e-06	1.6479e-06	39	20	39	2	0	0	1.4e-15	9.1e-15
18	9.2693e-06	1.6479e-06	41	21	41	2	0	0	1.4e-15	7.9e-15
19	1.0917e-05	1.6479e-06	43	22	43	2	0	0	1.8e-15	8e-15
20	1.2565e-05	1.6479e-06	45	23	45	2	0	0	1.5e-15	7.2e-15
21	1.4213e-05	1.6479e-06	47	24	47	2	0	0	1.5e-15	6.7e-15
22	1.7509e-05	3.2957e-06	49	25	49	2	0	0	1.5e-15	9.5e-15
23	2.0804e-05	3.2957e-06	51	26	51	2	0	0	1.6e-15	7.9e-15
24	2.41e-05	3.2957e-06	53	27	53	2	0	0	2.1e-15	7.7e-15
25	2.7396e-05	3.2957e-06	55	28	55	2	0	0	1.8e-15	7.3e-15
26	3.3987e-05	6.5915e-06	57	29	57	2	0	0	2.5e-15	1e-14
27	4.0579e-05	6.5915e-06	59	30	59	2	0	0	2.3e-15	9.3e-15
28	4.717e-05	6.5915e-06	61	31	61	2	0	0	2.2e-15	9.3e-15
29	5.3762e-05	6.5915e-06	63	32	63	2	0	0	1.6e-15	8.6e-15
30	6.0353e-05	6.5915e-06	65	33	65	2	0	0	1.4e-15	7.4e-15
31	7.3536e-05	1.3183e-05	67	34	67	2	0	0	2.8e-15	1.1e-14
32	8.6719e-05	1.3183e-05	69	35	69	2	0	0	1.1e-15	9.6e-15
33	9.9902e-05	1.3183e-05	71	36	71	2	0	0	1.5e-15	9.6e-15
34	0.00011309	1.3183e-05	73	37	73	2	0	0	2.6e-15	9e-15
35	0.00013945	2.6366e-05	75	38	75	2	0	0	2e-15	1.3e-14
36	0.00016582	2.6366e-05	77	39	77	2	0	0	3.2e-15	1.1e-14
37	0.00019218	2.6366e-05	79	40	79	2	0	0	2.4e-15	1.2e-14
38	0.00021855	2.6366e-05	81	41	81	2	0	0	2.8e-15	1.1e-14
39	0.00027128	5.2732e-05	83	42	83	2	0	0	3e-15	1.5e-14

40	0.00032401	5.2732e-05	85	43	85	2	0	0	2.1e-15	1.3e-14
41	0.00037675	5.2732e-05	87	44	87	2	0	0	2.4e-15	1.3e-14
42	0.00048221	0.00010546	89	45	89	2	0	0	3.5e-15	1.9e-14
43	0.00058767	0.00010546	91	46	91	2	0	0	3.2e-15	1.6e-14
44	0.00069314	0.00010546	93	47	93	2	0	0	2.5e-15	1.6e-14
45	0.0007986	0.00010546	95	48	95	2	0	0	1.8e-15	1.4e-14
46	0.0010095	0.00021093	97	49	97	2	0	0	3.5e-15	2e-14
47	0.0012205	0.00021093	99	50	99	2	0	0	2e-15	1.7e-14
48	0.0016423	0.00042186	101	51	101	2	0	0	4.4e-15	2.8e-14
49	0.0020642	0.00042186	103	52	103	2	0	0	2.1e-15	2.5e-14
50	0.002486	0.00042186	105	53	105	2	0	0	3.7e-15	2.4e-14
51	0.0033297	0.00084371	107	54	107	2	0	0	6.3e-15	3.3e-14
52	0.0041734	0.00084371	109	55	109	2	0	0	3.3e-15	2.6e-14
-	0.005	-	out							
53	0.0050172	0.00084371	111	56	111	2	0	0	6.4e-15	2.8e-14
54	0.0067046	0.0016874	113	57	113	2	0	0	3.8e-15	4.4e-14
55	0.008392	0.0016874	115	58	115	2	0	0	3e-15	3.7e-14
-	0.01	-	out							
56	0.011767	0.0033748	117	59	117	2	0	0	4.7e-15	5.7e-14
-	0.015	-	out							
57	0.015142	0.0033748	119	60	119	2	0	0	5.1e-15	4.9e-14
58	0.018517	0.0033748	121	61	121	2	0	0	5.3e-15	4.8e-14
-	0.02	-	out							
59	0.021891	0.0033748	123	62	123	2	0	0	4.7e-15	4e-14
-	0.025	-	out							
60	0.028641	0.0067497	125	63	125	2	0	0	6.1e-15	5.6e-14
-	0.03	-	out							
-	0.035	-	out							
61	0.035391	0.0067497	127	64	127	2	0	0	2.8e-15	4.5e-14
-	0.04	-	out							
-	0.045	-	out							
62	0.04889	0.013499	129	65	129	2	0	0	2.6e-15	7.8e-14
-	0.05	-	out							
-	0.055	-	out							
-	0.06	-	out							
63	0.06239	0.013499	131	66	131	2	0	0	9.1e-15	6.3e-14
-	0.065	-	out							
-	0.07	-	out							
-	0.075	-	out							
64	0.075889	0.013499	133	67	133	2	0	0	7.7e-15	6e-14
-	0.08	-	out							
-	0.085	-	out							
-	0.09	-	out							
-	0.095	-	out							
-	0.1	-	out							
65	0.10289	0.026999	135	68	135	2	0	0	6.7e-15	6.6e-14
-	0.105	-	out							
-	0.11	-	out							
-	0.115	-	out							
-	0.12	-	out							
-	0.125	-	out							
-	0.13	-	out							
-	0.135	-	out							
-	0.14	-	out							
-	0.145	-	out							
-	0.15	-	out							

-	0.155	- out								
66	0.15689	0.053998	137	69	137	1	0	0	4.5e-15	5.5e-14
-	0.16	- out								
-	0.165	- out								
-	0.17	- out								
-	0.175	- out								
-	0.18	- out								
-	0.185	- out								
-	0.19	- out								
-	0.195	- out								
-	0.2	- out								
-	0.205	- out								
-	0.21	- out								
67	0.21088	0.053998	139	70	139	1	0	0	1.6e-15	3.2e-14
-	0.215	- out								
-	0.22	- out								
-	0.225	- out								
-	0.23	- out								
-	0.235	- out								
-	0.24	- out								
-	0.245	- out								
-	0.25	- out								
-	0.25	- out								
-	0.27	- out								
-	0.29	- out								
68	0.30988	0.099	141	71	141	1	0	0	6e-15	4e-14
-	0.31	- out								
-	0.33	- out								
-	0.35	- out								
-	0.37	- out								
-	0.39	- out								
69	0.40888	0.099	143	72	143	1	0	0	2.4e-14	3.5e-14
-	0.41	- out								
-	0.43	- out								
-	0.45	- out								
-	0.47	- out								
-	0.49	- out								
70	0.50788	0.099	145	73	145	1	0	0	7.4e-15	2.8e-14
-	0.51	- out								
-	0.53	- out								
-	0.55	- out								
-	0.57	- out								
-	0.59	- out								
71	0.60688	0.099	147	74	147	1	0	0	7e-15	2.5e-14
-	0.61	- out								
-	0.63	- out								
-	0.65	- out								
-	0.67	- out								
-	0.69	- out								
72	0.70588	0.099	148	75	148	1	0	0	7.1e-15	2e-14
-	0.71	- out								
-	0.73	- out								
-	0.75	- out								
-	0.77	- out								
-	0.79	- out								
73	0.80488	0.099	149	76	149	1	0	0	7.5e-15	1.9e-14

```
   -        0.81          - out
   -        0.83          - out
   -        0.85          - out
   -        0.87          - out
   -        0.89          - out
  74     0.90388       0.099      150    77   150      2      0       0   5.5e-15   1.5e-14
   -        0.91          - out
   -        0.93          - out
   -        0.95          - out
   -        0.97          - out
   -        0.99          - out
  75      1.0029       0.099      151    78   151      2      0       0   5.2e-15   9.9e-15
Time-stepping completed.
Solution time: 13 s.
Physical memory: 1.07 GB
Virtual memory: 1.05 GB
Ended at May 28, 2023, 4:03:03 PM.
----- Time-Dependent Solver 1 in Oxygen Pulse/Solution 958 (sol958) ----------->
```

k0Sub=0, d=2E-6 (su4)

GENERAL

Description	Value
Solution	k0Sub=0, d=2E-6

LOG

```
<---- Compile Equations: Stationary in Oxygen Pulse/Solution 958 (sol958) ------
Started at May 28, 2023, 4:03:06 PM.
Parameter k0Sub = 0.0 (m/s).
Parameter d = 2.0E-6 (m).
Geometry shape function: Linear Lagrange
Number of vertex elements: 10
Number of boundary elements: 889
Number of elements: 34960
Minimum element quality: 0.4112
Number of vertex elements: 10
Minimum element quality: 0.4112
Time: 2 s.
Physical memory: 1.06 GB
Virtual memory: 1.04 GB
Ended at May 28, 2023, 4:03:08 PM.
----- Compile Equations: Stationary in Oxygen Pulse/Solution 958 (sol958) ----->

<---- Dependent Variables 1 in Oxygen Pulse/Solution 958 (sol958) --------------
Started at May 28, 2023, 4:03:08 PM.
Solution time: 0 s.
Physical memory: 1.06 GB
Virtual memory: 1.03 GB
Ended at May 28, 2023, 4:03:08 PM.
----- Dependent Variables 1 in Oxygen Pulse/Solution 958 (sol958) ------------->

<---- Stationary Solver 1 in Oxygen Pulse/Solution 958 (sol958) ----------------
Started at May 28, 2023, 4:03:08 PM.
Linear solver
Number of degrees of freedom solved for: 17907 (plus 938 internal DOFs).
Symmetric matrices found.
Scales for dependent variables:
Concentration (comp2.Oxi): 0.23
Orthonormal null-space function used.
Iter      SolEst     Damping    Stepsize #Res #Jac #Sol   LinErr   LinRes
   1     2.5e-16   1.0000000     2.5e-16    1    1    1  7.2e-14    3e-16
Solution time: 0 s.
Physical memory: 1.07 GB
Virtual memory: 1.04 GB
Ended at May 28, 2023, 4:03:09 PM.
----- Stationary Solver 1 in Oxygen Pulse/Solution 958 (sol958) --------------->

<---- Compile Equations: Oxygen Probe Pulse in Oxygen Pulse/Solution 958
      (sol958) -----------------------------------------------------------------
Started at May 28, 2023, 4:03:09 PM.
Geometry shape function: Linear Lagrange
Time: 1 s.
Physical memory: 1.07 GB
Virtual memory: 1.05 GB
Ended at May 28, 2023, 4:03:10 PM.
----- Compile Equations: Oxygen Probe Pulse in Oxygen Pulse/Solution 958
      (sol958) ---------------------------------------------------------------->

<---- Dependent Variables 2 in Oxygen Pulse/Solution 958 (sol958) --------------
Started at May 28, 2023, 4:03:10 PM.
Initial values of variables solved for: Solution 958 (sol958), Solution Store 1 (sol
985).
```

Values of variables not solved for: Solution 958 (sol958), Solution Store 1 (sol985)
.
Solution time: 0 s.
Physical memory: 1.07 GB
Virtual memory: 1.05 GB
Ended at May 28, 2023, 4:03:10 PM.
----- Dependent Variables 2 in Oxygen Pulse/Solution 958 (sol958) ------------->

<---- Time-Dependent Solver 1 in Oxygen Pulse/Solution 958 (sol958) ------------
Started at May 28, 2023, 4:03:10 PM.
Time-dependent solver (BDF)
Number of degrees of freedom solved for: 17907 (plus 35898 internal DOFs).
Symmetric matrices found.
Scales for dependent variables:
Concentration (comp2.Oxi): 0.23

Step	Time	Stepsize	Res	Jac	Sol	Order	Tfail	NLfail	LinErr	LinRes
0	0	- out	5	3	5			0	6e-16	1.3e-15
1	2.5768e-08	2.5768e-08	7	4	7	1	0	0	3.4e-16	1e-15
2	5.1536e-08	2.5768e-08	9	5	9	1	0	0	3.2e-16	1.2e-15
3	1.0307e-07	5.1536e-08	11	6	11	2	0	0	3.7e-16	1.3e-15
4	2.0615e-07	1.0307e-07	13	7	13	2	0	0	4.9e-16	1.7e-15
5	4.1229e-07	2.0615e-07	15	8	15	2	0	0	6.5e-16	3.4e-15
6	6.1844e-07	2.0615e-07	17	9	17	2	0	0	5.9e-16	3.7e-15
7	8.2458e-07	2.0615e-07	19	10	19	2	0	0	5.6e-16	4.1e-15
8	1.0307e-06	2.0615e-07	21	11	21	2	0	0	5.9e-16	4e-15
9	1.443e-06	4.1229e-07	23	12	23	2	0	0	1.1e-15	6.5e-15
10	1.8553e-06	4.1229e-07	25	13	25	2	0	0	8.7e-16	6e-15
11	2.2676e-06	4.1229e-07	27	14	27	2	0	0	7.5e-16	6.2e-15
12	2.6799e-06	4.1229e-07	29	15	29	2	0	0	8.2e-16	5.6e-15
13	3.5045e-06	8.2458e-07	31	16	31	2	0	0	9.7e-16	8.4e-15
14	4.3291e-06	8.2458e-07	33	17	33	2	0	0	1e-15	7.3e-15
15	5.1536e-06	8.2458e-07	35	18	35	2	0	0	1e-15	7.2e-15
16	5.9782e-06	8.2458e-07	37	19	37	2	0	0	8.7e-16	6.6e-15
17	7.6274e-06	1.6492e-06	39	20	39	2	0	0	1e-15	9.5e-15
18	9.2766e-06	1.6492e-06	41	21	41	2	0	0	1.6e-15	8.5e-15
19	1.0926e-05	1.6492e-06	43	22	43	2	0	0	9.8e-16	8.2e-15
20	1.2575e-05	1.6492e-06	45	23	45	2	0	0	1.2e-15	7.3e-15
21	1.4224e-05	1.6492e-06	47	24	47	2	0	0	1.3e-15	6.5e-15
22	1.7522e-05	3.2983e-06	49	25	49	2	0	0	2e-15	9.6e-15
23	2.0821e-05	3.2983e-06	51	26	51	2	0	0	1.9e-15	8.4e-15
24	2.4119e-05	3.2983e-06	53	27	53	2	0	0	1.5e-15	8.3e-15
25	2.7417e-05	3.2983e-06	55	28	55	2	0	0	1.4e-15	7.7e-15
26	3.4014e-05	6.5967e-06	57	29	57	2	0	0	2e-15	1.1e-14
27	4.0611e-05	6.5967e-06	59	30	59	2	0	0	1.5e-15	9.3e-15
28	4.7207e-05	6.5967e-06	61	31	61	2	0	0	2.1e-15	9.2e-15
29	5.3804e-05	6.5967e-06	63	32	63	2	0	0	2.7e-15	8.9e-15
30	6.0401e-05	6.5967e-06	65	33	65	2	0	0	2.1e-15	7.5e-15
31	7.3594e-05	1.3193e-05	67	34	67	2	0	0	1.3e-15	1.1e-14
32	8.6787e-05	1.3193e-05	69	35	69	2	0	0	1.6e-15	9.7e-15
33	9.9981e-05	1.3193e-05	71	36	71	2	0	0	1.8e-15	9.8e-15
34	0.00011317	1.3193e-05	73	37	73	2	0	0	3.1e-15	9.2e-15
35	0.00013956	2.6387e-05	75	38	75	2	0	0	3.6e-15	1.4e-14
36	0.00016595	2.6387e-05	77	39	77	2	0	0	2.6e-15	1.2e-14
37	0.00019233	2.6387e-05	79	40	79	2	0	0	2.1e-15	1.1e-14
38	0.00021872	2.6387e-05	81	41	81	2	0	0	1.9e-15	1e-14
39	0.00027149	5.2773e-05	83	42	83	2	0	0	4.4e-15	1.5e-14

40	0.00032427	5.2773e-05		85	43	85	2	0	0	5.4e-15	1.3e-14
41	0.00037704	5.2773e-05		87	44	87	2	0	0	3.2e-15	1.2e-14
42	0.00048259	0.00010555		89	45	89	2	0	0	2.5e-15	1.8e-14
43	0.00058813	0.00010555		91	46	91	2	0	0	2.6e-15	1.5e-14
44	0.00069368	0.00010555		93	47	93	2	0	0	2.7e-15	1.5e-14
45	0.00079923	0.00010555		95	48	95	2	0	0	1.6e-15	1.3e-14
46	0.0010103	0.00021109		97	49	97	2	0	0	6e-15	1.8e-14
47	0.0012214	0.00021109		99	50	99	2	0	0	5.2e-15	1.6e-14
48	0.0016436	0.00042219		101	51	101	2	0	0	2.8e-15	2.8e-14
49	0.0020658	0.00042219		103	52	103	2	0	0	3.8e-15	2.6e-14
50	0.002488	0.00042219		105	53	105	2	0	0	3.5e-15	2.3e-14
51	0.0033323	0.00084437		107	54	107	2	0	0	4.4e-15	3e-14
52	0.0041767	0.00084437		109	55	109	2	0	0	2.3e-15	2.1e-14
-	0.005	-	out								
53	0.0058655	0.0016887		111	56	111	2	0	0	2.1e-15	3.4e-14
54	0.0075542	0.0016887		113	57	113	2	0	0	2.2e-15	3.5e-14
55	0.009243	0.0016887		115	58	115	2	0	0	1.7e-15	3.5e-14
-	0.01	-	out								
56	0.010932	0.0016887		117	59	117	2	0	0	2.6e-15	3.4e-14
57	0.014309	0.0033775		119	60	119	2	0	0	3.3e-15	4.6e-14
-	0.015	-	out								
58	0.017687	0.0033775		121	61	121	2	0	0	5.2e-15	3.4e-14
-	0.02	-	out								
59	0.024442	0.006755		123	62	123	2	0	0	3.2e-15	6e-14
-	0.025	-	out								
-	0.03	-	out								
60	0.031197	0.006755		125	63	125	2	0	0	2.8e-15	5.1e-14
-	0.035	-	out								
61	0.037952	0.006755		127	64	127	2	0	0	4.5e-15	5.1e-14
-	0.04	-	out								
-	0.045	-	out								
-	0.05	-	out								
62	0.051462	0.01351		129	65	129	2	0	0	5.8e-15	7e-14
-	0.055	-	out								
-	0.06	-	out								
63	0.064972	0.01351		131	66	131	2	0	0	3.6e-15	5.2e-14
-	0.065	-	out								
-	0.07	-	out								
-	0.075	-	out								
-	0.08	-	out								
-	0.085	-	out								
-	0.09	-	out								
64	0.091991	0.02702		133	67	133	2	0	0	6.7e-15	8.1e-14
-	0.095	-	out								
-	0.1	-	out								
-	0.105	-	out								
-	0.11	-	out								
-	0.115	-	out								
65	0.11901	0.02702		135	68	135	1	0	0	9.9e-15	5.6e-14
-	0.12	-	out								
-	0.125	-	out								
-	0.13	-	out								
-	0.135	-	out								
-	0.14	-	out								
-	0.145	-	out								
-	0.15	-	out								

-	0.155	- out								
-	0.16	- out								
-	0.165	- out								
-	0.17	- out								
66	0.17305	0.05404	137	69	137	1	0	0	3.2e-15	3e-14
-	0.175	- out								
-	0.18	- out								
-	0.185	- out								
-	0.19	- out								
-	0.195	- out								
-	0.2	- out								
-	0.205	- out								
-	0.21	- out								
-	0.215	- out								
-	0.22	- out								
-	0.225	- out								
-	0.23	- out								
-	0.235	- out								
-	0.24	- out								
-	0.245	- out								
-	0.25	- out								
-	0.25	- out								
-	0.27	- out								
67	0.27205	0.099	139	70	139	1	0	0	7.3e-15	4.4e-14
-	0.29	- out								
-	0.31	- out								
-	0.33	- out								
-	0.35	- out								
-	0.37	- out								
68	0.37105	0.099	141	71	141	1	0	0	1.3e-14	4.2e-14
-	0.39	- out								
-	0.41	- out								
-	0.43	- out								
-	0.45	- out								
-	0.47	- out								
69	0.47005	0.099	143	72	143	1	0	0	1e-14	3.4e-14
-	0.49	- out								
-	0.51	- out								
-	0.53	- out								
-	0.55	- out								
70	0.56905	0.099	145	73	145	1	0	0	5.3e-15	2.7e-14
-	0.57	- out								
-	0.59	- out								
-	0.61	- out								
-	0.63	- out								
-	0.65	- out								
71	0.66805	0.099	146	74	146	1	0	0	4.1e-15	2.3e-14
-	0.67	- out								
-	0.69	- out								
-	0.71	- out								
-	0.73	- out								
-	0.75	- out								
72	0.76705	0.099	147	75	147	1	0	0	3.2e-15	2.1e-14
-	0.77	- out								
-	0.79	- out								
-	0.81	- out								

```
   -        0.83           - out
   -        0.85           - out
  73     0.86605       0.099      148   76  148     2     0      0  1.4e-14  1.6e-14
   -        0.87           - out
   -        0.89           - out
   -        0.91           - out
   -        0.93           - out
   -        0.95           - out
  74     0.96505       0.099      149   77  149     2     0      0  1.3e-14  1.1e-14
   -        0.97           - out
   -        0.99           - out
  75      1.0641       0.099      150   78  150     2     0      0  1.1e-14    1e-14
Time-stepping completed.
Solution time: 13 s.
Physical memory: 1.09 GB
Virtual memory: 1.07 GB
Ended at May 28, 2023, 4:03:22 PM.
----- Time-Dependent Solver 1 in Oxygen Pulse/Solution 958 (sol958) ----------->
```

k0Sub=0, d=2.4E-6 (su5)

GENERAL

Description	Value
Solution	k0Sub=0, d=2.4E-6

LOG

```
<---- Compile Equations: Stationary in Oxygen Pulse/Solution 958 (sol958) ------
Started at May 28, 2023, 4:03:25 PM.
Parameter k0Sub = 0.0 (m/s).
Parameter d = 2.4E-6 (m).
Geometry shape function: Linear Lagrange
Number of vertex elements: 10
Number of boundary elements: 890
Number of elements: 35147
Minimum element quality: 0.4544
Number of vertex elements: 10
Minimum element quality: 0.4544
Time: 2 s.
Physical memory: 1.1 GB
Virtual memory: 1.07 GB
Ended at May 28, 2023, 4:03:27 PM.
----- Compile Equations: Stationary in Oxygen Pulse/Solution 958 (sol958) ----->

<---- Dependent Variables 1 in Oxygen Pulse/Solution 958 (sol958) --------------
Started at May 28, 2023, 4:03:27 PM.
Solution time: 0 s.
Physical memory: 1.05 GB
Virtual memory: 1.03 GB
Ended at May 28, 2023, 4:03:27 PM.
----- Dependent Variables 1 in Oxygen Pulse/Solution 958 (sol958) ------------->

<---- Stationary Solver 1 in Oxygen Pulse/Solution 958 (sol958) ----------------
Started at May 28, 2023, 4:03:28 PM.
Linear solver
Number of degrees of freedom solved for: 18002 (plus 937 internal DOFs).
Symmetric matrices found.
Scales for dependent variables:
Concentration (comp2.Oxi): 0.23
Orthonormal null-space function used.
Iter      SolEst     Damping    Stepsize #Res #Jac #Sol   LinErr   LinRes
   1     2.2e-16   1.0000000     2.2e-16    1    1    1  5.1e-14  2.9e-16
Solution time: 0 s.
Physical memory: 1.07 GB
Virtual memory: 1.05 GB
Ended at May 28, 2023, 4:03:28 PM.
----- Stationary Solver 1 in Oxygen Pulse/Solution 958 (sol958) --------------->

<---- Compile Equations: Oxygen Probe Pulse in Oxygen Pulse/Solution 958
      (sol958) -----------------------------------------------------------------
Started at May 28, 2023, 4:03:28 PM.
Geometry shape function: Linear Lagrange
Time: 1 s.
Physical memory: 1.08 GB
Virtual memory: 1.06 GB
Ended at May 28, 2023, 4:03:29 PM.
----- Compile Equations: Oxygen Probe Pulse in Oxygen Pulse/Solution 958
      (sol958) ---------------------------------------------------------------->

<---- Dependent Variables 2 in Oxygen Pulse/Solution 958 (sol958) --------------
Started at May 28, 2023, 4:03:29 PM.
Initial values of variables solved for: Solution 958 (sol958), Solution Store 1 (sol
985).
```

Values of variables not solved for: Solution 958 (sol958), Solution Store 1 (sol985)
.
Solution time: 0 s.
Physical memory: 1.09 GB
Virtual memory: 1.06 GB
Ended at May 28, 2023, 4:03:29 PM.
----- Dependent Variables 2 in Oxygen Pulse/Solution 958 (sol958) ------------->

<---- Time-Dependent Solver 1 in Oxygen Pulse/Solution 958 (sol958) ------------
Started at May 28, 2023, 4:03:29 PM.
Time-dependent solver (BDF)
Number of degrees of freedom solved for: 18002 (plus 36084 internal DOFs).
Symmetric matrices found.
Scales for dependent variables:
Concentration (comp2.Oxi): 0.23

Step	Time	Stepsize	Res	Jac	Sol	Order	Tfail	NLfail	LinErr	LinRes
0	0	- out	5	3	5			0	7.9e-16	1.5e-15
1	2.5893e-08	2.5893e-08	7	4	7	1	0	0	3.6e-16	1e-15
2	5.1785e-08	2.5893e-08	9	5	9	1	0	0	3.7e-16	1.2e-15
3	1.0357e-07	5.1785e-08	11	6	11	2	0	0	4.2e-16	1.4e-15
4	2.0714e-07	1.0357e-07	13	7	13	2	0	0	4.7e-16	1.7e-15
5	4.1428e-07	2.0714e-07	15	8	15	2	0	0	5.7e-16	3.5e-15
6	6.2142e-07	2.0714e-07	17	9	17	2	0	0	7.5e-16	3.8e-15
7	8.2856e-07	2.0714e-07	19	10	19	2	0	0	6.1e-16	4e-15
8	1.0357e-06	2.0714e-07	21	11	21	2	0	0	6.1e-16	4e-15
9	1.45e-06	4.1428e-07	23	12	23	2	0	0	7.1e-16	6.2e-15
10	1.8643e-06	4.1428e-07	25	13	25	2	0	0	9e-16	6.3e-15
11	2.2785e-06	4.1428e-07	27	14	27	2	0	0	8.1e-16	6.1e-15
12	2.6928e-06	4.1428e-07	29	15	29	2	0	0	1e-15	5.8e-15
13	3.5214e-06	8.2856e-07	31	16	31	2	0	0	1.3e-15	8.6e-15
14	4.35e-06	8.2856e-07	33	17	33	2	0	0	9.6e-16	7.6e-15
15	5.1785e-06	8.2856e-07	35	18	35	2	0	0	1e-15	7.4e-15
16	6.0071e-06	8.2856e-07	37	19	37	2	0	0	1.2e-15	6.5e-15
17	7.6642e-06	1.6571e-06	39	20	39	2	0	0	1.4e-15	9.8e-15
18	9.3213e-06	1.6571e-06	41	21	41	2	0	0	9.5e-16	8.3e-15
19	1.0978e-05	1.6571e-06	43	22	43	2	0	0	1.4e-15	8.2e-15
20	1.2636e-05	1.6571e-06	45	23	45	2	0	0	1.2e-15	7.4e-15
21	1.4293e-05	1.6571e-06	47	24	47	2	0	0	9.9e-16	6.7e-15
22	1.7607e-05	3.3142e-06	49	25	49	2	0	0	1.5e-15	9.9e-15
23	2.0921e-05	3.3142e-06	51	26	51	2	0	0	1.6e-15	8.7e-15
24	2.4235e-05	3.3142e-06	53	27	53	2	0	0	1.8e-15	8.6e-15
25	2.755e-05	3.3142e-06	55	28	55	2	0	0	1.7e-15	7.9e-15
26	3.4178e-05	6.6285e-06	57	29	57	2	0	0	1.8e-15	1.1e-14
27	4.0807e-05	6.6285e-06	59	30	59	2	0	0	2.2e-15	1e-14
28	4.7435e-05	6.6285e-06	61	31	61	2	0	0	2e-15	9.8e-15
29	5.4064e-05	6.6285e-06	63	32	63	2	0	0	1.7e-15	8.8e-15
30	6.0692e-05	6.6285e-06	65	33	65	2	0	0	1.9e-15	8e-15
31	7.3949e-05	1.3257e-05	67	34	67	2	0	0	2.8e-15	1.2e-14
32	8.7206e-05	1.3257e-05	69	35	69	2	0	0	1.7e-15	9.9e-15
33	0.00010046	1.3257e-05	71	36	71	2	0	0	1.7e-15	1e-14
34	0.00011372	1.3257e-05	73	37	73	2	0	0	1.5e-15	9.7e-15
35	0.00014023	2.6514e-05	75	38	75	2	0	0	3.5e-15	1.3e-14
36	0.00016675	2.6514e-05	77	39	77	2	0	0	1.8e-15	1.2e-14
37	0.00019326	2.6514e-05	79	40	79	2	0	0	1.6e-15	1.2e-14
38	0.00021978	2.6514e-05	81	41	81	2	0	0	2e-15	1.1e-14
39	0.0002728	5.3028e-05	83	42	83	2	0	0	2e-15	1.6e-14

40	0.00032583	5.3028e-05	85	43	85	2	0	0	2.1e-15	1.3e-14
41	0.00037886	5.3028e-05	87	44	87	2	0	0	1.6e-15	1.3e-14
42	0.00048492	0.00010606	89	45	89	2	0	0	2.6e-15	2e-14
43	0.00059097	0.00010606	91	46	91	2	0	0	1.6e-15	1.6e-14
44	0.00069703	0.00010606	93	47	93	2	0	0	2.3e-15	1.6e-14
45	0.00080308	0.00010606	95	48	95	2	0	0	2.1e-15	1.3e-14
46	0.0010152	0.00021211	97	49	97	2	0	0	2.1e-15	1.9e-14
47	0.0012273	0.00021211	99	50	99	2	0	0	2.5e-15	1.5e-14
48	0.0016515	0.00042422	101	51	101	2	0	0	3.8e-15	2.6e-14
49	0.0020758	0.00042422	103	52	103	2	0	0	2.6e-15	2.4e-14
50	0.0025	0.00042422	105	53	105	2	0	0	4.4e-15	2.6e-14
51	0.0029242	0.00042422	107	54	107	2	0	0	5.4e-15	2e-14
52	0.0037727	0.00084845	109	55	109	2	0	0	2.2e-15	2.5e-14
53	0.0046211	0.00084845	111	56	111	2	0	0	5.7e-15	1.8e-14
-	0.005	- out								
54	0.006318	0.0016969	113	57	113	2	0	0	6.3e-15	3.3e-14
55	0.0080149	0.0016969	115	58	115	2	0	0	6.9e-15	3.2e-14
-	0.01	- out								
56	0.011409	0.0033938	117	59	117	2	0	0	7.7e-15	5.1e-14
57	0.014802	0.0033938	119	60	119	2	0	0	4.4e-15	4.7e-14
-	0.015	- out								
58	0.018196	0.0033938	121	61	121	2	0	0	3e-15	4.6e-14
-	0.02	- out								
59	0.024984	0.0067876	123	62	123	2	0	0	8.1e-15	5.9e-14
-	0.025	- out								
-	0.03	- out								
60	0.031771	0.0067876	125	63	125	2	0	0	3.8e-15	4.5e-14
-	0.035	- out								
-	0.04	- out								
-	0.045	- out								
61	0.045347	0.013575	127	64	127	2	0	0	3.3e-15	7.9e-14
-	0.05	- out								
-	0.055	- out								
62	0.058922	0.013575	129	65	129	2	0	0	6.3e-15	6.6e-14
-	0.06	- out								
-	0.065	- out								
-	0.07	- out								
63	0.072497	0.013575	131	66	131	2	0	0	2.1e-15	6.7e-14
-	0.075	- out								
-	0.08	- out								
-	0.085	- out								
64	0.086072	0.013575	133	67	133	2	0	0	3.1e-15	4.8e-14
-	0.09	- out								
-	0.095	- out								
-	0.1	- out								
-	0.105	- out								
-	0.11	- out								
65	0.11322	0.02715	135	68	135	2	0	0	3.8e-15	4.7e-14
-	0.115	- out								
-	0.12	- out								
-	0.125	- out								
-	0.13	- out								
-	0.135	- out								
-	0.14	- out								
-	0.145	- out								
-	0.15	- out								

-	0.155	- out								
-	0.16	- out								
-	0.165	- out								
66	0.16752	0.054301	137	69	137	1	0	0	6.7e-15	5.2e-14
-	0.17	- out								
-	0.175	- out								
-	0.18	- out								
-	0.185	- out								
-	0.19	- out								
-	0.195	- out								
-	0.2	- out								
-	0.205	- out								
-	0.21	- out								
-	0.215	- out								
-	0.22	- out								
67	0.22182	0.054301	139	70	139	1	0	0	1.7e-14	3.6e-14
-	0.225	- out								
-	0.23	- out								
-	0.235	- out								
-	0.24	- out								
-	0.245	- out								
-	0.25	- out								
-	0.25	- out								
-	0.27	- out								
-	0.29	- out								
-	0.31	- out								
68	0.32082	0.099	141	71	141	1	0	0	1.6e-14	4.1e-14
-	0.33	- out								
-	0.35	- out								
-	0.37	- out								
-	0.39	- out								
-	0.41	- out								
69	0.41982	0.099	143	72	143	1	0	0	8.8e-15	3.6e-14
-	0.43	- out								
-	0.45	- out								
-	0.47	- out								
-	0.49	- out								
-	0.51	- out								
70	0.51882	0.099	145	73	145	1	0	0	2.7e-15	2.9e-14
-	0.53	- out								
-	0.55	- out								
-	0.57	- out								
-	0.59	- out								
-	0.61	- out								
71	0.61782	0.099	147	74	147	1	0	0	1.4e-14	2.5e-14
-	0.63	- out								
-	0.65	- out								
-	0.67	- out								
-	0.69	- out								
-	0.71	- out								
72	0.71682	0.099	148	75	148	1	0	0	1.7e-15	2.1e-14
-	0.73	- out								
-	0.75	- out								
-	0.77	- out								
-	0.79	- out								
-	0.81	- out								

```
  73     0.81582       0.099      149   76  149     1     0      0  4.9e-15  1.9e-14
   -        0.83           - out
   -        0.85           - out
   -        0.87           - out
   -        0.89           - out
   -        0.91           - out
  74     0.91482       0.099      150   77  150     2     0      0  5.4e-15  1.6e-14
   -        0.93           - out
   -        0.95           - out
   -        0.97           - out
   -        0.99           - out
  75      1.0138       0.099      151   78  151     2     0      0    1e-14    1e-14
Time-stepping completed.
Solution time: 13 s.
Physical memory: 1.11 GB
Virtual memory: 1.09 GB
Ended at May 28, 2023, 4:03:41 PM.
----- Time-Dependent Solver 1 in Oxygen Pulse/Solution 958 (sol958) ----------->
```

k0Sub=0, d=3E-6 (su6)

GENERAL

Description	Value
Solution	k0Sub=0, d=3E-6

LOG

```
<---- Compile Equations: Stationary in Oxygen Pulse/Solution 958 (sol958) ------
Started at May 28, 2023, 4:03:44 PM.
Parameter k0Sub = 0.0 (m/s).
Parameter d = 3.0E-6 (m).
Geometry shape function: Linear Lagrange
Number of vertex elements: 10
Number of boundary elements: 890
Number of elements: 45121
Minimum element quality: 0.4275
Number of vertex elements: 10
Minimum element quality: 0.4275
Time: 5 s.
Physical memory: 1.07 GB
Virtual memory: 1.05 GB
Ended at May 28, 2023, 4:03:49 PM.
----- Compile Equations: Stationary in Oxygen Pulse/Solution 958 (sol958) ----->

<---- Dependent Variables 1 in Oxygen Pulse/Solution 958 (sol958) --------------
Started at May 28, 2023, 4:03:49 PM.
Solution time: 0 s.
Physical memory: 1.06 GB
Virtual memory: 1.04 GB
Ended at May 28, 2023, 4:03:49 PM.
----- Dependent Variables 1 in Oxygen Pulse/Solution 958 (sol958) ------------->

<---- Stationary Solver 1 in Oxygen Pulse/Solution 958 (sol958) ----------------
Started at May 28, 2023, 4:03:49 PM.
Linear solver
Number of degrees of freedom solved for: 22990 (plus 935 internal DOFs).
Symmetric matrices found.
Scales for dependent variables:
Concentration (comp2.Oxi): 0.23
Orthonormal null-space function used.
Iter      SolEst     Damping    Stepsize #Res #Jac #Sol   LinErr   LinRes
   1     2.3e-16   1.0000000     2.3e-16    1    1    1  1.9e-14  2.8e-16
Solution time: 1 s.
Physical memory: 1.06 GB
Virtual memory: 1.04 GB
Ended at May 28, 2023, 4:03:50 PM.
----- Stationary Solver 1 in Oxygen Pulse/Solution 958 (sol958) --------------->

<---- Compile Equations: Oxygen Probe Pulse in Oxygen Pulse/Solution 958
      (sol958) -----------------------------------------------------------------
Started at May 28, 2023, 4:03:50 PM.
Geometry shape function: Linear Lagrange
Time: 1 s.
Physical memory: 1.06 GB
Virtual memory: 1.04 GB
Ended at May 28, 2023, 4:03:50 PM.
----- Compile Equations: Oxygen Probe Pulse in Oxygen Pulse/Solution 958
      (sol958) ---------------------------------------------------------------->

<---- Dependent Variables 2 in Oxygen Pulse/Solution 958 (sol958) --------------
Started at May 28, 2023, 4:03:50 PM.
Initial values of variables solved for: Solution 958 (sol958), Solution Store 1 (sol
985).
```

Values of variables not solved for: Solution 958 (sol958), Solution Store 1 (sol985)
.
Solution time: 0 s.
Physical memory: 1.06 GB
Virtual memory: 1.04 GB
Ended at May 28, 2023, 4:03:50 PM.
----- Dependent Variables 2 in Oxygen Pulse/Solution 958 (sol958) ------------>

<---- Time-Dependent Solver 1 in Oxygen Pulse/Solution 958 (sol958) ------------
Started at May 28, 2023, 4:03:50 PM.
Time-dependent solver (BDF)
Number of degrees of freedom solved for: 22990 (plus 46056 internal DOFs).
Symmetric matrices found.
Scales for dependent variables:
Concentration (comp2.Oxi): 0.23

Step	Time	Stepsize	Res	Jac	Sol	Order	Tfail	NLfail	LinErr	LinRes
0	0	- out	5	3	5			0	7.1e-16	8.9e-16
1	3.5548e-08	3.5548e-08	7	4	7	1	0	0	3.2e-16	9.2e-16
2	7.1096e-08	3.5548e-08	9	5	9	1	0	0	3.1e-16	9.7e-16
3	1.4219e-07	7.1096e-08	11	6	11	2	0	0	3.9e-16	1.1e-15
4	2.8439e-07	1.4219e-07	13	7	13	2	0	0	3.7e-16	1.3e-15
5	5.6877e-07	2.8439e-07	15	8	15	2	0	0	6.2e-16	2.4e-15
6	8.5316e-07	2.8439e-07	17	9	17	2	0	0	5.6e-16	2.8e-15
7	1.1375e-06	2.8439e-07	19	10	19	2	0	0	5.8e-16	3e-15
8	1.4219e-06	2.8439e-07	21	11	21	2	0	0	4.7e-16	3.1e-15
9	1.9907e-06	5.6877e-07	23	12	23	2	0	0	6.1e-16	5e-15
10	2.5595e-06	5.6877e-07	25	13	25	2	0	0	7.3e-16	4.7e-15
11	3.1282e-06	5.6877e-07	27	14	27	2	0	0	6.6e-16	4.9e-15
12	3.697e-06	5.6877e-07	29	15	29	2	0	0	6.4e-16	4.6e-15
13	4.8345e-06	1.1375e-06	31	16	31	2	0	0	8.1e-16	6.9e-15
14	5.9721e-06	1.1375e-06	33	17	33	2	0	0	9.5e-16	6.4e-15
15	7.1096e-06	1.1375e-06	35	18	35	2	0	0	7.7e-16	6.3e-15
16	8.2472e-06	1.1375e-06	37	19	37	2	0	0	7.5e-16	5.6e-15
17	1.0522e-05	2.2751e-06	39	20	39	2	0	0	1.3e-15	8.9e-15
18	1.2797e-05	2.2751e-06	41	21	41	2	0	0	1e-15	7.8e-15
19	1.5072e-05	2.2751e-06	43	22	43	2	0	0	9.9e-16	8e-15
20	1.7347e-05	2.2751e-06	45	23	45	2	0	0	1e-15	7.1e-15
21	2.1898e-05	4.5502e-06	47	24	47	2	0	0	1.5e-15	1.1e-14
22	2.6448e-05	4.5502e-06	49	25	49	2	0	0	1.7e-15	9.8e-15
23	3.0998e-05	4.5502e-06	51	26	51	2	0	0	1.7e-15	9.7e-15
24	3.5548e-05	4.5502e-06	53	27	53	2	0	0	9.5e-16	9.1e-15
25	4.4648e-05	9.1003e-06	55	28	55	2	0	0	1.5e-15	1.4e-14
26	5.3749e-05	9.1003e-06	57	29	57	2	0	0	1.2e-15	1.2e-14
27	6.2849e-05	9.1003e-06	59	30	59	2	0	0	2e-15	1.2e-14
28	7.1949e-05	9.1003e-06	61	31	61	2	0	0	1.2e-15	1.1e-14
29	9.015e-05	1.8201e-05	63	32	63	2	0	0	2e-15	1.6e-14
30	0.00010835	1.8201e-05	65	33	65	2	0	0	2.3e-15	1.3e-14
31	0.00012655	1.8201e-05	67	34	67	2	0	0	2.3e-15	1.3e-14
32	0.00014475	1.8201e-05	69	35	69	2	0	0	1.9e-15	1.2e-14
33	0.00018115	3.6401e-05	71	36	71	2	0	0	1.2e-15	1.8e-14
34	0.00021755	3.6401e-05	73	37	73	2	0	0	3.9e-15	1.5e-14
35	0.00025396	3.6401e-05	75	38	75	2	0	0	2.7e-15	1.5e-14
36	0.00029036	3.6401e-05	77	39	77	2	0	0	1.6e-15	1.4e-14
37	0.00036316	7.2803e-05	79	40	79	2	0	0	3.5e-15	2e-14
38	0.00043596	7.2803e-05	81	41	81	2	0	0	4.4e-15	1.7e-14
39	0.00058157	0.00014561	83	42	83	2	0	0	3.2e-15	2.7e-14

40	0.00072717	0.00014561	85	43	85	2	0	0	2.7e-15	2.2e-14
41	0.00087278	0.00014561	87	44	87	2	0	0	1.9e-15	2.3e-14
42	0.0010184	0.00014561	89	45	89	2	0	0	3.2e-15	1.9e-14
43	0.0013096	0.00029121	91	46	91	2	0	0	3.1e-15	2.6e-14
44	0.0016008	0.00029121	93	47	93	2	0	0	6.5e-15	2e-14
45	0.0021832	0.00058242	95	48	95	2	0	0	6.1e-15	3.3e-14
46	0.0027656	0.00058242	97	49	97	2	0	0	2.7e-15	3.2e-14
47	0.0033481	0.00058242	99	50	99	2	0	0	3e-15	3.4e-14
48	0.0045129	0.0011648	101	51	101	2	0	0	7.1e-15	4.2e-14
-	0.005	-	out							
49	0.0056777	0.0011648	103	52	103	2	0	0	2.2e-15	2.7e-14
50	0.0080074	0.0023297	105	53	105	2	0	0	4.6e-15	4.7e-14
-	0.01	-	out							
51	0.010337	0.0023297	107	54	107	2	0	0	2.7e-15	4.6e-14
52	0.012667	0.0023297	109	55	109	2	0	0	3.2e-15	4.6e-14
-	0.015	-	out							
53	0.017326	0.0046594	111	56	111	2	0	0	3.2e-15	6.3e-14
-	0.02	-	out							
54	0.021986	0.0046594	113	57	113	2	0	0	6.1e-15	5.1e-14
-	0.025	-	out							
-	0.03	-	out							
55	0.031304	0.0093187	115	58	115	2	0	0	2.5e-15	8.1e-14
-	0.035	-	out							
-	0.04	-	out							
56	0.040623	0.0093187	117	59	117	2	0	0	4.3e-15	7.2e-14
-	0.045	-	out							
57	0.049942	0.0093187	119	60	119	2	0	0	7.1e-15	7.4e-14
-	0.05	-	out							
-	0.055	-	out							
-	0.06	-	out							
-	0.065	-	out							
58	0.068579	0.018637	121	61	121	2	0	0	8.3e-15	1e-13
-	0.07	-	out							
-	0.075	-	out							
-	0.08	-	out							
-	0.085	-	out							
59	0.087217	0.018637	123	62	123	1	0	0	8.3e-15	8.5e-14
-	0.09	-	out							
-	0.095	-	out							
-	0.1	-	out							
-	0.105	-	out							
-	0.11	-	out							
-	0.115	-	out							
-	0.12	-	out							
60	0.12449	0.037275	125	63	125	1	0	0	5.7e-15	6.5e-14
-	0.125	-	out							
-	0.13	-	out							
-	0.135	-	out							
-	0.14	-	out							
-	0.145	-	out							
-	0.15	-	out							
-	0.155	-	out							
-	0.16	-	out							
-	0.165	-	out							
-	0.17	-	out							
-	0.175	-	out							

-	0.18	- out								
-	0.185	- out								
-	0.19	- out								
-	0.195	- out								
61	0.19904	0.07455	127	64	127	1	0	0	1.8e-14	8.7e-14
-	0.2	- out								
-	0.205	- out								
-	0.21	- out								
-	0.215	- out								
-	0.22	- out								
-	0.225	- out								
-	0.23	- out								
-	0.235	- out								
-	0.24	- out								
-	0.245	- out								
-	0.25	- out								
-	0.25	- out								
-	0.27	- out								
62	0.27359	0.07455	129	65	129	1	0	0	1.5e-14	7.2e-14
-	0.29	- out								
-	0.31	- out								
-	0.33	- out								
-	0.35	- out								
-	0.37	- out								
63	0.37259	0.099	131	66	131	1	0	0	3.5e-15	7e-14
-	0.39	- out								
-	0.41	- out								
-	0.43	- out								
-	0.45	- out								
-	0.47	- out								
64	0.47159	0.099	133	67	133	1	0	0	2e-14	5.4e-14
-	0.49	- out								
-	0.51	- out								
-	0.53	- out								
-	0.55	- out								
-	0.57	- out								
65	0.57059	0.099	135	68	135	1	0	0	3.4e-15	4.2e-14
-	0.59	- out								
-	0.61	- out								
-	0.63	- out								
-	0.65	- out								
66	0.66959	0.099	136	69	136	1	0	0	9.2e-15	3.6e-14
-	0.67	- out								
-	0.69	- out								
-	0.71	- out								
-	0.73	- out								
-	0.75	- out								
67	0.76859	0.099	137	70	137	1	0	0	6.7e-15	3.1e-14
-	0.77	- out								
-	0.79	- out								
-	0.81	- out								
-	0.83	- out								
-	0.85	- out								
68	0.86759	0.099	138	71	138	2	0	0	9.5e-15	2.5e-14
-	0.87	- out								
-	0.89	- out								

```
   -        0.91           - out
   -        0.93           - out
   -        0.95           - out
  69     0.96659       0.099      139   72  139      2      0       0  8.3e-15  1.8e-14
   -        0.97           - out
   -        0.99           - out
  70      1.0656       0.099      140   73  140      2      0       0  2.3e-14  1.6e-14
Time-stepping completed.
Solution time: 15 s.
Physical memory: 1.11 GB
Virtual memory: 1.1 GB
Ended at May 28, 2023, 4:04:06 PM.
----- Time-Dependent Solver 1 in Oxygen Pulse/Solution 958 (sol958) ----------->
```

k0Sub=0, d=4E-6 (su7)

GENERAL

Description	Value
Solution	k0Sub=0, d=4E-6

LOG

```
<---- Compile Equations: Stationary in Oxygen Pulse/Solution 958 (sol958) ------
Started at May 28, 2023, 4:04:09 PM.
Parameter k0Sub = 0.0 (m/s).
Parameter d = 4.0E-6 (m).
Geometry shape function: Linear Lagrange
Number of vertex elements: 10
Number of boundary elements: 892
Number of elements: 35502
Minimum element quality: 0.4317
Number of vertex elements: 10
Minimum element quality: 0.4317
Time: 2 s.
Physical memory: 1.12 GB
Virtual memory: 1.1 GB
Ended at May 28, 2023, 4:04:11 PM.
----- Compile Equations: Stationary in Oxygen Pulse/Solution 958 (sol958) ----->

<---- Dependent Variables 1 in Oxygen Pulse/Solution 958 (sol958) --------------
Started at May 28, 2023, 4:04:11 PM.
Solution time: 0 s.
Physical memory: 1.12 GB
Virtual memory: 1.1 GB
Ended at May 28, 2023, 4:04:11 PM.
----- Dependent Variables 1 in Oxygen Pulse/Solution 958 (sol958) ------------->

<---- Stationary Solver 1 in Oxygen Pulse/Solution 958 (sol958) ----------------
Started at May 28, 2023, 4:04:11 PM.
Linear solver
Number of degrees of freedom solved for: 18182 (plus 936 internal DOFs).
Symmetric matrices found.
Scales for dependent variables:
Concentration (comp2.Oxi): 0.23
Orthonormal null-space function used.
Iter      SolEst     Damping    Stepsize #Res #Jac #Sol   LinErr   LinRes
   1     2.1e-16   1.0000000     2.1e-16    1    1    1  5.9e-14    3e-16
Solution time: 0 s.
Physical memory: 1.13 GB
Virtual memory: 1.1 GB
Ended at May 28, 2023, 4:04:12 PM.
----- Stationary Solver 1 in Oxygen Pulse/Solution 958 (sol958) --------------->

<---- Compile Equations: Oxygen Probe Pulse in Oxygen Pulse/Solution 958
      (sol958) -----------------------------------------------------------------
Started at May 28, 2023, 4:04:12 PM.
Geometry shape function: Linear Lagrange
Time: 1 s.
Physical memory: 1.13 GB
Virtual memory: 1.11 GB
Ended at May 28, 2023, 4:04:12 PM.
----- Compile Equations: Oxygen Probe Pulse in Oxygen Pulse/Solution 958
      (sol958) ---------------------------------------------------------------->

<---- Dependent Variables 2 in Oxygen Pulse/Solution 958 (sol958) --------------
Started at May 28, 2023, 4:04:12 PM.
Initial values of variables solved for: Solution 958 (sol958), Solution Store 1 (sol
985).
```

Values of variables not solved for: Solution 958 (sol958), Solution Store 1 (sol985)
.
Solution time: 0 s.
Physical memory: 1.13 GB
Virtual memory: 1.11 GB
Ended at May 28, 2023, 4:04:12 PM.
----- Dependent Variables 2 in Oxygen Pulse/Solution 958 (sol958) ------------->

<---- Time-Dependent Solver 1 in Oxygen Pulse/Solution 958 (sol958) ------------
Started at May 28, 2023, 4:04:12 PM.
Time-dependent solver (BDF)
Number of degrees of freedom solved for: 18182 (plus 36438 internal DOFs).
Symmetric matrices found.
Scales for dependent variables:
Concentration (comp2.Oxi): 0.23

Step	Time	Stepsize	Res	Jac	Sol	Order	Tfail	NLfail	LinErr	LinRes
0	0	- out	5	3	5			0	7.7e-16	1.4e-15
1	2.614e-08	2.614e-08	7	4	7	1	0	0	3.3e-16	1e-15
2	5.2279e-08	2.614e-08	9	5	9	1	0	0	3.8e-16	1.2e-15
3	1.0456e-07	5.2279e-08	11	6	11	2	0	0	3.6e-16	1.3e-15
4	2.0912e-07	1.0456e-07	13	7	13	2	0	0	5.1e-16	1.7e-15
5	4.1823e-07	2.0912e-07	15	8	15	2	0	0	6e-16	3.2e-15
6	6.2735e-07	2.0912e-07	17	9	17	2	0	0	6.1e-16	3.8e-15
7	8.3647e-07	2.0912e-07	19	10	19	2	0	0	5.9e-16	3.9e-15
8	1.0456e-06	2.0912e-07	21	11	21	2	0	0	6e-16	4e-15
9	1.4638e-06	4.1823e-07	23	12	23	2	0	0	7.3e-16	6.2e-15
10	1.882e-06	4.1823e-07	25	13	25	2	0	0	8.6e-16	6e-15
11	2.3003e-06	4.1823e-07	27	14	27	2	0	0	9.8e-16	6.1e-15
12	2.7185e-06	4.1823e-07	29	15	29	2	0	0	8.4e-16	5.6e-15
13	3.555e-06	8.3647e-07	31	16	31	2	0	0	1.4e-15	8.6e-15
14	4.3914e-06	8.3647e-07	33	17	33	2	0	0	1.2e-15	7.5e-15
15	5.2279e-06	8.3647e-07	35	18	35	2	0	0	1.1e-15	7.4e-15
16	6.0644e-06	8.3647e-07	37	19	37	2	0	0	1.1e-15	6.6e-15
17	7.7373e-06	1.6729e-06	39	20	39	2	0	0	1.2e-15	9.6e-15
18	9.4102e-06	1.6729e-06	41	21	41	2	0	0	1.1e-15	8.3e-15
19	1.1083e-05	1.6729e-06	43	22	43	2	0	0	1.3e-15	8.4e-15
20	1.2756e-05	1.6729e-06	45	23	45	2	0	0	1.3e-15	7.4e-15
21	1.4429e-05	1.6729e-06	47	24	47	2	0	0	8.6e-16	6.5e-15
22	1.7775e-05	3.3459e-06	49	25	49	2	0	0	1.3e-15	1e-14
23	2.1121e-05	3.3459e-06	51	26	51	2	0	0	1.6e-15	8.7e-15
24	2.4467e-05	3.3459e-06	53	27	53	2	0	0	1.8e-15	8.5e-15
25	2.7812e-05	3.3459e-06	55	28	55	2	0	0	1.1e-15	7.9e-15
26	3.4504e-05	6.6917e-06	57	29	57	2	0	0	2e-15	1.1e-14
27	4.1196e-05	6.6917e-06	59	30	59	2	0	0	1.7e-15	1e-14
28	4.7888e-05	6.6917e-06	61	31	61	2	0	0	1.7e-15	1e-14
29	5.4579e-05	6.6917e-06	63	32	63	2	0	0	2.2e-15	9.1e-15
30	6.1271e-05	6.6917e-06	65	33	65	2	0	0	1.3e-15	8.1e-15
31	7.4655e-05	1.3383e-05	67	34	67	2	0	0	1.4e-15	1.2e-14
32	8.8038e-05	1.3383e-05	69	35	69	2	0	0	2e-15	1e-14
33	0.00010142	1.3383e-05	71	36	71	2	0	0	2e-15	1e-14
34	0.0001148	1.3383e-05	73	37	73	2	0	0	1e-15	9.6e-15
35	0.00014157	2.6767e-05	75	38	75	2	0	0	1.5e-15	1.4e-14
36	0.00016834	2.6767e-05	77	39	77	2	0	0	2.5e-15	1.2e-14
37	0.00019511	2.6767e-05	79	40	79	2	0	0	1.7e-15	1.2e-14
38	0.00022187	2.6767e-05	81	41	81	2	0	0	1.8e-15	1.1e-14
39	0.00027541	5.3534e-05	83	42	83	2	0	0	2.3e-15	1.5e-14

40	0.00032894	5.3534e-05		85	43	85	2	0	0	1.7e-15	1.3e-14
41	0.00038247	5.3534e-05		87	44	87	2	0	0	4.4e-15	1.4e-14
42	0.00048954	0.00010707		89	45	89	2	0	0	3.4e-15	1.9e-14
43	0.00059661	0.00010707		91	46	91	2	0	0	1.7e-15	1.5e-14
44	0.00070368	0.00010707		93	47	93	2	0	0	2.7e-15	1.5e-14
45	0.00081074	0.00010707		95	48	95	2	0	0	4e-15	1.4e-14
46	0.0010249	0.00021414		97	49	97	2	0	0	5e-15	1.9e-14
47	0.001239	0.00021414		99	50	99	2	0	0	2.1e-15	1.6e-14
48	0.0016673	0.00042827		101	51	101	2	0	0	1.9e-15	2.6e-14
49	0.0020956	0.00042827		103	52	103	2	0	0	4.1e-15	2.2e-14
50	0.0025238	0.00042827		105	53	105	2	0	0	2.6e-15	2.2e-14
51	0.0029521	0.00042827		107	54	107	2	0	0	4.1e-15	2e-14
52	0.0038086	0.00085654		109	55	109	2	0	0	2.2e-15	2.6e-14
53	0.0046652	0.00085654		111	56	111	2	0	0	1.5e-15	2e-14
-	0.005	-	out								
54	0.0063783	0.0017131		113	57	113	2	0	0	3.1e-15	3.4e-14
55	0.0080913	0.0017131		115	58	115	2	0	0	5.6e-15	3e-14
-	0.01	-	out								
56	0.011518	0.0034262		117	59	117	2	0	0	4.1e-15	4.4e-14
57	0.014944	0.0034262		119	60	119	1	0	0	3.9e-15	5.6e-14
-	0.015	-	out								
58	0.01837	0.0034262		121	61	121	1	0	0	3.5e-15	4.5e-14
-	0.02	-	out								
-	0.025	-	out								
59	0.025222	0.0068523		123	62	123	1	0	0	4.8e-15	7.2e-14
-	0.03	-	out								
60	0.032074	0.0068523		125	63	125	1	0	0	7.4e-15	6.8e-14
-	0.035	-	out								
-	0.04	-	out								
-	0.045	-	out								
61	0.045779	0.013705		127	64	127	1	0	0	3.3e-15	1e-13
-	0.05	-	out								
-	0.055	-	out								
62	0.059484	0.013705		129	65	129	1	0	0	7.4e-15	9e-14
-	0.06	-	out								
-	0.065	-	out								
-	0.07	-	out								
-	0.075	-	out								
-	0.08	-	out								
-	0.085	-	out								
63	0.086893	0.027409		131	66	131	1	0	0	8.9e-15	1.1e-13
-	0.09	-	out								
-	0.095	-	out								
-	0.1	-	out								
-	0.105	-	out								
-	0.11	-	out								
64	0.1143	0.027409		133	67	133	1	0	0	5.6e-15	8.4e-14
-	0.115	-	out								
-	0.12	-	out								
-	0.125	-	out								
-	0.13	-	out								
-	0.135	-	out								
-	0.14	-	out								
-	0.145	-	out								
-	0.15	-	out								
-	0.155	-	out								

-	0.16	-	out							
-	0.165	-	out							
65	0.16912	0.054819	135	68	135	1	0	0	5.7e-15	8.8e-14
-	0.17	-	out							
-	0.175	-	out							
-	0.18	-	out							
-	0.185	-	out							
-	0.19	-	out							
-	0.195	-	out							
-	0.2	-	out							
-	0.205	-	out							
-	0.21	-	out							
-	0.215	-	out							
-	0.22	-	out							
66	0.22394	0.054819	137	69	137	1	0	0	7e-15	6.5e-14
-	0.225	-	out							
-	0.23	-	out							
-	0.235	-	out							
-	0.24	-	out							
-	0.245	-	out							
-	0.25	-	out							
-	0.25	-	out							
-	0.27	-	out							
-	0.29	-	out							
-	0.31	-	out							
67	0.32294	0.099	139	70	139	1	0	0	1.1e-14	6.6e-14
-	0.33	-	out							
-	0.35	-	out							
-	0.37	-	out							
-	0.39	-	out							
-	0.41	-	out							
68	0.42194	0.099	141	71	141	1	0	0	1.8e-14	4.9e-14
-	0.43	-	out							
-	0.45	-	out							
-	0.47	-	out							
-	0.49	-	out							
-	0.51	-	out							
69	0.52094	0.099	143	72	143	1	0	0	3.6e-15	3.8e-14
-	0.53	-	out							
-	0.55	-	out							
-	0.57	-	out							
-	0.59	-	out							
-	0.61	-	out							
70	0.61994	0.099	145	73	145	1	0	0	2.5e-15	3e-14
-	0.63	-	out							
-	0.65	-	out							
-	0.67	-	out							
-	0.69	-	out							
-	0.71	-	out							
71	0.71894	0.099	146	74	146	1	0	0	3.4e-15	2.4e-14
-	0.73	-	out							
-	0.75	-	out							
-	0.77	-	out							
-	0.79	-	out							
-	0.81	-	out							
72	0.81794	0.099	147	75	147	1	0	0	9.1e-15	2.1e-14

```
   -        0.83           - out
   -        0.85           - out
   -        0.87           - out
   -        0.89           - out
   -        0.91           - out
  73     0.91694       0.099      148    76   148      2      0       0  8.4e-15  1.8e-14
   -        0.93           - out
   -        0.95           - out
   -        0.97           - out
   -        0.99           - out
  74      1.0159       0.099      149    77   149      2      0       0  3.5e-15  1.2e-14
Time-stepping completed.
Solution time: 13 s.
Physical memory: 1.15 GB
Virtual memory: 1.12 GB
Ended at May 28, 2023, 4:04:25 PM.
----- Time-Dependent Solver 1 in Oxygen Pulse/Solution 958 (sol958) ----------->
```

k0Sub=0, d=5E-6 (su8)

GENERAL

Description	Value
Solution	k0Sub=0, d=5E-6

LOG

```
<---- Compile Equations: Stationary in Oxygen Pulse/Solution 958 (sol958) ------
Started at May 28, 2023, 4:04:28 PM.
Parameter k0Sub = 0.0 (m/s).
Parameter d = 4.9999999999999996E-6 (m).
Geometry shape function: Linear Lagrange
Number of vertex elements: 10
Number of boundary elements: 893
Number of elements: 35744
Minimum element quality: 0.4532
Number of vertex elements: 10
Minimum element quality: 0.4532
Time: 2 s.
Physical memory: 1.11 GB
Virtual memory: 1.09 GB
Ended at May 28, 2023, 4:04:30 PM.
----- Compile Equations: Stationary in Oxygen Pulse/Solution 958 (sol958) ----->

<---- Dependent Variables 1 in Oxygen Pulse/Solution 958 (sol958) --------------
Started at May 28, 2023, 4:04:30 PM.
Solution time: 0 s.
Physical memory: 1.11 GB
Virtual memory: 1.09 GB
Ended at May 28, 2023, 4:04:30 PM.
----- Dependent Variables 1 in Oxygen Pulse/Solution 958 (sol958) ------------->

<---- Stationary Solver 1 in Oxygen Pulse/Solution 958 (sol958) ----------------
Started at May 28, 2023, 4:04:30 PM.
Linear solver
Number of degrees of freedom solved for: 18304 (plus 936 internal DOFs).
Symmetric matrices found.
Scales for dependent variables:
Concentration (comp2.Oxi): 0.23
Orthonormal null-space function used.
Iter      SolEst     Damping    Stepsize #Res #Jac #Sol   LinErr   LinRes
   1     2.2e-16   1.0000000     2.2e-16    1    1    1    3e-14  2.9e-16
Solution time: 0 s.
Physical memory: 1.12 GB
Virtual memory: 1.1 GB
Ended at May 28, 2023, 4:04:31 PM.
----- Stationary Solver 1 in Oxygen Pulse/Solution 958 (sol958) --------------->

<---- Compile Equations: Oxygen Probe Pulse in Oxygen Pulse/Solution 958
      (sol958) -----------------------------------------------------------------
Started at May 28, 2023, 4:04:31 PM.
Geometry shape function: Linear Lagrange
Time: 1 s.
Physical memory: 1.12 GB
Virtual memory: 1.1 GB
Ended at May 28, 2023, 4:04:31 PM.
----- Compile Equations: Oxygen Probe Pulse in Oxygen Pulse/Solution 958
      (sol958) ---------------------------------------------------------------->

<---- Dependent Variables 2 in Oxygen Pulse/Solution 958 (sol958) --------------
Started at May 28, 2023, 4:04:31 PM.
Initial values of variables solved for: Solution 958 (sol958), Solution Store 1 (sol
985).
```

Values of variables not solved for: Solution 958 (sol958), Solution Store 1 (sol985)
.
Solution time: 0 s.
Physical memory: 1.12 GB
Virtual memory: 1.1 GB
Ended at May 28, 2023, 4:04:31 PM.
----- Dependent Variables 2 in Oxygen Pulse/Solution 958 (sol958) ------------->

<---- Time-Dependent Solver 1 in Oxygen Pulse/Solution 958 (sol958) ------------
Started at May 28, 2023, 4:04:31 PM.
Time-dependent solver (BDF)
Number of degrees of freedom solved for: 18304 (plus 36680 internal DOFs).
Symmetric matrices found.
Scales for dependent variables:
Concentration (comp2.Oxi): 0.23

Step	Time	Stepsize	Res	Jac	Sol	Order	Tfail	NLfail	LinErr	LinRes
0	0	- out	5	3	5			0	9.6e-16	1.5e-15
1	2.6321e-08	2.6321e-08	7	4	7	1	0	0	3.5e-16	1e-15
2	5.2641e-08	2.6321e-08	9	5	9	1	0	0	3.7e-16	1.2e-15
3	1.0528e-07	5.2641e-08	11	6	11	2	0	0	4e-16	1.4e-15
4	2.1056e-07	1.0528e-07	13	7	13	2	0	0	4.6e-16	1.8e-15
5	4.2113e-07	2.1056e-07	15	8	15	2	0	0	6e-16	3.3e-15
6	6.3169e-07	2.1056e-07	17	9	17	2	0	0	6e-16	3.8e-15
7	8.4226e-07	2.1056e-07	19	10	19	2	0	0	6.8e-16	3.8e-15
8	1.0528e-06	2.1056e-07	21	11	21	2	0	0	6.8e-16	4e-15
9	1.474e-06	4.2113e-07	23	12	23	2	0	0	9.4e-16	6.5e-15
10	1.8951e-06	4.2113e-07	25	13	25	2	0	0	9.3e-16	5.9e-15
11	2.3162e-06	4.2113e-07	27	14	27	2	0	0	8.1e-16	6.1e-15
12	2.7373e-06	4.2113e-07	29	15	29	2	0	0	8.7e-16	5.6e-15
13	3.5796e-06	8.4226e-07	31	16	31	2	0	0	1.1e-15	8.9e-15
14	4.4219e-06	8.4226e-07	33	17	33	2	0	0	1.1e-15	7.5e-15
15	5.2641e-06	8.4226e-07	35	18	35	2	0	0	9.6e-16	7.6e-15
16	6.1064e-06	8.4226e-07	37	19	37	2	0	0	8.9e-16	6.9e-15
17	7.7909e-06	1.6845e-06	39	20	39	2	0	0	1.4e-15	9.9e-15
18	9.4754e-06	1.6845e-06	41	21	41	2	0	0	1.1e-15	8.5e-15
19	1.116e-05	1.6845e-06	43	22	43	2	0	0	1.2e-15	8.5e-15
20	1.2844e-05	1.6845e-06	45	23	45	2	0	0	1.2e-15	7.5e-15
21	1.4529e-05	1.6845e-06	47	24	47	2	0	0	1.3e-15	6.7e-15
22	1.7898e-05	3.369e-06	49	25	49	2	0	0	1.8e-15	9.8e-15
23	2.1267e-05	3.369e-06	51	26	51	2	0	0	1.7e-15	8.7e-15
24	2.4636e-05	3.369e-06	53	27	53	2	0	0	1.8e-15	8.8e-15
25	2.8005e-05	3.369e-06	55	28	55	2	0	0	1.7e-15	8.1e-15
26	3.4743e-05	6.7381e-06	57	29	57	2	0	0	1.4e-15	1.2e-14
27	4.1481e-05	6.7381e-06	59	30	59	2	0	0	1.4e-15	1e-14
28	4.8219e-05	6.7381e-06	61	31	61	2	0	0	2.1e-15	1e-14
29	5.4957e-05	6.7381e-06	63	32	63	2	0	0	1.3e-15	9.4e-15
30	6.1695e-05	6.7381e-06	65	33	65	2	0	0	2e-15	8.4e-15
31	7.5171e-05	1.3476e-05	67	34	67	2	0	0	2.2e-15	1.2e-14
32	8.8648e-05	1.3476e-05	69	35	69	2	0	0	2.1e-15	1.1e-14
33	0.00010212	1.3476e-05	71	36	71	2	0	0	2.1e-15	1.1e-14
34	0.0001156	1.3476e-05	73	37	73	2	0	0	2.2e-15	1e-14
35	0.00014255	2.6952e-05	75	38	75	2	0	0	1.4e-15	1.5e-14
36	0.0001695	2.6952e-05	77	39	77	2	0	0	2.3e-15	1.2e-14
37	0.00019646	2.6952e-05	79	40	79	2	0	0	2.9e-15	1.3e-14
38	0.00022341	2.6952e-05	81	41	81	2	0	0	2.8e-15	1.2e-14
39	0.00027731	5.3904e-05	83	42	83	2	0	0	2.8e-15	1.7e-14

40	0.00033122	5.3904e-05		85	43	85	2	0	0	3.4e-15	1.4e-14
41	0.00038512	5.3904e-05		87	44	87	2	0	0	3.9e-15	1.4e-14
42	0.00049293	0.00010781		89	45	89	2	0	0	2.7e-15	1.9e-14
43	0.00060074	0.00010781		91	46	91	2	0	0	5e-15	1.6e-14
44	0.00070855	0.00010781		93	47	93	2	0	0	4.8e-15	1.6e-14
45	0.00081636	0.00010781		95	48	95	2	0	0	1.9e-15	1.5e-14
46	0.001032	0.00021562		97	49	97	2	0	0	1.9e-15	1.9e-14
47	0.0012476	0.00021562		99	50	99	2	0	0	2.2e-15	1.6e-14
48	0.0016788	0.00043124		101	51	101	2	0	0	6.9e-15	2.6e-14
49	0.0021101	0.00043124		103	52	103	2	0	0	5.9e-15	2.2e-14
50	0.0025413	0.00043124		105	53	105	2	0	0	4.6e-15	2.3e-14
51	0.0029725	0.00043124		107	54	107	2	0	0	3.4e-15	2e-14
52	0.003835	0.00086247		109	55	109	2	0	0	2e-15	2.6e-14
53	0.0046975	0.00086247		111	56	111	2	0	0	2.6e-15	2.1e-14
-	0.005	-	out								
54	0.0064224	0.0017249		113	57	113	2	0	0	7.6e-15	3.4e-14
55	0.0081474	0.0017249		115	58	115	2	0	0	1.3e-15	2.9e-14
-	0.01	-	out								
56	0.011597	0.0034499		117	59	117	2	0	0	2.9e-15	4.3e-14
-	0.015	-	out								
57	0.015047	0.0034499		119	60	119	1	0	0	9.4e-15	5e-14
58	0.018497	0.0034499		121	61	121	1	0	0	2.8e-15	4.1e-14
-	0.02	-	out								
-	0.025	-	out								
59	0.025397	0.0068998		123	62	123	1	0	0	4.3e-15	6.8e-14
-	0.03	-	out								
60	0.032297	0.0068998		125	63	125	1	0	0	3.6e-15	6.4e-14
-	0.035	-	out								
-	0.04	-	out								
-	0.045	-	out								
61	0.046096	0.0138		127	64	127	1	0	0	6.3e-15	9e-14
-	0.05	-	out								
-	0.055	-	out								
62	0.059896	0.0138		129	65	129	1	0	0	3.6e-15	8.3e-14
-	0.06	-	out								
-	0.065	-	out								
-	0.07	-	out								
-	0.075	-	out								
-	0.08	-	out								
-	0.085	-	out								
63	0.087495	0.027599		131	66	131	1	0	0	7.2e-15	1.1e-13
-	0.09	-	out								
-	0.095	-	out								
-	0.1	-	out								
-	0.105	-	out								
-	0.11	-	out								
-	0.115	-	out								
64	0.11509	0.027599		133	67	133	1	0	0	3.4e-15	8e-14
-	0.12	-	out								
-	0.125	-	out								
-	0.13	-	out								
-	0.135	-	out								
-	0.14	-	out								
-	0.145	-	out								
-	0.15	-	out								
-	0.155	-	out								

-	0.16	-	out							
-	0.165	-	out							
-	0.17	-	out							
65	0.17029	0.055198	135	68	135	1	0	0	5.9e-15	8.9e-14
-	0.175	-	out							
-	0.18	-	out							
-	0.185	-	out							
-	0.19	-	out							
-	0.195	-	out							
-	0.2	-	out							
-	0.205	-	out							
-	0.21	-	out							
-	0.215	-	out							
-	0.22	-	out							
-	0.225	-	out							
66	0.22549	0.055198	137	69	137	1	0	0	4.2e-15	6.8e-14
-	0.23	-	out							
-	0.235	-	out							
-	0.24	-	out							
-	0.245	-	out							
-	0.25	-	out							
-	0.25	-	out							
-	0.27	-	out							
-	0.29	-	out							
-	0.31	-	out							
67	0.32449	0.099	139	70	139	1	0	0	1.1e-14	6.6e-14
-	0.33	-	out							
-	0.35	-	out							
-	0.37	-	out							
-	0.39	-	out							
-	0.41	-	out							
68	0.42349	0.099	141	71	141	1	0	0	1.3e-14	5.1e-14
-	0.43	-	out							
-	0.45	-	out							
-	0.47	-	out							
-	0.49	-	out							
-	0.51	-	out							
69	0.52249	0.099	143	72	143	1	0	0	9.5e-15	3.8e-14
-	0.53	-	out							
-	0.55	-	out							
-	0.57	-	out							
-	0.59	-	out							
-	0.61	-	out							
70	0.62149	0.099	145	73	145	1	0	0	2e-14	3.1e-14
-	0.63	-	out							
-	0.65	-	out							
-	0.67	-	out							
-	0.69	-	out							
-	0.71	-	out							
71	0.72049	0.099	146	74	146	1	0	0	9.2e-15	2.5e-14
-	0.73	-	out							
-	0.75	-	out							
-	0.77	-	out							
-	0.79	-	out							
-	0.81	-	out							
72	0.81949	0.099	147	75	147	1	0	0	9.5e-15	2.2e-14

```
   -        0.83           - out
   -        0.85           - out
   -        0.87           - out
   -        0.89           - out
   -        0.91           - out
  73     0.91849       0.099      148    76   148      2      0       0  3.8e-15  1.8e-14
   -        0.93           - out
   -        0.95           - out
   -        0.97           - out
   -        0.99           - out
  74      1.0175       0.099      149    77   149      2      0       0  2.2e-15  1.2e-14
Time-stepping completed.
Solution time: 14 s.
Physical memory: 1.14 GB
Virtual memory: 1.12 GB
Ended at May 28, 2023, 4:04:46 PM.
----- Time-Dependent Solver 1 in Oxygen Pulse/Solution 958 (sol958) ----------->
```

k0Sub=0, d=6E-6 (su9)

GENERAL

Description	Value
Solution	k0Sub=0, d=6E-6

LOG

```
<---- Compile Equations: Stationary in Oxygen Pulse/Solution 958 (sol958) ------
Started at May 28, 2023, 4:04:48 PM.
Parameter k0Sub = 0.0 (m/s).
Parameter d = 6.0E-6 (m).
Geometry shape function: Linear Lagrange
Number of vertex elements: 10
Number of boundary elements: 894
Number of elements: 35904
Minimum element quality: 0.432
Number of vertex elements: 10
Minimum element quality: 0.432
Time: 2 s.
Physical memory: 1.13 GB
Virtual memory: 1.11 GB
Ended at May 28, 2023, 4:04:50 PM.
----- Compile Equations: Stationary in Oxygen Pulse/Solution 958 (sol958) ----->

<---- Dependent Variables 1 in Oxygen Pulse/Solution 958 (sol958) --------------
Started at May 28, 2023, 4:04:50 PM.
Solution time: 0 s.
Physical memory: 1.13 GB
Virtual memory: 1.11 GB
Ended at May 28, 2023, 4:04:50 PM.
----- Dependent Variables 1 in Oxygen Pulse/Solution 958 (sol958) ------------->

<---- Stationary Solver 1 in Oxygen Pulse/Solution 958 (sol958) ----------------
Started at May 28, 2023, 4:04:51 PM.
Linear solver
Number of degrees of freedom solved for: 18385 (plus 936 internal DOFs).
Symmetric matrices found.
Scales for dependent variables:
Concentration (comp2.Oxi): 0.23
Orthonormal null-space function used.
Iter      SolEst     Damping    Stepsize #Res #Jac #Sol   LinErr   LinRes
   1     2.7e-16   1.0000000     2.7e-16    1    1    1  5.6e-14    3e-16
Solution time: 0 s.
Physical memory: 1.13 GB
Virtual memory: 1.11 GB
Ended at May 28, 2023, 4:04:51 PM.
----- Stationary Solver 1 in Oxygen Pulse/Solution 958 (sol958) --------------->

<---- Compile Equations: Oxygen Probe Pulse in Oxygen Pulse/Solution 958
      (sol958) -----------------------------------------------------------------
Started at May 28, 2023, 4:04:51 PM.
Geometry shape function: Linear Lagrange
Time: 0 s.
Physical memory: 1.1 GB
Virtual memory: 1.08 GB
Ended at May 28, 2023, 4:04:52 PM.
----- Compile Equations: Oxygen Probe Pulse in Oxygen Pulse/Solution 958
      (sol958) ---------------------------------------------------------------->

<---- Dependent Variables 2 in Oxygen Pulse/Solution 958 (sol958) --------------
Started at May 28, 2023, 4:04:52 PM.
Initial values of variables solved for: Solution 958 (sol958), Solution Store 1 (sol
985).
```

Values of variables not solved for: Solution 958 (sol958), Solution Store 1 (sol985)
.
Solution time: 0 s.
Physical memory: 1.11 GB
Virtual memory: 1.08 GB
Ended at May 28, 2023, 4:04:52 PM.
----- Dependent Variables 2 in Oxygen Pulse/Solution 958 (sol958) ------------->

<---- Time-Dependent Solver 1 in Oxygen Pulse/Solution 958 (sol958) ------------
Started at May 28, 2023, 4:04:52 PM.
Time-dependent solver (BDF)
Number of degrees of freedom solved for: 18385 (plus 36840 internal DOFs).
Symmetric matrices found.
Scales for dependent variables:
Concentration (comp2.Oxi): 0.23

Step	Time	Stepsize	Res	Jac	Sol	Order	Tfail	NLfail	LinErr	LinRes
0	0	- out	5	3	5			0	8.2e-16	1.4e-15
1	2.6429e-08	2.6429e-08	7	4	7	1	0	0	3.5e-16	1.1e-15
2	5.2857e-08	2.6429e-08	9	5	9	1	0	0	3.6e-16	1.1e-15
3	1.0571e-07	5.2857e-08	11	6	11	2	0	0	3.3e-16	1.3e-15
4	2.1143e-07	1.0571e-07	13	7	13	2	0	0	4.7e-16	1.6e-15
5	4.2286e-07	2.1143e-07	15	8	15	2	0	0	5.9e-16	3.3e-15
6	6.3429e-07	2.1143e-07	17	9	17	2	0	0	8.1e-16	3.8e-15
7	8.4572e-07	2.1143e-07	19	10	19	2	0	0	8.3e-16	3.9e-15
8	1.0571e-06	2.1143e-07	21	11	21	2	0	0	6.4e-16	3.9e-15
9	1.48e-06	4.2286e-07	23	12	23	2	0	0	7.4e-16	6.2e-15
10	1.9029e-06	4.2286e-07	25	13	25	2	0	0	9.2e-16	5.8e-15
11	2.3257e-06	4.2286e-07	27	14	27	2	0	0	8.8e-16	6e-15
12	2.7486e-06	4.2286e-07	29	15	29	2	0	0	7.8e-16	5.7e-15
13	3.5943e-06	8.4572e-07	31	16	31	2	0	0	1e-15	8.8e-15
14	4.44e-06	8.4572e-07	33	17	33	2	0	0	8.6e-16	7.3e-15
15	5.2857e-06	8.4572e-07	35	18	35	2	0	0	1.2e-15	7.5e-15
16	6.1315e-06	8.4572e-07	37	19	37	2	0	0	1.3e-15	6.6e-15
17	7.8229e-06	1.6914e-06	39	20	39	2	0	0	2e-15	9.7e-15
18	9.5143e-06	1.6914e-06	41	21	41	2	0	0	1.2e-15	8.1e-15
19	1.1206e-05	1.6914e-06	43	22	43	2	0	0	1.2e-15	8e-15
20	1.2897e-05	1.6914e-06	45	23	45	2	0	0	1e-15	7.2e-15
21	1.4589e-05	1.6914e-06	47	24	47	2	0	0	1.5e-15	6.4e-15
22	1.7971e-05	3.3829e-06	49	25	49	2	0	0	2.7e-15	9.6e-15
23	2.1354e-05	3.3829e-06	51	26	51	2	0	0	1.5e-15	8.3e-15
24	2.4737e-05	3.3829e-06	53	27	53	2	0	0	1.8e-15	8.3e-15
25	2.812e-05	3.3829e-06	55	28	55	2	0	0	1.3e-15	7.9e-15
26	3.4886e-05	6.7657e-06	57	29	57	2	0	0	2.5e-15	1.2e-14
27	4.1652e-05	6.7657e-06	59	30	59	2	0	0	1.8e-15	1e-14
28	4.8417e-05	6.7657e-06	61	31	61	2	0	0	1.8e-15	1e-14
29	5.5183e-05	6.7657e-06	63	32	63	2	0	0	1.6e-15	9.2e-15
30	6.1949e-05	6.7657e-06	65	33	65	2	0	0	1.2e-15	8.4e-15
31	7.548e-05	1.3531e-05	67	34	67	2	0	0	1.5e-15	1.2e-14
32	8.9012e-05	1.3531e-05	69	35	69	2	0	0	1.7e-15	1.1e-14
33	0.00010254	1.3531e-05	71	36	71	2	0	0	1.5e-15	1.1e-14
34	0.00011607	1.3531e-05	73	37	73	2	0	0	1.5e-15	9.9e-15
35	0.00014314	2.7063e-05	75	38	75	2	0	0	3e-15	1.5e-14
36	0.0001702	2.7063e-05	77	39	77	2	0	0	3.7e-15	1.3e-14
37	0.00019726	2.7063e-05	79	40	79	2	0	0	2e-15	1.3e-14
38	0.00022433	2.7063e-05	81	41	81	2	0	0	1.9e-15	1.1e-14
39	0.00027845	5.4126e-05	83	42	83	2	0	0	2.1e-15	1.7e-14

40	0.00033258	5.4126e-05		85	43	85	2	0	0	3.7e-15	1.4e-14
41	0.0003867	5.4126e-05		87	44	87	2	0	0	2.9e-15	1.4e-14
42	0.00049496	0.00010825		89	45	89	2	0	0	2.9e-15	2e-14
43	0.00060321	0.00010825		91	46	91	2	0	0	2.8e-15	1.7e-14
44	0.00071146	0.00010825		93	47	93	2	0	0	1.5e-15	1.7e-14
45	0.00081971	0.00010825		95	48	95	2	0	0	2.3e-15	1.4e-14
46	0.0010362	0.0002165		97	49	97	2	0	0	4.4e-15	2e-14
47	0.0012527	0.0002165		99	50	99	2	0	0	3.8e-15	1.6e-14
48	0.0016857	0.00043301		101	51	101	2	0	0	3.4e-15	2.6e-14
49	0.0021187	0.00043301		103	52	103	2	0	0	3.4e-15	2.3e-14
50	0.0025517	0.00043301		105	53	105	2	0	0	2.7e-15	2.3e-14
51	0.0029847	0.00043301		107	54	107	2	0	0	4.9e-15	2e-14
52	0.0038508	0.00086601		109	55	109	2	0	0	5.6e-15	2.6e-14
53	0.0047168	0.00086601		111	56	111	2	0	0	6.6e-15	2e-14
-	0.005	-	out								
54	0.0064488	0.001732		113	57	113	2	0	0	4e-15	3.4e-14
55	0.0081808	0.001732		115	58	115	2	0	0	2.6e-15	3.1e-14
-	0.01	-	out								
56	0.011645	0.0034641		117	59	117	2	0	0	1.9e-15	4.4e-14
-	0.015	-	out								
57	0.015109	0.0034641		119	60	119	1	0	0	3.7e-15	4.8e-14
58	0.018573	0.0034641		121	61	121	1	0	0	5.1e-15	3.9e-14
-	0.02	-	out								
-	0.025	-	out								
59	0.025501	0.0069281		123	62	123	1	0	0	7.7e-15	6.3e-14
-	0.03	-	out								
60	0.032429	0.0069281		125	63	125	1	0	0	4.2e-15	6.1e-14
-	0.035	-	out								
-	0.04	-	out								
-	0.045	-	out								
61	0.046285	0.013856		127	64	127	1	0	0	6.7e-15	8.8e-14
-	0.05	-	out								
-	0.055	-	out								
-	0.06	-	out								
62	0.060142	0.013856		129	65	129	1	0	0	2.3e-15	7.6e-14
-	0.065	-	out								
-	0.07	-	out								
-	0.075	-	out								
-	0.08	-	out								
-	0.085	-	out								
63	0.087854	0.027712		131	66	131	1	0	0	1.1e-14	1e-13
-	0.09	-	out								
-	0.095	-	out								
-	0.1	-	out								
-	0.105	-	out								
-	0.11	-	out								
-	0.115	-	out								
64	0.11557	0.027712		133	67	133	1	0	0	9.4e-15	8.2e-14
-	0.12	-	out								
-	0.125	-	out								
-	0.13	-	out								
-	0.135	-	out								
-	0.14	-	out								
-	0.145	-	out								
-	0.15	-	out								
-	0.155	-	out								

-	0.16	- out								
-	0.165	- out								
-	0.17	- out								
65	0.17099	0.055425	135	68	135	1	0	0	3.8e-15	9.3e-14
-	0.175	- out								
-	0.18	- out								
-	0.185	- out								
-	0.19	- out								
-	0.195	- out								
-	0.2	- out								
-	0.205	- out								
-	0.21	- out								
-	0.215	- out								
-	0.22	- out								
-	0.225	- out								
66	0.22642	0.055425	137	69	137	1	0	0	3.8e-15	6.9e-14
-	0.23	- out								
-	0.235	- out								
-	0.24	- out								
-	0.245	- out								
-	0.25	- out								
-	0.25	- out								
-	0.27	- out								
-	0.29	- out								
-	0.31	- out								
67	0.32542	0.099	139	70	139	1	0	0	4.4e-15	7.1e-14
-	0.33	- out								
-	0.35	- out								
-	0.37	- out								
-	0.39	- out								
-	0.41	- out								
68	0.42442	0.099	141	71	141	1	0	0	2.8e-14	5.4e-14
-	0.43	- out								
-	0.45	- out								
-	0.47	- out								
-	0.49	- out								
-	0.51	- out								
69	0.52342	0.099	143	72	143	1	0	0	1e-14	4.2e-14
-	0.53	- out								
-	0.55	- out								
-	0.57	- out								
-	0.59	- out								
-	0.61	- out								
70	0.62242	0.099	145	73	145	1	0	0	1.4e-14	3.2e-14
-	0.63	- out								
-	0.65	- out								
-	0.67	- out								
-	0.69	- out								
-	0.71	- out								
71	0.72142	0.099	146	74	146	1	0	0	1e-14	2.6e-14
-	0.73	- out								
-	0.75	- out								
-	0.77	- out								
-	0.79	- out								
-	0.81	- out								
72	0.82042	0.099	147	75	147	1	0	0	7.9e-15	2.3e-14

```
  -        0.83           - out
  -        0.85           - out
  -        0.87           - out
  -        0.89           - out
  -        0.91           - out
 73     0.91942       0.099      148   76  148     2     0      0  7.3e-15  1.8e-14
  -        0.93           - out
  -        0.95           - out
  -        0.97           - out
  -        0.99           - out
 74      1.0184       0.099      149   77  149     2     0      0  4.3e-15  1.2e-14
Time-stepping completed.
Solution time: 13 s.
Physical memory: 1.16 GB
Virtual memory: 1.14 GB
Ended at May 28, 2023, 4:05:05 PM.
----- Time-Dependent Solver 1 in Oxygen Pulse/Solution 958 (sol958) ----------->
```

k0Sub=0, d=8E-6 (su10)

GENERAL

Description	Value
Solution	k0Sub=0, d=8E-6

LOG

```
<---- Compile Equations: Stationary in Oxygen Pulse/Solution 958 (sol958) ------
Started at May 28, 2023, 4:05:07 PM.
Parameter k0Sub = 0.0 (m/s).
Parameter d = 8.0E-6 (m).
Geometry shape function: Linear Lagrange
Number of vertex elements: 10
Number of boundary elements: 899
Number of elements: 46729
Minimum element quality: 0.4468
Number of vertex elements: 10
Minimum element quality: 0.4468
Time: 5 s.
Physical memory: 1.16 GB
Virtual memory: 1.14 GB
Ended at May 28, 2023, 4:05:12 PM.
----- Compile Equations: Stationary in Oxygen Pulse/Solution 958 (sol958) ----->

<---- Dependent Variables 1 in Oxygen Pulse/Solution 958 (sol958) --------------
Started at May 28, 2023, 4:05:12 PM.
Solution time: 0 s.
Physical memory: 1.16 GB
Virtual memory: 1.14 GB
Ended at May 28, 2023, 4:05:12 PM.
----- Dependent Variables 1 in Oxygen Pulse/Solution 958 (sol958) ------------->

<---- Stationary Solver 1 in Oxygen Pulse/Solution 958 (sol958) ----------------
Started at May 28, 2023, 4:05:13 PM.
Linear solver
Number of degrees of freedom solved for: 23800 (plus 941 internal DOFs).
Symmetric matrices found.
Scales for dependent variables:
Concentration (comp2.Oxi): 0.23
Orthonormal null-space function used.
Iter      SolEst     Damping    Stepsize #Res #Jac #Sol   LinErr   LinRes
   1     2.3e-16   1.0000000     2.3e-16    1    1    1  6.4e-14  2.8e-16
Solution time: 1 s.
Physical memory: 1.16 GB
Virtual memory: 1.13 GB
Ended at May 28, 2023, 4:05:13 PM.
----- Stationary Solver 1 in Oxygen Pulse/Solution 958 (sol958) --------------->

<---- Compile Equations: Oxygen Probe Pulse in Oxygen Pulse/Solution 958
      (sol958) ----------------------------------------------------------------
Started at May 28, 2023, 4:05:13 PM.
Geometry shape function: Linear Lagrange
Time: 1 s.
Physical memory: 1.11 GB
Virtual memory: 1.09 GB
Ended at May 28, 2023, 4:05:14 PM.
----- Compile Equations: Oxygen Probe Pulse in Oxygen Pulse/Solution 958
      (sol958) --------------------------------------------------------------->

<---- Dependent Variables 2 in Oxygen Pulse/Solution 958 (sol958) --------------
Started at May 28, 2023, 4:05:14 PM.
Initial values of variables solved for: Solution 958 (sol958), Solution Store 1 (sol
985).
```

Values of variables not solved for: Solution 958 (sol958), Solution Store 1 (sol985)
.
Solution time: 0 s.
Physical memory: 1.11 GB
Virtual memory: 1.09 GB
Ended at May 28, 2023, 4:05:14 PM.
----- Dependent Variables 2 in Oxygen Pulse/Solution 958 (sol958) ------------->

<---- Time-Dependent Solver 1 in Oxygen Pulse/Solution 958 (sol958) ------------
Started at May 28, 2023, 4:05:14 PM.
Time-dependent solver (BDF)
Number of degrees of freedom solved for: 23800 (plus 47670 internal DOFs).
Symmetric matrices found.
Scales for dependent variables:
Concentration (comp2.Oxi): 0.23

Step	Time	Stepsize	Res	Jac	Sol	Order	Tfail	NLfail	LinErr	LinRes
0	0	- out	5	3	5			0	5.1e-16	9.2e-16
1	3.6251e-08	3.6251e-08	7	4	7	1	0	0	3.2e-16	9.6e-16
2	7.2501e-08	3.6251e-08	9	5	9	1	0	0	3.2e-16	9.5e-16
3	1.45e-07	7.2501e-08	11	6	11	2	0	0	3.4e-16	1.1e-15
4	2.9e-07	1.45e-07	13	7	13	2	0	0	4.2e-16	1.3e-15
5	5.8001e-07	2.9e-07	15	8	15	2	0	0	4.7e-16	2.5e-15
6	8.7001e-07	2.9e-07	17	9	17	2	0	0	6.6e-16	2.9e-15
7	1.16e-06	2.9e-07	19	10	19	2	0	0	5.6e-16	3.2e-15
8	1.45e-06	2.9e-07	21	11	21	2	0	0	4.9e-16	3.2e-15
9	2.03e-06	5.8001e-07	23	12	23	2	0	0	7.2e-16	5.1e-15
10	2.61e-06	5.8001e-07	25	13	25	2	0	0	6.6e-16	4.6e-15
11	3.1901e-06	5.8001e-07	27	14	27	2	0	0	5.9e-16	4.6e-15
12	3.7701e-06	5.8001e-07	29	15	29	2	0	0	6.2e-16	4.3e-15
13	4.9301e-06	1.16e-06	31	16	31	2	0	0	1e-15	7e-15
14	6.0901e-06	1.16e-06	33	17	33	2	0	0	9.7e-16	6.4e-15
15	7.2501e-06	1.16e-06	35	18	35	2	0	0	9.3e-16	6e-15
16	8.4101e-06	1.16e-06	37	19	37	2	0	0	9.8e-16	5.6e-15
17	1.073e-05	2.32e-06	39	20	39	2	0	0	1.1e-15	8.6e-15
18	1.305e-05	2.32e-06	41	21	41	2	0	0	9.9e-16	7.7e-15
19	1.537e-05	2.32e-06	43	22	43	2	0	0	1.2e-15	7.5e-15
20	1.769e-05	2.32e-06	45	23	45	2	0	0	1.2e-15	7.3e-15
21	2.233e-05	4.6401e-06	47	24	47	2	0	0	1.4e-15	1.1e-14
22	2.697e-05	4.6401e-06	49	25	49	2	0	0	1.1e-15	9.8e-15
23	3.161e-05	4.6401e-06	51	26	51	2	0	0	1.2e-15	9.4e-15
24	3.6251e-05	4.6401e-06	53	27	53	2	0	0	1.1e-15	8.7e-15
25	4.5531e-05	9.2801e-06	55	28	55	2	0	0	1.5e-15	1.3e-14
26	5.4811e-05	9.2801e-06	57	29	57	2	0	0	1.6e-15	1.2e-14
27	6.4091e-05	9.2801e-06	59	30	59	2	0	0	1.6e-15	1.1e-14
28	7.3371e-05	9.2801e-06	61	31	61	2	0	0	1.1e-15	1e-14
29	9.1931e-05	1.856e-05	63	32	63	2	0	0	2e-15	1.5e-14
30	0.00011049	1.856e-05	65	33	65	2	0	0	2.8e-15	1.3e-14
31	0.00012905	1.856e-05	67	34	67	2	0	0	3.9e-15	1.4e-14
32	0.00014761	1.856e-05	69	35	69	2	0	0	2.1e-15	1.3e-14
33	0.00018473	3.7121e-05	71	36	71	2	0	0	2.3e-15	1.8e-14
34	0.00022185	3.7121e-05	73	37	73	2	0	0	1.5e-15	1.5e-14
35	0.00025897	3.7121e-05	75	38	75	2	0	0	2.3e-15	1.6e-14
36	0.00029609	3.7121e-05	77	39	77	2	0	0	2.7e-15	1.4e-14
37	0.00037034	7.4241e-05	79	40	79	2	0	0	3.7e-15	2e-14
38	0.00044458	7.4241e-05	81	41	81	2	0	0	3.3e-15	1.6e-14
39	0.00059306	0.00014848	83	42	83	2	0	0	4e-15	2.7e-14

40	0.00074154	0.00014848		85	43	85	2	0	0	5.7e-15	2.3e-14
41	0.00089002	0.00014848		87	44	87	2	0	0	2e-15	2.4e-14
42	0.0010385	0.00014848		89	45	89	2	0	0	4.3e-15	2e-14
43	0.0013355	0.00029696		91	46	91	2	0	0	6.8e-15	2.6e-14
44	0.0016324	0.00029696		93	47	93	2	0	0	3.4e-15	1.9e-14
45	0.0022264	0.00059393		95	48	95	2	0	0	3.3e-15	3.2e-14
46	0.0028203	0.00059393		97	49	97	2	0	0	1.9e-15	2.7e-14
47	0.0034142	0.00059393		99	50	99	2	0	0	2.6e-15	2.8e-14
48	0.0046021	0.0011879		101	51	101	2	0	0	1.9e-15	3.7e-14
-	0.005	-	out								
49	0.0057899	0.0011879		103	52	103	2	0	0	2.2e-15	2.6e-14
50	0.0081657	0.0023757		105	53	105	2	0	0	2.4e-15	4.3e-14
-	0.01	-	out								
51	0.010541	0.0023757		107	54	107	1	0	0	3.7e-15	4.9e-14
52	0.012917	0.0023757		109	55	109	1	0	0	6.1e-15	3.4e-14
-	0.015	-	out								
53	0.017669	0.0047514		111	56	111	1	0	0	4.4e-15	5.4e-14
-	0.02	-	out								
-	0.025	-	out								
54	0.027171	0.0095029		113	57	113	1	0	0	6.1e-15	8.7e-14
-	0.03	-	out								
-	0.035	-	out								
55	0.036674	0.0095029		115	58	115	1	0	0	8e-15	8.3e-14
-	0.04	-	out								
-	0.045	-	out								
-	0.05	-	out								
-	0.055	-	out								
56	0.05568	0.019006		117	59	117	1	0	0	5.1e-15	1.2e-13
-	0.06	-	out								
-	0.065	-	out								
-	0.07	-	out								
57	0.074686	0.019006		119	60	119	1	0	0	4.9e-15	1.2e-13
-	0.075	-	out								
-	0.08	-	out								
-	0.085	-	out								
-	0.09	-	out								
-	0.095	-	out								
-	0.1	-	out								
-	0.105	-	out								
-	0.11	-	out								
58	0.1127	0.038011		121	61	121	1	0	0	5.6e-15	1.4e-13
-	0.115	-	out								
-	0.12	-	out								
-	0.125	-	out								
-	0.13	-	out								
-	0.135	-	out								
-	0.14	-	out								
-	0.145	-	out								
-	0.15	-	out								
59	0.15071	0.038011		123	62	123	1	0	0	4e-15	1.1e-13
-	0.155	-	out								
-	0.16	-	out								
-	0.165	-	out								
-	0.17	-	out								
-	0.175	-	out								
-	0.18	-	out								

-	0.185	-	out							
-	0.19	-	out							
-	0.195	-	out							
-	0.2	-	out							
-	0.205	-	out							
-	0.21	-	out							
-	0.215	-	out							
-	0.22	-	out							
-	0.225	-	out							
60	0.22673	0.076023	125	63	125	1	0	0	1e-14	1.2e-13
-	0.23	-	out							
-	0.235	-	out							
-	0.24	-	out							
-	0.245	-	out							
-	0.25	-	out							
-	0.25	-	out							
-	0.27	-	out							
-	0.29	-	out							
61	0.30275	0.076023	127	64	127	1	0	0	5.9e-15	9.5e-14
-	0.31	-	out							
-	0.33	-	out							
-	0.35	-	out							
-	0.37	-	out							
-	0.39	-	out							
62	0.40175	0.099	129	65	129	1	0	0	4.2e-15	8.5e-14
-	0.41	-	out							
-	0.43	-	out							
-	0.45	-	out							
-	0.47	-	out							
-	0.49	-	out							
63	0.50075	0.099	131	66	131	1	0	0	7.2e-15	6.2e-14
-	0.51	-	out							
-	0.53	-	out							
-	0.55	-	out							
-	0.57	-	out							
-	0.59	-	out							
64	0.59975	0.099	133	67	133	1	0	0	1.1e-14	5e-14
-	0.61	-	out							
-	0.63	-	out							
-	0.65	-	out							
-	0.67	-	out							
-	0.69	-	out							
65	0.69875	0.099	134	68	134	1	0	0	1.3e-14	3.9e-14
-	0.71	-	out							
-	0.73	-	out							
-	0.75	-	out							
-	0.77	-	out							
-	0.79	-	out							
66	0.79775	0.099	135	69	135	1	0	0	4.8e-15	3.6e-14
-	0.81	-	out							
-	0.83	-	out							
-	0.85	-	out							
-	0.87	-	out							
-	0.89	-	out							
67	0.89675	0.099	136	70	136	2	0	0	5.7e-15	2.8e-14
-	0.91	-	out							

```
   -        0.93           - out
   -        0.95           - out
   -        0.97           - out
   -        0.99           - out
  68     0.99575       0.099      137    71   137      2      0       0  7.1e-15  1.9e-14
Time-stepping completed.
Solution time: 15 s.
Physical memory: 1.17 GB
Virtual memory: 1.15 GB
Ended at May 28, 2023, 4:05:29 PM.
----- Time-Dependent Solver 1 in Oxygen Pulse/Solution 958 (sol958) ----------->
```

k0Sub=0, d=1.2E-5 (su11)

GENERAL

Description	Value
Solution	k0Sub=0, d=1.2E-5

LOG

```
<---- Compile Equations: Stationary in Oxygen Pulse/Solution 958 (sol958) ------
Started at May 28, 2023, 4:05:32 PM.
Parameter k0Sub = 0.0 (m/s).
Parameter d = 1.2E-5 (m).
Geometry shape function: Linear Lagrange
Number of vertex elements: 10
Number of boundary elements: 906
Number of elements: 36660
Minimum element quality: 0.4505
Number of vertex elements: 10
Minimum element quality: 0.4505
Time: 2 s.
Physical memory: 1.17 GB
Virtual memory: 1.15 GB
Ended at May 28, 2023, 4:05:34 PM.
----- Compile Equations: Stationary in Oxygen Pulse/Solution 958 (sol958) ----->

<---- Dependent Variables 1 in Oxygen Pulse/Solution 958 (sol958) --------------
Started at May 28, 2023, 4:05:34 PM.
Solution time: 0 s.
Physical memory: 1.11 GB
Virtual memory: 1.08 GB
Ended at May 28, 2023, 4:05:34 PM.
----- Dependent Variables 1 in Oxygen Pulse/Solution 958 (sol958) ------------->

<---- Stationary Solver 1 in Oxygen Pulse/Solution 958 (sol958) ----------------
Started at May 28, 2023, 4:05:34 PM.
Linear solver
Number of degrees of freedom solved for: 18769 (plus 948 internal DOFs).
Symmetric matrices found.
Scales for dependent variables:
Concentration (comp2.Oxi): 0.23
Orthonormal null-space function used.
Iter      SolEst     Damping    Stepsize #Res #Jac #Sol   LinErr   LinRes
   1     2.1e-16   1.0000000     2.1e-16    1    1    1    2e-14    3e-16
Solution time: 0 s.
Physical memory: 1.13 GB
Virtual memory: 1.11 GB
Ended at May 28, 2023, 4:05:35 PM.
----- Stationary Solver 1 in Oxygen Pulse/Solution 958 (sol958) --------------->

<---- Compile Equations: Oxygen Probe Pulse in Oxygen Pulse/Solution 958
      (sol958) -----------------------------------------------------------------
Started at May 28, 2023, 4:05:35 PM.
Geometry shape function: Linear Lagrange
Time: 1 s.
Physical memory: 1.15 GB
Virtual memory: 1.13 GB
Ended at May 28, 2023, 4:05:35 PM.
----- Compile Equations: Oxygen Probe Pulse in Oxygen Pulse/Solution 958
      (sol958) ---------------------------------------------------------------->

<---- Dependent Variables 2 in Oxygen Pulse/Solution 958 (sol958) --------------
Started at May 28, 2023, 4:05:35 PM.
Initial values of variables solved for: Solution 958 (sol958), Solution Store 1 (sol
985).
```

Values of variables not solved for: Solution 958 (sol958), Solution Store 1 (sol985)
.
Solution time: 0 s.
Physical memory: 1.15 GB
Virtual memory: 1.13 GB
Ended at May 28, 2023, 4:05:35 PM.
----- Dependent Variables 2 in Oxygen Pulse/Solution 958 (sol958) ------------->

<---- Time-Dependent Solver 1 in Oxygen Pulse/Solution 958 (sol958) ------------
Started at May 28, 2023, 4:05:35 PM.
Time-dependent solver (BDF)
Number of degrees of freedom solved for: 18769 (plus 37608 internal DOFs).
Symmetric matrices found.
Scales for dependent variables:
Concentration (comp2.Oxi): 0.23

Step	Time	Stepsize	Res	Jac	Sol	Order	Tfail	NLfail	LinErr	LinRes
0	0	- out	5	3	5			0	7e-16	1.4e-15
1	2.659e-08	2.659e-08	7	4	7	1	0	0	3.6e-16	1.1e-15
2	5.3179e-08	2.659e-08	9	5	9	1	0	0	3.4e-16	1.1e-15
3	1.0636e-07	5.3179e-08	11	6	11	2	0	0	4e-16	1.3e-15
4	2.1272e-07	1.0636e-07	13	7	13	2	0	0	5e-16	1.7e-15
5	4.2543e-07	2.1272e-07	15	8	15	2	0	0	7.6e-16	3.4e-15
6	6.3815e-07	2.1272e-07	17	9	17	2	0	0	6.2e-16	3.9e-15
7	8.5087e-07	2.1272e-07	19	10	19	2	0	0	7.5e-16	4e-15
8	1.0636e-06	2.1272e-07	21	11	21	2	0	0	5.8e-16	3.9e-15
9	1.489e-06	4.2543e-07	23	12	23	2	0	0	7e-16	6.6e-15
10	1.9145e-06	4.2543e-07	25	13	25	2	0	0	9.9e-16	6.1e-15
11	2.3399e-06	4.2543e-07	27	14	27	2	0	0	8.5e-16	6.1e-15
12	2.7653e-06	4.2543e-07	29	15	29	2	0	0	8.3e-16	5.6e-15
13	3.6162e-06	8.5087e-07	31	16	31	2	0	0	1.1e-15	8.5e-15
14	4.4671e-06	8.5087e-07	33	17	33	2	0	0	1.1e-15	7.2e-15
15	5.3179e-06	8.5087e-07	35	18	35	2	0	0	1e-15	7.1e-15
16	6.1688e-06	8.5087e-07	37	19	37	2	0	0	9.5e-16	6.5e-15
17	7.8705e-06	1.7017e-06	39	20	39	2	0	0	1.8e-15	9.7e-15
18	9.5723e-06	1.7017e-06	41	21	41	2	0	0	1.3e-15	8.3e-15
19	1.1274e-05	1.7017e-06	43	22	43	2	0	0	1.4e-15	8.3e-15
20	1.2976e-05	1.7017e-06	45	23	45	2	0	0	1.4e-15	7.4e-15
21	1.4677e-05	1.7017e-06	47	24	47	2	0	0	1.4e-15	6.6e-15
22	1.8081e-05	3.4035e-06	49	25	49	2	0	0	1.4e-15	9.7e-15
23	2.1484e-05	3.4035e-06	51	26	51	2	0	0	1.6e-15	8.4e-15
24	2.4888e-05	3.4035e-06	53	27	53	2	0	0	1.4e-15	8.2e-15
25	2.8291e-05	3.4035e-06	55	28	55	2	0	0	1.1e-15	7.7e-15
26	3.5098e-05	6.8069e-06	57	29	57	2	0	0	1.3e-15	1.1e-14
27	4.1905e-05	6.8069e-06	59	30	59	2	0	0	1.1e-15	9.9e-15
28	4.8712e-05	6.8069e-06	61	31	61	2	0	0	1.6e-15	9.7e-15
29	5.5519e-05	6.8069e-06	63	32	63	2	0	0	1.7e-15	8.5e-15
30	6.2326e-05	6.8069e-06	65	33	65	2	0	0	1.2e-15	7.9e-15
31	7.594e-05	1.3614e-05	67	34	67	2	0	0	1.3e-15	1.2e-14
32	8.9554e-05	1.3614e-05	69	35	69	2	0	0	2.2e-15	1.1e-14
33	0.00010317	1.3614e-05	71	36	71	2	0	0	2.1e-15	1.1e-14
34	0.00011678	1.3614e-05	73	37	73	2	0	0	1.6e-15	9.6e-15
35	0.00014401	2.7228e-05	75	38	75	2	0	0	1.9e-15	1.4e-14
36	0.00017124	2.7228e-05	77	39	77	2	0	0	2.4e-15	1.3e-14
37	0.00019847	2.7228e-05	79	40	79	2	0	0	1.9e-15	1.2e-14
38	0.00022569	2.7228e-05	81	41	81	2	0	0	2.9e-15	1.1e-14
39	0.00028015	5.4456e-05	83	42	83	2	0	0	1.8e-15	1.6e-14

40	0.0003346	5.4456e-05		85	43	85	2	0	0	1.7e-15	1.4e-14
41	0.00038906	5.4456e-05		87	44	87	2	0	0	4.1e-15	1.3e-14
42	0.00049797	0.00010891		89	45	89	2	0	0	2.2e-15	1.9e-14
43	0.00060688	0.00010891		91	46	91	2	0	0	1.5e-15	1.6e-14
44	0.00071579	0.00010891		93	47	93	2	0	0	1.3e-15	1.6e-14
45	0.0008247	0.00010891		95	48	95	2	0	0	2e-15	1.5e-14
46	0.0010425	0.00021782		97	49	97	2	0	0	2.9e-15	2e-14
47	0.0012603	0.00021782		99	50	99	2	0	0	1.5e-15	1.7e-14
48	0.001696	0.00043564		101	51	101	2	0	0	3.1e-15	2.6e-14
49	0.0021316	0.00043564		103	52	103	2	0	0	3.7e-15	2.3e-14
50	0.0025673	0.00043564		105	53	105	2	0	0	2e-15	2.3e-14
51	0.0030029	0.00043564		107	54	107	2	0	0	3.8e-15	1.9e-14
52	0.0038742	0.00087129		109	55	109	2	0	0	2.9e-15	2.5e-14
53	0.0047455	0.00087129		111	56	111	2	0	0	3.1e-15	1.8e-14
-	0.005	-	out								
54	0.0064881	0.0017426		113	57	113	2	0	0	2.8e-15	3.1e-14
55	0.0082307	0.0017426		115	58	115	2	0	0	6e-15	2.6e-14
-	0.01	-	out								
56	0.011716	0.0034852		117	59	117	2	0	0	1.9e-15	3.9e-14
-	0.015	-	out								
57	0.015201	0.0034852		119	60	119	1	0	0	3e-15	4.3e-14
58	0.018686	0.0034852		121	61	121	1	0	0	4e-15	2.4e-14
-	0.02	-	out								
-	0.025	-	out								
59	0.025656	0.0069703		123	62	123	1	0	0	3.9e-15	4e-14
-	0.03	-	out								
-	0.035	-	out								
60	0.039597	0.013941		125	63	125	1	0	0	4.8e-15	5.9e-14
-	0.04	-	out								
-	0.045	-	out								
-	0.05	-	out								
61	0.053538	0.013941		127	64	127	1	0	0	4.8e-15	6.1e-14
-	0.055	-	out								
-	0.06	-	out								
-	0.065	-	out								
-	0.07	-	out								
-	0.075	-	out								
-	0.08	-	out								
62	0.081419	0.027881		129	65	129	1	0	0	2.8e-15	8.7e-14
-	0.085	-	out								
-	0.09	-	out								
-	0.095	-	out								
-	0.1	-	out								
-	0.105	-	out								
63	0.1093	0.027881		131	66	131	1	0	0	4.1e-15	7.5e-14
-	0.11	-	out								
-	0.115	-	out								
-	0.12	-	out								
-	0.125	-	out								
-	0.13	-	out								
-	0.135	-	out								
-	0.14	-	out								
-	0.145	-	out								
-	0.15	-	out								
-	0.155	-	out								
-	0.16	-	out								

-	0.165	-	out							
64	0.16506	0.055763	133	67	133	1	0	0	4.5e-15	9.5e-14
-	0.17	-	out							
-	0.175	-	out							
-	0.18	-	out							
-	0.185	-	out							
-	0.19	-	out							
-	0.195	-	out							
-	0.2	-	out							
-	0.205	-	out							
-	0.21	-	out							
-	0.215	-	out							
-	0.22	-	out							
65	0.22083	0.055763	135	68	135	1	0	0	3.5e-15	7.4e-14
-	0.225	-	out							
-	0.23	-	out							
-	0.235	-	out							
-	0.24	-	out							
-	0.245	-	out							
-	0.25	-	out							
-	0.25	-	out							
-	0.27	-	out							
-	0.29	-	out							
-	0.31	-	out							
66	0.31983	0.099	137	69	137	1	0	0	4.1e-15	8.2e-14
-	0.33	-	out							
-	0.35	-	out							
-	0.37	-	out							
-	0.39	-	out							
-	0.41	-	out							
67	0.41883	0.099	139	70	139	1	0	0	1.1e-14	6.1e-14
-	0.43	-	out							
-	0.45	-	out							
-	0.47	-	out							
-	0.49	-	out							
-	0.51	-	out							
68	0.51783	0.099	141	71	141	1	0	0	6.2e-15	4.9e-14
-	0.53	-	out							
-	0.55	-	out							
-	0.57	-	out							
-	0.59	-	out							
-	0.61	-	out							
69	0.61683	0.099	143	72	143	1	0	0	8.3e-15	3.9e-14
-	0.63	-	out							
-	0.65	-	out							
-	0.67	-	out							
-	0.69	-	out							
-	0.71	-	out							
70	0.71583	0.099	144	73	144	1	0	0	7e-15	3e-14
-	0.73	-	out							
-	0.75	-	out							
-	0.77	-	out							
-	0.79	-	out							
-	0.81	-	out							
71	0.81483	0.099	145	74	145	1	0	0	3.3e-15	2.7e-14
-	0.83	-	out							

```
   -       0.85           - out
   -       0.87           - out
   -       0.89           - out
   -       0.91           - out
  72    0.91383       0.099      146   75  146     2     0      0  7.1e-15  2.2e-14
   -       0.93           - out
   -       0.95           - out
   -       0.97           - out
   -       0.99           - out
  73     1.0128       0.099      147   76  147     2     0      0  1.1e-14  1.4e-14
Time-stepping completed.
Solution time: 13 s.
Physical memory: 1.18 GB
Virtual memory: 1.17 GB
Ended at May 28, 2023, 4:05:48 PM.
----- Time-Dependent Solver 1 in Oxygen Pulse/Solution 958 (sol958) ----------->
```

k0Sub=0, d=1.5E-5 (su12)

GENERAL

Description	Value
Solution	k0Sub=0, d=1.5E-5

LOG

```
<---- Compile Equations: Stationary in Oxygen Pulse/Solution 958 (sol958) ------
Started at May 28, 2023, 4:05:51 PM.
Parameter k0Sub = 0.0 (m/s).
Parameter d = 1.4999999999999999E-5 (m).
Geometry shape function: Linear Lagrange
Number of vertex elements: 10
Number of boundary elements: 910
Number of elements: 36954
Minimum element quality: 0.4444
Number of vertex elements: 10
Minimum element quality: 0.4444
Time: 2 s.
Physical memory: 1.13 GB
Virtual memory: 1.1 GB
Ended at May 28, 2023, 4:05:53 PM.
----- Compile Equations: Stationary in Oxygen Pulse/Solution 958 (sol958) ----->

<---- Dependent Variables 1 in Oxygen Pulse/Solution 958 (sol958) --------------
Started at May 28, 2023, 4:05:53 PM.
Solution time: 0 s.
Physical memory: 1.13 GB
Virtual memory: 1.1 GB
Ended at May 28, 2023, 4:05:53 PM.
----- Dependent Variables 1 in Oxygen Pulse/Solution 958 (sol958) ------------->

<---- Stationary Solver 1 in Oxygen Pulse/Solution 958 (sol958) ----------------
Started at May 28, 2023, 4:05:54 PM.
Linear solver
Number of degrees of freedom solved for: 18918 (plus 952 internal DOFs).
Symmetric matrices found.
Scales for dependent variables:
Concentration (comp2.Oxi): 0.23
Orthonormal null-space function used.
Iter      SolEst     Damping    Stepsize #Res #Jac #Sol   LinErr   LinRes
   1     2.2e-16   1.0000000     2.2e-16    1    1    1  2.2e-14  2.9e-16
Solution time: 0 s.
Physical memory: 1.13 GB
Virtual memory: 1.11 GB
Ended at May 28, 2023, 4:05:54 PM.
----- Stationary Solver 1 in Oxygen Pulse/Solution 958 (sol958) --------------->

<---- Compile Equations: Oxygen Probe Pulse in Oxygen Pulse/Solution 958
      (sol958) -----------------------------------------------------------------
Started at May 28, 2023, 4:05:54 PM.
Geometry shape function: Linear Lagrange
Time: 1 s.
Physical memory: 1.13 GB
Virtual memory: 1.1 GB
Ended at May 28, 2023, 4:05:55 PM.
----- Compile Equations: Oxygen Probe Pulse in Oxygen Pulse/Solution 958
      (sol958) ---------------------------------------------------------------->

<---- Dependent Variables 2 in Oxygen Pulse/Solution 958 (sol958) --------------
Started at May 28, 2023, 4:05:55 PM.
Initial values of variables solved for: Solution 958 (sol958), Solution Store 1 (sol
985).
```

Values of variables not solved for: Solution 958 (sol958), Solution Store 1 (sol985)
.
Solution time: 0 s.
Physical memory: 1.13 GB
Virtual memory: 1.1 GB
Ended at May 28, 2023, 4:05:55 PM.
----- Dependent Variables 2 in Oxygen Pulse/Solution 958 (sol958) ------------->

<---- Time-Dependent Solver 1 in Oxygen Pulse/Solution 958 (sol958) ------------
Started at May 28, 2023, 4:05:55 PM.
Time-dependent solver (BDF)
Number of degrees of freedom solved for: 18918 (plus 37906 internal DOFs).
Symmetric matrices found.
Scales for dependent variables:
Concentration (comp2.Oxi): 0.23

Step	Time	Stepsize	Res	Jac	Sol	Order	Tfail	NLfail	LinErr	LinRes
0	0	- out	5	3	5			0	9.4e-16	1.4e-15
1	2.6779e-08	2.6779e-08	7	4	7	1	0	0	3.6e-16	1.1e-15
2	5.3558e-08	2.6779e-08	9	5	9	1	0	0	3.3e-16	1.2e-15
3	1.0712e-07	5.3558e-08	11	6	11	2	0	0	3.7e-16	1.3e-15
4	2.1423e-07	1.0712e-07	13	7	13	2	0	0	4.9e-16	1.8e-15
5	4.2847e-07	2.1423e-07	15	8	15	2	0	0	5.9e-16	3.4e-15
6	6.427e-07	2.1423e-07	17	9	17	2	0	0	6e-16	3.6e-15
7	8.5693e-07	2.1423e-07	19	10	19	2	0	0	7.3e-16	3.9e-15
8	1.0712e-06	2.1423e-07	21	11	21	2	0	0	5.8e-16	4e-15
9	1.4996e-06	4.2847e-07	23	12	23	2	0	0	7.9e-16	6.3e-15
10	1.9281e-06	4.2847e-07	25	13	25	2	0	0	1e-15	5.9e-15
11	2.3566e-06	4.2847e-07	27	14	27	2	0	0	8.2e-16	6.3e-15
12	2.785e-06	4.2847e-07	29	15	29	2	0	0	8.1e-16	5.8e-15
13	3.642e-06	8.5693e-07	31	16	31	2	0	0	1.2e-15	8.6e-15
14	4.4989e-06	8.5693e-07	33	17	33	2	0	0	9.8e-16	7.5e-15
15	5.3558e-06	8.5693e-07	35	18	35	2	0	0	7.4e-16	7.5e-15
16	6.2128e-06	8.5693e-07	37	19	37	2	0	0	8.8e-16	6.9e-15
17	7.9266e-06	1.7139e-06	39	20	39	2	0	0	1.4e-15	9.8e-15
18	9.6405e-06	1.7139e-06	41	21	41	2	0	0	1.3e-15	8.2e-15
19	1.1354e-05	1.7139e-06	43	22	43	2	0	0	1.5e-15	8.1e-15
20	1.3068e-05	1.7139e-06	45	23	45	2	0	0	1.5e-15	7.4e-15
21	1.4782e-05	1.7139e-06	47	24	47	2	0	0	1.4e-15	6.8e-15
22	1.821e-05	3.4277e-06	49	25	49	2	0	0	1.6e-15	9.8e-15
23	2.1638e-05	3.4277e-06	51	26	51	2	0	0	1.2e-15	8.5e-15
24	2.5065e-05	3.4277e-06	53	27	53	2	0	0	2.2e-15	8.5e-15
25	2.8493e-05	3.4277e-06	55	28	55	2	0	0	2.1e-15	8e-15
26	3.5349e-05	6.8555e-06	57	29	57	2	0	0	2.8e-15	1.2e-14
27	4.2204e-05	6.8555e-06	59	30	59	2	0	0	2.9e-15	1e-14
28	4.9059e-05	6.8555e-06	61	31	61	2	0	0	1.7e-15	9.8e-15
29	5.5915e-05	6.8555e-06	63	32	63	2	0	0	2.3e-15	9.1e-15
30	6.277e-05	6.8555e-06	65	33	65	2	0	0	1.6e-15	8.2e-15
31	7.6481e-05	1.3711e-05	67	34	67	2	0	0	2.7e-15	1.2e-14
32	9.0192e-05	1.3711e-05	69	35	69	2	0	0	1.8e-15	1.1e-14
33	0.0001039	1.3711e-05	71	36	71	2	0	0	1.4e-15	1e-14
34	0.00011761	1.3711e-05	73	37	73	2	0	0	2.3e-15	9.8e-15
35	0.00014504	2.7422e-05	75	38	75	2	0	0	3e-15	1.4e-14
36	0.00017246	2.7422e-05	77	39	77	2	0	0	1.8e-15	1.2e-14
37	0.00019988	2.7422e-05	79	40	79	2	0	0	2.1e-15	1.3e-14
38	0.0002273	2.7422e-05	81	41	81	2	0	0	2.4e-15	1.1e-14
39	0.00028215	5.4844e-05	83	42	83	2	0	0	2.2e-15	1.7e-14

40	0.00033699	5.4844e-05		85	43	85	2	0	0	2.9e-15	1.4e-14
41	0.00039183	5.4844e-05		87	44	87	2	0	0	2.3e-15	1.4e-14
42	0.00050152	0.00010969		89	45	89	2	0	0	2.3e-15	2e-14
43	0.00061121	0.00010969		91	46	91	2	0	0	2.5e-15	1.7e-14
44	0.0007209	0.00010969		93	47	93	2	0	0	2.4e-15	1.6e-14
45	0.00083058	0.00010969		95	48	95	2	0	0	1.8e-15	1.5e-14
46	0.00105	0.00021938		97	49	97	2	0	0	1.9e-15	2e-14
47	0.0012693	0.00021938		99	50	99	2	0	0	3.1e-15	1.7e-14
48	0.0017081	0.00043875		101	51	101	2	0	0	7.6e-15	2.6e-14
49	0.0021468	0.00043875		103	52	103	2	0	0	5e-15	2.3e-14
50	0.0025856	0.00043875		105	53	105	2	0	0	5.6e-15	2.3e-14
51	0.0030243	0.00043875		107	54	107	2	0	0	1.2e-15	1.9e-14
52	0.0039018	0.0008775		109	55	109	2	0	0	5.3e-15	2.4e-14
53	0.0047793	0.0008775		111	56	111	2	0	0	2.2e-15	1.7e-14
-	0.005	-	out								
54	0.0065343	0.001755		113	57	113	2	0	0	5.3e-15	2.9e-14
55	0.0082893	0.001755		115	58	115	2	0	0	4.4e-15	2.5e-14
-	0.01	-	out								
56	0.011799	0.00351		117	59	117	2	0	0	2.2e-15	3.8e-14
-	0.015	-	out								
57	0.015309	0.00351		119	60	119	1	0	0	6.6e-15	4.1e-14
58	0.018819	0.00351		121	61	121	1	0	0	2.1e-15	2.3e-14
-	0.02	-	out								
-	0.025	-	out								
59	0.025839	0.00702		123	62	123	1	0	0	4.2e-15	3.6e-14
-	0.03	-	out								
-	0.035	-	out								
60	0.039879	0.01404		125	63	125	1	0	0	1.4e-14	5.4e-14
-	0.04	-	out								
-	0.045	-	out								
-	0.05	-	out								
61	0.053919	0.01404		127	64	127	1	0	0	1.1e-14	5e-14
-	0.055	-	out								
-	0.06	-	out								
-	0.065	-	out								
-	0.07	-	out								
-	0.075	-	out								
-	0.08	-	out								
62	0.081999	0.02808		129	65	129	1	0	0	3.5e-15	7.1e-14
-	0.085	-	out								
-	0.09	-	out								
-	0.095	-	out								
-	0.1	-	out								
-	0.105	-	out								
-	0.11	-	out								
63	0.11008	0.02808		131	66	131	1	0	0	4.2e-15	6.7e-14
-	0.115	-	out								
-	0.12	-	out								
-	0.125	-	out								
-	0.13	-	out								
-	0.135	-	out								
-	0.14	-	out								
-	0.145	-	out								
-	0.15	-	out								
-	0.155	-	out								
-	0.16	-	out								

-	0.165	-	out							
64	0.16624	0.05616	133	67	133	1	0	0	3.9e-15	8.5e-14
-	0.17	-	out							
-	0.175	-	out							
-	0.18	-	out							
-	0.185	-	out							
-	0.19	-	out							
-	0.195	-	out							
-	0.2	-	out							
-	0.205	-	out							
-	0.21	-	out							
-	0.215	-	out							
-	0.22	-	out							
65	0.2224	0.05616	135	68	135	1	0	0	1e-14	7.3e-14
-	0.225	-	out							
-	0.23	-	out							
-	0.235	-	out							
-	0.24	-	out							
-	0.245	-	out							
-	0.25	-	out							
-	0.25	-	out							
-	0.27	-	out							
-	0.29	-	out							
-	0.31	-	out							
66	0.3214	0.099	137	69	137	1	0	0	3.7e-15	7.7e-14
-	0.33	-	out							
-	0.35	-	out							
-	0.37	-	out							
-	0.39	-	out							
-	0.41	-	out							
67	0.4204	0.099	139	70	139	1	0	0	5.2e-15	6.1e-14
-	0.43	-	out							
-	0.45	-	out							
-	0.47	-	out							
-	0.49	-	out							
-	0.51	-	out							
68	0.5194	0.099	141	71	141	1	0	0	3.8e-15	5e-14
-	0.53	-	out							
-	0.55	-	out							
-	0.57	-	out							
-	0.59	-	out							
-	0.61	-	out							
69	0.6184	0.099	143	72	143	1	0	0	5.3e-15	3.8e-14
-	0.63	-	out							
-	0.65	-	out							
-	0.67	-	out							
-	0.69	-	out							
-	0.71	-	out							
70	0.7174	0.099	144	73	144	1	0	0	2.7e-15	3.1e-14
-	0.73	-	out							
-	0.75	-	out							
-	0.77	-	out							
-	0.79	-	out							
-	0.81	-	out							
71	0.8164	0.099	145	74	145	1	0	0	2.3e-15	2.8e-14
-	0.83	-	out							

```
   -        0.85           - out
   -        0.87           - out
   -        0.89           - out
   -        0.91           - out
  72      0.9154        0.099      146    75  146      2      0       0  8.7e-15  2.3e-14
   -        0.93           - out
   -        0.95           - out
   -        0.97           - out
   -        0.99           - out
  73      1.0144        0.099      147    76  147      2      0       0  7.3e-15  1.4e-14
Time-stepping completed.
Solution time: 13 s.
Physical memory: 1.17 GB
Virtual memory: 1.15 GB
Ended at May 28, 2023, 4:06:08 PM.
----- Time-Dependent Solver 1 in Oxygen Pulse/Solution 958 (sol958) ----------->
```

k0Sub=1.51E-9, d=5E-7 (su13)

GENERAL

Description	Value
Solution	k0Sub=1.51E-9, d=5E-7

LOG

```
<---- Compile Equations: Stationary in Oxygen Pulse/Solution 958 (sol958) ------
Started at May 28, 2023, 4:06:10 PM.
Parameter k0Sub = 1.51E-9 (m/s).
Parameter d = 5.0E-7 (m).
Geometry shape function: Linear Lagrange
Number of vertex elements: 10
Number of boundary elements: 920
Number of elements: 34573
Minimum element quality: 0.3539
Number of vertex elements: 10
Minimum element quality: 0.3539
Time: 2 s.
Physical memory: 1.19 GB
Virtual memory: 1.17 GB
Ended at May 28, 2023, 4:06:12 PM.
----- Compile Equations: Stationary in Oxygen Pulse/Solution 958 (sol958) ----->

<---- Dependent Variables 1 in Oxygen Pulse/Solution 958 (sol958) --------------
Started at May 28, 2023, 4:06:12 PM.
Solution time: 0 s.
Physical memory: 1.14 GB
Virtual memory: 1.12 GB
Ended at May 28, 2023, 4:06:12 PM.
----- Dependent Variables 1 in Oxygen Pulse/Solution 958 (sol958) ------------->

<---- Stationary Solver 1 in Oxygen Pulse/Solution 958 (sol958) ----------------
Started at May 28, 2023, 4:06:12 PM.
Linear solver
Number of degrees of freedom solved for: 17710 (plus 1007 internal DOFs).
Symmetric matrices found.
Scales for dependent variables:
Concentration (comp2.Oxi): 0.23
Orthonormal null-space function used.
Iter      SolEst     Damping    Stepsize #Res #Jac #Sol   LinErr   LinRes
   1     2.1e-06   1.0000000     2.1e-06    1    1    1  5.7e-13  1.5e-13
Solution time: 1 s.
Physical memory: 1.16 GB
Virtual memory: 1.14 GB
Ended at May 28, 2023, 4:06:13 PM.
----- Stationary Solver 1 in Oxygen Pulse/Solution 958 (sol958) --------------->

<---- Compile Equations: Oxygen Probe Pulse in Oxygen Pulse/Solution 958
      (sol958) -----------------------------------------------------------------
Started at May 28, 2023, 4:06:13 PM.
Geometry shape function: Linear Lagrange
Time: 0 s.
Physical memory: 1.16 GB
Virtual memory: 1.14 GB
Ended at May 28, 2023, 4:06:14 PM.
----- Compile Equations: Oxygen Probe Pulse in Oxygen Pulse/Solution 958
      (sol958) ---------------------------------------------------------------->

<---- Dependent Variables 2 in Oxygen Pulse/Solution 958 (sol958) --------------
Started at May 28, 2023, 4:06:14 PM.
Initial values of variables solved for: Solution 958 (sol958), Solution Store 1 (sol
985).
```

Values of variables not solved for: Solution 958 (sol958), Solution Store 1 (sol985)
.
Solution time: 0 s.
Physical memory: 1.12 GB
Virtual memory: 1.11 GB
Ended at May 28, 2023, 4:06:14 PM.
----- Dependent Variables 2 in Oxygen Pulse/Solution 958 (sol958) ------------->

<---- Time-Dependent Solver 1 in Oxygen Pulse/Solution 958 (sol958) ------------
Started at May 28, 2023, 4:06:14 PM.
Time-dependent solver (BDF)
Number of degrees of freedom solved for: 17710 (plus 35580 internal DOFs).
Symmetric matrices found.
Scales for dependent variables:
Concentration (comp2.Oxi): 0.23

Step	Time	Stepsize	Res	Jac	Sol	Order	Tfail	NLfail	LinErr	LinRes
0	0	- out	5	3	5			0	7.3e-16	1.5e-15
1	2.5603e-08	2.5603e-08	7	4	7	1	0	0	3.4e-16	1.1e-15
2	5.1207e-08	2.5603e-08	9	5	9	1	0	0	3.3e-16	1.1e-15
3	1.0241e-07	5.1207e-08	11	6	11	2	0	0	3.7e-16	1.3e-15
4	2.0483e-07	1.0241e-07	13	7	13	2	0	0	5.1e-16	1.8e-15
5	4.0965e-07	2.0483e-07	15	8	15	2	0	0	7.2e-16	3.5e-15
6	6.1448e-07	2.0483e-07	17	9	17	2	0	0	6.4e-16	3.8e-15
7	8.1931e-07	2.0483e-07	19	10	19	2	0	0	6.1e-16	3.9e-15
8	1.0241e-06	2.0483e-07	21	11	21	2	0	0	6e-16	3.9e-15
9	1.4338e-06	4.0965e-07	23	12	23	2	0	0	8e-16	5.8e-15
10	1.8434e-06	4.0965e-07	25	13	25	2	0	0	7.6e-16	5.6e-15
11	2.2531e-06	4.0965e-07	27	14	27	2	0	0	8.8e-16	5.5e-15
12	2.6627e-06	4.0965e-07	29	15	29	2	0	0	8e-16	5.1e-15
13	3.4821e-06	8.1931e-07	31	16	31	2	0	0	1.4e-15	7.8e-15
14	4.3014e-06	8.1931e-07	33	17	33	2	0	0	1.2e-15	6.6e-15
15	5.1207e-06	8.1931e-07	35	18	35	2	0	0	1.3e-15	6.4e-15
16	5.94e-06	8.1931e-07	37	19	37	2	0	0	1.1e-15	5.9e-15
17	7.5786e-06	1.6386e-06	39	20	39	2	0	0	1.4e-15	8.4e-15
18	9.2172e-06	1.6386e-06	41	21	41	2	0	0	2.1e-15	7.8e-15
19	1.0856e-05	1.6386e-06	43	22	43	2	0	0	1.3e-15	7.8e-15
20	1.2494e-05	1.6386e-06	45	23	45	2	0	0	1.3e-15	7.2e-15
21	1.4133e-05	1.6386e-06	47	24	47	2	0	0	9.2e-16	6.4e-15
22	1.741e-05	3.2772e-06	49	25	49	2	0	0	1.7e-15	9.7e-15
23	2.0687e-05	3.2772e-06	51	26	51	2	0	0	2.5e-15	8.3e-15
24	2.3965e-05	3.2772e-06	53	27	53	2	0	0	1.3e-15	8.7e-15
25	2.7242e-05	3.2772e-06	55	28	55	2	0	0	1.1e-15	7.8e-15
26	3.3796e-05	6.5545e-06	57	29	57	2	0	0	1.6e-15	1.2e-14
27	4.0351e-05	6.5545e-06	59	30	59	2	0	0	1.7e-15	1e-14
28	4.6905e-05	6.5545e-06	61	31	61	2	0	0	1.8e-15	1e-14
29	5.346e-05	6.5545e-06	63	32	63	2	0	0	1.7e-15	9.2e-15
30	6.0014e-05	6.5545e-06	65	33	65	2	0	0	1.6e-15	7.9e-15
31	7.3123e-05	1.3109e-05	67	34	67	2	0	0	1.9e-15	1.2e-14
32	8.6232e-05	1.3109e-05	69	35	69	2	0	0	1.6e-15	1.1e-14
33	9.9341e-05	1.3109e-05	71	36	71	2	0	0	1.6e-15	1.1e-14
34	0.00011245	1.3109e-05	73	37	73	2	0	0	1.2e-15	1e-14
35	0.00013867	2.6218e-05	75	38	75	2	0	0	1.5e-15	1.6e-14
36	0.00016489	2.6218e-05	77	39	77	2	0	0	1.5e-15	1.3e-14
37	0.0001911	2.6218e-05	79	40	79	2	0	0	1.7e-15	1.2e-14
38	0.00021732	2.6218e-05	81	41	81	2	0	0	2.1e-15	1.1e-14
39	0.00026976	5.2436e-05	83	42	83	2	0	0	2.5e-15	1.6e-14

40	0.00032219	5.2436e-05	85	43	85	2	0	0	2.3e-15	1.4e-14
41	0.00037463	5.2436e-05	87	44	87	2	0	0	3e-15	1.4e-14
42	0.0004795	0.00010487	89	45	89	2	0	0	4.7e-15	2.1e-14
43	0.00058437	0.00010487	91	46	91	2	0	0	5.1e-15	1.6e-14
44	0.00068924	0.00010487	93	47	93	2	0	0	4e-15	1.6e-14
45	0.00089898	0.00020974	95	48	95	2	0	0	2.7e-15	2.4e-14
46	0.0011087	0.00020974	97	49	97	2	0	0	2.2e-15	2e-14
47	0.0013185	0.00020974	99	50	99	2	0	0	3.3e-15	2e-14
48	0.0015282	0.00020974	101	51	101	2	0	0	5.8e-15	2.1e-14
49	0.0019477	0.00041949	103	52	103	2	0	0	2.2e-15	3e-14
50	0.0023672	0.00041949	105	53	105	2	0	0	3.1e-15	2.7e-14
51	0.0027867	0.00041949	107	54	107	2	0	0	2.1e-15	2.7e-14
52	0.0036256	0.00083897	109	55	109	2	0	0	3.6e-15	4e-14
53	0.0044646	0.00083897	111	56	111	2	0	0	5e-15	3.3e-14
-	0.005	- out								
54	0.0053036	0.00083897	113	57	113	2	0	0	1.9e-15	3.5e-14
55	0.0069815	0.0016779	115	58	115	2	0	0	5e-15	5e-14
56	0.0086595	0.0016779	117	59	117	2	0	0	2.9e-15	4.4e-14
-	0.01	- out								
57	0.012015	0.0033559	119	60	119	2	0	0	3.3e-15	6.4e-14
-	0.015	- out								
58	0.015371	0.0033559	121	61	121	2	0	0	3.9e-15	5.1e-14
59	0.018727	0.0033559	123	62	123	2	0	0	2.7e-15	5e-14
-	0.02	- out								
60	0.022083	0.0033559	125	63	125	2	0	0	2.2e-15	4.5e-14
-	0.025	- out								
61	0.028795	0.0067118	127	64	127	2	0	0	1.9e-15	5.9e-14
-	0.03	- out								
-	0.035	- out								
62	0.035507	0.0067118	129	65	129	2	0	0	9.7e-15	5.1e-14
-	0.04	- out								
-	0.045	- out								
63	0.04893	0.013424	131	66	131	2	0	0	9e-15	8.3e-14
-	0.05	- out								
-	0.055	- out								
-	0.06	- out								
64	0.062354	0.013424	133	67	133	1	0	0	1e-14	7.2e-14
-	0.065	- out								
-	0.07	- out								
-	0.075	- out								
65	0.075777	0.013424	135	68	135	1	0	0	4e-15	2.7e-14
-	0.08	- out								
-	0.085	- out								
-	0.09	- out								
-	0.095	- out								
-	0.1	- out								
66	0.10262	0.026847	137	69	137	1	0	0	4.8e-15	3.9e-14
-	0.105	- out								
-	0.11	- out								
-	0.115	- out								
-	0.12	- out								
-	0.125	- out								
-	0.13	- out								
-	0.135	- out								
-	0.14	- out								
-	0.145	- out								

-	0.15	-	out							
-	0.155	-	out							
67	0.15632	0.053694	139	70	139	1	0	0	2.6e-15	5e-14
-	0.16	-	out							
-	0.165	-	out							
-	0.17	-	out							
-	0.175	-	out							
-	0.18	-	out							
-	0.185	-	out							
-	0.19	-	out							
-	0.195	-	out							
-	0.2	-	out							
-	0.205	-	out							
-	0.21	-	out							
-	0.215	-	out							
-	0.22	-	out							
-	0.225	-	out							
-	0.23	-	out							
-	0.235	-	out							
-	0.24	-	out							
-	0.245	-	out							
-	0.25	-	out							
-	0.25	-	out							
68	0.25532	0.099	141	71	141	1	0	0	1.1e-14	5.6e-14
-	0.27	-	out							
-	0.29	-	out							
-	0.31	-	out							
-	0.33	-	out							
-	0.35	-	out							
69	0.35432	0.099	143	72	143	1	0	0	4.3e-15	4.5e-14
-	0.37	-	out							
-	0.39	-	out							
-	0.41	-	out							
-	0.43	-	out							
-	0.45	-	out							
70	0.45332	0.099	145	73	145	1	0	0	1e-14	3.5e-14
-	0.47	-	out							
-	0.49	-	out							
-	0.51	-	out							
-	0.53	-	out							
-	0.55	-	out							
71	0.55232	0.099	147	74	147	1	0	0	2.3e-14	2.7e-14
-	0.57	-	out							
-	0.59	-	out							
-	0.61	-	out							
-	0.63	-	out							
-	0.65	-	out							
72	0.65132	0.099	148	75	148	1	0	0	1.6e-14	2.2e-14
-	0.67	-	out							
-	0.69	-	out							
-	0.71	-	out							
-	0.73	-	out							
-	0.75	-	out							
73	0.75032	0.099	149	76	149	1	0	0	6.5e-15	1.9e-14
-	0.77	-	out							
-	0.79	-	out							

```
   -        0.81            - out
   -        0.83            - out
  74     0.84932        0.099      150    77   150      2      0       0  4.6e-15  1.6e-14
   -        0.85            - out
   -        0.87            - out
   -        0.89            - out
   -        0.91            - out
   -        0.93            - out
  75     0.94832        0.099      151    78   151      2      0       0  5.5e-15  1.1e-14
   -        0.95            - out
   -        0.97            - out
   -        0.99            - out
  76      1.0473        0.099      152    79   152      2      0       0  3.2e-15    1e-14
Time-stepping completed.
Solution time: 13 s.
Physical memory: 1.17 GB
Virtual memory: 1.15 GB
Ended at May 28, 2023, 4:06:26 PM.
----- Time-Dependent Solver 1 in Oxygen Pulse/Solution 958 (sol958) ----------->
```

k0Sub=1.51E-9, d=1E-6 (su14)

GENERAL

Description	Value
Solution	k0Sub=1.51E-9, d=1E-6

LOG

```
<---- Compile Equations: Stationary in Oxygen Pulse/Solution 958 (sol958) ------
Started at May 28, 2023, 4:06:29 PM.
Parameter k0Sub = 1.51E-9 (m/s).
Parameter d = 1.0E-6 (m).
Geometry shape function: Linear Lagrange
Number of vertex elements: 10
Minimum element quality: 0.4361
Time: 1 s.
Physical memory: 1.19 GB
Virtual memory: 1.17 GB
Ended at May 28, 2023, 4:06:29 PM.
----- Compile Equations: Stationary in Oxygen Pulse/Solution 958 (sol958) ----->

<---- Dependent Variables 1 in Oxygen Pulse/Solution 958 (sol958) --------------
Started at May 28, 2023, 4:06:29 PM.
Solution time: 0 s.
Physical memory: 1.19 GB
Virtual memory: 1.17 GB
Ended at May 28, 2023, 4:06:29 PM.
----- Dependent Variables 1 in Oxygen Pulse/Solution 958 (sol958) ------------->

<---- Stationary Solver 1 in Oxygen Pulse/Solution 958 (sol958) ----------------
Started at May 28, 2023, 4:06:29 PM.
Linear solver
Number of degrees of freedom solved for: 17724 (plus 963 internal DOFs).
Symmetric matrices found.
Scales for dependent variables:
Concentration (comp2.Oxi): 0.23
Orthonormal null-space function used.
Iter      SolEst     Damping    Stepsize #Res #Jac #Sol   LinErr   LinRes
   1     1.8e-06   1.0000000     1.8e-06    1    1    1  4.4e-13  1.3e-13
Solution time: 0 s.
Physical memory: 1.19 GB
Virtual memory: 1.17 GB
Ended at May 28, 2023, 4:06:30 PM.
----- Stationary Solver 1 in Oxygen Pulse/Solution 958 (sol958) --------------->

<---- Compile Equations: Oxygen Probe Pulse in Oxygen Pulse/Solution 958
      (sol958) ----------------------------------------------------------------
Started at May 28, 2023, 4:06:30 PM.
Geometry shape function: Linear Lagrange
Time: 1 s.
Physical memory: 1.2 GB
Virtual memory: 1.17 GB
Ended at May 28, 2023, 4:06:30 PM.
----- Compile Equations: Oxygen Probe Pulse in Oxygen Pulse/Solution 958
      (sol958) --------------------------------------------------------------->

<---- Dependent Variables 2 in Oxygen Pulse/Solution 958 (sol958) --------------
Started at May 28, 2023, 4:06:30 PM.
Initial values of variables solved for: Solution 958 (sol958), Solution Store 1 (sol
985).
Values of variables not solved for: Solution 958 (sol958), Solution Store 1 (sol985)
.
Solution time: 0 s.
Physical memory: 1.2 GB
```

Virtual memory: 1.17 GB
Ended at May 28, 2023, 4:06:30 PM.
----- Dependent Variables 2 in Oxygen Pulse/Solution 958 (sol958) ------------->

<---- Time-Dependent Solver 1 in Oxygen Pulse/Solution 958 (sol958) ------------
Started at May 28, 2023, 4:06:30 PM.
Time-dependent solver (BDF)
Number of degrees of freedom solved for: 17724 (plus 35562 internal DOFs).
Symmetric matrices found.
Scales for dependent variables:
Concentration (comp2.Oxi): 0.23

Step	Time	Stepsize		Res	Jac	Sol	Order	Tfail	NLfail	LinErr	LinRes
0	0	-	out	5	3	5			0	8.3e-16	1.4e-15
1	2.5901e-08	2.5901e-08		7	4	7	1	0	0	3.4e-16	1e-15
2	5.1803e-08	2.5901e-08		9	5	9	1	0	0	3.5e-16	1.2e-15
3	1.0361e-07	5.1803e-08		11	6	11	2	0	0	4e-16	1.3e-15
4	2.0721e-07	1.0361e-07		13	7	13	2	0	0	5.4e-16	1.7e-15
5	4.1442e-07	2.0721e-07		15	8	15	2	0	0	6.4e-16	3.4e-15
6	6.2163e-07	2.0721e-07		17	9	17	2	0	0	6e-16	3.8e-15
7	8.2884e-07	2.0721e-07		19	10	19	2	0	0	6.9e-16	3.9e-15
8	1.0361e-06	2.0721e-07		21	11	21	2	0	0	6.3e-16	3.8e-15
9	1.4505e-06	4.1442e-07		23	12	23	2	0	0	9.6e-16	6.3e-15
10	1.8649e-06	4.1442e-07		25	13	25	2	0	0	8.3e-16	5.8e-15
11	2.2793e-06	4.1442e-07		27	14	27	2	0	0	8.5e-16	5.9e-15
12	2.6937e-06	4.1442e-07		29	15	29	2	0	0	8.3e-16	5.4e-15
13	3.5226e-06	8.2884e-07		31	16	31	2	0	0	9e-16	8.1e-15
14	4.3514e-06	8.2884e-07		33	17	33	2	0	0	1.1e-15	6.7e-15
15	5.1803e-06	8.2884e-07		35	18	35	2	0	0	1.3e-15	6.9e-15
16	6.0091e-06	8.2884e-07		37	19	37	2	0	0	1e-15	6.2e-15
17	7.6668e-06	1.6577e-06		39	20	39	2	0	0	1.3e-15	9.2e-15
18	9.3245e-06	1.6577e-06		41	21	41	2	0	0	1.2e-15	7.9e-15
19	1.0982e-05	1.6577e-06		43	22	43	2	0	0	1e-15	7.7e-15
20	1.264e-05	1.6577e-06		45	23	45	2	0	0	1.2e-15	7e-15
21	1.4298e-05	1.6577e-06		47	24	47	2	0	0	1e-15	6.2e-15
22	1.7613e-05	3.3154e-06		49	25	49	2	0	0	1.9e-15	9e-15
23	2.0928e-05	3.3154e-06		51	26	51	2	0	0	2.4e-15	7.7e-15
24	2.4244e-05	3.3154e-06		53	27	53	2	0	0	1.5e-15	7.9e-15
25	2.7559e-05	3.3154e-06		55	28	55	2	0	0	1e-15	7.2e-15
26	3.419e-05	6.6307e-06		57	29	57	2	0	0	2e-15	1.1e-14
27	4.082e-05	6.6307e-06		59	30	59	2	0	0	2.3e-15	9e-15
28	4.7451e-05	6.6307e-06		61	31	61	2	0	0	1.6e-15	9.2e-15
29	5.4082e-05	6.6307e-06		63	32	63	2	0	0	1.7e-15	8.4e-15
30	6.0713e-05	6.6307e-06		65	33	65	2	0	0	2e-15	7.6e-15
31	7.3974e-05	1.3261e-05		67	34	67	2	0	0	2.1e-15	1.2e-14
32	8.7236e-05	1.3261e-05		69	35	69	2	0	0	1.5e-15	1e-14
33	0.0001005	1.3261e-05		71	36	71	2	0	0	1.3e-15	9.7e-15
34	0.00011376	1.3261e-05		73	37	73	2	0	0	1.8e-15	9.3e-15
35	0.00014028	2.6523e-05		75	38	75	2	0	0	1.7e-15	1.4e-14
36	0.0001668	2.6523e-05		77	39	77	2	0	0	2.6e-15	1.2e-14
37	0.00019333	2.6523e-05		79	40	79	2	0	0	1.5e-15	1.2e-14
38	0.00021985	2.6523e-05		81	41	81	2	0	0	2.4e-15	1.1e-14
39	0.0002729	5.3046e-05		83	42	83	2	0	0	3.6e-15	1.6e-14
40	0.00032594	5.3046e-05		85	43	85	2	0	0	1.4e-15	1.3e-14
41	0.00037899	5.3046e-05		87	44	87	2	0	0	2.7e-15	1.3e-14
42	0.00048508	0.00010609		89	45	89	2	0	0	2e-15	1.9e-14
43	0.00059117	0.00010609		91	46	91	2	0	0	2.3e-15	1.6e-14

44	0.00069726	0.00010609		93	47	93	2	0	0	2.8e-15	1.6e-14
45	0.00080335	0.00010609		95	48	95	2	0	0	2.9e-15	1.4e-14
46	0.0010155	0.00021218		97	49	97	2	0	0	3.2e-15	2e-14
47	0.0012277	0.00021218		99	50	99	2	0	0	2.5e-15	1.6e-14
48	0.0016521	0.00042437		101	51	101	2	0	0	3.9e-15	2.7e-14
49	0.0020765	0.00042437		103	52	103	2	0	0	5.7e-15	2.5e-14
50	0.0025008	0.00042437		105	53	105	2	0	0	5.4e-15	2.5e-14
51	0.0029252	0.00042437		107	54	107	2	0	0	3.6e-15	2.3e-14
52	0.0037739	0.00084873		109	55	109	2	0	0	7.9e-15	3.2e-14
53	0.0046227	0.00084873		111	56	111	2	0	0	1.3e-15	2.8e-14
-	0.005	-	out								
54	0.0063201	0.0016975		113	57	113	2	0	0	2.2e-15	4.6e-14
55	0.0080176	0.0016975		115	58	115	2	0	0	2.6e-15	4.3e-14
56	0.0097151	0.0016975		117	59	117	2	0	0	4e-15	4.2e-14
-	0.01	-	out								
57	0.011413	0.0016975		119	60	119	2	0	0	2e-15	3.9e-14
58	0.014807	0.0033949		121	61	121	2	0	0	2.5e-15	4.8e-14
-	0.015	-	out								
59	0.018202	0.0033949		123	62	123	2	0	0	4.8e-15	3.6e-14
-	0.02	-	out								
60	0.024992	0.0067899		125	63	125	2	0	0	3.7e-15	6.2e-14
-	0.025	-	out								
-	0.03	-	out								
61	0.031782	0.0067899		127	64	127	2	0	0	7.7e-15	5.7e-14
-	0.035	-	out								
62	0.038572	0.0067899		129	65	129	2	0	0	1.7e-15	5.9e-14
-	0.04	-	out								
-	0.045	-	out								
63	0.045362	0.0067899		131	66	131	2	0	0	2.5e-15	5.4e-14
-	0.05	-	out								
-	0.055	-	out								
64	0.058942	0.01358		133	67	133	2	0	0	4.2e-15	6.5e-14
-	0.06	-	out								
-	0.065	-	out								
-	0.07	-	out								
65	0.072521	0.01358		135	68	135	2	0	0	6.6e-15	4.4e-14
-	0.075	-	out								
-	0.08	-	out								
-	0.085	-	out								
-	0.09	-	out								
-	0.095	-	out								
66	0.099681	0.027159		137	69	137	2	0	0	1.6e-14	6.2e-14
-	0.1	-	out								
-	0.105	-	out								
-	0.11	-	out								
-	0.115	-	out								
-	0.12	-	out								
-	0.125	-	out								
-	0.13	-	out								
-	0.135	-	out								
-	0.14	-	out								
-	0.145	-	out								
-	0.15	-	out								
67	0.154	0.054319		139	70	139	1	0	0	6.2e-15	5.9e-14
-	0.155	-	out								
-	0.16	-	out								

-	0.165	-	out							
-	0.17	-	out							
-	0.175	-	out							
-	0.18	-	out							
-	0.185	-	out							
-	0.19	-	out							
-	0.195	-	out							
-	0.2	-	out							
-	0.205	-	out							
68	0.20832	0.054319	141	71	141	1	0	0	4.2e-15	3e-14
-	0.21	-	out							
-	0.215	-	out							
-	0.22	-	out							
-	0.225	-	out							
-	0.23	-	out							
-	0.235	-	out							
-	0.24	-	out							
-	0.245	-	out							
-	0.25	-	out							
-	0.25	-	out							
-	0.27	-	out							
-	0.29	-	out							
69	0.30732	0.099	143	72	143	1	0	0	3.6e-15	3.9e-14
-	0.31	-	out							
-	0.33	-	out							
-	0.35	-	out							
-	0.37	-	out							
-	0.39	-	out							
70	0.40632	0.099	145	73	145	1	0	0	8.8e-15	3.4e-14
-	0.41	-	out							
-	0.43	-	out							
-	0.45	-	out							
-	0.47	-	out							
-	0.49	-	out							
71	0.50532	0.099	147	74	147	1	0	0	1.4e-14	3e-14
-	0.51	-	out							
-	0.53	-	out							
-	0.55	-	out							
-	0.57	-	out							
-	0.59	-	out							
72	0.60432	0.099	149	75	149	1	0	0	7.4e-15	2.4e-14
-	0.61	-	out							
-	0.63	-	out							
-	0.65	-	out							
-	0.67	-	out							
-	0.69	-	out							
73	0.70332	0.099	150	76	150	1	0	0	1.8e-14	2e-14
-	0.71	-	out							
-	0.73	-	out							
-	0.75	-	out							
-	0.77	-	out							
-	0.79	-	out							
74	0.80232	0.099	151	77	151	1	0	0	6.7e-15	1.8e-14
-	0.81	-	out							
-	0.83	-	out							
-	0.85	-	out							

```
   -        0.87            - out
   -        0.89            - out
  75     0.90132        0.099      152   78  152      2      0       0  1.4e-14  1.4e-14
   -        0.91            - out
   -        0.93            - out
   -        0.95            - out
   -        0.97            - out
   -        0.99            - out
  76      1.0003        0.099      153   79  153      2      0       0  1.3e-14  9.3e-15
Time-stepping completed.
Solution time: 13 s.
Physical memory: 1.21 GB
Virtual memory: 1.19 GB
Ended at May 28, 2023, 4:06:43 PM.
----- Time-Dependent Solver 1 in Oxygen Pulse/Solution 958 (sol958) ----------->
```

k0Sub=1.51E-9, d=1.5E-6 (su15)

GENERAL

Description	Value
Solution	k0Sub=1.51E-9, d=1.5E-6

LOG

```
<---- Compile Equations: Stationary in Oxygen Pulse/Solution 958 (sol958) ------
Started at May 28, 2023, 4:06:46 PM.
Parameter k0Sub = 1.51E-9 (m/s).
Parameter d = 1.5E-6 (m).
Geometry shape function: Linear Lagrange
Number of vertex elements: 10
Minimum element quality: 0.395
Time: 1 s.
Physical memory: 1.18 GB
Virtual memory: 1.16 GB
Ended at May 28, 2023, 4:06:47 PM.
----- Compile Equations: Stationary in Oxygen Pulse/Solution 958 (sol958) ----->

<---- Dependent Variables 1 in Oxygen Pulse/Solution 958 (sol958) --------------
Started at May 28, 2023, 4:06:47 PM.
Solution time: 0 s.
Physical memory: 1.18 GB
Virtual memory: 1.16 GB
Ended at May 28, 2023, 4:06:47 PM.
----- Dependent Variables 1 in Oxygen Pulse/Solution 958 (sol958) ------------->

<---- Stationary Solver 1 in Oxygen Pulse/Solution 958 (sol958) ----------------
Started at May 28, 2023, 4:06:47 PM.
Linear solver
Number of degrees of freedom solved for: 17823 (plus 950 internal DOFs).
Symmetric matrices found.
Scales for dependent variables:
Concentration (comp2.Oxi): 0.23
Orthonormal null-space function used.
Iter      SolEst     Damping    Stepsize #Res #Jac #Sol   LinErr   LinRes
   1     1.5e-06   1.0000000     1.5e-06    1    1    1  4.1e-13  1.2e-13
Solution time: 1 s.
Physical memory: 1.19 GB
Virtual memory: 1.16 GB
Ended at May 28, 2023, 4:06:47 PM.
----- Stationary Solver 1 in Oxygen Pulse/Solution 958 (sol958) --------------->

<---- Compile Equations: Oxygen Probe Pulse in Oxygen Pulse/Solution 958
      (sol958) -----------------------------------------------------------------
Started at May 28, 2023, 4:06:47 PM.
Geometry shape function: Linear Lagrange
Time: 1 s.
Physical memory: 1.19 GB
Virtual memory: 1.16 GB
Ended at May 28, 2023, 4:06:48 PM.
----- Compile Equations: Oxygen Probe Pulse in Oxygen Pulse/Solution 958
      (sol958) ---------------------------------------------------------------->

<---- Dependent Variables 2 in Oxygen Pulse/Solution 958 (sol958) --------------
Started at May 28, 2023, 4:06:48 PM.
Initial values of variables solved for: Solution 958 (sol958), Solution Store 1 (sol
985).
Values of variables not solved for: Solution 958 (sol958), Solution Store 1 (sol985)
.
Solution time: 0 s.
Physical memory: 1.19 GB
```

Virtual memory: 1.16 GB
Ended at May 28, 2023, 4:06:48 PM.
----- Dependent Variables 2 in Oxygen Pulse/Solution 958 (sol958) ------------->

<---- Time-Dependent Solver 1 in Oxygen Pulse/Solution 958 (sol958) ------------
Started at May 28, 2023, 4:06:48 PM.
Time-dependent solver (BDF)
Number of degrees of freedom solved for: 17823 (plus 35744 internal DOFs).
Symmetric matrices found.
Scales for dependent variables:
Concentration (comp2.Oxi): 0.23

Step	Time	Stepsize	Res	Jac	Sol	Order	Tfail	NLfail	LinErr	LinRes
0	0	- out	5	3	5			0	8.6e-16	1.4e-15
1	2.5748e-08	2.5748e-08	7	4	7	1	0	0	3.6e-16	1e-15
2	5.1496e-08	2.5748e-08	9	5	9	1	0	0	3.9e-16	1.2e-15
3	1.0299e-07	5.1496e-08	11	6	11	2	0	0	3.6e-16	1.3e-15
4	2.0598e-07	1.0299e-07	13	7	13	2	0	0	4.5e-16	1.8e-15
5	4.1197e-07	2.0598e-07	15	8	15	2	0	0	7.9e-16	3.4e-15
6	6.1795e-07	2.0598e-07	17	9	17	2	0	0	6.4e-16	3.9e-15
7	8.2394e-07	2.0598e-07	19	10	19	2	0	0	5.8e-16	4e-15
8	1.0299e-06	2.0598e-07	21	11	21	2	0	0	6e-16	3.9e-15
9	1.4419e-06	4.1197e-07	23	12	23	2	0	0	6.7e-16	6.3e-15
10	1.8539e-06	4.1197e-07	25	13	25	2	0	0	7.7e-16	6e-15
11	2.2658e-06	4.1197e-07	27	14	27	2	0	0	8.1e-16	5.9e-15
12	2.6778e-06	4.1197e-07	29	15	29	2	0	0	9.9e-16	5.6e-15
13	3.5017e-06	8.2394e-07	31	16	31	2	0	0	1.5e-15	8.3e-15
14	4.3257e-06	8.2394e-07	33	17	33	2	0	0	1.1e-15	7.2e-15
15	5.1496e-06	8.2394e-07	35	18	35	2	0	0	1e-15	6.8e-15
16	5.9736e-06	8.2394e-07	37	19	37	2	0	0	1.1e-15	6.3e-15
17	7.6214e-06	1.6479e-06	39	20	39	2	0	0	1.4e-15	9.2e-15
18	9.2693e-06	1.6479e-06	41	21	41	2	0	0	1.3e-15	8.2e-15
19	1.0917e-05	1.6479e-06	43	22	43	2	0	0	1.4e-15	8.1e-15
20	1.2565e-05	1.6479e-06	45	23	45	2	0	0	1e-15	7.2e-15
21	1.4213e-05	1.6479e-06	47	24	47	2	0	0	1.2e-15	6.5e-15
22	1.7509e-05	3.2958e-06	49	25	49	2	0	0	1.5e-15	9.4e-15
23	2.0804e-05	3.2958e-06	51	26	51	2	0	0	1.9e-15	8.1e-15
24	2.41e-05	3.2958e-06	53	27	53	2	0	0	1.3e-15	8.4e-15
25	2.7396e-05	3.2958e-06	55	28	55	2	0	0	1.2e-15	7.2e-15
26	3.3987e-05	6.5915e-06	57	29	57	2	0	0	1.3e-15	1.1e-14
27	4.0579e-05	6.5915e-06	59	30	59	2	0	0	1.1e-15	9.2e-15
28	4.717e-05	6.5915e-06	61	31	61	2	0	0	1.7e-15	9.2e-15
29	5.3762e-05	6.5915e-06	63	32	63	2	0	0	1.3e-15	8.2e-15
30	6.0353e-05	6.5915e-06	65	33	65	2	0	0	2.3e-15	7.6e-15
31	7.3536e-05	1.3183e-05	67	34	67	2	0	0	1.9e-15	1.1e-14
32	8.6719e-05	1.3183e-05	69	35	69	2	0	0	2e-15	9.7e-15
33	9.9903e-05	1.3183e-05	71	36	71	2	0	0	2e-15	9.5e-15
34	0.00011309	1.3183e-05	73	37	73	2	0	0	1.3e-15	9.1e-15
35	0.00013945	2.6366e-05	75	38	75	2	0	0	2e-15	1.3e-14
36	0.00016582	2.6366e-05	77	39	77	2	0	0	1.4e-15	1.1e-14
37	0.00019218	2.6366e-05	79	40	79	2	0	0	3.4e-15	1.1e-14
38	0.00021855	2.6366e-05	81	41	81	2	0	0	1.8e-15	1.1e-14
39	0.00027128	5.2732e-05	83	42	83	2	0	0	3e-15	1.5e-14
40	0.00032401	5.2732e-05	85	43	85	2	0	0	1.9e-15	1.3e-14
41	0.00037675	5.2732e-05	87	44	87	2	0	0	2.2e-15	1.3e-14
42	0.00048221	0.00010546	89	45	89	2	0	0	3.6e-15	1.9e-14
43	0.00058767	0.00010546	91	46	91	2	0	0	3e-15	1.5e-14

44	0.00069314	0.00010546		93	47	93	2	0	0	3.1e-15	1.5e-14
45	0.0007986	0.00010546		95	48	95	2	0	0	1.9e-15	1.4e-14
46	0.0010095	0.00021093		97	49	97	2	0	0	3.7e-15	1.9e-14
47	0.0012205	0.00021093		99	50	99	2	0	0	2.9e-15	1.8e-14
48	0.0016423	0.00042186		101	51	101	2	0	0	3e-15	2.9e-14
49	0.0020642	0.00042186		103	52	103	2	0	0	4.8e-15	2.4e-14
50	0.002486	0.00042186		105	53	105	2	0	0	1.9e-15	2.4e-14
51	0.0033297	0.00084371		107	54	107	2	0	0	6.6e-15	3.2e-14
52	0.0041735	0.00084371		109	55	109	2	0	0	3.4e-15	2.5e-14
-	0.005	-	out								
53	0.0050172	0.00084371		111	56	111	2	0	0	5e-15	2.7e-14
54	0.0067046	0.0016874		113	57	113	2	0	0	7.2e-15	4.2e-14
55	0.008392	0.0016874		115	58	115	2	0	0	4.6e-15	3.7e-14
-	0.01	-	out								
56	0.011767	0.0033749		117	59	117	2	0	0	3.9e-15	5.9e-14
-	0.015	-	out								
57	0.015142	0.0033749		119	60	119	2	0	0	2.1e-15	4.9e-14
58	0.018517	0.0033749		121	61	121	2	0	0	7.1e-15	4.9e-14
-	0.02	-	out								
59	0.021891	0.0033749		123	62	123	2	0	0	5e-15	4.1e-14
-	0.025	-	out								
60	0.028641	0.0067497		125	63	125	2	0	0	9.3e-15	5.6e-14
-	0.03	-	out								
-	0.035	-	out								
61	0.035391	0.0067497		127	64	127	2	0	0	4.2e-15	4.5e-14
-	0.04	-	out								
-	0.045	-	out								
62	0.04889	0.013499		129	65	129	2	0	0	7.1e-15	7.5e-14
-	0.05	-	out								
-	0.055	-	out								
-	0.06	-	out								
63	0.06239	0.013499		131	66	131	2	0	0	4.2e-15	6.4e-14
-	0.065	-	out								
-	0.07	-	out								
-	0.075	-	out								
64	0.075889	0.013499		133	67	133	2	0	0	3e-15	6.1e-14
-	0.08	-	out								
-	0.085	-	out								
-	0.09	-	out								
-	0.095	-	out								
-	0.1	-	out								
65	0.10289	0.026999		135	68	135	2	0	0	8.2e-15	6.6e-14
-	0.105	-	out								
-	0.11	-	out								
-	0.115	-	out								
-	0.12	-	out								
-	0.125	-	out								
-	0.13	-	out								
-	0.135	-	out								
-	0.14	-	out								
-	0.145	-	out								
-	0.15	-	out								
-	0.155	-	out								
66	0.15689	0.053998		137	69	137	1	0	0	5.8e-15	5.9e-14
-	0.16	-	out								
-	0.165	-	out								

-	0.17	-	out							
-	0.175	-	out							
-	0.18	-	out							
-	0.185	-	out							
-	0.19	-	out							
-	0.195	-	out							
-	0.2	-	out							
-	0.205	-	out							
-	0.21	-	out							
67	0.21088	0.053998	139	70	139	1	0	0	3.2e-15	3e-14
-	0.215	-	out							
-	0.22	-	out							
-	0.225	-	out							
-	0.23	-	out							
-	0.235	-	out							
-	0.24	-	out							
-	0.245	-	out							
-	0.25	-	out							
-	0.25	-	out							
-	0.27	-	out							
-	0.29	-	out							
68	0.30988	0.099	141	71	141	1	0	0	6.3e-15	3.9e-14
-	0.31	-	out							
-	0.33	-	out							
-	0.35	-	out							
-	0.37	-	out							
-	0.39	-	out							
69	0.40888	0.099	143	72	143	1	0	0	1.3e-14	3.5e-14
-	0.41	-	out							
-	0.43	-	out							
-	0.45	-	out							
-	0.47	-	out							
-	0.49	-	out							
70	0.50788	0.099	145	73	145	1	0	0	3.9e-15	2.9e-14
-	0.51	-	out							
-	0.53	-	out							
-	0.55	-	out							
-	0.57	-	out							
-	0.59	-	out							
71	0.60688	0.099	147	74	147	1	0	0	8.6e-15	2.4e-14
-	0.61	-	out							
-	0.63	-	out							
-	0.65	-	out							
-	0.67	-	out							
-	0.69	-	out							
72	0.70588	0.099	148	75	148	1	0	0	1.1e-14	2.1e-14
-	0.71	-	out							
-	0.73	-	out							
-	0.75	-	out							
-	0.77	-	out							
-	0.79	-	out							
73	0.80488	0.099	149	76	149	1	0	0	5.2e-15	1.8e-14
-	0.81	-	out							
-	0.83	-	out							
-	0.85	-	out							
-	0.87	-	out							

```
   -        0.89            - out
  74      0.90388        0.099       150    77   150      2      0       0  1.3e-14  1.5e-14
   -        0.91            - out
   -        0.93            - out
   -        0.95            - out
   -        0.97            - out
   -        0.99            - out
  75       1.0029        0.099       151    78   151      2      0       0  4.7e-15  9.5e-15
Time-stepping completed.
Solution time: 13 s.
Physical memory: 1.21 GB
Virtual memory: 1.18 GB
Ended at May 28, 2023, 4:07:00 PM.
----- Time-Dependent Solver 1 in Oxygen Pulse/Solution 958 (sol958) ----------->
```

k0Sub=1.51E-9, d=2E-6 (su16)

GENERAL

Description	Value
Solution	k0Sub=1.51E-9, d=2E-6

LOG

```
<---- Compile Equations: Stationary in Oxygen Pulse/Solution 958 (sol958) ------
Started at May 28, 2023, 4:07:03 PM.
Parameter k0Sub = 1.51E-9 (m/s).
Parameter d = 2.0E-6 (m).
Geometry shape function: Linear Lagrange
Number of vertex elements: 10
Minimum element quality: 0.4112
Time: 1 s.
Physical memory: 1.14 GB
Virtual memory: 1.12 GB
Ended at May 28, 2023, 4:07:04 PM.
----- Compile Equations: Stationary in Oxygen Pulse/Solution 958 (sol958) ----->

<---- Dependent Variables 1 in Oxygen Pulse/Solution 958 (sol958) --------------
Started at May 28, 2023, 4:07:04 PM.
Solution time: 0 s.
Physical memory: 1.14 GB
Virtual memory: 1.12 GB
Ended at May 28, 2023, 4:07:04 PM.
----- Dependent Variables 1 in Oxygen Pulse/Solution 958 (sol958) ------------->

<---- Stationary Solver 1 in Oxygen Pulse/Solution 958 (sol958) ----------------
Started at May 28, 2023, 4:07:04 PM.
Linear solver
Number of degrees of freedom solved for: 17907 (plus 938 internal DOFs).
Symmetric matrices found.
Scales for dependent variables:
Concentration (comp2.Oxi): 0.23
Orthonormal null-space function used.
Iter      SolEst     Damping    Stepsize #Res #Jac #Sol   LinErr   LinRes
   1     1.4e-06   1.0000000     1.4e-06    1    1    1  4.3e-13    1e-13
Solution time: 1 s.
Physical memory: 1.14 GB
Virtual memory: 1.12 GB
Ended at May 28, 2023, 4:07:04 PM.
----- Stationary Solver 1 in Oxygen Pulse/Solution 958 (sol958) --------------->

<---- Compile Equations: Oxygen Probe Pulse in Oxygen Pulse/Solution 958
      (sol958) ----------------------------------------------------------------
Started at May 28, 2023, 4:07:04 PM.
Geometry shape function: Linear Lagrange
Time: 1 s.
Physical memory: 1.15 GB
Virtual memory: 1.13 GB
Ended at May 28, 2023, 4:07:05 PM.
----- Compile Equations: Oxygen Probe Pulse in Oxygen Pulse/Solution 958
      (sol958) --------------------------------------------------------------->

<---- Dependent Variables 2 in Oxygen Pulse/Solution 958 (sol958) --------------
Started at May 28, 2023, 4:07:05 PM.
Initial values of variables solved for: Solution 958 (sol958), Solution Store 1 (sol
985).
Values of variables not solved for: Solution 958 (sol958), Solution Store 1 (sol985)
.
Solution time: 0 s.
Physical memory: 1.15 GB
```

Virtual memory: 1.13 GB
Ended at May 28, 2023, 4:07:05 PM.
----- Dependent Variables 2 in Oxygen Pulse/Solution 958 (sol958) ------------->

<---- Time-Dependent Solver 1 in Oxygen Pulse/Solution 958 (sol958) ------------
Started at May 28, 2023, 4:07:05 PM.
Time-dependent solver (BDF)
Number of degrees of freedom solved for: 17907 (plus 35898 internal DOFs).
Symmetric matrices found.
Scales for dependent variables:
Concentration (comp2.Oxi): 0.23

Step	Time	Stepsize		Res	Jac	Sol	Order	Tfail	NLfail	LinErr	LinRes
0	0	-	out	5	3	5			0	7.8e-16	1.5e-15
1	2.5768e-08	2.5768e-08		7	4	7	1	0	0	3.6e-16	1e-15
2	5.1536e-08	2.5768e-08		9	5	9	1	0	0	3.7e-16	1.1e-15
3	1.0307e-07	5.1536e-08		11	6	11	2	0	0	3.5e-16	1.3e-15
4	2.0615e-07	1.0307e-07		13	7	13	2	0	0	5.1e-16	1.8e-15
5	4.1229e-07	2.0615e-07		15	8	15	2	0	0	7.1e-16	3.3e-15
6	6.1844e-07	2.0615e-07		17	9	17	2	0	0	6.4e-16	3.7e-15
7	8.2458e-07	2.0615e-07		19	10	19	2	0	0	7.2e-16	4.1e-15
8	1.0307e-06	2.0615e-07		21	11	21	2	0	0	6.7e-16	3.9e-15
9	1.443e-06	4.1229e-07		23	12	23	2	0	0	9.3e-16	6.4e-15
10	1.8553e-06	4.1229e-07		25	13	25	2	0	0	8.4e-16	6.2e-15
11	2.2676e-06	4.1229e-07		27	14	27	2	0	0	9e-16	6e-15
12	2.6799e-06	4.1229e-07		29	15	29	2	0	0	1.1e-15	5.6e-15
13	3.5045e-06	8.2458e-07		31	16	31	2	0	0	9e-16	8.4e-15
14	4.3291e-06	8.2458e-07		33	17	33	2	0	0	9.7e-16	7.4e-15
15	5.1536e-06	8.2458e-07		35	18	35	2	0	0	1.1e-15	7.1e-15
16	5.9782e-06	8.2458e-07		37	19	37	2	0	0	1.3e-15	6.6e-15
17	7.6274e-06	1.6492e-06		39	20	39	2	0	0	1.1e-15	9.9e-15
18	9.2766e-06	1.6492e-06		41	21	41	2	0	0	1.5e-15	8.5e-15
19	1.0926e-05	1.6492e-06		43	22	43	2	0	0	1.4e-15	8.2e-15
20	1.2575e-05	1.6492e-06		45	23	45	2	0	0	1.2e-15	7.6e-15
21	1.4224e-05	1.6492e-06		47	24	47	2	0	0	1.2e-15	6.7e-15
22	1.7522e-05	3.2983e-06		49	25	49	2	0	0	1.4e-15	9.8e-15
23	2.0821e-05	3.2983e-06		51	26	51	2	0	0	1.4e-15	8.5e-15
24	2.4119e-05	3.2983e-06		53	27	53	2	0	0	2.1e-15	8.7e-15
25	2.7417e-05	3.2983e-06		55	28	55	2	0	0	1.8e-15	7.7e-15
26	3.4014e-05	6.5967e-06		57	29	57	2	0	0	1.7e-15	1.1e-14
27	4.0611e-05	6.5967e-06		59	30	59	2	0	0	2.4e-15	9.6e-15
28	4.7207e-05	6.5967e-06		61	31	61	2	0	0	2e-15	9.7e-15
29	5.3804e-05	6.5967e-06		63	32	63	2	0	0	2e-15	8.7e-15
30	6.0401e-05	6.5967e-06		65	33	65	2	0	0	2e-15	7.4e-15
31	7.3594e-05	1.3193e-05		67	34	67	2	0	0	1.7e-15	1.1e-14
32	8.6787e-05	1.3193e-05		69	35	69	2	0	0	1.6e-15	1e-14
33	9.9981e-05	1.3193e-05		71	36	71	2	0	0	2.5e-15	9.7e-15
34	0.00011317	1.3193e-05		73	37	73	2	0	0	1.4e-15	9e-15
35	0.00013956	2.6387e-05		75	38	75	2	0	0	1.7e-15	1.3e-14
36	0.00016595	2.6387e-05		77	39	77	2	0	0	3.2e-15	1.1e-14
37	0.00019233	2.6387e-05		79	40	79	2	0	0	2e-15	1.1e-14
38	0.00021872	2.6387e-05		81	41	81	2	0	0	2.4e-15	1e-14
39	0.00027149	5.2773e-05		83	42	83	2	0	0	1.9e-15	1.5e-14
40	0.00032427	5.2773e-05		85	43	85	2	0	0	4e-15	1.3e-14
41	0.00037704	5.2773e-05		87	44	87	2	0	0	2.7e-15	1.3e-14
42	0.00048259	0.00010555		89	45	89	2	0	0	2.2e-15	1.8e-14
43	0.00058813	0.00010555		91	46	91	2	0	0	2.2e-15	1.5e-14

44	0.00069368	0.00010555		93	47	93	2	0	0	1.7e-15	1.5e-14
45	0.00079923	0.00010555		95	48	95	2	0	0	2.3e-15	1.2e-14
46	0.0010103	0.00021109		97	49	97	2	0	0	2.1e-15	1.8e-14
47	0.0012214	0.00021109		99	50	99	2	0	0	3.1e-15	1.6e-14
48	0.0016436	0.00042219		101	51	101	2	0	0	4.4e-15	2.6e-14
49	0.0020658	0.00042219		103	52	103	2	0	0	2.4e-15	2.5e-14
50	0.002488	0.00042219		105	53	105	2	0	0	3.1e-15	2.4e-14
51	0.0033323	0.00084437		107	54	107	2	0	0	3e-15	2.9e-14
52	0.0041767	0.00084437		109	55	109	2	0	0	2.2e-15	2.1e-14
-	0.005	-	out								
53	0.0058655	0.0016887		111	56	111	2	0	0	1.5e-15	3.4e-14
54	0.0075542	0.0016887		113	57	113	2	0	0	5.6e-15	3.5e-14
55	0.009243	0.0016887		115	58	115	2	0	0	5e-15	3.6e-14
-	0.01	-	out								
56	0.010932	0.0016887		117	59	117	2	0	0	4.5e-15	3.4e-14
57	0.014309	0.0033775		119	60	119	2	0	0	2.1e-15	4.5e-14
-	0.015	-	out								
58	0.017687	0.0033775		121	61	121	2	0	0	3.1e-15	3.4e-14
-	0.02	-	out								
59	0.024442	0.006755		123	62	123	2	0	0	2.6e-15	5.8e-14
-	0.025	-	out								
-	0.03	-	out								
60	0.031197	0.006755		125	63	125	2	0	0	1.9e-15	5.4e-14
-	0.035	-	out								
61	0.037952	0.006755		127	64	127	2	0	0	4.1e-15	5.2e-14
-	0.04	-	out								
-	0.045	-	out								
-	0.05	-	out								
62	0.051462	0.01351		129	65	129	2	0	0	5.1e-15	7.3e-14
-	0.055	-	out								
-	0.06	-	out								
63	0.064972	0.01351		131	66	131	2	0	0	6.8e-15	5.5e-14
-	0.065	-	out								
-	0.07	-	out								
-	0.075	-	out								
-	0.08	-	out								
-	0.085	-	out								
-	0.09	-	out								
64	0.091992	0.02702		133	67	133	2	0	0	7.2e-15	8.1e-14
-	0.095	-	out								
-	0.1	-	out								
-	0.105	-	out								
-	0.11	-	out								
-	0.115	-	out								
65	0.11901	0.02702		135	68	135	1	0	0	4.7e-15	5.7e-14
-	0.12	-	out								
-	0.125	-	out								
-	0.13	-	out								
-	0.135	-	out								
-	0.14	-	out								
-	0.145	-	out								
-	0.15	-	out								
-	0.155	-	out								
-	0.16	-	out								
-	0.165	-	out								
-	0.17	-	out								

66	0.17305	0.05404		137	69	137	1	0	0	2.3e-15	3e-14
-	0.175	-	out								
-	0.18	-	out								
-	0.185	-	out								
-	0.19	-	out								
-	0.195	-	out								
-	0.2	-	out								
-	0.205	-	out								
-	0.21	-	out								
-	0.215	-	out								
-	0.22	-	out								
-	0.225	-	out								
-	0.23	-	out								
-	0.235	-	out								
-	0.24	-	out								
-	0.245	-	out								
-	0.25	-	out								
-	0.25	-	out								
-	0.27	-	out								
67	0.27205	0.099		139	70	139	1	0	0	3.2e-15	4.6e-14
-	0.29	-	out								
-	0.31	-	out								
-	0.33	-	out								
-	0.35	-	out								
-	0.37	-	out								
68	0.37105	0.099		141	71	141	1	0	0	6.6e-15	4e-14
-	0.39	-	out								
-	0.41	-	out								
-	0.43	-	out								
-	0.45	-	out								
-	0.47	-	out								
69	0.47005	0.099		143	72	143	1	0	0	9.6e-15	3.5e-14
-	0.49	-	out								
-	0.51	-	out								
-	0.53	-	out								
-	0.55	-	out								
70	0.56905	0.099		145	73	145	1	0	0	3.7e-15	2.8e-14
-	0.57	-	out								
-	0.59	-	out								
-	0.61	-	out								
-	0.63	-	out								
-	0.65	-	out								
71	0.66805	0.099		146	74	146	1	0	0	9.2e-15	2.4e-14
-	0.67	-	out								
-	0.69	-	out								
-	0.71	-	out								
-	0.73	-	out								
-	0.75	-	out								
72	0.76705	0.099		147	75	147	1	0	0	1.1e-14	2.1e-14
-	0.77	-	out								
-	0.79	-	out								
-	0.81	-	out								
-	0.83	-	out								
-	0.85	-	out								
73	0.86605	0.099		148	76	148	2	0	0	1.4e-14	1.7e-14
-	0.87	-	out								

```
   -        0.89            - out
   -        0.91            - out
   -        0.93            - out
   -        0.95            - out
  74     0.96505        0.099      149   77  149      2      0       0  1.2e-14  1.1e-14
   -        0.97            - out
   -        0.99            - out
  75      1.0641        0.099      150   78  150      2      0       0  2.1e-14    1e-14
Time-stepping completed.
Solution time: 13 s.
Physical memory: 1.19 GB
Virtual memory: 1.17 GB
Ended at May 28, 2023, 4:07:18 PM.
----- Time-Dependent Solver 1 in Oxygen Pulse/Solution 958 (sol958) ----------->
```

k0Sub=1.51E-9, d=2.4E-6 (su17)

GENERAL

Description	Value
Solution	k0Sub=1.51E-9, d=2.4E-6

LOG

```
<---- Compile Equations: Stationary in Oxygen Pulse/Solution 958 (sol958) ------
Started at May 28, 2023, 4:07:20 PM.
Parameter k0Sub = 1.51E-9 (m/s).
Parameter d = 2.4E-6 (m).
Geometry shape function: Linear Lagrange
Number of vertex elements: 10
Minimum element quality: 0.4544
Time: 1 s.
Physical memory: 1.13 GB
Virtual memory: 1.1 GB
Ended at May 28, 2023, 4:07:21 PM.
----- Compile Equations: Stationary in Oxygen Pulse/Solution 958 (sol958) ----->

<---- Dependent Variables 1 in Oxygen Pulse/Solution 958 (sol958) --------------
Started at May 28, 2023, 4:07:21 PM.
Solution time: 0 s.
Physical memory: 1.13 GB
Virtual memory: 1.11 GB
Ended at May 28, 2023, 4:07:21 PM.
----- Dependent Variables 1 in Oxygen Pulse/Solution 958 (sol958) ------------->

<---- Stationary Solver 1 in Oxygen Pulse/Solution 958 (sol958) ----------------
Started at May 28, 2023, 4:07:21 PM.
Linear solver
Number of degrees of freedom solved for: 18002 (plus 937 internal DOFs).
Symmetric matrices found.
Scales for dependent variables:
Concentration (comp2.Oxi): 0.23
Orthonormal null-space function used.
Iter      SolEst     Damping    Stepsize #Res #Jac #Sol   LinErr   LinRes
   1     1.2e-06   1.0000000     1.2e-06    1    1    1  4.3e-13  9.7e-14
Solution time: 1 s.
Physical memory: 1.16 GB
Virtual memory: 1.14 GB
Ended at May 28, 2023, 4:07:21 PM.
----- Stationary Solver 1 in Oxygen Pulse/Solution 958 (sol958) --------------->

<---- Compile Equations: Oxygen Probe Pulse in Oxygen Pulse/Solution 958
      (sol958) ----------------------------------------------------------------
Started at May 28, 2023, 4:07:21 PM.
Geometry shape function: Linear Lagrange
Time: 1 s.
Physical memory: 1.16 GB
Virtual memory: 1.14 GB
Ended at May 28, 2023, 4:07:22 PM.
----- Compile Equations: Oxygen Probe Pulse in Oxygen Pulse/Solution 958
      (sol958) --------------------------------------------------------------->

<---- Dependent Variables 2 in Oxygen Pulse/Solution 958 (sol958) --------------
Started at May 28, 2023, 4:07:22 PM.
Initial values of variables solved for: Solution 958 (sol958), Solution Store 1 (sol
985).
Values of variables not solved for: Solution 958 (sol958), Solution Store 1 (sol985)
.
Solution time: 0 s.
Physical memory: 1.17 GB
```

```
Virtual memory: 1.14 GB
Ended at May 28, 2023, 4:07:22 PM.
----- Dependent Variables 2 in Oxygen Pulse/Solution 958 (sol958) ------------->

<---- Time-Dependent Solver 1 in Oxygen Pulse/Solution 958 (sol958) ------------
Started at May 28, 2023, 4:07:22 PM.
Time-dependent solver (BDF)
Number of degrees of freedom solved for: 18002 (plus 36084 internal DOFs).
Symmetric matrices found.
Scales for dependent variables:
Concentration (comp2.Oxi): 0.23
Step       Time    Stepsize      Res   Jac   Sol Order Tfail NLfail   LinErr   LinRes
   0          0          - out     5     3     5                    0  8.1e-16  1.5e-15
   1 2.5893e-08  2.5893e-08        7     4     7     1     0        0  3.5e-16    1e-15
   2 5.1785e-08  2.5893e-08        9     5     9     1     0        0  3.6e-16  1.2e-15
   3 1.0357e-07  5.1785e-08       11     6    11     2     0        0  3.8e-16  1.3e-15
   4 2.0714e-07  1.0357e-07       13     7    13     2     0        0  5.4e-16  1.7e-15
   5 4.1428e-07  2.0714e-07       15     8    15     2     0        0  5.7e-16  3.3e-15
   6 6.2142e-07  2.0714e-07       17     9    17     2     0        0  8.2e-16  3.7e-15
   7 8.2856e-07  2.0714e-07       19    10    19     2     0        0  6.7e-16    4e-15
   8 1.0357e-06  2.0714e-07       21    11    21     2     0        0    7e-16  4.1e-15
   9   1.45e-06  4.1428e-07       23    12    23     2     0        0  8.1e-16  6.4e-15
  10 1.8643e-06  4.1428e-07       25    13    25     2     0        0  7.8e-16    6e-15
  11 2.2785e-06  4.1428e-07       27    14    27     2     0        0    8e-16    6e-15
  12 2.6928e-06  4.1428e-07       29    15    29     2     0        0  7.8e-16  5.7e-15
  13 3.5214e-06  8.2856e-07       31    16    31     2     0        0  8.8e-16  8.7e-15
  14   4.35e-06  8.2856e-07       33    17    33     2     0        0  1.1e-15  7.4e-15
  15 5.1785e-06  8.2856e-07       35    18    35     2     0        0  1.1e-15  7.3e-15
  16 6.0071e-06  8.2856e-07       37    19    37     2     0        0  1.1e-15  6.7e-15
  17 7.6642e-06  1.6571e-06       39    20    39     2     0        0  1.5e-15  9.9e-15
  18 9.3213e-06  1.6571e-06       41    21    41     2     0        0  1.2e-15  8.4e-15
  19 1.0978e-05  1.6571e-06       43    22    43     2     0        0  1.1e-15  8.5e-15
  20 1.2636e-05  1.6571e-06       45    23    45     2     0        0  1.2e-15  7.7e-15
  21 1.4293e-05  1.6571e-06       47    24    47     2     0        0  1.5e-15  6.6e-15
  22 1.7607e-05  3.3143e-06       49    25    49     2     0        0  1.5e-15  9.8e-15
  23 2.0921e-05  3.3143e-06       51    26    51     2     0        0  1.2e-15  8.3e-15
  24 2.4235e-05  3.3143e-06       53    27    53     2     0        0  1.5e-15  8.6e-15
  25  2.755e-05  3.3143e-06       55    28    55     2     0        0  1.3e-15    8e-15
  26 3.4178e-05  6.6285e-06       57    29    57     2     0        0  1.7e-15  1.1e-14
  27 4.0807e-05  6.6285e-06       59    30    59     2     0        0  1.8e-15  9.9e-15
  28 4.7435e-05  6.6285e-06       61    31    61     2     0        0  1.6e-15  9.7e-15
  29 5.4064e-05  6.6285e-06       63    32    63     2     0        0  1.5e-15    9e-15
  30 6.0692e-05  6.6285e-06       65    33    65     2     0        0  1.8e-15    8e-15
  31 7.3949e-05  1.3257e-05       67    34    67     2     0        0  2.4e-15  1.1e-14
  32 8.7206e-05  1.3257e-05       69    35    69     2     0        0    2e-15    1e-14
  33 0.00010046  1.3257e-05       71    36    71     2     0        0  1.6e-15    1e-14
  34 0.00011372  1.3257e-05       73    37    73     2     0        0  1.8e-15  9.3e-15
  35 0.00014023  2.6514e-05       75    38    75     2     0        0  2.1e-15  1.4e-14
  36 0.00016675  2.6514e-05       77    39    77     2     0        0  1.3e-15  1.2e-14
  37 0.00019326  2.6514e-05       79    40    79     2     0        0  2.1e-15  1.2e-14
  38 0.00021978  2.6514e-05       81    41    81     2     0        0  2.3e-15    1e-14
  39  0.0002728  5.3028e-05       83    42    83     2     0        0  1.9e-15  1.5e-14
  40 0.00032583  5.3028e-05       85    43    85     2     0        0  1.9e-15  1.3e-14
  41 0.00037886  5.3028e-05       87    44    87     2     0        0  1.9e-15  1.3e-14
  42 0.00048492  0.00010606       89    45    89     2     0        0  1.7e-15  1.9e-14
  43 0.00059097  0.00010606       91    46    91     2     0        0  3.9e-15  1.5e-14
```

44	0.00069703	0.00010606		93	47	93	2	0	0	3.1e-15	1.6e-14
45	0.00080308	0.00010606		95	48	95	2	0	0	2.1e-15	1.3e-14
46	0.0010152	0.00021211		97	49	97	2	0	0	3.6e-15	1.8e-14
47	0.0012273	0.00021211		99	50	99	2	0	0	1.5e-15	1.6e-14
48	0.0016515	0.00042422		101	51	101	2	0	0	1.8e-15	2.6e-14
49	0.0020758	0.00042422		103	52	103	2	0	0	4e-15	2.5e-14
50	0.0025	0.00042422		105	53	105	2	0	0	1.8e-15	2.4e-14
51	0.0029242	0.00042422		107	54	107	2	0	0	4.7e-15	2.2e-14
52	0.0037727	0.00084845		109	55	109	2	0	0	1.8e-15	2.5e-14
53	0.0046211	0.00084845		111	56	111	2	0	0	3.7e-15	1.9e-14
-	0.005	-	out								
54	0.006318	0.0016969		113	57	113	2	0	0	3.2e-15	3.2e-14
55	0.0080149	0.0016969		115	58	115	2	0	0	6e-15	3.4e-14
-	0.01	-	out								
56	0.011409	0.0033938		117	59	117	2	0	0	3.5e-15	5.1e-14
57	0.014802	0.0033938		119	60	119	2	0	0	5.3e-15	4.7e-14
-	0.015	-	out								
58	0.018196	0.0033938		121	61	121	2	0	0	4.2e-15	4.5e-14
-	0.02	-	out								
59	0.024984	0.0067876		123	62	123	2	0	0	7e-15	5.9e-14
-	0.025	-	out								
-	0.03	-	out								
60	0.031771	0.0067876		125	63	125	2	0	0	4e-15	4.7e-14
-	0.035	-	out								
-	0.04	-	out								
-	0.045	-	out								
61	0.045347	0.013575		127	64	127	2	0	0	3.2e-15	7.6e-14
-	0.05	-	out								
-	0.055	-	out								
62	0.058922	0.013575		129	65	129	2	0	0	7.2e-15	6.4e-14
-	0.06	-	out								
-	0.065	-	out								
-	0.07	-	out								
63	0.072497	0.013575		131	66	131	2	0	0	3.1e-15	6.6e-14
-	0.075	-	out								
-	0.08	-	out								
-	0.085	-	out								
64	0.086072	0.013575		133	67	133	2	0	0	3e-15	5.1e-14
-	0.09	-	out								
-	0.095	-	out								
-	0.1	-	out								
-	0.105	-	out								
-	0.11	-	out								
65	0.11322	0.02715		135	68	135	2	0	0	5.9e-15	4.7e-14
-	0.115	-	out								
-	0.12	-	out								
-	0.125	-	out								
-	0.13	-	out								
-	0.135	-	out								
-	0.14	-	out								
-	0.145	-	out								
-	0.15	-	out								
-	0.155	-	out								
-	0.16	-	out								
-	0.165	-	out								
66	0.16752	0.054301		137	69	137	1	0	0	2.6e-15	5.1e-14

-	0.17	-	out							
-	0.175	-	out							
-	0.18	-	out							
-	0.185	-	out							
-	0.19	-	out							
-	0.195	-	out							
-	0.2	-	out							
-	0.205	-	out							
-	0.21	-	out							
-	0.215	-	out							
-	0.22	-	out							
67	0.22182	0.054301	139	70	139	1	0	0	8.8e-15	3.3e-14
-	0.225	-	out							
-	0.23	-	out							
-	0.235	-	out							
-	0.24	-	out							
-	0.245	-	out							
-	0.25	-	out							
-	0.25	-	out							
-	0.27	-	out							
-	0.29	-	out							
-	0.31	-	out							
68	0.32082	0.099	141	71	141	1	0	0	3.4e-15	4.1e-14
-	0.33	-	out							
-	0.35	-	out							
-	0.37	-	out							
-	0.39	-	out							
-	0.41	-	out							
69	0.41982	0.099	143	72	143	1	0	0	1.1e-14	3.6e-14
-	0.43	-	out							
-	0.45	-	out							
-	0.47	-	out							
-	0.49	-	out							
-	0.51	-	out							
70	0.51882	0.099	145	73	145	1	0	0	1.3e-14	3e-14
-	0.53	-	out							
-	0.55	-	out							
-	0.57	-	out							
-	0.59	-	out							
-	0.61	-	out							
71	0.61782	0.099	147	74	147	1	0	0	1e-14	2.6e-14
-	0.63	-	out							
-	0.65	-	out							
-	0.67	-	out							
-	0.69	-	out							
-	0.71	-	out							
72	0.71682	0.099	148	75	148	1	0	0	3.7e-15	2.1e-14
-	0.73	-	out							
-	0.75	-	out							
-	0.77	-	out							
-	0.79	-	out							
-	0.81	-	out							
73	0.81582	0.099	149	76	149	1	0	0	5.7e-15	2e-14
-	0.83	-	out							
-	0.85	-	out							
-	0.87	-	out							

```
   -        0.89           - out
   -        0.91           - out
  74     0.91482       0.099      150   77  150      2      0       0  1.1e-14  1.6e-14
   -        0.93           - out
   -        0.95           - out
   -        0.97           - out
   -        0.99           - out
  75      1.0138       0.099      151   78  151      2      0       0  1.9e-14  9.5e-15
Time-stepping completed.
Solution time: 13 s.
Physical memory: 1.19 GB
Virtual memory: 1.17 GB
Ended at May 28, 2023, 4:07:35 PM.
----- Time-Dependent Solver 1 in Oxygen Pulse/Solution 958 (sol958) ----------->
```

k0Sub=1.51E-9, d=3E-6 (su18)

GENERAL

Description	Value
Solution	k0Sub=1.51E-9, d=3E-6

LOG

```
<---- Compile Equations: Stationary in Oxygen Pulse/Solution 958 (sol958) ------
Started at May 28, 2023, 4:07:37 PM.
Parameter k0Sub = 1.51E-9 (m/s).
Parameter d = 3.0E-6 (m).
Geometry shape function: Linear Lagrange
Number of vertex elements: 10
Minimum element quality: 0.4275
Time: 1 s.
Physical memory: 1.18 GB
Virtual memory: 1.16 GB
Ended at May 28, 2023, 4:07:38 PM.
----- Compile Equations: Stationary in Oxygen Pulse/Solution 958 (sol958) ----->

<---- Dependent Variables 1 in Oxygen Pulse/Solution 958 (sol958) --------------
Started at May 28, 2023, 4:07:38 PM.
Solution time: 0 s.
Physical memory: 1.18 GB
Virtual memory: 1.16 GB
Ended at May 28, 2023, 4:07:38 PM.
----- Dependent Variables 1 in Oxygen Pulse/Solution 958 (sol958) ------------->

<---- Stationary Solver 1 in Oxygen Pulse/Solution 958 (sol958) ----------------
Started at May 28, 2023, 4:07:38 PM.
Linear solver
Number of degrees of freedom solved for: 22990 (plus 935 internal DOFs).
Symmetric matrices found.
Scales for dependent variables:
Concentration (comp2.Oxi): 0.23
Orthonormal null-space function used.
Iter      SolEst     Damping    Stepsize #Res #Jac #Sol   LinErr   LinRes
   1     9.5e-07   1.0000000     9.5e-07    1    1    1  4.5e-13  9.3e-14
Solution time: 1 s.
Physical memory: 1.19 GB
Virtual memory: 1.17 GB
Ended at May 28, 2023, 4:07:38 PM.
----- Stationary Solver 1 in Oxygen Pulse/Solution 958 (sol958) --------------->

<---- Compile Equations: Oxygen Probe Pulse in Oxygen Pulse/Solution 958
      (sol958) ----------------------------------------------------------------
Started at May 28, 2023, 4:07:38 PM.
Geometry shape function: Linear Lagrange
Time: 1 s.
Physical memory: 1.19 GB
Virtual memory: 1.17 GB
Ended at May 28, 2023, 4:07:39 PM.
----- Compile Equations: Oxygen Probe Pulse in Oxygen Pulse/Solution 958
      (sol958) --------------------------------------------------------------->

<---- Dependent Variables 2 in Oxygen Pulse/Solution 958 (sol958) --------------
Started at May 28, 2023, 4:07:39 PM.
Initial values of variables solved for: Solution 958 (sol958), Solution Store 1 (sol
985).
Values of variables not solved for: Solution 958 (sol958), Solution Store 1 (sol985)
.
Solution time: 0 s.
Physical memory: 1.19 GB
```

Virtual memory: 1.17 GB
Ended at May 28, 2023, 4:07:39 PM.
----- Dependent Variables 2 in Oxygen Pulse/Solution 958 (sol958) ------------->

<---- Time-Dependent Solver 1 in Oxygen Pulse/Solution 958 (sol958) ------------
Started at May 28, 2023, 4:07:39 PM.
Time-dependent solver (BDF)
Number of degrees of freedom solved for: 22990 (plus 46056 internal DOFs).
Symmetric matrices found.
Scales for dependent variables:
Concentration (comp2.Oxi): 0.23

Step	Time	Stepsize	Res	Jac	Sol	Order	Tfail	NLfail	LinErr	LinRes
0	0	- out	5	3	5			0	5.5e-16	8.8e-16
1	3.5548e-08	3.5548e-08	7	4	7	1	0	0	3.1e-16	9.1e-16
2	7.1096e-08	3.5548e-08	9	5	9	1	0	0	3.3e-16	1e-15
3	1.4219e-07	7.1096e-08	11	6	11	2	0	0	3.2e-16	9.9e-16
4	2.8439e-07	1.4219e-07	13	7	13	2	0	0	4.2e-16	1.3e-15
5	5.6877e-07	2.8439e-07	15	8	15	2	0	0	4.4e-16	2.3e-15
6	8.5316e-07	2.8439e-07	17	9	17	2	0	0	4.8e-16	2.7e-15
7	1.1375e-06	2.8439e-07	19	10	19	2	0	0	5.2e-16	3.1e-15
8	1.4219e-06	2.8439e-07	21	11	21	2	0	0	5.3e-16	3.2e-15
9	1.9907e-06	5.6877e-07	23	12	23	2	0	0	7.7e-16	5e-15
10	2.5595e-06	5.6877e-07	25	13	25	2	0	0	6.7e-16	4.7e-15
11	3.1282e-06	5.6877e-07	27	14	27	2	0	0	7.4e-16	4.7e-15
12	3.697e-06	5.6877e-07	29	15	29	2	0	0	5.5e-16	4.4e-15
13	4.8346e-06	1.1375e-06	31	16	31	2	0	0	8.6e-16	7.2e-15
14	5.9721e-06	1.1375e-06	33	17	33	2	0	0	9e-16	6.2e-15
15	7.1096e-06	1.1375e-06	35	18	35	2	0	0	8.3e-16	6.3e-15
16	8.2472e-06	1.1375e-06	37	19	37	2	0	0	8.5e-16	5.8e-15
17	1.0522e-05	2.2751e-06	39	20	39	2	0	0	1.2e-15	9.1e-15
18	1.2797e-05	2.2751e-06	41	21	41	2	0	0	1.2e-15	7.9e-15
19	1.5072e-05	2.2751e-06	43	22	43	2	0	0	1.1e-15	7.7e-15
20	1.7348e-05	2.2751e-06	45	23	45	2	0	0	1.2e-15	7.2e-15
21	2.1898e-05	4.5502e-06	47	24	47	2	0	0	1.1e-15	1.1e-14
22	2.6448e-05	4.5502e-06	49	25	49	2	0	0	2.1e-15	1e-14
23	3.0998e-05	4.5502e-06	51	26	51	2	0	0	1.7e-15	9.7e-15
24	3.5548e-05	4.5502e-06	53	27	53	2	0	0	1.2e-15	9e-15
25	4.4648e-05	9.1003e-06	55	28	55	2	0	0	1.7e-15	1.4e-14
26	5.3749e-05	9.1003e-06	57	29	57	2	0	0	1.5e-15	1.1e-14
27	6.2849e-05	9.1003e-06	59	30	59	2	0	0	2.3e-15	1.2e-14
28	7.1949e-05	9.1003e-06	61	31	61	2	0	0	9.2e-16	1.1e-14
29	9.015e-05	1.8201e-05	63	32	63	2	0	0	2.3e-15	1.5e-14
30	0.00010835	1.8201e-05	65	33	65	2	0	0	1.7e-15	1.3e-14
31	0.00012655	1.8201e-05	67	34	67	2	0	0	1.6e-15	1.4e-14
32	0.00014475	1.8201e-05	69	35	69	2	0	0	1.5e-15	1.3e-14
33	0.00018115	3.6401e-05	71	36	71	2	0	0	2.9e-15	1.8e-14
34	0.00021755	3.6401e-05	73	37	73	2	0	0	2.5e-15	1.5e-14
35	0.00025396	3.6401e-05	75	38	75	2	0	0	2.5e-15	1.5e-14
36	0.00029036	3.6401e-05	77	39	77	2	0	0	1.6e-15	1.4e-14
37	0.00036316	7.2803e-05	79	40	79	2	0	0	1.3e-15	2.1e-14
38	0.00043596	7.2803e-05	81	41	81	2	0	0	5.4e-15	1.8e-14
39	0.00058157	0.00014561	83	42	83	2	0	0	4.9e-15	2.8e-14
40	0.00072717	0.00014561	85	43	85	2	0	0	3.2e-15	2.3e-14
41	0.00087278	0.00014561	87	44	87	2	0	0	5.1e-15	2.4e-14
42	0.0010184	0.00014561	89	45	89	2	0	0	2.3e-15	1.9e-14
43	0.0013096	0.00029121	91	46	91	2	0	0	1.6e-15	2.5e-14

44	0.0016008	0.00029121		93	47	93	2	0	0	1.5e-15	1.9e-14
45	0.0021832	0.00058242		95	48	95	2	0	0	6.2e-15	3.2e-14
46	0.0027656	0.00058242		97	49	97	2	0	0	3e-15	3.3e-14
47	0.0033481	0.00058242		99	50	99	2	0	0	2.6e-15	3.3e-14
48	0.0045129	0.0011648		101	51	101	2	0	0	2.1e-15	4.2e-14
-	0.005	-	out								
49	0.0056778	0.0011648		103	52	103	2	0	0	4.2e-15	2.7e-14
50	0.0080074	0.0023297		105	53	105	2	0	0	6.8e-15	4.6e-14
-	0.01	-	out								
51	0.010337	0.0023297		107	54	107	2	0	0	2.5e-15	4.4e-14
52	0.012667	0.0023297		109	55	109	2	0	0	3.7e-15	4.7e-14
-	0.015	-	out								
53	0.017326	0.0046594		111	56	111	2	0	0	9.2e-15	6.4e-14
-	0.02	-	out								
54	0.021986	0.0046594		113	57	113	2	0	0	2.2e-15	4.8e-14
-	0.025	-	out								
-	0.03	-	out								
55	0.031304	0.0093187		115	58	115	2	0	0	6.3e-15	8.1e-14
-	0.035	-	out								
-	0.04	-	out								
56	0.040623	0.0093187		117	59	117	2	0	0	1.1e-14	7.2e-14
-	0.045	-	out								
57	0.049942	0.0093187		119	60	119	2	0	0	5.2e-15	7.2e-14
-	0.05	-	out								
-	0.055	-	out								
-	0.06	-	out								
-	0.065	-	out								
58	0.068579	0.018637		121	61	121	2	0	0	5.2e-15	9.6e-14
-	0.07	-	out								
-	0.075	-	out								
-	0.08	-	out								
-	0.085	-	out								
59	0.087217	0.018637		123	62	123	1	0	0	1.2e-14	8.5e-14
-	0.09	-	out								
-	0.095	-	out								
-	0.1	-	out								
-	0.105	-	out								
-	0.11	-	out								
-	0.115	-	out								
-	0.12	-	out								
60	0.12449	0.037275		125	63	125	1	0	0	2.4e-14	6.3e-14
-	0.125	-	out								
-	0.13	-	out								
-	0.135	-	out								
-	0.14	-	out								
-	0.145	-	out								
-	0.15	-	out								
-	0.155	-	out								
-	0.16	-	out								
-	0.165	-	out								
-	0.17	-	out								
-	0.175	-	out								
-	0.18	-	out								
-	0.185	-	out								
-	0.19	-	out								
-	0.195	-	out								

61	0.19904	0.07455		127	64	127	1	0	0	4.9e-15	8.4e-14
-	0.2	-	out								
-	0.205	-	out								
-	0.21	-	out								
-	0.215	-	out								
-	0.22	-	out								
-	0.225	-	out								
-	0.23	-	out								
-	0.235	-	out								
-	0.24	-	out								
-	0.245	-	out								
-	0.25	-	out								
-	0.25	-	out								
-	0.27	-	out								
62	0.27359	0.07455		129	65	129	1	0	0	6.6e-15	7e-14
-	0.29	-	out								
-	0.31	-	out								
-	0.33	-	out								
-	0.35	-	out								
-	0.37	-	out								
63	0.37259	0.099		131	66	131	1	0	0	5.1e-15	6.7e-14
-	0.39	-	out								
-	0.41	-	out								
-	0.43	-	out								
-	0.45	-	out								
-	0.47	-	out								
64	0.47159	0.099		133	67	133	1	0	0	5.2e-15	5.3e-14
-	0.49	-	out								
-	0.51	-	out								
-	0.53	-	out								
-	0.55	-	out								
-	0.57	-	out								
65	0.57059	0.099		135	68	135	1	0	0	1.6e-14	4.2e-14
-	0.59	-	out								
-	0.61	-	out								
-	0.63	-	out								
-	0.65	-	out								
66	0.66959	0.099		136	69	136	1	0	0	4.7e-15	3.6e-14
-	0.67	-	out								
-	0.69	-	out								
-	0.71	-	out								
-	0.73	-	out								
-	0.75	-	out								
67	0.76859	0.099		137	70	137	1	0	0	4.9e-15	3e-14
-	0.77	-	out								
-	0.79	-	out								
-	0.81	-	out								
-	0.83	-	out								
-	0.85	-	out								
68	0.86759	0.099		138	71	138	2	0	0	8.5e-15	2.5e-14
-	0.87	-	out								
-	0.89	-	out								
-	0.91	-	out								
-	0.93	-	out								
-	0.95	-	out								
69	0.96659	0.099		139	72	139	2	0	0	5.7e-15	1.7e-14

```
   -        0.97           - out
   -        0.99           - out
  70      1.0656       0.099      140    73  140      2      0       0  3.1e-15  1.5e-14
Time-stepping completed.
Solution time: 16 s.
Physical memory: 1.21 GB
Virtual memory: 1.2 GB
Ended at May 28, 2023, 4:07:55 PM.
----- Time-Dependent Solver 1 in Oxygen Pulse/Solution 958 (sol958) ----------->
```

k0Sub=1.51E-9, d=4E-6 (su19)

GENERAL

Description	Value
Solution	k0Sub=1.51E-9, d=4E-6

LOG

```
<---- Compile Equations: Stationary in Oxygen Pulse/Solution 958 (sol958) ------
Started at May 28, 2023, 4:07:58 PM.
Parameter k0Sub = 1.51E-9 (m/s).
Parameter d = 4.0E-6 (m).
Geometry shape function: Linear Lagrange
Number of vertex elements: 10
Minimum element quality: 0.4317
Time: 1 s.
Physical memory: 1.18 GB
Virtual memory: 1.17 GB
Ended at May 28, 2023, 4:07:58 PM.
----- Compile Equations: Stationary in Oxygen Pulse/Solution 958 (sol958) ----->

<---- Dependent Variables 1 in Oxygen Pulse/Solution 958 (sol958) --------------
Started at May 28, 2023, 4:07:58 PM.
Solution time: 0 s.
Physical memory: 1.18 GB
Virtual memory: 1.17 GB
Ended at May 28, 2023, 4:07:58 PM.
----- Dependent Variables 1 in Oxygen Pulse/Solution 958 (sol958) ------------->

<---- Stationary Solver 1 in Oxygen Pulse/Solution 958 (sol958) ----------------
Started at May 28, 2023, 4:07:58 PM.
Linear solver
Number of degrees of freedom solved for: 18182 (plus 936 internal DOFs).
Symmetric matrices found.
Scales for dependent variables:
Concentration (comp2.Oxi): 0.23
Orthonormal null-space function used.
Iter      SolEst     Damping    Stepsize #Res #Jac #Sol   LinErr   LinRes
   1     9.2e-07   1.0000000     9.2e-07    1    1    1  3.4e-13  7.7e-14
Solution time: 1 s.
Physical memory: 1.18 GB
Virtual memory: 1.17 GB
Ended at May 28, 2023, 4:07:59 PM.
----- Stationary Solver 1 in Oxygen Pulse/Solution 958 (sol958) --------------->

<---- Compile Equations: Oxygen Probe Pulse in Oxygen Pulse/Solution 958
      (sol958) ----------------------------------------------------------------
Started at May 28, 2023, 4:07:59 PM.
Geometry shape function: Linear Lagrange
Time: 1 s.
Physical memory: 1.18 GB
Virtual memory: 1.15 GB
Ended at May 28, 2023, 4:07:59 PM.
----- Compile Equations: Oxygen Probe Pulse in Oxygen Pulse/Solution 958
      (sol958) --------------------------------------------------------------->

<---- Dependent Variables 2 in Oxygen Pulse/Solution 958 (sol958) --------------
Started at May 28, 2023, 4:07:59 PM.
Initial values of variables solved for: Solution 958 (sol958), Solution Store 1 (sol
985).
Values of variables not solved for: Solution 958 (sol958), Solution Store 1 (sol985)
.
Solution time: 0 s.
Physical memory: 1.18 GB
```

Virtual memory: 1.15 GB
Ended at May 28, 2023, 4:07:59 PM.
----- Dependent Variables 2 in Oxygen Pulse/Solution 958 (sol958) ------------->

<---- Time-Dependent Solver 1 in Oxygen Pulse/Solution 958 (sol958) ------------
Started at May 28, 2023, 4:07:59 PM.
Time-dependent solver (BDF)
Number of degrees of freedom solved for: 18182 (plus 36438 internal DOFs).
Symmetric matrices found.
Scales for dependent variables:
Concentration (comp2.Oxi): 0.23

Step	Time	Stepsize		Res	Jac	Sol	Order	Tfail	NLfail	LinErr	LinRes
0	0	-	out	5	3	5			0	7.7e-16	1.4e-15
1	2.614e-08	2.614e-08		7	4	7	1	0	0	3.4e-16	1e-15
2	5.2279e-08	2.614e-08		9	5	9	1	0	0	3.6e-16	1.2e-15
3	1.0456e-07	5.2279e-08		11	6	11	2	0	0	3.8e-16	1.3e-15
4	2.0912e-07	1.0456e-07		13	7	13	2	0	0	4.9e-16	1.8e-15
5	4.1823e-07	2.0912e-07		15	8	15	2	0	0	5.6e-16	3.2e-15
6	6.2735e-07	2.0912e-07		17	9	17	2	0	0	6.9e-16	3.6e-15
7	8.3647e-07	2.0912e-07		19	10	19	2	0	0	6.5e-16	4e-15
8	1.0456e-06	2.0912e-07		21	11	21	2	0	0	6.7e-16	3.9e-15
9	1.4638e-06	4.1823e-07		23	12	23	2	0	0	7.6e-16	6.3e-15
10	1.882e-06	4.1823e-07		25	13	25	2	0	0	7.8e-16	6.2e-15
11	2.3003e-06	4.1823e-07		27	14	27	2	0	0	8.2e-16	5.9e-15
12	2.7185e-06	4.1823e-07		29	15	29	2	0	0	8e-16	5.8e-15
13	3.555e-06	8.3647e-07		31	16	31	2	0	0	9e-16	8.3e-15
14	4.3914e-06	8.3647e-07		33	17	33	2	0	0	1.1e-15	7.5e-15
15	5.2279e-06	8.3647e-07		35	18	35	2	0	0	1.5e-15	7.2e-15
16	6.0644e-06	8.3647e-07		37	19	37	2	0	0	1.1e-15	6.6e-15
17	7.7373e-06	1.6729e-06		39	20	39	2	0	0	1.3e-15	9.7e-15
18	9.4102e-06	1.6729e-06		41	21	41	2	0	0	1e-15	8.3e-15
19	1.1083e-05	1.6729e-06		43	22	43	2	0	0	1.5e-15	8.1e-15
20	1.2756e-05	1.6729e-06		45	23	45	2	0	0	8.2e-16	7.6e-15
21	1.4429e-05	1.6729e-06		47	24	47	2	0	0	2e-15	6.8e-15
22	1.7775e-05	3.3459e-06		49	25	49	2	0	0	1.9e-15	9.7e-15
23	2.1121e-05	3.3459e-06		51	26	51	2	0	0	1.7e-15	8.8e-15
24	2.4467e-05	3.3459e-06		53	27	53	2	0	0	2.2e-15	8.6e-15
25	2.7813e-05	3.3459e-06		55	28	55	2	0	0	1.4e-15	7.7e-15
26	3.4504e-05	6.6917e-06		57	29	57	2	0	0	1.5e-15	1.1e-14
27	4.1196e-05	6.6917e-06		59	30	59	2	0	0	2.1e-15	9.8e-15
28	4.7888e-05	6.6917e-06		61	31	61	2	0	0	1.9e-15	1e-14
29	5.4579e-05	6.6917e-06		63	32	63	2	0	0	2.3e-15	8.8e-15
30	6.1271e-05	6.6917e-06		65	33	65	2	0	0	1.6e-15	7.9e-15
31	7.4655e-05	1.3383e-05		67	34	67	2	0	0	2.1e-15	1.2e-14
32	8.8038e-05	1.3383e-05		69	35	69	2	0	0	1.5e-15	1.1e-14
33	0.00010142	1.3383e-05		71	36	71	2	0	0	1.2e-15	1e-14
34	0.00011481	1.3383e-05		73	37	73	2	0	0	2.2e-15	9.5e-15
35	0.00014157	2.6767e-05		75	38	75	2	0	0	1.7e-15	1.4e-14
36	0.00016834	2.6767e-05		77	39	77	2	0	0	2.8e-15	1.2e-14
37	0.00019511	2.6767e-05		79	40	79	2	0	0	2e-15	1.2e-14
38	0.00022187	2.6767e-05		81	41	81	2	0	0	1.9e-15	1.1e-14
39	0.00027541	5.3534e-05		83	42	83	2	0	0	2.3e-15	1.6e-14
40	0.00032894	5.3534e-05		85	43	85	2	0	0	2.1e-15	1.3e-14
41	0.00038247	5.3534e-05		87	44	87	2	0	0	4e-15	1.3e-14
42	0.00048954	0.00010707		89	45	89	2	0	0	3.1e-15	1.9e-14
43	0.00059661	0.00010707		91	46	91	2	0	0	1.5e-15	1.6e-14

44	0.00070368	0.00010707		93	47	93	2	0	0	4.7e-15	1.6e-14
45	0.00081075	0.00010707		95	48	95	2	0	0	2.1e-15	1.4e-14
46	0.0010249	0.00021414		97	49	97	2	0	0	4.9e-15	1.9e-14
47	0.001239	0.00021414		99	50	99	2	0	0	5.1e-15	1.6e-14
48	0.0016673	0.00042827		101	51	101	2	0	0	4.8e-15	2.6e-14
49	0.0020956	0.00042827		103	52	103	2	0	0	2.1e-15	2.3e-14
50	0.0025238	0.00042827		105	53	105	2	0	0	3.9e-15	2.3e-14
51	0.0029521	0.00042827		107	54	107	2	0	0	2.8e-15	2e-14
52	0.0038086	0.00085654		109	55	109	2	0	0	3.3e-15	2.8e-14
53	0.0046652	0.00085654		111	56	111	2	0	0	3.2e-15	2.2e-14
-	0.005	-	out								
54	0.0063783	0.0017131		113	57	113	2	0	0	8.7e-15	3.3e-14
55	0.0080913	0.0017131		115	58	115	2	0	0	2.1e-15	2.9e-14
-	0.01	-	out								
56	0.011518	0.0034262		117	59	117	2	0	0	6.5e-15	4.4e-14
57	0.014944	0.0034262		119	60	119	1	0	0	5.5e-15	5.4e-14
-	0.015	-	out								
58	0.01837	0.0034262		121	61	121	1	0	0	2.3e-15	4.5e-14
-	0.02	-	out								
-	0.025	-	out								
59	0.025222	0.0068523		123	62	123	1	0	0	3.4e-15	7.2e-14
-	0.03	-	out								
60	0.032075	0.0068523		125	63	125	1	0	0	9.2e-15	6.8e-14
-	0.035	-	out								
-	0.04	-	out								
-	0.045	-	out								
61	0.045779	0.013705		127	64	127	1	0	0	5.9e-15	9.8e-14
-	0.05	-	out								
-	0.055	-	out								
62	0.059484	0.013705		129	65	129	1	0	0	4e-15	8.7e-14
-	0.06	-	out								
-	0.065	-	out								
-	0.07	-	out								
-	0.075	-	out								
-	0.08	-	out								
-	0.085	-	out								
63	0.086893	0.027409		131	66	131	1	0	0	1.6e-15	1.1e-13
-	0.09	-	out								
-	0.095	-	out								
-	0.1	-	out								
-	0.105	-	out								
-	0.11	-	out								
64	0.1143	0.027409		133	67	133	1	0	0	5.8e-15	8.5e-14
-	0.115	-	out								
-	0.12	-	out								
-	0.125	-	out								
-	0.13	-	out								
-	0.135	-	out								
-	0.14	-	out								
-	0.145	-	out								
-	0.15	-	out								
-	0.155	-	out								
-	0.16	-	out								
-	0.165	-	out								
65	0.16912	0.054819		135	68	135	1	0	0	1.9e-14	8.5e-14
-	0.17	-	out								

-	0.175	- out								
-	0.18	- out								
-	0.185	- out								
-	0.19	- out								
-	0.195	- out								
-	0.2	- out								
-	0.205	- out								
-	0.21	- out								
-	0.215	- out								
-	0.22	- out								
66	0.22394	0.054819	137	69	137	1	0	0	1.3e-14	6.5e-14
-	0.225	- out								
-	0.23	- out								
-	0.235	- out								
-	0.24	- out								
-	0.245	- out								
-	0.25	- out								
-	0.25	- out								
-	0.27	- out								
-	0.29	- out								
-	0.31	- out								
67	0.32294	0.099	139	70	139	1	0	0	1.9e-14	6.9e-14
-	0.33	- out								
-	0.35	- out								
-	0.37	- out								
-	0.39	- out								
-	0.41	- out								
68	0.42194	0.099	141	71	141	1	0	0	2.6e-14	4.9e-14
-	0.43	- out								
-	0.45	- out								
-	0.47	- out								
-	0.49	- out								
-	0.51	- out								
69	0.52094	0.099	143	72	143	1	0	0	1.5e-14	3.8e-14
-	0.53	- out								
-	0.55	- out								
-	0.57	- out								
-	0.59	- out								
-	0.61	- out								
70	0.61994	0.099	145	73	145	1	0	0	7.1e-15	2.8e-14
-	0.63	- out								
-	0.65	- out								
-	0.67	- out								
-	0.69	- out								
-	0.71	- out								
71	0.71894	0.099	146	74	146	1	0	0	9.2e-15	2.4e-14
-	0.73	- out								
-	0.75	- out								
-	0.77	- out								
-	0.79	- out								
-	0.81	- out								
72	0.81794	0.099	147	75	147	1	0	0	1.1e-14	2.1e-14
-	0.83	- out								
-	0.85	- out								
-	0.87	- out								
-	0.89	- out								

```
  -       0.91          - out
 73     0.91694       0.099      148   76  148     2     0      0  7.5e-15  1.8e-14
  -       0.93          - out
  -       0.95          - out
  -       0.97          - out
  -       0.99          - out
 74      1.0159       0.099      149   77  149     2     0      0  1.2e-14  1.1e-14
Time-stepping completed.
Solution time: 13 s.
Physical memory: 1.21 GB
Virtual memory: 1.19 GB
Ended at May 28, 2023, 4:08:12 PM.
----- Time-Dependent Solver 1 in Oxygen Pulse/Solution 958 (sol958) ----------->
```

k0Sub=1.51E-9, d=5E-6 (su20)

GENERAL

Description	Value
Solution	k0Sub=1.51E-9, d=5E-6

LOG

```
<---- Compile Equations: Stationary in Oxygen Pulse/Solution 958 (sol958) ------
Started at May 28, 2023, 4:08:15 PM.
Parameter k0Sub = 1.51E-9 (m/s).
Parameter d = 4.9999999999999996E-6 (m).
Geometry shape function: Linear Lagrange
Number of vertex elements: 10
Minimum element quality: 0.4532
Time: 1 s.
Physical memory: 1.18 GB
Virtual memory: 1.16 GB
Ended at May 28, 2023, 4:08:15 PM.
----- Compile Equations: Stationary in Oxygen Pulse/Solution 958 (sol958) ----->

<---- Dependent Variables 1 in Oxygen Pulse/Solution 958 (sol958) --------------
Started at May 28, 2023, 4:08:15 PM.
Solution time: 0 s.
Physical memory: 1.18 GB
Virtual memory: 1.16 GB
Ended at May 28, 2023, 4:08:15 PM.
----- Dependent Variables 1 in Oxygen Pulse/Solution 958 (sol958) ------------->

<---- Stationary Solver 1 in Oxygen Pulse/Solution 958 (sol958) ----------------
Started at May 28, 2023, 4:08:15 PM.
Linear solver
Number of degrees of freedom solved for: 18304 (plus 936 internal DOFs).
Symmetric matrices found.
Scales for dependent variables:
Concentration (comp2.Oxi): 0.23
Orthonormal null-space function used.
Iter      SolEst     Damping    Stepsize #Res #Jac #Sol   LinErr   LinRes
   1     7.9e-07   1.0000000     7.9e-07    1    1    1  4.1e-13    7e-14
Solution time: 1 s.
Physical memory: 1.2 GB
Virtual memory: 1.18 GB
Ended at May 28, 2023, 4:08:16 PM.
----- Stationary Solver 1 in Oxygen Pulse/Solution 958 (sol958) --------------->

<---- Compile Equations: Oxygen Probe Pulse in Oxygen Pulse/Solution 958
      (sol958) ----------------------------------------------------------------
Started at May 28, 2023, 4:08:16 PM.
Geometry shape function: Linear Lagrange
Time: 1 s.
Physical memory: 1.21 GB
Virtual memory: 1.19 GB
Ended at May 28, 2023, 4:08:16 PM.
----- Compile Equations: Oxygen Probe Pulse in Oxygen Pulse/Solution 958
      (sol958) --------------------------------------------------------------->

<---- Dependent Variables 2 in Oxygen Pulse/Solution 958 (sol958) --------------
Started at May 28, 2023, 4:08:16 PM.
Initial values of variables solved for: Solution 958 (sol958), Solution Store 1 (sol
985).
Values of variables not solved for: Solution 958 (sol958), Solution Store 1 (sol985)
.
Solution time: 0 s.
Physical memory: 1.21 GB
```

```
Virtual memory: 1.19 GB
Ended at May 28, 2023, 4:08:16 PM.
----- Dependent Variables 2 in Oxygen Pulse/Solution 958 (sol958) ------------->

<---- Time-Dependent Solver 1 in Oxygen Pulse/Solution 958 (sol958) ------------
Started at May 28, 2023, 4:08:16 PM.
Time-dependent solver (BDF)
Number of degrees of freedom solved for: 18304 (plus 36680 internal DOFs).
Symmetric matrices found.
Scales for dependent variables:
Concentration (comp2.Oxi): 0.23
Step       Time    Stepsize     Res  Jac  Sol Order Tfail NLfail   LinErr   LinRes
   0          0           - out   5    3    5                 0  6.5e-16  1.4e-15
   1 2.6321e-08  2.6321e-08       7    4    7     1     0     0  3.4e-16    1e-15
   2 5.2641e-08  2.6321e-08       9    5    9     1     0     0  3.8e-16  1.2e-15
   3 1.0528e-07  5.2641e-08      11    6   11     2     0     0  3.5e-16  1.3e-15
   4 2.1056e-07  1.0528e-07      13    7   13     2     0     0    5e-16  1.7e-15
   5 4.2113e-07  2.1056e-07      15    8   15     2     0     0  5.7e-16  3.2e-15
   6 6.3169e-07  2.1056e-07      17    9   17     2     0     0  5.4e-16  3.7e-15
   7 8.4226e-07  2.1056e-07      19   10   19     2     0     0  8.1e-16  3.9e-15
   8 1.0528e-06  2.1056e-07      21   11   21     2     0     0  5.9e-16  3.9e-15
   9  1.474e-06  4.2113e-07      23   12   23     2     0     0  8.1e-16  6.2e-15
  10 1.8951e-06  4.2113e-07      25   13   25     2     0     0  8.7e-16    6e-15
  11 2.3162e-06  4.2113e-07      27   14   27     2     0     0  1.1e-15  6.1e-15
  12 2.7373e-06  4.2113e-07      29   15   29     2     0     0  9.7e-16  5.7e-15
  13 3.5796e-06  8.4226e-07      31   16   31     2     0     0  8.3e-16  8.6e-15
  14 4.4219e-06  8.4226e-07      33   17   33     2     0     0  9.7e-16  7.6e-15
  15 5.2641e-06  8.4226e-07      35   18   35     2     0     0  1.2e-15  7.3e-15
  16 6.1064e-06  8.4226e-07      37   19   37     2     0     0    1e-15  6.8e-15
  17 7.7909e-06  1.6845e-06      39   20   39     2     0     0  1.4e-15  9.8e-15
  18 9.4754e-06  1.6845e-06      41   21   41     2     0     0  1.7e-15  8.4e-15
  19  1.116e-05  1.6845e-06      43   22   43     2     0     0  1.2e-15  8.7e-15
  20 1.2844e-05  1.6845e-06      45   23   45     2     0     0  1.3e-15  7.9e-15
  21 1.4529e-05  1.6845e-06      47   24   47     2     0     0  8.9e-16  6.6e-15
  22 1.7898e-05   3.369e-06      49   25   49     2     0     0  2.4e-15    1e-14
  23 2.1267e-05   3.369e-06      51   26   51     2     0     0  1.4e-15  8.9e-15
  24 2.4636e-05   3.369e-06      53   27   53     2     0     0  1.5e-15  8.9e-15
  25 2.8005e-05   3.369e-06      55   28   55     2     0     0  1.4e-15    8e-15
  26 3.4743e-05  6.7381e-06      57   29   57     2     0     0  1.6e-15  1.2e-14
  27 4.1481e-05  6.7381e-06      59   30   59     2     0     0  1.5e-15    1e-14
  28 4.8219e-05  6.7381e-06      61   31   61     2     0     0  3.2e-15    1e-14
  29 5.4957e-05  6.7381e-06      63   32   63     2     0     0  1.5e-15  9.1e-15
  30 6.1695e-05  6.7381e-06      65   33   65     2     0     0  1.4e-15  8.1e-15
  31 7.5172e-05  1.3476e-05      67   34   67     2     0     0  2.4e-15  1.3e-14
  32 8.8648e-05  1.3476e-05      69   35   69     2     0     0  2.5e-15  1.1e-14
  33 0.00010212  1.3476e-05      71   36   71     2     0     0    2e-15  1.1e-14
  34  0.0001156  1.3476e-05      73   37   73     2     0     0  1.7e-15  9.9e-15
  35 0.00014255  2.6952e-05      75   38   75     2     0     0  3.1e-15  1.4e-14
  36  0.0001695  2.6952e-05      77   39   77     2     0     0  1.9e-15  1.3e-14
  37 0.00019646  2.6952e-05      79   40   79     2     0     0  1.6e-15  1.2e-14
  38 0.00022341  2.6952e-05      81   41   81     2     0     0  1.6e-15  1.1e-14
  39 0.00027731  5.3904e-05      83   42   83     2     0     0  1.8e-15  1.6e-14
  40 0.00033122  5.3904e-05      85   43   85     2     0     0  3.2e-15  1.4e-14
  41 0.00038512  5.3904e-05      87   44   87     2     0     0  2.2e-15  1.4e-14
  42 0.00049293  0.00010781      89   45   89     2     0     0  2.1e-15    2e-14
  43 0.00060074  0.00010781      91   46   91     2     0     0  1.7e-15  1.7e-14
```

44	0.00070855	0.00010781		93	47	93	2	0	0	1.8e-15	1.6e-14
45	0.00081636	0.00010781		95	48	95	2	0	0	3.1e-15	1.4e-14
46	0.001032	0.00021562		97	49	97	2	0	0	5.1e-15	2e-14
47	0.0012476	0.00021562		99	50	99	2	0	0	2.5e-15	1.7e-14
48	0.0016788	0.00043124		101	51	101	2	0	0	1.3e-15	2.6e-14
49	0.0021101	0.00043124		103	52	103	2	0	0	2e-15	2.3e-14
50	0.0025413	0.00043124		105	53	105	2	0	0	2.7e-15	2.3e-14
51	0.0029725	0.00043124		107	54	107	2	0	0	3.3e-15	2e-14
52	0.003835	0.00086247		109	55	109	2	0	0	5.1e-15	2.6e-14
53	0.0046975	0.00086247		111	56	111	2	0	0	5.8e-15	2.1e-14
-	0.005	-	out								
54	0.0064224	0.0017249		113	57	113	2	0	0	2.2e-15	3.3e-14
55	0.0081474	0.0017249		115	58	115	2	0	0	2.7e-15	3e-14
-	0.01	-	out								
56	0.011597	0.0034499		117	59	117	2	0	0	1.1e-14	4.4e-14
-	0.015	-	out								
57	0.015047	0.0034499		119	60	119	1	0	0	2.8e-15	5.1e-14
58	0.018497	0.0034499		121	61	121	1	0	0	6.2e-15	4.1e-14
-	0.02	-	out								
-	0.025	-	out								
59	0.025397	0.0068998		123	62	123	1	0	0	4.4e-15	6.5e-14
-	0.03	-	out								
60	0.032297	0.0068998		125	63	125	1	0	0	4.4e-15	6.2e-14
-	0.035	-	out								
-	0.04	-	out								
-	0.045	-	out								
61	0.046096	0.0138		127	64	127	1	0	0	5.7e-15	9.2e-14
-	0.05	-	out								
-	0.055	-	out								
62	0.059896	0.0138		129	65	129	1	0	0	4e-15	8.6e-14
-	0.06	-	out								
-	0.065	-	out								
-	0.07	-	out								
-	0.075	-	out								
-	0.08	-	out								
-	0.085	-	out								
63	0.087495	0.027599		131	66	131	1	0	0	8.9e-15	1.1e-13
-	0.09	-	out								
-	0.095	-	out								
-	0.1	-	out								
-	0.105	-	out								
-	0.11	-	out								
-	0.115	-	out								
64	0.11509	0.027599		133	67	133	1	0	0	6.7e-15	8.4e-14
-	0.12	-	out								
-	0.125	-	out								
-	0.13	-	out								
-	0.135	-	out								
-	0.14	-	out								
-	0.145	-	out								
-	0.15	-	out								
-	0.155	-	out								
-	0.16	-	out								
-	0.165	-	out								
-	0.17	-	out								
65	0.17029	0.055198		135	68	135	1	0	0	1.7e-14	9e-14

-	0.175	- out								
-	0.18	- out								
-	0.185	- out								
-	0.19	- out								
-	0.195	- out								
-	0.2	- out								
-	0.205	- out								
-	0.21	- out								
-	0.215	- out								
-	0.22	- out								
-	0.225	- out								
66	0.22549	0.055198	137	69	137	1	0	0	7e-15	6.7e-14
-	0.23	- out								
-	0.235	- out								
-	0.24	- out								
-	0.245	- out								
-	0.25	- out								
-	0.25	- out								
-	0.27	- out								
-	0.29	- out								
-	0.31	- out								
67	0.32449	0.099	139	70	139	1	0	0	1.2e-14	6.9e-14
-	0.33	- out								
-	0.35	- out								
-	0.37	- out								
-	0.39	- out								
-	0.41	- out								
68	0.42349	0.099	141	71	141	1	0	0	8.6e-15	5.3e-14
-	0.43	- out								
-	0.45	- out								
-	0.47	- out								
-	0.49	- out								
-	0.51	- out								
69	0.52249	0.099	143	72	143	1	0	0	3.2e-15	3.9e-14
-	0.53	- out								
-	0.55	- out								
-	0.57	- out								
-	0.59	- out								
-	0.61	- out								
70	0.62149	0.099	145	73	145	1	0	0	4.4e-15	3.1e-14
-	0.63	- out								
-	0.65	- out								
-	0.67	- out								
-	0.69	- out								
-	0.71	- out								
71	0.72049	0.099	146	74	146	1	0	0	3.6e-15	2.5e-14
-	0.73	- out								
-	0.75	- out								
-	0.77	- out								
-	0.79	- out								
-	0.81	- out								
72	0.81949	0.099	147	75	147	1	0	0	1.5e-14	2.2e-14
-	0.83	- out								
-	0.85	- out								
-	0.87	- out								
-	0.89	- out								

```
   -        0.91           - out
  73     0.91849       0.099       148    76   148      2      0       0  9.4e-15  1.9e-14
   -        0.93           - out
   -        0.95           - out
   -        0.97           - out
   -        0.99           - out
  74      1.0175       0.099       149    77   149      2      0       0  1.3e-15  1.1e-14
Time-stepping completed.
Solution time: 13 s.
Physical memory: 1.22 GB
Virtual memory: 1.2 GB
Ended at May 28, 2023, 4:08:29 PM.
----- Time-Dependent Solver 1 in Oxygen Pulse/Solution 958 (sol958) ----------->
```

k0Sub=1.51E-9, d=6E-6 (su21)

GENERAL

Description	Value
Solution	k0Sub=1.51E-9, d=6E-6

LOG

```
<---- Compile Equations: Stationary in Oxygen Pulse/Solution 958 (sol958) ------
Started at May 28, 2023, 4:08:32 PM.
Parameter k0Sub = 1.51E-9 (m/s).
Parameter d = 6.0E-6 (m).
Geometry shape function: Linear Lagrange
Number of vertex elements: 10
Minimum element quality: 0.432
Time: 1 s.
Physical memory: 1.19 GB
Virtual memory: 1.17 GB
Ended at May 28, 2023, 4:08:33 PM.
----- Compile Equations: Stationary in Oxygen Pulse/Solution 958 (sol958) ----->

<---- Dependent Variables 1 in Oxygen Pulse/Solution 958 (sol958) --------------
Started at May 28, 2023, 4:08:33 PM.
Solution time: 0 s.
Physical memory: 1.19 GB
Virtual memory: 1.17 GB
Ended at May 28, 2023, 4:08:33 PM.
----- Dependent Variables 1 in Oxygen Pulse/Solution 958 (sol958) ------------->

<---- Stationary Solver 1 in Oxygen Pulse/Solution 958 (sol958) ----------------
Started at May 28, 2023, 4:08:33 PM.
Linear solver
Number of degrees of freedom solved for: 18385 (plus 936 internal DOFs).
Symmetric matrices found.
Scales for dependent variables:
Concentration (comp2.Oxi): 0.23
Orthonormal null-space function used.
Iter      SolEst     Damping    Stepsize #Res #Jac #Sol   LinErr   LinRes
   1       7e-07   1.0000000       7e-07    1    1    1  2.9e-13  6.3e-14
Solution time: 1 s.
Physical memory: 1.2 GB
Virtual memory: 1.18 GB
Ended at May 28, 2023, 4:08:33 PM.
----- Stationary Solver 1 in Oxygen Pulse/Solution 958 (sol958) --------------->

<---- Compile Equations: Oxygen Probe Pulse in Oxygen Pulse/Solution 958
      (sol958) ----------------------------------------------------------------
Started at May 28, 2023, 4:08:33 PM.
Geometry shape function: Linear Lagrange
Time: 0 s.
Physical memory: 1.2 GB
Virtual memory: 1.18 GB
Ended at May 28, 2023, 4:08:34 PM.
----- Compile Equations: Oxygen Probe Pulse in Oxygen Pulse/Solution 958
      (sol958) --------------------------------------------------------------->

<---- Dependent Variables 2 in Oxygen Pulse/Solution 958 (sol958) --------------
Started at May 28, 2023, 4:08:34 PM.
Initial values of variables solved for: Solution 958 (sol958), Solution Store 1 (sol
985).
Values of variables not solved for: Solution 958 (sol958), Solution Store 1 (sol985)
.
Solution time: 0 s.
Physical memory: 1.2 GB
```

Virtual memory: 1.18 GB
Ended at May 28, 2023, 4:08:34 PM.
----- Dependent Variables 2 in Oxygen Pulse/Solution 958 (sol958) ------------->

<---- Time-Dependent Solver 1 in Oxygen Pulse/Solution 958 (sol958) ------------
Started at May 28, 2023, 4:08:34 PM.
Time-dependent solver (BDF)
Number of degrees of freedom solved for: 18385 (plus 36840 internal DOFs).
Symmetric matrices found.
Scales for dependent variables:
Concentration (comp2.Oxi): 0.23

Step	Time	Stepsize	Res	Jac	Sol	Order	Tfail	NLfail	LinErr	LinRes
0	0	- out	5	3	5			0	1.1e-15	1.4e-15
1	2.6429e-08	2.6429e-08	7	4	7	1	0	0	3.2e-16	1.1e-15
2	5.2857e-08	2.6429e-08	9	5	9	1	0	0	3.9e-16	1.2e-15
3	1.0571e-07	5.2857e-08	11	6	11	2	0	0	3.6e-16	1.3e-15
4	2.1143e-07	1.0571e-07	13	7	13	2	0	0	4.7e-16	1.7e-15
5	4.2286e-07	2.1143e-07	15	8	15	2	0	0	5.3e-16	3.3e-15
6	6.3429e-07	2.1143e-07	17	9	17	2	0	0	7.3e-16	3.8e-15
7	8.4572e-07	2.1143e-07	19	10	19	2	0	0	6.5e-16	3.8e-15
8	1.0571e-06	2.1143e-07	21	11	21	2	0	0	5.4e-16	3.8e-15
9	1.48e-06	4.2286e-07	23	12	23	2	0	0	7.2e-16	6e-15
10	1.9029e-06	4.2286e-07	25	13	25	2	0	0	6.6e-16	5.8e-15
11	2.3257e-06	4.2286e-07	27	14	27	2	0	0	9.5e-16	6e-15
12	2.7486e-06	4.2286e-07	29	15	29	2	0	0	8.6e-16	5.6e-15
13	3.5943e-06	8.4572e-07	31	16	31	2	0	0	1.2e-15	8.5e-15
14	4.44e-06	8.4572e-07	33	17	33	2	0	0	1e-15	7.3e-15
15	5.2857e-06	8.4572e-07	35	18	35	2	0	0	1.3e-15	7.3e-15
16	6.1315e-06	8.4572e-07	37	19	37	2	0	0	9.9e-16	6.6e-15
17	7.8229e-06	1.6914e-06	39	20	39	2	0	0	1e-15	9.6e-15
18	9.5143e-06	1.6914e-06	41	21	41	2	0	0	1.3e-15	8.3e-15
19	1.1206e-05	1.6914e-06	43	22	43	2	0	0	1.3e-15	8e-15
20	1.2897e-05	1.6914e-06	45	23	45	2	0	0	1.5e-15	7.7e-15
21	1.4589e-05	1.6914e-06	47	24	47	2	0	0	1.3e-15	6.5e-15
22	1.7972e-05	3.3829e-06	49	25	49	2	0	0	1.3e-15	9.7e-15
23	2.1354e-05	3.3829e-06	51	26	51	2	0	0	1.6e-15	8.4e-15
24	2.4737e-05	3.3829e-06	53	27	53	2	0	0	1.7e-15	8.6e-15
25	2.812e-05	3.3829e-06	55	28	55	2	0	0	1.4e-15	7.8e-15
26	3.4886e-05	6.7657e-06	57	29	57	2	0	0	2e-15	1.2e-14
27	4.1652e-05	6.7657e-06	59	30	59	2	0	0	1.9e-15	9.8e-15
28	4.8417e-05	6.7657e-06	61	31	61	2	0	0	1.8e-15	1e-14
29	5.5183e-05	6.7657e-06	63	32	63	2	0	0	1.5e-15	9.1e-15
30	6.1949e-05	6.7657e-06	65	33	65	2	0	0	1.7e-15	8.2e-15
31	7.548e-05	1.3531e-05	67	34	67	2	0	0	2e-15	1.2e-14
32	8.9012e-05	1.3531e-05	69	35	69	2	0	0	1.9e-15	1e-14
33	0.00010254	1.3531e-05	71	36	71	2	0	0	1.4e-15	1.1e-14
34	0.00011607	1.3531e-05	73	37	73	2	0	0	2e-15	9.9e-15
35	0.00014314	2.7063e-05	75	38	75	2	0	0	2.2e-15	1.4e-14
36	0.0001702	2.7063e-05	77	39	77	2	0	0	1.8e-15	1.3e-14
37	0.00019726	2.7063e-05	79	40	79	2	0	0	2.4e-15	1.2e-14
38	0.00022433	2.7063e-05	81	41	81	2	0	0	1.6e-15	1.2e-14
39	0.00027845	5.4126e-05	83	42	83	2	0	0	2.2e-15	1.6e-14
40	0.00033258	5.4126e-05	85	43	85	2	0	0	2e-15	1.4e-14
41	0.0003867	5.4126e-05	87	44	87	2	0	0	3.2e-15	1.4e-14
42	0.00049496	0.00010825	89	45	89	2	0	0	2.1e-15	2.1e-14
43	0.00060321	0.00010825	91	46	91	2	0	0	2.6e-15	1.7e-14

44	0.00071146	0.00010825		93	47	93	2	0	0	2.4e-15	1.7e-14
45	0.00081971	0.00010825		95	48	95	2	0	0	2.7e-15	1.5e-14
46	0.0010362	0.0002165		97	49	97	2	0	0	2.4e-15	2e-14
47	0.0012527	0.0002165		99	50	99	2	0	0	2.5e-15	1.6e-14
48	0.0016857	0.00043301		101	51	101	2	0	0	2.8e-15	2.6e-14
49	0.0021187	0.00043301		103	52	103	2	0	0	2.1e-15	2.2e-14
50	0.0025517	0.00043301		105	53	105	2	0	0	2.4e-15	2.2e-14
51	0.0029847	0.00043301		107	54	107	2	0	0	4.1e-15	2e-14
52	0.0038508	0.00086601		109	55	109	2	0	0	5.2e-15	2.7e-14
53	0.0047168	0.00086601		111	56	111	2	0	0	3.3e-15	2.1e-14
-	0.005	-	out								
54	0.0064488	0.001732		113	57	113	2	0	0	1.5e-15	3.5e-14
55	0.0081808	0.001732		115	58	115	2	0	0	2e-15	3.2e-14
-	0.01	-	out								
56	0.011645	0.0034641		117	59	117	2	0	0	2.1e-15	4.6e-14
-	0.015	-	out								
57	0.015109	0.0034641		119	60	119	1	0	0	1.9e-15	4.7e-14
58	0.018573	0.0034641		121	61	121	1	0	0	3.6e-15	3.7e-14
-	0.02	-	out								
-	0.025	-	out								
59	0.025501	0.0069281		123	62	123	1	0	0	3.6e-15	6.1e-14
-	0.03	-	out								
60	0.032429	0.0069281		125	63	125	1	0	0	3.5e-15	6e-14
-	0.035	-	out								
-	0.04	-	out								
-	0.045	-	out								
61	0.046285	0.013856		127	64	127	1	0	0	1.4e-14	9e-14
-	0.05	-	out								
-	0.055	-	out								
-	0.06	-	out								
62	0.060142	0.013856		129	65	129	1	0	0	6.2e-15	8e-14
-	0.065	-	out								
-	0.07	-	out								
-	0.075	-	out								
-	0.08	-	out								
-	0.085	-	out								
63	0.087854	0.027712		131	66	131	1	0	0	4.8e-15	1e-13
-	0.09	-	out								
-	0.095	-	out								
-	0.1	-	out								
-	0.105	-	out								
-	0.11	-	out								
-	0.115	-	out								
64	0.11557	0.027712		133	67	133	1	0	0	9.1e-15	8.1e-14
-	0.12	-	out								
-	0.125	-	out								
-	0.13	-	out								
-	0.135	-	out								
-	0.14	-	out								
-	0.145	-	out								
-	0.15	-	out								
-	0.155	-	out								
-	0.16	-	out								
-	0.165	-	out								
-	0.17	-	out								
65	0.17099	0.055425		135	68	135	1	0	0	3e-15	9.4e-14

-	0.175	-	out								
-	0.18	-	out								
-	0.185	-	out								
-	0.19	-	out								
-	0.195	-	out								
-	0.2	-	out								
-	0.205	-	out								
-	0.21	-	out								
-	0.215	-	out								
-	0.22	-	out								
-	0.225	-	out								
66	0.22642	0.055425		137	69	137	1	0	0	2.9e-15	7.2e-14
-	0.23	-	out								
-	0.235	-	out								
-	0.24	-	out								
-	0.245	-	out								
-	0.25	-	out								
-	0.25	-	out								
-	0.27	-	out								
-	0.29	-	out								
-	0.31	-	out								
67	0.32542	0.099		139	70	139	1	0	0	5.2e-15	6.9e-14
-	0.33	-	out								
-	0.35	-	out								
-	0.37	-	out								
-	0.39	-	out								
-	0.41	-	out								
68	0.42442	0.099		141	71	141	1	0	0	4.5e-15	5e-14
-	0.43	-	out								
-	0.45	-	out								
-	0.47	-	out								
-	0.49	-	out								
-	0.51	-	out								
69	0.52342	0.099		143	72	143	1	0	0	1.9e-14	4.2e-14
-	0.53	-	out								
-	0.55	-	out								
-	0.57	-	out								
-	0.59	-	out								
-	0.61	-	out								
70	0.62242	0.099		145	73	145	1	0	0	4e-15	3.1e-14
-	0.63	-	out								
-	0.65	-	out								
-	0.67	-	out								
-	0.69	-	out								
-	0.71	-	out								
71	0.72142	0.099		146	74	146	1	0	0	2.2e-15	2.6e-14
-	0.73	-	out								
-	0.75	-	out								
-	0.77	-	out								
-	0.79	-	out								
-	0.81	-	out								
72	0.82042	0.099		147	75	147	1	0	0	6e-15	2.3e-14
-	0.83	-	out								
-	0.85	-	out								
-	0.87	-	out								
-	0.89	-	out								

```
  -        0.91           - out
 73     0.91942       0.099       148    76   148      2      0       0  8.8e-15    2e-14
  -        0.93           - out
  -        0.95           - out
  -        0.97           - out
  -        0.99           - out
 74      1.0184       0.099       149    77   149      2      0       0  5.6e-15  1.3e-14
Time-stepping completed.
Solution time: 13 s.
Physical memory: 1.22 GB
Virtual memory: 1.2 GB
Ended at May 28, 2023, 4:08:47 PM.
----- Time-Dependent Solver 1 in Oxygen Pulse/Solution 958 (sol958) ----------->
```

k0Sub=1.51E-9, d=8E-6 (su22)

GENERAL

Description	Value
Solution	k0Sub=1.51E-9, d=8E-6

LOG

```
<---- Compile Equations: Stationary in Oxygen Pulse/Solution 958 (sol958) ------
Started at May 28, 2023, 4:08:49 PM.
Parameter k0Sub = 1.51E-9 (m/s).
Parameter d = 8.0E-6 (m).
Geometry shape function: Linear Lagrange
Number of vertex elements: 10
Minimum element quality: 0.4468
Time: 0 s.
Physical memory: 1.19 GB
Virtual memory: 1.16 GB
Ended at May 28, 2023, 4:08:50 PM.
----- Compile Equations: Stationary in Oxygen Pulse/Solution 958 (sol958) ----->

<---- Dependent Variables 1 in Oxygen Pulse/Solution 958 (sol958) --------------
Started at May 28, 2023, 4:08:50 PM.
Solution time: 0 s.
Physical memory: 1.19 GB
Virtual memory: 1.16 GB
Ended at May 28, 2023, 4:08:50 PM.
----- Dependent Variables 1 in Oxygen Pulse/Solution 958 (sol958) ------------->

<---- Stationary Solver 1 in Oxygen Pulse/Solution 958 (sol958) ----------------
Started at May 28, 2023, 4:08:50 PM.
Linear solver
Number of degrees of freedom solved for: 23800 (plus 941 internal DOFs).
Symmetric matrices found.
Scales for dependent variables:
Concentration (comp2.Oxi): 0.23
Orthonormal null-space function used.
Iter      SolEst     Damping    Stepsize #Res #Jac #Sol   LinErr   LinRes
   1       5e-07   1.0000000       5e-07    1    1    1    3e-13  6.1e-14
Solution time: 1 s.
Physical memory: 1.19 GB
Virtual memory: 1.17 GB
Ended at May 28, 2023, 4:08:50 PM.
----- Stationary Solver 1 in Oxygen Pulse/Solution 958 (sol958) --------------->

<---- Compile Equations: Oxygen Probe Pulse in Oxygen Pulse/Solution 958
      (sol958) ----------------------------------------------------------------
Started at May 28, 2023, 4:08:50 PM.
Geometry shape function: Linear Lagrange
Time: 0 s.
Physical memory: 1.2 GB
Virtual memory: 1.17 GB
Ended at May 28, 2023, 4:08:51 PM.
----- Compile Equations: Oxygen Probe Pulse in Oxygen Pulse/Solution 958
      (sol958) --------------------------------------------------------------->

<---- Dependent Variables 2 in Oxygen Pulse/Solution 958 (sol958) --------------
Started at May 28, 2023, 4:08:51 PM.
Initial values of variables solved for: Solution 958 (sol958), Solution Store 1 (sol
985).
Values of variables not solved for: Solution 958 (sol958), Solution Store 1 (sol985)
.
Solution time: 0 s.
Physical memory: 1.2 GB
```

Virtual memory: 1.17 GB
Ended at May 28, 2023, 4:08:51 PM.
----- Dependent Variables 2 in Oxygen Pulse/Solution 958 (sol958) ------------->

<---- Time-Dependent Solver 1 in Oxygen Pulse/Solution 958 (sol958) ------------
Started at May 28, 2023, 4:08:51 PM.
Time-dependent solver (BDF)
Number of degrees of freedom solved for: 23800 (plus 47670 internal DOFs).
Symmetric matrices found.
Scales for dependent variables:
Concentration (comp2.Oxi): 0.23

Step	Time	Stepsize	Res	Jac	Sol	Order	Tfail	NLfail	LinErr	LinRes
0	0	- out	5	3	5			0	6.4e-16	8.9e-16
1	3.6251e-08	3.6251e-08	7	4	7	1	0	0	3.3e-16	9.5e-16
2	7.2501e-08	3.6251e-08	9	5	9	1	0	0	3.4e-16	1e-15
3	1.45e-07	7.2501e-08	11	6	11	2	0	0	3.5e-16	1.1e-15
4	2.9e-07	1.45e-07	13	7	13	2	0	0	4e-16	1.3e-15
5	5.8001e-07	2.9e-07	15	8	15	2	0	0	5.5e-16	2.4e-15
6	8.7001e-07	2.9e-07	17	9	17	2	0	0	5.5e-16	2.9e-15
7	1.16e-06	2.9e-07	19	10	19	2	0	0	4.4e-16	3.2e-15
8	1.45e-06	2.9e-07	21	11	21	2	0	0	5.1e-16	3.3e-15
9	2.03e-06	5.8001e-07	23	12	23	2	0	0	7.7e-16	5.1e-15
10	2.61e-06	5.8001e-07	25	13	25	2	0	0	7.7e-16	4.7e-15
11	3.1901e-06	5.8001e-07	27	14	27	2	0	0	8e-16	4.7e-15
12	3.7701e-06	5.8001e-07	29	15	29	2	0	0	6.5e-16	4.4e-15
13	4.9301e-06	1.16e-06	31	16	31	2	0	0	8.1e-16	7.1e-15
14	6.0901e-06	1.16e-06	33	17	33	2	0	0	7.9e-16	6.4e-15
15	7.2501e-06	1.16e-06	35	18	35	2	0	0	6.6e-16	6.3e-15
16	8.4101e-06	1.16e-06	37	19	37	2	0	0	9.6e-16	5.8e-15
17	1.073e-05	2.32e-06	39	20	39	2	0	0	1e-15	8.7e-15
18	1.305e-05	2.32e-06	41	21	41	2	0	0	9.3e-16	7.6e-15
19	1.537e-05	2.32e-06	43	22	43	2	0	0	1e-15	7.7e-15
20	1.769e-05	2.32e-06	45	23	45	2	0	0	9.1e-16	7e-15
21	2.233e-05	4.6401e-06	47	24	47	2	0	0	1.3e-15	1.1e-14
22	2.697e-05	4.6401e-06	49	25	49	2	0	0	1.7e-15	9.5e-15
23	3.1611e-05	4.6401e-06	51	26	51	2	0	0	1.6e-15	9.4e-15
24	3.6251e-05	4.6401e-06	53	27	53	2	0	0	1.3e-15	8.9e-15
25	4.5531e-05	9.2801e-06	55	28	55	2	0	0	1.4e-15	1.3e-14
26	5.4811e-05	9.2801e-06	57	29	57	2	0	0	1.1e-15	1.1e-14
27	6.4091e-05	9.2801e-06	59	30	59	2	0	0	1.9e-15	1.1e-14
28	7.3371e-05	9.2801e-06	61	31	61	2	0	0	1.4e-15	1.1e-14
29	9.1931e-05	1.856e-05	63	32	63	2	0	0	2.1e-15	1.6e-14
30	0.00011049	1.856e-05	65	33	65	2	0	0	1.6e-15	1.4e-14
31	0.00012905	1.856e-05	67	34	67	2	0	0	3.5e-15	1.4e-14
32	0.00014761	1.856e-05	69	35	69	2	0	0	2.9e-15	1.2e-14
33	0.00018473	3.7121e-05	71	36	71	2	0	0	1.2e-15	1.8e-14
34	0.00022185	3.7121e-05	73	37	73	2	0	0	1.3e-15	1.6e-14
35	0.00025897	3.7121e-05	75	38	75	2	0	0	2.4e-15	1.6e-14
36	0.00029609	3.7121e-05	77	39	77	2	0	0	3.3e-15	1.4e-14
37	0.00037034	7.4241e-05	79	40	79	2	0	0	3.8e-15	2.1e-14
38	0.00044458	7.4241e-05	81	41	81	2	0	0	2.7e-15	1.7e-14
39	0.00059306	0.00014848	83	42	83	2	0	0	3.1e-15	2.7e-14
40	0.00074154	0.00014848	85	43	85	2	0	0	2.5e-15	2.4e-14
41	0.00089002	0.00014848	87	44	87	2	0	0	3.1e-15	2.3e-14
42	0.0010385	0.00014848	89	45	89	2	0	0	3.1e-15	2e-14
43	0.0013355	0.00029696	91	46	91	2	0	0	2.7e-15	2.5e-14

44	0.0016324	0.00029696		93	47	93	2	0	0	3.9e-15	2e-14
45	0.0022264	0.00059393		95	48	95	2	0	0	5.5e-15	3.2e-14
46	0.0028203	0.00059393		97	49	97	2	0	0	2e-15	2.7e-14
47	0.0034142	0.00059393		99	50	99	2	0	0	3.8e-15	2.8e-14
48	0.0046021	0.0011879		101	51	101	2	0	0	2.3e-15	3.7e-14
-	0.005	-	out								
49	0.0057899	0.0011879		103	52	103	2	0	0	3.7e-15	2.7e-14
50	0.0081657	0.0023757		105	53	105	2	0	0	1.9e-15	4.2e-14
-	0.01	-	out								
51	0.010541	0.0023757		107	54	107	1	0	0	8.5e-15	5e-14
52	0.012917	0.0023757		109	55	109	1	0	0	2.5e-15	3.3e-14
-	0.015	-	out								
53	0.017669	0.0047514		111	56	111	1	0	0	7.5e-15	5.5e-14
-	0.02	-	out								
-	0.025	-	out								
54	0.027171	0.0095029		113	57	113	1	0	0	5.4e-15	8.8e-14
-	0.03	-	out								
-	0.035	-	out								
55	0.036674	0.0095029		115	58	115	1	0	0	8.6e-15	8.7e-14
-	0.04	-	out								
-	0.045	-	out								
-	0.05	-	out								
-	0.055	-	out								
56	0.05568	0.019006		117	59	117	1	0	0	1.9e-14	1.2e-13
-	0.06	-	out								
-	0.065	-	out								
-	0.07	-	out								
57	0.074686	0.019006		119	60	119	1	0	0	1.2e-14	1.1e-13
-	0.075	-	out								
-	0.08	-	out								
-	0.085	-	out								
-	0.09	-	out								
-	0.095	-	out								
-	0.1	-	out								
-	0.105	-	out								
-	0.11	-	out								
58	0.1127	0.038011		121	61	121	1	0	0	1.5e-14	1.4e-13
-	0.115	-	out								
-	0.12	-	out								
-	0.125	-	out								
-	0.13	-	out								
-	0.135	-	out								
-	0.14	-	out								
-	0.145	-	out								
-	0.15	-	out								
59	0.15071	0.038011		123	62	123	1	0	0	7.1e-15	1.1e-13
-	0.155	-	out								
-	0.16	-	out								
-	0.165	-	out								
-	0.17	-	out								
-	0.175	-	out								
-	0.18	-	out								
-	0.185	-	out								
-	0.19	-	out								
-	0.195	-	out								
-	0.2	-	out								

-	0.205	- out								
-	0.21	- out								
-	0.215	- out								
-	0.22	- out								
-	0.225	- out								
60	0.22673	0.076023	125	63	125	1	0	0	3.3e-15	1.3e-13
-	0.23	- out								
-	0.235	- out								
-	0.24	- out								
-	0.245	- out								
-	0.25	- out								
-	0.25	- out								
-	0.27	- out								
-	0.29	- out								
61	0.30275	0.076023	127	64	127	1	0	0	4e-15	9.6e-14
-	0.31	- out								
-	0.33	- out								
-	0.35	- out								
-	0.37	- out								
-	0.39	- out								
62	0.40175	0.099	129	65	129	1	0	0	2.7e-15	8.7e-14
-	0.41	- out								
-	0.43	- out								
-	0.45	- out								
-	0.47	- out								
-	0.49	- out								
63	0.50075	0.099	131	66	131	1	0	0	6.7e-15	6.4e-14
-	0.51	- out								
-	0.53	- out								
-	0.55	- out								
-	0.57	- out								
-	0.59	- out								
64	0.59975	0.099	133	67	133	1	0	0	3.3e-15	5e-14
-	0.61	- out								
-	0.63	- out								
-	0.65	- out								
-	0.67	- out								
-	0.69	- out								
65	0.69875	0.099	134	68	134	1	0	0	7.6e-15	4.1e-14
-	0.71	- out								
-	0.73	- out								
-	0.75	- out								
-	0.77	- out								
-	0.79	- out								
66	0.79775	0.099	135	69	135	1	0	0	1.1e-14	3.5e-14
-	0.81	- out								
-	0.83	- out								
-	0.85	- out								
-	0.87	- out								
-	0.89	- out								
67	0.89675	0.099	136	70	136	2	0	0	6.1e-15	2.9e-14
-	0.91	- out								
-	0.93	- out								
-	0.95	- out								
-	0.97	- out								
-	0.99	- out								

```
  68      0.99575        0.099      137    71   137     2     0       0  6.6e-15  1.9e-14
Time-stepping completed.
Solution time: 15 s.
Physical memory: 1.22 GB
Virtual memory: 1.21 GB
Ended at May 28, 2023, 4:09:06 PM.
----- Time-Dependent Solver 1 in Oxygen Pulse/Solution 958 (sol958) ----------->
```

k0Sub=1.51E-9, d=1.2E-5 (su23)

GENERAL

Description	Value
Solution	k0Sub=1.51E-9, d=1.2E-5

LOG

```
<---- Compile Equations: Stationary in Oxygen Pulse/Solution 958 (sol958) ------
Started at May 28, 2023, 4:09:09 PM.
Parameter k0Sub = 1.51E-9 (m/s).
Parameter d = 1.2E-5 (m).
Geometry shape function: Linear Lagrange
Number of vertex elements: 10
Minimum element quality: 0.4505
Time: 1 s.
Physical memory: 1.18 GB
Virtual memory: 1.16 GB
Ended at May 28, 2023, 4:09:10 PM.
----- Compile Equations: Stationary in Oxygen Pulse/Solution 958 (sol958) ----->

<---- Dependent Variables 1 in Oxygen Pulse/Solution 958 (sol958) --------------
Started at May 28, 2023, 4:09:10 PM.
Solution time: 0 s.
Physical memory: 1.18 GB
Virtual memory: 1.16 GB
Ended at May 28, 2023, 4:09:10 PM.
----- Dependent Variables 1 in Oxygen Pulse/Solution 958 (sol958) ------------->

<---- Stationary Solver 1 in Oxygen Pulse/Solution 958 (sol958) ----------------
Started at May 28, 2023, 4:09:10 PM.
Linear solver
Number of degrees of freedom solved for: 18769 (plus 948 internal DOFs).
Symmetric matrices found.
Scales for dependent variables:
Concentration (comp2.Oxi): 0.23
Orthonormal null-space function used.
Iter      SolEst     Damping    Stepsize #Res #Jac #Sol   LinErr   LinRes
   1     4.3e-07   1.0000000     4.3e-07    1    1    1  2.3e-13  5.1e-14
Solution time: 1 s.
Physical memory: 1.18 GB
Virtual memory: 1.16 GB
Ended at May 28, 2023, 4:09:10 PM.
----- Stationary Solver 1 in Oxygen Pulse/Solution 958 (sol958) --------------->

<---- Compile Equations: Oxygen Probe Pulse in Oxygen Pulse/Solution 958
      (sol958) ----------------------------------------------------------------
Started at May 28, 2023, 4:09:10 PM.
Geometry shape function: Linear Lagrange
Time: 0 s.
Physical memory: 1.18 GB
Virtual memory: 1.16 GB
Ended at May 28, 2023, 4:09:11 PM.
----- Compile Equations: Oxygen Probe Pulse in Oxygen Pulse/Solution 958
      (sol958) --------------------------------------------------------------->

<---- Dependent Variables 2 in Oxygen Pulse/Solution 958 (sol958) --------------
Started at May 28, 2023, 4:09:11 PM.
Initial values of variables solved for: Solution 958 (sol958), Solution Store 1 (sol
985).
Values of variables not solved for: Solution 958 (sol958), Solution Store 1 (sol985)
.
Solution time: 0 s.
Physical memory: 1.19 GB
```

Virtual memory: 1.16 GB
Ended at May 28, 2023, 4:09:11 PM.
----- Dependent Variables 2 in Oxygen Pulse/Solution 958 (sol958) ------------->

<---- Time-Dependent Solver 1 in Oxygen Pulse/Solution 958 (sol958) ------------
Started at May 28, 2023, 4:09:11 PM.
Time-dependent solver (BDF)
Number of degrees of freedom solved for: 18769 (plus 37608 internal DOFs).
Symmetric matrices found.
Scales for dependent variables:
Concentration (comp2.Oxi): 0.23

Step	Time	Stepsize	Res	Jac	Sol	Order	Tfail	NLfail	LinErr	LinRes
0	0	- out	5	3	5			0	8.8e-16	1.3e-15
1	2.659e-08	2.659e-08	7	4	7	1	0	0	3.8e-16	1.1e-15
2	5.3179e-08	2.659e-08	9	5	9	1	0	0	3.3e-16	1.1e-15
3	1.0636e-07	5.3179e-08	11	6	11	2	0	0	4.1e-16	1.3e-15
4	2.1272e-07	1.0636e-07	13	7	13	2	0	0	5.2e-16	1.8e-15
5	4.2543e-07	2.1272e-07	15	8	15	2	0	0	4.8e-16	3.2e-15
6	6.3815e-07	2.1272e-07	17	9	17	2	0	0	5.3e-16	3.8e-15
7	8.5087e-07	2.1272e-07	19	10	19	2	0	0	5.9e-16	3.9e-15
8	1.0636e-06	2.1272e-07	21	11	21	2	0	0	7.3e-16	3.9e-15
9	1.489e-06	4.2543e-07	23	12	23	2	0	0	8.7e-16	6.4e-15
10	1.9145e-06	4.2543e-07	25	13	25	2	0	0	9.1e-16	6.2e-15
11	2.3399e-06	4.2543e-07	27	14	27	2	0	0	9.8e-16	6.3e-15
12	2.7653e-06	4.2543e-07	29	15	29	2	0	0	9.6e-16	5.7e-15
13	3.6162e-06	8.5087e-07	31	16	31	2	0	0	1.1e-15	8.6e-15
14	4.4671e-06	8.5087e-07	33	17	33	2	0	0	1e-15	7.3e-15
15	5.3179e-06	8.5087e-07	35	18	35	2	0	0	1.3e-15	7.3e-15
16	6.1688e-06	8.5087e-07	37	19	37	2	0	0	7.9e-16	6.5e-15
17	7.8705e-06	1.7017e-06	39	20	39	2	0	0	1.4e-15	9.3e-15
18	9.5723e-06	1.7017e-06	41	21	41	2	0	0	1.3e-15	8.3e-15
19	1.1274e-05	1.7017e-06	43	22	43	2	0	0	1.2e-15	8.3e-15
20	1.2976e-05	1.7017e-06	45	23	45	2	0	0	1.6e-15	7.3e-15
21	1.4677e-05	1.7017e-06	47	24	47	2	0	0	1.8e-15	6.6e-15
22	1.8081e-05	3.4035e-06	49	25	49	2	0	0	2e-15	9.8e-15
23	2.1484e-05	3.4035e-06	51	26	51	2	0	0	1.6e-15	8.3e-15
24	2.4888e-05	3.4035e-06	53	27	53	2	0	0	1.1e-15	8.3e-15
25	2.8291e-05	3.4035e-06	55	28	55	2	0	0	1.6e-15	7.4e-15
26	3.5098e-05	6.807e-06	57	29	57	2	0	0	1.9e-15	1.1e-14
27	4.1905e-05	6.807e-06	59	30	59	2	0	0	1.8e-15	9.5e-15
28	4.8712e-05	6.807e-06	61	31	61	2	0	0	1.1e-15	9.7e-15
29	5.5519e-05	6.807e-06	63	32	63	2	0	0	1.6e-15	8.9e-15
30	6.2326e-05	6.807e-06	65	33	65	2	0	0	2.1e-15	7.8e-15
31	7.594e-05	1.3614e-05	67	34	67	2	0	0	1.5e-15	1.2e-14
32	8.9554e-05	1.3614e-05	69	35	69	2	0	0	2.3e-15	1e-14
33	0.00010317	1.3614e-05	71	36	71	2	0	0	2.7e-15	1e-14
34	0.00011678	1.3614e-05	73	37	73	2	0	0	2.1e-15	9.6e-15
35	0.00014401	2.7228e-05	75	38	75	2	0	0	1.8e-15	1.4e-14
36	0.00017124	2.7228e-05	77	39	77	2	0	0	2.1e-15	1.2e-14
37	0.00019847	2.7228e-05	79	40	79	2	0	0	2.1e-15	1.2e-14
38	0.00022569	2.7228e-05	81	41	81	2	0	0	1.5e-15	1.1e-14
39	0.00028015	5.4456e-05	83	42	83	2	0	0	1.8e-15	1.6e-14
40	0.0003346	5.4456e-05	85	43	85	2	0	0	1.8e-15	1.3e-14
41	0.00038906	5.4456e-05	87	44	87	2	0	0	2.1e-15	1.4e-14
42	0.00049797	0.00010891	89	45	89	2	0	0	2.7e-15	2e-14
43	0.00060688	0.00010891	91	46	91	2	0	0	4e-15	1.7e-14

44	0.00071579	0.00010891	93	47	93	2	0	0	2.9e-15	1.6e-14
45	0.0008247	0.00010891	95	48	95	2	0	0	1.5e-15	1.5e-14
46	0.0010425	0.00021782	97	49	97	2	0	0	2.4e-15	2e-14
47	0.0012603	0.00021782	99	50	99	2	0	0	4.9e-15	1.7e-14
48	0.001696	0.00043564	101	51	101	2	0	0	3.5e-15	2.7e-14
49	0.0021316	0.00043564	103	52	103	2	0	0	3.4e-15	2.2e-14
50	0.0025673	0.00043564	105	53	105	2	0	0	1.8e-15	2.3e-14
51	0.0030029	0.00043564	107	54	107	2	0	0	3e-15	1.9e-14
52	0.0038742	0.00087129	109	55	109	2	0	0	3.1e-15	2.4e-14
53	0.0047455	0.00087129	111	56	111	2	0	0	2e-15	1.8e-14
-	0.005	-	out							
54	0.0064881	0.0017426	113	57	113	2	0	0	3.2e-15	3e-14
55	0.0082307	0.0017426	115	58	115	2	0	0	4.6e-15	2.6e-14
-	0.01	-	out							
56	0.011716	0.0034852	117	59	117	2	0	0	3.2e-15	4.1e-14
-	0.015	-	out							
57	0.015201	0.0034852	119	60	119	1	0	0	6.1e-15	4.3e-14
58	0.018686	0.0034852	121	61	121	1	0	0	2.5e-15	2.5e-14
-	0.02	-	out							
-	0.025	-	out							
59	0.025656	0.0069703	123	62	123	1	0	0	5.3e-15	4e-14
-	0.03	-	out							
-	0.035	-	out							
60	0.039597	0.013941	125	63	125	1	0	0	8.2e-15	6.2e-14
-	0.04	-	out							
-	0.045	-	out							
-	0.05	-	out							
61	0.053538	0.013941	127	64	127	1	0	0	9.7e-15	5.9e-14
-	0.055	-	out							
-	0.06	-	out							
-	0.065	-	out							
-	0.07	-	out							
-	0.075	-	out							
-	0.08	-	out							
62	0.081419	0.027881	129	65	129	1	0	0	4.6e-15	8.7e-14
-	0.085	-	out							
-	0.09	-	out							
-	0.095	-	out							
-	0.1	-	out							
-	0.105	-	out							
63	0.1093	0.027881	131	66	131	1	0	0	3.1e-15	7.7e-14
-	0.11	-	out							
-	0.115	-	out							
-	0.12	-	out							
-	0.125	-	out							
-	0.13	-	out							
-	0.135	-	out							
-	0.14	-	out							
-	0.145	-	out							
-	0.15	-	out							
-	0.155	-	out							
-	0.16	-	out							
-	0.165	-	out							
64	0.16506	0.055763	133	67	133	1	0	0	6.4e-15	9.6e-14
-	0.17	-	out							
-	0.175	-	out							

-	0.18	-	out							
-	0.185	-	out							
-	0.19	-	out							
-	0.195	-	out							
-	0.2	-	out							
-	0.205	-	out							
-	0.21	-	out							
-	0.215	-	out							
-	0.22	-	out							
65	0.22083	0.055763		135	68	135	1	0	0	3.5e-15	7.7e-14
-	0.225	-	out							
-	0.23	-	out							
-	0.235	-	out							
-	0.24	-	out							
-	0.245	-	out							
-	0.25	-	out							
-	0.25	-	out							
-	0.27	-	out							
-	0.29	-	out							
-	0.31	-	out							
66	0.31983	0.099		137	69	137	1	0	0	5.9e-15	8e-14
-	0.33	-	out							
-	0.35	-	out							
-	0.37	-	out							
-	0.39	-	out							
-	0.41	-	out							
67	0.41883	0.099		139	70	139	1	0	0	5.4e-15	6.2e-14
-	0.43	-	out							
-	0.45	-	out							
-	0.47	-	out							
-	0.49	-	out							
-	0.51	-	out							
68	0.51783	0.099		141	71	141	1	0	0	1e-14	4.8e-14
-	0.53	-	out							
-	0.55	-	out							
-	0.57	-	out							
-	0.59	-	out							
-	0.61	-	out							
69	0.61683	0.099		143	72	143	1	0	0	9.3e-15	3.8e-14
-	0.63	-	out							
-	0.65	-	out							
-	0.67	-	out							
-	0.69	-	out							
-	0.71	-	out							
70	0.71583	0.099		144	73	144	1	0	0	7.1e-15	3e-14
-	0.73	-	out							
-	0.75	-	out							
-	0.77	-	out							
-	0.79	-	out							
-	0.81	-	out							
71	0.81483	0.099		145	74	145	1	0	0	1.3e-14	2.6e-14
-	0.83	-	out							
-	0.85	-	out							
-	0.87	-	out							
-	0.89	-	out							
-	0.91	-	out							

```
  72      0.91383       0.099      146   75  146     2     0      0  3.3e-15  2.2e-14
   -         0.93           - out
   -         0.95           - out
   -         0.97           - out
   -         0.99           - out
  73       1.0128       0.099      147   76  147     2     0      0  6.6e-15  1.5e-14
Time-stepping completed.
Solution time: 13 s.
Physical memory: 1.22 GB
Virtual memory: 1.21 GB
Ended at May 28, 2023, 4:09:24 PM.
----- Time-Dependent Solver 1 in Oxygen Pulse/Solution 958 (sol958) ----------->
```

k0Sub=1.51E-9, d=1.5E-5 (su24)

GENERAL

Description	Value
Solution	k0Sub=1.51E-9, d=1.5E-5

LOG

```
<---- Compile Equations: Stationary in Oxygen Pulse/Solution 958 (sol958) ------
Started at May 28, 2023, 4:09:27 PM.
Parameter k0Sub = 1.51E-9 (m/s).
Parameter d = 1.4999999999999999E-5 (m).
Geometry shape function: Linear Lagrange
Number of vertex elements: 10
Minimum element quality: 0.4444
Time: 1 s.
Physical memory: 1.2 GB
Virtual memory: 1.18 GB
Ended at May 28, 2023, 4:09:28 PM.
----- Compile Equations: Stationary in Oxygen Pulse/Solution 958 (sol958) ----->

<---- Dependent Variables 1 in Oxygen Pulse/Solution 958 (sol958) --------------
Started at May 28, 2023, 4:09:28 PM.
Solution time: 0 s.
Physical memory: 1.2 GB
Virtual memory: 1.18 GB
Ended at May 28, 2023, 4:09:28 PM.
----- Dependent Variables 1 in Oxygen Pulse/Solution 958 (sol958) ------------->

<---- Stationary Solver 1 in Oxygen Pulse/Solution 958 (sol958) ----------------
Started at May 28, 2023, 4:09:28 PM.
Linear solver
Number of degrees of freedom solved for: 18918 (plus 952 internal DOFs).
Symmetric matrices found.
Scales for dependent variables:
Concentration (comp2.Oxi): 0.23
Orthonormal null-space function used.
Iter      SolEst     Damping    Stepsize #Res #Jac #Sol   LinErr   LinRes
   1     3.8e-07   1.0000000     3.8e-07    1    1    1    2e-13  4.6e-14
Solution time: 1 s.
Physical memory: 1.21 GB
Virtual memory: 1.19 GB
Ended at May 28, 2023, 4:09:28 PM.
----- Stationary Solver 1 in Oxygen Pulse/Solution 958 (sol958) --------------->

<---- Compile Equations: Oxygen Probe Pulse in Oxygen Pulse/Solution 958
      (sol958) ----------------------------------------------------------------
Started at May 28, 2023, 4:09:28 PM.
Geometry shape function: Linear Lagrange
Time: 1 s.
Physical memory: 1.21 GB
Virtual memory: 1.19 GB
Ended at May 28, 2023, 4:09:29 PM.
----- Compile Equations: Oxygen Probe Pulse in Oxygen Pulse/Solution 958
      (sol958) --------------------------------------------------------------->

<---- Dependent Variables 2 in Oxygen Pulse/Solution 958 (sol958) --------------
Started at May 28, 2023, 4:09:29 PM.
Initial values of variables solved for: Solution 958 (sol958), Solution Store 1 (sol
985).
Values of variables not solved for: Solution 958 (sol958), Solution Store 1 (sol985)
.
Solution time: 0 s.
Physical memory: 1.21 GB
```

Virtual memory: 1.19 GB
Ended at May 28, 2023, 4:09:29 PM.
----- Dependent Variables 2 in Oxygen Pulse/Solution 958 (sol958) ------------->

<---- Time-Dependent Solver 1 in Oxygen Pulse/Solution 958 (sol958) ------------
Started at May 28, 2023, 4:09:29 PM.
Time-dependent solver (BDF)
Number of degrees of freedom solved for: 18918 (plus 37906 internal DOFs).
Symmetric matrices found.
Scales for dependent variables:
Concentration (comp2.Oxi): 0.23

Step	Time	Stepsize	Res	Jac	Sol	Order	Tfail	NLfail	LinErr	LinRes
0	0	- out	5	3	5			0	7.9e-16	1.3e-15
1	2.6779e-08	2.6779e-08	7	4	7	1	0	0	3.6e-16	1.1e-15
2	5.3558e-08	2.6779e-08	9	5	9	1	0	0	3.6e-16	1.2e-15
3	1.0712e-07	5.3558e-08	11	6	11	2	0	0	3.9e-16	1.3e-15
4	2.1423e-07	1.0712e-07	13	7	13	2	0	0	4.4e-16	1.8e-15
5	4.2847e-07	2.1423e-07	15	8	15	2	0	0	6.5e-16	3.4e-15
6	6.427e-07	2.1423e-07	17	9	17	2	0	0	5.1e-16	3.7e-15
7	8.5693e-07	2.1423e-07	19	10	19	2	0	0	6.8e-16	3.8e-15
8	1.0712e-06	2.1423e-07	21	11	21	2	0	0	5.7e-16	4.1e-15
9	1.4996e-06	4.2847e-07	23	12	23	2	0	0	9.1e-16	6.3e-15
10	1.9281e-06	4.2847e-07	25	13	25	2	0	0	7.1e-16	5.8e-15
11	2.3566e-06	4.2847e-07	27	14	27	2	0	0	8.4e-16	6.1e-15
12	2.785e-06	4.2847e-07	29	15	29	2	0	0	7.9e-16	5.6e-15
13	3.642e-06	8.5693e-07	31	16	31	2	0	0	1.3e-15	8.6e-15
14	4.4989e-06	8.5693e-07	33	17	33	2	0	0	8.8e-16	7.3e-15
15	5.3558e-06	8.5693e-07	35	18	35	2	0	0	1.2e-15	7.2e-15
16	6.2128e-06	8.5693e-07	37	19	37	2	0	0	1e-15	6.6e-15
17	7.9266e-06	1.7139e-06	39	20	39	2	0	0	1.2e-15	9.6e-15
18	9.6405e-06	1.7139e-06	41	21	41	2	0	0	1.4e-15	8.2e-15
19	1.1354e-05	1.7139e-06	43	22	43	2	0	0	1.2e-15	8.2e-15
20	1.3068e-05	1.7139e-06	45	23	45	2	0	0	1.1e-15	7.2e-15
21	1.4782e-05	1.7139e-06	47	24	47	2	0	0	1.5e-15	6.6e-15
22	1.821e-05	3.4277e-06	49	25	49	2	0	0	1.7e-15	9.8e-15
23	2.1638e-05	3.4277e-06	51	26	51	2	0	0	1.7e-15	8.4e-15
24	2.5065e-05	3.4277e-06	53	27	53	2	0	0	1.4e-15	8.6e-15
25	2.8493e-05	3.4277e-06	55	28	55	2	0	0	8.7e-16	7.6e-15
26	3.5349e-05	6.8555e-06	57	29	57	2	0	0	1.7e-15	1.2e-14
27	4.2204e-05	6.8555e-06	59	30	59	2	0	0	1.7e-15	9.8e-15
28	4.9059e-05	6.8555e-06	61	31	61	2	0	0	1.9e-15	9.9e-15
29	5.5915e-05	6.8555e-06	63	32	63	2	0	0	1.7e-15	9e-15
30	6.277e-05	6.8555e-06	65	33	65	2	0	0	1.7e-15	8e-15
31	7.6481e-05	1.3711e-05	67	34	67	2	0	0	1.8e-15	1.2e-14
32	9.0192e-05	1.3711e-05	69	35	69	2	0	0	2.3e-15	1e-14
33	0.0001039	1.3711e-05	71	36	71	2	0	0	1.9e-15	1e-14
34	0.00011761	1.3711e-05	73	37	73	2	0	0	1.6e-15	9.7e-15
35	0.00014504	2.7422e-05	75	38	75	2	0	0	1.9e-15	1.4e-14
36	0.00017246	2.7422e-05	77	39	77	2	0	0	2.9e-15	1.3e-14
37	0.00019988	2.7422e-05	79	40	79	2	0	0	1.7e-15	1.2e-14
38	0.0002273	2.7422e-05	81	41	81	2	0	0	1.9e-15	1.1e-14
39	0.00028215	5.4844e-05	83	42	83	2	0	0	3.3e-15	1.7e-14
40	0.00033699	5.4844e-05	85	43	85	2	0	0	2.1e-15	1.4e-14
41	0.00039183	5.4844e-05	87	44	87	2	0	0	1.8e-15	1.4e-14
42	0.00050152	0.00010969	89	45	89	2	0	0	4.2e-15	2e-14
43	0.00061121	0.00010969	91	46	91	2	0	0	3.4e-15	1.7e-14

44	0.0007209	0.00010969		93	47	93	2	0	0	1.7e-15	1.7e-14
45	0.00083058	0.00010969		95	48	95	2	0	0	2.3e-15	1.5e-14
46	0.00105	0.00021938		97	49	97	2	0	0	2.8e-15	2.1e-14
47	0.0012693	0.00021938		99	50	99	2	0	0	2.3e-15	1.6e-14
48	0.0017081	0.00043875		101	51	101	2	0	0	8.2e-15	2.8e-14
49	0.0021468	0.00043875		103	52	103	2	0	0	1.8e-15	2.4e-14
50	0.0025856	0.00043875		105	53	105	2	0	0	3.1e-15	2.3e-14
51	0.0030243	0.00043875		107	54	107	2	0	0	2.4e-15	1.9e-14
52	0.0039018	0.0008775		109	55	109	2	0	0	3.6e-15	2.4e-14
53	0.0047793	0.0008775		111	56	111	2	0	0	2.5e-15	1.8e-14
-	0.005	-	out								
54	0.0065343	0.001755		113	57	113	2	0	0	3.8e-15	2.9e-14
55	0.0082893	0.001755		115	58	115	2	0	0	3.1e-15	2.6e-14
-	0.01	-	out								
56	0.011799	0.00351		117	59	117	2	0	0	5.6e-15	3.8e-14
-	0.015	-	out								
57	0.015309	0.00351		119	60	119	1	0	0	3e-15	4.1e-14
58	0.018819	0.00351		121	61	121	1	0	0	7.9e-15	2.4e-14
-	0.02	-	out								
-	0.025	-	out								
59	0.025839	0.00702		123	62	123	1	0	0	3.3e-15	3.7e-14
-	0.03	-	out								
-	0.035	-	out								
60	0.039879	0.01404		125	63	125	1	0	0	2.8e-15	5e-14
-	0.04	-	out								
-	0.045	-	out								
-	0.05	-	out								
61	0.053919	0.01404		127	64	127	1	0	0	4.4e-15	5.1e-14
-	0.055	-	out								
-	0.06	-	out								
-	0.065	-	out								
-	0.07	-	out								
-	0.075	-	out								
-	0.08	-	out								
62	0.081999	0.02808		129	65	129	1	0	0	5.7e-15	7.1e-14
-	0.085	-	out								
-	0.09	-	out								
-	0.095	-	out								
-	0.1	-	out								
-	0.105	-	out								
-	0.11	-	out								
63	0.11008	0.02808		131	66	131	1	0	0	1.2e-14	6.7e-14
-	0.115	-	out								
-	0.12	-	out								
-	0.125	-	out								
-	0.13	-	out								
-	0.135	-	out								
-	0.14	-	out								
-	0.145	-	out								
-	0.15	-	out								
-	0.155	-	out								
-	0.16	-	out								
-	0.165	-	out								
64	0.16624	0.05616		133	67	133	1	0	0	1.1e-14	8.9e-14
-	0.17	-	out								
-	0.175	-	out								

-	0.18	- out								
-	0.185	- out								
-	0.19	- out								
-	0.195	- out								
-	0.2	- out								
-	0.205	- out								
-	0.21	- out								
-	0.215	- out								
-	0.22	- out								
65	0.2224	0.05616	135	68	135	1	0	0	1.2e-14	7.3e-14
-	0.225	- out								
-	0.23	- out								
-	0.235	- out								
-	0.24	- out								
-	0.245	- out								
-	0.25	- out								
-	0.25	- out								
-	0.27	- out								
-	0.29	- out								
-	0.31	- out								
66	0.3214	0.099	137	69	137	1	0	0	5.6e-15	7.6e-14
-	0.33	- out								
-	0.35	- out								
-	0.37	- out								
-	0.39	- out								
-	0.41	- out								
67	0.4204	0.099	139	70	139	1	0	0	5.3e-15	6.1e-14
-	0.43	- out								
-	0.45	- out								
-	0.47	- out								
-	0.49	- out								
-	0.51	- out								
68	0.5194	0.099	141	71	141	1	0	0	5.6e-15	4.8e-14
-	0.53	- out								
-	0.55	- out								
-	0.57	- out								
-	0.59	- out								
-	0.61	- out								
69	0.6184	0.099	143	72	143	1	0	0	5e-15	3.7e-14
-	0.63	- out								
-	0.65	- out								
-	0.67	- out								
-	0.69	- out								
-	0.71	- out								
70	0.7174	0.099	144	73	144	1	0	0	3.2e-15	3.2e-14
-	0.73	- out								
-	0.75	- out								
-	0.77	- out								
-	0.79	- out								
-	0.81	- out								
71	0.8164	0.099	145	74	145	1	0	0	7.1e-15	2.8e-14
-	0.83	- out								
-	0.85	- out								
-	0.87	- out								
-	0.89	- out								
-	0.91	- out								

```
  72      0.9154       0.099      146   75  146     2     0      0  6.7e-15  2.3e-14
   -        0.93           - out
   -        0.95           - out
   -        0.97           - out
   -        0.99           - out
  73      1.0144       0.099      147   76  147     2     0      0  6.4e-15  1.5e-14
Time-stepping completed.
Solution time: 14 s.
Physical memory: 1.24 GB
Virtual memory: 1.22 GB
Ended at May 28, 2023, 4:09:43 PM.
----- Time-Dependent Solver 1 in Oxygen Pulse/Solution 958 (sol958) ----------->
```

k0Sub=1.51E-8, d=5E-7 (su25)

GENERAL

Description	Value
Solution	k0Sub=1.51E-8, d=5E-7

LOG

```
<---- Compile Equations: Stationary in Oxygen Pulse/Solution 958 (sol958) ------
Started at May 28, 2023, 4:09:45 PM.
Parameter k0Sub = 1.51E-8 (m/s).
Parameter d = 5.0E-7 (m).
Geometry shape function: Linear Lagrange
Number of vertex elements: 10
Minimum element quality: 0.3539
Time: 1 s.
Physical memory: 1.16 GB
Virtual memory: 1.14 GB
Ended at May 28, 2023, 4:09:46 PM.
----- Compile Equations: Stationary in Oxygen Pulse/Solution 958 (sol958) ----->

<---- Dependent Variables 1 in Oxygen Pulse/Solution 958 (sol958) --------------
Started at May 28, 2023, 4:09:46 PM.
Solution time: 0 s.
Physical memory: 1.16 GB
Virtual memory: 1.14 GB
Ended at May 28, 2023, 4:09:46 PM.
----- Dependent Variables 1 in Oxygen Pulse/Solution 958 (sol958) ------------->

<---- Stationary Solver 1 in Oxygen Pulse/Solution 958 (sol958) ----------------
Started at May 28, 2023, 4:09:46 PM.
Linear solver
Number of degrees of freedom solved for: 17710 (plus 1007 internal DOFs).
Symmetric matrices found.
Scales for dependent variables:
Concentration (comp2.Oxi): 0.23
Orthonormal null-space function used.
Iter      SolEst     Damping    Stepsize #Res #Jac #Sol   LinErr   LinRes
   1     2.1e-05   1.0000000     2.1e-05    1    1    1  5.2e-13  1.5e-13
Solution time: 1 s.
Physical memory: 1.18 GB
Virtual memory: 1.16 GB
Ended at May 28, 2023, 4:09:46 PM.
----- Stationary Solver 1 in Oxygen Pulse/Solution 958 (sol958) --------------->

<---- Compile Equations: Oxygen Probe Pulse in Oxygen Pulse/Solution 958
      (sol958) ----------------------------------------------------------------
Started at May 28, 2023, 4:09:46 PM.
Geometry shape function: Linear Lagrange
Time: 0 s.
Physical memory: 1.19 GB
Virtual memory: 1.17 GB
Ended at May 28, 2023, 4:09:47 PM.
----- Compile Equations: Oxygen Probe Pulse in Oxygen Pulse/Solution 958
      (sol958) --------------------------------------------------------------->

<---- Dependent Variables 2 in Oxygen Pulse/Solution 958 (sol958) --------------
Started at May 28, 2023, 4:09:47 PM.
Initial values of variables solved for: Solution 958 (sol958), Solution Store 1 (sol
985).
Values of variables not solved for: Solution 958 (sol958), Solution Store 1 (sol985)
.
Solution time: 0 s.
Physical memory: 1.19 GB
```

```
Virtual memory: 1.17 GB
Ended at May 28, 2023, 4:09:47 PM.
----- Dependent Variables 2 in Oxygen Pulse/Solution 958 (sol958) ------------->

<---- Time-Dependent Solver 1 in Oxygen Pulse/Solution 958 (sol958) ------------
Started at May 28, 2023, 4:09:47 PM.
Time-dependent solver (BDF)
Number of degrees of freedom solved for: 17710 (plus 35580 internal DOFs).
Symmetric matrices found.
Scales for dependent variables:
Concentration (comp2.Oxi): 0.23
Step        Time    Stepsize      Res  Jac   Sol Order Tfail NLfail   LinErr   LinRes
   0           0          - out     5    3     5                  0  8.4e-16  1.5e-15
   1  2.5604e-08  2.5604e-08        7    4     7     1     0      0  3.6e-16  1.1e-15
   2  5.1207e-08  2.5604e-08        9    5     9     1     0      0  3.4e-16  1.2e-15
   3  1.0241e-07  5.1207e-08       11    6    11     2     0      0  3.5e-16  1.3e-15
   4  2.0483e-07  1.0241e-07       13    7    13     2     0      0  4.8e-16  1.8e-15
   5  4.0966e-07  2.0483e-07       15    8    15     2     0      0  6.8e-16  3.3e-15
   6  6.1449e-07  2.0483e-07       17    9    17     2     0      0  5.9e-16  3.6e-15
   7  8.1932e-07  2.0483e-07       19   10    19     2     0      0  5.2e-16  3.9e-15
   8  1.0241e-06  2.0483e-07       21   11    21     2     0      0  6.6e-16  3.8e-15
   9  1.4338e-06  4.0966e-07       23   12    23     2     0      0  6.6e-16  5.8e-15
  10  1.8435e-06  4.0966e-07       25   13    25     2     0      0  8.3e-16  5.7e-15
  11  2.2531e-06  4.0966e-07       27   14    27     2     0      0  8.5e-16  5.5e-15
  12  2.6628e-06  4.0966e-07       29   15    29     2     0      0  8.6e-16  5.3e-15
  13  3.4821e-06  8.1932e-07       31   16    31     2     0      0    1e-15  7.8e-15
  14  4.3014e-06  8.1932e-07       33   17    33     2     0      0  1.2e-15  6.8e-15
  15  5.1207e-06  8.1932e-07       35   18    35     2     0      0  1.2e-15  6.4e-15
  16    5.94e-06  8.1932e-07       37   19    37     2     0      0    1e-15  5.9e-15
  17  7.5787e-06  1.6386e-06       39   20    39     2     0      0  1.2e-15  8.9e-15
  18  9.2173e-06  1.6386e-06       41   21    41     2     0      0  1.3e-15  7.4e-15
  19  1.0856e-05  1.6386e-06       43   22    43     2     0      0  1.1e-15  7.9e-15
  20  1.2495e-05  1.6386e-06       45   23    45     2     0      0  1.2e-15  7.1e-15
  21  1.4133e-05  1.6386e-06       47   24    47     2     0      0    1e-15  6.4e-15
  22   1.741e-05  3.2773e-06       49   25    49     2     0      0  1.4e-15  9.9e-15
  23  2.0688e-05  3.2773e-06       51   26    51     2     0      0  1.5e-15  8.4e-15
  24  2.3965e-05  3.2773e-06       53   27    53     2     0      0  1.5e-15  8.3e-15
  25  2.7242e-05  3.2773e-06       55   28    55     2     0      0  1.8e-15  7.8e-15
  26  3.3797e-05  6.5545e-06       57   29    57     2     0      0  2.9e-15  1.2e-14
  27  4.0351e-05  6.5545e-06       59   30    59     2     0      0  1.7e-15    1e-14
  28  4.6906e-05  6.5545e-06       61   31    61     2     0      0  1.6e-15    1e-14
  29   5.346e-05  6.5545e-06       63   32    63     2     0      0  1.1e-15  9.1e-15
  30  6.0015e-05  6.5545e-06       65   33    65     2     0      0  1.5e-15    8e-15
  31  7.3124e-05  1.3109e-05       67   34    67     2     0      0  1.8e-15  1.2e-14
  32  8.6233e-05  1.3109e-05       69   35    69     2     0      0    2e-15    1e-14
  33  9.9342e-05  1.3109e-05       71   36    71     2     0      0  1.9e-15  1.1e-14
  34  0.00011245  1.3109e-05       73   37    73     2     0      0  1.2e-15  1.1e-14
  35  0.00013867  2.6218e-05       75   38    75     2     0      0  2.1e-15  1.7e-14
  36  0.00016489  2.6218e-05       77   39    77     2     0      0  2.3e-15  1.3e-14
  37  0.00019111  2.6218e-05       79   40    79     2     0      0  1.9e-15  1.2e-14
  38  0.00021732  2.6218e-05       81   41    81     2     0      0  3.4e-15  1.2e-14
  39  0.00026976  5.2436e-05       83   42    83     2     0      0  1.8e-15  1.7e-14
  40   0.0003222  5.2436e-05       85   43    85     2     0      0  2.1e-15  1.4e-14
  41  0.00037463  5.2436e-05       87   44    87     2     0      0  3.5e-15  1.5e-14
  42  0.00047951  0.00010487       89   45    89     2     0      0  2.5e-15  2.1e-14
  43  0.00058438  0.00010487       91   46    91     2     0      0  3.6e-15  1.7e-14
```

44	0.00068925	0.00010487		93	47	93	2	0	0	3.3e-15	1.7e-14
45	0.000899	0.00020975		95	48	95	2	0	0	4.3e-15	2.4e-14
46	0.0011087	0.00020975		97	49	97	2	0	0	1.4e-15	2e-14
47	0.0013185	0.00020975		99	50	99	2	0	0	2.1e-15	2.1e-14
48	0.0015282	0.00020975		101	51	101	2	0	0	1.9e-15	2e-14
49	0.0019477	0.00041949		103	52	103	2	0	0	7e-15	3e-14
50	0.0023672	0.00041949		105	53	105	2	0	0	3.2e-15	2.6e-14
51	0.0027867	0.00041949		107	54	107	2	0	0	2.2e-15	2.6e-14
52	0.0036257	0.00083898		109	55	109	2	0	0	5.8e-15	3.9e-14
53	0.0044647	0.00083898		111	56	111	2	0	0	3.6e-15	3.4e-14
-	0.005	-	out								
54	0.0053036	0.00083898		113	57	113	2	0	0	1.5e-15	3.4e-14
55	0.0069816	0.001678		115	58	115	2	0	0	3.2e-15	4.9e-14
56	0.0086596	0.001678		117	59	117	2	0	0	3.1e-15	4e-14
-	0.01	-	out								
57	0.012015	0.0033559		119	60	119	2	0	0	3.9e-15	6.7e-14
-	0.015	-	out								
58	0.015371	0.0033559		121	61	121	2	0	0	5.4e-15	5e-14
59	0.018727	0.0033559		123	62	123	2	0	0	2.6e-15	5e-14
-	0.02	-	out								
60	0.022083	0.0033559		125	63	125	2	0	0	5.7e-15	4.3e-14
-	0.025	-	out								
61	0.028795	0.0067118		127	64	127	2	0	0	4.2e-15	6.2e-14
-	0.03	-	out								
-	0.035	-	out								
62	0.035507	0.0067118		129	65	129	2	0	0	3.5e-15	5.1e-14
-	0.04	-	out								
-	0.045	-	out								
63	0.048931	0.013424		131	66	131	2	0	0	1.5e-14	8.2e-14
-	0.05	-	out								
-	0.055	-	out								
-	0.06	-	out								
64	0.062354	0.013424		133	67	133	1	0	0	1.1e-14	7e-14
-	0.065	-	out								
-	0.07	-	out								
-	0.075	-	out								
65	0.075778	0.013424		135	68	135	1	0	0	3.9e-15	2.8e-14
-	0.08	-	out								
-	0.085	-	out								
-	0.09	-	out								
-	0.095	-	out								
-	0.1	-	out								
66	0.10263	0.026847		137	69	137	1	0	0	3.8e-15	3.9e-14
-	0.105	-	out								
-	0.11	-	out								
-	0.115	-	out								
-	0.12	-	out								
-	0.125	-	out								
-	0.13	-	out								
-	0.135	-	out								
-	0.14	-	out								
-	0.145	-	out								
-	0.15	-	out								
-	0.155	-	out								
67	0.15632	0.053695		139	70	139	1	0	0	4.7e-15	4.9e-14
-	0.16	-	out								

-	0.165	- out								
-	0.17	- out								
-	0.175	- out								
-	0.18	- out								
-	0.185	- out								
-	0.19	- out								
-	0.195	- out								
-	0.2	- out								
-	0.205	- out								
-	0.21	- out								
-	0.215	- out								
-	0.22	- out								
-	0.225	- out								
-	0.23	- out								
-	0.235	- out								
-	0.24	- out								
-	0.245	- out								
-	0.25	- out								
-	0.25	- out								
68	0.25532	0.099	141	71	141	1	0	0	1.4e-14	5.7e-14
-	0.27	- out								
-	0.29	- out								
-	0.31	- out								
-	0.33	- out								
-	0.35	- out								
69	0.35432	0.099	143	72	143	1	0	0	2.9e-14	4.6e-14
-	0.37	- out								
-	0.39	- out								
-	0.41	- out								
-	0.43	- out								
-	0.45	- out								
70	0.45332	0.099	145	73	145	1	0	0	1.2e-14	3.4e-14
-	0.47	- out								
-	0.49	- out								
-	0.51	- out								
-	0.53	- out								
-	0.55	- out								
71	0.55232	0.099	147	74	147	1	0	0	5.6e-15	2.7e-14
-	0.57	- out								
-	0.59	- out								
-	0.61	- out								
-	0.63	- out								
-	0.65	- out								
72	0.65132	0.099	148	75	148	1	0	0	3.8e-15	2.2e-14
-	0.67	- out								
-	0.69	- out								
-	0.71	- out								
-	0.73	- out								
-	0.75	- out								
73	0.75032	0.099	149	76	149	1	0	0	5.4e-15	2.1e-14
-	0.77	- out								
-	0.79	- out								
-	0.81	- out								
-	0.83	- out								
74	0.84932	0.099	150	77	150	2	0	0	5.8e-15	1.6e-14
-	0.85	- out								

```
   -        0.87           - out
   -        0.89           - out
   -        0.91           - out
   -        0.93           - out
  75     0.94832       0.099      151    78  151      2      0       0  5.9e-15    1e-14
   -        0.95           - out
   -        0.97           - out
   -        0.99           - out
  76      1.0473       0.099      152    79  152      2      0       0  8.5e-15  9.9e-15
Time-stepping completed.
Solution time: 13 s.
Physical memory: 1.23 GB
Virtual memory: 1.21 GB
Ended at May 28, 2023, 4:10:00 PM.
----- Time-Dependent Solver 1 in Oxygen Pulse/Solution 958 (sol958) ----------->
```

k0Sub=1.51E-8, d=1E-6 (su26)

GENERAL

Description	Value
Solution	k0Sub=1.51E-8, d=1E-6

LOG

```
<---- Compile Equations: Stationary in Oxygen Pulse/Solution 958 (sol958) ------
Started at May 28, 2023, 4:10:03 PM.
Parameter k0Sub = 1.51E-8 (m/s).
Parameter d = 1.0E-6 (m).
Geometry shape function: Linear Lagrange
Number of vertex elements: 10
Minimum element quality: 0.4361
Time: 1 s.
Physical memory: 1.19 GB
Virtual memory: 1.17 GB
Ended at May 28, 2023, 4:10:03 PM.
----- Compile Equations: Stationary in Oxygen Pulse/Solution 958 (sol958) ----->

<---- Dependent Variables 1 in Oxygen Pulse/Solution 958 (sol958) --------------
Started at May 28, 2023, 4:10:03 PM.
Solution time: 0 s.
Physical memory: 1.19 GB
Virtual memory: 1.17 GB
Ended at May 28, 2023, 4:10:03 PM.
----- Dependent Variables 1 in Oxygen Pulse/Solution 958 (sol958) ------------->

<---- Stationary Solver 1 in Oxygen Pulse/Solution 958 (sol958) ----------------
Started at May 28, 2023, 4:10:03 PM.
Linear solver
Number of degrees of freedom solved for: 17724 (plus 963 internal DOFs).
Symmetric matrices found.
Scales for dependent variables:
Concentration (comp2.Oxi): 0.23
Orthonormal null-space function used.
Iter      SolEst     Damping    Stepsize #Res #Jac #Sol   LinErr   LinRes
   1     1.8e-05   1.0000000     1.8e-05    1    1    1  4.7e-13  1.3e-13
Solution time: 1 s.
Physical memory: 1.2 GB
Virtual memory: 1.18 GB
Ended at May 28, 2023, 4:10:04 PM.
----- Stationary Solver 1 in Oxygen Pulse/Solution 958 (sol958) --------------->

<---- Compile Equations: Oxygen Probe Pulse in Oxygen Pulse/Solution 958
      (sol958) ----------------------------------------------------------------
Started at May 28, 2023, 4:10:04 PM.
Geometry shape function: Linear Lagrange
Time: 1 s.
Physical memory: 1.21 GB
Virtual memory: 1.19 GB
Ended at May 28, 2023, 4:10:04 PM.
----- Compile Equations: Oxygen Probe Pulse in Oxygen Pulse/Solution 958
      (sol958) --------------------------------------------------------------->

<---- Dependent Variables 2 in Oxygen Pulse/Solution 958 (sol958) --------------
Started at May 28, 2023, 4:10:04 PM.
Initial values of variables solved for: Solution 958 (sol958), Solution Store 1 (sol
985).
Values of variables not solved for: Solution 958 (sol958), Solution Store 1 (sol985)
.
Solution time: 0 s.
Physical memory: 1.21 GB
```

```
Virtual memory: 1.19 GB
Ended at May 28, 2023, 4:10:04 PM.
----- Dependent Variables 2 in Oxygen Pulse/Solution 958 (sol958) ------------->

<---- Time-Dependent Solver 1 in Oxygen Pulse/Solution 958 (sol958) ------------
Started at May 28, 2023, 4:10:04 PM.
Time-dependent solver (BDF)
Number of degrees of freedom solved for: 17724 (plus 35562 internal DOFs).
Symmetric matrices found.
Scales for dependent variables:
Concentration (comp2.Oxi): 0.23
Step       Time    Stepsize     Res   Jac   Sol Order Tfail NLfail   LinErr   LinRes
   0          0          - out    5     3     5                  0  8.2e-16  1.4e-15
   1 2.5902e-08  2.5902e-08       7     4     7     1     0      0  3.7e-16  1.1e-15
   2 5.1803e-08  2.5902e-08       9     5     9     1     0      0  3.6e-16  1.1e-15
   3 1.0361e-07  5.1803e-08      11     6    11     2     0      0  3.5e-16  1.2e-15
   4 2.0721e-07  1.0361e-07      13     7    13     2     0      0  4.8e-16  1.7e-15
   5 4.1442e-07  2.0721e-07      15     8    15     2     0      0  5.5e-16  3.2e-15
   6 6.2164e-07  2.0721e-07      17     9    17     2     0      0    7e-16  3.7e-15
   7 8.2885e-07  2.0721e-07      19    10    19     2     0      0  6.3e-16    4e-15
   8 1.0361e-06  2.0721e-07      21    11    21     2     0      0  6.6e-16  3.9e-15
   9 1.4505e-06  4.1442e-07      23    12    23     2     0      0  7.1e-16  6.4e-15
  10 1.8649e-06  4.1442e-07      25    13    25     2     0      0    8e-16  5.8e-15
  11 2.2793e-06  4.1442e-07      27    14    27     2     0      0  6.9e-16  5.7e-15
  12 2.6938e-06  4.1442e-07      29    15    29     2     0      0  8.1e-16  5.3e-15
  13 3.5226e-06  8.2885e-07      31    16    31     2     0      0  9.8e-16    8e-15
  14 4.3515e-06  8.2885e-07      33    17    33     2     0      0    1e-15    7e-15
  15 5.1803e-06  8.2885e-07      35    18    35     2     0      0  1.3e-15  6.8e-15
  16 6.0092e-06  8.2885e-07      37    19    37     2     0      0  1.1e-15  6.4e-15
  17 7.6669e-06  1.6577e-06      39    20    39     2     0      0  1.7e-15  9.4e-15
  18 9.3246e-06  1.6577e-06      41    21    41     2     0      0  1.1e-15  8.3e-15
  19 1.0982e-05  1.6577e-06      43    22    43     2     0      0  1.6e-15  7.8e-15
  20  1.264e-05  1.6577e-06      45    23    45     2     0      0  1.2e-15  7.2e-15
  21 1.4298e-05  1.6577e-06      47    24    47     2     0      0  1.5e-15  6.2e-15
  22 1.7613e-05  3.3154e-06      49    25    49     2     0      0    2e-15  9.1e-15
  23 2.0928e-05  3.3154e-06      51    26    51     2     0      0  1.2e-15    8e-15
  24 2.4244e-05  3.3154e-06      53    27    53     2     0      0  1.6e-15  7.6e-15
  25 2.7559e-05  3.3154e-06      55    28    55     2     0      0  1.1e-15  6.9e-15
  26  3.419e-05  6.6308e-06      57    29    57     2     0      0  1.8e-15  1.1e-14
  27 4.0821e-05  6.6308e-06      59    30    59     2     0      0  1.8e-15  9.1e-15
  28 4.7452e-05  6.6308e-06      61    31    61     2     0      0  2.2e-15  9.4e-15
  29 5.4082e-05  6.6308e-06      63    32    63     2     0      0  2.3e-15  8.6e-15
  30 6.0713e-05  6.6308e-06      65    33    65     2     0      0  1.9e-15  7.8e-15
  31 7.3975e-05  1.3262e-05      67    34    67     2     0      0  1.5e-15  1.2e-14
  32 8.7236e-05  1.3262e-05      69    35    69     2     0      0  1.6e-15    1e-14
  33  0.0001005  1.3262e-05      71    36    71     2     0      0  1.7e-15    1e-14
  34 0.00011376  1.3262e-05      73    37    73     2     0      0    2e-15  9.5e-15
  35 0.00014028  2.6523e-05      75    38    75     2     0      0  1.7e-15  1.4e-14
  36 0.00016681  2.6523e-05      77    39    77     2     0      0  2.8e-15  1.2e-14
  37 0.00019333  2.6523e-05      79    40    79     2     0      0  1.3e-15  1.2e-14
  38 0.00021985  2.6523e-05      81    41    81     2     0      0  3.9e-15  1.1e-14
  39  0.0002729  5.3046e-05      83    42    83     2     0      0  2.5e-15  1.6e-14
  40 0.00032595  5.3046e-05      85    43    85     2     0      0  2.7e-15  1.3e-14
  41 0.00037899  5.3046e-05      87    44    87     2     0      0    2e-15  1.3e-14
  42 0.00048508  0.00010609      89    45    89     2     0      0  1.9e-15  1.8e-14
  43 0.00059118  0.00010609      91    46    91     2     0      0  1.6e-15  1.6e-14
```

44	0.00069727	0.00010609	93	47	93	2	0	0	1.4e-15	1.6e-14
45	0.00080336	0.00010609	95	48	95	2	0	0	1.5e-15	1.4e-14
46	0.0010155	0.00021219	97	49	97	2	0	0	2.3e-15	2e-14
47	0.0012277	0.00021219	99	50	99	2	0	0	3.9e-15	1.6e-14
48	0.0016521	0.00042437	101	51	101	2	0	0	2.4e-15	2.7e-14
49	0.0020765	0.00042437	103	52	103	2	0	0	4.2e-15	2.6e-14
50	0.0025008	0.00042437	105	53	105	2	0	0	3.2e-15	2.6e-14
51	0.0029252	0.00042437	107	54	107	2	0	0	1.9e-15	2.3e-14
52	0.003774	0.00084874	109	55	109	2	0	0	3.5e-15	3.3e-14
53	0.0046227	0.00084874	111	56	111	2	0	0	2.9e-15	2.8e-14
-	0.005	-	out							
54	0.0063202	0.0016975	113	57	113	2	0	0	4.8e-15	4.5e-14
55	0.0080177	0.0016975	115	58	115	2	0	0	4.6e-15	4.3e-14
56	0.0097152	0.0016975	117	59	117	2	0	0	3.1e-15	4.4e-14
-	0.01	-	out							
57	0.011413	0.0016975	119	60	119	2	0	0	7.4e-15	3.9e-14
58	0.014808	0.003395	121	61	121	2	0	0	7.2e-15	4.8e-14
-	0.015	-	out							
59	0.018203	0.003395	123	62	123	2	0	0	1.6e-15	3.6e-14
-	0.02	-	out							
60	0.024993	0.0067899	125	63	125	2	0	0	5.6e-15	6.3e-14
-	0.025	-	out							
-	0.03	-	out							
61	0.031782	0.0067899	127	64	127	2	0	0	2.1e-15	5.6e-14
-	0.035	-	out							
62	0.038572	0.0067899	129	65	129	2	0	0	8.1e-15	5.9e-14
-	0.04	-	out							
-	0.045	-	out							
63	0.045362	0.0067899	131	66	131	2	0	0	6.6e-15	5.3e-14
-	0.05	-	out							
-	0.055	-	out							
64	0.058942	0.01358	133	67	133	2	0	0	5e-15	6.4e-14
-	0.06	-	out							
-	0.065	-	out							
-	0.07	-	out							
65	0.072522	0.01358	135	68	135	2	0	0	3.3e-15	4.2e-14
-	0.075	-	out							
-	0.08	-	out							
-	0.085	-	out							
-	0.09	-	out							
-	0.095	-	out							
66	0.099682	0.02716	137	69	137	2	0	0	4.8e-15	6.2e-14
-	0.1	-	out							
-	0.105	-	out							
-	0.11	-	out							
-	0.115	-	out							
-	0.12	-	out							
-	0.125	-	out							
-	0.13	-	out							
-	0.135	-	out							
-	0.14	-	out							
-	0.145	-	out							
-	0.15	-	out							
67	0.154	0.054319	139	70	139	1	0	0	6e-15	5.8e-14
-	0.155	-	out							
-	0.16	-	out							

-	0.165	-	out							
-	0.17	-	out							
-	0.175	-	out							
-	0.18	-	out							
-	0.185	-	out							
-	0.19	-	out							
-	0.195	-	out							
-	0.2	-	out							
-	0.205	-	out							
68	0.20832	0.054319	141	71	141	1	0	0	1.6e-15	3.1e-14
-	0.21	-	out							
-	0.215	-	out							
-	0.22	-	out							
-	0.225	-	out							
-	0.23	-	out							
-	0.235	-	out							
-	0.24	-	out							
-	0.245	-	out							
-	0.25	-	out							
-	0.25	-	out							
-	0.27	-	out							
-	0.29	-	out							
69	0.30732	0.099	143	72	143	1	0	0	5.6e-15	3.9e-14
-	0.31	-	out							
-	0.33	-	out							
-	0.35	-	out							
-	0.37	-	out							
-	0.39	-	out							
70	0.40632	0.099	145	73	145	1	0	0	1.2e-14	3.4e-14
-	0.41	-	out							
-	0.43	-	out							
-	0.45	-	out							
-	0.47	-	out							
-	0.49	-	out							
71	0.50532	0.099	147	74	147	1	0	0	8.1e-15	2.9e-14
-	0.51	-	out							
-	0.53	-	out							
-	0.55	-	out							
-	0.57	-	out							
-	0.59	-	out							
72	0.60432	0.099	149	75	149	1	0	0	2.5e-15	2.4e-14
-	0.61	-	out							
-	0.63	-	out							
-	0.65	-	out							
-	0.67	-	out							
-	0.69	-	out							
73	0.70332	0.099	150	76	150	1	0	0	5.8e-15	2e-14
-	0.71	-	out							
-	0.73	-	out							
-	0.75	-	out							
-	0.77	-	out							
-	0.79	-	out							
74	0.80232	0.099	151	77	151	1	0	0	1.5e-14	1.8e-14
-	0.81	-	out							
-	0.83	-	out							
-	0.85	-	out							

```
   -        0.87           - out
   -        0.89           - out
  75      0.90132       0.099      152    78   152      2      0       0  1.4e-14  1.5e-14
   -        0.91           - out
   -        0.93           - out
   -        0.95           - out
   -        0.97           - out
   -        0.99           - out
  76       1.0003       0.099      153    79   153      2      0       0  7.9e-15  8.8e-15
Time-stepping completed.
Solution time: 13 s.
Physical memory: 1.23 GB
Virtual memory: 1.22 GB
Ended at May 28, 2023, 4:10:17 PM.
----- Time-Dependent Solver 1 in Oxygen Pulse/Solution 958 (sol958) ----------->
```

k0Sub=1.51E-8, d=1.5E-6 (su27)

GENERAL

Description	Value
Solution	k0Sub=1.51E-8, d=1.5E-6

LOG

```
<---- Compile Equations: Stationary in Oxygen Pulse/Solution 958 (sol958) ------
Started at May 28, 2023, 4:10:20 PM.
Parameter k0Sub = 1.51E-8 (m/s).
Parameter d = 1.5E-6 (m).
Geometry shape function: Linear Lagrange
Number of vertex elements: 10
Minimum element quality: 0.395
Time: 1 s.
Physical memory: 1.2 GB
Virtual memory: 1.18 GB
Ended at May 28, 2023, 4:10:21 PM.
----- Compile Equations: Stationary in Oxygen Pulse/Solution 958 (sol958) ----->

<---- Dependent Variables 1 in Oxygen Pulse/Solution 958 (sol958) --------------
Started at May 28, 2023, 4:10:21 PM.
Solution time: 0 s.
Physical memory: 1.2 GB
Virtual memory: 1.18 GB
Ended at May 28, 2023, 4:10:21 PM.
----- Dependent Variables 1 in Oxygen Pulse/Solution 958 (sol958) ------------->

<---- Stationary Solver 1 in Oxygen Pulse/Solution 958 (sol958) ----------------
Started at May 28, 2023, 4:10:21 PM.
Linear solver
Number of degrees of freedom solved for: 17823 (plus 950 internal DOFs).
Symmetric matrices found.
Scales for dependent variables:
Concentration (comp2.Oxi): 0.23
Orthonormal null-space function used.
Iter      SolEst     Damping    Stepsize #Res #Jac #Sol   LinErr   LinRes
   1     1.5e-05   1.0000000     1.5e-05    1    1    1  4.1e-13  1.2e-13
Solution time: 1 s.
Physical memory: 1.21 GB
Virtual memory: 1.19 GB
Ended at May 28, 2023, 4:10:21 PM.
----- Stationary Solver 1 in Oxygen Pulse/Solution 958 (sol958) --------------->

<---- Compile Equations: Oxygen Probe Pulse in Oxygen Pulse/Solution 958
      (sol958) -----------------------------------------------------------------
Started at May 28, 2023, 4:10:21 PM.
Geometry shape function: Linear Lagrange
Time: 1 s.
Physical memory: 1.21 GB
Virtual memory: 1.19 GB
Ended at May 28, 2023, 4:10:22 PM.
----- Compile Equations: Oxygen Probe Pulse in Oxygen Pulse/Solution 958
      (sol958) ---------------------------------------------------------------->

<---- Dependent Variables 2 in Oxygen Pulse/Solution 958 (sol958) --------------
Started at May 28, 2023, 4:10:22 PM.
Initial values of variables solved for: Solution 958 (sol958), Solution Store 1 (sol
985).
Values of variables not solved for: Solution 958 (sol958), Solution Store 1 (sol985)
.
Solution time: 0 s.
Physical memory: 1.21 GB
```

Virtual memory: 1.19 GB
Ended at May 28, 2023, 4:10:22 PM.
----- Dependent Variables 2 in Oxygen Pulse/Solution 958 (sol958) ------------->

<---- Time-Dependent Solver 1 in Oxygen Pulse/Solution 958 (sol958) ------------
Started at May 28, 2023, 4:10:22 PM.
Time-dependent solver (BDF)
Number of degrees of freedom solved for: 17823 (plus 35744 internal DOFs).
Symmetric matrices found.
Scales for dependent variables:
Concentration (comp2.Oxi): 0.23

Step	Time	Stepsize		Res	Jac	Sol	Order	Tfail	NLfail	LinErr	LinRes
0	0	-	out	5	3	5			0	9.1e-16	1.4e-15
1	2.5748e-08	2.5748e-08		7	4	7	1	0	0	3.2e-16	9.9e-16
2	5.1497e-08	2.5748e-08		9	5	9	1	0	0	3.5e-16	1.2e-15
3	1.0299e-07	5.1497e-08		11	6	11	2	0	0	3.5e-16	1.3e-15
4	2.0599e-07	1.0299e-07		13	7	13	2	0	0	4.3e-16	1.8e-15
5	4.1197e-07	2.0599e-07		15	8	15	2	0	0	6.9e-16	3.4e-15
6	6.1796e-07	2.0599e-07		17	9	17	2	0	0	6.5e-16	3.7e-15
7	8.2395e-07	2.0599e-07		19	10	19	2	0	0	6.4e-16	3.8e-15
8	1.0299e-06	2.0599e-07		21	11	21	2	0	0	5.9e-16	4e-15
9	1.4419e-06	4.1197e-07		23	12	23	2	0	0	8e-16	6.3e-15
10	1.8539e-06	4.1197e-07		25	13	25	2	0	0	8.7e-16	6.1e-15
11	2.2659e-06	4.1197e-07		27	14	27	2	0	0	7.7e-16	5.9e-15
12	2.6778e-06	4.1197e-07		29	15	29	2	0	0	7.9e-16	5.7e-15
13	3.5018e-06	8.2395e-07		31	16	31	2	0	0	1.3e-15	8.3e-15
14	4.3257e-06	8.2395e-07		33	17	33	2	0	0	9.7e-16	7e-15
15	5.1497e-06	8.2395e-07		35	18	35	2	0	0	9.6e-16	6.7e-15
16	5.9736e-06	8.2395e-07		37	19	37	2	0	0	9.1e-16	6.2e-15
17	7.6215e-06	1.6479e-06		39	20	39	2	0	0	1.7e-15	9.8e-15
18	9.2694e-06	1.6479e-06		41	21	41	2	0	0	1.3e-15	8.1e-15
19	1.0917e-05	1.6479e-06		43	22	43	2	0	0	1.5e-15	8.1e-15
20	1.2565e-05	1.6479e-06		45	23	45	2	0	0	1.2e-15	7.4e-15
21	1.4213e-05	1.6479e-06		47	24	47	2	0	0	1.1e-15	6.4e-15
22	1.7509e-05	3.2958e-06		49	25	49	2	0	0	1.5e-15	9.4e-15
23	2.0805e-05	3.2958e-06		51	26	51	2	0	0	1.4e-15	8.3e-15
24	2.41e-05	3.2958e-06		53	27	53	2	0	0	1.5e-15	8.1e-15
25	2.7396e-05	3.2958e-06		55	28	55	2	0	0	1.2e-15	7.5e-15
26	3.3988e-05	6.5916e-06		57	29	57	2	0	0	1.7e-15	1e-14
27	4.0579e-05	6.5916e-06		59	30	59	2	0	0	1.4e-15	9.2e-15
28	4.7171e-05	6.5916e-06		61	31	61	2	0	0	1.5e-15	9.3e-15
29	5.3762e-05	6.5916e-06		63	32	63	2	0	0	1.8e-15	8.4e-15
30	6.0354e-05	6.5916e-06		65	33	65	2	0	0	1.4e-15	7.4e-15
31	7.3537e-05	1.3183e-05		67	34	67	2	0	0	1.4e-15	1.1e-14
32	8.672e-05	1.3183e-05		69	35	69	2	0	0	2.1e-15	9.6e-15
33	9.9903e-05	1.3183e-05		71	36	71	2	0	0	2.4e-15	9.4e-15
34	0.00011309	1.3183e-05		73	37	73	2	0	0	1.7e-15	9.1e-15
35	0.00013945	2.6366e-05		75	38	75	2	0	0	1.2e-15	1.3e-14
36	0.00016582	2.6366e-05		77	39	77	2	0	0	2e-15	1.1e-14
37	0.00019219	2.6366e-05		79	40	79	2	0	0	1.6e-15	1.2e-14
38	0.00021855	2.6366e-05		81	41	81	2	0	0	2.4e-15	1.1e-14
39	0.00027128	5.2733e-05		83	42	83	2	0	0	1.5e-15	1.6e-14
40	0.00032402	5.2733e-05		85	43	85	2	0	0	2.2e-15	1.3e-14
41	0.00037675	5.2733e-05		87	44	87	2	0	0	2.3e-15	1.3e-14
42	0.00048221	0.00010547		89	45	89	2	0	0	3.3e-15	1.9e-14
43	0.00058768	0.00010547		91	46	91	2	0	0	3.6e-15	1.6e-14

44	0.00069314	0.00010547	93	47	93	2	0	0	2.6e-15	1.5e-14
45	0.00079861	0.00010547	95	48	95	2	0	0	1.7e-15	1.4e-14
46	0.0010095	0.00021093	97	49	97	2	0	0	2.5e-15	2e-14
47	0.0012205	0.00021093	99	50	99	2	0	0	1.2e-15	1.7e-14
48	0.0016423	0.00042186	101	51	101	2	0	0	3.2e-15	2.9e-14
49	0.0020642	0.00042186	103	52	103	2	0	0	1.6e-15	2.5e-14
50	0.0024861	0.00042186	105	53	105	2	0	0	2.1e-15	2.4e-14
51	0.0033298	0.00084372	107	54	107	2	0	0	1.8e-15	3.2e-14
52	0.0041735	0.00084372	109	55	109	2	0	0	2.6e-15	2.6e-14
-	0.005	-	out							
53	0.0050172	0.00084372	111	56	111	2	0	0	4.8e-15	2.7e-14
54	0.0067047	0.0016874	113	57	113	2	0	0	2.8e-15	4.1e-14
55	0.0083921	0.0016874	115	58	115	2	0	0	2.1e-15	3.7e-14
-	0.01	-	out							
56	0.011767	0.0033749	117	59	117	2	0	0	1.1e-14	5.5e-14
-	0.015	-	out							
57	0.015142	0.0033749	119	60	119	2	0	0	7.2e-15	5.1e-14
58	0.018517	0.0033749	121	61	121	2	0	0	4.7e-15	4.9e-14
-	0.02	-	out							
59	0.021892	0.0033749	123	62	123	2	0	0	3.2e-15	4e-14
-	0.025	-	out							
60	0.028641	0.0067498	125	63	125	2	0	0	5.8e-15	5.6e-14
-	0.03	-	out							
-	0.035	-	out							
61	0.035391	0.0067498	127	64	127	2	0	0	5.4e-15	4.7e-14
-	0.04	-	out							
-	0.045	-	out							
62	0.048891	0.0135	129	65	129	2	0	0	6.6e-15	7.7e-14
-	0.05	-	out							
-	0.055	-	out							
-	0.06	-	out							
63	0.06239	0.0135	131	66	131	2	0	0	4.4e-15	6.4e-14
-	0.065	-	out							
-	0.07	-	out							
-	0.075	-	out							
64	0.07589	0.0135	133	67	133	2	0	0	4.3e-15	6.1e-14
-	0.08	-	out							
-	0.085	-	out							
-	0.09	-	out							
-	0.095	-	out							
-	0.1	-	out							
65	0.10289	0.026999	135	68	135	2	0	0	5.1e-15	6.7e-14
-	0.105	-	out							
-	0.11	-	out							
-	0.115	-	out							
-	0.12	-	out							
-	0.125	-	out							
-	0.13	-	out							
-	0.135	-	out							
-	0.14	-	out							
-	0.145	-	out							
-	0.15	-	out							
-	0.155	-	out							
66	0.15689	0.053998	137	69	137	1	0	0	1.1e-14	5.5e-14
-	0.16	-	out							
-	0.165	-	out							

-	0.17	-	out							
-	0.175	-	out							
-	0.18	-	out							
-	0.185	-	out							
-	0.19	-	out							
-	0.195	-	out							
-	0.2	-	out							
-	0.205	-	out							
-	0.21	-	out							
67	0.21088	0.053998	139	70	139	1	0	0	4.2e-15	3.1e-14
-	0.215	-	out							
-	0.22	-	out							
-	0.225	-	out							
-	0.23	-	out							
-	0.235	-	out							
-	0.24	-	out							
-	0.245	-	out							
-	0.25	-	out							
-	0.25	-	out							
-	0.27	-	out							
-	0.29	-	out							
68	0.30988	0.099	141	71	141	1	0	0	4.5e-15	4.1e-14
-	0.31	-	out							
-	0.33	-	out							
-	0.35	-	out							
-	0.37	-	out							
-	0.39	-	out							
69	0.40888	0.099	143	72	143	1	0	0	5.3e-15	3.4e-14
-	0.41	-	out							
-	0.43	-	out							
-	0.45	-	out							
-	0.47	-	out							
-	0.49	-	out							
70	0.50788	0.099	145	73	145	1	0	0	3.7e-15	3e-14
-	0.51	-	out							
-	0.53	-	out							
-	0.55	-	out							
-	0.57	-	out							
-	0.59	-	out							
71	0.60688	0.099	147	74	147	1	0	0	8.2e-15	2.4e-14
-	0.61	-	out							
-	0.63	-	out							
-	0.65	-	out							
-	0.67	-	out							
-	0.69	-	out							
72	0.70588	0.099	148	75	148	1	0	0	1.7e-14	2.1e-14
-	0.71	-	out							
-	0.73	-	out							
-	0.75	-	out							
-	0.77	-	out							
-	0.79	-	out							
73	0.80488	0.099	149	76	149	1	0	0	6.4e-15	1.8e-14
-	0.81	-	out							
-	0.83	-	out							
-	0.85	-	out							
-	0.87	-	out							

```
    -        0.89           - out
   74      0.90388        0.099      150    77   150      2      0       0     2e-14  1.5e-14
    -        0.91           - out
    -        0.93           - out
    -        0.95           - out
    -        0.97           - out
    -        0.99           - out
   75       1.0029        0.099      151    78   151      2      0       0  1.3e-14  9.7e-15
Time-stepping completed.
Solution time: 13 s.
Physical memory: 1.23 GB
Virtual memory: 1.21 GB
Ended at May 28, 2023, 4:10:34 PM.
----- Time-Dependent Solver 1 in Oxygen Pulse/Solution 958 (sol958) ----------->
```

k0Sub=1.51E-8, d=2E-6 (su28)

GENERAL

Description	Value
Solution	k0Sub=1.51E-8, d=2E-6

LOG

```
<---- Compile Equations: Stationary in Oxygen Pulse/Solution 958 (sol958) ------
Started at May 28, 2023, 4:10:37 PM.
Parameter k0Sub = 1.51E-8 (m/s).
Parameter d = 2.0E-6 (m).
Geometry shape function: Linear Lagrange
Number of vertex elements: 10
Minimum element quality: 0.4112
Time: 0 s.
Physical memory: 1.2 GB
Virtual memory: 1.18 GB
Ended at May 28, 2023, 4:10:38 PM.
----- Compile Equations: Stationary in Oxygen Pulse/Solution 958 (sol958) ----->

<---- Dependent Variables 1 in Oxygen Pulse/Solution 958 (sol958) --------------
Started at May 28, 2023, 4:10:38 PM.
Solution time: 0 s.
Physical memory: 1.2 GB
Virtual memory: 1.18 GB
Ended at May 28, 2023, 4:10:38 PM.
----- Dependent Variables 1 in Oxygen Pulse/Solution 958 (sol958) ------------->

<---- Stationary Solver 1 in Oxygen Pulse/Solution 958 (sol958) ----------------
Started at May 28, 2023, 4:10:38 PM.
Linear solver
Number of degrees of freedom solved for: 17907 (plus 938 internal DOFs).
Symmetric matrices found.
Scales for dependent variables:
Concentration (comp2.Oxi): 0.23
Orthonormal null-space function used.
Iter      SolEst     Damping    Stepsize #Res #Jac #Sol   LinErr   LinRes
   1     1.4e-05   1.0000000     1.4e-05    1    1    1  4.2e-13    1e-13
Solution time: 1 s.
Physical memory: 1.21 GB
Virtual memory: 1.19 GB
Ended at May 28, 2023, 4:10:38 PM.
----- Stationary Solver 1 in Oxygen Pulse/Solution 958 (sol958) --------------->

<---- Compile Equations: Oxygen Probe Pulse in Oxygen Pulse/Solution 958
      (sol958) -----------------------------------------------------------------
Started at May 28, 2023, 4:10:38 PM.
Geometry shape function: Linear Lagrange
Time: 0 s.
Physical memory: 1.21 GB
Virtual memory: 1.19 GB
Ended at May 28, 2023, 4:10:39 PM.
----- Compile Equations: Oxygen Probe Pulse in Oxygen Pulse/Solution 958
      (sol958) ---------------------------------------------------------------->

<---- Dependent Variables 2 in Oxygen Pulse/Solution 958 (sol958) --------------
Started at May 28, 2023, 4:10:39 PM.
Initial values of variables solved for: Solution 958 (sol958), Solution Store 1 (sol
985).
Values of variables not solved for: Solution 958 (sol958), Solution Store 1 (sol985)
.
Solution time: 0 s.
Physical memory: 1.21 GB
```

```
Virtual memory: 1.19 GB
Ended at May 28, 2023, 4:10:39 PM.
----- Dependent Variables 2 in Oxygen Pulse/Solution 958 (sol958) ------------->

<---- Time-Dependent Solver 1 in Oxygen Pulse/Solution 958 (sol958) ------------
Started at May 28, 2023, 4:10:39 PM.
Time-dependent solver (BDF)
Number of degrees of freedom solved for: 17907 (plus 35898 internal DOFs).
Symmetric matrices found.
Scales for dependent variables:
Concentration (comp2.Oxi): 0.23
Step        Time    Stepsize      Res   Jac   Sol Order Tfail NLfail   LinErr   LinRes
   0           0           - out    5     3     5                  0    9e-16  1.4e-15
   1  2.5768e-08  2.5768e-08        7     4     7     1     0      0  3.2e-16    1e-15
   2  5.1537e-08  2.5768e-08        9     5     9     1     0      0  3.4e-16  1.2e-15
   3  1.0307e-07  5.1537e-08       11     6    11     2     0      0  3.6e-16  1.2e-15
   4  2.0615e-07  1.0307e-07       13     7    13     2     0      0  4.6e-16  1.7e-15
   5   4.123e-07  2.0615e-07       15     8    15     2     0      0  6.4e-16  3.3e-15
   6  6.1844e-07  2.0615e-07       17     9    17     2     0      0  6.7e-16  3.7e-15
   7  8.2459e-07  2.0615e-07       19    10    19     2     0      0  5.9e-16  3.9e-15
   8  1.0307e-06  2.0615e-07       21    11    21     2     0      0  6.9e-16    4e-15
   9   1.443e-06   4.123e-07       23    12    23     2     0      0  8.9e-16  6.4e-15
  10  1.8553e-06   4.123e-07       25    13    25     2     0      0  1.1e-15  6.3e-15
  11  2.2676e-06   4.123e-07       27    14    27     2     0      0  9.6e-16  5.8e-15
  12  2.6799e-06   4.123e-07       29    15    29     2     0      0  8.9e-16  5.8e-15
  13  3.5045e-06  8.2459e-07       31    16    31     2     0      0  1.2e-15  8.2e-15
  14  4.3291e-06  8.2459e-07       33    17    33     2     0      0  8.7e-16  7.3e-15
  15  5.1537e-06  8.2459e-07       35    18    35     2     0      0  1.1e-15  7.5e-15
  16  5.9783e-06  8.2459e-07       37    19    37     2     0      0  8.9e-16  6.7e-15
  17  7.6275e-06  1.6492e-06       39    20    39     2     0      0  1.3e-15  9.7e-15
  18  9.2766e-06  1.6492e-06       41    21    41     2     0      0  1.2e-15  8.4e-15
  19  1.0926e-05  1.6492e-06       43    22    43     2     0      0  1.8e-15  8.6e-15
  20  1.2575e-05  1.6492e-06       45    23    45     2     0      0  1.2e-15  7.2e-15
  21  1.4224e-05  1.6492e-06       47    24    47     2     0      0  1.4e-15  6.6e-15
  22  1.7523e-05  3.2984e-06       49    25    49     2     0      0  2.2e-15  9.6e-15
  23  2.0821e-05  3.2984e-06       51    26    51     2     0      0  1.8e-15  8.5e-15
  24  2.4119e-05  3.2984e-06       53    27    53     2     0      0  1.4e-15  8.4e-15
  25  2.7418e-05  3.2984e-06       55    28    55     2     0      0  1.9e-15  7.6e-15
  26  3.4014e-05  6.5967e-06       57    29    57     2     0      0  2.1e-15  1.1e-14
  27  4.0611e-05  6.5967e-06       59    30    59     2     0      0  1.4e-15  9.6e-15
  28  4.7208e-05  6.5967e-06       61    31    61     2     0      0    2e-15  9.4e-15
  29  5.3805e-05  6.5967e-06       63    32    63     2     0      0  2.4e-15  8.6e-15
  30  6.0401e-05  6.5967e-06       65    33    65     2     0      0  1.8e-15  7.6e-15
  31  7.3595e-05  1.3193e-05       67    34    67     2     0      0    2e-15  1.1e-14
  32  8.6788e-05  1.3193e-05       69    35    69     2     0      0  1.7e-15    1e-14
  33  9.9982e-05  1.3193e-05       71    36    71     2     0      0  1.7e-15  9.8e-15
  34  0.00011317  1.3193e-05       73    37    73     2     0      0  1.7e-15  9.1e-15
  35  0.00013956  2.6387e-05       75    38    75     2     0      0  2.3e-15  1.3e-14
  36  0.00016595  2.6387e-05       77    39    77     2     0      0  2.5e-15  1.1e-14
  37  0.00019234  2.6387e-05       79    40    79     2     0      0  1.7e-15  1.2e-14
  38  0.00021872  2.6387e-05       81    41    81     2     0      0  2.1e-15    1e-14
  39   0.0002715  5.2774e-05       83    42    83     2     0      0  2.3e-15  1.5e-14
  40  0.00032427  5.2774e-05       85    43    85     2     0      0  2.3e-15  1.2e-14
  41  0.00037704  5.2774e-05       87    44    87     2     0      0  4.1e-15  1.2e-14
  42  0.00048259  0.00010555       89    45    89     2     0      0  2.7e-15  1.9e-14
  43  0.00058814  0.00010555       91    46    91     2     0      0    2e-15  1.5e-14
```

44	0.00069369	0.00010555	93	47	93	2	0	0	2.5e-15	1.5e-14
45	0.00079923	0.00010555	95	48	95	2	0	0	2.8e-15	1.3e-14
46	0.0010103	0.0002111	97	49	97	2	0	0	2.9e-15	1.8e-14
47	0.0012214	0.0002111	99	50	99	2	0	0	2.3e-15	1.5e-14
48	0.0016436	0.00042219	101	51	101	2	0	0	3.9e-15	2.5e-14
49	0.0020658	0.00042219	103	52	103	2	0	0	6.3e-15	2.3e-14
50	0.002488	0.00042219	105	53	105	2	0	0	4.4e-15	2.4e-14
51	0.0033324	0.00084438	107	54	107	2	0	0	2.6e-15	3e-14
52	0.0041768	0.00084438	109	55	109	2	0	0	6.5e-15	2.1e-14
-	0.005	- out								
53	0.0058655	0.0016888	111	56	111	2	0	0	1.7e-15	3.5e-14
54	0.0075543	0.0016888	113	57	113	2	0	0	2.8e-15	3.5e-14
55	0.009243	0.0016888	115	58	115	2	0	0	5.6e-15	3.6e-14
-	0.01	- out								
56	0.010932	0.0016888	117	59	117	2	0	0	3.4e-15	3.4e-14
57	0.014309	0.0033775	119	60	119	2	0	0	7.7e-15	4.4e-14
-	0.015	- out								
58	0.017687	0.0033775	121	61	121	2	0	0	3.1e-15	3.4e-14
-	0.02	- out								
59	0.024442	0.006755	123	62	123	2	0	0	3.5e-15	5.7e-14
-	0.025	- out								
-	0.03	- out								
60	0.031197	0.006755	125	63	125	2	0	0	6e-15	5e-14
-	0.035	- out								
61	0.037952	0.006755	127	64	127	2	0	0	3.8e-15	5.3e-14
-	0.04	- out								
-	0.045	- out								
-	0.05	- out								
62	0.051462	0.01351	129	65	129	2	0	0	5.5e-15	7e-14
-	0.055	- out								
-	0.06	- out								
63	0.064972	0.01351	131	66	131	2	0	0	6.6e-15	5.4e-14
-	0.065	- out								
-	0.07	- out								
-	0.075	- out								
-	0.08	- out								
-	0.085	- out								
-	0.09	- out								
64	0.091992	0.02702	133	67	133	2	0	0	1.1e-14	8e-14
-	0.095	- out								
-	0.1	- out								
-	0.105	- out								
-	0.11	- out								
-	0.115	- out								
65	0.11901	0.02702	135	68	135	1	0	0	4e-15	5.7e-14
-	0.12	- out								
-	0.125	- out								
-	0.13	- out								
-	0.135	- out								
-	0.14	- out								
-	0.145	- out								
-	0.15	- out								
-	0.155	- out								
-	0.16	- out								
-	0.165	- out								
-	0.17	- out								

66	0.17305	0.05404		137	69	137	1	0	0	1e-14	3e-14
-	0.175	-	out								
-	0.18	-	out								
-	0.185	-	out								
-	0.19	-	out								
-	0.195	-	out								
-	0.2	-	out								
-	0.205	-	out								
-	0.21	-	out								
-	0.215	-	out								
-	0.22	-	out								
-	0.225	-	out								
-	0.23	-	out								
-	0.235	-	out								
-	0.24	-	out								
-	0.245	-	out								
-	0.25	-	out								
-	0.25	-	out								
-	0.27	-	out								
67	0.27205	0.099		139	70	139	1	0	0	2.5e-15	4.5e-14
-	0.29	-	out								
-	0.31	-	out								
-	0.33	-	out								
-	0.35	-	out								
-	0.37	-	out								
68	0.37105	0.099		141	71	141	1	0	0	7.8e-15	3.9e-14
-	0.39	-	out								
-	0.41	-	out								
-	0.43	-	out								
-	0.45	-	out								
-	0.47	-	out								
69	0.47005	0.099		143	72	143	1	0	0	6e-15	3.4e-14
-	0.49	-	out								
-	0.51	-	out								
-	0.53	-	out								
-	0.55	-	out								
70	0.56905	0.099		145	73	145	1	0	0	4e-15	2.8e-14
-	0.57	-	out								
-	0.59	-	out								
-	0.61	-	out								
-	0.63	-	out								
-	0.65	-	out								
71	0.66805	0.099		146	74	146	1	0	0	1.3e-14	2.4e-14
-	0.67	-	out								
-	0.69	-	out								
-	0.71	-	out								
-	0.73	-	out								
-	0.75	-	out								
72	0.76705	0.099		147	75	147	1	0	0	1.7e-14	2e-14
-	0.77	-	out								
-	0.79	-	out								
-	0.81	-	out								
-	0.83	-	out								
-	0.85	-	out								
73	0.86605	0.099		148	76	148	2	0	0	1.1e-14	1.7e-14
-	0.87	-	out								

```
   -        0.89           - out
   -        0.91           - out
   -        0.93           - out
   -        0.95           - out
  74     0.96505       0.099      149   77  149     2     0      0  2.2e-14  1.1e-14
   -        0.97           - out
   -        0.99           - out
  75      1.0641       0.099      150   78  150     2     0      0  8.2e-15  1.1e-14
Time-stepping completed.
Solution time: 13 s.
Physical memory: 1.23 GB
Virtual memory: 1.21 GB
Ended at May 28, 2023, 4:10:52 PM.
----- Time-Dependent Solver 1 in Oxygen Pulse/Solution 958 (sol958) ----------->
```

k0Sub=1.51E-8, d=2.4E-6 (su29)

GENERAL

Description	Value
Solution	k0Sub=1.51E-8, d=2.4E-6

LOG

```
<---- Compile Equations: Stationary in Oxygen Pulse/Solution 958 (sol958) ------
Started at May 28, 2023, 4:10:54 PM.
Parameter k0Sub = 1.51E-8 (m/s).
Parameter d = 2.4E-6 (m).
Geometry shape function: Linear Lagrange
Number of vertex elements: 10
Minimum element quality: 0.4544
Time: 0 s.
Physical memory: 1.15 GB
Virtual memory: 1.12 GB
Ended at May 28, 2023, 4:10:55 PM.
----- Compile Equations: Stationary in Oxygen Pulse/Solution 958 (sol958) ----->

<---- Dependent Variables 1 in Oxygen Pulse/Solution 958 (sol958) --------------
Started at May 28, 2023, 4:10:55 PM.
Solution time: 0 s.
Physical memory: 1.15 GB
Virtual memory: 1.13 GB
Ended at May 28, 2023, 4:10:55 PM.
----- Dependent Variables 1 in Oxygen Pulse/Solution 958 (sol958) ------------->

<---- Stationary Solver 1 in Oxygen Pulse/Solution 958 (sol958) ----------------
Started at May 28, 2023, 4:10:55 PM.
Linear solver
Number of degrees of freedom solved for: 18002 (plus 937 internal DOFs).
Symmetric matrices found.
Scales for dependent variables:
Concentration (comp2.Oxi): 0.23
Orthonormal null-space function used.
Iter      SolEst     Damping    Stepsize #Res #Jac #Sol   LinErr   LinRes
   1     1.2e-05   1.0000000     1.2e-05    1    1    1  4.3e-13  9.8e-14
Solution time: 1 s.
Physical memory: 1.18 GB
Virtual memory: 1.16 GB
Ended at May 28, 2023, 4:10:55 PM.
----- Stationary Solver 1 in Oxygen Pulse/Solution 958 (sol958) --------------->

<---- Compile Equations: Oxygen Probe Pulse in Oxygen Pulse/Solution 958
      (sol958) -----------------------------------------------------------------
Started at May 28, 2023, 4:10:55 PM.
Geometry shape function: Linear Lagrange
Time: 0 s.
Physical memory: 1.18 GB
Virtual memory: 1.16 GB
Ended at May 28, 2023, 4:10:56 PM.
----- Compile Equations: Oxygen Probe Pulse in Oxygen Pulse/Solution 958
      (sol958) ---------------------------------------------------------------->

<---- Dependent Variables 2 in Oxygen Pulse/Solution 958 (sol958) --------------
Started at May 28, 2023, 4:10:56 PM.
Initial values of variables solved for: Solution 958 (sol958), Solution Store 1 (sol
985).
Values of variables not solved for: Solution 958 (sol958), Solution Store 1 (sol985)
.
Solution time: 0 s.
Physical memory: 1.18 GB
```

Virtual memory: 1.16 GB
Ended at May 28, 2023, 4:10:56 PM.
----- Dependent Variables 2 in Oxygen Pulse/Solution 958 (sol958) ------------->

<---- Time-Dependent Solver 1 in Oxygen Pulse/Solution 958 (sol958) ------------
Started at May 28, 2023, 4:10:56 PM.
Time-dependent solver (BDF)
Number of degrees of freedom solved for: 18002 (plus 36084 internal DOFs).
Symmetric matrices found.
Scales for dependent variables:
Concentration (comp2.Oxi): 0.23

Step	Time	Stepsize		Res	Jac	Sol	Order	Tfail	NLfail	LinErr	LinRes
0	0	-	out	5	3	5			0	8.7e-16	1.4e-15
1	2.5893e-08	2.5893e-08		7	4	7	1	0	0	3.8e-16	1.1e-15
2	5.1786e-08	2.5893e-08		9	5	9	1	0	0	3.7e-16	1.2e-15
3	1.0357e-07	5.1786e-08		11	6	11	2	0	0	4.3e-16	1.4e-15
4	2.0714e-07	1.0357e-07		13	7	13	2	0	0	4.8e-16	1.7e-15
5	4.1428e-07	2.0714e-07		15	8	15	2	0	0	6.6e-16	3.4e-15
6	6.2143e-07	2.0714e-07		17	9	17	2	0	0	8.3e-16	3.9e-15
7	8.2857e-07	2.0714e-07		19	10	19	2	0	0	7e-16	4e-15
8	1.0357e-06	2.0714e-07		21	11	21	2	0	0	6.2e-16	4.1e-15
9	1.45e-06	4.1428e-07		23	12	23	2	0	0	9.2e-16	6.6e-15
10	1.8643e-06	4.1428e-07		25	13	25	2	0	0	8.8e-16	6e-15
11	2.2786e-06	4.1428e-07		27	14	27	2	0	0	8.2e-16	6.2e-15
12	2.6928e-06	4.1428e-07		29	15	29	2	0	0	8.3e-16	5.8e-15
13	3.5214e-06	8.2857e-07		31	16	31	2	0	0	9.5e-16	8.8e-15
14	4.35e-06	8.2857e-07		33	17	33	2	0	0	1.1e-15	7.7e-15
15	5.1786e-06	8.2857e-07		35	18	35	2	0	0	1.1e-15	7.3e-15
16	6.0071e-06	8.2857e-07		37	19	37	2	0	0	1e-15	6.6e-15
17	7.6643e-06	1.6571e-06		39	20	39	2	0	0	1.2e-15	9.8e-15
18	9.3214e-06	1.6571e-06		41	21	41	2	0	0	1.3e-15	8.3e-15
19	1.0979e-05	1.6571e-06		43	22	43	2	0	0	1.2e-15	8.3e-15
20	1.2636e-05	1.6571e-06		45	23	45	2	0	0	1.5e-15	7.6e-15
21	1.4293e-05	1.6571e-06		47	24	47	2	0	0	1.5e-15	6.8e-15
22	1.7607e-05	3.3143e-06		49	25	49	2	0	0	1.9e-15	9.9e-15
23	2.0921e-05	3.3143e-06		51	26	51	2	0	0	1.8e-15	8.5e-15
24	2.4236e-05	3.3143e-06		53	27	53	2	0	0	1.9e-15	8.4e-15
25	2.755e-05	3.3143e-06		55	28	55	2	0	0	1.6e-15	7.9e-15
26	3.4178e-05	6.6286e-06		57	29	57	2	0	0	2.6e-15	1.2e-14
27	4.0807e-05	6.6286e-06		59	30	59	2	0	0	2.6e-15	1e-14
28	4.7436e-05	6.6286e-06		61	31	61	2	0	0	1.6e-15	9.8e-15
29	5.4064e-05	6.6286e-06		63	32	63	2	0	0	1.7e-15	8.8e-15
30	6.0693e-05	6.6286e-06		65	33	65	2	0	0	1.7e-15	8.2e-15
31	7.395e-05	1.3257e-05		67	34	67	2	0	0	2e-15	1.2e-14
32	8.7207e-05	1.3257e-05		69	35	69	2	0	0	2e-15	1e-14
33	0.00010046	1.3257e-05		71	36	71	2	0	0	3e-15	1e-14
34	0.00011372	1.3257e-05		73	37	73	2	0	0	1.9e-15	9.7e-15
35	0.00014024	2.6514e-05		75	38	75	2	0	0	1.8e-15	1.4e-14
36	0.00016675	2.6514e-05		77	39	77	2	0	0	1.9e-15	1.2e-14
37	0.00019326	2.6514e-05		79	40	79	2	0	0	2.9e-15	1.2e-14
38	0.00021978	2.6514e-05		81	41	81	2	0	0	2.5e-15	1.1e-14
39	0.00027281	5.3028e-05		83	42	83	2	0	0	2.5e-15	1.6e-14
40	0.00032583	5.3028e-05		85	43	85	2	0	0	4.1e-15	1.3e-14
41	0.00037886	5.3028e-05		87	44	87	2	0	0	4e-15	1.3e-14
42	0.00048492	0.00010606		89	45	89	2	0	0	2.4e-15	1.9e-14
43	0.00059098	0.00010606		91	46	91	2	0	0	8.2e-15	1.5e-14

44	0.00069703	0.00010606		93	47	93	2	0	0	3.6e-15	1.5e-14
45	0.00080309	0.00010606		95	48	95	2	0	0	2.6e-15	1.4e-14
46	0.0010152	0.00021211		97	49	97	2	0	0	2.1e-15	1.9e-14
47	0.0012273	0.00021211		99	50	99	2	0	0	1.3e-15	1.6e-14
48	0.0016515	0.00042423		101	51	101	2	0	0	2.4e-15	2.5e-14
49	0.0020758	0.00042423		103	52	103	2	0	0	3.2e-15	2.5e-14
50	0.0025	0.00042423		105	53	105	2	0	0	9.8e-15	2.4e-14
51	0.0029242	0.00042423		107	54	107	2	0	0	2e-15	2.1e-14
52	0.0037727	0.00084845		109	55	109	2	0	0	2.2e-15	2.5e-14
53	0.0046211	0.00084845		111	56	111	2	0	0	6.6e-15	1.9e-14
-	0.005	-	out								
54	0.006318	0.0016969		113	57	113	2	0	0	8.3e-15	3.3e-14
55	0.008015	0.0016969		115	58	115	2	0	0	3.8e-15	3.4e-14
-	0.01	-	out								
56	0.011409	0.0033938		117	59	117	2	0	0	6.1e-15	5e-14
57	0.014803	0.0033938		119	60	119	2	0	0	2.1e-15	4.5e-14
-	0.015	-	out								
58	0.018196	0.0033938		121	61	121	2	0	0	2.7e-15	4.7e-14
-	0.02	-	out								
59	0.024984	0.0067876		123	62	123	2	0	0	2.7e-15	6e-14
-	0.025	-	out								
-	0.03	-	out								
60	0.031772	0.0067876		125	63	125	2	0	0	3.2e-15	4.6e-14
-	0.035	-	out								
-	0.04	-	out								
-	0.045	-	out								
61	0.045347	0.013575		127	64	127	2	0	0	1.2e-14	7.8e-14
-	0.05	-	out								
-	0.055	-	out								
62	0.058922	0.013575		129	65	129	2	0	0	4.6e-15	6.5e-14
-	0.06	-	out								
-	0.065	-	out								
-	0.07	-	out								
63	0.072497	0.013575		131	66	131	2	0	0	3.7e-15	6.5e-14
-	0.075	-	out								
-	0.08	-	out								
-	0.085	-	out								
64	0.086073	0.013575		133	67	133	2	0	0	3e-15	5e-14
-	0.09	-	out								
-	0.095	-	out								
-	0.1	-	out								
-	0.105	-	out								
-	0.11	-	out								
65	0.11322	0.027151		135	68	135	2	0	0	1.9e-14	4.9e-14
-	0.115	-	out								
-	0.12	-	out								
-	0.125	-	out								
-	0.13	-	out								
-	0.135	-	out								
-	0.14	-	out								
-	0.145	-	out								
-	0.15	-	out								
-	0.155	-	out								
-	0.16	-	out								
-	0.165	-	out								
66	0.16752	0.054301		137	69	137	1	0	0	1.5e-14	5.2e-14

-	0.17	- out								
-	0.175	- out								
-	0.18	- out								
-	0.185	- out								
-	0.19	- out								
-	0.195	- out								
-	0.2	- out								
-	0.205	- out								
-	0.21	- out								
-	0.215	- out								
-	0.22	- out								
67	0.22183	0.054301	139	70	139	1	0	0	1.7e-14	3.3e-14
-	0.225	- out								
-	0.23	- out								
-	0.235	- out								
-	0.24	- out								
-	0.245	- out								
-	0.25	- out								
-	0.25	- out								
-	0.27	- out								
-	0.29	- out								
-	0.31	- out								
68	0.32083	0.099	141	71	141	1	0	0	5.6e-15	4.1e-14
-	0.33	- out								
-	0.35	- out								
-	0.37	- out								
-	0.39	- out								
-	0.41	- out								
69	0.41983	0.099	143	72	143	1	0	0	5.9e-15	3.5e-14
-	0.43	- out								
-	0.45	- out								
-	0.47	- out								
-	0.49	- out								
-	0.51	- out								
70	0.51883	0.099	145	73	145	1	0	0	1.3e-14	3e-14
-	0.53	- out								
-	0.55	- out								
-	0.57	- out								
-	0.59	- out								
-	0.61	- out								
71	0.61783	0.099	147	74	147	1	0	0	1.1e-14	2.4e-14
-	0.63	- out								
-	0.65	- out								
-	0.67	- out								
-	0.69	- out								
-	0.71	- out								
72	0.71683	0.099	148	75	148	1	0	0	8.3e-15	2.1e-14
-	0.73	- out								
-	0.75	- out								
-	0.77	- out								
-	0.79	- out								
-	0.81	- out								
73	0.81583	0.099	149	76	149	1	0	0	7.3e-15	1.9e-14
-	0.83	- out								
-	0.85	- out								
-	0.87	- out								

```
   -       0.89            - out
   -       0.91            - out
  74    0.91483        0.099      150   77  150      2      0       0  9.3e-15  1.5e-14
   -       0.93            - out
   -       0.95            - out
   -       0.97            - out
   -       0.99            - out
  75     1.0138        0.099      151   78  151      2      0       0  3.1e-15  9.8e-15
Time-stepping completed.
Solution time: 13 s.
Physical memory: 1.22 GB
Virtual memory: 1.2 GB
Ended at May 28, 2023, 4:11:09 PM.
----- Time-Dependent Solver 1 in Oxygen Pulse/Solution 958 (sol958) ------------>
```

k0Sub=1.51E-8, d=3E-6 (su30)

GENERAL

Description	Value
Solution	k0Sub=1.51E-8, d=3E-6

LOG

```
<---- Compile Equations: Stationary in Oxygen Pulse/Solution 958 (sol958) ------
Started at May 28, 2023, 4:11:12 PM.
Parameter k0Sub = 1.51E-8 (m/s).
Parameter d = 3.0E-6 (m).
Geometry shape function: Linear Lagrange
Number of vertex elements: 10
Minimum element quality: 0.4275
Time: 1 s.
Physical memory: 1.21 GB
Virtual memory: 1.19 GB
Ended at May 28, 2023, 4:11:12 PM.
----- Compile Equations: Stationary in Oxygen Pulse/Solution 958 (sol958) ----->

<---- Dependent Variables 1 in Oxygen Pulse/Solution 958 (sol958) --------------
Started at May 28, 2023, 4:11:12 PM.
Solution time: 0 s.
Physical memory: 1.21 GB
Virtual memory: 1.19 GB
Ended at May 28, 2023, 4:11:12 PM.
----- Dependent Variables 1 in Oxygen Pulse/Solution 958 (sol958) ------------->

<---- Stationary Solver 1 in Oxygen Pulse/Solution 958 (sol958) ----------------
Started at May 28, 2023, 4:11:12 PM.
Linear solver
Number of degrees of freedom solved for: 22990 (plus 935 internal DOFs).
Symmetric matrices found.
Scales for dependent variables:
Concentration (comp2.Oxi): 0.23
Orthonormal null-space function used.
Iter      SolEst     Damping    Stepsize #Res #Jac #Sol   LinErr   LinRes
   1     9.5e-06   1.0000000     9.5e-06    1    1    1  3.8e-13  9.2e-14
Solution time: 1 s.
Physical memory: 1.22 GB
Virtual memory: 1.2 GB
Ended at May 28, 2023, 4:11:13 PM.
----- Stationary Solver 1 in Oxygen Pulse/Solution 958 (sol958) --------------->

<---- Compile Equations: Oxygen Probe Pulse in Oxygen Pulse/Solution 958
      (sol958) ----------------------------------------------------------------
Started at May 28, 2023, 4:11:13 PM.
Geometry shape function: Linear Lagrange
Time: 0 s.
Physical memory: 1.22 GB
Virtual memory: 1.2 GB
Ended at May 28, 2023, 4:11:13 PM.
----- Compile Equations: Oxygen Probe Pulse in Oxygen Pulse/Solution 958
      (sol958) --------------------------------------------------------------->

<---- Dependent Variables 2 in Oxygen Pulse/Solution 958 (sol958) --------------
Started at May 28, 2023, 4:11:13 PM.
Initial values of variables solved for: Solution 958 (sol958), Solution Store 1 (sol
985).
Values of variables not solved for: Solution 958 (sol958), Solution Store 1 (sol985)
.
Solution time: 0 s.
Physical memory: 1.22 GB
```

```
Virtual memory: 1.2 GB
Ended at May 28, 2023, 4:11:13 PM.
----- Dependent Variables 2 in Oxygen Pulse/Solution 958 (sol958) ------------->

<---- Time-Dependent Solver 1 in Oxygen Pulse/Solution 958 (sol958) ------------
Started at May 28, 2023, 4:11:13 PM.
Time-dependent solver (BDF)
Number of degrees of freedom solved for: 22990 (plus 46056 internal DOFs).
Symmetric matrices found.
Scales for dependent variables:
Concentration (comp2.Oxi): 0.23
Step       Time    Stepsize      Res  Jac   Sol Order Tfail NLfail   LinErr   LinRes
   0          0          - out     5    3     5                  0  5.9e-16  8.3e-16
   1 3.5548e-08  3.5548e-08        7    4     7     1     0      0  3.1e-16  9.1e-16
   2 7.1097e-08  3.5548e-08        9    5     9     1     0      0  2.8e-16  9.3e-16
   3 1.4219e-07  7.1097e-08       11    6    11     2     0      0  3.2e-16    1e-15
   4 2.8439e-07  1.4219e-07       13    7    13     2     0      0  3.9e-16  1.3e-15
   5 5.6877e-07  2.8439e-07       15    8    15     2     0      0  5.7e-16  2.4e-15
   6 8.5316e-07  2.8439e-07       17    9    17     2     0      0  5.6e-16  2.8e-15
   7 1.1375e-06  2.8439e-07       19   10    19     2     0      0  5.5e-16  3.2e-15
   8 1.4219e-06  2.8439e-07       21   11    21     2     0      0  4.9e-16  3.3e-15
   9 1.9907e-06  5.6877e-07       23   12    23     2     0      0  6.8e-16    5e-15
  10 2.5595e-06  5.6877e-07       25   13    25     2     0      0  6.9e-16  4.6e-15
  11 3.1283e-06  5.6877e-07       27   14    27     2     0      0  7.7e-16    5e-15
  12  3.697e-06  5.6877e-07       29   15    29     2     0      0    7e-16  4.7e-15
  13 4.8346e-06  1.1375e-06       31   16    31     2     0      0  8.8e-16  6.9e-15
  14 5.9721e-06  1.1375e-06       33   17    33     2     0      0  7.4e-16  6.2e-15
  15 7.1097e-06  1.1375e-06       35   18    35     2     0      0  8.6e-16  6.4e-15
  16 8.2472e-06  1.1375e-06       37   19    37     2     0      0  8.2e-16  5.8e-15
  17 1.0522e-05  2.2751e-06       39   20    39     2     0      0    1e-15  9.1e-15
  18 1.2797e-05  2.2751e-06       41   21    41     2     0      0  1.2e-15    8e-15
  19 1.5073e-05  2.2751e-06       43   22    43     2     0      0  9.4e-16  7.7e-15
  20 1.7348e-05  2.2751e-06       45   23    45     2     0      0  8.8e-16  7.2e-15
  21 2.1898e-05  4.5502e-06       47   24    47     2     0      0  1.3e-15  1.1e-14
  22 2.6448e-05  4.5502e-06       49   25    49     2     0      0  1.5e-15  9.8e-15
  23 3.0998e-05  4.5502e-06       51   26    51     2     0      0  1.5e-15  9.9e-15
  24 3.5548e-05  4.5502e-06       53   27    53     2     0      0  1.3e-15  8.6e-15
  25 4.4649e-05  9.1004e-06       55   28    55     2     0      0  1.2e-15  1.3e-14
  26 5.3749e-05  9.1004e-06       57   29    57     2     0      0  1.3e-15  1.2e-14
  27  6.285e-05  9.1004e-06       59   30    59     2     0      0  8.5e-16  1.1e-14
  28  7.195e-05  9.1004e-06       61   31    61     2     0      0  1.6e-15  1.1e-14
  29 9.0151e-05  1.8201e-05       63   32    63     2     0      0  2.1e-15  1.6e-14
  30 0.00010835  1.8201e-05       65   33    65     2     0      0  1.6e-15  1.4e-14
  31 0.00012655  1.8201e-05       67   34    67     2     0      0  1.8e-15  1.3e-14
  32 0.00014475  1.8201e-05       69   35    69     2     0      0  1.4e-15  1.2e-14
  33 0.00018115  3.6402e-05       71   36    71     2     0      0  4.2e-15  1.7e-14
  34 0.00021756  3.6402e-05       73   37    73     2     0      0  1.1e-15  1.4e-14
  35 0.00025396  3.6402e-05       75   38    75     2     0      0  1.3e-15  1.5e-14
  36 0.00029036  3.6402e-05       77   39    77     2     0      0  2.9e-15  1.4e-14
  37 0.00036316  7.2803e-05       79   40    79     2     0      0  2.4e-15    2e-14
  38 0.00043597  7.2803e-05       81   41    81     2     0      0  3.9e-15  1.7e-14
  39 0.00058157  0.00014561       83   42    83     2     0      0    2e-15  2.7e-14
  40 0.00072718  0.00014561       85   43    85     2     0      0  2.6e-15  2.3e-14
  41 0.00087278  0.00014561       87   44    87     2     0      0  2.1e-15  2.3e-14
  42  0.0010184  0.00014561       89   45    89     2     0      0  5.8e-15    2e-14
  43  0.0013096  0.00029121       91   46    91     2     0      0    4e-15  2.6e-14
```

44	0.0016008	0.00029121		93	47	93	2	0	0	3.9e-15	2.1e-14
45	0.0021832	0.00058243		95	48	95	2	0	0	2.8e-15	3.3e-14
46	0.0027657	0.00058243		97	49	97	2	0	0	2.5e-15	3.1e-14
47	0.0033481	0.00058243		99	50	99	2	0	0	5.8e-15	3.3e-14
48	0.0045129	0.0011649		101	51	101	2	0	0	5.8e-15	4.1e-14
-	0.005	-	out								
49	0.0056778	0.0011649		103	52	103	2	0	0	4.3e-15	2.8e-14
50	0.0080075	0.0023297		105	53	105	2	0	0	3.4e-15	4.5e-14
-	0.01	-	out								
51	0.010337	0.0023297		107	54	107	2	0	0	3.1e-15	4.5e-14
52	0.012667	0.0023297		109	55	109	2	0	0	2.5e-15	4.7e-14
-	0.015	-	out								
53	0.017326	0.0046594		111	56	111	2	0	0	4.7e-15	6.5e-14
-	0.02	-	out								
54	0.021986	0.0046594		113	57	113	2	0	0	3e-15	4.8e-14
-	0.025	-	out								
-	0.03	-	out								
55	0.031304	0.0093188		115	58	115	2	0	0	6.2e-15	7.9e-14
-	0.035	-	out								
-	0.04	-	out								
56	0.040623	0.0093188		117	59	117	2	0	0	6e-15	7.1e-14
-	0.045	-	out								
57	0.049942	0.0093188		119	60	119	2	0	0	1e-14	7.3e-14
-	0.05	-	out								
-	0.055	-	out								
-	0.06	-	out								
-	0.065	-	out								
58	0.06858	0.018638		121	61	121	2	0	0	6.8e-15	9.7e-14
-	0.07	-	out								
-	0.075	-	out								
-	0.08	-	out								
-	0.085	-	out								
59	0.087217	0.018638		123	62	123	1	0	0	8e-15	8.4e-14
-	0.09	-	out								
-	0.095	-	out								
-	0.1	-	out								
-	0.105	-	out								
-	0.11	-	out								
-	0.115	-	out								
-	0.12	-	out								
60	0.12449	0.037275		125	63	125	1	0	0	4.9e-15	6.4e-14
-	0.125	-	out								
-	0.13	-	out								
-	0.135	-	out								
-	0.14	-	out								
-	0.145	-	out								
-	0.15	-	out								
-	0.155	-	out								
-	0.16	-	out								
-	0.165	-	out								
-	0.17	-	out								
-	0.175	-	out								
-	0.18	-	out								
-	0.185	-	out								
-	0.19	-	out								
-	0.195	-	out								

61	0.19904	0.07455		127	64	127	1	0	0	1.4e-14	8.5e-14
-	0.2	-	out								
-	0.205	-	out								
-	0.21	-	out								
-	0.215	-	out								
-	0.22	-	out								
-	0.225	-	out								
-	0.23	-	out								
-	0.235	-	out								
-	0.24	-	out								
-	0.245	-	out								
-	0.25	-	out								
-	0.25	-	out								
-	0.27	-	out								
62	0.27359	0.07455		129	65	129	1	0	0	2.2e-14	7.2e-14
-	0.29	-	out								
-	0.31	-	out								
-	0.33	-	out								
-	0.35	-	out								
-	0.37	-	out								
63	0.37259	0.099		131	66	131	1	0	0	2e-14	6.8e-14
-	0.39	-	out								
-	0.41	-	out								
-	0.43	-	out								
-	0.45	-	out								
-	0.47	-	out								
64	0.47159	0.099		133	67	133	1	0	0	1.4e-14	5.1e-14
-	0.49	-	out								
-	0.51	-	out								
-	0.53	-	out								
-	0.55	-	out								
-	0.57	-	out								
65	0.57059	0.099		135	68	135	1	0	0	2.8e-15	4.2e-14
-	0.59	-	out								
-	0.61	-	out								
-	0.63	-	out								
-	0.65	-	out								
66	0.66959	0.099		136	69	136	1	0	0	7.4e-15	3.6e-14
-	0.67	-	out								
-	0.69	-	out								
-	0.71	-	out								
-	0.73	-	out								
-	0.75	-	out								
67	0.76859	0.099		137	70	137	1	0	0	2.2e-14	3.1e-14
-	0.77	-	out								
-	0.79	-	out								
-	0.81	-	out								
-	0.83	-	out								
-	0.85	-	out								
68	0.86759	0.099		138	71	138	2	0	0	1e-14	2.6e-14
-	0.87	-	out								
-	0.89	-	out								
-	0.91	-	out								
-	0.93	-	out								
-	0.95	-	out								
69	0.96659	0.099		139	72	139	2	0	0	1.1e-14	1.7e-14

```
   -        0.97            - out
   -        0.99            - out
  70      1.0656       0.099      140   73  140     2     0      0  3.7e-15  1.6e-14
Time-stepping completed.
Solution time: 16 s.
Physical memory: 1.24 GB
Virtual memory: 1.23 GB
Ended at May 28, 2023, 4:11:29 PM.
----- Time-Dependent Solver 1 in Oxygen Pulse/Solution 958 (sol958) ----------->
```

k0Sub=1.51E-8, d=4E-6 (su31)

GENERAL

Description	Value
Solution	k0Sub=1.51E-8, d=4E-6

LOG

```
<---- Compile Equations: Stationary in Oxygen Pulse/Solution 958 (sol958) ------
Started at May 28, 2023, 4:11:32 PM.
Parameter k0Sub = 1.51E-8 (m/s).
Parameter d = 4.0E-6 (m).
Geometry shape function: Linear Lagrange
Number of vertex elements: 10
Minimum element quality: 0.4317
Time: 1 s.
Physical memory: 1.16 GB
Virtual memory: 1.14 GB
Ended at May 28, 2023, 4:11:33 PM.
----- Compile Equations: Stationary in Oxygen Pulse/Solution 958 (sol958) ----->

<---- Dependent Variables 1 in Oxygen Pulse/Solution 958 (sol958) --------------
Started at May 28, 2023, 4:11:33 PM.
Solution time: 0 s.
Physical memory: 1.16 GB
Virtual memory: 1.14 GB
Ended at May 28, 2023, 4:11:33 PM.
----- Dependent Variables 1 in Oxygen Pulse/Solution 958 (sol958) ------------->

<---- Stationary Solver 1 in Oxygen Pulse/Solution 958 (sol958) ----------------
Started at May 28, 2023, 4:11:33 PM.
Linear solver
Number of degrees of freedom solved for: 18182 (plus 936 internal DOFs).
Symmetric matrices found.
Scales for dependent variables:
Concentration (comp2.Oxi): 0.23
Orthonormal null-space function used.
Iter      SolEst     Damping    Stepsize #Res #Jac #Sol   LinErr   LinRes
   1     9.2e-06   1.0000000     9.2e-06    1    1    1    4e-13  7.5e-14
Solution time: 1 s.
Physical memory: 1.18 GB
Virtual memory: 1.16 GB
Ended at May 28, 2023, 4:11:33 PM.
----- Stationary Solver 1 in Oxygen Pulse/Solution 958 (sol958) --------------->

<---- Compile Equations: Oxygen Probe Pulse in Oxygen Pulse/Solution 958
      (sol958) ----------------------------------------------------------------
Started at May 28, 2023, 4:11:33 PM.
Geometry shape function: Linear Lagrange
Time: 1 s.
Physical memory: 1.2 GB
Virtual memory: 1.18 GB
Ended at May 28, 2023, 4:11:34 PM.
----- Compile Equations: Oxygen Probe Pulse in Oxygen Pulse/Solution 958
      (sol958) --------------------------------------------------------------->

<---- Dependent Variables 2 in Oxygen Pulse/Solution 958 (sol958) --------------
Started at May 28, 2023, 4:11:34 PM.
Initial values of variables solved for: Solution 958 (sol958), Solution Store 1 (sol
985).
Values of variables not solved for: Solution 958 (sol958), Solution Store 1 (sol985)
.
Solution time: 0 s.
Physical memory: 1.21 GB
```

Virtual memory: 1.18 GB
Ended at May 28, 2023, 4:11:34 PM.
----- Dependent Variables 2 in Oxygen Pulse/Solution 958 (sol958) ------------->

<---- Time-Dependent Solver 1 in Oxygen Pulse/Solution 958 (sol958) ------------
Started at May 28, 2023, 4:11:34 PM.
Time-dependent solver (BDF)
Number of degrees of freedom solved for: 18182 (plus 36438 internal DOFs).
Symmetric matrices found.
Scales for dependent variables:
Concentration (comp2.Oxi): 0.23

Step	Time	Stepsize		Res	Jac	Sol	Order	Tfail	NLfail	LinErr	LinRes
0	0	-	out	5	3	5			0	7.9e-16	1.4e-15
1	2.614e-08	2.614e-08		7	4	7	1	0	0	3.6e-16	1e-15
2	5.2279e-08	2.614e-08		9	5	9	1	0	0	3.6e-16	1.2e-15
3	1.0456e-07	5.2279e-08		11	6	11	2	0	0	3.6e-16	1.3e-15
4	2.0912e-07	1.0456e-07		13	7	13	2	0	0	4.6e-16	1.8e-15
5	4.1824e-07	2.0912e-07		15	8	15	2	0	0	7.1e-16	3.4e-15
6	6.2735e-07	2.0912e-07		17	9	17	2	0	0	6.5e-16	3.7e-15
7	8.3647e-07	2.0912e-07		19	10	19	2	0	0	6.4e-16	4e-15
8	1.0456e-06	2.0912e-07		21	11	21	2	0	0	7e-16	4e-15
9	1.4638e-06	4.1824e-07		23	12	23	2	0	0	7.7e-16	6.4e-15
10	1.8821e-06	4.1824e-07		25	13	25	2	0	0	7.1e-16	5.8e-15
11	2.3003e-06	4.1824e-07		27	14	27	2	0	0	6.6e-16	5.9e-15
12	2.7185e-06	4.1824e-07		29	15	29	2	0	0	7.6e-16	5.7e-15
13	3.555e-06	8.3647e-07		31	16	31	2	0	0	1e-15	8.5e-15
14	4.3915e-06	8.3647e-07		33	17	33	2	0	0	1e-15	7.3e-15
15	5.2279e-06	8.3647e-07		35	18	35	2	0	0	7.4e-16	7.3e-15
16	6.0644e-06	8.3647e-07		37	19	37	2	0	0	1.1e-15	6.6e-15
17	7.7374e-06	1.6729e-06		39	20	39	2	0	0	1.2e-15	9.9e-15
18	9.4103e-06	1.6729e-06		41	21	41	2	0	0	9.4e-16	8.2e-15
19	1.1083e-05	1.6729e-06		43	22	43	2	0	0	8.7e-16	8.1e-15
20	1.2756e-05	1.6729e-06		45	23	45	2	0	0	1.1e-15	7.5e-15
21	1.4429e-05	1.6729e-06		47	24	47	2	0	0	8.2e-16	6.5e-15
22	1.7775e-05	3.3459e-06		49	25	49	2	0	0	1.5e-15	9.7e-15
23	2.1121e-05	3.3459e-06		51	26	51	2	0	0	1.5e-15	8.6e-15
24	2.4467e-05	3.3459e-06		53	27	53	2	0	0	1.9e-15	8.4e-15
25	2.7813e-05	3.3459e-06		55	28	55	2	0	0	1.6e-15	7.9e-15
26	3.4504e-05	6.6918e-06		57	29	57	2	0	0	1.5e-15	1.2e-14
27	4.1196e-05	6.6918e-06		59	30	59	2	0	0	1.5e-15	9.5e-15
28	4.7888e-05	6.6918e-06		61	31	61	2	0	0	2e-15	1e-14
29	5.458e-05	6.6918e-06		63	32	63	2	0	0	1.4e-15	8.9e-15
30	6.1271e-05	6.6918e-06		65	33	65	2	0	0	1.7e-15	7.8e-15
31	7.4655e-05	1.3384e-05		67	34	67	2	0	0	1.5e-15	1.2e-14
32	8.8039e-05	1.3384e-05		69	35	69	2	0	0	1.5e-15	1e-14
33	0.00010142	1.3384e-05		71	36	71	2	0	0	1.3e-15	1.1e-14
34	0.00011481	1.3384e-05		73	37	73	2	0	0	1.2e-15	9.6e-15
35	0.00014157	2.6767e-05		75	38	75	2	0	0	3.2e-15	1.5e-14
36	0.00016834	2.6767e-05		77	39	77	2	0	0	2e-15	1.2e-14
37	0.00019511	2.6767e-05		79	40	79	2	0	0	1.8e-15	1.2e-14
38	0.00022187	2.6767e-05		81	41	81	2	0	0	1.5e-15	1.1e-14
39	0.00027541	5.3534e-05		83	42	83	2	0	0	2.1e-15	1.6e-14
40	0.00032894	5.3534e-05		85	43	85	2	0	0	2.8e-15	1.4e-14
41	0.00038248	5.3534e-05		87	44	87	2	0	0	3.9e-15	1.3e-14
42	0.00048954	0.00010707		89	45	89	2	0	0	4.2e-15	1.9e-14
43	0.00059661	0.00010707		91	46	91	2	0	0	5.1e-15	1.6e-14

44	0.00070368	0.00010707		93	47	93	2	0	0	4e-15	1.6e-14
45	0.00081075	0.00010707		95	48	95	2	0	0	4e-15	1.4e-14
46	0.0010249	0.00021414		97	49	97	2	0	0	1.6e-15	1.9e-14
47	0.001239	0.00021414		99	50	99	2	0	0	3.4e-15	1.7e-14
48	0.0016673	0.00042827		101	51	101	2	0	0	1.9e-15	2.5e-14
49	0.0020956	0.00042827		103	52	103	2	0	0	4.1e-15	2.2e-14
50	0.0025238	0.00042827		105	53	105	2	0	0	2.8e-15	2.3e-14
51	0.0029521	0.00042827		107	54	107	2	0	0	2.9e-15	2.1e-14
52	0.0038087	0.00085655		109	55	109	2	0	0	3.5e-15	2.7e-14
53	0.0046652	0.00085655		111	56	111	2	0	0	2.6e-15	2.1e-14
-	0.005	-	out								
54	0.0063783	0.0017131		113	57	113	2	0	0	4.2e-15	3.2e-14
55	0.0080914	0.0017131		115	58	115	2	0	0	1.9e-15	2.9e-14
-	0.01	-	out								
56	0.011518	0.0034262		117	59	117	2	0	0	2e-15	4.3e-14
57	0.014944	0.0034262		119	60	119	1	0	0	6.7e-15	5.5e-14
-	0.015	-	out								
58	0.01837	0.0034262		121	61	121	1	0	0	4.4e-15	4.5e-14
-	0.02	-	out								
-	0.025	-	out								
59	0.025222	0.0068524		123	62	123	1	0	0	2.9e-15	7.3e-14
-	0.03	-	out								
60	0.032075	0.0068524		125	63	125	1	0	0	5.7e-15	7.3e-14
-	0.035	-	out								
-	0.04	-	out								
-	0.045	-	out								
61	0.045779	0.013705		127	64	127	1	0	0	1.6e-14	1e-13
-	0.05	-	out								
-	0.055	-	out								
62	0.059484	0.013705		129	65	129	1	0	0	6.2e-15	8.9e-14
-	0.06	-	out								
-	0.065	-	out								
-	0.07	-	out								
-	0.075	-	out								
-	0.08	-	out								
-	0.085	-	out								
63	0.086894	0.027409		131	66	131	1	0	0	7.9e-15	1.1e-13
-	0.09	-	out								
-	0.095	-	out								
-	0.1	-	out								
-	0.105	-	out								
-	0.11	-	out								
64	0.1143	0.027409		133	67	133	1	0	0	1e-14	8e-14
-	0.115	-	out								
-	0.12	-	out								
-	0.125	-	out								
-	0.13	-	out								
-	0.135	-	out								
-	0.14	-	out								
-	0.145	-	out								
-	0.15	-	out								
-	0.155	-	out								
-	0.16	-	out								
-	0.165	-	out								
65	0.16912	0.054819		135	68	135	1	0	0	1.3e-14	8.8e-14
-	0.17	-	out								

-	0.175	- out								
-	0.18	- out								
-	0.185	- out								
-	0.19	- out								
-	0.195	- out								
-	0.2	- out								
-	0.205	- out								
-	0.21	- out								
-	0.215	- out								
-	0.22	- out								
66	0.22394	0.054819	137	69	137	1	0	0	9.9e-15	6.2e-14
-	0.225	- out								
-	0.23	- out								
-	0.235	- out								
-	0.24	- out								
-	0.245	- out								
-	0.25	- out								
-	0.25	- out								
-	0.27	- out								
-	0.29	- out								
-	0.31	- out								
67	0.32294	0.099	139	70	139	1	0	0	8.4e-15	6.5e-14
-	0.33	- out								
-	0.35	- out								
-	0.37	- out								
-	0.39	- out								
-	0.41	- out								
68	0.42194	0.099	141	71	141	1	0	0	7.2e-15	4.7e-14
-	0.43	- out								
-	0.45	- out								
-	0.47	- out								
-	0.49	- out								
-	0.51	- out								
69	0.52094	0.099	143	72	143	1	0	0	7.2e-15	3.6e-14
-	0.53	- out								
-	0.55	- out								
-	0.57	- out								
-	0.59	- out								
-	0.61	- out								
70	0.61994	0.099	145	73	145	1	0	0	2.3e-15	3e-14
-	0.63	- out								
-	0.65	- out								
-	0.67	- out								
-	0.69	- out								
-	0.71	- out								
71	0.71894	0.099	146	74	146	1	0	0	7.9e-15	2.4e-14
-	0.73	- out								
-	0.75	- out								
-	0.77	- out								
-	0.79	- out								
-	0.81	- out								
72	0.81794	0.099	147	75	147	1	0	0	1.4e-14	2.2e-14
-	0.83	- out								
-	0.85	- out								
-	0.87	- out								
-	0.89	- out								

```
   -        0.91            - out
  73     0.91694        0.099       148    76   148      2      0       0  8.1e-15  1.7e-14
   -        0.93            - out
   -        0.95            - out
   -        0.97            - out
   -        0.99            - out
  74      1.0159        0.099       149    77   149      2      0       0  6.2e-15  1.1e-14
Time-stepping completed.
Solution time: 14 s.
Physical memory: 1.24 GB
Virtual memory: 1.22 GB
Ended at May 28, 2023, 4:11:48 PM.
----- Time-Dependent Solver 1 in Oxygen Pulse/Solution 958 (sol958) ----------->
```

k0Sub=1.51E-8, d=5E-6 (su32)

GENERAL

Description	Value
Solution	k0Sub=1.51E-8, d=5E-6

LOG

```
<---- Compile Equations: Stationary in Oxygen Pulse/Solution 958 (sol958) ------
Started at May 28, 2023, 4:11:51 PM.
Parameter k0Sub = 1.51E-8 (m/s).
Parameter d = 4.9999999999999996E-6 (m).
Geometry shape function: Linear Lagrange
Number of vertex elements: 10
Minimum element quality: 0.4532
Time: 1 s.
Physical memory: 1.2 GB
Virtual memory: 1.18 GB
Ended at May 28, 2023, 4:11:51 PM.
----- Compile Equations: Stationary in Oxygen Pulse/Solution 958 (sol958) ----->

<---- Dependent Variables 1 in Oxygen Pulse/Solution 958 (sol958) --------------
Started at May 28, 2023, 4:11:51 PM.
Solution time: 0 s.
Physical memory: 1.2 GB
Virtual memory: 1.18 GB
Ended at May 28, 2023, 4:11:51 PM.
----- Dependent Variables 1 in Oxygen Pulse/Solution 958 (sol958) ------------->

<---- Stationary Solver 1 in Oxygen Pulse/Solution 958 (sol958) ----------------
Started at May 28, 2023, 4:11:51 PM.
Linear solver
Number of degrees of freedom solved for: 18304 (plus 936 internal DOFs).
Symmetric matrices found.
Scales for dependent variables:
Concentration (comp2.Oxi): 0.23
Orthonormal null-space function used.
Iter      SolEst     Damping    Stepsize #Res #Jac #Sol   LinErr   LinRes
   1     7.9e-06   1.0000000     7.9e-06    1    1    1  3.2e-13    7e-14
Solution time: 1 s.
Physical memory: 1.21 GB
Virtual memory: 1.19 GB
Ended at May 28, 2023, 4:11:52 PM.
----- Stationary Solver 1 in Oxygen Pulse/Solution 958 (sol958) --------------->

<---- Compile Equations: Oxygen Probe Pulse in Oxygen Pulse/Solution 958
      (sol958) -----------------------------------------------------------------
Started at May 28, 2023, 4:11:52 PM.
Geometry shape function: Linear Lagrange
Time: 1 s.
Physical memory: 1.21 GB
Virtual memory: 1.19 GB
Ended at May 28, 2023, 4:11:52 PM.
----- Compile Equations: Oxygen Probe Pulse in Oxygen Pulse/Solution 958
      (sol958) ---------------------------------------------------------------->

<---- Dependent Variables 2 in Oxygen Pulse/Solution 958 (sol958) --------------
Started at May 28, 2023, 4:11:52 PM.
Initial values of variables solved for: Solution 958 (sol958), Solution Store 1 (sol
985).
Values of variables not solved for: Solution 958 (sol958), Solution Store 1 (sol985)
.
Solution time: 0 s.
Physical memory: 1.21 GB
```

Virtual memory: 1.19 GB
Ended at May 28, 2023, 4:11:53 PM.
----- Dependent Variables 2 in Oxygen Pulse/Solution 958 (sol958) ------------->

<---- Time-Dependent Solver 1 in Oxygen Pulse/Solution 958 (sol958) ------------
Started at May 28, 2023, 4:11:53 PM.
Time-dependent solver (BDF)
Number of degrees of freedom solved for: 18304 (plus 36680 internal DOFs).
Symmetric matrices found.
Scales for dependent variables:
Concentration (comp2.Oxi): 0.23

Step	Time	Stepsize		Res	Jac	Sol	Order	Tfail	NLfail	LinErr	LinRes
0	0	-	out	5	3	5			0	8.4e-16	1.5e-15
1	2.6321e-08	2.6321e-08		7	4	7	1	0	0	3.4e-16	9.7e-16
2	5.2641e-08	2.6321e-08		9	5	9	1	0	0	3.5e-16	1.1e-15
3	1.0528e-07	5.2641e-08		11	6	11	2	0	0	4e-16	1.3e-15
4	2.1057e-07	1.0528e-07		13	7	13	2	0	0	5.7e-16	1.7e-15
5	4.2113e-07	2.1057e-07		15	8	15	2	0	0	7.1e-16	3.3e-15
6	6.317e-07	2.1057e-07		17	9	17	2	0	0	5.9e-16	3.7e-15
7	8.4226e-07	2.1057e-07		19	10	19	2	0	0	7.2e-16	3.8e-15
8	1.0528e-06	2.1057e-07		21	11	21	2	0	0	7.2e-16	4e-15
9	1.474e-06	4.2113e-07		23	12	23	2	0	0	9.2e-16	6.4e-15
10	1.8951e-06	4.2113e-07		25	13	25	2	0	0	7.8e-16	6.2e-15
11	2.3162e-06	4.2113e-07		27	14	27	2	0	0	9.4e-16	6e-15
12	2.7373e-06	4.2113e-07		29	15	29	2	0	0	7.5e-16	5.8e-15
13	3.5796e-06	8.4226e-07		31	16	31	2	0	0	1.4e-15	8.9e-15
14	4.4219e-06	8.4226e-07		33	17	33	2	0	0	1.5e-15	7.5e-15
15	5.2641e-06	8.4226e-07		35	18	35	2	0	0	1.1e-15	7.3e-15
16	6.1064e-06	8.4226e-07		37	19	37	2	0	0	9.7e-16	6.9e-15
17	7.7909e-06	1.6845e-06		39	20	39	2	0	0	1.3e-15	9.9e-15
18	9.4754e-06	1.6845e-06		41	21	41	2	0	0	1.5e-15	8.4e-15
19	1.116e-05	1.6845e-06		43	22	43	2	0	0	1.3e-15	8.5e-15
20	1.2844e-05	1.6845e-06		45	23	45	2	0	0	1.1e-15	7.5e-15
21	1.4529e-05	1.6845e-06		47	24	47	2	0	0	1.5e-15	6.8e-15
22	1.7898e-05	3.369e-06		49	25	49	2	0	0	1.8e-15	1e-14
23	2.1267e-05	3.369e-06		51	26	51	2	0	0	1.4e-15	9.1e-15
24	2.4636e-05	3.369e-06		53	27	53	2	0	0	1.5e-15	8.4e-15
25	2.8005e-05	3.369e-06		55	28	55	2	0	0	2.1e-15	8.2e-15
26	3.4743e-05	6.7381e-06		57	29	57	2	0	0	3.1e-15	1.2e-14
27	4.1481e-05	6.7381e-06		59	30	59	2	0	0	1.5e-15	1e-14
28	4.8219e-05	6.7381e-06		61	31	61	2	0	0	1.6e-15	1e-14
29	5.4958e-05	6.7381e-06		63	32	63	2	0	0	1.6e-15	9.4e-15
30	6.1696e-05	6.7381e-06		65	33	65	2	0	0	1.7e-15	8.1e-15
31	7.5172e-05	1.3476e-05		67	34	67	2	0	0	1.9e-15	1.2e-14
32	8.8648e-05	1.3476e-05		69	35	69	2	0	0	1.5e-15	1.1e-14
33	0.00010212	1.3476e-05		71	36	71	2	0	0	2.3e-15	1.1e-14
34	0.0001156	1.3476e-05		73	37	73	2	0	0	2.5e-15	9.9e-15
35	0.00014255	2.6952e-05		75	38	75	2	0	0	1.5e-15	1.5e-14
36	0.00016951	2.6952e-05		77	39	77	2	0	0	1.1e-15	1.3e-14
37	0.00019646	2.6952e-05		79	40	79	2	0	0	1.6e-15	1.2e-14
38	0.00022341	2.6952e-05		81	41	81	2	0	0	2.5e-15	1.1e-14
39	0.00027731	5.3905e-05		83	42	83	2	0	0	3e-15	1.7e-14
40	0.00033122	5.3905e-05		85	43	85	2	0	0	2.7e-15	1.4e-14
41	0.00038512	5.3905e-05		87	44	87	2	0	0	1.2e-15	1.4e-14
42	0.00049293	0.00010781		89	45	89	2	0	0	2.8e-15	2.1e-14
43	0.00060074	0.00010781		91	46	91	2	0	0	2.3e-15	1.7e-14

44	0.00070855	0.00010781	93	47	93	2	0	0	3.1e-15	1.7e-14
45	0.00081636	0.00010781	95	48	95	2	0	0	2.8e-15	1.5e-14
46	0.001032	0.00021562	97	49	97	2	0	0	1.6e-15	2e-14
47	0.0012476	0.00021562	99	50	99	2	0	0	3.7e-15	1.6e-14
48	0.0016788	0.00043124	101	51	101	2	0	0	2.7e-15	2.6e-14
49	0.0021101	0.00043124	103	52	103	2	0	0	1.8e-15	2.2e-14
50	0.0025413	0.00043124	105	53	105	2	0	0	2.9e-15	2.3e-14
51	0.0029726	0.00043124	107	54	107	2	0	0	6.3e-15	2e-14
52	0.003835	0.00086248	109	55	109	2	0	0	8e-15	2.7e-14
53	0.0046975	0.00086248	111	56	111	2	0	0	2.5e-15	2.1e-14
-	0.005	- out								
54	0.0064225	0.001725	113	57	113	2	0	0	3.1e-15	3.4e-14
55	0.0081474	0.001725	115	58	115	2	0	0	3.5e-15	3e-14
-	0.01	- out								
56	0.011597	0.0034499	117	59	117	2	0	0	3.3e-15	4.4e-14
-	0.015	- out								
57	0.015047	0.0034499	119	60	119	1	0	0	6.7e-15	5.2e-14
58	0.018497	0.0034499	121	61	121	1	0	0	5.8e-15	3.9e-14
-	0.02	- out								
-	0.025	- out								
59	0.025397	0.0068998	123	62	123	1	0	0	7.9e-15	6.6e-14
-	0.03	- out								
60	0.032297	0.0068998	125	63	125	1	0	0	6.4e-15	6.5e-14
-	0.035	- out								
-	0.04	- out								
-	0.045	- out								
61	0.046096	0.0138	127	64	127	1	0	0	6e-15	9.4e-14
-	0.05	- out								
-	0.055	- out								
62	0.059896	0.0138	129	65	129	1	0	0	4.6e-15	8.5e-14
-	0.06	- out								
-	0.065	- out								
-	0.07	- out								
-	0.075	- out								
-	0.08	- out								
-	0.085	- out								
63	0.087495	0.027599	131	66	131	1	0	0	1.6e-14	1.1e-13
-	0.09	- out								
-	0.095	- out								
-	0.1	- out								
-	0.105	- out								
-	0.11	- out								
-	0.115	- out								
64	0.11509	0.027599	133	67	133	1	0	0	9.3e-15	8.5e-14
-	0.12	- out								
-	0.125	- out								
-	0.13	- out								
-	0.135	- out								
-	0.14	- out								
-	0.145	- out								
-	0.15	- out								
-	0.155	- out								
-	0.16	- out								
-	0.165	- out								
-	0.17	- out								
65	0.17029	0.055198	135	68	135	1	0	0	5e-15	8.8e-14

-	0.175	- out								
-	0.18	- out								
-	0.185	- out								
-	0.19	- out								
-	0.195	- out								
-	0.2	- out								
-	0.205	- out								
-	0.21	- out								
-	0.215	- out								
-	0.22	- out								
-	0.225	- out								
66	0.22549	0.055198	137	69	137	1	0	0	1.3e-14	6.9e-14
-	0.23	- out								
-	0.235	- out								
-	0.24	- out								
-	0.245	- out								
-	0.25	- out								
-	0.25	- out								
-	0.27	- out								
-	0.29	- out								
-	0.31	- out								
67	0.32449	0.099	139	70	139	1	0	0	1.2e-14	7e-14
-	0.33	- out								
-	0.35	- out								
-	0.37	- out								
-	0.39	- out								
-	0.41	- out								
68	0.42349	0.099	141	71	141	1	0	0	7.3e-15	5e-14
-	0.43	- out								
-	0.45	- out								
-	0.47	- out								
-	0.49	- out								
-	0.51	- out								
69	0.52249	0.099	143	72	143	1	0	0	6.4e-15	3.9e-14
-	0.53	- out								
-	0.55	- out								
-	0.57	- out								
-	0.59	- out								
-	0.61	- out								
70	0.62149	0.099	145	73	145	1	0	0	6e-15	3.1e-14
-	0.63	- out								
-	0.65	- out								
-	0.67	- out								
-	0.69	- out								
-	0.71	- out								
71	0.72049	0.099	146	74	146	1	0	0	1e-14	2.6e-14
-	0.73	- out								
-	0.75	- out								
-	0.77	- out								
-	0.79	- out								
-	0.81	- out								
72	0.81949	0.099	147	75	147	1	0	0	1.2e-14	2.1e-14
-	0.83	- out								
-	0.85	- out								
-	0.87	- out								
-	0.89	- out								

```
   -        0.91           - out
   73     0.91849       0.099      148   76  148     2     0      0  8.2e-15  1.8e-14
   -        0.93           - out
   -        0.95           - out
   -        0.97           - out
   -        0.99           - out
   74      1.0175       0.099      149   77  149     2     0      0    3e-15  1.1e-14
Time-stepping completed.
Solution time: 15 s.
Physical memory: 1.24 GB
Virtual memory: 1.22 GB
Ended at May 28, 2023, 4:12:07 PM.
----- Time-Dependent Solver 1 in Oxygen Pulse/Solution 958 (sol958) ----------->
```

k0Sub=1.51E-8, d=6E-6 (su33)

GENERAL

Description	Value
Solution	k0Sub=1.51E-8, d=6E-6

LOG

```
<---- Compile Equations: Stationary in Oxygen Pulse/Solution 958 (sol958) ------
Started at May 28, 2023, 4:12:10 PM.
Parameter k0Sub = 1.51E-8 (m/s).
Parameter d = 6.0E-6 (m).
Geometry shape function: Linear Lagrange
Number of vertex elements: 10
Minimum element quality: 0.432
Time: 1 s.
Physical memory: 1.23 GB
Virtual memory: 1.21 GB
Ended at May 28, 2023, 4:12:11 PM.
----- Compile Equations: Stationary in Oxygen Pulse/Solution 958 (sol958) ----->

<---- Dependent Variables 1 in Oxygen Pulse/Solution 958 (sol958) --------------
Started at May 28, 2023, 4:12:11 PM.
Solution time: 0 s.
Physical memory: 1.23 GB
Virtual memory: 1.21 GB
Ended at May 28, 2023, 4:12:11 PM.
----- Dependent Variables 1 in Oxygen Pulse/Solution 958 (sol958) ------------->

<---- Stationary Solver 1 in Oxygen Pulse/Solution 958 (sol958) ----------------
Started at May 28, 2023, 4:12:11 PM.
Linear solver
Number of degrees of freedom solved for: 18385 (plus 936 internal DOFs).
Symmetric matrices found.
Scales for dependent variables:
Concentration (comp2.Oxi): 0.23
Orthonormal null-space function used.
Iter      SolEst     Damping    Stepsize #Res #Jac #Sol   LinErr   LinRes
   1       7e-06   1.0000000       7e-06    1    1    1  2.6e-13  6.4e-14
Solution time: 1 s.
Physical memory: 1.24 GB
Virtual memory: 1.22 GB
Ended at May 28, 2023, 4:12:11 PM.
----- Stationary Solver 1 in Oxygen Pulse/Solution 958 (sol958) --------------->

<---- Compile Equations: Oxygen Probe Pulse in Oxygen Pulse/Solution 958
      (sol958) ----------------------------------------------------------------
Started at May 28, 2023, 4:12:11 PM.
Geometry shape function: Linear Lagrange
Time: 1 s.
Physical memory: 1.24 GB
Virtual memory: 1.22 GB
Ended at May 28, 2023, 4:12:12 PM.
----- Compile Equations: Oxygen Probe Pulse in Oxygen Pulse/Solution 958
      (sol958) --------------------------------------------------------------->

<---- Dependent Variables 2 in Oxygen Pulse/Solution 958 (sol958) --------------
Started at May 28, 2023, 4:12:12 PM.
Initial values of variables solved for: Solution 958 (sol958), Solution Store 1 (sol
985).
Values of variables not solved for: Solution 958 (sol958), Solution Store 1 (sol985)
.
Solution time: 0 s.
Physical memory: 1.24 GB
```

Virtual memory: 1.22 GB
Ended at May 28, 2023, 4:12:12 PM.
----- Dependent Variables 2 in Oxygen Pulse/Solution 958 (sol958) ------------->

<---- Time-Dependent Solver 1 in Oxygen Pulse/Solution 958 (sol958) ------------
Started at May 28, 2023, 4:12:12 PM.
Time-dependent solver (BDF)
Number of degrees of freedom solved for: 18385 (plus 36840 internal DOFs).
Symmetric matrices found.
Scales for dependent variables:
Concentration (comp2.Oxi): 0.23

Step	Time	Stepsize	Res	Jac	Sol	Order	Tfail	NLfail	LinErr	LinRes
0	0	- out	5	3	5			0	1e-15	1.4e-15
1	2.6429e-08	2.6429e-08	7	4	7	1	0	0	3.6e-16	1.1e-15
2	5.2858e-08	2.6429e-08	9	5	9	1	0	0	3.9e-16	1.2e-15
3	1.0572e-07	5.2858e-08	11	6	11	2	0	0	3.7e-16	1.3e-15
4	2.1143e-07	1.0572e-07	13	7	13	2	0	0	5e-16	1.7e-15
5	4.2286e-07	2.1143e-07	15	8	15	2	0	0	5e-16	3.5e-15
6	6.3429e-07	2.1143e-07	17	9	17	2	0	0	8.4e-16	3.7e-15
7	8.4572e-07	2.1143e-07	19	10	19	2	0	0	6.6e-16	3.8e-15
8	1.0572e-06	2.1143e-07	21	11	21	2	0	0	6e-16	3.9e-15
9	1.48e-06	4.2286e-07	23	12	23	2	0	0	9e-16	6.2e-15
10	1.9029e-06	4.2286e-07	25	13	25	2	0	0	8.1e-16	6.2e-15
11	2.3257e-06	4.2286e-07	27	14	27	2	0	0	9.5e-16	5.9e-15
12	2.7486e-06	4.2286e-07	29	15	29	2	0	0	8.5e-16	5.5e-15
13	3.5943e-06	8.4572e-07	31	16	31	2	0	0	1.1e-15	8.6e-15
14	4.44e-06	8.4572e-07	33	17	33	2	0	0	1.1e-15	7.6e-15
15	5.2858e-06	8.4572e-07	35	18	35	2	0	0	1.1e-15	7.3e-15
16	6.1315e-06	8.4572e-07	37	19	37	2	0	0	1e-15	6.6e-15
17	7.8229e-06	1.6914e-06	39	20	39	2	0	0	1.3e-15	1e-14
18	9.5144e-06	1.6914e-06	41	21	41	2	0	0	1.6e-15	8.2e-15
19	1.1206e-05	1.6914e-06	43	22	43	2	0	0	1.6e-15	8e-15
20	1.2897e-05	1.6914e-06	45	23	45	2	0	0	1.8e-15	7.3e-15
21	1.4589e-05	1.6914e-06	47	24	47	2	0	0	1.4e-15	6.5e-15
22	1.7972e-05	3.3829e-06	49	25	49	2	0	0	1.2e-15	9.8e-15
23	2.1354e-05	3.3829e-06	51	26	51	2	0	0	2.8e-15	8.4e-15
24	2.4737e-05	3.3829e-06	53	27	53	2	0	0	1.5e-15	8.1e-15
25	2.812e-05	3.3829e-06	55	28	55	2	0	0	1.3e-15	7.9e-15
26	3.4886e-05	6.7658e-06	57	29	57	2	0	0	2.4e-15	1.2e-14
27	4.1652e-05	6.7658e-06	59	30	59	2	0	0	2.5e-15	1e-14
28	4.8418e-05	6.7658e-06	61	31	61	2	0	0	2.6e-15	1e-14
29	5.5183e-05	6.7658e-06	63	32	63	2	0	0	1.5e-15	9.1e-15
30	6.1949e-05	6.7658e-06	65	33	65	2	0	0	1.5e-15	8.2e-15
31	7.5481e-05	1.3532e-05	67	34	67	2	0	0	1.9e-15	1.2e-14
32	8.9012e-05	1.3532e-05	69	35	69	2	0	0	1.3e-15	1.1e-14
33	0.00010254	1.3532e-05	71	36	71	2	0	0	2e-15	1.1e-14
34	0.00011608	1.3532e-05	73	37	73	2	0	0	1.7e-15	9.9e-15
35	0.00014314	2.7063e-05	75	38	75	2	0	0	1.3e-15	1.5e-14
36	0.0001702	2.7063e-05	77	39	77	2	0	0	3.5e-15	1.2e-14
37	0.00019726	2.7063e-05	79	40	79	2	0	0	2.2e-15	1.2e-14
38	0.00022433	2.7063e-05	81	41	81	2	0	0	2.3e-15	1.1e-14
39	0.00027845	5.4126e-05	83	42	83	2	0	0	1.9e-15	1.7e-14
40	0.00033258	5.4126e-05	85	43	85	2	0	0	2.2e-15	1.4e-14
41	0.00038671	5.4126e-05	87	44	87	2	0	0	1.8e-15	1.4e-14
42	0.00049496	0.00010825	89	45	89	2	0	0	3.5e-15	2e-14
43	0.00060321	0.00010825	91	46	91	2	0	0	1.5e-15	1.7e-14

44	0.00071146	0.00010825		93	47	93	2	0	0	2e-15	1.7e-14
45	0.00081971	0.00010825		95	48	95	2	0	0	2.1e-15	1.4e-14
46	0.0010362	0.0002165		97	49	97	2	0	0	2.5e-15	2e-14
47	0.0012527	0.0002165		99	50	99	2	0	0	5.5e-15	1.7e-14
48	0.0016857	0.00043301		101	51	101	2	0	0	5.3e-15	2.6e-14
49	0.0021187	0.00043301		103	52	103	2	0	0	4.7e-15	2.2e-14
50	0.0025518	0.00043301		105	53	105	2	0	0	2.7e-15	2.3e-14
51	0.0029848	0.00043301		107	54	107	2	0	0	6.9e-15	2e-14
52	0.0038508	0.00086602		109	55	109	2	0	0	1.9e-15	2.6e-14
53	0.0047168	0.00086602		111	56	111	2	0	0	1.7e-15	2e-14
-	0.005	-	out								
54	0.0064488	0.001732		113	57	113	2	0	0	3e-15	3.3e-14
55	0.0081809	0.001732		115	58	115	2	0	0	4.6e-15	3.1e-14
-	0.01	-	out								
56	0.011645	0.0034641		117	59	117	2	0	0	7e-15	4.4e-14
-	0.015	-	out								
57	0.015109	0.0034641		119	60	119	1	0	0	4.5e-15	4.8e-14
58	0.018573	0.0034641		121	61	121	1	0	0	2.3e-15	3.7e-14
-	0.02	-	out								
-	0.025	-	out								
59	0.025501	0.0069281		123	62	123	1	0	0	5.2e-15	6.4e-14
-	0.03	-	out								
60	0.032429	0.0069281		125	63	125	1	0	0	6.2e-15	6.2e-14
-	0.035	-	out								
-	0.04	-	out								
-	0.045	-	out								
61	0.046286	0.013856		127	64	127	1	0	0	3.6e-15	8.8e-14
-	0.05	-	out								
-	0.055	-	out								
-	0.06	-	out								
62	0.060142	0.013856		129	65	129	1	0	0	5.1e-15	7.9e-14
-	0.065	-	out								
-	0.07	-	out								
-	0.075	-	out								
-	0.08	-	out								
-	0.085	-	out								
63	0.087855	0.027713		131	66	131	1	0	0	2.5e-15	1e-13
-	0.09	-	out								
-	0.095	-	out								
-	0.1	-	out								
-	0.105	-	out								
-	0.11	-	out								
-	0.115	-	out								
64	0.11557	0.027713		133	67	133	1	0	0	3.9e-15	8.1e-14
-	0.12	-	out								
-	0.125	-	out								
-	0.13	-	out								
-	0.135	-	out								
-	0.14	-	out								
-	0.145	-	out								
-	0.15	-	out								
-	0.155	-	out								
-	0.16	-	out								
-	0.165	-	out								
-	0.17	-	out								
65	0.17099	0.055425		135	68	135	1	0	0	7.9e-15	9e-14

-	0.175	-	out							
-	0.18	-	out							
-	0.185	-	out							
-	0.19	-	out							
-	0.195	-	out							
-	0.2	-	out							
-	0.205	-	out							
-	0.21	-	out							
-	0.215	-	out							
-	0.22	-	out							
-	0.225	-	out							
66	0.22642	0.055425	137	69	137	1	0	0	5.1e-15	6.8e-14
-	0.23	-	out							
-	0.235	-	out							
-	0.24	-	out							
-	0.245	-	out							
-	0.25	-	out							
-	0.25	-	out							
-	0.27	-	out							
-	0.29	-	out							
-	0.31	-	out							
67	0.32542	0.099	139	70	139	1	0	0	2.5e-14	7e-14
-	0.33	-	out							
-	0.35	-	out							
-	0.37	-	out							
-	0.39	-	out							
-	0.41	-	out							
68	0.42442	0.099	141	71	141	1	0	0	7.6e-15	5.3e-14
-	0.43	-	out							
-	0.45	-	out							
-	0.47	-	out							
-	0.49	-	out							
-	0.51	-	out							
69	0.52342	0.099	143	72	143	1	0	0	5.6e-15	4e-14
-	0.53	-	out							
-	0.55	-	out							
-	0.57	-	out							
-	0.59	-	out							
-	0.61	-	out							
70	0.62242	0.099	145	73	145	1	0	0	3.2e-15	3.1e-14
-	0.63	-	out							
-	0.65	-	out							
-	0.67	-	out							
-	0.69	-	out							
-	0.71	-	out							
71	0.72142	0.099	146	74	146	1	0	0	5e-15	2.7e-14
-	0.73	-	out							
-	0.75	-	out							
-	0.77	-	out							
-	0.79	-	out							
-	0.81	-	out							
72	0.82042	0.099	147	75	147	1	0	0	1.9e-14	2.4e-14
-	0.83	-	out							
-	0.85	-	out							
-	0.87	-	out							
-	0.89	-	out							

```
  -        0.91           - out
 73      0.91942       0.099      148   76  148     2     0       0  2.6e-15  1.9e-14
  -        0.93           - out
  -        0.95           - out
  -        0.97           - out
  -        0.99           - out
 74       1.0184       0.099      149   77  149     2     0       0  3.4e-15  1.3e-14
Time-stepping completed.
Solution time: 15 s.
Physical memory: 1.25 GB
Virtual memory: 1.23 GB
Ended at May 28, 2023, 4:12:27 PM.
----- Time-Dependent Solver 1 in Oxygen Pulse/Solution 958 (sol958) ----------->
```

k0Sub=1.51E-8, d=8E-6 (su34)

GENERAL

Description	Value
Solution	k0Sub=1.51E-8, d=8E-6

LOG

```
<---- Compile Equations: Stationary in Oxygen Pulse/Solution 958 (sol958) ------
Started at May 28, 2023, 4:12:30 PM.
Parameter k0Sub = 1.51E-8 (m/s).
Parameter d = 8.0E-6 (m).
Geometry shape function: Linear Lagrange
Number of vertex elements: 10
Minimum element quality: 0.4468
Time: 1 s.
Physical memory: 1.2 GB
Virtual memory: 1.19 GB
Ended at May 28, 2023, 4:12:30 PM.
----- Compile Equations: Stationary in Oxygen Pulse/Solution 958 (sol958) ----->

<---- Dependent Variables 1 in Oxygen Pulse/Solution 958 (sol958) --------------
Started at May 28, 2023, 4:12:30 PM.
Solution time: 0 s.
Physical memory: 1.2 GB
Virtual memory: 1.19 GB
Ended at May 28, 2023, 4:12:30 PM.
----- Dependent Variables 1 in Oxygen Pulse/Solution 958 (sol958) ------------->

<---- Stationary Solver 1 in Oxygen Pulse/Solution 958 (sol958) ----------------
Started at May 28, 2023, 4:12:30 PM.
Linear solver
Number of degrees of freedom solved for: 23800 (plus 941 internal DOFs).
Symmetric matrices found.
Scales for dependent variables:
Concentration (comp2.Oxi): 0.23
Orthonormal null-space function used.
Iter      SolEst     Damping    Stepsize #Res #Jac #Sol   LinErr   LinRes
   1       5e-06   1.0000000       5e-06    1    1    1    3e-13    6e-14
Solution time: 1 s.
Physical memory: 1.22 GB
Virtual memory: 1.2 GB
Ended at May 28, 2023, 4:12:31 PM.
----- Stationary Solver 1 in Oxygen Pulse/Solution 958 (sol958) --------------->

<---- Compile Equations: Oxygen Probe Pulse in Oxygen Pulse/Solution 958
      (sol958) -----------------------------------------------------------------
Started at May 28, 2023, 4:12:31 PM.
Geometry shape function: Linear Lagrange
Time: 0 s.
Physical memory: 1.22 GB
Virtual memory: 1.2 GB
Ended at May 28, 2023, 4:12:31 PM.
----- Compile Equations: Oxygen Probe Pulse in Oxygen Pulse/Solution 958
      (sol958) ---------------------------------------------------------------->

<---- Dependent Variables 2 in Oxygen Pulse/Solution 958 (sol958) --------------
Started at May 28, 2023, 4:12:31 PM.
Initial values of variables solved for: Solution 958 (sol958), Solution Store 1 (sol
985).
Values of variables not solved for: Solution 958 (sol958), Solution Store 1 (sol985)
.
Solution time: 0 s.
Physical memory: 1.22 GB
```

Virtual memory: 1.2 GB
Ended at May 28, 2023, 4:12:31 PM.
----- Dependent Variables 2 in Oxygen Pulse/Solution 958 (sol958) ------------->

<---- Time-Dependent Solver 1 in Oxygen Pulse/Solution 958 (sol958) ------------
Started at May 28, 2023, 4:12:31 PM.
Time-dependent solver (BDF)
Number of degrees of freedom solved for: 23800 (plus 47670 internal DOFs).
Symmetric matrices found.
Scales for dependent variables:
Concentration (comp2.Oxi): 0.23

Step	Time	Stepsize	Res	Jac	Sol	Order	Tfail	NLfail	LinErr	LinRes
0	0	- out	5	3	5			0	6.1e-16	8.3e-16
1	3.6251e-08	3.6251e-08	7	4	7	1	0	0	3.5e-16	9.3e-16
2	7.2501e-08	3.6251e-08	9	5	9	1	0	0	3.4e-16	1e-15
3	1.45e-07	7.2501e-08	11	6	11	2	0	0	3.3e-16	9.6e-16
4	2.9001e-07	1.45e-07	13	7	13	2	0	0	4.6e-16	1.4e-15
5	5.8001e-07	2.9001e-07	15	8	15	2	0	0	5e-16	2.4e-15
6	8.7002e-07	2.9001e-07	17	9	17	2	0	0	5.8e-16	2.8e-15
7	1.16e-06	2.9001e-07	19	10	19	2	0	0	5.8e-16	3.2e-15
8	1.45e-06	2.9001e-07	21	11	21	2	0	0	4.6e-16	3.3e-15
9	2.03e-06	5.8001e-07	23	12	23	2	0	0	6.6e-16	4.9e-15
10	2.6101e-06	5.8001e-07	25	13	25	2	0	0	7.9e-16	4.8e-15
11	3.1901e-06	5.8001e-07	27	14	27	2	0	0	6.5e-16	4.6e-15
12	3.7701e-06	5.8001e-07	29	15	29	2	0	0	7.7e-16	4.5e-15
13	4.9301e-06	1.16e-06	31	16	31	2	0	0	6.6e-16	7.1e-15
14	6.0901e-06	1.16e-06	33	17	33	2	0	0	8e-16	6.2e-15
15	7.2501e-06	1.16e-06	35	18	35	2	0	0	9.3e-16	6e-15
16	8.4102e-06	1.16e-06	37	19	37	2	0	0	8.8e-16	6e-15
17	1.073e-05	2.32e-06	39	20	39	2	0	0	9.9e-16	8.9e-15
18	1.305e-05	2.32e-06	41	21	41	2	0	0	9.2e-16	7.9e-15
19	1.537e-05	2.32e-06	43	22	43	2	0	0	1e-15	7.6e-15
20	1.769e-05	2.32e-06	45	23	45	2	0	0	1e-15	6.9e-15
21	2.233e-05	4.6401e-06	47	24	47	2	0	0	1.1e-15	1.1e-14
22	2.6971e-05	4.6401e-06	49	25	49	2	0	0	2e-15	9.7e-15
23	3.1611e-05	4.6401e-06	51	26	51	2	0	0	1.2e-15	9.5e-15
24	3.6251e-05	4.6401e-06	53	27	53	2	0	0	9.3e-16	8.9e-15
25	4.5531e-05	9.2802e-06	55	28	55	2	0	0	1.2e-15	1.3e-14
26	5.4811e-05	9.2802e-06	57	29	57	2	0	0	2.4e-15	1.1e-14
27	6.4091e-05	9.2802e-06	59	30	59	2	0	0	1.3e-15	1.1e-14
28	7.3371e-05	9.2802e-06	61	31	61	2	0	0	1.3e-15	1.1e-14
29	9.1932e-05	1.856e-05	63	32	63	2	0	0	1.5e-15	1.6e-14
30	0.00011049	1.856e-05	65	33	65	2	0	0	3e-15	1.3e-14
31	0.00012905	1.856e-05	67	34	67	2	0	0	1.6e-15	1.3e-14
32	0.00014761	1.856e-05	69	35	69	2	0	0	2.9e-15	1.2e-14
33	0.00018473	3.7121e-05	71	36	71	2	0	0	1.5e-15	1.8e-14
34	0.00022185	3.7121e-05	73	37	73	2	0	0	4.1e-15	1.6e-14
35	0.00025897	3.7121e-05	75	38	75	2	0	0	2.2e-15	1.6e-14
36	0.0002961	3.7121e-05	77	39	77	2	0	0	2e-15	1.4e-14
37	0.00037034	7.4241e-05	79	40	79	2	0	0	2.7e-15	2.1e-14
38	0.00044458	7.4241e-05	81	41	81	2	0	0	2.5e-15	1.7e-14
39	0.00059306	0.00014848	83	42	83	2	0	0	1.5e-15	2.7e-14
40	0.00074154	0.00014848	85	43	85	2	0	0	4e-15	2.4e-14
41	0.00089003	0.00014848	87	44	87	2	0	0	2.3e-15	2.3e-14
42	0.0010385	0.00014848	89	45	89	2	0	0	2.4e-15	2e-14
43	0.0013355	0.00029697	91	46	91	2	0	0	1.9e-15	2.6e-14

44	0.0016324	0.00029697	93	47	93	2	0	0	3.2e-15	1.9e-14
45	0.0022264	0.00059393	95	48	95	2	0	0	2.9e-15	3.1e-14
46	0.0028203	0.00059393	97	49	97	2	0	0	2.3e-15	2.9e-14
47	0.0034142	0.00059393	99	50	99	2	0	0	6.6e-15	2.9e-14
48	0.0046021	0.0011879	101	51	101	2	0	0	4.9e-15	3.8e-14
-	0.005	-	out							
49	0.00579	0.0011879	103	52	103	2	0	0	3.8e-15	2.7e-14
50	0.0081657	0.0023757	105	53	105	2	0	0	3.8e-15	4.2e-14
-	0.01	-	out							
51	0.010541	0.0023757	107	54	107	1	0	0	4.1e-15	4.9e-14
52	0.012917	0.0023757	109	55	109	1	0	0	3.5e-15	3.3e-14
-	0.015	-	out							
53	0.017669	0.0047515	111	56	111	1	0	0	2.7e-15	5.3e-14
-	0.02	-	out							
-	0.025	-	out							
54	0.027171	0.0095029	113	57	113	1	0	0	4.6e-15	8.6e-14
-	0.03	-	out							
-	0.035	-	out							
55	0.036674	0.0095029	115	58	115	1	0	0	9.2e-15	8.5e-14
-	0.04	-	out							
-	0.045	-	out							
-	0.05	-	out							
-	0.055	-	out							
56	0.05568	0.019006	117	59	117	1	0	0	9.5e-15	1.2e-13
-	0.06	-	out							
-	0.065	-	out							
-	0.07	-	out							
57	0.074686	0.019006	119	60	119	1	0	0	3.6e-15	1.1e-13
-	0.075	-	out							
-	0.08	-	out							
-	0.085	-	out							
-	0.09	-	out							
-	0.095	-	out							
-	0.1	-	out							
-	0.105	-	out							
-	0.11	-	out							
58	0.1127	0.038012	121	61	121	1	0	0	8.3e-15	1.4e-13
-	0.115	-	out							
-	0.12	-	out							
-	0.125	-	out							
-	0.13	-	out							
-	0.135	-	out							
-	0.14	-	out							
-	0.145	-	out							
-	0.15	-	out							
59	0.15071	0.038012	123	62	123	1	0	0	3.4e-15	1.1e-13
-	0.155	-	out							
-	0.16	-	out							
-	0.165	-	out							
-	0.17	-	out							
-	0.175	-	out							
-	0.18	-	out							
-	0.185	-	out							
-	0.19	-	out							
-	0.195	-	out							
-	0.2	-	out							

-	0.205	- out								
-	0.21	- out								
-	0.215	- out								
-	0.22	- out								
-	0.225	- out								
60	0.22673	0.076023	125	63	125	1	0	0	5.7e-15	1.2e-13
-	0.23	- out								
-	0.235	- out								
-	0.24	- out								
-	0.245	- out								
-	0.25	- out								
-	0.25	- out								
-	0.27	- out								
-	0.29	- out								
61	0.30276	0.076023	127	64	127	1	0	0	4.6e-15	9.6e-14
-	0.31	- out								
-	0.33	- out								
-	0.35	- out								
-	0.37	- out								
-	0.39	- out								
62	0.40176	0.099	129	65	129	1	0	0	4.4e-15	8.2e-14
-	0.41	- out								
-	0.43	- out								
-	0.45	- out								
-	0.47	- out								
-	0.49	- out								
63	0.50076	0.099	131	66	131	1	0	0	4.5e-15	6.3e-14
-	0.51	- out								
-	0.53	- out								
-	0.55	- out								
-	0.57	- out								
-	0.59	- out								
64	0.59976	0.099	133	67	133	1	0	0	1.2e-14	4.8e-14
-	0.61	- out								
-	0.63	- out								
-	0.65	- out								
-	0.67	- out								
-	0.69	- out								
65	0.69876	0.099	134	68	134	1	0	0	6.8e-15	4.1e-14
-	0.71	- out								
-	0.73	- out								
-	0.75	- out								
-	0.77	- out								
-	0.79	- out								
66	0.79776	0.099	135	69	135	1	0	0	5.1e-15	3.5e-14
-	0.81	- out								
-	0.83	- out								
-	0.85	- out								
-	0.87	- out								
-	0.89	- out								
67	0.89676	0.099	136	70	136	2	0	0	4.2e-15	3e-14
-	0.91	- out								
-	0.93	- out								
-	0.95	- out								
-	0.97	- out								
-	0.99	- out								

```
  68      0.99576        0.099      137    71   137      2      0       0  1.2e-14    2e-14
Time-stepping completed.
Solution time: 18 s.
Physical memory: 1.25 GB
Virtual memory: 1.24 GB
Ended at May 28, 2023, 4:12:49 PM.
----- Time-Dependent Solver 1 in Oxygen Pulse/Solution 958 (sol958) ----------->
```

k0Sub=1.51E-8, d=1.2E-5 (su35)

GENERAL

Description	Value
Solution	k0Sub=1.51E-8, d=1.2E-5

LOG

```
<---- Compile Equations: Stationary in Oxygen Pulse/Solution 958 (sol958) ------
Started at May 28, 2023, 4:12:52 PM.
Parameter k0Sub = 1.51E-8 (m/s).
Parameter d = 1.2E-5 (m).
Geometry shape function: Linear Lagrange
Number of vertex elements: 10
Minimum element quality: 0.4505
Time: 1 s.
Physical memory: 1.23 GB
Virtual memory: 1.21 GB
Ended at May 28, 2023, 4:12:53 PM.
----- Compile Equations: Stationary in Oxygen Pulse/Solution 958 (sol958) ----->

<---- Dependent Variables 1 in Oxygen Pulse/Solution 958 (sol958) --------------
Started at May 28, 2023, 4:12:53 PM.
Solution time: 0 s.
Physical memory: 1.23 GB
Virtual memory: 1.21 GB
Ended at May 28, 2023, 4:12:53 PM.
----- Dependent Variables 1 in Oxygen Pulse/Solution 958 (sol958) ------------->

<---- Stationary Solver 1 in Oxygen Pulse/Solution 958 (sol958) ----------------
Started at May 28, 2023, 4:12:53 PM.
Linear solver
Number of degrees of freedom solved for: 18769 (plus 948 internal DOFs).
Symmetric matrices found.
Scales for dependent variables:
Concentration (comp2.Oxi): 0.23
Orthonormal null-space function used.
Iter      SolEst     Damping    Stepsize #Res #Jac #Sol   LinErr   LinRes
   1     4.3e-06   1.0000000     4.3e-06    1    1    1  2.4e-13    5e-14
Solution time: 1 s.
Physical memory: 1.24 GB
Virtual memory: 1.22 GB
Ended at May 28, 2023, 4:12:53 PM.
----- Stationary Solver 1 in Oxygen Pulse/Solution 958 (sol958) --------------->

<---- Compile Equations: Oxygen Probe Pulse in Oxygen Pulse/Solution 958
      (sol958) -----------------------------------------------------------------
Started at May 28, 2023, 4:12:53 PM.
Geometry shape function: Linear Lagrange
Time: 0 s.
Physical memory: 1.24 GB
Virtual memory: 1.22 GB
Ended at May 28, 2023, 4:12:54 PM.
----- Compile Equations: Oxygen Probe Pulse in Oxygen Pulse/Solution 958
      (sol958) ---------------------------------------------------------------->

<---- Dependent Variables 2 in Oxygen Pulse/Solution 958 (sol958) --------------
Started at May 28, 2023, 4:12:54 PM.
Initial values of variables solved for: Solution 958 (sol958), Solution Store 1 (sol
985).
Values of variables not solved for: Solution 958 (sol958), Solution Store 1 (sol985)
.
Solution time: 0 s.
Physical memory: 1.24 GB
```

Virtual memory: 1.22 GB
Ended at May 28, 2023, 4:12:54 PM.
----- Dependent Variables 2 in Oxygen Pulse/Solution 958 (sol958) ------------->

<---- Time-Dependent Solver 1 in Oxygen Pulse/Solution 958 (sol958) ------------
Started at May 28, 2023, 4:12:54 PM.
Time-dependent solver (BDF)
Number of degrees of freedom solved for: 18769 (plus 37608 internal DOFs).
Symmetric matrices found.
Scales for dependent variables:
Concentration (comp2.Oxi): 0.23

Step	Time	Stepsize		Res	Jac	Sol	Order	Tfail	NLfail	LinErr	LinRes
0	0	-	out	5	3	5			0	8.2e-16	1.4e-15
1	2.659e-08	2.659e-08		7	4	7	1	0	0	3.3e-16	1e-15
2	5.3179e-08	2.659e-08		9	5	9	1	0	0	3.4e-16	1.1e-15
3	1.0636e-07	5.3179e-08		11	6	11	2	0	0	3.7e-16	1.3e-15
4	2.1272e-07	1.0636e-07		13	7	13	2	0	0	4.9e-16	1.7e-15
5	4.2544e-07	2.1272e-07		15	8	15	2	0	0	6e-16	3.3e-15
6	6.3815e-07	2.1272e-07		17	9	17	2	0	0	6.2e-16	3.7e-15
7	8.5087e-07	2.1272e-07		19	10	19	2	0	0	5.7e-16	3.9e-15
8	1.0636e-06	2.1272e-07		21	11	21	2	0	0	6.7e-16	4e-15
9	1.489e-06	4.2544e-07		23	12	23	2	0	0	8.8e-16	6.4e-15
10	1.9145e-06	4.2544e-07		25	13	25	2	0	0	7.3e-16	5.9e-15
11	2.3399e-06	4.2544e-07		27	14	27	2	0	0	7.2e-16	5.9e-15
12	2.7653e-06	4.2544e-07		29	15	29	2	0	0	8.2e-16	5.7e-15
13	3.6162e-06	8.5087e-07		31	16	31	2	0	0	9.9e-16	8.6e-15
14	4.4671e-06	8.5087e-07		33	17	33	2	0	0	1.2e-15	7.1e-15
15	5.3179e-06	8.5087e-07		35	18	35	2	0	0	1.1e-15	7.3e-15
16	6.1688e-06	8.5087e-07		37	19	37	2	0	0	9.6e-16	6.4e-15
17	7.8706e-06	1.7017e-06		39	20	39	2	0	0	1.1e-15	9.4e-15
18	9.5723e-06	1.7017e-06		41	21	41	2	0	0	1.3e-15	8.1e-15
19	1.1274e-05	1.7017e-06		43	22	43	2	0	0	1.3e-15	8e-15
20	1.2976e-05	1.7017e-06		45	23	45	2	0	0	1.1e-15	7.2e-15
21	1.4678e-05	1.7017e-06		47	24	47	2	0	0	1e-15	6.7e-15
22	1.8081e-05	3.4035e-06		49	25	49	2	0	0	1.6e-15	9.9e-15
23	2.1484e-05	3.4035e-06		51	26	51	2	0	0	1.1e-15	8.3e-15
24	2.4888e-05	3.4035e-06		53	27	53	2	0	0	1.5e-15	8.4e-15
25	2.8291e-05	3.4035e-06		55	28	55	2	0	0	1.3e-15	7.6e-15
26	3.5098e-05	6.807e-06		57	29	57	2	0	0	2.3e-15	1.1e-14
27	4.1905e-05	6.807e-06		59	30	59	2	0	0	1.7e-15	9.7e-15
28	4.8712e-05	6.807e-06		61	31	61	2	0	0	2.1e-15	9.5e-15
29	5.5519e-05	6.807e-06		63	32	63	2	0	0	1.6e-15	8.6e-15
30	6.2326e-05	6.807e-06		65	33	65	2	0	0	1.6e-15	7.7e-15
31	7.594e-05	1.3614e-05		67	34	67	2	0	0	2.7e-15	1.2e-14
32	8.9554e-05	1.3614e-05		69	35	69	2	0	0	1.8e-15	1.1e-14
33	0.00010317	1.3614e-05		71	36	71	2	0	0	3.1e-15	1e-14
34	0.00011678	1.3614e-05		73	37	73	2	0	0	1.3e-15	9.7e-15
35	0.00014401	2.7228e-05		75	38	75	2	0	0	1.5e-15	1.4e-14
36	0.00017124	2.7228e-05		77	39	77	2	0	0	2.9e-15	1.2e-14
37	0.00019847	2.7228e-05		79	40	79	2	0	0	2.7e-15	1.2e-14
38	0.00022569	2.7228e-05		81	41	81	2	0	0	2.6e-15	1.1e-14
39	0.00028015	5.4456e-05		83	42	83	2	0	0	1.8e-15	1.6e-14
40	0.0003346	5.4456e-05		85	43	85	2	0	0	1.7e-15	1.3e-14
41	0.00038906	5.4456e-05		87	44	87	2	0	0	2.6e-15	1.4e-14
42	0.00049797	0.00010891		89	45	89	2	0	0	4.1e-15	2e-14
43	0.00060688	0.00010891		91	46	91	2	0	0	2.4e-15	1.7e-14

44	0.00071579	0.00010891		93	47	93	2	0	0	3.7e-15	1.6e-14
45	0.00082471	0.00010891		95	48	95	2	0	0	6.1e-15	1.4e-14
46	0.0010425	0.00021782		97	49	97	2	0	0	2.5e-15	2e-14
47	0.0012604	0.00021782		99	50	99	2	0	0	4e-15	1.7e-14
48	0.001696	0.00043565		101	51	101	2	0	0	3.1e-15	2.7e-14
49	0.0021316	0.00043565		103	52	103	2	0	0	2.1e-15	2.3e-14
50	0.0025673	0.00043565		105	53	105	2	0	0	3.4e-15	2.3e-14
51	0.0030029	0.00043565		107	54	107	2	0	0	3.7e-15	2e-14
52	0.0038742	0.00087129		109	55	109	2	0	0	4e-15	2.5e-14
53	0.0047455	0.00087129		111	56	111	2	0	0	2.9e-15	1.8e-14
-	0.005	-	out								
54	0.0064881	0.0017426		113	57	113	2	0	0	2.5e-15	3e-14
55	0.0082307	0.0017426		115	58	115	2	0	0	3.3e-15	2.5e-14
-	0.01	-	out								
56	0.011716	0.0034852		117	59	117	2	0	0	4.5e-15	4e-14
-	0.015	-	out								
57	0.015201	0.0034852		119	60	119	1	0	0	6.1e-15	4.4e-14
58	0.018686	0.0034852		121	61	121	1	0	0	3.1e-15	2.6e-14
-	0.02	-	out								
-	0.025	-	out								
59	0.025657	0.0069703		123	62	123	1	0	0	4.5e-15	3.8e-14
-	0.03	-	out								
-	0.035	-	out								
60	0.039597	0.013941		125	63	125	1	0	0	3.1e-15	6.1e-14
-	0.04	-	out								
-	0.045	-	out								
-	0.05	-	out								
61	0.053538	0.013941		127	64	127	1	0	0	6.5e-15	6.2e-14
-	0.055	-	out								
-	0.06	-	out								
-	0.065	-	out								
-	0.07	-	out								
-	0.075	-	out								
-	0.08	-	out								
62	0.081419	0.027881		129	65	129	1	0	0	4e-15	8.6e-14
-	0.085	-	out								
-	0.09	-	out								
-	0.095	-	out								
-	0.1	-	out								
-	0.105	-	out								
63	0.1093	0.027881		131	66	131	1	0	0	8.6e-15	7.5e-14
-	0.11	-	out								
-	0.115	-	out								
-	0.12	-	out								
-	0.125	-	out								
-	0.13	-	out								
-	0.135	-	out								
-	0.14	-	out								
-	0.145	-	out								
-	0.15	-	out								
-	0.155	-	out								
-	0.16	-	out								
-	0.165	-	out								
64	0.16506	0.055763		133	67	133	1	0	0	5e-15	9.8e-14
-	0.17	-	out								
-	0.175	-	out								

-	0.18	-	out							
-	0.185	-	out							
-	0.19	-	out							
-	0.195	-	out							
-	0.2	-	out							
-	0.205	-	out							
-	0.21	-	out							
-	0.215	-	out							
-	0.22	-	out							
65	0.22083	0.055763	135	68	135	1	0	0	4.1e-15	7.5e-14
-	0.225	-	out							
-	0.23	-	out							
-	0.235	-	out							
-	0.24	-	out							
-	0.245	-	out							
-	0.25	-	out							
-	0.25	-	out							
-	0.27	-	out							
-	0.29	-	out							
-	0.31	-	out							
66	0.31983	0.099	137	69	137	1	0	0	1.5e-14	7.9e-14
-	0.33	-	out							
-	0.35	-	out							
-	0.37	-	out							
-	0.39	-	out							
-	0.41	-	out							
67	0.41883	0.099	139	70	139	1	0	0	1.3e-14	6.1e-14
-	0.43	-	out							
-	0.45	-	out							
-	0.47	-	out							
-	0.49	-	out							
-	0.51	-	out							
68	0.51783	0.099	141	71	141	1	0	0	5.1e-15	4.8e-14
-	0.53	-	out							
-	0.55	-	out							
-	0.57	-	out							
-	0.59	-	out							
-	0.61	-	out							
69	0.61683	0.099	143	72	143	1	0	0	8e-15	3.8e-14
-	0.63	-	out							
-	0.65	-	out							
-	0.67	-	out							
-	0.69	-	out							
-	0.71	-	out							
70	0.71583	0.099	144	73	144	1	0	0	5.2e-15	3.1e-14
-	0.73	-	out							
-	0.75	-	out							
-	0.77	-	out							
-	0.79	-	out							
-	0.81	-	out							
71	0.81483	0.099	145	74	145	1	0	0	4.9e-15	2.7e-14
-	0.83	-	out							
-	0.85	-	out							
-	0.87	-	out							
-	0.89	-	out							
-	0.91	-	out							

```
  72     0.91383       0.099      146   75  146     2     0      0  8.3e-15  2.3e-14
   -        0.93           - out
   -        0.95           - out
   -        0.97           - out
   -        0.99           - out
  73      1.0128       0.099      147   76  147     2     0      0    9e-15  1.4e-14
Time-stepping completed.
Solution time: 15 s.
Physical memory: 1.26 GB
Virtual memory: 1.25 GB
Ended at May 28, 2023, 4:13:09 PM.
----- Time-Dependent Solver 1 in Oxygen Pulse/Solution 958 (sol958) ----------->
```

k0Sub=1.51E-8, d=1.5E-5 (su36)

GENERAL

Description	Value
Solution	k0Sub=1.51E-8, d=1.5E-5

LOG

```
<---- Compile Equations: Stationary in Oxygen Pulse/Solution 958 (sol958) ------
Started at May 28, 2023, 4:13:12 PM.
Parameter k0Sub = 1.51E-8 (m/s).
Parameter d = 1.4999999999999999E-5 (m).
Geometry shape function: Linear Lagrange
Number of vertex elements: 10
Minimum element quality: 0.4444
Time: 1 s.
Physical memory: 1.16 GB
Virtual memory: 1.14 GB
Ended at May 28, 2023, 4:13:13 PM.
----- Compile Equations: Stationary in Oxygen Pulse/Solution 958 (sol958) ----->

<---- Dependent Variables 1 in Oxygen Pulse/Solution 958 (sol958) --------------
Started at May 28, 2023, 4:13:13 PM.
Solution time: 0 s.
Physical memory: 1.16 GB
Virtual memory: 1.14 GB
Ended at May 28, 2023, 4:13:13 PM.
----- Dependent Variables 1 in Oxygen Pulse/Solution 958 (sol958) ------------->

<---- Stationary Solver 1 in Oxygen Pulse/Solution 958 (sol958) ----------------
Started at May 28, 2023, 4:13:13 PM.
Linear solver
Number of degrees of freedom solved for: 18918 (plus 952 internal DOFs).
Symmetric matrices found.
Scales for dependent variables:
Concentration (comp2.Oxi): 0.23
Orthonormal null-space function used.
Iter      SolEst     Damping    Stepsize #Res #Jac #Sol   LinErr   LinRes
   1     3.8e-06   1.0000000     3.8e-06    1    1    1  1.9e-13  4.7e-14
Solution time: 1 s.
Physical memory: 1.19 GB
Virtual memory: 1.17 GB
Ended at May 28, 2023, 4:13:13 PM.
----- Stationary Solver 1 in Oxygen Pulse/Solution 958 (sol958) --------------->

<---- Compile Equations: Oxygen Probe Pulse in Oxygen Pulse/Solution 958
      (sol958) -----------------------------------------------------------------
Started at May 28, 2023, 4:13:13 PM.
Geometry shape function: Linear Lagrange
Time: 0 s.
Physical memory: 1.2 GB
Virtual memory: 1.17 GB
Ended at May 28, 2023, 4:13:14 PM.
----- Compile Equations: Oxygen Probe Pulse in Oxygen Pulse/Solution 958
      (sol958) ---------------------------------------------------------------->

<---- Dependent Variables 2 in Oxygen Pulse/Solution 958 (sol958) --------------
Started at May 28, 2023, 4:13:14 PM.
Initial values of variables solved for: Solution 958 (sol958), Solution Store 1 (sol
985).
Values of variables not solved for: Solution 958 (sol958), Solution Store 1 (sol985)
.
Solution time: 0 s.
Physical memory: 1.2 GB
```

Virtual memory: 1.18 GB
Ended at May 28, 2023, 4:13:14 PM.
----- Dependent Variables 2 in Oxygen Pulse/Solution 958 (sol958) ------------->

<---- Time-Dependent Solver 1 in Oxygen Pulse/Solution 958 (sol958) ------------
Started at May 28, 2023, 4:13:14 PM.
Time-dependent solver (BDF)
Number of degrees of freedom solved for: 18918 (plus 37906 internal DOFs).
Symmetric matrices found.
Scales for dependent variables:
Concentration (comp2.Oxi): 0.23

Step	Time	Stepsize	Res	Jac	Sol	Order	Tfail	NLfail	LinErr	LinRes
0	0	- out	5	3	5			0	6.7e-16	1.4e-15
1	2.6779e-08	2.6779e-08	7	4	7	1	0	0	3.7e-16	1.1e-15
2	5.3558e-08	2.6779e-08	9	5	9	1	0	0	3.8e-16	1.2e-15
3	1.0712e-07	5.3558e-08	11	6	11	2	0	0	3.8e-16	1.3e-15
4	2.1423e-07	1.0712e-07	13	7	13	2	0	0	5.1e-16	1.8e-15
5	4.2847e-07	2.1423e-07	15	8	15	2	0	0	5.3e-16	3.4e-15
6	6.427e-07	2.1423e-07	17	9	17	2	0	0	5.6e-16	3.8e-15
7	8.5694e-07	2.1423e-07	19	10	19	2	0	0	5.5e-16	3.9e-15
8	1.0712e-06	2.1423e-07	21	11	21	2	0	0	6.4e-16	4e-15
9	1.4996e-06	4.2847e-07	23	12	23	2	0	0	8e-16	6.5e-15
10	1.9281e-06	4.2847e-07	25	13	25	2	0	0	8.8e-16	6.3e-15
11	2.3566e-06	4.2847e-07	27	14	27	2	0	0	9.4e-16	5.9e-15
12	2.785e-06	4.2847e-07	29	15	29	2	0	0	8.5e-16	5.7e-15
13	3.642e-06	8.5694e-07	31	16	31	2	0	0	1.2e-15	8.6e-15
14	4.4989e-06	8.5694e-07	33	17	33	2	0	0	1.1e-15	7.6e-15
15	5.3558e-06	8.5694e-07	35	18	35	2	0	0	9.8e-16	7.4e-15
16	6.2128e-06	8.5694e-07	37	19	37	2	0	0	1.2e-15	6.8e-15
17	7.9267e-06	1.7139e-06	39	20	39	2	0	0	1.4e-15	9.8e-15
18	9.6405e-06	1.7139e-06	41	21	41	2	0	0	1.7e-15	8.5e-15
19	1.1354e-05	1.7139e-06	43	22	43	2	0	0	1e-15	8.2e-15
20	1.3068e-05	1.7139e-06	45	23	45	2	0	0	1.8e-15	7.4e-15
21	1.4782e-05	1.7139e-06	47	24	47	2	0	0	1.4e-15	6.8e-15
22	1.821e-05	3.4277e-06	49	25	49	2	0	0	1.3e-15	9.8e-15
23	2.1638e-05	3.4277e-06	51	26	51	2	0	0	1.6e-15	8.7e-15
24	2.5065e-05	3.4277e-06	53	27	53	2	0	0	1.8e-15	8.7e-15
25	2.8493e-05	3.4277e-06	55	28	55	2	0	0	1.9e-15	8e-15
26	3.5349e-05	6.8555e-06	57	29	57	2	0	0	1.9e-15	1.2e-14
27	4.2204e-05	6.8555e-06	59	30	59	2	0	0	2e-15	1e-14
28	4.906e-05	6.8555e-06	61	31	61	2	0	0	1.9e-15	9.9e-15
29	5.5915e-05	6.8555e-06	63	32	63	2	0	0	1.9e-15	8.9e-15
30	6.2771e-05	6.8555e-06	65	33	65	2	0	0	1.7e-15	8.1e-15
31	7.6481e-05	1.3711e-05	67	34	67	2	0	0	1.9e-15	1.2e-14
32	9.0192e-05	1.3711e-05	69	35	69	2	0	0	1.5e-15	1.1e-14
33	0.0001039	1.3711e-05	71	36	71	2	0	0	1.8e-15	1.1e-14
34	0.00011761	1.3711e-05	73	37	73	2	0	0	2.3e-15	9.5e-15
35	0.00014504	2.7422e-05	75	38	75	2	0	0	2.6e-15	1.4e-14
36	0.00017246	2.7422e-05	77	39	77	2	0	0	1.7e-15	1.2e-14
37	0.00019988	2.7422e-05	79	40	79	2	0	0	1.9e-15	1.2e-14
38	0.0002273	2.7422e-05	81	41	81	2	0	0	2.5e-15	1.1e-14
39	0.00028215	5.4844e-05	83	42	83	2	0	0	2e-15	1.5e-14
40	0.00033699	5.4844e-05	85	43	85	2	0	0	1.4e-15	1.4e-14
41	0.00039183	5.4844e-05	87	44	87	2	0	0	2.2e-15	1.4e-14
42	0.00050152	0.00010969	89	45	89	2	0	0	4.7e-15	2.1e-14
43	0.00061121	0.00010969	91	46	91	2	0	0	2.3e-15	1.7e-14

44	0.0007209	0.00010969	93	47	93	2	0	0	2.3e-15	1.7e-14
45	0.00083058	0.00010969	95	48	95	2	0	0	1.9e-15	1.5e-14
46	0.00105	0.00021938	97	49	97	2	0	0	1.2e-14	2.1e-14
47	0.0012693	0.00021938	99	50	99	2	0	0	4.5e-15	1.6e-14
48	0.0017081	0.00043875	101	51	101	2	0	0	2.2e-15	2.6e-14
49	0.0021468	0.00043875	103	52	103	2	0	0	1.8e-15	2.3e-14
50	0.0025856	0.00043875	105	53	105	2	0	0	3.9e-15	2.2e-14
51	0.0030243	0.00043875	107	54	107	2	0	0	3.1e-15	1.9e-14
52	0.0039018	0.0008775	109	55	109	2	0	0	2.7e-15	2.4e-14
53	0.0047793	0.0008775	111	56	111	2	0	0	4e-15	1.9e-14
-	0.005	- out								
54	0.0065343	0.001755	113	57	113	2	0	0	5.3e-15	2.9e-14
55	0.0082894	0.001755	115	58	115	2	0	0	2.5e-15	2.5e-14
-	0.01	- out								
56	0.011799	0.00351	117	59	117	2	0	0	3.3e-15	3.8e-14
-	0.015	- out								
57	0.015309	0.00351	119	60	119	1	0	0	7.6e-15	3.9e-14
58	0.018819	0.00351	121	61	121	1	0	0	2.7e-15	2.3e-14
-	0.02	- out								
-	0.025	- out								
59	0.025839	0.00702	123	62	123	1	0	0	3.2e-15	3.6e-14
-	0.03	- out								
-	0.035	- out								
60	0.039879	0.01404	125	63	125	1	0	0	2.8e-15	5.3e-14
-	0.04	- out								
-	0.045	- out								
-	0.05	- out								
61	0.053919	0.01404	127	64	127	1	0	0	5e-15	5.2e-14
-	0.055	- out								
-	0.06	- out								
-	0.065	- out								
-	0.07	- out								
-	0.075	- out								
-	0.08	- out								
62	0.082	0.02808	129	65	129	1	0	0	6.5e-15	7.2e-14
-	0.085	- out								
-	0.09	- out								
-	0.095	- out								
-	0.1	- out								
-	0.105	- out								
-	0.11	- out								
63	0.11008	0.02808	131	66	131	1	0	0	6.1e-15	6.6e-14
-	0.115	- out								
-	0.12	- out								
-	0.125	- out								
-	0.13	- out								
-	0.135	- out								
-	0.14	- out								
-	0.145	- out								
-	0.15	- out								
-	0.155	- out								
-	0.16	- out								
-	0.165	- out								
64	0.16624	0.05616	133	67	133	1	0	0	2.9e-15	8.7e-14
-	0.17	- out								
-	0.175	- out								

-	0.18	- out								
-	0.185	- out								
-	0.19	- out								
-	0.195	- out								
-	0.2	- out								
-	0.205	- out								
-	0.21	- out								
-	0.215	- out								
-	0.22	- out								
65	0.2224	0.05616	135	68	135	1	0	0	5.6e-15	7.1e-14
-	0.225	- out								
-	0.23	- out								
-	0.235	- out								
-	0.24	- out								
-	0.245	- out								
-	0.25	- out								
-	0.25	- out								
-	0.27	- out								
-	0.29	- out								
-	0.31	- out								
66	0.3214	0.099	137	69	137	1	0	0	5.4e-15	7.9e-14
-	0.33	- out								
-	0.35	- out								
-	0.37	- out								
-	0.39	- out								
-	0.41	- out								
67	0.4204	0.099	139	70	139	1	0	0	1.1e-14	6.2e-14
-	0.43	- out								
-	0.45	- out								
-	0.47	- out								
-	0.49	- out								
-	0.51	- out								
68	0.5194	0.099	141	71	141	1	0	0	7.4e-15	4.9e-14
-	0.53	- out								
-	0.55	- out								
-	0.57	- out								
-	0.59	- out								
-	0.61	- out								
69	0.6184	0.099	143	72	143	1	0	0	4.3e-15	3.9e-14
-	0.63	- out								
-	0.65	- out								
-	0.67	- out								
-	0.69	- out								
-	0.71	- out								
70	0.7174	0.099	144	73	144	1	0	0	9.9e-15	3.2e-14
-	0.73	- out								
-	0.75	- out								
-	0.77	- out								
-	0.79	- out								
-	0.81	- out								
71	0.8164	0.099	145	74	145	1	0	0	3.9e-15	2.8e-14
-	0.83	- out								
-	0.85	- out								
-	0.87	- out								
-	0.89	- out								
-	0.91	- out								

```
  72       0.9154       0.099      146   75  146     2     0      0  3.7e-15  2.3e-14
   -         0.93           - out
   -         0.95           - out
   -         0.97           - out
   -         0.99           - out
  73       1.0144       0.099      147   76  147     2     0      0  2.1e-15  1.5e-14
Time-stepping completed.
Solution time: 16 s.
Physical memory: 1.25 GB
Virtual memory: 1.24 GB
Ended at May 28, 2023, 4:13:29 PM.
----- Time-Dependent Solver 1 in Oxygen Pulse/Solution 958 (sol958) ----------->
```

k0Sub=1.51E-7, d=5E-7 (su37)

GENERAL

Description	Value
Solution	k0Sub=1.51E-7, d=5E-7

LOG

```
<---- Compile Equations: Stationary in Oxygen Pulse/Solution 958 (sol958) ------
Started at May 28, 2023, 4:13:32 PM.
Parameter k0Sub = 1.51E-7 (m/s).
Parameter d = 5.0E-7 (m).
Geometry shape function: Linear Lagrange
Number of vertex elements: 10
Minimum element quality: 0.3539
Time: 1 s.
Physical memory: 1.23 GB
Virtual memory: 1.21 GB
Ended at May 28, 2023, 4:13:33 PM.
----- Compile Equations: Stationary in Oxygen Pulse/Solution 958 (sol958) ----->

<---- Dependent Variables 1 in Oxygen Pulse/Solution 958 (sol958) --------------
Started at May 28, 2023, 4:13:33 PM.
Solution time: 0 s.
Physical memory: 1.23 GB
Virtual memory: 1.21 GB
Ended at May 28, 2023, 4:13:33 PM.
----- Dependent Variables 1 in Oxygen Pulse/Solution 958 (sol958) ------------->

<---- Stationary Solver 1 in Oxygen Pulse/Solution 958 (sol958) ----------------
Started at May 28, 2023, 4:13:33 PM.
Linear solver
Number of degrees of freedom solved for: 17710 (plus 1007 internal DOFs).
Symmetric matrices found.
Scales for dependent variables:
Concentration (comp2.Oxi): 0.23
Orthonormal null-space function used.
Iter      SolEst     Damping    Stepsize #Res #Jac #Sol   LinErr   LinRes
   1     0.00021   1.0000000     0.00021    1    1    1  5.6e-13  1.5e-13
Solution time: 1 s.
Physical memory: 1.23 GB
Virtual memory: 1.22 GB
Ended at May 28, 2023, 4:13:33 PM.
----- Stationary Solver 1 in Oxygen Pulse/Solution 958 (sol958) --------------->

<---- Compile Equations: Oxygen Probe Pulse in Oxygen Pulse/Solution 958
      (sol958) ----------------------------------------------------------------
Started at May 28, 2023, 4:13:33 PM.
Geometry shape function: Linear Lagrange
Time: 0 s.
Physical memory: 1.24 GB
Virtual memory: 1.22 GB
Ended at May 28, 2023, 4:13:34 PM.
----- Compile Equations: Oxygen Probe Pulse in Oxygen Pulse/Solution 958
      (sol958) --------------------------------------------------------------->

<---- Dependent Variables 2 in Oxygen Pulse/Solution 958 (sol958) --------------
Started at May 28, 2023, 4:13:34 PM.
Initial values of variables solved for: Solution 958 (sol958), Solution Store 1 (sol
985).
Values of variables not solved for: Solution 958 (sol958), Solution Store 1 (sol985)
.
Solution time: 0 s.
Physical memory: 1.24 GB
```

```
Virtual memory: 1.22 GB
Ended at May 28, 2023, 4:13:34 PM.
----- Dependent Variables 2 in Oxygen Pulse/Solution 958 (sol958) ------------->

<---- Time-Dependent Solver 1 in Oxygen Pulse/Solution 958 (sol958) ------------
Started at May 28, 2023, 4:13:34 PM.
Time-dependent solver (BDF)
Number of degrees of freedom solved for: 17710 (plus 35580 internal DOFs).
Symmetric matrices found.
Scales for dependent variables:
Concentration (comp2.Oxi): 0.23
Step         Time    Stepsize      Res  Jac  Sol Order Tfail NLfail   LinErr   LinRes
   0            0          - out    5    3    5                   0  7.5e-16  1.5e-15
   1  2.5607e-08  2.5607e-08        7    4    7     1     0       0  4.1e-16  1.1e-15
   2  5.1214e-08  2.5607e-08        9    5    9     1     0       0  3.6e-16  1.2e-15
   3  1.0243e-07  5.1214e-08       11    6   11     2     0       0  3.7e-16  1.3e-15
   4  2.0486e-07  1.0243e-07       13    7   13     2     0       0  4.7e-16  1.7e-15
   5  4.0971e-07  2.0486e-07       15    8   15     2     0       0  5.8e-16  3.4e-15
   6  6.1457e-07  2.0486e-07       17    9   17     2     0       0  6.3e-16  3.9e-15
   7  8.1942e-07  2.0486e-07       19   10   19     2     0       0  6.1e-16  3.9e-15
   8  1.0243e-06  2.0486e-07       21   11   21     2     0       0  7.7e-16  3.9e-15
   9   1.434e-06  4.0971e-07       23   12   23     2     0       0  7.8e-16  5.9e-15
  10  1.8437e-06  4.0971e-07       25   13   25     2     0       0  6.4e-16  5.4e-15
  11  2.2534e-06  4.0971e-07       27   14   27     2     0       0  6.8e-16  5.4e-15
  12  2.6631e-06  4.0971e-07       29   15   29     2     0       0  7.9e-16  5.3e-15
  13  3.4825e-06  8.1942e-07       31   16   31     2     0       0  8.8e-16  7.8e-15
  14   4.302e-06  8.1942e-07       33   17   33     2     0       0  9.3e-16  6.6e-15
  15  5.1214e-06  8.1942e-07       35   18   35     2     0       0  1.3e-15  6.5e-15
  16  5.9408e-06  8.1942e-07       37   19   37     2     0       0  9.6e-16  5.9e-15
  17  7.5796e-06  1.6388e-06       39   20   39     2     0       0  1.2e-15  8.6e-15
  18  9.2185e-06  1.6388e-06       41   21   41     2     0       0  1.3e-15  7.9e-15
  19  1.0857e-05  1.6388e-06       43   22   43     2     0       0  1.2e-15  7.6e-15
  20  1.2496e-05  1.6388e-06       45   23   45     2     0       0  1.3e-15  7.1e-15
  21  1.4135e-05  1.6388e-06       47   24   47     2     0       0  1.2e-15  6.5e-15
  22  1.7413e-05  3.2777e-06       49   25   49     2     0       0    2e-15  9.6e-15
  23   2.069e-05  3.2777e-06       51   26   51     2     0       0  2.4e-15  8.6e-15
  24  2.3968e-05  3.2777e-06       53   27   53     2     0       0  1.7e-15  8.5e-15
  25  2.7246e-05  3.2777e-06       55   28   55     2     0       0  1.7e-15  7.7e-15
  26  3.3801e-05  6.5554e-06       57   29   57     2     0       0  1.6e-15  1.2e-14
  27  4.0356e-05  6.5554e-06       59   30   59     2     0       0  2.4e-15    1e-14
  28  4.6912e-05  6.5554e-06       61   31   61     2     0       0  1.5e-15  9.7e-15
  29  5.3467e-05  6.5554e-06       63   32   63     2     0       0    2e-15    9e-15
  30  6.0023e-05  6.5554e-06       65   33   65     2     0       0  2.2e-15  8.3e-15
  31  7.3133e-05  1.3111e-05       67   34   67     2     0       0  3.1e-15  1.2e-14
  32  8.6244e-05  1.3111e-05       69   35   69     2     0       0  1.7e-15    1e-14
  33  9.9355e-05  1.3111e-05       71   36   71     2     0       0  1.7e-15  1.1e-14
  34  0.00011247  1.3111e-05       73   37   73     2     0       0  1.6e-15  1.1e-14
  35  0.00013869  2.6221e-05       75   38   75     2     0       0  1.4e-15  1.6e-14
  36  0.00016491  2.6221e-05       77   39   77     2     0       0  1.8e-15  1.3e-14
  37  0.00019113  2.6221e-05       79   40   79     2     0       0  1.9e-15  1.3e-14
  38  0.00021735  2.6221e-05       81   41   81     2     0       0  9.7e-16  1.1e-14
  39  0.00026979  5.2443e-05       83   42   83     2     0       0  2.8e-15  1.6e-14
  40  0.00032224  5.2443e-05       85   43   85     2     0       0    2e-15  1.4e-14
  41  0.00037468  5.2443e-05       87   44   87     2     0       0  1.9e-15  1.4e-14
  42  0.00047957  0.00010489       89   45   89     2     0       0  4.6e-15  2.1e-14
  43  0.00058445  0.00010489       91   46   91     2     0       0  3.2e-15  1.7e-14
```

44	0.00068934	0.00010489		93	47	93	2	0	0	3.9e-15	1.7e-14
45	0.00089911	0.00020977		95	48	95	2	0	0	3.8e-15	2.4e-14
46	0.0011089	0.00020977		97	49	97	2	0	0	2.1e-15	2e-14
47	0.0013187	0.00020977		99	50	99	2	0	0	6e-15	2.2e-14
48	0.0015284	0.00020977		101	51	101	2	0	0	2.3e-15	2.1e-14
49	0.001948	0.00041954		103	52	103	2	0	0	6.5e-15	3e-14
50	0.0023675	0.00041954		105	53	105	2	0	0	3.1e-15	2.7e-14
51	0.0027871	0.00041954		107	54	107	2	0	0	4e-15	2.6e-14
52	0.0036261	0.00083909		109	55	109	2	0	0	5e-15	3.8e-14
53	0.0044652	0.00083909		111	56	111	2	0	0	3.3e-15	3.4e-14
-	0.005	-	out								
54	0.0053043	0.00083909		113	57	113	2	0	0	1.6e-15	3.6e-14
55	0.0069825	0.0016782		115	58	115	2	0	0	3.9e-15	4.9e-14
56	0.0086607	0.0016782		117	59	117	2	0	0	2.4e-15	4.1e-14
-	0.01	-	out								
57	0.012017	0.0033563		119	60	119	2	0	0	1.4e-15	6.5e-14
-	0.015	-	out								
58	0.015373	0.0033563		121	61	121	2	0	0	5.7e-15	5.1e-14
59	0.01873	0.0033563		123	62	123	2	0	0	5.8e-15	5e-14
-	0.02	-	out								
60	0.022086	0.0033563		125	63	125	2	0	0	4.9e-15	4.4e-14
-	0.025	-	out								
61	0.028799	0.0067127		127	64	127	2	0	0	1e-14	6.4e-14
-	0.03	-	out								
-	0.035	-	out								
62	0.035511	0.0067127		129	65	129	2	0	0	3.3e-15	5.2e-14
-	0.04	-	out								
-	0.045	-	out								
63	0.048937	0.013425		131	66	131	2	0	0	1.4e-14	8e-14
-	0.05	-	out								
-	0.055	-	out								
-	0.06	-	out								
64	0.062362	0.013425		133	67	133	1	0	0	2.3e-14	7.4e-14
-	0.065	-	out								
-	0.07	-	out								
-	0.075	-	out								
65	0.075788	0.013425		135	68	135	1	0	0	1.6e-14	2.8e-14
-	0.08	-	out								
-	0.085	-	out								
-	0.09	-	out								
-	0.095	-	out								
-	0.1	-	out								
66	0.10264	0.026851		137	69	137	1	0	0	5.8e-15	3.9e-14
-	0.105	-	out								
-	0.11	-	out								
-	0.115	-	out								
-	0.12	-	out								
-	0.125	-	out								
-	0.13	-	out								
-	0.135	-	out								
-	0.14	-	out								
-	0.145	-	out								
-	0.15	-	out								
-	0.155	-	out								
67	0.15634	0.053702		139	70	139	1	0	0	7.4e-15	4.8e-14
-	0.16	-	out								

-	0.165	-	out							
-	0.17	-	out							
-	0.175	-	out							
-	0.18	-	out							
-	0.185	-	out							
-	0.19	-	out							
-	0.195	-	out							
-	0.2	-	out							
-	0.205	-	out							
-	0.21	-	out							
-	0.215	-	out							
-	0.22	-	out							
-	0.225	-	out							
-	0.23	-	out							
-	0.235	-	out							
-	0.24	-	out							
-	0.245	-	out							
-	0.25	-	out							
-	0.25	-	out							
68	0.25534	0.099	141	71	141	1	0	0	1.1e-14	5.6e-14
-	0.27	-	out							
-	0.29	-	out							
-	0.31	-	out							
-	0.33	-	out							
-	0.35	-	out							
69	0.35434	0.099	143	72	143	1	0	0	7e-15	4.4e-14
-	0.37	-	out							
-	0.39	-	out							
-	0.41	-	out							
-	0.43	-	out							
-	0.45	-	out							
70	0.45334	0.099	145	73	145	1	0	0	1e-14	3.5e-14
-	0.47	-	out							
-	0.49	-	out							
-	0.51	-	out							
-	0.53	-	out							
-	0.55	-	out							
71	0.55234	0.099	147	74	147	1	0	0	9.4e-15	2.7e-14
-	0.57	-	out							
-	0.59	-	out							
-	0.61	-	out							
-	0.63	-	out							
-	0.65	-	out							
72	0.65134	0.099	148	75	148	1	0	0	1.7e-14	2.2e-14
-	0.67	-	out							
-	0.69	-	out							
-	0.71	-	out							
-	0.73	-	out							
-	0.75	-	out							
73	0.75034	0.099	149	76	149	1	0	0	8.7e-15	1.9e-14
-	0.77	-	out							
-	0.79	-	out							
-	0.81	-	out							
-	0.83	-	out							
74	0.84934	0.099	150	77	150	2	0	0	6.4e-15	1.6e-14
-	0.85	-	out							

```
   -        0.87           - out
   -        0.89           - out
   -        0.91           - out
   -        0.93           - out
  75     0.94834       0.099      151    78   151      2      0       0  8.6e-15  1.1e-14
   -        0.95           - out
   -        0.97           - out
   -        0.99           - out
  76      1.0473       0.099      152    79   152      2      0       0  8.9e-15  9.9e-15
Time-stepping completed.
Solution time: 14 s.
Physical memory: 1.25 GB
Virtual memory: 1.24 GB
Ended at May 28, 2023, 4:13:48 PM.
----- Time-Dependent Solver 1 in Oxygen Pulse/Solution 958 (sol958) ------------&gt;
```

k0Sub=1.51E-7, d=1E-6 (su38)

GENERAL

Description	Value
Solution	k0Sub=1.51E-7, d=1E-6

LOG

```
<---- Compile Equations: Stationary in Oxygen Pulse/Solution 958 (sol958) ------
Started at May 28, 2023, 4:13:51 PM.
Parameter k0Sub = 1.51E-7 (m/s).
Parameter d = 1.0E-6 (m).
Geometry shape function: Linear Lagrange
Number of vertex elements: 10
Minimum element quality: 0.4361
Time: 1 s.
Physical memory: 1.21 GB
Virtual memory: 1.19 GB
Ended at May 28, 2023, 4:13:51 PM.
----- Compile Equations: Stationary in Oxygen Pulse/Solution 958 (sol958) ----->

<---- Dependent Variables 1 in Oxygen Pulse/Solution 958 (sol958) --------------
Started at May 28, 2023, 4:13:51 PM.
Solution time: 0 s.
Physical memory: 1.17 GB
Virtual memory: 1.14 GB
Ended at May 28, 2023, 4:13:51 PM.
----- Dependent Variables 1 in Oxygen Pulse/Solution 958 (sol958) ------------->

<---- Stationary Solver 1 in Oxygen Pulse/Solution 958 (sol958) ----------------
Started at May 28, 2023, 4:13:51 PM.
Linear solver
Number of degrees of freedom solved for: 17724 (plus 963 internal DOFs).
Symmetric matrices found.
Scales for dependent variables:
Concentration (comp2.Oxi): 0.23
Orthonormal null-space function used.
Iter      SolEst     Damping    Stepsize #Res #Jac #Sol   LinErr   LinRes
   1     0.00018   1.0000000     0.00018    1    1    1  4.7e-13  1.3e-13
Solution time: 1 s.
Physical memory: 1.2 GB
Virtual memory: 1.17 GB
Ended at May 28, 2023, 4:13:52 PM.
----- Stationary Solver 1 in Oxygen Pulse/Solution 958 (sol958) --------------->

<---- Compile Equations: Oxygen Probe Pulse in Oxygen Pulse/Solution 958
      (sol958) -----------------------------------------------------------------
Started at May 28, 2023, 4:13:52 PM.
Geometry shape function: Linear Lagrange
Time: 0 s.
Physical memory: 1.21 GB
Virtual memory: 1.19 GB
Ended at May 28, 2023, 4:13:52 PM.
----- Compile Equations: Oxygen Probe Pulse in Oxygen Pulse/Solution 958
      (sol958) ---------------------------------------------------------------->

<---- Dependent Variables 2 in Oxygen Pulse/Solution 958 (sol958) --------------
Started at May 28, 2023, 4:13:52 PM.
Initial values of variables solved for: Solution 958 (sol958), Solution Store 1 (sol
985).
Values of variables not solved for: Solution 958 (sol958), Solution Store 1 (sol985)
.
Solution time: 0 s.
Physical memory: 1.22 GB
```

Virtual memory: 1.19 GB
Ended at May 28, 2023, 4:13:52 PM.
----- Dependent Variables 2 in Oxygen Pulse/Solution 958 (sol958) ------------->

<---- Time-Dependent Solver 1 in Oxygen Pulse/Solution 958 (sol958) ------------
Started at May 28, 2023, 4:13:52 PM.
Time-dependent solver (BDF)
Number of degrees of freedom solved for: 17724 (plus 35562 internal DOFs).
Symmetric matrices found.
Scales for dependent variables:
Concentration (comp2.Oxi): 0.23

Step	Time	Stepsize		Res	Jac	Sol	Order	Tfail	NLfail	LinErr	LinRes
0	0	-	out	5	3	5			0	1.1e-15	1.5e-15
1	2.5904e-08	2.5904e-08		7	4	7	1	0	0	3.4e-16	1e-15
2	5.1809e-08	2.5904e-08		9	5	9	1	0	0	4.4e-16	1.2e-15
3	1.0362e-07	5.1809e-08		11	6	11	2	0	0	3.7e-16	1.2e-15
4	2.0723e-07	1.0362e-07		13	7	13	2	0	0	4.5e-16	1.7e-15
5	4.1447e-07	2.0723e-07		15	8	15	2	0	0	5.6e-16	3.3e-15
6	6.217e-07	2.0723e-07		17	9	17	2	0	0	6.4e-16	3.5e-15
7	8.2894e-07	2.0723e-07		19	10	19	2	0	0	6e-16	3.8e-15
8	1.0362e-06	2.0723e-07		21	11	21	2	0	0	7.6e-16	3.8e-15
9	1.4506e-06	4.1447e-07		23	12	23	2	0	0	9.1e-16	6.1e-15
10	1.8651e-06	4.1447e-07		25	13	25	2	0	0	1e-15	6.1e-15
11	2.2796e-06	4.1447e-07		27	14	27	2	0	0	7.6e-16	6e-15
12	2.694e-06	4.1447e-07		29	15	29	2	0	0	8.4e-16	5.5e-15
13	3.523e-06	8.2894e-07		31	16	31	2	0	0	8.6e-16	7.9e-15
14	4.3519e-06	8.2894e-07		33	17	33	2	0	0	1.3e-15	7e-15
15	5.1809e-06	8.2894e-07		35	18	35	2	0	0	1.5e-15	7.2e-15
16	6.0098e-06	8.2894e-07		37	19	37	2	0	0	9.3e-16	6.3e-15
17	7.6677e-06	1.6579e-06		39	20	39	2	0	0	1.5e-15	9.7e-15
18	9.3255e-06	1.6579e-06		41	21	41	2	0	0	1.1e-15	8.2e-15
19	1.0983e-05	1.6579e-06		43	22	43	2	0	0	1.4e-15	7.5e-15
20	1.2641e-05	1.6579e-06		45	23	45	2	0	0	1.3e-15	7e-15
21	1.4299e-05	1.6579e-06		47	24	47	2	0	0	1.5e-15	6.1e-15
22	1.7615e-05	3.3157e-06		49	25	49	2	0	0	1.3e-15	8.9e-15
23	2.0931e-05	3.3157e-06		51	26	51	2	0	0	1.2e-15	8e-15
24	2.4246e-05	3.3157e-06		53	27	53	2	0	0	1.2e-15	7.9e-15
25	2.7562e-05	3.3157e-06		55	28	55	2	0	0	2.3e-15	7.1e-15
26	3.4194e-05	6.6315e-06		57	29	57	2	0	0	1.8e-15	1e-14
27	4.0825e-05	6.6315e-06		59	30	59	2	0	0	2.5e-15	9.3e-15
28	4.7457e-05	6.6315e-06		61	31	61	2	0	0	1.8e-15	9.5e-15
29	5.4088e-05	6.6315e-06		63	32	63	2	0	0	1.8e-15	8.5e-15
30	6.072e-05	6.6315e-06		65	33	65	2	0	0	1.5e-15	7.6e-15
31	7.3983e-05	1.3263e-05		67	34	67	2	0	0	2.7e-15	1.2e-14
32	8.7246e-05	1.3263e-05		69	35	69	2	0	0	1.7e-15	9.8e-15
33	0.00010051	1.3263e-05		71	36	71	2	0	0	1.4e-15	1e-14
34	0.00011377	1.3263e-05		73	37	73	2	0	0	1.7e-15	9.4e-15
35	0.0001403	2.6526e-05		75	38	75	2	0	0	1.7e-15	1.3e-14
36	0.00016682	2.6526e-05		77	39	77	2	0	0	2.3e-15	1.2e-14
37	0.00019335	2.6526e-05		79	40	79	2	0	0	1.7e-15	1.2e-14
38	0.00021988	2.6526e-05		81	41	81	2	0	0	1.4e-15	1.1e-14
39	0.00027293	5.3052e-05		83	42	83	2	0	0	2.2e-15	1.6e-14
40	0.00032598	5.3052e-05		85	43	85	2	0	0	2.8e-15	1.3e-14
41	0.00037903	5.3052e-05		87	44	87	2	0	0	2.6e-15	1.3e-14
42	0.00048514	0.0001061		89	45	89	2	0	0	1.8e-15	1.8e-14
43	0.00059124	0.0001061		91	46	91	2	0	0	2.8e-15	1.6e-14

44	0.00069734	0.0001061	93	47	93	2	0	0	2.5e-15	1.6e-14
45	0.00080345	0.0001061	95	48	95	2	0	0	1.9e-15	1.4e-14
46	0.0010157	0.00021221	97	49	97	2	0	0	2.5e-15	2e-14
47	0.0012279	0.00021221	99	50	99	2	0	0	5.3e-15	1.6e-14
48	0.0016523	0.00042442	101	51	101	2	0	0	2e-15	2.8e-14
49	0.0020767	0.00042442	103	52	103	2	0	0	1.4e-15	2.5e-14
50	0.0025011	0.00042442	105	53	105	2	0	0	4.3e-15	2.5e-14
51	0.0029255	0.00042442	107	54	107	2	0	0	3.3e-15	2.4e-14
52	0.0037744	0.00084883	109	55	109	2	0	0	3.3e-15	3.2e-14
53	0.0046232	0.00084883	111	56	111	2	0	0	3.2e-15	2.8e-14
-	0.005	-	out							
54	0.0063209	0.0016977	113	57	113	2	0	0	8.3e-15	4.5e-14
55	0.0080185	0.0016977	115	58	115	2	0	0	5.9e-15	4.3e-14
56	0.0097162	0.0016977	117	59	117	2	0	0	4.6e-15	4.5e-14
-	0.01	-	out							
57	0.011414	0.0016977	119	60	119	2	0	0	6.6e-15	4e-14
58	0.014809	0.0033953	121	61	121	2	0	0	8.9e-15	4.9e-14
-	0.015	-	out							
59	0.018204	0.0033953	123	62	123	2	0	0	4.3e-15	3.7e-14
-	0.02	-	out							
60	0.024995	0.0067907	125	63	125	2	0	0	3.1e-15	6.2e-14
-	0.025	-	out							
-	0.03	-	out							
61	0.031786	0.0067907	127	64	127	2	0	0	8.1e-15	6e-14
-	0.035	-	out							
62	0.038576	0.0067907	129	65	129	2	0	0	1.1e-14	5.9e-14
-	0.04	-	out							
-	0.045	-	out							
63	0.045367	0.0067907	131	66	131	2	0	0	3.8e-15	5.2e-14
-	0.05	-	out							
-	0.055	-	out							
64	0.058948	0.013581	133	67	133	2	0	0	4.9e-15	6.7e-14
-	0.06	-	out							
-	0.065	-	out							
-	0.07	-	out							
65	0.07253	0.013581	135	68	135	2	0	0	5.1e-15	4.1e-14
-	0.075	-	out							
-	0.08	-	out							
-	0.085	-	out							
-	0.09	-	out							
-	0.095	-	out							
66	0.099692	0.027163	137	69	137	2	0	0	9.7e-15	6.3e-14
-	0.1	-	out							
-	0.105	-	out							
-	0.11	-	out							
-	0.115	-	out							
-	0.12	-	out							
-	0.125	-	out							
-	0.13	-	out							
-	0.135	-	out							
-	0.14	-	out							
-	0.145	-	out							
-	0.15	-	out							
67	0.15402	0.054325	139	70	139	1	0	0	9.5e-15	5.9e-14
-	0.155	-	out							
-	0.16	-	out							

-	0.165	-	out							
-	0.17	-	out							
-	0.175	-	out							
-	0.18	-	out							
-	0.185	-	out							
-	0.19	-	out							
-	0.195	-	out							
-	0.2	-	out							
-	0.205	-	out							
68	0.20834	0.054325	141	71	141	1	0	0	6.8e-15	3e-14
-	0.21	-	out							
-	0.215	-	out							
-	0.22	-	out							
-	0.225	-	out							
-	0.23	-	out							
-	0.235	-	out							
-	0.24	-	out							
-	0.245	-	out							
-	0.25	-	out							
-	0.25	-	out							
-	0.27	-	out							
-	0.29	-	out							
69	0.30734	0.099	143	72	143	1	0	0	1.7e-14	3.6e-14
-	0.31	-	out							
-	0.33	-	out							
-	0.35	-	out							
-	0.37	-	out							
-	0.39	-	out							
70	0.40634	0.099	145	73	145	1	0	0	4.7e-15	3.4e-14
-	0.41	-	out							
-	0.43	-	out							
-	0.45	-	out							
-	0.47	-	out							
-	0.49	-	out							
71	0.50534	0.099	147	74	147	1	0	0	1.6e-14	2.9e-14
-	0.51	-	out							
-	0.53	-	out							
-	0.55	-	out							
-	0.57	-	out							
-	0.59	-	out							
72	0.60434	0.099	149	75	149	1	0	0	3.5e-15	2.4e-14
-	0.61	-	out							
-	0.63	-	out							
-	0.65	-	out							
-	0.67	-	out							
-	0.69	-	out							
73	0.70334	0.099	150	76	150	1	0	0	2e-14	2e-14
-	0.71	-	out							
-	0.73	-	out							
-	0.75	-	out							
-	0.77	-	out							
-	0.79	-	out							
74	0.80234	0.099	151	77	151	1	0	0	3.1e-15	1.8e-14
-	0.81	-	out							
-	0.83	-	out							
-	0.85	-	out							

```
   -        0.87            - out
   -        0.89            - out
  75      0.90134        0.099      152    78   152      2      0       0  6.5e-15  1.5e-14
   -        0.91            - out
   -        0.93            - out
   -        0.95            - out
   -        0.97            - out
   -        0.99            - out
  76       1.0003        0.099      153    79   153      2      0       0  3.5e-15  9.4e-15
Time-stepping completed.
Solution time: 14 s.
Physical memory: 1.24 GB
Virtual memory: 1.23 GB
Ended at May 28, 2023, 4:14:07 PM.
----- Time-Dependent Solver 1 in Oxygen Pulse/Solution 958 (sol958) ----------->
```

k0Sub=1.51E-7, d=1.5E-6 (su39)

GENERAL

Description	Value
Solution	k0Sub=1.51E-7, d=1.5E-6

LOG

```
<---- Compile Equations: Stationary in Oxygen Pulse/Solution 958 (sol958) ------
Started at May 28, 2023, 4:14:10 PM.
Parameter k0Sub = 1.51E-7 (m/s).
Parameter d = 1.5E-6 (m).
Geometry shape function: Linear Lagrange
Number of vertex elements: 10
Minimum element quality: 0.395
Time: 1 s.
Physical memory: 1.21 GB
Virtual memory: 1.2 GB
Ended at May 28, 2023, 4:14:11 PM.
----- Compile Equations: Stationary in Oxygen Pulse/Solution 958 (sol958) ----->

<---- Dependent Variables 1 in Oxygen Pulse/Solution 958 (sol958) --------------
Started at May 28, 2023, 4:14:11 PM.
Solution time: 0 s.
Physical memory: 1.22 GB
Virtual memory: 1.2 GB
Ended at May 28, 2023, 4:14:11 PM.
----- Dependent Variables 1 in Oxygen Pulse/Solution 958 (sol958) ------------->

<---- Stationary Solver 1 in Oxygen Pulse/Solution 958 (sol958) ----------------
Started at May 28, 2023, 4:14:11 PM.
Linear solver
Number of degrees of freedom solved for: 17823 (plus 950 internal DOFs).
Symmetric matrices found.
Scales for dependent variables:
Concentration (comp2.Oxi): 0.23
Orthonormal null-space function used.
Iter      SolEst     Damping    Stepsize #Res #Jac #Sol   LinErr   LinRes
   1     0.00015   1.0000000     0.00015    1    1    1  4.3e-13  1.1e-13
Solution time: 1 s.
Physical memory: 1.23 GB
Virtual memory: 1.21 GB
Ended at May 28, 2023, 4:14:12 PM.
----- Stationary Solver 1 in Oxygen Pulse/Solution 958 (sol958) --------------->

<---- Compile Equations: Oxygen Probe Pulse in Oxygen Pulse/Solution 958
      (sol958) ----------------------------------------------------------------
Started at May 28, 2023, 4:14:12 PM.
Geometry shape function: Linear Lagrange
Time: 1 s.
Physical memory: 1.24 GB
Virtual memory: 1.22 GB
Ended at May 28, 2023, 4:14:12 PM.
----- Compile Equations: Oxygen Probe Pulse in Oxygen Pulse/Solution 958
      (sol958) --------------------------------------------------------------->

<---- Dependent Variables 2 in Oxygen Pulse/Solution 958 (sol958) --------------
Started at May 28, 2023, 4:14:12 PM.
Initial values of variables solved for: Solution 958 (sol958), Solution Store 1 (sol
985).
Values of variables not solved for: Solution 958 (sol958), Solution Store 1 (sol985)
.
Solution time: 0 s.
Physical memory: 1.24 GB
```

```
Virtual memory: 1.22 GB
Ended at May 28, 2023, 4:14:12 PM.
----- Dependent Variables 2 in Oxygen Pulse/Solution 958 (sol958) ------------->

<---- Time-Dependent Solver 1 in Oxygen Pulse/Solution 958 (sol958) ------------
Started at May 28, 2023, 4:14:12 PM.
Time-dependent solver (BDF)
Number of degrees of freedom solved for: 17823 (plus 35744 internal DOFs).
Symmetric matrices found.
Scales for dependent variables:
Concentration (comp2.Oxi): 0.23
Step        Time    Stepsize      Res  Jac   Sol Order Tfail NLfail   LinErr   LinRes
   0           0           - out    5    3     5                 0    1e-15  1.5e-15
   1  2.5751e-08  2.5751e-08        7    4     7     1     0     0  3.4e-16    1e-15
   2  5.1501e-08  2.5751e-08        9    5     9     1     0     0  3.7e-16  1.2e-15
   3    1.03e-07  5.1501e-08       11    6    11     2     0     0  3.7e-16  1.2e-15
   4  2.0601e-07    1.03e-07       13    7    13     2     0     0    5e-16  1.7e-15
   5  4.1201e-07  2.0601e-07       15    8    15     2     0     0  5.7e-16  3.3e-15
   6  6.1802e-07  2.0601e-07       17    9    17     2     0     0  7.1e-16    4e-15
   7  8.2402e-07  2.0601e-07       19   10    19     2     0     0  6.3e-16  3.9e-15
   8    1.03e-06  2.0601e-07       21   11    21     2     0     0    6e-16  3.9e-15
   9   1.442e-06  4.1201e-07       23   12    23     2     0     0  8.4e-16  6.2e-15
  10   1.854e-06  4.1201e-07       25   13    25     2     0     0  8.2e-16  5.9e-15
  11  2.2661e-06  4.1201e-07       27   14    27     2     0     0  1.1e-15    6e-15
  12  2.6781e-06  4.1201e-07       29   15    29     2     0     0  8.4e-16  5.7e-15
  13  3.5021e-06  8.2402e-07       31   16    31     2     0     0  1.1e-15  8.5e-15
  14  4.3261e-06  8.2402e-07       33   17    33     2     0     0    1e-15    7e-15
  15  5.1501e-06  8.2402e-07       35   18    35     2     0     0  1.2e-15  6.7e-15
  16  5.9741e-06  8.2402e-07       37   19    37     2     0     0  1.3e-15  6.2e-15
  17  7.6222e-06   1.648e-06       39   20    39     2     0     0  1.4e-15  9.4e-15
  18  9.2702e-06   1.648e-06       41   21    41     2     0     0  1.7e-15    8e-15
  19  1.0918e-05   1.648e-06       43   22    43     2     0     0  1.7e-15  7.9e-15
  20  1.2566e-05   1.648e-06       45   23    45     2     0     0  1.5e-15  7.1e-15
  21  1.4214e-05   1.648e-06       47   24    47     2     0     0  1.8e-15  6.5e-15
  22   1.751e-05  3.2961e-06       49   25    49     2     0     0  1.4e-15  9.3e-15
  23  2.0807e-05  3.2961e-06       51   26    51     2     0     0  1.9e-15  8.2e-15
  24  2.4103e-05  3.2961e-06       53   27    53     2     0     0  1.5e-15    8e-15
  25  2.7399e-05  3.2961e-06       55   28    55     2     0     0  1.7e-15  7.4e-15
  26  3.3991e-05  6.5922e-06       57   29    57     2     0     0  1.6e-15  1.1e-14
  27  4.0583e-05  6.5922e-06       59   30    59     2     0     0  1.9e-15  8.8e-15
  28  4.7175e-05  6.5922e-06       61   31    61     2     0     0  1.4e-15  9.3e-15
  29  5.3767e-05  6.5922e-06       63   32    63     2     0     0  2.3e-15  8.3e-15
  30  6.0359e-05  6.5922e-06       65   33    65     2     0     0  1.7e-15  7.6e-15
  31  7.3544e-05  1.3184e-05       67   34    67     2     0     0  1.8e-15  1.1e-14
  32  8.6728e-05  1.3184e-05       69   35    69     2     0     0  1.3e-15  9.6e-15
  33  9.9912e-05  1.3184e-05       71   36    71     2     0     0  2.5e-15  9.8e-15
  34   0.0001131  1.3184e-05       73   37    73     2     0     0  1.9e-15  8.8e-15
  35  0.00013947  2.6369e-05       75   38    75     2     0     0    2e-15  1.3e-14
  36  0.00016583  2.6369e-05       77   39    77     2     0     0  3.7e-15  1.1e-14
  37   0.0001922  2.6369e-05       79   40    79     2     0     0  2.1e-15  1.1e-14
  38  0.00021857  2.6369e-05       81   41    81     2     0     0  2.9e-15  1.1e-14
  39  0.00027131  5.2737e-05       83   42    83     2     0     0  1.9e-15  1.5e-14
  40  0.00032405  5.2737e-05       85   43    85     2     0     0    2e-15  1.3e-14
  41  0.00037678  5.2737e-05       87   44    87     2     0     0    3e-15  1.3e-14
  42  0.00048226  0.00010547       89   45    89     2     0     0  6.2e-15  1.9e-14
  43  0.00058773  0.00010547       91   46    91     2     0     0  3.6e-15  1.5e-14
```

44	0.00069321	0.00010547		93	47	93	2	0	0	2.5e-15	1.6e-14
45	0.00079868	0.00010547		95	48	95	2	0	0	1.6e-15	1.4e-14
46	0.0010096	0.00021095		97	49	97	2	0	0	6.3e-15	2e-14
47	0.0012206	0.00021095		99	50	99	2	0	0	1.7e-15	1.8e-14
48	0.0016425	0.0004219		101	51	101	2	0	0	3.2e-15	2.8e-14
49	0.0020644	0.0004219		103	52	103	2	0	0	2.3e-15	2.5e-14
50	0.0024863	0.0004219		105	53	105	2	0	0	5.1e-15	2.4e-14
51	0.0033301	0.0008438		107	54	107	2	0	0	6.8e-15	3.2e-14
52	0.0041739	0.0008438		109	55	109	2	0	0	2.6e-15	2.6e-14
-	0.005	-	out								
53	0.0050177	0.0008438		111	56	111	2	0	0	2.7e-15	2.7e-14
54	0.0067053	0.0016876		113	57	113	2	0	0	5.3e-15	4.2e-14
55	0.0083929	0.0016876		115	58	115	2	0	0	1.9e-15	3.7e-14
-	0.01	-	out								
56	0.011768	0.0033752		117	59	117	2	0	0	5.4e-15	5.8e-14
-	0.015	-	out								
57	0.015143	0.0033752		119	60	119	2	0	0	4.6e-15	4.9e-14
58	0.018518	0.0033752		121	61	121	2	0	0	4e-15	5e-14
-	0.02	-	out								
59	0.021894	0.0033752		123	62	123	2	0	0	4.4e-15	4e-14
-	0.025	-	out								
60	0.028644	0.0067504		125	63	125	2	0	0	8.7e-15	5.5e-14
-	0.03	-	out								
-	0.035	-	out								
61	0.035394	0.0067504		127	64	127	2	0	0	3.6e-15	4.7e-14
-	0.04	-	out								
-	0.045	-	out								
62	0.048895	0.013501		129	65	129	2	0	0	8.8e-15	7.5e-14
-	0.05	-	out								
-	0.055	-	out								
-	0.06	-	out								
63	0.062396	0.013501		131	66	131	2	0	0	4.1e-15	6.6e-14
-	0.065	-	out								
-	0.07	-	out								
-	0.075	-	out								
64	0.075897	0.013501		133	67	133	2	0	0	4.8e-15	5.9e-14
-	0.08	-	out								
-	0.085	-	out								
-	0.09	-	out								
-	0.095	-	out								
-	0.1	-	out								
65	0.1029	0.027002		135	68	135	2	0	0	8.9e-15	6.4e-14
-	0.105	-	out								
-	0.11	-	out								
-	0.115	-	out								
-	0.12	-	out								
-	0.125	-	out								
-	0.13	-	out								
-	0.135	-	out								
-	0.14	-	out								
-	0.145	-	out								
-	0.15	-	out								
-	0.155	-	out								
66	0.1569	0.054003		137	69	137	1	0	0	7.8e-15	5.7e-14
-	0.16	-	out								
-	0.165	-	out								

-	0.17	-	out							
-	0.175	-	out							
-	0.18	-	out							
-	0.185	-	out							
-	0.19	-	out							
-	0.195	-	out							
-	0.2	-	out							
-	0.205	-	out							
-	0.21	-	out							
67	0.2109	0.054003	139	70	139	1	0	0	9.1e-15	3.1e-14
-	0.215	-	out							
-	0.22	-	out							
-	0.225	-	out							
-	0.23	-	out							
-	0.235	-	out							
-	0.24	-	out							
-	0.245	-	out							
-	0.25	-	out							
-	0.25	-	out							
-	0.27	-	out							
-	0.29	-	out							
68	0.3099	0.099	141	71	141	1	0	0	6.4e-15	3.8e-14
-	0.31	-	out							
-	0.33	-	out							
-	0.35	-	out							
-	0.37	-	out							
-	0.39	-	out							
69	0.4089	0.099	143	72	143	1	0	0	7.3e-15	3.2e-14
-	0.41	-	out							
-	0.43	-	out							
-	0.45	-	out							
-	0.47	-	out							
-	0.49	-	out							
70	0.5079	0.099	145	73	145	1	0	0	3.2e-15	2.9e-14
-	0.51	-	out							
-	0.53	-	out							
-	0.55	-	out							
-	0.57	-	out							
-	0.59	-	out							
71	0.6069	0.099	147	74	147	1	0	0	1.1e-14	2.4e-14
-	0.61	-	out							
-	0.63	-	out							
-	0.65	-	out							
-	0.67	-	out							
-	0.69	-	out							
72	0.7059	0.099	148	75	148	1	0	0	2.4e-15	2.1e-14
-	0.71	-	out							
-	0.73	-	out							
-	0.75	-	out							
-	0.77	-	out							
-	0.79	-	out							
73	0.8049	0.099	149	76	149	1	0	0	8.7e-15	1.8e-14
-	0.81	-	out							
-	0.83	-	out							
-	0.85	-	out							
-	0.87	-	out							

```
  -       0.89          - out
 74     0.9039      0.099      150   77  150     2     0      0    2e-15  1.5e-14
  -       0.91          - out
  -       0.93          - out
  -       0.95          - out
  -       0.97          - out
  -       0.99          - out
 75     1.0029      0.099      151   78  151     2     0      0    6e-15  9.8e-15
Time-stepping completed.
Solution time: 17 s.
Physical memory: 1.26 GB
Virtual memory: 1.24 GB
Ended at May 28, 2023, 4:14:29 PM.
----- Time-Dependent Solver 1 in Oxygen Pulse/Solution 958 (sol958) ----------->
```

k0Sub=1.51E-7, d=2E-6 (su40)

GENERAL

Description	Value
Solution	k0Sub=1.51E-7, d=2E-6

LOG

```
<---- Compile Equations: Stationary in Oxygen Pulse/Solution 958 (sol958) ------
Started at May 28, 2023, 4:14:32 PM.
Parameter k0Sub = 1.51E-7 (m/s).
Parameter d = 2.0E-6 (m).
Geometry shape function: Linear Lagrange
Number of vertex elements: 10
Minimum element quality: 0.4112
Time: 1 s.
Physical memory: 1.22 GB
Virtual memory: 1.2 GB
Ended at May 28, 2023, 4:14:33 PM.
----- Compile Equations: Stationary in Oxygen Pulse/Solution 958 (sol958) ----->

<---- Dependent Variables 1 in Oxygen Pulse/Solution 958 (sol958) --------------
Started at May 28, 2023, 4:14:33 PM.
Solution time: 0 s.
Physical memory: 1.22 GB
Virtual memory: 1.2 GB
Ended at May 28, 2023, 4:14:33 PM.
----- Dependent Variables 1 in Oxygen Pulse/Solution 958 (sol958) ------------->

<---- Stationary Solver 1 in Oxygen Pulse/Solution 958 (sol958) ----------------
Started at May 28, 2023, 4:14:33 PM.
Linear solver
Number of degrees of freedom solved for: 17907 (plus 938 internal DOFs).
Symmetric matrices found.
Scales for dependent variables:
Concentration (comp2.Oxi): 0.23
Orthonormal null-space function used.
Iter      SolEst     Damping    Stepsize #Res #Jac #Sol   LinErr   LinRes
   1     0.00014   1.0000000     0.00014    1    1    1  4.3e-13    1e-13
Solution time: 1 s.
Physical memory: 1.23 GB
Virtual memory: 1.21 GB
Ended at May 28, 2023, 4:14:33 PM.
----- Stationary Solver 1 in Oxygen Pulse/Solution 958 (sol958) --------------->

<---- Compile Equations: Oxygen Probe Pulse in Oxygen Pulse/Solution 958
      (sol958) ----------------------------------------------------------------
Started at May 28, 2023, 4:14:33 PM.
Geometry shape function: Linear Lagrange
Time: 1 s.
Physical memory: 1.24 GB
Virtual memory: 1.21 GB
Ended at May 28, 2023, 4:14:34 PM.
----- Compile Equations: Oxygen Probe Pulse in Oxygen Pulse/Solution 958
      (sol958) --------------------------------------------------------------->

<---- Dependent Variables 2 in Oxygen Pulse/Solution 958 (sol958) --------------
Started at May 28, 2023, 4:14:34 PM.
Initial values of variables solved for: Solution 958 (sol958), Solution Store 1 (sol
985).
Values of variables not solved for: Solution 958 (sol958), Solution Store 1 (sol985)
.
Solution time: 0 s.
Physical memory: 1.24 GB
```

Virtual memory: 1.21 GB
Ended at May 28, 2023, 4:14:34 PM.
----- Dependent Variables 2 in Oxygen Pulse/Solution 958 (sol958) ------------->

<---- Time-Dependent Solver 1 in Oxygen Pulse/Solution 958 (sol958) ------------
Started at May 28, 2023, 4:14:34 PM.
Time-dependent solver (BDF)
Number of degrees of freedom solved for: 17907 (plus 35898 internal DOFs).
Symmetric matrices found.
Scales for dependent variables:
Concentration (comp2.Oxi): 0.23

Step	Time	Stepsize	Res	Jac	Sol	Order	Tfail	NLfail	LinErr	LinRes
0	0	- out	5	3	5			0	7.7e-16	1.4e-15
1	2.577e-08	2.577e-08	7	4	7	1	0	0	3e-16	9.8e-16
2	5.1541e-08	2.577e-08	9	5	9	1	0	0	3.8e-16	1.2e-15
3	1.0308e-07	5.1541e-08	11	6	11	2	0	0	3.6e-16	1.2e-15
4	2.0616e-07	1.0308e-07	13	7	13	2	0	0	4.9e-16	1.7e-15
5	4.1233e-07	2.0616e-07	15	8	15	2	0	0	6.8e-16	3.4e-15
6	6.1849e-07	2.0616e-07	17	9	17	2	0	0	7.4e-16	3.8e-15
7	8.2466e-07	2.0616e-07	19	10	19	2	0	0	6.7e-16	3.9e-15
8	1.0308e-06	2.0616e-07	21	11	21	2	0	0	6.4e-16	4.1e-15
9	1.4431e-06	4.1233e-07	23	12	23	2	0	0	8.9e-16	6.3e-15
10	1.8555e-06	4.1233e-07	25	13	25	2	0	0	7.3e-16	6.1e-15
11	2.2678e-06	4.1233e-07	27	14	27	2	0	0	7e-16	5.9e-15
12	2.6801e-06	4.1233e-07	29	15	29	2	0	0	8.3e-16	5.7e-15
13	3.5048e-06	8.2466e-07	31	16	31	2	0	0	1e-15	8.2e-15
14	4.3294e-06	8.2466e-07	33	17	33	2	0	0	1.1e-15	7.5e-15
15	5.1541e-06	8.2466e-07	35	18	35	2	0	0	1.3e-15	7.2e-15
16	5.9788e-06	8.2466e-07	37	19	37	2	0	0	1.2e-15	6.6e-15
17	7.6281e-06	1.6493e-06	39	20	39	2	0	0	1.4e-15	9.9e-15
18	9.2774e-06	1.6493e-06	41	21	41	2	0	0	1.1e-15	8.2e-15
19	1.0927e-05	1.6493e-06	43	22	43	2	0	0	1.2e-15	8.5e-15
20	1.2576e-05	1.6493e-06	45	23	45	2	0	0	1.5e-15	7.4e-15
21	1.4225e-05	1.6493e-06	47	24	47	2	0	0	1.2e-15	6.9e-15
22	1.7524e-05	3.2986e-06	49	25	49	2	0	0	1.2e-15	9.7e-15
23	2.0823e-05	3.2986e-06	51	26	51	2	0	0	1.2e-15	8.7e-15
24	2.4121e-05	3.2986e-06	53	27	53	2	0	0	1.5e-15	8.5e-15
25	2.742e-05	3.2986e-06	55	28	55	2	0	0	1.8e-15	8.1e-15
26	3.4017e-05	6.5972e-06	57	29	57	2	0	0	2.3e-15	1.1e-14
27	4.0614e-05	6.5972e-06	59	30	59	2	0	0	1.5e-15	9.3e-15
28	4.7212e-05	6.5972e-06	61	31	61	2	0	0	3.3e-15	9.7e-15
29	5.3809e-05	6.5972e-06	63	32	63	2	0	0	2.5e-15	8.5e-15
30	6.0406e-05	6.5972e-06	65	33	65	2	0	0	1.4e-15	7.9e-15
31	7.36e-05	1.3194e-05	67	34	67	2	0	0	1.7e-15	1.1e-14
32	8.6795e-05	1.3194e-05	69	35	69	2	0	0	1.7e-15	1e-14
33	9.9989e-05	1.3194e-05	71	36	71	2	0	0	2.5e-15	9.9e-15
34	0.00011318	1.3194e-05	73	37	73	2	0	0	2.3e-15	9e-15
35	0.00013957	2.6389e-05	75	38	75	2	0	0	1.6e-15	1.3e-14
36	0.00016596	2.6389e-05	77	39	77	2	0	0	1.9e-15	1.2e-14
37	0.00019235	2.6389e-05	79	40	79	2	0	0	3.1e-15	1.1e-14
38	0.00021874	2.6389e-05	81	41	81	2	0	0	2.2e-15	1e-14
39	0.00027152	5.2778e-05	83	42	83	2	0	0	2.2e-15	1.5e-14
40	0.0003243	5.2778e-05	85	43	85	2	0	0	2.6e-15	1.2e-14
41	0.00037707	5.2778e-05	87	44	87	2	0	0	1.5e-15	1.2e-14
42	0.00048263	0.00010556	89	45	89	2	0	0	4.2e-15	1.8e-14
43	0.00058819	0.00010556	91	46	91	2	0	0	4e-15	1.5e-14

44	0.00069374	0.00010556	93	47	93	2	0	0	4e-15	1.5e-14
45	0.0007993	0.00010556	95	48	95	2	0	0	3e-15	1.3e-14
46	0.0010104	0.00021111	97	49	97	2	0	0	2.1e-15	1.9e-14
47	0.0012215	0.00021111	99	50	99	2	0	0	1.5e-15	1.6e-14
48	0.0016437	0.00042222	101	51	101	2	0	0	4e-15	2.5e-14
49	0.002066	0.00042222	103	52	103	2	0	0	3.9e-15	2.4e-14
50	0.0024882	0.00042222	105	53	105	2	0	0	3e-15	2.3e-14
51	0.0033326	0.00084445	107	54	107	2	0	0	3.5e-15	2.9e-14
52	0.0041771	0.00084445	109	55	109	2	0	0	1.9e-15	2.2e-14
-	0.005	- out								
53	0.005866	0.0016889	111	56	111	2	0	0	5.2e-15	3.4e-14
54	0.0075549	0.0016889	113	57	113	2	0	0	3.6e-15	3.5e-14
55	0.0092438	0.0016889	115	58	115	2	0	0	6.7e-15	3.8e-14
-	0.01	- out								
56	0.010933	0.0016889	117	59	117	2	0	0	3.3e-15	3.4e-14
57	0.01431	0.0033778	119	60	119	2	0	0	2.7e-15	4.6e-14
-	0.015	- out								
58	0.017688	0.0033778	121	61	121	2	0	0	2.6e-15	3.3e-14
-	0.02	- out								
59	0.024444	0.0067556	123	62	123	2	0	0	3.3e-15	5.8e-14
-	0.025	- out								
-	0.03	- out								
60	0.031199	0.0067556	125	63	125	2	0	0	4.8e-15	5.1e-14
-	0.035	- out								
61	0.037955	0.0067556	127	64	127	2	0	0	2.6e-15	5.4e-14
-	0.04	- out								
-	0.045	- out								
-	0.05	- out								
62	0.051466	0.013511	129	65	129	2	0	0	3.4e-15	7.5e-14
-	0.055	- out								
-	0.06	- out								
63	0.064977	0.013511	131	66	131	2	0	0	2.9e-15	5.3e-14
-	0.065	- out								
-	0.07	- out								
-	0.075	- out								
-	0.08	- out								
-	0.085	- out								
-	0.09	- out								
64	0.092	0.027022	133	67	133	2	0	0	1.4e-14	8.3e-14
-	0.095	- out								
-	0.1	- out								
-	0.105	- out								
-	0.11	- out								
-	0.115	- out								
65	0.11902	0.027022	135	68	135	1	0	0	9.7e-15	5.7e-14
-	0.12	- out								
-	0.125	- out								
-	0.13	- out								
-	0.135	- out								
-	0.14	- out								
-	0.145	- out								
-	0.15	- out								
-	0.155	- out								
-	0.16	- out								
-	0.165	- out								
-	0.17	- out								

66	0.17307	0.054045	137	69	137	1	0	0	1.3e-14	2.9e-14
-	0.175	- out								
-	0.18	- out								
-	0.185	- out								
-	0.19	- out								
-	0.195	- out								
-	0.2	- out								
-	0.205	- out								
-	0.21	- out								
-	0.215	- out								
-	0.22	- out								
-	0.225	- out								
-	0.23	- out								
-	0.235	- out								
-	0.24	- out								
-	0.245	- out								
-	0.25	- out								
-	0.25	- out								
-	0.27	- out								
67	0.27207	0.099	139	70	139	1	0	0	1.5e-14	4.7e-14
-	0.29	- out								
-	0.31	- out								
-	0.33	- out								
-	0.35	- out								
-	0.37	- out								
68	0.37107	0.099	141	71	141	1	0	0	1.6e-14	4.3e-14
-	0.39	- out								
-	0.41	- out								
-	0.43	- out								
-	0.45	- out								
-	0.47	- out								
69	0.47007	0.099	143	72	143	1	0	0	1.1e-14	3.5e-14
-	0.49	- out								
-	0.51	- out								
-	0.53	- out								
-	0.55	- out								
70	0.56907	0.099	145	73	145	1	0	0	4.2e-15	2.7e-14
-	0.57	- out								
-	0.59	- out								
-	0.61	- out								
-	0.63	- out								
-	0.65	- out								
71	0.66807	0.099	146	74	146	1	0	0	4.6e-15	2.3e-14
-	0.67	- out								
-	0.69	- out								
-	0.71	- out								
-	0.73	- out								
-	0.75	- out								
72	0.76707	0.099	147	75	147	1	0	0	3.3e-15	2.1e-14
-	0.77	- out								
-	0.79	- out								
-	0.81	- out								
-	0.83	- out								
-	0.85	- out								
73	0.86607	0.099	148	76	148	2	0	0	4.9e-15	1.7e-14
-	0.87	- out								

```
   -        0.89           - out
   -        0.91           - out
   -        0.93           - out
   -        0.95           - out
  74     0.96507       0.099      149   77  149     2     0      0  1.4e-14  1.1e-14
   -        0.97           - out
   -        0.99           - out
  75      1.0641       0.099      150   78  150     2     0      0  1.3e-14    1e-14
Time-stepping completed.
Solution time: 16 s.
Physical memory: 1.25 GB
Virtual memory: 1.24 GB
Ended at May 28, 2023, 4:14:49 PM.
----- Time-Dependent Solver 1 in Oxygen Pulse/Solution 958 (sol958) ----------->
```

k0Sub=1.51E-7, d=2.4E-6 (su41)

GENERAL

Description	Value
Solution	k0Sub=1.51E-7, d=2.4E-6

LOG

```
<---- Compile Equations: Stationary in Oxygen Pulse/Solution 958 (sol958) ------
Started at May 28, 2023, 4:14:52 PM.
Parameter k0Sub = 1.51E-7 (m/s).
Parameter d = 2.4E-6 (m).
Geometry shape function: Linear Lagrange
Number of vertex elements: 10
Minimum element quality: 0.4544
Time: 1 s.
Physical memory: 1.22 GB
Virtual memory: 1.2 GB
Ended at May 28, 2023, 4:14:53 PM.
----- Compile Equations: Stationary in Oxygen Pulse/Solution 958 (sol958) ----->

<---- Dependent Variables 1 in Oxygen Pulse/Solution 958 (sol958) --------------
Started at May 28, 2023, 4:14:53 PM.
Solution time: 0 s.
Physical memory: 1.22 GB
Virtual memory: 1.2 GB
Ended at May 28, 2023, 4:14:53 PM.
----- Dependent Variables 1 in Oxygen Pulse/Solution 958 (sol958) ------------->

<---- Stationary Solver 1 in Oxygen Pulse/Solution 958 (sol958) ----------------
Started at May 28, 2023, 4:14:53 PM.
Linear solver
Number of degrees of freedom solved for: 18002 (plus 937 internal DOFs).
Symmetric matrices found.
Scales for dependent variables:
Concentration (comp2.Oxi): 0.23
Orthonormal null-space function used.
Iter      SolEst     Damping    Stepsize #Res #Jac #Sol   LinErr   LinRes
   1     0.00012   1.0000000     0.00012    1    1    1  4.7e-13  9.6e-14
Solution time: 1 s.
Physical memory: 1.22 GB
Virtual memory: 1.2 GB
Ended at May 28, 2023, 4:14:53 PM.
----- Stationary Solver 1 in Oxygen Pulse/Solution 958 (sol958) --------------->

<---- Compile Equations: Oxygen Probe Pulse in Oxygen Pulse/Solution 958
      (sol958) ----------------------------------------------------------------
Started at May 28, 2023, 4:14:53 PM.
Geometry shape function: Linear Lagrange
Time: 1 s.
Physical memory: 1.2 GB
Virtual memory: 1.17 GB
Ended at May 28, 2023, 4:14:54 PM.
----- Compile Equations: Oxygen Probe Pulse in Oxygen Pulse/Solution 958
      (sol958) --------------------------------------------------------------->

<---- Dependent Variables 2 in Oxygen Pulse/Solution 958 (sol958) --------------
Started at May 28, 2023, 4:14:54 PM.
Initial values of variables solved for: Solution 958 (sol958), Solution Store 1 (sol
985).
Values of variables not solved for: Solution 958 (sol958), Solution Store 1 (sol985)
.
Solution time: 0 s.
Physical memory: 1.2 GB
```

```
Virtual memory: 1.18 GB
Ended at May 28, 2023, 4:14:54 PM.
----- Dependent Variables 2 in Oxygen Pulse/Solution 958 (sol958) ------------->

<---- Time-Dependent Solver 1 in Oxygen Pulse/Solution 958 (sol958) ------------
Started at May 28, 2023, 4:14:54 PM.
Time-dependent solver (BDF)
Number of degrees of freedom solved for: 18002 (plus 36084 internal DOFs).
Symmetric matrices found.
Scales for dependent variables:
Concentration (comp2.Oxi): 0.23
Step       Time    Stepsize      Res  Jac  Sol Order Tfail NLfail   LinErr   LinRes
   0          0          - out     5    3    5                 0  6.4e-16  1.5e-15
   1 2.5895e-08  2.5895e-08        7    4    7     1     0      0  3.6e-16  1.1e-15
   2 5.1789e-08  2.5895e-08        9    5    9     1     0      0  3.5e-16  1.2e-15
   3 1.0358e-07  5.1789e-08       11    6   11     2     0      0  3.8e-16  1.3e-15
   4 2.0716e-07  1.0358e-07       13    7   13     2     0      0  4.9e-16  1.7e-15
   5 4.1431e-07  2.0716e-07       15    8   15     2     0      0  6.5e-16  3.4e-15
   6 6.2147e-07  2.0716e-07       17    9   17     2     0      0  7.5e-16  3.9e-15
   7 8.2863e-07  2.0716e-07       19   10   19     2     0      0  5.8e-16  4.1e-15
   8 1.0358e-06  2.0716e-07       21   11   21     2     0      0  5.9e-16    4e-15
   9 1.4501e-06  4.1431e-07       23   12   23     2     0      0  8.9e-16  6.3e-15
  10 1.8644e-06  4.1431e-07       25   13   25     2     0      0  8.8e-16  6.1e-15
  11 2.2787e-06  4.1431e-07       27   14   27     2     0      0  7.7e-16  5.9e-15
  12  2.693e-06  4.1431e-07       29   15   29     2     0      0  8.4e-16  5.8e-15
  13 3.5217e-06  8.2863e-07       31   16   31     2     0      0  1.2e-15  8.3e-15
  14 4.3503e-06  8.2863e-07       33   17   33     2     0      0  1.3e-15  7.3e-15
  15 5.1789e-06  8.2863e-07       35   18   35     2     0      0  1.1e-15  7.3e-15
  16 6.0076e-06  8.2863e-07       37   19   37     2     0      0  1.1e-15  6.6e-15
  17 7.6648e-06  1.6573e-06       39   20   39     2     0      0  1.3e-15    1e-14
  18 9.3221e-06  1.6573e-06       41   21   41     2     0      0  1.9e-15  8.7e-15
  19 1.0979e-05  1.6573e-06       43   22   43     2     0      0  1.3e-15  8.5e-15
  20 1.2637e-05  1.6573e-06       45   23   45     2     0      0  1.7e-15    8e-15
  21 1.4294e-05  1.6573e-06       47   24   47     2     0      0  1.6e-15  6.9e-15
  22 1.7608e-05  3.3145e-06       49   25   49     2     0      0  1.8e-15  9.7e-15
  23 2.0923e-05  3.3145e-06       51   26   51     2     0      0  1.6e-15  8.7e-15
  24 2.4237e-05  3.3145e-06       53   27   53     2     0      0  1.9e-15  8.4e-15
  25 2.7552e-05  3.3145e-06       55   28   55     2     0      0  1.5e-15  8.1e-15
  26 3.4181e-05   6.629e-06       57   29   57     2     0      0  2.6e-15  1.2e-14
  27  4.081e-05   6.629e-06       59   30   59     2     0      0  1.9e-15  1.1e-14
  28 4.7439e-05   6.629e-06       61   31   61     2     0      0  1.3e-15    1e-14
  29 5.4068e-05   6.629e-06       63   32   63     2     0      0  2.3e-15  8.9e-15
  30 6.0697e-05   6.629e-06       65   33   65     2     0      0  1.6e-15  8.2e-15
  31 7.3955e-05  1.3258e-05       67   34   67     2     0      0  1.7e-15  1.2e-14
  32 8.7213e-05  1.3258e-05       69   35   69     2     0      0    3e-15    1e-14
  33 0.00010047  1.3258e-05       71   36   71     2     0      0  1.8e-15    1e-14
  34 0.00011373  1.3258e-05       73   37   73     2     0      0  1.7e-15  9.6e-15
  35 0.00014025  2.6516e-05       75   38   75     2     0      0  1.6e-15  1.4e-14
  36 0.00016676  2.6516e-05       77   39   77     2     0      0  2.9e-15  1.1e-14
  37 0.00019328  2.6516e-05       79   40   79     2     0      0  1.5e-15  1.2e-14
  38 0.00021979  2.6516e-05       81   41   81     2     0      0  2.7e-15  1.1e-14
  39 0.00027283  5.3032e-05       83   42   83     2     0      0  1.8e-15  1.5e-14
  40 0.00032586  5.3032e-05       85   43   85     2     0      0    2e-15  1.3e-14
  41 0.00037889  5.3032e-05       87   44   87     2     0      0  2.2e-15  1.3e-14
  42 0.00048495  0.00010606       89   45   89     2     0      0  2.3e-15  1.9e-14
  43 0.00059102  0.00010606       91   46   91     2     0      0  2.1e-15  1.5e-14
```

44	0.00069708	0.00010606		93	47	93	2	0	0	4.7e-15	1.5e-14
45	0.00080315	0.00010606		95	48	95	2	0	0	3.1e-15	1.3e-14
46	0.0010153	0.00021213		97	49	97	2	0	0	1.9e-15	1.9e-14
47	0.0012274	0.00021213		99	50	99	2	0	0	1.7e-15	1.5e-14
48	0.0016517	0.00042426		101	51	101	2	0	0	3.9e-15	2.6e-14
49	0.0020759	0.00042426		103	52	103	2	0	0	4.3e-15	2.5e-14
50	0.0025002	0.00042426		105	53	105	2	0	0	6e-15	2.5e-14
51	0.0029244	0.00042426		107	54	107	2	0	0	1.1e-14	2.1e-14
52	0.003773	0.00084852		109	55	109	2	0	0	2.7e-15	2.6e-14
53	0.0046215	0.00084852		111	56	111	2	0	0	2.4e-15	2e-14
-	0.005	-	out								
54	0.0063185	0.001697		113	57	113	2	0	0	4e-15	3.2e-14
55	0.0080155	0.001697		115	58	115	2	0	0	5e-15	3.4e-14
-	0.01	-	out								
56	0.01141	0.0033941		117	59	117	2	0	0	2.1e-15	5.2e-14
57	0.014804	0.0033941		119	60	119	2	0	0	2.9e-15	4.8e-14
-	0.015	-	out								
58	0.018198	0.0033941		121	61	121	2	0	0	4.2e-15	4.9e-14
-	0.02	-	out								
59	0.024986	0.0067881		123	62	123	2	0	0	3.9e-15	5.9e-14
-	0.025	-	out								
-	0.03	-	out								
60	0.031774	0.0067881		125	63	125	2	0	0	8.6e-15	4.5e-14
-	0.035	-	out								
-	0.04	-	out								
-	0.045	-	out								
61	0.04535	0.013576		127	64	127	2	0	0	6.8e-15	7.8e-14
-	0.05	-	out								
-	0.055	-	out								
62	0.058926	0.013576		129	65	129	2	0	0	4.2e-15	6.6e-14
-	0.06	-	out								
-	0.065	-	out								
-	0.07	-	out								
63	0.072503	0.013576		131	66	131	2	0	0	6.3e-15	6.6e-14
-	0.075	-	out								
-	0.08	-	out								
-	0.085	-	out								
64	0.086079	0.013576		133	67	133	2	0	0	4.6e-15	4.9e-14
-	0.09	-	out								
-	0.095	-	out								
-	0.1	-	out								
-	0.105	-	out								
-	0.11	-	out								
65	0.11323	0.027152		135	68	135	2	0	0	3.2e-15	4.8e-14
-	0.115	-	out								
-	0.12	-	out								
-	0.125	-	out								
-	0.13	-	out								
-	0.135	-	out								
-	0.14	-	out								
-	0.145	-	out								
-	0.15	-	out								
-	0.155	-	out								
-	0.16	-	out								
-	0.165	-	out								
66	0.16754	0.054305		137	69	137	1	0	0	6.6e-15	5.1e-14

-	0.17	-	out							
-	0.175	-	out							
-	0.18	-	out							
-	0.185	-	out							
-	0.19	-	out							
-	0.195	-	out							
-	0.2	-	out							
-	0.205	-	out							
-	0.21	-	out							
-	0.215	-	out							
-	0.22	-	out							
67	0.22184	0.054305	139	70	139	1	0	0	1.3e-14	3.5e-14
-	0.225	-	out							
-	0.23	-	out							
-	0.235	-	out							
-	0.24	-	out							
-	0.245	-	out							
-	0.25	-	out							
-	0.25	-	out							
-	0.27	-	out							
-	0.29	-	out							
-	0.31	-	out							
68	0.32084	0.099	141	71	141	1	0	0	6e-15	4.2e-14
-	0.33	-	out							
-	0.35	-	out							
-	0.37	-	out							
-	0.39	-	out							
-	0.41	-	out							
69	0.41984	0.099	143	72	143	1	0	0	7.1e-15	3.6e-14
-	0.43	-	out							
-	0.45	-	out							
-	0.47	-	out							
-	0.49	-	out							
-	0.51	-	out							
70	0.51884	0.099	145	73	145	1	0	0	7.7e-15	3e-14
-	0.53	-	out							
-	0.55	-	out							
-	0.57	-	out							
-	0.59	-	out							
-	0.61	-	out							
71	0.61784	0.099	147	74	147	1	0	0	1.3e-14	2.6e-14
-	0.63	-	out							
-	0.65	-	out							
-	0.67	-	out							
-	0.69	-	out							
-	0.71	-	out							
72	0.71684	0.099	148	75	148	1	0	0	1e-14	2e-14
-	0.73	-	out							
-	0.75	-	out							
-	0.77	-	out							
-	0.79	-	out							
-	0.81	-	out							
73	0.81584	0.099	149	76	149	1	0	0	8.4e-15	1.9e-14
-	0.83	-	out							
-	0.85	-	out							
-	0.87	-	out							

```
   -        0.89           - out
   -        0.91           - out
  74     0.91484       0.099      150    77   150      2      0       0  2.4e-14   1.6e-14
   -        0.93           - out
   -        0.95           - out
   -        0.97           - out
   -        0.99           - out
  75      1.0138       0.099      151    78   151      2      0       0  8.2e-15   9.9e-15
Time-stepping completed.
Solution time: 15 s.
Physical memory: 1.24 GB
Virtual memory: 1.22 GB
Ended at May 28, 2023, 4:15:09 PM.
----- Time-Dependent Solver 1 in Oxygen Pulse/Solution 958 (sol958) ----------->
```

k0Sub=1.51E-7, d=3E-6 (su42)

GENERAL

Description	Value
Solution	k0Sub=1.51E-7, d=3E-6

LOG

```
<---- Compile Equations: Stationary in Oxygen Pulse/Solution 958 (sol958) ------
Started at May 28, 2023, 4:15:12 PM.
Parameter k0Sub = 1.51E-7 (m/s).
Parameter d = 3.0E-6 (m).
Geometry shape function: Linear Lagrange
Number of vertex elements: 10
Minimum element quality: 0.4275
Time: 1 s.
Physical memory: 1.22 GB
Virtual memory: 1.2 GB
Ended at May 28, 2023, 4:15:13 PM.
----- Compile Equations: Stationary in Oxygen Pulse/Solution 958 (sol958) ----->

<---- Dependent Variables 1 in Oxygen Pulse/Solution 958 (sol958) --------------
Started at May 28, 2023, 4:15:13 PM.
Solution time: 0 s.
Physical memory: 1.22 GB
Virtual memory: 1.2 GB
Ended at May 28, 2023, 4:15:13 PM.
----- Dependent Variables 1 in Oxygen Pulse/Solution 958 (sol958) ------------->

<---- Stationary Solver 1 in Oxygen Pulse/Solution 958 (sol958) ----------------
Started at May 28, 2023, 4:15:13 PM.
Linear solver
Number of degrees of freedom solved for: 22990 (plus 935 internal DOFs).
Symmetric matrices found.
Scales for dependent variables:
Concentration (comp2.Oxi): 0.23
Orthonormal null-space function used.
Iter      SolEst     Damping    Stepsize #Res #Jac #Sol   LinErr   LinRes
   1     9.5e-05   1.0000000     9.5e-05    1    1    1  4.2e-13  9.2e-14
Solution time: 1 s.
Physical memory: 1.23 GB
Virtual memory: 1.21 GB
Ended at May 28, 2023, 4:15:13 PM.
----- Stationary Solver 1 in Oxygen Pulse/Solution 958 (sol958) --------------->

<---- Compile Equations: Oxygen Probe Pulse in Oxygen Pulse/Solution 958
      (sol958) -----------------------------------------------------------------
Started at May 28, 2023, 4:15:13 PM.
Geometry shape function: Linear Lagrange
Time: 1 s.
Physical memory: 1.23 GB
Virtual memory: 1.21 GB
Ended at May 28, 2023, 4:15:14 PM.
----- Compile Equations: Oxygen Probe Pulse in Oxygen Pulse/Solution 958
      (sol958) ---------------------------------------------------------------->

<---- Dependent Variables 2 in Oxygen Pulse/Solution 958 (sol958) --------------
Started at May 28, 2023, 4:15:14 PM.
Initial values of variables solved for: Solution 958 (sol958), Solution Store 1 (sol
985).
Values of variables not solved for: Solution 958 (sol958), Solution Store 1 (sol985)
.
Solution time: 0 s.
Physical memory: 1.2 GB
```

```
Virtual memory: 1.18 GB
Ended at May 28, 2023, 4:15:14 PM.
----- Dependent Variables 2 in Oxygen Pulse/Solution 958 (sol958) ------------->

<---- Time-Dependent Solver 1 in Oxygen Pulse/Solution 958 (sol958) ------------
Started at May 28, 2023, 4:15:14 PM.
Time-dependent solver (BDF)
Number of degrees of freedom solved for: 22990 (plus 46056 internal DOFs).
Symmetric matrices found.
Scales for dependent variables:
Concentration (comp2.Oxi): 0.23
Step        Time    Stepsize      Res  Jac  Sol Order Tfail NLfail   LinErr   LinRes
   0           0           - out    5    3    5                 0  6.5e-16    9e-16
   1  3.5551e-08  3.5551e-08        7    4    7     1     0      0  3.1e-16  9.4e-16
   2  7.1102e-08  3.5551e-08        9    5    9     1     0      0  3.3e-16  9.7e-16
   3   1.422e-07  7.1102e-08       11    6   11     2     0      0  3.8e-16    1e-15
   4  2.8441e-07   1.422e-07       13    7   13     2     0      0  3.8e-16  1.3e-15
   5  5.6881e-07  2.8441e-07       15    8   15     2     0      0  5.3e-16  2.3e-15
   6  8.5322e-07  2.8441e-07       17    9   17     2     0      0  5.8e-16  2.9e-15
   7  1.1376e-06  2.8441e-07       19   10   19     2     0      0  4.6e-16  3.1e-15
   8   1.422e-06  2.8441e-07       21   11   21     2     0      0  6.2e-16  3.2e-15
   9  1.9908e-06  5.6881e-07       23   12   23     2     0      0  7.4e-16  5.2e-15
  10  2.5597e-06  5.6881e-07       25   13   25     2     0      0  6.4e-16  4.7e-15
  11  3.1285e-06  5.6881e-07       27   14   27     2     0      0  7.1e-16  4.8e-15
  12  3.6973e-06  5.6881e-07       29   15   29     2     0      0  6.7e-16  4.5e-15
  13  4.8349e-06  1.1376e-06       31   16   31     2     0      0  7.4e-16  6.9e-15
  14  5.9725e-06  1.1376e-06       33   17   33     2     0      0  8.5e-16  6.3e-15
  15  7.1102e-06  1.1376e-06       35   18   35     2     0      0  8.3e-16  6.2e-15
  16  8.2478e-06  1.1376e-06       37   19   37     2     0      0  9.1e-16    6e-15
  17  1.0523e-05  2.2752e-06       39   20   39     2     0      0  9.7e-16  8.8e-15
  18  1.2798e-05  2.2752e-06       41   21   41     2     0      0  1.3e-15  7.9e-15
  19  1.5074e-05  2.2752e-06       43   22   43     2     0      0  9.2e-16  7.9e-15
  20  1.7349e-05  2.2752e-06       45   23   45     2     0      0    1e-15  7.4e-15
  21  2.1899e-05  4.5505e-06       47   24   47     2     0      0  1.2e-15  1.1e-14
  22   2.645e-05  4.5505e-06       49   25   49     2     0      0  1.6e-15  9.7e-15
  23     3.1e-05  4.5505e-06       51   26   51     2     0      0  1.1e-15  9.8e-15
  24  3.5551e-05  4.5505e-06       53   27   53     2     0      0  1.7e-15  8.9e-15
  25  4.4652e-05   9.101e-06       55   28   55     2     0      0  2.4e-15  1.3e-14
  26  5.3753e-05   9.101e-06       57   29   57     2     0      0  1.2e-15  1.2e-14
  27  6.2854e-05   9.101e-06       59   30   59     2     0      0  1.4e-15  1.2e-14
  28  7.1955e-05   9.101e-06       61   31   61     2     0      0  1.4e-15  1.1e-14
  29  9.0157e-05  1.8202e-05       63   32   63     2     0      0  1.7e-15  1.6e-14
  30  0.00010836  1.8202e-05       65   33   65     2     0      0  1.2e-15  1.3e-14
  31  0.00012656  1.8202e-05       67   34   67     2     0      0  1.8e-15  1.4e-14
  32  0.00014476  1.8202e-05       69   35   69     2     0      0  1.6e-15  1.2e-14
  33  0.00018117  3.6404e-05       71   36   71     2     0      0  2.8e-15  1.8e-14
  34  0.00021757  3.6404e-05       73   37   73     2     0      0  2.1e-15  1.5e-14
  35  0.00025397  3.6404e-05       75   38   75     2     0      0  1.6e-15  1.5e-14
  36  0.00029038  3.6404e-05       77   39   77     2     0      0  2.3e-15  1.4e-14
  37  0.00036319  7.2808e-05       79   40   79     2     0      0  1.8e-15    2e-14
  38  0.00043599  7.2808e-05       81   41   81     2     0      0  2.3e-15  1.8e-14
  39  0.00058161  0.00014562       83   42   83     2     0      0  2.2e-15  2.7e-14
  40  0.00072723  0.00014562       85   43   85     2     0      0  1.8e-15  2.4e-14
  41  0.00087284  0.00014562       87   44   87     2     0      0    2e-15  2.3e-14
  42   0.0010185  0.00014562       89   45   89     2     0      0  5.5e-15    2e-14
  43   0.0013097  0.00029123       91   46   91     2     0      0  4.5e-15  2.6e-14
```

44	0.0016009	0.00029123		93	47	93	2	0	0	3.7e-15	2e-14
45	0.0021834	0.00058246		95	48	95	2	0	0	8.5e-15	3.4e-14
46	0.0027659	0.00058246		97	49	97	2	0	0	2.4e-15	3.5e-14
47	0.0033483	0.00058246		99	50	99	2	0	0	5.6e-15	3.4e-14
48	0.0045132	0.0011649		101	51	101	2	0	0	3.2e-15	4.2e-14
-	0.005	-	out								
49	0.0056782	0.0011649		103	52	103	2	0	0	3.2e-15	2.8e-14
50	0.008008	0.0023299		105	53	105	2	0	0	3.8e-15	4.8e-14
-	0.01	-	out								
51	0.010338	0.0023299		107	54	107	2	0	0	4.2e-15	4.5e-14
52	0.012668	0.0023299		109	55	109	2	0	0	2.6e-15	4.7e-14
-	0.015	-	out								
53	0.017327	0.0046597		111	56	111	2	0	0	3.4e-15	6.5e-14
-	0.02	-	out								
54	0.021987	0.0046597		113	57	113	2	0	0	7.7e-15	4.8e-14
-	0.025	-	out								
-	0.03	-	out								
55	0.031307	0.0093194		115	58	115	2	0	0	7.7e-15	8.1e-14
-	0.035	-	out								
-	0.04	-	out								
56	0.040626	0.0093194		117	59	117	2	0	0	4e-15	7.2e-14
-	0.045	-	out								
57	0.049945	0.0093194		119	60	119	2	0	0	1e-14	7.5e-14
-	0.05	-	out								
-	0.055	-	out								
-	0.06	-	out								
-	0.065	-	out								
58	0.068584	0.018639		121	61	121	2	0	0	7.6e-15	1e-13
-	0.07	-	out								
-	0.075	-	out								
-	0.08	-	out								
-	0.085	-	out								
59	0.087223	0.018639		123	62	123	1	0	0	8.7e-15	8.6e-14
-	0.09	-	out								
-	0.095	-	out								
-	0.1	-	out								
-	0.105	-	out								
-	0.11	-	out								
-	0.115	-	out								
-	0.12	-	out								
60	0.1245	0.037278		125	63	125	1	0	0	8.6e-15	6.6e-14
-	0.125	-	out								
-	0.13	-	out								
-	0.135	-	out								
-	0.14	-	out								
-	0.145	-	out								
-	0.15	-	out								
-	0.155	-	out								
-	0.16	-	out								
-	0.165	-	out								
-	0.17	-	out								
-	0.175	-	out								
-	0.18	-	out								
-	0.185	-	out								
-	0.19	-	out								
-	0.195	-	out								

61	0.19906	0.074555		127	64	127	1	0	0	1.2e-14	8.5e-14
-	0.2	-	out								
-	0.205	-	out								
-	0.21	-	out								
-	0.215	-	out								
-	0.22	-	out								
-	0.225	-	out								
-	0.23	-	out								
-	0.235	-	out								
-	0.24	-	out								
-	0.245	-	out								
-	0.25	-	out								
-	0.25	-	out								
-	0.27	-	out								
62	0.27361	0.074555		129	65	129	1	0	0	5.9e-15	7.4e-14
-	0.29	-	out								
-	0.31	-	out								
-	0.33	-	out								
-	0.35	-	out								
-	0.37	-	out								
63	0.37261	0.099		131	66	131	1	0	0	1.5e-14	7e-14
-	0.39	-	out								
-	0.41	-	out								
-	0.43	-	out								
-	0.45	-	out								
-	0.47	-	out								
64	0.47161	0.099		133	67	133	1	0	0	6.1e-15	5.6e-14
-	0.49	-	out								
-	0.51	-	out								
-	0.53	-	out								
-	0.55	-	out								
-	0.57	-	out								
65	0.57061	0.099		135	68	135	1	0	0	5e-15	4.3e-14
-	0.59	-	out								
-	0.61	-	out								
-	0.63	-	out								
-	0.65	-	out								
66	0.66961	0.099		136	69	136	1	0	0	7.3e-15	3.4e-14
-	0.67	-	out								
-	0.69	-	out								
-	0.71	-	out								
-	0.73	-	out								
-	0.75	-	out								
67	0.76861	0.099		137	70	137	1	0	0	3.5e-15	3.1e-14
-	0.77	-	out								
-	0.79	-	out								
-	0.81	-	out								
-	0.83	-	out								
-	0.85	-	out								
68	0.86761	0.099		138	71	138	2	0	0	1.1e-14	2.6e-14
-	0.87	-	out								
-	0.89	-	out								
-	0.91	-	out								
-	0.93	-	out								
-	0.95	-	out								
69	0.96661	0.099		139	72	139	2	0	0	1.6e-14	1.7e-14

```
    -         0.97           - out
    -         0.99           - out
   70       1.0656       0.099      140   73  140      2     0      0  1.2e-14  1.6e-14
Time-stepping completed.
Solution time: 19 s.
Physical memory: 1.25 GB
Virtual memory: 1.23 GB
Ended at May 28, 2023, 4:15:33 PM.
----- Time-Dependent Solver 1 in Oxygen Pulse/Solution 958 (sol958) ----------->
```

k0Sub=1.51E-7, d=4E-6 (su43)

GENERAL

Description	Value
Solution	k0Sub=1.51E-7, d=4E-6

LOG

```
<---- Compile Equations: Stationary in Oxygen Pulse/Solution 958 (sol958) ------
Started at May 28, 2023, 4:15:36 PM.
Parameter k0Sub = 1.51E-7 (m/s).
Parameter d = 4.0E-6 (m).
Geometry shape function: Linear Lagrange
Number of vertex elements: 10
Minimum element quality: 0.4317
Time: 1 s.
Physical memory: 1.23 GB
Virtual memory: 1.23 GB
Ended at May 28, 2023, 4:15:36 PM.
----- Compile Equations: Stationary in Oxygen Pulse/Solution 958 (sol958) ----->

<---- Dependent Variables 1 in Oxygen Pulse/Solution 958 (sol958) --------------
Started at May 28, 2023, 4:15:36 PM.
Solution time: 0 s.
Physical memory: 1.23 GB
Virtual memory: 1.23 GB
Ended at May 28, 2023, 4:15:36 PM.
----- Dependent Variables 1 in Oxygen Pulse/Solution 958 (sol958) ------------->

<---- Stationary Solver 1 in Oxygen Pulse/Solution 958 (sol958) ----------------
Started at May 28, 2023, 4:15:36 PM.
Linear solver
Number of degrees of freedom solved for: 18182 (plus 936 internal DOFs).
Symmetric matrices found.
Scales for dependent variables:
Concentration (comp2.Oxi): 0.23
Orthonormal null-space function used.
Iter      SolEst     Damping    Stepsize #Res #Jac #Sol   LinErr   LinRes
   1     9.2e-05   1.0000000     9.2e-05    1    1    1  3.4e-13  7.6e-14
Solution time: 1 s.
Physical memory: 1.24 GB
Virtual memory: 1.23 GB
Ended at May 28, 2023, 4:15:37 PM.
----- Stationary Solver 1 in Oxygen Pulse/Solution 958 (sol958) --------------->

<---- Compile Equations: Oxygen Probe Pulse in Oxygen Pulse/Solution 958
      (sol958) ----------------------------------------------------------------
Started at May 28, 2023, 4:15:37 PM.
Geometry shape function: Linear Lagrange
Time: 1 s.
Physical memory: 1.25 GB
Virtual memory: 1.24 GB
Ended at May 28, 2023, 4:15:38 PM.
----- Compile Equations: Oxygen Probe Pulse in Oxygen Pulse/Solution 958
      (sol958) --------------------------------------------------------------->

<---- Dependent Variables 2 in Oxygen Pulse/Solution 958 (sol958) --------------
Started at May 28, 2023, 4:15:38 PM.
Initial values of variables solved for: Solution 958 (sol958), Solution Store 1 (sol
985).
Values of variables not solved for: Solution 958 (sol958), Solution Store 1 (sol985)
.
Solution time: 0 s.
Physical memory: 1.25 GB
```

Virtual memory: 1.24 GB
Ended at May 28, 2023, 4:15:38 PM.
----- Dependent Variables 2 in Oxygen Pulse/Solution 958 (sol958) ------------->

<---- Time-Dependent Solver 1 in Oxygen Pulse/Solution 958 (sol958) ------------
Started at May 28, 2023, 4:15:38 PM.
Time-dependent solver (BDF)
Number of degrees of freedom solved for: 18182 (plus 36438 internal DOFs).
Symmetric matrices found.
Scales for dependent variables:
Concentration (comp2.Oxi): 0.23

Step	Time	Stepsize		Res	Jac	Sol	Order	Tfail	NLfail	LinErr	LinRes
0	0	-	out	5	3	5			0	7.7e-16	1.5e-15
1	2.6141e-08	2.6141e-08		7	4	7	1	0	0	3.8e-16	1.1e-15
2	5.2282e-08	2.6141e-08		9	5	9	1	0	0	3.5e-16	1.2e-15
3	1.0456e-07	5.2282e-08		11	6	11	2	0	0	3.8e-16	1.3e-15
4	2.0913e-07	1.0456e-07		13	7	13	2	0	0	4.3e-16	1.7e-15
5	4.1826e-07	2.0913e-07		15	8	15	2	0	0	6.1e-16	3.7e-15
6	6.2739e-07	2.0913e-07		17	9	17	2	0	0	5.6e-16	3.7e-15
7	8.3651e-07	2.0913e-07		19	10	19	2	0	0	5.8e-16	3.9e-15
8	1.0456e-06	2.0913e-07		21	11	21	2	0	0	6.2e-16	3.9e-15
9	1.4639e-06	4.1826e-07		23	12	23	2	0	0	7.8e-16	6.3e-15
10	1.8822e-06	4.1826e-07		25	13	25	2	0	0	9.2e-16	6e-15
11	2.3004e-06	4.1826e-07		27	14	27	2	0	0	8.4e-16	6e-15
12	2.7187e-06	4.1826e-07		29	15	29	2	0	0	7.3e-16	5.6e-15
13	3.5552e-06	8.3651e-07		31	16	31	2	0	0	1.3e-15	8.4e-15
14	4.3917e-06	8.3651e-07		33	17	33	2	0	0	1.4e-15	7.3e-15
15	5.2282e-06	8.3651e-07		35	18	35	2	0	0	1.2e-15	7.3e-15
16	6.0647e-06	8.3651e-07		37	19	37	2	0	0	1.2e-15	6.6e-15
17	7.7378e-06	1.673e-06		39	20	39	2	0	0	1.2e-15	9.8e-15
18	9.4108e-06	1.673e-06		41	21	41	2	0	0	1.1e-15	8.3e-15
19	1.1084e-05	1.673e-06		43	22	43	2	0	0	1.1e-15	8.1e-15
20	1.2757e-05	1.673e-06		45	23	45	2	0	0	1.4e-15	7.5e-15
21	1.443e-05	1.673e-06		47	24	47	2	0	0	1e-15	6.7e-15
22	1.7776e-05	3.3461e-06		49	25	49	2	0	0	1.4e-15	9.8e-15
23	2.1122e-05	3.3461e-06		51	26	51	2	0	0	1.3e-15	8.6e-15
24	2.4468e-05	3.3461e-06		53	27	53	2	0	0	1.5e-15	8.6e-15
25	2.7814e-05	3.3461e-06		55	28	55	2	0	0	1.9e-15	8e-15
26	3.4506e-05	6.6921e-06		57	29	57	2	0	0	2.5e-15	1.2e-14
27	4.1198e-05	6.6921e-06		59	30	59	2	0	0	1.8e-15	9.9e-15
28	4.789e-05	6.6921e-06		61	31	61	2	0	0	1.7e-15	9.2e-15
29	5.4583e-05	6.6921e-06		63	32	63	2	0	0	1.9e-15	8.9e-15
30	6.1275e-05	6.6921e-06		65	33	65	2	0	0	2.1e-15	7.9e-15
31	7.4659e-05	1.3384e-05		67	34	67	2	0	0	1.4e-15	1.2e-14
32	8.8043e-05	1.3384e-05		69	35	69	2	0	0	1.4e-15	1.1e-14
33	0.00010143	1.3384e-05		71	36	71	2	0	0	2.2e-15	1e-14
34	0.00011481	1.3384e-05		73	37	73	2	0	0	2.1e-15	9.8e-15
35	0.00014158	2.6768e-05		75	38	75	2	0	0	1.9e-15	1.4e-14
36	0.00016835	2.6768e-05		77	39	77	2	0	0	1.4e-15	1.2e-14
37	0.00019512	2.6768e-05		79	40	79	2	0	0	2.5e-15	1.2e-14
38	0.00022189	2.6768e-05		81	41	81	2	0	0	2.3e-15	1.1e-14
39	0.00027542	5.3537e-05		83	42	83	2	0	0	4.4e-15	1.6e-14
40	0.00032896	5.3537e-05		85	43	85	2	0	0	1.9e-15	1.3e-14
41	0.0003825	5.3537e-05		87	44	87	2	0	0	4.1e-15	1.3e-14
42	0.00048957	0.00010707		89	45	89	2	0	0	2.9e-15	1.9e-14
43	0.00059664	0.00010707		91	46	91	2	0	0	1.9e-15	1.6e-14

44	0.00070372	0.00010707		93	47	93	2	0	0	1.8e-15	1.6e-14
45	0.00081079	0.00010707		95	48	95	2	0	0	1.5e-15	1.4e-14
46	0.0010249	0.00021415		97	49	97	2	0	0	1.8e-15	2e-14
47	0.0012391	0.00021415		99	50	99	2	0	0	2.3e-15	1.6e-14
48	0.0016674	0.0004283		101	51	101	2	0	0	5.1e-15	2.5e-14
49	0.0020957	0.0004283		103	52	103	2	0	0	4.7e-15	2.3e-14
50	0.002524	0.0004283		105	53	105	2	0	0	2.9e-15	2.3e-14
51	0.0029523	0.0004283		107	54	107	2	0	0	9.8e-15	2.1e-14
52	0.0038089	0.00085659		109	55	109	2	0	0	3.3e-15	2.7e-14
53	0.0046654	0.00085659		111	56	111	2	0	0	4.4e-15	2.1e-14
-	0.005	-	out								
54	0.0063786	0.0017132		113	57	113	2	0	0	2.8e-15	3.3e-14
55	0.0080918	0.0017132		115	58	115	2	0	0	5.3e-15	3e-14
-	0.01	-	out								
56	0.011518	0.0034264		117	59	117	2	0	0	5.1e-15	4.5e-14
57	0.014945	0.0034264		119	60	119	1	0	0	2.2e-15	5.5e-14
-	0.015	-	out								
58	0.018371	0.0034264		121	61	121	1	0	0	6.3e-15	4.4e-14
-	0.02	-	out								
-	0.025	-	out								
59	0.025224	0.0068527		123	62	123	1	0	0	5.3e-15	7.3e-14
-	0.03	-	out								
60	0.032076	0.0068527		125	63	125	1	0	0	1.3e-14	6.7e-14
-	0.035	-	out								
-	0.04	-	out								
-	0.045	-	out								
61	0.045782	0.013705		127	64	127	1	0	0	1.3e-14	1e-13
-	0.05	-	out								
-	0.055	-	out								
62	0.059487	0.013705		129	65	129	1	0	0	6.1e-15	8.7e-14
-	0.06	-	out								
-	0.065	-	out								
-	0.07	-	out								
-	0.075	-	out								
-	0.08	-	out								
-	0.085	-	out								
63	0.086898	0.027411		131	66	131	1	0	0	4.6e-15	1.1e-13
-	0.09	-	out								
-	0.095	-	out								
-	0.1	-	out								
-	0.105	-	out								
-	0.11	-	out								
64	0.11431	0.027411		133	67	133	1	0	0	8.7e-15	8.2e-14
-	0.115	-	out								
-	0.12	-	out								
-	0.125	-	out								
-	0.13	-	out								
-	0.135	-	out								
-	0.14	-	out								
-	0.145	-	out								
-	0.15	-	out								
-	0.155	-	out								
-	0.16	-	out								
-	0.165	-	out								
65	0.16913	0.054822		135	68	135	1	0	0	1.8e-14	8.7e-14
-	0.17	-	out								

-	0.175	- out								
-	0.18	- out								
-	0.185	- out								
-	0.19	- out								
-	0.195	- out								
-	0.2	- out								
-	0.205	- out								
-	0.21	- out								
-	0.215	- out								
-	0.22	- out								
66	0.22395	0.054822	137	69	137	1	0	0	4.2e-15	6.4e-14
-	0.225	- out								
-	0.23	- out								
-	0.235	- out								
-	0.24	- out								
-	0.245	- out								
-	0.25	- out								
-	0.25	- out								
-	0.27	- out								
-	0.29	- out								
-	0.31	- out								
67	0.32295	0.099	139	70	139	1	0	0	7.8e-15	6.4e-14
-	0.33	- out								
-	0.35	- out								
-	0.37	- out								
-	0.39	- out								
-	0.41	- out								
68	0.42195	0.099	141	71	141	1	0	0	1.8e-14	5e-14
-	0.43	- out								
-	0.45	- out								
-	0.47	- out								
-	0.49	- out								
-	0.51	- out								
69	0.52095	0.099	143	72	143	1	0	0	4.8e-15	3.7e-14
-	0.53	- out								
-	0.55	- out								
-	0.57	- out								
-	0.59	- out								
-	0.61	- out								
70	0.61995	0.099	145	73	145	1	0	0	1.2e-14	2.9e-14
-	0.63	- out								
-	0.65	- out								
-	0.67	- out								
-	0.69	- out								
-	0.71	- out								
71	0.71895	0.099	146	74	146	1	0	0	4.1e-15	2.4e-14
-	0.73	- out								
-	0.75	- out								
-	0.77	- out								
-	0.79	- out								
-	0.81	- out								
72	0.81795	0.099	147	75	147	1	0	0	6.4e-15	2.1e-14
-	0.83	- out								
-	0.85	- out								
-	0.87	- out								
-	0.89	- out								

```
  -        0.91           - out
 73      0.91695        0.099      148   76  148     2     0      0  5.1e-15  1.8e-14
  -        0.93           - out
  -        0.95           - out
  -        0.97           - out
  -        0.99           - out
 74       1.016         0.099      149   77  149     2     0      0  3.5e-15  1.1e-14
Time-stepping completed.
Solution time: 15 s.
Physical memory: 1.27 GB
Virtual memory: 1.26 GB
Ended at May 28, 2023, 4:15:53 PM.
----- Time-Dependent Solver 1 in Oxygen Pulse/Solution 958 (sol958) ----------->
```

k0Sub=1.51E-7, d=5E-6 (su44)

GENERAL

Description	Value
Solution	k0Sub=1.51E-7, d=5E-6

LOG

```
<---- Compile Equations: Stationary in Oxygen Pulse/Solution 958 (sol958) ------
Started at May 28, 2023, 4:15:56 PM.
Parameter k0Sub = 1.51E-7 (m/s).
Parameter d = 4.9999999999999996E-6 (m).
Geometry shape function: Linear Lagrange
Number of vertex elements: 10
Minimum element quality: 0.4532
Time: 1 s.
Physical memory: 1.19 GB
Virtual memory: 1.17 GB
Ended at May 28, 2023, 4:15:56 PM.
----- Compile Equations: Stationary in Oxygen Pulse/Solution 958 (sol958) ----->

<---- Dependent Variables 1 in Oxygen Pulse/Solution 958 (sol958) --------------
Started at May 28, 2023, 4:15:56 PM.
Solution time: 0 s.
Physical memory: 1.19 GB
Virtual memory: 1.17 GB
Ended at May 28, 2023, 4:15:56 PM.
----- Dependent Variables 1 in Oxygen Pulse/Solution 958 (sol958) ------------->

<---- Stationary Solver 1 in Oxygen Pulse/Solution 958 (sol958) ----------------
Started at May 28, 2023, 4:15:56 PM.
Linear solver
Number of degrees of freedom solved for: 18304 (plus 936 internal DOFs).
Symmetric matrices found.
Scales for dependent variables:
Concentration (comp2.Oxi): 0.23
Orthonormal null-space function used.
Iter      SolEst     Damping    Stepsize #Res #Jac #Sol   LinErr   LinRes
   1     7.9e-05   1.0000000     7.9e-05    1    1    1  3.6e-13    7e-14
Solution time: 1 s.
Physical memory: 1.21 GB
Virtual memory: 1.19 GB
Ended at May 28, 2023, 4:15:57 PM.
----- Stationary Solver 1 in Oxygen Pulse/Solution 958 (sol958) --------------->

<---- Compile Equations: Oxygen Probe Pulse in Oxygen Pulse/Solution 958
      (sol958) -----------------------------------------------------------------
Started at May 28, 2023, 4:15:57 PM.
Geometry shape function: Linear Lagrange
Time: 0 s.
Physical memory: 1.22 GB
Virtual memory: 1.19 GB
Ended at May 28, 2023, 4:15:58 PM.
----- Compile Equations: Oxygen Probe Pulse in Oxygen Pulse/Solution 958
      (sol958) ---------------------------------------------------------------->

<---- Dependent Variables 2 in Oxygen Pulse/Solution 958 (sol958) --------------
Started at May 28, 2023, 4:15:58 PM.
Initial values of variables solved for: Solution 958 (sol958), Solution Store 1 (sol
985).
Values of variables not solved for: Solution 958 (sol958), Solution Store 1 (sol985)
.
Solution time: 0 s.
Physical memory: 1.22 GB
```

Virtual memory: 1.19 GB
Ended at May 28, 2023, 4:15:58 PM.
----- Dependent Variables 2 in Oxygen Pulse/Solution 958 (sol958) ------------->

<---- Time-Dependent Solver 1 in Oxygen Pulse/Solution 958 (sol958) ------------
Started at May 28, 2023, 4:15:58 PM.
Time-dependent solver (BDF)
Number of degrees of freedom solved for: 18304 (plus 36680 internal DOFs).
Symmetric matrices found.
Scales for dependent variables:
Concentration (comp2.Oxi): 0.23

Step	Time	Stepsize		Res	Jac	Sol	Order	Tfail	NLfail	LinErr	LinRes
0	0	-	out	5	3	5			0	9.5e-16	1.5e-15
1	2.6322e-08	2.6322e-08		7	4	7	1	0	0	3.3e-16	1.1e-15
2	5.2644e-08	2.6322e-08		9	5	9	1	0	0	3.8e-16	1.2e-15
3	1.0529e-07	5.2644e-08		11	6	11	2	0	0	3.7e-16	1.3e-15
4	2.1057e-07	1.0529e-07		13	7	13	2	0	0	4.7e-16	1.7e-15
5	4.2115e-07	2.1057e-07		15	8	15	2	0	0	7.2e-16	3.2e-15
6	6.3172e-07	2.1057e-07		17	9	17	2	0	0	5.7e-16	3.7e-15
7	8.423e-07	2.1057e-07		19	10	19	2	0	0	7.2e-16	4e-15
8	1.0529e-06	2.1057e-07		21	11	21	2	0	0	7.3e-16	3.9e-15
9	1.474e-06	4.2115e-07		23	12	23	2	0	0	8.1e-16	6.3e-15
10	1.8952e-06	4.2115e-07		25	13	25	2	0	0	7.3e-16	6e-15
11	2.3163e-06	4.2115e-07		27	14	27	2	0	0	9.1e-16	5.9e-15
12	2.7375e-06	4.2115e-07		29	15	29	2	0	0	8.1e-16	5.6e-15
13	3.5798e-06	8.423e-07		31	16	31	2	0	0	1e-15	8.6e-15
14	4.4221e-06	8.423e-07		33	17	33	2	0	0	1.1e-15	7.6e-15
15	5.2644e-06	8.423e-07		35	18	35	2	0	0	8.1e-16	7.4e-15
16	6.1067e-06	8.423e-07		37	19	37	2	0	0	8.5e-16	6.7e-15
17	7.7913e-06	1.6846e-06		39	20	39	2	0	0	1.1e-15	9.9e-15
18	9.4759e-06	1.6846e-06		41	21	41	2	0	0	1.5e-15	8.6e-15
19	1.116e-05	1.6846e-06		43	22	43	2	0	0	1.6e-15	8.3e-15
20	1.2845e-05	1.6846e-06		45	23	45	2	0	0	1.2e-15	7.5e-15
21	1.453e-05	1.6846e-06		47	24	47	2	0	0	1.3e-15	6.8e-15
22	1.7899e-05	3.3692e-06		49	25	49	2	0	0	1.9e-15	9.8e-15
23	2.1268e-05	3.3692e-06		51	26	51	2	0	0	1.2e-15	8.6e-15
24	2.4637e-05	3.3692e-06		53	27	53	2	0	0	1.6e-15	8.7e-15
25	2.8006e-05	3.3692e-06		55	28	55	2	0	0	1.4e-15	7.9e-15
26	3.4745e-05	6.7384e-06		57	29	57	2	0	0	1.9e-15	1.2e-14
27	4.1483e-05	6.7384e-06		59	30	59	2	0	0	2.2e-15	1e-14
28	4.8222e-05	6.7384e-06		61	31	61	2	0	0	1.8e-15	1e-14
29	5.496e-05	6.7384e-06		63	32	63	2	0	0	1.7e-15	9.1e-15
30	6.1698e-05	6.7384e-06		65	33	65	2	0	0	1.6e-15	8.2e-15
31	7.5175e-05	1.3477e-05		67	34	67	2	0	0	2.1e-15	1.2e-14
32	8.8652e-05	1.3477e-05		69	35	69	2	0	0	1.6e-15	1.1e-14
33	0.00010213	1.3477e-05		71	36	71	2	0	0	1.5e-15	1.1e-14
34	0.00011561	1.3477e-05		73	37	73	2	0	0	1.2e-15	9.7e-15
35	0.00014256	2.6954e-05		75	38	75	2	0	0	2.9e-15	1.5e-14
36	0.00016951	2.6954e-05		77	39	77	2	0	0	2.6e-15	1.3e-14
37	0.00019647	2.6954e-05		79	40	79	2	0	0	2.9e-15	1.3e-14
38	0.00022342	2.6954e-05		81	41	81	2	0	0	1.8e-15	1.2e-14
39	0.00027733	5.3907e-05		83	42	83	2	0	0	3e-15	1.6e-14
40	0.00033123	5.3907e-05		85	43	85	2	0	0	2.7e-15	1.4e-14
41	0.00038514	5.3907e-05		87	44	87	2	0	0	2.8e-15	1.3e-14
42	0.00049295	0.00010781		89	45	89	2	0	0	2.6e-15	1.9e-14
43	0.00060077	0.00010781		91	46	91	2	0	0	4.2e-15	1.6e-14

44	0.00070858	0.00010781		93	47	93	2	0	0	2.8e-15	1.7e-14
45	0.0008164	0.00010781		95	48	95	2	0	0	2.1e-15	1.4e-14
46	0.001032	0.00021563		97	49	97	2	0	0	3.1e-15	2e-14
47	0.0012477	0.00021563		99	50	99	2	0	0	2.3e-15	1.7e-14
48	0.0016789	0.00043126		101	51	101	2	0	0	4.4e-15	2.7e-14
49	0.0021102	0.00043126		103	52	103	2	0	0	2.1e-15	2.2e-14
50	0.0025414	0.00043126		105	53	105	2	0	0	2.4e-15	2.3e-14
51	0.0029727	0.00043126		107	54	107	2	0	0	7.5e-15	2e-14
52	0.0038352	0.00086251		109	55	109	2	0	0	2.9e-15	2.6e-14
53	0.0046977	0.00086251		111	56	111	2	0	0	4.5e-15	2e-14
-	0.005	-	out								
54	0.0064227	0.001725		113	57	113	2	0	0	4.6e-15	3.3e-14
55	0.0081478	0.001725		115	58	115	2	0	0	3.9e-15	3e-14
-	0.01	-	out								
56	0.011598	0.0034501		117	59	117	2	0	0	8.4e-15	4.6e-14
-	0.015	-	out								
57	0.015048	0.0034501		119	60	119	1	0	0	3.7e-15	5.1e-14
58	0.018498	0.0034501		121	61	121	1	0	0	3.3e-15	4.2e-14
-	0.02	-	out								
-	0.025	-	out								
59	0.025398	0.0069001		123	62	123	1	0	0	3.9e-15	6.7e-14
-	0.03	-	out								
60	0.032298	0.0069001		125	63	125	1	0	0	7.1e-15	6.3e-14
-	0.035	-	out								
-	0.04	-	out								
-	0.045	-	out								
61	0.046098	0.0138		127	64	127	1	0	0	7.2e-15	9.2e-14
-	0.05	-	out								
-	0.055	-	out								
62	0.059899	0.0138		129	65	129	1	0	0	9.5e-15	8.4e-14
-	0.06	-	out								
-	0.065	-	out								
-	0.07	-	out								
-	0.075	-	out								
-	0.08	-	out								
-	0.085	-	out								
63	0.087499	0.0276		131	66	131	1	0	0	5.6e-15	1.1e-13
-	0.09	-	out								
-	0.095	-	out								
-	0.1	-	out								
-	0.105	-	out								
-	0.11	-	out								
-	0.115	-	out								
64	0.1151	0.0276		133	67	133	1	0	0	1.3e-14	8.3e-14
-	0.12	-	out								
-	0.125	-	out								
-	0.13	-	out								
-	0.135	-	out								
-	0.14	-	out								
-	0.145	-	out								
-	0.15	-	out								
-	0.155	-	out								
-	0.16	-	out								
-	0.165	-	out								
-	0.17	-	out								
65	0.1703	0.055201		135	68	135	1	0	0	9.3e-15	9.1e-14

-	0.175	- out								
-	0.18	- out								
-	0.185	- out								
-	0.19	- out								
-	0.195	- out								
-	0.2	- out								
-	0.205	- out								
-	0.21	- out								
-	0.215	- out								
-	0.22	- out								
-	0.225	- out								
66	0.2255	0.055201	137	69	137	1	0	0	9.4e-15	6.6e-14
-	0.23	- out								
-	0.235	- out								
-	0.24	- out								
-	0.245	- out								
-	0.25	- out								
-	0.25	- out								
-	0.27	- out								
-	0.29	- out								
-	0.31	- out								
67	0.3245	0.099	139	70	139	1	0	0	8.4e-15	6.7e-14
-	0.33	- out								
-	0.35	- out								
-	0.37	- out								
-	0.39	- out								
-	0.41	- out								
68	0.4235	0.099	141	71	141	1	0	0	8.1e-15	5.1e-14
-	0.43	- out								
-	0.45	- out								
-	0.47	- out								
-	0.49	- out								
-	0.51	- out								
69	0.5225	0.099	143	72	143	1	0	0	5.9e-15	3.8e-14
-	0.53	- out								
-	0.55	- out								
-	0.57	- out								
-	0.59	- out								
-	0.61	- out								
70	0.6215	0.099	145	73	145	1	0	0	3.8e-15	3.1e-14
-	0.63	- out								
-	0.65	- out								
-	0.67	- out								
-	0.69	- out								
-	0.71	- out								
71	0.7205	0.099	146	74	146	1	0	0	5.5e-15	2.6e-14
-	0.73	- out								
-	0.75	- out								
-	0.77	- out								
-	0.79	- out								
-	0.81	- out								
72	0.8195	0.099	147	75	147	1	0	0	7.4e-15	2.2e-14
-	0.83	- out								
-	0.85	- out								
-	0.87	- out								
-	0.89	- out								

```
  -        0.91           - out
 73      0.9185       0.099       148    76   148      2      0       0  8.3e-15  1.8e-14
  -        0.93           - out
  -        0.95           - out
  -        0.97           - out
  -        0.99           - out
 74      1.0175       0.099       149    77   149      2      0       0  4.4e-15  1.2e-14
Time-stepping completed.
Solution time: 15 s.
Physical memory: 1.25 GB
Virtual memory: 1.23 GB
Ended at May 28, 2023, 4:16:13 PM.
----- Time-Dependent Solver 1 in Oxygen Pulse/Solution 958 (sol958) ----------->
```

k0Sub=1.51E-7, d=6E-6 (su45)

GENERAL

Description	Value
Solution	k0Sub=1.51E-7, d=6E-6

LOG

```
<---- Compile Equations: Stationary in Oxygen Pulse/Solution 958 (sol958) ------
Started at May 28, 2023, 4:16:15 PM.
Parameter k0Sub = 1.51E-7 (m/s).
Parameter d = 6.0E-6 (m).
Geometry shape function: Linear Lagrange
Number of vertex elements: 10
Minimum element quality: 0.432
Time: 0 s.
Physical memory: 1.22 GB
Virtual memory: 1.21 GB
Ended at May 28, 2023, 4:16:16 PM.
----- Compile Equations: Stationary in Oxygen Pulse/Solution 958 (sol958) ----->

<---- Dependent Variables 1 in Oxygen Pulse/Solution 958 (sol958) --------------
Started at May 28, 2023, 4:16:16 PM.
Solution time: 0 s.
Physical memory: 1.22 GB
Virtual memory: 1.21 GB
Ended at May 28, 2023, 4:16:16 PM.
----- Dependent Variables 1 in Oxygen Pulse/Solution 958 (sol958) ------------->

<---- Stationary Solver 1 in Oxygen Pulse/Solution 958 (sol958) ----------------
Started at May 28, 2023, 4:16:16 PM.
Linear solver
Number of degrees of freedom solved for: 18385 (plus 936 internal DOFs).
Symmetric matrices found.
Scales for dependent variables:
Concentration (comp2.Oxi): 0.23
Orthonormal null-space function used.
Iter      SolEst     Damping    Stepsize #Res #Jac #Sol   LinErr   LinRes
   1       7e-05   1.0000000       7e-05    1    1    1  2.7e-13  6.3e-14
Solution time: 1 s.
Physical memory: 1.23 GB
Virtual memory: 1.22 GB
Ended at May 28, 2023, 4:16:16 PM.
----- Stationary Solver 1 in Oxygen Pulse/Solution 958 (sol958) --------------->

<---- Compile Equations: Oxygen Probe Pulse in Oxygen Pulse/Solution 958
      (sol958) ----------------------------------------------------------------
Started at May 28, 2023, 4:16:16 PM.
Geometry shape function: Linear Lagrange
Time: 0 s.
Physical memory: 1.23 GB
Virtual memory: 1.22 GB
Ended at May 28, 2023, 4:16:17 PM.
----- Compile Equations: Oxygen Probe Pulse in Oxygen Pulse/Solution 958
      (sol958) --------------------------------------------------------------->

<---- Dependent Variables 2 in Oxygen Pulse/Solution 958 (sol958) --------------
Started at May 28, 2023, 4:16:17 PM.
Initial values of variables solved for: Solution 958 (sol958), Solution Store 1 (sol
985).
Values of variables not solved for: Solution 958 (sol958), Solution Store 1 (sol985)
.
Solution time: 0 s.
Physical memory: 1.24 GB
```

Virtual memory: 1.22 GB
Ended at May 28, 2023, 4:16:17 PM.
----- Dependent Variables 2 in Oxygen Pulse/Solution 958 (sol958) ------------->

<---- Time-Dependent Solver 1 in Oxygen Pulse/Solution 958 (sol958) ------------
Started at May 28, 2023, 4:16:17 PM.
Time-dependent solver (BDF)
Number of degrees of freedom solved for: 18385 (plus 36840 internal DOFs).
Symmetric matrices found.
Scales for dependent variables:
Concentration (comp2.Oxi): 0.23

Step	Time	Stepsize	Res	Jac	Sol	Order	Tfail	NLfail	LinErr	LinRes
0	0	- out	5	3	5			0	9.2e-16	1.4e-15
1	2.643e-08	2.643e-08	7	4	7	1	0	0	3.6e-16	1e-15
2	5.286e-08	2.643e-08	9	5	9	1	0	0	3.6e-16	1.2e-15
3	1.0572e-07	5.286e-08	11	6	11	2	0	0	3.8e-16	1.3e-15
4	2.1144e-07	1.0572e-07	13	7	13	2	0	0	4.8e-16	1.8e-15
5	4.2288e-07	2.1144e-07	15	8	15	2	0	0	5.8e-16	3.2e-15
6	6.3431e-07	2.1144e-07	17	9	17	2	0	0	6.3e-16	3.6e-15
7	8.4575e-07	2.1144e-07	19	10	19	2	0	0	6.9e-16	3.8e-15
8	1.0572e-06	2.1144e-07	21	11	21	2	0	0	6.1e-16	3.8e-15
9	1.4801e-06	4.2288e-07	23	12	23	2	0	0	6.6e-16	6e-15
10	1.9029e-06	4.2288e-07	25	13	25	2	0	0	7.9e-16	5.9e-15
11	2.3258e-06	4.2288e-07	27	14	27	2	0	0	8.6e-16	6.1e-15
12	2.7487e-06	4.2288e-07	29	15	29	2	0	0	7.8e-16	5.7e-15
13	3.5944e-06	8.4575e-07	31	16	31	2	0	0	1.1e-15	8.6e-15
14	4.4402e-06	8.4575e-07	33	17	33	2	0	0	1.1e-15	7.5e-15
15	5.286e-06	8.4575e-07	35	18	35	2	0	0	9e-16	7.2e-15
16	6.1317e-06	8.4575e-07	37	19	37	2	0	0	9.9e-16	6.4e-15
17	7.8232e-06	1.6915e-06	39	20	39	2	0	0	1.2e-15	9.7e-15
18	9.5147e-06	1.6915e-06	41	21	41	2	0	0	1.2e-15	8.4e-15
19	1.1206e-05	1.6915e-06	43	22	43	2	0	0	1.7e-15	8.5e-15
20	1.2898e-05	1.6915e-06	45	23	45	2	0	0	1.4e-15	7.3e-15
21	1.4589e-05	1.6915e-06	47	24	47	2	0	0	1.3e-15	6.5e-15
22	1.7972e-05	3.383e-06	49	25	49	2	0	0	1.8e-15	1e-14
23	2.1355e-05	3.383e-06	51	26	51	2	0	0	1.9e-15	8.4e-15
24	2.4738e-05	3.383e-06	53	27	53	2	0	0	1.7e-15	8.6e-15
25	2.8121e-05	3.383e-06	55	28	55	2	0	0	1.4e-15	7.7e-15
26	3.4887e-05	6.766e-06	57	29	57	2	0	0	1.8e-15	1.1e-14
27	4.1653e-05	6.766e-06	59	30	59	2	0	0	1.7e-15	9.8e-15
28	4.8419e-05	6.766e-06	61	31	61	2	0	0	1.7e-15	1e-14
29	5.5185e-05	6.766e-06	63	32	63	2	0	0	1.6e-15	9.2e-15
30	6.1951e-05	6.766e-06	65	33	65	2	0	0	1.5e-15	8.1e-15
31	7.5483e-05	1.3532e-05	67	34	67	2	0	0	2.1e-15	1.2e-14
32	8.9015e-05	1.3532e-05	69	35	69	2	0	0	1.3e-15	1e-14
33	0.00010255	1.3532e-05	71	36	71	2	0	0	2.3e-15	1.1e-14
34	0.00011608	1.3532e-05	73	37	73	2	0	0	2.5e-15	1e-14
35	0.00014314	2.7064e-05	75	38	75	2	0	0	2.8e-15	1.5e-14
36	0.00017021	2.7064e-05	77	39	77	2	0	0	1.3e-15	1.3e-14
37	0.00019727	2.7064e-05	79	40	79	2	0	0	1.6e-15	1.2e-14
38	0.00022434	2.7064e-05	81	41	81	2	0	0	3e-15	1.1e-14
39	0.00027846	5.4128e-05	83	42	83	2	0	0	1.7e-15	1.6e-14
40	0.00033259	5.4128e-05	85	43	85	2	0	0	2.1e-15	1.4e-14
41	0.00038672	5.4128e-05	87	44	87	2	0	0	1.9e-15	1.4e-14
42	0.00049498	0.00010826	89	45	89	2	0	0	3.9e-15	2.1e-14
43	0.00060323	0.00010826	91	46	91	2	0	0	2.7e-15	1.6e-14

44	0.00071149	0.00010826	93	47	93	2	0	0	3.7e-15	1.6e-14
45	0.00081975	0.00010826	95	48	95	2	0	0	3.9e-15	1.4e-14
46	0.0010363	0.00021651	97	49	97	2	0	0	1.8e-15	2e-14
47	0.0012528	0.00021651	99	50	99	2	0	0	1.8e-15	1.6e-14
48	0.0016858	0.00043303	101	51	101	2	0	0	5.6e-15	2.5e-14
49	0.0021188	0.00043303	103	52	103	2	0	0	3.2e-15	2.2e-14
50	0.0025518	0.00043303	105	53	105	2	0	0	3.4e-15	2.3e-14
51	0.0029849	0.00043303	107	54	107	2	0	0	5.2e-15	2e-14
52	0.0038509	0.00086605	109	55	109	2	0	0	3.1e-15	2.6e-14
53	0.004717	0.00086605	111	56	111	2	0	0	2.4e-15	2.1e-14
-	0.005	- out								
54	0.0064491	0.0017321	113	57	113	2	0	0	1.7e-15	3.4e-14
55	0.0081812	0.0017321	115	58	115	2	0	0	2e-15	3e-14
-	0.01	- out								
56	0.011645	0.0034642	117	59	117	2	0	0	6.2e-15	4.6e-14
-	0.015	- out								
57	0.01511	0.0034642	119	60	119	1	0	0	4.6e-15	4.7e-14
58	0.018574	0.0034642	121	61	121	1	0	0	3.4e-15	3.7e-14
-	0.02	- out								
-	0.025	- out								
59	0.025502	0.0069284	123	62	123	1	0	0	5.7e-15	6.5e-14
-	0.03	- out								
60	0.032431	0.0069284	125	63	125	1	0	0	2.8e-15	6.1e-14
-	0.035	- out								
-	0.04	- out								
-	0.045	- out								
61	0.046287	0.013857	127	64	127	1	0	0	2.6e-15	8.7e-14
-	0.05	- out								
-	0.055	- out								
-	0.06	- out								
62	0.060144	0.013857	129	65	129	1	0	0	3.7e-15	7.7e-14
-	0.065	- out								
-	0.07	- out								
-	0.075	- out								
-	0.08	- out								
-	0.085	- out								
63	0.087858	0.027714	131	66	131	1	0	0	4.2e-15	9.9e-14
-	0.09	- out								
-	0.095	- out								
-	0.1	- out								
-	0.105	- out								
-	0.11	- out								
-	0.115	- out								
64	0.11557	0.027714	133	67	133	1	0	0	1.3e-14	8.2e-14
-	0.12	- out								
-	0.125	- out								
-	0.13	- out								
-	0.135	- out								
-	0.14	- out								
-	0.145	- out								
-	0.15	- out								
-	0.155	- out								
-	0.16	- out								
-	0.165	- out								
-	0.17	- out								
65	0.171	0.055427	135	68	135	1	0	0	8.3e-15	9.1e-14

-	0.175	- out								
-	0.18	- out								
-	0.185	- out								
-	0.19	- out								
-	0.195	- out								
-	0.2	- out								
-	0.205	- out								
-	0.21	- out								
-	0.215	- out								
-	0.22	- out								
-	0.225	- out								
66	0.22643	0.055427	137	69	137	1	0	0	7.1e-15	7.1e-14
-	0.23	- out								
-	0.235	- out								
-	0.24	- out								
-	0.245	- out								
-	0.25	- out								
-	0.25	- out								
-	0.27	- out								
-	0.29	- out								
-	0.31	- out								
67	0.32543	0.099	139	70	139	1	0	0	1e-14	7.4e-14
-	0.33	- out								
-	0.35	- out								
-	0.37	- out								
-	0.39	- out								
-	0.41	- out								
68	0.42443	0.099	141	71	141	1	0	0	2.7e-15	5.2e-14
-	0.43	- out								
-	0.45	- out								
-	0.47	- out								
-	0.49	- out								
-	0.51	- out								
69	0.52343	0.099	143	72	143	1	0	0	8e-15	3.9e-14
-	0.53	- out								
-	0.55	- out								
-	0.57	- out								
-	0.59	- out								
-	0.61	- out								
70	0.62243	0.099	145	73	145	1	0	0	4e-15	3.2e-14
-	0.63	- out								
-	0.65	- out								
-	0.67	- out								
-	0.69	- out								
-	0.71	- out								
71	0.72143	0.099	146	74	146	1	0	0	1.1e-14	2.6e-14
-	0.73	- out								
-	0.75	- out								
-	0.77	- out								
-	0.79	- out								
-	0.81	- out								
72	0.82043	0.099	147	75	147	1	0	0	1.5e-14	2.3e-14
-	0.83	- out								
-	0.85	- out								
-	0.87	- out								
-	0.89	- out								

```
   -        0.91           - out
  73     0.91943       0.099      148   76  148     2     0      0  6.1e-15  1.8e-14
   -        0.93           - out
   -        0.95           - out
   -        0.97           - out
   -        0.99           - out
  74      1.0184       0.099      149   77  149     2     0      0  7.1e-15  1.2e-14
Time-stepping completed.
Solution time: 13 s.
Physical memory: 1.26 GB
Virtual memory: 1.24 GB
Ended at May 28, 2023, 4:16:30 PM.
----- Time-Dependent Solver 1 in Oxygen Pulse/Solution 958 (sol958) ----------->
```

k0Sub=1.51E-7, d=8E-6 (su46)

GENERAL

Description	Value
Solution	k0Sub=1.51E-7, d=8E-6

LOG

```
<---- Compile Equations: Stationary in Oxygen Pulse/Solution 958 (sol958) ------
Started at May 28, 2023, 4:16:33 PM.
Parameter k0Sub = 1.51E-7 (m/s).
Parameter d = 8.0E-6 (m).
Geometry shape function: Linear Lagrange
Number of vertex elements: 10
Minimum element quality: 0.4468
Time: 0 s.
Physical memory: 1.24 GB
Virtual memory: 1.22 GB
Ended at May 28, 2023, 4:16:33 PM.
----- Compile Equations: Stationary in Oxygen Pulse/Solution 958 (sol958) ----->

<---- Dependent Variables 1 in Oxygen Pulse/Solution 958 (sol958) --------------
Started at May 28, 2023, 4:16:33 PM.
Solution time: 0 s.
Physical memory: 1.24 GB
Virtual memory: 1.22 GB
Ended at May 28, 2023, 4:16:33 PM.
----- Dependent Variables 1 in Oxygen Pulse/Solution 958 (sol958) ------------->

<---- Stationary Solver 1 in Oxygen Pulse/Solution 958 (sol958) ----------------
Started at May 28, 2023, 4:16:33 PM.
Linear solver
Number of degrees of freedom solved for: 23800 (plus 941 internal DOFs).
Symmetric matrices found.
Scales for dependent variables:
Concentration (comp2.Oxi): 0.23
Orthonormal null-space function used.
Iter      SolEst     Damping    Stepsize #Res #Jac #Sol   LinErr   LinRes
   1       5e-05   1.0000000       5e-05    1    1    1  3.4e-13    6e-14
Solution time: 1 s.
Physical memory: 1.25 GB
Virtual memory: 1.23 GB
Ended at May 28, 2023, 4:16:34 PM.
----- Stationary Solver 1 in Oxygen Pulse/Solution 958 (sol958) --------------->

<---- Compile Equations: Oxygen Probe Pulse in Oxygen Pulse/Solution 958
      (sol958) ----------------------------------------------------------------
Started at May 28, 2023, 4:16:34 PM.
Geometry shape function: Linear Lagrange
Time: 1 s.
Physical memory: 1.25 GB
Virtual memory: 1.23 GB
Ended at May 28, 2023, 4:16:34 PM.
----- Compile Equations: Oxygen Probe Pulse in Oxygen Pulse/Solution 958
      (sol958) --------------------------------------------------------------->

<---- Dependent Variables 2 in Oxygen Pulse/Solution 958 (sol958) --------------
Started at May 28, 2023, 4:16:34 PM.
Initial values of variables solved for: Solution 958 (sol958), Solution Store 1 (sol
985).
Values of variables not solved for: Solution 958 (sol958), Solution Store 1 (sol985)
.
Solution time: 0 s.
Physical memory: 1.25 GB
```

Virtual memory: 1.23 GB
Ended at May 28, 2023, 4:16:34 PM.
----- Dependent Variables 2 in Oxygen Pulse/Solution 958 (sol958) ------------->

<---- Time-Dependent Solver 1 in Oxygen Pulse/Solution 958 (sol958) ------------
Started at May 28, 2023, 4:16:34 PM.
Time-dependent solver (BDF)
Number of degrees of freedom solved for: 23800 (plus 47670 internal DOFs).
Symmetric matrices found.
Scales for dependent variables:
Concentration (comp2.Oxi): 0.23

Step	Time	Stepsize	Res	Jac	Sol	Order	Tfail	NLfail	LinErr	LinRes
0	0	- out	5	3	5			0	5.6e-16	8.7e-16
1	3.6252e-08	3.6252e-08	7	4	7	1	0	0	2.9e-16	8.9e-16
2	7.2504e-08	3.6252e-08	9	5	9	1	0	0	3.1e-16	1e-15
3	1.4501e-07	7.2504e-08	11	6	11	2	0	0	3.2e-16	1.1e-15
4	2.9001e-07	1.4501e-07	13	7	13	2	0	0	4.2e-16	1.3e-15
5	5.8003e-07	2.9001e-07	15	8	15	2	0	0	4.6e-16	2.4e-15
6	8.7004e-07	2.9001e-07	17	9	17	2	0	0	5.3e-16	2.9e-15
7	1.1601e-06	2.9001e-07	19	10	19	2	0	0	4.3e-16	3e-15
8	1.4501e-06	2.9001e-07	21	11	21	2	0	0	5.1e-16	3.2e-15
9	2.0301e-06	5.8003e-07	23	12	23	2	0	0	7.9e-16	5.2e-15
10	2.6101e-06	5.8003e-07	25	13	25	2	0	0	7.9e-16	4.6e-15
11	3.1902e-06	5.8003e-07	27	14	27	2	0	0	6.9e-16	4.8e-15
12	3.7702e-06	5.8003e-07	29	15	29	2	0	0	6.3e-16	4.4e-15
13	4.9302e-06	1.1601e-06	31	16	31	2	0	0	7.2e-16	6.9e-15
14	6.0903e-06	1.1601e-06	33	17	33	2	0	0	1.1e-15	6.6e-15
15	7.2504e-06	1.1601e-06	35	18	35	2	0	0	9e-16	6.3e-15
16	8.4104e-06	1.1601e-06	37	19	37	2	0	0	9.7e-16	5.7e-15
17	1.0731e-05	2.3201e-06	39	20	39	2	0	0	1.1e-15	8.5e-15
18	1.3051e-05	2.3201e-06	41	21	41	2	0	0	9.9e-16	7.7e-15
19	1.5371e-05	2.3201e-06	43	22	43	2	0	0	9.9e-16	7.8e-15
20	1.7691e-05	2.3201e-06	45	23	45	2	0	0	1e-15	7.1e-15
21	2.2331e-05	4.6402e-06	47	24	47	2	0	0	1.5e-15	1.1e-14
22	2.6971e-05	4.6402e-06	49	25	49	2	0	0	1e-15	9.4e-15
23	3.1612e-05	4.6402e-06	51	26	51	2	0	0	1.1e-15	9.4e-15
24	3.6252e-05	4.6402e-06	53	27	53	2	0	0	1e-15	8.8e-15
25	4.5532e-05	9.2805e-06	55	28	55	2	0	0	1.5e-15	1.3e-14
26	5.4813e-05	9.2805e-06	57	29	57	2	0	0	2.5e-15	1.2e-14
27	6.4093e-05	9.2805e-06	59	30	59	2	0	0	1.4e-15	1.2e-14
28	7.3374e-05	9.2805e-06	61	31	61	2	0	0	1.2e-15	1e-14
29	9.1935e-05	1.8561e-05	63	32	63	2	0	0	1.7e-15	1.6e-14
30	0.0001105	1.8561e-05	65	33	65	2	0	0	1.1e-15	1.4e-14
31	0.00012906	1.8561e-05	67	34	67	2	0	0	1.2e-15	1.4e-14
32	0.00014762	1.8561e-05	69	35	69	2	0	0	2.5e-15	1.3e-14
33	0.00018474	3.7122e-05	71	36	71	2	0	0	3.5e-15	1.8e-14
34	0.00022186	3.7122e-05	73	37	73	2	0	0	1.4e-15	1.5e-14
35	0.00025898	3.7122e-05	75	38	75	2	0	0	1.7e-15	1.6e-14
36	0.0002961	3.7122e-05	77	39	77	2	0	0	4.5e-15	1.4e-14
37	0.00037035	7.4244e-05	79	40	79	2	0	0	2.2e-15	2e-14
38	0.00044459	7.4244e-05	81	41	81	2	0	0	2.8e-15	1.7e-14
39	0.00059308	0.00014849	83	42	83	2	0	0	1.9e-15	2.6e-14
40	0.00074157	0.00014849	85	43	85	2	0	0	4.1e-15	2.3e-14
41	0.00089005	0.00014849	87	44	87	2	0	0	1.9e-15	2.3e-14
42	0.0010385	0.00014849	89	45	89	2	0	0	2e-15	2e-14
43	0.0013355	0.00029697	91	46	91	2	0	0	3.6e-15	2.6e-14

44	0.0016325	0.00029697		93	47	93	2	0	0	3.7e-15	2e-14
45	0.0022264	0.00059395		95	48	95	2	0	0	2.6e-15	3.2e-14
46	0.0028204	0.00059395		97	49	97	2	0	0	3e-15	2.8e-14
47	0.0034143	0.00059395		99	50	99	2	0	0	2e-15	2.8e-14
48	0.0046022	0.0011879		101	51	101	2	0	0	4.4e-15	3.7e-14
-	0.005	-	out								
49	0.0057901	0.0011879		103	52	103	2	0	0	3.4e-15	2.7e-14
50	0.0081659	0.0023758		105	53	105	2	0	0	6.5e-15	4.2e-14
-	0.01	-	out								
51	0.010542	0.0023758		107	54	107	1	0	0	6.3e-15	5e-14
52	0.012918	0.0023758		109	55	109	1	0	0	4.2e-15	3.3e-14
-	0.015	-	out								
53	0.017669	0.0047516		111	56	111	1	0	0	5.5e-15	5.3e-14
-	0.02	-	out								
-	0.025	-	out								
54	0.027172	0.0095032		113	57	113	1	0	0	4.3e-15	8.4e-14
-	0.03	-	out								
-	0.035	-	out								
55	0.036676	0.0095032		115	58	115	1	0	0	3.7e-15	8.4e-14
-	0.04	-	out								
-	0.045	-	out								
-	0.05	-	out								
-	0.055	-	out								
56	0.055682	0.019006		117	59	117	1	0	0	7.5e-15	1.2e-13
-	0.06	-	out								
-	0.065	-	out								
-	0.07	-	out								
57	0.074688	0.019006		119	60	119	1	0	0	2e-15	1.1e-13
-	0.075	-	out								
-	0.08	-	out								
-	0.085	-	out								
-	0.09	-	out								
-	0.095	-	out								
-	0.1	-	out								
-	0.105	-	out								
-	0.11	-	out								
58	0.1127	0.038013		121	61	121	1	0	0	5e-15	1.4e-13
-	0.115	-	out								
-	0.12	-	out								
-	0.125	-	out								
-	0.13	-	out								
-	0.135	-	out								
-	0.14	-	out								
-	0.145	-	out								
-	0.15	-	out								
59	0.15071	0.038013		123	62	123	1	0	0	5e-15	1.2e-13
-	0.155	-	out								
-	0.16	-	out								
-	0.165	-	out								
-	0.17	-	out								
-	0.175	-	out								
-	0.18	-	out								
-	0.185	-	out								
-	0.19	-	out								
-	0.195	-	out								
-	0.2	-	out								

-	0.205	-	out							
-	0.21	-	out							
-	0.215	-	out							
-	0.22	-	out							
-	0.225	-	out							
60	0.22674	0.076026		125	63	125	1	0	0	9.6e-15	1.2e-13
-	0.23	-	out							
-	0.235	-	out							
-	0.24	-	out							
-	0.245	-	out							
-	0.25	-	out							
-	0.25	-	out							
-	0.27	-	out							
-	0.29	-	out							
61	0.30276	0.076026		127	64	127	1	0	0	9.8e-15	9.6e-14
-	0.31	-	out							
-	0.33	-	out							
-	0.35	-	out							
-	0.37	-	out							
-	0.39	-	out							
62	0.40176	0.099		129	65	129	1	0	0	5.5e-15	8.2e-14
-	0.41	-	out							
-	0.43	-	out							
-	0.45	-	out							
-	0.47	-	out							
-	0.49	-	out							
63	0.50076	0.099		131	66	131	1	0	0	3e-15	6.3e-14
-	0.51	-	out							
-	0.53	-	out							
-	0.55	-	out							
-	0.57	-	out							
-	0.59	-	out							
64	0.59976	0.099		133	67	133	1	0	0	5.1e-15	4.9e-14
-	0.61	-	out							
-	0.63	-	out							
-	0.65	-	out							
-	0.67	-	out							
-	0.69	-	out							
65	0.69876	0.099		134	68	134	1	0	0	6.9e-15	4.1e-14
-	0.71	-	out							
-	0.73	-	out							
-	0.75	-	out							
-	0.77	-	out							
-	0.79	-	out							
66	0.79776	0.099		135	69	135	1	0	0	5.7e-15	3.6e-14
-	0.81	-	out							
-	0.83	-	out							
-	0.85	-	out							
-	0.87	-	out							
-	0.89	-	out							
67	0.89676	0.099		136	70	136	2	0	0	1.1e-14	3e-14
-	0.91	-	out							
-	0.93	-	out							
-	0.95	-	out							
-	0.97	-	out							
-	0.99	-	out							

```
  68     0.99576       0.099      137   71  137     2     0      0  7.3e-15  1.9e-14
Time-stepping completed.
Solution time: 16 s.
Physical memory: 1.28 GB
Virtual memory: 1.27 GB
Ended at May 28, 2023, 4:16:51 PM.
----- Time-Dependent Solver 1 in Oxygen Pulse/Solution 958 (sol958) ----------->
```

k0Sub=1.51E-7, d=1.2E-5 (su47)

GENERAL

Description	Value
Solution	k0Sub=1.51E-7, d=1.2E-5

LOG

```
<---- Compile Equations: Stationary in Oxygen Pulse/Solution 958 (sol958) ------
Started at May 28, 2023, 4:16:54 PM.
Parameter k0Sub = 1.51E-7 (m/s).
Parameter d = 1.2E-5 (m).
Geometry shape function: Linear Lagrange
Number of vertex elements: 10
Minimum element quality: 0.4505
Time: 0 s.
Physical memory: 1.24 GB
Virtual memory: 1.23 GB
Ended at May 28, 2023, 4:16:54 PM.
----- Compile Equations: Stationary in Oxygen Pulse/Solution 958 (sol958) ----->

<---- Dependent Variables 1 in Oxygen Pulse/Solution 958 (sol958) --------------
Started at May 28, 2023, 4:16:54 PM.
Solution time: 0 s.
Physical memory: 1.24 GB
Virtual memory: 1.23 GB
Ended at May 28, 2023, 4:16:54 PM.
----- Dependent Variables 1 in Oxygen Pulse/Solution 958 (sol958) ------------->

<---- Stationary Solver 1 in Oxygen Pulse/Solution 958 (sol958) ----------------
Started at May 28, 2023, 4:16:54 PM.
Linear solver
Number of degrees of freedom solved for: 18769 (plus 948 internal DOFs).
Symmetric matrices found.
Scales for dependent variables:
Concentration (comp2.Oxi): 0.23
Orthonormal null-space function used.
Iter      SolEst     Damping    Stepsize #Res #Jac #Sol   LinErr   LinRes
   1     4.3e-05   1.0000000     4.3e-05    1    1    1  2.5e-13    5e-14
Solution time: 1 s.
Physical memory: 1.24 GB
Virtual memory: 1.23 GB
Ended at May 28, 2023, 4:16:55 PM.
----- Stationary Solver 1 in Oxygen Pulse/Solution 958 (sol958) --------------->

<---- Compile Equations: Oxygen Probe Pulse in Oxygen Pulse/Solution 958
      (sol958) -----------------------------------------------------------------
Started at May 28, 2023, 4:16:55 PM.
Geometry shape function: Linear Lagrange
Time: 0 s.
Physical memory: 1.22 GB
Virtual memory: 1.2 GB
Ended at May 28, 2023, 4:16:55 PM.
----- Compile Equations: Oxygen Probe Pulse in Oxygen Pulse/Solution 958
      (sol958) ---------------------------------------------------------------->

<---- Dependent Variables 2 in Oxygen Pulse/Solution 958 (sol958) --------------
Started at May 28, 2023, 4:16:55 PM.
Initial values of variables solved for: Solution 958 (sol958), Solution Store 1 (sol
985).
Values of variables not solved for: Solution 958 (sol958), Solution Store 1 (sol985)
.
Solution time: 0 s.
Physical memory: 1.23 GB
```

Virtual memory: 1.2 GB
Ended at May 28, 2023, 4:16:55 PM.
----- Dependent Variables 2 in Oxygen Pulse/Solution 958 (sol958) ------------->

<---- Time-Dependent Solver 1 in Oxygen Pulse/Solution 958 (sol958) ------------
Started at May 28, 2023, 4:16:55 PM.
Time-dependent solver (BDF)
Number of degrees of freedom solved for: 18769 (plus 37608 internal DOFs).
Symmetric matrices found.
Scales for dependent variables:
Concentration (comp2.Oxi): 0.23

Step	Time	Stepsize	Res	Jac	Sol	Order	Tfail	NLfail	LinErr	LinRes
0	0	- out	5	3	5			0	1.1e-15	1.4e-15
1	2.659e-08	2.659e-08	7	4	7	1	0	0	3.5e-16	1.1e-15
2	5.318e-08	2.659e-08	9	5	9	1	0	0	3.3e-16	1.2e-15
3	1.0636e-07	5.318e-08	11	6	11	2	0	0	3.5e-16	1.2e-15
4	2.1272e-07	1.0636e-07	13	7	13	2	0	0	5.2e-16	1.8e-15
5	4.2544e-07	2.1272e-07	15	8	15	2	0	0	5.7e-16	3.5e-15
6	6.3816e-07	2.1272e-07	17	9	17	2	0	0	6.9e-16	3.8e-15
7	8.5089e-07	2.1272e-07	19	10	19	2	0	0	6.6e-16	3.9e-15
8	1.0636e-06	2.1272e-07	21	11	21	2	0	0	5.9e-16	4e-15
9	1.4891e-06	4.2544e-07	23	12	23	2	0	0	9.3e-16	6.3e-15
10	1.9145e-06	4.2544e-07	25	13	25	2	0	0	9.1e-16	6.1e-15
11	2.3399e-06	4.2544e-07	27	14	27	2	0	0	7.9e-16	6.2e-15
12	2.7654e-06	4.2544e-07	29	15	29	2	0	0	1.1e-15	5.5e-15
13	3.6163e-06	8.5089e-07	31	16	31	2	0	0	1e-15	8.6e-15
14	4.4672e-06	8.5089e-07	33	17	33	2	0	0	8.5e-16	7.2e-15
15	5.318e-06	8.5089e-07	35	18	35	2	0	0	8.3e-16	7e-15
16	6.1689e-06	8.5089e-07	37	19	37	2	0	0	1e-15	6.4e-15
17	7.8707e-06	1.7018e-06	39	20	39	2	0	0	8.9e-16	9.3e-15
18	9.5725e-06	1.7018e-06	41	21	41	2	0	0	1.1e-15	7.9e-15
19	1.1274e-05	1.7018e-06	43	22	43	2	0	0	1.3e-15	8.1e-15
20	1.2976e-05	1.7018e-06	45	23	45	2	0	0	2e-15	7.2e-15
21	1.4678e-05	1.7018e-06	47	24	47	2	0	0	1.3e-15	6.6e-15
22	1.8081e-05	3.4035e-06	49	25	49	2	0	0	1.5e-15	9.6e-15
23	2.1485e-05	3.4035e-06	51	26	51	2	0	0	2.2e-15	8.4e-15
24	2.4888e-05	3.4035e-06	53	27	53	2	0	0	1.9e-15	8.3e-15
25	2.8292e-05	3.4035e-06	55	28	55	2	0	0	1.6e-15	7.4e-15
26	3.5099e-05	6.8071e-06	57	29	57	2	0	0	1.3e-15	1.1e-14
27	4.1906e-05	6.8071e-06	59	30	59	2	0	0	1.3e-15	9.9e-15
28	4.8713e-05	6.8071e-06	61	31	61	2	0	0	1.9e-15	9.6e-15
29	5.552e-05	6.8071e-06	63	32	63	2	0	0	1.6e-15	8.8e-15
30	6.2327e-05	6.8071e-06	65	33	65	2	0	0	1.8e-15	7.9e-15
31	7.5942e-05	1.3614e-05	67	34	67	2	0	0	2.1e-15	1.2e-14
32	8.9556e-05	1.3614e-05	69	35	69	2	0	0	1.8e-15	1e-14
33	0.00010317	1.3614e-05	71	36	71	2	0	0	1.2e-15	1e-14
34	0.00011678	1.3614e-05	73	37	73	2	0	0	2.1e-15	9.2e-15
35	0.00014401	2.7228e-05	75	38	75	2	0	0	2.2e-15	1.4e-14
36	0.00017124	2.7228e-05	77	39	77	2	0	0	2.3e-15	1.2e-14
37	0.00019847	2.7228e-05	79	40	79	2	0	0	1.8e-15	1.2e-14
38	0.0002257	2.7228e-05	81	41	81	2	0	0	2.2e-15	1.1e-14
39	0.00028015	5.4457e-05	83	42	83	2	0	0	3.1e-15	1.6e-14
40	0.00033461	5.4457e-05	85	43	85	2	0	0	1.5e-15	1.4e-14
41	0.00038907	5.4457e-05	87	44	87	2	0	0	1.3e-15	1.4e-14
42	0.00049798	0.00010891	89	45	89	2	0	0	1.2e-15	2e-14
43	0.00060689	0.00010891	91	46	91	2	0	0	2.8e-15	1.7e-14

44	0.00071581	0.00010891		93	47	93	2	0	0	5.7e-15	1.7e-14
45	0.00082472	0.00010891		95	48	95	2	0	0	2.3e-15	1.4e-14
46	0.0010425	0.00021783		97	49	97	2	0	0	2.8e-15	2e-14
47	0.0012604	0.00021783		99	50	99	2	0	0	3.5e-15	1.7e-14
48	0.001696	0.00043565		101	51	101	2	0	0	5.2e-15	2.7e-14
49	0.0021317	0.00043565		103	52	103	2	0	0	2.8e-15	2.3e-14
50	0.0025673	0.00043565		105	53	105	2	0	0	2.5e-15	2.2e-14
51	0.003003	0.00043565		107	54	107	2	0	0	2e-15	1.9e-14
52	0.0038743	0.00087131		109	55	109	2	0	0	3.7e-15	2.4e-14
53	0.0047456	0.00087131		111	56	111	2	0	0	1.6e-15	1.8e-14
-	0.005	-	out								
54	0.0064882	0.0017426		113	57	113	2	0	0	4.9e-15	2.9e-14
55	0.0082308	0.0017426		115	58	115	2	0	0	1.8e-15	2.7e-14
-	0.01	-	out								
56	0.011716	0.0034852		117	59	117	2	0	0	7.3e-15	4e-14
-	0.015	-	out								
57	0.015201	0.0034852		119	60	119	1	0	0	7.7e-15	4.2e-14
58	0.018687	0.0034852		121	61	121	1	0	0	3.2e-15	2.6e-14
-	0.02	-	out								
-	0.025	-	out								
59	0.025657	0.0069705		123	62	123	1	0	0	2.6e-15	4.1e-14
-	0.03	-	out								
-	0.035	-	out								
60	0.039598	0.013941		125	63	125	1	0	0	3.1e-15	6.2e-14
-	0.04	-	out								
-	0.045	-	out								
-	0.05	-	out								
61	0.053539	0.013941		127	64	127	1	0	0	5.5e-15	6.1e-14
-	0.055	-	out								
-	0.06	-	out								
-	0.065	-	out								
-	0.07	-	out								
-	0.075	-	out								
-	0.08	-	out								
62	0.081421	0.027882		129	65	129	1	0	0	8.3e-15	8.7e-14
-	0.085	-	out								
-	0.09	-	out								
-	0.095	-	out								
-	0.1	-	out								
-	0.105	-	out								
63	0.1093	0.027882		131	66	131	1	0	0	2.9e-15	7.8e-14
-	0.11	-	out								
-	0.115	-	out								
-	0.12	-	out								
-	0.125	-	out								
-	0.13	-	out								
-	0.135	-	out								
-	0.14	-	out								
-	0.145	-	out								
-	0.15	-	out								
-	0.155	-	out								
-	0.16	-	out								
-	0.165	-	out								
64	0.16507	0.055764		133	67	133	1	0	0	6.5e-15	9.5e-14
-	0.17	-	out								
-	0.175	-	out								

-	0.18	- out								
-	0.185	- out								
-	0.19	- out								
-	0.195	- out								
-	0.2	- out								
-	0.205	- out								
-	0.21	- out								
-	0.215	- out								
-	0.22	- out								
65	0.22083	0.055764	135	68	135	1	0	0	1.8e-14	7.7e-14
-	0.225	- out								
-	0.23	- out								
-	0.235	- out								
-	0.24	- out								
-	0.245	- out								
-	0.25	- out								
-	0.25	- out								
-	0.27	- out								
-	0.29	- out								
-	0.31	- out								
66	0.31983	0.099	137	69	137	1	0	0	7.3e-15	7.9e-14
-	0.33	- out								
-	0.35	- out								
-	0.37	- out								
-	0.39	- out								
-	0.41	- out								
67	0.41883	0.099	139	70	139	1	0	0	1.3e-14	6.1e-14
-	0.43	- out								
-	0.45	- out								
-	0.47	- out								
-	0.49	- out								
-	0.51	- out								
68	0.51783	0.099	141	71	141	1	0	0	9.6e-15	4.7e-14
-	0.53	- out								
-	0.55	- out								
-	0.57	- out								
-	0.59	- out								
-	0.61	- out								
69	0.61683	0.099	143	72	143	1	0	0	7.3e-15	3.8e-14
-	0.63	- out								
-	0.65	- out								
-	0.67	- out								
-	0.69	- out								
-	0.71	- out								
70	0.71583	0.099	144	73	144	1	0	0	1.1e-14	3.1e-14
-	0.73	- out								
-	0.75	- out								
-	0.77	- out								
-	0.79	- out								
-	0.81	- out								
71	0.81483	0.099	145	74	145	1	0	0	1.4e-14	2.7e-14
-	0.83	- out								
-	0.85	- out								
-	0.87	- out								
-	0.89	- out								
-	0.91	- out								

```
  72      0.91383       0.099      146   75  146     2     0      0  6.7e-15  2.2e-14
   -         0.93           - out
   -         0.95           - out
   -         0.97           - out
   -         0.99           - out
  73       1.0128       0.099      147   76  147     2     0      0  1.2e-14  1.4e-14
Time-stepping completed.
Solution time: 14 s.
Physical memory: 1.26 GB
Virtual memory: 1.25 GB
Ended at May 28, 2023, 4:17:09 PM.
----- Time-Dependent Solver 1 in Oxygen Pulse/Solution 958 (sol958) ----------->
```

k0Sub=1.51E-7, d=1.5E-5 (su48)

GENERAL

Description	Value
Solution	k0Sub=1.51E-7, d=1.5E-5

LOG

```
<---- Compile Equations: Stationary in Oxygen Pulse/Solution 958 (sol958) ------
Started at May 28, 2023, 4:17:12 PM.
Parameter k0Sub = 1.51E-7 (m/s).
Parameter d = 1.4999999999999999E-5 (m).
Geometry shape function: Linear Lagrange
Number of vertex elements: 10
Minimum element quality: 0.4444
Time: 0 s.
Physical memory: 1.24 GB
Virtual memory: 1.23 GB
Ended at May 28, 2023, 4:17:12 PM.
----- Compile Equations: Stationary in Oxygen Pulse/Solution 958 (sol958) ----->

<---- Dependent Variables 1 in Oxygen Pulse/Solution 958 (sol958) --------------
Started at May 28, 2023, 4:17:12 PM.
Solution time: 0 s.
Physical memory: 1.24 GB
Virtual memory: 1.23 GB
Ended at May 28, 2023, 4:17:12 PM.
----- Dependent Variables 1 in Oxygen Pulse/Solution 958 (sol958) ------------->

<---- Stationary Solver 1 in Oxygen Pulse/Solution 958 (sol958) ----------------
Started at May 28, 2023, 4:17:12 PM.
Linear solver
Number of degrees of freedom solved for: 18918 (plus 952 internal DOFs).
Symmetric matrices found.
Scales for dependent variables:
Concentration (comp2.Oxi): 0.23
Orthonormal null-space function used.
Iter      SolEst     Damping    Stepsize #Res #Jac #Sol   LinErr   LinRes
   1     3.8e-05   1.0000000     3.8e-05    1    1    1  2.2e-13  4.7e-14
Solution time: 1 s.
Physical memory: 1.25 GB
Virtual memory: 1.24 GB
Ended at May 28, 2023, 4:17:13 PM.
----- Stationary Solver 1 in Oxygen Pulse/Solution 958 (sol958) --------------->

<---- Compile Equations: Oxygen Probe Pulse in Oxygen Pulse/Solution 958
      (sol958) -----------------------------------------------------------------
Started at May 28, 2023, 4:17:13 PM.
Geometry shape function: Linear Lagrange
Time: 1 s.
Physical memory: 1.27 GB
Virtual memory: 1.25 GB
Ended at May 28, 2023, 4:17:13 PM.
----- Compile Equations: Oxygen Probe Pulse in Oxygen Pulse/Solution 958
      (sol958) ---------------------------------------------------------------->

<---- Dependent Variables 2 in Oxygen Pulse/Solution 958 (sol958) --------------
Started at May 28, 2023, 4:17:13 PM.
Initial values of variables solved for: Solution 958 (sol958), Solution Store 1 (sol
985).
Values of variables not solved for: Solution 958 (sol958), Solution Store 1 (sol985)
.
Solution time: 0 s.
Physical memory: 1.27 GB
```

Virtual memory: 1.25 GB
Ended at May 28, 2023, 4:17:13 PM.
----- Dependent Variables 2 in Oxygen Pulse/Solution 958 (sol958) ------------->

<---- Time-Dependent Solver 1 in Oxygen Pulse/Solution 958 (sol958) ------------
Started at May 28, 2023, 4:17:13 PM.
Time-dependent solver (BDF)
Number of degrees of freedom solved for: 18918 (plus 37906 internal DOFs).
Symmetric matrices found.
Scales for dependent variables:
Concentration (comp2.Oxi): 0.23

Step	Time	Stepsize		Res	Jac	Sol	Order	Tfail	NLfail	LinErr	LinRes
0	0	-	out	5	3	5			0	7.7e-16	1.5e-15
1	2.678e-08	2.678e-08		7	4	7	1	0	0	3.7e-16	1.1e-15
2	5.3559e-08	2.678e-08		9	5	9	1	0	0	3.9e-16	1.2e-15
3	1.0712e-07	5.3559e-08		11	6	11	2	0	0	4e-16	1.3e-15
4	2.1424e-07	1.0712e-07		13	7	13	2	0	0	4.6e-16	1.8e-15
5	4.2847e-07	2.1424e-07		15	8	15	2	0	0	5.6e-16	3.6e-15
6	6.4271e-07	2.1424e-07		17	9	17	2	0	0	6.6e-16	3.8e-15
7	8.5695e-07	2.1424e-07		19	10	19	2	0	0	5.5e-16	3.8e-15
8	1.0712e-06	2.1424e-07		21	11	21	2	0	0	5.6e-16	3.9e-15
9	1.4997e-06	4.2847e-07		23	12	23	2	0	0	9.5e-16	6.2e-15
10	1.9281e-06	4.2847e-07		25	13	25	2	0	0	8.7e-16	6.1e-15
11	2.3566e-06	4.2847e-07		27	14	27	2	0	0	7.7e-16	5.8e-15
12	2.7851e-06	4.2847e-07		29	15	29	2	0	0	1e-15	5.7e-15
13	3.642e-06	8.5695e-07		31	16	31	2	0	0	9.4e-16	8.7e-15
14	4.499e-06	8.5695e-07		33	17	33	2	0	0	8.4e-16	7.6e-15
15	5.3559e-06	8.5695e-07		35	18	35	2	0	0	1e-15	7.3e-15
16	6.2129e-06	8.5695e-07		37	19	37	2	0	0	1.1e-15	6.4e-15
17	7.9268e-06	1.7139e-06		39	20	39	2	0	0	1.5e-15	9.8e-15
18	9.6407e-06	1.7139e-06		41	21	41	2	0	0	1.9e-15	8.2e-15
19	1.1355e-05	1.7139e-06		43	22	43	2	0	0	1.4e-15	8.1e-15
20	1.3068e-05	1.7139e-06		45	23	45	2	0	0	1.6e-15	7.5e-15
21	1.4782e-05	1.7139e-06		47	24	47	2	0	0	1.1e-15	6.3e-15
22	1.821e-05	3.4278e-06		49	25	49	2	0	0	1.3e-15	9.5e-15
23	2.1638e-05	3.4278e-06		51	26	51	2	0	0	1.1e-15	8.4e-15
24	2.5066e-05	3.4278e-06		53	27	53	2	0	0	1.3e-15	8.6e-15
25	2.8494e-05	3.4278e-06		55	28	55	2	0	0	1.7e-15	7.7e-15
26	3.5349e-05	6.8556e-06		57	29	57	2	0	0	2.6e-15	1.2e-14
27	4.2205e-05	6.8556e-06		59	30	59	2	0	0	1.9e-15	1e-14
28	4.906e-05	6.8556e-06		61	31	61	2	0	0	1.8e-15	9.8e-15
29	5.5916e-05	6.8556e-06		63	32	63	2	0	0	1.7e-15	9e-15
30	6.2771e-05	6.8556e-06		65	33	65	2	0	0	1.8e-15	8.2e-15
31	7.6483e-05	1.3711e-05		67	34	67	2	0	0	1.7e-15	1.2e-14
32	9.0194e-05	1.3711e-05		69	35	69	2	0	0	2e-15	1e-14
33	0.0001039	1.3711e-05		71	36	71	2	0	0	2.8e-15	1e-14
34	0.00011762	1.3711e-05		73	37	73	2	0	0	2.2e-15	9.6e-15
35	0.00014504	2.7422e-05		75	38	75	2	0	0	2e-15	1.4e-14
36	0.00017246	2.7422e-05		77	39	77	2	0	0	1.9e-15	1.2e-14
37	0.00019988	2.7422e-05		79	40	79	2	0	0	5.2e-15	1.2e-14
38	0.00022731	2.7422e-05		81	41	81	2	0	0	1.9e-15	1.1e-14
39	0.00028215	5.4845e-05		83	42	83	2	0	0	2.2e-15	1.6e-14
40	0.00033699	5.4845e-05		85	43	85	2	0	0	2.2e-15	1.4e-14
41	0.00039184	5.4845e-05		87	44	87	2	0	0	1.5e-15	1.4e-14
42	0.00050153	0.00010969		89	45	89	2	0	0	3.8e-15	2e-14
43	0.00061122	0.00010969		91	46	91	2	0	0	1.5e-15	1.7e-14

44	0.00072091	0.00010969		93	47	93	2	0	0	4.2e-15	1.6e-14
45	0.0008306	0.00010969		95	48	95	2	0	0	2e-15	1.4e-14
46	0.00105	0.00021938		97	49	97	2	0	0	3.1e-15	2.1e-14
47	0.0012694	0.00021938		99	50	99	2	0	0	5.4e-15	1.6e-14
48	0.0017081	0.00043876		101	51	101	2	0	0	1.7e-15	2.6e-14
49	0.0021469	0.00043876		103	52	103	2	0	0	2.1e-15	2.4e-14
50	0.0025856	0.00043876		105	53	105	2	0	0	1.9e-15	2.2e-14
51	0.0030244	0.00043876		107	54	107	2	0	0	2.5e-15	1.9e-14
52	0.0039019	0.00087751		109	55	109	2	0	0	2.6e-15	2.5e-14
53	0.0047794	0.00087751		111	56	111	2	0	0	5.7e-15	1.9e-14
-	0.005	-	out								
54	0.0065344	0.001755		113	57	113	2	0	0	2.5e-15	2.9e-14
55	0.0082895	0.001755		115	58	115	2	0	0	2.5e-15	2.4e-14
-	0.01	-	out								
56	0.0118	0.0035101		117	59	117	2	0	0	6.1e-15	3.8e-14
-	0.015	-	out								
57	0.01531	0.0035101		119	60	119	1	0	0	2.6e-15	4e-14
58	0.01882	0.0035101		121	61	121	1	0	0	2.9e-15	2.4e-14
-	0.02	-	out								
-	0.025	-	out								
59	0.02584	0.0070201		123	62	123	1	0	0	5.2e-15	3.8e-14
-	0.03	-	out								
-	0.035	-	out								
60	0.03988	0.01404		125	63	125	1	0	0	1e-14	5.4e-14
-	0.04	-	out								
-	0.045	-	out								
-	0.05	-	out								
61	0.05392	0.01404		127	64	127	1	0	0	4.3e-15	5e-14
-	0.055	-	out								
-	0.06	-	out								
-	0.065	-	out								
-	0.07	-	out								
-	0.075	-	out								
-	0.08	-	out								
62	0.082001	0.02808		129	65	129	1	0	0	2e-15	7.1e-14
-	0.085	-	out								
-	0.09	-	out								
-	0.095	-	out								
-	0.1	-	out								
-	0.105	-	out								
-	0.11	-	out								
63	0.11008	0.02808		131	66	131	1	0	0	5.1e-15	6.9e-14
-	0.115	-	out								
-	0.12	-	out								
-	0.125	-	out								
-	0.13	-	out								
-	0.135	-	out								
-	0.14	-	out								
-	0.145	-	out								
-	0.15	-	out								
-	0.155	-	out								
-	0.16	-	out								
-	0.165	-	out								
64	0.16624	0.056161		133	67	133	1	0	0	7.1e-15	8.4e-14
-	0.17	-	out								
-	0.175	-	out								

-	0.18	- out								
-	0.185	- out								
-	0.19	- out								
-	0.195	- out								
-	0.2	- out								
-	0.205	- out								
-	0.21	- out								
-	0.215	- out								
-	0.22	- out								
65	0.2224	0.056161	135	68	135	1	0	0	5.2e-15	7.2e-14
-	0.225	- out								
-	0.23	- out								
-	0.235	- out								
-	0.24	- out								
-	0.245	- out								
-	0.25	- out								
-	0.25	- out								
-	0.27	- out								
-	0.29	- out								
-	0.31	- out								
66	0.3214	0.099	137	69	137	1	0	0	5.2e-15	7.7e-14
-	0.33	- out								
-	0.35	- out								
-	0.37	- out								
-	0.39	- out								
-	0.41	- out								
67	0.4204	0.099	139	70	139	1	0	0	7e-15	6.1e-14
-	0.43	- out								
-	0.45	- out								
-	0.47	- out								
-	0.49	- out								
-	0.51	- out								
68	0.5194	0.099	141	71	141	1	0	0	2.6e-14	4.7e-14
-	0.53	- out								
-	0.55	- out								
-	0.57	- out								
-	0.59	- out								
-	0.61	- out								
69	0.6184	0.099	143	72	143	1	0	0	6.7e-15	4e-14
-	0.63	- out								
-	0.65	- out								
-	0.67	- out								
-	0.69	- out								
-	0.71	- out								
70	0.7174	0.099	144	73	144	1	0	0	2.8e-15	3.2e-14
-	0.73	- out								
-	0.75	- out								
-	0.77	- out								
-	0.79	- out								
-	0.81	- out								
71	0.8164	0.099	145	74	145	1	0	0	4.9e-15	2.7e-14
-	0.83	- out								
-	0.85	- out								
-	0.87	- out								
-	0.89	- out								
-	0.91	- out								

```
  72      0.9154       0.099      146   75  146     2     0      0  9.1e-15  2.3e-14
   -        0.93           - out
   -        0.95           - out
   -        0.97           - out
   -        0.99           - out
  73      1.0144       0.099      147   76  147     2     0      0  1.1e-14  1.4e-14
Time-stepping completed.
Solution time: 14 s.
Physical memory: 1.3 GB
Virtual memory: 1.29 GB
Ended at May 28, 2023, 4:17:28 PM.
----- Time-Dependent Solver 1 in Oxygen Pulse/Solution 958 (sol958) ----------->
```

k0Sub=1.51E-6, d=5E-7 (su49)

GENERAL

Description	Value
Solution	k0Sub=1.51E-6, d=5E-7

LOG

```
<---- Compile Equations: Stationary in Oxygen Pulse/Solution 958 (sol958) ------
Started at May 28, 2023, 4:17:30 PM.
Parameter k0Sub = 1.51E-6 (m/s).
Parameter d = 5.0E-7 (m).
Geometry shape function: Linear Lagrange
Number of vertex elements: 10
Minimum element quality: 0.3539
Time: 1 s.
Physical memory: 1.23 GB
Virtual memory: 1.22 GB
Ended at May 28, 2023, 4:17:31 PM.
----- Compile Equations: Stationary in Oxygen Pulse/Solution 958 (sol958) ----->

<---- Dependent Variables 1 in Oxygen Pulse/Solution 958 (sol958) --------------
Started at May 28, 2023, 4:17:31 PM.
Solution time: 0 s.
Physical memory: 1.24 GB
Virtual memory: 1.22 GB
Ended at May 28, 2023, 4:17:31 PM.
----- Dependent Variables 1 in Oxygen Pulse/Solution 958 (sol958) ------------->

<---- Stationary Solver 1 in Oxygen Pulse/Solution 958 (sol958) ----------------
Started at May 28, 2023, 4:17:31 PM.
Linear solver
Number of degrees of freedom solved for: 17710 (plus 1007 internal DOFs).
Symmetric matrices found.
Scales for dependent variables:
Concentration (comp2.Oxi): 0.23
Orthonormal null-space function used.
Iter      SolEst     Damping    Stepsize #Res #Jac #Sol   LinErr   LinRes
   1      0.0021   1.0000000      0.0021    1    1    1  4.4e-13  1.5e-13
Solution time: 1 s.
Physical memory: 1.25 GB
Virtual memory: 1.23 GB
Ended at May 28, 2023, 4:17:32 PM.
----- Stationary Solver 1 in Oxygen Pulse/Solution 958 (sol958) --------------->

<---- Compile Equations: Oxygen Probe Pulse in Oxygen Pulse/Solution 958
      (sol958) ----------------------------------------------------------------
Started at May 28, 2023, 4:17:32 PM.
Geometry shape function: Linear Lagrange
Time: 0 s.
Physical memory: 1.26 GB
Virtual memory: 1.24 GB
Ended at May 28, 2023, 4:17:32 PM.
----- Compile Equations: Oxygen Probe Pulse in Oxygen Pulse/Solution 958
      (sol958) --------------------------------------------------------------->

<---- Dependent Variables 2 in Oxygen Pulse/Solution 958 (sol958) --------------
Started at May 28, 2023, 4:17:32 PM.
Initial values of variables solved for: Solution 958 (sol958), Solution Store 1 (sol
985).
Values of variables not solved for: Solution 958 (sol958), Solution Store 1 (sol985)
.
Solution time: 0 s.
Physical memory: 1.26 GB
```

```
Virtual memory: 1.24 GB
Ended at May 28, 2023, 4:17:32 PM.
----- Dependent Variables 2 in Oxygen Pulse/Solution 958 (sol958) ------------->

<---- Time-Dependent Solver 1 in Oxygen Pulse/Solution 958 (sol958) ------------
Started at May 28, 2023, 4:17:32 PM.
Time-dependent solver (BDF)
Number of degrees of freedom solved for: 17710 (plus 35580 internal DOFs).
Symmetric matrices found.
Scales for dependent variables:
Concentration (comp2.Oxi): 0.23
Step        Time    Stepsize      Res  Jac   Sol Order Tfail NLfail   LinErr   LinRes
   0           0           - out    5    3     5                  0  5.9e-16  1.6e-15
   1  2.5639e-08  2.5639e-08        7    4     7     1     0      0  3.5e-16  1.1e-15
   2  5.1278e-08  2.5639e-08        9    5     9     1     0      0  3.5e-16  1.2e-15
   3  1.0256e-07  5.1278e-08       11    6    11     2     0      0  3.5e-16  1.2e-15
   4  2.0511e-07  1.0256e-07       13    7    13     2     0      0  4.7e-16  1.7e-15
   5  4.1022e-07  2.0511e-07       15    8    15     2     0      0  5.9e-16  3.2e-15
   6  6.1534e-07  2.0511e-07       17    9    17     2     0      0  5.7e-16  3.7e-15
   7  8.2045e-07  2.0511e-07       19   10    19     2     0      0  6.7e-16    4e-15
   8  1.0256e-06  2.0511e-07       21   11    21     2     0      0  7.9e-16  3.9e-15
   9  1.4358e-06  4.1022e-07       23   12    23     2     0      0  7.5e-16  5.9e-15
  10   1.846e-06  4.1022e-07       25   13    25     2     0      0  8.2e-16  5.5e-15
  11  2.2562e-06  4.1022e-07       27   14    27     2     0      0  7.9e-16  5.6e-15
  12  2.6665e-06  4.1022e-07       29   15    29     2     0      0  7.3e-16  5.2e-15
  13  3.4869e-06  8.2045e-07       31   16    31     2     0      0  1.1e-15  7.6e-15
  14  4.3074e-06  8.2045e-07       33   17    33     2     0      0  1.1e-15  6.4e-15
  15  5.1278e-06  8.2045e-07       35   18    35     2     0      0  1.1e-15  6.6e-15
  16  5.9483e-06  8.2045e-07       37   19    37     2     0      0  1.2e-15  5.9e-15
  17  7.5892e-06  1.6409e-06       39   20    39     2     0      0  1.3e-15  8.8e-15
  18  9.2301e-06  1.6409e-06       41   21    41     2     0      0  1.2e-15  7.6e-15
  19  1.0871e-05  1.6409e-06       43   22    43     2     0      0  1.3e-15  7.9e-15
  20  1.2512e-05  1.6409e-06       45   23    45     2     0      0  1.1e-15  7.1e-15
  21  1.4153e-05  1.6409e-06       47   24    47     2     0      0  1.3e-15  6.6e-15
  22  1.7435e-05  3.2818e-06       49   25    49     2     0      0  2.2e-15  9.4e-15
  23  2.0716e-05  3.2818e-06       51   26    51     2     0      0  2.1e-15  8.5e-15
  24  2.3998e-05  3.2818e-06       53   27    53     2     0      0  2.4e-15  8.5e-15
  25   2.728e-05  3.2818e-06       55   28    55     2     0      0  1.4e-15  7.9e-15
  26  3.3844e-05  6.5636e-06       57   29    57     2     0      0  1.4e-15  1.2e-14
  27  4.0407e-05  6.5636e-06       59   30    59     2     0      0  1.5e-15    1e-14
  28  4.6971e-05  6.5636e-06       61   31    61     2     0      0  1.5e-15  9.7e-15
  29  5.3534e-05  6.5636e-06       63   32    63     2     0      0  1.6e-15  9.3e-15
  30  6.0098e-05  6.5636e-06       65   33    65     2     0      0  1.5e-15    8e-15
  31  7.3225e-05  1.3127e-05       67   34    67     2     0      0  1.9e-15  1.2e-14
  32  8.6352e-05  1.3127e-05       69   35    69     2     0      0  1.8e-15  1.1e-14
  33  9.9479e-05  1.3127e-05       71   36    71     2     0      0  1.5e-15  1.1e-14
  34  0.00011261  1.3127e-05       73   37    73     2     0      0  2.1e-15    1e-14
  35  0.00013886  2.6254e-05       75   38    75     2     0      0  2.3e-15  1.6e-14
  36  0.00016512  2.6254e-05       77   39    77     2     0      0  1.4e-15  1.3e-14
  37  0.00019137  2.6254e-05       79   40    79     2     0      0  2.8e-15  1.3e-14
  38  0.00021762  2.6254e-05       81   41    81     2     0      0  2.8e-15  1.1e-14
  39  0.00027013  5.2509e-05       83   42    83     2     0      0  2.5e-15  1.7e-14
  40  0.00032264  5.2509e-05       85   43    85     2     0      0  1.7e-15  1.4e-14
  41  0.00037515  5.2509e-05       87   44    87     2     0      0  2.2e-15  1.4e-14
  42  0.00048017  0.00010502       89   45    89     2     0      0  2.8e-15  2.1e-14
  43  0.00058519  0.00010502       91   46    91     2     0      0    2e-15  1.6e-14
```

44	0.0006902	0.00010502		93	47	93	2	0	0	2.3e-15	1.7e-14
45	0.00090024	0.00021004		95	48	95	2	0	0	2.1e-15	2.3e-14
46	0.0011103	0.00021004		97	49	97	2	0	0	2.6e-15	2e-14
47	0.0013203	0.00021004		99	50	99	2	0	0	2.1e-15	2.1e-14
48	0.0015303	0.00021004		101	51	101	2	0	0	4.4e-15	2e-14
49	0.0019504	0.00042007		103	52	103	2	0	0	2.2e-15	2.9e-14
50	0.0023705	0.00042007		105	53	105	2	0	0	1.8e-15	2.6e-14
51	0.0032106	0.00084014		107	54	107	2	0	0	3.7e-15	4.1e-14
52	0.0040508	0.00084014		109	55	109	2	0	0	2.8e-15	3.7e-14
53	0.0048909	0.00084014		111	56	111	2	0	0	1.8e-15	3.8e-14
-	0.005	-	out								
54	0.005731	0.00084014		113	57	113	2	0	0	1.4e-15	3.2e-14
55	0.0074113	0.0016803		115	58	115	2	0	0	5.6e-15	4.6e-14
56	0.0090916	0.0016803		117	59	117	2	0	0	5e-15	3.8e-14
-	0.01	-	out								
57	0.012452	0.0033606		119	60	119	2	0	0	3.3e-15	6e-14
-	0.015	-	out								
58	0.015813	0.0033606		121	61	121	2	0	0	1e-14	5e-14
59	0.019173	0.0033606		123	62	123	2	0	0	4.2e-15	5e-14
-	0.02	-	out								
-	0.025	-	out								
60	0.025894	0.0067211		125	63	125	2	0	0	4.1e-15	7.1e-14
-	0.03	-	out								
61	0.032616	0.0067211		127	64	127	2	0	0	6.8e-15	5.7e-14
-	0.035	-	out								
62	0.039337	0.0067211		129	65	129	2	0	0	5.6e-15	5.9e-14
-	0.04	-	out								
-	0.045	-	out								
-	0.05	-	out								
63	0.052779	0.013442		131	66	131	2	0	0	1.5e-14	7.8e-14
-	0.055	-	out								
-	0.06	-	out								
-	0.065	-	out								
64	0.066221	0.013442		133	67	133	1	0	0	6.7e-15	6.2e-14
-	0.07	-	out								
-	0.075	-	out								
65	0.079663	0.013442		135	68	135	1	0	0	6.2e-15	2.5e-14
-	0.08	-	out								
-	0.085	-	out								
-	0.09	-	out								
-	0.095	-	out								
-	0.1	-	out								
-	0.105	-	out								
66	0.10655	0.026884		137	69	137	1	0	0	5.9e-15	3.5e-14
-	0.11	-	out								
-	0.115	-	out								
-	0.12	-	out								
-	0.125	-	out								
-	0.13	-	out								
-	0.135	-	out								
-	0.14	-	out								
-	0.145	-	out								
-	0.15	-	out								
-	0.155	-	out								
-	0.16	-	out								
67	0.16032	0.053769		139	70	139	1	0	0	1.5e-14	4.8e-14

-	0.165	- out							
-	0.17	- out							
-	0.175	- out							
-	0.18	- out							
-	0.185	- out							
-	0.19	- out							
-	0.195	- out							
-	0.2	- out							
-	0.205	- out							
-	0.21	- out							
-	0.215	- out							
-	0.22	- out							
-	0.225	- out							
-	0.23	- out							
-	0.235	- out							
-	0.24	- out							
-	0.245	- out							
-	0.25	- out							
-	0.25	- out							
68	0.25932	0.099	141	71	141	1	0	0	1.4e-14 5.3e-14
-	0.27	- out							
-	0.29	- out							
-	0.31	- out							
-	0.33	- out							
-	0.35	- out							
69	0.35832	0.099	143	72	143	1	0	0	1.2e-14 4.4e-14
-	0.37	- out							
-	0.39	- out							
-	0.41	- out							
-	0.43	- out							
-	0.45	- out							
70	0.45732	0.099	145	73	145	1	0	0	6.9e-15 3.4e-14
-	0.47	- out							
-	0.49	- out							
-	0.51	- out							
-	0.53	- out							
-	0.55	- out							
71	0.55632	0.099	147	74	147	1	0	0	5.8e-15 2.6e-14
-	0.57	- out							
-	0.59	- out							
-	0.61	- out							
-	0.63	- out							
-	0.65	- out							
72	0.65532	0.099	148	75	148	1	0	0	2.3e-14 2.2e-14
-	0.67	- out							
-	0.69	- out							
-	0.71	- out							
-	0.73	- out							
-	0.75	- out							
73	0.75432	0.099	149	76	149	1	0	0	1.7e-14 1.9e-14
-	0.77	- out							
-	0.79	- out							
-	0.81	- out							
-	0.83	- out							
-	0.85	- out							
74	0.85332	0.099	150	77	150	2	0	0	1.1e-14 1.6e-14

```
   -        0.87           - out
   -        0.89           - out
   -        0.91           - out
   -        0.93           - out
   -        0.95           - out
  75     0.95232       0.099      151   78  151     2     0      0  1.2e-14  1.1e-14
   -        0.97           - out
   -        0.99           - out
  76      1.0513       0.099      152   79  152     2     0      0  5.5e-15    1e-14
Time-stepping completed.
Solution time: 14 s.
Physical memory: 1.28 GB
Virtual memory: 1.27 GB
Ended at May 28, 2023, 4:17:46 PM.
----- Time-Dependent Solver 1 in Oxygen Pulse/Solution 958 (sol958) ----------->
```

k0Sub=1.51E-6, d=1E-6 (su50)

GENERAL

Description	Value
Solution	k0Sub=1.51E-6, d=1E-6

LOG

```
<---- Compile Equations: Stationary in Oxygen Pulse/Solution 958 (sol958) ------
Started at May 28, 2023, 4:17:48 PM.
Parameter k0Sub = 1.51E-6 (m/s).
Parameter d = 1.0E-6 (m).
Geometry shape function: Linear Lagrange
Number of vertex elements: 10
Minimum element quality: 0.4361
Time: 0 s.
Physical memory: 1.26 GB
Virtual memory: 1.24 GB
Ended at May 28, 2023, 4:17:49 PM.
----- Compile Equations: Stationary in Oxygen Pulse/Solution 958 (sol958) ----->

<---- Dependent Variables 1 in Oxygen Pulse/Solution 958 (sol958) --------------
Started at May 28, 2023, 4:17:49 PM.
Solution time: 0 s.
Physical memory: 1.21 GB
Virtual memory: 1.18 GB
Ended at May 28, 2023, 4:17:49 PM.
----- Dependent Variables 1 in Oxygen Pulse/Solution 958 (sol958) ------------->

<---- Stationary Solver 1 in Oxygen Pulse/Solution 958 (sol958) ----------------
Started at May 28, 2023, 4:17:49 PM.
Linear solver
Number of degrees of freedom solved for: 17724 (plus 963 internal DOFs).
Symmetric matrices found.
Scales for dependent variables:
Concentration (comp2.Oxi): 0.23
Orthonormal null-space function used.
Iter      SolEst     Damping    Stepsize #Res #Jac #Sol   LinErr   LinRes
   1      0.0018   1.0000000      0.0018    1    1    1  5.1e-13  1.3e-13
Solution time: 1 s.
Physical memory: 1.23 GB
Virtual memory: 1.21 GB
Ended at May 28, 2023, 4:17:49 PM.
----- Stationary Solver 1 in Oxygen Pulse/Solution 958 (sol958) --------------->

<---- Compile Equations: Oxygen Probe Pulse in Oxygen Pulse/Solution 958
      (sol958) ----------------------------------------------------------------
Started at May 28, 2023, 4:17:49 PM.
Geometry shape function: Linear Lagrange
Time: 0 s.
Physical memory: 1.23 GB
Virtual memory: 1.21 GB
Ended at May 28, 2023, 4:17:50 PM.
----- Compile Equations: Oxygen Probe Pulse in Oxygen Pulse/Solution 958
      (sol958) --------------------------------------------------------------->

<---- Dependent Variables 2 in Oxygen Pulse/Solution 958 (sol958) --------------
Started at May 28, 2023, 4:17:50 PM.
Initial values of variables solved for: Solution 958 (sol958), Solution Store 1 (sol
985).
Values of variables not solved for: Solution 958 (sol958), Solution Store 1 (sol985)
.
Solution time: 0 s.
Physical memory: 1.24 GB
```

Virtual memory: 1.21 GB
Ended at May 28, 2023, 4:17:50 PM.
----- Dependent Variables 2 in Oxygen Pulse/Solution 958 (sol958) ------------->

<---- Time-Dependent Solver 1 in Oxygen Pulse/Solution 958 (sol958) ------------
Started at May 28, 2023, 4:17:50 PM.
Time-dependent solver (BDF)
Number of degrees of freedom solved for: 17724 (plus 35562 internal DOFs).
Symmetric matrices found.
Scales for dependent variables:
Concentration (comp2.Oxi): 0.23

Step	Time	Stepsize	Res	Jac	Sol	Order	Tfail	NLfail	LinErr	LinRes
0	0	- out	5	3	5			0	8.5e-16	1.5e-15
1	2.5932e-08	2.5932e-08	7	4	7	1	0	0	3.8e-16	1.1e-15
2	5.1863e-08	2.5932e-08	9	5	9	1	0	0	3.7e-16	1.2e-15
3	1.0373e-07	5.1863e-08	11	6	11	2	0	0	3.6e-16	1.3e-15
4	2.0745e-07	1.0373e-07	13	7	13	2	0	0	5.3e-16	1.8e-15
5	4.1491e-07	2.0745e-07	15	8	15	2	0	0	5.3e-16	3.3e-15
6	6.2236e-07	2.0745e-07	17	9	17	2	0	0	7.1e-16	3.8e-15
7	8.2981e-07	2.0745e-07	19	10	19	2	0	0	5.4e-16	3.9e-15
8	1.0373e-06	2.0745e-07	21	11	21	2	0	0	6.4e-16	4e-15
9	1.4522e-06	4.1491e-07	23	12	23	2	0	0	1.1e-15	6.3e-15
10	1.8671e-06	4.1491e-07	25	13	25	2	0	0	8e-16	5.9e-15
11	2.282e-06	4.1491e-07	27	14	27	2	0	0	9.2e-16	5.8e-15
12	2.6969e-06	4.1491e-07	29	15	29	2	0	0	7.8e-16	5.6e-15
13	3.5267e-06	8.2981e-07	31	16	31	2	0	0	1.3e-15	8.2e-15
14	4.3565e-06	8.2981e-07	33	17	33	2	0	0	1.2e-15	6.8e-15
15	5.1863e-06	8.2981e-07	35	18	35	2	0	0	9.8e-16	6.9e-15
16	6.0161e-06	8.2981e-07	37	19	37	2	0	0	8.4e-16	6.3e-15
17	7.6758e-06	1.6596e-06	39	20	39	2	0	0	1.2e-15	9.5e-15
18	9.3354e-06	1.6596e-06	41	21	41	2	0	0	1e-15	7.9e-15
19	1.0995e-05	1.6596e-06	43	22	43	2	0	0	9.8e-16	7.7e-15
20	1.2655e-05	1.6596e-06	45	23	45	2	0	0	1.2e-15	7.1e-15
21	1.4314e-05	1.6596e-06	47	24	47	2	0	0	1.5e-15	5.9e-15
22	1.7634e-05	3.3192e-06	49	25	49	2	0	0	1.9e-15	8.9e-15
23	2.0953e-05	3.3192e-06	51	26	51	2	0	0	1.6e-15	7.8e-15
24	2.4272e-05	3.3192e-06	53	27	53	2	0	0	2.3e-15	7.8e-15
25	2.7591e-05	3.3192e-06	55	28	55	2	0	0	1.3e-15	7.2e-15
26	3.423e-05	6.6385e-06	57	29	57	2	0	0	2e-15	1e-14
27	4.0868e-05	6.6385e-06	59	30	59	2	0	0	1.9e-15	9.1e-15
28	4.7507e-05	6.6385e-06	61	31	61	2	0	0	1.8e-15	9.3e-15
29	5.4145e-05	6.6385e-06	63	32	63	2	0	0	2.1e-15	8.4e-15
30	6.0784e-05	6.6385e-06	65	33	65	2	0	0	2.2e-15	7.5e-15
31	7.4061e-05	1.3277e-05	67	34	67	2	0	0	1.7e-15	1.2e-14
32	8.7338e-05	1.3277e-05	69	35	69	2	0	0	2.6e-15	1e-14
33	0.00010061	1.3277e-05	71	36	71	2	0	0	1.5e-15	1e-14
34	0.00011389	1.3277e-05	73	37	73	2	0	0	1.9e-15	9.2e-15
35	0.00014045	2.6554e-05	75	38	75	2	0	0	1.6e-15	1.3e-14
36	0.000167	2.6554e-05	77	39	77	2	0	0	2.6e-15	1.2e-14
37	0.00019355	2.6554e-05	79	40	79	2	0	0	2.4e-15	1.2e-14
38	0.00022011	2.6554e-05	81	41	81	2	0	0	2.5e-15	1.1e-14
39	0.00027322	5.3108e-05	83	42	83	2	0	0	3e-15	1.6e-14
40	0.00032632	5.3108e-05	85	43	85	2	0	0	2.4e-15	1.3e-14
41	0.00037943	5.3108e-05	87	44	87	2	0	0	3.2e-15	1.3e-14
42	0.00048565	0.00010622	89	45	89	2	0	0	1.8e-15	1.8e-14
43	0.00059186	0.00010622	91	46	91	2	0	0	2e-15	1.6e-14

44	0.00069808	0.00010622		93	47	93	2	0	0	2.7e-15	1.6e-14
45	0.0008043	0.00010622		95	48	95	2	0	0	1.8e-15	1.5e-14
46	0.0010167	0.00021243		97	49	97	2	0	0	4.5e-15	2e-14
47	0.0012292	0.00021243		99	50	99	2	0	0	1.3e-15	1.6e-14
48	0.001654	0.00042486		101	51	101	2	0	0	5e-15	2.8e-14
49	0.0020789	0.00042486		103	52	103	2	0	0	6.3e-15	2.5e-14
50	0.0025038	0.00042486		105	53	105	2	0	0	5.3e-15	2.6e-14
51	0.0029286	0.00042486		107	54	107	2	0	0	3.1e-15	2.3e-14
52	0.0037783	0.00084973		109	55	109	2	0	0	2.1e-15	3.3e-14
53	0.0046281	0.00084973		111	56	111	2	0	0	4e-15	2.8e-14
-	0.005	-	out								
54	0.0063275	0.0016995		113	57	113	2	0	0	5.5e-15	4.7e-14
55	0.008027	0.0016995		115	58	115	2	0	0	2e-15	4.2e-14
56	0.0097264	0.0016995		117	59	117	2	0	0	4e-15	4.5e-14
-	0.01	-	out								
57	0.011426	0.0016995		119	60	119	2	0	0	3.3e-15	3.9e-14
58	0.014825	0.0033989		121	61	121	2	0	0	3.7e-15	4.5e-14
-	0.015	-	out								
59	0.018224	0.0033989		123	62	123	2	0	0	7.4e-15	3.7e-14
-	0.02	-	out								
-	0.025	-	out								
60	0.025022	0.0067978		125	63	125	2	0	0	6.8e-15	6.2e-14
-	0.03	-	out								
61	0.031819	0.0067978		127	64	127	2	0	0	2.4e-15	5.3e-14
-	0.035	-	out								
62	0.038617	0.0067978		129	65	129	2	0	0	2.9e-15	5.8e-14
-	0.04	-	out								
-	0.045	-	out								
63	0.045415	0.0067978		131	66	131	2	0	0	9.8e-15	5.3e-14
-	0.05	-	out								
-	0.055	-	out								
64	0.059011	0.013596		133	67	133	2	0	0	9e-15	6.3e-14
-	0.06	-	out								
-	0.065	-	out								
-	0.07	-	out								
65	0.072606	0.013596		135	68	135	2	0	0	9.3e-15	4.1e-14
-	0.075	-	out								
-	0.08	-	out								
-	0.085	-	out								
-	0.09	-	out								
-	0.095	-	out								
66	0.099798	0.027191		137	69	137	2	0	0	4.6e-15	6e-14
-	0.1	-	out								
-	0.105	-	out								
-	0.11	-	out								
-	0.115	-	out								
-	0.12	-	out								
-	0.125	-	out								
-	0.13	-	out								
-	0.135	-	out								
-	0.14	-	out								
-	0.145	-	out								
-	0.15	-	out								
67	0.15418	0.054383		139	70	139	1	0	0	3.3e-15	6e-14
-	0.155	-	out								
-	0.16	-	out								

-	0.165	-	out							
-	0.17	-	out							
-	0.175	-	out							
-	0.18	-	out							
-	0.185	-	out							
-	0.19	-	out							
-	0.195	-	out							
-	0.2	-	out							
-	0.205	-	out							
68	0.20856	0.054383	141	71	141	1	0	0	7.6e-15	3e-14
-	0.21	-	out							
-	0.215	-	out							
-	0.22	-	out							
-	0.225	-	out							
-	0.23	-	out							
-	0.235	-	out							
-	0.24	-	out							
-	0.245	-	out							
-	0.25	-	out							
-	0.25	-	out							
-	0.27	-	out							
-	0.29	-	out							
69	0.30756	0.099	143	72	143	1	0	0	8.1e-15	3.7e-14
-	0.31	-	out							
-	0.33	-	out							
-	0.35	-	out							
-	0.37	-	out							
-	0.39	-	out							
70	0.40656	0.099	145	73	145	1	0	0	9.8e-15	3.3e-14
-	0.41	-	out							
-	0.43	-	out							
-	0.45	-	out							
-	0.47	-	out							
-	0.49	-	out							
71	0.50556	0.099	147	74	147	1	0	0	7.3e-15	2.8e-14
-	0.51	-	out							
-	0.53	-	out							
-	0.55	-	out							
-	0.57	-	out							
-	0.59	-	out							
72	0.60456	0.099	148	75	148	1	0	0	3.9e-15	2.3e-14
-	0.61	-	out							
-	0.63	-	out							
-	0.65	-	out							
-	0.67	-	out							
-	0.69	-	out							
73	0.70356	0.099	149	76	149	1	0	0	5.7e-15	2e-14
-	0.71	-	out							
-	0.73	-	out							
-	0.75	-	out							
-	0.77	-	out							
-	0.79	-	out							
74	0.80256	0.099	150	77	150	2	0	0	2.2e-15	1.7e-14
-	0.81	-	out							
-	0.83	-	out							
-	0.85	-	out							

```
   -        0.87           - out
   -        0.89           - out
  75     0.90156       0.099      151    78   151      2      0       0  1.1e-14  1.2e-14
   -        0.91           - out
   -        0.93           - out
   -        0.95           - out
   -        0.97           - out
   -        0.99           - out
  76      1.0006       0.099      152    79   152      2      0       0  8.3e-15  1.1e-14
Time-stepping completed.
Solution time: 13 s.
Physical memory: 1.26 GB
Virtual memory: 1.24 GB
Ended at May 28, 2023, 4:18:03 PM.
----- Time-Dependent Solver 1 in Oxygen Pulse/Solution 958 (sol958) ----------->
```

k0Sub=1.51E-6, d=1.5E-6 (su51)

GENERAL

Description	Value
Solution	k0Sub=1.51E-6, d=1.5E-6

LOG

```
<---- Compile Equations: Stationary in Oxygen Pulse/Solution 958 (sol958) ------
Started at May 28, 2023, 4:18:06 PM.
Parameter k0Sub = 1.51E-6 (m/s).
Parameter d = 1.5E-6 (m).
Geometry shape function: Linear Lagrange
Number of vertex elements: 10
Minimum element quality: 0.395
Time: 0 s.
Physical memory: 1.23 GB
Virtual memory: 1.21 GB
Ended at May 28, 2023, 4:18:07 PM.
----- Compile Equations: Stationary in Oxygen Pulse/Solution 958 (sol958) ----->

<---- Dependent Variables 1 in Oxygen Pulse/Solution 958 (sol958) --------------
Started at May 28, 2023, 4:18:07 PM.
Solution time: 0 s.
Physical memory: 1.23 GB
Virtual memory: 1.21 GB
Ended at May 28, 2023, 4:18:07 PM.
----- Dependent Variables 1 in Oxygen Pulse/Solution 958 (sol958) ------------->

<---- Stationary Solver 1 in Oxygen Pulse/Solution 958 (sol958) ----------------
Started at May 28, 2023, 4:18:07 PM.
Linear solver
Number of degrees of freedom solved for: 17823 (plus 950 internal DOFs).
Symmetric matrices found.
Scales for dependent variables:
Concentration (comp2.Oxi): 0.23
Orthonormal null-space function used.
Iter      SolEst     Damping    Stepsize #Res #Jac #Sol   LinErr   LinRes
   1      0.0015   1.0000000      0.0015    1    1    1  3.9e-13  1.2e-13
Solution time: 1 s.
Physical memory: 1.24 GB
Virtual memory: 1.22 GB
Ended at May 28, 2023, 4:18:07 PM.
----- Stationary Solver 1 in Oxygen Pulse/Solution 958 (sol958) --------------->

<---- Compile Equations: Oxygen Probe Pulse in Oxygen Pulse/Solution 958
      (sol958) ----------------------------------------------------------------
Started at May 28, 2023, 4:18:07 PM.
Geometry shape function: Linear Lagrange
Time: 0 s.
Physical memory: 1.24 GB
Virtual memory: 1.22 GB
Ended at May 28, 2023, 4:18:08 PM.
----- Compile Equations: Oxygen Probe Pulse in Oxygen Pulse/Solution 958
      (sol958) --------------------------------------------------------------->

<---- Dependent Variables 2 in Oxygen Pulse/Solution 958 (sol958) --------------
Started at May 28, 2023, 4:18:08 PM.
Initial values of variables solved for: Solution 958 (sol958), Solution Store 1 (sol
985).
Values of variables not solved for: Solution 958 (sol958), Solution Store 1 (sol985)
.
Solution time: 0 s.
Physical memory: 1.24 GB
```

Virtual memory: 1.22 GB
Ended at May 28, 2023, 4:18:08 PM.
----- Dependent Variables 2 in Oxygen Pulse/Solution 958 (sol958) ------------->

<---- Time-Dependent Solver 1 in Oxygen Pulse/Solution 958 (sol958) ------------
Started at May 28, 2023, 4:18:08 PM.
Time-dependent solver (BDF)
Number of degrees of freedom solved for: 17823 (plus 35744 internal DOFs).
Symmetric matrices found.
Scales for dependent variables:
Concentration (comp2.Oxi): 0.23

Step	Time	Stepsize	Res	Jac	Sol	Order	Tfail	NLfail	LinErr	LinRes
0	0	- out	5	3	5			0	9.7e-16	1.4e-15
1	2.5774e-08	2.5774e-08	7	4	7	1	0	0	3.4e-16	1.1e-15
2	5.1548e-08	2.5774e-08	9	5	9	1	0	0	3.6e-16	1.1e-15
3	1.031e-07	5.1548e-08	11	6	11	2	0	0	3.7e-16	1.3e-15
4	2.0619e-07	1.031e-07	13	7	13	2	0	0	4.7e-16	1.8e-15
5	4.1238e-07	2.0619e-07	15	8	15	2	0	0	4.9e-16	3.3e-15
6	6.1858e-07	2.0619e-07	17	9	17	2	0	0	7.1e-16	3.7e-15
7	8.2477e-07	2.0619e-07	19	10	19	2	0	0	7.4e-16	3.9e-15
8	1.031e-06	2.0619e-07	21	11	21	2	0	0	6.2e-16	3.9e-15
9	1.4433e-06	4.1238e-07	23	12	23	2	0	0	7.6e-16	6.2e-15
10	1.8557e-06	4.1238e-07	25	13	25	2	0	0	8.9e-16	5.9e-15
11	2.2681e-06	4.1238e-07	27	14	27	2	0	0	9.5e-16	6e-15
12	2.6805e-06	4.1238e-07	29	15	29	2	0	0	8.8e-16	5.6e-15
13	3.5053e-06	8.2477e-07	31	16	31	2	0	0	1e-15	8.4e-15
14	4.33e-06	8.2477e-07	33	17	33	2	0	0	1.1e-15	7.1e-15
15	5.1548e-06	8.2477e-07	35	18	35	2	0	0	1e-15	7e-15
16	5.9796e-06	8.2477e-07	37	19	37	2	0	0	1.2e-15	6.2e-15
17	7.6291e-06	1.6495e-06	39	20	39	2	0	0	1.6e-15	9.4e-15
18	9.2786e-06	1.6495e-06	41	21	41	2	0	0	1.5e-15	8e-15
19	1.0928e-05	1.6495e-06	43	22	43	2	0	0	1e-15	7.9e-15
20	1.2578e-05	1.6495e-06	45	23	45	2	0	0	1.2e-15	7.2e-15
21	1.4227e-05	1.6495e-06	47	24	47	2	0	0	1.2e-15	6.5e-15
22	1.7526e-05	3.2991e-06	49	25	49	2	0	0	1.1e-15	9.3e-15
23	2.0825e-05	3.2991e-06	51	26	51	2	0	0	1.7e-15	8.1e-15
24	2.4124e-05	3.2991e-06	53	27	53	2	0	0	1.4e-15	7.8e-15
25	2.7424e-05	3.2991e-06	55	28	55	2	0	0	1.3e-15	7.4e-15
26	3.4022e-05	6.5981e-06	57	29	57	2	0	0	1.4e-15	1.1e-14
27	4.062e-05	6.5981e-06	59	30	59	2	0	0	2.3e-15	9.1e-15
28	4.7218e-05	6.5981e-06	61	31	61	2	0	0	2.2e-15	9.3e-15
29	5.3816e-05	6.5981e-06	63	32	63	2	0	0	1.2e-15	8.5e-15
30	6.0414e-05	6.5981e-06	65	33	65	2	0	0	1.9e-15	7.5e-15
31	7.361e-05	1.3196e-05	67	34	67	2	0	0	1.4e-15	1.1e-14
32	8.6807e-05	1.3196e-05	69	35	69	2	0	0	2e-15	9.5e-15
33	0.0001	1.3196e-05	71	36	71	2	0	0	2.7e-15	9.5e-15
34	0.0001132	1.3196e-05	73	37	73	2	0	0	2.4e-15	9e-15
35	0.00013959	2.6393e-05	75	38	75	2	0	0	1.8e-15	1.3e-14
36	0.00016598	2.6393e-05	77	39	77	2	0	0	2.2e-15	1.2e-14
37	0.00019238	2.6393e-05	79	40	79	2	0	0	3.5e-15	1.2e-14
38	0.00021877	2.6393e-05	81	41	81	2	0	0	1.3e-15	1.1e-14
39	0.00027155	5.2785e-05	83	42	83	2	0	0	1.8e-15	1.5e-14
40	0.00032434	5.2785e-05	85	43	85	2	0	0	3.7e-15	1.3e-14
41	0.00037712	5.2785e-05	87	44	87	2	0	0	2.2e-15	1.3e-14
42	0.0004827	0.00010557	89	45	89	2	0	0	1.6e-15	1.9e-14
43	0.00058827	0.00010557	91	46	91	2	0	0	2.8e-15	1.6e-14

44	0.00069384	0.00010557		93	47	93	2	0	0	2.8e-15	1.5e-14
45	0.00079941	0.00010557		95	48	95	2	0	0	1.5e-15	1.4e-14
46	0.0010105	0.00021114		97	49	97	2	0	0	3.2e-15	2e-14
47	0.0012217	0.00021114		99	50	99	2	0	0	3.1e-15	1.7e-14
48	0.001644	0.00042228		101	51	101	2	0	0	3.4e-15	2.8e-14
49	0.0020662	0.00042228		103	52	103	2	0	0	3.4e-15	2.5e-14
50	0.0024885	0.00042228		105	53	105	2	0	0	2.1e-15	2.4e-14
51	0.0033331	0.00084456		107	54	107	2	0	0	2.3e-15	3.1e-14
52	0.0041777	0.00084456		109	55	109	2	0	0	3.1e-15	2.5e-14
-	0.005	-	out								
53	0.0050222	0.00084456		111	56	111	2	0	0	3.1e-15	2.7e-14
54	0.0067113	0.0016891		113	57	113	2	0	0	3e-15	4.1e-14
55	0.0084005	0.0016891		115	58	115	2	0	0	1e-14	3.7e-14
-	0.01	-	out								
56	0.011779	0.0033782		117	59	117	2	0	0	4e-15	5.7e-14
-	0.015	-	out								
57	0.015157	0.0033782		119	60	119	2	0	0	4.9e-15	5e-14
58	0.018535	0.0033782		121	61	121	2	0	0	1.8e-15	4.8e-14
-	0.02	-	out								
59	0.021913	0.0033782		123	62	123	2	0	0	1.8e-15	3.9e-14
-	0.025	-	out								
60	0.02867	0.0067565		125	63	125	2	0	0	3.8e-15	5.3e-14
-	0.03	-	out								
-	0.035	-	out								
61	0.035426	0.0067565		127	64	127	2	0	0	3.2e-15	4.8e-14
-	0.04	-	out								
-	0.045	-	out								
62	0.048939	0.013513		129	65	129	2	0	0	5.6e-15	7.6e-14
-	0.05	-	out								
-	0.055	-	out								
-	0.06	-	out								
63	0.062452	0.013513		131	66	131	2	0	0	4.5e-15	6.6e-14
-	0.065	-	out								
-	0.07	-	out								
-	0.075	-	out								
64	0.075965	0.013513		133	67	133	2	0	0	6.8e-15	6.1e-14
-	0.08	-	out								
-	0.085	-	out								
-	0.09	-	out								
-	0.095	-	out								
-	0.1	-	out								
65	0.10299	0.027026		135	68	135	2	0	0	1.2e-14	6.5e-14
-	0.105	-	out								
-	0.11	-	out								
-	0.115	-	out								
-	0.12	-	out								
-	0.125	-	out								
-	0.13	-	out								
-	0.135	-	out								
-	0.14	-	out								
-	0.145	-	out								
-	0.15	-	out								
-	0.155	-	out								
66	0.15704	0.054052		137	69	137	1	0	0	1.8e-14	5.7e-14
-	0.16	-	out								
-	0.165	-	out								

-	0.17	-	out							
-	0.175	-	out							
-	0.18	-	out							
-	0.185	-	out							
-	0.19	-	out							
-	0.195	-	out							
-	0.2	-	out							
-	0.205	-	out							
-	0.21	-	out							
67	0.2111	0.054052	139	70	139	1	0	0	2.1e-14	3.3e-14
-	0.215	-	out							
-	0.22	-	out							
-	0.225	-	out							
-	0.23	-	out							
-	0.235	-	out							
-	0.24	-	out							
-	0.245	-	out							
-	0.25	-	out							
-	0.25	-	out							
-	0.27	-	out							
-	0.29	-	out							
-	0.31	-	out							
68	0.3101	0.099	141	71	141	1	0	0	9.5e-15	4.1e-14
-	0.33	-	out							
-	0.35	-	out							
-	0.37	-	out							
-	0.39	-	out							
69	0.4091	0.099	143	72	143	1	0	0	4.6e-15	3.4e-14
-	0.41	-	out							
-	0.43	-	out							
-	0.45	-	out							
-	0.47	-	out							
-	0.49	-	out							
70	0.5081	0.099	145	73	145	1	0	0	1.8e-14	2.9e-14
-	0.51	-	out							
-	0.53	-	out							
-	0.55	-	out							
-	0.57	-	out							
-	0.59	-	out							
71	0.6071	0.099	147	74	147	1	0	0	1.5e-14	2.5e-14
-	0.61	-	out							
-	0.63	-	out							
-	0.65	-	out							
-	0.67	-	out							
-	0.69	-	out							
72	0.7061	0.099	148	75	148	1	0	0	5.7e-15	2e-14
-	0.71	-	out							
-	0.73	-	out							
-	0.75	-	out							
-	0.77	-	out							
-	0.79	-	out							
73	0.8051	0.099	149	76	149	1	0	0	8.3e-15	1.8e-14
-	0.81	-	out							
-	0.83	-	out							
-	0.85	-	out							
-	0.87	-	out							

```
    -         0.89            - out
   74       0.9041        0.099       150    77   150      2      0       0  1.2e-14  1.5e-14
    -         0.91            - out
    -         0.93            - out
    -         0.95            - out
    -         0.97            - out
    -         0.99            - out
   75       1.0031        0.099       151    78   151      2      0       0  5.3e-15  9.5e-15
Time-stepping completed.
Solution time: 14 s.
Physical memory: 1.27 GB
Virtual memory: 1.25 GB
Ended at May 28, 2023, 4:18:21 PM.
----- Time-Dependent Solver 1 in Oxygen Pulse/Solution 958 (sol958) ----------->
```

k0Sub=1.51E-6, d=2E-6 (su52)

GENERAL

Description	Value
Solution	k0Sub=1.51E-6, d=2E-6

LOG

```
<---- Compile Equations: Stationary in Oxygen Pulse/Solution 958 (sol958) ------
Started at May 28, 2023, 4:18:24 PM.
Parameter k0Sub = 1.51E-6 (m/s).
Parameter d = 2.0E-6 (m).
Geometry shape function: Linear Lagrange
Number of vertex elements: 10
Minimum element quality: 0.4112
Time: 0 s.
Physical memory: 1.24 GB
Virtual memory: 1.22 GB
Ended at May 28, 2023, 4:18:24 PM.
----- Compile Equations: Stationary in Oxygen Pulse/Solution 958 (sol958) ----->

<---- Dependent Variables 1 in Oxygen Pulse/Solution 958 (sol958) --------------
Started at May 28, 2023, 4:18:24 PM.
Solution time: 0 s.
Physical memory: 1.24 GB
Virtual memory: 1.22 GB
Ended at May 28, 2023, 4:18:24 PM.
----- Dependent Variables 1 in Oxygen Pulse/Solution 958 (sol958) ------------->

<---- Stationary Solver 1 in Oxygen Pulse/Solution 958 (sol958) ----------------
Started at May 28, 2023, 4:18:24 PM.
Linear solver
Number of degrees of freedom solved for: 17907 (plus 938 internal DOFs).
Symmetric matrices found.
Scales for dependent variables:
Concentration (comp2.Oxi): 0.23
Orthonormal null-space function used.
Iter      SolEst     Damping    Stepsize #Res #Jac #Sol   LinErr   LinRes
   1      0.0014   1.0000000      0.0014    1    1    1    5e-13    1e-13
Solution time: 1 s.
Physical memory: 1.25 GB
Virtual memory: 1.23 GB
Ended at May 28, 2023, 4:18:25 PM.
----- Stationary Solver 1 in Oxygen Pulse/Solution 958 (sol958) --------------->

<---- Compile Equations: Oxygen Probe Pulse in Oxygen Pulse/Solution 958
      (sol958) -----------------------------------------------------------------
Started at May 28, 2023, 4:18:25 PM.
Geometry shape function: Linear Lagrange
Time: 1 s.
Physical memory: 1.25 GB
Virtual memory: 1.23 GB
Ended at May 28, 2023, 4:18:25 PM.
----- Compile Equations: Oxygen Probe Pulse in Oxygen Pulse/Solution 958
      (sol958) ---------------------------------------------------------------->

<---- Dependent Variables 2 in Oxygen Pulse/Solution 958 (sol958) --------------
Started at May 28, 2023, 4:18:25 PM.
Initial values of variables solved for: Solution 958 (sol958), Solution Store 1 (sol
985).
Values of variables not solved for: Solution 958 (sol958), Solution Store 1 (sol985)
.
Solution time: 0 s.
Physical memory: 1.25 GB
```

Virtual memory: 1.23 GB
Ended at May 28, 2023, 4:18:25 PM.
----- Dependent Variables 2 in Oxygen Pulse/Solution 958 (sol958) ------------->

<---- Time-Dependent Solver 1 in Oxygen Pulse/Solution 958 (sol958) ------------
Started at May 28, 2023, 4:18:25 PM.
Time-dependent solver (BDF)
Number of degrees of freedom solved for: 17907 (plus 35898 internal DOFs).
Symmetric matrices found.
Scales for dependent variables:
Concentration (comp2.Oxi): 0.23

Step	Time	Stepsize		Res	Jac	Sol	Order	Tfail	NLfail	LinErr	LinRes
0	0	-	out	5	3	5			0	9.3e-16	1.4e-15
1	2.5791e-08	2.5791e-08		7	4	7	1	0	0	3.4e-16	1e-15
2	5.1582e-08	2.5791e-08		9	5	9	1	0	0	3.3e-16	1.2e-15
3	1.0316e-07	5.1582e-08		11	6	11	2	0	0	3.8e-16	1.2e-15
4	2.0633e-07	1.0316e-07		13	7	13	2	0	0	4.9e-16	1.8e-15
5	4.1265e-07	2.0633e-07		15	8	15	2	0	0	6.1e-16	3.4e-15
6	6.1898e-07	2.0633e-07		17	9	17	2	0	0	5.3e-16	3.8e-15
7	8.2531e-07	2.0633e-07		19	10	19	2	0	0	6e-16	4.1e-15
8	1.0316e-06	2.0633e-07		21	11	21	2	0	0	6.4e-16	4.2e-15
9	1.4443e-06	4.1265e-07		23	12	23	2	0	0	8.4e-16	6.5e-15
10	1.8569e-06	4.1265e-07		25	13	25	2	0	0	7.7e-16	6e-15
11	2.2696e-06	4.1265e-07		27	14	27	2	0	0	8e-16	5.9e-15
12	2.6823e-06	4.1265e-07		29	15	29	2	0	0	7.7e-16	5.5e-15
13	3.5076e-06	8.2531e-07		31	16	31	2	0	0	1.1e-15	8.4e-15
14	4.3329e-06	8.2531e-07		33	17	33	2	0	0	1.3e-15	7.5e-15
15	5.1582e-06	8.2531e-07		35	18	35	2	0	0	9.4e-16	7.1e-15
16	5.9835e-06	8.2531e-07		37	19	37	2	0	0	9.6e-16	6.5e-15
17	7.6341e-06	1.6506e-06		39	20	39	2	0	0	1.2e-15	9.7e-15
18	9.2847e-06	1.6506e-06		41	21	41	2	0	0	1.1e-15	8.1e-15
19	1.0935e-05	1.6506e-06		43	22	43	2	0	0	1.2e-15	8.3e-15
20	1.2586e-05	1.6506e-06		45	23	45	2	0	0	1.5e-15	7.5e-15
21	1.4237e-05	1.6506e-06		47	24	47	2	0	0	1.2e-15	6.6e-15
22	1.7538e-05	3.3012e-06		49	25	49	2	0	0	1.5e-15	9.7e-15
23	2.0839e-05	3.3012e-06		51	26	51	2	0	0	1.1e-15	8.4e-15
24	2.414e-05	3.3012e-06		53	27	53	2	0	0	1.4e-15	8.5e-15
25	2.7442e-05	3.3012e-06		55	28	55	2	0	0	1.1e-15	7.7e-15
26	3.4044e-05	6.6025e-06		57	29	57	2	0	0	1.5e-15	1.1e-14
27	4.0646e-05	6.6025e-06		59	30	59	2	0	0	2.3e-15	1e-14
28	4.7249e-05	6.6025e-06		61	31	61	2	0	0	1.5e-15	9.4e-15
29	5.3851e-05	6.6025e-06		63	32	63	2	0	0	1.3e-15	8.6e-15
30	6.0454e-05	6.6025e-06		65	33	65	2	0	0	1.4e-15	7.7e-15
31	7.3659e-05	1.3205e-05		67	34	67	2	0	0	1.5e-15	1.1e-14
32	8.6864e-05	1.3205e-05		69	35	69	2	0	0	1.5e-15	9.9e-15
33	0.00010007	1.3205e-05		71	36	71	2	0	0	1.8e-15	9.9e-15
34	0.00011327	1.3205e-05		73	37	73	2	0	0	1.4e-15	9e-15
35	0.00013968	2.641e-05		75	38	75	2	0	0	2.5e-15	1.4e-14
36	0.00016609	2.641e-05		77	39	77	2	0	0	2.2e-15	1.1e-14
37	0.0001925	2.641e-05		79	40	79	2	0	0	2.4e-15	1.1e-14
38	0.00021891	2.641e-05		81	41	81	2	0	0	2.8e-15	1e-14
39	0.00027173	5.282e-05		83	42	83	2	0	0	2.2e-15	1.5e-14
40	0.00032455	5.282e-05		85	43	85	2	0	0	2.1e-15	1.2e-14
41	0.00037737	5.282e-05		87	44	87	2	0	0	3.2e-15	1.3e-14
42	0.00048301	0.00010564		89	45	89	2	0	0	3.7e-15	1.8e-14
43	0.00058865	0.00010564		91	46	91	2	0	0	1.9e-15	1.5e-14

44	0.00069429	0.00010564		93	47	93	2	0	0	3.7e-15	1.5e-14
45	0.00079993	0.00010564		95	48	95	2	0	0	3.1e-15	1.3e-14
46	0.0010112	0.00021128		97	49	97	2	0	0	2.4e-15	1.9e-14
47	0.0012225	0.00021128		99	50	99	2	0	0	2.5e-15	1.6e-14
48	0.001645	0.00042256		101	51	101	2	0	0	2.6e-15	2.6e-14
49	0.0020676	0.00042256		103	52	103	2	0	0	2e-15	2.5e-14
50	0.0024902	0.00042256		105	53	105	2	0	0	1.7e-15	2.4e-14
51	0.0033353	0.00084512		107	54	107	2	0	0	2.8e-15	3e-14
52	0.0041804	0.00084512		109	55	109	2	0	0	2.2e-15	2.1e-14
-	0.005	-	out								
53	0.0058706	0.0016902		111	56	111	2	0	0	2.2e-15	3.5e-14
54	0.0075609	0.0016902		113	57	113	2	0	0	1.8e-15	3.3e-14
55	0.0092511	0.0016902		115	58	115	2	0	0	2.6e-15	3.7e-14
-	0.01	-	out								
56	0.010941	0.0016902		117	59	117	2	0	0	1.7e-15	3.5e-14
57	0.014322	0.0033805		119	60	119	2	0	0	4e-15	4.5e-14
-	0.015	-	out								
58	0.017702	0.0033805		121	61	121	2	0	0	3e-15	3.6e-14
-	0.02	-	out								
59	0.024463	0.0067609		123	62	123	2	0	0	3.1e-15	5.8e-14
-	0.025	-	out								
-	0.03	-	out								
60	0.031224	0.0067609		125	63	125	2	0	0	2.6e-15	5.5e-14
-	0.035	-	out								
61	0.037985	0.0067609		127	64	127	2	0	0	4.1e-15	5.6e-14
-	0.04	-	out								
-	0.045	-	out								
-	0.05	-	out								
62	0.051507	0.013522		129	65	129	2	0	0	4.4e-15	7.2e-14
-	0.055	-	out								
-	0.06	-	out								
-	0.065	-	out								
63	0.065029	0.013522		131	66	131	2	0	0	5.8e-15	5.3e-14
-	0.07	-	out								
-	0.075	-	out								
-	0.08	-	out								
-	0.085	-	out								
-	0.09	-	out								
64	0.092072	0.027044		133	67	133	2	0	0	1.1e-14	8.1e-14
-	0.095	-	out								
-	0.1	-	out								
-	0.105	-	out								
-	0.11	-	out								
-	0.115	-	out								
65	0.11912	0.027044		135	68	135	1	0	0	8e-15	5.6e-14
-	0.12	-	out								
-	0.125	-	out								
-	0.13	-	out								
-	0.135	-	out								
-	0.14	-	out								
-	0.145	-	out								
-	0.15	-	out								
-	0.155	-	out								
-	0.16	-	out								
-	0.165	-	out								
-	0.17	-	out								

66	0.1732	0.054087	137	69	137	1	0	0	1e-14	3.1e-14
-	0.175	- out								
-	0.18	- out								
-	0.185	- out								
-	0.19	- out								
-	0.195	- out								
-	0.2	- out								
-	0.205	- out								
-	0.21	- out								
-	0.215	- out								
-	0.22	- out								
-	0.225	- out								
-	0.23	- out								
-	0.235	- out								
-	0.24	- out								
-	0.245	- out								
-	0.25	- out								
-	0.25	- out								
-	0.27	- out								
67	0.2722	0.099	139	70	139	1	0	0	6.7e-15	4.5e-14
-	0.29	- out								
-	0.31	- out								
-	0.33	- out								
-	0.35	- out								
-	0.37	- out								
68	0.3712	0.099	141	71	141	1	0	0	8.1e-15	4.1e-14
-	0.39	- out								
-	0.41	- out								
-	0.43	- out								
-	0.45	- out								
-	0.47	- out								
69	0.4702	0.099	143	72	143	1	0	0	8.8e-15	3.4e-14
-	0.49	- out								
-	0.51	- out								
-	0.53	- out								
-	0.55	- out								
70	0.5692	0.099	145	73	145	1	0	0	3.5e-15	2.7e-14
-	0.57	- out								
-	0.59	- out								
-	0.61	- out								
-	0.63	- out								
-	0.65	- out								
71	0.6682	0.099	146	74	146	1	0	0	2.8e-15	2.3e-14
-	0.67	- out								
-	0.69	- out								
-	0.71	- out								
-	0.73	- out								
-	0.75	- out								
72	0.7672	0.099	147	75	147	1	0	0	4.4e-15	2.1e-14
-	0.77	- out								
-	0.79	- out								
-	0.81	- out								
-	0.83	- out								
-	0.85	- out								
73	0.8662	0.099	148	76	148	2	0	0	9e-15	1.7e-14
-	0.87	- out								

```
    -        0.89           - out
    -        0.91           - out
    -        0.93           - out
    -        0.95           - out
   74      0.9652       0.099      149   77  149     2     0      0    1e-14  1.1e-14
    -        0.97           - out
    -        0.99           - out
   75      1.0642       0.099      150   78  150     2     0      0  6.5e-15  1.1e-14
Time-stepping completed.
Solution time: 14 s.
Physical memory: 1.28 GB
Virtual memory: 1.26 GB
Ended at May 28, 2023, 4:18:39 PM.
----- Time-Dependent Solver 1 in Oxygen Pulse/Solution 958 (sol958) ----------->
```

k0Sub=1.51E-6, d=2.4E-6 (su53)

GENERAL

Description	Value
Solution	k0Sub=1.51E-6, d=2.4E-6

LOG

```
<---- Compile Equations: Stationary in Oxygen Pulse/Solution 958 (sol958) ------
Started at May 28, 2023, 4:18:42 PM.
Parameter k0Sub = 1.51E-6 (m/s).
Parameter d = 2.4E-6 (m).
Geometry shape function: Linear Lagrange
Number of vertex elements: 10
Minimum element quality: 0.4544
Time: 0 s.
Physical memory: 1.26 GB
Virtual memory: 1.24 GB
Ended at May 28, 2023, 4:18:42 PM.
----- Compile Equations: Stationary in Oxygen Pulse/Solution 958 (sol958) ----->

<---- Dependent Variables 1 in Oxygen Pulse/Solution 958 (sol958) --------------
Started at May 28, 2023, 4:18:42 PM.
Solution time: 0 s.
Physical memory: 1.26 GB
Virtual memory: 1.24 GB
Ended at May 28, 2023, 4:18:42 PM.
----- Dependent Variables 1 in Oxygen Pulse/Solution 958 (sol958) ------------->

<---- Stationary Solver 1 in Oxygen Pulse/Solution 958 (sol958) ----------------
Started at May 28, 2023, 4:18:42 PM.
Linear solver
Number of degrees of freedom solved for: 18002 (plus 937 internal DOFs).
Symmetric matrices found.
Scales for dependent variables:
Concentration (comp2.Oxi): 0.23
Orthonormal null-space function used.
Iter      SolEst     Damping    Stepsize #Res #Jac #Sol   LinErr   LinRes
   1      0.0012   1.0000000      0.0012    1    1    1  4.5e-13  9.6e-14
Solution time: 1 s.
Physical memory: 1.26 GB
Virtual memory: 1.24 GB
Ended at May 28, 2023, 4:18:43 PM.
----- Stationary Solver 1 in Oxygen Pulse/Solution 958 (sol958) --------------->

<---- Compile Equations: Oxygen Probe Pulse in Oxygen Pulse/Solution 958
      (sol958) -----------------------------------------------------------------
Started at May 28, 2023, 4:18:43 PM.
Geometry shape function: Linear Lagrange
Time: 1 s.
Physical memory: 1.26 GB
Virtual memory: 1.24 GB
Ended at May 28, 2023, 4:18:43 PM.
----- Compile Equations: Oxygen Probe Pulse in Oxygen Pulse/Solution 958
      (sol958) ---------------------------------------------------------------->

<---- Dependent Variables 2 in Oxygen Pulse/Solution 958 (sol958) --------------
Started at May 28, 2023, 4:18:43 PM.
Initial values of variables solved for: Solution 958 (sol958), Solution Store 1 (sol
985).
Values of variables not solved for: Solution 958 (sol958), Solution Store 1 (sol985)
.
Solution time: 0 s.
Physical memory: 1.26 GB
```

```
Virtual memory: 1.24 GB
Ended at May 28, 2023, 4:18:43 PM.
----- Dependent Variables 2 in Oxygen Pulse/Solution 958 (sol958) ------------->

<---- Time-Dependent Solver 1 in Oxygen Pulse/Solution 958 (sol958) ------------
Started at May 28, 2023, 4:18:43 PM.
Time-dependent solver (BDF)
Number of degrees of freedom solved for: 18002 (plus 36084 internal DOFs).
Symmetric matrices found.
Scales for dependent variables:
Concentration (comp2.Oxi): 0.23
Step       Time    Stepsize      Res  Jac   Sol Order Tfail NLfail   LinErr   LinRes
   0          0           - out    5    3     5                  0  1.1e-15  1.4e-15
   1 2.5913e-08  2.5913e-08        7    4     7     1     0      0  3.5e-16  1.1e-15
   2 5.1826e-08  2.5913e-08        9    5     9     1     0      0  3.6e-16  1.2e-15
   3 1.0365e-07  5.1826e-08       11    6    11     2     0      0    4e-16  1.3e-15
   4 2.0731e-07  1.0365e-07       13    7    13     2     0      0  4.6e-16  1.7e-15
   5 4.1461e-07  2.0731e-07       15    8    15     2     0      0  5.1e-16  3.5e-15
   6 6.2192e-07  2.0731e-07       17    9    17     2     0      0  4.5e-16  3.7e-15
   7 8.2922e-07  2.0731e-07       19   10    19     2     0      0  6.5e-16    4e-15
   8 1.0365e-06  2.0731e-07       21   11    21     2     0      0  6.2e-16    4e-15
   9 1.4511e-06  4.1461e-07       23   12    23     2     0      0  8.9e-16  6.5e-15
  10 1.8658e-06  4.1461e-07       25   13    25     2     0      0  7.3e-16  6.1e-15
  11 2.2804e-06  4.1461e-07       27   14    27     2     0      0    1e-15  6.3e-15
  12  2.695e-06  4.1461e-07       29   15    29     2     0      0  8.8e-16  5.8e-15
  13 3.5242e-06  8.2922e-07       31   16    31     2     0      0  1.1e-15  8.8e-15
  14 4.3534e-06  8.2922e-07       33   17    33     2     0      0  1.1e-15  7.6e-15
  15 5.1826e-06  8.2922e-07       35   18    35     2     0      0  1.3e-15  7.2e-15
  16 6.0119e-06  8.2922e-07       37   19    37     2     0      0  1.1e-15  6.7e-15
  17 7.6703e-06  1.6584e-06       39   20    39     2     0      0  1.6e-15  9.9e-15
  18 9.3288e-06  1.6584e-06       41   21    41     2     0      0  1.3e-15  8.5e-15
  19 1.0987e-05  1.6584e-06       43   22    43     2     0      0  1.3e-15  8.6e-15
  20 1.2646e-05  1.6584e-06       45   23    45     2     0      0  1.2e-15  7.9e-15
  21 1.4304e-05  1.6584e-06       47   24    47     2     0      0  1.7e-15  6.8e-15
  22 1.7621e-05  3.3169e-06       49   25    49     2     0      0  1.4e-15  9.8e-15
  23 2.0938e-05  3.3169e-06       51   26    51     2     0      0  1.5e-15  8.7e-15
  24 2.4255e-05  3.3169e-06       53   27    53     2     0      0  1.6e-15  8.8e-15
  25 2.7572e-05  3.3169e-06       55   28    55     2     0      0  1.5e-15  7.8e-15
  26 3.4205e-05  6.6338e-06       57   29    57     2     0      0  2.2e-15  1.2e-14
  27 4.0839e-05  6.6338e-06       59   30    59     2     0      0  2.1e-15    1e-14
  28 4.7473e-05  6.6338e-06       61   31    61     2     0      0  2.4e-15    1e-14
  29 5.4107e-05  6.6338e-06       63   32    63     2     0      0  1.9e-15  9.1e-15
  30 6.0741e-05  6.6338e-06       65   33    65     2     0      0    2e-15  7.9e-15
  31 7.4008e-05  1.3268e-05       67   34    67     2     0      0  2.8e-15  1.2e-14
  32 8.7276e-05  1.3268e-05       69   35    69     2     0      0  3.3e-15  9.8e-15
  33 0.00010054  1.3268e-05       71   36    71     2     0      0  2.1e-15  9.9e-15
  34 0.00011381  1.3268e-05       73   37    73     2     0      0  1.5e-15  9.3e-15
  35 0.00014035  2.6535e-05       75   38    75     2     0      0  1.7e-15  1.4e-14
  36 0.00016688  2.6535e-05       77   39    77     2     0      0  2.8e-15  1.2e-14
  37 0.00019342  2.6535e-05       79   40    79     2     0      0  1.6e-15  1.2e-14
  38 0.00021995  2.6535e-05       81   41    81     2     0      0  2.1e-15  1.1e-14
  39 0.00027302   5.307e-05       83   42    83     2     0      0  2.6e-15  1.5e-14
  40 0.00032609   5.307e-05       85   43    85     2     0      0  2.2e-15  1.3e-14
  41 0.00037916   5.307e-05       87   44    87     2     0      0  2.2e-15  1.3e-14
  42  0.0004853  0.00010614       89   45    89     2     0      0    4e-15  1.9e-14
  43 0.00059144  0.00010614       91   46    91     2     0      0  4.6e-15  1.5e-14
```

44	0.00069758	0.00010614		93	47	93	2	0	0	2.4e-15	1.6e-14
45	0.00080372	0.00010614		95	48	95	2	0	0	2.4e-15	1.4e-14
46	0.001016	0.00021228		97	49	97	2	0	0	2.3e-15	1.8e-14
47	0.0012283	0.00021228		99	50	99	2	0	0	3.7e-15	1.6e-14
48	0.0016528	0.00042456		101	51	101	2	0	0	7.8e-15	2.6e-14
49	0.0020774	0.00042456		103	52	103	2	0	0	4.5e-15	2.4e-14
50	0.002502	0.00042456		105	53	105	2	0	0	4e-15	2.6e-14
51	0.0029265	0.00042456		107	54	107	2	0	0	2.2e-15	2e-14
52	0.0037757	0.00084912		109	55	109	2	0	0	2.8e-15	2.5e-14
53	0.0046248	0.00084912		111	56	111	2	0	0	2e-15	2e-14
-	0.005	-	out								
54	0.006323	0.0016982		113	57	113	2	0	0	3e-15	3.3e-14
55	0.0080213	0.0016982		115	58	115	2	0	0	2.1e-15	3.4e-14
-	0.01	-	out								
56	0.011418	0.0033965		117	59	117	2	0	0	7.4e-15	5.1e-14
57	0.014814	0.0033965		119	60	119	2	0	0	3.4e-15	4.7e-14
-	0.015	-	out								
58	0.018211	0.0033965		121	61	121	2	0	0	4.9e-15	4.6e-14
-	0.02	-	out								
-	0.025	-	out								
59	0.025004	0.006793		123	62	123	2	0	0	4.8e-15	6e-14
-	0.03	-	out								
60	0.031797	0.006793		125	63	125	2	0	0	7.3e-15	4.5e-14
-	0.035	-	out								
-	0.04	-	out								
-	0.045	-	out								
61	0.045383	0.013586		127	64	127	2	0	0	4.4e-15	7.8e-14
-	0.05	-	out								
-	0.055	-	out								
62	0.058969	0.013586		129	65	129	2	0	0	6.3e-15	6.6e-14
-	0.06	-	out								
-	0.065	-	out								
-	0.07	-	out								
63	0.072555	0.013586		131	66	131	2	0	0	5.5e-15	6.7e-14
-	0.075	-	out								
-	0.08	-	out								
-	0.085	-	out								
64	0.086141	0.013586		133	67	133	2	0	0	7.9e-15	5e-14
-	0.09	-	out								
-	0.095	-	out								
-	0.1	-	out								
-	0.105	-	out								
-	0.11	-	out								
65	0.11331	0.027172		135	68	135	2	0	0	3.5e-15	4.7e-14
-	0.115	-	out								
-	0.12	-	out								
-	0.125	-	out								
-	0.13	-	out								
-	0.135	-	out								
-	0.14	-	out								
-	0.145	-	out								
-	0.15	-	out								
-	0.155	-	out								
-	0.16	-	out								
-	0.165	-	out								
66	0.16766	0.054344		137	69	137	1	0	0	1.2e-14	5.3e-14

-	0.17	- out								
-	0.175	- out								
-	0.18	- out								
-	0.185	- out								
-	0.19	- out								
-	0.195	- out								
-	0.2	- out								
-	0.205	- out								
-	0.21	- out								
-	0.215	- out								
-	0.22	- out								
67	0.222	0.054344	139	70	139	1	0	0	8.2e-15	3.4e-14
-	0.225	- out								
-	0.23	- out								
-	0.235	- out								
-	0.24	- out								
-	0.245	- out								
-	0.25	- out								
-	0.25	- out								
-	0.27	- out								
-	0.29	- out								
-	0.31	- out								
68	0.321	0.099	141	71	141	1	0	0	6.3e-15	4.2e-14
-	0.33	- out								
-	0.35	- out								
-	0.37	- out								
-	0.39	- out								
-	0.41	- out								
69	0.42	0.099	143	72	143	1	0	0	1.6e-14	3.7e-14
-	0.43	- out								
-	0.45	- out								
-	0.47	- out								
-	0.49	- out								
-	0.51	- out								
70	0.519	0.099	145	73	145	1	0	0	9.3e-15	3e-14
-	0.53	- out								
-	0.55	- out								
-	0.57	- out								
-	0.59	- out								
-	0.61	- out								
71	0.618	0.099	147	74	147	1	0	0	1.4e-14	2.4e-14
-	0.63	- out								
-	0.65	- out								
-	0.67	- out								
-	0.69	- out								
-	0.71	- out								
72	0.717	0.099	148	75	148	1	0	0	4.1e-15	2.1e-14
-	0.73	- out								
-	0.75	- out								
-	0.77	- out								
-	0.79	- out								
-	0.81	- out								
73	0.816	0.099	149	76	149	1	0	0	6e-15	1.9e-14
-	0.83	- out								
-	0.85	- out								
-	0.87	- out								

```
   -         0.89           - out
   -         0.91           - out
  74        0.915       0.099      150    77   150      2      0       0  5.4e-15  1.6e-14
   -         0.93           - out
   -         0.95           - out
   -         0.97           - out
   -         0.99           - out
  75        1.014       0.099      151    78   151      2      0       0  5.1e-15  9.8e-15
Time-stepping completed.
Solution time: 14 s.
Physical memory: 1.29 GB
Virtual memory: 1.27 GB
Ended at May 28, 2023, 4:18:57 PM.
----- Time-Dependent Solver 1 in Oxygen Pulse/Solution 958 (sol958) ----------->
```

k0Sub=1.51E-6, d=3E-6 (su54)

GENERAL

Description	Value
Solution	k0Sub=1.51E-6, d=3E-6

LOG

```
<---- Compile Equations: Stationary in Oxygen Pulse/Solution 958 (sol958) ------
Started at May 28, 2023, 4:19:00 PM.
Parameter k0Sub = 1.51E-6 (m/s).
Parameter d = 3.0E-6 (m).
Geometry shape function: Linear Lagrange
Number of vertex elements: 10
Minimum element quality: 0.4275
Time: 1 s.
Physical memory: 1.27 GB
Virtual memory: 1.25 GB
Ended at May 28, 2023, 4:19:00 PM.
----- Compile Equations: Stationary in Oxygen Pulse/Solution 958 (sol958) ----->

<---- Dependent Variables 1 in Oxygen Pulse/Solution 958 (sol958) --------------
Started at May 28, 2023, 4:19:00 PM.
Solution time: 0 s.
Physical memory: 1.27 GB
Virtual memory: 1.25 GB
Ended at May 28, 2023, 4:19:00 PM.
----- Dependent Variables 1 in Oxygen Pulse/Solution 958 (sol958) ------------->

<---- Stationary Solver 1 in Oxygen Pulse/Solution 958 (sol958) ----------------
Started at May 28, 2023, 4:19:00 PM.
Linear solver
Number of degrees of freedom solved for: 22990 (plus 935 internal DOFs).
Symmetric matrices found.
Scales for dependent variables:
Concentration (comp2.Oxi): 0.23
Orthonormal null-space function used.
Iter      SolEst     Damping    Stepsize #Res #Jac #Sol   LinErr   LinRes
   1     0.00095   1.0000000     0.00095    1    1    1    4e-13  8.9e-14
Solution time: 1 s.
Physical memory: 1.27 GB
Virtual memory: 1.25 GB
Ended at May 28, 2023, 4:19:01 PM.
----- Stationary Solver 1 in Oxygen Pulse/Solution 958 (sol958) --------------->

<---- Compile Equations: Oxygen Probe Pulse in Oxygen Pulse/Solution 958
      (sol958) ----------------------------------------------------------------
Started at May 28, 2023, 4:19:01 PM.
Geometry shape function: Linear Lagrange
Time: 0 s.
Physical memory: 1.24 GB
Virtual memory: 1.22 GB
Ended at May 28, 2023, 4:19:02 PM.
----- Compile Equations: Oxygen Probe Pulse in Oxygen Pulse/Solution 958
      (sol958) --------------------------------------------------------------->

<---- Dependent Variables 2 in Oxygen Pulse/Solution 958 (sol958) --------------
Started at May 28, 2023, 4:19:02 PM.
Initial values of variables solved for: Solution 958 (sol958), Solution Store 1 (sol
985).
Values of variables not solved for: Solution 958 (sol958), Solution Store 1 (sol985)
.
Solution time: 0 s.
Physical memory: 1.24 GB
```

```
Virtual memory: 1.22 GB
Ended at May 28, 2023, 4:19:02 PM.
----- Dependent Variables 2 in Oxygen Pulse/Solution 958 (sol958) ------------->

<---- Time-Dependent Solver 1 in Oxygen Pulse/Solution 958 (sol958) ------------
Started at May 28, 2023, 4:19:02 PM.
Time-dependent solver (BDF)
Number of degrees of freedom solved for: 22990 (plus 46056 internal DOFs).
Symmetric matrices found.
Scales for dependent variables:
Concentration (comp2.Oxi): 0.23
Step       Time    Stepsize      Res  Jac  Sol Order Tfail NLfail   LinErr   LinRes
   0          0         - out      5    3    5                 0    5e-16  8.7e-16
   1 3.5574e-08  3.5574e-08        7    4    7     1     0      0  2.9e-16  8.8e-16
   2 7.1149e-08  3.5574e-08        9    5    9     1     0      0  3.3e-16    1e-15
   3  1.423e-07  7.1149e-08       11    6   11     2     0      0  3.5e-16  1.1e-15
   4  2.846e-07   1.423e-07       13    7   13     2     0      0  3.8e-16  1.3e-15
   5 5.6919e-07   2.846e-07       15    8   15     2     0      0  5.1e-16  2.4e-15
   6 8.5379e-07   2.846e-07       17    9   17     2     0      0  4.9e-16  2.8e-15
   7 1.1384e-06   2.846e-07       19   10   19     2     0      0  5.8e-16  3.1e-15
   8  1.423e-06   2.846e-07       21   11   21     2     0      0  4.6e-16  3.1e-15
   9 1.9922e-06  5.6919e-07       23   12   23     2     0      0    6e-16    5e-15
  10 2.5614e-06  5.6919e-07       25   13   25     2     0      0  6.3e-16  4.6e-15
  11 3.1306e-06  5.6919e-07       27   14   27     2     0      0  6.8e-16  4.9e-15
  12 3.6997e-06  5.6919e-07       29   15   29     2     0      0  6.7e-16  4.7e-15
  13 4.8381e-06  1.1384e-06       31   16   31     2     0      0  8.7e-16    7e-15
  14 5.9765e-06  1.1384e-06       33   17   33     2     0      0  7.9e-16  6.1e-15
  15 7.1149e-06  1.1384e-06       35   18   35     2     0      0  8.3e-16  6.2e-15
  16 8.2533e-06  1.1384e-06       37   19   37     2     0      0  7.4e-16  5.7e-15
  17  1.053e-05  2.2768e-06       39   20   39     2     0      0  1.1e-15    9e-15
  18 1.2807e-05  2.2768e-06       41   21   41     2     0      0  1.2e-15  7.8e-15
  19 1.5084e-05  2.2768e-06       43   22   43     2     0      0  9.6e-16  7.9e-15
  20  1.736e-05  2.2768e-06       45   23   45     2     0      0  1.3e-15  7.3e-15
  21 2.1914e-05  4.5535e-06       47   24   47     2     0      0  1.4e-15  1.1e-14
  22 2.6467e-05  4.5535e-06       49   25   49     2     0      0  1.5e-15  9.6e-15
  23 3.1021e-05  4.5535e-06       51   26   51     2     0      0  1.4e-15  9.7e-15
  24 3.5574e-05  4.5535e-06       53   27   53     2     0      0  1.4e-15  9.2e-15
  25 4.4682e-05  9.1071e-06       55   28   55     2     0      0  1.7e-15  1.4e-14
  26 5.3789e-05  9.1071e-06       57   29   57     2     0      0  1.3e-15  1.2e-14
  27 6.2896e-05  9.1071e-06       59   30   59     2     0      0  1.5e-15  1.2e-14
  28 7.2003e-05  9.1071e-06       61   31   61     2     0      0    3e-15  1.1e-14
  29 9.0217e-05  1.8214e-05       63   32   63     2     0      0  1.9e-15  1.6e-14
  30 0.00010843  1.8214e-05       65   33   65     2     0      0  2.2e-15  1.4e-14
  31 0.00012665  1.8214e-05       67   34   67     2     0      0  1.9e-15  1.4e-14
  32 0.00014486  1.8214e-05       69   35   69     2     0      0  1.2e-15  1.3e-14
  33 0.00018129  3.6428e-05       71   36   71     2     0      0  2.3e-15  1.8e-14
  34 0.00021772  3.6428e-05       73   37   73     2     0      0  2.9e-15  1.5e-14
  35 0.00025414  3.6428e-05       75   38   75     2     0      0  1.9e-15  1.5e-14
  36 0.00029057  3.6428e-05       77   39   77     2     0      0  2.1e-15  1.4e-14
  37 0.00036343  7.2857e-05       79   40   79     2     0      0  2.1e-15  2.1e-14
  38 0.00043629  7.2857e-05       81   41   81     2     0      0  1.2e-15  1.8e-14
  39   0.000582  0.00014571       83   42   83     2     0      0  1.9e-15  2.7e-14
  40 0.00072771  0.00014571       85   43   85     2     0      0  1.9e-15  2.4e-14
  41 0.00087343  0.00014571       87   44   87     2     0      0  2.2e-15  2.3e-14
  42  0.0010191  0.00014571       89   45   89     2     0      0  3.9e-15    2e-14
  43  0.0013106  0.00029143       91   46   91     2     0      0    3e-15  2.5e-14
```

44	0.001602	0.00029143		93	47	93	2	0	0	3.1e-15	2e-14
45	0.0021848	0.00058285		95	48	95	2	0	0	9e-15	3.4e-14
46	0.0027677	0.00058285		97	49	97	2	0	0	5.2e-15	3.3e-14
47	0.0033505	0.00058285		99	50	99	2	0	0	3.6e-15	3.2e-14
48	0.0045163	0.0011657		101	51	101	2	0	0	2.2e-15	4e-14
-	0.005	-	out								
49	0.005682	0.0011657		103	52	103	2	0	0	4.1e-15	2.8e-14
50	0.0080134	0.0023314		105	53	105	2	0	0	3.2e-15	4.6e-14
-	0.01	-	out								
51	0.010345	0.0023314		107	54	107	2	0	0	4e-15	4.4e-14
52	0.012676	0.0023314		109	55	109	2	0	0	8.3e-15	4.7e-14
-	0.015	-	out								
53	0.017339	0.0046628		111	56	111	2	0	0	4.2e-15	6.6e-14
-	0.02	-	out								
54	0.022002	0.0046628		113	57	113	2	0	0	2.1e-15	5e-14
-	0.025	-	out								
-	0.03	-	out								
55	0.031327	0.0093256		115	58	115	2	0	0	3.5e-15	8e-14
-	0.035	-	out								
-	0.04	-	out								
56	0.040653	0.0093256		117	59	117	2	0	0	8.6e-15	7e-14
-	0.045	-	out								
57	0.049979	0.0093256		119	60	119	2	0	0	7.4e-15	7.3e-14
-	0.05	-	out								
-	0.055	-	out								
-	0.06	-	out								
-	0.065	-	out								
58	0.06863	0.018651		121	61	121	2	0	0	5.3e-15	9.6e-14
-	0.07	-	out								
-	0.075	-	out								
-	0.08	-	out								
-	0.085	-	out								
59	0.087281	0.018651		123	62	123	1	0	0	3.2e-15	8.6e-14
-	0.09	-	out								
-	0.095	-	out								
-	0.1	-	out								
-	0.105	-	out								
-	0.11	-	out								
-	0.115	-	out								
-	0.12	-	out								
60	0.12458	0.037303		125	63	125	1	0	0	5.4e-15	6.5e-14
-	0.125	-	out								
-	0.13	-	out								
-	0.135	-	out								
-	0.14	-	out								
-	0.145	-	out								
-	0.15	-	out								
-	0.155	-	out								
-	0.16	-	out								
-	0.165	-	out								
-	0.17	-	out								
-	0.175	-	out								
-	0.18	-	out								
-	0.185	-	out								
-	0.19	-	out								
-	0.195	-	out								

61	0.19919	0.074605	127	64	127	1	0	0	1.2e-14	8.6e-14
-	0.2	- out								
-	0.205	- out								
-	0.21	- out								
-	0.215	- out								
-	0.22	- out								
-	0.225	- out								
-	0.23	- out								
-	0.235	- out								
-	0.24	- out								
-	0.245	- out								
-	0.25	- out								
-	0.25	- out								
-	0.27	- out								
62	0.27379	0.074605	129	65	129	1	0	0	4.3e-15	7.1e-14
-	0.29	- out								
-	0.31	- out								
-	0.33	- out								
-	0.35	- out								
-	0.37	- out								
63	0.37279	0.099	131	66	131	1	0	0	2.1e-14	6.7e-14
-	0.39	- out								
-	0.41	- out								
-	0.43	- out								
-	0.45	- out								
-	0.47	- out								
64	0.47179	0.099	133	67	133	1	0	0	2.2e-14	5.3e-14
-	0.49	- out								
-	0.51	- out								
-	0.53	- out								
-	0.55	- out								
-	0.57	- out								
65	0.57079	0.099	135	68	135	1	0	0	6e-15	4.4e-14
-	0.59	- out								
-	0.61	- out								
-	0.63	- out								
-	0.65	- out								
66	0.66979	0.099	136	69	136	1	0	0	8.5e-15	3.6e-14
-	0.67	- out								
-	0.69	- out								
-	0.71	- out								
-	0.73	- out								
-	0.75	- out								
67	0.76879	0.099	137	70	137	1	0	0	6.9e-15	3.2e-14
-	0.77	- out								
-	0.79	- out								
-	0.81	- out								
-	0.83	- out								
-	0.85	- out								
68	0.86779	0.099	138	71	138	2	0	0	4.1e-15	2.6e-14
-	0.87	- out								
-	0.89	- out								
-	0.91	- out								
-	0.93	- out								
-	0.95	- out								
69	0.96679	0.099	139	72	139	2	0	0	1.2e-14	1.7e-14

```
   -        0.97           - out
   -        0.99           - out
  70      1.0658       0.099      140   73  140     2     0      0  9.8e-15  1.6e-14
Time-stepping completed.
Solution time: 16 s.
Physical memory: 1.3 GB
Virtual memory: 1.29 GB
Ended at May 28, 2023, 4:19:17 PM.
----- Time-Dependent Solver 1 in Oxygen Pulse/Solution 958 (sol958) ----------->
```

k0Sub=1.51E-6, d=4E-6 (su55)

GENERAL

Description	Value
Solution	k0Sub=1.51E-6, d=4E-6

LOG

```
<---- Compile Equations: Stationary in Oxygen Pulse/Solution 958 (sol958) ------
Started at May 28, 2023, 4:19:20 PM.
Parameter k0Sub = 1.51E-6 (m/s).
Parameter d = 4.0E-6 (m).
Geometry shape function: Linear Lagrange
Number of vertex elements: 10
Minimum element quality: 0.4317
Time: 1 s.
Physical memory: 1.28 GB
Virtual memory: 1.27 GB
Ended at May 28, 2023, 4:19:21 PM.
----- Compile Equations: Stationary in Oxygen Pulse/Solution 958 (sol958) ----->

<---- Dependent Variables 1 in Oxygen Pulse/Solution 958 (sol958) --------------
Started at May 28, 2023, 4:19:21 PM.
Solution time: 0 s.
Physical memory: 1.24 GB
Virtual memory: 1.22 GB
Ended at May 28, 2023, 4:19:21 PM.
----- Dependent Variables 1 in Oxygen Pulse/Solution 958 (sol958) ------------->

<---- Stationary Solver 1 in Oxygen Pulse/Solution 958 (sol958) ----------------
Started at May 28, 2023, 4:19:21 PM.
Linear solver
Number of degrees of freedom solved for: 18182 (plus 936 internal DOFs).
Symmetric matrices found.
Scales for dependent variables:
Concentration (comp2.Oxi): 0.23
Orthonormal null-space function used.
Iter      SolEst     Damping    Stepsize #Res #Jac #Sol   LinErr   LinRes
   1     0.00092   1.0000000     0.00092    1    1    1  3.3e-13  7.6e-14
Solution time: 1 s.
Physical memory: 1.27 GB
Virtual memory: 1.25 GB
Ended at May 28, 2023, 4:19:22 PM.
----- Stationary Solver 1 in Oxygen Pulse/Solution 958 (sol958) --------------->

<---- Compile Equations: Oxygen Probe Pulse in Oxygen Pulse/Solution 958
      (sol958) ----------------------------------------------------------------
Started at May 28, 2023, 4:19:22 PM.
Geometry shape function: Linear Lagrange
Time: 0 s.
Physical memory: 1.28 GB
Virtual memory: 1.25 GB
Ended at May 28, 2023, 4:19:22 PM.
----- Compile Equations: Oxygen Probe Pulse in Oxygen Pulse/Solution 958
      (sol958) --------------------------------------------------------------->

<---- Dependent Variables 2 in Oxygen Pulse/Solution 958 (sol958) --------------
Started at May 28, 2023, 4:19:22 PM.
Initial values of variables solved for: Solution 958 (sol958), Solution Store 1 (sol
985).
Values of variables not solved for: Solution 958 (sol958), Solution Store 1 (sol985)
.
Solution time: 0 s.
Physical memory: 1.28 GB
```

Virtual memory: 1.25 GB
Ended at May 28, 2023, 4:19:22 PM.
----- Dependent Variables 2 in Oxygen Pulse/Solution 958 (sol958) ------------->

<---- Time-Dependent Solver 1 in Oxygen Pulse/Solution 958 (sol958) ------------
Started at May 28, 2023, 4:19:22 PM.
Time-dependent solver (BDF)
Number of degrees of freedom solved for: 18182 (plus 36438 internal DOFs).
Symmetric matrices found.
Scales for dependent variables:
Concentration (comp2.Oxi): 0.23

Step	Time	Stepsize		Res	Jac	Sol	Order	Tfail	NLfail	LinErr	LinRes
0	0	-	out	5	3	5			0	8.2e-16	1.5e-15
1	2.6154e-08	2.6154e-08		7	4	7	1	0	0	3.6e-16	1.1e-15
2	5.2309e-08	2.6154e-08		9	5	9	1	0	0	3.8e-16	1.2e-15
3	1.0462e-07	5.2309e-08		11	6	11	2	0	0	4e-16	1.3e-15
4	2.0924e-07	1.0462e-07		13	7	13	2	0	0	4.7e-16	1.8e-15
5	4.1847e-07	2.0924e-07		15	8	15	2	0	0	7e-16	3.5e-15
6	6.2771e-07	2.0924e-07		17	9	17	2	0	0	6.2e-16	4e-15
7	8.3694e-07	2.0924e-07		19	10	19	2	0	0	6.6e-16	4e-15
8	1.0462e-06	2.0924e-07		21	11	21	2	0	0	6.8e-16	4e-15
9	1.4647e-06	4.1847e-07		23	12	23	2	0	0	9.2e-16	6.2e-15
10	1.8831e-06	4.1847e-07		25	13	25	2	0	0	8.5e-16	6e-15
11	2.3016e-06	4.1847e-07		27	14	27	2	0	0	1e-15	6e-15
12	2.7201e-06	4.1847e-07		29	15	29	2	0	0	7.1e-16	5.5e-15
13	3.557e-06	8.3694e-07		31	16	31	2	0	0	1e-15	8.7e-15
14	4.394e-06	8.3694e-07		33	17	33	2	0	0	9.6e-16	7.4e-15
15	5.2309e-06	8.3694e-07		35	18	35	2	0	0	9.7e-16	7.2e-15
16	6.0678e-06	8.3694e-07		37	19	37	2	0	0	1.1e-15	6.7e-15
17	7.7417e-06	1.6739e-06		39	20	39	2	0	0	9e-16	9.5e-15
18	9.4156e-06	1.6739e-06		41	21	41	2	0	0	9.7e-16	8.2e-15
19	1.109e-05	1.6739e-06		43	22	43	2	0	0	1.5e-15	8e-15
20	1.2763e-05	1.6739e-06		45	23	45	2	0	0	1.3e-15	7.3e-15
21	1.4437e-05	1.6739e-06		47	24	47	2	0	0	1.1e-15	6.7e-15
22	1.7785e-05	3.3478e-06		49	25	49	2	0	0	1.7e-15	1e-14
23	2.1133e-05	3.3478e-06		51	26	51	2	0	0	1.7e-15	8.7e-15
24	2.4481e-05	3.3478e-06		53	27	53	2	0	0	1.5e-15	8.4e-15
25	2.7828e-05	3.3478e-06		55	28	55	2	0	0	1.1e-15	7.9e-15
26	3.4524e-05	6.6955e-06		57	29	57	2	0	0	2.1e-15	1.2e-14
27	4.1219e-05	6.6955e-06		59	30	59	2	0	0	1.9e-15	9.8e-15
28	4.7915e-05	6.6955e-06		61	31	61	2	0	0	3.1e-15	9.8e-15
29	5.4611e-05	6.6955e-06		63	32	63	2	0	0	2.1e-15	9.3e-15
30	6.1306e-05	6.6955e-06		65	33	65	2	0	0	2.4e-15	7.6e-15
31	7.4697e-05	1.3391e-05		67	34	67	2	0	0	2.4e-15	1.2e-14
32	8.8088e-05	1.3391e-05		69	35	69	2	0	0	1.9e-15	1e-14
33	0.00010148	1.3391e-05		71	36	71	2	0	0	1.7e-15	1e-14
34	0.00011487	1.3391e-05		73	37	73	2	0	0	1.9e-15	9.5e-15
35	0.00014165	2.6782e-05		75	38	75	2	0	0	1.6e-15	1.4e-14
36	0.00016843	2.6782e-05		77	39	77	2	0	0	1.9e-15	1.2e-14
37	0.00019522	2.6782e-05		79	40	79	2	0	0	1.9e-15	1.2e-14
38	0.000222	2.6782e-05		81	41	81	2	0	0	1.9e-15	1.1e-14
39	0.00027556	5.3564e-05		83	42	83	2	0	0	1.8e-15	1.5e-14
40	0.00032913	5.3564e-05		85	43	85	2	0	0	3.9e-15	1.3e-14
41	0.00038269	5.3564e-05		87	44	87	2	0	0	1.8e-15	1.3e-14
42	0.00048982	0.00010713		89	45	89	2	0	0	2.1e-15	1.9e-14
43	0.00059695	0.00010713		91	46	91	2	0	0	3.1e-15	1.5e-14

44	0.00070408	0.00010713		93	47	93	2	0	0	2.2e-15	1.6e-14
45	0.00081121	0.00010713		95	48	95	2	0	0	1.9e-15	1.4e-14
46	0.0010255	0.00021426		97	49	97	2	0	0	1.7e-15	2e-14
47	0.0012397	0.00021426		99	50	99	2	0	0	3.2e-15	1.6e-14
48	0.0016682	0.00042852		101	51	101	2	0	0	2.3e-15	2.6e-14
49	0.0020968	0.00042852		103	52	103	2	0	0	3.3e-15	2.3e-14
50	0.0025253	0.00042852		105	53	105	2	0	0	8.3e-15	2.4e-14
51	0.0029538	0.00042852		107	54	107	2	0	0	2e-15	1.9e-14
52	0.0038108	0.00085703		109	55	109	2	0	0	5.6e-15	2.7e-14
53	0.0046678	0.00085703		111	56	111	2	0	0	2.7e-15	2.2e-14
-	0.005	-	out								
54	0.0063819	0.0017141		113	57	113	2	0	0	3.4e-15	3.4e-14
55	0.008096	0.0017141		115	58	115	2	0	0	3.9e-15	2.8e-14
-	0.01	-	out								
56	0.011524	0.0034281		117	59	117	2	0	0	2.3e-15	4.4e-14
57	0.014952	0.0034281		119	60	119	1	0	0	1.5e-15	5.5e-14
-	0.015	-	out								
58	0.01838	0.0034281		121	61	121	1	0	0	2.9e-15	4.3e-14
-	0.02	-	out								
-	0.025	-	out								
59	0.025237	0.0068562		123	62	123	1	0	0	5.2e-15	7e-14
-	0.03	-	out								
60	0.032093	0.0068562		125	63	125	1	0	0	3.5e-15	6.9e-14
-	0.035	-	out								
-	0.04	-	out								
-	0.045	-	out								
61	0.045805	0.013712		127	64	127	1	0	0	5.3e-15	9.9e-14
-	0.05	-	out								
-	0.055	-	out								
62	0.059518	0.013712		129	65	129	1	0	0	6.2e-15	8.5e-14
-	0.06	-	out								
-	0.065	-	out								
-	0.07	-	out								
-	0.075	-	out								
-	0.08	-	out								
-	0.085	-	out								
63	0.086943	0.027425		131	66	131	1	0	0	7.2e-15	1.1e-13
-	0.09	-	out								
-	0.095	-	out								
-	0.1	-	out								
-	0.105	-	out								
-	0.11	-	out								
64	0.11437	0.027425		133	67	133	1	0	0	8.2e-15	8.5e-14
-	0.115	-	out								
-	0.12	-	out								
-	0.125	-	out								
-	0.13	-	out								
-	0.135	-	out								
-	0.14	-	out								
-	0.145	-	out								
-	0.15	-	out								
-	0.155	-	out								
-	0.16	-	out								
-	0.165	-	out								
65	0.16922	0.05485		135	68	135	1	0	0	2e-14	8.6e-14
-	0.17	-	out								

-	0.175	- out								
-	0.18	- out								
-	0.185	- out								
-	0.19	- out								
-	0.195	- out								
-	0.2	- out								
-	0.205	- out								
-	0.21	- out								
-	0.215	- out								
-	0.22	- out								
66	0.22407	0.05485	137	69	137	1	0	0	1.3e-14	6.5e-14
-	0.225	- out								
-	0.23	- out								
-	0.235	- out								
-	0.24	- out								
-	0.245	- out								
-	0.25	- out								
-	0.25	- out								
-	0.27	- out								
-	0.29	- out								
-	0.31	- out								
67	0.32307	0.099	139	70	139	1	0	0	2e-14	6.4e-14
-	0.33	- out								
-	0.35	- out								
-	0.37	- out								
-	0.39	- out								
-	0.41	- out								
68	0.42207	0.099	141	71	141	1	0	0	6e-15	4.9e-14
-	0.43	- out								
-	0.45	- out								
-	0.47	- out								
-	0.49	- out								
-	0.51	- out								
69	0.52107	0.099	143	72	143	1	0	0	8.9e-15	3.6e-14
-	0.53	- out								
-	0.55	- out								
-	0.57	- out								
-	0.59	- out								
-	0.61	- out								
70	0.62007	0.099	145	73	145	1	0	0	5.1e-15	2.9e-14
-	0.63	- out								
-	0.65	- out								
-	0.67	- out								
-	0.69	- out								
-	0.71	- out								
71	0.71907	0.099	146	74	146	1	0	0	2e-15	2.4e-14
-	0.73	- out								
-	0.75	- out								
-	0.77	- out								
-	0.79	- out								
-	0.81	- out								
72	0.81807	0.099	147	75	147	1	0	0	7.4e-15	2.1e-14
-	0.83	- out								
-	0.85	- out								
-	0.87	- out								
-	0.89	- out								

```
  -        0.91           - out
 73     0.91707       0.099      148   76  148     2     0      0  1.7e-14  1.7e-14
  -        0.93           - out
  -        0.95           - out
  -        0.97           - out
  -        0.99           - out
 74      1.0161       0.099      149   77  149     2     0      0  1.8e-14  1.1e-14
Time-stepping completed.
Solution time: 14 s.
Physical memory: 1.32 GB
Virtual memory: 1.29 GB
Ended at May 28, 2023, 4:19:36 PM.
----- Time-Dependent Solver 1 in Oxygen Pulse/Solution 958 (sol958) ----------->
```

k0Sub=1.51E-6, d=5E-6 (su56)

GENERAL

Description	Value
Solution	k0Sub=1.51E-6, d=5E-6

LOG

```
<---- Compile Equations: Stationary in Oxygen Pulse/Solution 958 (sol958) ------
Started at May 28, 2023, 4:19:38 PM.
Parameter k0Sub = 1.51E-6 (m/s).
Parameter d = 4.9999999999999996E-6 (m).
Geometry shape function: Linear Lagrange
Number of vertex elements: 10
Minimum element quality: 0.4532
Time: 1 s.
Physical memory: 1.28 GB
Virtual memory: 1.26 GB
Ended at May 28, 2023, 4:19:39 PM.
----- Compile Equations: Stationary in Oxygen Pulse/Solution 958 (sol958) ----->

<---- Dependent Variables 1 in Oxygen Pulse/Solution 958 (sol958) --------------
Started at May 28, 2023, 4:19:39 PM.
Solution time: 0 s.
Physical memory: 1.28 GB
Virtual memory: 1.27 GB
Ended at May 28, 2023, 4:19:39 PM.
----- Dependent Variables 1 in Oxygen Pulse/Solution 958 (sol958) ------------->

<---- Stationary Solver 1 in Oxygen Pulse/Solution 958 (sol958) ----------------
Started at May 28, 2023, 4:19:39 PM.
Linear solver
Number of degrees of freedom solved for: 18304 (plus 936 internal DOFs).
Symmetric matrices found.
Scales for dependent variables:
Concentration (comp2.Oxi): 0.23
Orthonormal null-space function used.
Iter      SolEst     Damping    Stepsize #Res #Jac #Sol   LinErr   LinRes
   1     0.00079   1.0000000     0.00079    1    1    1  3.3e-13  6.8e-14
Solution time: 1 s.
Physical memory: 1.29 GB
Virtual memory: 1.27 GB
Ended at May 28, 2023, 4:19:40 PM.
----- Stationary Solver 1 in Oxygen Pulse/Solution 958 (sol958) --------------->

<---- Compile Equations: Oxygen Probe Pulse in Oxygen Pulse/Solution 958
      (sol958) ----------------------------------------------------------------
Started at May 28, 2023, 4:19:40 PM.
Geometry shape function: Linear Lagrange
Time: 0 s.
Physical memory: 1.3 GB
Virtual memory: 1.28 GB
Ended at May 28, 2023, 4:19:40 PM.
----- Compile Equations: Oxygen Probe Pulse in Oxygen Pulse/Solution 958
      (sol958) --------------------------------------------------------------->

<---- Dependent Variables 2 in Oxygen Pulse/Solution 958 (sol958) --------------
Started at May 28, 2023, 4:19:40 PM.
Initial values of variables solved for: Solution 958 (sol958), Solution Store 1 (sol
985).
Values of variables not solved for: Solution 958 (sol958), Solution Store 1 (sol985)
.
Solution time: 0 s.
Physical memory: 1.3 GB
```

```
Virtual memory: 1.28 GB
Ended at May 28, 2023, 4:19:40 PM.
----- Dependent Variables 2 in Oxygen Pulse/Solution 958 (sol958) ------------->

<---- Time-Dependent Solver 1 in Oxygen Pulse/Solution 958 (sol958) ------------
Started at May 28, 2023, 4:19:40 PM.
Time-dependent solver (BDF)
Number of degrees of freedom solved for: 18304 (plus 36680 internal DOFs).
Symmetric matrices found.
Scales for dependent variables:
Concentration (comp2.Oxi): 0.23
Step        Time    Stepsize      Res  Jac  Sol Order Tfail NLfail   LinErr   LinRes
   0           0          - out     5    3    5                 0  1.2e-15  1.4e-15
   1  2.6333e-08  2.6333e-08        7    4    7     1     0     0  3.4e-16  1.1e-15
   2  5.2666e-08  2.6333e-08        9    5    9     1     0     0  3.7e-16  1.2e-15
   3  1.0533e-07  5.2666e-08       11    6   11     2     0     0  4.2e-16  1.4e-15
   4  2.1067e-07  1.0533e-07       13    7   13     2     0     0  4.5e-16  1.7e-15
   5  4.2133e-07  2.1067e-07       15    8   15     2     0     0  5.4e-16  3.4e-15
   6    6.32e-07  2.1067e-07       17    9   17     2     0     0  6.4e-16  3.9e-15
   7  8.4266e-07  2.1067e-07       19   10   19     2     0     0  6.5e-16  3.8e-15
   8  1.0533e-06  2.1067e-07       21   11   21     2     0     0  6.5e-16    4e-15
   9  1.4747e-06  4.2133e-07       23   12   23     2     0     0    1e-15  6.3e-15
  10   1.896e-06  4.2133e-07       25   13   25     2     0     0  8.4e-16  6.1e-15
  11  2.3173e-06  4.2133e-07       27   14   27     2     0     0  9.6e-16  6.1e-15
  12  2.7387e-06  4.2133e-07       29   15   29     2     0     0  8.2e-16  5.6e-15
  13  3.5813e-06  8.4266e-07       31   16   31     2     0     0  1.3e-15  8.7e-15
  14   4.424e-06  8.4266e-07       33   17   33     2     0     0  1.2e-15  7.8e-15
  15  5.2666e-06  8.4266e-07       35   18   35     2     0     0  1.1e-15  7.4e-15
  16  6.1093e-06  8.4266e-07       37   19   37     2     0     0  9.8e-16  6.8e-15
  17  7.7946e-06  1.6853e-06       39   20   39     2     0     0  1.3e-15    1e-14
  18  9.4799e-06  1.6853e-06       41   21   41     2     0     0  1.5e-15  8.7e-15
  19  1.1165e-05  1.6853e-06       43   22   43     2     0     0  1.2e-15  8.3e-15
  20  1.2851e-05  1.6853e-06       45   23   45     2     0     0  1.3e-15  7.5e-15
  21  1.4536e-05  1.6853e-06       47   24   47     2     0     0    1e-15  6.5e-15
  22  1.7907e-05  3.3706e-06       49   25   49     2     0     0  1.9e-15    1e-14
  23  2.1277e-05  3.3706e-06       51   26   51     2     0     0  1.4e-15  8.7e-15
  24  2.4648e-05  3.3706e-06       53   27   53     2     0     0  1.8e-15  8.9e-15
  25  2.8019e-05  3.3706e-06       55   28   55     2     0     0  1.6e-15  7.8e-15
  26   3.476e-05  6.7413e-06       57   29   57     2     0     0  1.9e-15  1.2e-14
  27  4.1501e-05  6.7413e-06       59   30   59     2     0     0  2.2e-15    1e-14
  28  4.8242e-05  6.7413e-06       61   31   61     2     0     0  1.8e-15    1e-14
  29  5.4984e-05  6.7413e-06       63   32   63     2     0     0  1.8e-15  9.4e-15
  30  6.1725e-05  6.7413e-06       65   33   65     2     0     0  1.2e-15  8.5e-15
  31  7.5208e-05  1.3483e-05       67   34   67     2     0     0  1.4e-15  1.2e-14
  32   8.869e-05  1.3483e-05       69   35   69     2     0     0    2e-15  1.1e-14
  33  0.00010217  1.3483e-05       71   36   71     2     0     0  1.9e-15  1.1e-14
  34  0.00011566  1.3483e-05       73   37   73     2     0     0  2.1e-15  9.7e-15
  35  0.00014262  2.6965e-05       75   38   75     2     0     0  1.9e-15  1.5e-14
  36  0.00016959  2.6965e-05       77   39   77     2     0     0  1.5e-15  1.3e-14
  37  0.00019655  2.6965e-05       79   40   79     2     0     0  1.9e-15  1.2e-14
  38  0.00022352  2.6965e-05       81   41   81     2     0     0  2.2e-15  1.2e-14
  39  0.00027745   5.393e-05       83   42   83     2     0     0  2.5e-15  1.6e-14
  40  0.00033138   5.393e-05       85   43   85     2     0     0  1.5e-15  1.4e-14
  41  0.00038531   5.393e-05       87   44   87     2     0     0  2.3e-15  1.4e-14
  42  0.00049317  0.00010786       89   45   89     2     0     0    2e-15    2e-14
  43  0.00060103  0.00010786       91   46   91     2     0     0  1.9e-15  1.6e-14
```

44	0.00070889	0.00010786		93	47	93	2	0	0	2e-15	1.7e-14
45	0.00081675	0.00010786		95	48	95	2	0	0	2e-15	1.5e-14
46	0.0010325	0.00021572		97	49	97	2	0	0	1.7e-15	2.1e-14
47	0.0012482	0.00021572		99	50	99	2	0	0	1.9e-15	1.6e-14
48	0.0016796	0.00043144		101	51	101	2	0	0	3.3e-15	2.5e-14
49	0.0021111	0.00043144		103	52	103	2	0	0	3.1e-15	2.3e-14
50	0.0025425	0.00043144		105	53	105	2	0	0	4.4e-15	2.3e-14
51	0.002974	0.00043144		107	54	107	2	0	0	2.3e-15	2e-14
52	0.0038368	0.00086289		109	55	109	2	0	0	3.6e-15	2.6e-14
53	0.0046997	0.00086289		111	56	111	2	0	0	1.5e-15	2.1e-14
-	0.005	-	out								
54	0.0064255	0.0017258		113	57	113	2	0	0	1.6e-15	3.5e-14
55	0.0081513	0.0017258		115	58	115	2	0	0	1.9e-15	3.1e-14
-	0.01	-	out								
56	0.011603	0.0034515		117	59	117	2	0	0	3.8e-15	4.3e-14
-	0.015	-	out								
57	0.015054	0.0034515		119	60	119	1	0	0	1.6e-15	5.3e-14
58	0.018506	0.0034515		121	61	121	1	0	0	5e-15	4e-14
-	0.02	-	out								
-	0.025	-	out								
59	0.025409	0.0069031		123	62	123	1	0	0	4.5e-15	6.8e-14
-	0.03	-	out								
60	0.032312	0.0069031		125	63	125	1	0	0	8.2e-15	6.2e-14
-	0.035	-	out								
-	0.04	-	out								
-	0.045	-	out								
61	0.046118	0.013806		127	64	127	1	0	0	7.6e-15	9.8e-14
-	0.05	-	out								
-	0.055	-	out								
62	0.059924	0.013806		129	65	129	1	0	0	3.3e-15	8.5e-14
-	0.06	-	out								
-	0.065	-	out								
-	0.07	-	out								
-	0.075	-	out								
-	0.08	-	out								
-	0.085	-	out								
63	0.087537	0.027612		131	66	131	1	0	0	8.1e-15	1.1e-13
-	0.09	-	out								
-	0.095	-	out								
-	0.1	-	out								
-	0.105	-	out								
-	0.11	-	out								
-	0.115	-	out								
64	0.11515	0.027612		133	67	133	1	0	0	3.3e-15	8e-14
-	0.12	-	out								
-	0.125	-	out								
-	0.13	-	out								
-	0.135	-	out								
-	0.14	-	out								
-	0.145	-	out								
-	0.15	-	out								
-	0.155	-	out								
-	0.16	-	out								
-	0.165	-	out								
-	0.17	-	out								
65	0.17037	0.055225		135	68	135	1	0	0	7.9e-15	8.9e-14

-	0.175	- out								
-	0.18	- out								
-	0.185	- out								
-	0.19	- out								
-	0.195	- out								
-	0.2	- out								
-	0.205	- out								
-	0.21	- out								
-	0.215	- out								
-	0.22	- out								
-	0.225	- out								
66	0.2256	0.055225	137	69	137	1	0	0	9.6e-15	6.7e-14
-	0.23	- out								
-	0.235	- out								
-	0.24	- out								
-	0.245	- out								
-	0.25	- out								
-	0.25	- out								
-	0.27	- out								
-	0.29	- out								
-	0.31	- out								
67	0.3246	0.099	139	70	139	1	0	0	8.8e-15	7e-14
-	0.33	- out								
-	0.35	- out								
-	0.37	- out								
-	0.39	- out								
-	0.41	- out								
68	0.4236	0.099	141	71	141	1	0	0	9.5e-15	5.1e-14
-	0.43	- out								
-	0.45	- out								
-	0.47	- out								
-	0.49	- out								
-	0.51	- out								
69	0.5226	0.099	143	72	143	1	0	0	4.8e-15	3.9e-14
-	0.53	- out								
-	0.55	- out								
-	0.57	- out								
-	0.59	- out								
-	0.61	- out								
70	0.6216	0.099	145	73	145	1	0	0	1.6e-14	3.1e-14
-	0.63	- out								
-	0.65	- out								
-	0.67	- out								
-	0.69	- out								
-	0.71	- out								
71	0.7206	0.099	146	74	146	1	0	0	5.7e-15	2.6e-14
-	0.73	- out								
-	0.75	- out								
-	0.77	- out								
-	0.79	- out								
-	0.81	- out								
72	0.8196	0.099	147	75	147	1	0	0	4.5e-15	2.2e-14
-	0.83	- out								
-	0.85	- out								
-	0.87	- out								
-	0.89	- out								

```
  -        0.91           - out
 73      0.9186       0.099      148   76  148     2     0      0  1.8e-15  1.8e-14
  -        0.93           - out
  -        0.95           - out
  -        0.97           - out
  -        0.99           - out
 74      1.0176       0.099      149   77  149     2     0      0  1.9e-14  1.2e-14
Time-stepping completed.
Solution time: 14 s.
Physical memory: 1.33 GB
Virtual memory: 1.31 GB
Ended at May 28, 2023, 4:19:54 PM.
----- Time-Dependent Solver 1 in Oxygen Pulse/Solution 958 (sol958) ----------->
```

k0Sub=1.51E-6, d=6E-6 (su57)

GENERAL

Description	Value
Solution	k0Sub=1.51E-6, d=6E-6

LOG

```
<---- Compile Equations: Stationary in Oxygen Pulse/Solution 958 (sol958) ------
Started at May 28, 2023, 4:19:57 PM.
Parameter k0Sub = 1.51E-6 (m/s).
Parameter d = 6.0E-6 (m).
Geometry shape function: Linear Lagrange
Number of vertex elements: 10
Minimum element quality: 0.432
Time: 0 s.
Physical memory: 1.25 GB
Virtual memory: 1.22 GB
Ended at May 28, 2023, 4:19:57 PM.
----- Compile Equations: Stationary in Oxygen Pulse/Solution 958 (sol958) ----->

<---- Dependent Variables 1 in Oxygen Pulse/Solution 958 (sol958) --------------
Started at May 28, 2023, 4:19:57 PM.
Solution time: 0 s.
Physical memory: 1.25 GB
Virtual memory: 1.22 GB
Ended at May 28, 2023, 4:19:57 PM.
----- Dependent Variables 1 in Oxygen Pulse/Solution 958 (sol958) ------------->

<---- Stationary Solver 1 in Oxygen Pulse/Solution 958 (sol958) ----------------
Started at May 28, 2023, 4:19:57 PM.
Linear solver
Number of degrees of freedom solved for: 18385 (plus 936 internal DOFs).
Symmetric matrices found.
Scales for dependent variables:
Concentration (comp2.Oxi): 0.23
Orthonormal null-space function used.
Iter      SolEst     Damping    Stepsize #Res #Jac #Sol   LinErr   LinRes
   1      0.0007   1.0000000      0.0007    1    1    1  2.8e-13  6.2e-14
Solution time: 1 s.
Physical memory: 1.27 GB
Virtual memory: 1.25 GB
Ended at May 28, 2023, 4:19:58 PM.
----- Stationary Solver 1 in Oxygen Pulse/Solution 958 (sol958) --------------->

<---- Compile Equations: Oxygen Probe Pulse in Oxygen Pulse/Solution 958
      (sol958) ----------------------------------------------------------------
Started at May 28, 2023, 4:19:58 PM.
Geometry shape function: Linear Lagrange
Time: 0 s.
Physical memory: 1.28 GB
Virtual memory: 1.26 GB
Ended at May 28, 2023, 4:19:58 PM.
----- Compile Equations: Oxygen Probe Pulse in Oxygen Pulse/Solution 958
      (sol958) --------------------------------------------------------------->

<---- Dependent Variables 2 in Oxygen Pulse/Solution 958 (sol958) --------------
Started at May 28, 2023, 4:19:58 PM.
Initial values of variables solved for: Solution 958 (sol958), Solution Store 1 (sol
985).
Values of variables not solved for: Solution 958 (sol958), Solution Store 1 (sol985)
.
Solution time: 0 s.
Physical memory: 1.28 GB
```

Virtual memory: 1.26 GB
Ended at May 28, 2023, 4:19:58 PM.
----- Dependent Variables 2 in Oxygen Pulse/Solution 958 (sol958) ------------->

<---- Time-Dependent Solver 1 in Oxygen Pulse/Solution 958 (sol958) ------------
Started at May 28, 2023, 4:19:58 PM.
Time-dependent solver (BDF)
Number of degrees of freedom solved for: 18385 (plus 36840 internal DOFs).
Symmetric matrices found.
Scales for dependent variables:
Concentration (comp2.Oxi): 0.23

Step	Time	Stepsize		Res	Jac	Sol	Order	Tfail	NLfail	LinErr	LinRes
0	0	-	out	5	3	5			0	9.4e-16	1.5e-15
1	2.644e-08	2.644e-08		7	4	7	1	0	0	3.5e-16	1.1e-15
2	5.2879e-08	2.644e-08		9	5	9	1	0	0	3.5e-16	1.2e-15
3	1.0576e-07	5.2879e-08		11	6	11	2	0	0	4e-16	1.3e-15
4	2.1152e-07	1.0576e-07		13	7	13	2	0	0	5.2e-16	1.9e-15
5	4.2303e-07	2.1152e-07		15	8	15	2	0	0	6.1e-16	3.4e-15
6	6.3455e-07	2.1152e-07		17	9	17	2	0	0	5.5e-16	3.8e-15
7	8.4607e-07	2.1152e-07		19	10	19	2	0	0	5.8e-16	3.8e-15
8	1.0576e-06	2.1152e-07		21	11	21	2	0	0	6.6e-16	3.9e-15
9	1.4806e-06	4.2303e-07		23	12	23	2	0	0	8.6e-16	6.2e-15
10	1.9036e-06	4.2303e-07		25	13	25	2	0	0	8.5e-16	5.9e-15
11	2.3267e-06	4.2303e-07		27	14	27	2	0	0	7.9e-16	5.8e-15
12	2.7497e-06	4.2303e-07		29	15	29	2	0	0	7.9e-16	5.6e-15
13	3.5958e-06	8.4607e-07		31	16	31	2	0	0	9e-16	8.7e-15
14	4.4418e-06	8.4607e-07		33	17	33	2	0	0	1.3e-15	7.6e-15
15	5.2879e-06	8.4607e-07		35	18	35	2	0	0	1e-15	7.3e-15
16	6.134e-06	8.4607e-07		37	19	37	2	0	0	8.3e-16	6.5e-15
17	7.8261e-06	1.6921e-06		39	20	39	2	0	0	1.7e-15	9.9e-15
18	9.5182e-06	1.6921e-06		41	21	41	2	0	0	1.4e-15	8.3e-15
19	1.121e-05	1.6921e-06		43	22	43	2	0	0	1.4e-15	7.9e-15
20	1.2902e-05	1.6921e-06		45	23	45	2	0	0	1.2e-15	7.4e-15
21	1.4595e-05	1.6921e-06		47	24	47	2	0	0	1.3e-15	6.6e-15
22	1.7979e-05	3.3843e-06		49	25	49	2	0	0	1.4e-15	9.7e-15
23	2.1363e-05	3.3843e-06		51	26	51	2	0	0	2e-15	8.9e-15
24	2.4747e-05	3.3843e-06		53	27	53	2	0	0	1.4e-15	8.7e-15
25	2.8132e-05	3.3843e-06		55	28	55	2	0	0	1.8e-15	7.8e-15
26	3.49e-05	6.7685e-06		57	29	57	2	0	0	1.4e-15	1.2e-14
27	4.1669e-05	6.7685e-06		59	30	59	2	0	0	1.4e-15	1.1e-14
28	4.8437e-05	6.7685e-06		61	31	61	2	0	0	1.3e-15	9.8e-15
29	5.5206e-05	6.7685e-06		63	32	63	2	0	0	2e-15	9.2e-15
30	6.1974e-05	6.7685e-06		65	33	65	2	0	0	1.9e-15	8.2e-15
31	7.5511e-05	1.3537e-05		67	34	67	2	0	0	1.3e-15	1.2e-14
32	8.9048e-05	1.3537e-05		69	35	69	2	0	0	1.4e-15	1.1e-14
33	0.00010259	1.3537e-05		71	36	71	2	0	0	1.5e-15	1.1e-14
34	0.00011612	1.3537e-05		73	37	73	2	0	0	1.4e-15	9.9e-15
35	0.0001432	2.7074e-05		75	38	75	2	0	0	1.6e-15	1.5e-14
36	0.00017027	2.7074e-05		77	39	77	2	0	0	2.3e-15	1.3e-14
37	0.00019734	2.7074e-05		79	40	79	2	0	0	2.5e-15	1.2e-14
38	0.00022442	2.7074e-05		81	41	81	2	0	0	1.5e-15	1.1e-14
39	0.00027857	5.4148e-05		83	42	83	2	0	0	2.6e-15	1.7e-14
40	0.00033272	5.4148e-05		85	43	85	2	0	0	2.1e-15	1.4e-14
41	0.00038686	5.4148e-05		87	44	87	2	0	0	3.2e-15	1.4e-14
42	0.00049516	0.0001083		89	45	89	2	0	0	5.5e-15	2e-14
43	0.00060346	0.0001083		91	46	91	2	0	0	1.8e-15	1.7e-14

44	0.00071175	0.0001083		93	47	93	2	0	0	2.8e-15	1.7e-14
45	0.00082005	0.0001083		95	48	95	2	0	0	5.7e-15	1.5e-14
46	0.0010366	0.00021659		97	49	97	2	0	0	3.3e-15	2e-14
47	0.0012532	0.00021659		99	50	99	2	0	0	2.9e-15	1.6e-14
48	0.0016864	0.00043319		101	51	101	2	0	0	6.8e-15	2.7e-14
49	0.0021196	0.00043319		103	52	103	2	0	0	3.1e-15	2.2e-14
50	0.0025528	0.00043319		105	53	105	2	0	0	3.5e-15	2.3e-14
51	0.002986	0.00043319		107	54	107	2	0	0	1.8e-15	2e-14
52	0.0038523	0.00086637		109	55	109	2	0	0	2.2e-15	2.6e-14
53	0.0047187	0.00086637		111	56	111	2	0	0	3.3e-15	2e-14
-	0.005	-	out								
54	0.0064515	0.0017327		113	57	113	2	0	0	2e-15	3.5e-14
55	0.0081842	0.0017327		115	58	115	2	0	0	2.2e-15	3.1e-14
-	0.01	-	out								
56	0.01165	0.0034655		117	59	117	2	0	0	4.7e-15	4.5e-14
-	0.015	-	out								
57	0.015115	0.0034655		119	60	119	1	0	0	3.3e-15	5e-14
58	0.018581	0.0034655		121	61	121	1	0	0	5.3e-15	3.8e-14
-	0.02	-	out								
-	0.025	-	out								
59	0.025512	0.006931		123	62	123	1	0	0	4.9e-15	6.4e-14
-	0.03	-	out								
60	0.032443	0.006931		125	63	125	1	0	0	4.5e-15	6.1e-14
-	0.035	-	out								
-	0.04	-	out								
-	0.045	-	out								
61	0.046305	0.013862		127	64	127	1	0	0	4.6e-15	9e-14
-	0.05	-	out								
-	0.055	-	out								
-	0.06	-	out								
62	0.060166	0.013862		129	65	129	1	0	0	3.3e-15	7.7e-14
-	0.065	-	out								
-	0.07	-	out								
-	0.075	-	out								
-	0.08	-	out								
-	0.085	-	out								
63	0.08789	0.027724		131	66	131	1	0	0	2.9e-15	9.9e-14
-	0.09	-	out								
-	0.095	-	out								
-	0.1	-	out								
-	0.105	-	out								
-	0.11	-	out								
-	0.115	-	out								
64	0.11561	0.027724		133	67	133	1	0	0	7.5e-15	8.3e-14
-	0.12	-	out								
-	0.125	-	out								
-	0.13	-	out								
-	0.135	-	out								
-	0.14	-	out								
-	0.145	-	out								
-	0.15	-	out								
-	0.155	-	out								
-	0.16	-	out								
-	0.165	-	out								
-	0.17	-	out								
65	0.17106	0.055448		135	68	135	1	0	0	8.3e-15	9.4e-14

-	0.175	- out								
-	0.18	- out								
-	0.185	- out								
-	0.19	- out								
-	0.195	- out								
-	0.2	- out								
-	0.205	- out								
-	0.21	- out								
-	0.215	- out								
-	0.22	- out								
-	0.225	- out								
66	0.22651	0.055448	137	69	137	1	0	0	1.1e-14	7e-14
-	0.23	- out								
-	0.235	- out								
-	0.24	- out								
-	0.245	- out								
-	0.25	- out								
-	0.25	- out								
-	0.27	- out								
-	0.29	- out								
-	0.31	- out								
67	0.32551	0.099	139	70	139	1	0	0	7.1e-15	7.1e-14
-	0.33	- out								
-	0.35	- out								
-	0.37	- out								
-	0.39	- out								
-	0.41	- out								
68	0.42451	0.099	141	71	141	1	0	0	1.6e-14	5.4e-14
-	0.43	- out								
-	0.45	- out								
-	0.47	- out								
-	0.49	- out								
-	0.51	- out								
69	0.52351	0.099	143	72	143	1	0	0	1.3e-14	3.9e-14
-	0.53	- out								
-	0.55	- out								
-	0.57	- out								
-	0.59	- out								
-	0.61	- out								
70	0.62251	0.099	145	73	145	1	0	0	1.2e-14	3.1e-14
-	0.63	- out								
-	0.65	- out								
-	0.67	- out								
-	0.69	- out								
-	0.71	- out								
71	0.72151	0.099	146	74	146	1	0	0	6.5e-15	2.6e-14
-	0.73	- out								
-	0.75	- out								
-	0.77	- out								
-	0.79	- out								
-	0.81	- out								
72	0.82051	0.099	147	75	147	1	0	0	1.4e-14	2.4e-14
-	0.83	- out								
-	0.85	- out								
-	0.87	- out								
-	0.89	- out								

```
   -        0.91           - out
  73      0.91951       0.099      148    76   148      2      0       0     9e-15  1.9e-14
   -        0.93           - out
   -        0.95           - out
   -        0.97           - out
   -        0.99           - out
  74       1.0185       0.099      149    77   149      2      0       0  4.6e-15  1.3e-14
Time-stepping completed.
Solution time: 13 s.
Physical memory: 1.32 GB
Virtual memory: 1.3 GB
Ended at May 28, 2023, 4:20:12 PM.
----- Time-Dependent Solver 1 in Oxygen Pulse/Solution 958 (sol958) ----------->
```

k0Sub=1.51E-6, d=8E-6 (su58)

GENERAL

Description	Value
Solution	k0Sub=1.51E-6, d=8E-6

LOG

```
<---- Compile Equations: Stationary in Oxygen Pulse/Solution 958 (sol958) ------
Started at May 28, 2023, 4:20:14 PM.
Parameter k0Sub = 1.51E-6 (m/s).
Parameter d = 8.0E-6 (m).
Geometry shape function: Linear Lagrange
Number of vertex elements: 10
Minimum element quality: 0.4468
Time: 0 s.
Physical memory: 1.3 GB
Virtual memory: 1.28 GB
Ended at May 28, 2023, 4:20:15 PM.
----- Compile Equations: Stationary in Oxygen Pulse/Solution 958 (sol958) ----->

<---- Dependent Variables 1 in Oxygen Pulse/Solution 958 (sol958) --------------
Started at May 28, 2023, 4:20:15 PM.
Solution time: 0 s.
Physical memory: 1.3 GB
Virtual memory: 1.28 GB
Ended at May 28, 2023, 4:20:15 PM.
----- Dependent Variables 1 in Oxygen Pulse/Solution 958 (sol958) ------------->

<---- Stationary Solver 1 in Oxygen Pulse/Solution 958 (sol958) ----------------
Started at May 28, 2023, 4:20:15 PM.
Linear solver
Number of degrees of freedom solved for: 23800 (plus 941 internal DOFs).
Symmetric matrices found.
Scales for dependent variables:
Concentration (comp2.Oxi): 0.23
Orthonormal null-space function used.
Iter      SolEst     Damping    Stepsize #Res #Jac #Sol   LinErr   LinRes
   1      0.0005   1.0000000      0.0005    1    1    1  3.1e-13  5.9e-14
Solution time: 1 s.
Physical memory: 1.31 GB
Virtual memory: 1.29 GB
Ended at May 28, 2023, 4:20:16 PM.
----- Stationary Solver 1 in Oxygen Pulse/Solution 958 (sol958) --------------->

<---- Compile Equations: Oxygen Probe Pulse in Oxygen Pulse/Solution 958
      (sol958) -----------------------------------------------------------------
Started at May 28, 2023, 4:20:16 PM.
Geometry shape function: Linear Lagrange
Time: 0 s.
Physical memory: 1.31 GB
Virtual memory: 1.29 GB
Ended at May 28, 2023, 4:20:16 PM.
----- Compile Equations: Oxygen Probe Pulse in Oxygen Pulse/Solution 958
      (sol958) ---------------------------------------------------------------->

<---- Dependent Variables 2 in Oxygen Pulse/Solution 958 (sol958) --------------
Started at May 28, 2023, 4:20:16 PM.
Initial values of variables solved for: Solution 958 (sol958), Solution Store 1 (sol
985).
Values of variables not solved for: Solution 958 (sol958), Solution Store 1 (sol985)
.
Solution time: 0 s.
Physical memory: 1.31 GB
```

Virtual memory: 1.29 GB
Ended at May 28, 2023, 4:20:16 PM.
----- Dependent Variables 2 in Oxygen Pulse/Solution 958 (sol958) ------------->

<---- Time-Dependent Solver 1 in Oxygen Pulse/Solution 958 (sol958) ------------
Started at May 28, 2023, 4:20:16 PM.
Time-dependent solver (BDF)
Number of degrees of freedom solved for: 23800 (plus 47670 internal DOFs).
Symmetric matrices found.
Scales for dependent variables:
Concentration (comp2.Oxi): 0.23

Step	Time	Stepsize		Res	Jac	Sol	Order	Tfail	NLfail	LinErr	LinRes
0	0	-	out	5	3	5			0	6.2e-16	9.1e-16
1	3.6263e-08	3.6263e-08		7	4	7	1	0	0	3.2e-16	9.3e-16
2	7.2526e-08	3.6263e-08		9	5	9	1	0	0	3.3e-16	9.9e-16
3	1.4505e-07	7.2526e-08		11	6	11	2	0	0	3.4e-16	1.1e-15
4	2.901e-07	1.4505e-07		13	7	13	2	0	0	4e-16	1.3e-15
5	5.8021e-07	2.901e-07		15	8	15	2	0	0	5.8e-16	2.5e-15
6	8.7031e-07	2.901e-07		17	9	17	2	0	0	4.7e-16	2.9e-15
7	1.1604e-06	2.901e-07		19	10	19	2	0	0	4.7e-16	3e-15
8	1.4505e-06	2.901e-07		21	11	21	2	0	0	5.7e-16	3.2e-15
9	2.0307e-06	5.8021e-07		23	12	23	2	0	0	8.1e-16	4.8e-15
10	2.6109e-06	5.8021e-07		25	13	25	2	0	0	6.8e-16	4.8e-15
11	3.1911e-06	5.8021e-07		27	14	27	2	0	0	7.9e-16	4.6e-15
12	3.7713e-06	5.8021e-07		29	15	29	2	0	0	6.1e-16	4.5e-15
13	4.9317e-06	1.1604e-06		31	16	31	2	0	0	8.7e-16	6.9e-15
14	6.0922e-06	1.1604e-06		33	17	33	2	0	0	9.8e-16	6.2e-15
15	7.2526e-06	1.1604e-06		35	18	35	2	0	0	7.4e-16	6.5e-15
16	8.413e-06	1.1604e-06		37	19	37	2	0	0	8e-16	5.8e-15
17	1.0734e-05	2.3208e-06		39	20	39	2	0	0	1.7e-15	8.8e-15
18	1.3055e-05	2.3208e-06		41	21	41	2	0	0	1.1e-15	7.5e-15
19	1.5375e-05	2.3208e-06		43	22	43	2	0	0	1.2e-15	7.8e-15
20	1.7696e-05	2.3208e-06		45	23	45	2	0	0	1e-15	7e-15
21	2.2338e-05	4.6416e-06		47	24	47	2	0	0	1e-15	1.1e-14
22	2.698e-05	4.6416e-06		49	25	49	2	0	0	1.6e-15	9.4e-15
23	3.1621e-05	4.6416e-06		51	26	51	2	0	0	1.5e-15	9.4e-15
24	3.6263e-05	4.6416e-06		53	27	53	2	0	0	1e-15	8.8e-15
25	4.5546e-05	9.2833e-06		55	28	55	2	0	0	1.4e-15	1.3e-14
26	5.4829e-05	9.2833e-06		57	29	57	2	0	0	1.4e-15	1.2e-14
27	6.4113e-05	9.2833e-06		59	30	59	2	0	0	1.1e-15	1.1e-14
28	7.3396e-05	9.2833e-06		61	31	61	2	0	0	1.6e-15	1.1e-14
29	9.1963e-05	1.8567e-05		63	32	63	2	0	0	2.2e-15	1.6e-14
30	0.00011053	1.8567e-05		65	33	65	2	0	0	4.2e-15	1.4e-14
31	0.0001291	1.8567e-05		67	34	67	2	0	0	1.9e-15	1.3e-14
32	0.00014766	1.8567e-05		69	35	69	2	0	0	1.5e-15	1.2e-14
33	0.0001848	3.7133e-05		71	36	71	2	0	0	4.3e-15	1.8e-14
34	0.00022193	3.7133e-05		73	37	73	2	0	0	1.4e-15	1.6e-14
35	0.00025906	3.7133e-05		75	38	75	2	0	0	1.3e-15	1.5e-14
36	0.00029619	3.7133e-05		77	39	77	2	0	0	1.4e-15	1.4e-14
37	0.00037046	7.4266e-05		79	40	79	2	0	0	2.6e-15	2e-14
38	0.00044473	7.4266e-05		81	41	81	2	0	0	2.3e-15	1.7e-14
39	0.00059326	0.00014853		83	42	83	2	0	0	1.6e-15	2.8e-14
40	0.00074179	0.00014853		85	43	85	2	0	0	3.2e-15	2.3e-14
41	0.00089032	0.00014853		87	44	87	2	0	0	4.8e-15	2.3e-14
42	0.0010389	0.00014853		89	45	89	2	0	0	1.3e-15	2.1e-14
43	0.0013359	0.00029707		91	46	91	2	0	0	2.8e-15	2.6e-14

44	0.001633	0.00029707	93	47	93	2	0	0	4.2e-15	1.9e-14
45	0.0022271	0.00059413	95	48	95	2	0	0	6.7e-15	3.3e-14
46	0.0028212	0.00059413	97	49	97	2	0	0	8.5e-15	2.9e-14
47	0.0034154	0.00059413	99	50	99	2	0	0	5.8e-15	2.8e-14
48	0.0046036	0.0011883	101	51	101	2	0	0	5.6e-15	3.7e-14
-	0.005	-	out							
49	0.0057919	0.0011883	103	52	103	2	0	0	2.7e-15	2.6e-14
50	0.0081684	0.0023765	105	53	105	2	0	0	4.5e-15	4.2e-14
-	0.01	-	out							
51	0.010545	0.0023765	107	54	107	1	0	0	6e-15	4.9e-14
52	0.012921	0.0023765	109	55	109	1	0	0	2.1e-15	3.4e-14
-	0.015	-	out							
53	0.017675	0.004753	111	56	111	1	0	0	9.9e-15	5.4e-14
-	0.02	-	out							
-	0.025	-	out							
54	0.027181	0.0095061	113	57	113	1	0	0	3.9e-15	8.5e-14
-	0.03	-	out							
-	0.035	-	out							
55	0.036687	0.0095061	115	58	115	1	0	0	8.1e-15	8.3e-14
-	0.04	-	out							
-	0.045	-	out							
-	0.05	-	out							
-	0.055	-	out							
56	0.055699	0.019012	117	59	117	1	0	0	6.9e-15	1.2e-13
-	0.06	-	out							
-	0.065	-	out							
-	0.07	-	out							
57	0.074711	0.019012	119	60	119	1	0	0	1e-14	1.1e-13
-	0.075	-	out							
-	0.08	-	out							
-	0.085	-	out							
-	0.09	-	out							
-	0.095	-	out							
-	0.1	-	out							
-	0.105	-	out							
-	0.11	-	out							
58	0.11274	0.038024	121	61	121	1	0	0	5.6e-15	1.4e-13
-	0.115	-	out							
-	0.12	-	out							
-	0.125	-	out							
-	0.13	-	out							
-	0.135	-	out							
-	0.14	-	out							
-	0.145	-	out							
-	0.15	-	out							
59	0.15076	0.038024	123	62	123	1	0	0	1.1e-14	1.1e-13
-	0.155	-	out							
-	0.16	-	out							
-	0.165	-	out							
-	0.17	-	out							
-	0.175	-	out							
-	0.18	-	out							
-	0.185	-	out							
-	0.19	-	out							
-	0.195	-	out							
-	0.2	-	out							

-	0.205	- out								
-	0.21	- out								
-	0.215	- out								
-	0.22	- out								
-	0.225	- out								
60	0.22681	0.076049	125	63	125	1	0	0	6e-15	1.2e-13
-	0.23	- out								
-	0.235	- out								
-	0.24	- out								
-	0.245	- out								
-	0.25	- out								
-	0.25	- out								
-	0.27	- out								
-	0.29	- out								
61	0.30286	0.076049	127	64	127	1	0	0	1.8e-14	9.4e-14
-	0.31	- out								
-	0.33	- out								
-	0.35	- out								
-	0.37	- out								
-	0.39	- out								
62	0.40186	0.099	129	65	129	1	0	0	1.3e-14	8.1e-14
-	0.41	- out								
-	0.43	- out								
-	0.45	- out								
-	0.47	- out								
-	0.49	- out								
63	0.50086	0.099	131	66	131	1	0	0	5.9e-15	6.6e-14
-	0.51	- out								
-	0.53	- out								
-	0.55	- out								
-	0.57	- out								
-	0.59	- out								
64	0.59986	0.099	133	67	133	1	0	0	5.3e-15	5e-14
-	0.61	- out								
-	0.63	- out								
-	0.65	- out								
-	0.67	- out								
-	0.69	- out								
65	0.69886	0.099	134	68	134	1	0	0	2.3e-15	4.1e-14
-	0.71	- out								
-	0.73	- out								
-	0.75	- out								
-	0.77	- out								
-	0.79	- out								
66	0.79786	0.099	135	69	135	1	0	0	7.5e-15	3.5e-14
-	0.81	- out								
-	0.83	- out								
-	0.85	- out								
-	0.87	- out								
-	0.89	- out								
67	0.89686	0.099	136	70	136	2	0	0	6.8e-15	2.9e-14
-	0.91	- out								
-	0.93	- out								
-	0.95	- out								
-	0.97	- out								
-	0.99	- out								

```
  68     0.99586       0.099      137   71  137     2     0      0  5.6e-15  1.9e-14
Time-stepping completed.
Solution time: 16 s.
Physical memory: 1.35 GB
Virtual memory: 1.33 GB
Ended at May 28, 2023, 4:20:32 PM.
----- Time-Dependent Solver 1 in Oxygen Pulse/Solution 958 (sol958) ----------->
```

k0Sub=1.51E-6, d=1.2E-5 (su59)

GENERAL

Description	Value
Solution	k0Sub=1.51E-6, d=1.2E-5

LOG

```
<---- Compile Equations: Stationary in Oxygen Pulse/Solution 958 (sol958) ------
Started at May 28, 2023, 4:20:36 PM.
Parameter k0Sub = 1.51E-6 (m/s).
Parameter d = 1.2E-5 (m).
Geometry shape function: Linear Lagrange
Number of vertex elements: 10
Minimum element quality: 0.4505
Time: 1 s.
Physical memory: 1.31 GB
Virtual memory: 1.29 GB
Ended at May 28, 2023, 4:20:36 PM.
----- Compile Equations: Stationary in Oxygen Pulse/Solution 958 (sol958) ----->

<---- Dependent Variables 1 in Oxygen Pulse/Solution 958 (sol958) --------------
Started at May 28, 2023, 4:20:36 PM.
Solution time: 0 s.
Physical memory: 1.31 GB
Virtual memory: 1.29 GB
Ended at May 28, 2023, 4:20:36 PM.
----- Dependent Variables 1 in Oxygen Pulse/Solution 958 (sol958) ------------->

<---- Stationary Solver 1 in Oxygen Pulse/Solution 958 (sol958) ----------------
Started at May 28, 2023, 4:20:36 PM.
Linear solver
Number of degrees of freedom solved for: 18769 (plus 948 internal DOFs).
Symmetric matrices found.
Scales for dependent variables:
Concentration (comp2.Oxi): 0.23
Orthonormal null-space function used.
Iter      SolEst     Damping    Stepsize #Res #Jac #Sol   LinErr   LinRes
   1     0.00043   1.0000000     0.00043    1    1    1  2.4e-13  5.1e-14
Solution time: 1 s.
Physical memory: 1.32 GB
Virtual memory: 1.3 GB
Ended at May 28, 2023, 4:20:37 PM.
----- Stationary Solver 1 in Oxygen Pulse/Solution 958 (sol958) --------------->

<---- Compile Equations: Oxygen Probe Pulse in Oxygen Pulse/Solution 958
      (sol958) -----------------------------------------------------------------
Started at May 28, 2023, 4:20:37 PM.
Geometry shape function: Linear Lagrange
Time: 0 s.
Physical memory: 1.32 GB
Virtual memory: 1.3 GB
Ended at May 28, 2023, 4:20:37 PM.
----- Compile Equations: Oxygen Probe Pulse in Oxygen Pulse/Solution 958
      (sol958) ---------------------------------------------------------------->

<---- Dependent Variables 2 in Oxygen Pulse/Solution 958 (sol958) --------------
Started at May 28, 2023, 4:20:37 PM.
Initial values of variables solved for: Solution 958 (sol958), Solution Store 1 (sol
985).
Values of variables not solved for: Solution 958 (sol958), Solution Store 1 (sol985)
.
Solution time: 0 s.
Physical memory: 1.32 GB
```

Virtual memory: 1.3 GB
Ended at May 28, 2023, 4:20:37 PM.
----- Dependent Variables 2 in Oxygen Pulse/Solution 958 (sol958) ------------->

<---- Time-Dependent Solver 1 in Oxygen Pulse/Solution 958 (sol958) ------------
Started at May 28, 2023, 4:20:37 PM.
Time-dependent solver (BDF)
Number of degrees of freedom solved for: 18769 (plus 37608 internal DOFs).
Symmetric matrices found.
Scales for dependent variables:
Concentration (comp2.Oxi): 0.23

Step	Time	Stepsize	Res	Jac	Sol	Order	Tfail	NLfail	LinErr	LinRes
0	0	- out	5	3	5			0	7.9e-16	1.4e-15
1	2.6595e-08	2.6595e-08	7	4	7	1	0	0	3.6e-16	1e-15
2	5.319e-08	2.6595e-08	9	5	9	1	0	0	3.3e-16	1.2e-15
3	1.0638e-07	5.319e-08	11	6	11	2	0	0	3.6e-16	1.2e-15
4	2.1276e-07	1.0638e-07	13	7	13	2	0	0	5e-16	1.8e-15
5	4.2552e-07	2.1276e-07	15	8	15	2	0	0	6.1e-16	3.5e-15
6	6.3828e-07	2.1276e-07	17	9	17	2	0	0	6.2e-16	3.8e-15
7	8.5105e-07	2.1276e-07	19	10	19	2	0	0	6.1e-16	4.1e-15
8	1.0638e-06	2.1276e-07	21	11	21	2	0	0	6.7e-16	3.8e-15
9	1.4893e-06	4.2552e-07	23	12	23	2	0	0	7.9e-16	6.5e-15
10	1.9149e-06	4.2552e-07	25	13	25	2	0	0	8.1e-16	6e-15
11	2.3404e-06	4.2552e-07	27	14	27	2	0	0	8.3e-16	5.9e-15
12	2.7659e-06	4.2552e-07	29	15	29	2	0	0	8.2e-16	5.6e-15
13	3.6169e-06	8.5105e-07	31	16	31	2	0	0	9.4e-16	8.4e-15
14	4.468e-06	8.5105e-07	33	17	33	2	0	0	1.4e-15	7.4e-15
15	5.319e-06	8.5105e-07	35	18	35	2	0	0	1.3e-15	7.1e-15
16	6.1701e-06	8.5105e-07	37	19	37	2	0	0	8.8e-16	6.4e-15
17	7.8722e-06	1.7021e-06	39	20	39	2	0	0	1.2e-15	9.4e-15
18	9.5743e-06	1.7021e-06	41	21	41	2	0	0	1.3e-15	8.2e-15
19	1.1276e-05	1.7021e-06	43	22	43	2	0	0	1.2e-15	8.3e-15
20	1.2978e-05	1.7021e-06	45	23	45	2	0	0	1.6e-15	7.5e-15
21	1.4681e-05	1.7021e-06	47	24	47	2	0	0	1.6e-15	6.7e-15
22	1.8085e-05	3.4042e-06	49	25	49	2	0	0	1.5e-15	9.8e-15
23	2.1489e-05	3.4042e-06	51	26	51	2	0	0	1.3e-15	8.3e-15
24	2.4893e-05	3.4042e-06	53	27	53	2	0	0	1.3e-15	8.2e-15
25	2.8297e-05	3.4042e-06	55	28	55	2	0	0	1.7e-15	7.5e-15
26	3.5106e-05	6.8084e-06	57	29	57	2	0	0	1.7e-15	1.1e-14
27	4.1914e-05	6.8084e-06	59	30	59	2	0	0	1.7e-15	9.5e-15
28	4.8722e-05	6.8084e-06	61	31	61	2	0	0	2e-15	9.9e-15
29	5.5531e-05	6.8084e-06	63	32	63	2	0	0	2.2e-15	8.4e-15
30	6.9148e-05	1.3617e-05	65	33	65	2	0	0	2e-15	1.3e-14
31	8.2764e-05	1.3617e-05	67	34	67	2	0	0	1.9e-15	1.1e-14
32	9.6381e-05	1.3617e-05	69	35	69	2	0	0	3.1e-15	1.1e-14
33	0.00011	1.3617e-05	71	36	71	2	0	0	1.6e-15	9.9e-15
34	0.00013723	2.7233e-05	73	37	73	2	0	0	2e-15	1.4e-14
35	0.00016446	2.7233e-05	75	38	75	2	0	0	2.8e-15	1.2e-14
36	0.0001917	2.7233e-05	77	39	77	2	0	0	1.3e-15	1.2e-14
37	0.00021893	2.7233e-05	79	40	79	2	0	0	1.8e-15	1.1e-14
38	0.0002734	5.4467e-05	81	41	81	2	0	0	2.5e-15	1.6e-14
39	0.00032787	5.4467e-05	83	42	83	2	0	0	3.9e-15	1.4e-14
40	0.00038233	5.4467e-05	85	43	85	2	0	0	3.2e-15	1.3e-14
41	0.00049127	0.00010893	87	44	87	2	0	0	5.2e-15	2e-14
42	0.0006002	0.00010893	89	45	89	2	0	0	2.1e-15	1.7e-14
43	0.00070913	0.00010893	91	46	91	2	0	0	1.6e-15	1.7e-14

44	0.00081807	0.00010893		93	47	93	2	0	0	2e-15	1.5e-14
45	0.0010359	0.00021787		95	48	95	2	0	0	6.3e-15	2e-14
46	0.0012538	0.00021787		97	49	97	2	0	0	3.1e-15	1.6e-14
47	0.0016895	0.00043574		99	50	99	2	0	0	2.2e-15	2.7e-14
48	0.0021253	0.00043574		101	51	101	2	0	0	1.9e-15	2.4e-14
49	0.002561	0.00043574		103	52	103	2	0	0	3.3e-15	2.2e-14
50	0.0029967	0.00043574		105	53	105	2	0	0	3.2e-15	2e-14
51	0.0038682	0.00087147		107	54	107	2	0	0	3.4e-15	2.4e-14
52	0.0047397	0.00087147		109	55	109	2	0	0	3.7e-15	1.8e-14
-	0.005	-	out								
53	0.0064826	0.0017429		111	56	111	2	0	0	3e-15	3.1e-14
54	0.0082256	0.0017429		113	57	113	2	0	0	1.8e-15	2.7e-14
-	0.01	-	out								
55	0.011711	0.0034859		115	58	115	2	0	0	4.6e-15	3.9e-14
-	0.015	-	out								
56	0.015197	0.0034859		117	59	117	1	0	0	3.7e-15	4.3e-14
57	0.018683	0.0034859		119	60	119	1	0	0	2.4e-15	2.4e-14
-	0.02	-	out								
-	0.025	-	out								
58	0.025655	0.0069718		121	61	121	1	0	0	4.6e-15	4e-14
-	0.03	-	out								
-	0.035	-	out								
59	0.039599	0.013944		123	62	123	1	0	0	3.4e-15	6.2e-14
-	0.04	-	out								
-	0.045	-	out								
-	0.05	-	out								
60	0.053542	0.013944		125	63	125	1	0	0	4.8e-15	6.1e-14
-	0.055	-	out								
-	0.06	-	out								
-	0.065	-	out								
-	0.07	-	out								
-	0.075	-	out								
-	0.08	-	out								
61	0.081429	0.027887		127	64	127	1	0	0	5.9e-15	8.7e-14
-	0.085	-	out								
-	0.09	-	out								
-	0.095	-	out								
-	0.1	-	out								
-	0.105	-	out								
62	0.10932	0.027887		129	65	129	1	0	0	6.1e-15	7.7e-14
-	0.11	-	out								
-	0.115	-	out								
-	0.12	-	out								
-	0.125	-	out								
-	0.13	-	out								
-	0.135	-	out								
-	0.14	-	out								
-	0.145	-	out								
-	0.15	-	out								
-	0.155	-	out								
-	0.16	-	out								
-	0.165	-	out								
63	0.16509	0.055774		131	66	131	1	0	0	7.9e-15	9.3e-14
-	0.17	-	out								
-	0.175	-	out								
-	0.18	-	out								

-	0.185	- out								
-	0.19	- out								
-	0.195	- out								
-	0.2	- out								
-	0.205	- out								
-	0.21	- out								
-	0.215	- out								
-	0.22	- out								
64	0.22086	0.055774	133	67	133	1	0	0	9.4e-15	7.8e-14
-	0.225	- out								
-	0.23	- out								
-	0.235	- out								
-	0.24	- out								
-	0.245	- out								
-	0.25	- out								
-	0.25	- out								
-	0.27	- out								
-	0.29	- out								
-	0.31	- out								
65	0.31986	0.099	135	68	135	1	0	0	7.6e-15	7.9e-14
-	0.33	- out								
-	0.35	- out								
-	0.37	- out								
-	0.39	- out								
-	0.41	- out								
66	0.41886	0.099	137	69	137	1	0	0	7.9e-15	6.2e-14
-	0.43	- out								
-	0.45	- out								
-	0.47	- out								
-	0.49	- out								
-	0.51	- out								
67	0.51786	0.099	139	70	139	1	0	0	1.2e-14	4.7e-14
-	0.53	- out								
-	0.55	- out								
-	0.57	- out								
-	0.59	- out								
-	0.61	- out								
68	0.61686	0.099	141	71	141	1	0	0	6.1e-15	3.8e-14
-	0.63	- out								
-	0.65	- out								
-	0.67	- out								
-	0.69	- out								
-	0.71	- out								
69	0.71586	0.099	142	72	142	1	0	0	1.1e-14	3.1e-14
-	0.73	- out								
-	0.75	- out								
-	0.77	- out								
-	0.79	- out								
-	0.81	- out								
70	0.81486	0.099	143	73	143	1	0	0	6.9e-15	2.6e-14
-	0.83	- out								
-	0.85	- out								
-	0.87	- out								
-	0.89	- out								
-	0.91	- out								
71	0.91386	0.099	144	74	144	2	0	0	8.5e-15	2.2e-14

```
    -        0.93           - out
    -        0.95           - out
    -        0.97           - out
    -        0.99           - out
   72      1.0129       0.099      145    75  145      2      0       0  3.9e-15  1.4e-14
Time-stepping completed.
Solution time: 14 s.
Physical memory: 1.35 GB
Virtual memory: 1.33 GB
Ended at May 28, 2023, 4:20:51 PM.
----- Time-Dependent Solver 1 in Oxygen Pulse/Solution 958 (sol958) ----------->
```

k0Sub=1.51E-6, d=1.5E-5 (su60)

GENERAL

Description	Value
Solution	k0Sub=1.51E-6, d=1.5E-5

LOG

```
<---- Compile Equations: Stationary in Oxygen Pulse/Solution 958 (sol958) ------
Started at May 28, 2023, 4:20:54 PM.
Parameter k0Sub = 1.51E-6 (m/s).
Parameter d = 1.4999999999999999E-5 (m).
Geometry shape function: Linear Lagrange
Number of vertex elements: 10
Minimum element quality: 0.4444
Time: 1 s.
Physical memory: 1.26 GB
Virtual memory: 1.24 GB
Ended at May 28, 2023, 4:20:55 PM.
----- Compile Equations: Stationary in Oxygen Pulse/Solution 958 (sol958) ----->

<---- Dependent Variables 1 in Oxygen Pulse/Solution 958 (sol958) --------------
Started at May 28, 2023, 4:20:55 PM.
Solution time: 0 s.
Physical memory: 1.26 GB
Virtual memory: 1.24 GB
Ended at May 28, 2023, 4:20:55 PM.
----- Dependent Variables 1 in Oxygen Pulse/Solution 958 (sol958) ------------->

<---- Stationary Solver 1 in Oxygen Pulse/Solution 958 (sol958) ----------------
Started at May 28, 2023, 4:20:55 PM.
Linear solver
Number of degrees of freedom solved for: 18918 (plus 952 internal DOFs).
Symmetric matrices found.
Scales for dependent variables:
Concentration (comp2.Oxi): 0.23
Orthonormal null-space function used.
Iter      SolEst     Damping    Stepsize #Res #Jac #Sol   LinErr   LinRes
   1     0.00037   1.0000000     0.00037    1    1    1  2.1e-13  4.5e-14
Solution time: 1 s.
Physical memory: 1.29 GB
Virtual memory: 1.27 GB
Ended at May 28, 2023, 4:20:55 PM.
----- Stationary Solver 1 in Oxygen Pulse/Solution 958 (sol958) --------------->

<---- Compile Equations: Oxygen Probe Pulse in Oxygen Pulse/Solution 958
      (sol958) ----------------------------------------------------------------
Started at May 28, 2023, 4:20:55 PM.
Geometry shape function: Linear Lagrange
Time: 1 s.
Physical memory: 1.29 GB
Virtual memory: 1.27 GB
Ended at May 28, 2023, 4:20:56 PM.
----- Compile Equations: Oxygen Probe Pulse in Oxygen Pulse/Solution 958
      (sol958) --------------------------------------------------------------->

<---- Dependent Variables 2 in Oxygen Pulse/Solution 958 (sol958) --------------
Started at May 28, 2023, 4:20:56 PM.
Initial values of variables solved for: Solution 958 (sol958), Solution Store 1 (sol
985).
Values of variables not solved for: Solution 958 (sol958), Solution Store 1 (sol985)
.
Solution time: 0 s.
Physical memory: 1.3 GB
```

```
Virtual memory: 1.27 GB
Ended at May 28, 2023, 4:20:56 PM.
----- Dependent Variables 2 in Oxygen Pulse/Solution 958 (sol958) ------------->

<---- Time-Dependent Solver 1 in Oxygen Pulse/Solution 958 (sol958) ------------
Started at May 28, 2023, 4:20:56 PM.
Time-dependent solver (BDF)
Number of degrees of freedom solved for: 18918 (plus 37906 internal DOFs).
Symmetric matrices found.
Scales for dependent variables:
Concentration (comp2.Oxi): 0.23
Step        Time    Stepsize      Res  Jac  Sol Order Tfail NLfail   LinErr   LinRes
   0           0          - out     5    3    5                 0  6.4e-16  1.3e-15
   1  2.6784e-08  2.6784e-08        7    4    7     1     0     0  3.6e-16    1e-15
   2  5.3567e-08  2.6784e-08        9    5    9     1     0     0  3.7e-16  1.2e-15
   3  1.0713e-07  5.3567e-08       11    6   11     2     0     0  3.5e-16  1.3e-15
   4  2.1427e-07  1.0713e-07       13    7   13     2     0     0    5e-16  1.8e-15
   5  4.2854e-07  2.1427e-07       15    8   15     2     0     0  6.1e-16  3.4e-15
   6  6.4281e-07  2.1427e-07       17    9   17     2     0     0  7.3e-16  3.8e-15
   7  8.5707e-07  2.1427e-07       19   10   19     2     0     0  5.9e-16  4.1e-15
   8  1.0713e-06  2.1427e-07       21   11   21     2     0     0  6.6e-16  4.1e-15
   9  1.4999e-06  4.2854e-07       23   12   23     2     0     0  1.1e-15  6.3e-15
  10  1.9284e-06  4.2854e-07       25   13   25     2     0     0  9.8e-16  6.1e-15
  11   2.357e-06  4.2854e-07       27   14   27     2     0     0    1e-15  5.9e-15
  12  2.7855e-06  4.2854e-07       29   15   29     2     0     0  8.2e-16  5.9e-15
  13  3.6426e-06  8.5707e-07       31   16   31     2     0     0  8.2e-16  8.5e-15
  14  4.4996e-06  8.5707e-07       33   17   33     2     0     0  1.1e-15  7.2e-15
  15  5.3567e-06  8.5707e-07       35   18   35     2     0     0  1.1e-15  7.3e-15
  16  6.2138e-06  8.5707e-07       37   19   37     2     0     0  9.4e-16  6.6e-15
  17  7.9279e-06  1.7141e-06       39   20   39     2     0     0  9.6e-16  9.6e-15
  18  9.6421e-06  1.7141e-06       41   21   41     2     0     0  1.6e-15  8.4e-15
  19  1.1356e-05  1.7141e-06       43   22   43     2     0     0  1.5e-15  8.2e-15
  20   1.307e-05  1.7141e-06       45   23   45     2     0     0  1.4e-15  7.3e-15
  21  1.4785e-05  1.7141e-06       47   24   47     2     0     0  1.8e-15  6.6e-15
  22  1.8213e-05  3.4283e-06       49   25   49     2     0     0  1.5e-15  9.8e-15
  23  2.1641e-05  3.4283e-06       51   26   51     2     0     0  1.8e-15  8.7e-15
  24  2.5069e-05  3.4283e-06       53   27   53     2     0     0  1.4e-15  8.3e-15
  25  2.8498e-05  3.4283e-06       55   28   55     2     0     0  1.6e-15  7.8e-15
  26  3.5354e-05  6.8566e-06       57   29   57     2     0     0  1.9e-15  1.1e-14
  27  4.2211e-05  6.8566e-06       59   30   59     2     0     0  1.8e-15  9.8e-15
  28  4.9068e-05  6.8566e-06       61   31   61     2     0     0  2.2e-15  9.7e-15
  29  5.5924e-05  6.8566e-06       63   32   63     2     0     0  1.5e-15  8.8e-15
  30  6.2781e-05  6.8566e-06       65   33   65     2     0     0  1.6e-15  8.1e-15
  31  7.6494e-05  1.3713e-05       67   34   67     2     0     0  1.7e-15  1.2e-14
  32  9.0207e-05  1.3713e-05       69   35   69     2     0     0  2.3e-15  1.1e-14
  33  0.00010392  1.3713e-05       71   36   71     2     0     0  1.8e-15    1e-14
  34  0.00011763  1.3713e-05       73   37   73     2     0     0  1.5e-15  9.7e-15
  35  0.00014506  2.7426e-05       75   38   75     2     0     0  1.7e-15  1.4e-14
  36  0.00017249  2.7426e-05       77   39   77     2     0     0  2.6e-15  1.3e-14
  37  0.00019991  2.7426e-05       79   40   79     2     0     0  2.5e-15  1.2e-14
  38  0.00022734  2.7426e-05       81   41   81     2     0     0  1.6e-15  1.1e-14
  39  0.00028219  5.4853e-05       83   42   83     2     0     0  1.5e-15  1.6e-14
  40  0.00033704  5.4853e-05       85   43   85     2     0     0  1.5e-15  1.4e-14
  41   0.0003919  5.4853e-05       87   44   87     2     0     0  1.2e-15  1.4e-14
  42   0.0005016  0.00010971       89   45   89     2     0     0  2.9e-15    2e-14
  43  0.00061131  0.00010971       91   46   91     2     0     0  2.4e-15  1.6e-14
```

44	0.00072101	0.00010971		93	47	93	2	0	0	5.2e-15	1.6e-14
45	0.00083072	0.00010971		95	48	95	2	0	0	1.8e-15	1.5e-14
46	0.0010501	0.00021941		97	49	97	2	0	0	1.8e-15	2e-14
47	0.0012695	0.00021941		99	50	99	2	0	0	2e-15	1.7e-14
48	0.0017084	0.00043882		101	51	101	2	0	0	2.8e-15	2.8e-14
49	0.0021472	0.00043882		103	52	103	2	0	0	6.7e-15	2.3e-14
50	0.002586	0.00043882		105	53	105	2	0	0	2.8e-15	2.3e-14
51	0.0030248	0.00043882		107	54	107	2	0	0	4.6e-15	1.9e-14
52	0.0039025	0.00087764		109	55	109	2	0	0	9.9e-15	2.4e-14
53	0.0047801	0.00087764		111	56	111	2	0	0	2.5e-15	1.8e-14
-	0.005	-	out								
54	0.0065354	0.0017553		113	57	113	2	0	0	2.4e-15	2.9e-14
55	0.0082907	0.0017553		115	58	115	2	0	0	1.6e-15	2.4e-14
-	0.01	-	out								
56	0.011801	0.0035106		117	59	117	2	0	0	3.8e-15	3.7e-14
-	0.015	-	out								
57	0.015312	0.0035106		119	60	119	1	0	0	2.5e-15	4.2e-14
58	0.018822	0.0035106		121	61	121	1	0	0	5.3e-15	2.5e-14
-	0.02	-	out								
-	0.025	-	out								
59	0.025844	0.0070212		123	62	123	1	0	0	2e-15	3.7e-14
-	0.03	-	out								
-	0.035	-	out								
60	0.039886	0.014042		125	63	125	1	0	0	8.5e-15	5.3e-14
-	0.04	-	out								
-	0.045	-	out								
-	0.05	-	out								
61	0.053928	0.014042		127	64	127	1	0	0	7.1e-15	4.9e-14
-	0.055	-	out								
-	0.06	-	out								
-	0.065	-	out								
-	0.07	-	out								
-	0.075	-	out								
-	0.08	-	out								
62	0.082013	0.028085		129	65	129	1	0	0	3.5e-15	7.3e-14
-	0.085	-	out								
-	0.09	-	out								
-	0.095	-	out								
-	0.1	-	out								
-	0.105	-	out								
-	0.11	-	out								
63	0.1101	0.028085		131	66	131	1	0	0	4.1e-15	6.4e-14
-	0.115	-	out								
-	0.12	-	out								
-	0.125	-	out								
-	0.13	-	out								
-	0.135	-	out								
-	0.14	-	out								
-	0.145	-	out								
-	0.15	-	out								
-	0.155	-	out								
-	0.16	-	out								
-	0.165	-	out								
64	0.16627	0.056169		133	67	133	1	0	0	5.9e-15	8.4e-14
-	0.17	-	out								
-	0.175	-	out								

-	0.18	- out								
-	0.185	- out								
-	0.19	- out								
-	0.195	- out								
-	0.2	- out								
-	0.205	- out								
-	0.21	- out								
-	0.215	- out								
-	0.22	- out								
65	0.22244	0.056169	135	68	135	1	0	0	1.2e-14	7e-14
-	0.225	- out								
-	0.23	- out								
-	0.235	- out								
-	0.24	- out								
-	0.245	- out								
-	0.25	- out								
-	0.25	- out								
-	0.27	- out								
-	0.29	- out								
-	0.31	- out								
66	0.32144	0.099	137	69	137	1	0	0	4.2e-15	7.6e-14
-	0.33	- out								
-	0.35	- out								
-	0.37	- out								
-	0.39	- out								
-	0.41	- out								
67	0.42044	0.099	139	70	139	1	0	0	1.5e-14	6.2e-14
-	0.43	- out								
-	0.45	- out								
-	0.47	- out								
-	0.49	- out								
-	0.51	- out								
68	0.51944	0.099	141	71	141	1	0	0	1.6e-14	4.9e-14
-	0.53	- out								
-	0.55	- out								
-	0.57	- out								
-	0.59	- out								
-	0.61	- out								
69	0.61844	0.099	143	72	143	1	0	0	1.4e-14	3.8e-14
-	0.63	- out								
-	0.65	- out								
-	0.67	- out								
-	0.69	- out								
-	0.71	- out								
70	0.71744	0.099	144	73	144	1	0	0	4.6e-15	3.3e-14
-	0.73	- out								
-	0.75	- out								
-	0.77	- out								
-	0.79	- out								
-	0.81	- out								
71	0.81644	0.099	145	74	145	1	0	0	9.8e-15	2.8e-14
-	0.83	- out								
-	0.85	- out								
-	0.87	- out								
-	0.89	- out								
-	0.91	- out								

```
  72      0.91544       0.099      146   75  146     2     0      0  2.4e-15  2.3e-14
   -         0.93           - out
   -         0.95           - out
   -         0.97           - out
   -         0.99           - out
  73       1.0144       0.099      147   76  147     2     0      0  8.1e-15  1.4e-14
Time-stepping completed.
Solution time: 14 s.
Physical memory: 1.33 GB
Virtual memory: 1.31 GB
Ended at May 28, 2023, 4:21:10 PM.
----- Time-Dependent Solver 1 in Oxygen Pulse/Solution 958 (sol958) ----------->
```

k0Sub=1.51E-5, d=5E-7 (su61)

GENERAL

Description	Value
Solution	k0Sub=1.51E-5, d=5E-7

LOG

```
<---- Compile Equations: Stationary in Oxygen Pulse/Solution 958 (sol958) ------
Started at May 28, 2023, 4:21:12 PM.
Parameter k0Sub = 1.51E-5 (m/s).
Parameter d = 5.0E-7 (m).
Geometry shape function: Linear Lagrange
Number of vertex elements: 10
Minimum element quality: 0.3539
Time: 1 s.
Physical memory: 1.3 GB
Virtual memory: 1.29 GB
Ended at May 28, 2023, 4:21:13 PM.
----- Compile Equations: Stationary in Oxygen Pulse/Solution 958 (sol958) ----->

<---- Dependent Variables 1 in Oxygen Pulse/Solution 958 (sol958) --------------
Started at May 28, 2023, 4:21:13 PM.
Solution time: 0 s.
Physical memory: 1.3 GB
Virtual memory: 1.29 GB
Ended at May 28, 2023, 4:21:13 PM.
----- Dependent Variables 1 in Oxygen Pulse/Solution 958 (sol958) ------------->

<---- Stationary Solver 1 in Oxygen Pulse/Solution 958 (sol958) ----------------
Started at May 28, 2023, 4:21:13 PM.
Linear solver
Number of degrees of freedom solved for: 17710 (plus 1007 internal DOFs).
Symmetric matrices found.
Scales for dependent variables:
Concentration (comp2.Oxi): 0.23
Orthonormal null-space function used.
Iter      SolEst     Damping    Stepsize #Res #Jac #Sol   LinErr   LinRes
   1       0.021   1.0000000       0.021    1    1    1  4.8e-13  1.5e-13
Solution time: 1 s.
Physical memory: 1.32 GB
Virtual memory: 1.3 GB
Ended at May 28, 2023, 4:21:14 PM.
----- Stationary Solver 1 in Oxygen Pulse/Solution 958 (sol958) --------------->

<---- Compile Equations: Oxygen Probe Pulse in Oxygen Pulse/Solution 958
      (sol958) ----------------------------------------------------------------
Started at May 28, 2023, 4:21:14 PM.
Geometry shape function: Linear Lagrange
Time: 0 s.
Physical memory: 1.32 GB
Virtual memory: 1.3 GB
Ended at May 28, 2023, 4:21:14 PM.
----- Compile Equations: Oxygen Probe Pulse in Oxygen Pulse/Solution 958
      (sol958) --------------------------------------------------------------->

<---- Dependent Variables 2 in Oxygen Pulse/Solution 958 (sol958) --------------
Started at May 28, 2023, 4:21:14 PM.
Initial values of variables solved for: Solution 958 (sol958), Solution Store 1 (sol
985).
Values of variables not solved for: Solution 958 (sol958), Solution Store 1 (sol985)
.
Solution time: 0 s.
Physical memory: 1.32 GB
```

```
Virtual memory: 1.3 GB
Ended at May 28, 2023, 4:21:14 PM.
----- Dependent Variables 2 in Oxygen Pulse/Solution 958 (sol958) ------------->

<---- Time-Dependent Solver 1 in Oxygen Pulse/Solution 958 (sol958) ------------
Started at May 28, 2023, 4:21:14 PM.
Time-dependent solver (BDF)
Number of degrees of freedom solved for: 17710 (plus 35580 internal DOFs).
Symmetric matrices found.
Scales for dependent variables:
Concentration (comp2.Oxi): 0.23
Step        Time    Stepsize      Res  Jac  Sol Order Tfail NLfail   LinErr   LinRes
   0           0          - out     5    3    5                  0  7.1e-16  1.5e-15
   1   2.596e-08   2.596e-08        7    4    7     1     0      0  3.4e-16  1.1e-15
   2  5.1921e-08   2.596e-08        9    5    9     1     0      0  3.6e-16  1.1e-15
   3  1.0384e-07  5.1921e-08       11    6   11     2     0      0  3.8e-16  1.3e-15
   4  2.0768e-07  1.0384e-07       13    7   13     2     0      0  4.6e-16  1.8e-15
   5  4.1537e-07  2.0768e-07       15    8   15     2     0      0  7.1e-16  3.4e-15
   6  6.2305e-07  2.0768e-07       17    9   17     2     0      0  6.9e-16  3.9e-15
   7  8.3073e-07  2.0768e-07       19   10   19     2     0      0  6.2e-16  3.9e-15
   8  1.0384e-06  2.0768e-07       21   11   21     2     0      0  7.3e-16  3.9e-15
   9  1.4538e-06  4.1537e-07       23   12   23     2     0      0    1e-15  5.8e-15
  10  1.8692e-06  4.1537e-07       25   13   25     2     0      0  8.4e-16  5.6e-15
  11  2.2845e-06  4.1537e-07       27   14   27     2     0      0  8.2e-16  5.6e-15
  12  2.6999e-06  4.1537e-07       29   15   29     2     0      0    7e-16  5.3e-15
  13  3.5306e-06  8.3073e-07       31   16   31     2     0      0  1.4e-15  7.6e-15
  14  4.3614e-06  8.3073e-07       33   17   33     2     0      0  1.6e-15  6.8e-15
  15  5.1921e-06  8.3073e-07       35   18   35     2     0      0  8.2e-16  6.4e-15
  16  6.0228e-06  8.3073e-07       37   19   37     2     0      0  7.6e-16    6e-15
  17  7.6843e-06  1.6615e-06       39   20   39     2     0      0  1.6e-15  9.1e-15
  18  9.3458e-06  1.6615e-06       41   21   41     2     0      0  1.7e-15  7.7e-15
  19  1.1007e-05  1.6615e-06       43   22   43     2     0      0  1.7e-15  7.8e-15
  20  1.2669e-05  1.6615e-06       45   23   45     2     0      0  1.3e-15  7.4e-15
  21   1.433e-05  1.6615e-06       47   24   47     2     0      0  1.3e-15  6.5e-15
  22  1.7653e-05  3.3229e-06       49   25   49     2     0      0  1.4e-15    1e-14
  23  2.0976e-05  3.3229e-06       51   26   51     2     0      0  1.2e-15  8.5e-15
  24  2.4299e-05  3.3229e-06       53   27   53     2     0      0  1.3e-15  8.4e-15
  25  2.7622e-05  3.3229e-06       55   28   55     2     0      0  1.7e-15  7.9e-15
  26  3.4268e-05  6.6459e-06       57   29   57     2     0      0  1.6e-15  1.1e-14
  27  4.0914e-05  6.6459e-06       59   30   59     2     0      0    2e-15  9.9e-15
  28   4.756e-05  6.6459e-06       61   31   61     2     0      0  1.8e-15  9.9e-15
  29  5.4205e-05  6.6459e-06       63   32   63     2     0      0  1.5e-15    9e-15
  30  6.7497e-05  1.3292e-05       65   33   65     2     0      0  2.9e-15  1.3e-14
  31  8.0789e-05  1.3292e-05       67   34   67     2     0      0  1.8e-15  1.1e-14
  32  9.4081e-05  1.3292e-05       69   35   69     2     0      0  2.3e-15  1.1e-14
  33  0.00010737  1.3292e-05       71   36   71     2     0      0  2.8e-15    1e-14
  34  0.00013396  2.6583e-05       73   37   73     2     0      0    3e-15  1.6e-14
  35  0.00016054  2.6583e-05       75   38   75     2     0      0    3e-15  1.4e-14
  36  0.00018712  2.6583e-05       77   39   77     2     0      0  1.4e-15  1.3e-14
  37  0.00021371  2.6583e-05       79   40   79     2     0      0  1.6e-15  1.1e-14
  38  0.00026687  5.3167e-05       81   41   81     2     0      0  1.8e-15  1.7e-14
  39  0.00032004  5.3167e-05       83   42   83     2     0      0  1.7e-15  1.4e-14
  40  0.00037321  5.3167e-05       85   43   85     2     0      0  2.9e-15  1.5e-14
  41  0.00042637  5.3167e-05       87   44   87     2     0      0  1.7e-15  1.4e-14
  42  0.00053271  0.00010633       89   45   89     2     0      0  2.4e-15  1.9e-14
  43  0.00063904  0.00010633       91   46   91     2     0      0  2.9e-15  1.4e-14
```

44	0.00085171	0.00021267		93	47	93	2	0	0	3.1e-15	2.4e-14
45	0.0010644	0.00021267		95	48	95	2	0	0	1.7e-15	2.2e-14
46	0.001277	0.00021267		97	49	97	2	0	0	3.7e-15	2.2e-14
47	0.0014897	0.00021267		99	50	99	2	0	0	3.5e-15	2.1e-14
48	0.001915	0.00042534		101	51	101	2	0	0	4.8e-15	3.1e-14
49	0.0023404	0.00042534		103	52	103	2	0	0	2.6e-15	2.6e-14
50	0.0027657	0.00042534		105	53	105	2	0	0	2.8e-15	2.8e-14
51	0.0036164	0.00085067		107	54	107	2	0	0	6.9e-15	4e-14
52	0.0044671	0.00085067		109	55	109	2	0	0	6.6e-15	3.5e-14
-	0.005	-	out								
53	0.0053177	0.00085067		111	56	111	2	0	0	2.9e-15	3.6e-14
54	0.0070191	0.0017013		113	57	113	2	0	0	4.5e-15	4.9e-14
55	0.0087204	0.0017013		115	58	115	2	0	0	3.1e-15	4.1e-14
-	0.01	-	out								
56	0.012123	0.0034027		117	59	117	2	0	0	2.4e-15	6.3e-14
-	0.015	-	out								
57	0.015526	0.0034027		119	60	119	2	0	0	4.7e-15	5.1e-14
58	0.018928	0.0034027		121	61	121	2	0	0	4.3e-15	5.1e-14
-	0.02	-	out								
59	0.022331	0.0034027		123	62	123	2	0	0	2.2e-15	4.3e-14
-	0.025	-	out								
60	0.029137	0.0068054		125	63	125	2	0	0	3.5e-15	6e-14
-	0.03	-	out								
-	0.035	-	out								
61	0.035942	0.0068054		127	64	127	2	0	0	1e-14	5.1e-14
-	0.04	-	out								
-	0.045	-	out								
62	0.049553	0.013611		129	65	129	2	0	0	3.9e-15	8.3e-14
-	0.05	-	out								
-	0.055	-	out								
-	0.06	-	out								
63	0.063163	0.013611		131	66	131	1	0	0	8.7e-15	6.9e-14
-	0.065	-	out								
-	0.07	-	out								
-	0.075	-	out								
64	0.076774	0.013611		133	67	133	1	0	0	5.4e-15	2.5e-14
-	0.08	-	out								
-	0.085	-	out								
-	0.09	-	out								
-	0.095	-	out								
-	0.1	-	out								
65	0.104	0.027221		135	68	135	1	0	0	6e-15	3.6e-14
-	0.105	-	out								
-	0.11	-	out								
-	0.115	-	out								
-	0.12	-	out								
-	0.125	-	out								
-	0.13	-	out								
-	0.135	-	out								
-	0.14	-	out								
-	0.145	-	out								
-	0.15	-	out								
-	0.155	-	out								
66	0.15844	0.054443		137	69	137	1	0	0	1.2e-14	5e-14
-	0.16	-	out								
-	0.165	-	out								

-	0.17	- out								
-	0.175	- out								
-	0.18	- out								
-	0.185	- out								
-	0.19	- out								
-	0.195	- out								
-	0.2	- out								
-	0.205	- out								
-	0.21	- out								
-	0.215	- out								
-	0.22	- out								
-	0.225	- out								
-	0.23	- out								
-	0.235	- out								
-	0.24	- out								
-	0.245	- out								
-	0.25	- out								
-	0.25	- out								
67	0.25744	0.099	139	70	139	1	0	0	2e-14	5.7e-14
-	0.27	- out								
-	0.29	- out								
-	0.31	- out								
-	0.33	- out								
-	0.35	- out								
68	0.35644	0.099	141	71	141	1	0	0	1.4e-14	4.3e-14
-	0.37	- out								
-	0.39	- out								
-	0.41	- out								
-	0.43	- out								
-	0.45	- out								
69	0.45544	0.099	143	72	143	1	0	0	1.1e-14	3.5e-14
-	0.47	- out								
-	0.49	- out								
-	0.51	- out								
-	0.53	- out								
-	0.55	- out								
70	0.55444	0.099	145	73	145	1	0	0	1.1e-14	2.6e-14
-	0.57	- out								
-	0.59	- out								
-	0.61	- out								
-	0.63	- out								
-	0.65	- out								
71	0.65344	0.099	146	74	146	1	0	0	9.9e-15	2.2e-14
-	0.67	- out								
-	0.69	- out								
-	0.71	- out								
-	0.73	- out								
-	0.75	- out								
72	0.75244	0.099	147	75	147	1	0	0	3.2e-15	2e-14
-	0.77	- out								
-	0.79	- out								
-	0.81	- out								
-	0.83	- out								
-	0.85	- out								
73	0.85144	0.099	148	76	148	2	0	0	2.2e-15	1.6e-14
-	0.87	- out								

```
     -        0.89           - out
     -        0.91           - out
     -        0.93           - out
     -        0.95           - out
    74     0.95044       0.099       149    77  149      2      0       0  1.2e-14  1.1e-14
     -        0.97           - out
     -        0.99           - out
    75      1.0494       0.099       150    78  150      2      0       0  4.5e-15    1e-14
Time-stepping completed.
Solution time: 13 s.
Physical memory: 1.34 GB
Virtual memory: 1.33 GB
Ended at May 28, 2023, 4:21:27 PM.
----- Time-Dependent Solver 1 in Oxygen Pulse/Solution 958 (sol958) ----------->
```

k0Sub=1.51E-5, d=1E-6 (su62)

GENERAL

Description	Value
Solution	k0Sub=1.51E-5, d=1E-6

LOG

```
<---- Compile Equations: Stationary in Oxygen Pulse/Solution 958 (sol958) ------
Started at May 28, 2023, 4:21:30 PM.
Parameter k0Sub = 1.51E-5 (m/s).
Parameter d = 1.0E-6 (m).
Geometry shape function: Linear Lagrange
Number of vertex elements: 10
Minimum element quality: 0.4361
Time: 0 s.
Physical memory: 1.31 GB
Virtual memory: 1.29 GB
Ended at May 28, 2023, 4:21:31 PM.
----- Compile Equations: Stationary in Oxygen Pulse/Solution 958 (sol958) ----->

<---- Dependent Variables 1 in Oxygen Pulse/Solution 958 (sol958) --------------
Started at May 28, 2023, 4:21:31 PM.
Solution time: 0 s.
Physical memory: 1.31 GB
Virtual memory: 1.29 GB
Ended at May 28, 2023, 4:21:31 PM.
----- Dependent Variables 1 in Oxygen Pulse/Solution 958 (sol958) ------------->

<---- Stationary Solver 1 in Oxygen Pulse/Solution 958 (sol958) ----------------
Started at May 28, 2023, 4:21:31 PM.
Linear solver
Number of degrees of freedom solved for: 17724 (plus 963 internal DOFs).
Symmetric matrices found.
Scales for dependent variables:
Concentration (comp2.Oxi): 0.23
Orthonormal null-space function used.
Iter      SolEst     Damping    Stepsize #Res #Jac #Sol   LinErr   LinRes
   1       0.018   1.0000000       0.018    1    1    1  4.7e-13  1.2e-13
Solution time: 1 s.
Physical memory: 1.32 GB
Virtual memory: 1.3 GB
Ended at May 28, 2023, 4:21:31 PM.
----- Stationary Solver 1 in Oxygen Pulse/Solution 958 (sol958) --------------->

<---- Compile Equations: Oxygen Probe Pulse in Oxygen Pulse/Solution 958
      (sol958) ----------------------------------------------------------------
Started at May 28, 2023, 4:21:31 PM.
Geometry shape function: Linear Lagrange
Time: 0 s.
Physical memory: 1.32 GB
Virtual memory: 1.3 GB
Ended at May 28, 2023, 4:21:32 PM.
----- Compile Equations: Oxygen Probe Pulse in Oxygen Pulse/Solution 958
      (sol958) --------------------------------------------------------------->

<---- Dependent Variables 2 in Oxygen Pulse/Solution 958 (sol958) --------------
Started at May 28, 2023, 4:21:32 PM.
Initial values of variables solved for: Solution 958 (sol958), Solution Store 1 (sol
985).
Values of variables not solved for: Solution 958 (sol958), Solution Store 1 (sol985)
.
Solution time: 0 s.
Physical memory: 1.32 GB
```

Virtual memory: 1.3 GB
Ended at May 28, 2023, 4:21:32 PM.
----- Dependent Variables 2 in Oxygen Pulse/Solution 958 (sol958) ------------->

<---- Time-Dependent Solver 1 in Oxygen Pulse/Solution 958 (sol958) ------------
Started at May 28, 2023, 4:21:32 PM.
Time-dependent solver (BDF)
Number of degrees of freedom solved for: 17724 (plus 35562 internal DOFs).
Symmetric matrices found.
Scales for dependent variables:
Concentration (comp2.Oxi): 0.23

Step	Time	Stepsize	Res	Jac	Sol	Order	Tfail	NLfail	LinErr	LinRes
0	0	- out	5	3	5			0	6.8e-16	1.4e-15
1	2.6204e-08	2.6204e-08	7	4	7	1	0	0	3.5e-16	1e-15
2	5.2408e-08	2.6204e-08	9	5	9	1	0	0	3.6e-16	1.2e-15
3	1.0482e-07	5.2408e-08	11	6	11	2	0	0	4e-16	1.3e-15
4	2.0963e-07	1.0482e-07	13	7	13	2	0	0	5e-16	1.8e-15
5	4.1927e-07	2.0963e-07	15	8	15	2	0	0	6.8e-16	3.4e-15
6	6.289e-07	2.0963e-07	17	9	17	2	0	0	7.2e-16	3.7e-15
7	8.3854e-07	2.0963e-07	19	10	19	2	0	0	6.7e-16	3.8e-15
8	1.0482e-06	2.0963e-07	21	11	21	2	0	0	6.1e-16	4.2e-15
9	1.4674e-06	4.1927e-07	23	12	23	2	0	0	9.5e-16	6.5e-15
10	1.8867e-06	4.1927e-07	25	13	25	2	0	0	1e-15	5.9e-15
11	2.306e-06	4.1927e-07	27	14	27	2	0	0	1e-15	5.7e-15
12	2.7252e-06	4.1927e-07	29	15	29	2	0	0	7.6e-16	5.3e-15
13	3.5638e-06	8.3854e-07	31	16	31	2	0	0	1.2e-15	8.3e-15
14	4.4023e-06	8.3854e-07	33	17	33	2	0	0	1.6e-15	7.1e-15
15	5.2408e-06	8.3854e-07	35	18	35	2	0	0	9.7e-16	7.1e-15
16	6.0794e-06	8.3854e-07	37	19	37	2	0	0	9.2e-16	6.4e-15
17	7.7564e-06	1.6771e-06	39	20	39	2	0	0	1.4e-15	9.6e-15
18	9.4335e-06	1.6771e-06	41	21	41	2	0	0	1.2e-15	8.1e-15
19	1.1111e-05	1.6771e-06	43	22	43	2	0	0	1.7e-15	7.9e-15
20	1.2788e-05	1.6771e-06	45	23	45	2	0	0	1.2e-15	7.3e-15
21	1.4465e-05	1.6771e-06	47	24	47	2	0	0	1.3e-15	6.1e-15
22	1.7819e-05	3.3541e-06	49	25	49	2	0	0	1.3e-15	9.2e-15
23	2.1173e-05	3.3541e-06	51	26	51	2	0	0	1.3e-15	7.8e-15
24	2.4527e-05	3.3541e-06	53	27	53	2	0	0	1.8e-15	7.7e-15
25	2.7881e-05	3.3541e-06	55	28	55	2	0	0	1.6e-15	7.3e-15
26	3.459e-05	6.7083e-06	57	29	57	2	0	0	1.5e-15	1.1e-14
27	4.1298e-05	6.7083e-06	59	30	59	2	0	0	1.8e-15	9.3e-15
28	4.8006e-05	6.7083e-06	61	31	61	2	0	0	1.3e-15	9.1e-15
29	5.4714e-05	6.7083e-06	63	32	63	2	0	0	1.5e-15	8.3e-15
30	6.1423e-05	6.7083e-06	65	33	65	2	0	0	1.8e-15	7.8e-15
31	7.4839e-05	1.3417e-05	67	34	67	2	0	0	2.8e-15	1.2e-14
32	8.8256e-05	1.3417e-05	69	35	69	2	0	0	1.7e-15	1e-14
33	0.00010167	1.3417e-05	71	36	71	2	0	0	1e-15	1e-14
34	0.00011509	1.3417e-05	73	37	73	2	0	0	1.6e-15	9.6e-15
35	0.00014192	2.6833e-05	75	38	75	2	0	0	1.4e-15	1.4e-14
36	0.00016876	2.6833e-05	77	39	77	2	0	0	4.3e-15	1.2e-14
37	0.00019559	2.6833e-05	79	40	79	2	0	0	2e-15	1.2e-14
38	0.00022242	2.6833e-05	81	41	81	2	0	0	3.6e-15	1.1e-14
39	0.00027609	5.3666e-05	83	42	83	2	0	0	1.6e-15	1.6e-14
40	0.00032975	5.3666e-05	85	43	85	2	0	0	2.1e-15	1.3e-14
41	0.00038342	5.3666e-05	87	44	87	2	0	0	1.9e-15	1.3e-14
42	0.00049075	0.00010733	89	45	89	2	0	0	7.5e-15	1.9e-14
43	0.00059809	0.00010733	91	46	91	2	0	0	2.2e-15	1.6e-14

44	0.00070542	0.00010733		93	47	93	2	0	0	2.2e-15	1.6e-14
45	0.00081275	0.00010733		95	48	95	2	0	0	1.7e-15	1.5e-14
46	0.0010274	0.00021466		97	49	97	2	0	0	2.1e-15	2e-14
47	0.0012421	0.00021466		99	50	99	2	0	0	2.8e-15	1.7e-14
48	0.0016714	0.00042933		101	51	101	2	0	0	5.4e-15	2.8e-14
49	0.0021007	0.00042933		103	52	103	2	0	0	4.7e-15	2.6e-14
50	0.0025301	0.00042933		105	53	105	2	0	0	4.2e-15	2.6e-14
51	0.0029594	0.00042933		107	54	107	2	0	0	3.7e-15	2.2e-14
52	0.0038181	0.00085866		109	55	109	2	0	0	4.1e-15	3.2e-14
53	0.0046767	0.00085866		111	56	111	2	0	0	3.6e-15	2.8e-14
-	0.005	-	out								
54	0.006394	0.0017173		113	57	113	2	0	0	2.6e-15	4.3e-14
55	0.0081114	0.0017173		115	58	115	2	0	0	7.2e-15	4.4e-14
56	0.0098287	0.0017173		117	59	117	2	0	0	5.7e-15	4.2e-14
-	0.01	-	out								
57	0.011546	0.0017173		119	60	119	2	0	0	3.8e-15	3.8e-14
58	0.014981	0.0034346		121	61	121	2	0	0	7.8e-15	4.8e-14
-	0.015	-	out								
59	0.018415	0.0034346		123	62	123	2	0	0	1.5e-15	3.5e-14
-	0.02	-	out								
-	0.025	-	out								
60	0.025285	0.0068693		125	63	125	2	0	0	6.2e-15	6.4e-14
-	0.03	-	out								
61	0.032154	0.0068693		127	64	127	2	0	0	3.3e-15	5.8e-14
-	0.035	-	out								
62	0.039023	0.0068693		129	65	129	2	0	0	5.5e-15	5.7e-14
-	0.04	-	out								
-	0.045	-	out								
-	0.05	-	out								
63	0.052762	0.013739		131	66	131	2	0	0	3.2e-15	8e-14
-	0.055	-	out								
-	0.06	-	out								
-	0.065	-	out								
64	0.0665	0.013739		133	67	133	1	0	0	2.1e-14	6.4e-14
-	0.07	-	out								
-	0.075	-	out								
-	0.08	-	out								
65	0.080239	0.013739		135	68	135	1	0	0	1.2e-14	2.7e-14
-	0.085	-	out								
-	0.09	-	out								
-	0.095	-	out								
-	0.1	-	out								
-	0.105	-	out								
66	0.10772	0.027477		137	69	137	1	0	0	7.5e-15	3.8e-14
-	0.11	-	out								
-	0.115	-	out								
-	0.12	-	out								
-	0.125	-	out								
-	0.13	-	out								
-	0.135	-	out								
-	0.14	-	out								
-	0.145	-	out								
-	0.15	-	out								
-	0.155	-	out								
-	0.16	-	out								
67	0.16267	0.054954		139	70	139	1	0	0	1.1e-14	5e-14

-	0.165	-	out							
-	0.17	-	out							
-	0.175	-	out							
-	0.18	-	out							
-	0.185	-	out							
-	0.19	-	out							
-	0.195	-	out							
-	0.2	-	out							
-	0.205	-	out							
-	0.21	-	out							
-	0.215	-	out							
-	0.22	-	out							
-	0.225	-	out							
-	0.23	-	out							
-	0.235	-	out							
-	0.24	-	out							
-	0.245	-	out							
-	0.25	-	out							
-	0.25	-	out							
68	0.26167	0.099	141	71	141	1	0	0	8.6e-15	5.5e-14
-	0.27	-	out							
-	0.29	-	out							
-	0.31	-	out							
-	0.33	-	out							
-	0.35	-	out							
69	0.36067	0.099	143	72	143	1	0	0	5.9e-15	4.4e-14
-	0.37	-	out							
-	0.39	-	out							
-	0.41	-	out							
-	0.43	-	out							
-	0.45	-	out							
70	0.45967	0.099	145	73	145	1	0	0	2.9e-15	3.4e-14
-	0.47	-	out							
-	0.49	-	out							
-	0.51	-	out							
-	0.53	-	out							
-	0.55	-	out							
71	0.55867	0.099	147	74	147	1	0	0	2.7e-15	2.7e-14
-	0.57	-	out							
-	0.59	-	out							
-	0.61	-	out							
-	0.63	-	out							
-	0.65	-	out							
72	0.65767	0.099	148	75	148	1	0	0	2.3e-14	2.2e-14
-	0.67	-	out							
-	0.69	-	out							
-	0.71	-	out							
-	0.73	-	out							
-	0.75	-	out							
73	0.75667	0.099	149	76	149	1	0	0	1.8e-14	2e-14
-	0.77	-	out							
-	0.79	-	out							
-	0.81	-	out							
-	0.83	-	out							
-	0.85	-	out							
74	0.85567	0.099	150	77	150	2	0	0	3.6e-15	1.6e-14

```
   -        0.87           - out
   -        0.89           - out
   -        0.91           - out
   -        0.93           - out
   -        0.95           - out
  75     0.95467       0.099      151    78  151      2      0       0  1.7e-14  1.1e-14
   -        0.97           - out
   -        0.99           - out
  76      1.0537       0.099      152    79  152      2      0       0  5.3e-15    1e-14
Time-stepping completed.
Solution time: 14 s.
Physical memory: 1.34 GB
Virtual memory: 1.32 GB
Ended at May 28, 2023, 4:21:45 PM.
----- Time-Dependent Solver 1 in Oxygen Pulse/Solution 958 (sol958) ------------>
```

k0Sub=1.51E-5, d=1.5E-6 (su63)

GENERAL

Description	Value
Solution	k0Sub=1.51E-5, d=1.5E-6

LOG

```
<---- Compile Equations: Stationary in Oxygen Pulse/Solution 958 (sol958) ------
Started at May 28, 2023, 4:21:48 PM.
Parameter k0Sub = 1.51E-5 (m/s).
Parameter d = 1.5E-6 (m).
Geometry shape function: Linear Lagrange
Number of vertex elements: 10
Minimum element quality: 0.395
Time: 0 s.
Physical memory: 1.31 GB
Virtual memory: 1.29 GB
Ended at May 28, 2023, 4:21:49 PM.
----- Compile Equations: Stationary in Oxygen Pulse/Solution 958 (sol958) ----->

<---- Dependent Variables 1 in Oxygen Pulse/Solution 958 (sol958) --------------
Started at May 28, 2023, 4:21:49 PM.
Solution time: 0 s.
Physical memory: 1.31 GB
Virtual memory: 1.29 GB
Ended at May 28, 2023, 4:21:49 PM.
----- Dependent Variables 1 in Oxygen Pulse/Solution 958 (sol958) ------------->

<---- Stationary Solver 1 in Oxygen Pulse/Solution 958 (sol958) ----------------
Started at May 28, 2023, 4:21:49 PM.
Linear solver
Number of degrees of freedom solved for: 17823 (plus 950 internal DOFs).
Symmetric matrices found.
Scales for dependent variables:
Concentration (comp2.Oxi): 0.23
Orthonormal null-space function used.
Iter      SolEst     Damping    Stepsize #Res #Jac #Sol   LinErr   LinRes
   1       0.015   1.0000000       0.015    1    1    1  3.7e-13  1.1e-13
Solution time: 1 s.
Physical memory: 1.32 GB
Virtual memory: 1.3 GB
Ended at May 28, 2023, 4:21:49 PM.
----- Stationary Solver 1 in Oxygen Pulse/Solution 958 (sol958) --------------->

<---- Compile Equations: Oxygen Probe Pulse in Oxygen Pulse/Solution 958
      (sol958) -----------------------------------------------------------------
Started at May 28, 2023, 4:21:49 PM.
Geometry shape function: Linear Lagrange
Time: 0 s.
Physical memory: 1.32 GB
Virtual memory: 1.3 GB
Ended at May 28, 2023, 4:21:50 PM.
----- Compile Equations: Oxygen Probe Pulse in Oxygen Pulse/Solution 958
      (sol958) ---------------------------------------------------------------->

<---- Dependent Variables 2 in Oxygen Pulse/Solution 958 (sol958) --------------
Started at May 28, 2023, 4:21:50 PM.
Initial values of variables solved for: Solution 958 (sol958), Solution Store 1 (sol
985).
Values of variables not solved for: Solution 958 (sol958), Solution Store 1 (sol985)
.
Solution time: 0 s.
Physical memory: 1.32 GB
```

Virtual memory: 1.3 GB
Ended at May 28, 2023, 4:21:50 PM.
----- Dependent Variables 2 in Oxygen Pulse/Solution 958 (sol958) ------------->

<---- Time-Dependent Solver 1 in Oxygen Pulse/Solution 958 (sol958) ------------
Started at May 28, 2023, 4:21:50 PM.
Time-dependent solver (BDF)
Number of degrees of freedom solved for: 17823 (plus 35744 internal DOFs).
Symmetric matrices found.
Scales for dependent variables:
Concentration (comp2.Oxi): 0.23

Step	Time	Stepsize		Res	Jac	Sol	Order	Tfail	NLfail	LinErr	LinRes
0	0	-	out	5	3	5			0	8.3e-16	1.4e-15
1	2.6006e-08	2.6006e-08		7	4	7	1	0	0	3.5e-16	1.1e-15
2	5.2013e-08	2.6006e-08		9	5	9	1	0	0	3.8e-16	1.2e-15
3	1.0403e-07	5.2013e-08		11	6	11	2	0	0	3.7e-16	1.3e-15
4	2.0805e-07	1.0403e-07		13	7	13	2	0	0	5.4e-16	1.7e-15
5	4.161e-07	2.0805e-07		15	8	15	2	0	0	7.1e-16	3.3e-15
6	6.2415e-07	2.0805e-07		17	9	17	2	0	0	6.4e-16	3.8e-15
7	8.322e-07	2.0805e-07		19	10	19	2	0	0	5.8e-16	3.9e-15
8	1.0403e-06	2.0805e-07		21	11	21	2	0	0	6.8e-16	4e-15
9	1.4564e-06	4.161e-07		23	12	23	2	0	0	8.3e-16	6.3e-15
10	1.8725e-06	4.161e-07		25	13	25	2	0	0	7.8e-16	6e-15
11	2.2886e-06	4.161e-07		27	14	27	2	0	0	9.3e-16	6e-15
12	2.7047e-06	4.161e-07		29	15	29	2	0	0	8.1e-16	5.7e-15
13	3.5369e-06	8.322e-07		31	16	31	2	0	0	1e-15	8.1e-15
14	4.3691e-06	8.322e-07		33	17	33	2	0	0	1.2e-15	7e-15
15	5.2013e-06	8.322e-07		35	18	35	2	0	0	1e-15	6.7e-15
16	6.0335e-06	8.322e-07		37	19	37	2	0	0	1.3e-15	6.4e-15
17	7.6979e-06	1.6644e-06		39	20	39	2	0	0	2.3e-15	9.6e-15
18	9.3623e-06	1.6644e-06		41	21	41	2	0	0	1.2e-15	7.9e-15
19	1.1027e-05	1.6644e-06		43	22	43	2	0	0	1.7e-15	7.9e-15
20	1.2691e-05	1.6644e-06		45	23	45	2	0	0	1.4e-15	7.2e-15
21	1.4355e-05	1.6644e-06		47	24	47	2	0	0	1.2e-15	6.5e-15
22	1.7684e-05	3.3288e-06		49	25	49	2	0	0	1.5e-15	9.2e-15
23	2.1013e-05	3.3288e-06		51	26	51	2	0	0	1.1e-15	8.3e-15
24	2.4342e-05	3.3288e-06		53	27	53	2	0	0	1.5e-15	7.9e-15
25	2.7671e-05	3.3288e-06		55	28	55	2	0	0	1.2e-15	7.2e-15
26	3.4328e-05	6.6576e-06		57	29	57	2	0	0	1.9e-15	1.1e-14
27	4.0986e-05	6.6576e-06		59	30	59	2	0	0	1.7e-15	9.2e-15
28	4.7644e-05	6.6576e-06		61	31	61	2	0	0	1.8e-15	9.3e-15
29	5.4301e-05	6.6576e-06		63	32	63	2	0	0	2.1e-15	8.5e-15
30	6.0959e-05	6.6576e-06		65	33	65	2	0	0	2.1e-15	7.6e-15
31	7.4274e-05	1.3315e-05		67	34	67	2	0	0	1.9e-15	1.1e-14
32	8.7589e-05	1.3315e-05		69	35	69	2	0	0	2.4e-15	9.5e-15
33	0.0001009	1.3315e-05		71	36	71	2	0	0	1.8e-15	9.4e-15
34	0.00011422	1.3315e-05		73	37	73	2	0	0	2e-15	9e-15
35	0.00014085	2.663e-05		75	38	75	2	0	0	2.1e-15	1.3e-14
36	0.00016748	2.663e-05		77	39	77	2	0	0	2.7e-15	1.2e-14
37	0.00019411	2.663e-05		79	40	79	2	0	0	1.6e-15	1.1e-14
38	0.00022074	2.663e-05		81	41	81	2	0	0	1.3e-15	1.1e-14
39	0.000274	5.3261e-05		83	42	83	2	0	0	1.7e-15	1.6e-14
40	0.00032726	5.3261e-05		85	43	85	2	0	0	1.8e-15	1.3e-14
41	0.00038052	5.3261e-05		87	44	87	2	0	0	2e-15	1.3e-14
42	0.00048705	0.00010652		89	45	89	2	0	0	3.5e-15	1.9e-14
43	0.00059357	0.00010652		91	46	91	2	0	0	1.8e-15	1.5e-14

44	0.00070009	0.00010652	93	47	93	2	0	0	4e-15	1.5e-14
45	0.00080661	0.00010652	95	48	95	2	0	0	3.1e-15	1.4e-14
46	0.0010197	0.00021304	97	49	97	2	0	0	3.4e-15	2e-14
47	0.0012327	0.00021304	99	50	99	2	0	0	2.1e-15	1.7e-14
48	0.0016588	0.00042609	101	51	101	2	0	0	2.4e-15	2.8e-14
49	0.0020849	0.00042609	103	52	103	2	0	0	2e-15	2.5e-14
50	0.002511	0.00042609	105	53	105	2	0	0	4.9e-15	2.3e-14
51	0.0033631	0.00085218	107	54	107	2	0	0	7.2e-15	3.2e-14
52	0.0042153	0.00085218	109	55	109	2	0	0	3.6e-15	2.5e-14
-	0.005	-	out							
53	0.0050675	0.00085218	111	56	111	2	0	0	4.6e-15	2.8e-14
54	0.0067718	0.0017044	113	57	113	2	0	0	3.1e-15	4.1e-14
55	0.0084762	0.0017044	115	58	115	2	0	0	1.7e-15	3.7e-14
-	0.01	-	out							
56	0.011885	0.0034087	117	59	117	2	0	0	2.1e-15	5.6e-14
-	0.015	-	out							
57	0.015294	0.0034087	119	60	119	2	0	0	2.6e-15	4.9e-14
58	0.018702	0.0034087	121	61	121	2	0	0	5.4e-15	4.8e-14
-	0.02	-	out							
-	0.025	-	out							
59	0.02552	0.0068174	123	62	123	2	0	0	7.2e-15	6.1e-14
-	0.03	-	out							
60	0.032337	0.0068174	125	63	125	2	0	0	5.7e-15	5.1e-14
-	0.035	-	out							
-	0.04	-	out							
-	0.045	-	out							
61	0.045972	0.013635	127	64	127	2	0	0	3.4e-15	8.2e-14
-	0.05	-	out							
-	0.055	-	out							
62	0.059607	0.013635	129	65	129	1	0	0	4.2e-15	8.1e-14
-	0.06	-	out							
-	0.065	-	out							
-	0.07	-	out							
63	0.073241	0.013635	131	66	131	1	0	0	1.5e-14	3.1e-14
-	0.075	-	out							
-	0.08	-	out							
-	0.085	-	out							
-	0.09	-	out							
-	0.095	-	out							
-	0.1	-	out							
64	0.10051	0.02727	133	67	133	1	0	0	1.9e-14	4.6e-14
-	0.105	-	out							
-	0.11	-	out							
-	0.115	-	out							
-	0.12	-	out							
-	0.125	-	out							
-	0.13	-	out							
-	0.135	-	out							
-	0.14	-	out							
-	0.145	-	out							
-	0.15	-	out							
-	0.155	-	out							
65	0.15505	0.054539	135	68	135	1	0	0	5e-15	5.7e-14
-	0.16	-	out							
-	0.165	-	out							
-	0.17	-	out							

-	0.175	- out								
-	0.18	- out								
-	0.185	- out								
-	0.19	- out								
-	0.195	- out								
-	0.2	- out								
-	0.205	- out								
66	0.20959	0.054539	137	69	137	1	0	0	8.2e-15	4.9e-14
-	0.21	- out								
-	0.215	- out								
-	0.22	- out								
-	0.225	- out								
-	0.23	- out								
-	0.235	- out								
-	0.24	- out								
-	0.245	- out								
-	0.25	- out								
-	0.25	- out								
-	0.27	- out								
-	0.29	- out								
67	0.30859	0.099	139	70	139	1	0	0	9.5e-15	4.9e-14
-	0.31	- out								
-	0.33	- out								
-	0.35	- out								
-	0.37	- out								
-	0.39	- out								
68	0.40759	0.099	141	71	141	1	0	0	1.1e-14	4e-14
-	0.41	- out								
-	0.43	- out								
-	0.45	- out								
-	0.47	- out								
-	0.49	- out								
69	0.50659	0.099	143	72	143	1	0	0	2.2e-14	3.1e-14
-	0.51	- out								
-	0.53	- out								
-	0.55	- out								
-	0.57	- out								
-	0.59	- out								
70	0.60559	0.099	145	73	145	1	0	0	4.6e-15	2.5e-14
-	0.61	- out								
-	0.63	- out								
-	0.65	- out								
-	0.67	- out								
-	0.69	- out								
71	0.70459	0.099	146	74	146	1	0	0	2.1e-15	2.1e-14
-	0.71	- out								
-	0.73	- out								
-	0.75	- out								
-	0.77	- out								
-	0.79	- out								
72	0.80359	0.099	147	75	147	1	0	0	1.7e-14	1.8e-14
-	0.81	- out								
-	0.83	- out								
-	0.85	- out								
-	0.87	- out								
-	0.89	- out								

```
  73      0.90259       0.099       148    76   148      2      0       0     4e-15   1.6e-14
   -         0.91           - out
   -         0.93           - out
   -         0.95           - out
   -         0.97           - out
   -         0.99           - out
  74       1.0016       0.099       149    77   149      2      0       0     7e-15   9.5e-15
Time-stepping completed.
Solution time: 13 s.
Physical memory: 1.34 GB
Virtual memory: 1.32 GB
Ended at May 28, 2023, 4:22:03 PM.
----- Time-Dependent Solver 1 in Oxygen Pulse/Solution 958 (sol958) ----------->
```

k0Sub=1.51E-5, d=2E-6 (su64)

GENERAL

Description	Value
Solution	k0Sub=1.51E-5, d=2E-6

LOG

```
<---- Compile Equations: Stationary in Oxygen Pulse/Solution 958 (sol958) ------
Started at May 28, 2023, 4:22:06 PM.
Parameter k0Sub = 1.51E-5 (m/s).
Parameter d = 2.0E-6 (m).
Geometry shape function: Linear Lagrange
Number of vertex elements: 10
Minimum element quality: 0.4112
Time: 0 s.
Physical memory: 1.31 GB
Virtual memory: 1.29 GB
Ended at May 28, 2023, 4:22:06 PM.
----- Compile Equations: Stationary in Oxygen Pulse/Solution 958 (sol958) ----->

<---- Dependent Variables 1 in Oxygen Pulse/Solution 958 (sol958) --------------
Started at May 28, 2023, 4:22:06 PM.
Solution time: 0 s.
Physical memory: 1.31 GB
Virtual memory: 1.29 GB
Ended at May 28, 2023, 4:22:06 PM.
----- Dependent Variables 1 in Oxygen Pulse/Solution 958 (sol958) ------------->

<---- Stationary Solver 1 in Oxygen Pulse/Solution 958 (sol958) ----------------
Started at May 28, 2023, 4:22:06 PM.
Linear solver
Number of degrees of freedom solved for: 17907 (plus 938 internal DOFs).
Symmetric matrices found.
Scales for dependent variables:
Concentration (comp2.Oxi): 0.23
Orthonormal null-space function used.
Iter      SolEst     Damping    Stepsize #Res #Jac #Sol   LinErr   LinRes
   1       0.013   1.0000000       0.013    1    1    1  4.3e-13    1e-13
Solution time: 1 s.
Physical memory: 1.32 GB
Virtual memory: 1.3 GB
Ended at May 28, 2023, 4:22:07 PM.
----- Stationary Solver 1 in Oxygen Pulse/Solution 958 (sol958) --------------->

<---- Compile Equations: Oxygen Probe Pulse in Oxygen Pulse/Solution 958
      (sol958) ----------------------------------------------------------------
Started at May 28, 2023, 4:22:07 PM.
Geometry shape function: Linear Lagrange
Time: 0 s.
Physical memory: 1.32 GB
Virtual memory: 1.3 GB
Ended at May 28, 2023, 4:22:07 PM.
----- Compile Equations: Oxygen Probe Pulse in Oxygen Pulse/Solution 958
      (sol958) --------------------------------------------------------------->

<---- Dependent Variables 2 in Oxygen Pulse/Solution 958 (sol958) --------------
Started at May 28, 2023, 4:22:07 PM.
Initial values of variables solved for: Solution 958 (sol958), Solution Store 1 (sol
985).
Values of variables not solved for: Solution 958 (sol958), Solution Store 1 (sol985)
.
Solution time: 0 s.
Physical memory: 1.32 GB
```

```
Virtual memory: 1.3 GB
Ended at May 28, 2023, 4:22:07 PM.
----- Dependent Variables 2 in Oxygen Pulse/Solution 958 (sol958) ------------->

<---- Time-Dependent Solver 1 in Oxygen Pulse/Solution 958 (sol958) ------------
Started at May 28, 2023, 4:22:07 PM.
Time-dependent solver (BDF)
Number of degrees of freedom solved for: 17907 (plus 35898 internal DOFs).
Symmetric matrices found.
Scales for dependent variables:
Concentration (comp2.Oxi): 0.23
Step        Time    Stepsize      Res  Jac  Sol Order Tfail NLfail   LinErr   LinRes
   0           0           - out    5    3    5                  0  9.2e-16  1.4e-15
   1  2.5994e-08  2.5994e-08        7    4    7     1     0      0  3.5e-16  1.1e-15
   2  5.1987e-08  2.5994e-08        9    5    9     1     0      0  3.4e-16  1.2e-15
   3  1.0397e-07  5.1987e-08       11    6   11     2     0      0  3.8e-16  1.2e-15
   4  2.0795e-07  1.0397e-07       13    7   13     2     0      0  4.6e-16  1.8e-15
   5   4.159e-07  2.0795e-07       15    8   15     2     0      0  5.4e-16  3.2e-15
   6  6.2385e-07  2.0795e-07       17    9   17     2     0      0  6.1e-16    4e-15
   7   8.318e-07  2.0795e-07       19   10   19     2     0      0  6.5e-16  4.1e-15
   8  1.0397e-06  2.0795e-07       21   11   21     2     0      0  6.8e-16  4.1e-15
   9  1.4556e-06   4.159e-07       23   12   23     2     0      0  8.3e-16  6.4e-15
  10  1.8715e-06   4.159e-07       25   13   25     2     0      0    7e-16  5.8e-15
  11  2.2874e-06   4.159e-07       27   14   27     2     0      0  8.4e-16    6e-15
  12  2.7033e-06   4.159e-07       29   15   29     2     0      0  7.5e-16  5.5e-15
  13  3.5351e-06   8.318e-07       31   16   31     2     0      0  1.2e-15  8.5e-15
  14  4.3669e-06   8.318e-07       33   17   33     2     0      0    8e-16  7.2e-15
  15  5.1987e-06   8.318e-07       35   18   35     2     0      0  1.1e-15  7.1e-15
  16  6.0305e-06   8.318e-07       37   19   37     2     0      0    1e-15  6.5e-15
  17  7.6941e-06  1.6636e-06       39   20   39     2     0      0  1.6e-15  9.7e-15
  18  9.3577e-06  1.6636e-06       41   21   41     2     0      0  1.6e-15  8.4e-15
  19  1.1021e-05  1.6636e-06       43   22   43     2     0      0  1.3e-15  8.2e-15
  20  1.2685e-05  1.6636e-06       45   23   45     2     0      0  1.1e-15  7.4e-15
  21  1.4349e-05  1.6636e-06       47   24   47     2     0      0    1e-15  6.4e-15
  22  1.7676e-05  3.3272e-06       49   25   49     2     0      0  1.1e-15  9.7e-15
  23  2.1003e-05  3.3272e-06       51   26   51     2     0      0  1.8e-15  8.3e-15
  24   2.433e-05  3.3272e-06       53   27   53     2     0      0  1.5e-15  8.2e-15
  25  2.7657e-05  3.3272e-06       55   28   55     2     0      0  1.8e-15  7.6e-15
  26  3.4312e-05  6.6544e-06       57   29   57     2     0      0  1.7e-15  1.1e-14
  27  4.0966e-05  6.6544e-06       59   30   59     2     0      0  2.4e-15  9.5e-15
  28  4.7621e-05  6.6544e-06       61   31   61     2     0      0  1.7e-15  9.4e-15
  29  5.4275e-05  6.6544e-06       63   32   63     2     0      0  2.3e-15  8.6e-15
  30  6.0929e-05  6.6544e-06       65   33   65     2     0      0  1.6e-15  7.5e-15
  31  7.4238e-05  1.3309e-05       67   34   67     2     0      0  1.7e-15  1.1e-14
  32  8.7547e-05  1.3309e-05       69   35   69     2     0      0  2.4e-15  9.8e-15
  33  0.00010086  1.3309e-05       71   36   71     2     0      0  1.6e-15  9.4e-15
  34  0.00011416  1.3309e-05       73   37   73     2     0      0    2e-15  9.3e-15
  35  0.00014078  2.6618e-05       75   38   75     2     0      0  2.5e-15  1.3e-14
  36   0.0001674  2.6618e-05       77   39   77     2     0      0  2.5e-15  1.1e-14
  37  0.00019402  2.6618e-05       79   40   79     2     0      0  1.6e-15  1.2e-14
  38  0.00022063  2.6618e-05       81   41   81     2     0      0  1.4e-15    1e-14
  39  0.00027387  5.3235e-05       83   42   83     2     0      0  1.9e-15  1.4e-14
  40  0.00032711  5.3235e-05       85   43   85     2     0      0    1e-15  1.2e-14
  41  0.00038034  5.3235e-05       87   44   87     2     0      0    2e-15  1.2e-14
  42  0.00048681  0.00010647       89   45   89     2     0      0  2.7e-15  1.8e-14
  43  0.00059328  0.00010647       91   46   91     2     0      0  3.8e-15  1.5e-14
```

44	0.00069975	0.00010647		93	47	93	2	0	0	2.8e-15	1.4e-14
45	0.00080622	0.00010647		95	48	95	2	0	0	2.3e-15	1.3e-14
46	0.0010192	0.00021294		97	49	97	2	0	0	7.8e-15	1.8e-14
47	0.0012321	0.00021294		99	50	99	2	0	0	3.7e-15	1.5e-14
48	0.001658	0.00042588		101	51	101	2	0	0	1.8e-15	2.7e-14
49	0.0020839	0.00042588		103	52	103	2	0	0	3.2e-15	2.5e-14
50	0.0025097	0.00042588		105	53	105	2	0	0	2.7e-15	2.3e-14
51	0.0033615	0.00085176		107	54	107	2	0	0	3.2e-15	3e-14
52	0.0042133	0.00085176		109	55	109	2	0	0	5.4e-15	2.1e-14
-	0.005	-	out								
53	0.0059168	0.0017035		111	56	111	2	0	0	6.2e-15	3.6e-14
54	0.0076203	0.0017035		113	57	113	2	0	0	6.8e-15	3.6e-14
55	0.0093239	0.0017035		115	58	115	2	0	0	2.3e-15	3.5e-14
-	0.01	-	out								
56	0.011027	0.0017035		117	59	117	2	0	0	9.7e-15	3.4e-14
57	0.014434	0.0034071		119	60	119	2	0	0	3.4e-15	4.6e-14
-	0.015	-	out								
58	0.017841	0.0034071		121	61	121	2	0	0	2.2e-15	3.4e-14
-	0.02	-	out								
59	0.024656	0.0068141		123	62	123	2	0	0	3.6e-15	5.7e-14
-	0.025	-	out								
-	0.03	-	out								
60	0.03147	0.0068141		125	63	125	2	0	0	5.7e-15	5.2e-14
-	0.035	-	out								
61	0.038284	0.0068141		127	64	127	2	0	0	2.7e-15	5.3e-14
-	0.04	-	out								
-	0.045	-	out								
-	0.05	-	out								
62	0.051912	0.013628		129	65	129	2	0	0	6.1e-15	7e-14
-	0.055	-	out								
-	0.06	-	out								
-	0.065	-	out								
63	0.06554	0.013628		131	66	131	1	0	0	6.7e-15	6.7e-14
-	0.07	-	out								
-	0.075	-	out								
64	0.079168	0.013628		133	67	133	1	0	0	1.3e-15	3.3e-14
-	0.08	-	out								
-	0.085	-	out								
-	0.09	-	out								
-	0.095	-	out								
-	0.1	-	out								
-	0.105	-	out								
65	0.10642	0.027256		135	68	135	1	0	0	1.1e-14	4.6e-14
-	0.11	-	out								
-	0.115	-	out								
-	0.12	-	out								
-	0.125	-	out								
-	0.13	-	out								
-	0.135	-	out								
-	0.14	-	out								
-	0.145	-	out								
-	0.15	-	out								
-	0.155	-	out								
-	0.16	-	out								
66	0.16094	0.054513		137	69	137	1	0	0	1e-14	5.9e-14
-	0.165	-	out								

-	0.17	-	out							
-	0.175	-	out							
-	0.18	-	out							
-	0.185	-	out							
-	0.19	-	out							
-	0.195	-	out							
-	0.2	-	out							
-	0.205	-	out							
-	0.21	-	out							
-	0.215	-	out							
67	0.21545	0.054513	139	70	139	1	0	0	8.9e-15	4.9e-14
-	0.22	-	out							
-	0.225	-	out							
-	0.23	-	out							
-	0.235	-	out							
-	0.24	-	out							
-	0.245	-	out							
-	0.25	-	out							
-	0.25	-	out							
-	0.27	-	out							
-	0.29	-	out							
-	0.31	-	out							
68	0.31445	0.099	141	71	141	1	0	0	2.7e-15	5.1e-14
-	0.33	-	out							
-	0.35	-	out							
-	0.37	-	out							
-	0.39	-	out							
-	0.41	-	out							
69	0.41345	0.099	143	72	143	1	0	0	1.1e-14	4e-14
-	0.43	-	out							
-	0.45	-	out							
-	0.47	-	out							
-	0.49	-	out							
-	0.51	-	out							
70	0.51245	0.099	145	73	145	1	0	0	1.2e-14	3e-14
-	0.53	-	out							
-	0.55	-	out							
-	0.57	-	out							
-	0.59	-	out							
-	0.61	-	out							
71	0.61145	0.099	147	74	147	1	0	0	1.3e-14	2.7e-14
-	0.63	-	out							
-	0.65	-	out							
-	0.67	-	out							
-	0.69	-	out							
-	0.71	-	out							
72	0.71045	0.099	148	75	148	1	0	0	1.7e-14	2.2e-14
-	0.73	-	out							
-	0.75	-	out							
-	0.77	-	out							
-	0.79	-	out							
73	0.80945	0.099	149	76	149	1	0	0	4.2e-15	1.9e-14
-	0.81	-	out							
-	0.83	-	out							
-	0.85	-	out							
-	0.87	-	out							

```
   -        0.89           - out
  74      0.90845       0.099       150    77   150      2      0       0  6.4e-15  1.5e-14
   -        0.91           - out
   -        0.93           - out
   -        0.95           - out
   -        0.97           - out
   -        0.99           - out
  75       1.0075       0.099       151    78   151      2      0       0  7.9e-15    1e-14
Time-stepping completed.
Solution time: 13 s.
Physical memory: 1.34 GB
Virtual memory: 1.32 GB
Ended at May 28, 2023, 4:22:21 PM.
----- Time-Dependent Solver 1 in Oxygen Pulse/Solution 958 (sol958) ----------->
```

k0Sub=1.51E-5, d=2.4E-6 (su65)

GENERAL

Description	Value
Solution	k0Sub=1.51E-5, d=2.4E-6

LOG

```
<---- Compile Equations: Stationary in Oxygen Pulse/Solution 958 (sol958) ------
Started at May 28, 2023, 4:22:24 PM.
Parameter k0Sub = 1.51E-5 (m/s).
Parameter d = 2.4E-6 (m).
Geometry shape function: Linear Lagrange
Number of vertex elements: 10
Minimum element quality: 0.4544
Time: 1 s.
Physical memory: 1.31 GB
Virtual memory: 1.29 GB
Ended at May 28, 2023, 4:22:24 PM.
----- Compile Equations: Stationary in Oxygen Pulse/Solution 958 (sol958) ----->

<---- Dependent Variables 1 in Oxygen Pulse/Solution 958 (sol958) --------------
Started at May 28, 2023, 4:22:24 PM.
Solution time: 0 s.
Physical memory: 1.31 GB
Virtual memory: 1.29 GB
Ended at May 28, 2023, 4:22:24 PM.
----- Dependent Variables 1 in Oxygen Pulse/Solution 958 (sol958) ------------->

<---- Stationary Solver 1 in Oxygen Pulse/Solution 958 (sol958) ----------------
Started at May 28, 2023, 4:22:24 PM.
Linear solver
Number of degrees of freedom solved for: 18002 (plus 937 internal DOFs).
Symmetric matrices found.
Scales for dependent variables:
Concentration (comp2.Oxi): 0.23
Orthonormal null-space function used.
Iter      SolEst     Damping    Stepsize #Res #Jac #Sol   LinErr   LinRes
   1       0.012   1.0000000       0.012    1    1    1  3.5e-13  9.3e-14
Solution time: 1 s.
Physical memory: 1.32 GB
Virtual memory: 1.3 GB
Ended at May 28, 2023, 4:22:25 PM.
----- Stationary Solver 1 in Oxygen Pulse/Solution 958 (sol958) --------------->

<---- Compile Equations: Oxygen Probe Pulse in Oxygen Pulse/Solution 958
      (sol958) ----------------------------------------------------------------
Started at May 28, 2023, 4:22:25 PM.
Geometry shape function: Linear Lagrange
Time: 0 s.
Physical memory: 1.32 GB
Virtual memory: 1.3 GB
Ended at May 28, 2023, 4:22:25 PM.
----- Compile Equations: Oxygen Probe Pulse in Oxygen Pulse/Solution 958
      (sol958) --------------------------------------------------------------->

<---- Dependent Variables 2 in Oxygen Pulse/Solution 958 (sol958) --------------
Started at May 28, 2023, 4:22:25 PM.
Initial values of variables solved for: Solution 958 (sol958), Solution Store 1 (sol
985).
Values of variables not solved for: Solution 958 (sol958), Solution Store 1 (sol985)
.
Solution time: 0 s.
Physical memory: 1.32 GB
```

Virtual memory: 1.3 GB
Ended at May 28, 2023, 4:22:25 PM.
----- Dependent Variables 2 in Oxygen Pulse/Solution 958 (sol958) ------------->

<---- Time-Dependent Solver 1 in Oxygen Pulse/Solution 958 (sol958) ------------
Started at May 28, 2023, 4:22:25 PM.
Time-dependent solver (BDF)
Number of degrees of freedom solved for: 18002 (plus 36084 internal DOFs).
Symmetric matrices found.
Scales for dependent variables:
Concentration (comp2.Oxi): 0.23

Step	Time	Stepsize		Res	Jac	Sol	Order	Tfail	NLfail	LinErr	LinRes
0	0	-	out	5	3	5			0	7.9e-16	1.5e-15
1	2.6098e-08	2.6098e-08		7	4	7	1	0	0	3.5e-16	1.1e-15
2	5.2195e-08	2.6098e-08		9	5	9	1	0	0	3.3e-16	1.1e-15
3	1.0439e-07	5.2195e-08		11	6	11	2	0	0	3.9e-16	1.3e-15
4	2.0878e-07	1.0439e-07		13	7	13	2	0	0	5.1e-16	1.8e-15
5	4.1756e-07	2.0878e-07		15	8	15	2	0	0	6.7e-16	3.5e-15
6	6.2634e-07	2.0878e-07		17	9	17	2	0	0	6.3e-16	3.8e-15
7	8.3512e-07	2.0878e-07		19	10	19	2	0	0	7e-16	4e-15
8	1.0439e-06	2.0878e-07		21	11	21	2	0	0	6e-16	3.9e-15
9	1.4615e-06	4.1756e-07		23	12	23	2	0	0	1.1e-15	6.5e-15
10	1.879e-06	4.1756e-07		25	13	25	2	0	0	7.9e-16	6.1e-15
11	2.2966e-06	4.1756e-07		27	14	27	2	0	0	8.6e-16	6.1e-15
12	2.7141e-06	4.1756e-07		29	15	29	2	0	0	7.2e-16	5.8e-15
13	3.5493e-06	8.3512e-07		31	16	31	2	0	0	1.1e-15	8.7e-15
14	4.3844e-06	8.3512e-07		33	17	33	2	0	0	1.1e-15	7.4e-15
15	5.2195e-06	8.3512e-07		35	18	35	2	0	0	1e-15	7.5e-15
16	6.0546e-06	8.3512e-07		37	19	37	2	0	0	1.1e-15	6.7e-15
17	7.7249e-06	1.6702e-06		39	20	39	2	0	0	1.3e-15	9.9e-15
18	9.3951e-06	1.6702e-06		41	21	41	2	0	0	1.2e-15	8.7e-15
19	1.1065e-05	1.6702e-06		43	22	43	2	0	0	1.1e-15	8.6e-15
20	1.2736e-05	1.6702e-06		45	23	45	2	0	0	1.2e-15	7.7e-15
21	1.4406e-05	1.6702e-06		47	24	47	2	0	0	2.1e-15	6.9e-15
22	1.7746e-05	3.3405e-06		49	25	49	2	0	0	1.5e-15	1e-14
23	2.1087e-05	3.3405e-06		51	26	51	2	0	0	1.4e-15	8.7e-15
24	2.4427e-05	3.3405e-06		53	27	53	2	0	0	1.7e-15	8.6e-15
25	2.7768e-05	3.3405e-06		55	28	55	2	0	0	1.5e-15	7.9e-15
26	3.4449e-05	6.681e-06		57	29	57	2	0	0	1.5e-15	1.1e-14
27	4.113e-05	6.681e-06		59	30	59	2	0	0	1.2e-15	1e-14
28	4.7811e-05	6.681e-06		61	31	61	2	0	0	2.2e-15	9.9e-15
29	5.4492e-05	6.681e-06		63	32	63	2	0	0	1.4e-15	8.8e-15
30	6.1173e-05	6.681e-06		65	33	65	2	0	0	1.4e-15	7.9e-15
31	7.4534e-05	1.3362e-05		67	34	67	2	0	0	1.7e-15	1.2e-14
32	8.7896e-05	1.3362e-05		69	35	69	2	0	0	1.4e-15	1e-14
33	0.00010126	1.3362e-05		71	36	71	2	0	0	1.6e-15	1e-14
34	0.00011462	1.3362e-05		73	37	73	2	0	0	1.3e-15	9.5e-15
35	0.00014134	2.6724e-05		75	38	75	2	0	0	2.3e-15	1.5e-14
36	0.00016807	2.6724e-05		77	39	77	2	0	0	3.2e-15	1.1e-14
37	0.00019479	2.6724e-05		79	40	79	2	0	0	1.9e-15	1.2e-14
38	0.00022152	2.6724e-05		81	41	81	2	0	0	1.8e-15	1.1e-14
39	0.00027496	5.3448e-05		83	42	83	2	0	0	2.8e-15	1.5e-14
40	0.00032841	5.3448e-05		85	43	85	2	0	0	3e-15	1.3e-14
41	0.00038186	5.3448e-05		87	44	87	2	0	0	3.9e-15	1.3e-14
42	0.00048875	0.0001069		89	45	89	2	0	0	2.8e-15	2e-14
43	0.00059565	0.0001069		91	46	91	2	0	0	1.9e-15	1.6e-14

44	0.00070254	0.0001069		93	47	93	2	0	0	3.1e-15	1.6e-14
45	0.00080944	0.0001069		95	48	95	2	0	0	1.6e-15	1.3e-14
46	0.0010232	0.00021379		97	49	97	2	0	0	2e-15	1.9e-14
47	0.001237	0.00021379		99	50	99	2	0	0	3.6e-15	1.6e-14
48	0.0016646	0.00042758		101	51	101	2	0	0	4.2e-15	2.5e-14
49	0.0020922	0.00042758		103	52	103	2	0	0	3.5e-15	2.5e-14
50	0.0025198	0.00042758		105	53	105	2	0	0	3.5e-15	2.5e-14
51	0.0029473	0.00042758		107	54	107	2	0	0	2.7e-15	2.1e-14
52	0.0038025	0.00085516		109	55	109	2	0	0	2.6e-15	2.6e-14
53	0.0046577	0.00085516		111	56	111	2	0	0	2.5e-15	2e-14
-	0.005	-	out								
54	0.006368	0.0017103		113	57	113	2	0	0	3.2e-15	3.3e-14
55	0.0080783	0.0017103		115	58	115	2	0	0	1.8e-15	3.3e-14
-	0.01	-	out								
56	0.011499	0.0034207		117	59	117	2	0	0	3e-15	5.1e-14
57	0.01492	0.0034207		119	60	119	2	0	0	5.4e-15	4.6e-14
-	0.015	-	out								
58	0.01834	0.0034207		121	61	121	2	0	0	4.9e-15	4.8e-14
-	0.02	-	out								
-	0.025	-	out								
59	0.025182	0.0068413		123	62	123	2	0	0	5.7e-15	5.8e-14
-	0.03	-	out								
60	0.032023	0.0068413		125	63	125	2	0	0	4.3e-15	4.5e-14
-	0.035	-	out								
-	0.04	-	out								
-	0.045	-	out								
61	0.045705	0.013683		127	64	127	2	0	0	3.6e-15	7.7e-14
-	0.05	-	out								
-	0.055	-	out								
62	0.059388	0.013683		129	65	129	2	0	0	1.5e-14	6.4e-14
-	0.06	-	out								
-	0.065	-	out								
-	0.07	-	out								
63	0.073071	0.013683		131	66	131	2	0	0	5.9e-15	6.3e-14
-	0.075	-	out								
-	0.08	-	out								
-	0.085	-	out								
64	0.086753	0.013683		133	67	133	2	0	0	1.1e-14	4.9e-14
-	0.09	-	out								
-	0.095	-	out								
-	0.1	-	out								
-	0.105	-	out								
-	0.11	-	out								
65	0.11412	0.027365		135	68	135	2	0	0	8.3e-15	4.7e-14
-	0.115	-	out								
-	0.12	-	out								
-	0.125	-	out								
-	0.13	-	out								
-	0.135	-	out								
-	0.14	-	out								
-	0.145	-	out								
-	0.15	-	out								
-	0.155	-	out								
-	0.16	-	out								
-	0.165	-	out								
66	0.16885	0.05473		137	69	137	1	0	0	1.7e-14	5.1e-14

-	0.17	-	out							
-	0.175	-	out							
-	0.18	-	out							
-	0.185	-	out							
-	0.19	-	out							
-	0.195	-	out							
-	0.2	-	out							
-	0.205	-	out							
-	0.21	-	out							
-	0.215	-	out							
-	0.22	-	out							
67	0.22358	0.05473	139	70	139	1	0	0	8.8e-15	3.1e-14
-	0.225	-	out							
-	0.23	-	out							
-	0.235	-	out							
-	0.24	-	out							
-	0.245	-	out							
-	0.25	-	out							
-	0.25	-	out							
-	0.27	-	out							
-	0.29	-	out							
-	0.31	-	out							
68	0.32258	0.099	141	71	141	1	0	0	7.1e-15	4.2e-14
-	0.33	-	out							
-	0.35	-	out							
-	0.37	-	out							
-	0.39	-	out							
-	0.41	-	out							
69	0.42158	0.099	143	72	143	1	0	0	4.3e-15	3.6e-14
-	0.43	-	out							
-	0.45	-	out							
-	0.47	-	out							
-	0.49	-	out							
-	0.51	-	out							
70	0.52058	0.099	145	73	145	1	0	0	5.7e-15	3e-14
-	0.53	-	out							
-	0.55	-	out							
-	0.57	-	out							
-	0.59	-	out							
-	0.61	-	out							
71	0.61958	0.099	147	74	147	1	0	0	1.3e-14	2.5e-14
-	0.63	-	out							
-	0.65	-	out							
-	0.67	-	out							
-	0.69	-	out							
-	0.71	-	out							
72	0.71858	0.099	148	75	148	1	0	0	4.7e-15	2.1e-14
-	0.73	-	out							
-	0.75	-	out							
-	0.77	-	out							
-	0.79	-	out							
-	0.81	-	out							
73	0.81758	0.099	149	76	149	1	0	0	4.1e-15	1.8e-14
-	0.83	-	out							
-	0.85	-	out							
-	0.87	-	out							

```
  -       0.89           - out
  -       0.91           - out
 74    0.91658       0.099      150   77  150     2     0      0  3.7e-15  1.5e-14
  -       0.93           - out
  -       0.95           - out
  -       0.97           - out
  -       0.99           - out
 75     1.0156       0.099      151   78  151     2     0      0  4.1e-15  9.4e-15
Time-stepping completed.
Solution time: 14 s.
Physical memory: 1.35 GB
Virtual memory: 1.33 GB
Ended at May 28, 2023, 4:22:39 PM.
----- Time-Dependent Solver 1 in Oxygen Pulse/Solution 958 (sol958) ----------->
```

k0Sub=1.51E-5, d=3E-6 (su66)

GENERAL

Description	Value
Solution	k0Sub=1.51E-5, d=3E-6

LOG

```
<---- Compile Equations: Stationary in Oxygen Pulse/Solution 958 (sol958) ------
Started at May 28, 2023, 4:22:41 PM.
Parameter k0Sub = 1.51E-5 (m/s).
Parameter d = 3.0E-6 (m).
Geometry shape function: Linear Lagrange
Number of vertex elements: 10
Minimum element quality: 0.4275
Time: 1 s.
Physical memory: 1.32 GB
Virtual memory: 1.3 GB
Ended at May 28, 2023, 4:22:42 PM.
----- Compile Equations: Stationary in Oxygen Pulse/Solution 958 (sol958) ----->

<---- Dependent Variables 1 in Oxygen Pulse/Solution 958 (sol958) --------------
Started at May 28, 2023, 4:22:42 PM.
Solution time: 0 s.
Physical memory: 1.32 GB
Virtual memory: 1.3 GB
Ended at May 28, 2023, 4:22:42 PM.
----- Dependent Variables 1 in Oxygen Pulse/Solution 958 (sol958) ------------->

<---- Stationary Solver 1 in Oxygen Pulse/Solution 958 (sol958) ----------------
Started at May 28, 2023, 4:22:42 PM.
Linear solver
Number of degrees of freedom solved for: 22990 (plus 935 internal DOFs).
Symmetric matrices found.
Scales for dependent variables:
Concentration (comp2.Oxi): 0.23
Orthonormal null-space function used.
Iter      SolEst     Damping    Stepsize #Res #Jac #Sol   LinErr   LinRes
   1      0.0094   1.0000000      0.0094    1    1    1  4.1e-13    9e-14
Solution time: 1 s.
Physical memory: 1.33 GB
Virtual memory: 1.31 GB
Ended at May 28, 2023, 4:22:43 PM.
----- Stationary Solver 1 in Oxygen Pulse/Solution 958 (sol958) --------------->

<---- Compile Equations: Oxygen Probe Pulse in Oxygen Pulse/Solution 958
      (sol958) ----------------------------------------------------------------
Started at May 28, 2023, 4:22:43 PM.
Geometry shape function: Linear Lagrange
Time: 0 s.
Physical memory: 1.33 GB
Virtual memory: 1.31 GB
Ended at May 28, 2023, 4:22:43 PM.
----- Compile Equations: Oxygen Probe Pulse in Oxygen Pulse/Solution 958
      (sol958) --------------------------------------------------------------->

<---- Dependent Variables 2 in Oxygen Pulse/Solution 958 (sol958) --------------
Started at May 28, 2023, 4:22:43 PM.
Initial values of variables solved for: Solution 958 (sol958), Solution Store 1 (sol
985).
Values of variables not solved for: Solution 958 (sol958), Solution Store 1 (sol985)
.
Solution time: 0 s.
Physical memory: 1.33 GB
```

Virtual memory: 1.31 GB
Ended at May 28, 2023, 4:22:43 PM.
----- Dependent Variables 2 in Oxygen Pulse/Solution 958 (sol958) ------------->

<---- Time-Dependent Solver 1 in Oxygen Pulse/Solution 958 (sol958) ------------
Started at May 28, 2023, 4:22:43 PM.
Time-dependent solver (BDF)
Number of degrees of freedom solved for: 22990 (plus 46056 internal DOFs).
Symmetric matrices found.
Scales for dependent variables:
Concentration (comp2.Oxi): 0.23

Step	Time	Stepsize	Res	Jac	Sol	Order	Tfail	NLfail	LinErr	LinRes
0	0	- out	5	3	5			0	5.7e-16	8.7e-16
1	3.581e-08	3.581e-08	7	4	7	1	0	0	3.1e-16	8.9e-16
2	7.1619e-08	3.581e-08	9	5	9	1	0	0	3.2e-16	1e-15
3	1.4324e-07	7.1619e-08	11	6	11	2	0	0	2.9e-16	9.7e-16
4	2.8648e-07	1.4324e-07	13	7	13	2	0	0	4.5e-16	1.4e-15
5	5.7295e-07	2.8648e-07	15	8	15	2	0	0	4.6e-16	2.4e-15
6	8.5943e-07	2.8648e-07	17	9	17	2	0	0	4.8e-16	2.7e-15
7	1.1459e-06	2.8648e-07	19	10	19	2	0	0	5.1e-16	3e-15
8	1.4324e-06	2.8648e-07	21	11	21	2	0	0	5e-16	3.2e-15
9	2.0053e-06	5.7295e-07	23	12	23	2	0	0	6.4e-16	5e-15
10	2.5783e-06	5.7295e-07	25	13	25	2	0	0	6.8e-16	4.6e-15
11	3.1512e-06	5.7295e-07	27	14	27	2	0	0	7.2e-16	4.8e-15
12	3.7242e-06	5.7295e-07	29	15	29	2	0	0	6.1e-16	4.5e-15
13	4.8701e-06	1.1459e-06	31	16	31	2	0	0	8.2e-16	6.6e-15
14	6.016e-06	1.1459e-06	33	17	33	2	0	0	7.1e-16	6e-15
15	7.1619e-06	1.1459e-06	35	18	35	2	0	0	8.5e-16	6.1e-15
16	8.3078e-06	1.1459e-06	37	19	37	2	0	0	7.5e-16	5.7e-15
17	1.06e-05	2.2918e-06	39	20	39	2	0	0	1.2e-15	8.7e-15
18	1.2891e-05	2.2918e-06	41	21	41	2	0	0	1.1e-15	7.4e-15
19	1.5183e-05	2.2918e-06	43	22	43	2	0	0	1.3e-15	8.3e-15
20	1.7475e-05	2.2918e-06	45	23	45	2	0	0	1.2e-15	7.5e-15
21	2.2059e-05	4.5836e-06	47	24	47	2	0	0	1.3e-15	1.1e-14
22	2.6642e-05	4.5836e-06	49	25	49	2	0	0	1.6e-15	9.7e-15
23	3.1226e-05	4.5836e-06	51	26	51	2	0	0	1.6e-15	9.6e-15
24	3.581e-05	4.5836e-06	53	27	53	2	0	0	9.7e-16	9e-15
25	4.4977e-05	9.1673e-06	55	28	55	2	0	0	1.5e-15	1.3e-14
26	5.4144e-05	9.1673e-06	57	29	57	2	0	0	1.3e-15	1.1e-14
27	6.3311e-05	9.1673e-06	59	30	59	2	0	0	1.8e-15	1.2e-14
28	7.2479e-05	9.1673e-06	61	31	61	2	0	0	1.4e-15	1.1e-14
29	9.0813e-05	1.8335e-05	63	32	63	2	0	0	1.8e-15	1.6e-14
30	0.00010915	1.8335e-05	65	33	65	2	0	0	3.6e-15	1.3e-14
31	0.00012748	1.8335e-05	67	34	67	2	0	0	2.3e-15	1.4e-14
32	0.00014582	1.8335e-05	69	35	69	2	0	0	1.6e-15	1.3e-14
33	0.00018249	3.6669e-05	71	36	71	2	0	0	2.5e-15	1.8e-14
34	0.00021916	3.6669e-05	73	37	73	2	0	0	3.3e-15	1.5e-14
35	0.00025582	3.6669e-05	75	38	75	2	0	0	2e-15	1.5e-14
36	0.00029249	3.6669e-05	77	39	77	2	0	0	2.2e-15	1.4e-14
37	0.00036583	7.3338e-05	79	40	79	2	0	0	2.1e-15	2.1e-14
38	0.00043917	7.3338e-05	81	41	81	2	0	0	3.6e-15	1.7e-14
39	0.00058585	0.00014668	83	42	83	2	0	0	2.9e-15	2.7e-14
40	0.00073252	0.00014668	85	43	85	2	0	0	3.7e-15	2.4e-14
41	0.0008792	0.00014668	87	44	87	2	0	0	2.8e-15	2.4e-14
42	0.0010259	0.00014668	89	45	89	2	0	0	3.9e-15	2e-14
43	0.0013192	0.00029335	91	46	91	2	0	0	5.5e-15	2.5e-14

44	0.0016126	0.00029335		93	47	93	2	0	0	2.8e-15	2e-14
45	0.0021993	0.00058671		95	48	95	2	0	0	2.6e-15	3.4e-14
46	0.002786	0.00058671		97	49	97	2	0	0	4.2e-15	3.4e-14
47	0.0033727	0.00058671		99	50	99	2	0	0	2.6e-15	3.3e-14
48	0.0045461	0.0011734		101	51	101	2	0	0	3.6e-15	4.3e-14
-	0.005	-	out								
49	0.0057195	0.0011734		103	52	103	2	0	0	2.6e-15	2.8e-14
50	0.0080663	0.0023468		105	53	105	2	0	0	9.8e-15	4.6e-14
-	0.01	-	out								
51	0.010413	0.0023468		107	54	107	2	0	0	3.7e-15	4.8e-14
52	0.01276	0.0023468		109	55	109	2	0	0	3.5e-15	4.7e-14
-	0.015	-	out								
53	0.017454	0.0046936		111	56	111	2	0	0	6.5e-15	6.4e-14
-	0.02	-	out								
54	0.022147	0.0046936		113	57	113	2	0	0	4.5e-15	4.9e-14
-	0.025	-	out								
-	0.03	-	out								
55	0.031535	0.0093873		115	58	115	2	0	0	1.6e-14	8.1e-14
-	0.035	-	out								
-	0.04	-	out								
56	0.040922	0.0093873		117	59	117	2	0	0	3.8e-15	7e-14
-	0.045	-	out								
-	0.05	-	out								
57	0.050309	0.0093873		119	60	119	2	0	0	3.4e-15	7.4e-14
-	0.055	-	out								
-	0.06	-	out								
-	0.065	-	out								
58	0.069084	0.018775		121	61	121	2	0	0	9.1e-15	9.9e-14
-	0.07	-	out								
-	0.075	-	out								
-	0.08	-	out								
-	0.085	-	out								
59	0.087858	0.018775		123	62	123	1	0	0	5e-15	8.3e-14
-	0.09	-	out								
-	0.095	-	out								
-	0.1	-	out								
-	0.105	-	out								
-	0.11	-	out								
-	0.115	-	out								
-	0.12	-	out								
-	0.125	-	out								
60	0.12541	0.037549		125	63	125	1	0	0	1.8e-14	6.4e-14
-	0.13	-	out								
-	0.135	-	out								
-	0.14	-	out								
-	0.145	-	out								
-	0.15	-	out								
-	0.155	-	out								
-	0.16	-	out								
-	0.165	-	out								
-	0.17	-	out								
-	0.175	-	out								
-	0.18	-	out								
-	0.185	-	out								
-	0.19	-	out								
-	0.195	-	out								

-	0.2	-	out							
61	0.20051	0.075098		127	64	127	1	0	0	9.8e-15	8.5e-14
-	0.205	-	out							
-	0.21	-	out							
-	0.215	-	out							
-	0.22	-	out							
-	0.225	-	out							
-	0.23	-	out							
-	0.235	-	out							
-	0.24	-	out							
-	0.245	-	out							
-	0.25	-	out							
-	0.25	-	out							
-	0.27	-	out							
62	0.2756	0.075098		129	65	129	1	0	0	9.9e-15	7.1e-14
-	0.29	-	out							
-	0.31	-	out							
-	0.33	-	out							
-	0.35	-	out							
-	0.37	-	out							
63	0.3746	0.099		131	66	131	1	0	0	2.5e-14	6.6e-14
-	0.39	-	out							
-	0.41	-	out							
-	0.43	-	out							
-	0.45	-	out							
-	0.47	-	out							
64	0.4736	0.099		133	67	133	1	0	0	1.8e-14	5.3e-14
-	0.49	-	out							
-	0.51	-	out							
-	0.53	-	out							
-	0.55	-	out							
-	0.57	-	out							
65	0.5726	0.099		135	68	135	1	0	0	3.7e-15	4.1e-14
-	0.59	-	out							
-	0.61	-	out							
-	0.63	-	out							
-	0.65	-	out							
-	0.67	-	out							
66	0.6716	0.099		136	69	136	1	0	0	1.5e-14	3.5e-14
-	0.69	-	out							
-	0.71	-	out							
-	0.73	-	out							
-	0.75	-	out							
-	0.77	-	out							
67	0.7706	0.099		137	70	137	1	0	0	9.6e-15	3e-14
-	0.79	-	out							
-	0.81	-	out							
-	0.83	-	out							
-	0.85	-	out							
68	0.8696	0.099		138	71	138	2	0	0	1.9e-14	2.5e-14
-	0.87	-	out							
-	0.89	-	out							
-	0.91	-	out							
-	0.93	-	out							
-	0.95	-	out							
69	0.9686	0.099		139	72	139	2	0	0	6.3e-15	1.6e-14

```
   -        0.97           - out
   -        0.99           - out
  70      1.0676       0.099      140    73  140      2      0       0     1e-14  1.6e-14
Time-stepping completed.
Solution time: 16 s.
Physical memory: 1.36 GB
Virtual memory: 1.34 GB
Ended at May 28, 2023, 4:22:59 PM.
----- Time-Dependent Solver 1 in Oxygen Pulse/Solution 958 (sol958) ----------->
```

k0Sub=1.51E-5, d=4E-6 (su67)

GENERAL

Description	Value
Solution	k0Sub=1.51E-5, d=4E-6

LOG

```
<---- Compile Equations: Stationary in Oxygen Pulse/Solution 958 (sol958) ------
Started at May 28, 2023, 4:23:02 PM.
Parameter k0Sub = 1.51E-5 (m/s).
Parameter d = 4.0E-6 (m).
Geometry shape function: Linear Lagrange
Number of vertex elements: 10
Minimum element quality: 0.4317
Time: 1 s.
Physical memory: 1.32 GB
Virtual memory: 1.31 GB
Ended at May 28, 2023, 4:23:03 PM.
----- Compile Equations: Stationary in Oxygen Pulse/Solution 958 (sol958) ----->

<---- Dependent Variables 1 in Oxygen Pulse/Solution 958 (sol958) --------------
Started at May 28, 2023, 4:23:03 PM.
Solution time: 0 s.
Physical memory: 1.27 GB
Virtual memory: 1.24 GB
Ended at May 28, 2023, 4:23:03 PM.
----- Dependent Variables 1 in Oxygen Pulse/Solution 958 (sol958) ------------->

<---- Stationary Solver 1 in Oxygen Pulse/Solution 958 (sol958) ----------------
Started at May 28, 2023, 4:23:03 PM.
Linear solver
Number of degrees of freedom solved for: 18182 (plus 936 internal DOFs).
Symmetric matrices found.
Scales for dependent variables:
Concentration (comp2.Oxi): 0.23
Orthonormal null-space function used.
Iter      SolEst     Damping    Stepsize #Res #Jac #Sol   LinErr   LinRes
   1      0.0091   1.0000000      0.0091    1    1    1  3.5e-13  7.5e-14
Solution time: 1 s.
Physical memory: 1.3 GB
Virtual memory: 1.27 GB
Ended at May 28, 2023, 4:23:04 PM.
----- Stationary Solver 1 in Oxygen Pulse/Solution 958 (sol958) --------------->

<---- Compile Equations: Oxygen Probe Pulse in Oxygen Pulse/Solution 958
      (sol958) ----------------------------------------------------------------
Started at May 28, 2023, 4:23:04 PM.
Geometry shape function: Linear Lagrange
Time: 0 s.
Physical memory: 1.3 GB
Virtual memory: 1.28 GB
Ended at May 28, 2023, 4:23:04 PM.
----- Compile Equations: Oxygen Probe Pulse in Oxygen Pulse/Solution 958
      (sol958) --------------------------------------------------------------->

<---- Dependent Variables 2 in Oxygen Pulse/Solution 958 (sol958) --------------
Started at May 28, 2023, 4:23:04 PM.
Initial values of variables solved for: Solution 958 (sol958), Solution Store 1 (sol
985).
Values of variables not solved for: Solution 958 (sol958), Solution Store 1 (sol985)
.
Solution time: 0 s.
Physical memory: 1.31 GB
```

Virtual memory: 1.28 GB
Ended at May 28, 2023, 4:23:04 PM.
----- Dependent Variables 2 in Oxygen Pulse/Solution 958 (sol958) ------------->

<---- Time-Dependent Solver 1 in Oxygen Pulse/Solution 958 (sol958) ------------
Started at May 28, 2023, 4:23:04 PM.
Time-dependent solver (BDF)
Number of degrees of freedom solved for: 18182 (plus 36438 internal DOFs).
Symmetric matrices found.
Scales for dependent variables:
Concentration (comp2.Oxi): 0.23

Step	Time	Stepsize		Res	Jac	Sol	Order	Tfail	NLfail	LinErr	LinRes
0	0	-	out	5	3	5			0	9.5e-16	1.4e-15
1	2.6287e-08	2.6287e-08		7	4	7	1	0	0	3.6e-16	1.1e-15
2	5.2575e-08	2.6287e-08		9	5	9	1	0	0	3.8e-16	1.2e-15
3	1.0515e-07	5.2575e-08		11	6	11	2	0	0	3.8e-16	1.3e-15
4	2.103e-07	1.0515e-07		13	7	13	2	0	0	4.8e-16	1.7e-15
5	4.206e-07	2.103e-07		15	8	15	2	0	0	5.2e-16	3.4e-15
6	6.309e-07	2.103e-07		17	9	17	2	0	0	6e-16	3.8e-15
7	8.412e-07	2.103e-07		19	10	19	2	0	0	6.2e-16	4e-15
8	1.0515e-06	2.103e-07		21	11	21	2	0	0	6.4e-16	4e-15
9	1.4721e-06	4.206e-07		23	12	23	2	0	0	9.5e-16	6.4e-15
10	1.8927e-06	4.206e-07		25	13	25	2	0	0	8.7e-16	6e-15
11	2.3133e-06	4.206e-07		27	14	27	2	0	0	8.7e-16	6.1e-15
12	2.7339e-06	4.206e-07		29	15	29	2	0	0	9.9e-16	5.7e-15
13	3.5751e-06	8.412e-07		31	16	31	2	0	0	1e-15	8.4e-15
14	4.4163e-06	8.412e-07		33	17	33	2	0	0	1.4e-15	7.3e-15
15	5.2575e-06	8.412e-07		35	18	35	2	0	0	1.4e-15	7.3e-15
16	6.0987e-06	8.412e-07		37	19	37	2	0	0	1.1e-15	6.6e-15
17	7.7811e-06	1.6824e-06		39	20	39	2	0	0	1.4e-15	1e-14
18	9.4635e-06	1.6824e-06		41	21	41	2	0	0	1.1e-15	8.4e-15
19	1.1146e-05	1.6824e-06		43	22	43	2	0	0	1.1e-15	8.1e-15
20	1.2828e-05	1.6824e-06		45	23	45	2	0	0	9.1e-16	7.5e-15
21	1.4511e-05	1.6824e-06		47	24	47	2	0	0	1e-15	6.7e-15
22	1.7875e-05	3.3648e-06		49	25	49	2	0	0	1.5e-15	1e-14
23	2.124e-05	3.3648e-06		51	26	51	2	0	0	1.7e-15	8.7e-15
24	2.4605e-05	3.3648e-06		53	27	53	2	0	0	1.3e-15	8.8e-15
25	2.797e-05	3.3648e-06		55	28	55	2	0	0	1.4e-15	7.9e-15
26	3.4699e-05	6.7296e-06		57	29	57	2	0	0	2.3e-15	1.2e-14
27	4.1429e-05	6.7296e-06		59	30	59	2	0	0	2.4e-15	1e-14
28	4.8159e-05	6.7296e-06		61	31	61	2	0	0	2.1e-15	9.7e-15
29	5.4888e-05	6.7296e-06		63	32	63	2	0	0	1.5e-15	8.9e-15
30	6.1618e-05	6.7296e-06		65	33	65	2	0	0	1.7e-15	8.3e-15
31	7.5077e-05	1.3459e-05		67	34	67	2	0	0	2.1e-15	1.2e-14
32	8.8536e-05	1.3459e-05		69	35	69	2	0	0	1.5e-15	1.1e-14
33	0.000102	1.3459e-05		71	36	71	2	0	0	1.8e-15	1e-14
34	0.00011545	1.3459e-05		73	37	73	2	0	0	1.4e-15	9.5e-15
35	0.00014237	2.6918e-05		75	38	75	2	0	0	2.5e-15	1.4e-14
36	0.00016929	2.6918e-05		77	39	77	2	0	0	1.8e-15	1.2e-14
37	0.00019621	2.6918e-05		79	40	79	2	0	0	1.8e-15	1.2e-14
38	0.00022313	2.6918e-05		81	41	81	2	0	0	2.7e-15	1.1e-14
39	0.00027696	5.3837e-05		83	42	83	2	0	0	2.3e-15	1.6e-14
40	0.0003308	5.3837e-05		85	43	85	2	0	0	2.9e-15	1.3e-14
41	0.00038464	5.3837e-05		87	44	87	2	0	0	2.2e-15	1.3e-14
42	0.00049231	0.00010767		89	45	89	2	0	0	2.1e-15	2e-14
43	0.00059998	0.00010767		91	46	91	2	0	0	2.4e-15	1.6e-14

44	0.00070766	0.00010767		93	47	93	2	0	0	2.3e-15	1.6e-14
45	0.00081533	0.00010767		95	48	95	2	0	0	2.5e-15	1.4e-14
46	0.0010307	0.00021535		97	49	97	2	0	0	2.5e-15	1.9e-14
47	0.001246	0.00021535		99	50	99	2	0	0	3.1e-15	1.5e-14
48	0.0016767	0.00043069		101	51	101	2	0	0	4.6e-15	2.6e-14
49	0.0021074	0.00043069		103	52	103	2	0	0	4e-15	2.2e-14
50	0.0025381	0.00043069		105	53	105	2	0	0	2.4e-15	2.3e-14
51	0.0029688	0.00043069		107	54	107	2	0	0	2.6e-15	2.1e-14
52	0.0038302	0.00086139		109	55	109	2	0	0	2.4e-15	2.8e-14
53	0.0046916	0.00086139		111	56	111	2	0	0	1.4e-15	2.1e-14
-	0.005	-	out								
54	0.0064143	0.0017228		113	57	113	2	0	0	2.6e-15	3.2e-14
55	0.0081371	0.0017228		115	58	115	2	0	0	2.7e-15	3e-14
-	0.01	-	out								
56	0.011583	0.0034455		117	59	117	2	0	0	2.7e-15	4.4e-14
-	0.015	-	out								
57	0.015028	0.0034455		119	60	119	1	0	0	4.7e-15	5.6e-14
58	0.018474	0.0034455		121	61	121	1	0	0	2.3e-15	4.5e-14
-	0.02	-	out								
-	0.025	-	out								
59	0.025365	0.0068911		123	62	123	1	0	0	3.6e-15	6.9e-14
-	0.03	-	out								
60	0.032256	0.0068911		125	63	125	1	0	0	5.3e-15	6.9e-14
-	0.035	-	out								
-	0.04	-	out								
-	0.045	-	out								
61	0.046038	0.013782		127	64	127	1	0	0	6.8e-15	1e-13
-	0.05	-	out								
-	0.055	-	out								
62	0.05982	0.013782		129	65	129	1	0	0	3.9e-15	8.3e-14
-	0.06	-	out								
-	0.065	-	out								
-	0.07	-	out								
-	0.075	-	out								
-	0.08	-	out								
-	0.085	-	out								
63	0.087385	0.027564		131	66	131	1	0	0	1.7e-14	1e-13
-	0.09	-	out								
-	0.095	-	out								
-	0.1	-	out								
-	0.105	-	out								
-	0.11	-	out								
64	0.11495	0.027564		133	67	133	1	0	0	5.6e-15	8.1e-14
-	0.115	-	out								
-	0.12	-	out								
-	0.125	-	out								
-	0.13	-	out								
-	0.135	-	out								
-	0.14	-	out								
-	0.145	-	out								
-	0.15	-	out								
-	0.155	-	out								
-	0.16	-	out								
-	0.165	-	out								
-	0.17	-	out								
65	0.17008	0.055129		135	68	135	1	0	0	6e-15	8.8e-14

-	0.175	-	out								
-	0.18	-	out								
-	0.185	-	out								
-	0.19	-	out								
-	0.195	-	out								
-	0.2	-	out								
-	0.205	-	out								
-	0.21	-	out								
-	0.215	-	out								
-	0.22	-	out								
-	0.225	-	out								
66	0.22521	0.055129		137	69	137	1	0	0	1.5e-14	6.3e-14
-	0.23	-	out								
-	0.235	-	out								
-	0.24	-	out								
-	0.245	-	out								
-	0.25	-	out								
-	0.25	-	out								
-	0.27	-	out								
-	0.29	-	out								
-	0.31	-	out								
67	0.32421	0.099		139	70	139	1	0	0	1.7e-14	6.4e-14
-	0.33	-	out								
-	0.35	-	out								
-	0.37	-	out								
-	0.39	-	out								
-	0.41	-	out								
68	0.42321	0.099		141	71	141	1	0	0	3.3e-15	4.7e-14
-	0.43	-	out								
-	0.45	-	out								
-	0.47	-	out								
-	0.49	-	out								
-	0.51	-	out								
69	0.52221	0.099		143	72	143	1	0	0	1.7e-14	3.7e-14
-	0.53	-	out								
-	0.55	-	out								
-	0.57	-	out								
-	0.59	-	out								
-	0.61	-	out								
70	0.62121	0.099		145	73	145	1	0	0	1.7e-14	2.8e-14
-	0.63	-	out								
-	0.65	-	out								
-	0.67	-	out								
-	0.69	-	out								
-	0.71	-	out								
71	0.72021	0.099		146	74	146	1	0	0	1.1e-14	2.3e-14
-	0.73	-	out								
-	0.75	-	out								
-	0.77	-	out								
-	0.79	-	out								
-	0.81	-	out								
72	0.81921	0.099		147	75	147	1	0	0	4.8e-15	2.2e-14
-	0.83	-	out								
-	0.85	-	out								
-	0.87	-	out								
-	0.89	-	out								

```
   -       0.91           - out
  73     0.91821       0.099      148    76  148     2     0      0  1.5e-14  1.7e-14
   -       0.93           - out
   -       0.95           - out
   -       0.97           - out
   -       0.99           - out
  74      1.0172       0.099      149    77  149     2     0      0  9.6e-15  1.1e-14
Time-stepping completed.
Solution time: 13 s.
Physical memory: 1.34 GB
Virtual memory: 1.32 GB
Ended at May 28, 2023, 4:23:18 PM.
----- Time-Dependent Solver 1 in Oxygen Pulse/Solution 958 (sol958) ----------->
```

k0Sub=1.51E-5, d=5E-6 (su68)

GENERAL

Description	Value
Solution	k0Sub=1.51E-5, d=5E-6

LOG

```
<---- Compile Equations: Stationary in Oxygen Pulse/Solution 958 (sol958) ------
Started at May 28, 2023, 4:23:20 PM.
Parameter k0Sub = 1.51E-5 (m/s).
Parameter d = 4.9999999999999996E-6 (m).
Geometry shape function: Linear Lagrange
Number of vertex elements: 10
Minimum element quality: 0.4532
Time: 1 s.
Physical memory: 1.33 GB
Virtual memory: 1.31 GB
Ended at May 28, 2023, 4:23:21 PM.
----- Compile Equations: Stationary in Oxygen Pulse/Solution 958 (sol958) ----->

<---- Dependent Variables 1 in Oxygen Pulse/Solution 958 (sol958) --------------
Started at May 28, 2023, 4:23:21 PM.
Solution time: 0 s.
Physical memory: 1.33 GB
Virtual memory: 1.31 GB
Ended at May 28, 2023, 4:23:21 PM.
----- Dependent Variables 1 in Oxygen Pulse/Solution 958 (sol958) ------------->

<---- Stationary Solver 1 in Oxygen Pulse/Solution 958 (sol958) ----------------
Started at May 28, 2023, 4:23:21 PM.
Linear solver
Number of degrees of freedom solved for: 18304 (plus 936 internal DOFs).
Symmetric matrices found.
Scales for dependent variables:
Concentration (comp2.Oxi): 0.23
Orthonormal null-space function used.
Iter      SolEst     Damping    Stepsize #Res #Jac #Sol   LinErr   LinRes
   1      0.0079   1.0000000      0.0079    1    1    1  3.4e-13  6.8e-14
Solution time: 1 s.
Physical memory: 1.34 GB
Virtual memory: 1.31 GB
Ended at May 28, 2023, 4:23:21 PM.
----- Stationary Solver 1 in Oxygen Pulse/Solution 958 (sol958) --------------->

<---- Compile Equations: Oxygen Probe Pulse in Oxygen Pulse/Solution 958
      (sol958) ----------------------------------------------------------------
Started at May 28, 2023, 4:23:22 PM.
Geometry shape function: Linear Lagrange
Time: 0 s.
Physical memory: 1.35 GB
Virtual memory: 1.32 GB
Ended at May 28, 2023, 4:23:22 PM.
----- Compile Equations: Oxygen Probe Pulse in Oxygen Pulse/Solution 958
      (sol958) --------------------------------------------------------------->

<---- Dependent Variables 2 in Oxygen Pulse/Solution 958 (sol958) --------------
Started at May 28, 2023, 4:23:22 PM.
Initial values of variables solved for: Solution 958 (sol958), Solution Store 1 (sol
985).
Values of variables not solved for: Solution 958 (sol958), Solution Store 1 (sol985)
.
Solution time: 0 s.
Physical memory: 1.35 GB
```

```
Virtual memory: 1.32 GB
Ended at May 28, 2023, 4:23:22 PM.
----- Dependent Variables 2 in Oxygen Pulse/Solution 958 (sol958) ------------->

<---- Time-Dependent Solver 1 in Oxygen Pulse/Solution 958 (sol958) ------------
Started at May 28, 2023, 4:23:22 PM.
Time-dependent solver (BDF)
Number of degrees of freedom solved for: 18304 (plus 36680 internal DOFs).
Symmetric matrices found.
Scales for dependent variables:
Concentration (comp2.Oxi): 0.23
Step        Time    Stepsize      Res   Jac   Sol Order Tfail NLfail   LinErr   LinRes
   0           0           - out    5     3     5                 0  9.3e-16  1.4e-15
   1  2.6446e-08  2.6446e-08         7     4     7     1     0      0  3.5e-16  1.1e-15
   2  5.2891e-08  2.6446e-08         9     5     9     1     0      0    4e-16  1.2e-15
   3  1.0578e-07  5.2891e-08        11     6    11     2     0      0  3.5e-16  1.2e-15
   4  2.1157e-07  1.0578e-07        13     7    13     2     0      0  4.7e-16  1.8e-15
   5  4.2313e-07  2.1157e-07        15     8    15     2     0      0  6.2e-16  3.3e-15
   6   6.347e-07  2.1157e-07        17     9    17     2     0      0  6.6e-16  3.8e-15
   7  8.4626e-07  2.1157e-07        19    10    19     2     0      0  6.2e-16  3.8e-15
   8  1.0578e-06  2.1157e-07        21    11    21     2     0      0  7.5e-16    4e-15
   9   1.481e-06  4.2313e-07        23    12    23     2     0      0  7.3e-16  6.1e-15
  10  1.9041e-06  4.2313e-07        25    13    25     2     0      0  8.3e-16    6e-15
  11  2.3272e-06  4.2313e-07        27    14    27     2     0      0  9.9e-16    6e-15
  12  2.7503e-06  4.2313e-07        29    15    29     2     0      0  7.8e-16    6e-15
  13  3.5966e-06  8.4626e-07        31    16    31     2     0      0  1.2e-15  8.6e-15
  14  4.4429e-06  8.4626e-07        33    17    33     2     0      0  1.2e-15  7.8e-15
  15  5.2891e-06  8.4626e-07        35    18    35     2     0      0  9.8e-16  7.6e-15
  16  6.1354e-06  8.4626e-07        37    19    37     2     0      0  1.2e-15  6.8e-15
  17  7.8279e-06  1.6925e-06        39    20    39     2     0      0  2.2e-15  9.8e-15
  18  9.5204e-06  1.6925e-06        41    21    41     2     0      0  1.5e-15  8.5e-15
  19  1.1213e-05  1.6925e-06        43    22    43     2     0      0  1.6e-15  8.2e-15
  20  1.2905e-05  1.6925e-06        45    23    45     2     0      0  1.5e-15  7.4e-15
  21  1.4598e-05  1.6925e-06        47    24    47     2     0      0  1.3e-15  6.9e-15
  22  1.7983e-05   3.385e-06        49    25    49     2     0      0  1.9e-15    1e-14
  23  2.1368e-05   3.385e-06        51    26    51     2     0      0  1.3e-15  8.9e-15
  24  2.4753e-05   3.385e-06        53    27    53     2     0      0  1.5e-15  8.6e-15
  25  2.8138e-05   3.385e-06        55    28    55     2     0      0  2.1e-15  7.9e-15
  26  3.4908e-05  6.7701e-06        57    29    57     2     0      0  1.9e-15  1.1e-14
  27  4.1678e-05  6.7701e-06        59    30    59     2     0      0  1.4e-15  9.9e-15
  28  4.8448e-05  6.7701e-06        61    31    61     2     0      0    2e-15    1e-14
  29  5.5218e-05  6.7701e-06        63    32    63     2     0      0  1.5e-15  8.9e-15
  30  6.1989e-05  6.7701e-06        65    33    65     2     0      0  1.1e-15  8.2e-15
  31  7.5529e-05   1.354e-05        67    34    67     2     0      0  1.3e-15  1.3e-14
  32  8.9069e-05   1.354e-05        69    35    69     2     0      0  1.9e-15  1.1e-14
  33  0.00010261   1.354e-05        71    36    71     2     0      0  2.1e-15  1.1e-14
  34  0.00011615   1.354e-05        73    37    73     2     0      0  2.6e-15    1e-14
  35  0.00014323   2.708e-05        75    38    75     2     0      0  2.1e-15  1.5e-14
  36  0.00017031   2.708e-05        77    39    77     2     0      0  1.7e-15  1.2e-14
  37  0.00019739   2.708e-05        79    40    79     2     0      0  1.7e-15  1.3e-14
  38  0.00022447   2.708e-05        81    41    81     2     0      0  1.6e-15  1.1e-14
  39  0.00027863  5.4161e-05        83    42    83     2     0      0  1.6e-15  1.6e-14
  40  0.00033279  5.4161e-05        85    43    85     2     0      0  1.7e-15  1.4e-14
  41  0.00038695  5.4161e-05        87    44    87     2     0      0  2.4e-15  1.4e-14
  42  0.00049527  0.00010832        89    45    89     2     0      0    2e-15    2e-14
  43  0.00060359  0.00010832        91    46    91     2     0      0  1.4e-15  1.6e-14
```

44	0.00071192	0.00010832	93	47	93	2	0	0	2.6e-15	1.7e-14
45	0.00082024	0.00010832	95	48	95	2	0	0	5e-15	1.5e-14
46	0.0010369	0.00021664	97	49	97	2	0	0	4.6e-15	2e-14
47	0.0012535	0.00021664	99	50	99	2	0	0	2.8e-15	1.6e-14
48	0.0016868	0.00043329	101	51	101	2	0	0	5e-15	2.6e-14
49	0.0021201	0.00043329	103	52	103	2	0	0	3.8e-15	2.3e-14
50	0.0025534	0.00043329	105	53	105	2	0	0	6.3e-15	2.3e-14
51	0.0029867	0.00043329	107	54	107	2	0	0	1.6e-15	2e-14
52	0.0038532	0.00086657	109	55	109	2	0	0	7.2e-15	2.6e-14
53	0.0047198	0.00086657	111	56	111	2	0	0	7.2e-15	2.1e-14
-	0.005	- out								
54	0.0064529	0.0017331	113	57	113	2	0	0	3.9e-15	3.6e-14
55	0.0081861	0.0017331	115	58	115	2	0	0	4.7e-15	3e-14
-	0.01	- out								
56	0.011652	0.0034663	117	59	117	2	0	0	5.5e-15	4.3e-14
-	0.015	- out								
57	0.015119	0.0034663	119	60	119	1	0	0	5.6e-15	5.4e-14
58	0.018585	0.0034663	121	61	121	1	0	0	6.8e-15	4.1e-14
-	0.02	- out								
-	0.025	- out								
59	0.025517	0.0069326	123	62	123	1	0	0	3.7e-15	6.6e-14
-	0.03	- out								
60	0.03245	0.0069326	125	63	125	1	0	0	6.2e-15	6.7e-14
-	0.035	- out								
-	0.04	- out								
-	0.045	- out								
61	0.046315	0.013865	127	64	127	1	0	0	1.1e-14	9.4e-14
-	0.05	- out								
-	0.055	- out								
-	0.06	- out								
62	0.06018	0.013865	129	65	129	1	0	0	1e-14	8.6e-14
-	0.065	- out								
-	0.07	- out								
-	0.075	- out								
-	0.08	- out								
-	0.085	- out								
63	0.087911	0.02773	131	66	131	1	0	0	3.9e-15	1e-13
-	0.09	- out								
-	0.095	- out								
-	0.1	- out								
-	0.105	- out								
-	0.11	- out								
-	0.115	- out								
64	0.11564	0.02773	133	67	133	1	0	0	3.5e-15	8.2e-14
-	0.12	- out								
-	0.125	- out								
-	0.13	- out								
-	0.135	- out								
-	0.14	- out								
-	0.145	- out								
-	0.15	- out								
-	0.155	- out								
-	0.16	- out								
-	0.165	- out								
-	0.17	- out								
65	0.1711	0.05546	135	68	135	1	0	0	1.1e-14	8.9e-14

-	0.175	-	out							
-	0.18	-	out							
-	0.185	-	out							
-	0.19	-	out							
-	0.195	-	out							
-	0.2	-	out							
-	0.205	-	out							
-	0.21	-	out							
-	0.215	-	out							
-	0.22	-	out							
-	0.225	-	out							
66	0.22656	0.05546	137	69	137	1	0	0	1.2e-14	6.7e-14
-	0.23	-	out							
-	0.235	-	out							
-	0.24	-	out							
-	0.245	-	out							
-	0.25	-	out							
-	0.25	-	out							
-	0.27	-	out							
-	0.29	-	out							
-	0.31	-	out							
67	0.32556	0.099	139	70	139	1	0	0	6e-15	6.9e-14
-	0.33	-	out							
-	0.35	-	out							
-	0.37	-	out							
-	0.39	-	out							
-	0.41	-	out							
68	0.42456	0.099	141	71	141	1	0	0	5.2e-15	4.9e-14
-	0.43	-	out							
-	0.45	-	out							
-	0.47	-	out							
-	0.49	-	out							
-	0.51	-	out							
69	0.52356	0.099	143	72	143	1	0	0	3.5e-15	3.8e-14
-	0.53	-	out							
-	0.55	-	out							
-	0.57	-	out							
-	0.59	-	out							
-	0.61	-	out							
70	0.62256	0.099	145	73	145	1	0	0	1.5e-14	3e-14
-	0.63	-	out							
-	0.65	-	out							
-	0.67	-	out							
-	0.69	-	out							
-	0.71	-	out							
71	0.72156	0.099	146	74	146	1	0	0	5.7e-15	2.5e-14
-	0.73	-	out							
-	0.75	-	out							
-	0.77	-	out							
-	0.79	-	out							
-	0.81	-	out							
72	0.82056	0.099	147	75	147	1	0	0	8.4e-15	2e-14
-	0.83	-	out							
-	0.85	-	out							
-	0.87	-	out							
-	0.89	-	out							

```
   -        0.91           - out
  73      0.91956       0.099      148    76   148      2      0       0  7.5e-15  1.8e-14
   -        0.93           - out
   -        0.95           - out
   -        0.97           - out
   -        0.99           - out
  74       1.0186       0.099      149    77   149      2      0       0  4.2e-15  1.2e-14
Time-stepping completed.
Solution time: 14 s.
Physical memory: 1.36 GB
Virtual memory: 1.34 GB
Ended at May 28, 2023, 4:23:36 PM.
----- Time-Dependent Solver 1 in Oxygen Pulse/Solution 958 (sol958) ----------->
```

k0Sub=1.51E-5, d=6E-6 (su69)

GENERAL

Description	Value
Solution	k0Sub=1.51E-5, d=6E-6

LOG

```
<---- Compile Equations: Stationary in Oxygen Pulse/Solution 958 (sol958) ------
Started at May 28, 2023, 4:23:39 PM.
Parameter k0Sub = 1.51E-5 (m/s).
Parameter d = 6.0E-6 (m).
Geometry shape function: Linear Lagrange
Number of vertex elements: 10
Minimum element quality: 0.432
Time: 0 s.
Physical memory: 1.32 GB
Virtual memory: 1.3 GB
Ended at May 28, 2023, 4:23:39 PM.
----- Compile Equations: Stationary in Oxygen Pulse/Solution 958 (sol958) ----->

<---- Dependent Variables 1 in Oxygen Pulse/Solution 958 (sol958) --------------
Started at May 28, 2023, 4:23:39 PM.
Solution time: 0 s.
Physical memory: 1.33 GB
Virtual memory: 1.31 GB
Ended at May 28, 2023, 4:23:39 PM.
----- Dependent Variables 1 in Oxygen Pulse/Solution 958 (sol958) ------------->

<---- Stationary Solver 1 in Oxygen Pulse/Solution 958 (sol958) ----------------
Started at May 28, 2023, 4:23:39 PM.
Linear solver
Number of degrees of freedom solved for: 18385 (plus 936 internal DOFs).
Symmetric matrices found.
Scales for dependent variables:
Concentration (comp2.Oxi): 0.23
Orthonormal null-space function used.
Iter      SolEst     Damping    Stepsize #Res #Jac #Sol   LinErr   LinRes
   1      0.0069   1.0000000      0.0069    1    1    1  3.1e-13  6.2e-14
Solution time: 1 s.
Physical memory: 1.33 GB
Virtual memory: 1.31 GB
Ended at May 28, 2023, 4:23:40 PM.
----- Stationary Solver 1 in Oxygen Pulse/Solution 958 (sol958) --------------->

<---- Compile Equations: Oxygen Probe Pulse in Oxygen Pulse/Solution 958
      (sol958) ----------------------------------------------------------------
Started at May 28, 2023, 4:23:40 PM.
Geometry shape function: Linear Lagrange
Time: 0 s.
Physical memory: 1.33 GB
Virtual memory: 1.31 GB
Ended at May 28, 2023, 4:23:40 PM.
----- Compile Equations: Oxygen Probe Pulse in Oxygen Pulse/Solution 958
      (sol958) --------------------------------------------------------------->

<---- Dependent Variables 2 in Oxygen Pulse/Solution 958 (sol958) --------------
Started at May 28, 2023, 4:23:40 PM.
Initial values of variables solved for: Solution 958 (sol958), Solution Store 1 (sol
985).
Values of variables not solved for: Solution 958 (sol958), Solution Store 1 (sol985)
.
Solution time: 0 s.
Physical memory: 1.33 GB
```

Virtual memory: 1.31 GB
Ended at May 28, 2023, 4:23:40 PM.
----- Dependent Variables 2 in Oxygen Pulse/Solution 958 (sol958) ------------->

<---- Time-Dependent Solver 1 in Oxygen Pulse/Solution 958 (sol958) ------------
Started at May 28, 2023, 4:23:40 PM.
Time-dependent solver (BDF)
Number of degrees of freedom solved for: 18385 (plus 36840 internal DOFs).
Symmetric matrices found.
Scales for dependent variables:
Concentration (comp2.Oxi): 0.23

Step	Time	Stepsize		Res	Jac	Sol	Order	Tfail	NLfail	LinErr	LinRes
0	0	-	out	5	3	5			0	9.2e-16	1.5e-15
1	2.6536e-08	2.6536e-08		7	4	7	1	0	0	3.5e-16	1e-15
2	5.3072e-08	2.6536e-08		9	5	9	1	0	0	3.7e-16	1.2e-15
3	1.0614e-07	5.3072e-08		11	6	11	2	0	0	4.1e-16	1.3e-15
4	2.1229e-07	1.0614e-07		13	7	13	2	0	0	4.8e-16	1.7e-15
5	4.2458e-07	2.1229e-07		15	8	15	2	0	0	5.7e-16	3.3e-15
6	6.3687e-07	2.1229e-07		17	9	17	2	0	0	6.6e-16	3.7e-15
7	8.4916e-07	2.1229e-07		19	10	19	2	0	0	5.4e-16	4e-15
8	1.0614e-06	2.1229e-07		21	11	21	2	0	0	6.6e-16	3.9e-15
9	1.486e-06	4.2458e-07		23	12	23	2	0	0	1e-15	6.4e-15
10	1.9106e-06	4.2458e-07		25	13	25	2	0	0	9.4e-16	6e-15
11	2.3352e-06	4.2458e-07		27	14	27	2	0	0	9e-16	5.9e-15
12	2.7598e-06	4.2458e-07		29	15	29	2	0	0	9.2e-16	5.5e-15
13	3.6089e-06	8.4916e-07		31	16	31	2	0	0	1.1e-15	8.5e-15
14	4.4581e-06	8.4916e-07		33	17	33	2	0	0	1e-15	7.4e-15
15	5.3072e-06	8.4916e-07		35	18	35	2	0	0	1.4e-15	7.1e-15
16	6.1564e-06	8.4916e-07		37	19	37	2	0	0	9.1e-16	6.6e-15
17	7.8547e-06	1.6983e-06		39	20	39	2	0	0	1.3e-15	9.6e-15
18	9.553e-06	1.6983e-06		41	21	41	2	0	0	1.5e-15	8.3e-15
19	1.1251e-05	1.6983e-06		43	22	43	2	0	0	1.7e-15	8.4e-15
20	1.295e-05	1.6983e-06		45	23	45	2	0	0	1.4e-15	7.4e-15
21	1.4648e-05	1.6983e-06		47	24	47	2	0	0	1.1e-15	6.6e-15
22	1.8045e-05	3.3966e-06		49	25	49	2	0	0	1.3e-15	1e-14
23	2.1441e-05	3.3966e-06		51	26	51	2	0	0	1.5e-15	8.5e-15
24	2.4838e-05	3.3966e-06		53	27	53	2	0	0	1.2e-15	8.3e-15
25	2.8235e-05	3.3966e-06		55	28	55	2	0	0	1.5e-15	7.8e-15
26	3.5028e-05	6.7933e-06		57	29	57	2	0	0	1.6e-15	1.1e-14
27	4.1821e-05	6.7933e-06		59	30	59	2	0	0	1.5e-15	9.9e-15
28	4.8614e-05	6.7933e-06		61	31	61	2	0	0	1.5e-15	9.9e-15
29	5.5408e-05	6.7933e-06		63	32	63	2	0	0	1.5e-15	9e-15
30	6.2201e-05	6.7933e-06		65	33	65	2	0	0	1.6e-15	8.5e-15
31	7.5787e-05	1.3587e-05		67	34	67	2	0	0	2.1e-15	1.2e-14
32	8.9374e-05	1.3587e-05		69	35	69	2	0	0	1.5e-15	1e-14
33	0.00010296	1.3587e-05		71	36	71	2	0	0	1.9e-15	1.1e-14
34	0.00011655	1.3587e-05		73	37	73	2	0	0	2.7e-15	9.7e-15
35	0.00014372	2.7173e-05		75	38	75	2	0	0	1.7e-15	1.4e-14
36	0.00017089	2.7173e-05		77	39	77	2	0	0	1.8e-15	1.3e-14
37	0.00019807	2.7173e-05		79	40	79	2	0	0	1.7e-15	1.2e-14
38	0.00022524	2.7173e-05		81	41	81	2	0	0	1.8e-15	1.2e-14
39	0.00027959	5.4346e-05		83	42	83	2	0	0	1.1e-15	1.6e-14
40	0.00033393	5.4346e-05		85	43	85	2	0	0	2e-15	1.4e-14
41	0.00038828	5.4346e-05		87	44	87	2	0	0	1.6e-15	1.3e-14
42	0.00049697	0.00010869		89	45	89	2	0	0	4.7e-15	2.1e-14
43	0.00060566	0.00010869		91	46	91	2	0	0	3.5e-15	1.7e-14

44	0.00071435	0.00010869		93	47	93	2	0	0	3.5e-15	1.7e-14
45	0.00082305	0.00010869		95	48	95	2	0	0	3.4e-15	1.4e-14
46	0.0010404	0.00021738		97	49	97	2	0	0	1.9e-15	2e-14
47	0.0012578	0.00021738		99	50	99	2	0	0	3.3e-15	1.6e-14
48	0.0016926	0.00043477		101	51	101	2	0	0	4.5e-15	2.5e-14
49	0.0021274	0.00043477		103	52	103	2	0	0	2.9e-15	2.3e-14
50	0.0025621	0.00043477		105	53	105	2	0	0	3.8e-15	2.3e-14
51	0.0029969	0.00043477		107	54	107	2	0	0	2.5e-15	2e-14
52	0.0038664	0.00086954		109	55	109	2	0	0	4.6e-15	2.7e-14
53	0.004736	0.00086954		111	56	111	2	0	0	2.9e-15	2e-14
-	0.005	-	out								
54	0.006475	0.0017391		113	57	113	2	0	0	7.6e-15	3.4e-14
55	0.0082141	0.0017391		115	58	115	2	0	0	3.4e-15	3e-14
-	0.01	-	out								
56	0.011692	0.0034782		117	59	117	2	0	0	4.4e-15	4.6e-14
-	0.015	-	out								
57	0.01517	0.0034782		119	60	119	1	0	0	3e-15	4.8e-14
58	0.018649	0.0034782		121	61	121	1	0	0	5.2e-15	3.8e-14
-	0.02	-	out								
-	0.025	-	out								
59	0.025605	0.0069563		123	62	123	1	0	0	4.2e-15	6.1e-14
-	0.03	-	out								
60	0.032561	0.0069563		125	63	125	1	0	0	8.8e-15	6.5e-14
-	0.035	-	out								
-	0.04	-	out								
-	0.045	-	out								
61	0.046474	0.013913		127	64	127	1	0	0	5.3e-15	9.3e-14
-	0.05	-	out								
-	0.055	-	out								
-	0.06	-	out								
62	0.060386	0.013913		129	65	129	1	0	0	3.3e-15	8e-14
-	0.065	-	out								
-	0.07	-	out								
-	0.075	-	out								
-	0.08	-	out								
-	0.085	-	out								
63	0.088212	0.027825		131	66	131	1	0	0	7e-15	1e-13
-	0.09	-	out								
-	0.095	-	out								
-	0.1	-	out								
-	0.105	-	out								
-	0.11	-	out								
-	0.115	-	out								
64	0.11604	0.027825		133	67	133	1	0	0	4.8e-15	7.7e-14
-	0.12	-	out								
-	0.125	-	out								
-	0.13	-	out								
-	0.135	-	out								
-	0.14	-	out								
-	0.145	-	out								
-	0.15	-	out								
-	0.155	-	out								
-	0.16	-	out								
-	0.165	-	out								
-	0.17	-	out								
65	0.17169	0.05565		135	68	135	1	0	0	1.4e-14	9.3e-14

-	0.175	- out								
-	0.18	- out								
-	0.185	- out								
-	0.19	- out								
-	0.195	- out								
-	0.2	- out								
-	0.205	- out								
-	0.21	- out								
-	0.215	- out								
-	0.22	- out								
-	0.225	- out								
66	0.22734	0.05565	137	69	137	1	0	0	7.4e-15	6.7e-14
-	0.23	- out								
-	0.235	- out								
-	0.24	- out								
-	0.245	- out								
-	0.25	- out								
-	0.25	- out								
-	0.27	- out								
-	0.29	- out								
-	0.31	- out								
67	0.32634	0.099	139	70	139	1	0	0	8e-15	7e-14
-	0.33	- out								
-	0.35	- out								
-	0.37	- out								
-	0.39	- out								
-	0.41	- out								
68	0.42534	0.099	141	71	141	1	0	0	3.7e-15	5.1e-14
-	0.43	- out								
-	0.45	- out								
-	0.47	- out								
-	0.49	- out								
-	0.51	- out								
69	0.52434	0.099	143	72	143	1	0	0	5e-15	4.1e-14
-	0.53	- out								
-	0.55	- out								
-	0.57	- out								
-	0.59	- out								
-	0.61	- out								
70	0.62334	0.099	145	73	145	1	0	0	3.2e-15	3.2e-14
-	0.63	- out								
-	0.65	- out								
-	0.67	- out								
-	0.69	- out								
-	0.71	- out								
71	0.72234	0.099	146	74	146	1	0	0	5.2e-15	2.6e-14
-	0.73	- out								
-	0.75	- out								
-	0.77	- out								
-	0.79	- out								
-	0.81	- out								
72	0.82134	0.099	147	75	147	1	0	0	3.9e-15	2.3e-14
-	0.83	- out								
-	0.85	- out								
-	0.87	- out								
-	0.89	- out								

```
  -        0.91           - out
 73     0.92034       0.099      148   76  148     2     0      0  4.9e-15  1.9e-14
  -        0.93           - out
  -        0.95           - out
  -        0.97           - out
  -        0.99           - out
 74      1.0193       0.099      149   77  149     2     0      0  8.3e-15  1.2e-14
Time-stepping completed.
Solution time: 14 s.
Physical memory: 1.36 GB
Virtual memory: 1.34 GB
Ended at May 28, 2023, 4:23:54 PM.
----- Time-Dependent Solver 1 in Oxygen Pulse/Solution 958 (sol958) ----------->
```

k0Sub=1.51E-5, d=8E-6 (su70)

GENERAL

Description	Value
Solution	k0Sub=1.51E-5, d=8E-6

LOG

```
<---- Compile Equations: Stationary in Oxygen Pulse/Solution 958 (sol958) ------
Started at May 28, 2023, 4:23:57 PM.
Parameter k0Sub = 1.51E-5 (m/s).
Parameter d = 8.0E-6 (m).
Geometry shape function: Linear Lagrange
Number of vertex elements: 10
Minimum element quality: 0.4468
Time: 0 s.
Physical memory: 1.33 GB
Virtual memory: 1.31 GB
Ended at May 28, 2023, 4:23:57 PM.
----- Compile Equations: Stationary in Oxygen Pulse/Solution 958 (sol958) ----->

<---- Dependent Variables 1 in Oxygen Pulse/Solution 958 (sol958) --------------
Started at May 28, 2023, 4:23:57 PM.
Solution time: 0 s.
Physical memory: 1.33 GB
Virtual memory: 1.31 GB
Ended at May 28, 2023, 4:23:57 PM.
----- Dependent Variables 1 in Oxygen Pulse/Solution 958 (sol958) ------------->

<---- Stationary Solver 1 in Oxygen Pulse/Solution 958 (sol958) ----------------
Started at May 28, 2023, 4:23:57 PM.
Linear solver
Number of degrees of freedom solved for: 23800 (plus 941 internal DOFs).
Symmetric matrices found.
Scales for dependent variables:
Concentration (comp2.Oxi): 0.23
Orthonormal null-space function used.
Iter      SolEst     Damping    Stepsize #Res #Jac #Sol   LinErr   LinRes
   1      0.0049   1.0000000      0.0049    1    1    1  2.9e-13    6e-14
Solution time: 1 s.
Physical memory: 1.35 GB
Virtual memory: 1.32 GB
Ended at May 28, 2023, 4:23:58 PM.
----- Stationary Solver 1 in Oxygen Pulse/Solution 958 (sol958) --------------->

<---- Compile Equations: Oxygen Probe Pulse in Oxygen Pulse/Solution 958
      (sol958) ----------------------------------------------------------------
Started at May 28, 2023, 4:23:58 PM.
Geometry shape function: Linear Lagrange
Time: 0 s.
Physical memory: 1.35 GB
Virtual memory: 1.32 GB
Ended at May 28, 2023, 4:23:58 PM.
----- Compile Equations: Oxygen Probe Pulse in Oxygen Pulse/Solution 958
      (sol958) --------------------------------------------------------------->

<---- Dependent Variables 2 in Oxygen Pulse/Solution 958 (sol958) --------------
Started at May 28, 2023, 4:23:58 PM.
Initial values of variables solved for: Solution 958 (sol958), Solution Store 1 (sol
985).
Values of variables not solved for: Solution 958 (sol958), Solution Store 1 (sol985)
.
Solution time: 0 s.
Physical memory: 1.35 GB
```

```
Virtual memory: 1.33 GB
Ended at May 28, 2023, 4:23:58 PM.
----- Dependent Variables 2 in Oxygen Pulse/Solution 958 (sol958) ------------->

<---- Time-Dependent Solver 1 in Oxygen Pulse/Solution 958 (sol958) ------------
Started at May 28, 2023, 4:23:58 PM.
Time-dependent solver (BDF)
Number of degrees of freedom solved for: 23800 (plus 47670 internal DOFs).
Symmetric matrices found.
Scales for dependent variables:
Concentration (comp2.Oxi): 0.23
Step         Time       Stepsize      Res   Jac   Sol Order Tfail NLfail   LinErr   LinRes
   0            0           - out     5     3     5                   0    5e-16  8.9e-16
   1  3.6372e-08  3.6372e-08          7     4     7     1     0       0  3.4e-16  9.8e-16
   2  7.2743e-08  3.6372e-08          9     5     9     1     0       0  3.1e-16  9.9e-16
   3  1.4549e-07  7.2743e-08         11     6    11     2     0       0  3.3e-16  1.1e-15
   4  2.9097e-07  1.4549e-07         13     7    13     2     0       0  3.9e-16  1.3e-15
   5  5.8194e-07  2.9097e-07         15     8    15     2     0       0  5.9e-16  2.4e-15
   6  8.7292e-07  2.9097e-07         17     9    17     2     0       0    5e-16  2.9e-15
   7  1.1639e-06  2.9097e-07         19    10    19     2     0       0  5.3e-16  3.2e-15
   8  1.4549e-06  2.9097e-07         21    11    21     2     0       0  5.3e-16  3.4e-15
   9  2.0368e-06  5.8194e-07         23    12    23     2     0       0  6.4e-16  5.2e-15
  10  2.6187e-06  5.8194e-07         25    13    25     2     0       0  6.2e-16  4.6e-15
  11  3.2007e-06  5.8194e-07         27    14    27     2     0       0  6.1e-16  4.7e-15
  12  3.7826e-06  5.8194e-07         29    15    29     2     0       0  7.4e-16  4.6e-15
  13  4.9465e-06  1.1639e-06         31    16    31     2     0       0  8.5e-16  6.7e-15
  14  6.1104e-06  1.1639e-06         33    17    33     2     0       0  7.4e-16  6.2e-15
  15  7.2743e-06  1.1639e-06         35    18    35     2     0       0  8.5e-16    6e-15
  16  8.4382e-06  1.1639e-06         37    19    37     2     0       0  5.8e-16  5.5e-15
  17  1.0766e-05  2.3278e-06         39    20    39     2     0       0  1.3e-15  8.7e-15
  18  1.3094e-05  2.3278e-06         41    21    41     2     0       0  1.2e-15  7.9e-15
  19  1.5422e-05  2.3278e-06         43    22    43     2     0       0  1.3e-15  7.8e-15
  20  1.7749e-05  2.3278e-06         45    23    45     2     0       0  1.2e-15  7.3e-15
  21  2.2405e-05  4.6556e-06         47    24    47     2     0       0    1e-15    1e-14
  22   2.706e-05  4.6556e-06         49    25    49     2     0       0  9.4e-16  9.6e-15
  23  3.1716e-05  4.6556e-06         51    26    51     2     0       0  1.2e-15  9.6e-15
  24  3.6372e-05  4.6556e-06         53    27    53     2     0       0  1.3e-15    9e-15
  25  4.5683e-05  9.3111e-06         55    28    55     2     0       0  2.1e-15  1.3e-14
  26  5.4994e-05  9.3111e-06         57    29    57     2     0       0  1.5e-15  1.1e-14
  27  6.4305e-05  9.3111e-06         59    30    59     2     0       0  1.7e-15  1.1e-14
  28  7.3616e-05  9.3111e-06         61    31    61     2     0       0  1.5e-15  1.1e-14
  29  9.2238e-05  1.8622e-05         63    32    63     2     0       0  2.2e-15  1.6e-14
  30  0.00011086  1.8622e-05         65    33    65     2     0       0  1.5e-15  1.4e-14
  31  0.00012948  1.8622e-05         67    34    67     2     0       0  1.3e-15  1.3e-14
  32   0.0001481  1.8622e-05         69    35    69     2     0       0  9.4e-16  1.2e-14
  33  0.00018535  3.7244e-05         71    36    71     2     0       0  2.1e-15  1.8e-14
  34  0.00022259  3.7244e-05         73    37    73     2     0       0  1.8e-15  1.5e-14
  35  0.00025984  3.7244e-05         75    38    75     2     0       0    3e-15  1.5e-14
  36  0.00029708  3.7244e-05         77    39    77     2     0       0  1.6e-15  1.4e-14
  37  0.00037157  7.4489e-05         79    40    79     2     0       0    4e-15    2e-14
  38  0.00044606  7.4489e-05         81    41    81     2     0       0    2e-15  1.7e-14
  39  0.00059504  0.00014898         83    42    83     2     0       0  2.5e-15  2.7e-14
  40  0.00074402  0.00014898         85    43    85     2     0       0  2.2e-15  2.4e-14
  41  0.00089299  0.00014898         87    44    87     2     0       0  1.8e-15  2.3e-14
  42    0.001042  0.00014898         89    45    89     2     0       0  2.2e-15    2e-14
  43   0.0013399  0.00029796         91    46    91     2     0       0  2.5e-15  2.6e-14
```

44	0.0016379	0.00029796		93	47	93	2	0	0	3.7e-15	1.9e-14
45	0.0022338	0.00059591		95	48	95	2	0	0	4.2e-15	3.2e-14
46	0.0028297	0.00059591		97	49	97	2	0	0	3.3e-15	2.7e-14
47	0.0034256	0.00059591		99	50	99	2	0	0	6.3e-15	2.9e-14
48	0.0046174	0.0011918		101	51	101	2	0	0	1.1e-14	3.6e-14
-	0.005	-	out								
49	0.0058093	0.0011918		103	52	103	2	0	0	2.3e-15	2.7e-14
50	0.0081929	0.0023836		105	53	105	2	0	0	5.5e-15	4.5e-14
-	0.01	-	out								
51	0.010577	0.0023836		107	54	107	1	0	0	2e-15	4.9e-14
52	0.01296	0.0023836		109	55	109	1	0	0	3e-15	3.2e-14
-	0.015	-	out								
53	0.017727	0.0047673		111	56	111	1	0	0	5.8e-15	5.4e-14
-	0.02	-	out								
-	0.025	-	out								
54	0.027262	0.0095346		113	57	113	1	0	0	2.4e-15	8.5e-14
-	0.03	-	out								
-	0.035	-	out								
55	0.036797	0.0095346		115	58	115	1	0	0	3.4e-15	8.5e-14
-	0.04	-	out								
-	0.045	-	out								
-	0.05	-	out								
-	0.055	-	out								
56	0.055866	0.019069		117	59	117	1	0	0	3.6e-15	1.2e-13
-	0.06	-	out								
-	0.065	-	out								
-	0.07	-	out								
57	0.074935	0.019069		119	60	119	1	0	0	5.8e-15	1.1e-13
-	0.075	-	out								
-	0.08	-	out								
-	0.085	-	out								
-	0.09	-	out								
-	0.095	-	out								
-	0.1	-	out								
-	0.105	-	out								
-	0.11	-	out								
58	0.11307	0.038138		121	61	121	1	0	0	7.7e-15	1.3e-13
-	0.115	-	out								
-	0.12	-	out								
-	0.125	-	out								
-	0.13	-	out								
-	0.135	-	out								
-	0.14	-	out								
-	0.145	-	out								
-	0.15	-	out								
59	0.15121	0.038138		123	62	123	1	0	0	9.5e-15	1.1e-13
-	0.155	-	out								
-	0.16	-	out								
-	0.165	-	out								
-	0.17	-	out								
-	0.175	-	out								
-	0.18	-	out								
-	0.185	-	out								
-	0.19	-	out								
-	0.195	-	out								
-	0.2	-	out								

-	0.205	- out								
-	0.21	- out								
-	0.215	- out								
-	0.22	- out								
-	0.225	- out								
60	0.22749	0.076277	125	63	125	1	0	0	1.2e-14	1.2e-13
-	0.23	- out								
-	0.235	- out								
-	0.24	- out								
-	0.245	- out								
-	0.25	- out								
-	0.25	- out								
-	0.27	- out								
-	0.29	- out								
61	0.30376	0.076277	127	64	127	1	0	0	3.9e-15	9.2e-14
-	0.31	- out								
-	0.33	- out								
-	0.35	- out								
-	0.37	- out								
-	0.39	- out								
62	0.40276	0.099	129	65	129	1	0	0	3.9e-15	8.4e-14
-	0.41	- out								
-	0.43	- out								
-	0.45	- out								
-	0.47	- out								
-	0.49	- out								
63	0.50176	0.099	131	66	131	1	0	0	6.7e-15	6.3e-14
-	0.51	- out								
-	0.53	- out								
-	0.55	- out								
-	0.57	- out								
-	0.59	- out								
64	0.60076	0.099	133	67	133	1	0	0	5.7e-15	4.9e-14
-	0.61	- out								
-	0.63	- out								
-	0.65	- out								
-	0.67	- out								
-	0.69	- out								
65	0.69976	0.099	134	68	134	1	0	0	8.3e-15	4e-14
-	0.71	- out								
-	0.73	- out								
-	0.75	- out								
-	0.77	- out								
-	0.79	- out								
66	0.79876	0.099	135	69	135	1	0	0	4.1e-15	3.6e-14
-	0.81	- out								
-	0.83	- out								
-	0.85	- out								
-	0.87	- out								
-	0.89	- out								
67	0.89776	0.099	136	70	136	2	0	0	3.4e-15	2.8e-14
-	0.91	- out								
-	0.93	- out								
-	0.95	- out								
-	0.97	- out								
-	0.99	- out								

```
  68      0.99676        0.099      137    71   137      2      0       0  5.3e-15   1.9e-14
Time-stepping completed.
Solution time: 16 s.
Physical memory: 1.37 GB
Virtual memory: 1.36 GB
Ended at May 28, 2023, 4:24:14 PM.
----- Time-Dependent Solver 1 in Oxygen Pulse/Solution 958 (sol958) ----------->
```

k0Sub=1.51E-5, d=1.2E-5 (su71)

GENERAL

Description	Value
Solution	k0Sub=1.51E-5, d=1.2E-5

LOG

```
<---- Compile Equations: Stationary in Oxygen Pulse/Solution 958 (sol958) ------
Started at May 28, 2023, 4:24:18 PM.
Parameter k0Sub = 1.51E-5 (m/s).
Parameter d = 1.2E-5 (m).
Geometry shape function: Linear Lagrange
Number of vertex elements: 10
Minimum element quality: 0.4505
Time: 1 s.
Physical memory: 1.34 GB
Virtual memory: 1.32 GB
Ended at May 28, 2023, 4:24:18 PM.
----- Compile Equations: Stationary in Oxygen Pulse/Solution 958 (sol958) ----->

<---- Dependent Variables 1 in Oxygen Pulse/Solution 958 (sol958) --------------
Started at May 28, 2023, 4:24:18 PM.
Solution time: 0 s.
Physical memory: 1.33 GB
Virtual memory: 1.32 GB
Ended at May 28, 2023, 4:24:18 PM.
----- Dependent Variables 1 in Oxygen Pulse/Solution 958 (sol958) ------------->

<---- Stationary Solver 1 in Oxygen Pulse/Solution 958 (sol958) ----------------
Started at May 28, 2023, 4:24:18 PM.
Linear solver
Number of degrees of freedom solved for: 18769 (plus 948 internal DOFs).
Symmetric matrices found.
Scales for dependent variables:
Concentration (comp2.Oxi): 0.23
Orthonormal null-space function used.
Iter      SolEst     Damping    Stepsize #Res #Jac #Sol   LinErr   LinRes
   1      0.0042   1.0000000      0.0042    1    1    1  2.5e-13  4.8e-14
Solution time: 1 s.
Physical memory: 1.34 GB
Virtual memory: 1.32 GB
Ended at May 28, 2023, 4:24:19 PM.
----- Stationary Solver 1 in Oxygen Pulse/Solution 958 (sol958) --------------->

<---- Compile Equations: Oxygen Probe Pulse in Oxygen Pulse/Solution 958
      (sol958) ----------------------------------------------------------------
Started at May 28, 2023, 4:24:19 PM.
Geometry shape function: Linear Lagrange
Time: 0 s.
Physical memory: 1.35 GB
Virtual memory: 1.33 GB
Ended at May 28, 2023, 4:24:20 PM.
----- Compile Equations: Oxygen Probe Pulse in Oxygen Pulse/Solution 958
      (sol958) --------------------------------------------------------------->

<---- Dependent Variables 2 in Oxygen Pulse/Solution 958 (sol958) --------------
Started at May 28, 2023, 4:24:20 PM.
Initial values of variables solved for: Solution 958 (sol958), Solution Store 1 (sol
985).
Values of variables not solved for: Solution 958 (sol958), Solution Store 1 (sol985)
.
Solution time: 0 s.
Physical memory: 1.35 GB
```

```
Virtual memory: 1.33 GB
Ended at May 28, 2023, 4:24:20 PM.
----- Dependent Variables 2 in Oxygen Pulse/Solution 958 (sol958) ------------->

<---- Time-Dependent Solver 1 in Oxygen Pulse/Solution 958 (sol958) ------------
Started at May 28, 2023, 4:24:20 PM.
Time-dependent solver (BDF)
Number of degrees of freedom solved for: 18769 (plus 37608 internal DOFs).
Symmetric matrices found.
Scales for dependent variables:
Concentration (comp2.Oxi): 0.23
Step        Time    Stepsize      Res  Jac  Sol Order Tfail NLfail   LinErr   LinRes
   0           0           - out    5    3    5                   0    1e-15  1.4e-15
   1  2.6644e-08  2.6644e-08        7    4    7     1     0       0  3.5e-16    1e-15
   2  5.3289e-08  2.6644e-08        9    5    9     1     0       0  3.6e-16  1.2e-15
   3  1.0658e-07  5.3289e-08       11    6   11     2     0       0  3.6e-16  1.3e-15
   4  2.1315e-07  1.0658e-07       13    7   13     2     0       0  5.1e-16  1.7e-15
   5  4.2631e-07  2.1315e-07       15    8   15     2     0       0  6.2e-16  3.3e-15
   6  6.3946e-07  2.1315e-07       17    9   17     2     0       0  7.5e-16  3.9e-15
   7  8.5262e-07  2.1315e-07       19   10   19     2     0       0  5.8e-16    4e-15
   8  1.0658e-06  2.1315e-07       21   11   21     2     0       0  7.1e-16  3.9e-15
   9  1.4921e-06  4.2631e-07       23   12   23     2     0       0  1.1e-15  6.4e-15
  10  1.9184e-06  4.2631e-07       25   13   25     2     0       0    1e-15    6e-15
  11  2.3447e-06  4.2631e-07       27   14   27     2     0       0  8.2e-16  5.7e-15
  12   2.771e-06  4.2631e-07       29   15   29     2     0       0    8e-16  5.7e-15
  13  3.6236e-06  8.5262e-07       31   16   31     2     0       0  9.1e-16  8.4e-15
  14  4.4762e-06  8.5262e-07       33   17   33     2     0       0    1e-15  7.2e-15
  15  5.3289e-06  8.5262e-07       35   18   35     2     0       0  1.1e-15  7.1e-15
  16  6.1815e-06  8.5262e-07       37   19   37     2     0       0  9.6e-16  6.3e-15
  17  7.8867e-06  1.7052e-06       39   20   39     2     0       0  1.8e-15  9.7e-15
  18   9.592e-06  1.7052e-06       41   21   41     2     0       0  1.3e-15  8.2e-15
  19  1.1297e-05  1.7052e-06       43   22   43     2     0       0  1.2e-15  7.8e-15
  20  1.3002e-05  1.7052e-06       45   23   45     2     0       0  1.2e-15  7.3e-15
  21  1.4708e-05  1.7052e-06       47   24   47     2     0       0  1.1e-15  6.5e-15
  22  1.8118e-05  3.4105e-06       49   25   49     2     0       0  1.5e-15  9.5e-15
  23  2.1529e-05  3.4105e-06       51   26   51     2     0       0  1.8e-15  8.6e-15
  24  2.4939e-05  3.4105e-06       53   27   53     2     0       0  1.3e-15  8.5e-15
  25   2.835e-05  3.4105e-06       55   28   55     2     0       0  1.5e-15  7.5e-15
  26  3.5171e-05   6.821e-06       57   29   57     2     0       0  2.5e-15  1.1e-14
  27  4.1991e-05   6.821e-06       59   30   59     2     0       0  1.6e-15  9.7e-15
  28  4.8812e-05   6.821e-06       61   31   61     2     0       0  2.2e-15  9.6e-15
  29  5.5633e-05   6.821e-06       63   32   63     2     0       0  1.9e-15  8.7e-15
  30  6.9275e-05  1.3642e-05       65   33   65     2     0       0  1.7e-15  1.3e-14
  31  8.2917e-05  1.3642e-05       67   34   67     2     0       0  2.8e-15  1.1e-14
  32  9.6559e-05  1.3642e-05       69   35   69     2     0       0  2.2e-15  1.1e-14
  33   0.0001102  1.3642e-05       71   36   71     2     0       0  2.5e-15    1e-14
  34  0.00013748  2.7284e-05       73   37   73     2     0       0  2.3e-15  1.5e-14
  35  0.00016477  2.7284e-05       75   38   75     2     0       0  1.8e-15  1.3e-14
  36  0.00019205  2.7284e-05       77   39   77     2     0       0  2.3e-15  1.2e-14
  37  0.00021934  2.7284e-05       79   40   79     2     0       0    2e-15  1.1e-14
  38   0.0002739  5.4568e-05       81   41   81     2     0       0  2.3e-15  1.7e-14
  39  0.00032847  5.4568e-05       83   42   83     2     0       0  1.6e-15  1.4e-14
  40  0.00038304  5.4568e-05       85   43   85     2     0       0    3e-15  1.4e-14
  41  0.00049217  0.00010914       87   44   87     2     0       0  2.1e-15    2e-14
  42  0.00060131  0.00010914       89   45   89     2     0       0  3.7e-15  1.6e-14
  43  0.00071044  0.00010914       91   46   91     2     0       0  2.1e-15  1.7e-14
```

44	0.00081958	0.00010914		93	47	93	2	0	0	1.5e-15	1.5e-14
45	0.0010379	0.00021827		95	48	95	2	0	0	5.9e-15	2.1e-14
46	0.0012561	0.00021827		97	49	97	2	0	0	2.5e-15	1.6e-14
47	0.0016927	0.00043654		99	50	99	2	0	0	3.9e-15	2.7e-14
48	0.0021292	0.00043654		101	51	101	2	0	0	2.5e-15	2.3e-14
49	0.0025657	0.00043654		103	52	103	2	0	0	3.6e-15	2.3e-14
50	0.0030023	0.00043654		105	53	105	2	0	0	4.1e-15	2e-14
51	0.0038754	0.00087308		107	54	107	2	0	0	3.7e-15	2.4e-14
52	0.0047484	0.00087308		109	55	109	2	0	0	1.4e-15	1.9e-14
-	0.005	-	out								
53	0.0064946	0.0017462		111	56	111	2	0	0	7.5e-15	2.9e-14
54	0.0082408	0.0017462		113	57	113	2	0	0	2.5e-15	2.6e-14
-	0.01	-	out								
55	0.011733	0.0034923		115	58	115	2	0	0	1.8e-15	4.1e-14
-	0.015	-	out								
56	0.015225	0.0034923		117	59	117	1	0	0	5.8e-15	4.3e-14
57	0.018718	0.0034923		119	60	119	1	0	0	3e-15	2.5e-14
-	0.02	-	out								
-	0.025	-	out								
58	0.025702	0.0069847		121	61	121	1	0	0	3e-15	4e-14
-	0.03	-	out								
-	0.035	-	out								
59	0.039672	0.013969		123	62	123	1	0	0	3.4e-15	6e-14
-	0.04	-	out								
-	0.045	-	out								
-	0.05	-	out								
60	0.053641	0.013969		125	63	125	1	0	0	7.2e-15	6.1e-14
-	0.055	-	out								
-	0.06	-	out								
-	0.065	-	out								
-	0.07	-	out								
-	0.075	-	out								
-	0.08	-	out								
61	0.08158	0.027939		127	64	127	1	0	0	3e-15	8.4e-14
-	0.085	-	out								
-	0.09	-	out								
-	0.095	-	out								
-	0.1	-	out								
-	0.105	-	out								
62	0.10952	0.027939		129	65	129	1	0	0	6.3e-15	7.5e-14
-	0.11	-	out								
-	0.115	-	out								
-	0.12	-	out								
-	0.125	-	out								
-	0.13	-	out								
-	0.135	-	out								
-	0.14	-	out								
-	0.145	-	out								
-	0.15	-	out								
-	0.155	-	out								
-	0.16	-	out								
-	0.165	-	out								
63	0.1654	0.055877		131	66	131	1	0	0	2.8e-15	9.4e-14
-	0.17	-	out								
-	0.175	-	out								
-	0.18	-	out								

-	0.185	-	out							
-	0.19	-	out							
-	0.195	-	out							
-	0.2	-	out							
-	0.205	-	out							
-	0.21	-	out							
-	0.215	-	out							
-	0.22	-	out							
64	0.22127	0.055877		133	67	133	1	0	0	1e-14	7.5e-14
-	0.225	-	out							
-	0.23	-	out							
-	0.235	-	out							
-	0.24	-	out							
-	0.245	-	out							
-	0.25	-	out							
-	0.25	-	out							
-	0.27	-	out							
-	0.29	-	out							
-	0.31	-	out							
65	0.32027	0.099		135	68	135	1	0	0	5.7e-15	8e-14
-	0.33	-	out							
-	0.35	-	out							
-	0.37	-	out							
-	0.39	-	out							
-	0.41	-	out							
66	0.41927	0.099		137	69	137	1	0	0	5.5e-15	6.1e-14
-	0.43	-	out							
-	0.45	-	out							
-	0.47	-	out							
-	0.49	-	out							
-	0.51	-	out							
67	0.51827	0.099		139	70	139	1	0	0	4.3e-15	4.6e-14
-	0.53	-	out							
-	0.55	-	out							
-	0.57	-	out							
-	0.59	-	out							
-	0.61	-	out							
68	0.61727	0.099		141	71	141	1	0	0	4.9e-15	3.6e-14
-	0.63	-	out							
-	0.65	-	out							
-	0.67	-	out							
-	0.69	-	out							
-	0.71	-	out							
69	0.71627	0.099		142	72	142	1	0	0	6.7e-15	3.1e-14
-	0.73	-	out							
-	0.75	-	out							
-	0.77	-	out							
-	0.79	-	out							
-	0.81	-	out							
70	0.81527	0.099		143	73	143	1	0	0	3.1e-15	2.7e-14
-	0.83	-	out							
-	0.85	-	out							
-	0.87	-	out							
-	0.89	-	out							
-	0.91	-	out							
71	0.91427	0.099		144	74	144	2	0	0	7.6e-15	2.2e-14

```
   -        0.93          - out
   -        0.95          - out
   -        0.97          - out
   -        0.99          - out
  72      1.0133       0.099      145   75  145      2      0       0  8.9e-15  1.4e-14
Time-stepping completed.
Solution time: 14 s.
Physical memory: 1.38 GB
Virtual memory: 1.36 GB
Ended at May 28, 2023, 4:24:34 PM.
----- Time-Dependent Solver 1 in Oxygen Pulse/Solution 958 (sol958) ----------->
```

k0Sub=1.51E-5, d=1.5E-5 (su72)

GENERAL

Description	Value
Solution	k0Sub=1.51E-5, d=1.5E-5

LOG

```
<---- Compile Equations: Stationary in Oxygen Pulse/Solution 958 (sol958) ------
Started at May 28, 2023, 4:24:36 PM.
Parameter k0Sub = 1.51E-5 (m/s).
Parameter d = 1.4999999999999999E-5 (m).
Geometry shape function: Linear Lagrange
Number of vertex elements: 10
Minimum element quality: 0.4444
Time: 1 s.
Physical memory: 1.34 GB
Virtual memory: 1.33 GB
Ended at May 28, 2023, 4:24:37 PM.
----- Compile Equations: Stationary in Oxygen Pulse/Solution 958 (sol958) ----->

<---- Dependent Variables 1 in Oxygen Pulse/Solution 958 (sol958) --------------
Started at May 28, 2023, 4:24:37 PM.
Solution time: 0 s.
Physical memory: 1.34 GB
Virtual memory: 1.33 GB
Ended at May 28, 2023, 4:24:37 PM.
----- Dependent Variables 1 in Oxygen Pulse/Solution 958 (sol958) ------------->

<---- Stationary Solver 1 in Oxygen Pulse/Solution 958 (sol958) ----------------
Started at May 28, 2023, 4:24:37 PM.
Linear solver
Number of degrees of freedom solved for: 18918 (plus 952 internal DOFs).
Symmetric matrices found.
Scales for dependent variables:
Concentration (comp2.Oxi): 0.23
Orthonormal null-space function used.
Iter      SolEst     Damping    Stepsize #Res #Jac #Sol   LinErr   LinRes
   1      0.0037   1.0000000      0.0037    1    1    1  2.3e-13  4.5e-14
Solution time: 1 s.
Physical memory: 1.35 GB
Virtual memory: 1.33 GB
Ended at May 28, 2023, 4:24:37 PM.
----- Stationary Solver 1 in Oxygen Pulse/Solution 958 (sol958) --------------->

<---- Compile Equations: Oxygen Probe Pulse in Oxygen Pulse/Solution 958
      (sol958) ----------------------------------------------------------------
Started at May 28, 2023, 4:24:37 PM.
Geometry shape function: Linear Lagrange
Time: 0 s.
Physical memory: 1.35 GB
Virtual memory: 1.33 GB
Ended at May 28, 2023, 4:24:38 PM.
----- Compile Equations: Oxygen Probe Pulse in Oxygen Pulse/Solution 958
      (sol958) --------------------------------------------------------------->

<---- Dependent Variables 2 in Oxygen Pulse/Solution 958 (sol958) --------------
Started at May 28, 2023, 4:24:38 PM.
Initial values of variables solved for: Solution 958 (sol958), Solution Store 1 (sol
985).
Values of variables not solved for: Solution 958 (sol958), Solution Store 1 (sol985)
.
Solution time: 0 s.
Physical memory: 1.35 GB
```

Virtual memory: 1.33 GB
Ended at May 28, 2023, 4:24:38 PM.
----- Dependent Variables 2 in Oxygen Pulse/Solution 958 (sol958) ------------->

<---- Time-Dependent Solver 1 in Oxygen Pulse/Solution 958 (sol958) ------------
Started at May 28, 2023, 4:24:38 PM.
Time-dependent solver (BDF)
Number of degrees of freedom solved for: 18918 (plus 37906 internal DOFs).
Symmetric matrices found.
Scales for dependent variables:
Concentration (comp2.Oxi): 0.23

Step	Time	Stepsize	Res	Jac	Sol	Order	Tfail	NLfail	LinErr	LinRes
0	0	- out	5	3	5			0	8.1e-16	1.4e-15
1	2.6822e-08	2.6822e-08	7	4	7	1	0	0	3.6e-16	1.1e-15
2	5.3645e-08	2.6822e-08	9	5	9	1	0	0	3.5e-16	1.2e-15
3	1.0729e-07	5.3645e-08	11	6	11	2	0	0	3.5e-16	1.3e-15
4	2.1458e-07	1.0729e-07	13	7	13	2	0	0	4.6e-16	1.8e-15
5	4.2916e-07	2.1458e-07	15	8	15	2	0	0	6.8e-16	3.3e-15
6	6.4374e-07	2.1458e-07	17	9	17	2	0	0	7.7e-16	3.8e-15
7	8.5832e-07	2.1458e-07	19	10	19	2	0	0	5.7e-16	3.9e-15
8	1.0729e-06	2.1458e-07	21	11	21	2	0	0	7.4e-16	4e-15
9	1.5021e-06	4.2916e-07	23	12	23	2	0	0	9.3e-16	6e-15
10	1.9312e-06	4.2916e-07	25	13	25	2	0	0	9.2e-16	6e-15
11	2.3604e-06	4.2916e-07	27	14	27	2	0	0	8.9e-16	6.1e-15
12	2.7895e-06	4.2916e-07	29	15	29	2	0	0	8.5e-16	5.8e-15
13	3.6478e-06	8.5832e-07	31	16	31	2	0	0	1.2e-15	8.7e-15
14	4.5062e-06	8.5832e-07	33	17	33	2	0	0	8.8e-16	7.5e-15
15	5.3645e-06	8.5832e-07	35	18	35	2	0	0	9.9e-16	7.4e-15
16	6.2228e-06	8.5832e-07	37	19	37	2	0	0	1.3e-15	6.6e-15
17	7.9394e-06	1.7166e-06	39	20	39	2	0	0	1.3e-15	1e-14
18	9.6561e-06	1.7166e-06	41	21	41	2	0	0	1.2e-15	8.4e-15
19	1.1373e-05	1.7166e-06	43	22	43	2	0	0	1.6e-15	8.3e-15
20	1.3089e-05	1.7166e-06	45	23	45	2	0	0	1.5e-15	7.5e-15
21	1.4806e-05	1.7166e-06	47	24	47	2	0	0	1.9e-15	6.5e-15
22	1.8239e-05	3.4333e-06	49	25	49	2	0	0	1.4e-15	1e-14
23	2.1672e-05	3.4333e-06	51	26	51	2	0	0	1.6e-15	8.4e-15
24	2.5106e-05	3.4333e-06	53	27	53	2	0	0	2e-15	8.8e-15
25	2.8539e-05	3.4333e-06	55	28	55	2	0	0	1.3e-15	7.8e-15
26	3.5406e-05	6.8665e-06	57	29	57	2	0	0	1.7e-15	1.2e-14
27	4.2272e-05	6.8665e-06	59	30	59	2	0	0	2.2e-15	9.9e-15
28	4.9139e-05	6.8665e-06	61	31	61	2	0	0	1.9e-15	9.9e-15
29	5.6005e-05	6.8665e-06	63	32	63	2	0	0	2.4e-15	9e-15
30	6.2872e-05	6.8665e-06	65	33	65	2	0	0	1.5e-15	7.9e-15
31	7.6605e-05	1.3733e-05	67	34	67	2	0	0	1.7e-15	1.2e-14
32	9.0338e-05	1.3733e-05	69	35	69	2	0	0	2.1e-15	1.1e-14
33	0.00010407	1.3733e-05	71	36	71	2	0	0	1.8e-15	1.1e-14
34	0.0001178	1.3733e-05	73	37	73	2	0	0	1.9e-15	9.7e-15
35	0.00014527	2.7466e-05	75	38	75	2	0	0	2e-15	1.4e-14
36	0.00017274	2.7466e-05	77	39	77	2	0	0	2.1e-15	1.2e-14
37	0.0002002	2.7466e-05	79	40	79	2	0	0	2.6e-15	1.2e-14
38	0.00022767	2.7466e-05	81	41	81	2	0	0	2.1e-15	1.1e-14
39	0.0002826	5.4932e-05	83	42	83	2	0	0	5.5e-15	1.6e-14
40	0.00033753	5.4932e-05	85	43	85	2	0	0	4.6e-15	1.3e-14
41	0.00039247	5.4932e-05	87	44	87	2	0	0	4.5e-15	1.4e-14
42	0.00050233	0.00010986	89	45	89	2	0	0	2.3e-15	2e-14
43	0.00061219	0.00010986	91	46	91	2	0	0	3.3e-15	1.7e-14

44	0.00072206	0.00010986	93	47	93	2	0	0	2.7e-15	1.7e-14
45	0.00083192	0.00010986	95	48	95	2	0	0	3.2e-15	1.4e-14
46	0.0010517	0.00021973	97	49	97	2	0	0	8e-15	2.1e-14
47	0.0012714	0.00021973	99	50	99	2	0	0	4.8e-15	1.7e-14
48	0.0017108	0.00043946	101	51	101	2	0	0	4.9e-15	2.5e-14
49	0.0021503	0.00043946	103	52	103	2	0	0	4.6e-15	2.3e-14
50	0.0025898	0.00043946	105	53	105	2	0	0	6.4e-15	2.2e-14
51	0.0030292	0.00043946	107	54	107	2	0	0	1.6e-15	1.8e-14
52	0.0039081	0.00087892	109	55	109	2	0	0	2.7e-15	2.4e-14
53	0.004787	0.00087892	111	56	111	2	0	0	4.6e-15	1.8e-14
-	0.005	- out								
54	0.0065449	0.0017578	113	57	113	2	0	0	3.9e-15	3e-14
55	0.0083027	0.0017578	115	58	115	2	0	0	1.4e-15	2.5e-14
-	0.01	- out								
56	0.011818	0.0035157	117	59	117	2	0	0	7.6e-15	3.8e-14
-	0.015	- out								
57	0.015334	0.0035157	119	60	119	1	0	0	7.5e-15	4.1e-14
58	0.01885	0.0035157	121	61	121	1	0	0	6.6e-15	2.4e-14
-	0.02	- out								
-	0.025	- out								
59	0.025881	0.0070313	123	62	123	1	0	0	1e-14	3.7e-14
-	0.03	- out								
-	0.035	- out								
60	0.039944	0.014063	125	63	125	1	0	0	4.1e-15	5.3e-14
-	0.04	- out								
-	0.045	- out								
-	0.05	- out								
61	0.054006	0.014063	127	64	127	1	0	0	6.4e-15	5e-14
-	0.055	- out								
-	0.06	- out								
-	0.065	- out								
-	0.07	- out								
-	0.075	- out								
-	0.08	- out								
62	0.082132	0.028125	129	65	129	1	0	0	4e-15	7.2e-14
-	0.085	- out								
-	0.09	- out								
-	0.095	- out								
-	0.1	- out								
-	0.105	- out								
-	0.11	- out								
63	0.11026	0.028125	131	66	131	1	0	0	6.2e-15	6.5e-14
-	0.115	- out								
-	0.12	- out								
-	0.125	- out								
-	0.13	- out								
-	0.135	- out								
-	0.14	- out								
-	0.145	- out								
-	0.15	- out								
-	0.155	- out								
-	0.16	- out								
-	0.165	- out								
64	0.16651	0.056251	133	67	133	1	0	0	2.2e-15	8.4e-14
-	0.17	- out								
-	0.175	- out								

-	0.18	- out								
-	0.185	- out								
-	0.19	- out								
-	0.195	- out								
-	0.2	- out								
-	0.205	- out								
-	0.21	- out								
-	0.215	- out								
-	0.22	- out								
65	0.22276	0.056251	135	68	135	1	0	0	6.3e-15	7.1e-14
-	0.225	- out								
-	0.23	- out								
-	0.235	- out								
-	0.24	- out								
-	0.245	- out								
-	0.25	- out								
-	0.25	- out								
-	0.27	- out								
-	0.29	- out								
-	0.31	- out								
66	0.32176	0.099	137	69	137	1	0	0	8.4e-15	7.6e-14
-	0.33	- out								
-	0.35	- out								
-	0.37	- out								
-	0.39	- out								
-	0.41	- out								
67	0.42076	0.099	139	70	139	1	0	0	1.1e-14	6e-14
-	0.43	- out								
-	0.45	- out								
-	0.47	- out								
-	0.49	- out								
-	0.51	- out								
68	0.51976	0.099	141	71	141	1	0	0	1e-14	4.9e-14
-	0.53	- out								
-	0.55	- out								
-	0.57	- out								
-	0.59	- out								
-	0.61	- out								
69	0.61876	0.099	143	72	143	1	0	0	9.9e-15	3.8e-14
-	0.63	- out								
-	0.65	- out								
-	0.67	- out								
-	0.69	- out								
-	0.71	- out								
70	0.71776	0.099	144	73	144	1	0	0	6e-15	3.2e-14
-	0.73	- out								
-	0.75	- out								
-	0.77	- out								
-	0.79	- out								
-	0.81	- out								
71	0.81676	0.099	145	74	145	1	0	0	6.4e-15	2.7e-14
-	0.83	- out								
-	0.85	- out								
-	0.87	- out								
-	0.89	- out								
-	0.91	- out								

```
  72     0.91576       0.099      146   75  146     2     0      0  3.3e-15  2.3e-14
   -        0.93           - out
   -        0.95           - out
   -        0.97           - out
   -        0.99           - out
  73      1.0148       0.099      147   76  147     2     0      0  5.5e-15  1.4e-14
Time-stepping completed.
Solution time: 14 s.
Physical memory: 1.37 GB
Virtual memory: 1.36 GB
Ended at May 28, 2023, 4:24:52 PM.
----- Time-Dependent Solver 1 in Oxygen Pulse/Solution 958 (sol958) ----------->
```

k0Sub=1.51E-4, d=5E-7 (su73)

GENERAL

Description	Value
Solution	k0Sub=1.51E-4, d=5E-7

LOG

```
<---- Compile Equations: Stationary in Oxygen Pulse/Solution 958 (sol958) ------
Started at May 28, 2023, 4:24:55 PM.
Parameter k0Sub = 1.51E-4 (m/s).
Parameter d = 5.0E-7 (m).
Geometry shape function: Linear Lagrange
Number of vertex elements: 10
Minimum element quality: 0.3539
Time: 1 s.
Physical memory: 1.28 GB
Virtual memory: 1.26 GB
Ended at May 28, 2023, 4:24:55 PM.
----- Compile Equations: Stationary in Oxygen Pulse/Solution 958 (sol958) ----->

<---- Dependent Variables 1 in Oxygen Pulse/Solution 958 (sol958) --------------
Started at May 28, 2023, 4:24:55 PM.
Solution time: 0 s.
Physical memory: 1.29 GB
Virtual memory: 1.26 GB
Ended at May 28, 2023, 4:24:55 PM.
----- Dependent Variables 1 in Oxygen Pulse/Solution 958 (sol958) ------------->

<---- Stationary Solver 1 in Oxygen Pulse/Solution 958 (sol958) ----------------
Started at May 28, 2023, 4:24:55 PM.
Linear solver
Number of degrees of freedom solved for: 17710 (plus 1007 internal DOFs).
Symmetric matrices found.
Scales for dependent variables:
Concentration (comp2.Oxi): 0.23
Orthonormal null-space function used.
Iter      SolEst     Damping    Stepsize #Res #Jac #Sol   LinErr   LinRes
   1        0.21   1.0000000        0.21    1    1    1  4.6e-13  1.1e-13
Solution time: 1 s.
Physical memory: 1.32 GB
Virtual memory: 1.29 GB
Ended at May 28, 2023, 4:24:56 PM.
----- Stationary Solver 1 in Oxygen Pulse/Solution 958 (sol958) --------------->

<---- Compile Equations: Oxygen Probe Pulse in Oxygen Pulse/Solution 958
      (sol958) -----------------------------------------------------------------
Started at May 28, 2023, 4:24:56 PM.
Geometry shape function: Linear Lagrange
Time: 0 s.
Physical memory: 1.32 GB
Virtual memory: 1.29 GB
Ended at May 28, 2023, 4:24:56 PM.
----- Compile Equations: Oxygen Probe Pulse in Oxygen Pulse/Solution 958
      (sol958) ---------------------------------------------------------------->

<---- Dependent Variables 2 in Oxygen Pulse/Solution 958 (sol958) --------------
Started at May 28, 2023, 4:24:56 PM.
Initial values of variables solved for: Solution 958 (sol958), Solution Store 1 (sol
985).
Values of variables not solved for: Solution 958 (sol958), Solution Store 1 (sol985)
.
Solution time: 0 s.
Physical memory: 1.32 GB
```

```
Virtual memory: 1.3 GB
Ended at May 28, 2023, 4:24:56 PM.
----- Dependent Variables 2 in Oxygen Pulse/Solution 958 (sol958) ------------->

<---- Time-Dependent Solver 1 in Oxygen Pulse/Solution 958 (sol958) ------------
Started at May 28, 2023, 4:24:56 PM.
Time-dependent solver (BDF)
Number of degrees of freedom solved for: 17710 (plus 35580 internal DOFs).
Symmetric matrices found.
Scales for dependent variables:
Concentration (comp2.Oxi): 0.23
Step        Time    Stepsize      Res  Jac   Sol Order Tfail NLfail   LinErr   LinRes
   0           0           - out    5    3     5                  0  7.7e-16  1.5e-15
   1  2.9139e-08  2.9139e-08        7    4     7     1     0      0  4.1e-16  1.2e-15
   2  5.8279e-08  2.9139e-08        9    5     9     1     0      0  3.8e-16  1.3e-15
   3  1.1656e-07  5.8279e-08       11    6    11     2     0      0    4e-16  1.5e-15
   4  2.3311e-07  1.1656e-07       13    7    13     2     0      0  5.2e-16    2e-15
   5  4.6623e-07  2.3311e-07       15    8    15     2     0      0  7.2e-16  3.7e-15
   6  6.9934e-07  2.3311e-07       17    9    17     2     0      0  6.4e-16  4.1e-15
   7  9.3246e-07  2.3311e-07       19   10    19     2     0      0  9.6e-16  4.3e-15
   8  1.1656e-06  2.3311e-07       21   11    21     2     0      0  6.4e-16  4.1e-15
   9  1.6318e-06  4.6623e-07       23   12    23     2     0      0  9.7e-16  6.2e-15
  10   2.098e-06  4.6623e-07       25   13    25     2     0      0  7.8e-16    6e-15
  11  2.5643e-06  4.6623e-07       27   14    27     2     0      0  7.9e-16  5.9e-15
  12  3.0305e-06  4.6623e-07       29   15    29     2     0      0  9.8e-16  5.6e-15
  13   3.963e-06  9.3246e-07       31   16    31     2     0      0  8.1e-16    8e-15
  14  4.8954e-06  9.3246e-07       33   17    33     2     0      0  1.6e-15    7e-15
  15  5.8279e-06  9.3246e-07       35   18    35     2     0      0  1.1e-15  6.7e-15
  16  6.7603e-06  9.3246e-07       37   19    37     2     0      0  1.1e-15  6.1e-15
  17  8.6253e-06  1.8649e-06       39   20    39     2     0      0  1.5e-15  9.1e-15
  18   1.049e-05  1.8649e-06       41   21    41     2     0      0  1.3e-15  8.1e-15
  19  1.2355e-05  1.8649e-06       43   22    43     2     0      0  1.2e-15    8e-15
  20   1.422e-05  1.8649e-06       45   23    45     2     0      0  1.1e-15  7.5e-15
  21   1.795e-05  3.7298e-06       47   24    47     2     0      0  1.8e-15  1.1e-14
  22   2.168e-05  3.7298e-06       49   25    49     2     0      0  1.2e-15  9.1e-15
  23   2.541e-05  3.7298e-06       51   26    51     2     0      0  1.5e-15  9.4e-15
  24  2.9139e-05  3.7298e-06       53   27    53     2     0      0  1.4e-15  8.4e-15
  25  3.6599e-05  7.4597e-06       55   28    55     2     0      0    2e-15  1.2e-14
  26  4.4059e-05  7.4597e-06       57   29    57     2     0      0    2e-15  1.1e-14
  27  5.1518e-05  7.4597e-06       59   30    59     2     0      0    2e-15    1e-14
  28  5.8978e-05  7.4597e-06       61   31    61     2     0      0    2e-15  9.9e-15
  29  7.3897e-05  1.4919e-05       63   32    63     2     0      0  1.7e-15  1.3e-14
  30  8.8817e-05  1.4919e-05       65   33    65     2     0      0  1.7e-15  1.2e-14
  31  0.00010374  1.4919e-05       67   34    67     2     0      0  2.4e-15  1.2e-14
  32  0.00011866  1.4919e-05       69   35    69     2     0      0  1.2e-15  1.1e-14
  33  0.00014849  2.9839e-05       71   36    71     2     0      0  2.4e-15  1.7e-14
  34  0.00017833  2.9839e-05       73   37    73     2     0      0  1.9e-15  1.4e-14
  35  0.00020817  2.9839e-05       75   38    75     2     0      0  1.5e-15  1.3e-14
  36  0.00023801  2.9839e-05       77   39    77     2     0      0  1.8e-15  1.2e-14
  37  0.00029769  5.9677e-05       79   40    79     2     0      0  2.1e-15  1.7e-14
  38  0.00035737  5.9677e-05       81   41    81     2     0      0  1.8e-15  1.4e-14
  39  0.00047672  0.00011935       83   42    83     2     0      0  2.2e-15  2.3e-14
  40  0.00059607  0.00011935       85   43    85     2     0      0  1.1e-15  2.1e-14
  41  0.00071543  0.00011935       87   44    87     2     0      0  3.7e-15    2e-14
  42  0.00083478  0.00011935       89   45    89     2     0      0    3e-15  1.6e-14
  43   0.0010735  0.00023871       91   46    91     2     0      0  3.2e-15  2.3e-14
```

44	0.0013122	0.00023871	93	47	93	2	0	0	2.2e-15	2e-14
45	0.0017896	0.00047742	95	48	95	2	0	0	3.2e-15	3.5e-14
46	0.002267	0.00047742	97	49	97	2	0	0	3.2e-15	3.4e-14
47	0.0027445	0.00047742	99	50	99	2	0	0	6.3e-15	3.4e-14
48	0.0032219	0.00047742	101	51	101	2	0	0	3.2e-15	3e-14
49	0.0041767	0.00095484	103	52	103	2	0	0	5e-15	3.8e-14
-	0.005	-	out							
50	0.0051316	0.00095484	105	53	105	2	0	0	2e-15	3.1e-14
51	0.0070412	0.0019097	107	54	107	2	0	0	3.5e-15	5.1e-14
52	0.0089509	0.0019097	109	55	109	2	0	0	4.3e-15	4.9e-14
-	0.01	-	out							
53	0.010861	0.0019097	111	56	111	2	0	0	4.7e-15	4.5e-14
54	0.01468	0.0038194	113	57	113	2	0	0	3.5e-15	5.8e-14
-	0.015	-	out							
55	0.018499	0.0038194	115	58	115	2	0	0	8.1e-15	4e-14
-	0.02	-	out							
-	0.025	-	out							
56	0.026138	0.0076387	117	59	117	2	0	0	4.3e-15	6.9e-14
-	0.03	-	out							
57	0.033777	0.0076387	119	60	119	2	0	0	3.2e-15	6e-14
-	0.035	-	out							
-	0.04	-	out							
58	0.041415	0.0076387	121	61	121	2	0	0	7.9e-15	6.2e-14
-	0.045	-	out							
-	0.05	-	out							
-	0.055	-	out							
59	0.056693	0.015277	123	62	123	2	0	0	9e-15	7.8e-14
-	0.06	-	out							
-	0.065	-	out							
-	0.07	-	out							
-	0.075	-	out							
-	0.08	-	out							
-	0.085	-	out							
60	0.087248	0.030555	125	63	125	1	0	0	5.5e-15	6.5e-14
-	0.09	-	out							
-	0.095	-	out							
-	0.1	-	out							
-	0.105	-	out							
-	0.11	-	out							
-	0.115	-	out							
61	0.1178	0.030555	127	64	127	1	0	0	1.2e-14	3e-14
-	0.12	-	out							
-	0.125	-	out							
-	0.13	-	out							
-	0.135	-	out							
-	0.14	-	out							
-	0.145	-	out							
-	0.15	-	out							
-	0.155	-	out							
-	0.16	-	out							
-	0.165	-	out							
-	0.17	-	out							
-	0.175	-	out							
62	0.17891	0.06111	129	65	129	1	0	0	4.3e-15	4.2e-14
-	0.18	-	out							
-	0.185	-	out							

-	0.19	-	out							
-	0.195	-	out							
-	0.2	-	out							
-	0.205	-	out							
-	0.21	-	out							
-	0.215	-	out							
-	0.22	-	out							
-	0.225	-	out							
-	0.23	-	out							
-	0.235	-	out							
-	0.24	-	out							
-	0.245	-	out							
-	0.25	-	out							
-	0.25	-	out							
-	0.27	-	out							
63	0.27791	0.099	131	66	131	1	0	0	1.5e-14	4.6e-14
-	0.29	-	out							
-	0.31	-	out							
-	0.33	-	out							
-	0.35	-	out							
-	0.37	-	out							
64	0.37691	0.099	133	67	133	1	0	0	1.6e-14	3.6e-14
-	0.39	-	out							
-	0.41	-	out							
-	0.43	-	out							
-	0.45	-	out							
-	0.47	-	out							
65	0.47591	0.099	135	68	135	1	0	0	1.7e-14	3.1e-14
-	0.49	-	out							
-	0.51	-	out							
-	0.53	-	out							
-	0.55	-	out							
-	0.57	-	out							
66	0.57491	0.099	136	69	136	1	0	0	5.9e-15	2.5e-14
-	0.59	-	out							
-	0.61	-	out							
-	0.63	-	out							
-	0.65	-	out							
-	0.67	-	out							
67	0.67391	0.099	137	70	137	1	0	0	5.4e-15	2.1e-14
-	0.69	-	out							
-	0.71	-	out							
-	0.73	-	out							
-	0.75	-	out							
-	0.77	-	out							
68	0.77291	0.099	138	71	138	2	0	0	3.4e-15	1.8e-14
-	0.79	-	out							
-	0.81	-	out							
-	0.83	-	out							
-	0.85	-	out							
-	0.87	-	out							
69	0.87191	0.099	139	72	139	2	0	0	1.3e-14	1.2e-14
-	0.89	-	out							
-	0.91	-	out							
-	0.93	-	out							
-	0.95	-	out							

```
  -        0.97           - out
  70     0.97091       0.099      140   73  140     2     0       0  5.8e-15  1.1e-14
  -        0.99           - out
  71      1.0699       0.099      141   74  141     2     0       0  5.6e-15    1e-14
Time-stepping completed.
Solution time: 13 s.
Physical memory: 1.34 GB
Virtual memory: 1.33 GB
Ended at May 28, 2023, 4:25:09 PM.
----- Time-Dependent Solver 1 in Oxygen Pulse/Solution 958 (sol958) ----------->
```

k0Sub=1.51E-4, d=1E-6 (su74)

GENERAL

Description	Value
Solution	k0Sub=1.51E-4, d=1E-6

LOG

```
<---- Compile Equations: Stationary in Oxygen Pulse/Solution 958 (sol958) ------
Started at May 28, 2023, 4:25:12 PM.
Parameter k0Sub = 1.51E-4 (m/s).
Parameter d = 1.0E-6 (m).
Geometry shape function: Linear Lagrange
Number of vertex elements: 10
Minimum element quality: 0.4361
Time: 0 s.
Physical memory: 1.33 GB
Virtual memory: 1.31 GB
Ended at May 28, 2023, 4:25:12 PM.
----- Compile Equations: Stationary in Oxygen Pulse/Solution 958 (sol958) ----->

<---- Dependent Variables 1 in Oxygen Pulse/Solution 958 (sol958) --------------
Started at May 28, 2023, 4:25:12 PM.
Solution time: 0 s.
Physical memory: 1.33 GB
Virtual memory: 1.31 GB
Ended at May 28, 2023, 4:25:12 PM.
----- Dependent Variables 1 in Oxygen Pulse/Solution 958 (sol958) ------------->

<---- Stationary Solver 1 in Oxygen Pulse/Solution 958 (sol958) ----------------
Started at May 28, 2023, 4:25:12 PM.
Linear solver
Number of degrees of freedom solved for: 17724 (plus 963 internal DOFs).
Symmetric matrices found.
Scales for dependent variables:
Concentration (comp2.Oxi): 0.23
Orthonormal null-space function used.
Iter      SolEst     Damping    Stepsize #Res #Jac #Sol   LinErr   LinRes
   1        0.17   1.0000000        0.17    1    1    1  4.1e-13  9.6e-14
Solution time: 1 s.
Physical memory: 1.34 GB
Virtual memory: 1.32 GB
Ended at May 28, 2023, 4:25:13 PM.
----- Stationary Solver 1 in Oxygen Pulse/Solution 958 (sol958) --------------->

<---- Compile Equations: Oxygen Probe Pulse in Oxygen Pulse/Solution 958
      (sol958) -----------------------------------------------------------------
Started at May 28, 2023, 4:25:13 PM.
Geometry shape function: Linear Lagrange
Time: 1 s.
Physical memory: 1.35 GB
Virtual memory: 1.32 GB
Ended at May 28, 2023, 4:25:13 PM.
----- Compile Equations: Oxygen Probe Pulse in Oxygen Pulse/Solution 958
      (sol958) ---------------------------------------------------------------->

<---- Dependent Variables 2 in Oxygen Pulse/Solution 958 (sol958) --------------
Started at May 28, 2023, 4:25:14 PM.
Initial values of variables solved for: Solution 958 (sol958), Solution Store 1 (sol
985).
Values of variables not solved for: Solution 958 (sol958), Solution Store 1 (sol985)
.
Solution time: 0 s.
Physical memory: 1.34 GB
```

```
Virtual memory: 1.32 GB
Ended at May 28, 2023, 4:25:14 PM.
----- Dependent Variables 2 in Oxygen Pulse/Solution 958 (sol958) ------------->

<---- Time-Dependent Solver 1 in Oxygen Pulse/Solution 958 (sol958) ------------
Started at May 28, 2023, 4:25:14 PM.
Time-dependent solver (BDF)
Number of degrees of freedom solved for: 17724 (plus 35562 internal DOFs).
Symmetric matrices found.
Scales for dependent variables:
Concentration (comp2.Oxi): 0.23
Step        Time    Stepsize      Res   Jac   Sol Order Tfail NLfail   LinErr   LinRes
   0           0          - out     5     3     5                 0  7.6e-16  1.4e-15
   1  2.8846e-08  2.8846e-08        7     4     7     1     0       0  3.3e-16  1.1e-15
   2  5.7692e-08  2.8846e-08        9     5     9     1     0       0  4.1e-16  1.3e-15
   3  1.1538e-07  5.7692e-08       11     6    11     2     0       0    4e-16  1.4e-15
   4  2.3077e-07  1.1538e-07       13     7    13     2     0       0  4.4e-16  1.8e-15
   5  4.6154e-07  2.3077e-07       15     8    15     2     0       0  6.1e-16  3.6e-15
   6   6.923e-07  2.3077e-07       17     9    17     2     0       0  7.3e-16  4.1e-15
   7  9.2307e-07  2.3077e-07       19    10    19     2     0       0  6.6e-16  4.2e-15
   8  1.1538e-06  2.3077e-07       21    11    21     2     0       0  7.5e-16  4.3e-15
   9  1.6154e-06  4.6154e-07       23    12    23     2     0       0  8.7e-16  6.8e-15
  10  2.0769e-06  4.6154e-07       25    13    25     2     0       0  8.8e-16  6.2e-15
  11  2.5384e-06  4.6154e-07       27    14    27     2     0       0  8.9e-16  6.2e-15
  12       3e-06  4.6154e-07       29    15    29     2     0       0  8.6e-16  5.8e-15
  13  3.9231e-06  9.2307e-07       31    16    31     2     0       0  1.2e-15  8.5e-15
  14  4.8461e-06  9.2307e-07       33    17    33     2     0       0  1.3e-15  7.3e-15
  15  5.7692e-06  9.2307e-07       35    18    35     2     0       0    1e-15  7.4e-15
  16  6.6923e-06  9.2307e-07       37    19    37     2     0       0  1.1e-15  6.5e-15
  17  8.5384e-06  1.8461e-06       39    20    39     2     0       0  1.2e-15  9.3e-15
  18  1.0385e-05  1.8461e-06       41    21    41     2     0       0  1.5e-15    8e-15
  19  1.2231e-05  1.8461e-06       43    22    43     2     0       0  1.3e-15  7.8e-15
  20  1.4077e-05  1.8461e-06       45    23    45     2     0       0  1.3e-15  7.2e-15
  21  1.7769e-05  3.6923e-06       47    24    47     2     0       0  1.4e-15    1e-14
  22  2.1461e-05  3.6923e-06       49    25    49     2     0       0  1.8e-15  8.6e-15
  23  2.5154e-05  3.6923e-06       51    26    51     2     0       0  1.6e-15  8.9e-15
  24  2.8846e-05  3.6923e-06       53    27    53     2     0       0  1.7e-15  7.8e-15
  25  3.2538e-05  3.6923e-06       55    28    55     2     0       0  1.4e-15  6.9e-15
  26  3.9923e-05  7.3846e-06       57    29    57     2     0       0  1.5e-15    1e-14
  27  4.7307e-05  7.3846e-06       59    30    59     2     0       0  1.8e-15    9e-15
  28  5.4692e-05  7.3846e-06       61    31    61     2     0       0  1.4e-15    9e-15
  29  6.2077e-05  7.3846e-06       63    32    63     2     0       0  2.7e-15  8.3e-15
  30  7.6846e-05  1.4769e-05       65    33    65     2     0       0  1.7e-15  1.2e-14
  31  9.1615e-05  1.4769e-05       67    34    67     2     0       0  1.6e-15  1.1e-14
  32  0.00010638  1.4769e-05       69    35    69     2     0       0  2.2e-15  1.1e-14
  33  0.00012115  1.4769e-05       71    36    71     2     0       0  1.8e-15  9.9e-15
  34  0.00015069  2.9538e-05       73    37    73     2     0       0  1.8e-15  1.4e-14
  35  0.00018023  2.9538e-05       75    38    75     2     0       0  1.7e-15  1.2e-14
  36  0.00020977  2.9538e-05       77    39    77     2     0       0  2.2e-15  1.2e-14
  37  0.00023931  2.9538e-05       79    40    79     2     0       0  1.8e-15  1.2e-14
  38  0.00029838  5.9077e-05       81    41    81     2     0       0  2.2e-15  1.6e-14
  39  0.00035746  5.9077e-05       83    42    83     2     0       0  1.4e-15  1.3e-14
  40  0.00047561  0.00011815       85    43    85     2     0       0  2.7e-15  2.1e-14
  41  0.00059377  0.00011815       87    44    87     2     0       0    3e-15  1.8e-14
  42  0.00071192  0.00011815       89    45    89     2     0       0  3.1e-15  1.8e-14
  43  0.00083007  0.00011815       91    46    91     2     0       0  2.2e-15  1.6e-14
```

44	0.0010664	0.00023631		93	47	93	2	0	0	2.1e-15	2.1e-14
45	0.0013027	0.00023631		95	48	95	2	0	0	1.5e-15	1.6e-14
46	0.0017753	0.00047261		97	49	97	2	0	0	3.4e-15	2.9e-14
47	0.0022479	0.00047261		99	50	99	2	0	0	2.2e-15	2.7e-14
48	0.0027205	0.00047261		101	51	101	2	0	0	1.5e-15	2.8e-14
49	0.0031931	0.00047261		103	52	103	2	0	0	3.4e-15	2.4e-14
50	0.0041384	0.00094522		105	53	105	2	0	0	8e-15	3.3e-14
-	0.005	-	out								
51	0.0050836	0.00094522		107	54	107	2	0	0	5.1e-15	2.9e-14
52	0.006974	0.0018904		109	55	109	2	0	0	3.2e-15	4.5e-14
53	0.0088645	0.0018904		111	56	111	2	0	0	4.6e-15	4.6e-14
-	0.01	-	out								
54	0.010755	0.0018904		113	57	113	2	0	0	4.2e-15	4.5e-14
55	0.014536	0.0037809		115	58	115	2	0	0	6.5e-15	5.9e-14
-	0.015	-	out								
56	0.018317	0.0037809		117	59	117	2	0	0	3.9e-15	4e-14
-	0.02	-	out								
-	0.025	-	out								
57	0.025879	0.0075618		119	60	119	2	0	0	2.4e-15	6.4e-14
-	0.03	-	out								
58	0.03344	0.0075618		121	61	121	2	0	0	2.8e-15	5.7e-14
-	0.035	-	out								
-	0.04	-	out								
59	0.041002	0.0075618		123	62	123	2	0	0	2.7e-15	5.9e-14
-	0.045	-	out								
-	0.05	-	out								
-	0.055	-	out								
60	0.056126	0.015124		125	63	125	2	0	0	6.4e-15	7.3e-14
-	0.06	-	out								
-	0.065	-	out								
-	0.07	-	out								
-	0.075	-	out								
-	0.08	-	out								
-	0.085	-	out								
61	0.086373	0.030247		127	64	127	1	0	0	2.4e-14	7.3e-14
-	0.09	-	out								
-	0.095	-	out								
-	0.1	-	out								
-	0.105	-	out								
-	0.11	-	out								
-	0.115	-	out								
62	0.11662	0.030247		129	65	129	1	0	0	2.1e-14	3.3e-14
-	0.12	-	out								
-	0.125	-	out								
-	0.13	-	out								
-	0.135	-	out								
-	0.14	-	out								
-	0.145	-	out								
-	0.15	-	out								
-	0.155	-	out								
-	0.16	-	out								
-	0.165	-	out								
-	0.17	-	out								
-	0.175	-	out								
63	0.17711	0.060494		131	66	131	1	0	0	4.8e-15	4.3e-14
-	0.18	-	out								

-	0.185	- out								
-	0.19	- out								
-	0.195	- out								
-	0.2	- out								
-	0.205	- out								
-	0.21	- out								
-	0.215	- out								
-	0.22	- out								
-	0.225	- out								
-	0.23	- out								
-	0.235	- out								
-	0.24	- out								
-	0.245	- out								
-	0.25	- out								
-	0.25	- out								
-	0.27	- out								
64	0.27611	0.099	133	67	133	1	0	0	7.3e-15	4.8e-14
-	0.29	- out								
-	0.31	- out								
-	0.33	- out								
-	0.35	- out								
-	0.37	- out								
65	0.37511	0.099	135	68	135	1	0	0	1.1e-14	4e-14
-	0.39	- out								
-	0.41	- out								
-	0.43	- out								
-	0.45	- out								
-	0.47	- out								
66	0.47411	0.099	137	69	137	1	0	0	2.8e-15	3.1e-14
-	0.49	- out								
-	0.51	- out								
-	0.53	- out								
-	0.55	- out								
-	0.57	- out								
67	0.57311	0.099	138	70	138	1	0	0	6.5e-15	2.6e-14
-	0.59	- out								
-	0.61	- out								
-	0.63	- out								
-	0.65	- out								
-	0.67	- out								
68	0.67211	0.099	139	71	139	1	0	0	4.8e-15	2.3e-14
-	0.69	- out								
-	0.71	- out								
-	0.73	- out								
-	0.75	- out								
-	0.77	- out								
69	0.77111	0.099	140	72	140	2	0	0	1.7e-14	1.8e-14
-	0.79	- out								
-	0.81	- out								
-	0.83	- out								
-	0.85	- out								
-	0.87	- out								
70	0.87011	0.099	141	73	141	2	0	0	4.3e-15	1.3e-14
-	0.89	- out								
-	0.91	- out								
-	0.93	- out								

```
   -        0.95            - out
  71      0.96911       0.099      142    74   142      2      0       0  1.3e-14  1.2e-14
   -        0.97            - out
   -        0.99            - out
  72       1.0681       0.099      143    75   143      2      0       0  7.2e-15    1e-14
Time-stepping completed.
Solution time: 13 s.
Physical memory: 1.36 GB
Virtual memory: 1.34 GB
Ended at May 28, 2023, 4:25:27 PM.
----- Time-Dependent Solver 1 in Oxygen Pulse/Solution 958 (sol958) ----------->
```

k0Sub=1.51E-4, d=1.5E-6 (su75)

GENERAL

Description	Value
Solution	k0Sub=1.51E-4, d=1.5E-6

LOG

```
<---- Compile Equations: Stationary in Oxygen Pulse/Solution 958 (sol958) ------
Started at May 28, 2023, 4:25:29 PM.
Parameter k0Sub = 1.51E-4 (m/s).
Parameter d = 1.5E-6 (m).
Geometry shape function: Linear Lagrange
Number of vertex elements: 10
Minimum element quality: 0.395
Time: 0 s.
Physical memory: 1.33 GB
Virtual memory: 1.31 GB
Ended at May 28, 2023, 4:25:30 PM.
----- Compile Equations: Stationary in Oxygen Pulse/Solution 958 (sol958) ----->

<---- Dependent Variables 1 in Oxygen Pulse/Solution 958 (sol958) --------------
Started at May 28, 2023, 4:25:30 PM.
Solution time: 0 s.
Physical memory: 1.33 GB
Virtual memory: 1.31 GB
Ended at May 28, 2023, 4:25:30 PM.
----- Dependent Variables 1 in Oxygen Pulse/Solution 958 (sol958) ------------->

<---- Stationary Solver 1 in Oxygen Pulse/Solution 958 (sol958) ----------------
Started at May 28, 2023, 4:25:30 PM.
Linear solver
Number of degrees of freedom solved for: 17823 (plus 950 internal DOFs).
Symmetric matrices found.
Scales for dependent variables:
Concentration (comp2.Oxi): 0.23
Orthonormal null-space function used.
Iter      SolEst     Damping    Stepsize #Res #Jac #Sol   LinErr   LinRes
   1        0.15   1.0000000        0.15    1    1    1  3.5e-13  8.8e-14
Solution time: 1 s.
Physical memory: 1.34 GB
Virtual memory: 1.31 GB
Ended at May 28, 2023, 4:25:30 PM.
----- Stationary Solver 1 in Oxygen Pulse/Solution 958 (sol958) --------------->

<---- Compile Equations: Oxygen Probe Pulse in Oxygen Pulse/Solution 958
      (sol958) ----------------------------------------------------------------
Started at May 28, 2023, 4:25:30 PM.
Geometry shape function: Linear Lagrange
Time: 0 s.
Physical memory: 1.34 GB
Virtual memory: 1.31 GB
Ended at May 28, 2023, 4:25:31 PM.
----- Compile Equations: Oxygen Probe Pulse in Oxygen Pulse/Solution 958
      (sol958) --------------------------------------------------------------->

<---- Dependent Variables 2 in Oxygen Pulse/Solution 958 (sol958) --------------
Started at May 28, 2023, 4:25:31 PM.
Initial values of variables solved for: Solution 958 (sol958), Solution Store 1 (sol
985).
Values of variables not solved for: Solution 958 (sol958), Solution Store 1 (sol985)
.
Solution time: 0 s.
Physical memory: 1.34 GB
```

Virtual memory: 1.31 GB
Ended at May 28, 2023, 4:25:31 PM.
----- Dependent Variables 2 in Oxygen Pulse/Solution 958 (sol958) ------------->

<---- Time-Dependent Solver 1 in Oxygen Pulse/Solution 958 (sol958) ------------
Started at May 28, 2023, 4:25:31 PM.
Time-dependent solver (BDF)
Number of degrees of freedom solved for: 17823 (plus 35744 internal DOFs).
Symmetric matrices found.
Scales for dependent variables:
Concentration (comp2.Oxi): 0.23

Step	Time	Stepsize		Res	Jac	Sol	Order	Tfail	NLfail	LinErr	LinRes
0	0	-	out	5	3	5			0	1.1e-15	1.4e-15
1	2.8223e-08	2.8223e-08		7	4	7	1	0	0	3.7e-16	1.1e-15
2	5.6447e-08	2.8223e-08		9	5	9	1	0	0	3.5e-16	1.2e-15
3	1.1289e-07	5.6447e-08		11	6	11	2	0	0	4.3e-16	1.4e-15
4	2.2579e-07	1.1289e-07		13	7	13	2	0	0	4.7e-16	1.9e-15
5	4.5158e-07	2.2579e-07		15	8	15	2	0	0	7.4e-16	3.5e-15
6	6.7736e-07	2.2579e-07		17	9	17	2	0	0	6.4e-16	4e-15
7	9.0315e-07	2.2579e-07		19	10	19	2	0	0	5.1e-16	4.2e-15
8	1.1289e-06	2.2579e-07		21	11	21	2	0	0	5.2e-16	4.2e-15
9	1.5805e-06	4.5158e-07		23	12	23	2	0	0	8.4e-16	6.8e-15
10	2.0321e-06	4.5158e-07		25	13	25	2	0	0	9.1e-16	6.4e-15
11	2.4837e-06	4.5158e-07		27	14	27	2	0	0	8.6e-16	6.2e-15
12	2.9352e-06	4.5158e-07		29	15	29	2	0	0	8.1e-16	5.6e-15
13	3.8384e-06	9.0315e-07		31	16	31	2	0	0	1.4e-15	8.3e-15
14	4.7415e-06	9.0315e-07		33	17	33	2	0	0	1.4e-15	7.3e-15
15	5.6447e-06	9.0315e-07		35	18	35	2	0	0	1e-15	7e-15
16	6.5478e-06	9.0315e-07		37	19	37	2	0	0	1.1e-15	6.3e-15
17	8.3541e-06	1.8063e-06		39	20	39	2	0	0	1.5e-15	9.2e-15
18	1.016e-05	1.8063e-06		41	21	41	2	0	0	1.4e-15	8.3e-15
19	1.1967e-05	1.8063e-06		43	22	43	2	0	0	1.6e-15	8e-15
20	1.3773e-05	1.8063e-06		45	23	45	2	0	0	1.2e-15	7.4e-15
21	1.5579e-05	1.8063e-06		47	24	47	2	0	0	1.1e-15	6.4e-15
22	1.9192e-05	3.6126e-06		49	25	49	2	0	0	1.1e-15	9.5e-15
23	2.2805e-05	3.6126e-06		51	26	51	2	0	0	1.3e-15	8.4e-15
24	2.6417e-05	3.6126e-06		53	27	53	2	0	0	1.9e-15	8.2e-15
25	3.003e-05	3.6126e-06		55	28	55	2	0	0	1.9e-15	7.6e-15
26	3.7255e-05	7.2252e-06		57	29	57	2	0	0	1.8e-15	1.1e-14
27	4.448e-05	7.2252e-06		59	30	59	2	0	0	1.2e-15	9.4e-15
28	5.1705e-05	7.2252e-06		61	31	61	2	0	0	1.8e-15	9.3e-15
29	5.8931e-05	7.2252e-06		63	32	63	2	0	0	1.6e-15	8.4e-15
30	7.3381e-05	1.445e-05		65	33	65	2	0	0	2.7e-15	1.3e-14
31	8.7831e-05	1.445e-05		67	34	67	2	0	0	1.9e-15	1.1e-14
32	0.00010228	1.445e-05		69	35	69	2	0	0	1.8e-15	1.1e-14
33	0.00011673	1.445e-05		71	36	71	2	0	0	1.7e-15	9.8e-15
34	0.00014563	2.8901e-05		73	37	73	2	0	0	2.6e-15	1.4e-14
35	0.00017453	2.8901e-05		75	38	75	2	0	0	2e-15	1.2e-14
36	0.00020343	2.8901e-05		77	39	77	2	0	0	1.3e-15	1.2e-14
37	0.00023234	2.8901e-05		79	40	79	2	0	0	2.7e-15	1.1e-14
38	0.00029014	5.7802e-05		81	41	81	2	0	0	2.2e-15	1.6e-14
39	0.00034794	5.7802e-05		83	42	83	2	0	0	2.2e-15	1.3e-14
40	0.00046354	0.0001156		85	43	85	2	0	0	3.4e-15	2.1e-14
41	0.00057915	0.0001156		87	44	87	2	0	0	3.9e-15	1.8e-14
42	0.00069475	0.0001156		89	45	89	2	0	0	4.6e-15	1.7e-14
43	0.00081035	0.0001156		91	46	91	2	0	0	3.5e-15	1.6e-14

44	0.0010416	0.00023121	93	47	93	2	0	0	2.9e-15	2e-14
45	0.0012728	0.00023121	95	48	95	2	0	0	3.9e-15	1.8e-14
46	0.0017352	0.00046241	97	49	97	2	0	0	1.5e-15	2.9e-14
47	0.0021976	0.00046241	99	50	99	2	0	0	4.2e-15	2.5e-14
48	0.00266	0.00046241	101	51	101	2	0	0	3.2e-15	2.4e-14
49	0.0031224	0.00046241	103	52	103	2	0	0	3.3e-15	2.1e-14
50	0.0040472	0.00092483	105	53	105	2	0	0	4.6e-15	2.9e-14
51	0.0049721	0.00092483	107	54	107	2	0	0	1.8e-15	2.7e-14
-	0.005	-	out							
52	0.0068217	0.0018497	109	55	109	2	0	0	3.5e-15	4.2e-14
53	0.0086714	0.0018497	111	56	111	2	0	0	5.6e-15	4.3e-14
-	0.01	-	out							
54	0.010521	0.0018497	113	57	113	2	0	0	2.1e-15	4.2e-14
55	0.01422	0.0036993	115	58	115	2	0	0	5.3e-15	5.6e-14
-	0.015	-	out							
56	0.01792	0.0036993	117	59	117	2	0	0	2.9e-15	4e-14
-	0.02	-	out							
-	0.025	-	out							
57	0.025318	0.0073986	119	60	119	2	0	0	4.7e-15	6.5e-14
-	0.03	-	out							
58	0.032717	0.0073986	121	61	121	2	0	0	5.7e-15	5.6e-14
-	0.035	-	out							
-	0.04	-	out							
59	0.040115	0.0073986	123	62	123	2	0	0	5.3e-15	5.7e-14
-	0.045	-	out							
-	0.05	-	out							
60	0.054913	0.014797	125	63	125	2	0	0	3.4e-15	7.1e-14
-	0.055	-	out							
-	0.06	-	out							
-	0.065	-	out							
-	0.07	-	out							
-	0.075	-	out							
-	0.08	-	out							
61	0.084507	0.029594	127	64	127	1	0	0	2.6e-15	7.5e-14
-	0.085	-	out							
-	0.09	-	out							
-	0.095	-	out							
-	0.1	-	out							
-	0.105	-	out							
-	0.11	-	out							
62	0.1141	0.029594	129	65	129	1	0	0	1.2e-14	3.8e-14
-	0.115	-	out							
-	0.12	-	out							
-	0.125	-	out							
-	0.13	-	out							
-	0.135	-	out							
-	0.14	-	out							
-	0.145	-	out							
-	0.15	-	out							
-	0.155	-	out							
-	0.16	-	out							
-	0.165	-	out							
-	0.17	-	out							
63	0.17329	0.059189	131	66	131	1	0	0	6.8e-15	4.7e-14
-	0.175	-	out							
-	0.18	-	out							

-	0.185	- out								
-	0.19	- out								
-	0.195	- out								
-	0.2	- out								
-	0.205	- out								
-	0.21	- out								
-	0.215	- out								
-	0.22	- out								
-	0.225	- out								
-	0.23	- out								
-	0.235	- out								
-	0.24	- out								
-	0.245	- out								
-	0.25	- out								
-	0.25	- out								
-	0.27	- out								
64	0.27229	0.099	133	67	133	1	0	0	4.1e-15	5.2e-14
-	0.29	- out								
-	0.31	- out								
-	0.33	- out								
-	0.35	- out								
-	0.37	- out								
65	0.37129	0.099	135	68	135	1	0	0	3.5e-15	4.2e-14
-	0.39	- out								
-	0.41	- out								
-	0.43	- out								
-	0.45	- out								
-	0.47	- out								
66	0.47029	0.099	137	69	137	1	0	0	1e-14	3.2e-14
-	0.49	- out								
-	0.51	- out								
-	0.53	- out								
-	0.55	- out								
67	0.56929	0.099	138	70	138	1	0	0	9.7e-15	2.8e-14
-	0.57	- out								
-	0.59	- out								
-	0.61	- out								
-	0.63	- out								
-	0.65	- out								
68	0.66829	0.099	139	71	139	1	0	0	4.8e-15	2.3e-14
-	0.67	- out								
-	0.69	- out								
-	0.71	- out								
-	0.73	- out								
-	0.75	- out								
69	0.76729	0.099	140	72	140	2	0	0	1.2e-14	1.8e-14
-	0.77	- out								
-	0.79	- out								
-	0.81	- out								
-	0.83	- out								
-	0.85	- out								
70	0.86629	0.099	141	73	141	2	0	0	6.2e-15	1.3e-14
-	0.87	- out								
-	0.89	- out								
-	0.91	- out								
-	0.93	- out								

```
   -        0.95           - out
  71     0.96529       0.099      142   74  142     2     0      0  9.2e-15  1.1e-14
   -        0.97           - out
   -        0.99           - out
  72      1.0643       0.099      143   75  143     2     0      0    6e-15    1e-14
Time-stepping completed.
Solution time: 13 s.
Physical memory: 1.35 GB
Virtual memory: 1.33 GB
Ended at May 28, 2023, 4:25:44 PM.
----- Time-Dependent Solver 1 in Oxygen Pulse/Solution 958 (sol958) ----------->
```

k0Sub=1.51E-4, d=2E-6 (su76)

GENERAL

Description	Value
Solution	k0Sub=1.51E-4, d=2E-6

LOG

```
<---- Compile Equations: Stationary in Oxygen Pulse/Solution 958 (sol958) ------
Started at May 28, 2023, 4:25:47 PM.
Parameter k0Sub = 1.51E-4 (m/s).
Parameter d = 2.0E-6 (m).
Geometry shape function: Linear Lagrange
Number of vertex elements: 10
Minimum element quality: 0.4112
Time: 0 s.
Physical memory: 1.35 GB
Virtual memory: 1.32 GB
Ended at May 28, 2023, 4:25:47 PM.
----- Compile Equations: Stationary in Oxygen Pulse/Solution 958 (sol958) ----->

<---- Dependent Variables 1 in Oxygen Pulse/Solution 958 (sol958) --------------
Started at May 28, 2023, 4:25:47 PM.
Solution time: 0 s.
Physical memory: 1.35 GB
Virtual memory: 1.32 GB
Ended at May 28, 2023, 4:25:47 PM.
----- Dependent Variables 1 in Oxygen Pulse/Solution 958 (sol958) ------------->

<---- Stationary Solver 1 in Oxygen Pulse/Solution 958 (sol958) ----------------
Started at May 28, 2023, 4:25:47 PM.
Linear solver
Number of degrees of freedom solved for: 17907 (plus 938 internal DOFs).
Symmetric matrices found.
Scales for dependent variables:
Concentration (comp2.Oxi): 0.23
Orthonormal null-space function used.
Iter      SolEst     Damping    Stepsize #Res #Jac #Sol   LinErr   LinRes
   1        0.13   1.0000000        0.13    1    1    1  4.2e-13    8e-14
Solution time: 1 s.
Physical memory: 1.35 GB
Virtual memory: 1.32 GB
Ended at May 28, 2023, 4:25:48 PM.
----- Stationary Solver 1 in Oxygen Pulse/Solution 958 (sol958) --------------->

<---- Compile Equations: Oxygen Probe Pulse in Oxygen Pulse/Solution 958
      (sol958) ----------------------------------------------------------------
Started at May 28, 2023, 4:25:48 PM.
Geometry shape function: Linear Lagrange
Time: 0 s.
Physical memory: 1.35 GB
Virtual memory: 1.32 GB
Ended at May 28, 2023, 4:25:48 PM.
----- Compile Equations: Oxygen Probe Pulse in Oxygen Pulse/Solution 958
      (sol958) --------------------------------------------------------------->

<---- Dependent Variables 2 in Oxygen Pulse/Solution 958 (sol958) --------------
Started at May 28, 2023, 4:25:48 PM.
Initial values of variables solved for: Solution 958 (sol958), Solution Store 1 (sol
985).
Values of variables not solved for: Solution 958 (sol958), Solution Store 1 (sol985)
.
Solution time: 0 s.
Physical memory: 1.35 GB
```

Virtual memory: 1.32 GB
Ended at May 28, 2023, 4:25:48 PM.
----- Dependent Variables 2 in Oxygen Pulse/Solution 958 (sol958) ------------->

<---- Time-Dependent Solver 1 in Oxygen Pulse/Solution 958 (sol958) ------------
Started at May 28, 2023, 4:25:48 PM.
Time-dependent solver (BDF)
Number of degrees of freedom solved for: 17907 (plus 35898 internal DOFs).
Symmetric matrices found.
Scales for dependent variables:
Concentration (comp2.Oxi): 0.23

Step	Time	Stepsize	Res	Jac	Sol	Order	Tfail	NLfail	LinErr	LinRes
0	0	- out	5	3	5			0	7.8e-16	1.4e-15
1	2.7904e-08	2.7904e-08	7	4	7	1	0	0	4e-16	1.1e-15
2	5.5808e-08	2.7904e-08	9	5	9	1	0	0	3.6e-16	1.2e-15
3	1.1162e-07	5.5808e-08	11	6	11	2	0	0	3.4e-16	1.3e-15
4	2.2323e-07	1.1162e-07	13	7	13	2	0	0	4.8e-16	1.8e-15
5	4.4647e-07	2.2323e-07	15	8	15	2	0	0	5.9e-16	3.5e-15
6	6.697e-07	2.2323e-07	17	9	17	2	0	0	6.9e-16	4.1e-15
7	8.9293e-07	2.2323e-07	19	10	19	2	0	0	6.9e-16	4.3e-15
8	1.1162e-06	2.2323e-07	21	11	21	2	0	0	6.6e-16	4.2e-15
9	1.5626e-06	4.4647e-07	23	12	23	2	0	0	7.2e-16	6.8e-15
10	2.0091e-06	4.4647e-07	25	13	25	2	0	0	7.5e-16	6.2e-15
11	2.4556e-06	4.4647e-07	27	14	27	2	0	0	6.2e-16	6.2e-15
12	2.902e-06	4.4647e-07	29	15	29	2	0	0	7.9e-16	5.6e-15
13	3.795e-06	8.9293e-07	31	16	31	2	0	0	1.2e-15	8.5e-15
14	4.6879e-06	8.9293e-07	33	17	33	2	0	0	1.4e-15	7.3e-15
15	5.5808e-06	8.9293e-07	35	18	35	2	0	0	1.3e-15	7.2e-15
16	6.4737e-06	8.9293e-07	37	19	37	2	0	0	1.4e-15	6.6e-15
17	8.2596e-06	1.7859e-06	39	20	39	2	0	0	1.1e-15	9.8e-15
18	1.0045e-05	1.7859e-06	41	21	41	2	0	0	1.7e-15	8.5e-15
19	1.1831e-05	1.7859e-06	43	22	43	2	0	0	1.7e-15	8.4e-15
20	1.3617e-05	1.7859e-06	45	23	45	2	0	0	1.4e-15	7.6e-15
21	1.5403e-05	1.7859e-06	47	24	47	2	0	0	1.1e-15	6.7e-15
22	1.8975e-05	3.5717e-06	49	25	49	2	0	0	1.7e-15	9.8e-15
23	2.2546e-05	3.5717e-06	51	26	51	2	0	0	1.5e-15	8.8e-15
24	2.6118e-05	3.5717e-06	53	27	53	2	0	0	1.7e-15	8.4e-15
25	2.969e-05	3.5717e-06	55	28	55	2	0	0	1.4e-15	7.8e-15
26	3.6833e-05	7.1434e-06	57	29	57	2	0	0	1.3e-15	1.2e-14
27	4.3977e-05	7.1434e-06	59	30	59	2	0	0	1.3e-15	9.6e-15
28	5.112e-05	7.1434e-06	61	31	61	2	0	0	1.8e-15	9.8e-15
29	5.8264e-05	7.1434e-06	63	32	63	2	0	0	2.4e-15	9.2e-15
30	7.2551e-05	1.4287e-05	65	33	65	2	0	0	1.7e-15	1.3e-14
31	8.6838e-05	1.4287e-05	67	34	67	2	0	0	2.7e-15	1.1e-14
32	0.00010112	1.4287e-05	69	35	69	2	0	0	1.6e-15	1e-14
33	0.00011541	1.4287e-05	71	36	71	2	0	0	2.2e-15	9.8e-15
34	0.00014399	2.8574e-05	73	37	73	2	0	0	4.4e-15	1.4e-14
35	0.00017256	2.8574e-05	75	38	75	2	0	0	2.6e-15	1.1e-14
36	0.00020113	2.8574e-05	77	39	77	2	0	0	3e-15	1.2e-14
37	0.00022971	2.8574e-05	79	40	79	2	0	0	2.8e-15	1.1e-14
38	0.00028685	5.7148e-05	81	41	81	2	0	0	2.4e-15	1.5e-14
39	0.000344	5.7148e-05	83	42	83	2	0	0	2.6e-15	1.2e-14
40	0.00040115	5.7148e-05	85	43	85	2	0	0	3.7e-15	1.3e-14
41	0.00051544	0.0001143	87	44	87	2	0	0	6.8e-15	1.9e-14
42	0.00062974	0.0001143	89	45	89	2	0	0	2.2e-15	1.5e-14
43	0.00074403	0.0001143	91	46	91	2	0	0	1.8e-15	1.4e-14

44	0.00097262	0.00022859		93	47	93	2	0	0	2e-15	2e-14
45	0.0012012	0.00022859		95	48	95	2	0	0	2.3e-15	1.7e-14
46	0.0014298	0.00022859		97	49	97	2	0	0	6.5e-15	1.9e-14
47	0.001887	0.00045718		99	50	99	2	0	0	2.5e-15	2.7e-14
48	0.0023442	0.00045718		101	51	101	2	0	0	3.5e-15	2.1e-14
49	0.0032585	0.00091436		103	52	103	2	0	0	3.7e-15	3.2e-14
50	0.0041729	0.00091436		105	53	105	2	0	0	6.2e-15	2.6e-14
-	0.005	-	out								
51	0.0050872	0.00091436		107	54	107	2	0	0	2.1e-15	2.7e-14
52	0.0060016	0.00091436		109	55	109	2	0	0	2.8e-15	2.4e-14
53	0.0078303	0.0018287		111	56	111	2	0	0	9e-15	3.5e-14
54	0.0096591	0.0018287		113	57	113	2	0	0	7.6e-15	3.3e-14
-	0.01	-	out								
55	0.013316	0.0036574		115	58	115	2	0	0	3.1e-15	5.1e-14
-	0.015	-	out								
56	0.016974	0.0036574		117	59	117	2	0	0	2.6e-15	4.4e-14
-	0.02	-	out								
57	0.024289	0.0073149		119	60	119	2	0	0	2.7e-15	6.4e-14
-	0.025	-	out								
-	0.03	-	out								
58	0.031604	0.0073149		121	61	121	2	0	0	4.1e-15	5.5e-14
-	0.035	-	out								
59	0.038919	0.0073149		123	62	123	2	0	0	5.9e-15	5.8e-14
-	0.04	-	out								
-	0.045	-	out								
-	0.05	-	out								
60	0.053548	0.01463		125	63	125	2	0	0	4.8e-15	7.2e-14
-	0.055	-	out								
-	0.06	-	out								
-	0.065	-	out								
61	0.068178	0.01463		127	64	127	1	0	0	5.3e-15	6.4e-14
-	0.07	-	out								
-	0.075	-	out								
-	0.08	-	out								
-	0.085	-	out								
-	0.09	-	out								
-	0.095	-	out								
62	0.097438	0.02926		129	65	129	1	0	0	4.4e-15	4.2e-14
-	0.1	-	out								
-	0.105	-	out								
-	0.11	-	out								
-	0.115	-	out								
-	0.12	-	out								
-	0.125	-	out								
-	0.13	-	out								
-	0.135	-	out								
-	0.14	-	out								
-	0.145	-	out								
-	0.15	-	out								
-	0.155	-	out								
63	0.15596	0.058519		131	66	131	1	0	0	6.1e-15	5.6e-14
-	0.16	-	out								
-	0.165	-	out								
-	0.17	-	out								
-	0.175	-	out								
-	0.18	-	out								

-	0.185	- out								
-	0.19	- out								
-	0.195	- out								
-	0.2	- out								
-	0.205	- out								
-	0.21	- out								
-	0.215	- out								
-	0.22	- out								
-	0.225	- out								
-	0.23	- out								
-	0.235	- out								
-	0.24	- out								
-	0.245	- out								
-	0.25	- out								
-	0.25	- out								
64	0.25496	0.099	133	67	133	1	0	0	5.1e-15	6.1e-14
-	0.27	- out								
-	0.29	- out								
-	0.31	- out								
-	0.33	- out								
-	0.35	- out								
65	0.35396	0.099	135	68	135	1	0	0	7e-15	4.8e-14
-	0.37	- out								
-	0.39	- out								
-	0.41	- out								
-	0.43	- out								
-	0.45	- out								
66	0.45296	0.099	137	69	137	1	0	0	4.6e-15	3.7e-14
-	0.47	- out								
-	0.49	- out								
-	0.51	- out								
-	0.53	- out								
-	0.55	- out								
67	0.55196	0.099	139	70	139	1	0	0	2.6e-15	2.9e-14
-	0.57	- out								
-	0.59	- out								
-	0.61	- out								
-	0.63	- out								
-	0.65	- out								
68	0.65096	0.099	140	71	140	1	0	0	4.9e-15	2.3e-14
-	0.67	- out								
-	0.69	- out								
-	0.71	- out								
-	0.73	- out								
69	0.74996	0.099	141	72	141	1	0	0	5.3e-15	2e-14
-	0.75	- out								
-	0.77	- out								
-	0.79	- out								
-	0.81	- out								
-	0.83	- out								
70	0.84896	0.099	142	73	142	2	0	0	6.1e-15	1.7e-14
-	0.85	- out								
-	0.87	- out								
-	0.89	- out								
-	0.91	- out								
-	0.93	- out								

```
  71      0.94796       0.099      143   74  143     2     0      0  2.9e-15  1.2e-14
   -         0.95           - out
   -         0.97           - out
   -         0.99           - out
  72       1.047        0.099      144   75  144     2     0      0  1.1e-14  1.1e-14
Time-stepping completed.
Solution time: 13 s.
Physical memory: 1.37 GB
Virtual memory: 1.35 GB
Ended at May 28, 2023, 4:26:02 PM.
----- Time-Dependent Solver 1 in Oxygen Pulse/Solution 958 (sol958) ----------->
```

k0Sub=1.51E-4, d=2.4E-6 (su77)

GENERAL

Description	Value
Solution	k0Sub=1.51E-4, d=2.4E-6

LOG

```
<---- Compile Equations: Stationary in Oxygen Pulse/Solution 958 (sol958) ------
Started at May 28, 2023, 4:26:04 PM.
Parameter k0Sub = 1.51E-4 (m/s).
Parameter d = 2.4E-6 (m).
Geometry shape function: Linear Lagrange
Number of vertex elements: 10
Minimum element quality: 0.4544
Time: 1 s.
Physical memory: 1.33 GB
Virtual memory: 1.31 GB
Ended at May 28, 2023, 4:26:05 PM.
----- Compile Equations: Stationary in Oxygen Pulse/Solution 958 (sol958) ----->

<---- Dependent Variables 1 in Oxygen Pulse/Solution 958 (sol958) --------------
Started at May 28, 2023, 4:26:05 PM.
Solution time: 0 s.
Physical memory: 1.28 GB
Virtual memory: 1.25 GB
Ended at May 28, 2023, 4:26:05 PM.
----- Dependent Variables 1 in Oxygen Pulse/Solution 958 (sol958) ------------->

<---- Stationary Solver 1 in Oxygen Pulse/Solution 958 (sol958) ----------------
Started at May 28, 2023, 4:26:05 PM.
Linear solver
Number of degrees of freedom solved for: 18002 (plus 937 internal DOFs).
Symmetric matrices found.
Scales for dependent variables:
Concentration (comp2.Oxi): 0.23
Orthonormal null-space function used.
Iter      SolEst     Damping    Stepsize #Res #Jac #Sol   LinErr   LinRes
   1        0.12   1.0000000        0.12    1    1    1  3.7e-13  7.6e-14
Solution time: 1 s.
Physical memory: 1.31 GB
Virtual memory: 1.28 GB
Ended at May 28, 2023, 4:26:06 PM.
----- Stationary Solver 1 in Oxygen Pulse/Solution 958 (sol958) --------------->

<---- Compile Equations: Oxygen Probe Pulse in Oxygen Pulse/Solution 958
      (sol958) -----------------------------------------------------------------
Started at May 28, 2023, 4:26:06 PM.
Geometry shape function: Linear Lagrange
Time: 0 s.
Physical memory: 1.31 GB
Virtual memory: 1.29 GB
Ended at May 28, 2023, 4:26:06 PM.
----- Compile Equations: Oxygen Probe Pulse in Oxygen Pulse/Solution 958
      (sol958) ---------------------------------------------------------------->

<---- Dependent Variables 2 in Oxygen Pulse/Solution 958 (sol958) --------------
Started at May 28, 2023, 4:26:06 PM.
Initial values of variables solved for: Solution 958 (sol958), Solution Store 1 (sol
985).
Values of variables not solved for: Solution 958 (sol958), Solution Store 1 (sol985)
.
Solution time: 0 s.
Physical memory: 1.31 GB
```

```
Virtual memory: 1.29 GB
Ended at May 28, 2023, 4:26:06 PM.
----- Dependent Variables 2 in Oxygen Pulse/Solution 958 (sol958) ------------->

<---- Time-Dependent Solver 1 in Oxygen Pulse/Solution 958 (sol958) ------------
Started at May 28, 2023, 4:26:06 PM.
Time-dependent solver (BDF)
Number of degrees of freedom solved for: 18002 (plus 36084 internal DOFs).
Symmetric matrices found.
Scales for dependent variables:
Concentration (comp2.Oxi): 0.23
Step       Time    Stepsize      Res  Jac  Sol Order Tfail NLfail   LinErr   LinRes
   0          0          - out     5    3    5                  0  9.1e-16  1.4e-15
   1 2.7818e-08  2.7818e-08        7    4    7     1     0      0  3.7e-16  1.1e-15
   2 5.5636e-08  2.7818e-08        9    5    9     1     0      0  3.8e-16  1.3e-15
   3 1.1127e-07  5.5636e-08       11    6   11     2     0      0  3.6e-16  1.3e-15
   4 2.2254e-07  1.1127e-07       13    7   13     2     0      0  4.7e-16  1.8e-15
   5 4.4509e-07  2.2254e-07       15    8   15     2     0      0  5.8e-16  3.6e-15
   6 6.6763e-07  2.2254e-07       17    9   17     2     0      0  6.7e-16  4.2e-15
   7 8.9017e-07  2.2254e-07       19   10   19     2     0      0  6.4e-16  4.3e-15
   8 1.1127e-06  2.2254e-07       21   11   21     2     0      0  6.5e-16  4.3e-15
   9 1.5578e-06  4.4509e-07       23   12   23     2     0      0    9e-16  6.7e-15
  10 2.0029e-06  4.4509e-07       25   13   25     2     0      0    1e-15  6.3e-15
  11  2.448e-06  4.4509e-07       27   14   27     2     0      0  9.2e-16  6.4e-15
  12 2.8931e-06  4.4509e-07       29   15   29     2     0      0  9.5e-16    6e-15
  13 3.7832e-06  8.9017e-07       31   16   31     2     0      0    1e-15  9.1e-15
  14 4.6734e-06  8.9017e-07       33   17   33     2     0      0  1.1e-15  7.7e-15
  15 5.5636e-06  8.9017e-07       35   18   35     2     0      0  1.1e-15  7.4e-15
  16 6.4537e-06  8.9017e-07       37   19   37     2     0      0  1.2e-15  6.8e-15
  17 8.2341e-06  1.7803e-06       39   20   39     2     0      0  1.4e-15    1e-14
  18 1.0014e-05  1.7803e-06       41   21   41     2     0      0  1.2e-15  8.5e-15
  19 1.1795e-05  1.7803e-06       43   22   43     2     0      0  1.5e-15  8.8e-15
  20 1.3575e-05  1.7803e-06       45   23   45     2     0      0  1.1e-15  7.7e-15
  21 1.5355e-05  1.7803e-06       47   24   47     2     0      0  1.4e-15  6.8e-15
  22 1.8916e-05  3.5607e-06       49   25   49     2     0      0  1.6e-15  9.9e-15
  23 2.2477e-05  3.5607e-06       51   26   51     2     0      0  1.5e-15  8.5e-15
  24 2.6038e-05  3.5607e-06       53   27   53     2     0      0  1.7e-15  8.5e-15
  25 2.9598e-05  3.5607e-06       55   28   55     2     0      0  1.5e-15  8.2e-15
  26  3.672e-05  7.1214e-06       57   29   57     2     0      0  1.5e-15  1.2e-14
  27 4.3841e-05  7.1214e-06       59   30   59     2     0      0  2.4e-15    1e-14
  28 5.0962e-05  7.1214e-06       61   31   61     2     0      0  1.9e-15  9.9e-15
  29 5.8084e-05  7.1214e-06       63   32   63     2     0      0  1.5e-15    9e-15
  30 7.2326e-05  1.4243e-05       65   33   65     2     0      0  2.1e-15  1.3e-14
  31 8.6569e-05  1.4243e-05       67   34   67     2     0      0  1.4e-15  1.1e-14
  32 0.00010081  1.4243e-05       69   35   69     2     0      0  2.7e-15  1.1e-14
  33 0.00011505  1.4243e-05       71   36   71     2     0      0  1.3e-15    1e-14
  34 0.00014354  2.8485e-05       73   37   73     2     0      0  2.2e-15  1.4e-14
  35 0.00017203  2.8485e-05       75   38   75     2     0      0  2.2e-15  1.2e-14
  36 0.00020051  2.8485e-05       77   39   77     2     0      0    2e-15  1.2e-14
  37   0.000229  2.8485e-05       79   40   79     2     0      0  2.7e-15  1.1e-14
  38 0.00028597  5.6971e-05       81   41   81     2     0      0  2.2e-15  1.6e-14
  39 0.00034294  5.6971e-05       83   42   83     2     0      0  1.5e-15  1.3e-14
  40 0.00039991  5.6971e-05       85   43   85     2     0      0    2e-15  1.4e-14
  41 0.00051385  0.00011394       87   44   87     2     0      0  3.2e-15  1.9e-14
  42 0.00062779  0.00011394       89   45   89     2     0      0  3.6e-15  1.5e-14
  43 0.00074174  0.00011394       91   46   91     2     0      0  4.5e-15  1.6e-14
```

44	0.00085568	0.00011394	93	47	93	2	0	0	3.7e-15	1.3e-14
45	0.0010836	0.00022788	95	48	95	2	0	0	2.1e-15	1.9e-14
46	0.0013114	0.00022788	97	49	97	2	0	0	1.8e-15	1.6e-14
47	0.0017672	0.00045577	99	50	99	2	0	0	4.8e-15	2.6e-14
48	0.002223	0.00045577	101	51	101	2	0	0	2.6e-15	2.5e-14
49	0.0026787	0.00045577	103	52	103	2	0	0	2.5e-15	2.4e-14
50	0.0035903	0.00091154	105	53	105	2	0	0	2.7e-15	3.2e-14
51	0.0045018	0.00091154	107	54	107	2	0	0	2.7e-15	2.2e-14
-	0.005	-	out							
52	0.0063249	0.0018231	109	55	109	2	0	0	9.2e-15	3.8e-14
53	0.008148	0.0018231	111	56	111	2	0	0	5e-15	3.5e-14
54	0.009971	0.0018231	113	57	113	2	0	0	3.9e-15	3.7e-14
-	0.01	-	out							
55	0.013617	0.0036461	115	58	115	2	0	0	4.2e-15	5.3e-14
-	0.015	-	out							
56	0.017263	0.0036461	117	59	117	2	0	0	6e-15	3.7e-14
-	0.02	-	out							
57	0.024556	0.0072923	119	60	119	2	0	0	7e-15	6.1e-14
-	0.025	-	out							
-	0.03	-	out							
58	0.031848	0.0072923	121	61	121	2	0	0	4.3e-15	5.5e-14
-	0.035	-	out							
59	0.03914	0.0072923	123	62	123	2	0	0	6e-15	5.4e-14
-	0.04	-	out							
-	0.045	-	out							
-	0.05	-	out							
60	0.053725	0.014585	125	63	125	2	0	0	8.3e-15	7.2e-14
-	0.055	-	out							
-	0.06	-	out							
-	0.065	-	out							
61	0.068309	0.014585	127	64	127	1	0	0	1.4e-14	6.2e-14
-	0.07	-	out							
-	0.075	-	out							
-	0.08	-	out							
-	0.085	-	out							
-	0.09	-	out							
-	0.095	-	out							
62	0.097478	0.029169	129	65	129	1	0	0	9e-15	4.3e-14
-	0.1	-	out							
-	0.105	-	out							
-	0.11	-	out							
-	0.115	-	out							
-	0.12	-	out							
-	0.125	-	out							
-	0.13	-	out							
-	0.135	-	out							
-	0.14	-	out							
-	0.145	-	out							
-	0.15	-	out							
-	0.155	-	out							
63	0.15582	0.058338	131	66	131	1	0	0	1.2e-14	6e-14
-	0.16	-	out							
-	0.165	-	out							
-	0.17	-	out							
-	0.175	-	out							
-	0.18	-	out							

-	0.185	-	out							
-	0.19	-	out							
-	0.195	-	out							
-	0.2	-	out							
-	0.205	-	out							
-	0.21	-	out							
-	0.215	-	out							
-	0.22	-	out							
-	0.225	-	out							
-	0.23	-	out							
-	0.235	-	out							
-	0.24	-	out							
-	0.245	-	out							
-	0.25	-	out							
-	0.25	-	out							
64	0.25482	0.099		133	67	133	1	0	0	1.6e-15	6.5e-14
-	0.27	-	out							
-	0.29	-	out							
-	0.31	-	out							
-	0.33	-	out							
-	0.35	-	out							
65	0.35382	0.099		135	68	135	1	0	0	9.2e-15	5.2e-14
-	0.37	-	out							
-	0.39	-	out							
-	0.41	-	out							
-	0.43	-	out							
-	0.45	-	out							
66	0.45282	0.099		137	69	137	1	0	0	4e-15	3.9e-14
-	0.47	-	out							
-	0.49	-	out							
-	0.51	-	out							
-	0.53	-	out							
-	0.55	-	out							
67	0.55182	0.099		139	70	139	1	0	0	8.4e-15	2.9e-14
-	0.57	-	out							
-	0.59	-	out							
-	0.61	-	out							
-	0.63	-	out							
-	0.65	-	out							
68	0.65082	0.099		140	71	140	1	0	0	5.8e-15	2.4e-14
-	0.67	-	out							
-	0.69	-	out							
-	0.71	-	out							
-	0.73	-	out							
69	0.74982	0.099		141	72	141	1	0	0	2.4e-15	2.1e-14
-	0.75	-	out							
-	0.77	-	out							
-	0.79	-	out							
-	0.81	-	out							
-	0.83	-	out							
70	0.84882	0.099		142	73	142	2	0	0	1.1e-14	1.8e-14
-	0.85	-	out							
-	0.87	-	out							
-	0.89	-	out							
-	0.91	-	out							
-	0.93	-	out							

```
  71      0.94782       0.099      143    74  143      2      0       0  5.3e-15  1.2e-14
   -         0.95           - out
   -         0.97           - out
   -         0.99           - out
  72       1.0468       0.099      144    75  144      2      0       0  5.2e-15  1.1e-14
Time-stepping completed.
Solution time: 13 s.
Physical memory: 1.36 GB
Virtual memory: 1.33 GB
Ended at May 28, 2023, 4:26:19 PM.
----- Time-Dependent Solver 1 in Oxygen Pulse/Solution 958 (sol958) ----------->
```

k0Sub=1.51E-4, d=3E-6 (su78)

GENERAL

Description	Value
Solution	k0Sub=1.51E-4, d=3E-6

LOG

```
<---- Compile Equations: Stationary in Oxygen Pulse/Solution 958 (sol958) ------
Started at May 28, 2023, 4:26:22 PM.
Parameter k0Sub = 1.51E-4 (m/s).
Parameter d = 3.0E-6 (m).
Geometry shape function: Linear Lagrange
Number of vertex elements: 10
Minimum element quality: 0.4275
Time: 1 s.
Physical memory: 1.33 GB
Virtual memory: 1.32 GB
Ended at May 28, 2023, 4:26:23 PM.
----- Compile Equations: Stationary in Oxygen Pulse/Solution 958 (sol958) ----->

<---- Dependent Variables 1 in Oxygen Pulse/Solution 958 (sol958) --------------
Started at May 28, 2023, 4:26:23 PM.
Solution time: 0 s.
Physical memory: 1.33 GB
Virtual memory: 1.32 GB
Ended at May 28, 2023, 4:26:23 PM.
----- Dependent Variables 1 in Oxygen Pulse/Solution 958 (sol958) ------------->

<---- Stationary Solver 1 in Oxygen Pulse/Solution 958 (sol958) ----------------
Started at May 28, 2023, 4:26:23 PM.
Linear solver
Number of degrees of freedom solved for: 22990 (plus 935 internal DOFs).
Symmetric matrices found.
Scales for dependent variables:
Concentration (comp2.Oxi): 0.23
Orthonormal null-space function used.
Iter      SolEst     Damping    Stepsize #Res #Jac #Sol   LinErr   LinRes
   1       0.088   1.0000000       0.088    1    1    1    4e-13  7.3e-14
Solution time: 1 s.
Physical memory: 1.33 GB
Virtual memory: 1.32 GB
Ended at May 28, 2023, 4:26:23 PM.
----- Stationary Solver 1 in Oxygen Pulse/Solution 958 (sol958) --------------->

<---- Compile Equations: Oxygen Probe Pulse in Oxygen Pulse/Solution 958
      (sol958) -----------------------------------------------------------------
Started at May 28, 2023, 4:26:23 PM.
Geometry shape function: Linear Lagrange
Time: 0 s.
Physical memory: 1.29 GB
Virtual memory: 1.26 GB
Ended at May 28, 2023, 4:26:24 PM.
----- Compile Equations: Oxygen Probe Pulse in Oxygen Pulse/Solution 958
      (sol958) ---------------------------------------------------------------->

<---- Dependent Variables 2 in Oxygen Pulse/Solution 958 (sol958) --------------
Started at May 28, 2023, 4:26:24 PM.
Initial values of variables solved for: Solution 958 (sol958), Solution Store 1 (sol
985).
Values of variables not solved for: Solution 958 (sol958), Solution Store 1 (sol985)
.
Solution time: 0 s.
Physical memory: 1.3 GB
```

Virtual memory: 1.27 GB
Ended at May 28, 2023, 4:26:24 PM.
----- Dependent Variables 2 in Oxygen Pulse/Solution 958 (sol958) ------------->

<---- Time-Dependent Solver 1 in Oxygen Pulse/Solution 958 (sol958) ------------
Started at May 28, 2023, 4:26:24 PM.
Time-dependent solver (BDF)
Number of degrees of freedom solved for: 22990 (plus 46056 internal DOFs).
Symmetric matrices found.
Scales for dependent variables:
Concentration (comp2.Oxi): 0.23

Step	Time	Stepsize	Res	Jac	Sol	Order	Tfail	NLfail	LinErr	LinRes
0	0	- out	5	3	5			0	4.4e-16	8.6e-16
1	3.7971e-08	3.7971e-08	7	4	7	1	0	0	3.6e-16	9.8e-16
2	7.5942e-08	3.7971e-08	9	5	9	1	0	0	3.6e-16	1e-15
3	1.5188e-07	7.5942e-08	11	6	11	2	0	0	3.4e-16	1.1e-15
4	3.0377e-07	1.5188e-07	13	7	13	2	0	0	4.2e-16	1.4e-15
5	6.0753e-07	3.0377e-07	15	8	15	2	0	0	4.8e-16	2.5e-15
6	9.113e-07	3.0377e-07	17	9	17	2	0	0	5.8e-16	2.9e-15
7	1.2151e-06	3.0377e-07	19	10	19	2	0	0	5.6e-16	3.2e-15
8	1.5188e-06	3.0377e-07	21	11	21	2	0	0	5.3e-16	3.2e-15
9	2.1264e-06	6.0753e-07	23	12	23	2	0	0	7.8e-16	5.1e-15
10	2.7339e-06	6.0753e-07	25	13	25	2	0	0	6.3e-16	5e-15
11	3.3414e-06	6.0753e-07	27	14	27	2	0	0	9.4e-16	4.9e-15
12	3.949e-06	6.0753e-07	29	15	29	2	0	0	6.5e-16	4.5e-15
13	5.164e-06	1.2151e-06	31	16	31	2	0	0	8.5e-16	7.1e-15
14	6.3791e-06	1.2151e-06	33	17	33	2	0	0	7e-16	6.3e-15
15	7.5942e-06	1.2151e-06	35	18	35	2	0	0	7.8e-16	6.5e-15
16	8.8092e-06	1.2151e-06	37	19	37	2	0	0	9.8e-16	5.9e-15
17	1.1239e-05	2.4301e-06	39	20	39	2	0	0	1.1e-15	9.3e-15
18	1.367e-05	2.4301e-06	41	21	41	2	0	0	1.1e-15	8.3e-15
19	1.61e-05	2.4301e-06	43	22	43	2	0	0	1.1e-15	7.7e-15
20	1.853e-05	2.4301e-06	45	23	45	2	0	0	1.1e-15	7.5e-15
21	2.339e-05	4.8603e-06	47	24	47	2	0	0	1.3e-15	1.1e-14
22	2.825e-05	4.8603e-06	49	25	49	2	0	0	1.2e-15	9.6e-15
23	3.3111e-05	4.8603e-06	51	26	51	2	0	0	1.7e-15	1e-14
24	3.7971e-05	4.8603e-06	53	27	53	2	0	0	1.5e-15	9.2e-15
25	4.7691e-05	9.7205e-06	55	28	55	2	0	0	1.6e-15	1.3e-14
26	5.7412e-05	9.7205e-06	57	29	57	2	0	0	1.6e-15	1.2e-14
27	6.7132e-05	9.7205e-06	59	30	59	2	0	0	2.2e-15	1.2e-14
28	7.6853e-05	9.7205e-06	61	31	61	2	0	0	1.8e-15	1.1e-14
29	9.6294e-05	1.9441e-05	63	32	63	2	0	0	1.5e-15	1.6e-14
30	0.00011574	1.9441e-05	65	33	65	2	0	0	1.9e-15	1.3e-14
31	0.00013518	1.9441e-05	67	34	67	2	0	0	1.8e-15	1.4e-14
32	0.00015462	1.9441e-05	69	35	69	2	0	0	1.7e-15	1.3e-14
33	0.0001935	3.8882e-05	71	36	71	2	0	0	1.5e-15	1.8e-14
34	0.00023238	3.8882e-05	73	37	73	2	0	0	1.4e-15	1.6e-14
35	0.00027126	3.8882e-05	75	38	75	2	0	0	2.2e-15	1.5e-14
36	0.00034903	7.7764e-05	77	39	77	2	0	0	1.6e-15	2.3e-14
37	0.00042679	7.7764e-05	79	40	79	2	0	0	2.4e-15	2e-14
38	0.00050456	7.7764e-05	81	41	81	2	0	0	2.5e-15	2e-14
39	0.00058232	7.7764e-05	83	42	83	2	0	0	1.9e-15	1.6e-14
40	0.00073785	0.00015553	85	43	85	2	0	0	1.9e-15	2.3e-14
41	0.00089338	0.00015553	87	44	87	2	0	0	2.4e-15	1.9e-14
42	0.0012044	0.00031106	89	45	89	2	0	0	3.3e-15	3e-14
43	0.0015155	0.00031106	91	46	91	2	0	0	2.2e-15	2.5e-14

44	0.0018266	0.00031106		93	47	93	2	0	0	6.2e-15	2.5e-14
45	0.0021376	0.00031106		95	48	95	2	0	0	2.9e-15	2.4e-14
46	0.0027597	0.00062211		97	49	97	2	0	0	1.1e-14	3.3e-14
47	0.0033818	0.00062211		99	50	99	2	0	0	5.1e-15	2.5e-14
48	0.0046261	0.0012442		101	51	101	2	0	0	3.3e-15	4.1e-14
-	0.005	-	out								
49	0.0058703	0.0012442		103	52	103	2	0	0	2.9e-15	3.3e-14
50	0.0083588	0.0024885		105	53	105	2	0	0	2.7e-15	5.1e-14
-	0.01	-	out								
51	0.010847	0.0024885		107	54	107	1	0	0	3.2e-15	6.8e-14
52	0.013336	0.0024885		109	55	109	1	0	0	3.1e-15	5e-14
-	0.015	-	out								
53	0.018313	0.0049769		111	56	111	1	0	0	7.1e-15	8.6e-14
-	0.02	-	out								
54	0.02329	0.0049769		113	57	113	1	0	0	6.9e-15	8.1e-14
-	0.025	-	out								
-	0.03	-	out								
55	0.033243	0.0099538		115	58	115	1	0	0	6.8e-15	1.2e-13
-	0.035	-	out								
-	0.04	-	out								
56	0.043197	0.0099538		117	59	117	1	0	0	4.8e-15	1.2e-13
-	0.045	-	out								
-	0.05	-	out								
-	0.055	-	out								
-	0.06	-	out								
57	0.063105	0.019908		119	60	119	1	0	0	4.5e-15	1.5e-13
-	0.065	-	out								
-	0.07	-	out								
-	0.075	-	out								
-	0.08	-	out								
58	0.083012	0.019908		121	61	121	1	0	0	6.2e-15	1.1e-13
-	0.085	-	out								
-	0.09	-	out								
-	0.095	-	out								
-	0.1	-	out								
-	0.105	-	out								
-	0.11	-	out								
-	0.115	-	out								
-	0.12	-	out								
59	0.12283	0.039815		123	62	123	1	0	0	5e-15	1.2e-13
-	0.125	-	out								
-	0.13	-	out								
-	0.135	-	out								
-	0.14	-	out								
-	0.145	-	out								
-	0.15	-	out								
-	0.155	-	out								
-	0.16	-	out								
60	0.16264	0.039815		125	63	125	1	0	0	9.7e-15	8.9e-14
-	0.165	-	out								
-	0.17	-	out								
-	0.175	-	out								
-	0.18	-	out								
-	0.185	-	out								
-	0.19	-	out								
-	0.195	-	out								

-	0.2	- out							
-	0.205	- out							
-	0.21	- out							
-	0.215	- out							
-	0.22	- out							
-	0.225	- out							
-	0.23	- out							
-	0.235	- out							
-	0.24	- out							
61	0.24227	0.079631	127	64	127	1	0	0	1.3e-14 9.7e-14
-	0.245	- out							
-	0.25	- out							
-	0.25	- out							
-	0.27	- out							
-	0.29	- out							
-	0.31	- out							
-	0.33	- out							
62	0.34127	0.099	129	65	129	1	0	0	4.1e-15 7.7e-14
-	0.35	- out							
-	0.37	- out							
-	0.39	- out							
-	0.41	- out							
-	0.43	- out							
63	0.44027	0.099	131	66	131	1	0	0	4.6e-15 5.8e-14
-	0.45	- out							
-	0.47	- out							
-	0.49	- out							
-	0.51	- out							
-	0.53	- out							
64	0.53927	0.099	133	67	133	1	0	0	8.3e-15 4.7e-14
-	0.55	- out							
-	0.57	- out							
-	0.59	- out							
-	0.61	- out							
-	0.63	- out							
65	0.63827	0.099	134	68	134	1	0	0	3.7e-15 3.6e-14
-	0.65	- out							
-	0.67	- out							
-	0.69	- out							
-	0.71	- out							
-	0.73	- out							
66	0.73727	0.099	135	69	135	1	0	0	1.2e-14 3.2e-14
-	0.75	- out							
-	0.77	- out							
-	0.79	- out							
-	0.81	- out							
-	0.83	- out							
67	0.83627	0.099	136	70	136	2	0	0	6.6e-15 2.6e-14
-	0.85	- out							
-	0.87	- out							
-	0.89	- out							
-	0.91	- out							
-	0.93	- out							
68	0.93527	0.099	137	71	137	2	0	0	1.3e-14 1.8e-14
-	0.95	- out							
-	0.97	- out							

```
   -        0.99            - out
  69      1.0343       0.099      138   72  138     2     0      0  5.6e-15  1.5e-14
Time-stepping completed.
Solution time: 16 s.
Physical memory: 1.36 GB
Virtual memory: 1.35 GB
Ended at May 28, 2023, 4:26:40 PM.
----- Time-Dependent Solver 1 in Oxygen Pulse/Solution 958 (sol958) ----------->
```

k0Sub=1.51E-4, d=4E-6 (su79)

GENERAL

Description	Value
Solution	k0Sub=1.51E-4, d=4E-6

LOG

```
<---- Compile Equations: Stationary in Oxygen Pulse/Solution 958 (sol958) ------
Started at May 28, 2023, 4:26:43 PM.
Parameter k0Sub = 1.51E-4 (m/s).
Parameter d = 4.0E-6 (m).
Geometry shape function: Linear Lagrange
Number of vertex elements: 10
Minimum element quality: 0.4317
Time: 0 s.
Physical memory: 1.33 GB
Virtual memory: 1.32 GB
Ended at May 28, 2023, 4:26:44 PM.
----- Compile Equations: Stationary in Oxygen Pulse/Solution 958 (sol958) ----->

<---- Dependent Variables 1 in Oxygen Pulse/Solution 958 (sol958) --------------
Started at May 28, 2023, 4:26:44 PM.
Solution time: 0 s.
Physical memory: 1.33 GB
Virtual memory: 1.32 GB
Ended at May 28, 2023, 4:26:44 PM.
----- Dependent Variables 1 in Oxygen Pulse/Solution 958 (sol958) ------------->

<---- Stationary Solver 1 in Oxygen Pulse/Solution 958 (sol958) ----------------
Started at May 28, 2023, 4:26:44 PM.
Linear solver
Number of degrees of freedom solved for: 18182 (plus 936 internal DOFs).
Symmetric matrices found.
Scales for dependent variables:
Concentration (comp2.Oxi): 0.23
Orthonormal null-space function used.
Iter      SolEst     Damping    Stepsize #Res #Jac #Sol   LinErr   LinRes
   1       0.084   1.0000000       0.084    1    1    1  3.6e-13    6e-14
Solution time: 1 s.
Physical memory: 1.34 GB
Virtual memory: 1.33 GB
Ended at May 28, 2023, 4:26:44 PM.
----- Stationary Solver 1 in Oxygen Pulse/Solution 958 (sol958) --------------->

<---- Compile Equations: Oxygen Probe Pulse in Oxygen Pulse/Solution 958
      (sol958) ----------------------------------------------------------------
Started at May 28, 2023, 4:26:44 PM.
Geometry shape function: Linear Lagrange
Time: 0 s.
Physical memory: 1.35 GB
Virtual memory: 1.34 GB
Ended at May 28, 2023, 4:26:45 PM.
----- Compile Equations: Oxygen Probe Pulse in Oxygen Pulse/Solution 958
      (sol958) --------------------------------------------------------------->

<---- Dependent Variables 2 in Oxygen Pulse/Solution 958 (sol958) --------------
Started at May 28, 2023, 4:26:45 PM.
Initial values of variables solved for: Solution 958 (sol958), Solution Store 1 (sol
985).
Values of variables not solved for: Solution 958 (sol958), Solution Store 1 (sol985)
.
Solution time: 0 s.
Physical memory: 1.36 GB
```

Virtual memory: 1.34 GB
Ended at May 28, 2023, 4:26:45 PM.
----- Dependent Variables 2 in Oxygen Pulse/Solution 958 (sol958) ------------->

<---- Time-Dependent Solver 1 in Oxygen Pulse/Solution 958 (sol958) ------------
Started at May 28, 2023, 4:26:45 PM.
Time-dependent solver (BDF)
Number of degrees of freedom solved for: 18182 (plus 36438 internal DOFs).
Symmetric matrices found.
Scales for dependent variables:
Concentration (comp2.Oxi): 0.23

Step	Time	Stepsize	Res	Jac	Sol	Order	Tfail	NLfail	LinErr	LinRes
0	0	- out	5	3	5			0	9.5e-16	1.5e-15
1	2.7495e-08	2.7495e-08	7	4	7	1	0	0	3.4e-16	1.1e-15
2	5.499e-08	2.7495e-08	9	5	9	1	0	0	3.5e-16	1.3e-15
3	1.0998e-07	5.499e-08	11	6	11	2	0	0	4.1e-16	1.4e-15
4	2.1996e-07	1.0998e-07	13	7	13	2	0	0	4.6e-16	1.8e-15
5	4.3992e-07	2.1996e-07	15	8	15	2	0	0	6.8e-16	3.7e-15
6	6.5988e-07	2.1996e-07	17	9	17	2	0	0	6.9e-16	3.8e-15
7	8.7984e-07	2.1996e-07	19	10	19	2	0	0	6.3e-16	4e-15
8	1.0998e-06	2.1996e-07	21	11	21	2	0	0	6.6e-16	4.2e-15
9	1.5397e-06	4.3992e-07	23	12	23	2	0	0	1.1e-15	6.3e-15
10	1.9796e-06	4.3992e-07	25	13	25	2	0	0	7.7e-16	6e-15
11	2.4196e-06	4.3992e-07	27	14	27	2	0	0	8.6e-16	6.2e-15
12	2.8595e-06	4.3992e-07	29	15	29	2	0	0	9.3e-16	6.1e-15
13	3.7393e-06	8.7984e-07	31	16	31	2	0	0	1.2e-15	8.7e-15
14	4.6192e-06	8.7984e-07	33	17	33	2	0	0	1.2e-15	7.6e-15
15	5.499e-06	8.7984e-07	35	18	35	2	0	0	1.1e-15	7.4e-15
16	6.3788e-06	8.7984e-07	37	19	37	2	0	0	1.1e-15	6.6e-15
17	8.1385e-06	1.7597e-06	39	20	39	2	0	0	1.5e-15	9.9e-15
18	9.8982e-06	1.7597e-06	41	21	41	2	0	0	1.3e-15	8.4e-15
19	1.1658e-05	1.7597e-06	43	22	43	2	0	0	1.1e-15	8.3e-15
20	1.3418e-05	1.7597e-06	45	23	45	2	0	0	1.2e-15	7.3e-15
21	1.5177e-05	1.7597e-06	47	24	47	2	0	0	1.1e-15	6.7e-15
22	1.8697e-05	3.5194e-06	49	25	49	2	0	0	1.7e-15	1e-14
23	2.2216e-05	3.5194e-06	51	26	51	2	0	0	1.6e-15	8.7e-15
24	2.5735e-05	3.5194e-06	53	27	53	2	0	0	2.2e-15	8.8e-15
25	2.9255e-05	3.5194e-06	55	28	55	2	0	0	1.4e-15	7.8e-15
26	3.6293e-05	7.0387e-06	57	29	57	2	0	0	1.6e-15	1.2e-14
27	4.3332e-05	7.0387e-06	59	30	59	2	0	0	1.8e-15	9.9e-15
28	5.0371e-05	7.0387e-06	61	31	61	2	0	0	9.5e-16	1e-14
29	5.7409e-05	7.0387e-06	63	32	63	2	0	0	1.5e-15	9e-15
30	7.1487e-05	1.4077e-05	65	33	65	2	0	0	2e-15	1.4e-14
31	8.5564e-05	1.4077e-05	67	34	67	2	0	0	1.8e-15	1.2e-14
32	9.9642e-05	1.4077e-05	69	35	69	2	0	0	1.8e-15	1.1e-14
33	0.00011372	1.4077e-05	71	36	71	2	0	0	1.4e-15	1e-14
34	0.00014187	2.8155e-05	73	37	73	2	0	0	2.5e-15	1.4e-14
35	0.00017003	2.8155e-05	75	38	75	2	0	0	2e-15	1.2e-14
36	0.00019818	2.8155e-05	77	39	77	2	0	0	3.6e-15	1.3e-14
37	0.00022634	2.8155e-05	79	40	79	2	0	0	2.2e-15	1.1e-14
38	0.00028265	5.631e-05	81	41	81	2	0	0	3.8e-15	1.6e-14
39	0.00033896	5.631e-05	83	42	83	2	0	0	2.4e-15	1.3e-14
40	0.00039527	5.631e-05	85	43	85	2	0	0	2.5e-15	1.4e-14
41	0.00050789	0.00011262	87	44	87	2	0	0	4.8e-15	1.9e-14
42	0.00062051	0.00011262	89	45	89	2	0	0	6.6e-15	1.6e-14
43	0.00073313	0.00011262	91	46	91	2	0	0	2.3e-15	1.6e-14

44	0.00084575	0.00011262		93	47	93	2	0	0	1.1e-15	1.4e-14
45	0.001071	0.00022524		95	48	95	2	0	0	3.4e-15	2e-14
46	0.0012962	0.00022524		97	49	97	2	0	0	2.3e-15	1.6e-14
47	0.0017467	0.00045048		99	50	99	2	0	0	4.2e-15	2.6e-14
48	0.0021972	0.00045048		101	51	101	2	0	0	6.7e-15	2.2e-14
49	0.0026477	0.00045048		103	52	103	2	0	0	1.8e-15	2.2e-14
50	0.0030981	0.00045048		105	53	105	2	0	0	2.1e-15	2.1e-14
51	0.0039991	0.00090095		107	54	107	2	0	0	5.8e-15	2.7e-14
52	0.0049	0.00090095		109	55	109	2	0	0	1.8e-15	2e-14
-	0.005	-	out								
53	0.006702	0.0018019		111	56	111	2	0	0	3.1e-15	3.4e-14
54	0.0085039	0.0018019		113	57	113	2	0	0	5.2e-15	3.1e-14
-	0.01	-	out								
55	0.012108	0.0036038		115	58	115	2	0	0	6.7e-15	4.5e-14
-	0.015	-	out								
56	0.015712	0.0036038		117	59	117	1	0	0	4.7e-15	5.8e-14
57	0.019315	0.0036038		119	60	119	1	0	0	7.6e-15	4.5e-14
-	0.02	-	out								
-	0.025	-	out								
58	0.026523	0.0072076		121	61	121	1	0	0	3.4e-15	7.4e-14
-	0.03	-	out								
59	0.033731	0.0072076		123	62	123	1	0	0	4.5e-15	6.8e-14
-	0.035	-	out								
-	0.04	-	out								
-	0.045	-	out								
60	0.048146	0.014415		125	63	125	1	0	0	6.2e-15	9.4e-14
-	0.05	-	out								
-	0.055	-	out								
-	0.06	-	out								
61	0.062561	0.014415		127	64	127	1	0	0	6.9e-15	7.9e-14
-	0.065	-	out								
-	0.07	-	out								
-	0.075	-	out								
-	0.08	-	out								
-	0.085	-	out								
-	0.09	-	out								
62	0.091392	0.028831		129	65	129	1	0	0	1.1e-14	9.5e-14
-	0.095	-	out								
-	0.1	-	out								
-	0.105	-	out								
-	0.11	-	out								
-	0.115	-	out								
-	0.12	-	out								
63	0.12022	0.028831		131	66	131	1	0	0	7.4e-15	7.3e-14
-	0.125	-	out								
-	0.13	-	out								
-	0.135	-	out								
-	0.14	-	out								
-	0.145	-	out								
-	0.15	-	out								
-	0.155	-	out								
-	0.16	-	out								
-	0.165	-	out								
-	0.17	-	out								
-	0.175	-	out								
64	0.17788	0.057661		133	67	133	1	0	0	5.6e-15	7.9e-14

-	0.18	-	out							
-	0.185	-	out							
-	0.19	-	out							
-	0.195	-	out							
-	0.2	-	out							
-	0.205	-	out							
-	0.21	-	out							
-	0.215	-	out							
-	0.22	-	out							
-	0.225	-	out							
-	0.23	-	out							
-	0.235	-	out							
65	0.23554	0.057661	135	68	135	1	0	0	1e-14	5.9e-14
-	0.24	-	out							
-	0.245	-	out							
-	0.25	-	out							
-	0.25	-	out							
-	0.27	-	out							
-	0.29	-	out							
-	0.31	-	out							
-	0.33	-	out							
66	0.33454	0.099	137	69	137	1	0	0	1.5e-14	5.8e-14
-	0.35	-	out							
-	0.37	-	out							
-	0.39	-	out							
-	0.41	-	out							
-	0.43	-	out							
67	0.43354	0.099	139	70	139	1	0	0	6.9e-15	4.3e-14
-	0.45	-	out							
-	0.47	-	out							
-	0.49	-	out							
-	0.51	-	out							
-	0.53	-	out							
68	0.53254	0.099	141	71	141	1	0	0	2.1e-15	3.1e-14
-	0.55	-	out							
-	0.57	-	out							
-	0.59	-	out							
-	0.61	-	out							
-	0.63	-	out							
69	0.63154	0.099	142	72	142	1	0	0	1.2e-14	2.7e-14
-	0.65	-	out							
-	0.67	-	out							
-	0.69	-	out							
-	0.71	-	out							
-	0.73	-	out							
70	0.73054	0.099	143	73	143	1	0	0	8e-15	2.3e-14
-	0.75	-	out							
-	0.77	-	out							
-	0.79	-	out							
-	0.81	-	out							
71	0.82954	0.099	144	74	144	2	0	0	4.9e-15	1.9e-14
-	0.83	-	out							
-	0.85	-	out							
-	0.87	-	out							
-	0.89	-	out							
-	0.91	-	out							

```
  72     0.92854       0.099      145   75  145     2     0      0  4.7e-15  1.3e-14
   -        0.93           - out
   -        0.95           - out
   -        0.97           - out
   -        0.99           - out
  73      1.0275       0.099      146   76  146     2     0      0  4.5e-15  1.2e-14
Time-stepping completed.
Solution time: 13 s.
Physical memory: 1.38 GB
Virtual memory: 1.36 GB
Ended at May 28, 2023, 4:26:58 PM.
----- Time-Dependent Solver 1 in Oxygen Pulse/Solution 958 (sol958) ----------->
```

k0Sub=1.51E-4, d=5E-6 (su80)

GENERAL

Description	Value
Solution	k0Sub=1.51E-4, d=5E-6

LOG

```
<---- Compile Equations: Stationary in Oxygen Pulse/Solution 958 (sol958) ------
Started at May 28, 2023, 4:27:01 PM.
Parameter k0Sub = 1.51E-4 (m/s).
Parameter d = 4.9999999999999996E-6 (m).
Geometry shape function: Linear Lagrange
Number of vertex elements: 10
Minimum element quality: 0.4532
Time: 1 s.
Physical memory: 1.29 GB
Virtual memory: 1.26 GB
Ended at May 28, 2023, 4:27:01 PM.
----- Compile Equations: Stationary in Oxygen Pulse/Solution 958 (sol958) ----->

<---- Dependent Variables 1 in Oxygen Pulse/Solution 958 (sol958) --------------
Started at May 28, 2023, 4:27:01 PM.
Solution time: 0 s.
Physical memory: 1.29 GB
Virtual memory: 1.27 GB
Ended at May 28, 2023, 4:27:01 PM.
----- Dependent Variables 1 in Oxygen Pulse/Solution 958 (sol958) ------------->

<---- Stationary Solver 1 in Oxygen Pulse/Solution 958 (sol958) ----------------
Started at May 28, 2023, 4:27:01 PM.
Linear solver
Number of degrees of freedom solved for: 18304 (plus 936 internal DOFs).
Symmetric matrices found.
Scales for dependent variables:
Concentration (comp2.Oxi): 0.23
Orthonormal null-space function used.
Iter      SolEst     Damping    Stepsize #Res #Jac #Sol   LinErr   LinRes
   1       0.072   1.0000000       0.072    1    1    1  3.1e-13  5.5e-14
Solution time: 1 s.
Physical memory: 1.32 GB
Virtual memory: 1.29 GB
Ended at May 28, 2023, 4:27:02 PM.
----- Stationary Solver 1 in Oxygen Pulse/Solution 958 (sol958) --------------->

<---- Compile Equations: Oxygen Probe Pulse in Oxygen Pulse/Solution 958
      (sol958) ----------------------------------------------------------------
Started at May 28, 2023, 4:27:02 PM.
Geometry shape function: Linear Lagrange
Time: 0 s.
Physical memory: 1.32 GB
Virtual memory: 1.3 GB
Ended at May 28, 2023, 4:27:02 PM.
----- Compile Equations: Oxygen Probe Pulse in Oxygen Pulse/Solution 958
      (sol958) --------------------------------------------------------------->

<---- Dependent Variables 2 in Oxygen Pulse/Solution 958 (sol958) --------------
Started at May 28, 2023, 4:27:02 PM.
Initial values of variables solved for: Solution 958 (sol958), Solution Store 1 (sol
985).
Values of variables not solved for: Solution 958 (sol958), Solution Store 1 (sol985)
.
Solution time: 0 s.
Physical memory: 1.33 GB
```

```
Virtual memory: 1.3 GB
Ended at May 28, 2023, 4:27:03 PM.
----- Dependent Variables 2 in Oxygen Pulse/Solution 958 (sol958) ------------->

<---- Time-Dependent Solver 1 in Oxygen Pulse/Solution 958 (sol958) ------------
Started at May 28, 2023, 4:27:03 PM.
Time-dependent solver (BDF)
Number of degrees of freedom solved for: 18304 (plus 36680 internal DOFs).
Symmetric matrices found.
Scales for dependent variables:
Concentration (comp2.Oxi): 0.23
Step        Time    Stepsize      Res  Jac  Sol Order Tfail NLfail   LinErr   LinRes
   0           0          - out     5    3    5                  0  6.4e-16  1.4e-15
   1  2.7455e-08  2.7455e-08        7    4    7     1     0      0  3.6e-16  1.1e-15
   2  5.4909e-08  2.7455e-08        9    5    9     1     0      0  3.4e-16  1.2e-15
   3  1.0982e-07  5.4909e-08       11    6   11     2     0      0  3.8e-16  1.4e-15
   4  2.1964e-07  1.0982e-07       13    7   13     2     0      0  4.9e-16  1.8e-15
   5  4.3927e-07  2.1964e-07       15    8   15     2     0      0  5.9e-16  3.4e-15
   6  6.5891e-07  2.1964e-07       17    9   17     2     0      0  7.3e-16  3.9e-15
   7  8.7855e-07  2.1964e-07       19   10   19     2     0      0  7.3e-16  3.8e-15
   8  1.0982e-06  2.1964e-07       21   11   21     2     0      0    6e-16    4e-15
   9  1.5375e-06  4.3927e-07       23   12   23     2     0      0  8.2e-16  6.4e-15
  10  1.9767e-06  4.3927e-07       25   13   25     2     0      0  8.8e-16  6.2e-15
  11   2.416e-06  4.3927e-07       27   14   27     2     0      0  7.4e-16  6.3e-15
  12  2.8553e-06  4.3927e-07       29   15   29     2     0      0    8e-16  5.9e-15
  13  3.7338e-06  8.7855e-07       31   16   31     2     0      0  1.3e-15  8.8e-15
  14  4.6124e-06  8.7855e-07       33   17   33     2     0      0  1.1e-15  7.7e-15
  15  5.4909e-06  8.7855e-07       35   18   35     2     0      0  1.1e-15  7.3e-15
  16  6.3695e-06  8.7855e-07       37   19   37     2     0      0  1.1e-15  6.9e-15
  17  8.1266e-06  1.7571e-06       39   20   39     2     0      0  1.4e-15    1e-14
  18  9.8836e-06  1.7571e-06       41   21   41     2     0      0  1.5e-15  8.5e-15
  19  1.1641e-05  1.7571e-06       43   22   43     2     0      0  1.2e-15  8.2e-15
  20  1.3398e-05  1.7571e-06       45   23   45     2     0      0    1e-15  7.6e-15
  21  1.5155e-05  1.7571e-06       47   24   47     2     0      0  1.2e-15  6.8e-15
  22  1.8669e-05  3.5142e-06       49   25   49     2     0      0  1.2e-15    1e-14
  23  2.2183e-05  3.5142e-06       51   26   51     2     0      0  1.6e-15  8.8e-15
  24  2.5697e-05  3.5142e-06       53   27   53     2     0      0  1.4e-15  8.5e-15
  25  2.9212e-05  3.5142e-06       55   28   55     2     0      0  1.4e-15  7.8e-15
  26   3.624e-05  7.0284e-06       57   29   57     2     0      0  2.2e-15  1.2e-14
  27  4.3268e-05  7.0284e-06       59   30   59     2     0      0  2.4e-15    1e-14
  28  5.0297e-05  7.0284e-06       61   31   61     2     0      0  1.3e-15    1e-14
  29  5.7325e-05  7.0284e-06       63   32   63     2     0      0  1.6e-15  9.3e-15
  30  7.1382e-05  1.4057e-05       65   33   65     2     0      0  1.6e-15  1.4e-14
  31  8.5439e-05  1.4057e-05       67   34   67     2     0      0  1.9e-15  1.2e-14
  32  9.9495e-05  1.4057e-05       69   35   69     2     0      0  1.5e-15  1.2e-14
  33  0.00011355  1.4057e-05       71   36   71     2     0      0  2.1e-15  1.1e-14
  34  0.00014167  2.8113e-05       73   37   73     2     0      0  1.8e-15  1.5e-14
  35  0.00016978  2.8113e-05       75   38   75     2     0      0    2e-15  1.3e-14
  36  0.00019789  2.8113e-05       77   39   77     2     0      0  1.8e-15  1.3e-14
  37  0.00022601  2.8113e-05       79   40   79     2     0      0  3.3e-15  1.2e-14
  38  0.00028223  5.6227e-05       81   41   81     2     0      0  2.4e-15  1.7e-14
  39  0.00033846  5.6227e-05       83   42   83     2     0      0  2.6e-15  1.4e-14
  40  0.00039469  5.6227e-05       85   43   85     2     0      0    2e-15  1.4e-14
  41  0.00050714  0.00011245       87   44   87     2     0      0  3.2e-15    2e-14
  42  0.00061959  0.00011245       89   45   89     2     0      0  2.7e-15  1.6e-14
  43  0.00073205  0.00011245       91   46   91     2     0      0  2.5e-15  1.7e-14
```

44	0.0008445	0.00011245		93	47	93	2	0	0	4.4e-15	1.5e-14
45	0.0010694	0.00022491		95	48	95	2	0	0	2.8e-15	2e-14
46	0.0012943	0.00022491		97	49	97	2	0	0	3.3e-15	1.6e-14
47	0.0017441	0.00044982		99	50	99	2	0	0	3e-15	2.6e-14
48	0.0021939	0.00044982		101	51	101	2	0	0	2.8e-15	2.2e-14
49	0.0026438	0.00044982		103	52	103	2	0	0	6.4e-15	2.2e-14
50	0.0030936	0.00044982		105	53	105	2	0	0	3.5e-15	2.1e-14
51	0.0039932	0.00089963		107	54	107	2	0	0	2.2e-15	2.6e-14
52	0.0048928	0.00089963		109	55	109	2	0	0	4.2e-15	2e-14
-	0.005	-	out								
53	0.0066921	0.0017993		111	56	111	2	0	0	1.6e-15	3.4e-14
54	0.0084914	0.0017993		113	57	113	2	0	0	1.8e-15	2.9e-14
-	0.01	-	out								
55	0.01209	0.0035985		115	58	115	2	0	0	5.6e-15	4.3e-14
-	0.015	-	out								
56	0.015688	0.0035985		117	59	117	1	0	0	4.9e-15	5.2e-14
57	0.019287	0.0035985		119	60	119	1	0	0	3e-15	4.1e-14
-	0.02	-	out								
-	0.025	-	out								
58	0.026484	0.0071971		121	61	121	1	0	0	3.6e-15	6.7e-14
-	0.03	-	out								
59	0.033681	0.0071971		123	62	123	1	0	0	4.2e-15	6.4e-14
-	0.035	-	out								
-	0.04	-	out								
-	0.045	-	out								
60	0.048075	0.014394		125	63	125	1	0	0	5.2e-15	9.2e-14
-	0.05	-	out								
-	0.055	-	out								
-	0.06	-	out								
61	0.062469	0.014394		127	64	127	1	0	0	7.3e-15	7.8e-14
-	0.065	-	out								
-	0.07	-	out								
-	0.075	-	out								
-	0.08	-	out								
-	0.085	-	out								
-	0.09	-	out								
62	0.091257	0.028788		129	65	129	1	0	0	8.6e-15	1e-13
-	0.095	-	out								
-	0.1	-	out								
-	0.105	-	out								
-	0.11	-	out								
-	0.115	-	out								
-	0.12	-	out								
63	0.12005	0.028788		131	66	131	1	0	0	8.3e-15	7.3e-14
-	0.125	-	out								
-	0.13	-	out								
-	0.135	-	out								
-	0.14	-	out								
-	0.145	-	out								
-	0.15	-	out								
-	0.155	-	out								
-	0.16	-	out								
-	0.165	-	out								
-	0.17	-	out								
-	0.175	-	out								
64	0.17762	0.057576		133	67	133	1	0	0	6.3e-15	8.4e-14

-	0.18	-	out							
-	0.185	-	out							
-	0.19	-	out							
-	0.195	-	out							
-	0.2	-	out							
-	0.205	-	out							
-	0.21	-	out							
-	0.215	-	out							
-	0.22	-	out							
-	0.225	-	out							
-	0.23	-	out							
-	0.235	-	out							
65	0.2352	0.057576	135	68	135	1	0	0	1.3e-14	6e-14
-	0.24	-	out							
-	0.245	-	out							
-	0.25	-	out							
-	0.25	-	out							
-	0.27	-	out							
-	0.29	-	out							
-	0.31	-	out							
-	0.33	-	out							
66	0.3342	0.099	137	69	137	1	0	0	8.3e-15	5.7e-14
-	0.35	-	out							
-	0.37	-	out							
-	0.39	-	out							
-	0.41	-	out							
-	0.43	-	out							
67	0.4332	0.099	139	70	139	1	0	0	1.8e-14	4.4e-14
-	0.45	-	out							
-	0.47	-	out							
-	0.49	-	out							
-	0.51	-	out							
-	0.53	-	out							
68	0.5322	0.099	141	71	141	1	0	0	1.7e-14	3.5e-14
-	0.55	-	out							
-	0.57	-	out							
-	0.59	-	out							
-	0.61	-	out							
-	0.63	-	out							
69	0.6312	0.099	142	72	142	1	0	0	2.7e-15	2.8e-14
-	0.65	-	out							
-	0.67	-	out							
-	0.69	-	out							
-	0.71	-	out							
-	0.73	-	out							
70	0.7302	0.099	143	73	143	1	0	0	6.9e-15	2.4e-14
-	0.75	-	out							
-	0.77	-	out							
-	0.79	-	out							
-	0.81	-	out							
71	0.8292	0.099	144	74	144	2	0	0	1.1e-14	2e-14
-	0.83	-	out							
-	0.85	-	out							
-	0.87	-	out							
-	0.89	-	out							
-	0.91	-	out							

```
  72       0.9282       0.099      145   75  145     2     0      0  6.8e-15  1.4e-14
   -         0.93           - out
   -         0.95           - out
   -         0.97           - out
   -         0.99           - out
  73       1.0272       0.099      146   76  146     2     0      0  5.3e-15  1.3e-14
Time-stepping completed.
Solution time: 14 s.
Physical memory: 1.36 GB
Virtual memory: 1.34 GB
Ended at May 28, 2023, 4:27:16 PM.
----- Time-Dependent Solver 1 in Oxygen Pulse/Solution 958 (sol958) ----------->
```

k0Sub=1.51E-4, d=6E-6 (su81)

GENERAL

Description	Value
Solution	k0Sub=1.51E-4, d=6E-6

LOG

```
<---- Compile Equations: Stationary in Oxygen Pulse/Solution 958 (sol958) ------
Started at May 28, 2023, 4:27:19 PM.
Parameter k0Sub = 1.51E-4 (m/s).
Parameter d = 6.0E-6 (m).
Geometry shape function: Linear Lagrange
Number of vertex elements: 10
Minimum element quality: 0.432
Time: 1 s.
Physical memory: 1.36 GB
Virtual memory: 1.34 GB
Ended at May 28, 2023, 4:27:19 PM.
----- Compile Equations: Stationary in Oxygen Pulse/Solution 958 (sol958) ----->

<---- Dependent Variables 1 in Oxygen Pulse/Solution 958 (sol958) --------------
Started at May 28, 2023, 4:27:19 PM.
Solution time: 0 s.
Physical memory: 1.36 GB
Virtual memory: 1.34 GB
Ended at May 28, 2023, 4:27:19 PM.
----- Dependent Variables 1 in Oxygen Pulse/Solution 958 (sol958) ------------->

<---- Stationary Solver 1 in Oxygen Pulse/Solution 958 (sol958) ----------------
Started at May 28, 2023, 4:27:19 PM.
Linear solver
Number of degrees of freedom solved for: 18385 (plus 936 internal DOFs).
Symmetric matrices found.
Scales for dependent variables:
Concentration (comp2.Oxi): 0.23
Orthonormal null-space function used.
Iter      SolEst     Damping    Stepsize #Res #Jac #Sol   LinErr   LinRes
   1       0.063   1.0000000       0.063    1    1    1    3e-13  5.1e-14
Solution time: 1 s.
Physical memory: 1.36 GB
Virtual memory: 1.34 GB
Ended at May 28, 2023, 4:27:20 PM.
----- Stationary Solver 1 in Oxygen Pulse/Solution 958 (sol958) --------------->

<---- Compile Equations: Oxygen Probe Pulse in Oxygen Pulse/Solution 958
      (sol958) ----------------------------------------------------------------
Started at May 28, 2023, 4:27:20 PM.
Geometry shape function: Linear Lagrange
Time: 0 s.
Physical memory: 1.36 GB
Virtual memory: 1.34 GB
Ended at May 28, 2023, 4:27:21 PM.
----- Compile Equations: Oxygen Probe Pulse in Oxygen Pulse/Solution 958
      (sol958) --------------------------------------------------------------->

<---- Dependent Variables 2 in Oxygen Pulse/Solution 958 (sol958) --------------
Started at May 28, 2023, 4:27:21 PM.
Initial values of variables solved for: Solution 958 (sol958), Solution Store 1 (sol
985).
Values of variables not solved for: Solution 958 (sol958), Solution Store 1 (sol985)
.
Solution time: 0 s.
Physical memory: 1.3 GB
```

Virtual memory: 1.27 GB
Ended at May 28, 2023, 4:27:21 PM.
----- Dependent Variables 2 in Oxygen Pulse/Solution 958 (sol958) ------------->

<---- Time-Dependent Solver 1 in Oxygen Pulse/Solution 958 (sol958) ------------
Started at May 28, 2023, 4:27:21 PM.
Time-dependent solver (BDF)
Number of degrees of freedom solved for: 18385 (plus 36840 internal DOFs).
Symmetric matrices found.
Scales for dependent variables:
Concentration (comp2.Oxi): 0.23

Step	Time	Stepsize	Res	Jac	Sol	Order	Tfail	NLfail	LinErr	LinRes
0	0	- out	5	3	5			0	1.2e-15	1.5e-15
1	2.7395e-08	2.7395e-08	7	4	7	1	0	0	3.2e-16	1e-15
2	5.479e-08	2.7395e-08	9	5	9	1	0	0	3.6e-16	1.2e-15
3	1.0958e-07	5.479e-08	11	6	11	2	0	0	3.7e-16	1.3e-15
4	2.1916e-07	1.0958e-07	13	7	13	2	0	0	5e-16	1.8e-15
5	4.3832e-07	2.1916e-07	15	8	15	2	0	0	5.8e-16	3.5e-15
6	6.5748e-07	2.1916e-07	17	9	17	2	0	0	5.9e-16	3.9e-15
7	8.7664e-07	2.1916e-07	19	10	19	2	0	0	6.1e-16	4e-15
8	1.0958e-06	2.1916e-07	21	11	21	2	0	0	5.9e-16	4e-15
9	1.5341e-06	4.3832e-07	23	12	23	2	0	0	8.8e-16	6.4e-15
10	1.9724e-06	4.3832e-07	25	13	25	2	0	0	9e-16	6.1e-15
11	2.4108e-06	4.3832e-07	27	14	27	2	0	0	8.4e-16	6.2e-15
12	2.8491e-06	4.3832e-07	29	15	29	2	0	0	8.9e-16	5.9e-15
13	3.7257e-06	8.7664e-07	31	16	31	2	0	0	1.2e-15	8.9e-15
14	4.6024e-06	8.7664e-07	33	17	33	2	0	0	9.8e-16	7.5e-15
15	5.479e-06	8.7664e-07	35	18	35	2	0	0	1.4e-15	7.4e-15
16	6.3557e-06	8.7664e-07	37	19	37	2	0	0	1.3e-15	6.7e-15
17	8.1089e-06	1.7533e-06	39	20	39	2	0	0	1.6e-15	9.4e-15
18	9.8622e-06	1.7533e-06	41	21	41	2	0	0	1.1e-15	8.3e-15
19	1.1616e-05	1.7533e-06	43	22	43	2	0	0	1.2e-15	8.4e-15
20	1.3369e-05	1.7533e-06	45	23	45	2	0	0	9.1e-16	7.4e-15
21	1.5122e-05	1.7533e-06	47	24	47	2	0	0	1.4e-15	6.6e-15
22	1.8629e-05	3.5066e-06	49	25	49	2	0	0	1e-15	1e-14
23	2.2135e-05	3.5066e-06	51	26	51	2	0	0	2e-15	8.4e-15
24	2.5642e-05	3.5066e-06	53	27	53	2	0	0	1.1e-15	8.4e-15
25	2.9148e-05	3.5066e-06	55	28	55	2	0	0	1.4e-15	7.8e-15
26	3.6161e-05	7.0131e-06	57	29	57	2	0	0	1.5e-15	1.2e-14
27	4.3175e-05	7.0131e-06	59	30	59	2	0	0	1.7e-15	9.9e-15
28	5.0188e-05	7.0131e-06	61	31	61	2	0	0	1.7e-15	1e-14
29	5.7201e-05	7.0131e-06	63	32	63	2	0	0	2.2e-15	9.1e-15
30	7.1227e-05	1.4026e-05	65	33	65	2	0	0	2.2e-15	1.3e-14
31	8.5253e-05	1.4026e-05	67	34	67	2	0	0	2.1e-15	1.1e-14
32	9.928e-05	1.4026e-05	69	35	69	2	0	0	2.2e-15	1.2e-14
33	0.00011331	1.4026e-05	71	36	71	2	0	0	1.7e-15	1e-14
34	0.00014136	2.8053e-05	73	37	73	2	0	0	1.4e-15	1.5e-14
35	0.00016941	2.8053e-05	75	38	75	2	0	0	1.3e-15	1.3e-14
36	0.00019746	2.8053e-05	77	39	77	2	0	0	2.5e-15	1.3e-14
37	0.00022552	2.8053e-05	79	40	79	2	0	0	3e-15	1.2e-14
38	0.00028162	5.6105e-05	81	41	81	2	0	0	3.1e-15	1.7e-14
39	0.00033773	5.6105e-05	83	42	83	2	0	0	2.7e-15	1.4e-14
40	0.00039383	5.6105e-05	85	43	85	2	0	0	3.3e-15	1.4e-14
41	0.00050604	0.00011221	87	44	87	2	0	0	2.3e-15	2e-14
42	0.00061825	0.00011221	89	45	89	2	0	0	2.3e-15	1.7e-14
43	0.00073046	0.00011221	91	46	91	2	0	0	3.9e-15	1.7e-14

44	0.00084267	0.00011221		93	47	93	2	0	0	2e-15	1.4e-14
45	0.0010671	0.00022442		95	48	95	2	0	0	1.5e-15	2e-14
46	0.0012915	0.00022442		97	49	97	2	0	0	2.9e-15	1.6e-14
47	0.0017404	0.00044884		99	50	99	2	0	0	5.2e-15	2.6e-14
48	0.0021892	0.00044884		101	51	101	2	0	0	5e-15	2.3e-14
49	0.002638	0.00044884		103	52	103	2	0	0	3.8e-15	2.3e-14
50	0.0030869	0.00044884		105	53	105	2	0	0	2.1e-15	2.1e-14
51	0.0039846	0.00089768		107	54	107	2	0	0	1.8e-15	2.6e-14
52	0.0048822	0.00089768		109	55	109	2	0	0	2.5e-15	2.1e-14
-	0.005	-	out								
53	0.0066776	0.0017954		111	56	111	2	0	0	7.9e-15	3.6e-14
54	0.008473	0.0017954		113	57	113	2	0	0	5.2e-15	3.1e-14
-	0.01	-	out								
55	0.012064	0.0035907		115	58	115	2	0	0	5e-15	4.6e-14
-	0.015	-	out								
56	0.015654	0.0035907		117	59	117	1	0	0	1.8e-15	4.9e-14
57	0.019245	0.0035907		119	60	119	1	0	0	3.2e-15	3.8e-14
-	0.02	-	out								
-	0.025	-	out								
58	0.026427	0.0071815		121	61	121	1	0	0	2.4e-15	6.1e-14
-	0.03	-	out								
59	0.033608	0.0071815		123	62	123	1	0	0	2.6e-15	6e-14
-	0.035	-	out								
-	0.04	-	out								
-	0.045	-	out								
60	0.047971	0.014363		125	63	125	1	0	0	4.4e-15	8.9e-14
-	0.05	-	out								
-	0.055	-	out								
-	0.06	-	out								
61	0.062334	0.014363		127	64	127	1	0	0	5.2e-15	7.3e-14
-	0.065	-	out								
-	0.07	-	out								
-	0.075	-	out								
-	0.08	-	out								
-	0.085	-	out								
-	0.09	-	out								
62	0.09106	0.028726		129	65	129	1	0	0	8.4e-15	9.3e-14
-	0.095	-	out								
-	0.1	-	out								
-	0.105	-	out								
-	0.11	-	out								
-	0.115	-	out								
63	0.11979	0.028726		131	66	131	1	0	0	3.5e-15	7.5e-14
-	0.12	-	out								
-	0.125	-	out								
-	0.13	-	out								
-	0.135	-	out								
-	0.14	-	out								
-	0.145	-	out								
-	0.15	-	out								
-	0.155	-	out								
-	0.16	-	out								
-	0.165	-	out								
-	0.17	-	out								
-	0.175	-	out								
64	0.17724	0.057452		133	67	133	1	0	0	4.1e-15	8.5e-14

-	0.18	- out								
-	0.185	- out								
-	0.19	- out								
-	0.195	- out								
-	0.2	- out								
-	0.205	- out								
-	0.21	- out								
-	0.215	- out								
-	0.22	- out								
-	0.225	- out								
-	0.23	- out								
65	0.23469	0.057452	135	68	135	1	0	0	7.5e-15	6.3e-14
-	0.235	- out								
-	0.24	- out								
-	0.245	- out								
-	0.25	- out								
-	0.25	- out								
-	0.27	- out								
-	0.29	- out								
-	0.31	- out								
-	0.33	- out								
66	0.33369	0.099	137	69	137	1	0	0	1e-14	6.3e-14
-	0.35	- out								
-	0.37	- out								
-	0.39	- out								
-	0.41	- out								
-	0.43	- out								
67	0.43269	0.099	139	70	139	1	0	0	7.6e-15	4.8e-14
-	0.45	- out								
-	0.47	- out								
-	0.49	- out								
-	0.51	- out								
-	0.53	- out								
68	0.53169	0.099	141	71	141	1	0	0	7e-15	3.7e-14
-	0.55	- out								
-	0.57	- out								
-	0.59	- out								
-	0.61	- out								
-	0.63	- out								
69	0.63069	0.099	143	72	143	1	0	0	6.5e-15	2.9e-14
-	0.65	- out								
-	0.67	- out								
-	0.69	- out								
-	0.71	- out								
70	0.72969	0.099	144	73	144	1	0	0	5.4e-15	2.5e-14
-	0.73	- out								
-	0.75	- out								
-	0.77	- out								
-	0.79	- out								
-	0.81	- out								
71	0.82869	0.099	145	74	145	1	0	0	7.2e-15	2.1e-14
-	0.83	- out								
-	0.85	- out								
-	0.87	- out								
-	0.89	- out								
-	0.91	- out								

```
  72      0.92769       0.099      146   75  146     2     0      0  3.7e-15  1.7e-14
   -         0.93           - out
   -         0.95           - out
   -         0.97           - out
   -         0.99           - out
  73       1.0267       0.099      147   76  147     2     0      0  5.6e-15  1.2e-14
Time-stepping completed.
Solution time: 14 s.
Physical memory: 1.36 GB
Virtual memory: 1.34 GB
Ended at May 28, 2023, 4:27:34 PM.
----- Time-Dependent Solver 1 in Oxygen Pulse/Solution 958 (sol958) ----------->
```

k0Sub=1.51E-4, d=8E-6 (su82)

GENERAL

Description	Value
Solution	k0Sub=1.51E-4, d=8E-6

LOG

```
<---- Compile Equations: Stationary in Oxygen Pulse/Solution 958 (sol958) ------
Started at May 28, 2023, 4:27:37 PM.
Parameter k0Sub = 1.51E-4 (m/s).
Parameter d = 8.0E-6 (m).
Geometry shape function: Linear Lagrange
Number of vertex elements: 10
Minimum element quality: 0.4468
Time: 0 s.
Physical memory: 1.36 GB
Virtual memory: 1.34 GB
Ended at May 28, 2023, 4:27:37 PM.
----- Compile Equations: Stationary in Oxygen Pulse/Solution 958 (sol958) ----->

<---- Dependent Variables 1 in Oxygen Pulse/Solution 958 (sol958) --------------
Started at May 28, 2023, 4:27:37 PM.
Solution time: 0 s.
Physical memory: 1.36 GB
Virtual memory: 1.34 GB
Ended at May 28, 2023, 4:27:37 PM.
----- Dependent Variables 1 in Oxygen Pulse/Solution 958 (sol958) ------------->

<---- Stationary Solver 1 in Oxygen Pulse/Solution 958 (sol958) ----------------
Started at May 28, 2023, 4:27:37 PM.
Linear solver
Number of degrees of freedom solved for: 23800 (plus 941 internal DOFs).
Symmetric matrices found.
Scales for dependent variables:
Concentration (comp2.Oxi): 0.23
Orthonormal null-space function used.
Iter      SolEst     Damping    Stepsize #Res #Jac #Sol   LinErr   LinRes
   1       0.044   1.0000000       0.044    1    1    1  2.9e-13  4.9e-14
Solution time: 1 s.
Physical memory: 1.37 GB
Virtual memory: 1.34 GB
Ended at May 28, 2023, 4:27:38 PM.
----- Stationary Solver 1 in Oxygen Pulse/Solution 958 (sol958) --------------->

<---- Compile Equations: Oxygen Probe Pulse in Oxygen Pulse/Solution 958
      (sol958) -----------------------------------------------------------------
Started at May 28, 2023, 4:27:38 PM.
Geometry shape function: Linear Lagrange
Time: 0 s.
Physical memory: 1.37 GB
Virtual memory: 1.34 GB
Ended at May 28, 2023, 4:27:39 PM.
----- Compile Equations: Oxygen Probe Pulse in Oxygen Pulse/Solution 958
      (sol958) ---------------------------------------------------------------->

<---- Dependent Variables 2 in Oxygen Pulse/Solution 958 (sol958) --------------
Started at May 28, 2023, 4:27:39 PM.
Initial values of variables solved for: Solution 958 (sol958), Solution Store 1 (sol
985).
Values of variables not solved for: Solution 958 (sol958), Solution Store 1 (sol985)
.
Solution time: 0 s.
Physical memory: 1.37 GB
```

Virtual memory: 1.34 GB
Ended at May 28, 2023, 4:27:39 PM.
----- Dependent Variables 2 in Oxygen Pulse/Solution 958 (sol958) ------------->

<---- Time-Dependent Solver 1 in Oxygen Pulse/Solution 958 (sol958) ------------
Started at May 28, 2023, 4:27:39 PM.
Time-dependent solver (BDF)
Number of degrees of freedom solved for: 23800 (plus 47670 internal DOFs).
Symmetric matrices found.
Scales for dependent variables:
Concentration (comp2.Oxi): 0.23

Step	Time	Stepsize	Res	Jac	Sol	Order	Tfail	NLfail	LinErr	LinRes
0	0	- out	5	3	5			0	5.2e-16	9.1e-16
1	3.7322e-08	3.7322e-08	7	4	7	1	0	0	2.9e-16	9.3e-16
2	7.4644e-08	3.7322e-08	9	5	9	1	0	0	3.3e-16	1e-15
3	1.4929e-07	7.4644e-08	11	6	11	2	0	0	3.2e-16	1.1e-15
4	2.9857e-07	1.4929e-07	13	7	13	2	0	0	3.7e-16	1.3e-15
5	5.9715e-07	2.9857e-07	15	8	15	2	0	0	5.7e-16	2.4e-15
6	8.9572e-07	2.9857e-07	17	9	17	2	0	0	6e-16	2.9e-15
7	1.1943e-06	2.9857e-07	19	10	19	2	0	0	5.9e-16	3.2e-15
8	1.4929e-06	2.9857e-07	21	11	21	2	0	0	4.8e-16	3.3e-15
9	2.09e-06	5.9715e-07	23	12	23	2	0	0	6.9e-16	5e-15
10	2.6872e-06	5.9715e-07	25	13	25	2	0	0	5.9e-16	4.8e-15
11	3.2843e-06	5.9715e-07	27	14	27	2	0	0	6.3e-16	4.9e-15
12	3.8815e-06	5.9715e-07	29	15	29	2	0	0	6.4e-16	4.6e-15
13	5.0758e-06	1.1943e-06	31	16	31	2	0	0	9.5e-16	7.2e-15
14	6.2701e-06	1.1943e-06	33	17	33	2	0	0	1.1e-15	6.1e-15
15	7.4644e-06	1.1943e-06	35	18	35	2	0	0	5.9e-16	6.2e-15
16	8.6587e-06	1.1943e-06	37	19	37	2	0	0	1e-15	5.9e-15
17	1.1047e-05	2.3886e-06	39	20	39	2	0	0	1.4e-15	8.9e-15
18	1.3436e-05	2.3886e-06	41	21	41	2	0	0	1.1e-15	7.9e-15
19	1.5824e-05	2.3886e-06	43	22	43	2	0	0	1.6e-15	7.7e-15
20	1.8213e-05	2.3886e-06	45	23	45	2	0	0	9.4e-16	7.5e-15
21	2.299e-05	4.7772e-06	47	24	47	2	0	0	1.2e-15	1.1e-14
22	2.7767e-05	4.7772e-06	49	25	49	2	0	0	1.5e-15	9.5e-15
23	3.2545e-05	4.7772e-06	51	26	51	2	0	0	1.3e-15	9.8e-15
24	3.7322e-05	4.7772e-06	53	27	53	2	0	0	1.2e-15	9.1e-15
25	4.6876e-05	9.5544e-06	55	28	55	2	0	0	1.6e-15	1.3e-14
26	5.6431e-05	9.5544e-06	57	29	57	2	0	0	1.8e-15	1.1e-14
27	6.5985e-05	9.5544e-06	59	30	59	2	0	0	1.9e-15	1.1e-14
28	7.5539e-05	9.5544e-06	61	31	61	2	0	0	1.6e-15	1e-14
29	9.4648e-05	1.9109e-05	63	32	63	2	0	0	1.8e-15	1.6e-14
30	0.00011376	1.9109e-05	65	33	65	2	0	0	1.9e-15	1.4e-14
31	0.00013287	1.9109e-05	67	34	67	2	0	0	1e-15	1.4e-14
32	0.00015197	1.9109e-05	69	35	69	2	0	0	1.6e-15	1.2e-14
33	0.00019019	3.8218e-05	71	36	71	2	0	0	3.5e-15	1.8e-14
34	0.00022841	3.8218e-05	73	37	73	2	0	0	1.4e-15	1.5e-14
35	0.00026663	3.8218e-05	75	38	75	2	0	0	2.5e-15	1.5e-14
36	0.00030484	3.8218e-05	77	39	77	2	0	0	2.5e-15	1.4e-14
37	0.00038128	7.6435e-05	79	40	79	2	0	0	2.5e-15	2e-14
38	0.00045772	7.6435e-05	81	41	81	2	0	0	1.7e-15	1.7e-14
39	0.00061059	0.00015287	83	42	83	2	0	0	1.8e-15	2.7e-14
40	0.00076346	0.00015287	85	43	85	2	0	0	2.9e-15	2.3e-14
41	0.00091633	0.00015287	87	44	87	2	0	0	3.7e-15	2.3e-14
42	0.0010692	0.00015287	89	45	89	2	0	0	4.8e-15	2e-14
43	0.0013749	0.00030574	91	46	91	2	0	0	3.2e-15	2.7e-14

44	0.0016807	0.00030574		93	47	93	2	0	0	2.8e-15	2e-14
45	0.0022922	0.00061148		95	48	95	2	0	0	2.1e-15	3.1e-14
46	0.0029036	0.00061148		97	49	97	2	0	0	3.1e-15	2.8e-14
47	0.0035151	0.00061148		99	50	99	2	0	0	6.1e-15	2.9e-14
48	0.0047381	0.001223		101	51	101	2	0	0	7.5e-15	3.6e-14
-	0.005	-	out								
49	0.005961	0.001223		103	52	103	2	0	0	4.1e-15	2.6e-14
50	0.008407	0.0024459		105	53	105	2	0	0	4.4e-15	4.5e-14
-	0.01	-	out								
51	0.010853	0.0024459		107	54	107	1	0	0	2.6e-15	4.8e-14
52	0.013299	0.0024459		109	55	109	1	0	0	4.2e-15	3.3e-14
-	0.015	-	out								
53	0.018191	0.0048919		111	56	111	1	0	0	6.6e-15	5.4e-14
-	0.02	-	out								
-	0.025	-	out								
54	0.027974	0.0097837		113	57	113	1	0	0	6.1e-15	8.8e-14
-	0.03	-	out								
-	0.035	-	out								
55	0.037758	0.0097837		115	58	115	1	0	0	4.4e-15	8.5e-14
-	0.04	-	out								
-	0.045	-	out								
-	0.05	-	out								
-	0.055	-	out								
56	0.057325	0.019567		117	59	117	1	0	0	1.2e-14	1.2e-13
-	0.06	-	out								
-	0.065	-	out								
-	0.07	-	out								
-	0.075	-	out								
57	0.076893	0.019567		119	60	119	1	0	0	4e-15	1.1e-13
-	0.08	-	out								
-	0.085	-	out								
-	0.09	-	out								
-	0.095	-	out								
-	0.1	-	out								
-	0.105	-	out								
-	0.11	-	out								
-	0.115	-	out								
58	0.11603	0.039135		121	61	121	1	0	0	8.6e-15	1.3e-13
-	0.12	-	out								
-	0.125	-	out								
-	0.13	-	out								
-	0.135	-	out								
-	0.14	-	out								
-	0.145	-	out								
-	0.15	-	out								
-	0.155	-	out								
59	0.15516	0.039135		123	62	123	1	0	0	4.6e-15	1e-13
-	0.16	-	out								
-	0.165	-	out								
-	0.17	-	out								
-	0.175	-	out								
-	0.18	-	out								
-	0.185	-	out								
-	0.19	-	out								
-	0.195	-	out								
-	0.2	-	out								

-	0.205	-	out						
-	0.21	-	out						
-	0.215	-	out						
-	0.22	-	out						
-	0.225	-	out						
-	0.23	-	out						
60	0.23343	0.07827	125	63	125	1	0	0	5.2e-15	1.1e-13
-	0.235	-	out						
-	0.24	-	out						
-	0.245	-	out						
-	0.25	-	out						
-	0.25	-	out						
-	0.27	-	out						
-	0.29	-	out						
-	0.31	-	out						
61	0.3117	0.07827	127	64	127	1	0	0	4.8e-15	8.7e-14
-	0.33	-	out						
-	0.35	-	out						
-	0.37	-	out						
-	0.39	-	out						
-	0.41	-	out						
62	0.4107	0.099	129	65	129	1	0	0	6e-15	7.4e-14
-	0.43	-	out						
-	0.45	-	out						
-	0.47	-	out						
-	0.49	-	out						
63	0.5097	0.099	131	66	131	1	0	0	4.1e-15	5.7e-14
-	0.51	-	out						
-	0.53	-	out						
-	0.55	-	out						
-	0.57	-	out						
-	0.59	-	out						
64	0.6087	0.099	133	67	133	1	0	0	1.3e-14	4.6e-14
-	0.61	-	out						
-	0.63	-	out						
-	0.65	-	out						
-	0.67	-	out						
-	0.69	-	out						
65	0.7077	0.099	134	68	134	1	0	0	5.6e-15	3.7e-14
-	0.71	-	out						
-	0.73	-	out						
-	0.75	-	out						
-	0.77	-	out						
-	0.79	-	out						
66	0.8067	0.099	135	69	135	1	0	0	4.9e-15	3.2e-14
-	0.81	-	out						
-	0.83	-	out						
-	0.85	-	out						
-	0.87	-	out						
-	0.89	-	out						
67	0.9057	0.099	136	70	136	2	0	0	3.1e-15	2.6e-14
-	0.91	-	out						
-	0.93	-	out						
-	0.95	-	out						
-	0.97	-	out						
-	0.99	-	out						

```
  68      1.0047       0.099      137   71  137     2     0      0  4.2e-15  1.7e-14
Time-stepping completed.
Solution time: 16 s.
Physical memory: 1.38 GB
Virtual memory: 1.37 GB
Ended at May 28, 2023, 4:27:55 PM.
----- Time-Dependent Solver 1 in Oxygen Pulse/Solution 958 (sol958) ----------->
```

k0Sub=1.51E-4, d=1.2E-5 (su83)

GENERAL

Description	Value
Solution	k0Sub=1.51E-4, d=1.2E-5

LOG

```
<---- Compile Equations: Stationary in Oxygen Pulse/Solution 958 (sol958) ------
Started at May 28, 2023, 4:27:58 PM.
Parameter k0Sub = 1.51E-4 (m/s).
Parameter d = 1.2E-5 (m).
Geometry shape function: Linear Lagrange
Number of vertex elements: 10
Minimum element quality: 0.4505
Time: 1 s.
Physical memory: 1.34 GB
Virtual memory: 1.32 GB
Ended at May 28, 2023, 4:27:59 PM.
----- Compile Equations: Stationary in Oxygen Pulse/Solution 958 (sol958) ----->

<---- Dependent Variables 1 in Oxygen Pulse/Solution 958 (sol958) --------------
Started at May 28, 2023, 4:27:59 PM.
Solution time: 0 s.
Physical memory: 1.34 GB
Virtual memory: 1.32 GB
Ended at May 28, 2023, 4:27:59 PM.
----- Dependent Variables 1 in Oxygen Pulse/Solution 958 (sol958) ------------->

<---- Stationary Solver 1 in Oxygen Pulse/Solution 958 (sol958) ----------------
Started at May 28, 2023, 4:27:59 PM.
Linear solver
Number of degrees of freedom solved for: 18769 (plus 948 internal DOFs).
Symmetric matrices found.
Scales for dependent variables:
Concentration (comp2.Oxi): 0.23
Orthonormal null-space function used.
Iter      SolEst     Damping    Stepsize #Res #Jac #Sol   LinErr   LinRes
   1       0.038   1.0000000       0.038    1    1    1  2.3e-13  4.2e-14
Solution time: 1 s.
Physical memory: 1.35 GB
Virtual memory: 1.33 GB
Ended at May 28, 2023, 4:28:00 PM.
----- Stationary Solver 1 in Oxygen Pulse/Solution 958 (sol958) --------------->

<---- Compile Equations: Oxygen Probe Pulse in Oxygen Pulse/Solution 958
      (sol958) ----------------------------------------------------------------
Started at May 28, 2023, 4:28:00 PM.
Geometry shape function: Linear Lagrange
Time: 0 s.
Physical memory: 1.35 GB
Virtual memory: 1.33 GB
Ended at May 28, 2023, 4:28:00 PM.
----- Compile Equations: Oxygen Probe Pulse in Oxygen Pulse/Solution 958
      (sol958) --------------------------------------------------------------->

<---- Dependent Variables 2 in Oxygen Pulse/Solution 958 (sol958) --------------
Started at May 28, 2023, 4:28:00 PM.
Initial values of variables solved for: Solution 958 (sol958), Solution Store 1 (sol
985).
Values of variables not solved for: Solution 958 (sol958), Solution Store 1 (sol985)
.
Solution time: 0 s.
Physical memory: 1.36 GB
```

Virtual memory: 1.33 GB
Ended at May 28, 2023, 4:28:00 PM.
----- Dependent Variables 2 in Oxygen Pulse/Solution 958 (sol958) ------------->

<---- Time-Dependent Solver 1 in Oxygen Pulse/Solution 958 (sol958) ------------
Started at May 28, 2023, 4:28:00 PM.
Time-dependent solver (BDF)
Number of degrees of freedom solved for: 18769 (plus 37608 internal DOFs).
Symmetric matrices found.
Scales for dependent variables:
Concentration (comp2.Oxi): 0.23

Step	Time	Stepsize		Res	Jac	Sol	Order	Tfail	NLfail	LinErr	LinRes
0	0	-	out	5	3	5			0	8.9e-16	1.4e-15
1	2.7067e-08	2.7067e-08		7	4	7	1	0	0	3.6e-16	1e-15
2	5.4135e-08	2.7067e-08		9	5	9	1	0	0	3.7e-16	1.2e-15
3	1.0827e-07	5.4135e-08		11	6	11	2	0	0	3.4e-16	1.3e-15
4	2.1654e-07	1.0827e-07		13	7	13	2	0	0	5.2e-16	1.8e-15
5	4.3308e-07	2.1654e-07		15	8	15	2	0	0	7.4e-16	3.6e-15
6	6.4962e-07	2.1654e-07		17	9	17	2	0	0	6.5e-16	3.8e-15
7	8.6616e-07	2.1654e-07		19	10	19	2	0	0	6.8e-16	4.1e-15
8	1.0827e-06	2.1654e-07		21	11	21	2	0	0	6.1e-16	4e-15
9	1.5158e-06	4.3308e-07		23	12	23	2	0	0	7.4e-16	6.3e-15
10	1.9489e-06	4.3308e-07		25	13	25	2	0	0	7.6e-16	5.8e-15
11	2.3819e-06	4.3308e-07		27	14	27	2	0	0	8.1e-16	6.1e-15
12	2.815e-06	4.3308e-07		29	15	29	2	0	0	9e-16	5.7e-15
13	3.6812e-06	8.6616e-07		31	16	31	2	0	0	1.1e-15	8.3e-15
14	4.5473e-06	8.6616e-07		33	17	33	2	0	0	1.2e-15	7.4e-15
15	5.4135e-06	8.6616e-07		35	18	35	2	0	0	1.1e-15	7.2e-15
16	6.2796e-06	8.6616e-07		37	19	37	2	0	0	1e-15	6.4e-15
17	8.0119e-06	1.7323e-06		39	20	39	2	0	0	1.4e-15	9.2e-15
18	9.7443e-06	1.7323e-06		41	21	41	2	0	0	1.1e-15	8.2e-15
19	1.1477e-05	1.7323e-06		43	22	43	2	0	0	9.4e-16	8.2e-15
20	1.3209e-05	1.7323e-06		45	23	45	2	0	0	1.4e-15	7.3e-15
21	1.4941e-05	1.7323e-06		47	24	47	2	0	0	1.5e-15	6.7e-15
22	1.8406e-05	3.4646e-06		49	25	49	2	0	0	1.7e-15	9.8e-15
23	2.187e-05	3.4646e-06		51	26	51	2	0	0	1.3e-15	8.3e-15
24	2.5335e-05	3.4646e-06		53	27	53	2	0	0	1.5e-15	8.2e-15
25	2.88e-05	3.4646e-06		55	28	55	2	0	0	1.8e-15	7.8e-15
26	3.5729e-05	6.9293e-06		57	29	57	2	0	0	2.4e-15	1.1e-14
27	4.2658e-05	6.9293e-06		59	30	59	2	0	0	1.5e-15	9.3e-15
28	4.9587e-05	6.9293e-06		61	31	61	2	0	0	1.6e-15	9.4e-15
29	5.6517e-05	6.9293e-06		63	32	63	2	0	0	1.6e-15	8.9e-15
30	7.0375e-05	1.3859e-05		65	33	65	2	0	0	1.5e-15	1.2e-14
31	8.4234e-05	1.3859e-05		67	34	67	2	0	0	2.2e-15	1.1e-14
32	9.8092e-05	1.3859e-05		69	35	69	2	0	0	2.8e-15	1.1e-14
33	0.00011195	1.3859e-05		71	36	71	2	0	0	1.5e-15	1e-14
34	0.00013967	2.7717e-05		73	37	73	2	0	0	2.3e-15	1.5e-14
35	0.00016738	2.7717e-05		75	38	75	2	0	0	1.9e-15	1.3e-14
36	0.0001951	2.7717e-05		77	39	77	2	0	0	2.1e-15	1.2e-14
37	0.00022282	2.7717e-05		79	40	79	2	0	0	1.9e-15	1.1e-14
38	0.00027825	5.5434e-05		81	41	81	2	0	0	1.9e-15	1.6e-14
39	0.00033369	5.5434e-05		83	42	83	2	0	0	1.5e-15	1.3e-14
40	0.00038912	5.5434e-05		85	43	85	2	0	0	1.3e-15	1.3e-14
41	0.00049999	0.00011087		87	44	87	2	0	0	1.9e-15	1.9e-14
42	0.00061086	0.00011087		89	45	89	2	0	0	5.6e-15	1.7e-14
43	0.00072172	0.00011087		91	46	91	2	0	0	2.1e-15	1.7e-14

44	0.00083259	0.00011087		93	47	93	2	0	0	3.8e-15	1.4e-14
45	0.0010543	0.00022174		95	48	95	2	0	0	5.3e-15	2.1e-14
46	0.0012761	0.00022174		97	49	97	2	0	0	1.4e-15	1.6e-14
47	0.0017195	0.00044347		99	50	99	2	0	0	8.4e-15	2.7e-14
48	0.002163	0.00044347		101	51	101	2	0	0	5.5e-15	2.4e-14
49	0.0026065	0.00044347		103	52	103	2	0	0	4.2e-15	2.2e-14
50	0.00305	0.00044347		105	53	105	2	0	0	2e-15	1.9e-14
51	0.0039369	0.00088694		107	54	107	2	0	0	2e-15	2.4e-14
52	0.0048238	0.00088694		109	55	109	2	0	0	4.6e-15	1.9e-14
-	0.005	-	out								
53	0.0065977	0.0017739		111	56	111	2	0	0	4.2e-15	3e-14
54	0.0083716	0.0017739		113	57	113	2	0	0	7.4e-15	2.7e-14
-	0.01	-	out								
55	0.011919	0.0035478		115	58	115	2	0	0	3.2e-15	4e-14
-	0.015	-	out								
56	0.015467	0.0035478		117	59	117	1	0	0	2.8e-15	4.3e-14
57	0.019015	0.0035478		119	60	119	1	0	0	2.3e-15	2.5e-14
-	0.02	-	out								
-	0.025	-	out								
58	0.026111	0.0070956		121	61	121	1	0	0	3.3e-15	3.9e-14
-	0.03	-	out								
-	0.035	-	out								
-	0.04	-	out								
59	0.040302	0.014191		123	62	123	1	0	0	4.7e-15	6.3e-14
-	0.045	-	out								
-	0.05	-	out								
60	0.054493	0.014191		125	63	125	1	0	0	5.1e-15	6.2e-14
-	0.055	-	out								
-	0.06	-	out								
-	0.065	-	out								
-	0.07	-	out								
-	0.075	-	out								
-	0.08	-	out								
61	0.082875	0.028382		127	64	127	1	0	0	6.4e-15	8.5e-14
-	0.085	-	out								
-	0.09	-	out								
-	0.095	-	out								
-	0.1	-	out								
-	0.105	-	out								
-	0.11	-	out								
62	0.11126	0.028382		129	65	129	1	0	0	5.2e-15	7.4e-14
-	0.115	-	out								
-	0.12	-	out								
-	0.125	-	out								
-	0.13	-	out								
-	0.135	-	out								
-	0.14	-	out								
-	0.145	-	out								
-	0.15	-	out								
-	0.155	-	out								
-	0.16	-	out								
-	0.165	-	out								
63	0.16802	0.056764		131	66	131	1	0	0	6.4e-15	9.2e-14
-	0.17	-	out								
-	0.175	-	out								
-	0.18	-	out								

-	0.185	-	out							
-	0.19	-	out							
-	0.195	-	out							
-	0.2	-	out							
-	0.205	-	out							
-	0.21	-	out							
-	0.215	-	out							
-	0.22	-	out							
64	0.22479	0.056764		133	67	133	1	0	0	4.4e-15	7.4e-14
-	0.225	-	out							
-	0.23	-	out							
-	0.235	-	out							
-	0.24	-	out							
-	0.245	-	out							
-	0.25	-	out							
-	0.25	-	out							
-	0.27	-	out							
-	0.29	-	out							
-	0.31	-	out							
65	0.32379	0.099		135	68	135	1	0	0	7.3e-15	7.7e-14
-	0.33	-	out							
-	0.35	-	out							
-	0.37	-	out							
-	0.39	-	out							
-	0.41	-	out							
66	0.42279	0.099		137	69	137	1	0	0	6.4e-15	5.6e-14
-	0.43	-	out							
-	0.45	-	out							
-	0.47	-	out							
-	0.49	-	out							
-	0.51	-	out							
67	0.52179	0.099		139	70	139	1	0	0	1.2e-14	4.6e-14
-	0.53	-	out							
-	0.55	-	out							
-	0.57	-	out							
-	0.59	-	out							
-	0.61	-	out							
68	0.62079	0.099		141	71	141	1	0	0	8.9e-15	3.7e-14
-	0.63	-	out							
-	0.65	-	out							
-	0.67	-	out							
-	0.69	-	out							
-	0.71	-	out							
69	0.71979	0.099		142	72	142	1	0	0	2.2e-15	2.9e-14
-	0.73	-	out							
-	0.75	-	out							
-	0.77	-	out							
-	0.79	-	out							
-	0.81	-	out							
70	0.81879	0.099		143	73	143	1	0	0	5.5e-15	2.5e-14
-	0.83	-	out							
-	0.85	-	out							
-	0.87	-	out							
-	0.89	-	out							
-	0.91	-	out							
71	0.91779	0.099		144	74	144	2	0	0	7.1e-15	2.2e-14

```
   -        0.93           - out
   -        0.95           - out
   -        0.97           - out
   -        0.99           - out
  72      1.0168        0.099      145    75  145      2      0       0  1.2e-14  1.4e-14
Time-stepping completed.
Solution time: 14 s.
Physical memory: 1.36 GB
Virtual memory: 1.34 GB
Ended at May 28, 2023, 4:28:14 PM.
----- Time-Dependent Solver 1 in Oxygen Pulse/Solution 958 (sol958) ----------->
```

k0Sub=1.51E-4, d=1.5E-5 (su84)

GENERAL

Description	Value
Solution	k0Sub=1.51E-4, d=1.5E-5

LOG

```
<---- Compile Equations: Stationary in Oxygen Pulse/Solution 958 (sol958) ------
Started at May 28, 2023, 4:28:17 PM.
Parameter k0Sub = 1.51E-4 (m/s).
Parameter d = 1.4999999999999999E-5 (m).
Geometry shape function: Linear Lagrange
Number of vertex elements: 10
Minimum element quality: 0.4444
Time: 1 s.
Physical memory: 1.37 GB
Virtual memory: 1.35 GB
Ended at May 28, 2023, 4:28:17 PM.
----- Compile Equations: Stationary in Oxygen Pulse/Solution 958 (sol958) ----->

<---- Dependent Variables 1 in Oxygen Pulse/Solution 958 (sol958) --------------
Started at May 28, 2023, 4:28:17 PM.
Solution time: 0 s.
Physical memory: 1.37 GB
Virtual memory: 1.35 GB
Ended at May 28, 2023, 4:28:17 PM.
----- Dependent Variables 1 in Oxygen Pulse/Solution 958 (sol958) ------------->

<---- Stationary Solver 1 in Oxygen Pulse/Solution 958 (sol958) ----------------
Started at May 28, 2023, 4:28:17 PM.
Linear solver
Number of degrees of freedom solved for: 18918 (plus 952 internal DOFs).
Symmetric matrices found.
Scales for dependent variables:
Concentration (comp2.Oxi): 0.23
Orthonormal null-space function used.
Iter      SolEst     Damping    Stepsize #Res #Jac #Sol   LinErr   LinRes
   1       0.033   1.0000000       0.033    1    1    1  2.1e-13  3.9e-14
Solution time: 1 s.
Physical memory: 1.38 GB
Virtual memory: 1.36 GB
Ended at May 28, 2023, 4:28:18 PM.
----- Stationary Solver 1 in Oxygen Pulse/Solution 958 (sol958) --------------->

<---- Compile Equations: Oxygen Probe Pulse in Oxygen Pulse/Solution 958
      (sol958) -----------------------------------------------------------------
Started at May 28, 2023, 4:28:18 PM.
Geometry shape function: Linear Lagrange
Time: 0 s.
Physical memory: 1.38 GB
Virtual memory: 1.36 GB
Ended at May 28, 2023, 4:28:18 PM.
----- Compile Equations: Oxygen Probe Pulse in Oxygen Pulse/Solution 958
      (sol958) ---------------------------------------------------------------->

<---- Dependent Variables 2 in Oxygen Pulse/Solution 958 (sol958) --------------
Started at May 28, 2023, 4:28:18 PM.
Initial values of variables solved for: Solution 958 (sol958), Solution Store 1 (sol
985).
Values of variables not solved for: Solution 958 (sol958), Solution Store 1 (sol985)
.
Solution time: 0 s.
Physical memory: 1.38 GB
```

```
Virtual memory: 1.36 GB
Ended at May 28, 2023, 4:28:18 PM.
----- Dependent Variables 2 in Oxygen Pulse/Solution 958 (sol958) ------------->

<---- Time-Dependent Solver 1 in Oxygen Pulse/Solution 958 (sol958) ------------
Started at May 28, 2023, 4:28:18 PM.
Time-dependent solver (BDF)
Number of degrees of freedom solved for: 18918 (plus 37906 internal DOFs).
Symmetric matrices found.
Scales for dependent variables:
Concentration (comp2.Oxi): 0.23
Step        Time    Stepsize      Res   Jac   Sol Order Tfail NLfail   LinErr   LinRes
   0           0           - out    5     3     5                  0  7.5e-16  1.4e-15
   1  2.7154e-08  2.7154e-08        7     4     7     1     0      0  3.2e-16    1e-15
   2  5.4308e-08  2.7154e-08        9     5     9     1     0      0  3.4e-16  1.2e-15
   3  1.0862e-07  5.4308e-08       11     6    11     2     0      0  3.6e-16  1.3e-15
   4  2.1723e-07  1.0862e-07       13     7    13     2     0      0  4.6e-16  1.8e-15
   5  4.3446e-07  2.1723e-07       15     8    15     2     0      0  6.8e-16  3.6e-15
   6  6.5169e-07  2.1723e-07       17     9    17     2     0      0  5.7e-16  3.8e-15
   7  8.6893e-07  2.1723e-07       19    10    19     2     0      0  5.5e-16    4e-15
   8  1.0862e-06  2.1723e-07       21    11    21     2     0      0  6.1e-16    4e-15
   9  1.5206e-06  4.3446e-07       23    12    23     2     0      0    1e-15  6.4e-15
  10  1.9551e-06  4.3446e-07       25    13    25     2     0      0    8e-16    6e-15
  11  2.3895e-06  4.3446e-07       27    14    27     2     0      0  8.7e-16    6e-15
  12   2.824e-06  4.3446e-07       29    15    29     2     0      0  7.7e-16  5.7e-15
  13  3.6929e-06  8.6893e-07       31    16    31     2     0      0    9e-16  8.5e-15
  14  4.5619e-06  8.6893e-07       33    17    33     2     0      0    1e-15  7.5e-15
  15  5.4308e-06  8.6893e-07       35    18    35     2     0      0  1.3e-15  7.3e-15
  16  6.2997e-06  8.6893e-07       37    19    37     2     0      0    1e-15  6.6e-15
  17  8.0376e-06  1.7379e-06       39    20    39     2     0      0  1.5e-15  9.5e-15
  18  9.7754e-06  1.7379e-06       41    21    41     2     0      0  1.8e-15  8.2e-15
  19  1.1513e-05  1.7379e-06       43    22    43     2     0      0  1.6e-15  8.2e-15
  20  1.3251e-05  1.7379e-06       45    23    45     2     0      0  1.3e-15  7.4e-15
  21  1.4989e-05  1.7379e-06       47    24    47     2     0      0  1.7e-15  6.6e-15
  22  1.8465e-05  3.4757e-06       49    25    49     2     0      0  1.7e-15  9.9e-15
  23   2.194e-05  3.4757e-06       51    26    51     2     0      0  1.7e-15  8.5e-15
  24  2.5416e-05  3.4757e-06       53    27    53     2     0      0  1.5e-15  8.6e-15
  25  2.8892e-05  3.4757e-06       55    28    55     2     0      0  1.6e-15    8e-15
  26  3.5843e-05  6.9514e-06       57    29    57     2     0      0  1.5e-15  1.2e-14
  27  4.2795e-05  6.9514e-06       59    30    59     2     0      0  1.8e-15  9.8e-15
  28  4.9746e-05  6.9514e-06       61    31    61     2     0      0  1.4e-15  9.9e-15
  29  5.6697e-05  6.9514e-06       63    32    63     2     0      0  1.6e-15  9.2e-15
  30    7.06e-05  1.3903e-05       65    33    65     2     0      0  1.7e-15  1.3e-14
  31  8.4503e-05  1.3903e-05       67    34    67     2     0      0  1.7e-15  1.1e-14
  32  9.8406e-05  1.3903e-05       69    35    69     2     0      0  1.7e-15  1.1e-14
  33  0.00011231  1.3903e-05       71    36    71     2     0      0  1.9e-15    1e-14
  34  0.00014011  2.7806e-05       73    37    73     2     0      0  2.4e-15  1.5e-14
  35  0.00016792  2.7806e-05       75    38    75     2     0      0  2.2e-15  1.2e-14
  36  0.00019573  2.7806e-05       77    39    77     2     0      0  2.1e-15  1.2e-14
  37  0.00022353  2.7806e-05       79    40    79     2     0      0  1.9e-15  1.1e-14
  38  0.00027914  5.5611e-05       81    41    81     2     0      0  2.4e-15  1.6e-14
  39  0.00033475  5.5611e-05       83    42    83     2     0      0  2.5e-15  1.4e-14
  40  0.00039036  5.5611e-05       85    43    85     2     0      0  1.2e-15  1.4e-14
  41  0.00050159  0.00011122       87    44    87     2     0      0  2.7e-15  2.1e-14
  42  0.00061281  0.00011122       89    45    89     2     0      0  2.9e-15  1.7e-14
  43  0.00072403  0.00011122       91    46    91     2     0      0  1.4e-15  1.7e-14
```

44	0.00083525	0.00011122		93	47	93	2	0	0	2.3e-15	1.5e-14
45	0.0010577	0.00022245		95	48	95	2	0	0	2.1e-15	2.1e-14
46	0.0012801	0.00022245		97	49	97	2	0	0	4.5e-15	1.6e-14
47	0.001725	0.00044489		99	50	99	2	0	0	2.1e-15	2.7e-14
48	0.0021699	0.00044489		101	51	101	2	0	0	4.9e-15	2.4e-14
49	0.0026148	0.00044489		103	52	103	2	0	0	2.1e-15	2.3e-14
50	0.0030597	0.00044489		105	53	105	2	0	0	3.5e-15	2e-14
51	0.0039495	0.00088978		107	54	107	2	0	0	1.4e-15	2.3e-14
52	0.0048393	0.00088978		109	55	109	2	0	0	1.9e-15	1.7e-14
-	0.005	-	out								
53	0.0066188	0.0017796		111	56	111	2	0	0	2.8e-15	3e-14
54	0.0083984	0.0017796		113	57	113	2	0	0	2.5e-15	2.6e-14
-	0.01	-	out								
55	0.011958	0.0035591		115	58	115	2	0	0	8.9e-15	3.7e-14
-	0.015	-	out								
56	0.015517	0.0035591		117	59	117	1	0	0	6.6e-15	4.1e-14
57	0.019076	0.0035591		119	60	119	1	0	0	2.9e-15	2.4e-14
-	0.02	-	out								
-	0.025	-	out								
58	0.026194	0.0071182		121	61	121	1	0	0	6.7e-15	3.6e-14
-	0.03	-	out								
-	0.035	-	out								
-	0.04	-	out								
59	0.04043	0.014236		123	62	123	1	0	0	3.7e-15	5.2e-14
-	0.045	-	out								
-	0.05	-	out								
60	0.054667	0.014236		125	63	125	1	0	0	5.5e-15	5e-14
-	0.055	-	out								
-	0.06	-	out								
-	0.065	-	out								
-	0.07	-	out								
-	0.075	-	out								
-	0.08	-	out								
61	0.08314	0.028473		127	64	127	1	0	0	3.9e-15	7e-14
-	0.085	-	out								
-	0.09	-	out								
-	0.095	-	out								
-	0.1	-	out								
-	0.105	-	out								
-	0.11	-	out								
62	0.11161	0.028473		129	65	129	1	0	0	8e-15	6.3e-14
-	0.115	-	out								
-	0.12	-	out								
-	0.125	-	out								
-	0.13	-	out								
-	0.135	-	out								
-	0.14	-	out								
-	0.145	-	out								
-	0.15	-	out								
-	0.155	-	out								
-	0.16	-	out								
-	0.165	-	out								
63	0.16856	0.056946		131	66	131	1	0	0	2.9e-15	8.4e-14
-	0.17	-	out								
-	0.175	-	out								
-	0.18	-	out								

-	0.185	-	out							
-	0.19	-	out							
-	0.195	-	out							
-	0.2	-	out							
-	0.205	-	out							
-	0.21	-	out							
-	0.215	-	out							
-	0.22	-	out							
-	0.225	-	out							
-	0.23	-	out							
-	0.235	-	out							
-	0.24	-	out							
-	0.245	-	out							
-	0.25	-	out							
-	0.25	-	out							
64	0.26756	0.099	133	67	133	1	0	0	7e-15	9.3e-14
-	0.27	-	out							
-	0.29	-	out							
-	0.31	-	out							
-	0.33	-	out							
-	0.35	-	out							
65	0.36656	0.099	135	68	135	1	0	0	1.1e-14	7.1e-14
-	0.37	-	out							
-	0.39	-	out							
-	0.41	-	out							
-	0.43	-	out							
-	0.45	-	out							
66	0.46556	0.099	137	69	137	1	0	0	3.9e-15	5.6e-14
-	0.47	-	out							
-	0.49	-	out							
-	0.51	-	out							
-	0.53	-	out							
-	0.55	-	out							
67	0.56456	0.099	139	70	139	1	0	0	5.9e-15	4.3e-14
-	0.57	-	out							
-	0.59	-	out							
-	0.61	-	out							
-	0.63	-	out							
-	0.65	-	out							
68	0.66356	0.099	141	71	141	1	0	0	1.1e-14	3.5e-14
-	0.67	-	out							
-	0.69	-	out							
-	0.71	-	out							
-	0.73	-	out							
-	0.75	-	out							
69	0.76256	0.099	142	72	142	1	0	0	1.1e-14	2.7e-14
-	0.77	-	out							
-	0.79	-	out							
-	0.81	-	out							
-	0.83	-	out							
-	0.85	-	out							
70	0.86156	0.099	143	73	143	1	0	0	6.9e-15	2.5e-14
-	0.87	-	out							
-	0.89	-	out							
-	0.91	-	out							
-	0.93	-	out							

```
   -       0.95           - out
  71     0.96056       0.099      144   74  144     2     0      0  5.7e-15    2e-14
   -       0.97           - out
   -       0.99           - out
  72      1.0596       0.099      145   75  145     2     0      0    1e-14  1.2e-14
Time-stepping completed.
Solution time: 15 s.
Physical memory: 1.4 GB
Virtual memory: 1.38 GB
Ended at May 28, 2023, 4:28:33 PM.
----- Time-Dependent Solver 1 in Oxygen Pulse/Solution 958 (sol958) ----------->
```

4 Results

4.1 DATA SETS

4.1.1 Probe Solution 2

SOLUTION

Description	Value
Solution	Solution 958
Component	Component 2 (comp2)

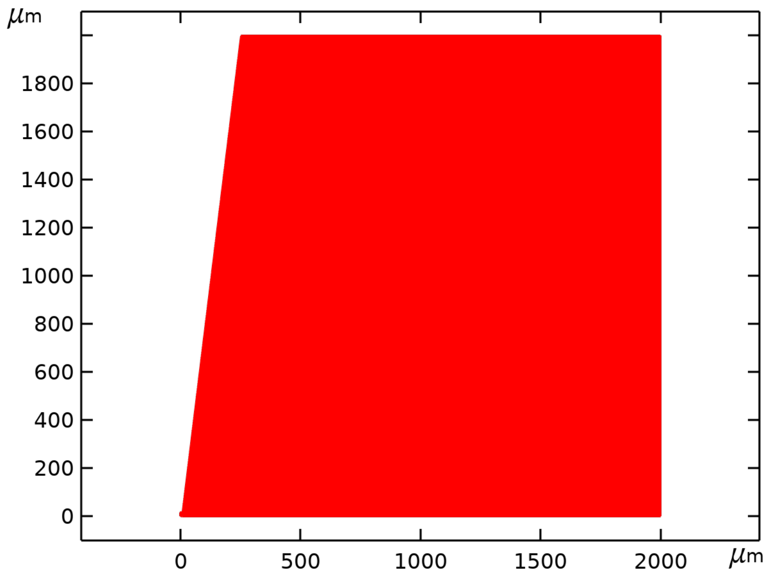

Dataset: Probe Solution 2

4.1.2 UME

SELECTION

Geometric entity level	Boundary
Selection	Geometry geom2: Dimension 1: Boundary 3

DATA

Description	Value
Dataset	Probe Solution 2

SETTINGS

Description	Value
Method	Integration
Integration order	4
Integration order	On

4.1.3 OxiMem

SELECTION

Geometric entity level	Boundary
Selection	Geometry geom2: Dimension 1: No boundaries

DATA

Description	Value
Dataset	Probe Solution 2

SETTINGS

Description	Value
Method	Integration
Integration order	4
Integration order	On

4.1.4 RedProbe

SELECTION

Geometric entity level	Domain
Selection	Geometry geom2

DATA

Description	Value
Dataset	Probe Solution 2

SETTINGS

Description	Value
Method	Integration
Integration order	4
Integration order	On

4.1.5 OxiNuc

SELECTION

Geometric entity level	Boundary
Selection	Geometry geom2: Dimension 1: Boundaries 10–11

DATA

Description	Value
Dataset	Probe Solution 2

SETTINGS

Description	Value
Method	Integration
Integration order	4

Description	Value
Integration order	On

4.1.6 RedSub

SELECTION

Geometric entity level	Domain
Selection	Geometry geom2

DATA

Description	Value
Dataset	Probe Solution 2

SETTINGS

Description	Value
Method	Integration
Integration order	4
Integration order	On

4.1.7 Oxygen Pulse/Solution 958

SOLUTION

Description	Value
Solution	Solution 958
Component	Component 2 (comp2)

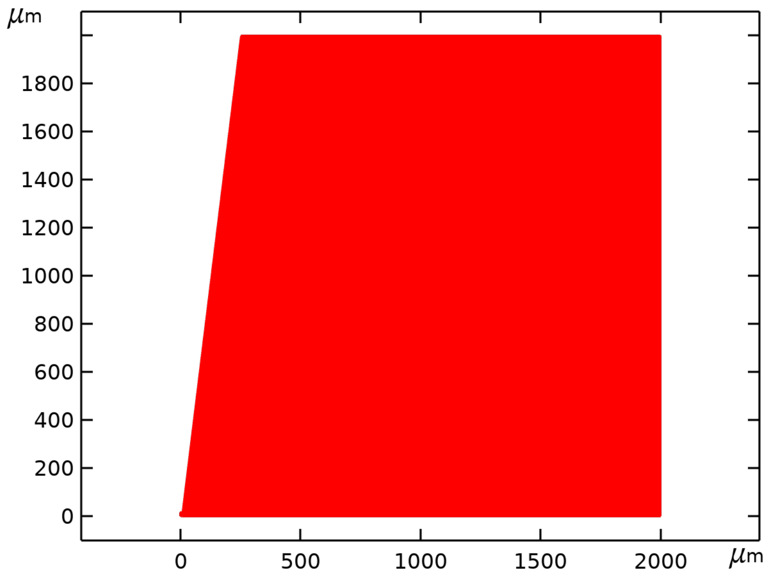

Dataset: Oxygen Pulse/Solution 958

4.1.8 Oxygen Pulse/Parametric Solutions 4

SOLUTION

Description	Value
Solution	Parametric Solutions 4
Component	Component 2 (comp2)

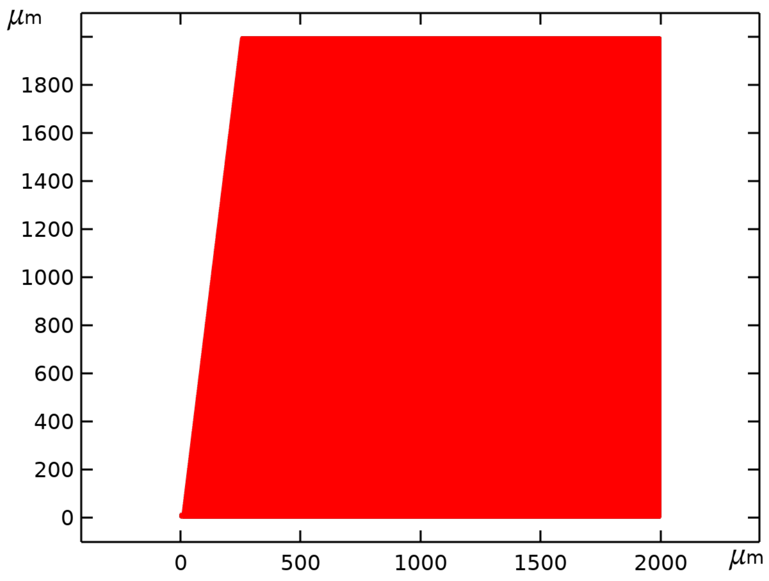

Dataset: Oxygen Pulse/Parametric Solutions 4

4.1.9 Revolution 2D 4

DATA

Description	Value
Dataset	Oxygen Pulse/Parametric Solutions 4

AXIS DATA

Description	Value
Axis entry method	Two points
Points	{{0, 0}, {0, 1}}

REVOLUTION LAYERS

Description	Value
Start angle	-90
Revolution angle	225

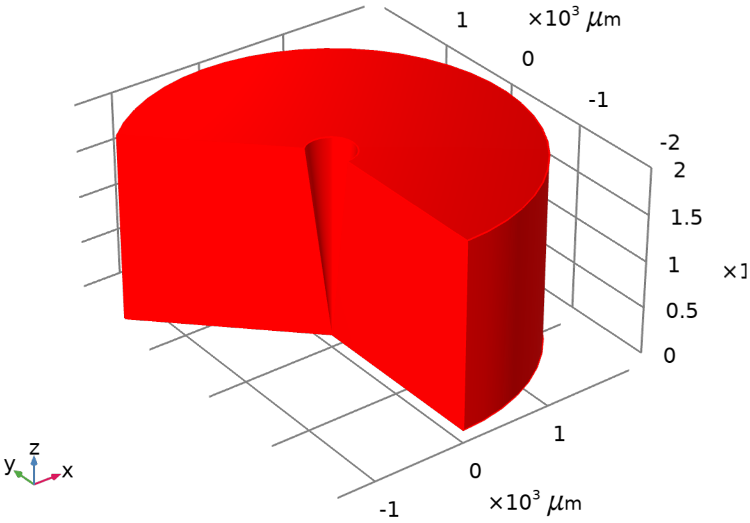

Dataset: Revolution 2D 4

4.1.10 Oxygen Pulse/Solution Store 1

SOLUTION

Description	Value
Solution	Solution Store 1
Component	Component 2 (comp2)

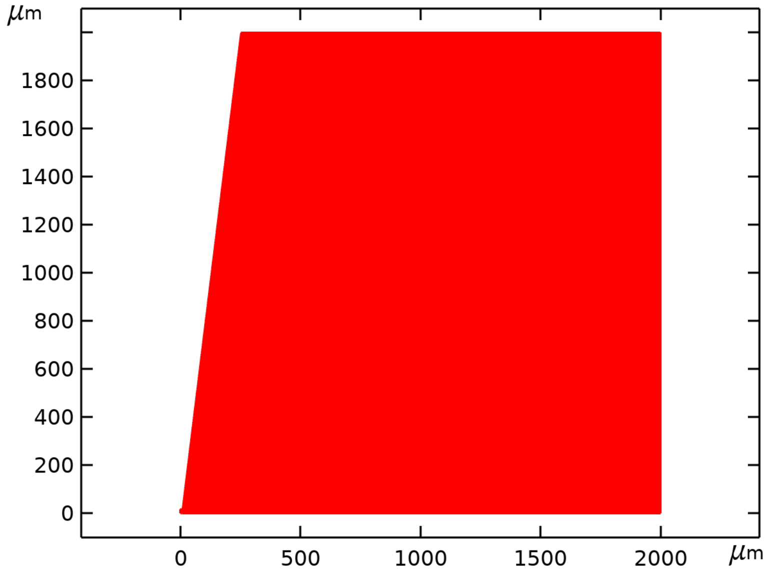

Dataset: Oxygen Pulse/Solution Store 1

4.1.11 CellToUME

DATA

Description	Value

Description	Value
Dataset	Oxygen Pulse/Parametric Solutions 4

LINE DATA

Description	Value
Line entry method	Two points
Points	{{0, CellHeight}, {0, CellHeight + 100}}
Bounded by points	Off

ADVANCED

Description	Value
Space variable	cln2x
Normal variables	{cln2nx, cln2ny}
Tangent variables	{cln2tx, cln2ty}

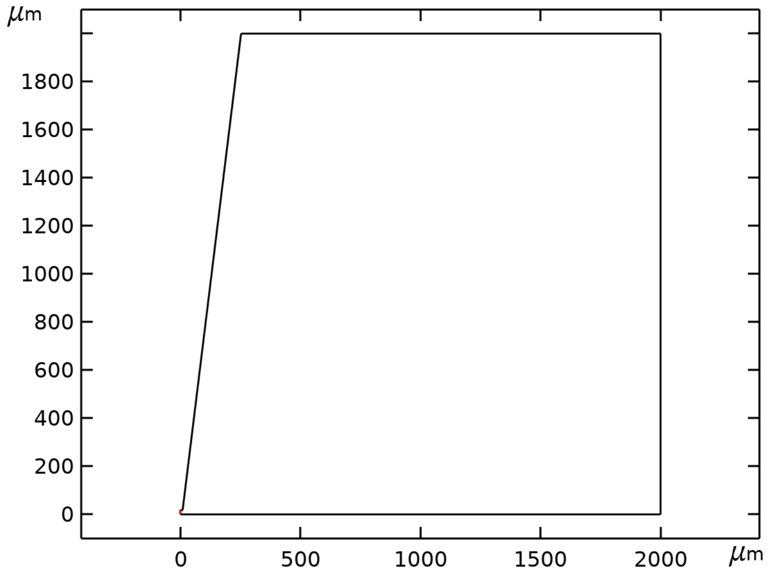

Dataset: CellToUME

4.1.12 UMEOCRmembrane

SELECTION

Geometric entity level	Boundary
Selection	Geometry geom2: Dimension 1: Boundary 11

DATA

Description	Value
Dataset	Probe Solution 2

SETTINGS

Description	Value
Method	Integration

Description	Value
Integration order	4
Integration order	On

4.1.13 UMEOCRnucleus

SELECTION

Geometric entity level	Boundary
Selection	Geometry geom2: Dimension 1: Boundary 10

DATA

Description	Value
Dataset	Probe Solution 2

SETTINGS

Description	Value
Method	Integration
Integration order	4
Integration order	On

4.1.14 No Solution (18)

SOLUTION

Description	Value
Component	Component 2 (comp2)

4.1.15 Revolution 2D 6

DATA

Description	Value
Dataset	No Solution (18)

AXIS DATA

Description	Value
Axis entry method	Two points
Points	{{0, 0}, {0, 1}}

REVOLUTION LAYERS

Description	Value
Start angle	-90
Revolution angle	225

4.1.16 No Solution (20)

SOLUTION

Description	Value

Description	Value
Component	Component 2 (comp2)

4.1.17 Revolution 2D 7

DATA

Description	Value
Dataset	No Solution (20)

AXIS DATA

Description	Value
Axis entry method	Two points
Points	{{0, 0}, {0, 1}}

REVOLUTION LAYERS

Description	Value
Start angle	-90
Revolution angle	225

4.1.18 Probe Solution 28

SOLUTION

Description	Value
Solution	Solution 958
Component	Component 2 (comp2)
Frame	Spatial (r, phi, z)

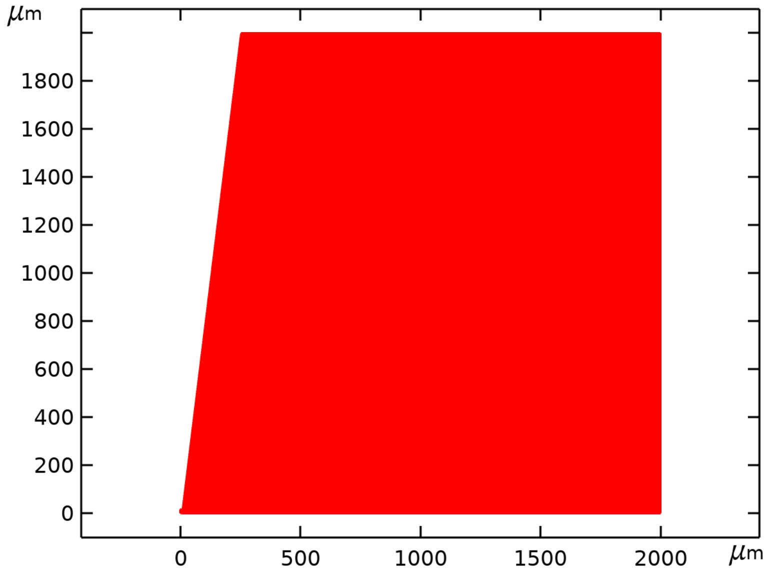

Dataset: Probe Solution 28

4.1.19 Domain Point Probe 1

DATA

Description	Value
Dataset	Probe Solution 28

POINT DATA

Description	Value
Entry method	Coordinates
r	0
z	CellHeight
Snapping	Snap to closest boundary

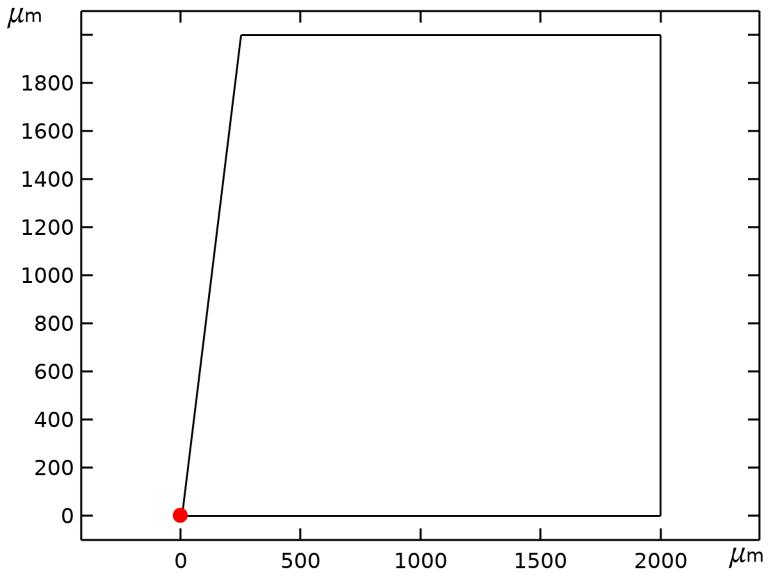

Dataset: Domain Point Probe 1

4.1.20 MemFlux

SELECTION

Geometric entity level	Boundary
Selection	Geometry geom2: Dimension 1: Boundary 11

DATA

Description	Value
Dataset	Probe Solution 2

SETTINGS

Description	Value
Method	Integration
Integration order	4
Integration order	On

4.1.21 NucFlux

SELECTION

Geometric entity level	Boundary
Selection	Geometry geom2: Dimension 1: Boundary 10

DATA

Description	Value
Dataset	Probe Solution 2

SETTINGS

Description	Value
Method	Integration
Integration order	4
Integration order	On

4.1.22 UMEToCell

DATA

Description	Value
Dataset	Oxygen Pulse/Parametric Solutions 4

LINE DATA

Description	Value
Line entry method	Two points
Points	{{0, d2}, {0, d2 - 100}}
Bounded by points	Off

ADVANCED

Description	Value
Space variable	cln2x
Normal variables	{cln2nx, cln2ny}
Tangent variables	{cln3tx, cln3ty}

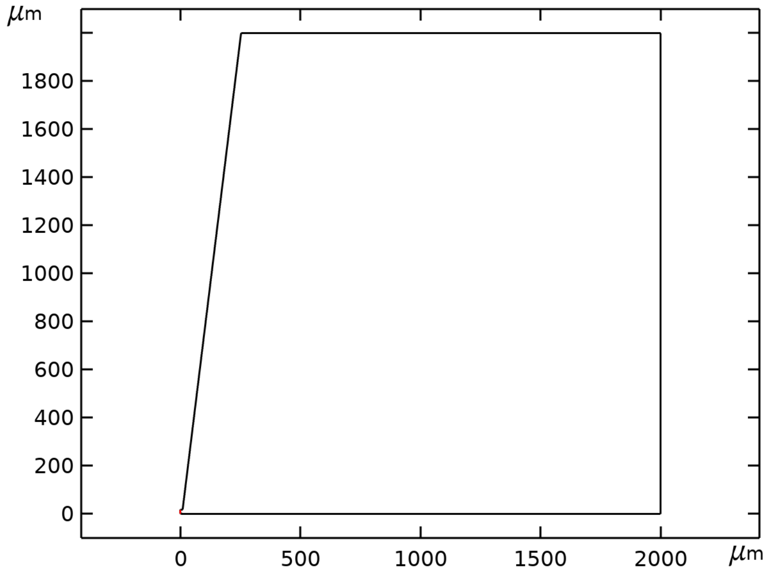

Dataset: UMEToCell

4.2 DERIVED VALUES

4.2.1 Global Variable Probe 2

OUTPUT

Evaluated in	Accumulated Probe Table 10

DATA

Description	Value
Dataset	Probe Solution 2

EXPRESSIONS

Expression	Unit	Description
Potential(t)	V	Interpolation 1

4.2.2 UME

OUTPUT

Evaluated in	Accumulated Probe Table 10

DATA

Description	Value
Dataset	UME

EXPRESSIONS

Expression	Unit	Description
-(tds2.ntflux_Oxi)*n*F	A	

4.2.3 OxiMem

OUTPUT

Evaluated in	Accumulated Probe Table 10

DATA

Description	Value
Dataset	OxiMem

EXPRESSIONS

Expression	Unit	Description
Oxi	M	SurfaceConcOxi

4.2.4 RedProbe

DATA

Description	Value
Dataset	RedProbe

EXPRESSIONS

Expression	Unit	Description
Red		SurfaceConcRed

4.2.5 OxiNuc

OUTPUT

Evaluated in	Accumulated Probe Table 10

DATA

Description	Value
Dataset	OxiNuc

EXPRESSIONS

Expression	Unit	Description
Oxi	M	SurfaceConcOxi

4.2.6 RedSub

DATA

Description	Value
Dataset	RedSub

EXPRESSIONS

Expression	Unit	Description
Red		SurfaceConcRed

4.2.7 Substrate Potential

OUTPUT

Evaluated in	Probe Table 7

DATA

Description	Value
Dataset	Probe Solution 2

EXPRESSIONS

Expression	Unit	Description
Epulse	V	Tip potential during pulse

4.2.8 k0Sub probe

OUTPUT

Evaluated in	Accumulated Probe Table 10

DATA

Description	Value
Dataset	Probe Solution 2

EXPRESSIONS

Expression	Unit	Description
k0Sub	m/s	Substrate Kinetics

4.2.9 UMEOCRmembrane

OUTPUT

Evaluated in	Accumulated Probe Table 10

DATA

Description	Value
Dataset	UMEOCRmembrane

EXPRESSIONS

Expression	Unit	Description
tds2.ntflux_Oxi	mol/s	Normal total flux

4.2.10 UMEOCRnucleus

OUTPUT

Evaluated in	Accumulated Probe Table 10

DATA

Description	Value
Dataset	UMEOCRnucleus

EXPRESSIONS

Expression	Unit	Description
tds2.tflux_Oxiz	mol/s	Normal total flux

4.2.11 CellTopConc

OUTPUT

Evaluated in	Accumulated Probe Table 10

DATA

Description	Value
Dataset	Domain Point Probe 1

EXPRESSIONS

Expression	Unit	Description
Oxi	mol/m^3	Concentration

4.2.12 MemFlux

OUTPUT

Evaluated in	Accumulated Probe Table 10

DATA

Description	Value
Dataset	MemFlux

EXPRESSIONS

Expression	Unit	Description
k0Sub*Oxi	mol/s	

4.2.13 NucFlux

OUTPUT

Evaluated in	Accumulated Probe Table 10

DATA

Description	Value
Dataset	NucFlux

EXPRESSIONS

Expression	Unit	Description
k0Sub*Oxi	mol/s	

4.2.14 CellTopFlux

OUTPUT

Evaluated in	Accumulated Probe Table 10

DATA

Description	Value
Dataset	Domain Point Probe 1

EXPRESSIONS

Expression	Unit	Description
tds2.tflux_Oxiz	mol/(m^2*s)	Normal total flux

4.2.15 UMERadius

OUTPUT

Evaluated in	Accumulated Probe Table 10

DATA

Description	Value
Dataset	Probe Solution 2

EXPRESSIONS

Expression	Unit	Description
Rb	µm	WElectrode radius

4.3 TABLES

4.3.1 Probe Table 1

Time (s)	-(tds2.ntflux_Oxi)*n*F (A), UME	SurfaceConcOxi (M), OxiMem	SurfaceConcOxi (M), OxiNuc	SubstrateKinetics (m/s)	Normal total flux (mol/s), UME OCR membrane	Normal total flux, UME OCRnucleus	Concentration (mol/m^3), Point: (0, CellHeight)	k0Sub*Oxi (mol/s), MemFlux	k0Sub*Oxi (mol/s), NucFlux	Normal total flux (mol/(m^2*s)), Point: (0, CellHeight)	WElectrode radius (µm)
0.0000	-6.8604E-9	NaN	2.0322E-4	1.5100E-4	-1.6547E-15	-1.0933E-15	0.19181	9.1907E-15	1.6547E-15	-1.8596E-5	1.0000
2.7154E-8	-6.7302E-9	NaN	2.0322E-4	1.5100E-4	-1.6547E-15	-1.0933E-15	0.19181	9.1907E-15	1.6547E-15	-1.8596E-5	1.0000
5.4308E-8	-6.6195E-9	NaN	2.0322E-4	1.5100E-4	-1.6547E-15	-1.0933E-15	0.19181	9.1907E-15	1.6547E-15	-1.8596E-5	1.0000
1.0862E-7	-6.4250E-9	NaN	2.0322E-4	1.5100E-4	-1.6547E-15	-1.0933E-15	0.19181	9.1907E-15	1.6547E-15	-1.8596E-5	1.0000
2.1723E-7	-6.116	NaN	2.0322E-4	1.5100E-4	-1.654	-1.093	0.19181	9.1907E-15	1.6547E-15	-1.859	1.0000

Time (s)	-(tds2.ntflux_Oxi)*n*F (A), UME	SurfaceConcOxi (M), OxiMem	SurfaceConcOxi (M), OxiNuc	Substrate Kinetics (m/s)	Normal total flux (mol/s), UME OCR membrane	Normal total flux, UME OCRnucleus	Concentration (mol/m^3), Point: (0, CellHeight)	k0Sub*Oxi (mol/s), MemFlux	k0Sub*Oxi (mol/s), NucFlux	Normal total flux (mol/(m^2*s)), Point: (0, CellHeight)	WElectrode radius (µm)
	4E-9				7E-15	3E-15				6E-5	
4.3446E-7	-5.6598E-9	NaN	2.0322E-4	1.5100E-4	-1.6547E-15	-1.0933E-15	0.19181	9.1907E-15	1.6547E-15	-1.8596E-5	1.0000
6.5169E-7	-5.3349E-9	NaN	2.0322E-4	1.5100E-4	-1.6547E-15	-1.0933E-15	0.19181	9.1907E-15	1.6547E-15	-1.8596E-5	1.0000
8.6893E-7	-5.0809E-9	NaN	2.0322E-4	1.5100E-4	-1.6547E-15	-1.0933E-15	0.19181	9.1907E-15	1.6547E-15	-1.8596E-5	1.0000
1.0862E-6	-4.8706E-9	NaN	2.0322E-4	1.5100E-4	-1.6547E-15	-1.0933E-15	0.19181	9.1907E-15	1.6547E-15	-1.8596E-5	1.0000
1.5206E-6	-4.5267E-9	NaN	2.0322E-4	1.5100E-4	-1.6547E-15	-1.0933E-15	0.19181	9.1907E-15	1.6547E-15	-1.8596E-5	1.0000
1.9551E-6	-4.2621E-9	NaN	2.0322E-4	1.5100E-4	-1.6547E-15	-1.0933E-15	0.19181	9.1907E-15	1.6547E-15	-1.8596E-5	1.0000
2.3895E-6	-4.0500E-9	NaN	2.0322E-4	1.5100E-4	-1.6547E-15	-1.0933E-15	0.19181	9.1907E-15	1.6547E-15	-1.8596E-5	1.0000
2.8240E-6	-3.8740E-9	NaN	2.0322E-4	1.5100E-4	-1.6547E-15	-1.0933E-15	0.19181	9.1907E-15	1.6547E-15	-1.8596E-5	1.0000
3.6929E-6	-3.5878E-9	NaN	2.0322E-4	1.5100E-4	-1.6547E-15	-1.0933E-15	0.19181	9.1907E-15	1.6547E-15	-1.8596E-5	1.0000
4.5619E-6	-3.3690E-9	NaN	2.0322E-4	1.5100E-4	-1.6547E-15	-1.0933E-15	0.19181	9.1907E-15	1.6547E-15	-1.8596E-5	1.0000
5.4308E-6	-3.1951E-9	NaN	2.0322E-4	1.5100E-4	-1.6547E-15	-1.0933E-15	0.19181	9.1907E-15	1.6547E-15	-1.8596E-5	1.0000
6.2997E-6	-3.0521E-9	NaN	2.0322E-4	1.5100E-4	-1.6547E-15	-1.0933E-15	0.19181	9.1907E-15	1.6547E-15	-1.8596E-5	1.0000
8.0376E-6	-2.8221E-9	NaN	2.0322E-4	1.5100E-4	-1.6547E-15	-1.0933E-15	0.19181	9.1907E-15	1.6547E-15	-1.8596E-5	1.0000

Time (s)	-(tds2.ntflux_Oxi)*n*F (A), UME	SurfaceConcOxi (M), OxiMem	SurfaceConcOxi (M), OxiNuc	Substrate Kinetics (m/s)	Normal total flux (mol/s), UME OCR membrane	Normal total flux, UME OCRnucleus	Concentration (mol/m^3), Point: (0, CellHeight)	k0Sub*Oxi (mol/s), MemFlux	k0Sub*Oxi (mol/s), NucFlux	Normal total flux (mol/(m^2*s)), Point: (0, CellHeight)	WElectrode radius (µm)
9.7754E-6	-2.6488E-9	NaN	2.0322E-4	1.5100E-4	-1.6547E-15	-1.0933E-15	0.19181	9.1907E-15	1.6547E-15	-1.8596E-5	1.0000
1.1513E-5	-2.5129E-9	NaN	2.0322E-4	1.5100E-4	-1.6547E-15	-1.0933E-15	0.19181	9.1907E-15	1.6547E-15	-1.8596E-5	1.0000
1.3251E-5	-2.4023E-9	NaN	2.0322E-4	1.5100E-4	-1.6547E-15	-1.0933E-15	0.19181	9.1907E-15	1.6547E-15	-1.8596E-5	1.0000
1.4989E-5	-2.3097E-9	NaN	2.0322E-4	1.5100E-4	-1.6547E-15	-1.0933E-15	0.19181	9.1907E-15	1.6547E-15	-1.8596E-5	1.0000
1.8465E-5	-2.1589E-9	NaN	2.0322E-4	1.5100E-4	-1.6547E-15	-1.0933E-15	0.19181	9.1907E-15	1.6547E-15	-1.8596E-5	1.0000
2.1940E-5	-2.0434E-9	NaN	2.0322E-4	1.5100E-4	-1.6547E-15	-1.0933E-15	0.19181	9.1907E-15	1.6547E-15	-1.8596E-5	1.0000
2.5416E-5	-1.9519E-9	NaN	2.0322E-4	1.5100E-4	-1.6547E-15	-1.0933E-15	0.19181	9.1907E-15	1.6547E-15	-1.8596E-5	1.0000
2.8892E-5	-1.8770E-9	NaN	2.0322E-4	1.5100E-4	-1.6547E-15	-1.0933E-15	0.19181	9.1907E-15	1.6547E-15	-1.8596E-5	1.0000
3.5843E-5	-1.7574E-9	NaN	2.0322E-4	1.5100E-4	-1.6547E-15	-1.0933E-15	0.19181	9.1907E-15	1.6547E-15	-1.8596E-5	1.0000
4.2795E-5	-1.6682E-9	NaN	2.0322E-4	1.5100E-4	-1.6547E-15	-1.0933E-15	0.19181	9.1907E-15	1.6547E-15	-1.8596E-5	1.0000
4.9746E-5	-1.5989E-9	NaN	2.0322E-4	1.5100E-4	-1.6547E-15	-1.0933E-15	0.19181	9.1907E-15	1.6547E-15	-1.8596E-5	1.0000
5.6697E-5	-1.5430E-9	NaN	2.0322E-4	1.5100E-4	-1.6547E-15	-1.0933E-15	0.19181	9.1907E-15	1.6547E-15	-1.8596E-5	1.0000
7.0600E-5	-1.4546E-9	NaN	2.0322E-4	1.5100E-4	-1.6547E-15	-1.0933E-15	0.19181	9.1907E-15	1.6547E-15	-1.8596E-5	1.0000
8.450	-	NaN	2.032	1.510	-	-	0.191	9.190	1.654	-	1.000

Time (s)	-(tds2.ntflux_Oxi)*n*F (A), UME	SurfaceConcOxi (M), OxiMem	SurfaceConcOxi (M), OxiNuc	Substrate Kinetics (m/s)	Normal total flux (mol/s), UME OCR membrane	Normal total flux, UME OCRnucleus	Concentration (mol/m^3), Point: (0, CellHeight)	k0Sub*Oxi (mol/s), MemFlux	k0Sub*Oxi (mol/s), NucFlux	Normal total flux (mol/(m^2*s)), Point: (0, CellHeight)	WElectrode radius (µm)
3E-5	1.3897E-9		2E-4	0E-4	1.6547E-15	1.0933E-15	81	7E-15	7E-15	1.8596E-5	0
9.8406E-5	-1.3399E-9	NaN	2.0322E-4	1.5100E-4	-1.6547E-15	-1.0933E-15	0.19181	9.1907E-15	1.6547E-15	-1.8596E-5	1.0000
1.1231E-4	-1.3001E-9	NaN	2.0322E-4	1.5100E-4	-1.6547E-15	-1.0933E-15	0.19181	9.1907E-15	1.6547E-15	-1.8596E-5	1.0000
1.4011E-4	-1.2376E-9	NaN	2.0322E-4	1.5100E-4	-1.6547E-15	-1.0933E-15	0.19181	9.1907E-15	1.6547E-15	-1.8596E-5	1.0000
1.6792E-4	-1.1922E-9	NaN	2.0322E-4	1.5100E-4	-1.6547E-15	-1.0933E-15	0.19181	9.1907E-15	1.6547E-15	-1.8596E-5	1.0000
1.9573E-4	-1.1576E-9	NaN	2.0322E-4	1.5100E-4	-1.6547E-15	-1.0933E-15	0.19181	9.1907E-15	1.6547E-15	-1.8596E-5	1.0000
2.2353E-4	-1.1300E-9	NaN	2.0322E-4	1.5100E-4	-1.6547E-15	-1.0933E-15	0.19181	9.1907E-15	1.6547E-15	-1.8596E-5	1.0000
2.7914E-4	-1.0869E-9	NaN	2.0322E-4	1.5100E-4	-1.6547E-15	-1.0933E-15	0.19181	9.1907E-15	1.6547E-15	-1.8596E-5	1.0000
3.3475E-4	-1.0556E-9	NaN	2.0322E-4	1.5100E-4	-1.6547E-15	-1.0933E-15	0.19181	9.1907E-15	1.6547E-15	-1.8596E-5	1.0000
3.9036E-4	-1.0318E-9	NaN	2.0322E-4	1.5100E-4	-1.6547E-15	-1.0933E-15	0.19181	9.1907E-15	1.6547E-15	-1.8596E-5	1.0000
5.0159E-4	-9.9581E-10	NaN	2.0322E-4	1.5100E-4	-1.6547E-15	-1.0933E-15	0.19181	9.1907E-15	1.6547E-15	-1.8596E-5	1.0000
6.1281E-4	-9.7087E-10	NaN	2.0322E-4	1.5100E-4	-1.6547E-15	-1.0933E-15	0.19181	9.1907E-15	1.6547E-15	-1.8596E-5	1.0000
7.2403E-4	-9.5242E-10	NaN	2.0322E-4	1.5100E-4	-1.6547E-15	-1.0933E-15	0.19181	9.1907E-15	1.6547E-15	-1.8596E-5	1.0000
8.3525E-4	-9.379	NaN	2.0322E-4	1.5100E-4	-1.654	-1.093	0.19181	9.1907E-15	1.6547E-15	-1.859	1.0000

Time (s)	-(tds2.ntflux_Oxi)*n*F (A), UME	SurfaceConcOxi (M), OxiMem	SurfaceConcOxi (M), OxiNuc	Substrate Kinetics (m/s)	Normal total flux (mol/s), UME OCR membrane	Normal total flux, UME OCRnucleus	Concentration (mol/m^3), Point: (0, CellHeight)	k0Sub*Oxi (mol/s), MemFlux	k0Sub*Oxi (mol/s), NucFlux	Normal total flux (mol/(m^2*s)), Point: (0, CellHeight)	WElectrode radius (µm)
	6E-10				7E-15	3E-15				6E-5	
0.0010577	-9.1550E-10	NaN	2.0322E-4	1.5100E-4	-1.6547E-15	-1.0933E-15	0.19181	9.1907E-15	1.6547E-15	-1.8596E-5	1.0000
0.0012801	-8.9941E-10	NaN	2.0322E-4	1.5100E-4	-1.6547E-15	-1.0933E-15	0.19181	9.1907E-15	1.6547E-15	-1.8596E-5	1.0000
0.0017250	-8.7642E-10	NaN	2.0322E-4	1.5100E-4	-1.6547E-15	-1.0933E-15	0.19181	9.1907E-15	1.6547E-15	-1.8596E-5	1.0000
0.0021699	-8.6160E-10	NaN	2.0322E-4	1.5100E-4	-1.6547E-15	-1.0933E-15	0.19181	9.1907E-15	1.6547E-15	-1.8596E-5	1.0000
0.0026148	-8.5113E-10	NaN	2.0322E-4	1.5100E-4	-1.6547E-15	-1.0933E-15	0.19181	9.1907E-15	1.6547E-15	-1.8596E-5	1.0000
0.0030597	-8.4309E-10	NaN	2.0322E-4	1.5100E-4	-1.6546E-15	-1.0933E-15	0.19181	9.1907E-15	1.6547E-15	-1.8594E-5	1.0000
0.0039495	-8.3076E-10	NaN	2.0321E-4	1.5100E-4	-1.6540E-15	-1.0932E-15	0.19181	9.1906E-15	1.6547E-15	-1.8588E-5	1.0000
0.0048393	-8.2209E-10	NaN	2.0321E-4	1.5100E-4	-1.6529E-15	-1.0931E-15	0.19179	9.1905E-15	1.6547E-15	-1.8576E-5	1.0000
0.0066188	-8.0988E-10	NaN	2.0319E-4	1.5100E-4	-1.6484E-15	-1.0925E-15	0.19174	9.1895E-15	1.6544E-15	-1.8534E-5	1.0000
0.0083984	-8.0219E-10	NaN	2.0314E-4	1.5100E-4	-1.6420E-15	-1.0914E-15	0.19163	9.1875E-15	1.6539E-15	-1.8476E-5	1.0000
0.011958	-7.9197E-10	NaN	2.0297E-4	1.5100E-4	-1.6289E-15	-1.0884E-15	0.19133	9.1801E-15	1.6521E-15	-1.8356E-5	1.0000
0.015517	-7.8731E-10	NaN	2.0269E-4	1.5100E-4	-1.6200E-15	-1.0849E-15	0.19093	9.1682E-15	1.6493E-15	-1.8256E-5	1.0000
0.019076	-7.8367E-10	NaN	2.0240E-4	1.5100E-4	-1.6145E-15	-1.0817E-15	0.19054	9.1553E-15	1.6465E-15	-1.8178E-5	1.0000

Time (s)	-(tds2.ntflux_Oxi)*n*F (A), UME	SurfaceConcOxi (M), OxiMem	SurfaceConcOxi (M), OxiNuc	Substrate Kinetics (m/s)	Normal total flux (mol/s), UME OCR membrane	Normal total flux, UME OCRnucleus	Concentration (mol/m^3), Point: (0, CellHeight)	k0Sub*Oxi (mol/s), MemFlux	k0Sub*Oxi (mol/s), NucFlux	Normal total flux (mol/(m^2*s)), Point: (0, CellHeight)	WElectrode radius (µm)
0.026194	-7.7879E-10	NaN	2.0182E-4	1.5100E-4	-1.6097E-15	-1.0765E-15	0.18984	9.1298E-15	1.6411E-15	-1.8071E-5	1.0000
0.040430	-7.7325E-10	NaN	2.0087E-4	1.5100E-4	-1.6069E-15	-1.0694E-15	0.18879	9.0878E-15	1.6326E-15	-1.7941E-5	1.0000
0.054667	-7.6975E-10	NaN	2.0013E-4	1.5100E-4	-1.6059E-15	-1.0643E-15	0.18800	9.0546E-15	1.6260E-15	-1.7856E-5	1.0000
0.083140	-7.6576E-10	NaN	1.9914E-4	1.5100E-4	-1.6043E-15	-1.0581E-15	0.18699	9.0105E-15	1.6176E-15	-1.7757E-5	1.0000
0.11161	-7.6319E-10	NaN	1.9846E-4	1.5100E-4	-1.6027E-15	-1.0540E-15	0.18631	8.9799E-15	1.6118E-15	-1.7693E-5	1.0000
0.16856	-7.6021E-10	NaN	1.9766E-4	1.5100E-4	-1.5997E-15	-1.0494E-15	0.18552	8.9438E-15	1.6051E-15	-1.7619E-5	1.0000
0.26756	-7.5745E-10	NaN	1.9691E-4	1.5100E-4	-1.5960E-15	-1.0452E-15	0.18480	8.9101E-15	1.5989E-15	-1.7552E-5	1.0000
0.36656	-7.5577E-10	NaN	1.9646E-4	1.5100E-4	-1.5934E-15	-1.0427E-15	0.18437	8.8897E-15	1.5952E-15	-1.7511E-5	1.0000
0.46556	-7.5464E-10	NaN	1.9616E-4	1.5100E-4	-1.5915E-15	-1.0411E-15	0.18409	8.8761E-15	1.5927E-15	-1.7484E-5	1.0000
0.56456	-7.5382E-10	NaN	1.9594E-4	1.5100E-4	-1.5901E-15	-1.0399E-15	0.18388	8.8663E-15	1.5909E-15	-1.7465E-5	1.0000
0.66356	-7.5320E-10	NaN	1.9578E-4	1.5100E-4	-1.5889E-15	-1.0390E-15	0.18372	8.8588E-15	1.5896E-15	-1.7450E-5	1.0000
0.76256	-7.5269E-10	NaN	1.9564E-4	1.5100E-4	-1.5880E-15	-1.0383E-15	0.18360	8.8528E-15	1.5885E-15	-1.7438E-5	1.0000
0.86156	-7.5228E-10	NaN	1.9554E-4	1.5100E-4	-1.5872E-15	-1.0377E-15	0.18350	8.8479E-15	1.5876E-15	-1.7429E-5	1.0000
0.960	-	NaN	1.954	1.510	-	-	0.183	8.843	1.586	-	1.000

Time (s)	-(tds2.ntflux_Oxi)*n*F (A), UME	SurfaceConcOxi (M), OxiMem	SurfaceConcOxi (M), OxiNuc	Substrate Kinetics (m/s)	Normal total flux (mol/s), UME OCR membrane	Normal total flux, UME OCRnucleus	Concentration (mol/m^3), Point: (0, CellHeight)	k0Sub*Oxi (mol/s), MemFlux	k0Sub*Oxi (mol/s), NucFlux	Normal total flux (mol/(m^2*s)), Point: (0, CellHeight)	WElectrode radius (µm)
56	7.5194E-10		4E-4	0E-4	1.5865E-15	1.0372E-15	41	8E-15	8E-15	1.7421E-5	0
1.0596	-7.5164E-10	NaN	1.9537E-4	1.5100E-4	-1.5859E-15	-1.0368E-15	0.18334	8.8403E-15	1.5862E-15	-1.7414E-5	1.0000

4.3.2 Table 3

Global Evaluation 3 (bnd4)

d (um)	k0Sub (cm/s)	Probe variable bnd4 (A)
100.00	10.000	-9.8325E-10
100.00	8.8889	-9.8325E-10
100.00	7.7778	-9.8325E-10
100.00	6.6667	-9.8325E-10
100.00	5.5556	-9.8325E-10
100.00	4.4444	-9.8325E-10
100.00	3.3333	-9.8325E-10
100.00	2.2222	-9.8325E-10
100.00	1.1111	-9.8325E-10
100.00	0.0000	-9.8277E-10
98.990	10.000	-9.8320E-10
98.990	8.8889	-9.8320E-10
98.990	7.7778	-9.8320E-10
98.990	6.6667	-9.8320E-10
98.990	5.5556	-9.8320E-10
98.990	4.4444	-9.8320E-10
98.990	3.3333	-9.8320E-10
98.990	2.2222	-9.8320E-10
98.990	1.1111	-9.8320E-10
98.990	0.0000	-9.8271E-10
97.981	10.000	-9.8329E-10
97.981	8.8889	-9.8329E-10
97.981	7.7778	-9.8329E-10
97.981	6.6667	-9.8329E-10

d (um)	k0Sub (cm/s)	Probe variable bnd4 (A)
97.981	5.5556	-9.8329E-10
97.981	4.4444	-9.8329E-10
97.981	3.3333	-9.8329E-10
97.981	2.2222	-9.8328E-10
97.981	1.1111	-9.8328E-10
97.981	0.0000	-9.8278E-10
96.971	10.000	-9.8330E-10
96.971	8.8889	-9.8330E-10
96.971	7.7778	-9.8330E-10
96.971	6.6667	-9.8330E-10
96.971	5.5556	-9.8330E-10
96.971	4.4444	-9.8330E-10
96.971	3.3333	-9.8330E-10
96.971	2.2222	-9.8330E-10
96.971	1.1111	-9.8330E-10
96.971	0.0000	-9.8279E-10
95.962	10.000	-9.8327E-10
95.962	8.8889	-9.8327E-10
95.962	7.7778	-9.8327E-10
95.962	6.6667	-9.8327E-10
95.962	5.5556	-9.8327E-10
95.962	4.4444	-9.8327E-10
95.962	3.3333	-9.8327E-10
95.962	2.2222	-9.8327E-10
95.962	1.1111	-9.8327E-10
95.962	0.0000	-9.8274E-10
94.952	10.000	-9.8326E-10
94.952	8.8889	-9.8326E-10
94.952	7.7778	-9.8326E-10
94.952	6.6667	-9.8326E-10
94.952	5.5556	-9.8326E-10
94.952	4.4444	-9.8326E-10
94.952	3.3333	-9.8326E-10
94.952	2.2222	-9.8326E-10
94.952	1.1111	-9.8326E-10
94.952	0.0000	-9.8272E-10
93.942	10.000	-9.8318E-10
93.942	8.8889	-9.8318E-10
93.942	7.7778	-9.8318E-10
93.942	6.6667	-9.8318E-10

d (um)	k0Sub (cm/s)	Probe variable bnd4 (A)
93.942	5.5556	-9.8318E-10
93.942	4.4444	-9.8318E-10
93.942	3.3333	-9.8318E-10
93.942	2.2222	-9.8318E-10
93.942	1.1111	-9.8318E-10
93.942	0.0000	-9.8262E-10
92.933	10.000	-9.8327E-10
92.933	8.8889	-9.8327E-10
92.933	7.7778	-9.8327E-10
92.933	6.6667	-9.8327E-10
92.933	5.5556	-9.8327E-10
92.933	4.4444	-9.8327E-10
92.933	3.3333	-9.8327E-10
92.933	2.2222	-9.8327E-10
92.933	1.1111	-9.8327E-10
92.933	0.0000	-9.8269E-10
91.923	10.000	-9.8330E-10
91.923	8.8889	-9.8330E-10
91.923	7.7778	-9.8330E-10
91.923	6.6667	-9.8330E-10
91.923	5.5556	-9.8330E-10
91.923	4.4444	-9.8330E-10
91.923	3.3333	-9.8330E-10
91.923	2.2222	-9.8330E-10
91.923	1.1111	-9.8330E-10
91.923	0.0000	-9.8272E-10
90.914	10.000	-9.8319E-10
90.914	8.8889	-9.8319E-10
90.914	7.7778	-9.8319E-10
90.914	6.6667	-9.8319E-10
90.914	5.5556	-9.8319E-10
90.914	4.4444	-9.8319E-10
90.914	3.3333	-9.8319E-10
90.914	2.2222	-9.8319E-10
90.914	1.1111	-9.8319E-10
90.914	0.0000	-9.8258E-10
89.904	10.000	-9.8322E-10
89.904	8.8889	-9.8322E-10
89.904	7.7778	-9.8322E-10
89.904	6.6667	-9.8322E-10

d (um)	k0Sub (cm/s)	Probe variable bnd4 (A)
89.904	5.5556	-9.8322E-10
89.904	4.4444	-9.8322E-10
89.904	3.3333	-9.8322E-10
89.904	2.2222	-9.8322E-10
89.904	1.1111	-9.8322E-10
89.904	0.0000	-9.8260E-10
88.894	10.000	-9.8333E-10
88.894	8.8889	-9.8333E-10
88.894	7.7778	-9.8333E-10
88.894	6.6667	-9.8333E-10
88.894	5.5556	-9.8333E-10
88.894	4.4444	-9.8333E-10
88.894	3.3333	-9.8333E-10
88.894	2.2222	-9.8333E-10
88.894	1.1111	-9.8333E-10
88.894	0.0000	-9.8269E-10
87.885	10.000	-9.8323E-10
87.885	8.8889	-9.8323E-10
87.885	7.7778	-9.8323E-10
87.885	6.6667	-9.8323E-10
87.885	5.5556	-9.8323E-10
87.885	4.4444	-9.8323E-10
87.885	3.3333	-9.8323E-10
87.885	2.2222	-9.8323E-10
87.885	1.1111	-9.8323E-10
87.885	0.0000	-9.8258E-10
86.875	10.000	-9.8313E-10
86.875	8.8889	-9.8313E-10
86.875	7.7778	-9.8313E-10
86.875	6.6667	-9.8313E-10
86.875	5.5556	-9.8313E-10
86.875	4.4444	-9.8313E-10
86.875	3.3333	-9.8313E-10
86.875	2.2222	-9.8313E-10
86.875	1.1111	-9.8313E-10
86.875	0.0000	-9.8246E-10
85.866	10.000	-9.8329E-10
85.866	8.8889	-9.8329E-10
85.866	7.7778	-9.8329E-10
85.866	6.6667	-9.8329E-10

d (um)	k0Sub (cm/s)	Probe variable bnd4 (A)
85.866	5.5556	-9.8329E-10
85.866	4.4444	-9.8329E-10
85.866	3.3333	-9.8329E-10
85.866	2.2222	-9.8329E-10
85.866	1.1111	-9.8329E-10
85.866	0.0000	-9.8260E-10
84.856	10.000	-9.8330E-10
84.856	8.8889	-9.8330E-10
84.856	7.7778	-9.8330E-10
84.856	6.6667	-9.8330E-10
84.856	5.5556	-9.8330E-10
84.856	4.4444	-9.8330E-10
84.856	3.3333	-9.8330E-10
84.856	2.2222	-9.8330E-10
84.856	1.1111	-9.8330E-10
84.856	0.0000	-9.8259E-10
83.846	10.000	-9.8329E-10
83.846	8.8889	-9.8329E-10
83.846	7.7778	-9.8329E-10
83.846	6.6667	-9.8329E-10
83.846	5.5556	-9.8329E-10
83.846	4.4444	-9.8329E-10
83.846	3.3333	-9.8329E-10
83.846	2.2222	-9.8329E-10
83.846	1.1111	-9.8329E-10
83.846	0.0000	-9.8256E-10
82.837	10.000	-9.8323E-10
82.837	8.8889	-9.8323E-10
82.837	7.7778	-9.8323E-10
82.837	6.6667	-9.8323E-10
82.837	5.5556	-9.8323E-10
82.837	4.4444	-9.8323E-10
82.837	3.3333	-9.8323E-10
82.837	2.2222	-9.8323E-10
82.837	1.1111	-9.8323E-10
82.837	0.0000	-9.8248E-10
81.827	10.000	-9.8320E-10
81.827	8.8889	-9.8320E-10
81.827	7.7778	-9.8320E-10
81.827	6.6667	-9.8320E-10

d (um)	k0Sub (cm/s)	Probe variable bnd4 (A)
81.827	5.5556	-9.8320E-10
81.827	4.4444	-9.8320E-10
81.827	3.3333	-9.8320E-10
81.827	2.2222	-9.8320E-10
81.827	1.1111	-9.8320E-10
81.827	0.0000	-9.8243E-10
80.818	10.000	-9.8326E-10
80.818	8.8889	-9.8326E-10
80.818	7.7778	-9.8326E-10
80.818	6.6667	-9.8326E-10
80.818	5.5556	-9.8326E-10
80.818	4.4444	-9.8326E-10
80.818	3.3333	-9.8326E-10
80.818	2.2222	-9.8326E-10
80.818	1.1111	-9.8326E-10
80.818	0.0000	-9.8247E-10
79.808	10.000	-9.8324E-10
79.808	8.8889	-9.8324E-10
79.808	7.7778	-9.8324E-10
79.808	6.6667	-9.8324E-10
79.808	5.5556	-9.8324E-10
79.808	4.4444	-9.8324E-10
79.808	3.3333	-9.8324E-10
79.808	2.2222	-9.8324E-10
79.808	1.1111	-9.8324E-10
79.808	0.0000	-9.8243E-10
78.798	10.000	-9.8323E-10
78.798	8.8889	-9.8323E-10
78.798	7.7778	-9.8323E-10
78.798	6.6667	-9.8323E-10
78.798	5.5556	-9.8323E-10
78.798	4.4444	-9.8323E-10
78.798	3.3333	-9.8323E-10
78.798	2.2222	-9.8323E-10
78.798	1.1111	-9.8323E-10
78.798	0.0000	-9.8240E-10
77.789	10.000	-9.8328E-10
77.789	8.8889	-9.8328E-10
77.789	7.7778	-9.8328E-10
77.789	6.6667	-9.8328E-10

d (um)	k0Sub (cm/s)	Probe variable bnd4 (A)
77.789	5.5556	-9.8328E-10
77.789	4.4444	-9.8328E-10
77.789	3.3333	-9.8328E-10
77.789	2.2222	-9.8328E-10
77.789	1.1111	-9.8328E-10
77.789	0.0000	-9.8242E-10
76.779	10.000	-9.8345E-10
76.779	8.8889	-9.8345E-10
76.779	7.7778	-9.8345E-10
76.779	6.6667	-9.8345E-10
76.779	5.5556	-9.8345E-10
76.779	4.4444	-9.8345E-10
76.779	3.3333	-9.8345E-10
76.779	2.2222	-9.8345E-10
76.779	1.1111	-9.8345E-10
76.779	0.0000	-9.8256E-10
75.770	10.000	-9.8343E-10
75.770	8.8889	-9.8343E-10
75.770	7.7778	-9.8343E-10
75.770	6.6667	-9.8343E-10
75.770	5.5556	-9.8343E-10
75.770	4.4444	-9.8343E-10
75.770	3.3333	-9.8343E-10
75.770	2.2222	-9.8343E-10
75.770	1.1111	-9.8343E-10
75.770	0.0000	-9.8252E-10
74.760	10.000	-9.8333E-10
74.760	8.8889	-9.8333E-10
74.760	7.7778	-9.8333E-10
74.760	6.6667	-9.8333E-10
74.760	5.5556	-9.8333E-10
74.760	4.4444	-9.8333E-10
74.760	3.3333	-9.8333E-10
74.760	2.2222	-9.8333E-10
74.760	1.1111	-9.8333E-10
74.760	0.0000	-9.8240E-10
73.751	10.000	-9.8346E-10
73.751	8.8889	-9.8346E-10
73.751	7.7778	-9.8346E-10
73.751	6.6667	-9.8346E-10

d (um)	k0Sub (cm/s)	Probe variable bnd4 (A)
73.751	5.5556	-9.8346E-10
73.751	4.4444	-9.8346E-10
73.751	3.3333	-9.8346E-10
73.751	2.2222	-9.8346E-10
73.751	1.1111	-9.8346E-10
73.751	0.0000	-9.8250E-10
72.741	10.000	-9.8330E-10
72.741	8.8889	-9.8330E-10
72.741	7.7778	-9.8330E-10
72.741	6.6667	-9.8330E-10
72.741	5.5556	-9.8330E-10
72.741	4.4444	-9.8330E-10
72.741	3.3333	-9.8330E-10
72.741	2.2222	-9.8330E-10
72.741	1.1111	-9.8330E-10
72.741	0.0000	-9.8231E-10
71.731	10.000	-9.8335E-10
71.731	8.8889	-9.8335E-10
71.731	7.7778	-9.8335E-10
71.731	6.6667	-9.8335E-10
71.731	5.5556	-9.8335E-10
71.731	4.4444	-9.8335E-10
71.731	3.3333	-9.8335E-10
71.731	2.2222	-9.8335E-10
71.731	1.1111	-9.8335E-10
71.731	0.0000	-9.8233E-10
70.722	10.000	-9.8330E-10
70.722	8.8889	-9.8330E-10
70.722	7.7778	-9.8330E-10
70.722	6.6667	-9.8330E-10
70.722	5.5556	-9.8330E-10
70.722	4.4444	-9.8330E-10
70.722	3.3333	-9.8330E-10
70.722	2.2222	-9.8330E-10
70.722	1.1111	-9.8330E-10
70.722	0.0000	-9.8226E-10
69.712	10.000	-9.8342E-10
69.712	8.8889	-9.8342E-10
69.712	7.7778	-9.8342E-10
69.712	6.6667	-9.8342E-10

d (um)	k0Sub (cm/s)	Probe variable bnd4 (A)
69.712	5.5556	-9.8342E-10
69.712	4.4444	-9.8342E-10
69.712	3.3333	-9.8342E-10
69.712	2.2222	-9.8342E-10
69.712	1.1111	-9.8342E-10
69.712	0.0000	-9.8234E-10
68.703	10.000	-9.8343E-10
68.703	8.8889	-9.8343E-10
68.703	7.7778	-9.8343E-10
68.703	6.6667	-9.8343E-10
68.703	5.5556	-9.8343E-10
68.703	4.4444	-9.8343E-10
68.703	3.3333	-9.8343E-10
68.703	2.2222	-9.8343E-10
68.703	1.1111	-9.8343E-10
68.703	0.0000	-9.8232E-10
67.693	10.000	-9.8336E-10
67.693	8.8889	-9.8336E-10
67.693	7.7778	-9.8336E-10
67.693	6.6667	-9.8336E-10
67.693	5.5556	-9.8336E-10
67.693	4.4444	-9.8336E-10
67.693	3.3333	-9.8336E-10
67.693	2.2222	-9.8336E-10
67.693	1.1111	-9.8336E-10
67.693	0.0000	-9.8222E-10
66.683	10.000	-9.8337E-10
66.683	8.8889	-9.8337E-10
66.683	7.7778	-9.8337E-10
66.683	6.6667	-9.8337E-10
66.683	5.5556	-9.8337E-10
66.683	4.4444	-9.8337E-10
66.683	3.3333	-9.8337E-10
66.683	2.2222	-9.8337E-10
66.683	1.1111	-9.8337E-10
66.683	0.0000	-9.8220E-10
65.674	10.000	-9.8353E-10
65.674	8.8889	-9.8353E-10
65.674	7.7778	-9.8353E-10
65.674	6.6667	-9.8353E-10

d (um)	k0Sub (cm/s)	Probe variable bnd4 (A)
65.674	5.5556	-9.8353E-10
65.674	4.4444	-9.8353E-10
65.674	3.3333	-9.8353E-10
65.674	2.2222	-9.8353E-10
65.674	1.1111	-9.8353E-10
65.674	0.0000	-9.8232E-10
64.664	10.000	-9.8352E-10
64.664	8.8889	-9.8352E-10
64.664	7.7778	-9.8352E-10
64.664	6.6667	-9.8352E-10
64.664	5.5556	-9.8352E-10
64.664	4.4444	-9.8352E-10
64.664	3.3333	-9.8352E-10
64.664	2.2222	-9.8352E-10
64.664	1.1111	-9.8352E-10
64.664	0.0000	-9.8227E-10
63.655	10.000	-9.8346E-10
63.655	8.8889	-9.8346E-10
63.655	7.7778	-9.8346E-10
63.655	6.6667	-9.8346E-10
63.655	5.5556	-9.8346E-10
63.655	4.4444	-9.8346E-10
63.655	3.3333	-9.8346E-10
63.655	2.2222	-9.8346E-10
63.655	1.1111	-9.8346E-10
63.655	0.0000	-9.8217E-10
62.645	10.000	-9.8347E-10
62.645	8.8889	-9.8347E-10
62.645	7.7778	-9.8347E-10
62.645	6.6667	-9.8347E-10
62.645	5.5556	-9.8347E-10
62.645	4.4444	-9.8347E-10
62.645	3.3333	-9.8347E-10
62.645	2.2222	-9.8347E-10
62.645	1.1111	-9.8347E-10
62.645	0.0000	-9.8213E-10
61.635	10.000	-9.8352E-10
61.635	8.8889	-9.8352E-10
61.635	7.7778	-9.8352E-10
61.635	6.6667	-9.8352E-10

d (um)	k0Sub (cm/s)	Probe variable bnd4 (A)
61.635	5.5556	-9.8352E-10
61.635	4.4444	-9.8352E-10
61.635	3.3333	-9.8352E-10
61.635	2.2222	-9.8352E-10
61.635	1.1111	-9.8352E-10
61.635	0.0000	-9.8215E-10
60.626	10.000	-9.8356E-10
60.626	8.8889	-9.8356E-10
60.626	7.7778	-9.8356E-10
60.626	6.6667	-9.8356E-10
60.626	5.5556	-9.8356E-10
60.626	4.4444	-9.8356E-10
60.626	3.3333	-9.8356E-10
60.626	2.2222	-9.8356E-10
60.626	1.1111	-9.8356E-10
60.626	0.0000	-9.8214E-10
59.616	10.000	-9.8184E-10
59.616	8.8889	-9.8184E-10
59.616	7.7778	-9.8184E-10
59.616	6.6667	-9.8184E-10
59.616	5.5556	-9.8184E-10
59.616	4.4444	-9.8184E-10
59.616	3.3333	-9.8184E-10
59.616	2.2222	-9.8184E-10
59.616	1.1111	-9.8184E-10
59.616	0.0000	-9.8038E-10
58.607	10.000	-9.8357E-10
58.607	8.8889	-9.8357E-10
58.607	7.7778	-9.8357E-10
58.607	6.6667	-9.8357E-10
58.607	5.5556	-9.8357E-10
58.607	4.4444	-9.8357E-10
58.607	3.3333	-9.8357E-10
58.607	2.2222	-9.8357E-10
58.607	1.1111	-9.8357E-10
58.607	0.0000	-9.8206E-10
57.597	10.000	-9.8356E-10
57.597	8.8889	-9.8356E-10
57.597	7.7778	-9.8356E-10
57.597	6.6667	-9.8356E-10

d (um)	k0Sub (cm/s)	Probe variable bnd4 (A)
57.597	5.5556	-9.8356E-10
57.597	4.4444	-9.8356E-10
57.597	3.3333	-9.8356E-10
57.597	2.2222	-9.8356E-10
57.597	1.1111	-9.8356E-10
57.597	0.0000	-9.8199E-10
56.587	10.000	-9.8356E-10
56.587	8.8889	-9.8356E-10
56.587	7.7778	-9.8356E-10
56.587	6.6667	-9.8356E-10
56.587	5.5556	-9.8356E-10
56.587	4.4444	-9.8356E-10
56.587	3.3333	-9.8356E-10
56.587	2.2222	-9.8356E-10
56.587	1.1111	-9.8356E-10
56.587	0.0000	-9.8194E-10
55.578	10.000	-9.8197E-10
55.578	8.8889	-9.8197E-10
55.578	7.7778	-9.8197E-10
55.578	6.6667	-9.8197E-10
55.578	5.5556	-9.8197E-10
55.578	4.4444	-9.8197E-10
55.578	3.3333	-9.8197E-10
55.578	2.2222	-9.8197E-10
55.578	1.1111	-9.8197E-10
55.578	0.0000	-9.8030E-10
54.568	10.000	-9.8375E-10
54.568	8.8889	-9.8375E-10
54.568	7.7778	-9.8375E-10
54.568	6.6667	-9.8375E-10
54.568	5.5556	-9.8375E-10
54.568	4.4444	-9.8375E-10
54.568	3.3333	-9.8375E-10
54.568	2.2222	-9.8375E-10
54.568	1.1111	-9.8375E-10
54.568	0.0000	-9.8202E-10
53.559	10.000	-9.8367E-10
53.559	8.8889	-9.8367E-10
53.559	7.7778	-9.8367E-10
53.559	6.6667	-9.8367E-10

d (um)	k0Sub (cm/s)	Probe variable bnd4 (A)
53.559	5.5556	-9.8367E-10
53.559	4.4444	-9.8367E-10
53.559	3.3333	-9.8367E-10
53.559	2.2222	-9.8367E-10
53.559	1.1111	-9.8367E-10
53.559	0.0000	-9.8188E-10
52.549	10.000	-9.8358E-10
52.549	8.8889	-9.8358E-10
52.549	7.7778	-9.8358E-10
52.549	6.6667	-9.8358E-10
52.549	5.5556	-9.8358E-10
52.549	4.4444	-9.8358E-10
52.549	3.3333	-9.8358E-10
52.549	2.2222	-9.8358E-10
52.549	1.1111	-9.8358E-10
52.549	0.0000	-9.8173E-10
51.539	10.000	-9.8370E-10
51.539	8.8889	-9.8370E-10
51.539	7.7778	-9.8370E-10
51.539	6.6667	-9.8370E-10
51.539	5.5556	-9.8370E-10
51.539	4.4444	-9.8370E-10
51.539	3.3333	-9.8370E-10
51.539	2.2222	-9.8370E-10
51.539	1.1111	-9.8370E-10
51.539	0.0000	-9.8178E-10
50.530	10.000	-9.8368E-10
50.530	8.8889	-9.8368E-10
50.530	7.7778	-9.8368E-10
50.530	6.6667	-9.8368E-10
50.530	5.5556	-9.8368E-10
50.530	4.4444	-9.8368E-10
50.530	3.3333	-9.8368E-10
50.530	2.2222	-9.8368E-10
50.530	1.1111	-9.8368E-10
50.530	0.0000	-9.8169E-10
49.520	10.000	-9.8379E-10
49.520	8.8889	-9.8379E-10
49.520	7.7778	-9.8379E-10
49.520	6.6667	-9.8379E-10

d (um)	k0Sub (cm/s)	Probe variable bnd4 (A)
49.520	5.5556	-9.8379E-10
49.520	4.4444	-9.8379E-10
49.520	3.3333	-9.8379E-10
49.520	2.2222	-9.8379E-10
49.520	1.1111	-9.8379E-10
49.520	0.0000	-9.8172E-10
48.511	10.000	-9.8367E-10
48.511	8.8889	-9.8367E-10
48.511	7.7778	-9.8367E-10
48.511	6.6667	-9.8367E-10
48.511	5.5556	-9.8367E-10
48.511	4.4444	-9.8367E-10
48.511	3.3333	-9.8367E-10
48.511	2.2222	-9.8367E-10
48.511	1.1111	-9.8367E-10
48.511	0.0000	-9.8153E-10
47.501	10.000	-9.8378E-10
47.501	8.8889	-9.8378E-10
47.501	7.7778	-9.8378E-10
47.501	6.6667	-9.8378E-10
47.501	5.5556	-9.8378E-10
47.501	4.4444	-9.8378E-10
47.501	3.3333	-9.8378E-10
47.501	2.2222	-9.8378E-10
47.501	1.1111	-9.8378E-10
47.501	0.0000	-9.8156E-10
46.491	10.000	-9.8392E-10
46.491	8.8889	-9.8392E-10
46.491	7.7778	-9.8392E-10
46.491	6.6667	-9.8392E-10
46.491	5.5556	-9.8392E-10
46.491	4.4444	-9.8392E-10
46.491	3.3333	-9.8392E-10
46.491	2.2222	-9.8392E-10
46.491	1.1111	-9.8392E-10
46.491	0.0000	-9.8162E-10
45.482	10.000	-9.8404E-10
45.482	8.8889	-9.8404E-10
45.482	7.7778	-9.8404E-10
45.482	6.6667	-9.8404E-10

d (um)	k0Sub (cm/s)	Probe variable bnd4 (A)
45.482	5.5556	-9.8404E-10
45.482	4.4444	-9.8404E-10
45.482	3.3333	-9.8404E-10
45.482	2.2222	-9.8404E-10
45.482	1.1111	-9.8404E-10
45.482	0.0000	-9.8164E-10
44.472	10.000	-9.8398E-10
44.472	8.8889	-9.8398E-10
44.472	7.7778	-9.8398E-10
44.472	6.6667	-9.8398E-10
44.472	5.5556	-9.8398E-10
44.472	4.4444	-9.8398E-10
44.472	3.3333	-9.8398E-10
44.472	2.2222	-9.8398E-10
44.472	1.1111	-9.8398E-10
44.472	0.0000	-9.8149E-10
43.463	10.000	-9.8413E-10
43.463	8.8889	-9.8413E-10
43.463	7.7778	-9.8413E-10
43.463	6.6667	-9.8413E-10
43.463	5.5556	-9.8413E-10
43.463	4.4444	-9.8413E-10
43.463	3.3333	-9.8413E-10
43.463	2.2222	-9.8413E-10
43.463	1.1111	-9.8413E-10
43.463	0.0000	-9.8154E-10
42.453	10.000	-9.8411E-10
42.453	8.8889	-9.8411E-10
42.453	7.7778	-9.8411E-10
42.453	6.6667	-9.8411E-10
42.453	5.5556	-9.8411E-10
42.453	4.4444	-9.8411E-10
42.453	3.3333	-9.8411E-10
42.453	2.2222	-9.8411E-10
42.453	1.1111	-9.8411E-10
42.453	0.0000	-9.8142E-10
41.443	10.000	-9.8425E-10
41.443	8.8889	-9.8425E-10
41.443	7.7778	-9.8425E-10
41.443	6.6667	-9.8425E-10

d (um)	k0Sub (cm/s)	Probe variable bnd4 (A)
41.443	5.5556	-9.8425E-10
41.443	4.4444	-9.8425E-10
41.443	3.3333	-9.8425E-10
41.443	2.2222	-9.8425E-10
41.443	1.1111	-9.8425E-10
41.443	0.0000	-9.8144E-10
40.434	10.000	-9.8424E-10
40.434	8.8889	-9.8424E-10
40.434	7.7778	-9.8424E-10
40.434	6.6667	-9.8424E-10
40.434	5.5556	-9.8424E-10
40.434	4.4444	-9.8424E-10
40.434	3.3333	-9.8424E-10
40.434	2.2222	-9.8424E-10
40.434	1.1111	-9.8424E-10
40.434	0.0000	-9.8132E-10
39.424	10.000	-9.8419E-10
39.424	8.8889	-9.8419E-10
39.424	7.7778	-9.8419E-10
39.424	6.6667	-9.8419E-10
39.424	5.5556	-9.8419E-10
39.424	4.4444	-9.8419E-10
39.424	3.3333	-9.8419E-10
39.424	2.2222	-9.8419E-10
39.424	1.1111	-9.8419E-10
39.424	0.0000	-9.8114E-10
38.415	10.000	-9.8415E-10
38.415	8.8889	-9.8415E-10
38.415	7.7778	-9.8415E-10
38.415	6.6667	-9.8415E-10
38.415	5.5556	-9.8415E-10
38.415	4.4444	-9.8415E-10
38.415	3.3333	-9.8415E-10
38.415	2.2222	-9.8415E-10
38.415	1.1111	-9.8415E-10
38.415	0.0000	-9.8097E-10
37.405	10.000	-9.8448E-10
37.405	8.8889	-9.8448E-10
37.405	7.7778	-9.8448E-10
37.405	6.6667	-9.8448E-10

d (um)	k0Sub (cm/s)	Probe variable bnd4 (A)
37.405	5.5556	-9.8448E-10
37.405	4.4444	-9.8448E-10
37.405	3.3333	-9.8448E-10
37.405	2.2222	-9.8448E-10
37.405	1.1111	-9.8448E-10
37.405	0.0000	-9.8116E-10
36.395	10.000	-9.8438E-10
36.395	8.8889	-9.8438E-10
36.395	7.7778	-9.8438E-10
36.395	6.6667	-9.8438E-10
36.395	5.5556	-9.8438E-10
36.395	4.4444	-9.8438E-10
36.395	3.3333	-9.8438E-10
36.395	2.2222	-9.8438E-10
36.395	1.1111	-9.8438E-10
36.395	0.0000	-9.8090E-10
35.386	10.000	-9.8461E-10
35.386	8.8889	-9.8461E-10
35.386	7.7778	-9.8461E-10
35.386	6.6667	-9.8461E-10
35.386	5.5556	-9.8461E-10
35.386	4.4444	-9.8461E-10
35.386	3.3333	-9.8461E-10
35.386	2.2222	-9.8461E-10
35.386	1.1111	-9.8461E-10
35.386	0.0000	-9.8098E-10
34.376	10.000	-9.8464E-10
34.376	8.8889	-9.8464E-10
34.376	7.7778	-9.8464E-10
34.376	6.6667	-9.8464E-10
34.376	5.5556	-9.8464E-10
34.376	4.4444	-9.8464E-10
34.376	3.3333	-9.8464E-10
34.376	2.2222	-9.8464E-10
34.376	1.1111	-9.8464E-10
34.376	0.0000	-9.8084E-10
33.367	10.000	-9.8469E-10
33.367	8.8889	-9.8469E-10
33.367	7.7778	-9.8469E-10
33.367	6.6667	-9.8469E-10

d (um)	k0Sub (cm/s)	Probe variable bnd4 (A)
33.367	5.5556	-9.8469E-10
33.367	4.4444	-9.8469E-10
33.367	3.3333	-9.8469E-10
33.367	2.2222	-9.8469E-10
33.367	1.1111	-9.8469E-10
33.367	0.0000	-9.8070E-10
32.357	10.000	-9.8481E-10
32.357	8.8889	-9.8481E-10
32.357	7.7778	-9.8481E-10
32.357	6.6667	-9.8481E-10
32.357	5.5556	-9.8481E-10
32.357	4.4444	-9.8481E-10
32.357	3.3333	-9.8481E-10
32.357	2.2222	-9.8481E-10
32.357	1.1111	-9.8481E-10
32.357	0.0000	-9.8062E-10
31.347	10.000	-9.8479E-10
31.347	8.8889	-9.8479E-10
31.347	7.7778	-9.8479E-10
31.347	6.6667	-9.8479E-10
31.347	5.5556	-9.8479E-10
31.347	4.4444	-9.8479E-10
31.347	3.3333	-9.8479E-10
31.347	2.2222	-9.8479E-10
31.347	1.1111	-9.8479E-10
31.347	0.0000	-9.8039E-10
30.338	10.000	-9.8498E-10
30.338	8.8889	-9.8498E-10
30.338	7.7778	-9.8498E-10
30.338	6.6667	-9.8498E-10
30.338	5.5556	-9.8498E-10
30.338	4.4444	-9.8498E-10
30.338	3.3333	-9.8498E-10
30.338	2.2222	-9.8498E-10
30.338	1.1111	-9.8498E-10
30.338	0.0000	-9.8035E-10
29.328	10.000	-9.8497E-10
29.328	8.8889	-9.8497E-10
29.328	7.7778	-9.8497E-10
29.328	6.6667	-9.8497E-10

d (um)	k0Sub (cm/s)	Probe variable bnd4 (A)
29.328	5.5556	-9.8497E-10
29.328	4.4444	-9.8497E-10
29.328	3.3333	-9.8497E-10
29.328	2.2222	-9.8497E-10
29.328	1.1111	-9.8497E-10
29.328	0.0000	-9.8008E-10
28.319	10.000	-9.8529E-10
28.319	8.8889	-9.8529E-10
28.319	7.7778	-9.8529E-10
28.319	6.6667	-9.8529E-10
28.319	5.5556	-9.8529E-10
28.319	4.4444	-9.8529E-10
28.319	3.3333	-9.8529E-10
28.319	2.2222	-9.8529E-10
28.319	1.1111	-9.8529E-10
28.319	0.0000	-9.8013E-10
27.309	10.000	-9.8538E-10
27.309	8.8889	-9.8538E-10
27.309	7.7778	-9.8538E-10
27.309	6.6667	-9.8538E-10
27.309	5.5556	-9.8538E-10
27.309	4.4444	-9.8538E-10
27.309	3.3333	-9.8538E-10
27.309	2.2222	-9.8538E-10
27.309	1.1111	-9.8538E-10
27.309	0.0000	-9.7993E-10
26.299	10.000	-9.8552E-10
26.299	8.8889	-9.8552E-10
26.299	7.7778	-9.8552E-10
26.299	6.6667	-9.8552E-10
26.299	5.5556	-9.8552E-10
26.299	4.4444	-9.8552E-10
26.299	3.3333	-9.8552E-10
26.299	2.2222	-9.8552E-10
26.299	1.1111	-9.8552E-10
26.299	0.0000	-9.7974E-10
25.290	10.000	-9.8571E-10
25.290	8.8889	-9.8571E-10
25.290	7.7778	-9.8571E-10
25.290	6.6667	-9.8571E-10

d (um)	k0Sub (cm/s)	Probe variable bnd4 (A)
25.290	5.5556	-9.8571E-10
25.290	4.4444	-9.8571E-10
25.290	3.3333	-9.8571E-10
25.290	2.2222	-9.8571E-10
25.290	1.1111	-9.8571E-10
25.290	0.0000	-9.7959E-10
24.280	10.000	-9.8580E-10
24.280	8.8889	-9.8580E-10
24.280	7.7778	-9.8580E-10
24.280	6.6667	-9.8580E-10
24.280	5.5556	-9.8580E-10
24.280	4.4444	-9.8580E-10
24.280	3.3333	-9.8580E-10
24.280	2.2222	-9.8580E-10
24.280	1.1111	-9.8580E-10
24.280	0.0000	-9.7929E-10
23.271	10.000	-9.8617E-10
23.271	8.8889	-9.8617E-10
23.271	7.7778	-9.8617E-10
23.271	6.6667	-9.8617E-10
23.271	5.5556	-9.8617E-10
23.271	4.4444	-9.8617E-10
23.271	3.3333	-9.8617E-10
23.271	2.2222	-9.8617E-10
23.271	1.1111	-9.8617E-10
23.271	0.0000	-9.7922E-10
22.261	10.000	-9.8632E-10
22.261	8.8889	-9.8632E-10
22.261	7.7778	-9.8632E-10
22.261	6.6667	-9.8632E-10
22.261	5.5556	-9.8632E-10
22.261	4.4444	-9.8632E-10
22.261	3.3333	-9.8632E-10
22.261	2.2222	-9.8632E-10
22.261	1.1111	-9.8632E-10
22.261	0.0000	-9.7890E-10
21.252	10.000	-9.8647E-10
21.252	8.8889	-9.8647E-10
21.252	7.7778	-9.8647E-10
21.252	6.6667	-9.8647E-10

d (um)	k0Sub (cm/s)	Probe variable bnd4 (A)
21.252	5.5556	-9.8647E-10
21.252	4.4444	-9.8647E-10
21.252	3.3333	-9.8647E-10
21.252	2.2222	-9.8647E-10
21.252	1.1111	-9.8647E-10
21.252	0.0000	-9.7852E-10
20.242	10.000	-9.8693E-10
20.242	8.8889	-9.8693E-10
20.242	7.7778	-9.8693E-10
20.242	6.6667	-9.8693E-10
20.242	5.5556	-9.8693E-10
20.242	4.4444	-9.8693E-10
20.242	3.3333	-9.8693E-10
20.242	2.2222	-9.8693E-10
20.242	1.1111	-9.8693E-10
20.242	0.0000	-9.7838E-10
19.232	10.000	-9.8705E-10
19.232	8.8889	-9.8705E-10
19.232	7.7778	-9.8705E-10
19.232	6.6667	-9.8705E-10
19.232	5.5556	-9.8705E-10
19.232	4.4444	-9.8705E-10
19.232	3.3333	-9.8705E-10
19.232	2.2222	-9.8705E-10
19.232	1.1111	-9.8705E-10
19.232	0.0000	-9.7784E-10
18.223	10.000	-9.8762E-10
18.223	8.8889	-9.8762E-10
18.223	7.7778	-9.8762E-10
18.223	6.6667	-9.8762E-10
18.223	5.5556	-9.8762E-10
18.223	4.4444	-9.8762E-10
18.223	3.3333	-9.8762E-10
18.223	2.2222	-9.8762E-10
18.223	1.1111	-9.8762E-10
18.223	0.0000	-9.7764E-10
17.213	10.000	-9.8786E-10
17.213	8.8889	-9.8786E-10
17.213	7.7778	-9.8786E-10
17.213	6.6667	-9.8786E-10

d (um)	k0Sub (cm/s)	Probe variable bnd4 (A)
17.213	5.5556	-9.8786E-10
17.213	4.4444	-9.8786E-10
17.213	3.3333	-9.8786E-10
17.213	2.2222	-9.8786E-10
17.213	1.1111	-9.8786E-10
17.213	0.0000	-9.7701E-10
16.204	10.000	-9.8840E-10
16.204	8.8889	-9.8840E-10
16.204	7.7778	-9.8840E-10
16.204	6.6667	-9.8840E-10
16.204	5.5556	-9.8840E-10
16.204	4.4444	-9.8840E-10
16.204	3.3333	-9.8840E-10
16.204	2.2222	-9.8840E-10
16.204	1.1111	-9.8840E-10
16.204	0.0000	-9.7654E-10
15.194	10.000	-9.8879E-10
15.194	8.8889	-9.8879E-10
15.194	7.7778	-9.8879E-10
15.194	6.6667	-9.8879E-10
15.194	5.5556	-9.8879E-10
15.194	4.4444	-9.8879E-10
15.194	3.3333	-9.8879E-10
15.194	2.2222	-9.8879E-10
15.194	1.1111	-9.8879E-10
15.194	0.0000	-9.7576E-10
14.184	10.000	-9.8971E-10
14.184	8.8889	-9.8971E-10
14.184	7.7778	-9.8971E-10
14.184	6.6667	-9.8971E-10
14.184	5.5556	-9.8971E-10
14.184	4.4444	-9.8971E-10
14.184	3.3333	-9.8971E-10
14.184	2.2222	-9.8971E-10
14.184	1.1111	-9.8971E-10
14.184	0.0000	-9.7528E-10
13.175	10.000	-9.9051E-10
13.175	8.8889	-9.9051E-10
13.175	7.7778	-9.9051E-10
13.175	6.6667	-9.9051E-10

d (um)	k0Sub (cm/s)	Probe variable bnd4 (A)
13.175	5.5556	-9.9051E-10
13.175	4.4444	-9.9051E-10
13.175	3.3333	-9.9051E-10
13.175	2.2222	-9.9051E-10
13.175	1.1111	-9.9051E-10
13.175	0.0000	-9.7443E-10
12.165	10.000	-9.9138E-10
12.165	8.8889	-9.9138E-10
12.165	7.7778	-9.9138E-10
12.165	6.6667	-9.9138E-10
12.165	5.5556	-9.9138E-10
12.165	4.4444	-9.9138E-10
12.165	3.3333	-9.9138E-10
12.165	2.2222	-9.9138E-10
12.165	1.1111	-9.9138E-10
12.165	0.0000	-9.7331E-10
11.156	10.000	-9.9255E-10
11.156	8.8889	-9.9255E-10
11.156	7.7778	-9.9255E-10
11.156	6.6667	-9.9255E-10
11.156	5.5556	-9.9255E-10
11.156	4.4444	-9.9255E-10
11.156	3.3333	-9.9255E-10
11.156	2.2222	-9.9255E-10
11.156	1.1111	-9.9255E-10
11.156	0.0000	-9.7202E-10
10.146	10.000	-9.9399E-10
10.146	8.8889	-9.9399E-10
10.146	7.7778	-9.9399E-10
10.146	6.6667	-9.9399E-10
10.146	5.5556	-9.9399E-10
10.146	4.4444	-9.9399E-10
10.146	3.3333	-9.9399E-10
10.146	2.2222	-9.9399E-10
10.146	1.1111	-9.9399E-10
10.146	0.0000	-9.7038E-10
9.1364	10.000	-9.9588E-10
9.1364	8.8889	-9.9588E-10
9.1364	7.7778	-9.9588E-10
9.1364	6.6667	-9.9588E-10

d (um)	k0Sub (cm/s)	Probe variable bnd4 (A)
9.1364	5.5556	-9.9588E-10
9.1364	4.4444	-9.9588E-10
9.1364	3.3333	-9.9588E-10
9.1364	2.2222	-9.9588E-10
9.1364	1.1111	-9.9588E-10
9.1364	0.0000	-9.6831E-10
8.1268	10.000	-9.9823E-10
8.1268	8.8889	-9.9823E-10
8.1268	7.7778	-9.9823E-10
8.1268	6.6667	-9.9823E-10
8.1268	5.5556	-9.9823E-10
8.1268	4.4444	-9.9823E-10
8.1268	3.3333	-9.9823E-10
8.1268	2.2222	-9.9823E-10
8.1268	1.1111	-9.9823E-10
8.1268	0.0000	-9.6543E-10
7.1172	10.000	-1.0015E-9
7.1172	8.8889	-1.0015E-9
7.1172	7.7778	-1.0015E-9
7.1172	6.6667	-1.0015E-9
7.1172	5.5556	-1.0015E-9
7.1172	4.4444	-1.0015E-9
7.1172	3.3333	-1.0015E-9
7.1172	2.2222	-1.0015E-9
7.1172	1.1111	-1.0015E-9
7.1172	0.0000	-9.6156E-10
6.1076	10.000	-1.0062E-9
6.1076	8.8889	-1.0062E-9
6.1076	7.7778	-1.0062E-9
6.1076	6.6667	-1.0062E-9
6.1076	5.5556	-1.0062E-9
6.1076	4.4444	-1.0062E-9
6.1076	3.3333	-1.0062E-9
6.1076	2.2222	-1.0062E-9
6.1076	1.1111	-1.0062E-9
6.1076	0.0000	-9.5598E-10
5.0980	10.000	-1.0130E-9
5.0980	8.8889	-1.0130E-9
5.0980	7.7778	-1.0130E-9
5.0980	6.6667	-1.0130E-9

d (um)	k0Sub (cm/s)	Probe variable bnd4 (A)
5.0980	5.5556	-1.0130E-9
5.0980	4.4444	-1.0130E-9
5.0980	3.3333	-1.0130E-9
5.0980	2.2222	-1.0130E-9
5.0980	1.1111	-1.0130E-9
5.0980	0.0000	-9.4728E-10
4.0884	10.000	-1.0237E-9
4.0884	8.8889	-1.0237E-9
4.0884	7.7778	-1.0237E-9
4.0884	6.6667	-1.0237E-9
4.0884	5.5556	-1.0237E-9
4.0884	4.4444	-1.0237E-9
4.0884	3.3333	-1.0237E-9
4.0884	2.2222	-1.0237E-9
4.0884	1.1111	-1.0237E-9
4.0884	0.0000	-9.3270E-10
3.0788	10.000	-1.0424E-9
3.0788	8.8889	-1.0424E-9
3.0788	7.7778	-1.0424E-9
3.0788	6.6667	-1.0424E-9
3.0788	5.5556	-1.0424E-9
3.0788	4.4444	-1.0424E-9
3.0788	3.3333	-1.0424E-9
3.0788	2.2222	-1.0424E-9
3.0788	1.1111	-1.0424E-9
3.0788	0.0000	-9.0570E-10
2.0692	10.000	-1.0819E-9
2.0692	8.8889	-1.0819E-9
2.0692	7.7778	-1.0819E-9
2.0692	6.6667	-1.0819E-9
2.0692	5.5556	-1.0819E-9
2.0692	4.4444	-1.0819E-9
2.0692	3.3333	-1.0819E-9
2.0692	2.2222	-1.0819E-9
2.0692	1.1111	-1.0818E-9
2.0692	0.0000	-8.4696E-10
1.0596	10.000	-1.2078E-9
1.0596	8.8889	-1.2077E-9
1.0596	7.7778	-1.2077E-9
1.0596	6.6667	-1.2077E-9

d (um)	k0Sub (cm/s)	Probe variable bnd4 (A)
1.0596	5.5556	-1.2077E-9
1.0596	4.4444	-1.2077E-9
1.0596	3.3333	-1.2076E-9
1.0596	2.2222	-1.2076E-9
1.0596	1.1111	-1.2073E-9
1.0596	0.0000	-6.8069E-10
0.050000	10.000	-8.0997E-9
0.050000	8.8889	-8.0900E-9
0.050000	7.7778	-8.0775E-9
0.050000	6.6667	-8.0611E-9
0.050000	5.5556	-8.0381E-9
0.050000	4.4444	-8.0042E-9
0.050000	3.3333	-7.9485E-9
0.050000	2.2222	-7.8408E-9
0.050000	1.1111	-7.5432E-9
0.050000	0.0000	-5.4551E-11

4.3.3 Table 4

Global Evaluation 3 (bnd4)

d (um)	k0Sub (cm/s)	Probe variable bnd4 (A)
25.000	1.0000	-9.8581E-10
25.000	0.88889	-9.8581E-10
25.000	0.77778	-9.8581E-10
25.000	0.66667	-9.8581E-10
25.000	0.55556	-9.8581E-10
25.000	0.44444	-9.8581E-10
25.000	0.33333	-9.8581E-10
25.000	0.22222	-9.8580E-10
25.000	0.11111	-9.8580E-10
25.000	0.0000	-9.7957E-10
24.748	1.0000	-9.8585E-10
24.748	0.88889	-9.8585E-10
24.748	0.77778	-9.8585E-10
24.748	0.66667	-9.8585E-10
24.748	0.55556	-9.8585E-10
24.748	0.44444	-9.8585E-10
24.748	0.33333	-9.8585E-10
24.748	0.22222	-9.8585E-10
24.748	0.11111	-9.8585E-10

d (um)	k0Sub (cm/s)	Probe variable bnd4 (A)
24.748	0.0000	-9.7952E-10
24.496	1.0000	-9.8578E-10
24.496	0.88889	-9.8578E-10
24.496	0.77778	-9.8578E-10
24.496	0.66667	-9.8578E-10
24.496	0.55556	-9.8578E-10
24.496	0.44444	-9.8578E-10
24.496	0.33333	-9.8578E-10
24.496	0.22222	-9.8578E-10
24.496	0.11111	-9.8578E-10
24.496	0.0000	-9.7935E-10
24.244	1.0000	-9.8578E-10
24.244	0.88889	-9.8578E-10
24.244	0.77778	-9.8578E-10
24.244	0.66667	-9.8578E-10
24.244	0.55556	-9.8578E-10
24.244	0.44444	-9.8578E-10
24.244	0.33333	-9.8578E-10
24.244	0.22222	-9.8578E-10
24.244	0.11111	-9.8578E-10
24.244	0.0000	-9.7926E-10
23.992	1.0000	-9.8587E-10
23.992	0.88889	-9.8587E-10
23.992	0.77778	-9.8587E-10
23.992	0.66667	-9.8587E-10
23.992	0.55556	-9.8587E-10
23.992	0.44444	-9.8587E-10
23.992	0.33333	-9.8586E-10
23.992	0.22222	-9.8586E-10
23.992	0.11111	-9.8586E-10
23.992	0.0000	-9.7923E-10
23.740	1.0000	-9.8591E-10
23.740	0.88889	-9.8591E-10
23.740	0.77778	-9.8591E-10
23.740	0.66667	-9.8591E-10
23.740	0.55556	-9.8591E-10
23.740	0.44444	-9.8591E-10
23.740	0.33333	-9.8591E-10
23.740	0.22222	-9.8591E-10
23.740	0.11111	-9.8590E-10

d (um)	k0Sub (cm/s)	Probe variable bnd4 (A)
23.740	0.0000	-9.7917E-10
23.488	1.0000	-9.8602E-10
23.488	0.88889	-9.8602E-10
23.488	0.77778	-9.8602E-10
23.488	0.66667	-9.8602E-10
23.488	0.55556	-9.8602E-10
23.488	0.44444	-9.8602E-10
23.488	0.33333	-9.8602E-10
23.488	0.22222	-9.8602E-10
23.488	0.11111	-9.8602E-10
23.488	0.0000	-9.7917E-10
23.236	1.0000	-9.8617E-10
23.236	0.88889	-9.8617E-10
23.236	0.77778	-9.8617E-10
23.236	0.66667	-9.8617E-10
23.236	0.55556	-9.8617E-10
23.236	0.44444	-9.8617E-10
23.236	0.33333	-9.8617E-10
23.236	0.22222	-9.8617E-10
23.236	0.11111	-9.8617E-10
23.236	0.0000	-9.7921E-10
22.984	1.0000	-9.8608E-10
22.984	0.88889	-9.8608E-10
22.984	0.77778	-9.8608E-10
22.984	0.66667	-9.8608E-10
22.984	0.55556	-9.8608E-10
22.984	0.44444	-9.8608E-10
22.984	0.33333	-9.8608E-10
22.984	0.22222	-9.8608E-10
22.984	0.11111	-9.8608E-10
22.984	0.0000	-9.7901E-10
22.732	1.0000	-9.8622E-10
22.732	0.88889	-9.8622E-10
22.732	0.77778	-9.8622E-10
22.732	0.66667	-9.8622E-10
22.732	0.55556	-9.8622E-10
22.732	0.44444	-9.8622E-10
22.732	0.33333	-9.8622E-10
22.732	0.22222	-9.8622E-10
22.732	0.11111	-9.8622E-10

d (um)	k0Sub (cm/s)	Probe variable bnd4 (A)
22.732	0.0000	-9.7903E-10
22.480	1.0000	-9.8624E-10
22.480	0.88889	-9.8624E-10
22.480	0.77778	-9.8624E-10
22.480	0.66667	-9.8624E-10
22.480	0.55556	-9.8624E-10
22.480	0.44444	-9.8624E-10
22.480	0.33333	-9.8624E-10
22.480	0.22222	-9.8624E-10
22.480	0.11111	-9.8623E-10
22.480	0.0000	-9.7893E-10
22.228	1.0000	-9.8626E-10
22.228	0.88889	-9.8626E-10
22.228	0.77778	-9.8626E-10
22.228	0.66667	-9.8626E-10
22.228	0.55556	-9.8626E-10
22.228	0.44444	-9.8626E-10
22.228	0.33333	-9.8626E-10
22.228	0.22222	-9.8626E-10
22.228	0.11111	-9.8626E-10
22.228	0.0000	-9.7883E-10
21.976	1.0000	-9.8636E-10
21.976	0.88889	-9.8636E-10
21.976	0.77778	-9.8636E-10
21.976	0.66667	-9.8636E-10
21.976	0.55556	-9.8636E-10
21.976	0.44444	-9.8636E-10
21.976	0.33333	-9.8636E-10
21.976	0.22222	-9.8636E-10
21.976	0.11111	-9.8635E-10
21.976	0.0000	-9.7880E-10
21.724	1.0000	-9.8653E-10
21.724	0.88889	-9.8653E-10
21.724	0.77778	-9.8653E-10
21.724	0.66667	-9.8653E-10
21.724	0.55556	-9.8653E-10
21.724	0.44444	-9.8653E-10
21.724	0.33333	-9.8653E-10
21.724	0.22222	-9.8653E-10
21.724	0.11111	-9.8652E-10

d (um)	k0Sub (cm/s)	Probe variable bnd4 (A)
21.724	0.0000	-9.7883E-10
21.472	1.0000	-9.8655E-10
21.472	0.88889	-9.8655E-10
21.472	0.77778	-9.8655E-10
21.472	0.66667	-9.8655E-10
21.472	0.55556	-9.8655E-10
21.472	0.44444	-9.8655E-10
21.472	0.33333	-9.8655E-10
21.472	0.22222	-9.8655E-10
21.472	0.11111	-9.8655E-10
21.472	0.0000	-9.7872E-10
21.220	1.0000	-9.8645E-10
21.220	0.88889	-9.8645E-10
21.220	0.77778	-9.8645E-10
21.220	0.66667	-9.8645E-10
21.220	0.55556	-9.8645E-10
21.220	0.44444	-9.8645E-10
21.220	0.33333	-9.8645E-10
21.220	0.22222	-9.8645E-10
21.220	0.11111	-9.8645E-10
21.220	0.0000	-9.7848E-10
20.968	1.0000	-9.8667E-10
20.968	0.88889	-9.8667E-10
20.968	0.77778	-9.8667E-10
20.968	0.66667	-9.8667E-10
20.968	0.55556	-9.8667E-10
20.968	0.44444	-9.8667E-10
20.968	0.33333	-9.8667E-10
20.968	0.22222	-9.8667E-10
20.968	0.11111	-9.8667E-10
20.968	0.0000	-9.7856E-10
20.716	1.0000	-9.8667E-10
20.716	0.88889	-9.8667E-10
20.716	0.77778	-9.8667E-10
20.716	0.66667	-9.8667E-10
20.716	0.55556	-9.8667E-10
20.716	0.44444	-9.8667E-10
20.716	0.33333	-9.8666E-10
20.716	0.22222	-9.8666E-10
20.716	0.11111	-9.8666E-10

d (um)	k0Sub (cm/s)	Probe variable bnd4 (A)
20.716	0.0000	-9.7841E-10
20.464	1.0000	-9.8678E-10
20.464	0.88889	-9.8678E-10
20.464	0.77778	-9.8678E-10
20.464	0.66667	-9.8678E-10
20.464	0.55556	-9.8678E-10
20.464	0.44444	-9.8678E-10
20.464	0.33333	-9.8678E-10
20.464	0.22222	-9.8678E-10
20.464	0.11111	-9.8677E-10
20.464	0.0000	-9.7837E-10
20.212	1.0000	-9.8690E-10
20.212	0.88889	-9.8690E-10
20.212	0.77778	-9.8690E-10
20.212	0.66667	-9.8690E-10
20.212	0.55556	-9.8690E-10
20.212	0.44444	-9.8690E-10
20.212	0.33333	-9.8690E-10
20.212	0.22222	-9.8690E-10
20.212	0.11111	-9.8690E-10
20.212	0.0000	-9.7834E-10
19.960	1.0000	-9.8709E-10
19.960	0.88889	-9.8709E-10
19.960	0.77778	-9.8709E-10
19.960	0.66667	-9.8709E-10
19.960	0.55556	-9.8709E-10
19.960	0.44444	-9.8709E-10
19.960	0.33333	-9.8709E-10
19.960	0.22222	-9.8709E-10
19.960	0.11111	-9.8708E-10
19.960	0.0000	-9.7836E-10
19.708	1.0000	-9.8695E-10
19.708	0.88889	-9.8695E-10
19.708	0.77778	-9.8695E-10
19.708	0.66667	-9.8695E-10
19.708	0.55556	-9.8695E-10
19.708	0.44444	-9.8695E-10
19.708	0.33333	-9.8695E-10
19.708	0.22222	-9.8695E-10
19.708	0.11111	-9.8694E-10

d (um)	k0Sub (cm/s)	Probe variable bnd4 (A)
19.708	0.0000	-9.7806E-10
19.456	1.0000	-9.8696E-10
19.456	0.88889	-9.8696E-10
19.456	0.77778	-9.8696E-10
19.456	0.66667	-9.8696E-10
19.456	0.55556	-9.8696E-10
19.456	0.44444	-9.8696E-10
19.456	0.33333	-9.8696E-10
19.456	0.22222	-9.8696E-10
19.456	0.11111	-9.8696E-10
19.456	0.0000	-9.7790E-10
19.204	1.0000	-9.8713E-10
19.204	0.88889	-9.8713E-10
19.204	0.77778	-9.8713E-10
19.204	0.66667	-9.8713E-10
19.204	0.55556	-9.8713E-10
19.204	0.44444	-9.8713E-10
19.204	0.33333	-9.8713E-10
19.204	0.22222	-9.8712E-10
19.204	0.11111	-9.8712E-10
19.204	0.0000	-9.7789E-10
18.952	1.0000	-9.8747E-10
18.952	0.88889	-9.8747E-10
18.952	0.77778	-9.8747E-10
18.952	0.66667	-9.8747E-10
18.952	0.55556	-9.8747E-10
18.952	0.44444	-9.8747E-10
18.952	0.33333	-9.8747E-10
18.952	0.22222	-9.8747E-10
18.952	0.11111	-9.8746E-10
18.952	0.0000	-9.7804E-10
18.699	1.0000	-9.8730E-10
18.699	0.88889	-9.8730E-10
18.699	0.77778	-9.8730E-10
18.699	0.66667	-9.8730E-10
18.699	0.55556	-9.8730E-10
18.699	0.44444	-9.8730E-10
18.699	0.33333	-9.8730E-10
18.699	0.22222	-9.8730E-10
18.699	0.11111	-9.8730E-10

d (um)	k0Sub (cm/s)	Probe variable bnd4 (A)
18.699	0.0000	-9.7770E-10
18.447	1.0000	-9.8746E-10
18.447	0.88889	-9.8746E-10
18.447	0.77778	-9.8746E-10
18.447	0.66667	-9.8746E-10
18.447	0.55556	-9.8746E-10
18.447	0.44444	-9.8746E-10
18.447	0.33333	-9.8746E-10
18.447	0.22222	-9.8746E-10
18.447	0.11111	-9.8746E-10
18.447	0.0000	-9.7766E-10
18.195	1.0000	-9.8754E-10
18.195	0.88889	-9.8754E-10
18.195	0.77778	-9.8754E-10
18.195	0.66667	-9.8754E-10
18.195	0.55556	-9.8754E-10
18.195	0.44444	-9.8754E-10
18.195	0.33333	-9.8754E-10
18.195	0.22222	-9.8754E-10
18.195	0.11111	-9.8753E-10
18.195	0.0000	-9.7753E-10
17.943	1.0000	-9.8768E-10
17.943	0.88889	-9.8768E-10
17.943	0.77778	-9.8768E-10
17.943	0.66667	-9.8768E-10
17.943	0.55556	-9.8768E-10
17.943	0.44444	-9.8768E-10
17.943	0.33333	-9.8768E-10
17.943	0.22222	-9.8767E-10
17.943	0.11111	-9.8767E-10
17.943	0.0000	-9.7746E-10
17.691	1.0000	-9.8768E-10
17.691	0.88889	-9.8768E-10
17.691	0.77778	-9.8768E-10
17.691	0.66667	-9.8768E-10
17.691	0.55556	-9.8768E-10
17.691	0.44444	-9.8768E-10
17.691	0.33333	-9.8768E-10
17.691	0.22222	-9.8768E-10
17.691	0.11111	-9.8767E-10

d (um)	k0Sub (cm/s)	Probe variable bnd4 (A)
17.691	0.0000	-9.7726E-10
17.439	1.0000	-9.8784E-10
17.439	0.88889	-9.8784E-10
17.439	0.77778	-9.8784E-10
17.439	0.66667	-9.8784E-10
17.439	0.55556	-9.8784E-10
17.439	0.44444	-9.8784E-10
17.439	0.33333	-9.8784E-10
17.439	0.22222	-9.8784E-10
17.439	0.11111	-9.8783E-10
17.439	0.0000	-9.7719E-10
17.187	1.0000	-9.8787E-10
17.187	0.88889	-9.8787E-10
17.187	0.77778	-9.8787E-10
17.187	0.66667	-9.8787E-10
17.187	0.55556	-9.8787E-10
17.187	0.44444	-9.8787E-10
17.187	0.33333	-9.8787E-10
17.187	0.22222	-9.8787E-10
17.187	0.11111	-9.8787E-10
17.187	0.0000	-9.7700E-10
16.935	1.0000	-9.8812E-10
16.935	0.88889	-9.8812E-10
16.935	0.77778	-9.8812E-10
16.935	0.66667	-9.8812E-10
16.935	0.55556	-9.8812E-10
16.935	0.44444	-9.8812E-10
16.935	0.33333	-9.8812E-10
16.935	0.22222	-9.8811E-10
16.935	0.11111	-9.8811E-10
16.935	0.0000	-9.7700E-10
16.683	1.0000	-9.8831E-10
16.683	0.88889	-9.8831E-10
16.683	0.77778	-9.8831E-10
16.683	0.66667	-9.8831E-10
16.683	0.55556	-9.8831E-10
16.683	0.44444	-9.8831E-10
16.683	0.33333	-9.8830E-10
16.683	0.22222	-9.8830E-10
16.683	0.11111	-9.8830E-10

d (um)	k0Sub (cm/s)	Probe variable bnd4 (A)
16.683	0.0000	-9.7694E-10
16.431	1.0000	-9.8832E-10
16.431	0.88889	-9.8832E-10
16.431	0.77778	-9.8832E-10
16.431	0.66667	-9.8832E-10
16.431	0.55556	-9.8832E-10
16.431	0.44444	-9.8832E-10
16.431	0.33333	-9.8832E-10
16.431	0.22222	-9.8832E-10
16.431	0.11111	-9.8831E-10
16.431	0.0000	-9.7670E-10
16.179	1.0000	-9.8847E-10
16.179	0.88889	-9.8847E-10
16.179	0.77778	-9.8847E-10
16.179	0.66667	-9.8847E-10
16.179	0.55556	-9.8847E-10
16.179	0.44444	-9.8847E-10
16.179	0.33333	-9.8847E-10
16.179	0.22222	-9.8847E-10
16.179	0.11111	-9.8847E-10
16.179	0.0000	-9.7658E-10
15.927	1.0000	-9.8868E-10
15.927	0.88889	-9.8868E-10
15.927	0.77778	-9.8868E-10
15.927	0.66667	-9.8868E-10
15.927	0.55556	-9.8868E-10
15.927	0.44444	-9.8868E-10
15.927	0.33333	-9.8868E-10
15.927	0.22222	-9.8868E-10
15.927	0.11111	-9.8867E-10
15.927	0.0000	-9.7651E-10
15.675	1.0000	-9.8862E-10
15.675	0.88889	-9.8862E-10
15.675	0.77778	-9.8862E-10
15.675	0.66667	-9.8862E-10
15.675	0.55556	-9.8862E-10
15.675	0.44444	-9.8862E-10
15.675	0.33333	-9.8862E-10
15.675	0.22222	-9.8862E-10
15.675	0.11111	-9.8861E-10

d (um)	k0Sub (cm/s)	Probe variable bnd4 (A)
15.675	0.0000	-9.7617E-10
15.423	1.0000	-9.8882E-10
15.423	0.88889	-9.8882E-10
15.423	0.77778	-9.8882E-10
15.423	0.66667	-9.8882E-10
15.423	0.55556	-9.8882E-10
15.423	0.44444	-9.8882E-10
15.423	0.33333	-9.8882E-10
15.423	0.22222	-9.8882E-10
15.423	0.11111	-9.8881E-10
15.423	0.0000	-9.7606E-10
15.171	1.0000	-9.8887E-10
15.171	0.88889	-9.8887E-10
15.171	0.77778	-9.8887E-10
15.171	0.66667	-9.8887E-10
15.171	0.55556	-9.8887E-10
15.171	0.44444	-9.8887E-10
15.171	0.33333	-9.8886E-10
15.171	0.22222	-9.8886E-10
15.171	0.11111	-9.8886E-10
15.171	0.0000	-9.7580E-10
14.919	1.0000	-9.8908E-10
14.919	0.88889	-9.8908E-10
14.919	0.77778	-9.8908E-10
14.919	0.66667	-9.8908E-10
14.919	0.55556	-9.8908E-10
14.919	0.44444	-9.8908E-10
14.919	0.33333	-9.8908E-10
14.919	0.22222	-9.8908E-10
14.919	0.11111	-9.8907E-10
14.919	0.0000	-9.7569E-10
14.667	1.0000	-9.8935E-10
14.667	0.88889	-9.8935E-10
14.667	0.77778	-9.8935E-10
14.667	0.66667	-9.8935E-10
14.667	0.55556	-9.8935E-10
14.667	0.44444	-9.8935E-10
14.667	0.33333	-9.8935E-10
14.667	0.22222	-9.8934E-10
14.667	0.11111	-9.8934E-10

d (um)	k0Sub (cm/s)	Probe variable bnd4 (A)
14.667	0.0000	-9.7561E-10
14.415	1.0000	-9.8948E-10
14.415	0.88889	-9.8948E-10
14.415	0.77778	-9.8948E-10
14.415	0.66667	-9.8948E-10
14.415	0.55556	-9.8948E-10
14.415	0.44444	-9.8948E-10
14.415	0.33333	-9.8947E-10
14.415	0.22222	-9.8947E-10
14.415	0.11111	-9.8947E-10
14.415	0.0000	-9.7539E-10
14.163	1.0000	-9.8967E-10
14.163	0.88889	-9.8967E-10
14.163	0.77778	-9.8967E-10
14.163	0.66667	-9.8967E-10
14.163	0.55556	-9.8967E-10
14.163	0.44444	-9.8967E-10
14.163	0.33333	-9.8967E-10
14.163	0.22222	-9.8966E-10
14.163	0.11111	-9.8966E-10
14.163	0.0000	-9.7521E-10
13.911	1.0000	-9.8982E-10
13.911	0.88889	-9.8982E-10
13.911	0.77778	-9.8981E-10
13.911	0.66667	-9.8981E-10
13.911	0.55556	-9.8981E-10
13.911	0.44444	-9.8981E-10
13.911	0.33333	-9.8981E-10
13.911	0.22222	-9.8981E-10
13.911	0.11111	-9.8980E-10
13.911	0.0000	-9.7497E-10
13.659	1.0000	-9.8992E-10
13.659	0.88889	-9.8992E-10
13.659	0.77778	-9.8992E-10
13.659	0.66667	-9.8992E-10
13.659	0.55556	-9.8992E-10
13.659	0.44444	-9.8992E-10
13.659	0.33333	-9.8992E-10
13.659	0.22222	-9.8991E-10
13.659	0.11111	-9.8991E-10

d (um)	k0Sub (cm/s)	Probe variable bnd4 (A)
13.659	0.0000	-9.7468E-10
13.407	1.0000	-9.9029E-10
13.407	0.88889	-9.9029E-10
13.407	0.77778	-9.9029E-10
13.407	0.66667	-9.9029E-10
13.407	0.55556	-9.9029E-10
13.407	0.44444	-9.9029E-10
13.407	0.33333	-9.9029E-10
13.407	0.22222	-9.9029E-10
13.407	0.11111	-9.9028E-10
13.407	0.0000	-9.7463E-10
13.155	1.0000	-9.9033E-10
13.155	0.88889	-9.9033E-10
13.155	0.77778	-9.9033E-10
13.155	0.66667	-9.9033E-10
13.155	0.55556	-9.9033E-10
13.155	0.44444	-9.9033E-10
13.155	0.33333	-9.9033E-10
13.155	0.22222	-9.9033E-10
13.155	0.11111	-9.9032E-10
13.155	0.0000	-9.7423E-10
12.903	1.0000	-9.9066E-10
12.903	0.88889	-9.9066E-10
12.903	0.77778	-9.9066E-10
12.903	0.66667	-9.9066E-10
12.903	0.55556	-9.9066E-10
12.903	0.44444	-9.9066E-10
12.903	0.33333	-9.9066E-10
12.903	0.22222	-9.9066E-10
12.903	0.11111	-9.9065E-10
12.903	0.0000	-9.7409E-10
12.651	1.0000	-9.9079E-10
12.651	0.88889	-9.9079E-10
12.651	0.77778	-9.9079E-10
12.651	0.66667	-9.9078E-10
12.651	0.55556	-9.9078E-10
12.651	0.44444	-9.9078E-10
12.651	0.33333	-9.9078E-10
12.651	0.22222	-9.9078E-10
12.651	0.11111	-9.9077E-10

d (um)	k0Sub (cm/s)	Probe variable bnd4 (A)
12.651	0.0000	-9.7373E-10
12.399	1.0000	-9.9116E-10
12.399	0.88889	-9.9115E-10
12.399	0.77778	-9.9115E-10
12.399	0.66667	-9.9115E-10
12.399	0.55556	-9.9115E-10
12.399	0.44444	-9.9115E-10
12.399	0.33333	-9.9115E-10
12.399	0.22222	-9.9115E-10
12.399	0.11111	-9.9114E-10
12.399	0.0000	-9.7358E-10
12.147	1.0000	-9.9137E-10
12.147	0.88889	-9.9137E-10
12.147	0.77778	-9.9137E-10
12.147	0.66667	-9.9137E-10
12.147	0.55556	-9.9137E-10
12.147	0.44444	-9.9136E-10
12.147	0.33333	-9.9136E-10
12.147	0.22222	-9.9136E-10
12.147	0.11111	-9.9135E-10
12.147	0.0000	-9.7326E-10
11.895	1.0000	-9.9170E-10
11.895	0.88889	-9.9170E-10
11.895	0.77778	-9.9170E-10
11.895	0.66667	-9.9170E-10
11.895	0.55556	-9.9170E-10
11.895	0.44444	-9.9170E-10
11.895	0.33333	-9.9170E-10
11.895	0.22222	-9.9169E-10
11.895	0.11111	-9.9168E-10
11.895	0.0000	-9.7302E-10
11.643	1.0000	-9.9190E-10
11.643	0.88889	-9.9190E-10
11.643	0.77778	-9.9190E-10
11.643	0.66667	-9.9190E-10
11.643	0.55556	-9.9189E-10
11.643	0.44444	-9.9189E-10
11.643	0.33333	-9.9189E-10
11.643	0.22222	-9.9189E-10
11.643	0.11111	-9.9188E-10

d (um)	k0Sub (cm/s)	Probe variable bnd4 (A)
11.643	0.0000	-9.7263E-10
11.391	1.0000	-9.9221E-10
11.391	0.88889	-9.9221E-10
11.391	0.77778	-9.9221E-10
11.391	0.66667	-9.9220E-10
11.391	0.55556	-9.9220E-10
11.391	0.44444	-9.9220E-10
11.391	0.33333	-9.9220E-10
11.391	0.22222	-9.9220E-10
11.391	0.11111	-9.9219E-10
11.391	0.0000	-9.7230E-10
11.139	1.0000	-9.9260E-10
11.139	0.88889	-9.9260E-10
11.139	0.77778	-9.9260E-10
11.139	0.66667	-9.9260E-10
11.139	0.55556	-9.9259E-10
11.139	0.44444	-9.9259E-10
11.139	0.33333	-9.9259E-10
11.139	0.22222	-9.9259E-10
11.139	0.11111	-9.9258E-10
11.139	0.0000	-9.7202E-10
10.887	1.0000	-9.9291E-10
10.887	0.88889	-9.9291E-10
10.887	0.77778	-9.9291E-10
10.887	0.66667	-9.9291E-10
10.887	0.55556	-9.9291E-10
10.887	0.44444	-9.9291E-10
10.887	0.33333	-9.9290E-10
10.887	0.22222	-9.9290E-10
10.887	0.11111	-9.9289E-10
10.887	0.0000	-9.7163E-10
10.635	1.0000	-9.9327E-10
10.635	0.88889	-9.9327E-10
10.635	0.77778	-9.9327E-10
10.635	0.66667	-9.9327E-10
10.635	0.55556	-9.9327E-10
10.635	0.44444	-9.9327E-10
10.635	0.33333	-9.9326E-10
10.635	0.22222	-9.9326E-10
10.635	0.11111	-9.9325E-10

d (um)	k0Sub (cm/s)	Probe variable bnd4 (A)
10.635	0.0000	-9.7125E-10
10.383	1.0000	-9.9360E-10
10.383	0.88889	-9.9360E-10
10.383	0.77778	-9.9360E-10
10.383	0.66667	-9.9360E-10
10.383	0.55556	-9.9360E-10
10.383	0.44444	-9.9359E-10
10.383	0.33333	-9.9359E-10
10.383	0.22222	-9.9359E-10
10.383	0.11111	-9.9357E-10
10.383	0.0000	-9.7078E-10
10.131	1.0000	-9.9401E-10
10.131	0.88889	-9.9401E-10
10.131	0.77778	-9.9401E-10
10.131	0.66667	-9.9401E-10
10.131	0.55556	-9.9401E-10
10.131	0.44444	-9.9401E-10
10.131	0.33333	-9.9400E-10
10.131	0.22222	-9.9400E-10
10.131	0.11111	-9.9398E-10
10.131	0.0000	-9.7035E-10
9.8788	1.0000	-9.9441E-10
9.8788	0.88889	-9.9441E-10
9.8788	0.77778	-9.9441E-10
9.8788	0.66667	-9.9440E-10
9.8788	0.55556	-9.9440E-10
9.8788	0.44444	-9.9440E-10
9.8788	0.33333	-9.9440E-10
9.8788	0.22222	-9.9439E-10
9.8788	0.11111	-9.9438E-10
9.8788	0.0000	-9.6985E-10
9.6268	1.0000	-9.9499E-10
9.6268	0.88889	-9.9499E-10
9.6268	0.77778	-9.9499E-10
9.6268	0.66667	-9.9499E-10
9.6268	0.55556	-9.9499E-10
9.6268	0.44444	-9.9499E-10
9.6268	0.33333	-9.9498E-10
9.6268	0.22222	-9.9498E-10
9.6268	0.11111	-9.9496E-10

d (um)	k0Sub (cm/s)	Probe variable bnd4 (A)
9.6268	0.0000	-9.6947E-10
9.3747	1.0000	-9.9544E-10
9.3747	0.88889	-9.9544E-10
9.3747	0.77778	-9.9544E-10
9.3747	0.66667	-9.9543E-10
9.3747	0.55556	-9.9543E-10
9.3747	0.44444	-9.9543E-10
9.3747	0.33333	-9.9543E-10
9.3747	0.22222	-9.9542E-10
9.3747	0.11111	-9.9541E-10
9.3747	0.0000	-9.6890E-10
9.1227	1.0000	-9.9593E-10
9.1227	0.88889	-9.9593E-10
9.1227	0.77778	-9.9593E-10
9.1227	0.66667	-9.9593E-10
9.1227	0.55556	-9.9593E-10
9.1227	0.44444	-9.9592E-10
9.1227	0.33333	-9.9592E-10
9.1227	0.22222	-9.9591E-10
9.1227	0.11111	-9.9590E-10
9.1227	0.0000	-9.6829E-10
8.8707	1.0000	-9.9651E-10
8.8707	0.88889	-9.9651E-10
8.8707	0.77778	-9.9651E-10
8.8707	0.66667	-9.9651E-10
8.8707	0.55556	-9.9650E-10
8.8707	0.44444	-9.9650E-10
8.8707	0.33333	-9.9650E-10
8.8707	0.22222	-9.9649E-10
8.8707	0.11111	-9.9647E-10
8.8707	0.0000	-9.6770E-10
8.6187	1.0000	-9.9708E-10
8.6187	0.88889	-9.9708E-10
8.6187	0.77778	-9.9708E-10
8.6187	0.66667	-9.9708E-10
8.6187	0.55556	-9.9708E-10
8.6187	0.44444	-9.9708E-10
8.6187	0.33333	-9.9707E-10
8.6187	0.22222	-9.9707E-10
8.6187	0.11111	-9.9704E-10

d (um)	k0Sub (cm/s)	Probe variable bnd4 (A)
8.6187	0.0000	-9.6702E-10
8.3667	1.0000	-9.9769E-10
8.3667	0.88889	-9.9769E-10
8.3667	0.77778	-9.9769E-10
8.3667	0.66667	-9.9769E-10
8.3667	0.55556	-9.9768E-10
8.3667	0.44444	-9.9768E-10
8.3667	0.33333	-9.9768E-10
8.3667	0.22222	-9.9767E-10
8.3667	0.11111	-9.9765E-10
8.3667	0.0000	-9.6627E-10
8.1146	1.0000	-9.9826E-10
8.1146	0.88889	-9.9826E-10
8.1146	0.77778	-9.9826E-10
8.1146	0.66667	-9.9826E-10
8.1146	0.55556	-9.9826E-10
8.1146	0.44444	-9.9826E-10
8.1146	0.33333	-9.9825E-10
8.1146	0.22222	-9.9824E-10
8.1146	0.11111	-9.9822E-10
8.1146	0.0000	-9.6540E-10
7.8626	1.0000	-9.9901E-10
7.8626	0.88889	-9.9901E-10
7.8626	0.77778	-9.9901E-10
7.8626	0.66667	-9.9901E-10
7.8626	0.55556	-9.9901E-10
7.8626	0.44444	-9.9901E-10
7.8626	0.33333	-9.9900E-10
7.8626	0.22222	-9.9899E-10
7.8626	0.11111	-9.9897E-10
7.8626	0.0000	-9.6457E-10
7.6106	1.0000	-9.9985E-10
7.6106	0.88889	-9.9984E-10
7.6106	0.77778	-9.9984E-10
7.6106	0.66667	-9.9984E-10
7.6106	0.55556	-9.9984E-10
7.6106	0.44444	-9.9984E-10
7.6106	0.33333	-9.9983E-10
7.6106	0.22222	-9.9982E-10
7.6106	0.11111	-9.9979E-10

d (um)	k0Sub (cm/s)	Probe variable bnd4 (A)
7.6106	0.0000	-9.6369E-10
7.3586	1.0000	-1.0006E-9
7.3586	0.88889	-1.0006E-9
7.3586	0.77778	-1.0006E-9
7.3586	0.66667	-1.0006E-9
7.3586	0.55556	-1.0006E-9
7.3586	0.44444	-1.0006E-9
7.3586	0.33333	-1.0006E-9
7.3586	0.22222	-1.0006E-9
7.3586	0.11111	-1.0006E-9
7.3586	0.0000	-9.6261E-10
7.1066	1.0000	-1.0016E-9
7.1066	0.88889	-1.0016E-9
7.1066	0.77778	-1.0016E-9
7.1066	0.66667	-1.0016E-9
7.1066	0.55556	-1.0016E-9
7.1066	0.44444	-1.0016E-9
7.1066	0.33333	-1.0016E-9
7.1066	0.22222	-1.0016E-9
7.1066	0.11111	-1.0016E-9
7.1066	0.0000	-9.6157E-10
6.8545	1.0000	-1.0027E-9
6.8545	0.88889	-1.0027E-9
6.8545	0.77778	-1.0027E-9
6.8545	0.66667	-1.0027E-9
6.8545	0.55556	-1.0027E-9
6.8545	0.44444	-1.0027E-9
6.8545	0.33333	-1.0026E-9
6.8545	0.22222	-1.0026E-9
6.8545	0.11111	-1.0026E-9
6.8545	0.0000	-9.6039E-10
6.6025	1.0000	-1.0036E-9
6.6025	0.88889	-1.0036E-9
6.6025	0.77778	-1.0036E-9
6.6025	0.66667	-1.0036E-9
6.6025	0.55556	-1.0036E-9
6.6025	0.44444	-1.0036E-9
6.6025	0.33333	-1.0036E-9
6.6025	0.22222	-1.0036E-9
6.6025	0.11111	-1.0036E-9

d (um)	k0Sub (cm/s)	Probe variable bnd4 (A)
6.6025	0.0000	-9.5893E-10
6.3505	1.0000	-1.0049E-9
6.3505	0.88889	-1.0049E-9
6.3505	0.77778	-1.0049E-9
6.3505	0.66667	-1.0049E-9
6.3505	0.55556	-1.0049E-9
6.3505	0.44444	-1.0049E-9
6.3505	0.33333	-1.0049E-9
6.3505	0.22222	-1.0048E-9
6.3505	0.11111	-1.0048E-9
6.3505	0.0000	-9.5750E-10
6.0985	1.0000	-1.0046E-9
6.0985	0.88889	-1.0046E-9
6.0985	0.77778	-1.0046E-9
6.0985	0.66667	-1.0046E-9
6.0985	0.55556	-1.0046E-9
6.0985	0.44444	-1.0046E-9
6.0985	0.33333	-1.0045E-9
6.0985	0.22222	-1.0045E-9
6.0985	0.11111	-1.0045E-9
6.0985	0.0000	-9.5434E-10
5.8465	1.0000	-1.0077E-9
5.8465	0.88889	-1.0077E-9
5.8465	0.77778	-1.0077E-9
5.8465	0.66667	-1.0077E-9
5.8465	0.55556	-1.0077E-9
5.8465	0.44444	-1.0077E-9
5.8465	0.33333	-1.0077E-9
5.8465	0.22222	-1.0077E-9
5.8465	0.11111	-1.0076E-9
5.8465	0.0000	-9.5413E-10
5.5944	1.0000	-1.0093E-9
5.5944	0.88889	-1.0093E-9
5.5944	0.77778	-1.0093E-9
5.5944	0.66667	-1.0093E-9
5.5944	0.55556	-1.0093E-9
5.5944	0.44444	-1.0093E-9
5.5944	0.33333	-1.0093E-9
5.5944	0.22222	-1.0093E-9
5.5944	0.11111	-1.0092E-9

d (um)	k0Sub (cm/s)	Probe variable bnd4 (A)
5.5944	0.0000	-9.5206E-10
5.3424	1.0000	-1.0111E-9
5.3424	0.88889	-1.0111E-9
5.3424	0.77778	-1.0111E-9
5.3424	0.66667	-1.0111E-9
5.3424	0.55556	-1.0111E-9
5.3424	0.44444	-1.0111E-9
5.3424	0.33333	-1.0111E-9
5.3424	0.22222	-1.0111E-9
5.3424	0.11111	-1.0110E-9
5.3424	0.0000	-9.4981E-10
5.0904	1.0000	-1.0130E-9
5.0904	0.88889	-1.0130E-9
5.0904	0.77778	-1.0130E-9
5.0904	0.66667	-1.0130E-9
5.0904	0.55556	-1.0130E-9
5.0904	0.44444	-1.0130E-9
5.0904	0.33333	-1.0130E-9
5.0904	0.22222	-1.0129E-9
5.0904	0.11111	-1.0129E-9
5.0904	0.0000	-9.4715E-10
4.8384	1.0000	-1.0154E-9
4.8384	0.88889	-1.0154E-9
4.8384	0.77778	-1.0154E-9
4.8384	0.66667	-1.0154E-9
4.8384	0.55556	-1.0154E-9
4.8384	0.44444	-1.0154E-9
4.8384	0.33333	-1.0154E-9
4.8384	0.22222	-1.0154E-9
4.8384	0.11111	-1.0153E-9
4.8384	0.0000	-9.4437E-10
4.5864	1.0000	-1.0177E-9
4.5864	0.88889	-1.0177E-9
4.5864	0.77778	-1.0177E-9
4.5864	0.66667	-1.0177E-9
4.5864	0.55556	-1.0177E-9
4.5864	0.44444	-1.0177E-9
4.5864	0.33333	-1.0177E-9
4.5864	0.22222	-1.0176E-9
4.5864	0.11111	-1.0175E-9

d (um)	k0Sub (cm/s)	Probe variable bnd4 (A)
4.5864	0.0000	-9.4085E-10
4.3343	1.0000	-1.0206E-9
4.3343	0.88889	-1.0206E-9
4.3343	0.77778	-1.0206E-9
4.3343	0.66667	-1.0206E-9
4.3343	0.55556	-1.0206E-9
4.3343	0.44444	-1.0206E-9
4.3343	0.33333	-1.0205E-9
4.3343	0.22222	-1.0205E-9
4.3343	0.11111	-1.0204E-9
4.3343	0.0000	-9.3706E-10
4.0823	1.0000	-1.0237E-9
4.0823	0.88889	-1.0237E-9
4.0823	0.77778	-1.0237E-9
4.0823	0.66667	-1.0237E-9
4.0823	0.55556	-1.0237E-9
4.0823	0.44444	-1.0237E-9
4.0823	0.33333	-1.0237E-9
4.0823	0.22222	-1.0236E-9
4.0823	0.11111	-1.0235E-9
4.0823	0.0000	-9.3257E-10
3.8303	1.0000	-1.0275E-9
3.8303	0.88889	-1.0275E-9
3.8303	0.77778	-1.0275E-9
3.8303	0.66667	-1.0275E-9
3.8303	0.55556	-1.0275E-9
3.8303	0.44444	-1.0275E-9
3.8303	0.33333	-1.0274E-9
3.8303	0.22222	-1.0274E-9
3.8303	0.11111	-1.0273E-9
3.8303	0.0000	-9.2748E-10
3.5783	1.0000	-1.0318E-9
3.5783	0.88889	-1.0318E-9
3.5783	0.77778	-1.0317E-9
3.5783	0.66667	-1.0317E-9
3.5783	0.55556	-1.0317E-9
3.5783	0.44444	-1.0317E-9
3.5783	0.33333	-1.0317E-9
3.5783	0.22222	-1.0316E-9
3.5783	0.11111	-1.0315E-9

d (um)	k0Sub (cm/s)	Probe variable bnd4 (A)
3.5783	0.0000	-9.2138E-10
3.3263	1.0000	-1.0368E-9
3.3263	0.88889	-1.0368E-9
3.3263	0.77778	-1.0368E-9
3.3263	0.66667	-1.0368E-9
3.3263	0.55556	-1.0368E-9
3.3263	0.44444	-1.0368E-9
3.3263	0.33333	-1.0367E-9
3.3263	0.22222	-1.0367E-9
3.3263	0.11111	-1.0365E-9
3.3263	0.0000	-9.1429E-10
3.0742	1.0000	-1.0425E-9
3.0742	0.88889	-1.0425E-9
3.0742	0.77778	-1.0425E-9
3.0742	0.66667	-1.0425E-9
3.0742	0.55556	-1.0424E-9
3.0742	0.44444	-1.0424E-9
3.0742	0.33333	-1.0424E-9
3.0742	0.22222	-1.0423E-9
3.0742	0.11111	-1.0421E-9
3.0742	0.0000	-9.0552E-10
2.8222	1.0000	-1.0495E-9
2.8222	0.88889	-1.0495E-9
2.8222	0.77778	-1.0495E-9
2.8222	0.66667	-1.0495E-9
2.8222	0.55556	-1.0495E-9
2.8222	0.44444	-1.0495E-9
2.8222	0.33333	-1.0494E-9
2.8222	0.22222	-1.0493E-9
2.8222	0.11111	-1.0490E-9
2.8222	0.0000	-8.9517E-10
2.5702	1.0000	-1.0581E-9
2.5702	0.88889	-1.0581E-9
2.5702	0.77778	-1.0581E-9
2.5702	0.66667	-1.0581E-9
2.5702	0.55556	-1.0581E-9
2.5702	0.44444	-1.0580E-9
2.5702	0.33333	-1.0580E-9
2.5702	0.22222	-1.0579E-9
2.5702	0.11111	-1.0575E-9

d (um)	k0Sub (cm/s)	Probe variable bnd4 (A)
2.5702	0.0000	-8.8249E-10
2.3182	1.0000	-1.0685E-9
2.3182	0.88889	-1.0685E-9
2.3182	0.77778	-1.0685E-9
2.3182	0.66667	-1.0685E-9
2.3182	0.55556	-1.0685E-9
2.3182	0.44444	-1.0684E-9
2.3182	0.33333	-1.0684E-9
2.3182	0.22222	-1.0682E-9
2.3182	0.11111	-1.0678E-9
2.3182	0.0000	-8.6662E-10
2.0662	1.0000	-1.0818E-9
2.0662	0.88889	-1.0818E-9
2.0662	0.77778	-1.0818E-9
2.0662	0.66667	-1.0818E-9
2.0662	0.55556	-1.0817E-9
2.0662	0.44444	-1.0817E-9
2.0662	0.33333	-1.0816E-9
2.0662	0.22222	-1.0814E-9
2.0662	0.11111	-1.0809E-9
2.0662	0.0000	-8.4660E-10
1.8141	1.0000	-1.0993E-9
1.8141	0.88889	-1.0992E-9
1.8141	0.77778	-1.0992E-9
1.8141	0.66667	-1.0992E-9
1.8141	0.55556	-1.0991E-9
1.8141	0.44444	-1.0991E-9
1.8141	0.33333	-1.0989E-9
1.8141	0.22222	-1.0987E-9
1.8141	0.11111	-1.0980E-9
1.8141	0.0000	-8.2100E-10
1.5621	1.0000	-1.1226E-9
1.5621	0.88889	-1.1226E-9
1.5621	0.77778	-1.1226E-9
1.5621	0.66667	-1.1225E-9
1.5621	0.55556	-1.1224E-9
1.5621	0.44444	-1.1223E-9
1.5621	0.33333	-1.1222E-9
1.5621	0.22222	-1.1218E-9
1.5621	0.11111	-1.1209E-9

d (um)	k0Sub (cm/s)	Probe variable bnd4 (A)
1.5621	0.0000	-7.8728E-10
1.3101	1.0000	-1.1562E-9
1.3101	0.88889	-1.1562E-9
1.3101	0.77778	-1.1561E-9
1.3101	0.66667	-1.1561E-9
1.3101	0.55556	-1.1560E-9
1.3101	0.44444	-1.1558E-9
1.3101	0.33333	-1.1556E-9
1.3101	0.22222	-1.1551E-9
1.3101	0.11111	-1.1536E-9
1.3101	0.0000	-7.4222E-10
1.0581	1.0000	-1.2077E-9
1.0581	0.88889	-1.2077E-9
1.0581	0.77778	-1.2076E-9
1.0581	0.66667	-1.2075E-9
1.0581	0.55556	-1.2073E-9
1.0581	0.44444	-1.2070E-9
1.0581	0.33333	-1.2066E-9
1.0581	0.22222	-1.2058E-9
1.0581	0.11111	-1.2034E-9
1.0581	0.0000	-6.8027E-10
0.80606	1.0000	-1.2960E-9
0.80606	0.88889	-1.2959E-9
0.80606	0.77778	-1.2957E-9
0.80606	0.66667	-1.2955E-9
0.80606	0.55556	-1.2952E-9
0.80606	0.44444	-1.2947E-9
0.80606	0.33333	-1.2939E-9
0.80606	0.22222	-1.2924E-9
0.80606	0.11111	-1.2879E-9
0.80606	0.0000	-5.9326E-10
0.55404	1.0000	-1.4754E-9
0.55404	0.88889	-1.4751E-9
0.55404	0.77778	-1.4747E-9
0.55404	0.66667	-1.4742E-9
0.55404	0.55556	-1.4735E-9
0.55404	0.44444	-1.4723E-9
0.55404	0.33333	-1.4705E-9
0.55404	0.22222	-1.4669E-9
0.55404	0.11111	-1.4563E-9

d (um)	k0Sub (cm/s)	Probe variable bnd4 (A)
0.55404	0.0000	-4.6869E-10
0.30202	1.0000	-1.9903E-9
0.30202	0.88889	-1.9890E-9
0.30202	0.77778	-1.9874E-9
0.30202	0.66667	-1.9852E-9
0.30202	0.55556	-1.9821E-9
0.30202	0.44444	-1.9775E-9
0.30202	0.33333	-1.9700E-9
0.30202	0.22222	-1.9554E-9
0.30202	0.11111	-1.9142E-9
0.30202	0.0000	-2.9184E-10
0.050000	1.0000	-7.4817E-9
0.050000	0.88889	-7.4070E-9
0.050000	0.77778	-7.3143E-9
0.050000	0.66667	-7.1961E-9
0.050000	0.55556	-7.0401E-9
0.050000	0.44444	-6.8244E-9
0.050000	0.33333	-6.5059E-9
0.050000	0.22222	-5.9843E-9
0.050000	0.11111	-4.9489E-9
0.050000	0.0000	-5.4551E-11

4.3.4 Table 5

Global Evaluation 3 (bnd4)

d (um)	k0Sub (cm/s)	Probe variable bnd4 (A)
20.000	1.0000	-9.8682E-10
20.000	0.50000	-9.8682E-10
20.000	0.10000	-9.8682E-10
20.000	0.050000	-9.8681E-10
20.000	0.010000	-9.8676E-10
20.000	0.0010000	-9.8626E-10
20.000	1.0000E-4	-9.8351E-10
20.000	0.0000	-9.7812E-10
19.798	1.0000	-9.8706E-10
19.798	0.50000	-9.8706E-10
19.798	0.10000	-9.8705E-10
19.798	0.050000	-9.8705E-10
19.798	0.010000	-9.8699E-10
19.798	0.0010000	-9.8648E-10

d (um)	k0Sub (cm/s)	Probe variable bnd4 (A)
19.798	1.0000E-4	-9.8368E-10
19.798	0.0000	-9.7822E-10
19.597	1.0000	-9.8686E-10
19.597	0.50000	-9.8686E-10
19.597	0.10000	-9.8685E-10
19.597	0.050000	-9.8685E-10
19.597	0.010000	-9.8679E-10
19.597	0.0010000	-9.8627E-10
19.597	1.0000E-4	-9.8342E-10
19.597	0.0000	-9.7789E-10
19.395	1.0000	-9.8704E-10
19.395	0.50000	-9.8704E-10
19.395	0.10000	-9.8703E-10
19.395	0.050000	-9.8702E-10
19.395	0.010000	-9.8697E-10
19.395	0.0010000	-9.8644E-10
19.395	1.0000E-4	-9.8353E-10
19.395	0.0000	-9.7793E-10
19.194	1.0000	-9.8711E-10
19.194	0.50000	-9.8711E-10
19.194	0.10000	-9.8711E-10
19.194	0.050000	-9.8710E-10
19.194	0.010000	-9.8705E-10
19.194	0.0010000	-9.8650E-10
19.194	1.0000E-4	-9.8353E-10
19.194	0.0000	-9.7787E-10
18.992	1.0000	-9.8725E-10
18.992	0.50000	-9.8725E-10
18.992	0.10000	-9.8725E-10
18.992	0.050000	-9.8724E-10
18.992	0.010000	-9.8718E-10
18.992	0.0010000	-9.8662E-10
18.992	1.0000E-4	-9.8360E-10
18.992	0.0000	-9.7786E-10
18.791	1.0000	-9.8732E-10
18.791	0.50000	-9.8732E-10
18.791	0.10000	-9.8731E-10
18.791	0.050000	-9.8730E-10
18.791	0.010000	-9.8725E-10
18.791	0.0010000	-9.8667E-10

d (um)	k0Sub (cm/s)	Probe variable bnd4 (A)
18.791	1.0000E-4	-9.8359E-10
18.791	0.0000	-9.7777E-10
18.589	1.0000	-9.8746E-10
18.589	0.50000	-9.8746E-10
18.589	0.10000	-9.8745E-10
18.589	0.050000	-9.8744E-10
18.589	0.010000	-9.8739E-10
18.589	0.0010000	-9.8680E-10
18.589	1.0000E-4	-9.8365E-10
18.589	0.0000	-9.7776E-10
18.388	1.0000	-9.8740E-10
18.388	0.50000	-9.8739E-10
18.388	0.10000	-9.8739E-10
18.388	0.050000	-9.8738E-10
18.388	0.010000	-9.8732E-10
18.388	0.0010000	-9.8672E-10
18.388	1.0000E-4	-9.8351E-10
18.388	0.0000	-9.7755E-10
18.186	1.0000	-9.8751E-10
18.186	0.50000	-9.8751E-10
18.186	0.10000	-9.8750E-10
18.186	0.050000	-9.8749E-10
18.186	0.010000	-9.8743E-10
18.186	0.0010000	-9.8681E-10
18.186	1.0000E-4	-9.8353E-10
18.186	0.0000	-9.7749E-10
17.985	1.0000	-9.8756E-10
17.985	0.50000	-9.8756E-10
17.985	0.10000	-9.8755E-10
17.985	0.050000	-9.8754E-10
17.985	0.010000	-9.8748E-10
17.985	0.0010000	-9.8684E-10
17.985	1.0000E-4	-9.8350E-10
17.985	0.0000	-9.7738E-10
17.783	1.0000	-9.8760E-10
17.783	0.50000	-9.8759E-10
17.783	0.10000	-9.8759E-10
17.783	0.050000	-9.8758E-10
17.783	0.010000	-9.8751E-10
17.783	0.0010000	-9.8686E-10

d (um)	k0Sub (cm/s)	Probe variable bnd4 (A)
17.783	1.0000E-4	-9.8345E-10
17.783	0.0000	-9.7725E-10
17.582	1.0000	-9.8770E-10
17.582	0.50000	-9.8770E-10
17.582	0.10000	-9.8769E-10
17.582	0.050000	-9.8768E-10
17.582	0.010000	-9.8762E-10
17.582	0.0010000	-9.8695E-10
17.582	1.0000E-4	-9.8347E-10
17.582	0.0000	-9.7718E-10
17.380	1.0000	-9.8786E-10
17.380	0.50000	-9.8786E-10
17.380	0.10000	-9.8786E-10
17.380	0.050000	-9.8785E-10
17.380	0.010000	-9.8778E-10
17.380	0.0010000	-9.8709E-10
17.380	1.0000E-4	-9.8353E-10
17.380	0.0000	-9.7716E-10
17.179	1.0000	-9.8791E-10
17.179	0.50000	-9.8791E-10
17.179	0.10000	-9.8790E-10
17.179	0.050000	-9.8789E-10
17.179	0.010000	-9.8782E-10
17.179	0.0010000	-9.8711E-10
17.179	1.0000E-4	-9.8348E-10
17.179	0.0000	-9.7702E-10
16.977	1.0000	-9.8806E-10
16.977	0.50000	-9.8806E-10
16.977	0.10000	-9.8805E-10
16.977	0.050000	-9.8804E-10
16.977	0.010000	-9.8797E-10
16.977	0.0010000	-9.8725E-10
16.977	1.0000E-4	-9.8353E-10
16.977	0.0000	-9.7698E-10
16.776	1.0000	-9.8821E-10
16.776	0.50000	-9.8821E-10
16.776	0.10000	-9.8820E-10
16.776	0.050000	-9.8819E-10
16.776	0.010000	-9.8811E-10
16.776	0.0010000	-9.8737E-10

d (um)	k0Sub (cm/s)	Probe variable bnd4 (A)
16.776	1.0000E-4	-9.8358E-10
16.776	0.0000	-9.7693E-10
16.574	1.0000	-9.8830E-10
16.574	0.50000	-9.8830E-10
16.574	0.10000	-9.8829E-10
16.574	0.050000	-9.8828E-10
16.574	0.010000	-9.8821E-10
16.574	0.0010000	-9.8744E-10
16.574	1.0000E-4	-9.8356E-10
16.574	0.0000	-9.7683E-10
16.373	1.0000	-9.8836E-10
16.373	0.50000	-9.8836E-10
16.373	0.10000	-9.8835E-10
16.373	0.050000	-9.8834E-10
16.373	0.010000	-9.8827E-10
16.373	0.0010000	-9.8748E-10
16.373	1.0000E-4	-9.8351E-10
16.373	0.0000	-9.7668E-10
16.171	1.0000	-9.8833E-10
16.171	0.50000	-9.8833E-10
16.171	0.10000	-9.8832E-10
16.171	0.050000	-9.8831E-10
16.171	0.010000	-9.8823E-10
16.171	0.0010000	-9.8742E-10
16.171	1.0000E-4	-9.8337E-10
16.171	0.0000	-9.7644E-10
15.970	1.0000	-9.8860E-10
15.970	0.50000	-9.8860E-10
15.970	0.10000	-9.8859E-10
15.970	0.050000	-9.8858E-10
15.970	0.010000	-9.8850E-10
15.970	0.0010000	-9.8767E-10
15.970	1.0000E-4	-9.8352E-10
15.970	0.0000	-9.7648E-10
15.768	1.0000	-9.8863E-10
15.768	0.50000	-9.8862E-10
15.768	0.10000	-9.8862E-10
15.768	0.050000	-9.8860E-10
15.768	0.010000	-9.8852E-10
15.768	0.0010000	-9.8766E-10

d (um)	k0Sub (cm/s)	Probe variable bnd4 (A)
15.768	1.0000E-4	-9.8341E-10
15.768	0.0000	-9.7628E-10
15.567	1.0000	-9.8875E-10
15.567	0.50000	-9.8874E-10
15.567	0.10000	-9.8874E-10
15.567	0.050000	-9.8872E-10
15.567	0.010000	-9.8863E-10
15.567	0.0010000	-9.8775E-10
15.567	1.0000E-4	-9.8341E-10
15.567	0.0000	-9.7616E-10
15.365	1.0000	-9.8895E-10
15.365	0.50000	-9.8895E-10
15.365	0.10000	-9.8894E-10
15.365	0.050000	-9.8893E-10
15.365	0.010000	-9.8884E-10
15.365	0.0010000	-9.8793E-10
15.365	1.0000E-4	-9.8348E-10
15.365	0.0000	-9.7612E-10
15.164	1.0000	-9.8891E-10
15.164	0.50000	-9.8891E-10
15.164	0.10000	-9.8890E-10
15.164	0.050000	-9.8889E-10
15.164	0.010000	-9.8880E-10
15.164	0.0010000	-9.8786E-10
15.164	1.0000E-4	-9.8330E-10
15.164	0.0000	-9.7584E-10
14.962	1.0000	-9.8913E-10
14.962	0.50000	-9.8913E-10
14.962	0.10000	-9.8912E-10
14.962	0.050000	-9.8911E-10
14.962	0.010000	-9.8901E-10
14.962	0.0010000	-9.8805E-10
14.962	1.0000E-4	-9.8338E-10
14.962	0.0000	-9.7580E-10
14.761	1.0000	-9.8925E-10
14.761	0.50000	-9.8925E-10
14.761	0.10000	-9.8924E-10
14.761	0.050000	-9.8923E-10
14.761	0.010000	-9.8913E-10
14.761	0.0010000	-9.8813E-10

d (um)	k0Sub (cm/s)	Probe variable bnd4 (A)
14.761	1.0000E-4	-9.8335E-10
14.761	0.0000	-9.7565E-10
14.559	1.0000	-9.8938E-10
14.559	0.50000	-9.8938E-10
14.559	0.10000	-9.8937E-10
14.559	0.050000	-9.8936E-10
14.559	0.010000	-9.8925E-10
14.559	0.0010000	-9.8822E-10
14.559	1.0000E-4	-9.8332E-10
14.559	0.0000	-9.7550E-10
14.358	1.0000	-9.8947E-10
14.358	0.50000	-9.8947E-10
14.358	0.10000	-9.8946E-10
14.358	0.050000	-9.8945E-10
14.358	0.010000	-9.8934E-10
14.358	0.0010000	-9.8828E-10
14.358	1.0000E-4	-9.8325E-10
14.358	0.0000	-9.7530E-10
14.156	1.0000	-9.8969E-10
14.156	0.50000	-9.8969E-10
14.156	0.10000	-9.8968E-10
14.156	0.050000	-9.8966E-10
14.156	0.010000	-9.8955E-10
14.156	0.0010000	-9.8846E-10
14.156	1.0000E-4	-9.8330E-10
14.156	0.0000	-9.7522E-10
13.955	1.0000	-9.8988E-10
13.955	0.50000	-9.8988E-10
13.955	0.10000	-9.8986E-10
13.955	0.050000	-9.8985E-10
13.955	0.010000	-9.8973E-10
13.955	0.0010000	-9.8860E-10
13.955	1.0000E-4	-9.8331E-10
13.955	0.0000	-9.7510E-10
13.753	1.0000	-9.8991E-10
13.753	0.50000	-9.8990E-10
13.753	0.10000	-9.8989E-10
13.753	0.050000	-9.8988E-10
13.753	0.010000	-9.8976E-10
13.753	0.0010000	-9.8859E-10

d (um)	k0Sub (cm/s)	Probe variable bnd4 (A)
13.753	1.0000E-4	-9.8315E-10
13.753	0.0000	-9.7482E-10
13.552	1.0000	-9.9003E-10
13.552	0.50000	-9.9003E-10
13.552	0.10000	-9.9002E-10
13.552	0.050000	-9.9000E-10
13.552	0.010000	-9.8988E-10
13.552	0.0010000	-9.8867E-10
13.552	1.0000E-4	-9.8309E-10
13.552	0.0000	-9.7461E-10
13.350	1.0000	-9.9036E-10
13.350	0.50000	-9.9035E-10
13.350	0.10000	-9.9034E-10
13.350	0.050000	-9.9032E-10
13.350	0.010000	-9.9020E-10
13.350	0.0010000	-9.8895E-10
13.350	1.0000E-4	-9.8321E-10
13.350	0.0000	-9.7459E-10
13.148	1.0000	-9.9032E-10
13.148	0.50000	-9.9032E-10
13.148	0.10000	-9.9031E-10
13.148	0.050000	-9.9029E-10
13.148	0.010000	-9.9016E-10
13.148	0.0010000	-9.8886E-10
13.148	1.0000E-4	-9.8297E-10
13.148	0.0000	-9.7421E-10
12.947	1.0000	-9.9066E-10
12.947	0.50000	-9.9065E-10
12.947	0.10000	-9.9064E-10
12.947	0.050000	-9.9062E-10
12.947	0.010000	-9.9048E-10
12.947	0.0010000	-9.8914E-10
12.947	1.0000E-4	-9.8309E-10
12.947	0.0000	-9.7417E-10
12.745	1.0000	-9.9082E-10
12.745	0.50000	-9.9082E-10
12.745	0.10000	-9.9081E-10
12.745	0.050000	-9.9079E-10
12.745	0.010000	-9.9064E-10
12.745	0.0010000	-9.8925E-10

d (um)	k0Sub (cm/s)	Probe variable bnd4 (A)
12.745	1.0000E-4	-9.8303E-10
12.745	0.0000	-9.7395E-10
12.544	1.0000	-9.9102E-10
12.544	0.50000	-9.9101E-10
12.544	0.10000	-9.9100E-10
12.544	0.050000	-9.9098E-10
12.544	0.010000	-9.9083E-10
12.544	0.0010000	-9.8939E-10
12.544	1.0000E-4	-9.8298E-10
12.544	0.0000	-9.7374E-10
12.342	1.0000	-9.9122E-10
12.342	0.50000	-9.9122E-10
12.342	0.10000	-9.9120E-10
12.342	0.050000	-9.9118E-10
12.342	0.010000	-9.9103E-10
12.342	0.0010000	-9.8953E-10
12.342	1.0000E-4	-9.8294E-10
12.342	0.0000	-9.7353E-10
12.141	1.0000	-9.9151E-10
12.141	0.50000	-9.9150E-10
12.141	0.10000	-9.9149E-10
12.141	0.050000	-9.9147E-10
12.141	0.010000	-9.9131E-10
12.141	0.0010000	-9.8975E-10
12.141	1.0000E-4	-9.8296E-10
12.141	0.0000	-9.7338E-10
11.939	1.0000	-9.9158E-10
11.939	0.50000	-9.9158E-10
11.939	0.10000	-9.9156E-10
11.939	0.050000	-9.9154E-10
11.939	0.010000	-9.9137E-10
11.939	0.0010000	-9.8976E-10
11.939	1.0000E-4	-9.8277E-10
11.939	0.0000	-9.7301E-10
11.738	1.0000	-9.9181E-10
11.738	0.50000	-9.9181E-10
11.738	0.10000	-9.9179E-10
11.738	0.050000	-9.9177E-10
11.738	0.010000	-9.9160E-10
11.738	0.0010000	-9.8992E-10

d (um)	k0Sub (cm/s)	Probe variable bnd4 (A)
11.738	1.0000E-4	-9.8271E-10
11.738	0.0000	-9.7277E-10
11.536	1.0000	-9.9211E-10
11.536	0.50000	-9.9211E-10
11.536	0.10000	-9.9209E-10
11.536	0.050000	-9.9207E-10
11.536	0.010000	-9.9188E-10
11.536	0.0010000	-9.9014E-10
11.536	1.0000E-4	-9.8271E-10
11.536	0.0000	-9.7258E-10
11.335	1.0000	-9.9225E-10
11.335	0.50000	-9.9225E-10
11.335	0.10000	-9.9223E-10
11.335	0.050000	-9.9221E-10
11.335	0.010000	-9.9202E-10
11.335	0.0010000	-9.9020E-10
11.335	1.0000E-4	-9.8254E-10
11.335	0.0000	-9.7221E-10
11.133	1.0000	-9.9261E-10
11.133	0.50000	-9.9261E-10
11.133	0.10000	-9.9259E-10
11.133	0.050000	-9.9256E-10
11.133	0.010000	-9.9236E-10
11.133	0.0010000	-9.9047E-10
11.133	1.0000E-4	-9.8256E-10
11.133	0.0000	-9.7202E-10
10.932	1.0000	-9.9290E-10
10.932	0.50000	-9.9289E-10
10.932	0.10000	-9.9287E-10
10.932	0.050000	-9.9284E-10
10.932	0.010000	-9.9264E-10
10.932	0.0010000	-9.9067E-10
10.932	1.0000E-4	-9.8250E-10
10.932	0.0000	-9.7175E-10
10.730	1.0000	-9.9309E-10
10.730	0.50000	-9.9308E-10
10.730	0.10000	-9.9306E-10
10.730	0.050000	-9.9303E-10
10.730	0.010000	-9.9282E-10
10.730	0.0010000	-9.9076E-10

d (um)	k0Sub (cm/s)	Probe variable bnd4 (A)
10.730	1.0000E-4	-9.8232E-10
10.730	0.0000	-9.7135E-10
10.529	1.0000	-9.9338E-10
10.529	0.50000	-9.9338E-10
10.529	0.10000	-9.9336E-10
10.529	0.050000	-9.9333E-10
10.529	0.010000	-9.9310E-10
10.529	0.0010000	-9.9096E-10
10.529	1.0000E-4	-9.8223E-10
10.529	0.0000	-9.7104E-10
10.327	1.0000	-9.9359E-10
10.327	0.50000	-9.9359E-10
10.327	0.10000	-9.9357E-10
10.327	0.050000	-9.9354E-10
10.327	0.010000	-9.9330E-10
10.327	0.0010000	-9.9106E-10
10.327	1.0000E-4	-9.8203E-10
10.327	0.0000	-9.7060E-10
10.126	1.0000	-9.9402E-10
10.126	0.50000	-9.9402E-10
10.126	0.10000	-9.9399E-10
10.126	0.050000	-9.9396E-10
10.126	0.010000	-9.9371E-10
10.126	0.0010000	-9.9137E-10
10.126	1.0000E-4	-9.8203E-10
10.126	0.0000	-9.7035E-10
9.9242	1.0000	-9.9442E-10
9.9242	0.50000	-9.9441E-10
9.9242	0.10000	-9.9439E-10
9.9242	0.050000	-9.9435E-10
9.9242	0.010000	-9.9409E-10
9.9242	0.0010000	-9.9164E-10
9.9242	1.0000E-4	-9.8197E-10
9.9242	0.0000	-9.7003E-10
9.7227	1.0000	-9.9477E-10
9.7227	0.50000	-9.9477E-10
9.7227	0.10000	-9.9474E-10
9.7227	0.050000	-9.9470E-10
9.7227	0.010000	-9.9443E-10
9.7227	0.0010000	-9.9186E-10

d (um)	k0Sub (cm/s)	Probe variable bnd4 (A)
9.7227	1.0000E-4	-9.8183E-10
9.7227	0.0000	-9.6963E-10
9.5212	1.0000	-9.9500E-10
9.5212	0.50000	-9.9500E-10
9.5212	0.10000	-9.9497E-10
9.5212	0.050000	-9.9493E-10
9.5212	0.010000	-9.9464E-10
9.5212	0.0010000	-9.9195E-10
9.5212	1.0000E-4	-9.8155E-10
9.5212	0.0000	-9.6907E-10
9.3197	1.0000	-9.9543E-10
9.3197	0.50000	-9.9543E-10
9.3197	0.10000	-9.9540E-10
9.3197	0.050000	-9.9536E-10
9.3197	0.010000	-9.9505E-10
9.3197	0.0010000	-9.9223E-10
9.3197	1.0000E-4	-9.8144E-10
9.3197	0.0000	-9.6866E-10
9.1182	1.0000	-9.9415E-10
9.1182	0.50000	-9.9415E-10
9.1182	0.10000	-9.9411E-10
9.1182	0.050000	-9.9407E-10
9.1182	0.010000	-9.9375E-10
9.1182	0.0010000	-9.9079E-10
9.1182	1.0000E-4	-9.7960E-10
9.1182	0.0000	-9.6653E-10
8.9167	1.0000	-9.9630E-10
8.9167	0.50000	-9.9629E-10
8.9167	0.10000	-9.9626E-10
8.9167	0.050000	-9.9622E-10
8.9167	0.010000	-9.9588E-10
8.9167	0.0010000	-9.9276E-10
8.9167	1.0000E-4	-9.8112E-10
8.9167	0.0000	-9.6772E-10
8.7152	1.0000	-9.9678E-10
8.7152	0.50000	-9.9677E-10
8.7152	0.10000	-9.9674E-10
8.7152	0.050000	-9.9669E-10
8.7152	0.010000	-9.9633E-10
8.7152	0.0010000	-9.9305E-10

d (um)	k0Sub (cm/s)	Probe variable bnd4 (A)
8.7152	1.0000E-4	-9.8094E-10
8.7152	0.0000	-9.6721E-10
8.5136	1.0000	-9.9732E-10
8.5136	0.50000	-9.9731E-10
8.5136	0.10000	-9.9727E-10
8.5136	0.050000	-9.9722E-10
8.5136	0.010000	-9.9685E-10
8.5136	0.0010000	-9.9339E-10
8.5136	1.0000E-4	-9.8078E-10
8.5136	0.0000	-9.6670E-10
8.3121	1.0000	-9.9779E-10
8.3121	0.50000	-9.9778E-10
8.3121	0.10000	-9.9774E-10
8.3121	0.050000	-9.9769E-10
8.3121	0.010000	-9.9729E-10
8.3121	0.0010000	-9.9365E-10
8.3121	1.0000E-4	-9.8051E-10
8.3121	0.0000	-9.6607E-10
8.1106	1.0000	-9.9826E-10
8.1106	0.50000	-9.9826E-10
8.1106	0.10000	-9.9821E-10
8.1106	0.050000	-9.9816E-10
8.1106	0.010000	-9.9773E-10
8.1106	0.0010000	-9.9389E-10
8.1106	1.0000E-4	-9.8020E-10
8.1106	0.0000	-9.6537E-10
7.9091	1.0000	-9.9899E-10
7.9091	0.50000	-9.9898E-10
7.9091	0.10000	-9.9893E-10
7.9091	0.050000	-9.9888E-10
7.9091	0.010000	-9.9842E-10
7.9091	0.0010000	-9.9436E-10
7.9091	1.0000E-4	-9.8006E-10
7.9091	0.0000	-9.6483E-10
7.7076	1.0000	-9.9951E-10
7.7076	0.50000	-9.9951E-10
7.7076	0.10000	-9.9946E-10
7.7076	0.050000	-9.9940E-10
7.7076	0.010000	-9.9892E-10
7.7076	0.0010000	-9.9461E-10

d (um)	k0Sub (cm/s)	Probe variable bnd4 (A)
7.7076	1.0000E-4	-9.7968E-10
7.7076	0.0000	-9.6402E-10
7.5061	1.0000	-1.0002E-9
7.5061	0.50000	-1.0002E-9
7.5061	0.10000	-1.0001E-9
7.5061	0.050000	-1.0001E-9
7.5061	0.010000	-9.9957E-10
7.5061	0.0010000	-9.9500E-10
7.5061	1.0000E-4	-9.7939E-10
7.5061	0.0000	-9.6329E-10
7.3045	1.0000	-1.0009E-9
7.3045	0.50000	-1.0008E-9
7.3045	0.10000	-1.0008E-9
7.3045	0.050000	-1.0007E-9
7.3045	0.010000	-1.0002E-9
7.3045	0.0010000	-9.9533E-10
7.3045	1.0000E-4	-9.7899E-10
7.3045	0.0000	-9.6242E-10
7.1030	1.0000	-1.0017E-9
7.1030	0.50000	-1.0017E-9
7.1030	0.10000	-1.0016E-9
7.1030	0.050000	-1.0016E-9
7.1030	0.010000	-1.0010E-9
7.1030	0.0010000	-9.9583E-10
7.1030	1.0000E-4	-9.7870E-10
7.1030	0.0000	-9.6162E-10
6.9015	1.0000	-1.0026E-9
6.9015	0.50000	-1.0026E-9
6.9015	0.10000	-1.0025E-9
6.9015	0.050000	-1.0024E-9
6.9015	0.010000	-1.0018E-9
6.9015	0.0010000	-9.9631E-10
6.9015	1.0000E-4	-9.7833E-10
6.9015	0.0000	-9.6071E-10
6.7000	1.0000	-1.0033E-9
6.7000	0.50000	-1.0033E-9
6.7000	0.10000	-1.0032E-9
6.7000	0.050000	-1.0032E-9
6.7000	0.010000	-1.0025E-9
6.7000	0.0010000	-9.9665E-10

d (um)	k0Sub (cm/s)	Probe variable bnd4 (A)
6.7000	1.0000E-4	-9.7775E-10
6.7000	0.0000	-9.5957E-10
6.4985	1.0000	-1.0042E-9
6.4985	0.50000	-1.0042E-9
6.4985	0.10000	-1.0041E-9
6.4985	0.050000	-1.0040E-9
6.4985	0.010000	-1.0033E-9
6.4985	0.0010000	-9.9705E-10
6.4985	1.0000E-4	-9.7717E-10
6.4985	0.0000	-9.5839E-10
6.2970	1.0000	-1.0052E-9
6.2970	0.50000	-1.0052E-9
6.2970	0.10000	-1.0051E-9
6.2970	0.050000	-1.0050E-9
6.2970	0.010000	-1.0042E-9
6.2970	0.0010000	-9.9754E-10
6.2970	1.0000E-4	-9.7661E-10
6.2970	0.0000	-9.5719E-10
6.0955	1.0000	-1.0061E-9
6.0955	0.50000	-1.0061E-9
6.0955	0.10000	-1.0060E-9
6.0955	0.050000	-1.0059E-9
6.0955	0.010000	-1.0051E-9
6.0955	0.0010000	-9.9793E-10
6.0955	1.0000E-4	-9.7587E-10
6.0955	0.0000	-9.5577E-10
5.8939	1.0000	-1.0073E-9
5.8939	0.50000	-1.0073E-9
5.8939	0.10000	-1.0072E-9
5.8939	0.050000	-1.0071E-9
5.8939	0.010000	-1.0062E-9
5.8939	0.0010000	-9.9856E-10
5.8939	1.0000E-4	-9.7523E-10
5.8939	0.0000	-9.5439E-10
5.6924	1.0000	-1.0086E-9
5.6924	0.50000	-1.0086E-9
5.6924	0.10000	-1.0085E-9
5.6924	0.050000	-1.0084E-9
5.6924	0.010000	-1.0074E-9
5.6924	0.0010000	-9.9917E-10

d (um)	k0Sub (cm/s)	Probe variable bnd4 (A)
5.6924	1.0000E-4	-9.7449E-10
5.6924	0.0000	-9.5286E-10
5.4909	1.0000	-1.0101E-9
5.4909	0.50000	-1.0100E-9
5.4909	0.10000	-1.0099E-9
5.4909	0.050000	-1.0098E-9
5.4909	0.010000	-1.0087E-9
5.4909	0.0010000	-9.9985E-10
5.4909	1.0000E-4	-9.7368E-10
5.4909	0.0000	-9.5120E-10
5.2894	1.0000	-1.0114E-9
5.2894	0.50000	-1.0114E-9
5.2894	0.10000	-1.0113E-9
5.2894	0.050000	-1.0111E-9
5.2894	0.010000	-1.0100E-9
5.2894	0.0010000	-1.0003E-9
5.2894	1.0000E-4	-9.7257E-10
5.2894	0.0000	-9.4917E-10
5.0879	1.0000	-1.0131E-9
5.0879	0.50000	-1.0131E-9
5.0879	0.10000	-1.0130E-9
5.0879	0.050000	-1.0128E-9
5.0879	0.010000	-1.0116E-9
5.0879	0.0010000	-1.0012E-9
5.0879	1.0000E-4	-9.7160E-10
5.0879	0.0000	-9.4721E-10
4.8864	1.0000	-1.0149E-9
4.8864	0.50000	-1.0148E-9
4.8864	0.10000	-1.0147E-9
4.8864	0.050000	-1.0145E-9
4.8864	0.010000	-1.0132E-9
4.8864	0.0010000	-1.0019E-9
4.8864	1.0000E-4	-9.7035E-10
4.8864	0.0000	-9.4487E-10
4.6848	1.0000	-1.0167E-9
4.6848	0.50000	-1.0167E-9
4.6848	0.10000	-1.0165E-9
4.6848	0.050000	-1.0163E-9
4.6848	0.010000	-1.0148E-9
4.6848	0.0010000	-1.0025E-9

d (um)	k0Sub (cm/s)	Probe variable bnd4 (A)
4.6848	1.0000E-4	-9.6888E-10
4.6848	0.0000	-9.4222E-10
4.4833	1.0000	-1.0188E-9
4.4833	0.50000	-1.0187E-9
4.4833	0.10000	-1.0186E-9
4.4833	0.050000	-1.0184E-9
4.4833	0.010000	-1.0167E-9
4.4833	0.0010000	-1.0033E-9
4.4833	1.0000E-4	-9.6726E-10
4.4833	0.0000	-9.3931E-10
4.2818	1.0000	-1.0211E-9
4.2818	0.50000	-1.0211E-9
4.2818	0.10000	-1.0209E-9
4.2818	0.050000	-1.0207E-9
4.2818	0.010000	-1.0188E-9
4.2818	0.0010000	-1.0042E-9
4.2818	1.0000E-4	-9.6548E-10
4.2818	0.0000	-9.3610E-10
4.0803	1.0000	-1.0238E-9
4.0803	0.50000	-1.0237E-9
4.0803	0.10000	-1.0235E-9
4.0803	0.050000	-1.0233E-9
4.0803	0.010000	-1.0212E-9
4.0803	0.0010000	-1.0051E-9
4.0803	1.0000E-4	-9.6348E-10
4.0803	0.0000	-9.3253E-10
3.8788	1.0000	-1.0267E-9
3.8788	0.50000	-1.0267E-9
3.8788	0.10000	-1.0264E-9
3.8788	0.050000	-1.0261E-9
3.8788	0.010000	-1.0239E-9
3.8788	0.0010000	-1.0061E-9
3.8788	1.0000E-4	-9.6117E-10
3.8788	0.0000	-9.2849E-10
3.6773	1.0000	-1.0301E-9
3.6773	0.50000	-1.0300E-9
3.6773	0.10000	-1.0298E-9
3.6773	0.050000	-1.0294E-9
3.6773	0.010000	-1.0269E-9
3.6773	0.0010000	-1.0072E-9

d (um)	k0Sub (cm/s)	Probe variable bnd4 (A)
3.6773	1.0000E-4	-9.5857E-10
3.6773	0.0000	-9.2393E-10
3.4758	1.0000	-1.0338E-9
3.4758	0.50000	-1.0338E-9
3.4758	0.10000	-1.0335E-9
3.4758	0.050000	-1.0331E-9
3.4758	0.010000	-1.0302E-9
3.4758	0.0010000	-1.0084E-9
3.4758	1.0000E-4	-9.5555E-10
3.4758	0.0000	-9.1874E-10
3.2742	1.0000	-1.0378E-9
3.2742	0.50000	-1.0378E-9
3.2742	0.10000	-1.0374E-9
3.2742	0.050000	-1.0370E-9
3.2742	0.010000	-1.0337E-9
3.2742	0.0010000	-1.0094E-9
3.2742	1.0000E-4	-9.5180E-10
3.2742	0.0000	-9.1255E-10
3.0727	1.0000	-1.0425E-9
3.0727	0.50000	-1.0425E-9
3.0727	0.10000	-1.0421E-9
3.0727	0.050000	-1.0416E-9
3.0727	0.010000	-1.0378E-9
3.0727	0.0010000	-1.0105E-9
3.0727	1.0000E-4	-9.4748E-10
3.0727	0.0000	-9.0545E-10
2.8712	1.0000	-1.0481E-9
2.8712	0.50000	-1.0481E-9
2.8712	0.10000	-1.0476E-9
2.8712	0.050000	-1.0470E-9
2.8712	0.010000	-1.0427E-9
2.8712	0.0010000	-1.0118E-9
2.8712	1.0000E-4	-9.4261E-10
2.8712	0.0000	-8.9741E-10
2.6697	1.0000	-1.0545E-9
2.6697	0.50000	-1.0545E-9
2.6697	0.10000	-1.0539E-9
2.6697	0.050000	-1.0532E-9
2.6697	0.010000	-1.0482E-9
2.6697	0.0010000	-1.0130E-9

d (um)	k0Sub (cm/s)	Probe variable bnd4 (A)
2.6697	1.0000E-4	-9.3668E-10
2.6697	0.0000	-8.8782E-10
2.4682	1.0000	-1.0621E-9
2.4682	0.50000	-1.0620E-9
2.4682	0.10000	-1.0613E-9
2.4682	0.050000	-1.0605E-9
2.4682	0.010000	-1.0546E-9
2.4682	0.0010000	-1.0143E-9
2.4682	1.0000E-4	-9.2957E-10
2.4682	0.0000	-8.7648E-10
2.2667	1.0000	-1.0710E-9
2.2667	0.50000	-1.0709E-9
2.2667	0.10000	-1.0701E-9
2.2667	0.050000	-1.0692E-9
2.2667	0.010000	-1.0621E-9
2.2667	0.0010000	-1.0154E-9
2.2667	1.0000E-4	-9.2093E-10
2.2667	0.0000	-8.6290E-10
2.0652	1.0000	-1.0820E-9
2.0652	0.50000	-1.0819E-9
2.0652	0.10000	-1.0810E-9
2.0652	0.050000	-1.0798E-9
2.0652	0.010000	-1.0712E-9
2.0652	0.0010000	-1.0165E-9
2.0652	1.0000E-4	-9.1048E-10
2.0652	0.0000	-8.4660E-10
1.8636	1.0000	-1.0956E-9
1.8636	0.50000	-1.0954E-9
1.8636	0.10000	-1.0942E-9
1.8636	0.050000	-1.0928E-9
1.8636	0.010000	-1.0822E-9
1.8636	0.0010000	-1.0172E-9
1.8636	1.0000E-4	-8.9748E-10
1.8636	0.0000	-8.2666E-10
1.6621	1.0000	-1.1125E-9
1.6621	0.50000	-1.1123E-9
1.6621	0.10000	-1.1107E-9
1.6621	0.050000	-1.1089E-9
1.6621	0.010000	-1.0954E-9
1.6621	0.0010000	-1.0172E-9

d (um)	k0Sub (cm/s)	Probe variable bnd4 (A)
1.6621	1.0000E-4	-8.8095E-10
1.6621	0.0000	-8.0181E-10
1.4606	1.0000	-1.1346E-9
1.4606	0.50000	-1.1344E-9
1.4606	0.10000	-1.1323E-9
1.4606	0.050000	-1.1298E-9
1.4606	0.010000	-1.1122E-9
1.4606	0.0010000	-1.0161E-9
1.4606	1.0000E-4	-8.5995E-10
1.4606	0.0000	-7.7072E-10
1.2591	1.0000	-1.1648E-9
1.2591	0.50000	-1.1645E-9
1.2591	0.10000	-1.1616E-9
1.2591	0.050000	-1.1581E-9
1.2591	0.010000	-1.1341E-9
1.2591	0.0010000	-1.0133E-9
1.2591	1.0000E-4	-8.3278E-10
1.2591	0.0000	-7.3126E-10
1.0576	1.0000	-1.2078E-9
1.0576	0.50000	-1.2073E-9
1.0576	0.10000	-1.2030E-9
1.0576	0.050000	-1.1979E-9
1.0576	0.010000	-1.1635E-9
1.0576	0.0010000	-1.0069E-9
1.0576	1.0000E-4	-7.9663E-10
1.0576	0.0000	-6.8013E-10
0.85606	1.0000	-1.2740E-9
0.85606	0.50000	-1.2731E-9
0.85606	0.10000	-1.2662E-9
0.85606	0.050000	-1.2579E-9
0.85606	0.010000	-1.2049E-9
0.85606	0.0010000	-9.9429E-10
0.85606	1.0000E-4	-7.4769E-10
0.85606	0.0000	-6.1307E-10
0.65455	1.0000	-1.3860E-9
0.65455	0.50000	-1.3843E-9
0.65455	0.10000	-1.3714E-9
0.65455	0.050000	-1.3564E-9
0.65455	0.010000	-1.2661E-9
0.65455	0.0010000	-9.6898E-10

d (um)	k0Sub (cm/s)	Probe variable bnd4 (A)
0.65455	1.0000E-4	-6.7943E-10
0.65455	0.0000	-5.2386E-10
0.45303	1.0000	-1.6094E-9
0.45303	0.50000	-1.6054E-9
0.45303	0.10000	-1.5754E-9
0.45303	0.050000	-1.5418E-9
0.45303	0.010000	-1.3627E-9
0.45303	0.0010000	-9.1763E-10
0.45303	1.0000E-4	-5.8163E-10
0.45303	0.0000	-4.0483E-10
0.25152	1.0000	-2.2242E-9
0.25152	0.50000	-2.2085E-9
0.25152	0.10000	-2.0974E-9
0.25152	0.050000	-1.9858E-9
0.25152	0.010000	-1.5275E-9
0.25152	0.0010000	-8.1157E-10
0.25152	1.0000E-4	-4.3812E-10
0.25152	0.0000	-2.4915E-10

4.3.5 Table 6

Global Evaluation 3 (bnd4)

d (um)	k0Sub (cm/s)	Probe variable bnd4 (A)
20.000	0.0050000	-9.8670E-10
20.000	0.0020000	-9.8653E-10
19.798	0.0050000	-9.8693E-10
19.798	0.0020000	-9.8676E-10
19.597	0.0050000	-9.8673E-10
19.597	0.0020000	-9.8655E-10
19.395	0.0050000	-9.8691E-10
19.395	0.0020000	-9.8672E-10
19.194	0.0050000	-9.8698E-10
19.194	0.0020000	-9.8679E-10
18.992	0.0050000	-9.8712E-10
18.992	0.0020000	-9.8692E-10
18.791	0.0050000	-9.8718E-10
18.791	0.0020000	-9.8698E-10
18.589	0.0050000	-9.8731E-10
18.589	0.0020000	-9.8711E-10
18.388	0.0050000	-9.8725E-10

d (um)	k0Sub (cm/s)	Probe variable bnd4 (A)
18.388	0.0020000	-9.8704E-10
18.186	0.0050000	-9.8735E-10
18.186	0.0020000	-9.8714E-10
17.985	0.0050000	-9.8740E-10
17.985	0.0020000	-9.8718E-10
17.783	0.0050000	-9.8744E-10
17.783	0.0020000	-9.8721E-10
17.582	0.0050000	-9.8754E-10
17.582	0.0020000	-9.8730E-10
17.380	0.0050000	-9.8770E-10
17.380	0.0020000	-9.8746E-10
17.179	0.0050000	-9.8773E-10
17.179	0.0020000	-9.8749E-10
16.977	0.0050000	-9.8788E-10
16.977	0.0020000	-9.8763E-10
16.776	0.0050000	-9.8802E-10
16.776	0.0020000	-9.8776E-10
16.574	0.0050000	-9.8811E-10
16.574	0.0020000	-9.8785E-10
16.373	0.0050000	-9.8817E-10
16.373	0.0020000	-9.8790E-10
16.171	0.0050000	-9.8813E-10
16.171	0.0020000	-9.8785E-10
15.970	0.0050000	-9.8840E-10
15.970	0.0020000	-9.8811E-10
15.768	0.0050000	-9.8841E-10
15.768	0.0020000	-9.8811E-10
15.567	0.0050000	-9.8853E-10
15.567	0.0020000	-9.8822E-10
15.365	0.0050000	-9.8873E-10
15.365	0.0020000	-9.8841E-10
15.164	0.0050000	-9.8868E-10
15.164	0.0020000	-9.8835E-10
14.962	0.0050000	-9.8889E-10
14.962	0.0020000	-9.8856E-10
14.761	0.0050000	-9.8900E-10
14.761	0.0020000	-9.8866E-10
14.559	0.0050000	-9.8912E-10
14.559	0.0020000	-9.8877E-10
14.358	0.0050000	-9.8921E-10

d (um)	k0Sub (cm/s)	Probe variable bnd4 (A)
14.358	0.0020000	-9.8884E-10
14.156	0.0050000	-9.8942E-10
14.156	0.0020000	-9.8903E-10
13.955	0.0050000	-9.8959E-10
13.955	0.0020000	-9.8920E-10
13.753	0.0050000	-9.8961E-10
13.753	0.0020000	-9.8920E-10
13.552	0.0050000	-9.8973E-10
13.552	0.0020000	-9.8930E-10
13.350	0.0050000	-9.9004E-10
13.350	0.0020000	-9.8960E-10
13.148	0.0050000	-9.9000E-10
13.148	0.0020000	-9.8954E-10
12.947	0.0050000	-9.9032E-10
12.947	0.0020000	-9.8984E-10
12.745	0.0050000	-9.9047E-10
12.745	0.0020000	-9.8998E-10
12.544	0.0050000	-9.9065E-10
12.544	0.0020000	-9.9014E-10
12.342	0.0050000	-9.9084E-10
12.342	0.0020000	-9.9031E-10
12.141	0.0050000	-9.9111E-10
12.141	0.0020000	-9.9056E-10
11.939	0.0050000	-9.9117E-10
11.939	0.0020000	-9.9060E-10
11.738	0.0050000	-9.9139E-10
11.738	0.0020000	-9.9079E-10
11.536	0.0050000	-9.9166E-10
11.536	0.0020000	-9.9105E-10
11.335	0.0050000	-9.9179E-10
11.335	0.0020000	-9.9114E-10
11.133	0.0050000	-9.9212E-10
11.133	0.0020000	-9.9145E-10
10.932	0.0050000	-9.9239E-10
10.932	0.0020000	-9.9169E-10
10.730	0.0050000	-9.9255E-10
10.730	0.0020000	-9.9183E-10
10.529	0.0050000	-9.9283E-10
10.529	0.0020000	-9.9207E-10
10.327	0.0050000	-9.9301E-10

d (um)	k0Sub (cm/s)	Probe variable bnd4 (A)
10.327	0.0020000	-9.9221E-10
10.126	0.0050000	-9.9341E-10
10.126	0.0020000	-9.9258E-10
9.9242	0.0050000	-9.9378E-10
9.9242	0.0020000	-9.9290E-10
9.7227	0.0050000	-9.9410E-10
9.7227	0.0020000	-9.9318E-10
9.5212	0.0050000	-9.9429E-10
9.5212	0.0020000	-9.9333E-10
9.3197	0.0050000	-9.9469E-10
9.3197	0.0020000	-9.9368E-10
9.1182	0.0050000	-9.9337E-10
9.1182	0.0020000	-9.9231E-10
8.9167	0.0050000	-9.9547E-10
8.9167	0.0020000	-9.9435E-10
8.7152	0.0050000	-9.9590E-10
8.7152	0.0020000	-9.9473E-10
8.5136	0.0050000	-9.9639E-10
8.5136	0.0020000	-9.9515E-10
8.3121	0.0050000	-9.9681E-10
8.3121	0.0020000	-9.9550E-10
8.1106	0.0050000	-9.9723E-10
8.1106	0.0020000	-9.9584E-10
7.9091	0.0050000	-9.9789E-10
7.9091	0.0020000	-9.9642E-10
7.7076	0.0050000	-9.9834E-10
7.7076	0.0020000	-9.9679E-10
7.5061	0.0050000	-9.9896E-10
7.5061	0.0020000	-9.9731E-10
7.3045	0.0050000	-9.9952E-10
7.3045	0.0020000	-9.9777E-10
7.1030	0.0050000	-1.0003E-9
7.1030	0.0020000	-9.9842E-10
6.9015	0.0050000	-1.0010E-9
6.9015	0.0020000	-9.9906E-10
6.7000	0.0050000	-1.0017E-9
6.7000	0.0020000	-9.9957E-10
6.4985	0.0050000	-1.0024E-9
6.4985	0.0020000	-1.0002E-9
6.2970	0.0050000	-1.0033E-9

d (um)	k0Sub (cm/s)	Probe variable bnd4 (A)
6.2970	0.0020000	-1.0009E-9
6.0955	0.0050000	-1.0041E-9
6.0955	0.0020000	-1.0015E-9
5.8939	0.0050000	-1.0052E-9
5.8939	0.0020000	-1.0024E-9
5.6924	0.0050000	-1.0063E-9
5.6924	0.0020000	-1.0033E-9
5.4909	0.0050000	-1.0075E-9
5.4909	0.0020000	-1.0042E-9
5.2894	0.0050000	-1.0086E-9
5.2894	0.0020000	-1.0051E-9
5.0879	0.0050000	-1.0101E-9
5.0879	0.0020000	-1.0063E-9
4.8864	0.0050000	-1.0116E-9
4.8864	0.0020000	-1.0074E-9
4.6848	0.0050000	-1.0131E-9
4.6848	0.0020000	-1.0085E-9
4.4833	0.0050000	-1.0148E-9
4.4833	0.0020000	-1.0098E-9
4.2818	0.0050000	-1.0167E-9
4.2818	0.0020000	-1.0113E-9
4.0803	0.0050000	-1.0189E-9
4.0803	0.0020000	-1.0129E-9
3.8788	0.0050000	-1.0212E-9
3.8788	0.0020000	-1.0146E-9
3.6773	0.0050000	-1.0240E-9
3.6773	0.0020000	-1.0166E-9
3.4758	0.0050000	-1.0270E-9
3.4758	0.0020000	-1.0187E-9
3.2742	0.0050000	-1.0300E-9
3.2742	0.0020000	-1.0208E-9
3.0727	0.0050000	-1.0336E-9
3.0727	0.0020000	-1.0233E-9
2.8712	0.0050000	-1.0379E-9
2.8712	0.0020000	-1.0262E-9
2.6697	0.0050000	-1.0427E-9
2.6697	0.0020000	-1.0292E-9
2.4682	0.0050000	-1.0482E-9
2.4682	0.0020000	-1.0327E-9
2.2667	0.0050000	-1.0545E-9

d (um)	k0Sub (cm/s)	Probe variable bnd4 (A)
2.2667	0.0020000	-1.0364E-9
2.0652	0.0050000	-1.0620E-9
2.0652	0.0020000	-1.0408E-9
1.8636	0.0050000	-1.0710E-9
1.8636	0.0020000	-1.0456E-9
1.6621	0.0050000	-1.0815E-9
1.6621	0.0020000	-1.0506E-9
1.4606	0.0050000	-1.0944E-9
1.4606	0.0020000	-1.0562E-9
1.2591	0.0050000	-1.1106E-9
1.2591	0.0020000	-1.0622E-9
1.0576	0.0050000	-1.1312E-9
1.0576	0.0020000	-1.0677E-9
0.85606	0.0050000	-1.1581E-9
0.85606	0.0020000	-1.0718E-9
0.65455	0.0050000	-1.1932E-9
0.65455	0.0020000	-1.0703E-9
0.45303	0.0050000	-1.2379E-9
0.45303	0.0020000	-1.0533E-9
0.25152	0.0050000	-1.2866E-9
0.25152	0.0020000	-9.9399E-10

4.3.6 Probe Table 7

Time (s)	Tip potential during pulse (V)
19.589	-0.65000
19.589	-0.65000
19.589	-0.65000
19.589	-0.65000
19.590	-0.65000
19.590	-0.65000
19.590	-0.65000
19.590	-0.65000
19.591	-0.65000
19.591	-0.65000
19.591	-0.65000
19.592	-0.65000
19.592	-0.65000
19.593	-0.65000
19.593	-0.65000
19.593	-0.65000

Time (s)	Tip potential during pulse (V)
19.594	-0.65000
19.594	-0.65000
19.594	-0.65000
19.594	-0.65000
19.595	-0.65000
19.595	-0.65000
19.595	-0.65000
19.595	-0.65000
19.596	-0.65000
19.596	-0.65000
19.596	-0.65000
19.596	-0.65000
19.597	-0.65000
19.597	-0.65000
19.597	-0.65000
19.598	-0.65000
19.598	-0.65000
19.598	-0.65000
19.598	-0.65000
19.599	-0.65000
19.599	-0.65000
19.599	-0.65000
19.600	-0.65000
19.600	-0.65000
19.600	-0.65000
19.600	-0.65000
19.601	-0.65000
19.601	-0.65000
19.601	-0.65000
19.601	-0.65000
19.602	-0.65000
19.602	-0.65000
19.602	-0.65000
19.602	-0.65000
19.603	-0.65000
19.603	-0.65000
19.603	-0.65000
19.604	-0.65000
19.604	-0.65000
19.604	-0.65000

Time (s)	Tip potential during pulse (V)
19.604	-0.65000
19.605	-0.65000
19.607	-0.65000
19.610	-0.65000
19.615	-0.65000
19.627	-0.65000
19.650	-0.65000
19.651	-0.65000
19.651	-0.65000
19.651	-0.65000
19.651	-0.65000
19.652	-0.65000
19.652	-0.65000
19.652	-0.65000
19.653	-0.65000
19.653	-0.65000
19.653	-0.65000
19.653	-0.65000
19.654	-0.65000
19.654	-0.65000
19.654	-0.65000
19.654	-0.65000
19.655	-0.65000
19.655	-0.65000
19.655	-0.65000
19.655	-0.65000
19.656	-0.65000
19.656	-0.65000
19.656	-0.65000
19.657	-0.65000
19.657	-0.65000
19.657	-0.65000
19.657	-0.65000
19.658	-0.65000
19.658	-0.65000
19.658	-0.65000
19.659	-0.65000
19.659	-0.65000
19.659	-0.65000
19.659	-0.65000

Time (s)	Tip potential during pulse (V)
19.660	-0.65000
19.660	-0.65000
19.660	-0.65000
19.660	-0.65000
19.661	-0.65000
19.661	-0.65000
19.661	-0.65000
19.662	-0.65000
19.662	-0.65000
19.662	-0.65000
19.662	-0.65000
19.663	-0.65000
19.663	-0.65000
19.663	-0.65000
19.664	-0.65000
19.664	-0.65000
19.664	-0.65000
19.664	-0.65000
19.665	-0.65000
19.665	-0.65000
19.665	-0.65000
19.665	-0.65000
19.666	-0.65000
19.666	-0.65000
19.666	-0.65000
19.666	-0.65000
19.667	-0.65000
19.667	-0.65000
19.667	-0.65000
19.668	-0.65000
19.668	-0.65000
19.668	-0.65000
19.668	-0.65000
19.669	-0.65000
19.669	-0.65000
19.669	-0.65000
19.670	-0.65000
19.670	-0.65000
19.670	-0.65000
19.670	-0.65000

Time (s)	Tip potential during pulse (V)
19.671	-0.65000
19.671	-0.65000
19.671	-0.65000
19.671	-0.65000
19.672	-0.65000
19.672	-0.65000
19.672	-0.65000
19.672	-0.65000
19.673	-0.65000
19.673	-0.65000
19.673	-0.65000
19.674	-0.65000
19.674	-0.65000
19.674	-0.65000
19.674	-0.65000
19.675	-0.65000
19.675	-0.65000
19.675	-0.65000
19.676	-0.65000
19.676	-0.65000
19.676	-0.65000
19.676	-0.65000
19.677	-0.65000
19.677	-0.65000
19.677	-0.65000
19.677	-0.65000
19.678	-0.65000
19.678	-0.65000
19.678	-0.65000
19.678	-0.65000
19.679	-0.65000
19.679	-0.65000
19.679	-0.65000
19.680	-0.65000
19.680	-0.65000
19.680	-0.65000
19.680	-0.65000
19.681	-0.65000
19.681	-0.65000
19.681	-0.65000

Time (s)	Tip potential during pulse (V)
19.682	-0.65000
19.682	-0.65000
19.682	-0.65000
19.682	-0.65000
19.683	-0.65000
19.683	-0.65000
19.683	-0.65000
19.683	-0.65000
19.684	-0.65000
19.684	-0.65000
19.684	-0.65000
19.684	-0.65000
19.685	-0.65000
19.685	-0.65000
19.685	-0.65000
19.686	-0.65000
19.686	-0.65000
19.686	-0.65000
19.686	-0.65000
19.687	-0.65000
19.687	-0.65000
19.687	-0.65000
19.688	-0.65000
19.688	-0.65000
19.688	-0.65000
19.688	-0.65000
19.689	-0.65000
19.689	-0.65000
19.689	-0.65000
19.689	-0.65000
19.690	-0.65000
19.690	-0.65000
19.690	-0.65000
19.690	-0.65000
19.691	-0.65000
19.691	-0.65000
19.691	-0.65000
19.692	-0.65000
19.692	-0.65000
19.692	-0.65000

Time (s)	Tip potential during pulse (V)
19.692	-0.65000
19.693	-0.65000
19.693	-0.65000
19.693	-0.65000
19.694	-0.65000
19.694	-0.65000
19.694	-0.65000
19.694	-0.65000
19.695	-0.65000
19.695	-0.65000
19.695	-0.65000
19.695	-0.65000
19.696	-0.65000
19.696	-0.65000
19.696	-0.65000
19.696	-0.65000
19.697	-0.65000
19.697	-0.65000
19.697	-0.65000
19.698	-0.65000
19.698	-0.65000
19.698	-0.65000
19.698	-0.65000
19.699	-0.65000
19.699	-0.65000
19.699	-0.65000
19.700	-0.65000
19.700	-0.65000
19.700	-0.65000
19.700	-0.65000
19.701	-0.65000
19.701	-0.65000
19.701	-0.65000
19.701	-0.65000
19.702	-0.65000
19.702	-0.65000
19.702	-0.65000
19.702	-0.65000
19.703	-0.65000
19.703	-0.65000

Time (s)	Tip potential during pulse (V)
19.703	-0.65000
19.704	-0.65000
19.704	-0.65000
19.704	-0.65000
19.704	-0.65000
19.705	-0.65000
19.705	-0.65000
19.705	-0.65000
19.706	-0.65000
19.706	-0.65000
19.706	-0.65000
19.706	-0.65000
19.707	-0.65000
19.707	-0.65000
19.707	-0.65000
19.707	-0.65000
19.708	-0.65000
19.708	-0.65000
19.708	-0.65000
19.708	-0.65000
19.709	-0.65000
19.709	-0.65000
19.709	-0.65000
19.710	-0.65000
19.710	-0.65000
19.710	-0.65000
19.710	-0.65000
19.711	-0.65000
19.711	-0.65000
19.711	-0.65000
19.712	-0.65000
19.712	-0.65000
19.712	-0.65000
19.712	-0.65000
19.713	-0.65000
19.713	-0.65000
19.713	-0.65000
19.713	-0.65000
19.714	-0.65000
19.714	-0.65000

Time (s)	Tip potential during pulse (V)
19.714	-0.65000
19.715	-0.65000
19.715	-0.65000
19.716	-0.65000
19.716	-0.65000
19.716	-0.65000
19.717	-0.65000
19.717	-0.65000
19.717	-0.65000
19.717	-0.65000
19.718	-0.65000
19.718	-0.65000
19.718	-0.65000
19.718	-0.65000
19.719	-0.65000
19.719	-0.65000
19.719	-0.65000
19.719	-0.65000
19.720	-0.65000
19.720	-0.65000
19.720	-0.65000
19.721	-0.65000
19.721	-0.65000
19.721	-0.65000
19.721	-0.65000
19.722	-0.65000
19.722	-0.65000
19.722	-0.65000
19.723	-0.65000
19.723	-0.65000
19.723	-0.65000
19.723	-0.65000
19.724	-0.65000
19.724	-0.65000
19.724	-0.65000
19.724	-0.65000
19.725	-0.65000
19.725	-0.65000
19.725	-0.65000
19.725	-0.65000

Time (s)	Tip potential during pulse (V)
19.726	-0.65000
19.726	-0.65000
19.726	-0.65000
19.727	-0.65000
19.727	-0.65000
19.727	-0.65000
19.727	-0.65000
19.728	-0.65000
19.728	-0.65000
19.728	-0.65000
19.729	-0.65000
19.729	-0.65000
19.729	-0.65000
19.729	-0.65000
19.730	-0.65000
19.730	-0.65000
19.730	-0.65000
19.730	-0.65000
19.731	-0.65000
19.731	-0.65000
19.731	-0.65000
19.731	-0.65000
19.732	-0.65000
19.732	-0.65000
19.732	-0.65000
19.733	-0.65000
19.733	-0.65000
19.733	-0.65000
19.733	-0.65000
19.734	-0.65000
19.734	-0.65000
19.734	-0.65000
19.735	-0.65000
19.735	-0.65000
19.735	-0.65000
19.735	-0.65000
19.736	-0.65000
19.736	-0.65000
19.736	-0.65000
19.736	-0.65000

Time (s)	Tip potential during pulse (V)
19.737	-0.65000
19.737	-0.65000
19.737	-0.65000
19.737	-0.65000
19.738	-0.65000
19.738	-0.65000
19.738	-0.65000
19.739	-0.65000
19.739	-0.65000
19.739	-0.65000
19.739	-0.65000
19.740	-0.65000
19.740	-0.65000
19.740	-0.65000
19.741	-0.65000
19.741	-0.65000
19.741	-0.65000
19.741	-0.65000
19.742	-0.65000
19.742	-0.65000
19.742	-0.65000
19.742	-0.65000
19.743	-0.65000
19.743	-0.65000
19.743	-0.65000
19.743	-0.65000
19.744	-0.65000
19.744	-0.65000
19.744	-0.65000
19.745	-0.65000
19.745	-0.65000
19.745	-0.65000
19.745	-0.65000
19.746	-0.65000
19.746	-0.65000
19.746	-0.65000
19.747	-0.65000
19.747	-0.65000
19.747	-0.65000
19.747	-0.65000

Time (s)	Tip potential during pulse (V)
19.748	-0.65000
19.748	-0.65000
19.748	-0.65000
19.748	-0.65000
19.749	-0.65000
19.749	-0.65000
19.749	-0.65000
19.749	-0.65000
19.750	-0.65000
19.750	-0.65000
19.750	-0.65000
19.751	-0.65000
19.751	-0.65000
19.751	-0.65000
19.751	-0.65000
19.752	-0.65000
19.752	-0.65000
19.752	-0.65000
19.753	-0.65000
19.753	-0.65000
19.753	-0.65000
19.753	-0.65000
19.754	-0.65000
19.754	-0.65000
19.754	-0.65000
19.754	-0.65000
19.755	-0.65000
19.755	-0.65000
19.755	-0.65000
19.755	-0.65000
19.756	-0.65000
19.756	-0.65000
19.756	-0.65000
19.757	-0.65000
19.757	-0.65000
19.757	-0.65000
19.757	-0.65000
19.758	-0.65000
19.758	-0.65000
19.758	-0.65000

Time (s)	Tip potential during pulse (V)
19.759	-0.65000
19.759	-0.65000
19.759	-0.65000
19.759	-0.65000
19.760	-0.65000
19.760	-0.65000
19.760	-0.65000
19.760	-0.65000
19.761	-0.65000
19.761	-0.65000
19.761	-0.65000
19.762	-0.65000
19.762	-0.65000
19.762	-0.65000
19.762	-0.65000
19.763	-0.65000
19.763	-0.65000
19.763	-0.65000
19.764	-0.65000
19.764	-0.65000
19.764	-0.65000
19.764	-0.65000
19.765	-0.65000
19.765	-0.65000
19.765	-0.65000
19.765	-0.65000
19.766	-0.65000
19.766	-0.65000
19.766	-0.65000
19.766	-0.65000
19.767	-0.65000
19.767	-0.65000
19.767	-0.65000
19.768	-0.65000
19.768	-0.65000
19.768	-0.65000
19.768	-0.65000
19.769	-0.65000
19.769	-0.65000
19.769	-0.65000

Time (s)	Tip potential during pulse (V)
19.770	-0.65000
19.770	-0.65000
19.770	-0.65000
19.770	-0.65000
19.771	-0.65000
19.771	-0.65000
19.771	-0.65000
19.771	-0.65000
19.772	-0.65000
19.772	-0.65000
19.772	-0.65000
19.772	-0.65000
19.773	-0.65000
19.773	-0.65000
19.773	-0.65000
19.774	-0.65000
19.774	-0.65000
19.774	-0.65000
19.774	-0.65000
19.775	-0.65000
19.775	-0.65000
19.775	-0.65000
19.776	-0.65000
19.776	-0.65000
19.776	-0.65000
19.776	-0.65000
19.777	-0.65000
19.777	-0.65000
19.777	-0.65000
19.777	-0.65000
19.778	-0.65000
19.778	-0.65000
19.778	-0.65000
19.778	-0.65000
19.779	-0.65000
19.779	-0.65000
19.779	-0.65000
19.780	-0.65000
19.780	-0.65000
19.780	-0.65000

Time (s)	Tip potential during pulse (V)
19.780	-0.65000
19.781	-0.65000
19.781	-0.65000
19.781	-0.65000
19.782	-0.65000
19.782	-0.65000
19.782	-0.65000
19.782	-0.65000
19.783	-0.65000
19.783	-0.65000
19.783	-0.65000
19.783	-0.65000
19.784	-0.65000
19.784	-0.65000
19.784	-0.65000
19.784	-0.65000
19.785	-0.65000
19.785	-0.65000
19.785	-0.65000
19.786	-0.65000
19.786	-0.65000
19.786	-0.65000
19.786	-0.65000
19.787	-0.65000
19.787	-0.65000
19.787	-0.65000
19.788	-0.65000
19.788	-0.65000
19.788	-0.65000
19.788	-0.65000
19.789	-0.65000
19.789	-0.65000
19.789	-0.65000
19.789	-0.65000
19.790	-0.65000
19.790	-0.65000
19.790	-0.65000
19.790	-0.65000
19.791	-0.65000
19.791	-0.65000

Time (s)	Tip potential during pulse (V)
19.791	-0.65000
19.792	-0.65000
19.792	-0.65000
19.792	-0.65000
19.792	-0.65000
19.793	-0.65000
19.793	-0.65000
19.793	-0.65000
19.794	-0.65000
19.794	-0.65000
19.794	-0.65000
19.794	-0.65000
19.795	-0.65000
19.795	-0.65000
19.795	-0.65000
19.795	-0.65000
19.796	-0.65000
19.796	-0.65000
19.796	-0.65000
19.796	-0.65000
19.797	-0.65000
19.797	-0.65000
19.797	-0.65000
19.798	-0.65000
19.798	-0.65000
19.798	-0.65000
19.798	-0.65000
19.799	-0.65000
19.799	-0.65000
19.799	-0.65000
19.800	-0.65000
19.800	-0.65000
19.800	-0.65000
19.800	-0.65000
19.801	-0.65000
19.801	-0.65000
19.801	-0.65000
19.801	-0.65000
19.802	-0.65000
19.802	-0.65000

Time (s)	Tip potential during pulse (V)
19.802	-0.65000
19.802	-0.65000
19.803	-0.65000
19.803	-0.65000
19.803	-0.65000
19.804	-0.65000
19.804	-0.65000
19.804	-0.65000
19.804	-0.65000
19.805	-0.65000
19.805	-0.65000
19.805	-0.65000
19.806	-0.65000
19.806	-0.65000
19.806	-0.65000
19.806	-0.65000
19.807	-0.65000
19.807	-0.65000
19.807	-0.65000
19.807	-0.65000
19.808	-0.65000
19.808	-0.65000
19.808	-0.65000
19.808	-0.65000
19.809	-0.65000
19.809	-0.65000
19.809	-0.65000
19.810	-0.65000
19.810	-0.65000
19.810	-0.65000
19.810	-0.65000
19.811	-0.65000
19.811	-0.65000
19.811	-0.65000
19.812	-0.65000
19.812	-0.65000
19.812	-0.65000
19.812	-0.65000
19.813	-0.65000
19.813	-0.65000

Time (s)	Tip potential during pulse (V)
19.813	-0.65000
19.813	-0.65000
19.814	-0.65000
19.814	-0.65000
19.814	-0.65000
19.815	-0.65000
19.815	-0.65000
19.815	-0.65000
19.815	-0.65000
19.816	-0.65000
19.816	-0.65000
19.816	-0.65000
19.817	-0.65000
19.817	-0.65000
19.817	-0.65000
19.817	-0.65000
19.818	-0.65000
19.818	-0.65000
19.818	-0.65000
19.818	-0.65000
19.819	-0.65000
19.819	-0.65000
19.819	-0.65000
19.819	-0.65000
19.820	-0.65000
19.820	-0.65000
19.820	-0.65000
19.821	-0.65000
19.821	-0.65000
19.821	-0.65000
19.821	-0.65000
19.822	-0.65000
19.822	-0.65000
19.822	-0.65000
19.823	-0.65000
19.823	-0.65000
19.823	-0.65000
19.823	-0.65000
19.824	-0.65000
19.824	-0.65000

Time (s)	Tip potential during pulse (V)
19.824	-0.65000
19.824	-0.65000
19.825	-0.65000
19.825	-0.65000
19.825	-0.65000
19.825	-0.65000
19.826	-0.65000
19.826	-0.65000
19.826	-0.65000
19.827	-0.65000
19.827	-0.65000
19.827	-0.65000
19.827	-0.65000
19.828	-0.65000
19.828	-0.65000
19.828	-0.65000
19.829	-0.65000
19.829	-0.65000
19.829	-0.65000
19.829	-0.65000
19.830	-0.65000
19.830	-0.65000
19.830	-0.65000
19.830	-0.65000
19.831	-0.65000
19.831	-0.65000
19.831	-0.65000
19.831	-0.65000
19.832	-0.65000
19.832	-0.65000
19.832	-0.65000
19.833	-0.65000
19.833	-0.65000
19.833	-0.65000
19.833	-0.65000
19.834	-0.65000
19.834	-0.65000
19.834	-0.65000
19.835	-0.65000
19.835	-0.65000

Time (s)	Tip potential during pulse (V)
19.835	-0.65000
19.835	-0.65000
19.836	-0.65000
19.836	-0.65000
19.836	-0.65000
19.836	-0.65000
19.836	-0.65000
19.837	-0.65000
19.837	-0.65000
19.837	-0.65000
19.838	-0.65000
19.838	-0.65000
19.838	-0.65000
19.838	-0.65000
19.838	-0.65000
19.839	-0.65000
19.839	-0.65000
19.839	-0.65000
19.839	-0.65000
19.840	-0.65000
19.840	-0.65000
19.840	-0.65000
19.841	-0.65000
19.841	-0.65000
19.841	-0.65000
19.841	-0.65000
19.842	-0.65000
19.842	-0.65000
19.842	-0.65000
19.842	-0.65000
19.842	-0.65000
19.842	-0.65000
19.843	-0.65000
19.843	-0.65000
19.843	-0.65000
19.843	-0.65000
19.844	-0.65000
19.844	-0.65000
19.844	-0.65000
19.844	-0.65000

Time (s)	Tip potential during pulse (V)
19.845	-0.65000
19.845	-0.65000
19.845	-0.65000
19.845	-0.65000
19.846	-0.65000
19.846	-0.65000
19.846	-0.65000
19.846	-0.65000
19.847	-0.65000
19.847	-0.65000
19.847	-0.65000
19.847	-0.65000
19.848	-0.65000
19.848	-0.65000
19.848	-0.65000
19.848	-0.65000
19.849	-0.65000
19.849	-0.65000
19.849	-0.65000
19.849	-0.65000
19.850	-0.65000
19.850	-0.65000
19.850	-0.65000
19.850	-0.65000
19.850	-0.65000
19.851	-0.65000
19.851	-0.65000
19.851	-0.65000
19.851	-0.65000
19.852	-0.65000
19.852	-0.65000
19.852	-0.65000
19.852	-0.65000
19.852	-0.65000
19.853	-0.65000
19.853	-0.65000
19.853	-0.65000
19.853	-0.65000
19.853	-0.65000
19.854	-0.65000

Time (s)	Tip potential during pulse (V)
19.854	-0.65000
19.854	-0.65000
19.854	-0.65000
19.854	-0.65000
19.854	-0.65000
19.855	-0.65000
19.855	-0.65000
19.855	-0.65000
19.855	-0.65000
19.855	-0.65000
19.856	-0.65000
19.856	-0.65000
19.856	-0.65000
19.856	-0.65000
19.856	-0.65000
19.857	-0.65000
19.857	-0.65000
19.857	-0.65000
19.857	-0.65000
19.857	-0.65000
19.857	-0.65000
19.858	-0.65000
19.858	-0.65000
19.858	-0.65000
19.858	-0.65000
19.858	-0.65000
19.859	-0.65000
19.859	-0.65000
19.859	-0.65000
19.859	-0.65000
19.860	-0.65000
19.860	-0.65000
19.860	-0.65000
19.860	-0.65000
19.861	-0.65000
19.861	-0.65000
19.861	-0.65000
19.861	-0.65000
19.862	-0.65000
19.862	-0.65000

Time (s)	Tip potential during pulse (V)
19.862	-0.65000
19.862	-0.65000
19.862	-0.65000
19.863	-0.65000
19.863	-0.65000
19.863	-0.65000
19.863	-0.65000
19.863	-0.65000
19.863	-0.65000
19.863	-0.65000
19.864	-0.65000
19.864	-0.65000
19.864	-0.65000
19.864	-0.65000
19.865	-0.65000
19.865	-0.65000
19.865	-0.65000
19.865	-0.65000
19.865	-0.65000
19.866	-0.65000
19.866	-0.65000
19.866	-0.65000
19.866	-0.65000
19.866	-0.65000
19.866	-0.65000
19.866	-0.65000
19.867	-0.65000
19.867	-0.65000
19.867	-0.65000
19.868	-0.65000
19.868	-0.65000
19.868	-0.65000
19.868	-0.65000
19.869	-0.65000
19.869	-0.65000
19.869	-0.65000
19.869	-0.65000
19.869	-0.65000
19.870	-0.65000
19.870	-0.65000

Time (s)	Tip potential during pulse (V)
19.870	-0.65000
19.870	-0.65000
19.871	-0.65000
19.871	-0.65000
19.871	-0.65000
19.871	-0.65000
19.871	-0.65000
19.872	-0.65000
19.872	-0.65000
19.872	-0.65000
19.873	-0.65000
19.873	-0.65000
19.873	-0.65000
19.873	-0.65000
19.874	-0.65000
19.874	-0.65000
19.874	-0.65000
19.874	-0.65000
19.874	-0.65000
19.875	-0.65000
19.875	-0.65000
19.875	-0.65000
19.875	-0.65000
19.875	-0.65000
19.876	-0.65000
19.876	-0.65000
19.876	-0.65000
19.876	-0.65000
19.876	-0.65000
19.877	-0.65000
19.877	-0.65000
19.877	-0.65000
19.877	-0.65000
19.878	-0.65000
19.878	-0.65000
19.878	-0.65000
19.878	-0.65000
19.878	-0.65000
19.878	-0.65000
19.878	-0.65000

Time (s)	Tip potential during pulse (V)
19.879	-0.65000
19.879	-0.65000
19.879	-0.65000
19.879	-0.65000
19.879	-0.65000
19.879	-0.65000
19.880	-0.65000
19.880	-0.65000
19.880	-0.65000
19.880	-0.65000
19.880	-0.65000
19.881	-0.65000
19.881	-0.65000
19.881	-0.65000
19.881	-0.65000
19.881	-0.65000
19.882	-0.65000
19.882	-0.65000
19.882	-0.65000
19.882	-0.65000
19.882	-0.65000
19.882	-0.65000
19.883	-0.65000
19.883	-0.65000
19.883	-0.65000
19.883	-0.65000
19.883	-0.65000
19.883	-0.65000
19.883	-0.65000
19.884	-0.65000
19.884	-0.65000
19.884	-0.65000
19.884	-0.65000
19.884	-0.65000
19.884	-0.65000
19.884	-0.65000
19.885	-0.65000
19.885	-0.65000
19.885	-0.65000
19.885	-0.65000

Time (s)	Tip potential during pulse (V)
19.885	-0.65000
19.885	-0.65000
19.885	-0.65000
19.886	-0.65000
19.886	-0.65000
19.886	-0.65000
19.886	-0.65000
19.886	-0.65000
19.886	-0.65000
19.886	-0.65000
19.886	-0.65000
19.887	-0.65000
19.887	-0.65000
19.887	-0.65000
19.887	-0.65000
19.887	-0.65000
19.887	-0.65000
19.887	-0.65000
19.888	-0.65000
19.888	-0.65000
19.888	-0.65000
19.888	-0.65000
19.888	-0.65000
19.889	-0.65000
19.889	-0.65000
19.889	-0.65000
19.889	-0.65000
19.890	-0.65000
19.890	-0.65000
19.890	-0.65000
19.890	-0.65000
19.890	-0.65000
19.891	-0.65000
19.891	-0.65000
19.891	-0.65000
19.891	-0.65000
19.891	-0.65000
19.891	-0.65000
19.891	-0.65000
19.892	-0.65000

Time (s)	Tip potential during pulse (V)
19.892	-0.65000
19.892	-0.65000
19.892	-0.65000
19.892	-0.65000
19.892	-0.65000
19.893	-0.65000
19.893	-0.65000
19.893	-0.65000
19.894	-0.65000
19.894	-0.65000
19.894	-0.65000
19.894	-0.65000
19.894	-0.65000
19.894	-0.65000
19.894	-0.65000
19.895	-0.65000
19.895	-0.65000
19.895	-0.65000
19.895	-0.65000
19.896	-0.65000
19.896	-0.65000
19.896	-0.65000
19.896	-0.65000
19.896	-0.65000
19.897	-0.65000
19.897	-0.65000
19.897	-0.65000
19.897	-0.65000
19.897	-0.65000
19.897	-0.65000
19.897	-0.65000
19.898	-0.65000
19.898	-0.65000
19.898	-0.65000
19.898	-0.65000
19.898	-0.65000
19.898	-0.65000
19.899	-0.65000
19.899	-0.65000
19.899	-0.65000

Time (s)	Tip potential during pulse (V)
19.899	-0.65000
19.899	-0.65000
19.900	-0.65000
19.900	-0.65000
19.900	-0.65000
19.900	-0.65000
19.901	-0.65000
19.901	-0.65000
19.901	-0.65000
19.901	-0.65000
19.901	-0.65000
19.902	-0.65000
19.902	-0.65000
19.902	-0.65000
19.902	-0.65000
19.902	-0.65000
19.902	-0.65000
19.902	-0.65000
19.903	-0.65000
19.903	-0.65000
19.903	-0.65000
19.903	-0.65000
19.904	-0.65000
19.904	-0.65000
19.904	-0.65000
19.904	-0.65000
19.904	-0.65000
19.904	-0.65000
19.904	-0.65000
19.905	-0.65000
19.905	-0.65000
19.905	-0.65000
19.905	-0.65000
19.905	-0.65000
19.905	-0.65000
19.905	-0.65000
19.906	-0.65000
19.906	-0.65000
19.906	-0.65000
19.906	-0.65000

Time (s)	Tip potential during pulse (V)
19.906	-0.65000
19.907	-0.65000
19.907	-0.65000
19.907	-0.65000
19.907	-0.65000
19.907	-0.65000
19.907	-0.65000
19.907	-0.65000
19.907	-0.65000
19.908	-0.65000
19.908	-0.65000
19.908	-0.65000
19.908	-0.65000
19.908	-0.65000
19.908	-0.65000
19.908	-0.65000
19.909	-0.65000
19.909	-0.65000
19.909	-0.65000
19.909	-0.65000
19.909	-0.65000
19.909	-0.65000
19.909	-0.65000
19.910	-0.65000
19.910	-0.65000
19.910	-0.65000
19.910	-0.65000
19.910	-0.65000
19.910	-0.65000
19.911	-0.65000
19.911	-0.65000
19.911	-0.65000
19.911	-0.65000
19.911	-0.65000
19.912	-0.65000
19.912	-0.65000
19.912	-0.65000
19.912	-0.65000
19.912	-0.65000
19.912	-0.65000

Time (s)	Tip potential during pulse (V)
19.912	-0.65000
19.913	-0.65000
19.913	-0.65000
19.913	-0.65000
19.913	-0.65000
19.913	-0.65000
19.914	-0.65000
19.914	-0.65000
19.914	-0.65000
19.914	-0.65000
19.914	-0.65000
19.915	-0.65000
19.915	-0.65000
19.915	-0.65000
19.915	-0.65000
19.915	-0.65000
19.915	-0.65000
19.915	-0.65000
19.915	-0.65000
19.916	-0.65000
19.916	-0.65000
19.916	-0.65000
19.916	-0.65000
19.916	-0.65000
19.917	-0.65000
19.917	-0.65000
19.917	-0.65000
19.917	-0.65000
19.917	-0.65000
19.917	-0.65000
19.917	-0.65000
19.918	-0.65000
19.918	-0.65000
19.918	-0.65000
19.918	-0.65000
19.918	-0.65000
19.918	-0.65000
19.918	-0.65000
19.918	-0.65000
19.919	-0.65000

Time (s)	Tip potential during pulse (V)
19.919	-0.65000
19.919	-0.65000
19.919	-0.65000
19.919	-0.65000
19.919	-0.65000
19.919	-0.65000
19.920	-0.65000
19.920	-0.65000
19.920	-0.65000
19.920	-0.65000
19.920	-0.65000
19.920	-0.65000
19.920	-0.65000
19.921	-0.65000
19.921	-0.65000
19.921	-0.65000
19.921	-0.65000
19.921	-0.65000
19.921	-0.65000
19.921	-0.65000
19.921	-0.65000
19.922	-0.65000
19.922	-0.65000
19.922	-0.65000
19.922	-0.65000
19.922	-0.65000
19.922	-0.65000
19.922	-0.65000
19.923	-0.65000
19.923	-0.65000
19.923	-0.65000
19.923	-0.65000
19.923	-0.65000
19.923	-0.65000
19.923	-0.65000
19.924	-0.65000
19.924	-0.65000
19.924	-0.65000
19.924	-0.65000
19.924	-0.65000

Time (s)	Tip potential during pulse (V)
19.924	-0.65000
19.925	-0.65000
19.925	-0.65000
19.925	-0.65000
19.925	-0.65000
19.925	-0.65000
19.925	-0.65000
19.925	-0.65000
19.926	-0.65000
19.926	-0.65000
19.926	-0.65000
19.926	-0.65000
19.926	-0.65000
19.926	-0.65000
19.926	-0.65000
19.927	-0.65000
19.927	-0.65000
19.927	-0.65000
19.927	-0.65000
19.927	-0.65000
19.927	-0.65000
19.928	-0.65000
19.928	-0.65000
19.928	-0.65000
19.928	-0.65000
19.928	-0.65000
19.928	-0.65000
19.928	-0.65000
19.929	-0.65000
19.929	-0.65000
19.929	-0.65000
19.929	-0.65000
19.929	-0.65000
19.929	-0.65000
19.929	-0.65000
19.930	-0.65000
19.930	-0.65000
19.930	-0.65000
19.930	-0.65000
19.930	-0.65000

Time (s)	Tip potential during pulse (V)
19.930	-0.65000
19.931	-0.65000
19.931	-0.65000
19.931	-0.65000
19.931	-0.65000
19.931	-0.65000
19.931	-0.65000
19.931	-0.65000
19.932	-0.65000
19.932	-0.65000
19.932	-0.65000
19.932	-0.65000
19.932	-0.65000
19.932	-0.65000
19.932	-0.65000
19.933	-0.65000
19.933	-0.65000
19.933	-0.65000
19.933	-0.65000
19.933	-0.65000
19.933	-0.65000
19.933	-0.65000
19.933	-0.65000
19.934	-0.65000
19.934	-0.65000
19.934	-0.65000
19.934	-0.65000
19.934	-0.65000
19.935	-0.65000
19.935	-0.65000
19.935	-0.65000
19.935	-0.65000
19.935	-0.65000
19.935	-0.65000
19.935	-0.65000
19.936	-0.65000
19.936	-0.65000
19.936	-0.65000
19.936	-0.65000
19.936	-0.65000

Time (s)	Tip potential during pulse (V)
19.936	-0.65000
19.937	-0.65000
19.937	-0.65000
19.937	-0.65000
19.937	-0.65000
19.937	-0.65000
19.937	-0.65000
19.937	-0.65000
19.938	-0.65000
19.938	-0.65000
19.938	-0.65000
19.938	-0.65000
19.938	-0.65000
19.939	-0.65000
19.939	-0.65000
19.939	-0.65000
19.939	-0.65000
19.939	-0.65000
19.939	-0.65000
19.939	-0.65000
19.940	-0.65000
19.940	-0.65000
19.940	-0.65000
19.940	-0.65000
19.941	-0.65000
19.941	-0.65000
19.941	-0.65000
19.941	-0.65000
19.941	-0.65000
19.942	-0.65000
19.942	-0.65000
19.942	-0.65000
19.942	-0.65000
19.942	-0.65000
19.942	-0.65000
19.942	-0.65000
19.942	-0.65000
19.943	-0.65000
19.943	-0.65000
19.943	-0.65000

Time (s)	Tip potential during pulse (V)
19.943	-0.65000
19.943	-0.65000
19.944	-0.65000
19.944	-0.65000
19.944	-0.65000
19.944	-0.65000
19.945	-0.65000
19.945	-0.65000
19.945	-0.65000
19.945	-0.65000
19.945	-0.65000
19.946	-0.65000
19.946	-0.65000
19.946	-0.65000
19.946	-0.65000
19.946	-0.65000
19.947	-0.65000
19.947	-0.65000
19.947	-0.65000
19.947	-0.65000
19.947	-0.65000
19.947	-0.65000
19.947	-0.65000
19.948	-0.65000
19.948	-0.65000
19.948	-0.65000
19.948	-0.65000
19.948	-0.65000
19.948	-0.65000
19.949	-0.65000
19.949	-0.65000
19.949	-0.65000
19.949	-0.65000
19.949	-0.65000
19.950	-0.65000
19.950	-0.65000
19.950	-0.65000
19.950	-0.65000
19.951	-0.65000
19.951	-0.65000

Time (s)	Tip potential during pulse (V)
19.951	-0.65000
19.951	-0.65000
19.951	-0.65000
19.952	-0.65000
19.952	-0.65000
19.952	-0.65000
19.952	-0.65000
19.952	-0.65000
19.952	-0.65000
19.952	-0.65000
19.953	-0.65000
19.953	-0.65000
19.953	-0.65000
19.953	-0.65000
19.953	-0.65000
19.954	-0.65000
19.954	-0.65000
19.954	-0.65000
19.954	-0.65000
19.954	-0.65000
19.954	-0.65000
19.954	-0.65000
19.955	-0.65000
19.955	-0.65000
19.955	-0.65000
19.955	-0.65000
19.955	-0.65000
19.955	-0.65000
19.956	-0.65000
19.956	-0.65000
19.956	-0.65000
19.957	-0.65000
19.957	-0.65000
19.957	-0.65000
19.957	-0.65000
19.957	-0.65000
19.958	-0.65000
19.958	-0.65000
19.958	-0.65000
19.958	-0.65000

Time (s)	Tip potential during pulse (V)
19.959	-0.65000
19.959	-0.65000
19.959	-0.65000
19.959	-0.65000
19.959	-0.65000
19.960	-0.65000
19.960	-0.65000
19.960	-0.65000
19.960	-0.65000
19.960	-0.65000
19.960	-0.65000
19.961	-0.65000
19.961	-0.65000
19.961	-0.65000
19.961	-0.65000
19.961	-0.65000
19.962	-0.65000
19.962	-0.65000
19.962	-0.65000
19.962	-0.65000
19.962	-0.65000
19.962	-0.65000
19.963	-0.65000
19.963	-0.65000
19.963	-0.65000
19.963	-0.65000
19.963	-0.65000
19.963	-0.65000
19.963	-0.65000
19.964	-0.65000
19.964	-0.65000
19.964	-0.65000
19.964	-0.65000
19.965	-0.65000
19.965	-0.65000
19.965	-0.65000
19.965	-0.65000
19.965	-0.65000
19.965	-0.65000
19.966	-0.65000

Time (s)	Tip potential during pulse (V)
19.966	-0.65000
19.966	-0.65000
19.966	-0.65000
19.966	-0.65000
19.967	-0.65000
19.967	-0.65000
19.967	-0.65000
19.967	-0.65000
19.967	-0.65000
19.968	-0.65000
19.968	-0.65000
19.968	-0.65000
19.968	-0.65000
19.968	-0.65000
19.968	-0.65000
19.968	-0.65000
19.968	-0.65000
19.969	-0.65000
19.969	-0.65000
19.969	-0.65000
19.969	-0.65000
19.969	-0.65000
19.970	-0.65000
19.970	-0.65000
19.970	-0.65000
19.970	-0.65000
19.970	-0.65000
19.970	-0.65000
19.970	-0.65000
19.971	-0.65000
19.971	-0.65000
19.971	-0.65000
19.971	-0.65000
19.971	-0.65000
19.971	-0.65000
19.971	-0.65000
19.971	-0.65000
19.972	-0.65000
19.972	-0.65000
19.972	-0.65000

Time (s)	Tip potential during pulse (V)
19.972	-0.65000
19.972	-0.65000
19.973	-0.65000
19.973	-0.65000
19.973	-0.65000
19.973	-0.65000
19.973	-0.65000
19.974	-0.65000
19.974	-0.65000
19.974	-0.65000
19.974	-0.65000
19.975	-0.65000
19.975	-0.65000
19.975	-0.65000
19.975	-0.65000
19.975	-0.65000
19.975	-0.65000
19.975	-0.65000
19.976	-0.65000
19.976	-0.65000
19.976	-0.65000
19.976	-0.65000
19.977	-0.65000
19.977	-0.65000
19.977	-0.65000
19.977	-0.65000
19.977	-0.65000
19.978	-0.65000
19.978	-0.65000
19.978	-0.65000
19.978	-0.65000
19.978	-0.65000
19.979	-0.65000
19.979	-0.65000
19.979	-0.65000
19.979	-0.65000
19.979	-0.65000
19.980	-0.65000
19.980	-0.65000
19.980	-0.65000

Time (s)	Tip potential during pulse (V)
19.980	-0.65000
19.980	-0.65000
19.981	-0.65000
19.981	-0.65000
19.981	-0.65000
19.981	-0.65000
19.981	-0.65000
19.981	-0.65000
19.982	-0.65000
19.982	-0.65000
19.982	-0.65000
19.982	-0.65000
19.983	-0.65000
19.983	-0.65000
19.983	-0.65000
19.983	-0.65000
19.983	-0.65000
19.983	-0.65000
19.983	-0.65000
19.984	-0.65000
19.984	-0.65000
19.984	-0.65000
19.984	-0.65000
19.984	-0.65000
19.985	-0.65000
19.985	-0.65000
19.985	-0.65000
19.985	-0.65000
19.985	-0.65000
19.986	-0.65000
19.986	-0.65000
19.986	-0.65000
19.986	-0.65000
19.986	-0.65000
19.987	-0.65000
19.987	-0.65000
19.987	-0.65000
19.987	-0.65000
19.987	-0.65000
19.987	-0.65000

Time (s)	Tip potential during pulse (V)
19.988	-0.65000
19.988	-0.65000
19.988	-0.65000
19.988	-0.65000
19.988	-0.65000
19.989	-0.65000
19.989	-0.65000
19.989	-0.65000
19.989	-0.65000
19.990	-0.65000
19.990	-0.65000
19.990	-0.65000
19.991	-0.65000
19.991	-0.65000
19.991	-0.65000
19.991	-0.65000
19.991	-0.65000
19.991	-0.65000
19.992	-0.65000
19.992	-0.65000
19.992	-0.65000
19.992	-0.65000
19.992	-0.65000
19.992	-0.65000
19.992	-0.65000
19.993	-0.65000
19.993	-0.65000
19.993	-0.65000
19.993	-0.65000
19.993	-0.65000
19.993	-0.65000
19.993	-0.65000
19.994	-0.65000
19.994	-0.65000
19.994	-0.65000
19.994	-0.65000
19.994	-0.65000
19.994	-0.65000
19.994	-0.65000
19.995	-0.65000

Time (s)	Tip potential during pulse (V)
19.995	-0.65000
19.995	-0.65000
19.995	-0.65000
19.995	-0.65000
19.995	-0.65000
19.995	-0.65000
19.995	-0.65000
19.996	-0.65000
19.996	-0.65000
19.996	-0.65000
19.996	-0.65000
19.996	-0.65000
19.997	-0.65000
19.997	-0.65000
19.997	-0.65000
19.997	-0.65000
19.997	-0.65000
19.997	-0.65000
19.997	-0.65000
19.998	-0.65000
19.998	-0.65000
19.998	-0.65000
19.998	-0.65000
19.998	-0.65000
19.998	-0.65000
19.998	-0.65000
19.999	-0.65000
19.999	-0.65000
19.999	-0.65000
19.999	-0.65000
19.999	-0.65000
19.999	-0.65000
20.000	-0.65000
20.000	-0.65000
20.000	-0.65000
20.000	-0.65000
20.000	-0.65000
20.000	-0.65000
20.000	-0.65000
20.001	-0.65000

Time (s)	Tip potential during pulse (V)
20.001	-0.65000
20.001	-0.65000
20.001	-0.65000
20.001	-0.65000
20.001	-0.65000
20.001	-0.65000
20.001	-0.65000
20.002	-0.65000
20.002	-0.65000
20.002	-0.65000
20.002	-0.65000
20.002	-0.65000
20.002	-0.65000
20.002	-0.65000
20.003	-0.65000
20.003	-0.65000
20.003	-0.65000
20.003	-0.65000
20.003	-0.65000
20.003	-0.65000
20.003	-0.65000
20.004	-0.65000
20.004	-0.65000
20.004	-0.65000
20.004	-0.65000
20.004	-0.65000
20.004	-0.65000
20.004	-0.65000
20.004	-0.65000
20.005	-0.65000
20.005	-0.65000
20.005	-0.65000
20.005	-0.65000
20.005	-0.65000
20.005	-0.65000
20.005	-0.65000
20.005	-0.65000
20.006	-0.65000
20.006	-0.65000
20.006	-0.65000

Time (s)	Tip potential during pulse (V)
20.006	-0.65000
20.006	-0.65000
20.007	-0.65000
20.007	-0.65000
20.007	-0.65000
20.007	-0.65000
20.007	-0.65000
20.007	-0.65000
20.007	-0.65000
20.008	-0.65000
20.008	-0.65000
20.008	-0.65000
20.008	-0.65000
20.008	-0.65000
20.008	-0.65000
20.008	-0.65000
20.009	-0.65000
20.009	-0.65000
20.009	-0.65000
20.009	-0.65000
20.009	-0.65000
20.009	-0.65000
20.010	-0.65000
20.010	-0.65000
20.010	-0.65000
20.011	-0.65000
20.011	-0.65000
20.011	-0.65000
20.011	-0.65000
20.011	-0.65000
20.011	-0.65000
20.012	-0.65000
20.012	-0.65000
20.012	-0.65000
20.012	-0.65000
20.012	-0.65000
20.013	-0.65000
20.013	-0.65000
20.013	-0.65000
20.013	-0.65000

Time (s)	Tip potential during pulse (V)
20.013	-0.65000
20.013	-0.65000
20.014	-0.65000
20.014	-0.65000
20.014	-0.65000
20.014	-0.65000
20.014	-0.65000
20.014	-0.65000
20.015	-0.65000
20.015	-0.65000
20.015	-0.65000
20.015	-0.65000
20.016	-0.65000
20.016	-0.65000
20.016	-0.65000
20.016	-0.65000
20.017	-0.65000
20.017	-0.65000
20.017	-0.65000
20.017	-0.65000
20.017	-0.65000
20.017	-0.65000
20.017	-0.65000
20.018	-0.65000
20.018	-0.65000
20.018	-0.65000
20.018	-0.65000
20.018	-0.65000
20.018	-0.65000
20.018	-0.65000
20.019	-0.65000
20.019	-0.65000
20.019	-0.65000
20.020	-0.65000
20.020	-0.65000
20.020	-0.65000
20.020	-0.65000
20.020	-0.65000
20.020	-0.65000
20.021	-0.65000

Time (s)	Tip potential during pulse (V)
20.021	-0.65000
20.021	-0.65000
20.021	-0.65000
20.021	-0.65000
20.022	-0.65000
20.022	-0.65000
20.022	-0.65000
20.022	-0.65000
20.022	-0.65000
20.023	-0.65000
20.023	-0.65000
20.023	-0.65000
20.023	-0.65000
20.023	-0.65000
20.024	-0.65000
20.024	-0.65000
20.024	-0.65000
20.024	-0.65000
20.025	-0.65000
20.025	-0.65000
20.025	-0.65000
20.025	-0.65000
20.025	-0.65000
20.025	-0.65000
20.026	-0.65000
20.026	-0.65000
20.026	-0.65000
20.026	-0.65000
20.026	-0.65000
20.026	-0.65000
20.026	-0.65000
20.027	-0.65000
20.027	-0.65000
20.027	-0.65000
20.027	-0.65000
20.027	-0.65000
20.028	-0.65000
20.028	-0.65000
20.028	-0.65000
20.029	-0.65000

Time (s)	Tip potential during pulse (V)
20.029	-0.65000
20.029	-0.65000
20.029	-0.65000
20.029	-0.65000
20.029	-0.65000
20.030	-0.65000
20.030	-0.65000
20.030	-0.65000
20.030	-0.65000
20.030	-0.65000
20.031	-0.65000
20.031	-0.65000
20.031	-0.65000
20.031	-0.65000
20.031	-0.65000
20.032	-0.65000
20.032	-0.65000
20.032	-0.65000
20.032	-0.65000
20.032	-0.65000
20.032	-0.65000
20.033	-0.65000
20.033	-0.65000
20.033	-0.65000
20.033	-0.65000
20.033	-0.65000
20.034	-0.65000
20.034	-0.65000
20.034	-0.65000
20.034	-0.65000
20.035	-0.65000
20.035	-0.65000
20.035	-0.65000
20.035	-0.65000
20.035	-0.65000
20.036	-0.65000
20.036	-0.65000
20.036	-0.65000
20.036	-0.65000
20.036	-0.65000

Time (s)	Tip potential during pulse (V)
20.037	-0.65000
20.037	-0.65000
20.037	-0.65000
20.037	-0.65000
20.038	-0.65000
20.038	-0.65000
20.038	-0.65000
20.038	-0.65000
20.039	-0.65000
20.039	-0.65000
20.039	-0.65000
20.039	-0.65000
20.040	-0.65000
20.040	-0.65000
20.040	-0.65000
20.040	-0.65000
20.040	-0.65000
20.041	-0.65000
20.041	-0.65000
20.041	-0.65000
20.041	-0.65000
20.041	-0.65000
20.041	-0.65000
20.041	-0.65000
20.042	-0.65000
20.042	-0.65000
20.042	-0.65000
20.042	-0.65000
20.043	-0.65000
20.043	-0.65000
20.043	-0.65000
20.043	-0.65000
20.043	-0.65000
20.044	-0.65000
20.044	-0.65000
20.044	-0.65000
20.045	-0.65000
20.045	-0.65000
20.045	-0.65000
20.045	-0.65000

Time (s)	Tip potential during pulse (V)
20.045	-0.65000
20.045	-0.65000
20.045	-0.65000
20.046	-0.65000
20.046	-0.65000
20.046	-0.65000
20.046	-0.65000
20.046	-0.65000
20.047	-0.65000
20.047	-0.65000
20.047	-0.65000
20.047	-0.65000
20.047	-0.65000
20.047	-0.65000
20.048	-0.65000
20.048	-0.65000
20.048	-0.65000
20.048	-0.65000
20.048	-0.65000
20.049	-0.65000
20.049	-0.65000
20.049	-0.65000
20.049	-0.65000
20.049	-0.65000
20.049	-0.65000
20.050	-0.65000
20.050	-0.65000
20.050	-0.65000
20.050	-0.65000
20.050	-0.65000
20.051	-0.65000
20.051	-0.65000
20.051	-0.65000
20.051	-0.65000
20.051	-0.65000
20.052	-0.65000
20.052	-0.65000
20.052	-0.65000
20.052	-0.65000
20.052	-0.65000

Time (s)	Tip potential during pulse (V)
20.052	-0.65000
20.052	-0.65000
20.053	-0.65000
20.053	-0.65000
20.053	-0.65000
20.053	-0.65000
20.054	-0.65000
20.054	-0.65000
20.054	-0.65000
20.054	-0.65000
20.054	-0.65000
20.054	-0.65000
20.054	-0.65000
20.055	-0.65000
20.055	-0.65000
20.055	-0.65000
20.055	-0.65000
20.055	-0.65000
20.055	-0.65000
20.055	-0.65000
20.055	-0.65000
20.055	-0.65000
20.056	-0.65000
20.056	-0.65000
20.056	-0.65000
20.056	-0.65000
20.056	-0.65000
20.056	-0.65000
20.056	-0.65000
20.057	-0.65000
20.057	-0.65000
20.057	-0.65000
20.057	-0.65000
20.057	-0.65000
20.058	-0.65000
20.058	-0.65000
20.058	-0.65000
20.058	-0.65000
20.058	-0.65000
20.058	-0.65000

Time (s)	Tip potential during pulse (V)
20.059	-0.65000
20.059	-0.65000
20.059	-0.65000
20.059	-0.65000
20.059	-0.65000
20.060	-0.65000
20.060	-0.65000
20.060	-0.65000
20.060	-0.65000
20.060	-0.65000
20.060	-0.65000
20.060	-0.65000
20.060	-0.65000
20.061	-0.65000
20.061	-0.65000
20.061	-0.65000
20.061	-0.65000
20.061	-0.65000
20.061	-0.65000
20.061	-0.65000
20.062	-0.65000
20.062	-0.65000
20.062	-0.65000
20.062	-0.65000
20.062	-0.65000
20.063	-0.65000
20.063	-0.65000
20.063	-0.65000
20.063	-0.65000
20.063	-0.65000
20.063	-0.65000
20.063	-0.65000
20.063	-0.65000
20.064	-0.65000
20.064	-0.65000
20.064	-0.65000
20.064	-0.65000
20.064	-0.65000
20.064	-0.65000
20.064	-0.65000

Time (s)	Tip potential during pulse (V)
20.065	-0.65000
20.065	-0.65000
20.065	-0.65000
20.065	-0.65000
20.065	-0.65000
20.065	-0.65000
20.065	-0.65000
20.066	-0.65000
20.066	-0.65000
20.066	-0.65000
20.066	-0.65000
20.066	-0.65000
20.067	-0.65000
20.067	-0.65000
20.067	-0.65000
20.067	-0.65000
20.067	-0.65000
20.067	-0.65000
20.067	-0.65000
20.068	-0.65000
20.068	-0.65000
20.068	-0.65000
20.068	-0.65000
20.068	-0.65000
20.068	-0.65000
20.068	-0.65000
20.069	-0.65000
20.069	-0.65000
20.069	-0.65000
20.069	-0.65000
20.069	-0.65000
20.070	-0.65000
20.070	-0.65000
20.070	-0.65000
20.070	-0.65000
20.070	-0.65000
20.070	-0.65000
20.070	-0.65000
20.070	-0.65000
20.071	-0.65000

Time (s)	Tip potential during pulse (V)
20.071	-0.65000
20.071	-0.65000
20.071	-0.65000
20.071	-0.65000
20.071	-0.65000
20.071	-0.65000
20.072	-0.65000
20.072	-0.65000
20.072	-0.65000
20.072	-0.65000
20.072	-0.65000
20.073	-0.65000
20.073	-0.65000
20.073	-0.65000
20.073	-0.65000
20.073	-0.65000
20.073	-0.65000
20.073	-0.65000
20.073	-0.65000
20.074	-0.65000
20.074	-0.65000
20.074	-0.65000
20.074	-0.65000
20.074	-0.65000
20.074	-0.65000
20.074	-0.65000
20.075	-0.65000
20.075	-0.65000
20.075	-0.65000
20.075	-0.65000
20.075	-0.65000
20.075	-0.65000
20.076	-0.65000
20.076	-0.65000
20.076	-0.65000
20.076	-0.65000
20.076	-0.65000
20.076	-0.65000
20.076	-0.65000
20.077	-0.65000

Time (s)	Tip potential during pulse (V)
20.077	-0.65000
20.077	-0.65000
20.077	-0.65000
20.077	-0.65000
20.077	-0.65000
20.077	-0.65000
20.078	-0.65000
20.078	-0.65000
20.078	-0.65000
20.078	-0.65000
20.079	-0.65000
20.079	-0.65000
20.079	-0.65000
20.079	-0.65000
20.079	-0.65000
20.079	-0.65000
20.079	-0.65000
20.080	-0.65000
20.080	-0.65000
20.080	-0.65000
20.080	-0.65000
20.080	-0.65000
20.080	-0.65000
20.080	-0.65000
20.081	-0.65000
20.081	-0.65000
20.081	-0.65000
20.081	-0.65000
20.082	-0.65000
20.082	-0.65000
20.082	-0.65000
20.082	-0.65000
20.082	-0.65000
20.082	-0.65000
20.082	-0.65000
20.083	-0.65000
20.083	-0.65000
20.083	-0.65000
20.083	-0.65000
20.083	-0.65000

Time (s)	Tip potential during pulse (V)
20.083	-0.65000
20.083	-0.65000
20.084	-0.65000
20.084	-0.65000
20.084	-0.65000
20.084	-0.65000
20.084	-0.65000
20.084	-0.65000
20.084	-0.65000
20.085	-0.65000
20.085	-0.65000
20.085	-0.65000
20.085	-0.65000
20.085	-0.65000
20.085	-0.65000
20.086	-0.65000
20.086	-0.65000
20.086	-0.65000
20.086	-0.65000
20.086	-0.65000
20.087	-0.65000
20.087	-0.65000
20.087	-0.65000
20.087	-0.65000
20.088	-0.65000
20.088	-0.65000
20.088	-0.65000
20.088	-0.65000
20.088	-0.65000
20.089	-0.65000
20.089	-0.65000
20.089	-0.65000
20.089	-0.65000
20.089	-0.65000
20.090	-0.65000
20.090	-0.65000
20.090	-0.65000
20.090	-0.65000
20.090	-0.65000
20.090	-0.65000

Time (s)	Tip potential during pulse (V)
20.091	-0.65000
20.091	-0.65000
20.091	-0.65000
20.091	-0.65000
20.091	-0.65000
20.091	-0.65000
20.092	-0.65000
20.092	-0.65000
20.092	-0.65000
20.092	-0.65000
20.092	-0.65000
20.093	-0.65000
20.093	-0.65000
20.093	-0.65000
20.093	-0.65000
20.093	-0.65000
20.093	-0.65000
20.094	-0.65000
20.094	-0.65000
20.094	-0.65000
20.094	-0.65000
20.094	-0.65000
20.094	-0.65000
20.094	-0.65000
20.095	-0.65000
20.095	-0.65000
20.095	-0.65000
20.095	-0.65000
20.095	-0.65000
20.096	-0.65000
20.096	-0.65000
20.096	-0.65000
20.096	-0.65000
20.096	-0.65000
20.097	-0.65000
20.097	-0.65000
20.097	-0.65000
20.097	-0.65000
20.097	-0.65000
20.097	-0.65000

Time (s)	Tip potential during pulse (V)
20.098	-0.65000
20.098	-0.65000
20.098	-0.65000
20.098	-0.65000
20.098	-0.65000
20.099	-0.65000
20.099	-0.65000
20.099	-0.65000
20.099	-0.65000
20.099	-0.65000
20.099	-0.65000
20.099	-0.65000
20.099	-0.65000
20.100	-0.65000
20.100	-0.65000
20.100	-0.65000
20.100	-0.65000
20.100	-0.65000
20.101	-0.65000
20.101	-0.65000
20.101	-0.65000
20.101	-0.65000
20.101	-0.65000
20.101	-0.65000
20.101	-0.65000
20.102	-0.65000
20.102	-0.65000
20.102	-0.65000
20.102	-0.65000
20.102	-0.65000
20.102	-0.65000
20.102	-0.65000
20.102	-0.65000
20.103	-0.65000
20.103	-0.65000
20.103	-0.65000
20.103	-0.65000
20.103	-0.65000
20.103	-0.65000
20.103	-0.65000

Time (s)	Tip potential during pulse (V)
20.104	-0.65000
20.104	-0.65000
20.104	-0.65000
20.104	-0.65000
20.104	-0.65000
20.104	-0.65000
20.104	-0.65000
20.105	-0.65000
20.105	-0.65000
20.105	-0.65000
20.105	-0.65000
20.105	-0.65000
20.105	-0.65000
20.105	-0.65000
20.105	-0.65000
20.106	-0.65000
20.106	-0.65000
20.106	-0.65000
20.106	-0.65000
20.106	-0.65000
20.106	-0.65000
20.106	-0.65000
20.107	-0.65000
20.107	-0.65000
20.107	-0.65000
20.107	-0.65000
20.107	-0.65000
20.107	-0.65000
20.107	-0.65000
20.108	-0.65000
20.108	-0.65000
20.108	-0.65000
20.108	-0.65000
20.108	-0.65000
20.108	-0.65000
20.109	-0.65000
20.109	-0.65000
20.109	-0.65000
20.109	-0.65000
20.109	-0.65000

Time (s)	Tip potential during pulse (V)
20.109	-0.65000
20.109	-0.65000
20.110	-0.65000
20.110	-0.65000
20.110	-0.65000
20.110	-0.65000
20.110	-0.65000
20.110	-0.65000
20.110	-0.65000
20.111	-0.65000
20.111	-0.65000
20.111	-0.65000
20.111	-0.65000
20.111	-0.65000
20.111	-0.65000
20.111	-0.65000
20.111	-0.65000
20.112	-0.65000
20.112	-0.65000
20.112	-0.65000
20.112	-0.65000
20.112	-0.65000
20.112	-0.65000
20.112	-0.65000
20.113	-0.65000
20.113	-0.65000
20.113	-0.65000
20.113	-0.65000
20.113	-0.65000
20.113	-0.65000
20.113	-0.65000
20.114	-0.65000
20.114	-0.65000
20.114	-0.65000
20.114	-0.65000
20.114	-0.65000
20.114	-0.65000
20.114	-0.65000
20.114	-0.65000
20.115	-0.65000

Time (s)	Tip potential during pulse (V)
20.115	-0.65000
20.115	-0.65000
20.115	-0.65000
20.115	-0.65000
20.115	-0.65000
20.115	-0.65000
20.116	-0.65000
20.116	-0.65000
20.116	-0.65000
20.116	-0.65000
20.116	-0.65000
20.117	-0.65000
20.117	-0.65000
20.117	-0.65000
20.117	-0.65000
20.117	-0.65000
20.117	-0.65000
20.117	-0.65000
20.117	-0.65000
20.118	-0.65000
20.118	-0.65000
20.118	-0.65000
20.118	-0.65000
20.118	-0.65000
20.118	-0.65000
20.118	-0.65000
20.119	-0.65000
20.119	-0.65000
20.119	-0.65000
20.119	-0.65000
20.119	-0.65000
20.119	-0.65000
20.119	-0.65000
20.120	-0.65000
20.120	-0.65000
20.120	-0.65000
20.120	-0.65000
20.120	-0.65000
20.120	-0.65000
20.120	-0.65000

Time (s)	Tip potential during pulse (V)
20.120	-0.65000
20.121	-0.65000
20.121	-0.65000
20.121	-0.65000
20.121	-0.65000
20.121	-0.65000
20.121	-0.65000
20.121	-0.65000
20.122	-0.65000
20.122	-0.65000
20.122	-0.65000
20.122	-0.65000
20.122	-0.65000
20.122	-0.65000
20.122	-0.65000
20.123	-0.65000
20.123	-0.65000
20.123	-0.65000
20.123	-0.65000
20.123	-0.65000
20.123	-0.65000
20.123	-0.65000
20.123	-0.65000
20.124	-0.65000
20.124	-0.65000
20.124	-0.65000
20.124	-0.65000
20.124	-0.65000
20.125	-0.65000
20.125	-0.65000
20.125	-0.65000
20.125	-0.65000
20.125	-0.65000
20.125	-0.65000
20.125	-0.65000
20.126	-0.65000
20.126	-0.65000
20.126	-0.65000
20.126	-0.65000
20.126	-0.65000

Time (s)	Tip potential during pulse (V)
20.126	-0.65000
20.127	-0.65000
20.127	-0.65000
20.127	-0.65000
20.127	-0.65000
20.127	-0.65000
20.127	-0.65000
20.127	-0.65000
20.128	-0.65000
20.128	-0.65000
20.128	-0.65000
20.128	-0.65000
20.128	-0.65000
20.128	-0.65000
20.128	-0.65000
20.129	-0.65000
20.129	-0.65000
20.129	-0.65000
20.129	-0.65000
20.129	-0.65000
20.129	-0.65000
20.129	-0.65000
20.129	-0.65000
20.130	-0.65000
20.130	-0.65000
20.130	-0.65000
20.130	-0.65000
20.130	-0.65000
20.130	-0.65000
20.130	-0.65000
20.131	-0.65000
20.131	-0.65000
20.131	-0.65000
20.131	-0.65000
20.131	-0.65000
20.131	-0.65000
20.131	-0.65000
20.132	-0.65000
20.132	-0.65000
20.132	-0.65000

Time (s)	Tip potential during pulse (V)
20.132	-0.65000
20.132	-0.65000
20.132	-0.65000
20.132	-0.65000
20.133	-0.65000
20.133	-0.65000
20.133	-0.65000
20.133	-0.65000
20.133	-0.65000
20.133	-0.65000
20.134	-0.65000
20.134	-0.65000
20.134	-0.65000
20.134	-0.65000
20.134	-0.65000
20.134	-0.65000
20.134	-0.65000
20.135	-0.65000
20.135	-0.65000
20.135	-0.65000
20.135	-0.65000
20.135	-0.65000
20.135	-0.65000
20.135	-0.65000
20.135	-0.65000
20.136	-0.65000
20.136	-0.65000
20.136	-0.65000
20.136	-0.65000
20.136	-0.65000
20.136	-0.65000
20.136	-0.65000
20.137	-0.65000
20.137	-0.65000
20.137	-0.65000
20.137	-0.65000
20.137	-0.65000
20.137	-0.65000
20.137	-0.65000
20.138	-0.65000

Time (s)	Tip potential during pulse (V)
20.138	-0.65000
20.138	-0.65000
20.138	-0.65000
20.138	-0.65000
20.138	-0.65000
20.138	-0.65000
20.138	-0.65000
20.139	-0.65000
20.139	-0.65000
20.139	-0.65000
20.139	-0.65000
20.139	-0.65000
20.139	-0.65000
20.139	-0.65000
20.140	-0.65000
20.140	-0.65000
20.140	-0.65000
20.140	-0.65000
20.140	-0.65000
20.140	-0.65000
20.140	-0.65000
20.141	-0.65000
20.141	-0.65000
20.141	-0.65000
20.141	-0.65000
20.141	-0.65000
20.142	-0.65000
20.142	-0.65000
20.142	-0.65000
20.142	-0.65000
20.142	-0.65000
20.143	-0.65000
20.143	-0.65000
20.143	-0.65000
20.143	-0.65000
20.143	-0.65000
20.143	-0.65000
20.143	-0.65000
20.143	-0.65000
20.144	-0.65000

Time (s)	Tip potential during pulse (V)
20.144	-0.65000
20.144	-0.65000
20.144	-0.65000
20.144	-0.65000
20.144	-0.65000
20.144	-0.65000
20.145	-0.65000
20.145	-0.65000
20.145	-0.65000
20.145	-0.65000
20.145	-0.65000
20.145	-0.65000
20.145	-0.65000
20.146	-0.65000
20.146	-0.65000
20.146	-0.65000
20.146	-0.65000
20.146	-0.65000
20.146	-0.65000
20.146	-0.65000
20.146	-0.65000
20.147	-0.65000
20.147	-0.65000
20.147	-0.65000
20.147	-0.65000
20.147	-0.65000
20.147	-0.65000
20.147	-0.65000
20.148	-0.65000
20.148	-0.65000
20.148	-0.65000
20.148	-0.65000
20.148	-0.65000
20.148	-0.65000
20.148	-0.65000
20.149	-0.65000
20.149	-0.65000
20.149	-0.65000
20.149	-0.65000
20.149	-0.65000

Time (s)	Tip potential during pulse (V)
20.149	-0.65000
20.150	-0.65000
20.150	-0.65000
20.150	-0.65000
20.150	-0.65000
20.150	-0.65000
20.150	-0.65000
20.150	-0.65000
20.151	-0.65000
20.151	-0.65000
20.151	-0.65000
20.151	-0.65000
20.151	-0.65000
20.151	-0.65000
20.151	-0.65000
20.152	-0.65000
20.152	-0.65000
20.152	-0.65000
20.152	-0.65000
20.152	-0.65000
20.152	-0.65000
20.152	-0.65000
20.152	-0.65000
20.153	-0.65000
20.153	-0.65000
20.153	-0.65000
20.153	-0.65000
20.153	-0.65000
20.153	-0.65000
20.153	-0.65000
20.154	-0.65000
20.154	-0.65000
20.154	-0.65000
20.154	-0.65000
20.154	-0.65000
20.154	-0.65000
20.154	-0.65000
20.155	-0.65000
20.155	-0.65000
20.155	-0.65000

Time (s)	Tip potential during pulse (V)
20.155	-0.65000
20.155	-0.65000
20.155	-0.65000
20.155	-0.65000
20.155	-0.65000
20.156	-0.65000
20.156	-0.65000
20.156	-0.65000
20.156	-0.65000
20.156	-0.65000
20.156	-0.65000
20.156	-0.65000
20.157	-0.65000
20.157	-0.65000
20.157	-0.65000
20.157	-0.65000
20.157	-0.65000
20.157	-0.65000
20.157	-0.65000
20.158	-0.65000
20.158	-0.65000
20.158	-0.65000
20.158	-0.65000
20.158	-0.65000
20.158	-0.65000
20.158	-0.65000
20.158	-0.65000
20.159	-0.65000
20.159	-0.65000
20.159	-0.65000
20.159	-0.65000
20.159	-0.65000
20.159	-0.65000
20.159	-0.65000
20.160	-0.65000
20.160	-0.65000
20.160	-0.65000
20.160	-0.65000
20.160	-0.65000
20.160	-0.65000

Time (s)	Tip potential during pulse (V)
20.160	-0.65000
20.161	-0.65000
20.161	-0.65000
20.161	-0.65000
20.161	-0.65000
20.161	-0.65000
20.161	-0.65000
20.161	-0.65000
20.161	-0.65000
20.162	-0.65000
20.162	-0.65000
20.162	-0.65000
20.162	-0.65000
20.162	-0.65000
20.162	-0.65000
20.162	-0.65000
20.163	-0.65000
20.163	-0.65000
20.163	-0.65000
20.163	-0.65000
20.163	-0.65000
20.163	-0.65000
20.163	-0.65000
20.164	-0.65000
20.164	-0.65000
20.164	-0.65000
20.164	-0.65000
20.164	-0.65000
20.164	-0.65000
20.164	-0.65000
20.164	-0.65000
20.165	-0.65000
20.165	-0.65000
20.165	-0.65000
20.165	-0.65000
20.165	-0.65000
20.165	-0.65000
20.165	-0.65000
20.166	-0.65000
20.166	-0.65000

Time (s)	Tip potential during pulse (V)
20.166	-0.65000
20.166	-0.65000
20.166	-0.65000
20.166	-0.65000
20.166	-0.65000
20.167	-0.65000
20.167	-0.65000
20.167	-0.65000
20.167	-0.65000
20.167	-0.65000
20.167	-0.65000
20.167	-0.65000
20.167	-0.65000
20.168	-0.65000
20.168	-0.65000
20.168	-0.65000
20.168	-0.65000
20.168	-0.65000
20.168	-0.65000
20.168	-0.65000
20.169	-0.65000
20.169	-0.65000
20.169	-0.65000
20.169	-0.65000
20.169	-0.65000
20.169	-0.65000
20.169	-0.65000
20.170	-0.65000
20.170	-0.65000
20.170	-0.65000
20.170	-0.65000
20.170	-0.65000
20.170	-0.65000
20.170	-0.65000
20.170	-0.65000
20.171	-0.65000
20.171	-0.65000
20.171	-0.65000
20.171	-0.65000
20.171	-0.65000

Time (s)	Tip potential during pulse (V)
20.171	-0.65000
20.171	-0.65000
20.172	-0.65000
20.172	-0.65000
20.172	-0.65000
20.172	-0.65000
20.172	-0.65000
20.172	-0.65000
20.172	-0.65000
20.173	-0.65000
20.173	-0.65000
20.173	-0.65000
20.173	-0.65000
20.173	-0.65000
20.173	-0.65000
20.173	-0.65000
20.173	-0.65000
20.174	-0.65000
20.174	-0.65000
20.174	-0.65000
20.174	-0.65000
20.174	-0.65000
20.174	-0.65000
20.174	-0.65000
20.175	-0.65000
20.175	-0.65000
20.175	-0.65000
20.175	-0.65000
20.175	-0.65000
20.175	-0.65000
20.175	-0.65000
20.176	-0.65000
20.176	-0.65000
20.176	-0.65000
20.176	-0.65000
20.176	-0.65000
20.176	-0.65000
20.176	-0.65000
20.176	-0.65000
20.177	-0.65000

Time (s)	Tip potential during pulse (V)
20.177	-0.65000
20.177	-0.65000
20.177	-0.65000
20.177	-0.65000
20.177	-0.65000
20.177	-0.65000
20.178	-0.65000
20.178	-0.65000
20.178	-0.65000
20.178	-0.65000
20.178	-0.65000
20.178	-0.65000
20.178	-0.65000
20.179	-0.65000
20.179	-0.65000
20.179	-0.65000
20.179	-0.65000
20.179	-0.65000
20.179	-0.65000
20.179	-0.65000
20.179	-0.65000
20.180	-0.65000
20.180	-0.65000
20.180	-0.65000
20.180	-0.65000
20.180	-0.65000
20.180	-0.65000
20.180	-0.65000
20.181	-0.65000
20.181	-0.65000
20.181	-0.65000
20.181	-0.65000
20.181	-0.65000
20.181	-0.65000
20.181	-0.65000
20.182	-0.65000
20.182	-0.65000
20.182	-0.65000
20.182	-0.65000
20.182	-0.65000

Time (s)	Tip potential during pulse (V)
20.182	-0.65000
20.182	-0.65000
20.182	-0.65000
20.183	-0.65000
20.183	-0.65000
20.183	-0.65000
20.183	-0.65000
20.183	-0.65000
20.183	-0.65000
20.183	-0.65000
20.183	-0.65000
20.183	-0.65000
20.184	-0.65000
20.184	-0.65000
20.184	-0.65000
20.184	-0.65000
20.184	-0.65000
20.184	-0.65000
20.184	-0.65000
20.185	-0.65000
20.185	-0.65000
20.185	-0.65000
20.185	-0.65000
20.185	-0.65000
20.185	-0.65000
20.185	-0.65000
20.185	-0.65000
20.186	-0.65000
20.186	-0.65000
20.186	-0.65000
20.186	-0.65000
20.186	-0.65000
20.186	-0.65000
20.186	-0.65000
20.187	-0.65000
20.187	-0.65000
20.187	-0.65000
20.187	-0.65000
20.187	-0.65000
20.187	-0.65000

Time (s)	Tip potential during pulse (V)
20.187	-0.65000
20.188	-0.65000
20.188	-0.65000
20.188	-0.65000
20.188	-0.65000
20.188	-0.65000
20.188	-0.65000
20.188	-0.65000
20.188	-0.65000
20.189	-0.65000
20.189	-0.65000
20.189	-0.65000
20.189	-0.65000
20.189	-0.65000
20.189	-0.65000
20.189	-0.65000
20.190	-0.65000
20.190	-0.65000
20.190	-0.65000
20.190	-0.65000
20.190	-0.65000
20.190	-0.65000
20.190	-0.65000
20.191	-0.65000
20.191	-0.65000
20.191	-0.65000
20.191	-0.65000
20.191	-0.65000
20.191	-0.65000
20.191	-0.65000
20.192	-0.65000
20.192	-0.65000
20.192	-0.65000
20.192	-0.65000
20.192	-0.65000
20.192	-0.65000
20.192	-0.65000
20.192	-0.65000
20.192	-0.65000
20.193	-0.65000

Time (s)	Tip potential during pulse (V)
20.193	-0.65000
20.193	-0.65000
20.193	-0.65000
20.193	-0.65000
20.193	-0.65000
20.193	-0.65000
20.193	-0.65000
20.194	-0.65000
20.194	-0.65000
20.194	-0.65000
20.194	-0.65000
20.194	-0.65000
20.194	-0.65000
20.194	-0.65000
20.195	-0.65000
20.195	-0.65000
20.195	-0.65000
20.195	-0.65000
20.195	-0.65000
20.195	-0.65000
20.195	-0.65000
20.196	-0.65000
20.196	-0.65000
20.196	-0.65000
20.196	-0.65000
20.196	-0.65000
20.196	-0.65000
20.196	-0.65000
20.196	-0.65000
20.197	-0.65000
20.197	-0.65000
20.197	-0.65000
20.197	-0.65000
20.197	-0.65000
20.197	-0.65000
20.197	-0.65000
20.198	-0.65000
20.198	-0.65000
20.198	-0.65000
20.198	-0.65000

Time (s)	Tip potential during pulse (V)
20.198	-0.65000
20.198	-0.65000
20.198	-0.65000
20.199	-0.65000
20.199	-0.65000
20.199	-0.65000
20.199	-0.65000
20.199	-0.65000
20.199	-0.65000
20.199	-0.65000
20.199	-0.65000
20.200	-0.65000
20.200	-0.65000
20.200	-0.65000
20.200	-0.65000
20.200	-0.65000
20.200	-0.65000
20.200	-0.65000
20.201	-0.65000
20.201	-0.65000
20.201	-0.65000
20.201	-0.65000
20.201	-0.65000
20.201	-0.65000
20.201	-0.65000
20.202	-0.65000
20.202	-0.65000
20.202	-0.65000
20.202	-0.65000
20.202	-0.65000
20.202	-0.65000
20.202	-0.65000
20.202	-0.65000
20.203	-0.65000
20.203	-0.65000
20.203	-0.65000
20.203	-0.65000
20.203	-0.65000
20.203	-0.65000
20.203	-0.65000

Time (s)	Tip potential during pulse (V)
20.204	-0.65000
20.204	-0.65000
20.204	-0.65000
20.204	-0.65000
20.204	-0.65000
20.204	-0.65000
20.204	-0.65000
20.204	-0.65000
20.204	-0.65000
20.205	-0.65000
20.205	-0.65000
20.205	-0.65000
20.205	-0.65000
20.205	-0.65000
20.205	-0.65000
20.205	-0.65000
20.206	-0.65000
20.206	-0.65000
20.206	-0.65000
20.206	-0.65000
20.206	-0.65000
20.206	-0.65000
20.206	-0.65000
20.207	-0.65000
20.207	-0.65000
20.207	-0.65000
20.207	-0.65000
20.207	-0.65000
20.207	-0.65000
20.207	-0.65000
20.207	-0.65000
20.208	-0.65000
20.208	-0.65000
20.208	-0.65000
20.208	-0.65000
20.208	-0.65000
20.208	-0.65000
20.208	-0.65000
20.209	-0.65000
20.209	-0.65000

Time (s)	Tip potential during pulse (V)
20.209	-0.65000
20.209	-0.65000
20.209	-0.65000
20.209	-0.65000
20.209	-0.65000
20.210	-0.65000
20.210	-0.65000
20.210	-0.65000
20.210	-0.65000
20.210	-0.65000
20.210	-0.65000
20.210	-0.65000
20.210	-0.65000
20.211	-0.65000
20.211	-0.65000
20.211	-0.65000
20.211	-0.65000
20.211	-0.65000
20.211	-0.65000
20.211	-0.65000
20.212	-0.65000
20.212	-0.65000
20.212	-0.65000
20.212	-0.65000
20.212	-0.65000
20.212	-0.65000
20.212	-0.65000
20.213	-0.65000
20.213	-0.65000
20.213	-0.65000
20.213	-0.65000
20.213	-0.65000
20.213	-0.65000
20.213	-0.65000
20.213	-0.65000
20.214	-0.65000
20.214	-0.65000
20.214	-0.65000
20.214	-0.65000
20.214	-0.65000

Time (s)	Tip potential during pulse (V)
20.214	-0.65000
20.214	-0.65000
20.215	-0.65000
20.215	-0.65000
20.215	-0.65000
20.215	-0.65000
20.215	-0.65000
20.215	-0.65000
20.215	-0.65000
20.216	-0.65000
20.216	-0.65000
20.216	-0.65000
20.216	-0.65000
20.216	-0.65000
20.216	-0.65000
20.216	-0.65000
20.216	-0.65000
20.217	-0.65000
20.217	-0.65000
20.217	-0.65000
20.217	-0.65000
20.217	-0.65000
20.217	-0.65000
20.217	-0.65000
20.217	-0.65000
20.218	-0.65000
20.218	-0.65000
20.218	-0.65000
20.218	-0.65000
20.218	-0.65000
20.218	-0.65000
20.218	-0.65000
20.219	-0.65000
20.219	-0.65000
20.219	-0.65000
20.219	-0.65000
20.219	-0.65000
20.219	-0.65000
20.219	-0.65000
20.220	-0.65000

Time (s)	Tip potential during pulse (V)
20.220	-0.65000
20.220	-0.65000
20.220	-0.65000
20.220	-0.65000
20.220	-0.65000
20.220	-0.65000
20.220	-0.65000
20.221	-0.65000
20.221	-0.65000
20.221	-0.65000
20.221	-0.65000
20.221	-0.65000
20.221	-0.65000
20.221	-0.65000
20.222	-0.65000
20.222	-0.65000
20.222	-0.65000
20.222	-0.65000
20.222	-0.65000
20.222	-0.65000
20.222	-0.65000
20.223	-0.65000
20.223	-0.65000
20.223	-0.65000
20.223	-0.65000
20.223	-0.65000
20.223	-0.65000
20.223	-0.65000
20.223	-0.65000
20.224	-0.65000
20.224	-0.65000
20.224	-0.65000
20.224	-0.65000
20.224	-0.65000
20.224	-0.65000
20.224	-0.65000
20.225	-0.65000
20.225	-0.65000
20.225	-0.65000
20.225	-0.65000

Time (s)	Tip potential during pulse (V)
20.225	-0.65000
20.225	-0.65000
20.225	-0.65000
20.226	-0.65000
20.226	-0.65000
20.226	-0.65000
20.226	-0.65000
20.226	-0.65000
20.226	-0.65000
20.226	-0.65000
20.226	-0.65000
20.227	-0.65000
20.227	-0.65000
20.227	-0.65000
20.227	-0.65000
20.227	-0.65000
20.227	-0.65000
20.227	-0.65000
20.228	-0.65000
20.228	-0.65000
20.228	-0.65000
20.228	-0.65000
20.228	-0.65000
20.228	-0.65000
20.228	-0.65000
20.229	-0.65000
20.229	-0.65000
20.229	-0.65000
20.229	-0.65000
20.229	-0.65000
20.229	-0.65000
20.229	-0.65000
20.229	-0.65000
20.230	-0.65000
20.230	-0.65000
20.230	-0.65000
20.230	-0.65000
20.230	-0.65000
20.230	-0.65000
20.230	-0.65000

Time (s)	Tip potential during pulse (V)
20.231	-0.65000
20.231	-0.65000
20.231	-0.65000
20.231	-0.65000
20.231	-0.65000
20.231	-0.65000
20.231	-0.65000
20.232	-0.65000
20.232	-0.65000
20.232	-0.65000
20.232	-0.65000
20.232	-0.65000
20.232	-0.65000
20.232	-0.65000
20.232	-0.65000
20.233	-0.65000
20.233	-0.65000
20.233	-0.65000
20.233	-0.65000
20.233	-0.65000
20.233	-0.65000
20.233	-0.65000
20.234	-0.65000
20.234	-0.65000
20.234	-0.65000
20.234	-0.65000
20.234	-0.65000
20.234	-0.65000
20.234	-0.65000
20.235	-0.65000
20.235	-0.65000
20.235	-0.65000
20.235	-0.65000
20.235	-0.65000
20.235	-0.65000
20.235	-0.65000
20.235	-0.65000
20.236	-0.65000
20.236	-0.65000
20.236	-0.65000

Time (s)	Tip potential during pulse (V)
20.236	-0.65000
20.236	-0.65000
20.236	-0.65000
20.236	-0.65000
20.237	-0.65000
20.237	-0.65000
20.237	-0.65000
20.237	-0.65000
20.237	-0.65000
20.237	-0.65000
20.237	-0.65000
20.238	-0.65000
20.238	-0.65000
20.238	-0.65000
20.238	-0.65000
20.238	-0.65000
20.238	-0.65000
20.238	-0.65000
20.238	-0.65000
20.238	-0.65000
20.239	-0.65000
20.239	-0.65000
20.239	-0.65000
20.239	-0.65000
20.239	-0.65000
20.239	-0.65000
20.239	-0.65000
20.240	-0.65000
20.240	-0.65000
20.240	-0.65000
20.240	-0.65000
20.240	-0.65000
20.240	-0.65000
20.240	-0.65000
20.240	-0.65000
20.241	-0.65000
20.241	-0.65000
20.241	-0.65000
20.241	-0.65000
20.241	-0.65000

Time (s)	Tip potential during pulse (V)
20.241	-0.65000
20.241	-0.65000
20.242	-0.65000
20.242	-0.65000
20.242	-0.65000
20.242	-0.65000
20.242	-0.65000
20.242	-0.65000
20.242	-0.65000
20.243	-0.65000
20.243	-0.65000
20.243	-0.65000
20.243	-0.65000
20.243	-0.65000
20.243	-0.65000
20.243	-0.65000
20.243	-0.65000
20.244	-0.65000
20.244	-0.65000
20.244	-0.65000
20.244	-0.65000
20.244	-0.65000
20.244	-0.65000
20.244	-0.65000
20.245	-0.65000
20.245	-0.65000
20.245	-0.65000
20.245	-0.65000
20.245	-0.65000
20.245	-0.65000
20.245	-0.65000
20.246	-0.65000
20.246	-0.65000
20.246	-0.65000
20.246	-0.65000
20.246	-0.65000
20.246	-0.65000
20.246	-0.65000
20.246	-0.65000
20.247	-0.65000

Time (s)	Tip potential during pulse (V)
20.247	-0.65000
20.247	-0.65000
20.247	-0.65000
20.247	-0.65000
20.248	-0.65000
20.248	-0.65000
20.248	-0.65000
20.248	-0.65000
20.248	-0.65000
20.248	-0.65000
20.248	-0.65000
20.249	-0.65000
20.249	-0.65000
20.249	-0.65000
20.249	-0.65000
20.249	-0.65000
20.249	-0.65000
20.249	-0.65000
20.249	-0.65000
20.250	-0.65000
20.250	-0.65000
20.250	-0.65000
20.250	-0.65000
20.250	-0.65000
20.250	-0.65000
20.250	-0.65000
20.251	-0.65000
20.251	-0.65000
20.251	-0.65000
20.251	-0.65000
20.251	-0.65000
20.251	-0.65000
20.251	-0.65000
20.252	-0.65000
20.252	-0.65000
20.252	-0.65000
20.252	-0.65000
20.252	-0.65000
20.252	-0.65000
20.252	-0.65000

Time (s)	Tip potential during pulse (V)
20.252	-0.65000
20.253	-0.65000
20.253	-0.65000
20.253	-0.65000
20.253	-0.65000
20.253	-0.65000
20.253	-0.65000
20.253	-0.65000
20.254	-0.65000
20.254	-0.65000
20.254	-0.65000
20.254	-0.65000
20.254	-0.65000
20.254	-0.65000
20.254	-0.65000
20.255	-0.65000
20.255	-0.65000
20.255	-0.65000
20.255	-0.65000
20.255	-0.65000
20.255	-0.65000
20.255	-0.65000
20.256	-0.65000
20.256	-0.65000
20.256	-0.65000
20.256	-0.65000
20.256	-0.65000
20.256	-0.65000
20.256	-0.65000
20.256	-0.65000
20.256	-0.65000
20.257	-0.65000
20.257	-0.65000
20.257	-0.65000
20.257	-0.65000
20.257	-0.65000
20.257	-0.65000
20.257	-0.65000
20.257	-0.65000
20.258	-0.65000

Time (s)	Tip potential during pulse (V)
20.258	-0.65000
20.258	-0.65000
20.258	-0.65000
20.258	-0.65000
20.258	-0.65000
20.258	-0.65000
20.259	-0.65000
20.259	-0.65000
20.259	-0.65000
20.259	-0.65000
20.259	-0.65000
20.259	-0.65000
20.259	-0.65000
20.260	-0.65000
20.260	-0.65000
20.260	-0.65000
20.260	-0.65000
20.260	-0.65000
20.260	-0.65000
20.260	-0.65000
20.260	-0.65000
20.261	-0.65000
20.261	-0.65000
20.261	-0.65000
20.261	-0.65000
20.261	-0.65000
20.261	-0.65000
20.261	-0.65000
20.262	-0.65000
20.262	-0.65000
20.262	-0.65000
20.262	-0.65000
20.262	-0.65000
20.262	-0.65000
20.262	-0.65000
20.263	-0.65000
20.263	-0.65000
20.263	-0.65000
20.263	-0.65000
20.263	-0.65000

Time (s)	Tip potential during pulse (V)
20.263	-0.65000
20.263	-0.65000
20.263	-0.65000
20.264	-0.65000
20.264	-0.65000
20.264	-0.65000
20.264	-0.65000
20.264	-0.65000
20.264	-0.65000
20.264	-0.65000
20.265	-0.65000
20.265	-0.65000
20.265	-0.65000
20.265	-0.65000
20.265	-0.65000
20.265	-0.65000
20.265	-0.65000
20.266	-0.65000
20.266	-0.65000
20.266	-0.65000
20.266	-0.65000
20.266	-0.65000
20.266	-0.65000
20.266	-0.65000
20.266	-0.65000
20.267	-0.65000
20.267	-0.65000
20.267	-0.65000
20.267	-0.65000
20.267	-0.65000
20.267	-0.65000
20.268	-0.65000
20.268	-0.65000
20.268	-0.65000
20.268	-0.65000
20.268	-0.65000
20.268	-0.65000
20.268	-0.65000
20.268	-0.65000
20.269	-0.65000

Time (s)	Tip potential during pulse (V)
20.269	-0.65000
20.269	-0.65000
20.269	-0.65000
20.269	-0.65000
20.269	-0.65000
20.269	-0.65000
20.270	-0.65000
20.270	-0.65000
20.270	-0.65000
20.270	-0.65000
20.270	-0.65000
20.270	-0.65000
20.270	-0.65000
20.271	-0.65000
20.271	-0.65000
20.271	-0.65000
20.271	-0.65000
20.271	-0.65000
20.271	-0.65000
20.271	-0.65000
20.271	-0.65000
20.272	-0.65000
20.272	-0.65000
20.272	-0.65000
20.272	-0.65000
20.272	-0.65000
20.272	-0.65000
20.272	-0.65000
20.273	-0.65000
20.273	-0.65000
20.273	-0.65000
20.273	-0.65000
20.273	-0.65000
20.273	-0.65000
20.273	-0.65000
20.273	-0.65000
20.273	-0.65000
20.274	-0.65000
20.274	-0.65000
20.274	-0.65000

Time (s)	Tip potential during pulse (V)
20.274	-0.65000
20.274	-0.65000
20.274	-0.65000
20.274	-0.65000
20.275	-0.65000
20.275	-0.65000
20.275	-0.65000
20.275	-0.65000
20.275	-0.65000
20.275	-0.65000
20.275	-0.65000
20.276	-0.65000
20.276	-0.65000
20.276	-0.65000
20.276	-0.65000
20.276	-0.65000
20.276	-0.65000
20.276	-0.65000
20.276	-0.65000
20.277	-0.65000
20.277	-0.65000
20.277	-0.65000
20.277	-0.65000
20.277	-0.65000
20.277	-0.65000
20.277	-0.65000
20.278	-0.65000
20.278	-0.65000
20.278	-0.65000
20.278	-0.65000
20.278	-0.65000
20.278	-0.65000
20.278	-0.65000
20.279	-0.65000
20.279	-0.65000
20.279	-0.65000
20.279	-0.65000
20.279	-0.65000
20.279	-0.65000
20.279	-0.65000

Time (s)	Tip potential during pulse (V)
20.279	-0.65000
20.280	-0.65000
20.280	-0.65000
20.280	-0.65000
20.280	-0.65000
20.280	-0.65000
20.280	-0.65000
20.280	-0.65000
20.280	-0.65000
20.281	-0.65000
20.281	-0.65000
20.281	-0.65000
20.281	-0.65000
20.281	-0.65000
20.281	-0.65000
20.281	-0.65000
20.282	-0.65000
20.282	-0.65000
20.282	-0.65000
20.282	-0.65000
20.282	-0.65000
20.282	-0.65000
20.282	-0.65000
20.283	-0.65000
20.283	-0.65000
20.283	-0.65000
20.283	-0.65000
20.283	-0.65000
20.283	-0.65000
20.283	-0.65000
20.283	-0.65000
20.284	-0.65000
20.284	-0.65000
20.284	-0.65000
20.284	-0.65000
20.284	-0.65000
20.284	-0.65000
20.284	-0.65000
20.285	-0.65000
20.285	-0.65000

Time (s)	Tip potential during pulse (V)
20.285	-0.65000
20.285	-0.65000
20.285	-0.65000
20.285	-0.65000
20.285	-0.65000
20.286	-0.65000
20.286	-0.65000
20.286	-0.65000
20.286	-0.65000
20.286	-0.65000
20.286	-0.65000
20.286	-0.65000
20.286	-0.65000
20.287	-0.65000
20.287	-0.65000
20.287	-0.65000
20.287	-0.65000
20.287	-0.65000
20.287	-0.65000
20.287	-0.65000
20.288	-0.65000
20.288	-0.65000
20.288	-0.65000
20.288	-0.65000
20.288	-0.65000
20.288	-0.65000
20.288	-0.65000
20.289	-0.65000
20.289	-0.65000
20.289	-0.65000
20.289	-0.65000
20.289	-0.65000
20.289	-0.65000
20.289	-0.65000
20.289	-0.65000
20.290	-0.65000
20.290	-0.65000
20.290	-0.65000
20.290	-0.65000
20.290	-0.65000

Time (s)	Tip potential during pulse (V)
20.290	-0.65000
20.290	-0.65000
20.291	-0.65000
20.291	-0.65000
20.291	-0.65000
20.291	-0.65000
20.291	-0.65000
20.291	-0.65000
20.291	-0.65000
20.292	-0.65000
20.292	-0.65000
20.292	-0.65000
20.292	-0.65000
20.292	-0.65000
20.292	-0.65000
20.292	-0.65000
20.292	-0.65000
20.293	-0.65000
20.293	-0.65000
20.293	-0.65000
20.293	-0.65000
20.293	-0.65000
20.293	-0.65000
20.293	-0.65000
20.294	-0.65000
20.294	-0.65000
20.294	-0.65000
20.294	-0.65000
20.294	-0.65000
20.294	-0.65000
20.294	-0.65000
20.295	-0.65000
20.295	-0.65000
20.295	-0.65000
20.295	-0.65000
20.295	-0.65000
20.295	-0.65000
20.295	-0.65000
20.295	-0.65000
20.296	-0.65000

Time (s)	Tip potential during pulse (V)
20.296	-0.65000
20.296	-0.65000
20.296	-0.65000
20.296	-0.65000
20.296	-0.65000
20.296	-0.65000
20.297	-0.65000
20.297	-0.65000
20.297	-0.65000
20.297	-0.65000
20.297	-0.65000
20.297	-0.65000
20.297	-0.65000
20.298	-0.65000
20.298	-0.65000
20.298	-0.65000
20.298	-0.65000
20.298	-0.65000
20.298	-0.65000
20.298	-0.65000
20.298	-0.65000
20.299	-0.65000
20.299	-0.65000
20.299	-0.65000
20.299	-0.65000
20.299	-0.65000
20.299	-0.65000
20.299	-0.65000
20.300	-0.65000
20.300	-0.65000
20.300	-0.65000
20.300	-0.65000
20.300	-0.65000
20.300	-0.65000
20.300	-0.65000
20.301	-0.65000
20.301	-0.65000
20.301	-0.65000
20.301	-0.65000
20.301	-0.65000

Time (s)	Tip potential during pulse (V)
20.301	-0.65000
20.301	-0.65000
20.301	-0.65000
20.302	-0.65000
20.302	-0.65000
20.302	-0.65000
20.302	-0.65000
20.302	-0.65000
20.302	-0.65000
20.302	-0.65000
20.303	-0.65000
20.303	-0.65000
20.303	-0.65000
20.303	-0.65000
20.303	-0.65000
20.303	-0.65000
20.303	-0.65000
20.304	-0.65000
20.304	-0.65000
20.304	-0.65000
20.304	-0.65000
20.304	-0.65000
20.304	-0.65000
20.304	-0.65000
20.304	-0.65000
20.305	-0.65000
20.305	-0.65000
20.305	-0.65000
20.305	-0.65000
20.305	-0.65000
20.305	-0.65000
20.305	-0.65000
20.306	-0.65000
20.306	-0.65000
20.306	-0.65000
20.306	-0.65000
20.306	-0.65000
20.306	-0.65000
20.306	-0.65000
20.307	-0.65000

Time (s)	Tip potential during pulse (V)
20.307	-0.65000
20.307	-0.65000
20.307	-0.65000
20.307	-0.65000
20.307	-0.65000
20.307	-0.65000
20.307	-0.65000
20.308	-0.65000
20.308	-0.65000
20.308	-0.65000
20.308	-0.65000
20.308	-0.65000
20.308	-0.65000
20.308	-0.65000
20.309	-0.65000
20.309	-0.65000
20.309	-0.65000
20.309	-0.65000
20.309	-0.65000
20.309	-0.65000
20.309	-0.65000
20.310	-0.65000
20.310	-0.65000
20.310	-0.65000
20.310	-0.65000
20.310	-0.65000
20.310	-0.65000
20.310	-0.65000
20.310	-0.65000
20.311	-0.65000
20.311	-0.65000
20.311	-0.65000
20.311	-0.65000
20.311	-0.65000
20.311	-0.65000
20.311	-0.65000
20.312	-0.65000
20.312	-0.65000
20.312	-0.65000
20.312	-0.65000

Time (s)	Tip potential during pulse (V)
20.312	-0.65000
20.312	-0.65000
20.312	-0.65000
20.313	-0.65000
20.313	-0.65000
20.313	-0.65000
20.313	-0.65000
20.313	-0.65000
20.313	-0.65000
20.313	-0.65000
20.313	-0.65000
20.314	-0.65000
20.314	-0.65000
20.314	-0.65000
20.314	-0.65000
20.314	-0.65000
20.314	-0.65000
20.314	-0.65000
20.315	-0.65000
20.315	-0.65000
20.315	-0.65000
20.315	-0.65000
20.315	-0.65000
20.315	-0.65000
20.315	-0.65000
20.316	-0.65000
20.316	-0.65000
20.316	-0.65000
20.316	-0.65000
20.316	-0.65000
20.316	-0.65000
20.316	-0.65000
20.316	-0.65000
20.317	-0.65000
20.317	-0.65000
20.317	-0.65000
20.317	-0.65000
20.317	-0.65000
20.317	-0.65000
20.317	-0.65000

Time (s)	Tip potential during pulse (V)
20.318	-0.65000
20.318	-0.65000
20.318	-0.65000
20.318	-0.65000
20.318	-0.65000
20.318	-0.65000
20.318	-0.65000
20.319	-0.65000
20.319	-0.65000
20.319	-0.65000
20.319	-0.65000
20.319	-0.65000
20.319	-0.65000
20.319	-0.65000
20.320	-0.65000
20.320	-0.65000
20.320	-0.65000
20.320	-0.65000
20.320	-0.65000
20.320	-0.65000
20.320	-0.65000
20.321	-0.65000
20.321	-0.65000
20.321	-0.65000
20.321	-0.65000
20.321	-0.65000
20.321	-0.65000
20.321	-0.65000
20.321	-0.65000
20.322	-0.65000
20.322	-0.65000
20.322	-0.65000
20.322	-0.65000
20.322	-0.65000
20.322	-0.65000
20.322	-0.65000
20.323	-0.65000
20.323	-0.65000
20.323	-0.65000
20.323	-0.65000

Time (s)	Tip potential during pulse (V)
20.323	-0.65000
20.323	-0.65000
20.323	-0.65000
20.324	-0.65000
20.324	-0.65000
20.324	-0.65000
20.324	-0.65000
20.324	-0.65000
20.324	-0.65000
20.324	-0.65000
20.324	-0.65000
20.325	-0.65000
20.325	-0.65000
20.325	-0.65000
20.325	-0.65000
20.325	-0.65000
20.325	-0.65000
20.325	-0.65000
20.326	-0.65000
20.326	-0.65000
20.326	-0.65000
20.326	-0.65000
20.326	-0.65000
20.326	-0.65000
20.326	-0.65000
20.327	-0.65000
20.327	-0.65000
20.327	-0.65000
20.327	-0.65000
20.327	-0.65000
20.327	-0.65000
20.327	-0.65000
20.327	-0.65000
20.328	-0.65000
20.328	-0.65000
20.328	-0.65000
20.328	-0.65000
20.328	-0.65000
20.328	-0.65000
20.328	-0.65000

Time (s)	Tip potential during pulse (V)
20.329	-0.65000
20.329	-0.65000
20.329	-0.65000
20.329	-0.65000
20.329	-0.65000
20.329	-0.65000
20.329	-0.65000
20.330	-0.65000
20.330	-0.65000
20.330	-0.65000
20.330	-0.65000
20.330	-0.65000
20.330	-0.65000
20.330	-0.65000
20.330	-0.65000
20.331	-0.65000
20.331	-0.65000
20.331	-0.65000
20.331	-0.65000
20.331	-0.65000
20.331	-0.65000
20.331	-0.65000
20.332	-0.65000
20.332	-0.65000
20.332	-0.65000
20.332	-0.65000
20.332	-0.65000
20.332	-0.65000
20.332	-0.65000
20.333	-0.65000
20.333	-0.65000
20.333	-0.65000
20.333	-0.65000
20.333	-0.65000
20.333	-0.65000
20.333	-0.65000
20.333	-0.65000
20.334	-0.65000
20.334	-0.65000
20.334	-0.65000

Time (s)	Tip potential during pulse (V)
20.334	-0.65000
20.334	-0.65000
20.334	-0.65000
20.334	-0.65000
20.335	-0.65000
20.335	-0.65000
20.335	-0.65000
20.335	-0.65000
20.335	-0.65000
20.335	-0.65000
20.335	-0.65000
20.336	-0.65000
20.336	-0.65000
20.336	-0.65000
20.336	-0.65000
20.336	-0.65000
20.336	-0.65000
20.336	-0.65000
20.336	-0.65000
20.336	-0.65000
20.337	-0.65000
20.337	-0.65000
20.337	-0.65000
20.337	-0.65000
20.337	-0.65000
20.337	-0.65000
20.337	-0.65000
20.338	-0.65000
20.338	-0.65000
20.338	-0.65000
20.338	-0.65000
20.338	-0.65000
20.338	-0.65000
20.338	-0.65000
20.338	-0.65000
20.339	-0.65000
20.339	-0.65000
20.339	-0.65000
20.339	-0.65000
20.339	-0.65000

Time (s)	Tip potential during pulse (V)
20.339	-0.65000
20.339	-0.65000
20.340	-0.65000
20.340	-0.65000
20.340	-0.65000
20.340	-0.65000
20.340	-0.65000
20.340	-0.65000
20.340	-0.65000
20.341	-0.65000
20.341	-0.65000
20.341	-0.65000
20.341	-0.65000
20.341	-0.65000
20.341	-0.65000
20.341	-0.65000
20.341	-0.65000
20.342	-0.65000
20.342	-0.65000
20.342	-0.65000
20.342	-0.65000
20.342	-0.65000
20.342	-0.65000
20.342	-0.65000
20.343	-0.65000
20.343	-0.65000
20.343	-0.65000
20.343	-0.65000
20.343	-0.65000
20.343	-0.65000
20.343	-0.65000
20.344	-0.65000
20.344	-0.65000
20.344	-0.65000
20.344	-0.65000
20.344	-0.65000
20.344	-0.65000
20.344	-0.65000
20.345	-0.65000
20.345	-0.65000

Time (s)	Tip potential during pulse (V)
20.345	-0.65000
20.345	-0.65000
20.345	-0.65000
20.345	-0.65000
20.345	-0.65000
20.346	-0.65000
20.346	-0.65000
20.346	-0.65000
20.346	-0.65000
20.346	-0.65000
20.346	-0.65000
20.346	-0.65000
20.346	-0.65000
20.347	-0.65000
20.347	-0.65000
20.347	-0.65000
20.347	-0.65000
20.347	-0.65000
20.347	-0.65000
20.347	-0.65000
20.348	-0.65000
20.348	-0.65000
20.348	-0.65000
20.348	-0.65000
20.348	-0.65000
20.348	-0.65000
20.348	-0.65000
20.349	-0.65000
20.349	-0.65000
20.349	-0.65000
20.349	-0.65000
20.349	-0.65000
20.349	-0.65000
20.349	-0.65000
20.349	-0.65000
20.350	-0.65000
20.350	-0.65000
20.350	-0.65000
20.350	-0.65000
20.350	-0.65000

Time (s)	Tip potential during pulse (V)
20.350	-0.65000
20.350	-0.65000
20.351	-0.65000
20.351	-0.65000
20.351	-0.65000
20.351	-0.65000
20.351	-0.65000
20.351	-0.65000
20.351	-0.65000
20.352	-0.65000
20.352	-0.65000
20.352	-0.65000
20.352	-0.65000
20.352	-0.65000
20.352	-0.65000
20.352	-0.65000
20.352	-0.65000
20.353	-0.65000
20.353	-0.65000
20.353	-0.65000
20.353	-0.65000
20.353	-0.65000
20.353	-0.65000
20.353	-0.65000
20.354	-0.65000
20.354	-0.65000
20.354	-0.65000
20.354	-0.65000
20.354	-0.65000
20.354	-0.65000
20.354	-0.65000
20.355	-0.65000
20.355	-0.65000
20.355	-0.65000
20.355	-0.65000
20.355	-0.65000
20.355	-0.65000
20.355	-0.65000
20.355	-0.65000
20.356	-0.65000

Time (s)	Tip potential during pulse (V)
20.356	-0.65000
20.356	-0.65000
20.356	-0.65000
20.356	-0.65000
20.356	-0.65000
20.356	-0.65000
20.357	-0.65000
20.357	-0.65000
20.357	-0.65000
20.357	-0.65000
20.357	-0.65000
20.357	-0.65000
20.357	-0.65000
20.358	-0.65000
20.358	-0.65000
20.358	-0.65000
20.358	-0.65000
20.358	-0.65000
20.358	-0.65000
20.358	-0.65000
20.358	-0.65000
20.359	-0.65000
20.359	-0.65000
20.359	-0.65000
20.359	-0.65000
20.359	-0.65000
20.359	-0.65000
20.359	-0.65000
20.360	-0.65000
20.360	-0.65000
20.360	-0.65000
20.360	-0.65000
20.360	-0.65000
20.360	-0.65000
20.360	-0.65000
20.361	-0.65000
20.361	-0.65000
20.361	-0.65000
20.361	-0.65000
20.361	-0.65000

Time (s)	Tip potential during pulse (V)
20.361	-0.65000
20.361	-0.65000
20.361	-0.65000
20.362	-0.65000
20.362	-0.65000
20.362	-0.65000
20.362	-0.65000
20.362	-0.65000
20.362	-0.65000
20.362	-0.65000
20.363	-0.65000
20.363	-0.65000
20.363	-0.65000
20.363	-0.65000
20.363	-0.65000
20.363	-0.65000
20.363	-0.65000
20.364	-0.65000
20.364	-0.65000
20.364	-0.65000
20.364	-0.65000
20.364	-0.65000
20.364	-0.65000
20.364	-0.65000
20.364	-0.65000
20.365	-0.65000
20.365	-0.65000
20.365	-0.65000
20.365	-0.65000
20.365	-0.65000
20.365	-0.65000
20.365	-0.65000
20.366	-0.65000
20.366	-0.65000
20.366	-0.65000
20.366	-0.65000
20.366	-0.65000
20.366	-0.65000
20.366	-0.65000
20.367	-0.65000

Time (s)	Tip potential during pulse (V)
20.367	-0.65000
20.367	-0.65000
20.367	-0.65000
20.367	-0.65000
20.367	-0.65000
20.367	-0.65000
20.367	-0.65000
20.368	-0.65000
20.368	-0.65000
20.368	-0.65000
20.368	-0.65000
20.368	-0.65000
20.368	-0.65000
20.368	-0.65000
20.369	-0.65000
20.369	-0.65000
20.369	-0.65000
20.369	-0.65000
20.369	-0.65000
20.369	-0.65000
20.369	-0.65000
20.370	-0.65000
20.370	-0.65000
20.370	-0.65000
20.370	-0.65000
20.370	-0.65000
20.370	-0.65000
20.370	-0.65000
20.370	-0.65000
20.371	-0.65000
20.371	-0.65000
20.371	-0.65000
20.371	-0.65000
20.371	-0.65000
20.371	-0.65000
20.371	-0.65000
20.372	-0.65000
20.372	-0.65000
20.372	-0.65000
20.372	-0.65000

Time (s)	Tip potential during pulse (V)
20.372	-0.65000
20.372	-0.65000
20.372	-0.65000
20.373	-0.65000
20.373	-0.65000
20.373	-0.65000
20.373	-0.65000
20.373	-0.65000
20.373	-0.65000
20.373	-0.65000
20.373	-0.65000
20.374	-0.65000
20.374	-0.65000
20.374	-0.65000
20.374	-0.65000
20.374	-0.65000
20.374	-0.65000
20.374	-0.65000
20.375	-0.65000
20.375	-0.65000
20.375	-0.65000
20.375	-0.65000
20.375	-0.65000
20.375	-0.65000
20.375	-0.65000
20.376	-0.65000
20.376	-0.65000
20.376	-0.65000
20.376	-0.65000
20.376	-0.65000
20.376	-0.65000
20.376	-0.65000
20.376	-0.65000
20.377	-0.65000
20.377	-0.65000
20.377	-0.65000
20.377	-0.65000
20.377	-0.65000
20.377	-0.65000
20.377	-0.65000

Time (s)	Tip potential during pulse (V)
20.378	-0.65000
20.378	-0.65000
20.378	-0.65000
20.378	-0.65000
20.378	-0.65000
20.378	-0.65000
20.378	-0.65000
20.379	-0.65000
20.379	-0.65000
20.379	-0.65000
20.379	-0.65000
20.379	-0.65000
20.379	-0.65000
20.379	-0.65000
20.379	-0.65000
20.380	-0.65000
20.380	-0.65000
20.380	-0.65000
20.380	-0.65000
20.380	-0.65000
20.380	-0.65000
20.380	-0.65000
20.381	-0.65000
20.381	-0.65000
20.381	-0.65000
20.381	-0.65000
20.381	-0.65000
20.381	-0.65000
20.381	-0.65000
20.382	-0.65000
20.382	-0.65000
20.382	-0.65000
20.382	-0.65000
20.382	-0.65000
20.382	-0.65000
20.382	-0.65000
20.382	-0.65000
20.383	-0.65000
20.383	-0.65000
20.383	-0.65000

Time (s)	Tip potential during pulse (V)
20.383	-0.65000
20.383	-0.65000
20.383	-0.65000
20.383	-0.65000
20.384	-0.65000
20.384	-0.65000
20.384	-0.65000
20.384	-0.65000
20.384	-0.65000
20.384	-0.65000
20.384	-0.65000
20.385	-0.65000
20.385	-0.65000
20.385	-0.65000
20.385	-0.65000
20.385	-0.65000
20.385	-0.65000
20.385	-0.65000
20.385	-0.65000
20.386	-0.65000
20.386	-0.65000
20.386	-0.65000
20.386	-0.65000
20.386	-0.65000
20.386	-0.65000
20.386	-0.65000
20.386	-0.65000
20.387	-0.65000
20.387	-0.65000
20.387	-0.65000
20.387	-0.65000
20.387	-0.65000
20.387	-0.65000
20.387	-0.65000
20.388	-0.65000
20.388	-0.65000
20.388	-0.65000
20.388	-0.65000
20.388	-0.65000
20.388	-0.65000

Time (s)	Tip potential during pulse (V)
20.388	-0.65000
20.389	-0.65000
20.389	-0.65000
20.389	-0.65000
20.389	-0.65000
20.389	-0.65000
20.389	-0.65000
20.389	-0.65000
20.389	-0.65000
20.390	-0.65000
20.390	-0.65000
20.390	-0.65000
20.390	-0.65000
20.390	-0.65000
20.390	-0.65000
20.390	-0.65000
20.391	-0.65000
20.391	-0.65000
20.391	-0.65000
20.391	-0.65000
20.391	-0.65000
20.391	-0.65000
20.391	-0.65000
20.391	-0.65000
20.391	-0.65000
20.392	-0.65000
20.392	-0.65000
20.392	-0.65000
20.392	-0.65000
20.392	-0.65000
20.392	-0.65000
20.392	-0.65000
20.393	-0.65000
20.393	-0.65000
20.393	-0.65000
20.393	-0.65000
20.393	-0.65000
20.393	-0.65000
20.393	-0.65000
20.394	-0.65000

Time (s)	Tip potential during pulse (V)
20.394	-0.65000
20.394	-0.65000
20.394	-0.65000
20.394	-0.65000
20.394	-0.65000
20.394	-0.65000
20.395	-0.65000
20.395	-0.65000
20.395	-0.65000
20.395	-0.65000
20.395	-0.65000
20.395	-0.65000
20.395	-0.65000
20.396	-0.65000
20.396	-0.65000
20.396	-0.65000
20.396	-0.65000
20.396	-0.65000
20.396	-0.65000
20.396	-0.65000
20.396	-0.65000
20.397	-0.65000
20.397	-0.65000
20.397	-0.65000
20.397	-0.65000
20.397	-0.65000
20.397	-0.65000
20.397	-0.65000
20.398	-0.65000
20.398	-0.65000
20.398	-0.65000
20.398	-0.65000
20.398	-0.65000
20.398	-0.65000
20.398	-0.65000
20.399	-0.65000
20.399	-0.65000
20.399	-0.65000
20.399	-0.65000
20.399	-0.65000

Time (s)	Tip potential during pulse (V)
20.399	-0.65000
20.399	-0.65000
20.399	-0.65000
20.399	-0.65000
20.399	-0.65000
20.400	-0.65000
20.400	-0.65000
20.400	-0.65000
20.400	-0.65000
20.400	-0.65000
20.400	-0.65000
20.400	-0.65000
20.401	-0.65000
20.401	-0.65000
20.401	-0.65000
20.401	-0.65000
20.401	-0.65000
20.401	-0.65000
20.401	-0.65000
20.402	-0.65000
20.402	-0.65000
20.402	-0.65000
20.402	-0.65000
20.402	-0.65000
20.402	-0.65000
20.402	-0.65000
20.402	-0.65000
20.403	-0.65000
20.403	-0.65000
20.403	-0.65000
20.403	-0.65000
20.403	-0.65000
20.403	-0.65000
20.403	-0.65000
20.404	-0.65000
20.404	-0.65000
20.404	-0.65000
20.404	-0.65000
20.404	-0.65000
20.404	-0.65000

Time (s)	Tip potential during pulse (V)
20.404	-0.65000
20.405	-0.65000
20.405	-0.65000
20.405	-0.65000
20.405	-0.65000
20.405	-0.65000
20.405	-0.65000
20.406	-0.65000
20.406	-0.65000
20.406	-0.65000
20.406	-0.65000
20.406	-0.65000
20.406	-0.65000
20.407	-0.65000
20.407	-0.65000
20.407	-0.65000
20.407	-0.65000
20.407	-0.65000
20.407	-0.65000
20.407	-0.65000
20.407	-0.65000
20.408	-0.65000
20.408	-0.65000
20.408	-0.65000
20.408	-0.65000
20.408	-0.65000
20.408	-0.65000
20.408	-0.65000
20.408	-0.65000
20.408	-0.65000
20.409	-0.65000
20.409	-0.65000
20.409	-0.65000
20.409	-0.65000
20.409	-0.65000
20.409	-0.65000
20.409	-0.65000
20.410	-0.65000
20.410	-0.65000
20.410	-0.65000

Time (s)	Tip potential during pulse (V)
20.410	-0.65000
20.410	-0.65000
20.410	-0.65000
20.410	-0.65000
20.410	-0.65000
20.411	-0.65000
20.411	-0.65000
20.411	-0.65000
20.411	-0.65000
20.411	-0.65000
20.411	-0.65000
20.411	-0.65000
20.412	-0.65000
20.412	-0.65000
20.412	-0.65000
20.412	-0.65000
20.412	-0.65000
20.412	-0.65000
20.412	-0.65000
20.413	-0.65000
20.413	-0.65000
20.413	-0.65000
20.413	-0.65000
20.413	-0.65000
20.413	-0.65000
20.413	-0.65000
20.413	-0.65000
20.414	-0.65000
20.414	-0.65000
20.414	-0.65000
20.414	-0.65000
20.414	-0.65000
20.414	-0.65000
20.414	-0.65000
20.415	-0.65000
20.415	-0.65000
20.415	-0.65000
20.415	-0.65000
20.415	-0.65000
20.415	-0.65000

Time (s)	Tip potential during pulse (V)
20.415	-0.65000
20.416	-0.65000
20.416	-0.65000
20.416	-0.65000
20.416	-0.65000
20.416	-0.65000
20.416	-0.65000
20.416	-0.65000
20.416	-0.65000
20.417	-0.65000
20.417	-0.65000
20.417	-0.65000
20.417	-0.65000
20.417	-0.65000
20.417	-0.65000
20.417	-0.65000
20.418	-0.65000
20.418	-0.65000
20.418	-0.65000
20.418	-0.65000
20.418	-0.65000
20.418	-0.65000
20.418	-0.65000
20.419	-0.65000
20.419	-0.65000
20.419	-0.65000
20.419	-0.65000
20.419	-0.65000
20.419	-0.65000
20.419	-0.65000
20.420	-0.65000
20.420	-0.65000
20.420	-0.65000
20.420	-0.65000
20.420	-0.65000
20.420	-0.65000
20.420	-0.65000
20.420	-0.65000
20.420	-0.65000
20.421	-0.65000

Time (s)	Tip potential during pulse (V)
20.421	-0.65000
20.421	-0.65000
20.421	-0.65000
20.421	-0.65000
20.421	-0.65000
20.421	-0.65000
20.421	-0.65000
20.422	-0.65000
20.422	-0.65000
20.422	-0.65000
20.422	-0.65000
20.422	-0.65000
20.422	-0.65000
20.422	-0.65000
20.423	-0.65000
20.423	-0.65000
20.423	-0.65000
20.423	-0.65000
20.423	-0.65000
20.423	-0.65000
20.423	-0.65000
20.424	-0.65000
20.424	-0.65000
20.424	-0.65000
20.424	-0.65000
20.424	-0.65000
20.424	-0.65000
20.424	-0.65000
20.424	-0.65000
20.425	-0.65000
20.425	-0.65000
20.425	-0.65000
20.425	-0.65000
20.425	-0.65000
20.425	-0.65000
20.425	-0.65000
20.426	-0.65000
20.426	-0.65000
20.426	-0.65000
20.426	-0.65000

Time (s)	Tip potential during pulse (V)
20.426	-0.65000
20.426	-0.65000
20.426	-0.65000
20.427	-0.65000
20.427	-0.65000
20.427	-0.65000
20.427	-0.65000
20.427	-0.65000
20.427	-0.65000
20.427	-0.65000
20.427	-0.65000
20.428	-0.65000
20.428	-0.65000
20.428	-0.65000
20.428	-0.65000
20.428	-0.65000
20.428	-0.65000
20.428	-0.65000
20.429	-0.65000
20.429	-0.65000
20.429	-0.65000
20.429	-0.65000
20.429	-0.65000
20.429	-0.65000
20.429	-0.65000
20.430	-0.65000
20.430	-0.65000
20.430	-0.65000
20.430	-0.65000
20.430	-0.65000
20.430	-0.65000
20.430	-0.65000
20.430	-0.65000
20.431	-0.65000
20.431	-0.65000
20.431	-0.65000
20.431	-0.65000
20.431	-0.65000
20.431	-0.65000
20.431	-0.65000

Time (s)	Tip potential during pulse (V)
20.432	-0.65000
20.432	-0.65000
20.432	-0.65000
20.432	-0.65000
20.432	-0.65000
20.432	-0.65000
20.432	-0.65000
20.433	-0.65000
20.433	-0.65000
20.433	-0.65000
20.433	-0.65000
20.433	-0.65000
20.433	-0.65000
20.433	-0.65000
20.434	-0.65000
20.434	-0.65000
20.434	-0.65000
20.434	-0.65000
20.434	-0.65000
20.434	-0.65000
20.434	-0.65000
20.435	-0.65000
20.435	-0.65000
20.435	-0.65000
20.435	-0.65000
20.435	-0.65000
20.435	-0.65000
20.435	-0.65000
20.435	-0.65000
20.436	-0.65000
20.436	-0.65000
20.436	-0.65000
20.436	-0.65000
20.436	-0.65000
20.436	-0.65000
20.436	-0.65000
20.437	-0.65000
20.437	-0.65000
20.437	-0.65000
20.437	-0.65000

Time (s)	Tip potential during pulse (V)
20.437	-0.65000
20.437	-0.65000
20.437	-0.65000
20.438	-0.65000
20.438	-0.65000
20.438	-0.65000
20.438	-0.65000
20.438	-0.65000
20.438	-0.65000
20.438	-0.65000
20.438	-0.65000
20.439	-0.65000
20.439	-0.65000
20.439	-0.65000
20.439	-0.65000
20.439	-0.65000
20.439	-0.65000
20.439	-0.65000
20.440	-0.65000
20.440	-0.65000
20.440	-0.65000
20.440	-0.65000
20.440	-0.65000
20.440	-0.65000
20.440	-0.65000
20.441	-0.65000
20.441	-0.65000
20.441	-0.65000
20.441	-0.65000
20.441	-0.65000
20.441	-0.65000
20.441	-0.65000
20.441	-0.65000
20.442	-0.65000
20.442	-0.65000
20.442	-0.65000
20.442	-0.65000
20.442	-0.65000
20.442	-0.65000
20.442	-0.65000

Time (s)	Tip potential during pulse (V)
20.443	-0.65000
20.443	-0.65000
20.443	-0.65000
20.443	-0.65000
20.443	-0.65000
20.443	-0.65000
20.443	-0.65000
20.444	-0.65000
20.444	-0.65000
20.444	-0.65000
20.444	-0.65000
20.444	-0.65000
20.444	-0.65000
20.444	-0.65000
20.444	-0.65000
20.445	-0.65000
20.445	-0.65000
20.445	-0.65000
20.445	-0.65000
20.445	-0.65000
20.445	-0.65000
20.445	-0.65000
20.446	-0.65000
20.446	-0.65000
20.446	-0.65000
20.446	-0.65000
20.446	-0.65000
20.446	-0.65000
20.446	-0.65000
20.447	-0.65000
20.447	-0.65000
20.447	-0.65000
20.447	-0.65000
20.447	-0.65000
20.447	-0.65000
20.447	-0.65000
20.447	-0.65000
20.448	-0.65000
20.448	-0.65000
20.448	-0.65000

Time (s)	Tip potential during pulse (V)
20.448	-0.65000
20.448	-0.65000
20.448	-0.65000
20.448	-0.65000
20.449	-0.65000
20.449	-0.65000
20.449	-0.65000
20.449	-0.65000
20.449	-0.65000
20.449	-0.65000
20.449	-0.65000
20.450	-0.65000
20.450	-0.65000
20.450	-0.65000
20.450	-0.65000
20.450	-0.65000
20.450	-0.65000
20.450	-0.65000
20.450	-0.65000
20.451	-0.65000
20.451	-0.65000
20.451	-0.65000
20.451	-0.65000
20.451	-0.65000
20.451	-0.65000
20.451	-0.65000
20.452	-0.65000
20.452	-0.65000
20.452	-0.65000
20.452	-0.65000
20.452	-0.65000
20.452	-0.65000
20.452	-0.65000
20.453	-0.65000
20.453	-0.65000
20.453	-0.65000
20.453	-0.65000
20.453	-0.65000
20.453	-0.65000
20.453	-0.65000

Time (s)	Tip potential during pulse (V)
20.453	-0.65000
20.454	-0.65000
20.454	-0.65000
20.454	-0.65000
20.454	-0.65000
20.454	-0.65000
20.454	-0.65000
20.454	-0.65000
20.455	-0.65000
20.455	-0.65000
20.455	-0.65000
20.455	-0.65000
20.455	-0.65000
20.455	-0.65000
20.455	-0.65000
20.456	-0.65000
20.456	-0.65000
20.456	-0.65000
20.456	-0.65000
20.456	-0.65000
20.456	-0.65000
20.456	-0.65000
20.456	-0.65000
20.457	-0.65000
20.457	-0.65000
20.457	-0.65000
20.457	-0.65000
20.457	-0.65000
20.457	-0.65000
20.457	-0.65000
20.458	-0.65000
20.458	-0.65000
20.458	-0.65000
20.458	-0.65000
20.458	-0.65000
20.458	-0.65000
20.458	-0.65000
20.459	-0.65000
20.459	-0.65000
20.459	-0.65000

Time (s)	Tip potential during pulse (V)
20.459	-0.65000
20.459	-0.65000
20.459	-0.65000
20.459	-0.65000
20.459	-0.65000
20.460	-0.65000
20.460	-0.65000
20.460	-0.65000
20.460	-0.65000
20.460	-0.65000
20.460	-0.65000
20.460	-0.65000
20.461	-0.65000
20.461	-0.65000
20.461	-0.65000
20.461	-0.65000
20.461	-0.65000
20.461	-0.65000
20.461	-0.65000
20.462	-0.65000
20.462	-0.65000
20.462	-0.65000
20.462	-0.65000
20.462	-0.65000
20.462	-0.65000
20.462	-0.65000
20.462	-0.65000
20.463	-0.65000
20.463	-0.65000
20.463	-0.65000
20.463	-0.65000
20.463	-0.65000
20.463	-0.65000
20.463	-0.65000
20.464	-0.65000
20.464	-0.65000
20.464	-0.65000
20.464	-0.65000
20.464	-0.65000
20.464	-0.65000

Time (s)	Tip potential during pulse (V)
20.464	-0.65000
20.465	-0.65000
20.465	-0.65000
20.465	-0.65000
20.465	-0.65000
20.465	-0.65000
20.465	-0.65000
20.465	-0.65000
20.465	-0.65000
20.466	-0.65000
20.466	-0.65000
20.466	-0.65000
20.466	-0.65000
20.466	-0.65000
20.466	-0.65000
20.466	-0.65000
20.467	-0.65000
20.467	-0.65000
20.467	-0.65000
20.467	-0.65000
20.467	-0.65000
20.467	-0.65000
20.467	-0.65000
20.468	-0.65000
20.468	-0.65000
20.468	-0.65000
20.468	-0.65000
20.468	-0.65000
20.468	-0.65000
20.468	-0.65000
20.468	-0.65000
20.469	-0.65000
20.469	-0.65000
20.469	-0.65000
20.469	-0.65000
20.469	-0.65000
20.469	-0.65000
20.469	-0.65000
20.470	-0.65000
20.470	-0.65000

Time (s)	Tip potential during pulse (V)
20.470	-0.65000
20.470	-0.65000
20.470	-0.65000
20.470	-0.65000
20.470	-0.65000
20.471	-0.65000
20.471	-0.65000
20.471	-0.65000
20.471	-0.65000
20.471	-0.65000
20.471	-0.65000
20.471	-0.65000
20.471	-0.65000
20.472	-0.65000
20.472	-0.65000
20.472	-0.65000
20.472	-0.65000
20.472	-0.65000
20.472	-0.65000
20.472	-0.65000
20.473	-0.65000
20.473	-0.65000
20.473	-0.65000
20.473	-0.65000
20.473	-0.65000
20.473	-0.65000
20.473	-0.65000
20.474	-0.65000
20.474	-0.65000
20.474	-0.65000
20.474	-0.65000
20.474	-0.65000
20.474	-0.65000
20.474	-0.65000
20.474	-0.65000
20.475	-0.65000
20.475	-0.65000
20.475	-0.65000
20.475	-0.65000
20.475	-0.65000

Time (s)	Tip potential during pulse (V)
20.475	-0.65000
20.475	-0.65000
20.476	-0.65000
20.476	-0.65000
20.476	-0.65000
20.476	-0.65000
20.476	-0.65000
20.476	-0.65000
20.476	-0.65000
20.476	-0.65000
20.476	-0.65000
20.476	-0.65000
20.477	-0.65000
20.477	-0.65000
20.477	-0.65000
20.477	-0.65000
20.477	-0.65000
20.477	-0.65000
20.477	-0.65000
20.478	-0.65000
20.478	-0.65000
20.478	-0.65000
20.478	-0.65000
20.478	-0.65000
20.478	-0.65000
20.478	-0.65000
20.478	-0.65000
20.479	-0.65000
20.479	-0.65000
20.479	-0.65000
20.479	-0.65000
20.479	-0.65000
20.479	-0.65000
20.479	-0.65000
20.480	-0.65000
20.480	-0.65000
20.480	-0.65000
20.480	-0.65000
20.480	-0.65000
20.480	-0.65000

Time (s)	Tip potential during pulse (V)
20.480	-0.65000
20.481	-0.65000
20.481	-0.65000
20.481	-0.65000
20.481	-0.65000
20.481	-0.65000
20.481	-0.65000
20.481	-0.65000
20.481	-0.65000
20.482	-0.65000
20.482	-0.65000
20.482	-0.65000
20.482	-0.65000
20.482	-0.65000
20.482	-0.65000
20.482	-0.65000
20.483	-0.65000
20.483	-0.65000
20.483	-0.65000
20.483	-0.65000
20.483	-0.65000
20.483	-0.65000
20.483	-0.65000
20.484	-0.65000
20.484	-0.65000
20.484	-0.65000
20.484	-0.65000
20.484	-0.65000
20.484	-0.65000
20.484	-0.65000
20.484	-0.65000
20.485	-0.65000
20.485	-0.65000
20.485	-0.65000
20.485	-0.65000
20.485	-0.65000
20.485	-0.65000
20.485	-0.65000
20.486	-0.65000
20.486	-0.65000

Time (s)	Tip potential during pulse (V)
20.486	-0.65000
20.486	-0.65000
20.486	-0.65000
20.486	-0.65000
20.486	-0.65000
20.487	-0.65000
20.487	-0.65000
20.487	-0.65000
20.487	-0.65000
20.487	-0.65000
20.487	-0.65000
20.487	-0.65000
20.487	-0.65000
20.488	-0.65000
20.488	-0.65000
20.488	-0.65000
20.488	-0.65000
20.488	-0.65000
20.488	-0.65000
20.488	-0.65000
20.489	-0.65000
20.489	-0.65000
20.489	-0.65000
20.489	-0.65000
20.489	-0.65000
20.489	-0.65000
20.489	-0.65000
20.490	-0.65000
20.490	-0.65000
20.490	-0.65000
20.490	-0.65000
20.490	-0.65000
20.490	-0.65000
20.490	-0.65000
20.490	-0.65000
20.490	-0.65000
20.491	-0.65000
20.491	-0.65000
20.491	-0.65000
20.491	-0.65000

Time (s)	Tip potential during pulse (V)
20.491	-0.65000
20.491	-0.65000
20.491	-0.65000
20.492	-0.65000
20.492	-0.65000
20.492	-0.65000
20.492	-0.65000
20.492	-0.65000
20.492	-0.65000
20.492	-0.65000
20.492	-0.65000
20.493	-0.65000
20.493	-0.65000
20.493	-0.65000
20.493	-0.65000
20.493	-0.65000
20.493	-0.65000
20.493	-0.65000
20.494	-0.65000
20.494	-0.65000
20.494	-0.65000
20.494	-0.65000
20.494	-0.65000
20.494	-0.65000
20.494	-0.65000
20.495	-0.65000
20.495	-0.65000
20.495	-0.65000
20.495	-0.65000
20.495	-0.65000
20.495	-0.65000
20.495	-0.65000
20.495	-0.65000
20.496	-0.65000
20.496	-0.65000
20.496	-0.65000
20.496	-0.65000
20.496	-0.65000
20.496	-0.65000
20.496	-0.65000

Time (s)	Tip potential during pulse (V)
20.496	-0.65000
20.497	-0.65000
20.497	-0.65000
20.497	-0.65000
20.497	-0.65000
20.497	-0.65000
20.497	-0.65000
20.497	-0.65000
20.497	-0.65000
20.498	-0.65000
20.498	-0.65000
20.498	-0.65000
20.498	-0.65000
20.498	-0.65000
20.498	-0.65000
20.498	-0.65000
20.499	-0.65000
20.499	-0.65000
20.499	-0.65000
20.499	-0.65000
20.499	-0.65000
20.499	-0.65000
20.499	-0.65000
20.500	-0.65000
20.500	-0.65000
20.500	-0.65000
20.500	-0.65000
20.500	-0.65000
20.500	-0.65000
20.500	-0.65000
20.500	-0.65000
20.501	-0.65000
20.501	-0.65000
20.501	-0.65000
20.501	-0.65000
20.501	-0.65000
20.501	-0.65000
20.501	-0.65000
20.502	-0.65000
20.502	-0.65000

Time (s)	Tip potential during pulse (V)
20.502	-0.65000
20.502	-0.65000
20.502	-0.65000
20.502	-0.65000
20.502	-0.65000
20.503	-0.65000
20.503	-0.65000
20.503	-0.65000
20.503	-0.65000
20.503	-0.65000
20.503	-0.65000
20.503	-0.65000
20.503	-0.65000
20.504	-0.65000
20.504	-0.65000
20.504	-0.65000
20.504	-0.65000
20.504	-0.65000
20.504	-0.65000
20.504	-0.65000
20.505	-0.65000
20.505	-0.65000
20.505	-0.65000
20.505	-0.65000
20.505	-0.65000
20.505	-0.65000
20.505	-0.65000
20.506	-0.65000
20.506	-0.65000
20.506	-0.65000
20.506	-0.65000
20.506	-0.65000
20.506	-0.65000
20.506	-0.65000
20.506	-0.65000
20.506	-0.65000
20.507	-0.65000
20.507	-0.65000
20.507	-0.65000
20.507	-0.65000

Time (s)	Tip potential during pulse (V)
20.507	-0.65000
20.507	-0.65000
20.507	-0.65000
20.508	-0.65000
20.508	-0.65000
20.508	-0.65000
20.508	-0.65000
20.508	-0.65000
20.508	-0.65000
20.508	-0.65000
20.508	-0.65000
20.509	-0.65000
20.509	-0.65000
20.509	-0.65000
20.509	-0.65000
20.509	-0.65000
20.509	-0.65000
20.509	-0.65000
20.510	-0.65000
20.510	-0.65000
20.510	-0.65000
20.510	-0.65000
20.510	-0.65000
20.510	-0.65000
20.510	-0.65000
20.511	-0.65000
20.511	-0.65000
20.511	-0.65000
20.511	-0.65000
20.511	-0.65000
20.511	-0.65000
20.511	-0.65000
20.511	-0.65000
20.512	-0.65000
20.512	-0.65000
20.512	-0.65000
20.512	-0.65000
20.512	-0.65000
20.512	-0.65000
20.512	-0.65000

Time (s)	Tip potential during pulse (V)
20.513	-0.65000
20.513	-0.65000
20.513	-0.65000
20.513	-0.65000
20.513	-0.65000
20.513	-0.65000
20.513	-0.65000
20.514	-0.65000
20.514	-0.65000
20.514	-0.65000
20.514	-0.65000
20.514	-0.65000
20.514	-0.65000
20.514	-0.65000
20.514	-0.65000
20.515	-0.65000
20.515	-0.65000
20.515	-0.65000
20.515	-0.65000
20.515	-0.65000
20.515	-0.65000
20.515	-0.65000
20.516	-0.65000
20.516	-0.65000
20.516	-0.65000
20.516	-0.65000
20.516	-0.65000
20.516	-0.65000
20.516	-0.65000
20.517	-0.65000
20.517	-0.65000
20.517	-0.65000
20.517	-0.65000
20.517	-0.65000
20.517	-0.65000
20.517	-0.65000
20.517	-0.65000
20.518	-0.65000
20.518	-0.65000
20.518	-0.65000

Time (s)	Tip potential during pulse (V)
20.518	-0.65000
20.518	-0.65000
20.518	-0.65000
20.518	-0.65000
20.519	-0.65000
20.519	-0.65000
20.519	-0.65000
20.519	-0.65000
20.519	-0.65000
20.519	-0.65000
20.519	-0.65000
20.520	-0.65000
20.520	-0.65000
20.520	-0.65000
20.520	-0.65000
20.520	-0.65000
20.520	-0.65000
20.520	-0.65000
20.520	-0.65000
20.520	-0.65000
20.521	-0.65000
20.521	-0.65000
20.521	-0.65000
20.521	-0.65000
20.521	-0.65000
20.521	-0.65000
20.521	-0.65000
20.522	-0.65000
20.522	-0.65000
20.522	-0.65000
20.522	-0.65000
20.522	-0.65000
20.522	-0.65000
20.522	-0.65000
20.522	-0.65000
20.523	-0.65000
20.523	-0.65000
20.523	-0.65000
20.523	-0.65000
20.523	-0.65000

Time (s)	Tip potential during pulse (V)
20.523	-0.65000
20.523	-0.65000
20.524	-0.65000
20.524	-0.65000
20.524	-0.65000
20.524	-0.65000
20.524	-0.65000
20.524	-0.65000
20.524	-0.65000
20.525	-0.65000
20.525	-0.65000
20.525	-0.65000
20.525	-0.65000
20.525	-0.65000
20.525	-0.65000
20.525	-0.65000
20.525	-0.65000
20.526	-0.65000
20.526	-0.65000
20.526	-0.65000
20.526	-0.65000
20.526	-0.65000
20.526	-0.65000
20.526	-0.65000
20.527	-0.65000
20.527	-0.65000
20.527	-0.65000
20.527	-0.65000
20.527	-0.65000
20.527	-0.65000
20.527	-0.65000
20.528	-0.65000
20.528	-0.65000
20.528	-0.65000
20.528	-0.65000
20.528	-0.65000
20.528	-0.65000
20.528	-0.65000
20.528	-0.65000
20.529	-0.65000

Time (s)	Tip potential during pulse (V)
20.529	-0.65000
20.529	-0.65000
20.529	-0.65000
20.529	-0.65000
20.529	-0.65000
20.529	-0.65000
20.530	-0.65000
20.530	-0.65000
20.530	-0.65000
20.530	-0.65000
20.530	-0.65000
20.530	-0.65000
20.530	-0.65000
20.531	-0.65000
20.531	-0.65000
20.531	-0.65000
20.531	-0.65000
20.531	-0.65000
20.531	-0.65000
20.531	-0.65000
20.531	-0.65000
20.532	-0.65000
20.532	-0.65000
20.532	-0.65000
20.532	-0.65000
20.532	-0.65000
20.532	-0.65000
20.532	-0.65000
20.532	-0.65000
20.533	-0.65000
20.533	-0.65000
20.533	-0.65000
20.533	-0.65000
20.533	-0.65000
20.533	-0.65000
20.533	-0.65000
20.534	-0.65000
20.534	-0.65000
20.534	-0.65000
20.534	-0.65000

Time (s)	Tip potential during pulse (V)
20.534	-0.65000
20.534	-0.65000
20.534	-0.65000
20.535	-0.65000
20.535	-0.65000
20.535	-0.65000
20.535	-0.65000
20.535	-0.65000
20.535	-0.65000
20.535	-0.65000
20.535	-0.65000
20.535	-0.65000
20.535	-0.65000
20.536	-0.65000
20.536	-0.65000
20.536	-0.65000
20.536	-0.65000
20.536	-0.65000
20.536	-0.65000
20.536	-0.65000
20.537	-0.65000
20.537	-0.65000
20.537	-0.65000
20.537	-0.65000
20.537	-0.65000
20.537	-0.65000
20.537	-0.65000
20.538	-0.65000
20.538	-0.65000
20.538	-0.65000
20.538	-0.65000
20.538	-0.65000
20.538	-0.65000
20.538	-0.65000
20.538	-0.65000
20.538	-0.65000
20.539	-0.65000
20.539	-0.65000
20.539	-0.65000
20.539	-0.65000

Time (s)	Tip potential during pulse (V)
20.539	-0.65000
20.539	-0.65000
20.539	-0.65000
20.540	-0.65000
20.540	-0.65000
20.540	-0.65000
20.540	-0.65000
20.540	-0.65000
20.540	-0.65000
20.540	-0.65000
20.540	-0.65000
20.541	-0.65000
20.541	-0.65000
20.541	-0.65000
20.541	-0.65000
20.541	-0.65000
20.541	-0.65000
20.541	-0.65000
20.542	-0.65000
20.542	-0.65000
20.542	-0.65000
20.542	-0.65000
20.542	-0.65000
20.542	-0.65000
20.542	-0.65000
20.543	-0.65000
20.543	-0.65000
20.543	-0.65000
20.543	-0.65000
20.543	-0.65000
20.543	-0.65000
20.543	-0.65000
20.543	-0.65000
20.544	-0.65000
20.544	-0.65000
20.544	-0.65000
20.544	-0.65000
20.544	-0.65000
20.544	-0.65000
20.544	-0.65000

Time (s)	Tip potential during pulse (V)
20.545	-0.65000
20.545	-0.65000
20.545	-0.65000
20.545	-0.65000
20.545	-0.65000
20.545	-0.65000
20.545	-0.65000
20.546	-0.65000
20.546	-0.65000
20.546	-0.65000
20.546	-0.65000
20.546	-0.65000
20.546	-0.65000
20.546	-0.65000
20.546	-0.65000
20.547	-0.65000
20.547	-0.65000
20.547	-0.65000
20.547	-0.65000
20.547	-0.65000
20.547	-0.65000
20.547	-0.65000
20.548	-0.65000
20.548	-0.65000
20.548	-0.65000
20.548	-0.65000
20.548	-0.65000
20.548	-0.65000
20.548	-0.65000
20.549	-0.65000
20.549	-0.65000
20.549	-0.65000
20.549	-0.65000
20.549	-0.65000
20.549	-0.65000
20.549	-0.65000
20.549	-0.65000
20.550	-0.65000
20.550	-0.65000
20.550	-0.65000

Time (s)	Tip potential during pulse (V)
20.550	-0.65000
20.550	-0.65000
20.550	-0.65000
20.550	-0.65000
20.551	-0.65000
20.551	-0.65000
20.551	-0.65000
20.551	-0.65000
20.551	-0.65000
20.551	-0.65000
20.551	-0.65000
20.552	-0.65000
20.552	-0.65000
20.552	-0.65000
20.552	-0.65000
20.552	-0.65000
20.552	-0.65000
20.552	-0.65000
20.552	-0.65000
20.553	-0.65000
20.553	-0.65000
20.553	-0.65000
20.553	-0.65000
20.553	-0.65000
20.553	-0.65000
20.553	-0.65000
20.554	-0.65000
20.554	-0.65000
20.554	-0.65000
20.554	-0.65000
20.554	-0.65000
20.554	-0.65000
20.554	-0.65000
20.555	-0.65000
20.555	-0.65000
20.555	-0.65000
20.555	-0.65000
20.555	-0.65000
20.555	-0.65000
20.555	-0.65000

Time (s)	Tip potential during pulse (V)
20.555	-0.65000
20.556	-0.65000
20.556	-0.65000
20.556	-0.65000
20.556	-0.65000
20.556	-0.65000
20.556	-0.65000
20.556	-0.65000
20.557	-0.65000
20.557	-0.65000
20.557	-0.65000
20.557	-0.65000
20.557	-0.65000
20.557	-0.65000
20.557	-0.65000
20.558	-0.65000
20.558	-0.65000
20.558	-0.65000
20.558	-0.65000
20.558	-0.65000
20.558	-0.65000
20.558	-0.65000
20.559	-0.65000
20.559	-0.65000
20.559	-0.65000
20.559	-0.65000
20.559	-0.65000
20.559	-0.65000
20.559	-0.65000
20.560	-0.65000
20.560	-0.65000
20.560	-0.65000
20.560	-0.65000
20.560	-0.65000
20.560	-0.65000
20.560	-0.65000
20.560	-0.65000
20.561	-0.65000
20.561	-0.65000
20.561	-0.65000

Time (s)	Tip potential during pulse (V)
20.561	-0.65000
20.561	-0.65000
20.561	-0.65000
20.561	-0.65000
20.562	-0.65000
20.562	-0.65000
20.562	-0.65000
20.562	-0.65000
20.562	-0.65000
20.562	-0.65000
20.562	-0.65000
20.563	-0.65000
20.563	-0.65000
20.563	-0.65000
20.563	-0.65000
20.563	-0.65000
20.563	-0.65000
20.563	-0.65000
20.563	-0.65000
20.564	-0.65000
20.564	-0.65000
20.564	-0.65000
20.564	-0.65000
20.564	-0.65000
20.564	-0.65000
20.564	-0.65000
20.565	-0.65000
20.565	-0.65000
20.565	-0.65000
20.565	-0.65000
20.565	-0.65000
20.565	-0.65000
20.565	-0.65000
20.566	-0.65000
20.566	-0.65000
20.566	-0.65000
20.566	-0.65000
20.566	-0.65000
20.566	-0.65000
20.566	-0.65000

Time (s)	Tip potential during pulse (V)
20.566	-0.65000
20.566	-0.65000
20.567	-0.65000
20.567	-0.65000
20.567	-0.65000
20.567	-0.65000
20.567	-0.65000
20.567	-0.65000
20.567	-0.65000
20.568	-0.65000
20.568	-0.65000
20.568	-0.65000
20.568	-0.65000
20.568	-0.65000
20.568	-0.65000
20.568	-0.65000
20.568	-0.65000
20.569	-0.65000
20.569	-0.65000
20.569	-0.65000
20.569	-0.65000
20.569	-0.65000
20.569	-0.65000
20.569	-0.65000
20.570	-0.65000
20.570	-0.65000
20.570	-0.65000
20.570	-0.65000
20.570	-0.65000
20.570	-0.65000
20.570	-0.65000
20.570	-0.65000
20.571	-0.65000
20.571	-0.65000
20.571	-0.65000
20.571	-0.65000
20.571	-0.65000
20.571	-0.65000
20.571	-0.65000
20.572	-0.65000

Time (s)	Tip potential during pulse (V)
20.572	-0.65000
20.572	-0.65000
20.572	-0.65000
20.572	-0.65000
20.572	-0.65000
20.572	-0.65000
20.572	-0.65000
20.573	-0.65000
20.573	-0.65000
20.573	-0.65000
20.573	-0.65000
20.573	-0.65000
20.573	-0.65000
20.573	-0.65000
20.574	-0.65000
20.574	-0.65000
20.574	-0.65000
20.574	-0.65000
20.574	-0.65000
20.574	-0.65000
20.574	-0.65000
20.575	-0.65000
20.575	-0.65000
20.575	-0.65000
20.575	-0.65000
20.575	-0.65000
20.575	-0.65000
20.575	-0.65000
20.575	-0.65000
20.576	-0.65000
20.576	-0.65000
20.576	-0.65000
20.576	-0.65000
20.576	-0.65000
20.576	-0.65000
20.576	-0.65000
20.577	-0.65000
20.577	-0.65000
20.577	-0.65000
20.577	-0.65000

Time (s)	Tip potential during pulse (V)
20.577	-0.65000
20.577	-0.65000
20.577	-0.65000
20.578	-0.65000
20.578	-0.65000
20.578	-0.65000
20.578	-0.65000
20.578	-0.65000
20.578	-0.65000
20.578	-0.65000
20.578	-0.65000
20.579	-0.65000
20.579	-0.65000
20.579	-0.65000
20.579	-0.65000
20.579	-0.65000
20.579	-0.65000
20.579	-0.65000
20.580	-0.65000
20.580	-0.65000
20.580	-0.65000
20.580	-0.65000
20.580	-0.65000
20.580	-0.65000
20.580	-0.65000
20.581	-0.65000
20.581	-0.65000
20.581	-0.65000
20.581	-0.65000
20.581	-0.65000
20.581	-0.65000
20.581	-0.65000
20.581	-0.65000
20.582	-0.65000
20.582	-0.65000
20.582	-0.65000
20.582	-0.65000
20.582	-0.65000
20.582	-0.65000
20.582	-0.65000

Time (s)	Tip potential during pulse (V)
20.583	-0.65000
20.583	-0.65000
20.583	-0.65000
20.583	-0.65000
20.583	-0.65000
20.583	-0.65000
20.583	-0.65000
20.584	-0.65000
20.584	-0.65000
20.584	-0.65000
20.584	-0.65000
20.584	-0.65000
20.584	-0.65000
20.584	-0.65000
20.584	-0.65000
20.585	-0.65000
20.585	-0.65000
20.585	-0.65000
20.585	-0.65000
20.585	-0.65000
20.585	-0.65000
20.585	-0.65000
20.585	-0.65000
20.586	-0.65000
20.586	-0.65000
20.586	-0.65000
20.586	-0.65000
20.586	-0.65000
20.586	-0.65000
20.586	-0.65000
20.586	-0.65000
20.587	-0.65000
20.587	-0.65000
20.587	-0.65000
20.587	-0.65000
20.587	-0.65000
20.587	-0.65000
20.587	-0.65000
20.588	-0.65000
20.588	-0.65000

Time (s)	Tip potential during pulse (V)
20.588	-0.65000
20.588	-0.65000
20.588	-0.65000
20.589	-0.65000
20.589	-0.65000
20.589	-0.65000
20.589	-0.65000
20.589	-0.65000
20.589	-0.65000
20.589	-0.65000
20.589	-0.65000
20.590	-0.65000
20.590	-0.65000
20.590	-0.65000
20.590	-0.65000
20.590	-0.65000
20.590	-0.65000
20.590	-0.65000
20.591	-0.65000
20.591	-0.65000
20.591	-0.65000
20.591	-0.65000
20.591	-0.65000
20.591	-0.65000
20.591	-0.65000
20.592	-0.65000
20.592	-0.65000
20.592	-0.65000
20.592	-0.65000
20.592	-0.65000
20.592	-0.65000
20.592	-0.65000
20.592	-0.65000
20.593	-0.65000
20.593	-0.65000
20.593	-0.65000
20.593	-0.65000
20.593	-0.65000
20.593	-0.65000
20.593	-0.65000

Time (s)	Tip potential during pulse (V)
20.594	-0.65000
20.594	-0.65000
20.594	-0.65000
20.594	-0.65000
20.594	-0.65000
20.594	-0.65000
20.594	-0.65000
20.595	-0.65000
20.595	-0.65000
20.595	-0.65000
20.595	-0.65000
20.595	-0.65000
20.595	-0.65000
20.595	-0.65000
20.595	-0.65000
20.596	-0.65000
20.596	-0.65000
20.596	-0.65000
20.596	-0.65000
20.596	-0.65000
20.596	-0.65000
20.596	-0.65000
20.597	-0.65000
20.597	-0.65000
20.597	-0.65000
20.597	-0.65000
20.597	-0.65000
20.597	-0.65000
20.597	-0.65000
20.598	-0.65000
20.598	-0.65000
20.598	-0.65000
20.598	-0.65000
20.598	-0.65000
20.598	-0.65000
20.598	-0.65000
20.598	-0.65000
20.599	-0.65000
20.599	-0.65000
20.599	-0.65000

Time (s)	Tip potential during pulse (V)
20.599	-0.65000
20.599	-0.65000
20.599	-0.65000
20.599	-0.65000
20.600	-0.65000
20.600	-0.65000
20.600	-0.65000
20.600	-0.65000
20.600	-0.65000
20.600	-0.65000
20.600	-0.65000
20.601	-0.65000
20.601	-0.65000
20.601	-0.65000
20.601	-0.65000
20.601	-0.65000
20.601	-0.65000
20.601	-0.65000
20.601	-0.65000
20.602	-0.65000
20.602	-0.65000
20.602	-0.65000
20.602	-0.65000
20.602	-0.65000
20.602	-0.65000
20.602	-0.65000
20.603	-0.65000
20.603	-0.65000
20.603	-0.65000
20.603	-0.65000
20.603	-0.65000
20.603	-0.65000
20.603	-0.65000
20.604	-0.65000
20.604	-0.65000
20.604	-0.65000
20.604	-0.65000
20.604	-0.65000
20.604	-0.65000
20.604	-0.65000

Time (s)	Tip potential during pulse (V)
20.604	-0.65000
20.605	-0.65000
20.605	-0.65000
20.605	-0.65000
20.605	-0.65000
20.605	-0.65000
20.605	-0.65000
20.605	-0.65000
20.606	-0.65000
20.606	-0.65000
20.606	-0.65000
20.606	-0.65000
20.606	-0.65000
20.606	-0.65000
20.606	-0.65000
20.606	-0.65000
20.607	-0.65000
20.607	-0.65000
20.607	-0.65000
20.607	-0.65000
20.607	-0.65000
20.607	-0.65000
20.607	-0.65000
20.608	-0.65000
20.608	-0.65000
20.608	-0.65000
20.608	-0.65000
20.608	-0.65000
20.608	-0.65000
20.608	-0.65000
20.608	-0.65000
20.609	-0.65000
20.609	-0.65000
20.609	-0.65000
20.609	-0.65000
20.609	-0.65000
20.609	-0.65000
20.609	-0.65000
20.609	-0.65000
20.610	-0.65000

Time (s)	Tip potential during pulse (V)
20.610	-0.65000
20.610	-0.65000
20.610	-0.65000
20.610	-0.65000
20.610	-0.65000
20.610	-0.65000
20.610	-0.65000
20.611	-0.65000
20.611	-0.65000
20.611	-0.65000
20.611	-0.65000
20.611	-0.65000
20.611	-0.65000
20.611	-0.65000
20.612	-0.65000
20.612	-0.65000
20.612	-0.65000
20.612	-0.65000
20.612	-0.65000
20.612	-0.65000
20.612	-0.65000
20.613	-0.65000
20.613	-0.65000
20.613	-0.65000
20.613	-0.65000
20.613	-0.65000
20.613	-0.65000
20.613	-0.65000
20.613	-0.65000
20.614	-0.65000
20.614	-0.65000
20.614	-0.65000
20.614	-0.65000
20.614	-0.65000
20.614	-0.65000
20.614	-0.65000
20.615	-0.65000
20.615	-0.65000
20.615	-0.65000
20.615	-0.65000

Time (s)	Tip potential during pulse (V)
20.615	-0.65000
20.615	-0.65000
20.615	-0.65000
20.616	-0.65000
20.616	-0.65000
20.616	-0.65000
20.616	-0.65000
20.616	-0.65000
20.616	-0.65000
20.616	-0.65000
20.616	-0.65000
20.617	-0.65000
20.617	-0.65000
20.617	-0.65000
20.617	-0.65000
20.617	-0.65000
20.617	-0.65000
20.617	-0.65000
20.617	-0.65000
20.617	-0.65000
20.618	-0.65000
20.618	-0.65000
20.618	-0.65000
20.618	-0.65000
20.618	-0.65000
20.618	-0.65000
20.618	-0.65000
20.619	-0.65000
20.619	-0.65000
20.619	-0.65000
20.619	-0.65000
20.619	-0.65000
20.619	-0.65000
20.619	-0.65000
20.619	-0.65000
20.620	-0.65000
20.620	-0.65000
20.620	-0.65000
20.620	-0.65000
20.620	-0.65000

Time (s)	Tip potential during pulse (V)
20.620	-0.65000
20.620	-0.65000
20.620	-0.65000
20.621	-0.65000
20.621	-0.65000
20.621	-0.65000
20.621	-0.65000
20.621	-0.65000
20.621	-0.65000
20.621	-0.65000
20.622	-0.65000
20.622	-0.65000
20.622	-0.65000
20.622	-0.65000
20.622	-0.65000
20.622	-0.65000
20.622	-0.65000
20.623	-0.65000
20.623	-0.65000
20.623	-0.65000
20.623	-0.65000
20.623	-0.65000
20.623	-0.65000
20.623	-0.65000
20.623	-0.65000
20.624	-0.65000
20.624	-0.65000
20.624	-0.65000
20.624	-0.65000
20.624	-0.65000
20.624	-0.65000
20.624	-0.65000
20.625	-0.65000
20.625	-0.65000
20.625	-0.65000
20.625	-0.65000
20.625	-0.65000
20.625	-0.65000
20.625	-0.65000
20.626	-0.65000

Time (s)	Tip potential during pulse (V)
20.626	-0.65000
20.626	-0.65000
20.626	-0.65000
20.626	-0.65000
20.626	-0.65000
20.626	-0.65000
20.626	-0.65000
20.627	-0.65000
20.627	-0.65000
20.627	-0.65000
20.627	-0.65000
20.627	-0.65000
20.627	-0.65000
20.627	-0.65000
20.628	-0.65000
20.628	-0.65000
20.628	-0.65000
20.628	-0.65000
20.628	-0.65000
20.628	-0.65000
20.628	-0.65000
20.629	-0.65000
20.629	-0.65000
20.629	-0.65000
20.629	-0.65000
20.629	-0.65000
20.629	-0.65000
20.629	-0.65000
20.629	-0.65000
20.630	-0.65000
20.630	-0.65000
20.630	-0.65000
20.630	-0.65000
20.630	-0.65000
20.630	-0.65000
20.630	-0.65000
20.630	-0.65000
20.631	-0.65000
20.631	-0.65000
20.631	-0.65000

Time (s)	Tip potential during pulse (V)
20.631	-0.65000
20.631	-0.65000
20.631	-0.65000
20.631	-0.65000
20.632	-0.65000
20.632	-0.65000
20.632	-0.65000
20.632	-0.65000
20.632	-0.65000
20.632	-0.65000
20.632	-0.65000
20.632	-0.65000
20.632	-0.65000
20.633	-0.65000
20.633	-0.65000
20.633	-0.65000
20.633	-0.65000
20.633	-0.65000
20.633	-0.65000
20.633	-0.65000
20.634	-0.65000
20.634	-0.65000
20.634	-0.65000
20.634	-0.65000
20.634	-0.65000
20.634	-0.65000
20.634	-0.65000
20.635	-0.65000
20.635	-0.65000
20.635	-0.65000
20.635	-0.65000
20.635	-0.65000
20.635	-0.65000
20.635	-0.65000
20.635	-0.65000
20.636	-0.65000
20.636	-0.65000
20.636	-0.65000
20.636	-0.65000
20.636	-0.65000

Time (s)	Tip potential during pulse (V)
20.636	-0.65000
20.636	-0.65000
20.637	-0.65000
20.637	-0.65000
20.637	-0.65000
20.637	-0.65000
20.637	-0.65000
20.637	-0.65000
20.637	-0.65000
20.638	-0.65000
20.638	-0.65000
20.638	-0.65000
20.638	-0.65000
20.638	-0.65000
20.638	-0.65000
20.638	-0.65000
20.638	-0.65000
20.639	-0.65000
20.639	-0.65000
20.639	-0.65000
20.639	-0.65000
20.639	-0.65000
20.639	-0.65000
20.639	-0.65000
20.639	-0.65000
20.640	-0.65000
20.640	-0.65000
20.640	-0.65000
20.640	-0.65000
20.640	-0.65000
20.640	-0.65000
20.640	-0.65000
20.641	-0.65000
20.641	-0.65000
20.641	-0.65000
20.641	-0.65000
20.641	-0.65000
20.641	-0.65000
20.641	-0.65000
20.642	-0.65000

Time (s)	Tip potential during pulse (V)
20.642	-0.65000
20.642	-0.65000
20.642	-0.65000
20.642	-0.65000
20.642	-0.65000
20.642	-0.65000
20.642	-0.65000
20.643	-0.65000
20.643	-0.65000
20.643	-0.65000
20.643	-0.65000
20.643	-0.65000
20.643	-0.65000
20.643	-0.65000
20.644	-0.65000
20.644	-0.65000
20.644	-0.65000
20.644	-0.65000
20.644	-0.65000
20.644	-0.65000
20.644	-0.65000
20.645	-0.65000
20.645	-0.65000
20.645	-0.65000
20.645	-0.65000
20.645	-0.65000
20.645	-0.65000
20.645	-0.65000
20.645	-0.65000
20.645	-0.65000
20.646	-0.65000
20.646	-0.65000
20.646	-0.65000
20.646	-0.65000
20.646	-0.65000
20.646	-0.65000
20.646	-0.65000
20.647	-0.65000
20.647	-0.65000
20.647	-0.65000

Time (s)	Tip potential during pulse (V)
20.647	-0.65000
20.647	-0.65000
20.647	-0.65000
20.647	-0.65000
20.648	-0.65000
20.648	-0.65000
20.648	-0.65000
20.648	-0.65000
20.648	-0.65000
20.648	-0.65000
20.648	-0.65000
20.648	-0.65000
20.648	-0.65000
20.649	-0.65000
20.649	-0.65000
20.649	-0.65000
20.649	-0.65000
20.649	-0.65000
20.649	-0.65000
20.649	-0.65000
20.650	-0.65000
20.650	-0.65000
20.650	-0.65000
20.650	-0.65000
20.650	-0.65000
20.650	-0.65000
20.650	-0.65000
20.651	-0.65000
20.651	-0.65000
20.651	-0.65000
20.651	-0.65000
20.651	-0.65000
20.651	-0.65000
20.651	-0.65000
20.651	-0.65000
20.652	-0.65000
20.652	-0.65000
20.652	-0.65000
20.652	-0.65000
20.652	-0.65000

Time (s)	Tip potential during pulse (V)
20.652	-0.65000
20.652	-0.65000
20.652	-0.65000
20.653	-0.65000
20.653	-0.65000
20.653	-0.65000
20.653	-0.65000
20.653	-0.65000
20.653	-0.65000
20.653	-0.65000
20.654	-0.65000
20.654	-0.65000
20.654	-0.65000
20.654	-0.65000
20.654	-0.65000
20.654	-0.65000
20.654	-0.65000
20.655	-0.65000
20.655	-0.65000
20.655	-0.65000
20.655	-0.65000
20.655	-0.65000
20.655	-0.65000
20.655	-0.65000
20.655	-0.65000
20.655	-0.65000
20.656	-0.65000
20.656	-0.65000
20.656	-0.65000
20.656	-0.65000
20.656	-0.65000
20.656	-0.65000
20.656	-0.65000
20.657	-0.65000
20.657	-0.65000
20.657	-0.65000
20.657	-0.65000
20.657	-0.65000
20.657	-0.65000
20.657	-0.65000

Time (s)	Tip potential during pulse (V)
20.657	-0.65000
20.658	-0.65000
20.658	-0.65000
20.658	-0.65000
20.658	-0.65000
20.658	-0.65000
20.658	-0.65000
20.658	-0.65000
20.659	-0.65000
20.659	-0.65000
20.659	-0.65000
20.659	-0.65000
20.659	-0.65000
20.659	-0.65000
20.659	-0.65000
20.660	-0.65000
20.660	-0.65000
20.660	-0.65000
20.660	-0.65000
20.660	-0.65000
20.660	-0.65000
20.660	-0.65000
20.660	-0.65000
20.661	-0.65000
20.661	-0.65000
20.661	-0.65000
20.661	-0.65000
20.661	-0.65000
20.661	-0.65000
20.661	-0.65000
20.662	-0.65000
20.662	-0.65000
20.662	-0.65000
20.662	-0.65000
20.662	-0.65000
20.662	-0.65000
20.662	-0.65000
20.663	-0.65000
20.663	-0.65000
20.663	-0.65000

Time (s)	Tip potential during pulse (V)
20.663	-0.65000
20.663	-0.65000
20.663	-0.65000
20.663	-0.65000
20.663	-0.65000
20.664	-0.65000
20.664	-0.65000
20.664	-0.65000
20.664	-0.65000
20.664	-0.65000
20.664	-0.65000
20.664	-0.65000
20.665	-0.65000
20.665	-0.65000
20.665	-0.65000
20.665	-0.65000
20.665	-0.65000
20.665	-0.65000
20.665	-0.65000
20.666	-0.65000
20.666	-0.65000
20.666	-0.65000
20.666	-0.65000
20.666	-0.65000
20.666	-0.65000
20.666	-0.65000
20.666	-0.65000
20.667	-0.65000
20.667	-0.65000
20.667	-0.65000
20.667	-0.65000
20.667	-0.65000
20.667	-0.65000
20.667	-0.65000
20.668	-0.65000
20.668	-0.65000
20.668	-0.65000
20.668	-0.65000
20.668	-0.65000
20.668	-0.65000

Time (s)	Tip potential during pulse (V)
20.668	-0.65000
20.669	-0.65000
20.669	-0.65000
20.669	-0.65000
20.669	-0.65000
20.669	-0.65000
20.669	-0.65000
20.669	-0.65000
20.669	-0.65000
20.670	-0.65000
20.670	-0.65000
20.670	-0.65000
20.670	-0.65000
20.670	-0.65000
20.670	-0.65000
20.670	-0.65000
20.671	-0.65000
20.671	-0.65000
20.671	-0.65000
20.671	-0.65000
20.671	-0.65000
20.671	-0.65000
20.671	-0.65000
20.672	-0.65000
20.672	-0.65000
20.672	-0.65000
20.672	-0.65000
20.672	-0.65000
20.672	-0.65000
20.672	-0.65000
20.673	-0.65000
20.673	-0.65000
20.673	-0.65000
20.673	-0.65000
20.673	-0.65000
20.673	-0.65000
20.673	-0.65000
20.674	-0.65000
20.674	-0.65000
20.674	-0.65000

Time (s)	Tip potential during pulse (V)
20.674	-0.65000
20.674	-0.65000
20.674	-0.65000
20.674	-0.65000
20.674	-0.65000
20.674	-0.65000
20.675	-0.65000
20.675	-0.65000
20.675	-0.65000
20.675	-0.65000
20.675	-0.65000
20.675	-0.65000
20.675	-0.65000
20.675	-0.65000
20.676	-0.65000
20.676	-0.65000
20.676	-0.65000
20.676	-0.65000
20.676	-0.65000
20.676	-0.65000
20.676	-0.65000
20.677	-0.65000
20.677	-0.65000
20.677	-0.65000
20.677	-0.65000
20.677	-0.65000
20.677	-0.65000
20.677	-0.65000
20.677	-0.65000
20.678	-0.65000
20.678	-0.65000
20.678	-0.65000
20.678	-0.65000
20.678	-0.65000
20.678	-0.65000
20.678	-0.65000
20.679	-0.65000
20.679	-0.65000
20.679	-0.65000
20.679	-0.65000

Time (s)	Tip potential during pulse (V)
20.679	-0.65000
20.679	-0.65000
20.679	-0.65000
20.680	-0.65000
20.680	-0.65000
20.680	-0.65000
20.680	-0.65000
20.680	-0.65000
20.680	-0.65000
20.680	-0.65000
20.680	-0.65000
20.680	-0.65000
20.681	-0.65000
20.681	-0.65000
20.681	-0.65000
20.681	-0.65000
20.681	-0.65000
20.681	-0.65000
20.681	-0.65000
20.682	-0.65000
20.682	-0.65000
20.682	-0.65000
20.682	-0.65000
20.682	-0.65000
20.682	-0.65000
20.682	-0.65000
20.682	-0.65000
20.683	-0.65000
20.683	-0.65000
20.683	-0.65000
20.683	-0.65000
20.683	-0.65000
20.683	-0.65000
20.683	-0.65000
20.683	-0.65000
20.684	-0.65000
20.684	-0.65000
20.684	-0.65000
20.684	-0.65000
20.684	-0.65000

Time (s)	Tip potential during pulse (V)
20.684	-0.65000
20.684	-0.65000
20.685	-0.65000
20.685	-0.65000
20.685	-0.65000
20.685	-0.65000
20.685	-0.65000
20.685	-0.65000
20.685	-0.65000
20.686	-0.65000
20.686	-0.65000
20.686	-0.65000
20.686	-0.65000
20.686	-0.65000
20.686	-0.65000
20.686	-0.65000
20.686	-0.65000
20.687	-0.65000
20.687	-0.65000
20.687	-0.65000
20.687	-0.65000
20.687	-0.65000
20.687	-0.65000
20.687	-0.65000
20.688	-0.65000
20.688	-0.65000
20.688	-0.65000
20.688	-0.65000
20.688	-0.65000
20.688	-0.65000
20.688	-0.65000
20.689	-0.65000
20.689	-0.65000
20.689	-0.65000
20.689	-0.65000
20.689	-0.65000
20.689	-0.65000
20.689	-0.65000
20.689	-0.65000
20.690	-0.65000

Time (s)	Tip potential during pulse (V)
20.690	-0.65000
20.690	-0.65000
20.690	-0.65000
20.690	-0.65000
20.690	-0.65000
20.690	-0.65000
20.691	-0.65000
20.691	-0.65000
20.691	-0.65000
20.691	-0.65000
20.691	-0.65000
20.691	-0.65000
20.691	-0.65000
20.692	-0.65000
20.692	-0.65000
20.692	-0.65000
20.692	-0.65000
20.692	-0.65000
20.692	-0.65000
20.692	-0.65000
20.692	-0.65000
20.693	-0.65000
20.693	-0.65000
20.693	-0.65000
20.693	-0.65000
20.693	-0.65000
20.693	-0.65000
20.693	-0.65000
20.694	-0.65000
20.694	-0.65000
20.694	-0.65000
20.694	-0.65000
20.694	-0.65000
20.694	-0.65000
20.694	-0.65000
20.695	-0.65000
20.695	-0.65000
20.695	-0.65000
20.695	-0.65000
20.695	-0.65000

Time (s)	Tip potential during pulse (V)
20.695	-0.65000
20.695	-0.65000
20.695	-0.65000
20.696	-0.65000
20.696	-0.65000
20.696	-0.65000
20.696	-0.65000
20.696	-0.65000
20.696	-0.65000
20.696	-0.65000
20.697	-0.65000
20.697	-0.65000
20.697	-0.65000
20.697	-0.65000
20.697	-0.65000
20.697	-0.65000
20.697	-0.65000
20.698	-0.65000
20.698	-0.65000
20.698	-0.65000
20.698	-0.65000
20.698	-0.65000
20.698	-0.65000
20.698	-0.65000
20.698	-0.65000
20.699	-0.65000
20.699	-0.65000
20.699	-0.65000
20.699	-0.65000
20.699	-0.65000
20.699	-0.65000
20.699	-0.65000
20.700	-0.65000
20.700	-0.65000
20.700	-0.65000
20.700	-0.65000
20.700	-0.65000
20.700	-0.65000
20.700	-0.65000
20.701	-0.65000

Time (s)	Tip potential during pulse (V)
20.701	-0.65000
20.701	-0.65000
20.701	-0.65000
20.701	-0.65000
20.701	-0.65000
20.701	-0.65000
20.701	-0.65000
20.702	-0.65000
20.702	-0.65000
20.702	-0.65000
20.702	-0.65000
20.702	-0.65000
20.702	-0.65000
20.702	-0.65000
20.703	-0.65000
20.703	-0.65000
20.703	-0.65000
20.703	-0.65000
20.703	-0.65000
20.703	-0.65000
20.703	-0.65000
20.704	-0.65000
20.704	-0.65000
20.704	-0.65000
20.704	-0.65000
20.704	-0.65000
20.704	-0.65000
20.704	-0.65000
20.704	-0.65000
20.705	-0.65000
20.705	-0.65000
20.705	-0.65000
20.705	-0.65000
20.705	-0.65000
20.705	-0.65000
20.705	-0.65000
20.705	-0.65000
20.706	-0.65000
20.706	-0.65000
20.706	-0.65000

Time (s)	Tip potential during pulse (V)
20.706	-0.65000
20.706	-0.65000
20.706	-0.65000
20.706	-0.65000
20.707	-0.65000
20.707	-0.65000
20.707	-0.65000
20.707	-0.65000
20.707	-0.65000
20.707	-0.65000
20.707	-0.65000
20.708	-0.65000
20.708	-0.65000
20.708	-0.65000
20.708	-0.65000
20.708	-0.65000
20.708	-0.65000
20.708	-0.65000
20.708	-0.65000
20.708	-0.65000
20.709	-0.65000
20.709	-0.65000
20.709	-0.65000
20.709	-0.65000
20.709	-0.65000
20.709	-0.65000
20.709	-0.65000
20.709	-0.65000
20.710	-0.65000
20.710	-0.65000
20.710	-0.65000
20.710	-0.65000
20.710	-0.65000
20.710	-0.65000
20.710	-0.65000
20.711	-0.65000
20.711	-0.65000
20.711	-0.65000
20.711	-0.65000
20.711	-0.65000

Time (s)	Tip potential during pulse (V)
20.711	-0.65000
20.711	-0.65000
20.711	-0.65000
20.712	-0.65000
20.712	-0.65000
20.712	-0.65000
20.712	-0.65000
20.712	-0.65000
20.712	-0.65000
20.712	-0.65000
20.713	-0.65000
20.713	-0.65000
20.713	-0.65000
20.713	-0.65000
20.713	-0.65000
20.713	-0.65000
20.713	-0.65000
20.714	-0.65000
20.714	-0.65000
20.714	-0.65000
20.714	-0.65000
20.714	-0.65000
20.714	-0.65000
20.714	-0.65000
20.714	-0.65000
20.715	-0.65000
20.715	-0.65000
20.715	-0.65000
20.715	-0.65000
20.715	-0.65000
20.715	-0.65000
20.715	-0.65000
20.716	-0.65000
20.716	-0.65000
20.716	-0.65000
20.716	-0.65000
20.716	-0.65000
20.716	-0.65000
20.716	-0.65000
20.717	-0.65000

Time (s)	Tip potential during pulse (V)
20.717	-0.65000
20.717	-0.65000
20.717	-0.65000
20.717	-0.65000
20.717	-0.65000
20.717	-0.65000
20.717	-0.65000
20.718	-0.65000
20.718	-0.65000
20.718	-0.65000
20.718	-0.65000
20.718	-0.65000
20.718	-0.65000
20.718	-0.65000
20.719	-0.65000
20.719	-0.65000
20.719	-0.65000
20.719	-0.65000
20.719	-0.65000
20.719	-0.65000
20.719	-0.65000
20.720	-0.65000
20.720	-0.65000
20.720	-0.65000
20.720	-0.65000
20.720	-0.65000
20.720	-0.65000
20.720	-0.65000
20.720	-0.65000
20.721	-0.65000
20.721	-0.65000
20.721	-0.65000
20.721	-0.65000
20.721	-0.65000
20.721	-0.65000
20.721	-0.65000
20.721	-0.65000
20.722	-0.65000
20.722	-0.65000
20.722	-0.65000

Time (s)	Tip potential during pulse (V)
20.722	-0.65000
20.722	-0.65000
20.722	-0.65000
20.722	-0.65000
20.722	-0.65000
20.723	-0.65000
20.723	-0.65000
20.723	-0.65000
20.723	-0.65000
20.723	-0.65000
20.723	-0.65000
20.723	-0.65000
20.723	-0.65000
20.723	-0.65000
20.724	-0.65000
20.724	-0.65000
20.724	-0.65000
20.724	-0.65000
20.724	-0.65000
20.724	-0.65000
20.724	-0.65000
20.725	-0.65000
20.725	-0.65000
20.725	-0.65000
20.725	-0.65000
20.725	-0.65000
20.725	-0.65000
20.725	-0.65000
20.725	-0.65000
20.726	-0.65000
20.726	-0.65000
20.726	-0.65000
20.726	-0.65000
20.726	-0.65000
20.726	-0.65000
20.726	-0.65000
20.727	-0.65000
20.727	-0.65000
20.727	-0.65000
20.727	-0.65000

Time (s)	Tip potential during pulse (V)
20.727	-0.65000
20.727	-0.65000
20.727	-0.65000
20.728	-0.65000
20.728	-0.65000
20.728	-0.65000
20.728	-0.65000
20.728	-0.65000
20.728	-0.65000
20.728	-0.65000
20.728	-0.65000
20.729	-0.65000
20.729	-0.65000
20.729	-0.65000
20.729	-0.65000
20.729	-0.65000
20.729	-0.65000
20.729	-0.65000
20.730	-0.65000
20.730	-0.65000
20.730	-0.65000
20.730	-0.65000
20.730	-0.65000
20.730	-0.65000
20.730	-0.65000
20.731	-0.65000
20.731	-0.65000
20.731	-0.65000
20.731	-0.65000
20.731	-0.65000
20.731	-0.65000
20.731	-0.65000
20.731	-0.65000
20.732	-0.65000
20.732	-0.65000
20.732	-0.65000
20.732	-0.65000
20.732	-0.65000
20.732	-0.65000
20.732	-0.65000

Time (s)	Tip potential during pulse (V)
20.733	-0.65000
20.733	-0.65000
20.733	-0.65000
20.733	-0.65000
20.733	-0.65000
20.733	-0.65000
20.733	-0.65000
20.733	-0.65000
20.733	-0.65000
20.734	-0.65000
20.734	-0.65000
20.734	-0.65000
20.734	-0.65000
20.734	-0.65000
20.734	-0.65000
20.734	-0.65000
20.735	-0.65000
20.735	-0.65000
20.735	-0.65000
20.735	-0.65000
20.735	-0.65000
20.735	-0.65000
20.735	-0.65000
20.736	-0.65000
20.736	-0.65000
20.736	-0.65000
20.736	-0.65000
20.736	-0.65000
20.736	-0.65000
20.736	-0.65000
20.737	-0.65000
20.737	-0.65000
20.737	-0.65000
20.737	-0.65000
20.737	-0.65000
20.737	-0.65000
20.737	-0.65000
20.738	-0.65000
20.738	-0.65000
20.738	-0.65000

Time (s)	Tip potential during pulse (V)
20.738	-0.65000
20.738	-0.65000
20.738	-0.65000
20.738	-0.65000
20.738	-0.65000
20.739	-0.65000
20.739	-0.65000
20.739	-0.65000
20.739	-0.65000
20.739	-0.65000
20.739	-0.65000
20.739	-0.65000
20.740	-0.65000
20.740	-0.65000
20.740	-0.65000
20.740	-0.65000
20.740	-0.65000
20.740	-0.65000
20.740	-0.65000
20.741	-0.65000
20.741	-0.65000
20.741	-0.65000
20.741	-0.65000
20.741	-0.65000
20.741	-0.65000
20.741	-0.65000
20.741	-0.65000
20.742	-0.65000
20.742	-0.65000
20.742	-0.65000
20.742	-0.65000
20.742	-0.65000
20.742	-0.65000
20.742	-0.65000
20.743	-0.65000
20.743	-0.65000
20.743	-0.65000
20.743	-0.65000
20.743	-0.65000
20.743	-0.65000

Time (s)	Tip potential during pulse (V)
20.743	-0.65000
20.743	-0.65000
20.743	-0.65000
20.744	-0.65000
20.744	-0.65000
20.744	-0.65000
20.744	-0.65000
20.744	-0.65000
20.744	-0.65000
20.744	-0.65000
20.745	-0.65000
20.745	-0.65000
20.745	-0.65000
20.745	-0.65000
20.745	-0.65000
20.745	-0.65000
20.745	-0.65000
20.746	-0.65000
20.746	-0.65000
20.746	-0.65000
20.746	-0.65000
20.746	-0.65000
20.746	-0.65000
20.746	-0.65000
20.746	-0.65000
20.747	-0.65000
20.747	-0.65000
20.747	-0.65000
20.747	-0.65000
20.747	-0.65000
20.747	-0.65000
20.747	-0.65000
20.748	-0.65000
20.748	-0.65000
20.748	-0.65000
20.748	-0.65000
20.748	-0.65000
20.748	-0.65000
20.748	-0.65000
20.749	-0.65000

Time (s)	Tip potential during pulse (V)
20.749	-0.65000
20.749	-0.65000
20.749	-0.65000
20.749	-0.65000
20.749	-0.65000
20.749	-0.65000
20.749	-0.65000
20.750	-0.65000
20.750	-0.65000
20.750	-0.65000
20.750	-0.65000
20.750	-0.65000
20.750	-0.65000
20.750	-0.65000
20.751	-0.65000
20.751	-0.65000
20.751	-0.65000
20.751	-0.65000
20.751	-0.65000
20.751	-0.65000
20.751	-0.65000
20.752	-0.65000
20.752	-0.65000
20.752	-0.65000
20.752	-0.65000
20.752	-0.65000
20.752	-0.65000
20.752	-0.65000
20.752	-0.65000
20.753	-0.65000
20.753	-0.65000
20.753	-0.65000
20.753	-0.65000
20.753	-0.65000
20.753	-0.65000
20.753	-0.65000
20.754	-0.65000
20.754	-0.65000
20.754	-0.65000
20.754	-0.65000

Time (s)	Tip potential during pulse (V)
20.754	-0.65000
20.754	-0.65000
20.754	-0.65000
20.755	-0.65000
20.755	-0.65000
20.755	-0.65000
20.755	-0.65000
20.755	-0.65000
20.755	-0.65000
20.755	-0.65000
20.755	-0.65000
20.756	-0.65000
20.756	-0.65000
20.756	-0.65000
20.756	-0.65000
20.756	-0.65000
20.756	-0.65000
20.756	-0.65000
20.757	-0.65000
20.757	-0.65000
20.757	-0.65000
20.757	-0.65000
20.757	-0.65000
20.757	-0.65000
20.757	-0.65000
20.757	-0.65000
20.758	-0.65000
20.758	-0.65000
20.758	-0.65000
20.758	-0.65000
20.758	-0.65000
20.758	-0.65000
20.758	-0.65000
20.759	-0.65000
20.759	-0.65000
20.759	-0.65000
20.759	-0.65000
20.759	-0.65000
20.759	-0.65000
20.759	-0.65000

Time (s)	Tip potential during pulse (V)
20.759	-0.65000
20.760	-0.65000
20.760	-0.65000
20.760	-0.65000
20.760	-0.65000
20.760	-0.65000
20.760	-0.65000
20.760	-0.65000
20.761	-0.65000
20.761	-0.65000
20.761	-0.65000
20.761	-0.65000
20.761	-0.65000
20.761	-0.65000
20.761	-0.65000
20.762	-0.65000
20.762	-0.65000
20.762	-0.65000
20.762	-0.65000
20.762	-0.65000
20.762	-0.65000
20.762	-0.65000
20.762	-0.65000
20.763	-0.65000
20.763	-0.65000
20.763	-0.65000
20.763	-0.65000
20.763	-0.65000
20.763	-0.65000
20.763	-0.65000
20.763	-0.65000
20.764	-0.65000
20.764	-0.65000
20.764	-0.65000
20.764	-0.65000
20.764	-0.65000
20.764	-0.65000
20.764	-0.65000
20.765	-0.65000
20.765	-0.65000

Time (s)	Tip potential during pulse (V)
20.765	-0.65000
20.765	-0.65000
20.765	-0.65000
20.765	-0.65000
20.765	-0.65000
20.766	-0.65000
20.766	-0.65000
20.766	-0.65000
20.766	-0.65000
20.766	-0.65000
20.766	-0.65000
20.766	-0.65000
20.766	-0.65000
20.767	-0.65000
20.767	-0.65000
20.767	-0.65000
20.767	-0.65000
20.767	-0.65000
20.767	-0.65000
20.767	-0.65000
20.768	-0.65000
20.768	-0.65000
20.768	-0.65000
20.768	-0.65000
20.768	-0.65000
20.768	-0.65000
20.768	-0.65000
20.769	-0.65000
20.769	-0.65000
20.769	-0.65000
20.769	-0.65000
20.769	-0.65000
20.769	-0.65000
20.769	-0.65000
20.769	-0.65000
20.770	-0.65000
20.770	-0.65000
20.770	-0.65000
20.770	-0.65000
20.770	-0.65000

Time (s)	Tip potential during pulse (V)
20.770	-0.65000
20.770	-0.65000
20.771	-0.65000
20.771	-0.65000
20.771	-0.65000
20.771	-0.65000
20.771	-0.65000
20.771	-0.65000
20.771	-0.65000
20.772	-0.65000
20.772	-0.65000
20.772	-0.65000
20.772	-0.65000
20.772	-0.65000
20.772	-0.65000
20.772	-0.65000
20.772	-0.65000
20.773	-0.65000
20.773	-0.65000
20.773	-0.65000
20.773	-0.65000
20.773	-0.65000
20.773	-0.65000
20.773	-0.65000
20.774	-0.65000
20.774	-0.65000
20.774	-0.65000
20.774	-0.65000
20.774	-0.65000
20.774	-0.65000
20.774	-0.65000
20.775	-0.65000
20.775	-0.65000
20.775	-0.65000
20.775	-0.65000
20.775	-0.65000
20.775	-0.65000
20.775	-0.65000
20.775	-0.65000
20.776	-0.65000

Time (s)	Tip potential during pulse (V)
20.776	-0.65000
20.776	-0.65000
20.776	-0.65000
20.776	-0.65000
20.776	-0.65000
20.776	-0.65000
20.777	-0.65000
20.777	-0.65000
20.777	-0.65000
20.777	-0.65000
20.777	-0.65000
20.777	-0.65000
20.777	-0.65000
20.778	-0.65000
20.778	-0.65000
20.778	-0.65000
20.778	-0.65000
20.778	-0.65000
20.778	-0.65000
20.778	-0.65000
20.778	-0.65000
20.779	-0.65000
20.779	-0.65000
20.779	-0.65000
20.779	-0.65000
20.779	-0.65000
20.779	-0.65000
20.779	-0.65000
20.780	-0.65000
20.780	-0.65000
20.780	-0.65000
20.780	-0.65000
20.780	-0.65000
20.780	-0.65000
20.780	-0.65000
20.781	-0.65000
20.781	-0.65000
20.781	-0.65000
20.781	-0.65000
20.781	-0.65000

Time (s)	Tip potential during pulse (V)
20.781	-0.65000
20.781	-0.65000
20.781	-0.65000
20.782	-0.65000
20.782	-0.65000
20.782	-0.65000
20.782	-0.65000
20.782	-0.65000
20.782	-0.65000
20.782	-0.65000
20.783	-0.65000
20.783	-0.65000
20.783	-0.65000
20.783	-0.65000
20.783	-0.65000
20.783	-0.65000
20.783	-0.65000
20.784	-0.65000
20.784	-0.65000
20.784	-0.65000
20.784	-0.65000
20.784	-0.65000
20.784	-0.65000
20.784	-0.65000
20.784	-0.65000
20.785	-0.65000
20.785	-0.65000
20.785	-0.65000
20.785	-0.65000
20.785	-0.65000
20.785	-0.65000
20.785	-0.65000
20.786	-0.65000
20.786	-0.65000
20.786	-0.65000
20.786	-0.65000
20.786	-0.65000
20.786	-0.65000
20.786	-0.65000
20.787	-0.65000

Time (s)	Tip potential during pulse (V)
20.787	-0.65000
20.787	-0.65000
20.787	-0.65000
20.787	-0.65000
20.787	-0.65000
20.787	-0.65000
20.787	-0.65000
20.788	-0.65000
20.788	-0.65000
20.788	-0.65000
20.788	-0.65000
20.788	-0.65000
20.788	-0.65000
20.788	-0.65000
20.789	-0.65000
20.789	-0.65000
20.789	-0.65000
20.789	-0.65000
20.789	-0.65000
20.789	-0.65000
20.789	-0.65000
20.790	-0.65000
20.790	-0.65000
20.790	-0.65000
20.790	-0.65000
20.790	-0.65000
20.790	-0.65000
20.790	-0.65000
20.790	-0.65000
20.791	-0.65000
20.791	-0.65000
20.791	-0.65000
20.791	-0.65000
20.791	-0.65000
20.791	-0.65000
20.791	-0.65000
20.791	-0.65000
20.792	-0.65000
20.792	-0.65000
20.792	-0.65000

Time (s)	Tip potential during pulse (V)
20.792	-0.65000
20.792	-0.65000
20.792	-0.65000
20.792	-0.65000
20.793	-0.65000
20.793	-0.65000
20.793	-0.65000
20.793	-0.65000
20.793	-0.65000
20.793	-0.65000
20.793	-0.65000
20.794	-0.65000
20.794	-0.65000
20.794	-0.65000
20.794	-0.65000
20.794	-0.65000
20.794	-0.65000
20.794	-0.65000
20.794	-0.65000
20.795	-0.65000
20.795	-0.65000
20.795	-0.65000
20.795	-0.65000
20.795	-0.65000
20.795	-0.65000
20.795	-0.65000
20.796	-0.65000
20.796	-0.65000
20.796	-0.65000
20.796	-0.65000
20.796	-0.65000
20.796	-0.65000
20.796	-0.65000
20.797	-0.65000
20.797	-0.65000
20.797	-0.65000
20.797	-0.65000
20.797	-0.65000
20.797	-0.65000
20.797	-0.65000

Time (s)	Tip potential during pulse (V)
20.797	-0.65000
20.798	-0.65000
20.798	-0.65000
20.798	-0.65000
20.798	-0.65000
20.798	-0.65000
20.798	-0.65000
20.798	-0.65000
20.799	-0.65000
20.799	-0.65000
20.799	-0.65000
20.799	-0.65000
20.799	-0.65000
20.799	-0.65000
20.799	-0.65000
20.800	-0.65000
20.800	-0.65000
20.800	-0.65000
20.800	-0.65000
20.800	-0.65000
20.800	-0.65000
20.800	-0.65000
20.800	-0.65000
20.801	-0.65000
20.801	-0.65000
20.801	-0.65000
20.801	-0.65000
20.801	-0.65000
20.801	-0.65000
20.801	-0.65000
20.802	-0.65000
20.802	-0.65000
20.802	-0.65000
20.802	-0.65000
20.802	-0.65000
20.802	-0.65000
20.802	-0.65000
20.803	-0.65000
20.803	-0.65000
20.803	-0.65000

Time (s)	Tip potential during pulse (V)
20.803	-0.65000
20.803	-0.65000
20.803	-0.65000
20.803	-0.65000
20.803	-0.65000
20.804	-0.65000
20.804	-0.65000
20.804	-0.65000
20.804	-0.65000
20.804	-0.65000
20.804	-0.65000
20.804	-0.65000
20.805	-0.65000
20.805	-0.65000
20.805	-0.65000
20.805	-0.65000
20.805	-0.65000
20.805	-0.65000
20.805	-0.65000
20.806	-0.65000
20.806	-0.65000
20.806	-0.65000
20.806	-0.65000
20.806	-0.65000
20.806	-0.65000
20.806	-0.65000
20.806	-0.65000
20.807	-0.65000
20.807	-0.65000
20.807	-0.65000
20.807	-0.65000
20.807	-0.65000
20.807	-0.65000
20.807	-0.65000
20.808	-0.65000
20.808	-0.65000
20.808	-0.65000
20.808	-0.65000
20.808	-0.65000
20.808	-0.65000

Time (s)	Tip potential during pulse (V)
20.808	-0.65000
20.809	-0.65000
20.809	-0.65000
20.809	-0.65000
20.809	-0.65000
20.809	-0.65000
20.809	-0.65000
20.809	-0.65000
20.809	-0.65000
20.810	-0.65000
20.810	-0.65000
20.810	-0.65000
20.810	-0.65000
20.810	-0.65000
20.810	-0.65000
20.810	-0.65000
20.811	-0.65000
20.811	-0.65000
20.811	-0.65000
20.811	-0.65000
20.811	-0.65000
20.811	-0.65000
20.811	-0.65000
20.812	-0.65000
20.812	-0.65000
20.812	-0.65000
20.812	-0.65000
20.812	-0.65000
20.812	-0.65000
20.812	-0.65000
20.812	-0.65000
20.813	-0.65000
20.813	-0.65000
20.813	-0.65000
20.813	-0.65000
20.813	-0.65000
20.813	-0.65000
20.813	-0.65000
20.814	-0.65000
20.814	-0.65000

Time (s)	Tip potential during pulse (V)
20.814	-0.65000
20.814	-0.65000
20.814	-0.65000
20.814	-0.65000
20.814	-0.65000
20.815	-0.65000
20.815	-0.65000
20.815	-0.65000
20.815	-0.65000
20.815	-0.65000
20.815	-0.65000
20.815	-0.65000
20.815	-0.65000
20.816	-0.65000
20.816	-0.65000
20.816	-0.65000
20.816	-0.65000
20.816	-0.65000
20.816	-0.65000
20.816	-0.65000
20.817	-0.65000
20.817	-0.65000
20.817	-0.65000
20.817	-0.65000
20.817	-0.65000
20.817	-0.65000
20.817	-0.65000
20.818	-0.65000
20.818	-0.65000
20.818	-0.65000
20.818	-0.65000
20.818	-0.65000
20.818	-0.65000
20.818	-0.65000
20.818	-0.65000
20.819	-0.65000
20.819	-0.65000
20.819	-0.65000
20.819	-0.65000
20.819	-0.65000

Time (s)	Tip potential during pulse (V)
20.819	-0.65000
20.819	-0.65000
20.820	-0.65000
20.820	-0.65000
20.820	-0.65000
20.820	-0.65000
20.820	-0.65000
20.820	-0.65000
20.820	-0.65000
20.821	-0.65000
20.821	-0.65000
20.821	-0.65000
20.821	-0.65000
20.821	-0.65000
20.821	-0.65000
20.821	-0.65000
20.821	-0.65000
20.822	-0.65000
20.822	-0.65000
20.822	-0.65000
20.822	-0.65000
20.822	-0.65000
20.822	-0.65000
20.822	-0.65000
20.823	-0.65000
20.823	-0.65000
20.823	-0.65000
20.823	-0.65000
20.823	-0.65000
20.823	-0.65000
20.823	-0.65000
20.824	-0.65000
20.824	-0.65000
20.824	-0.65000
20.824	-0.65000
20.824	-0.65000
20.824	-0.65000
20.824	-0.65000
20.824	-0.65000
20.825	-0.65000

Time (s)	Tip potential during pulse (V)
20.825	-0.65000
20.825	-0.65000
20.825	-0.65000
20.825	-0.65000
20.825	-0.65000
20.825	-0.65000
20.826	-0.65000
20.826	-0.65000
20.826	-0.65000
20.826	-0.65000
20.826	-0.65000
20.826	-0.65000
20.826	-0.65000
20.827	-0.65000
20.827	-0.65000
20.827	-0.65000
20.827	-0.65000
20.827	-0.65000
20.827	-0.65000
20.827	-0.65000
20.827	-0.65000
20.828	-0.65000
20.828	-0.65000
20.828	-0.65000
20.828	-0.65000
20.828	-0.65000
20.828	-0.65000
20.828	-0.65000
20.829	-0.65000
20.829	-0.65000
20.829	-0.65000
20.829	-0.65000
20.829	-0.65000
20.829	-0.65000
20.829	-0.65000
20.830	-0.65000
20.830	-0.65000
20.830	-0.65000
20.830	-0.65000
20.830	-0.65000

Time (s)	Tip potential during pulse (V)
20.830	-0.65000
20.830	-0.65000
20.830	-0.65000
20.831	-0.65000
20.831	-0.65000
20.831	-0.65000
20.831	-0.65000
20.831	-0.65000
20.831	-0.65000
20.831	-0.65000
20.832	-0.65000
20.832	-0.65000
20.832	-0.65000
20.832	-0.65000
20.832	-0.65000
20.832	-0.65000
20.832	-0.65000
20.833	-0.65000
20.833	-0.65000
20.833	-0.65000
20.833	-0.65000
20.833	-0.65000
20.833	-0.65000
20.833	-0.65000
20.833	-0.65000
20.834	-0.65000
20.834	-0.65000
20.834	-0.65000
20.834	-0.65000
20.834	-0.65000
20.834	-0.65000
20.834	-0.65000
20.835	-0.65000
20.835	-0.65000
20.835	-0.65000
20.835	-0.65000
20.835	-0.65000
20.835	-0.65000
20.835	-0.65000
20.836	-0.65000

Time (s)	Tip potential during pulse (V)
20.836	-0.65000
20.836	-0.65000
20.836	-0.65000
20.836	-0.65000
20.836	-0.65000
20.836	-0.65000
20.837	-0.65000
20.837	-0.65000
20.837	-0.65000
20.837	-0.65000
20.837	-0.65000
20.837	-0.65000
20.837	-0.65000
20.838	-0.65000
20.838	-0.65000
20.838	-0.65000
20.838	-0.65000
20.838	-0.65000
20.838	-0.65000
20.838	-0.65000
20.838	-0.65000
20.839	-0.65000
20.839	-0.65000
20.839	-0.65000
20.839	-0.65000
20.839	-0.65000
20.839	-0.65000
20.839	-0.65000
20.840	-0.65000
20.840	-0.65000
20.840	-0.65000
20.840	-0.65000
20.840	-0.65000
20.840	-0.65000
20.840	-0.65000
20.841	-0.65000
20.841	-0.65000
20.841	-0.65000
20.841	-0.65000
20.841	-0.65000

Time (s)	Tip potential during pulse (V)
20.841	-0.65000
20.841	-0.65000
20.841	-0.65000
20.842	-0.65000
20.842	-0.65000
20.842	-0.65000
20.842	-0.65000
20.842	-0.65000
20.842	-0.65000
20.842	-0.65000
20.843	-0.65000
20.843	-0.65000
20.843	-0.65000
20.843	-0.65000
20.843	-0.65000
20.843	-0.65000
20.843	-0.65000
20.843	-0.65000
20.843	-0.65000
20.844	-0.65000
20.844	-0.65000
20.844	-0.65000
20.844	-0.65000
20.844	-0.65000
20.844	-0.65000
20.844	-0.65000
20.845	-0.65000
20.845	-0.65000
20.845	-0.65000
20.845	-0.65000
20.845	-0.65000
20.845	-0.65000
20.845	-0.65000
20.846	-0.65000
20.846	-0.65000
20.846	-0.65000
20.846	-0.65000
20.846	-0.65000
20.846	-0.65000
20.846	-0.65000

Time (s)	Tip potential during pulse (V)
20.846	-0.65000
20.846	-0.65000
20.846	-0.65000
20.847	-0.65000
20.847	-0.65000
20.847	-0.65000
20.847	-0.65000
20.847	-0.65000
20.847	-0.65000
20.847	-0.65000
20.847	-0.65000
20.848	-0.65000
20.848	-0.65000
20.848	-0.65000
20.848	-0.65000
20.848	-0.65000
20.848	-0.65000
20.848	-0.65000
20.849	-0.65000
20.849	-0.65000
20.849	-0.65000
20.849	-0.65000
20.849	-0.65000
20.849	-0.65000
20.849	-0.65000
20.849	-0.65000
20.850	-0.65000
20.850	-0.65000
20.850	-0.65000
20.850	-0.65000
20.850	-0.65000
20.850	-0.65000
20.850	-0.65000
20.851	-0.65000
20.851	-0.65000
20.851	-0.65000
20.851	-0.65000
20.851	-0.65000
20.851	-0.65000
20.851	-0.65000

Time (s)	Tip potential during pulse (V)
20.851	-0.65000
20.852	-0.65000
20.852	-0.65000
20.852	-0.65000
20.852	-0.65000
20.852	-0.65000
20.852	-0.65000
20.852	-0.65000
20.853	-0.65000
20.853	-0.65000
20.853	-0.65000
20.853	-0.65000
20.853	-0.65000
20.853	-0.65000
20.853	-0.65000
20.853	-0.65000
20.853	-0.65000
20.854	-0.65000
20.854	-0.65000
20.854	-0.65000
20.854	-0.65000
20.854	-0.65000
20.854	-0.65000
20.854	-0.65000
20.855	-0.65000
20.855	-0.65000
20.855	-0.65000
20.855	-0.65000
20.855	-0.65000
20.855	-0.65000
20.855	-0.65000
20.856	-0.65000
20.856	-0.65000
20.856	-0.65000
20.856	-0.65000
20.856	-0.65000
20.856	-0.65000
20.856	-0.65000
20.856	-0.65000
20.857	-0.65000

Time (s)	Tip potential during pulse (V)
20.857	-0.65000
20.857	-0.65000
20.857	-0.65000
20.857	-0.65000
20.857	-0.65000
20.857	-0.65000
20.858	-0.65000
20.858	-0.65000
20.858	-0.65000
20.858	-0.65000
20.858	-0.65000
20.858	-0.65000
20.858	-0.65000
20.859	-0.65000
20.859	-0.65000
20.859	-0.65000
20.859	-0.65000
20.859	-0.65000
20.859	-0.65000
20.859	-0.65000
20.859	-0.65000
20.860	-0.65000
20.860	-0.65000
20.860	-0.65000
20.860	-0.65000
20.860	-0.65000
20.860	-0.65000
20.860	-0.65000
20.861	-0.65000
20.861	-0.65000
20.861	-0.65000
20.861	-0.65000
20.861	-0.65000
20.861	-0.65000
20.861	-0.65000
20.862	-0.65000
20.862	-0.65000
20.862	-0.65000
20.862	-0.65000
20.862	-0.65000

Time (s)	Tip potential during pulse (V)
20.862	-0.65000
20.862	-0.65000
20.862	-0.65000
20.863	-0.65000
20.863	-0.65000
20.863	-0.65000
20.863	-0.65000
20.863	-0.65000
20.863	-0.65000
20.863	-0.65000
20.864	-0.65000
20.864	-0.65000
20.864	-0.65000
20.864	-0.65000
20.864	-0.65000
20.864	-0.65000
20.864	-0.65000
20.865	-0.65000
20.865	-0.65000
20.865	-0.65000
20.865	-0.65000
20.865	-0.65000
20.865	-0.65000
20.865	-0.65000
20.865	-0.65000
20.866	-0.65000
20.866	-0.65000
20.866	-0.65000
20.866	-0.65000
20.866	-0.65000
20.866	-0.65000
20.866	-0.65000
20.867	-0.65000
20.867	-0.65000
20.867	-0.65000
20.867	-0.65000
20.867	-0.65000
20.867	-0.65000
20.867	-0.65000
20.868	-0.65000

Time (s)	Tip potential during pulse (V)
20.868	-0.65000
20.868	-0.65000
20.868	-0.65000
20.868	-0.65000
20.868	-0.65000
20.868	-0.65000
20.869	-0.65000
20.869	-0.65000
20.869	-0.65000
20.869	-0.65000
20.869	-0.65000
20.869	-0.65000
20.869	-0.65000
20.870	-0.65000
20.870	-0.65000
20.870	-0.65000
20.870	-0.65000
20.870	-0.65000
20.870	-0.65000
20.870	-0.65000
20.870	-0.65000
20.870	-0.65000
20.871	-0.65000
20.871	-0.65000
20.871	-0.65000
20.871	-0.65000
20.871	-0.65000
20.871	-0.65000
20.871	-0.65000
20.872	-0.65000
20.872	-0.65000
20.872	-0.65000
20.872	-0.65000
20.872	-0.65000
20.872	-0.65000
20.872	-0.65000
20.872	-0.65000
20.873	-0.65000
20.873	-0.65000
20.873	-0.65000

Time (s)	Tip potential during pulse (V)
20.873	-0.65000
20.873	-0.65000
20.873	-0.65000
20.873	-0.65000
20.874	-0.65000
20.874	-0.65000
20.874	-0.65000
20.874	-0.65000
20.874	-0.65000
20.874	-0.65000
20.874	-0.65000
20.875	-0.65000
20.875	-0.65000
20.875	-0.65000
20.875	-0.65000
20.875	-0.65000
20.875	-0.65000
20.875	-0.65000
20.875	-0.65000
20.876	-0.65000
20.876	-0.65000
20.876	-0.65000
20.876	-0.65000
20.876	-0.65000
20.876	-0.65000
20.876	-0.65000
20.876	-0.65000
20.877	-0.65000
20.877	-0.65000
20.877	-0.65000
20.877	-0.65000
20.877	-0.65000
20.877	-0.65000
20.877	-0.65000
20.878	-0.65000
20.878	-0.65000
20.878	-0.65000
20.878	-0.65000
20.878	-0.65000
20.878	-0.65000

Time (s)	Tip potential during pulse (V)
20.878	-0.65000
20.879	-0.65000
20.879	-0.65000
20.879	-0.65000
20.879	-0.65000
20.879	-0.65000
20.879	-0.65000
20.879	-0.65000
20.879	-0.65000
20.880	-0.65000
20.880	-0.65000
20.880	-0.65000
20.880	-0.65000
20.880	-0.65000
20.880	-0.65000
20.880	-0.65000
20.881	-0.65000
20.881	-0.65000
20.881	-0.65000
20.881	-0.65000
20.881	-0.65000
20.881	-0.65000
20.881	-0.65000
20.882	-0.65000
20.882	-0.65000
20.882	-0.65000
20.882	-0.65000
20.882	-0.65000
20.882	-0.65000
20.882	-0.65000
20.882	-0.65000
20.883	-0.65000
20.883	-0.65000
20.883	-0.65000
20.883	-0.65000
20.883	-0.65000
20.883	-0.65000
20.883	-0.65000
20.884	-0.65000
20.884	-0.65000

Time (s)	Tip potential during pulse (V)
20.884	-0.65000
20.884	-0.65000
20.884	-0.65000
20.884	-0.65000
20.884	-0.65000
20.884	-0.65000
20.884	-0.65000
20.885	-0.65000
20.885	-0.65000
20.885	-0.65000
20.885	-0.65000
20.885	-0.65000
20.885	-0.65000
20.885	-0.65000
20.886	-0.65000
20.886	-0.65000
20.886	-0.65000
20.886	-0.65000
20.886	-0.65000
20.886	-0.65000
20.886	-0.65000
20.886	-0.65000
20.886	-0.65000
20.887	-0.65000
20.887	-0.65000
20.887	-0.65000
20.887	-0.65000
20.887	-0.65000
20.887	-0.65000
20.887	-0.65000
20.887	-0.65000
20.888	-0.65000
20.888	-0.65000
20.888	-0.65000
20.888	-0.65000
20.888	-0.65000
20.888	-0.65000
20.888	-0.65000
20.888	-0.65000
20.889	-0.65000

Time (s)	Tip potential during pulse (V)
20.889	-0.65000
20.889	-0.65000
20.889	-0.65000
20.889	-0.65000
20.889	-0.65000
20.889	-0.65000
20.890	-0.65000
20.890	-0.65000
20.890	-0.65000
20.890	-0.65000
20.890	-0.65000
20.890	-0.65000
20.890	-0.65000
20.891	-0.65000
20.891	-0.65000
20.891	-0.65000
20.891	-0.65000
20.891	-0.65000
20.891	-0.65000
20.891	-0.65000
20.891	-0.65000
20.892	-0.65000
20.892	-0.65000
20.892	-0.65000
20.892	-0.65000
20.892	-0.65000
20.892	-0.65000
20.892	-0.65000
20.893	-0.65000
20.893	-0.65000
20.893	-0.65000
20.893	-0.65000
20.893	-0.65000
20.893	-0.65000
20.893	-0.65000
20.894	-0.65000
20.894	-0.65000
20.894	-0.65000
20.894	-0.65000
20.894	-0.65000

Time (s)	Tip potential during pulse (V)
20.894	-0.65000
20.894	-0.65000
20.894	-0.65000
20.895	-0.65000
20.895	-0.65000
20.895	-0.65000
20.895	-0.65000
20.895	-0.65000
20.895	-0.65000
20.895	-0.65000
20.896	-0.65000
20.896	-0.65000
20.896	-0.65000
20.896	-0.65000
20.896	-0.65000
20.896	-0.65000
20.896	-0.65000
20.897	-0.65000
20.897	-0.65000
20.897	-0.65000
20.897	-0.65000
20.897	-0.65000
20.897	-0.65000
20.897	-0.65000
20.897	-0.65000
20.898	-0.65000
20.898	-0.65000
20.898	-0.65000
20.898	-0.65000
20.898	-0.65000
20.898	-0.65000
20.898	-0.65000
20.899	-0.65000
20.899	-0.65000
20.899	-0.65000
20.899	-0.65000
20.899	-0.65000
20.899	-0.65000
20.899	-0.65000
20.900	-0.65000

Time (s)	Tip potential during pulse (V)
20.900	-0.65000
20.900	-0.65000
20.900	-0.65000
20.900	-0.65000
20.900	-0.65000
20.900	-0.65000
20.900	-0.65000
20.901	-0.65000
20.901	-0.65000
20.901	-0.65000
20.901	-0.65000
20.901	-0.65000
20.901	-0.65000
20.901	-0.65000
20.902	-0.65000
20.902	-0.65000
20.902	-0.65000
20.902	-0.65000
20.902	-0.65000
20.902	-0.65000
20.902	-0.65000
20.903	-0.65000
20.903	-0.65000
20.903	-0.65000
20.903	-0.65000
20.903	-0.65000
20.903	-0.65000
20.903	-0.65000
20.903	-0.65000
20.904	-0.65000
20.904	-0.65000
20.904	-0.65000
20.904	-0.65000
20.904	-0.65000
20.904	-0.65000
20.904	-0.65000
20.905	-0.65000
20.905	-0.65000
20.905	-0.65000
20.905	-0.65000

Time (s)	Tip potential during pulse (V)
20.905	-0.65000
20.905	-0.65000
20.905	-0.65000
20.906	-0.65000
20.906	-0.65000
20.906	-0.65000
20.906	-0.65000
20.906	-0.65000
20.906	-0.65000
20.906	-0.65000
20.906	-0.65000
20.907	-0.65000
20.907	-0.65000
20.907	-0.65000
20.907	-0.65000
20.907	-0.65000
20.907	-0.65000
20.907	-0.65000
20.907	-0.65000
20.908	-0.65000
20.908	-0.65000
20.908	-0.65000
20.908	-0.65000
20.908	-0.65000
20.908	-0.65000
20.908	-0.65000
20.908	-0.65000
20.909	-0.65000
20.909	-0.65000
20.909	-0.65000
20.909	-0.65000
20.909	-0.65000
20.909	-0.65000
20.909	-0.65000
20.910	-0.65000
20.910	-0.65000
20.910	-0.65000
20.910	-0.65000
20.910	-0.65000
20.910	-0.65000

Time (s)	Tip potential during pulse (V)
20.910	-0.65000
20.911	-0.65000
20.911	-0.65000
20.911	-0.65000
20.911	-0.65000
20.911	-0.65000
20.911	-0.65000
20.911	-0.65000
20.911	-0.65000
20.912	-0.65000
20.912	-0.65000
20.912	-0.65000
20.912	-0.65000
20.912	-0.65000
20.912	-0.65000
20.912	-0.65000
20.913	-0.65000
20.913	-0.65000
20.913	-0.65000
20.913	-0.65000
20.913	-0.65000
20.913	-0.65000
20.913	-0.65000
20.914	-0.65000
20.914	-0.65000
20.914	-0.65000
20.914	-0.65000
20.914	-0.65000
20.914	-0.65000
20.914	-0.65000
20.914	-0.65000
20.915	-0.65000
20.915	-0.65000
20.915	-0.65000
20.915	-0.65000
20.915	-0.65000
20.915	-0.65000
20.915	-0.65000
20.916	-0.65000
20.916	-0.65000

Time (s)	Tip potential during pulse (V)
20.916	-0.65000
20.916	-0.65000
20.916	-0.65000
20.916	-0.65000
20.916	-0.65000
20.917	-0.65000
20.917	-0.65000
20.917	-0.65000
20.917	-0.65000
20.917	-0.65000
20.917	-0.65000
20.917	-0.65000
20.917	-0.65000
20.918	-0.65000
20.918	-0.65000
20.918	-0.65000
20.918	-0.65000
20.918	-0.65000
20.918	-0.65000
20.918	-0.65000
20.918	-0.65000
20.919	-0.65000
20.919	-0.65000
20.919	-0.65000
20.919	-0.65000
20.919	-0.65000
20.919	-0.65000
20.919	-0.65000
20.920	-0.65000
20.920	-0.65000
20.920	-0.65000
20.920	-0.65000
20.920	-0.65000
20.920	-0.65000
20.920	-0.65000
20.921	-0.65000
20.921	-0.65000
20.921	-0.65000
20.921	-0.65000
20.921	-0.65000

Time (s)	Tip potential during pulse (V)
20.921	-0.65000
20.921	-0.65000
20.921	-0.65000
20.922	-0.65000
20.922	-0.65000
20.922	-0.65000
20.922	-0.65000
20.922	-0.65000
20.922	-0.65000
20.922	-0.65000
20.923	-0.65000
20.923	-0.65000
20.923	-0.65000
20.923	-0.65000
20.923	-0.65000
20.923	-0.65000
20.923	-0.65000
20.924	-0.65000
20.924	-0.65000
20.924	-0.65000
20.924	-0.65000
20.924	-0.65000
20.924	-0.65000
20.924	-0.65000
20.924	-0.65000
20.925	-0.65000
20.925	-0.65000
20.925	-0.65000
20.925	-0.65000
20.925	-0.65000
20.925	-0.65000
20.925	-0.65000
20.926	-0.65000
20.926	-0.65000
20.926	-0.65000
20.926	-0.65000
20.926	-0.65000
20.926	-0.65000
20.926	-0.65000
20.927	-0.65000

Time (s)	Tip potential during pulse (V)
20.927	-0.65000
20.927	-0.65000
20.927	-0.65000
20.927	-0.65000
20.927	-0.65000
20.927	-0.65000
20.927	-0.65000
20.928	-0.65000
20.928	-0.65000
20.928	-0.65000
20.928	-0.65000
20.928	-0.65000
20.928	-0.65000
20.928	-0.65000
20.929	-0.65000
20.929	-0.65000
20.929	-0.65000
20.929	-0.65000
20.929	-0.65000
20.929	-0.65000
20.929	-0.65000
20.930	-0.65000
20.930	-0.65000
20.930	-0.65000
20.930	-0.65000
20.930	-0.65000
20.930	-0.65000
20.930	-0.65000
20.930	-0.65000
20.931	-0.65000
20.931	-0.65000
20.931	-0.65000
20.931	-0.65000
20.931	-0.65000
20.931	-0.65000
20.931	-0.65000
20.932	-0.65000
20.932	-0.65000
20.932	-0.65000
20.932	-0.65000

Time (s)	Tip potential during pulse (V)
20.932	-0.65000
20.932	-0.65000
20.932	-0.65000
20.933	-0.65000
20.933	-0.65000
20.933	-0.65000
20.933	-0.65000
20.933	-0.65000
20.933	-0.65000
20.933	-0.65000
20.933	-0.65000
20.934	-0.65000
20.934	-0.65000
20.934	-0.65000
20.934	-0.65000
20.934	-0.65000
20.934	-0.65000
20.934	-0.65000
20.935	-0.65000
20.935	-0.65000
20.935	-0.65000
20.935	-0.65000
20.935	-0.65000
20.935	-0.65000
20.935	-0.65000
20.936	-0.65000
20.936	-0.65000
20.936	-0.65000
20.936	-0.65000
20.936	-0.65000
20.936	-0.65000
20.936	-0.65000
20.936	-0.65000
20.937	-0.65000
20.937	-0.65000
20.937	-0.65000
20.937	-0.65000
20.937	-0.65000
20.937	-0.65000
20.937	-0.65000

Time (s)	Tip potential during pulse (V)
20.938	-0.65000
20.938	-0.65000
20.938	-0.65000
20.938	-0.65000
20.938	-0.65000
20.938	-0.65000
20.938	-0.65000
20.939	-0.65000
20.939	-0.65000
20.939	-0.65000
20.939	-0.65000
20.939	-0.65000
20.939	-0.65000
20.939	-0.65000
20.939	-0.65000
20.940	-0.65000
20.940	-0.65000
20.940	-0.65000
20.940	-0.65000
20.940	-0.65000
20.940	-0.65000
20.940	-0.65000
20.941	-0.65000
20.941	-0.65000
20.941	-0.65000
20.941	-0.65000
20.941	-0.65000
20.941	-0.65000
20.941	-0.65000
20.942	-0.65000
20.942	-0.65000
20.942	-0.65000
20.942	-0.65000
20.942	-0.65000
20.942	-0.65000
20.942	-0.65000
20.942	-0.65000
20.943	-0.65000
20.943	-0.65000
20.943	-0.65000

Time (s)	Tip potential during pulse (V)
20.943	-0.65000
20.943	-0.65000
20.943	-0.65000
20.943	-0.65000
20.943	-0.65000
20.944	-0.65000
20.944	-0.65000
20.944	-0.65000
20.944	-0.65000
20.944	-0.65000
20.944	-0.65000
20.944	-0.65000
20.944	-0.65000
20.945	-0.65000
20.945	-0.65000
20.945	-0.65000
20.945	-0.65000
20.945	-0.65000
20.945	-0.65000
20.945	-0.65000
20.946	-0.65000
20.946	-0.65000
20.946	-0.65000
20.946	-0.65000
20.946	-0.65000
20.946	-0.65000
20.946	-0.65000
20.947	-0.65000
20.947	-0.65000
20.947	-0.65000
20.947	-0.65000
20.947	-0.65000
20.947	-0.65000
20.947	-0.65000
20.947	-0.65000
20.948	-0.65000
20.948	-0.65000
20.948	-0.65000
20.948	-0.65000
20.948	-0.65000

Time (s)	Tip potential during pulse (V)
20.948	-0.65000
20.948	-0.65000
20.949	-0.65000
20.949	-0.65000
20.949	-0.65000
20.949	-0.65000
20.949	-0.65000
20.949	-0.65000
20.949	-0.65000
20.950	-0.65000
20.950	-0.65000
20.950	-0.65000
20.950	-0.65000
20.950	-0.65000
20.950	-0.65000
20.950	-0.65000
20.950	-0.65000
20.951	-0.65000
20.951	-0.65000
20.951	-0.65000
20.951	-0.65000
20.951	-0.65000
20.951	-0.65000
20.951	-0.65000
20.952	-0.65000
20.952	-0.65000
20.952	-0.65000
20.952	-0.65000
20.952	-0.65000
20.952	-0.65000
20.952	-0.65000
20.952	-0.65000
20.952	-0.65000
20.952	-0.65000
20.953	-0.65000
20.953	-0.65000
20.953	-0.65000
20.953	-0.65000
20.953	-0.65000
20.953	-0.65000

Time (s)	Tip potential during pulse (V)
20.953	-0.65000
20.953	-0.65000
20.954	-0.65000
20.954	-0.65000
20.954	-0.65000
20.954	-0.65000
20.954	-0.65000
20.954	-0.65000
20.954	-0.65000
20.955	-0.65000
20.955	-0.65000
20.955	-0.65000
20.955	-0.65000
20.955	-0.65000
20.955	-0.65000
20.955	-0.65000
20.955	-0.65000
20.956	-0.65000
20.956	-0.65000
20.956	-0.65000
20.956	-0.65000
20.956	-0.65000
20.956	-0.65000
20.956	-0.65000
20.957	-0.65000
20.957	-0.65000
20.957	-0.65000
20.957	-0.65000
20.957	-0.65000
20.957	-0.65000
20.957	-0.65000
20.958	-0.65000
20.958	-0.65000
20.958	-0.65000
20.958	-0.65000
20.958	-0.65000
20.958	-0.65000
20.958	-0.65000
20.958	-0.65000
20.959	-0.65000

Time (s)	Tip potential during pulse (V)
20.959	-0.65000
20.959	-0.65000
20.959	-0.65000
20.959	-0.65000
20.959	-0.65000
20.959	-0.65000
20.960	-0.65000
20.960	-0.65000
20.960	-0.65000
20.960	-0.65000
20.960	-0.65000
20.960	-0.65000
20.960	-0.65000
20.960	-0.65000
20.960	-0.65000
20.960	-0.65000
20.961	-0.65000
20.961	-0.65000
20.961	-0.65000
20.961	-0.65000
20.961	-0.65000
20.961	-0.65000
20.961	-0.65000
20.962	-0.65000
20.962	-0.65000
20.962	-0.65000
20.962	-0.65000
20.962	-0.65000
20.962	-0.65000
20.962	-0.65000
20.962	-0.65000
20.963	-0.65000
20.963	-0.65000
20.963	-0.65000
20.963	-0.65000
20.963	-0.65000
20.963	-0.65000
20.963	-0.65000
20.964	-0.65000
20.964	-0.65000

Time (s)	Tip potential during pulse (V)
20.964	-0.65000
20.964	-0.65000
20.964	-0.65000
20.964	-0.65000
20.964	-0.65000
20.965	-0.65000
20.965	-0.65000
20.965	-0.65000
20.965	-0.65000
20.965	-0.65000
20.965	-0.65000
20.965	-0.65000
20.965	-0.65000
20.966	-0.65000
20.966	-0.65000
20.966	-0.65000
20.966	-0.65000
20.966	-0.65000
20.966	-0.65000
20.966	-0.65000
20.967	-0.65000
20.967	-0.65000
20.967	-0.65000
20.967	-0.65000
20.967	-0.65000
20.967	-0.65000
20.967	-0.65000
20.968	-0.65000
20.968	-0.65000
20.968	-0.65000
20.968	-0.65000
20.968	-0.65000
20.968	-0.65000
20.968	-0.65000
20.968	-0.65000
20.969	-0.65000
20.969	-0.65000
20.969	-0.65000
20.969	-0.65000
20.969	-0.65000

Time (s)	Tip potential during pulse (V)
20.969	-0.65000
20.970	-0.65000
20.970	-0.65000
20.970	-0.65000
20.970	-0.65000
20.970	-0.65000
20.970	-0.65000
20.970	-0.65000
20.970	-0.65000
20.971	-0.65000
20.971	-0.65000
20.971	-0.65000
20.971	-0.65000
20.971	-0.65000
20.971	-0.65000
20.971	-0.65000
20.972	-0.65000
20.972	-0.65000
20.972	-0.65000
20.972	-0.65000
20.972	-0.65000
20.972	-0.65000
20.972	-0.65000
20.973	-0.65000
20.973	-0.65000
20.973	-0.65000
20.973	-0.65000
20.973	-0.65000
20.973	-0.65000
20.973	-0.65000
20.973	-0.65000
20.974	-0.65000
20.974	-0.65000
20.974	-0.65000
20.974	-0.65000
20.974	-0.65000
20.974	-0.65000
20.974	-0.65000
20.975	-0.65000
20.975	-0.65000

Time (s)	Tip potential during pulse (V)
20.975	-0.65000
20.975	-0.65000
20.975	-0.65000
20.975	-0.65000
20.975	-0.65000
20.976	-0.65000
20.976	-0.65000
20.976	-0.65000
20.976	-0.65000
20.976	-0.65000
20.976	-0.65000
20.976	-0.65000
20.976	-0.65000
20.977	-0.65000
20.977	-0.65000
20.977	-0.65000
20.977	-0.65000
20.977	-0.65000
20.977	-0.65000
20.977	-0.65000
20.978	-0.65000
20.978	-0.65000
20.978	-0.65000
20.978	-0.65000
20.978	-0.65000
20.978	-0.65000
20.978	-0.65000
20.979	-0.65000
20.979	-0.65000
20.979	-0.65000
20.979	-0.65000
20.979	-0.65000
20.979	-0.65000
20.979	-0.65000
20.979	-0.65000
20.980	-0.65000
20.980	-0.65000
20.980	-0.65000
20.980	-0.65000
20.980	-0.65000

Time (s)	Tip potential during pulse (V)
20.980	-0.65000
20.980	-0.65000
20.981	-0.65000
20.981	-0.65000
20.981	-0.65000
20.981	-0.65000
20.981	-0.65000
20.981	-0.65000
20.981	-0.65000
20.981	-0.65000
20.981	-0.65000
20.982	-0.65000
20.982	-0.65000
20.982	-0.65000
20.982	-0.65000
20.982	-0.65000
20.982	-0.65000
20.982	-0.65000
20.983	-0.65000
20.983	-0.65000
20.983	-0.65000
20.983	-0.65000
20.983	-0.65000
20.983	-0.65000
20.983	-0.65000
20.984	-0.65000
20.984	-0.65000
20.984	-0.65000
20.984	-0.65000
20.984	-0.65000
20.984	-0.65000
20.984	-0.65000
20.984	-0.65000
20.985	-0.65000
20.985	-0.65000
20.985	-0.65000
20.985	-0.65000
20.985	-0.65000
20.985	-0.65000
20.985	-0.65000

Time (s)	Tip potential during pulse (V)
20.986	-0.65000
20.986	-0.65000
20.986	-0.65000
20.986	-0.65000
20.986	-0.65000
20.986	-0.65000
20.986	-0.65000
20.987	-0.65000
20.987	-0.65000
20.987	-0.65000
20.987	-0.65000
20.987	-0.65000
20.987	-0.65000
20.987	-0.65000
20.987	-0.65000
20.988	-0.65000
20.988	-0.65000
20.988	-0.65000
20.988	-0.65000
20.988	-0.65000
20.988	-0.65000
20.988	-0.65000
20.989	-0.65000
20.989	-0.65000
20.989	-0.65000
20.989	-0.65000
20.989	-0.65000
20.989	-0.65000
20.989	-0.65000
20.990	-0.65000
20.990	-0.65000
20.990	-0.65000
20.990	-0.65000
20.990	-0.65000
20.990	-0.65000
20.990	-0.65000
20.990	-0.65000
20.991	-0.65000
20.991	-0.65000
20.991	-0.65000

Time (s)	Tip potential during pulse (V)
20.991	-0.65000
20.991	-0.65000
20.991	-0.65000
20.991	-0.65000
20.992	-0.65000
20.992	-0.65000
20.992	-0.65000
20.992	-0.65000
20.992	-0.65000
20.992	-0.65000
20.992	-0.65000
20.993	-0.65000
20.993	-0.65000
20.993	-0.65000
20.993	-0.65000
20.993	-0.65000
20.993	-0.65000
20.993	-0.65000
20.993	-0.65000
20.994	-0.65000
20.994	-0.65000
20.994	-0.65000
20.994	-0.65000
20.994	-0.65000
20.994	-0.65000
20.995	-0.65000
20.995	-0.65000
20.995	-0.65000
20.995	-0.65000
20.995	-0.65000
20.995	-0.65000
20.995	-0.65000
20.995	-0.65000
20.996	-0.65000
20.996	-0.65000
20.996	-0.65000
20.996	-0.65000
20.996	-0.65000
20.996	-0.65000
20.996	-0.65000

Time (s)	Tip potential during pulse (V)
20.997	-0.65000
20.997	-0.65000
20.997	-0.65000
20.997	-0.65000
20.997	-0.65000
20.997	-0.65000
20.997	-0.65000
20.998	-0.65000
20.998	-0.65000
20.998	-0.65000
20.998	-0.65000
20.998	-0.65000
20.998	-0.65000
20.998	-0.65000
20.998	-0.65000
20.999	-0.65000
20.999	-0.65000
20.999	-0.65000
20.999	-0.65000
20.999	-0.65000
20.999	-0.65000
20.999	-0.65000
20.999	-0.65000
21.000	-0.65000
21.000	-0.65000
21.000	-0.65000
21.000	-0.65000
21.000	-0.65000
21.000	-0.65000
21.000	-0.65000
21.000	-0.65000
21.001	-0.65000
21.001	-0.65000
21.001	-0.65000
21.001	-0.65000
21.001	-0.65000
21.001	-0.65000
21.001	-0.65000
21.002	-0.65000
21.002	-0.65000

Time (s)	Tip potential during pulse (V)
21.002	-0.65000
21.002	-0.65000
21.002	-0.65000
21.002	-0.65000
21.002	-0.65000
21.003	-0.65000
21.003	-0.65000
21.003	-0.65000
21.003	-0.65000
21.003	-0.65000
21.003	-0.65000
21.003	-0.65000
21.003	-0.65000
21.004	-0.65000
21.004	-0.65000
21.004	-0.65000
21.004	-0.65000
21.004	-0.65000
21.004	-0.65000
21.004	-0.65000
21.005	-0.65000
21.005	-0.65000
21.005	-0.65000
21.005	-0.65000
21.005	-0.65000
21.005	-0.65000
21.005	-0.65000
21.006	-0.65000
21.006	-0.65000
21.006	-0.65000
21.006	-0.65000
21.006	-0.65000
21.006	-0.65000
21.006	-0.65000
21.006	-0.65000
21.007	-0.65000
21.007	-0.65000
21.007	-0.65000
21.007	-0.65000
21.007	-0.65000

Time (s)	Tip potential during pulse (V)
21.007	-0.65000
21.007	-0.65000
21.008	-0.65000
21.008	-0.65000
21.008	-0.65000
21.008	-0.65000
21.008	-0.65000
21.008	-0.65000
21.008	-0.65000
21.009	-0.65000
21.009	-0.65000
21.009	-0.65000
21.009	-0.65000
21.009	-0.65000
21.009	-0.65000
21.009	-0.65000
21.009	-0.65000
21.010	-0.65000
21.010	-0.65000
21.010	-0.65000
21.010	-0.65000
21.010	-0.65000
21.010	-0.65000
21.010	-0.65000
21.011	-0.65000
21.011	-0.65000
21.011	-0.65000
21.011	-0.65000
21.011	-0.65000
21.011	-0.65000
21.011	-0.65000
21.012	-0.65000
21.012	-0.65000
21.012	-0.65000
21.012	-0.65000
21.012	-0.65000
21.012	-0.65000
21.012	-0.65000
21.012	-0.65000
21.013	-0.65000

Time (s)	Tip potential during pulse (V)
21.013	-0.65000
21.013	-0.65000
21.013	-0.65000
21.013	-0.65000
21.013	-0.65000
21.013	-0.65000
21.014	-0.65000
21.014	-0.65000
21.014	-0.65000
21.014	-0.65000
21.014	-0.65000
21.014	-0.65000
21.014	-0.65000
21.015	-0.65000
21.015	-0.65000
21.015	-0.65000
21.015	-0.65000
21.015	-0.65000
21.015	-0.65000
21.015	-0.65000
21.015	-0.65000
21.016	-0.65000
21.016	-0.65000
21.016	-0.65000
21.016	-0.65000
21.016	-0.65000
21.016	-0.65000
21.016	-0.65000
21.017	-0.65000
21.017	-0.65000
21.017	-0.65000
21.017	-0.65000
21.017	-0.65000
21.017	-0.65000
21.017	-0.65000
21.018	-0.65000
21.018	-0.65000
21.018	-0.65000
21.018	-0.65000
21.018	-0.65000

Time (s)	Tip potential during pulse (V)
21.018	-0.65000
21.018	-0.65000
21.018	-0.65000
21.019	-0.65000
21.019	-0.65000
21.019	-0.65000
21.019	-0.65000
21.019	-0.65000
21.019	-0.65000
21.019	-0.65000
21.020	-0.65000
21.020	-0.65000
21.020	-0.65000
21.020	-0.65000
21.020	-0.65000
21.020	-0.65000
21.020	-0.65000
21.021	-0.65000
21.021	-0.65000
21.021	-0.65000
21.021	-0.65000
21.021	-0.65000
21.021	-0.65000
21.021	-0.65000
21.022	-0.65000
21.022	-0.65000
21.022	-0.65000
21.022	-0.65000
21.022	-0.65000
21.022	-0.65000
21.022	-0.65000
21.023	-0.65000
21.023	-0.65000
21.023	-0.65000
21.023	-0.65000
21.023	-0.65000
21.023	-0.65000
21.023	-0.65000
21.023	-0.65000
21.024	-0.65000

Time (s)	Tip potential during pulse (V)
21.024	-0.65000
21.024	-0.65000
21.024	-0.65000
21.024	-0.65000
21.024	-0.65000
21.024	-0.65000
21.025	-0.65000
21.025	-0.65000
21.025	-0.65000
21.025	-0.65000
21.025	-0.65000
21.025	-0.65000
21.025	-0.65000
21.026	-0.65000
21.026	-0.65000
21.026	-0.65000
21.026	-0.65000
21.026	-0.65000
21.026	-0.65000
21.026	-0.65000
21.026	-0.65000
21.027	-0.65000
21.027	-0.65000
21.027	-0.65000
21.027	-0.65000
21.027	-0.65000
21.027	-0.65000
21.027	-0.65000
21.028	-0.65000
21.028	-0.65000
21.028	-0.65000
21.028	-0.65000
21.028	-0.65000
21.028	-0.65000
21.028	-0.65000
21.029	-0.65000
21.029	-0.65000
21.029	-0.65000
21.029	-0.65000
21.029	-0.65000

Time (s)	Tip potential during pulse (V)
21.029	-0.65000
21.029	-0.65000
21.029	-0.65000
21.030	-0.65000
21.030	-0.65000
21.030	-0.65000
21.030	-0.65000
21.030	-0.65000
21.030	-0.65000
21.030	-0.65000
21.031	-0.65000
21.031	-0.65000
21.031	-0.65000
21.031	-0.65000
21.031	-0.65000
21.031	-0.65000
21.031	-0.65000
21.031	-0.65000
21.031	-0.65000
21.032	-0.65000
21.032	-0.65000
21.032	-0.65000
21.032	-0.65000
21.032	-0.65000
21.032	-0.65000
21.032	-0.65000
21.033	-0.65000
21.033	-0.65000
21.033	-0.65000
21.033	-0.65000
21.033	-0.65000
21.033	-0.65000
21.033	-0.65000
21.034	-0.65000
21.034	-0.65000
21.034	-0.65000
21.034	-0.65000
21.034	-0.65000
21.034	-0.65000
21.034	-0.65000

Time (s)	Tip potential during pulse (V)
21.034	-0.65000
21.035	-0.65000
21.035	-0.65000
21.035	-0.65000
21.035	-0.65000
21.035	-0.65000
21.035	-0.65000
21.035	-0.65000
21.036	-0.65000
21.036	-0.65000
21.036	-0.65000
21.036	-0.65000
21.036	-0.65000
21.036	-0.65000
21.036	-0.65000
21.037	-0.65000
21.037	-0.65000
21.037	-0.65000
21.037	-0.65000
21.037	-0.65000
21.037	-0.65000
21.037	-0.65000
21.037	-0.65000
21.038	-0.65000
21.038	-0.65000
21.038	-0.65000
21.038	-0.65000
21.038	-0.65000
21.038	-0.65000
21.038	-0.65000
21.039	-0.65000
21.039	-0.65000
21.039	-0.65000
21.039	-0.65000
21.039	-0.65000
21.039	-0.65000
21.039	-0.65000
21.039	-0.65000
21.039	-0.65000
21.040	-0.65000

Time (s)	Tip potential during pulse (V)
21.040	-0.65000
21.040	-0.65000
21.040	-0.65000
21.040	-0.65000
21.040	-0.65000
21.040	-0.65000
21.041	-0.65000
21.041	-0.65000
21.041	-0.65000
21.041	-0.65000
21.041	-0.65000
21.041	-0.65000
21.041	-0.65000
21.042	-0.65000
21.042	-0.65000
21.042	-0.65000
21.042	-0.65000
21.042	-0.65000
21.042	-0.65000
21.042	-0.65000
21.042	-0.65000
21.043	-0.65000
21.043	-0.65000
21.043	-0.65000
21.043	-0.65000
21.043	-0.65000
21.043	-0.65000
21.043	-0.65000
21.044	-0.65000
21.044	-0.65000
21.044	-0.65000
21.044	-0.65000
21.044	-0.65000
21.044	-0.65000
21.044	-0.65000
21.045	-0.65000
21.045	-0.65000
21.045	-0.65000
21.045	-0.65000
21.045	-0.65000

Time (s)	Tip potential during pulse (V)
21.045	-0.65000
21.045	-0.65000
21.045	-0.65000
21.046	-0.65000
21.046	-0.65000
21.046	-0.65000
21.046	-0.65000
21.046	-0.65000
21.046	-0.65000
21.046	-0.65000
21.046	-0.65000
21.047	-0.65000
21.047	-0.65000
21.047	-0.65000
21.047	-0.65000
21.047	-0.65000
21.047	-0.65000
21.047	-0.65000
21.048	-0.65000
21.048	-0.65000
21.048	-0.65000
21.048	-0.65000
21.048	-0.65000
21.048	-0.65000
21.048	-0.65000
21.049	-0.65000
21.049	-0.65000
21.049	-0.65000
21.049	-0.65000
21.049	-0.65000
21.049	-0.65000
21.049	-0.65000
21.049	-0.65000
21.050	-0.65000
21.050	-0.65000
21.050	-0.65000
21.050	-0.65000
21.050	-0.65000
21.050	-0.65000
21.050	-0.65000

Time (s)	Tip potential during pulse (V)
21.051	-0.65000
21.051	-0.65000
21.051	-0.65000
21.051	-0.65000
21.051	-0.65000
21.051	-0.65000
21.051	-0.65000
21.052	-0.65000
21.052	-0.65000
21.052	-0.65000
21.052	-0.65000
21.052	-0.65000
21.052	-0.65000
21.052	-0.65000
21.052	-0.65000
21.053	-0.65000
21.053	-0.65000
21.053	-0.65000
21.053	-0.65000
21.053	-0.65000
21.053	-0.65000
21.053	-0.65000
21.054	-0.65000
21.054	-0.65000
21.054	-0.65000
21.054	-0.65000
21.054	-0.65000
21.054	-0.65000
21.054	-0.65000
21.055	-0.65000
21.055	-0.65000
21.055	-0.65000
21.055	-0.65000
21.055	-0.65000
21.055	-0.65000
21.055	-0.65000
21.055	-0.65000
21.056	-0.65000
21.056	-0.65000
21.056	-0.65000

Time (s)	Tip potential during pulse (V)
21.056	-0.65000
21.056	-0.65000
21.056	-0.65000
21.056	-0.65000
21.056	-0.65000
21.057	-0.65000
21.057	-0.65000
21.057	-0.65000
21.057	-0.65000
21.057	-0.65000
21.057	-0.65000
21.057	-0.65000
21.057	-0.65000
21.058	-0.65000
21.058	-0.65000
21.058	-0.65000
21.058	-0.65000
21.058	-0.65000
21.058	-0.65000
21.059	-0.65000
21.059	-0.65000
21.059	-0.65000
21.059	-0.65000
21.059	-0.65000
21.059	-0.65000
21.059	-0.65000
21.059	-0.65000
21.060	-0.65000
21.060	-0.65000
21.060	-0.65000
21.060	-0.65000
21.060	-0.65000
21.060	-0.65000
21.060	-0.65000
21.060	-0.65000
21.060	-0.65000
21.061	-0.65000
21.061	-0.65000
21.061	-0.65000
21.061	-0.65000

Time (s)	Tip potential during pulse (V)
21.061	-0.65000
21.061	-0.65000
21.061	-0.65000
21.062	-0.65000
21.062	-0.65000
21.062	-0.65000
21.062	-0.65000
21.062	-0.65000
21.062	-0.65000
21.062	-0.65000
21.062	-0.65000
21.063	-0.65000
21.063	-0.65000
21.063	-0.65000
21.063	-0.65000
21.063	-0.65000
21.063	-0.65000
21.063	-0.65000
21.064	-0.65000
21.064	-0.65000
21.064	-0.65000
21.064	-0.65000
21.064	-0.65000
21.064	-0.65000
21.064	-0.65000
21.065	-0.65000
21.065	-0.65000
21.065	-0.65000
21.065	-0.65000
21.065	-0.65000
21.065	-0.65000
21.065	-0.65000
21.065	-0.65000
21.066	-0.65000
21.066	-0.65000
21.066	-0.65000
21.066	-0.65000
21.066	-0.65000
21.066	-0.65000
21.066	-0.65000

Time (s)	Tip potential during pulse (V)
21.067	-0.65000
21.067	-0.65000
21.067	-0.65000
21.067	-0.65000
21.067	-0.65000
21.067	-0.65000
21.067	-0.65000
21.067	-0.65000
21.067	-0.65000
21.068	-0.65000
21.068	-0.65000
21.068	-0.65000
21.068	-0.65000
21.068	-0.65000
21.068	-0.65000
21.068	-0.65000
21.069	-0.65000
21.069	-0.65000
21.069	-0.65000
21.069	-0.65000
21.069	-0.65000
21.069	-0.65000
21.069	-0.65000
21.070	-0.65000
21.070	-0.65000
21.070	-0.65000
21.070	-0.65000
21.070	-0.65000
21.070	-0.65000
21.070	-0.65000
21.070	-0.65000
21.071	-0.65000
21.071	-0.65000
21.071	-0.65000
21.071	-0.65000
21.071	-0.65000
21.071	-0.65000
21.071	-0.65000
21.072	-0.65000
21.072	-0.65000

Time (s)	Tip potential during pulse (V)
21.072	-0.65000
21.072	-0.65000
21.072	-0.65000
21.072	-0.65000
21.072	-0.65000
21.073	-0.65000
21.073	-0.65000
21.073	-0.65000
21.073	-0.65000
21.073	-0.65000
21.073	-0.65000
21.073	-0.65000
21.073	-0.65000
21.074	-0.65000
21.074	-0.65000
21.074	-0.65000
21.074	-0.65000
21.074	-0.65000
21.074	-0.65000
21.074	-0.65000
21.075	-0.65000
21.075	-0.65000
21.075	-0.65000
21.075	-0.65000
21.075	-0.65000
21.075	-0.65000
21.075	-0.65000
21.076	-0.65000
21.076	-0.65000
21.076	-0.65000
21.076	-0.65000
21.076	-0.65000
21.076	-0.65000
21.076	-0.65000
21.076	-0.65000
21.077	-0.65000
21.077	-0.65000
21.077	-0.65000
21.077	-0.65000
21.077	-0.65000

Time (s)	Tip potential during pulse (V)
21.077	-0.65000
21.077	-0.65000
21.078	-0.65000
21.078	-0.65000
21.078	-0.65000
21.078	-0.65000
21.078	-0.65000
21.078	-0.65000
21.078	-0.65000
21.079	-0.65000
21.079	-0.65000
21.079	-0.65000
21.079	-0.65000
21.079	-0.65000
21.079	-0.65000
21.079	-0.65000
21.079	-0.65000
21.080	-0.65000
21.080	-0.65000
21.080	-0.65000
21.080	-0.65000
21.080	-0.65000
21.080	-0.65000
21.080	-0.65000
21.081	-0.65000
21.081	-0.65000
21.081	-0.65000
21.081	-0.65000
21.081	-0.65000
21.081	-0.65000
21.081	-0.65000
21.082	-0.65000
21.082	-0.65000
21.082	-0.65000
21.082	-0.65000
21.082	-0.65000
21.082	-0.65000
21.082	-0.65000
21.082	-0.65000
21.083	-0.65000

Time (s)	Tip potential during pulse (V)
21.083	-0.65000
21.083	-0.65000
21.083	-0.65000
21.083	-0.65000
21.083	-0.65000
21.083	-0.65000
21.083	-0.65000
21.084	-0.65000
21.084	-0.65000
21.084	-0.65000
21.084	-0.65000
21.084	-0.65000
21.084	-0.65000
21.084	-0.65000
21.085	-0.65000
21.085	-0.65000
21.085	-0.65000
21.085	-0.65000
21.085	-0.65000
21.085	-0.65000
21.085	-0.65000
21.086	-0.65000
21.086	-0.65000
21.086	-0.65000
21.086	-0.65000
21.086	-0.65000
21.086	-0.65000
21.086	-0.65000
21.086	-0.65000
21.087	-0.65000
21.087	-0.65000
21.087	-0.65000
21.087	-0.65000
21.087	-0.65000
21.087	-0.65000
21.087	-0.65000
21.088	-0.65000
21.088	-0.65000
21.088	-0.65000
21.088	-0.65000

Time (s)	Tip potential during pulse (V)
21.088	-0.65000
21.088	-0.65000
21.088	-0.65000
21.089	-0.65000
21.089	-0.65000
21.089	-0.65000
21.089	-0.65000
21.089	-0.65000
21.089	-0.65000
21.089	-0.65000
21.089	-0.65000
21.090	-0.65000
21.090	-0.65000
21.090	-0.65000
21.090	-0.65000
21.090	-0.65000
21.090	-0.65000
21.090	-0.65000
21.091	-0.65000
21.091	-0.65000
21.091	-0.65000
21.091	-0.65000
21.091	-0.65000
21.091	-0.65000
21.091	-0.65000
21.092	-0.65000
21.092	-0.65000
21.092	-0.65000
21.092	-0.65000
21.092	-0.65000
21.092	-0.65000
21.092	-0.65000
21.092	-0.65000
21.093	-0.65000
21.093	-0.65000
21.093	-0.65000
21.093	-0.65000
21.093	-0.65000
21.093	-0.65000
21.093	-0.65000

Time (s)	Tip potential during pulse (V)
21.094	-0.65000
21.094	-0.65000
21.094	-0.65000
21.094	-0.65000
21.094	-0.65000
21.094	-0.65000
21.094	-0.65000
21.095	-0.65000
21.095	-0.65000
21.095	-0.65000
21.095	-0.65000
21.095	-0.65000
21.095	-0.65000
21.095	-0.65000
21.095	-0.65000
21.096	-0.65000
21.096	-0.65000
21.096	-0.65000
21.096	-0.65000
21.096	-0.65000
21.096	-0.65000
21.096	-0.65000
21.096	-0.65000
21.097	-0.65000
21.097	-0.65000
21.097	-0.65000
21.097	-0.65000
21.097	-0.65000
21.097	-0.65000
21.097	-0.65000
21.098	-0.65000
21.098	-0.65000
21.098	-0.65000
21.098	-0.65000
21.098	-0.65000
21.098	-0.65000
21.098	-0.65000
21.099	-0.65000
21.099	-0.65000
21.099	-0.65000

Time (s)	Tip potential during pulse (V)
21.099	-0.65000
21.099	-0.65000
21.099	-0.65000
21.099	-0.65000
21.099	-0.65000
21.100	-0.65000
21.100	-0.65000
21.100	-0.65000
21.100	-0.65000
21.100	-0.65000
21.100	-0.65000
21.100	-0.65000
21.101	-0.65000
21.101	-0.65000
21.101	-0.65000
21.101	-0.65000
21.101	-0.65000
21.101	-0.65000
21.101	-0.65000
21.101	-0.65000
21.101	-0.65000
21.102	-0.65000
21.102	-0.65000
21.102	-0.65000
21.102	-0.65000
21.102	-0.65000
21.102	-0.65000
21.102	-0.65000
21.103	-0.65000
21.103	-0.65000
21.103	-0.65000
21.103	-0.65000
21.103	-0.65000
21.103	-0.65000
21.103	-0.65000
21.104	-0.65000
21.104	-0.65000
21.104	-0.65000
21.104	-0.65000
21.104	-0.65000

Time (s)	Tip potential during pulse (V)
21.104	-0.65000
21.104	-0.65000
21.104	-0.65000
21.105	-0.65000
21.105	-0.65000
21.105	-0.65000
21.105	-0.65000
21.105	-0.65000
21.105	-0.65000
21.105	-0.65000
21.106	-0.65000
21.106	-0.65000
21.106	-0.65000
21.106	-0.65000
21.106	-0.65000
21.106	-0.65000
21.106	-0.65000
21.107	-0.65000
21.107	-0.65000
21.107	-0.65000
21.107	-0.65000
21.107	-0.65000
21.107	-0.65000
21.107	-0.65000
21.107	-0.65000
21.108	-0.65000
21.108	-0.65000
21.108	-0.65000
21.108	-0.65000
21.108	-0.65000
21.108	-0.65000
21.109	-0.65000
21.109	-0.65000
21.109	-0.65000
21.109	-0.65000
21.109	-0.65000
21.109	-0.65000
21.109	-0.65000
21.109	-0.65000
21.110	-0.65000

Time (s)	Tip potential during pulse (V)
21.110	-0.65000
21.110	-0.65000
21.110	-0.65000
21.110	-0.65000
21.110	-0.65000
21.110	-0.65000
21.111	-0.65000
21.111	-0.65000
21.111	-0.65000
21.111	-0.65000
21.111	-0.65000
21.111	-0.65000
21.111	-0.65000
21.112	-0.65000
21.112	-0.65000
21.112	-0.65000
21.112	-0.65000
21.112	-0.65000
21.112	-0.65000
21.112	-0.65000
21.112	-0.65000
21.113	-0.65000
21.113	-0.65000
21.113	-0.65000
21.113	-0.65000
21.113	-0.65000
21.113	-0.65000
21.113	-0.65000
21.114	-0.65000
21.114	-0.65000
21.114	-0.65000
21.114	-0.65000
21.114	-0.65000
21.114	-0.65000
21.114	-0.65000
21.115	-0.65000
21.115	-0.65000
21.115	-0.65000
21.115	-0.65000
21.115	-0.65000

Time (s)	Tip potential during pulse (V)
21.115	-0.65000
21.115	-0.65000
21.115	-0.65000
21.116	-0.65000
21.116	-0.65000
21.116	-0.65000
21.116	-0.65000
21.116	-0.65000
21.116	-0.65000
21.116	-0.65000
21.117	-0.65000
21.117	-0.65000
21.117	-0.65000
21.117	-0.65000
21.117	-0.65000
21.117	-0.65000
21.117	-0.65000
21.118	-0.65000
21.118	-0.65000
21.118	-0.65000
21.118	-0.65000
21.118	-0.65000
21.118	-0.65000
21.118	-0.65000
21.118	-0.65000
21.118	-0.65000
21.118	-0.65000
21.119	-0.65000
21.119	-0.65000
21.119	-0.65000
21.119	-0.65000
21.119	-0.65000
21.119	-0.65000
21.119	-0.65000
21.120	-0.65000
21.120	-0.65000
21.120	-0.65000
21.120	-0.65000
21.120	-0.65000
21.120	-0.65000

Time (s)	Tip potential during pulse (V)
21.120	-0.65000
21.121	-0.65000
21.121	-0.65000
21.121	-0.65000
21.121	-0.65000
21.121	-0.65000
21.121	-0.65000
21.121	-0.65000
21.122	-0.65000
21.122	-0.65000
21.122	-0.65000
21.122	-0.65000
21.122	-0.65000
21.122	-0.65000
21.122	-0.65000
21.123	-0.65000
21.123	-0.65000
21.123	-0.65000
21.123	-0.65000
21.123	-0.65000
21.123	-0.65000
21.123	-0.65000
21.123	-0.65000
21.124	-0.65000
21.124	-0.65000
21.124	-0.65000
21.124	-0.65000
21.124	-0.65000
21.124	-0.65000
21.124	-0.65000
21.125	-0.65000
21.125	-0.65000
21.125	-0.65000
21.125	-0.65000
21.125	-0.65000
21.125	-0.65000
21.125	-0.65000
21.126	-0.65000
21.126	-0.65000
21.126	-0.65000

Time (s)	Tip potential during pulse (V)
21.126	-0.65000
21.126	-0.65000
21.126	-0.65000
21.126	-0.65000
21.126	-0.65000
21.127	-0.65000
21.127	-0.65000
21.127	-0.65000
21.127	-0.65000
21.127	-0.65000
21.127	-0.65000
21.127	-0.65000
21.128	-0.65000
21.128	-0.65000
21.128	-0.65000
21.128	-0.65000
21.128	-0.65000
21.128	-0.65000
21.128	-0.65000
21.129	-0.65000
21.129	-0.65000
21.129	-0.65000
21.129	-0.65000
21.129	-0.65000
21.129	-0.65000
21.129	-0.65000
21.129	-0.65000
21.130	-0.65000
21.130	-0.65000
21.130	-0.65000
21.130	-0.65000
21.130	-0.65000
21.130	-0.65000
21.130	-0.65000
21.131	-0.65000
21.131	-0.65000
21.131	-0.65000
21.131	-0.65000
21.131	-0.65000
21.131	-0.65000

Time (s)	Tip potential during pulse (V)
21.131	-0.65000
21.132	-0.65000
21.132	-0.65000
21.132	-0.65000
21.132	-0.65000
21.132	-0.65000
21.132	-0.65000
21.132	-0.65000
21.132	-0.65000
21.133	-0.65000
21.133	-0.65000
21.133	-0.65000
21.133	-0.65000
21.133	-0.65000
21.133	-0.65000
21.133	-0.65000
21.134	-0.65000
21.134	-0.65000
21.134	-0.65000
21.134	-0.65000
21.134	-0.65000
21.134	-0.65000
21.134	-0.65000
21.135	-0.65000
21.135	-0.65000
21.135	-0.65000
21.135	-0.65000
21.135	-0.65000
21.135	-0.65000
21.135	-0.65000
21.135	-0.65000
21.136	-0.65000
21.136	-0.65000
21.136	-0.65000
21.136	-0.65000
21.136	-0.65000
21.136	-0.65000
21.136	-0.65000
21.137	-0.65000
21.137	-0.65000

Time (s)	Tip potential during pulse (V)
21.137	-0.65000
21.137	-0.65000
21.137	-0.65000
21.137	-0.65000
21.137	-0.65000
21.138	-0.65000
21.138	-0.65000
21.138	-0.65000
21.138	-0.65000
21.138	-0.65000
21.138	-0.65000
21.138	-0.65000
21.138	-0.65000
21.139	-0.65000
21.139	-0.65000
21.139	-0.65000
21.139	-0.65000
21.139	-0.65000
21.139	-0.65000
21.139	-0.65000
21.140	-0.65000
21.140	-0.65000
21.140	-0.65000
21.140	-0.65000
21.140	-0.65000
21.140	-0.65000
21.140	-0.65000
21.141	-0.65000
21.141	-0.65000
21.141	-0.65000
21.141	-0.65000
21.141	-0.65000
21.141	-0.65000
21.141	-0.65000
21.141	-0.65000
21.142	-0.65000
21.142	-0.65000
21.142	-0.65000
21.142	-0.65000
21.142	-0.65000

Time (s)	Tip potential during pulse (V)
21.142	-0.65000
21.142	-0.65000
21.143	-0.65000
21.143	-0.65000
21.143	-0.65000
21.143	-0.65000
21.143	-0.65000
21.143	-0.65000
21.143	-0.65000
21.143	-0.65000
21.143	-0.65000
21.144	-0.65000
21.144	-0.65000
21.144	-0.65000
21.144	-0.65000
21.144	-0.65000
21.144	-0.65000
21.144	-0.65000
21.145	-0.65000
21.145	-0.65000
21.145	-0.65000
21.145	-0.65000
21.145	-0.65000
21.145	-0.65000
21.145	-0.65000
21.146	-0.65000
21.146	-0.65000
21.146	-0.65000
21.146	-0.65000
21.146	-0.65000
21.146	-0.65000
21.146	-0.65000
21.147	-0.65000
21.147	-0.65000
21.147	-0.65000
21.147	-0.65000
21.147	-0.65000
21.147	-0.65000
21.147	-0.65000
21.147	-0.65000

Time (s)	Tip potential during pulse (V)
21.147	-0.65000
21.148	-0.65000
21.148	-0.65000
21.148	-0.65000
21.148	-0.65000
21.148	-0.65000
21.148	-0.65000
21.148	-0.65000
21.149	-0.65000
21.149	-0.65000
21.149	-0.65000
21.149	-0.65000
21.149	-0.65000
21.149	-0.65000
21.149	-0.65000
21.150	-0.65000
21.150	-0.65000
21.150	-0.65000
21.150	-0.65000
21.150	-0.65000
21.150	-0.65000
21.150	-0.65000
21.150	-0.65000
21.150	-0.65000

4.3.7 Table 8

Substrate Potential (cRedSub)

d (um)	Probe variable cRedSub (mol/m^3)
30.000	0.11307
29.799	0.11389
29.598	0.11474
29.397	0.11559
29.196	0.11645
28.995	0.11733
28.794	0.11818
28.593	0.11909
28.392	0.11982
28.191	0.12093
27.990	0.12182

d (um)	Probe variable cRedSub (mol/m^3)
27.789	0.12284
27.588	0.12379
27.387	0.12460
27.186	0.12578
26.985	0.12678
26.784	0.12779
26.583	0.12886
26.382	0.12989
26.181	0.13098
25.980	0.13202
25.779	0.13313
25.578	0.13423
25.377	0.13541
25.176	0.13655
24.975	0.13774
24.774	0.13890
24.573	0.14008
24.372	0.14134
24.171	0.14258
23.970	0.14384
23.769	0.14516
23.568	0.14643
23.367	0.14776
23.166	0.14909
22.965	0.15047
22.764	0.15187
22.563	0.15331
22.362	0.15474
22.161	0.15620
21.960	0.15768
21.759	0.15922
21.558	0.16077
21.357	0.16225
21.156	0.16395
20.955	0.16555
20.754	0.16726
20.553	0.16892
20.352	0.17068
20.151	0.17230
19.950	0.17420

d (um)	Probe variable cRedSub (mol/m^3)
19.749	0.17605
19.548	0.17791
19.347	0.17961
19.146	0.18170
18.945	0.18365
18.744	0.18570
18.543	0.18774
18.342	0.18980
18.141	0.19180
17.940	0.19409
17.739	0.19633
17.538	0.19856
17.337	0.20085
17.136	0.20318
16.935	0.20556
16.734	0.20802
16.533	0.21052
16.332	0.21310
16.131	0.21569
15.930	0.21835
15.729	0.22107
15.528	0.22383
15.327	0.22667
15.126	0.22953
14.924	0.23250
14.723	0.23559
14.522	0.23870
14.321	0.24183
14.120	0.24509
13.919	0.24844
13.718	0.25181
13.517	0.25536
13.316	0.25890
13.115	0.26251
12.914	0.26624
12.713	0.27004
12.512	0.27396
12.311	0.27802
12.110	0.28214
11.909	0.28639

d (um)	Probe variable cRedSub (mol/m^3)
11.708	0.29067
11.507	0.29506
11.306	0.29965
11.105	0.30434
10.904	0.30906
10.703	0.31406
10.502	0.31904
10.301	0.32422
10.100	0.32959
9.8993	0.33510
9.6983	0.34067
9.4973	0.34642
9.2963	0.35231
9.0953	0.35844
8.8943	0.36467
8.6933	0.37099
8.4923	0.37771
8.2913	0.38448
8.0903	0.39146
7.8893	0.39867
7.6883	0.40604
7.4872	0.41369
7.2862	0.42147
7.0852	0.42952
6.8842	0.43779
6.6832	0.44630
6.4822	0.45506
6.2812	0.46416
6.0802	0.47354
5.8792	0.48314
5.6782	0.49303
5.4772	0.50339
5.2762	0.51382
5.0752	0.52469
4.8742	0.53600
4.6732	0.54769
4.4721	0.55979
4.2711	0.57276
4.0701	0.58532
3.8691	0.59879

d (um)	Probe variable cRedSub (mol/m^3)
3.6681	0.61289
3.4671	0.62719
3.2661	0.64228
3.0651	0.65799
2.8641	0.67419
2.6631	0.69121
2.4621	0.70934
2.2611	0.72702
2.0601	0.74574
1.8591	0.76489
1.6581	0.78405
1.4570	0.80286
1.2560	0.82003
1.0550	0.83384
0.85403	0.84049
0.65302	0.83367
0.45201	0.80240
0.25101	0.72934
0.050000	0.59175

4.3.8 Table 9

Substrate Potential (EeqSub)

d (um)	EeqSub (V)
30.000	-0.084904
29.799	-0.085088
29.598	-0.085277
29.397	-0.085466
29.196	-0.085653
28.995	-0.085846
28.794	-0.086030
28.593	-0.086224
28.392	-0.086380
28.191	-0.086614
27.990	-0.086800
27.789	-0.087013
27.588	-0.087208
27.387	-0.087374
27.186	-0.087613
26.985	-0.087814

d (um)	EeqSub (V)
26.784	-0.088017
26.583	-0.088228
26.382	-0.088429
26.181	-0.088641
25.980	-0.088843
25.779	-0.089054
25.578	-0.089263
25.377	-0.089484
25.176	-0.089697
24.975	-0.089918
24.774	-0.090129
24.573	-0.090343
24.372	-0.090569
24.171	-0.090790
23.970	-0.091013
23.769	-0.091245
23.568	-0.091465
23.367	-0.091693
23.166	-0.091918
22.965	-0.092152
22.764	-0.092385
22.563	-0.092622
22.362	-0.092856
22.161	-0.093093
21.960	-0.093331
21.759	-0.093575
21.558	-0.093820
21.357	-0.094049
21.156	-0.094311
20.955	-0.094555
20.754	-0.094814
20.553	-0.095062
20.352	-0.095322
20.151	-0.095558
19.950	-0.095833
19.749	-0.096098
19.548	-0.096361
19.347	-0.096599
19.146	-0.096887
18.945	-0.097155

d (um)	EeqSub (V)
18.744	-0.097431
18.543	-0.097704
18.342	-0.097976
18.141	-0.098238
17.940	-0.098532
17.739	-0.098817
17.538	-0.099098
17.337	-0.099382
17.136	-0.099669
16.935	-0.099958
16.734	-0.10025
16.533	-0.10055
16.332	-0.10085
16.131	-0.10115
15.930	-0.10145
15.729	-0.10175
15.528	-0.10206
15.327	-0.10237
15.126	-0.10268
14.924	-0.10299
14.723	-0.10331
14.522	-0.10364
14.321	-0.10395
14.120	-0.10428
13.919	-0.10461
13.718	-0.10494
13.517	-0.10528
13.316	-0.10561
13.115	-0.10595
12.914	-0.10629
12.713	-0.10663
12.512	-0.10698
12.311	-0.10733
12.110	-0.10768
11.909	-0.10804
11.708	-0.10839
11.507	-0.10875
11.306	-0.10912
11.105	-0.10948
10.904	-0.10985

d (um)	EeqSub (V)
10.703	-0.11023
10.502	-0.11060
10.301	-0.11097
10.100	-0.11136
9.8993	-0.11174
9.6983	-0.11213
9.4973	-0.11251
9.2963	-0.11290
9.0953	-0.11330
8.8943	-0.11369
8.6933	-0.11408
8.4923	-0.11449
8.2913	-0.11489
8.0903	-0.11529
7.8893	-0.11570
7.6883	-0.11611
7.4872	-0.11652
7.2862	-0.11693
7.0852	-0.11734
6.8842	-0.11775
6.6832	-0.11816
6.4822	-0.11858
6.2812	-0.11899
6.0802	-0.11941
5.8792	-0.11983
5.6782	-0.12024
5.4772	-0.12066
5.2762	-0.12107
5.0752	-0.12148
4.8742	-0.12190
4.6732	-0.12231
4.4721	-0.12272
4.2711	-0.12315
4.0701	-0.12353
3.8691	-0.12394
3.6681	-0.12434
3.4671	-0.12473
3.2661	-0.12512
3.0651	-0.12552
2.8641	-0.12591

d (um)	EeqSub (V)
2.6631	-0.12630
2.4621	-0.12672
2.2611	-0.12710
2.0601	-0.12753
1.8591	-0.12798
1.6581	-0.12849
1.4570	-0.12909
1.2560	-0.12983
1.0550	-0.13083
0.85403	-0.13223
0.65302	-0.13444
0.45201	-0.13834
0.25101	-0.14678
0.050000	-0.17932

4.3.9 Accumulated Probe Table 10

k0Sub (m/s)	d (m)	Time (s)	-(tds2.ntflux_Oxi)*n*F (A), UME	Surface ConcOxi (M), Oxi Mem	Surface ConcOxi (M), OxiNuc	Substrate Kinetics (m/s), k0Sub probe	Normal total flux (mol/s), UME OCR membrane	Normal total flux (mol/s), UME OCR nucleus	Concentration (mol/m^3), Point: (5.5109E-16, 2.5)	k0Sub* Oxi (mol/s), Mem Flux	k0Sub* Oxi (mol/s), Nuc Flux	Normal total flux (mol/(m^2*s)), Point: (5.5109E-16, 2.5)	WElectrode radius (μm), UME Radius
0.0000	5.0000E-7	0.0000	-7.1493E-9	NaN	2.2700E-4	0.0000	3.5480E-18	-2.1981E-29	0.22697	0.0000	0.0000	9.5108E-7	1.0000
0.0000	5.0000E-7	0.005000	-7.4819E-10	NaN	2.0657E-4	0.0000	8.6907E-16	7.0680E-16	0.062313	0.0000	0.0000	2.2781E-4	1.0000
0.0000	5.0000E-7	0.010000	-7.0196E-10	NaN	1.9858E-4	0.0000	4.5894E-16	7.0445E-16	0.058402	0.0000	0.0000	2.1349E-4	1.0000
0.0000	5.0000E-7	0.015000	-6.7909E-10	NaN	1.9390E-4	0.0000	2.7183E-16	6.9542E-16	0.056480	0.0000	0.0000	2.0645E-4	1.0000
0.00	5.00	0.02	-	NaN	1.91	0.00	1.76	6.88	0.05	0.00	0.00	2.02	1.00

k0Sub (m/s)	d (m)	Time (s)	-(tds2.ntflux_Oxi)*n*F (A), UME	Surface ConcOxi (M), OxiMem	Surface ConcOxi (M), OxiNuc	Substrate Kinetics (m/s), k0Sub probe	Normal total flux (mol/s), UME OCR membrane	Normal total flux (mol/s), UME OCR nucleus	Concentration (mol/m^3), Point: (5.5109E-16, 2.5)	k0Sub*Oxi (mol/s), MemFlux	k0Sub*Oxi (mol/s), NucFlux	Normal total flux (mol/(m^2*s)), Point: (5.5109E-16, 2.5)	WElectrode radius (µm), UME Radius
00	00E-7	0000	6.6597E-10		00E-4	00	39E-16	28E-16	5380	00	00	43E-4	00
0.0000	5.0000E-7	0.025000	-6.5729E-10	NaN	1.8904E-4	0.0000	1.1877E-16	6.8326E-16	0.054653	0.0000	0.0000	1.9977E-4	1.0000
0.0000	5.0000E-7	0.030000	-6.5123E-10	NaN	1.8765E-4	0.0000	8.1417E-17	6.7960E-16	0.054146	0.0000	0.0000	1.9792E-4	1.0000
0.0000	5.0000E-7	0.035000	-6.4706E-10	NaN	1.8667E-4	0.0000	5.8336E-17	6.7688E-16	0.053797	0.0000	0.0000	1.9664E-4	1.0000
0.0000	5.0000E-7	0.040000	-6.4375E-10	NaN	1.8588E-4	0.0000	4.0851E-17	6.7463E-16	0.053520	0.0000	0.0000	1.9563E-4	1.0000
0.0000	5.0000E-7	0.045000	-6.4123E-10	NaN	1.8526E-4	0.0000	2.8581E-17	6.7284E-16	0.053309	0.0000	0.0000	1.9486E-4	1.0000
0.0000	5.0000E-7	0.050000	-6.3967E-10	NaN	1.8486E-4	0.0000	2.2613E-17	6.7159E-16	0.053179	0.0000	0.0000	1.9438E-4	1.0000
0.0000	5.0000E-7	0.055000	-6.3881E-10	NaN	1.8463E-4	0.0000	2.0542E-17	6.7081E-16	0.053107	0.0000	0.0000	1.9412E-4	1.0000
0.0000	5.0000E-7	0.060000	-6.3795E-10	NaN	1.8440E-4	0.0000	1.8471E-17	6.7003E-16	0.053036	0.0000	0.0000	1.9386E-4	1.0000
0.0000	5.0000E-7	0.065000	-6.3720E-10	NaN	1.8420E-4	0.0000	1.6734E-17	6.6935E-16	0.052974	0.0000	0.0000	1.9363E-4	1.0000

k0Sub (m/s)	d (m)	Time (s)	-(tds2.ntflux_Oxi)*n*F (A), UME	Surface ConcOxi (M), Oxi Mem	Surface ConcOxi (M), OxiNuc	Substrate Kinetics (m/s), k0Sub probe	Normal total flux (mol/s), UME OCR membrane	Normal total flux (mol/s), UME OCR nucleus	Concentration (mol/m^3), Point: (5.5109E-16, 2.5)	k0Sub*Oxi (mol/s), Mem Flux	k0Sub*Oxi (mol/s), Nuc Flux	Normal total flux (mol/(m^2*s)), Point: (5.5109E-16, 2.5)	WElectrode radius (μm), UME Radius
0.0000	5.0000E-7	0.070000	-6.3656E-10	NaN	1.8403E-4	0.0000	1.5252E-17	6.6876E-16	0.052920	0.0000	0.0000	1.9343E-4	1.0000
0.0000	5.0000E-7	0.075000	-6.3591E-10	NaN	1.8385E-4	0.0000	1.3771E-17	6.6817E-16	0.052866	0.0000	0.0000	1.9324E-4	1.0000
0.0000	5.0000E-7	0.080000	-6.3545E-10	NaN	1.8373E-4	0.0000	1.2825E-17	6.6775E-16	0.052828	0.0000	0.0000	1.9310E-4	1.0000
0.0000	5.0000E-7	0.085000	-6.3503E-10	NaN	1.8361E-4	0.0000	1.1950E-17	6.6736E-16	0.052793	0.0000	0.0000	1.9297E-4	1.0000
0.0000	5.0000E-7	0.090000	-6.3461E-10	NaN	1.8350E-4	0.0000	1.1076E-17	6.6697E-16	0.052758	0.0000	0.0000	1.9284E-4	1.0000
0.0000	5.0000E-7	0.095000	-6.3419E-10	NaN	1.8338E-4	0.0000	1.0202E-17	6.6658E-16	0.052723	0.0000	0.0000	1.9271E-4	1.0000
0.0000	5.0000E-7	0.10000	-6.3377E-10	NaN	1.8327E-4	0.0000	9.3277E-18	6.6619E-16	0.052687	0.0000	0.0000	1.9258E-4	1.0000
0.0000	5.0000E-7	0.10500	-6.3343E-10	NaN	1.8318E-4	0.0000	8.7098E-18	6.6588E-16	0.052660	0.0000	0.0000	1.9248E-4	1.0000
0.0000	5.0000E-7	0.11000	-6.3320E-10	NaN	1.8312E-4	0.0000	8.3352E-18	6.6566E-16	0.052641	0.0000	0.0000	1.9241E-4	1.0000
0.0000	5.0000E-7	0.11500	-6.3297E-	NaN	1.8305E-4	0.0000	7.9607E-18	6.6544E-16	0.052621	0.0000	0.0000	1.9234E-4	1.0000

k0Sub (m/s)	d (m)	Time (s)	-(tds2.ntflux_Oxi)*n*F (A), UME	Surface Con cOxi (M), Oxi Mem	Surface Con cOxi (M), OxiNuc	Substrate Kinetics (m/s), k0Sub probe	Normal total flux (mol/s), UME OCR membrane	Normal total flux (mol/s), UME OCR nucleus	Concentration (mol/m^3), Point: (5.5109E-16, 2.5)	k0Sub*Oxi (mol/s), Mem Flux	k0Sub*Oxi (mol/s), Nuc Flux	Normal total flux (mol/(m^2*s)), Point: (5.5109E-16, 2.5)	WElectrode radius (µm), UME Radius
			10										
0.0000	5.0000E-7	0.12000	-6.3274E-10	NaN	1.8299E-4	0.0000	7.5861E-18	6.6522E-16	0.052602	0.0000	0.0000	1.9227E-4	1.0000
0.0000	5.0000E-7	0.12500	-6.3251E-10	NaN	1.8293E-4	0.0000	7.2116E-18	6.6500E-16	0.052583	0.0000	0.0000	1.9220E-4	1.0000
0.0000	5.0000E-7	0.13000	-6.3228E-10	NaN	1.8286E-4	0.0000	6.8371E-18	6.6478E-16	0.052564	0.0000	0.0000	1.9213E-4	1.0000
0.0000	5.0000E-7	0.13500	-6.3205E-10	NaN	1.8280E-4	0.0000	6.4625E-18	6.6456E-16	0.052544	0.0000	0.0000	1.9206E-4	1.0000
0.0000	5.0000E-7	0.14000	-6.3182E-10	NaN	1.8273E-4	0.0000	6.0880E-18	6.6434E-16	0.052525	0.0000	0.0000	1.9199E-4	1.0000
0.0000	5.0000E-7	0.14500	-6.3159E-10	NaN	1.8267E-4	0.0000	5.7134E-18	6.6412E-16	0.052506	0.0000	0.0000	1.9192E-4	1.0000
0.0000	5.0000E-7	0.15000	-6.3135E-10	NaN	1.8261E-4	0.0000	5.3389E-18	6.6390E-16	0.052487	0.0000	0.0000	1.9185E-4	1.0000
0.0000	5.0000E-7	0.15500	-6.3112E-10	NaN	1.8254E-4	0.0000	4.9643E-18	6.6368E-16	0.052467	0.0000	0.0000	1.9178E-4	1.0000
0.0000	5.0000E-7	0.16000	-6.3098E-10	NaN	1.8250E-4	0.0000	4.7848E-18	6.6354E-16	0.052455	0.0000	0.0000	1.9173E-4	1.0000
0.00	5.0000E-	0.16	-6.30	NaN	1.8247E-	0.00	4.6587E-	6.6343E-	0.05	0.00	0.00	1.9170E-	1.00

k0Sub (m/s)	d (m)	Time (s)	-(tds2.ntflux_Oxi)*n*F (A), UME	Surface ConcOxi (M), OxiMem	Surface ConcOxi (M), OxiNuc	Substrate Kinetics (m/s), k0Sub probe	Normal total flux (mol/s), UME OCR membrane	Normal total flux (mol/s), UME OCR nucleus	Concentration (mol/m^3), Point: (5.5109E-16, 2.5)	k0Sub*Oxi (mol/s), Mem Flux	k0Sub*Oxi (mol/s), Nuc Flux	Normal total flux (mol/(m^2*s)), Point: (5.5109E-16, 2.5)	WElectrode radius (µm), UME Radius
00	7	500	87E-10		4	00	18	16	2446	00	00	4	00
0.0000	5.0000E-7	0.17000	-6.3075E-10	NaN	1.8244E-4	0.0000	4.5327E-18	6.6332E-16	0.052437	0.0000	0.0000	1.9167E-4	1.0000
0.0000	5.0000E-7	0.17500	-6.3064E-10	NaN	1.8241E-4	0.0000	4.4067E-18	6.6321E-16	0.052427	0.0000	0.0000	1.9163E-4	1.0000
0.0000	5.0000E-7	0.18000	-6.3053E-10	NaN	1.8238E-4	0.0000	4.2807E-18	6.6310E-16	0.052418	0.0000	0.0000	1.9160E-4	1.0000
0.0000	5.0000E-7	0.18500	-6.3041E-10	NaN	1.8235E-4	0.0000	4.1547E-18	6.6298E-16	0.052408	0.0000	0.0000	1.9156E-4	1.0000
0.0000	5.0000E-7	0.19000	-6.3030E-10	NaN	1.8231E-4	0.0000	4.0287E-18	6.6287E-16	0.052399	0.0000	0.0000	1.9153E-4	1.0000
0.0000	5.0000E-7	0.19500	-6.3019E-10	NaN	1.8228E-4	0.0000	3.9027E-18	6.6276E-16	0.052390	0.0000	0.0000	1.9149E-4	1.0000
0.0000	5.0000E-7	0.20000	-6.3008E-10	NaN	1.8225E-4	0.0000	3.7767E-18	6.6265E-16	0.052380	0.0000	0.0000	1.9146E-4	1.0000
0.0000	5.0000E-7	0.20500	-6.2996E-10	NaN	1.8222E-4	0.0000	3.6507E-18	6.6254E-16	0.052371	0.0000	0.0000	1.9142E-4	1.0000
0.0000	5.0000E-7	0.21000	-6.2985E-10	NaN	1.8219E-4	0.0000	3.5247E-18	6.6243E-16	0.052361	0.0000	0.0000	1.9139E-4	1.0000

k0Sub (m/s)	d (m)	Time (s)	-(tds2.ntflux_Oxi)*n*F (A), UME	Surface ConcOxi (M), OxiMem	Surface ConcOxi (M), OxiNuc	Substrate Kinetics (m/s), k0Sub probe	Normal total flux (mol/s), UME OCR membrane	Normal total flux (mol/s), UME OCR nucleus	Concentration (mol/m^3), Point: (5.5109E-16, 2.5)	k0Sub*Oxi (mol/s), Mem Flux	k0Sub*Oxi (mol/s), Nuc Flux	Normal total flux (mol/(m^2*s)), Point: (5.5109E-16, 2.5)	WEIectrode radius (µm), UME Radius
0.0000	5.0000E-7	0.21500	-6.2974E-10	NaN	1.8216E-4	0.0000	3.3987E-18	6.6232E-16	0.052352	0.0000	0.0000	1.9136E-4	1.0000
0.0000	5.0000E-7	0.22000	-6.2962E-10	NaN	1.8212E-4	0.0000	3.2727E-18	6.6221E-16	0.052343	0.0000	0.0000	1.9132E-4	1.0000
0.0000	5.0000E-7	0.22500	-6.2951E-10	NaN	1.8209E-4	0.0000	3.1467E-18	6.6210E-16	0.052333	0.0000	0.0000	1.9129E-4	1.0000
0.0000	5.0000E-7	0.23000	-6.2940E-10	NaN	1.8206E-4	0.0000	3.0206E-18	6.6199E-16	0.052324	0.0000	0.0000	1.9125E-4	1.0000
0.0000	5.0000E-7	0.23500	-6.2928E-10	NaN	1.8203E-4	0.0000	2.8946E-18	6.6187E-16	0.052314	0.0000	0.0000	1.9122E-4	1.0000
0.0000	5.0000E-7	0.24000	-6.2917E-10	NaN	1.8200E-4	0.0000	2.7686E-18	6.6176E-16	0.052305	0.0000	0.0000	1.9118E-4	1.0000
0.0000	5.0000E-7	0.24500	-6.2906E-10	NaN	1.8197E-4	0.0000	2.6426E-18	6.6165E-16	0.052296	0.0000	0.0000	1.9115E-4	1.0000
0.0000	5.0000E-7	0.25000	-6.2895E-10	NaN	1.8193E-4	0.0000	2.5166E-18	6.6154E-16	0.052286	0.0000	0.0000	1.9112E-4	1.0000
0.0000	5.0000E-7	0.25000	-6.2895E-10	NaN	1.8193E-4	0.0000	2.5166E-18	6.6154E-16	0.052286	0.0000	0.0000	1.9112E-4	1.0000
0.0000	5.0000E-7	0.27000	-6.2864E-	NaN	1.8185E-4	0.0000	2.2410E-18	6.6123E-16	0.052260	0.0000	0.0000	1.9102E-4	1.0000

k0Sub (m/s)	d (m)	Time (s)	-(tds2.ntflux_Oxi)*n*F (A), UME	Surface ConcOxi (M), OxiMem	Surface ConcOxi (M), OxiNuc	Substrate Kinetics (m/s), k0Sub probe	Normal total flux (mol/s), UME OCR membrane	Normal total flux (mol/s), UME OCR nucleus	Concentration (mol/m^3), Point: (5.5109E-16, 2.5)	k0Sub*Oxi (mol/s), Mem Flux	k0Sub*Oxi (mol/s), Nuc Flux	Normal total flux (mol/(m^2*s)), Point: (5.5109E-16, 2.5)	WElectrode radius (µm), UME Radius
			10										
0.0000	5.0000E-7	0.29000	-6.2838E-10	NaN	1.8177E-4	0.0000	2.0412E-18	6.6097E-16	0.052239	0.0000	0.0000	1.9094E-4	1.0000
0.0000	5.0000E-7	0.31000	-6.2812E-10	NaN	1.8170E-4	0.0000	1.8415E-18	6.6071E-16	0.052217	0.0000	0.0000	1.9086E-4	1.0000
0.0000	5.0000E-7	0.33000	-6.2786E-10	NaN	1.8163E-4	0.0000	1.6418E-18	6.6045E-16	0.052196	0.0000	0.0000	1.9078E-4	1.0000
0.0000	5.0000E-7	0.35000	-6.2760E-10	NaN	1.8155E-4	0.0000	1.4421E-18	6.6019E-16	0.052174	0.0000	0.0000	1.9071E-4	1.0000
0.0000	5.0000E-7	0.37000	-6.2741E-10	NaN	1.8150E-4	0.0000	1.3260E-18	6.6000E-16	0.052158	0.0000	0.0000	1.9065E-4	1.0000
0.0000	5.0000E-7	0.39000	-6.2724E-10	NaN	1.8145E-4	0.0000	1.2299E-18	6.5982E-16	0.052144	0.0000	0.0000	1.9060E-4	1.0000
0.0000	5.0000E-7	0.41000	-6.2707E-10	NaN	1.8140E-4	0.0000	1.1339E-18	6.5965E-16	0.052130	0.0000	0.0000	1.9055E-4	1.0000
0.0000	5.0000E-7	0.43000	-6.2690E-10	NaN	1.8136E-4	0.0000	1.0378E-18	6.5948E-16	0.052116	0.0000	0.0000	1.9049E-4	1.0000
0.0000	5.0000E-7	0.45000	-6.2673E-10	NaN	1.8131E-4	0.0000	9.4178E-19	6.5931E-16	0.052102	0.0000	0.0000	1.9044E-4	1.0000
0.00	5.0000E-	0.47	-6.26	NaN	1.8127E-	0.00	8.8273E-	6.5917E-	0.05	0.00	0.00	1.9040E-	1.00

k0Sub (m/s)	d (m)	Time (s)	-(tds2.ntflux_Oxi)*n*F (A), UME	Surface ConcOxi (M), OxiMem	Surface ConcOxi (M), OxiNuc	Substrate Kinetics (m/s), k0Sub probe	Normal total flux (mol/s), UME OCR membrane	Normal total flux (mol/s), UME OCR nucleus	Concentration (mol/m^3), Point: (5.5109E-16, 2.5)	k0Sub*Oxi (mol/s), Mem Flux	k0Sub*Oxi (mol/s), Nuc Flux	Normal total flux (mol/(m^2*s)), Point: (5.5109E-16, 2.5)	WElectrode radius (µm), UME Radius
00	7	000	60E-10		4	00	19	16	2091	00	00	4	00
0.0000	5.0000E-7	0.49000	-6.2648E-10	NaN	1.8124E-4	0.0000	8.2947E-19	6.5905E-16	0.052081	0.0000	0.0000	1.9037E-4	1.0000
0.0000	5.0000E-7	0.51000	-6.2636E-10	NaN	1.8120E-4	0.0000	7.7622E-19	6.5893E-16	0.052071	0.0000	0.0000	1.9033E-4	1.0000
0.0000	5.0000E-7	0.53000	-6.2624E-10	NaN	1.8117E-4	0.0000	7.2296E-19	6.5880E-16	0.052061	0.0000	0.0000	1.9029E-4	1.0000
0.0000	5.0000E-7	0.55000	-6.2612E-10	NaN	1.8113E-4	0.0000	6.6970E-19	6.5868E-16	0.052051	0.0000	0.0000	1.9026E-4	1.0000
0.0000	5.0000E-7	0.57000	-6.2602E-10	NaN	1.8110E-4	0.0000	6.3375E-19	6.5858E-16	0.052043	0.0000	0.0000	1.9023E-4	1.0000
0.0000	5.0000E-7	0.59000	-6.2593E-10	NaN	1.8108E-4	0.0000	5.9924E-19	6.5848E-16	0.052035	0.0000	0.0000	1.9020E-4	1.0000
0.0000	5.0000E-7	0.61000	-6.2583E-10	NaN	1.8105E-4	0.0000	5.6474E-19	6.5839E-16	0.052027	0.0000	0.0000	1.9017E-4	1.0000
0.0000	5.0000E-7	0.63000	-6.2574E-10	NaN	1.8102E-4	0.0000	5.3023E-19	6.5829E-16	0.052020	0.0000	0.0000	1.9014E-4	1.0000
0.0000	5.0000E-7	0.65000	-6.2565E-10	NaN	1.8100E-4	0.0000	4.9573E-19	6.5819E-16	0.052012	0.0000	0.0000	1.9011E-4	1.0000

k0Sub (m/s)	d (m)	Time (s)	-(tds2.ntflux_Oxi)*n*F (A), UME	Surface ConcOxi (M), OxiMem	Surface ConcOxi (M), OxiNuc	Substrate Kinetics (m/s), k0Sub probe	Normal total flux (mol/s), UME OCR membrane	Normal total flux (mol/s), UME OCR nucleus	Concentration (mol/m^3), Point: (5.5109E-16, 2.5)	k0Sub*Oxi (mol/s), Mem Flux	k0Sub*Oxi (mol/s), Nuc Flux	Normal total flux (mol/(m^2*s)), Point: (5.5109E-16, 2.5)	WElectrode radius (µm), UME Radius
0.0000	5.0000E-7	0.67000	-6.2557E-10	NaN	1.8098E-4	0.0000	4.7353E-19	6.5812E-16	0.052006	0.0000	0.0000	1.9009E-4	1.0000
0.0000	5.0000E-7	0.69000	-6.2550E-10	NaN	1.8096E-4	0.0000	4.5160E-19	6.5804E-16	0.052000	0.0000	0.0000	1.9007E-4	1.0000
0.0000	5.0000E-7	0.71000	-6.2543E-10	NaN	1.8093E-4	0.0000	4.2966E-19	6.5796E-16	0.051993	0.0000	0.0000	1.9005E-4	1.0000
0.0000	5.0000E-7	0.73000	-6.2535E-10	NaN	1.8091E-4	0.0000	4.0772E-19	6.5789E-16	0.051987	0.0000	0.0000	1.9002E-4	1.0000
0.0000	5.0000E-7	0.75000	-6.2528E-10	NaN	1.8089E-4	0.0000	3.8579E-19	6.5781E-16	0.051981	0.0000	0.0000	1.9000E-4	1.0000
0.0000	5.0000E-7	0.77000	-6.2522E-10	NaN	1.8087E-4	0.0000	3.6808E-19	6.5775E-16	0.051976	0.0000	0.0000	1.8998E-4	1.0000
0.0000	5.0000E-7	0.79000	-6.2515E-10	NaN	1.8086E-4	0.0000	3.5161E-19	6.5768E-16	0.051971	0.0000	0.0000	1.8996E-4	1.0000
0.0000	5.0000E-7	0.81000	-6.2509E-10	NaN	1.8084E-4	0.0000	3.3651E-19	6.5762E-16	0.051966	0.0000	0.0000	1.8994E-4	1.0000
0.0000	5.0000E-7	0.83000	-6.2504E-10	NaN	1.8082E-4	0.0000	3.2277E-19	6.5756E-16	0.051961	0.0000	0.0000	1.8993E-4	1.0000
0.0000	5.0000E-7	0.85000	-6.2498E-	NaN	1.8081E-4	0.0000	3.1031E-19	6.5750E-16	0.051956	0.0000	0.0000	1.8991E-4	1.0000

k0Sub (m/s)	d (m)	Time (s)	-(tds2.ntflux_Oxi)*n*F (A), UME	Surface ConcOxi (M), Oxi Mem	Surface ConcOxi (M), OxiNuc	Substrate Kinetics (m/s), k0Sub probe	Normal total flux (mol/s), UME OCR membrane	Normal total flux (mol/s), UME OCR nucleus	Concentration (mol/m^3), Point: (5.5109E-16, 2.5)	k0Sub*Oxi (mol/s), Mem Flux	k0Sub*Oxi (mol/s), Nuc Flux	Normal total flux (mol/(m^2*s)), Point: (5.5109E-16, 2.5)	WElectrode radius (µm), UME Radius
			10										
0.0000	5.0000E-7	0.87000	-6.2493E-10	NaN	1.8079E-4	0.0000	2.9770E-19	6.5745E-16	0.051952	0.0000	0.0000	1.8989E-4	1.0000
0.0000	5.0000E-7	0.89000	-6.2487E-10	NaN	1.8078E-4	0.0000	2.8593E-19	6.5739E-16	0.051947	0.0000	0.0000	1.8988E-4	1.0000
0.0000	5.0000E-7	0.91000	-6.2482E-10	NaN	1.8076E-4	0.0000	2.7500E-19	6.5734E-16	0.051943	0.0000	0.0000	1.8986E-4	1.0000
0.0000	5.0000E-7	0.93000	-6.2478E-10	NaN	1.8075E-4	0.0000	2.6491E-19	6.5729E-16	0.051939	0.0000	0.0000	1.8985E-4	1.0000
0.0000	5.0000E-7	0.95000	-6.2473E-10	NaN	1.8073E-4	0.0000	2.5556E-19	6.5724E-16	0.051935	0.0000	0.0000	1.8983E-4	1.0000
0.0000	5.0000E-7	0.97000	-6.2468E-10	NaN	1.8072E-4	0.0000	2.4631E-19	6.5719E-16	0.051932	0.0000	0.0000	1.8982E-4	1.0000
0.0000	5.0000E-7	0.99000	-6.2464E-10	NaN	1.8071E-4	0.0000	2.3762E-19	6.5715E-16	0.051928	0.0000	0.0000	1.8981E-4	1.0000
0.0000	1.0000E-6	0.0000	-7.1537E-9	NaN	2.2700E-4	0.0000	1.1043E-22	-1.1939E-29	0.22700	0.0000	0.0000	2.3845E-11	1.0000
0.0000	1.0000E-6	0.0050000	-7.9199E-10	NaN	2.1025E-4	0.0000	8.0011E-16	5.6570E-16	0.11268	0.0000	0.0000	1.4396E-4	1.0000
0.00	1.0000E-	0.01	-7.46	NaN	2.0278E-	0.00	4.3923E-	5.7403E-	0.10	0.00	0.00	1.3552E-	1.00

k0Sub (m/s)	d (m)	Time (s)	-(tds2.ntflux_Oxi)*n*F (A), UME	Surface ConcOxi (M), OxiMem	Surface ConcOxi (M), OxiNuc	Substrate Kinetics (m/s), k0Sub probe	Normal total flux (mol/s), UME OCR membrane	Normal total flux (mol/s), UME OCR nucleus	Concentration (mol/m^3), Point: (5.5109E-16, 2.5)	k0Sub*Oxi (mol/s), Mem Flux	k0Sub*Oxi (mol/s), Nuc Flux	Normal total flux (mol/(m^2*s)), Point: (5.5109E-16, 2.5)	WEIectrode radius (µm), UME Radius
00	6	0000	72E-10		4	00	16	16	612	00	00	4	00
0.0000	1.0000E-6	0.015000	-7.2418E-10	NaN	1.9831E-4	0.0000	2.7180E-16	5.6980E-16	0.10288	0.0000	0.0000	1.3137E-4	1.0000
0.0000	1.0000E-6	0.020000	-7.1001E-10	NaN	1.9536E-4	0.0000	1.7728E-16	5.6601E-16	0.10085	0.0000	0.0000	1.2877E-4	1.0000
0.0000	1.0000E-6	0.025000	-7.0046E-10	NaN	1.9331E-4	0.0000	1.1872E-16	5.6289E-16	0.099479	0.0000	0.0000	1.2702E-4	1.0000
0.0000	1.0000E-6	0.030000	-6.9382E-10	NaN	1.9183E-4	0.0000	8.1525E-17	5.6034E-16	0.098529	0.0000	0.0000	1.2580E-4	1.0000
0.0000	1.0000E-6	0.035000	-6.8941E-10	NaN	1.9082E-4	0.0000	6.0102E-17	5.5832E-16	0.097899	0.0000	0.0000	1.2499E-4	1.0000
0.0000	1.0000E-6	0.040000	-6.8627E-10	NaN	1.9007E-4	0.0000	4.6574E-17	5.5671E-16	0.097450	0.0000	0.0000	1.2442E-4	1.0000
0.0000	1.0000E-6	0.045000	-6.8387E-10	NaN	1.8949E-4	0.0000	3.7141E-17	5.5542E-16	0.097107	0.0000	0.0000	1.2398E-4	1.0000
0.0000	1.0000E-6	0.050000	-6.8183E-10	NaN	1.8900E-4	0.0000	2.9412E-17	5.5430E-16	0.096816	0.0000	0.0000	1.2361E-4	1.0000
0.0000	1.0000E-6	0.055000	-6.8015E-10	NaN	1.8859E-4	0.0000	2.3492E-17	5.5335E-16	0.096578	0.0000	0.0000	1.2330E-4	1.0000

k0Sub (m/s)	d (m)	Time (s)	-(tds2.ntflux_Oxi)*n*F (A), UME	Surface ConcOxi (M), OxiMem	Surface ConcOxi (M), OxiNuc	Substrate Kinetics (m/s), k0Sub probe	Normal total flux (mol/s), UME OCR membrane	Normal total flux (mol/s), UME OCR nucleus	Concentration (mol/m^3), Point: (5.5109E-16, 2.5)	k0Sub*Oxi (mol/s), MemFlux	k0Sub*Oxi (mol/s), NucFlux	Normal total flux (mol/(m^2*s)), Point: (5.5109E-16, 2.5)	WElectrode radius (µm), UME Radius
0.0000	1.0000E-6	0.060000	-6.7884E-10	NaN	1.8827E-4	0.0000	1.9298E-17	5.5258E-16	0.096390	0.0000	0.0000	1.2306E-4	1.0000
0.0000	1.0000E-6	0.065000	-6.7769E-10	NaN	1.8798E-4	0.0000	1.5827E-17	5.5188E-16	0.096226	0.0000	0.0000	1.2285E-4	1.0000
0.0000	1.0000E-6	0.070000	-6.7680E-10	NaN	1.8775E-4	0.0000	1.3607E-17	5.5130E-16	0.096099	0.0000	0.0000	1.2269E-4	1.0000
0.0000	1.0000E-6	0.075000	-6.7603E-10	NaN	1.8756E-4	0.0000	1.1892E-17	5.5080E-16	0.095990	0.0000	0.0000	1.2255E-4	1.0000
0.0000	1.0000E-6	0.080000	-6.7527E-10	NaN	1.8736E-4	0.0000	1.0004E-17	5.5030E-16	0.095881	0.0000	0.0000	1.2241E-4	1.0000
0.0000	1.0000E-6	0.085000	-6.7459E-10	NaN	1.8719E-4	0.0000	8.4490E-18	5.4985E-16	0.095785	0.0000	0.0000	1.2229E-4	1.0000
0.0000	1.0000E-6	0.090000	-6.7401E-10	NaN	1.8704E-4	0.0000	7.2272E-18	5.4946E-16	0.095702	0.0000	0.0000	1.2218E-4	1.0000
0.0000	1.0000E-6	0.095000	-6.7351E-10	NaN	1.8691E-4	0.0000	6.3383E-18	5.4912E-16	0.095632	0.0000	0.0000	1.2210E-4	1.0000
0.0000	1.0000E-6	0.10000	-6.7312E-10	NaN	1.8680E-4	0.0000	5.8084E-18	5.4883E-16	0.095576	0.0000	0.0000	1.2202E-4	1.0000
0.0000	1.0000E-6	0.10500	-6.7291E-	NaN	1.8675E-4	0.0000	5.6544E-18	5.4867E-16	0.095546	0.0000	0.0000	1.2199E-4	1.0000

k0Sub (m/s)	d (m)	Time (s)	-(tds2.ntflux_Oxi)*n*F (A), UME	Surface ConcOxi (M), OxiMem	Surface ConcOxi (M), OxiNuc	Substrate Kinetics (m/s), k0Sub probe	Normal total flux (mol/s), UME OCR membrane	Normal total flux (mol/s), UME OCR nucleus	Concentration (mol/m^3), Point: (5.5109E-16, 2.5)	k0Sub*Oxi (mol/s), Mem Flux	k0Sub*Oxi (mol/s), Nuc Flux	Normal total flux (mol/(m^2*s)), Point: (5.5109E-16, 2.5)	WEIectrode radius (μm), UME Radius
			10										
0.0000	1.0000E-6	0.11000	-6.7271E-10	NaN	1.8669E-4	0.0000	5.5004E-18	5.4851E-16	0.095517	0.0000	0.0000	1.2195E-4	1.0000
0.0000	1.0000E-6	0.11500	-6.7250E-10	NaN	1.8663E-4	0.0000	5.3464E-18	5.4835E-16	0.095488	0.0000	0.0000	1.2191E-4	1.0000
0.0000	1.0000E-6	0.12000	-6.7229E-10	NaN	1.8658E-4	0.0000	5.1924E-18	5.4819E-16	0.095458	0.0000	0.0000	1.2187E-4	1.0000
0.0000	1.0000E-6	0.12500	-6.7209E-10	NaN	1.8652E-4	0.0000	5.0384E-18	5.4803E-16	0.095429	0.0000	0.0000	1.2184E-4	1.0000
0.0000	1.0000E-6	0.13000	-6.7188E-10	NaN	1.8647E-4	0.0000	4.8844E-18	5.4787E-16	0.095399	0.0000	0.0000	1.2180E-4	1.0000
0.0000	1.0000E-6	0.13500	-6.7167E-10	NaN	1.8641E-4	0.0000	4.7304E-18	5.4771E-16	0.095370	0.0000	0.0000	1.2176E-4	1.0000
0.0000	1.0000E-6	0.14000	-6.7147E-10	NaN	1.8635E-4	0.0000	4.5764E-18	5.4755E-16	0.095341	0.0000	0.0000	1.2172E-4	1.0000
0.0000	1.0000E-6	0.14500	-6.7126E-10	NaN	1.8630E-4	0.0000	4.4224E-18	5.4739E-16	0.095311	0.0000	0.0000	1.2169E-4	1.0000
0.0000	1.0000E-6	0.15000	-6.7105E-10	NaN	1.8624E-4	0.0000	4.2684E-18	5.4723E-16	0.095282	0.0000	0.0000	1.2165E-4	1.0000
0.00	1.0000E-	0.15	-6.70	NaN	1.8619E-	0.00	4.1276E-	5.4708E-	0.09	0.00	0.00	1.2161E-	1.00

k0Sub (m/s)	d (m)	Time (s)	-(tds2.ntflux_Oxi)*n*F (A), UME	Surface ConcOxi (M), OxiMem	Surface ConcOxi (M), OxiNuc	Substrate Kinetics (m/s), k0Sub probe	Normal total flux (mol/s), UME OCR membrane	Normal total flux (mol/s), UME OCR nucleus	Concentration (mol/m^3), Point: (5.5109E-16, 2.5)	k0Sub*Oxi (mol/s), Mem Flux	k0Sub*Oxi (mol/s), Nuc Flux	Normal total flux (mol/(m^2*s)), Point: (5.5109E-16, 2.5)	WElectrode radius (μm), UME Radius
00	6	500	86E-10		4	00	18	16	5254	00	00	4	00
0.0000	1.0000E-6	0.16000	-6.7072E-10	NaN	1.8615E-4	0.0000	4.0162E-18	5.4697E-16	0.095234	0.0000	0.0000	1.2159E-4	1.0000
0.0000	1.0000E-6	0.16500	-6.7058E-10	NaN	1.8611E-4	0.0000	3.9048E-18	5.4687E-16	0.095214	0.0000	0.0000	1.2156E-4	1.0000
0.0000	1.0000E-6	0.17000	-6.7044E-10	NaN	1.8607E-4	0.0000	3.7934E-18	5.4676E-16	0.095194	0.0000	0.0000	1.2154E-4	1.0000
0.0000	1.0000E-6	0.17500	-6.7029E-10	NaN	1.8604E-4	0.0000	3.6820E-18	5.4665E-16	0.095174	0.0000	0.0000	1.2151E-4	1.0000
0.0000	1.0000E-6	0.18000	-6.7015E-10	NaN	1.8600E-4	0.0000	3.5706E-18	5.4654E-16	0.095154	0.0000	0.0000	1.2149E-4	1.0000
0.0000	1.0000E-6	0.18500	-6.7001E-10	NaN	1.8596E-4	0.0000	3.4592E-18	5.4643E-16	0.095134	0.0000	0.0000	1.2146E-4	1.0000
0.0000	1.0000E-6	0.19000	-6.6987E-10	NaN	1.8592E-4	0.0000	3.3478E-18	5.4632E-16	0.095114	0.0000	0.0000	1.2143E-4	1.0000
0.0000	1.0000E-6	0.19500	-6.6973E-10	NaN	1.8588E-4	0.0000	3.2364E-18	5.4622E-16	0.095094	0.0000	0.0000	1.2141E-4	1.0000
0.0000	1.0000E-6	0.20000	-6.6959E-10	NaN	1.8585E-4	0.0000	3.1250E-18	5.4611E-16	0.095074	0.0000	0.0000	1.2138E-4	1.0000

k0Sub (m/s)	d (m)	Time (s)	-(tds2.ntflux_Oxi)*n*F (A), UME	Surface ConcOxi (M), OxiMem	Surface ConcOxi (M), OxiNuc	Substrate Kinetics (m/s), k0Sub probe	Normal total flux (mol/s), UME OCR membrane	Normal total flux (mol/s), UME OCR nucleus	Concentration (mol/m^3), Point: (5.5109E-16, 2.5)	k0Sub*Oxi (mol/s), Mem Flux	k0Sub*Oxi (mol/s), Nuc Flux	Normal total flux (mol/(m^2*s)), Point: (5.5109E-16, 2.5)	WElectrode radius (µm), UME Radius
0.0000	1.0000E-6	0.20500	-6.6945E-10	NaN	1.8581E-4	0.0000	3.0136E-18	5.4600E-16	0.095054	0.0000	0.0000	1.2136E-4	1.0000
0.0000	1.0000E-6	0.21000	-6.6933E-10	NaN	1.8577E-4	0.0000	2.9235E-18	5.4591E-16	0.095037	0.0000	0.0000	1.2134E-4	1.0000
0.0000	1.0000E-6	0.21500	-6.6924E-10	NaN	1.8575E-4	0.0000	2.8640E-18	5.4584E-16	0.095025	0.0000	0.0000	1.2132E-4	1.0000
0.0000	1.0000E-6	0.22000	-6.6916E-10	NaN	1.8573E-4	0.0000	2.8045E-18	5.4578E-16	0.095012	0.0000	0.0000	1.2130E-4	1.0000
0.0000	1.0000E-6	0.22500	-6.6907E-10	NaN	1.8570E-4	0.0000	2.7450E-18	5.4571E-16	0.095000	0.0000	0.0000	1.2129E-4	1.0000
0.0000	1.0000E-6	0.23000	-6.6899E-10	NaN	1.8568E-4	0.0000	2.6855E-18	5.4564E-16	0.094988	0.0000	0.0000	1.2127E-4	1.0000
0.0000	1.0000E-6	0.23500	-6.6890E-10	NaN	1.8566E-4	0.0000	2.6260E-18	5.4558E-16	0.094976	0.0000	0.0000	1.2126E-4	1.0000
0.0000	1.0000E-6	0.24000	-6.6882E-10	NaN	1.8564E-4	0.0000	2.5665E-18	5.4551E-16	0.094964	0.0000	0.0000	1.2124E-4	1.0000
0.0000	1.0000E-6	0.24500	-6.6873E-10	NaN	1.8561E-4	0.0000	2.5070E-18	5.4545E-16	0.094952	0.0000	0.0000	1.2123E-4	1.0000
0.0000	1.0000E-6	0.25000	-6.6865E-	NaN	1.8559E-4	0.0000	2.4475E-18	5.4538E-16	0.094940	0.0000	0.0000	1.2121E-4	1.0000

k0Sub (m/s)	d (m)	Time (s)	-(tds2.ntflux_Oxi)*n*F (A), UME	Surface ConcOxi (M), OxiMem	Surface ConcOxi (M), OxiNuc	Substrate Kinetics (m/s), k0Sub probe	Normal total flux (mol/s), UME OCR membrane	Normal total flux (mol/s), UME OCR nucleus	Concentration (mol/m^3), Point: (5.5109E-16, 2.5)	k0Sub*Oxi (mol/s), MemFlux	k0Sub*Oxi (mol/s), NucFlux	Normal total flux (mol/(m^2*s)), Point: (5.5109E-16, 2.5)	WElectrode radius (µm), UME Radius
			10										
0.0000	1.0000E-6	0.25000	-6.6865E-10	NaN	1.8559E-4	0.0000	2.4475E-18	5.4538E-16	0.094940	0.0000	0.0000	1.2121E-4	1.0000
0.0000	1.0000E-6	0.27000	-6.6831E-10	NaN	1.8550E-4	0.0000	2.2095E-18	5.4512E-16	0.094892	0.0000	0.0000	1.2115E-4	1.0000
0.0000	1.0000E-6	0.29000	-6.6797E-10	NaN	1.8540E-4	0.0000	1.9715E-18	5.4486E-16	0.094843	0.0000	0.0000	1.2109E-4	1.0000
0.0000	1.0000E-6	0.31000	-6.6764E-10	NaN	1.8532E-4	0.0000	1.7508E-18	5.4461E-16	0.094797	0.0000	0.0000	1.2103E-4	1.0000
0.0000	1.0000E-6	0.33000	-6.6742E-10	NaN	1.8526E-4	0.0000	1.6269E-18	5.4443E-16	0.094766	0.0000	0.0000	1.2099E-4	1.0000
0.0000	1.0000E-6	0.35000	-6.6720E-10	NaN	1.8520E-4	0.0000	1.5029E-18	5.4426E-16	0.094735	0.0000	0.0000	1.2095E-4	1.0000
0.0000	1.0000E-6	0.37000	-6.6698E-10	NaN	1.8514E-4	0.0000	1.3789E-18	5.4409E-16	0.094703	0.0000	0.0000	1.2091E-4	1.0000
0.0000	1.0000E-6	0.39000	-6.6676E-10	NaN	1.8508E-4	0.0000	1.2549E-18	5.4392E-16	0.094672	0.0000	0.0000	1.2087E-4	1.0000
0.0000	1.0000E-6	0.41000	-6.6655E-10	NaN	1.8502E-4	0.0000	1.1422E-18	5.4375E-16	0.094642	0.0000	0.0000	1.2083E-4	1.0000
0.00	1.0000E-	0.43	-6.66	NaN	1.8498E-	0.00	1.0731E-	5.4363E-	0.09	0.00	0.00	1.2080E-	1.00

k0Sub (m/s)	d (m)	Time (s)	-(tds2.ntflux_Oxi)*n*F (A), UME	Surface ConcOxi (M), OxiMem	Surface ConcOxi (M), OxiNuc	Substrate Kinetics (m/s), k0Sub probe	Normal total flux (mol/s), UME OCR membrane	Normal total flux (mol/s), UME OCR nucleus	Concentration (mol/m^3), Point: (5.5109E-16, 2.5)	k0Sub*Oxi (mol/s), Mem Flux	k0Sub*Oxi (mol/s), Nuc Flux	Normal total flux (mol/(m^2*s)), Point: (5.5109E-16, 2.5)	WElectrode radius (µm), UME Radius
00	6	000	40E-10		4	00	18	16	4620	00	00	4	00
0.0000	1.0000E-6	0.45000	-6.6624E-10	NaN	1.8493E-4	0.0000	1.0041E-18	5.4351E-16	0.094598	0.0000	0.0000	1.2078E-4	1.0000
0.0000	1.0000E-6	0.47000	-6.6609E-10	NaN	1.8489E-4	0.0000	9.3500E-19	5.4339E-16	0.094576	0.0000	0.0000	1.2075E-4	1.0000
0.0000	1.0000E-6	0.49000	-6.6593E-10	NaN	1.8485E-4	0.0000	8.6594E-19	5.4327E-16	0.094554	0.0000	0.0000	1.2072E-4	1.0000
0.0000	1.0000E-6	0.51000	-6.6579E-10	NaN	1.8481E-4	0.0000	8.0396E-19	5.4315E-16	0.094534	0.0000	0.0000	1.2069E-4	1.0000
0.0000	1.0000E-6	0.53000	-6.6567E-10	NaN	1.8478E-4	0.0000	7.6218E-19	5.4306E-16	0.094517	0.0000	0.0000	1.2067E-4	1.0000
0.0000	1.0000E-6	0.55000	-6.6556E-10	NaN	1.8475E-4	0.0000	7.2040E-19	5.4297E-16	0.094501	0.0000	0.0000	1.2065E-4	1.0000
0.0000	1.0000E-6	0.57000	-6.6544E-10	NaN	1.8471E-4	0.0000	6.7862E-19	5.4288E-16	0.094485	0.0000	0.0000	1.2063E-4	1.0000
0.0000	1.0000E-6	0.59000	-6.6533E-10	NaN	1.8468E-4	0.0000	6.3684E-19	5.4279E-16	0.094468	0.0000	0.0000	1.2061E-4	1.0000
0.0000	1.0000E-6	0.61000	-6.6522E-10	NaN	1.8465E-4	0.0000	5.9926E-19	5.4270E-16	0.094453	0.0000	0.0000	1.2059E-4	1.0000

k0Sub (m/s)	d (m)	Time (s)	-(tds2.ntflux_Oxi)*n*F (A), UME	Surface ConcOxi (M), OxiMem	Surface ConcOxi (M), OxiNuc	Substrate Kinetics (m/s), k0Sub probe	Normal total flux (mol/s), UME OCR membrane	Normal total flux (mol/s), UME OCR nucleus	Concentration (mol/m^3), Point: (5.5109E-16, 2.5)	k0Sub*Oxi (mol/s), Mem Flux	k0Sub*Oxi (mol/s), Nuc Flux	Normal total flux (mol/(m^2*s)), Point: (5.5109E-16, 2.5)	WElectrode radius (µm), UME Radius
0.0000	1.0000E-6	0.63000	-6.6513E-10	NaN	1.8463E-4	0.0000	5.7080E-19	5.4263E-16	0.094440	0.0000	0.0000	1.2057E-4	1.0000
0.0000	1.0000E-6	0.65000	-6.6503E-10	NaN	1.8460E-4	0.0000	5.4233E-19	5.4256E-16	0.094427	0.0000	0.0000	1.2056E-4	1.0000
0.0000	1.0000E-6	0.67000	-6.6494E-10	NaN	1.8458E-4	0.0000	5.1387E-19	5.4248E-16	0.094414	0.0000	0.0000	1.2054E-4	1.0000
0.0000	1.0000E-6	0.69000	-6.6485E-10	NaN	1.8455E-4	0.0000	4.8541E-19	5.4241E-16	0.094401	0.0000	0.0000	1.2052E-4	1.0000
0.0000	1.0000E-6	0.71000	-6.6476E-10	NaN	1.8453E-4	0.0000	4.6043E-19	5.4234E-16	0.094389	0.0000	0.0000	1.2051E-4	1.0000
0.0000	1.0000E-6	0.73000	-6.6469E-10	NaN	1.8451E-4	0.0000	4.4139E-19	5.4228E-16	0.094378	0.0000	0.0000	1.2049E-4	1.0000
0.0000	1.0000E-6	0.75000	-6.6462E-10	NaN	1.8449E-4	0.0000	4.2235E-19	5.4222E-16	0.094368	0.0000	0.0000	1.2048E-4	1.0000
0.0000	1.0000E-6	0.77000	-6.6455E-10	NaN	1.8447E-4	0.0000	4.0331E-19	5.4217E-16	0.094357	0.0000	0.0000	1.2047E-4	1.0000
0.0000	1.0000E-6	0.79000	-6.6447E-10	NaN	1.8445E-4	0.0000	3.8426E-19	5.4211E-16	0.094347	0.0000	0.0000	1.2045E-4	1.0000
0.0000	1.0000E-6	0.81000	-6.6440E-	NaN	1.8443E-4	0.0000	3.6644E-19	5.4205E-16	0.094337	0.0000	0.0000	1.2044E-4	1.0000

k0Sub (m/s)	d (m)	Time (s)	-(tds2.ntflux_Oxi)*n*F (A), UME	Surface ConcOxi (M), OxiMem	Surface ConcOxi (M), OxiNuc	Substrate Kinetics (m/s), k0Sub probe	Normal total flux (mol/s), UME OCR membrane	Normal total flux (mol/s), UME OCR nucleus	Concentration (mol/m^3), Point: (5.5109E-16, 2.5)	k0Sub*Oxi (mol/s), Mem Flux	k0Sub*Oxi (mol/s), Nuc Flux	Normal total flux (mol/(m^2*s)), Point: (5.5109E-16, 2.5)	WElectrode radius (µm), UME Radius
			10										
0.0000	1.0000E-6	0.83000	-6.6434E-10	NaN	1.8441E-4	0.0000	3.5102E-19	5.4200E-16	0.094328	0.0000	0.0000	1.2043E-4	1.0000
0.0000	1.0000E-6	0.85000	-6.6427E-10	NaN	1.8439E-4	0.0000	3.3668E-19	5.4195E-16	0.094319	0.0000	0.0000	1.2042E-4	1.0000
0.0000	1.0000E-6	0.87000	-6.6421E-10	NaN	1.8438E-4	0.0000	3.2341E-19	5.4190E-16	0.094310	0.0000	0.0000	1.2041E-4	1.0000
0.0000	1.0000E-6	0.89000	-6.6416E-10	NaN	1.8436E-4	0.0000	3.1120E-19	5.4185E-16	0.094302	0.0000	0.0000	1.2040E-4	1.0000
0.0000	1.0000E-6	0.91000	-6.6410E-10	NaN	1.8435E-4	0.0000	2.9962E-19	5.4181E-16	0.094294	0.0000	0.0000	1.2039E-4	1.0000
0.0000	1.0000E-6	0.93000	-6.6404E-10	NaN	1.8433E-4	0.0000	2.8834E-19	5.4176E-16	0.094286	0.0000	0.0000	1.2038E-4	1.0000
0.0000	1.0000E-6	0.95000	-6.6399E-10	NaN	1.8432E-4	0.0000	2.7776E-19	5.4172E-16	0.094279	0.0000	0.0000	1.2037E-4	1.0000
0.0000	1.0000E-6	0.97000	-6.6394E-10	NaN	1.8430E-4	0.0000	2.6791E-19	5.4168E-16	0.094271	0.0000	0.0000	1.2036E-4	1.0000
0.0000	1.0000E-6	0.99000	-6.6389E-10	NaN	1.8429E-4	0.0000	2.5876E-19	5.4164E-16	0.094265	0.0000	0.0000	1.2035E-4	1.0000
0.00	1.5000E-	0.00	-7.15	NaN	2.2700E-	0.00	7.3358E-	1.4477E-	0.22	0.00	0.00	3.7290E-	1.00

k0Sub (m/s)	d (m)	Time (s)	-(tds2.ntflux_Oxi)*n*F (A), UME	Surface ConcOxi (M), Oxi Mem	Surface ConcOxi (M), OxiNuc	Substrate Kinetics (m/s), k0Sub probe	Normal total flux (mol/s), UME OCR membrane	Normal total flux (mol/s), UME OCR nucleus	Concentration (mol/m^3), Point: (5.5109E-16, 2.5)	k0Sub*Oxi (mol/s), Mem Flux	k0Sub*Oxi (mol/s), Nuc Flux	Normal total flux (mol/(m^2*s)), Point: (5.5109E-16, 2.5)	WEIectrode radius (μm), UME Radius
00	6	00	34E-9		4	00	28	29	700	00	00	16	00
0.0000	1.5000E-6	0.005000	-8.1796E-10	NaN	2.1331E-4	0.0000	7.3030E-16	4.5103E-16	0.14619	0.0000	0.0000	8.6780E-5	1.0000
0.0000	1.5000E-6	0.010000	-7.7421E-10	NaN	2.0632E-4	0.0000	4.0760E-16	4.6762E-16	0.13820	0.0000	0.0000	8.1990E-5	1.0000
0.0000	1.5000E-6	0.015000	-7.5228E-10	NaN	2.0205E-4	0.0000	2.5503E-16	4.6722E-16	0.13423	0.0000	0.0000	7.9625E-5	1.0000
0.0000	1.5000E-6	0.020000	-7.3906E-10	NaN	1.9927E-4	0.0000	1.7349E-16	4.6491E-16	0.13185	0.0000	0.0000	7.8206E-5	1.0000
0.0000	1.5000E-6	0.025000	-7.2994E-10	NaN	1.9730E-4	0.0000	1.2257E-16	4.6299E-16	0.13021	0.0000	0.0000	7.7229E-5	1.0000
0.0000	1.5000E-6	0.030000	-7.2332E-10	NaN	1.9585E-4	0.0000	8.8298E-17	4.6145E-16	0.12902	0.0000	0.0000	7.6519E-5	1.0000
0.0000	1.5000E-6	0.035000	-7.1858E-10	NaN	1.9478E-4	0.0000	6.6089E-17	4.6015E-16	0.12817	0.0000	0.0000	7.6011E-5	1.0000
0.0000	1.5000E-6	0.040000	-7.1469E-10	NaN	1.9390E-4	0.0000	4.8680E-17	4.5900E-16	0.12747	0.0000	0.0000	7.5596E-5	1.0000
0.0000	1.5000E-6	0.045000	-7.1167E-10	NaN	1.9320E-4	0.0000	3.6095E-17	4.5802E-16	0.12693	0.0000	0.0000	7.5273E-5	1.0000

k0Sub (m/s)	d (m)	Time (s)	-(tds2.ntflux_Oxi)*n*F (A), UME	Surface ConcOxi (M), OxiMem	Surface ConcOxi (M), OxiNuc	Substrate Kinetics (m/s), k0Sub probe	Normal total flux (mol/s), UME OCR membrane	Normal total flux (mol/s), UME OCR nucleus	Concentration (mol/m^3), Point: (5.5109E-16, 2.5)	k0Sub*Oxi (mol/s), Mem Flux	k0Sub*Oxi (mol/s), Nuc Flux	Normal total flux (mol/(m^2*s)), Point: (5.5109E-16, 2.5)	WElectrode radius (μm), UME Radius
0.0000	1.5000E-6	0.050000	-7.0945E-10	NaN	1.9268E-4	0.0000	2.8003E-17	4.5719E-16	0.12653	0.0000	0.0000	7.5036E-5	1.0000
0.0000	1.5000E-6	0.055000	-7.0753E-10	NaN	1.9222E-4	0.0000	2.1296E-17	4.5645E-16	0.12618	0.0000	0.0000	7.4832E-5	1.0000
0.0000	1.5000E-6	0.060000	-7.0618E-10	NaN	1.9189E-4	0.0000	1.7609E-17	4.5583E-16	0.12594	0.0000	0.0000	7.4688E-5	1.0000
0.0000	1.5000E-6	0.065000	-7.0512E-10	NaN	1.9162E-4	0.0000	1.5316E-17	4.5530E-16	0.12575	0.0000	0.0000	7.4576E-5	1.0000
0.0000	1.5000E-6	0.070000	-7.0415E-10	NaN	1.9138E-4	0.0000	1.3106E-17	4.5482E-16	0.12558	0.0000	0.0000	7.4472E-5	1.0000
0.0000	1.5000E-6	0.075000	-7.0342E-10	NaN	1.9119E-4	0.0000	1.1995E-17	4.5442E-16	0.12545	0.0000	0.0000	7.4395E-5	1.0000
0.0000	1.5000E-6	0.080000	-7.0269E-10	NaN	1.9100E-4	0.0000	1.0670E-17	4.5403E-16	0.12532	0.0000	0.0000	7.4318E-5	1.0000
0.0000	1.5000E-6	0.085000	-7.0200E-10	NaN	1.9082E-4	0.0000	9.3469E-18	4.5367E-16	0.12519	0.0000	0.0000	7.4244E-5	1.0000
0.0000	1.5000E-6	0.090000	-7.0138E-10	NaN	1.9067E-4	0.0000	8.2223E-18	4.5334E-16	0.12508	0.0000	0.0000	7.4178E-5	1.0000
0.0000	1.5000E-6	0.095000	-7.0083E-	NaN	1.9053E-4	0.0000	7.2962E-18	4.5305E-16	0.12498	0.0000	0.0000	7.4120E-5	1.0000

k0Sub (m/s)	d (m)	Time (s)	-(tds2.ntflux_Oxi)*n*F (A), UME	Surface ConcOxi (M), OxiMem	Surface ConcOxi (M), OxiNuc	Substrate Kinetics (m/s), k0Sub probe	Normal total flux (mol/s), UME OCR membrane	Normal total flux (mol/s), UME OCR nucleus	Concentration (mol/m^3), Point: (5.5109E-16, 2.5)	k0Sub*Oxi (mol/s), Mem Flux	k0Sub*Oxi (mol/s), Nuc Flux	Normal total flux (mol/(m^2*s)), Point: (5.5109E-16, 2.5)	WElectrode radius (µm), UME Radius
			10										
0.0000	1.5000E-6	0.10000	-7.0036E-10	NaN	1.9040E-4	0.0000	6.5687E-18	4.5280E-16	0.12490	0.0000	0.0000	7.4070E-5	1.0000
0.0000	1.5000E-6	0.10500	-7.0003E-10	NaN	1.9032E-4	0.0000	6.1713E-18	4.5261E-16	0.12484	0.0000	0.0000	7.4035E-5	1.0000
0.0000	1.5000E-6	0.11000	-6.9981E-10	NaN	1.9026E-4	0.0000	5.9986E-18	4.5248E-16	0.12480	0.0000	0.0000	7.4011E-5	1.0000
0.0000	1.5000E-6	0.11500	-6.9959E-10	NaN	1.9020E-4	0.0000	5.8260E-18	4.5234E-16	0.12476	0.0000	0.0000	7.3988E-5	1.0000
0.0000	1.5000E-6	0.12000	-6.9936E-10	NaN	1.9014E-4	0.0000	5.6533E-18	4.5221E-16	0.12472	0.0000	0.0000	7.3964E-5	1.0000
0.0000	1.5000E-6	0.12500	-6.9914E-10	NaN	1.9008E-4	0.0000	5.4806E-18	4.5208E-16	0.12468	0.0000	0.0000	7.3941E-5	1.0000
0.0000	1.5000E-6	0.13000	-6.9892E-10	NaN	1.9002E-4	0.0000	5.3080E-18	4.5195E-16	0.12464	0.0000	0.0000	7.3917E-5	1.0000
0.0000	1.5000E-6	0.13500	-6.9870E-10	NaN	1.8996E-4	0.0000	5.1353E-18	4.5181E-16	0.12460	0.0000	0.0000	7.3894E-5	1.0000
0.0000	1.5000E-6	0.14000	-6.9848E-10	NaN	1.8991E-4	0.0000	4.9626E-18	4.5168E-16	0.12456	0.0000	0.0000	7.3871E-5	1.0000
0.00	1.5000E-	0.14	-6.98	NaN	1.8985E-	0.00	4.7899E-	4.5155E-	0.12	0.00	0.00	7.3847E-	1.00

k0Sub (m/s)	d (m)	Time (s)	-(tds2.ntflux_Oxi)*n*F (A), UME	Surface ConcOxi (M), Oxi Mem	Surface ConcOxi (M), OxiNuc	Substrate Kinetics (m/s), k0Sub probe	Normal total flux (mol/s), UME OCR membrane	Normal total flux (mol/s), UME OCR nucleus	Concentration (mol/m^3), Point: (5.5109E-16, 2.5)	k0Sub*Oxi (mol/s), Mem Flux	k0Sub*Oxi (mol/s), Nuc Flux	Normal total flux (mol/(m^2*s)), Point: (5.5109E-16, 2.5)	WElectrode radius (µm), UME Radius
00	6	500	26E-10		4	00	18	16	452	00	00	5	00
0.0000	1.5000E-6	0.15000	-6.9803E-10	NaN	1.8979E-4	0.0000	4.6173E-18	4.5141E-16	0.12448	0.0000	0.0000	7.3824E-5	1.0000
0.0000	1.5000E-6	0.15500	-6.9781E-10	NaN	1.8973E-4	0.0000	4.4446E-18	4.5128E-16	0.12445	0.0000	0.0000	7.3800E-5	1.0000
0.0000	1.5000E-6	0.16000	-6.9763E-10	NaN	1.8968E-4	0.0000	4.3071E-18	4.5118E-16	0.12441	0.0000	0.0000	7.3781E-5	1.0000
0.0000	1.5000E-6	0.16500	-6.9748E-10	NaN	1.8964E-4	0.0000	4.1869E-18	4.5109E-16	0.12439	0.0000	0.0000	7.3765E-5	1.0000
0.0000	1.5000E-6	0.17000	-6.9733E-10	NaN	1.8960E-4	0.0000	4.0668E-18	4.5100E-16	0.12436	0.0000	0.0000	7.3749E-5	1.0000
0.0000	1.5000E-6	0.17500	-6.9718E-10	NaN	1.8956E-4	0.0000	3.9466E-18	4.5091E-16	0.12433	0.0000	0.0000	7.3733E-5	1.0000
0.0000	1.5000E-6	0.18000	-6.9703E-10	NaN	1.8952E-4	0.0000	3.8265E-18	4.5081E-16	0.12431	0.0000	0.0000	7.3717E-5	1.0000
0.0000	1.5000E-6	0.18500	-6.9688E-10	NaN	1.8948E-4	0.0000	3.7064E-18	4.5072E-16	0.12428	0.0000	0.0000	7.3701E-5	1.0000
0.0000	1.5000E-6	0.19000	-6.9672E-10	NaN	1.8944E-4	0.0000	3.5862E-18	4.5063E-16	0.12425	0.0000	0.0000	7.3685E-5	1.0000

k0Sub (m/s)	d (m)	Time (s)	-(tds2.ntflux_Oxi)*n*F (A), UME	Surface ConcOxi (M), OxiMem	Surface ConcOxi (M), OxiNuc	Substrate Kinetics (m/s), k0Sub probe	Normal total flux (mol/s), UME OCR membrane	Normal total flux (mol/s), UME OCR nucleus	Concentration (mol/m^3), Point: (5.5109E-16, 2.5)	k0Sub*Oxi (mol/s), Mem Flux	k0Sub*Oxi (mol/s), Nuc Flux	Normal total flux (mol/(m^2*s)), Point: (5.5109E-16, 2.5)	WElectrode radius (µm), UME Radius
0.0000	1.5000E-6	0.19500	-6.9657E-10	NaN	1.8940E-4	0.0000	3.4661E-18	4.5054E-16	0.12422	0.0000	0.0000	7.3669E-5	1.0000
0.0000	1.5000E-6	0.20000	-6.9642E-10	NaN	1.8936E-4	0.0000	3.3459E-18	4.5045E-16	0.12420	0.0000	0.0000	7.3653E-5	1.0000
0.0000	1.5000E-6	0.20500	-6.9627E-10	NaN	1.8932E-4	0.0000	3.2258E-18	4.5036E-16	0.12417	0.0000	0.0000	7.3637E-5	1.0000
0.0000	1.5000E-6	0.21000	-6.9612E-10	NaN	1.8928E-4	0.0000	3.1057E-18	4.5027E-16	0.12414	0.0000	0.0000	7.3621E-5	1.0000
0.0000	1.5000E-6	0.21500	-6.9602E-10	NaN	1.8925E-4	0.0000	3.0352E-18	4.5021E-16	0.12412	0.0000	0.0000	7.3610E-5	1.0000
0.0000	1.5000E-6	0.22000	-6.9593E-10	NaN	1.8923E-4	0.0000	2.9730E-18	4.5016E-16	0.12411	0.0000	0.0000	7.3600E-5	1.0000
0.0000	1.5000E-6	0.22500	-6.9583E-10	NaN	1.8921E-4	0.0000	2.9107E-18	4.5010E-16	0.12409	0.0000	0.0000	7.3591E-5	1.0000
0.0000	1.5000E-6	0.23000	-6.9574E-10	NaN	1.8918E-4	0.0000	2.8485E-18	4.5005E-16	0.12408	0.0000	0.0000	7.3581E-5	1.0000
0.0000	1.5000E-6	0.23500	-6.9565E-10	NaN	1.8916E-4	0.0000	2.7862E-18	4.4999E-16	0.12406	0.0000	0.0000	7.3571E-5	1.0000
0.0000	1.5000E-6	0.24000	-6.9556E-	NaN	1.8913E-4	0.0000	2.7239E-18	4.4994E-16	0.12404	0.0000	0.0000	7.3562E-5	1.0000

k0Sub (m/s)	d (m)	Time (s)	-(tds2.ntflux_Oxi)*n*F (A), UME	Surface ConcOxi (M), Oxi Mem	Surface ConcOxi (M), OxiNuc	Substrate Kinetics (m/s), k0Sub probe	Normal total flux (mol/s), UME OCR membrane	Normal total flux (mol/s), UME OCR nucleus	Concentration (mol/m^3), Point: (5.5109E-16, 2.5)	k0Sub*Oxi (mol/s), Mem Flux	k0Sub*Oxi (mol/s), Nuc Flux	Normal total flux (mol/(m^2*s)), Point: (5.5109E-16, 2.5)	WEIectrode radius (µm), UME Radius
			10										
0.0000	1.5000E-6	0.24500	-6.9547E-10	NaN	1.8911E-4	0.0000	2.6617E-18	4.4988E-16	0.12403	0.0000	0.0000	7.3552E-5	1.0000
0.0000	1.5000E-6	0.25000	-6.9538E-10	NaN	1.8908E-4	0.0000	2.5994E-18	4.4983E-16	0.12401	0.0000	0.0000	7.3542E-5	1.0000
0.0000	1.5000E-6	0.25000	-6.9538E-10	NaN	1.8908E-4	0.0000	2.5994E-18	4.4983E-16	0.12401	0.0000	0.0000	7.3542E-5	1.0000
0.0000	1.5000E-6	0.27000	-6.9501E-10	NaN	1.8899E-4	0.0000	2.3504E-18	4.4961E-16	0.12395	0.0000	0.0000	7.3503E-5	1.0000
0.0000	1.5000E-6	0.29000	-6.9465E-10	NaN	1.8889E-4	0.0000	2.1013E-18	4.4939E-16	0.12388	0.0000	0.0000	7.3465E-5	1.0000
0.0000	1.5000E-6	0.31000	-6.9428E-10	NaN	1.8879E-4	0.0000	1.8541E-18	4.4917E-16	0.12381	0.0000	0.0000	7.3426E-5	1.0000
0.0000	1.5000E-6	0.33000	-6.9404E-10	NaN	1.8873E-4	0.0000	1.7247E-18	4.4902E-16	0.12377	0.0000	0.0000	7.3401E-5	1.0000
0.0000	1.5000E-6	0.35000	-6.9381E-10	NaN	1.8867E-4	0.0000	1.5953E-18	4.4888E-16	0.12373	0.0000	0.0000	7.3376E-5	1.0000
0.0000	1.5000E-6	0.37000	-6.9357E-10	NaN	1.8860E-4	0.0000	1.4659E-18	4.4873E-16	0.12369	0.0000	0.0000	7.3351E-5	1.0000
0.00	1.5000E-	0.39	-6.93	NaN	1.8854E-	0.00	1.3365E-	4.4859E-	0.12	0.00	0.00	7.3325E-	1.00

k0Sub (m/s)	d (m)	Time (s)	-(tds2.ntflux_Oxi)*n*F (A), UME	Surface ConcOxi (M), OxiMem	Surface ConcOxi (M), OxiNuc	Substrate Kinetics (m/s), k0Sub probe	Normal total flux (mol/s), UME OCR membrane	Normal total flux (mol/s), UME OCR nucleus	Concentration (mol/m^3), Point: (5.5109E-16, 2.5)	k0Sub*Oxi (mol/s), MemFlux	k0Sub*Oxi (mol/s), NucFlux	Normal total flux (mol/(m^2*s)), Point: (5.5109E-16, 2.5)	WEIectrode radius (μm), UME Radius
00	6	000	33E-10		4	00	18	16	365	00	00	5	00
0.0000	1.5000E-6	0.41000	-6.9310E-10	NaN	1.8848E-4	0.0000	1.2109E-18	4.4844E-16	0.12360	0.0000	0.0000	7.3301E-5	1.0000
0.0000	1.5000E-6	0.43000	-6.9293E-10	NaN	1.8843E-4	0.0000	1.1385E-18	4.4834E-16	0.12357	0.0000	0.0000	7.3283E-5	1.0000
0.0000	1.5000E-6	0.45000	-6.9276E-10	NaN	1.8839E-4	0.0000	1.0662E-18	4.4824E-16	0.12354	0.0000	0.0000	7.3265E-5	1.0000
0.0000	1.5000E-6	0.47000	-6.9260E-10	NaN	1.8834E-4	0.0000	9.9390E-19	4.4813E-16	0.12351	0.0000	0.0000	7.3248E-5	1.0000
0.0000	1.5000E-6	0.49000	-6.9243E-10	NaN	1.8830E-4	0.0000	9.2158E-19	4.4803E-16	0.12348	0.0000	0.0000	7.3230E-5	1.0000
0.0000	1.5000E-6	0.51000	-6.9227E-10	NaN	1.8825E-4	0.0000	8.5264E-19	4.4793E-16	0.12346	0.0000	0.0000	7.3213E-5	1.0000
0.0000	1.5000E-6	0.53000	-6.9214E-10	NaN	1.8822E-4	0.0000	8.0878E-19	4.4785E-16	0.12343	0.0000	0.0000	7.3200E-5	1.0000
0.0000	1.5000E-6	0.55000	-6.9202E-10	NaN	1.8819E-4	0.0000	7.6492E-19	4.4778E-16	0.12341	0.0000	0.0000	7.3187E-5	1.0000
0.0000	1.5000E-6	0.57000	-6.9189E-10	NaN	1.8815E-4	0.0000	7.2106E-19	4.4770E-16	0.12339	0.0000	0.0000	7.3174E-5	1.0000

k0Sub (m/s)	d (m)	Time (s)	-(tds2.ntflux_Oxi)*n*F (A), UME	Surface ConcOxi (M), OxiMem	Surface ConcOxi (M), OxiNuc	Substrate Kinetics (m/s), k0Sub probe	Normal total flux (mol/s), UME OCR membrane	Normal total flux (mol/s), UME OCR nucleus	Concentration (mol/m^3), Point: (5.5109E-16, 2.5)	k0Sub*Oxi (mol/s), Mem Flux	k0Sub*Oxi (mol/s), Nuc Flux	Normal total flux (mol/(m^2*s)), Point: (5.5109E-16, 2.5)	WElectrode radius (μm), UME Radius
0.0000	1.5000E-6	0.59000	-6.9177E-10	NaN	1.8812E-4	0.0000	6.7720E-19	4.4762E-16	0.12337	0.0000	0.0000	7.3160E-5	1.0000
0.0000	1.5000E-6	0.61000	-6.9165E-10	NaN	1.8809E-4	0.0000	6.3573E-19	4.4755E-16	0.12335	0.0000	0.0000	7.3148E-5	1.0000
0.0000	1.5000E-6	0.63000	-6.9155E-10	NaN	1.8806E-4	0.0000	6.0581E-19	4.4748E-16	0.12333	0.0000	0.0000	7.3137E-5	1.0000
0.0000	1.5000E-6	0.65000	-6.9145E-10	NaN	1.8803E-4	0.0000	5.7590E-19	4.4742E-16	0.12331	0.0000	0.0000	7.3127E-5	1.0000
0.0000	1.5000E-6	0.67000	-6.9135E-10	NaN	1.8801E-4	0.0000	5.4599E-19	4.4736E-16	0.12329	0.0000	0.0000	7.3116E-5	1.0000
0.0000	1.5000E-6	0.69000	-6.9125E-10	NaN	1.8798E-4	0.0000	5.1607E-19	4.4730E-16	0.12327	0.0000	0.0000	7.3106E-5	1.0000
0.0000	1.5000E-6	0.71000	-6.9116E-10	NaN	1.8796E-4	0.0000	4.8837E-19	4.4724E-16	0.12326	0.0000	0.0000	7.3096E-5	1.0000
0.0000	1.5000E-6	0.73000	-6.9108E-10	NaN	1.8793E-4	0.0000	4.6833E-19	4.4719E-16	0.12324	0.0000	0.0000	7.3087E-5	1.0000
0.0000	1.5000E-6	0.75000	-6.9100E-10	NaN	1.8791E-4	0.0000	4.4829E-19	4.4714E-16	0.12323	0.0000	0.0000	7.3079E-5	1.0000
0.0000	1.5000E-6	0.77000	-6.9092E-	NaN	1.8789E-4	0.0000	4.2825E-19	4.4709E-16	0.12322	0.0000	0.0000	7.3071E-5	1.0000

k0Sub (m/s)	d (m)	Time (s)	-(tds2.ntflux_Oxi)*n*F (A), UME	Surface ConcOxi (M), Oxi Mem	Surface ConcOxi (M), OxiNuc	Substrate Kinetics (m/s), k0Sub probe	Normal total flux (mol/s), UME OCR membrane	Normal total flux (mol/s), UME OCR nucleus	Concentration (mol/m^3), Point: (5.5109E-16, 2.5)	k0Sub*Oxi (mol/s), Mem Flux	k0Sub*Oxi (mol/s), Nuc Flux	Normal total flux (mol/(m^2*s)), Point: (5.5109E-16, 2.5)	WElectrode radius (µm), UME Radius
			10										
0.0000	1.5000E-6	0.79000	-6.9084E-10	NaN	1.8787E-4	0.0000	4.0822E-19	4.4704E-16	0.12320	0.0000	0.0000	7.3062E-5	1.0000
0.0000	1.5000E-6	0.81000	-6.9077E-10	NaN	1.8785E-4	0.0000	3.8899E-19	4.4699E-16	0.12319	0.0000	0.0000	7.3054E-5	1.0000
0.0000	1.5000E-6	0.83000	-6.9070E-10	NaN	1.8783E-4	0.0000	3.7260E-19	4.4695E-16	0.12318	0.0000	0.0000	7.3047E-5	1.0000
0.0000	1.5000E-6	0.85000	-6.9063E-10	NaN	1.8781E-4	0.0000	3.5733E-19	4.4690E-16	0.12316	0.0000	0.0000	7.3040E-5	1.0000
0.0000	1.5000E-6	0.87000	-6.9056E-10	NaN	1.8779E-4	0.0000	3.4318E-19	4.4686E-16	0.12315	0.0000	0.0000	7.3033E-5	1.0000
0.0000	1.5000E-6	0.89000	-6.9050E-10	NaN	1.8778E-4	0.0000	3.3015E-19	4.4682E-16	0.12314	0.0000	0.0000	7.3026E-5	1.0000
0.0000	1.5000E-6	0.91000	-6.9044E-10	NaN	1.8776E-4	0.0000	3.1794E-19	4.4678E-16	0.12313	0.0000	0.0000	7.3019E-5	1.0000
0.0000	1.5000E-6	0.93000	-6.9038E-10	NaN	1.8775E-4	0.0000	3.0593E-19	4.4675E-16	0.12312	0.0000	0.0000	7.3013E-5	1.0000
0.0000	1.5000E-6	0.95000	-6.9032E-10	NaN	1.8773E-4	0.0000	2.9468E-19	4.4671E-16	0.12311	0.0000	0.0000	7.3007E-5	1.0000
0.00	1.5000E-	0.97	-6.90	NaN	1.8771E-	0.00	2.8418E-	4.4667E-	0.12	0.00	0.00	7.3001E-	1.00

k0Sub (m/s)	d (m)	Time (s)	-(tds2.ntflux_Oxi)*n*F (A), UME	Surface ConcOxi (M), OxiMem	Surface ConcOxi (M), OxiNuc	Substrate Kinetics (m/s), k0Sub probe	Normal total flux (mol/s), UME OCR membrane	Normal total flux (mol/s), UME OCR nucleus	Concentration (mol/m^3), Point: (5.5109E-16, 2.5)	k0Sub*Oxi (mol/s), Mem Flux	k0Sub*Oxi (mol/s), Nuc Flux	Normal total flux (mol/(m^2*s)), Point: (5.5109E-16, 2.5)	WEIectrode radius (µm), UME Radius
00	6	000	27E-10		4	00	19	16	310	00	00	5	00
0.0000	1.5000E-6	0.99000	-6.9021E-10	NaN	1.8770E-4	0.0000	2.7443E-19	4.4664E-16	0.12309	0.0000	0.0000	7.2996E-5	1.0000
0.0000	2.0000E-6	0.0000	-7.1541E-9	NaN	2.2700E-4	0.0000	-1.2387E-28	-3.7786E-30	0.22700	0.0000	0.0000	0.0000	1.0000
0.0000	2.0000E-6	0.00050000	-8.3272E-10	NaN	2.1578E-4	0.0000	6.6767E-16	3.6492E-16	0.16805	0.0000	0.0000	5.4329E-5	1.0000
0.0000	2.0000E-6	0.010000	-7.9304E-10	NaN	2.0938E-4	0.0000	3.8338E-16	3.8301E-16	0.15985	0.0000	0.0000	5.1661E-5	1.0000
0.0000	2.0000E-6	0.015000	-7.7241E-10	NaN	2.0537E-4	0.0000	2.4604E-16	3.8496E-16	0.15563	0.0000	0.0000	5.0291E-5	1.0000
0.0000	2.0000E-6	0.020000	-7.5904E-10	NaN	2.0263E-4	0.0000	1.6721E-16	3.8497E-16	0.15290	0.0000	0.0000	4.9405E-5	1.0000
0.0000	2.0000E-6	0.025000	-7.4975E-10	NaN	2.0066E-4	0.0000	1.1704E-16	3.8439E-16	0.15101	0.0000	0.0000	4.8791E-5	1.0000
0.0000	2.0000E-6	0.030000	-7.4316E-10	NaN	1.9922E-4	0.0000	8.4883E-17	3.8357E-16	0.14967	0.0000	0.0000	4.8357E-5	1.0000
0.0000	2.0000E-6	0.035000	-7.3850E-10	NaN	1.9817E-4	0.0000	6.4697E-17	3.8268E-16	0.14872	0.0000	0.0000	4.8050E-5	1.0000

k0Sub (m/s)	d (m)	Time (s)	-(tds2.ntflux_Oxi)*n*F (A), UME	Surface ConcOxi (M), Oxi Mem	Surface ConcOxi (M), OxiNuc	Substrate Kinetics (m/s), k0Sub probe	Normal total flux (mol/s), UME OCR membrane	Normal total flux (mol/s), UME OCR nucleus	Concentration (mol/m^3), Point: (5.5109E-16, 2.5)	k0Sub*Oxi (mol/s), Mem Flux	k0Sub*Oxi (mol/s), Nuc Flux	Normal total flux (mol/(m^2*s)), Point: (5.5109E-16, 2.5)	WElectrode radius (µm), UME Radius
0.0000	2.0000E-6	0.040000	-7.3497E-10	NaN	1.9735E-4	0.0000	5.0836E-17	3.8188E-16	0.14801	0.0000	0.0000	4.7818E-5	1.0000
0.0000	2.0000E-6	0.045000	-7.3192E-10	NaN	1.9665E-4	0.0000	3.9083E-17	3.8119E-16	0.14739	0.0000	0.0000	4.7618E-5	1.0000
0.0000	2.0000E-6	0.050000	-7.2958E-10	NaN	1.9610E-4	0.0000	3.0890E-17	3.8059E-16	0.14692	0.0000	0.0000	4.7464E-5	1.0000
0.0000	2.0000E-6	0.055000	-7.2765E-10	NaN	1.9564E-4	0.0000	2.4595E-17	3.8006E-16	0.14652	0.0000	0.0000	4.7337E-5	1.0000
0.0000	2.0000E-6	0.060000	-7.2609E-10	NaN	1.9526E-4	0.0000	2.0047E-17	3.7958E-16	0.14621	0.0000	0.0000	4.7235E-5	1.0000
0.0000	2.0000E-6	0.065000	-7.2498E-10	NaN	1.9499E-4	0.0000	1.7669E-17	3.7916E-16	0.14598	0.0000	0.0000	4.7163E-5	1.0000
0.0000	2.0000E-6	0.070000	-7.2377E-10	NaN	1.9469E-4	0.0000	1.4445E-17	3.7876E-16	0.14574	0.0000	0.0000	4.7083E-5	1.0000
0.0000	2.0000E-6	0.075000	-7.2270E-10	NaN	1.9443E-4	0.0000	1.1772E-17	3.7840E-16	0.14552	0.0000	0.0000	4.7013E-5	1.0000
0.0000	2.0000E-6	0.080000	-7.2178E-10	NaN	1.9420E-4	0.0000	9.6522E-18	3.7807E-16	0.14534	0.0000	0.0000	4.6953E-5	1.0000
0.0000	2.0000E-6	0.085000	-7.2102E-	NaN	1.9401E-4	0.0000	8.0842E-18	3.7778E-16	0.14518	0.0000	0.0000	4.6903E-5	1.0000

k0Sub (m/s)	d (m)	Time (s)	-(tds2.ntflux_Oxi)*n*F (A), UME	Surface ConcOxi (M), OxiMem	Surface ConcOxi (M), OxiNuc	Substrate Kinetics (m/s), k0Sub probe	Normal total flux (mol/s), UME OCR membrane	Normal total flux (mol/s), UME OCR nucleus	Concentration (mol/m^3), Point: (5.5109E-16, 2.5)	k0Sub*Oxi (mol/s), Mem Flux	k0Sub*Oxi (mol/s), Nuc Flux	Normal total flux (mol/(m^2*s)), Point: (5.5109E-16, 2.5)	WElectrode radius (µm), UME Radius
			10										
0.0000	2.0000E-6	0.090000	-7.2040E-10	NaN	1.9385E-4	0.0000	7.0684E-18	3.7753E-16	0.14506	0.0000	0.0000	4.6863E-5	1.0000
0.0000	2.0000E-6	0.095000	-7.2001E-10	NaN	1.9375E-4	0.0000	6.7474E-18	3.7734E-16	0.14498	0.0000	0.0000	4.6838E-5	1.0000
0.0000	2.0000E-6	0.10000	-7.1970E-10	NaN	1.9367E-4	0.0000	6.6093E-18	3.7719E-16	0.14492	0.0000	0.0000	4.6818E-5	1.0000
0.0000	2.0000E-6	0.10500	-7.1940E-10	NaN	1.9359E-4	0.0000	6.4712E-18	3.7703E-16	0.14486	0.0000	0.0000	4.6798E-5	1.0000
0.0000	2.0000E-6	0.11000	-7.1909E-10	NaN	1.9351E-4	0.0000	6.3331E-18	3.7688E-16	0.14479	0.0000	0.0000	4.6778E-5	1.0000
0.0000	2.0000E-6	0.11500	-7.1878E-10	NaN	1.9342E-4	0.0000	6.1950E-18	3.7672E-16	0.14473	0.0000	0.0000	4.6758E-5	1.0000
0.0000	2.0000E-6	0.12000	-7.1850E-10	NaN	1.9335E-4	0.0000	6.0592E-18	3.7658E-16	0.14467	0.0000	0.0000	4.6739E-5	1.0000
0.0000	2.0000E-6	0.12500	-7.1828E-10	NaN	1.9329E-4	0.0000	5.8917E-18	3.7648E-16	0.14463	0.0000	0.0000	4.6725E-5	1.0000
0.0000	2.0000E-6	0.13000	-7.1807E-10	NaN	1.9324E-4	0.0000	5.7241E-18	3.7638E-16	0.14459	0.0000	0.0000	4.6711E-5	1.0000
0.00	2.0000E-	0.13	-7.17	NaN	1.9318E-	0.00	5.5566E-	3.7628E-	0.14	0.00	0.00	4.6698E-	1.00

k0Sub (m/s)	d (m)	Time (s)	-(tds2.ntflux_Oxi)*n*F (A), UME	Surface ConcOxi (M), OxiMem	Surface ConcOxi (M), OxiNuc	Substrate Kinetics (m/s), k0Sub probe	Normal total flux (mol/s), UME OCR membrane	Normal total flux (mol/s), UME OCR nucleus	Concentration (mol/m^3), Point: (5.5109E-16, 2.5)	k0Sub*Oxi (mol/s), MemFlux	k0Sub*Oxi (mol/s), NucFlux	Normal total flux (mol/(m^2*s)), Point: (5.5109E-16, 2.5)	WElectrode radius (µm), UME Radius
00	6	500	86E-10		4	00	18	16	455	00	00	5	00
0.0000	2.0000E-6	0.14000	-7.1765E-10	NaN	1.9312E-4	0.0000	5.3890E-18	3.7618E-16	0.14450	0.0000	0.0000	4.6684E-5	1.0000
0.0000	2.0000E-6	0.14500	-7.1744E-10	NaN	1.9307E-4	0.0000	5.2215E-18	3.7607E-16	0.14446	0.0000	0.0000	4.6670E-5	1.0000
0.0000	2.0000E-6	0.15000	-7.1722E-10	NaN	1.9301E-4	0.0000	5.0540E-18	3.7597E-16	0.14442	0.0000	0.0000	4.6656E-5	1.0000
0.0000	2.0000E-6	0.15500	-7.1701E-10	NaN	1.9296E-4	0.0000	4.8864E-18	3.7587E-16	0.14438	0.0000	0.0000	4.6642E-5	1.0000
0.0000	2.0000E-6	0.16000	-7.1680E-10	NaN	1.9290E-4	0.0000	4.7189E-18	3.7577E-16	0.14433	0.0000	0.0000	4.6628E-5	1.0000
0.0000	2.0000E-6	0.16500	-7.1659E-10	NaN	1.9285E-4	0.0000	4.5513E-18	3.7567E-16	0.14429	0.0000	0.0000	4.6615E-5	1.0000
0.0000	2.0000E-6	0.17000	-7.1637E-10	NaN	1.9279E-4	0.0000	4.3838E-18	3.7557E-16	0.14425	0.0000	0.0000	4.6601E-5	1.0000
0.0000	2.0000E-6	0.17500	-7.1620E-10	NaN	1.9274E-4	0.0000	4.2525E-18	3.7549E-16	0.14421	0.0000	0.0000	4.6589E-5	1.0000
0.0000	2.0000E-6	0.18000	-7.1608E-10	NaN	1.9271E-4	0.0000	4.1593E-18	3.7543E-16	0.14419	0.0000	0.0000	4.6581E-5	1.0000

k0Sub (m/s)	d (m)	Time (s)	-(tds2.ntflux_Oxi)*n*F (A), UME	Surface ConcOxi (M), OxiMem	Surface ConcOxi (M), OxiNuc	Substrate Kinetics (m/s), k0Sub probe	Normal total flux (mol/s), UME OCR membrane	Normal total flux (mol/s), UME OCR nucleus	Concentration (mol/m^3), Point: (5.5109E-16, 2.5)	k0Sub*Oxi (mol/s), Mem Flux	k0Sub*Oxi (mol/s), Nuc Flux	Normal total flux (mol/(m^2*s)), Point: (5.5109E-16, 2.5)	WElectrode radius (µm), UME Radius
0.0000	2.0000E-6	0.18500	-7.1595E-10	NaN	1.9268E-4	0.0000	4.0660E-18	3.7537E-16	0.14416	0.0000	0.0000	4.6573E-5	1.0000
0.0000	2.0000E-6	0.19000	-7.1583E-10	NaN	1.9265E-4	0.0000	3.9728E-18	3.7531E-16	0.14414	0.0000	0.0000	4.6565E-5	1.0000
0.0000	2.0000E-6	0.19500	-7.1571E-10	NaN	1.9262E-4	0.0000	3.8796E-18	3.7526E-16	0.14411	0.0000	0.0000	4.6557E-5	1.0000
0.0000	2.0000E-6	0.20000	-7.1559E-10	NaN	1.9258E-4	0.0000	3.7864E-18	3.7520E-16	0.14409	0.0000	0.0000	4.6549E-5	1.0000
0.0000	2.0000E-6	0.20500	-7.1546E-10	NaN	1.9255E-4	0.0000	3.6932E-18	3.7514E-16	0.14406	0.0000	0.0000	4.6541E-5	1.0000
0.0000	2.0000E-6	0.21000	-7.1534E-10	NaN	1.9252E-4	0.0000	3.6000E-18	3.7508E-16	0.14404	0.0000	0.0000	4.6533E-5	1.0000
0.0000	2.0000E-6	0.21500	-7.1522E-10	NaN	1.9249E-4	0.0000	3.5068E-18	3.7503E-16	0.14401	0.0000	0.0000	4.6525E-5	1.0000
0.0000	2.0000E-6	0.22000	-7.1510E-10	NaN	1.9246E-4	0.0000	3.4136E-18	3.7497E-16	0.14399	0.0000	0.0000	4.6518E-5	1.0000
0.0000	2.0000E-6	0.22500	-7.1498E-10	NaN	1.9242E-4	0.0000	3.3204E-18	3.7491E-16	0.14396	0.0000	0.0000	4.6510E-5	1.0000
0.0000	2.0000E-6	0.23000	-7.1485E-	NaN	1.9239E-4	0.0000	3.2272E-18	3.7485E-16	0.14394	0.0000	0.0000	4.6502E-5	1.0000

k0Sub (m/s)	d (m)	Time (s)	-(tds2.ntflux_Oxi)*n*F (A), UME	Surface ConcOxi (M), OxiMem	Surface ConcOxi (M), OxiNuc	Substrate Kinetics (m/s), k0Sub probe	Normal total flux (mol/s), UME OCR membrane	Normal total flux (mol/s), UME OCR nucleus	Concentration (mol/m^3), Point: (5.5109E-16, 2.5)	k0Sub*Oxi (mol/s), MemFlux	k0Sub*Oxi (mol/s), NucFlux	Normal total flux (mol/(m^2*s)), Point: (5.5109E-16, 2.5)	WElectrode radius (µm), UME Radius
			10										
0.0000	2.0000E-6	0.23500	-7.1473E-10	NaN	1.9236E-4	0.0000	3.1340E-18	3.7479E-16	0.14392	0.0000	0.0000	4.6494E-5	1.0000
0.0000	2.0000E-6	0.24000	-7.1461E-10	NaN	1.9233E-4	0.0000	3.0408E-18	3.7474E-16	0.14389	0.0000	0.0000	4.6486E-5	1.0000
0.0000	2.0000E-6	0.24500	-7.1449E-10	NaN	1.9230E-4	0.0000	2.9476E-18	3.7468E-16	0.14387	0.0000	0.0000	4.6478E-5	1.0000
0.0000	2.0000E-6	0.25000	-7.1436E-10	NaN	1.9226E-4	0.0000	2.8544E-18	3.7462E-16	0.14384	0.0000	0.0000	4.6470E-5	1.0000
0.0000	2.0000E-6	0.25000	-7.1436E-10	NaN	1.9226E-4	0.0000	2.8544E-18	3.7462E-16	0.14384	0.0000	0.0000	4.6470E-5	1.0000
0.0000	2.0000E-6	0.27000	-7.1387E-10	NaN	1.9214E-4	0.0000	2.4815E-18	3.7439E-16	0.14374	0.0000	0.0000	4.6438E-5	1.0000
0.0000	2.0000E-6	0.29000	-7.1355E-10	NaN	1.9205E-4	0.0000	2.2796E-18	3.7423E-16	0.14368	0.0000	0.0000	4.6417E-5	1.0000
0.0000	2.0000E-6	0.31000	-7.1325E-10	NaN	1.9197E-4	0.0000	2.0930E-18	3.7409E-16	0.14362	0.0000	0.0000	4.6397E-5	1.0000
0.0000	2.0000E-6	0.33000	-7.1295E-10	NaN	1.9189E-4	0.0000	1.9064E-18	3.7394E-16	0.14356	0.0000	0.0000	4.6377E-5	1.0000
0.00	2.0000E-	0.35	-7.12	NaN	1.9181E-	0.00	1.7199E-	3.7379E-	0.14	0.00	0.00	4.6358E-	1.00

k0Sub (m/s)	d (m)	Time (s)	-(tds2.ntflux_Oxi)*n*F (A), UME	Surface ConcOxi (M), Oxi Mem	Surface ConcOxi (M), OxiNuc	Substrate Kinetics (m/s), k0Sub probe	Normal total flux (mol/s), UME OCR membrane	Normal total flux (mol/s), UME OCR nucleus	Concentration (mol/m^3), Point: (5.5109E-16, 2.5)	k0Sub* Oxi (mol/s), Mem Flux	k0Sub* Oxi (mol/s), Nuc Flux	Normal total flux (mol/(m^2*s)), Point: (5.5109E-16, 2.5)	WElectrode radius (µm), UME Radius
00	6	000	64E-10		4	00	18	16	349	00	00	5	00
0.0000	2.0000E-6	0.37000	-7.1234E-10	NaN	1.9173E-4	0.0000	1.5333E-18	3.7365E-16	0.14343	0.0000	0.0000	4.6338E-5	1.0000
0.0000	2.0000E-6	0.39000	-7.1213E-10	NaN	1.9167E-4	0.0000	1.4319E-18	3.7355E-16	0.14339	0.0000	0.0000	4.6324E-5	1.0000
0.0000	2.0000E-6	0.41000	-7.1192E-10	NaN	1.9162E-4	0.0000	1.3333E-18	3.7344E-16	0.14335	0.0000	0.0000	4.6311E-5	1.0000
0.0000	2.0000E-6	0.43000	-7.1172E-10	NaN	1.9157E-4	0.0000	1.2346E-18	3.7334E-16	0.14331	0.0000	0.0000	4.6297E-5	1.0000
0.0000	2.0000E-6	0.45000	-7.1151E-10	NaN	1.9151E-4	0.0000	1.1359E-18	3.7324E-16	0.14327	0.0000	0.0000	4.6284E-5	1.0000
0.0000	2.0000E-6	0.47000	-7.1131E-10	NaN	1.9146E-4	0.0000	1.0372E-18	3.7314E-16	0.14323	0.0000	0.0000	4.6271E-5	1.0000
0.0000	2.0000E-6	0.49000	-7.1116E-10	NaN	1.9142E-4	0.0000	9.8098E-19	3.7307E-16	0.14320	0.0000	0.0000	4.6261E-5	1.0000
0.0000	2.0000E-6	0.51000	-7.1101E-10	NaN	1.9138E-4	0.0000	9.2377E-19	3.7299E-16	0.14317	0.0000	0.0000	4.6251E-5	1.0000
0.0000	2.0000E-6	0.53000	-7.1086E-10	NaN	1.9134E-4	0.0000	8.6656E-19	3.7292E-16	0.14314	0.0000	0.0000	4.6242E-5	1.0000

k0Sub (m/s)	d (m)	Time (s)	-(tds2.ntflux_Oxi)*n*F (A), UME	Surface ConcOxi (M), Oxi Mem	Surface ConcOxi (M), OxiNuc	Substrate Kinetics (m/s), k0Sub probe	Normal total flux (mol/s), UME OCR membrane	Normal total flux (mol/s), UME OCR nucleus	Concentration (mol/m^3), Point: (5.5109E-16, 2.5)	k0Sub*Oxi (mol/s), Mem Flux	k0Sub*Oxi (mol/s), Nuc Flux	Normal total flux (mol/(m^2*s)), Point: (5.5109E-16, 2.5)	WElectrode radius (µm), UME Radius
0.0000	2.0000E-6	0.55000	-7.1072E-10	NaN	1.9130E-4	0.0000	8.0935E-19	3.7285E-16	0.14311	0.0000	0.0000	4.6232E-5	1.0000
0.0000	2.0000E-6	0.57000	-7.1057E-10	NaN	1.9126E-4	0.0000	7.5370E-19	3.7277E-16	0.14308	0.0000	0.0000	4.6223E-5	1.0000
0.0000	2.0000E-6	0.59000	-7.1045E-10	NaN	1.9123E-4	0.0000	7.1582E-19	3.7271E-16	0.14305	0.0000	0.0000	4.6215E-5	1.0000
0.0000	2.0000E-6	0.61000	-7.1034E-10	NaN	1.9120E-4	0.0000	6.7794E-19	3.7266E-16	0.14303	0.0000	0.0000	4.6207E-5	1.0000
0.0000	2.0000E-6	0.63000	-7.1022E-10	NaN	1.9117E-4	0.0000	6.4005E-19	3.7260E-16	0.14301	0.0000	0.0000	4.6200E-5	1.0000
0.0000	2.0000E-6	0.65000	-7.1010E-10	NaN	1.9114E-4	0.0000	6.0217E-19	3.7254E-16	0.14298	0.0000	0.0000	4.6192E-5	1.0000
0.0000	2.0000E-6	0.67000	-7.0999E-10	NaN	1.9111E-4	0.0000	5.6609E-19	3.7248E-16	0.14296	0.0000	0.0000	4.6185E-5	1.0000
0.0000	2.0000E-6	0.69000	-7.0990E-10	NaN	1.9108E-4	0.0000	5.4150E-19	3.7244E-16	0.14294	0.0000	0.0000	4.6179E-5	1.0000
0.0000	2.0000E-6	0.71000	-7.0981E-10	NaN	1.9106E-4	0.0000	5.1691E-19	3.7239E-16	0.14292	0.0000	0.0000	4.6173E-5	1.0000
0.0000	2.0000E-6	0.73000	-7.0972E-	NaN	1.9103E-4	0.0000	4.9232E-19	3.7234E-16	0.14291	0.0000	0.0000	4.6167E-5	1.0000

k0Sub (m/s)	d (m)	Time (s)	-(tds2.ntflux_Oxi)*n*F (A), UME	Surface ConcOxi (M), OxiMem	Surface ConcOxi (M), OxiNuc	Substrate Kinetics (m/s), k0Sub probe	Normal total flux (mol/s), UME OCR membrane	Normal total flux (mol/s), UME OCR nucleus	Concentration (mol/m^3), Point: (5.5109E-16, 2.5)	k0Sub*Oxi (mol/s), Mem Flux	k0Sub*Oxi (mol/s), Nuc Flux	Normal total flux (mol/(m^2*s)), Point: (5.5109E-16, 2.5)	WElectrode radius (µm), UME Radius
			10										
0.0000	2.0000E-6	0.75000	-7.0963E-10	NaN	1.9101E-4	0.0000	4.6773E-19	3.7230E-16	0.14289	0.0000	0.0000	4.6161E-5	1.0000
0.0000	2.0000E-6	0.77000	-7.0954E-10	NaN	1.9099E-4	0.0000	4.4386E-19	3.7225E-16	0.14287	0.0000	0.0000	4.6155E-5	1.0000
0.0000	2.0000E-6	0.79000	-7.0945E-10	NaN	1.9096E-4	0.0000	4.2392E-19	3.7221E-16	0.14285	0.0000	0.0000	4.6150E-5	1.0000
0.0000	2.0000E-6	0.81000	-7.0938E-10	NaN	1.9094E-4	0.0000	4.0546E-19	3.7217E-16	0.14284	0.0000	0.0000	4.6145E-5	1.0000
0.0000	2.0000E-6	0.83000	-7.0930E-10	NaN	1.9092E-4	0.0000	3.8848E-19	3.7213E-16	0.14282	0.0000	0.0000	4.6140E-5	1.0000
0.0000	2.0000E-6	0.85000	-7.0923E-10	NaN	1.9090E-4	0.0000	3.7296E-19	3.7210E-16	0.14281	0.0000	0.0000	4.6135E-5	1.0000
0.0000	2.0000E-6	0.87000	-7.0916E-10	NaN	1.9089E-4	0.0000	3.5862E-19	3.7206E-16	0.14279	0.0000	0.0000	4.6131E-5	1.0000
0.0000	2.0000E-6	0.89000	-7.0909E-10	NaN	1.9087E-4	0.0000	3.4434E-19	3.7203E-16	0.14278	0.0000	0.0000	4.6126E-5	1.0000
0.0000	2.0000E-6	0.91000	-7.0903E-10	NaN	1.9085E-4	0.0000	3.3100E-19	3.7199E-16	0.14277	0.0000	0.0000	4.6122E-5	1.0000
0.00	2.0000E-	0.93	-7.08	NaN	1.9083E-	0.00	3.1861E-	3.7196E-	0.14	0.00	0.00	4.6118E-	1.00

k0Sub (m/s)	d (m)	Time (s)	-(tds2.ntflux_Oxi)*n*F (A), UME	Surface ConcOxi (M), Oxi Mem	Surface ConcOxi (M), OxiNuc	Substrate Kinetics (m/s), k0Sub probe	Normal total flux (mol/s), UME OCR membrane	Normal total flux (mol/s), UME OCR nucleus	Concentration (mol/m^3), Point: (5.5109E-16, 2.5)	k0Sub*Oxi (mol/s), Mem Flux	k0Sub*Oxi (mol/s), Nuc Flux	Normal total flux (mol/(m^2*s)), Point: (5.5109E-16, 2.5)	WElectrode radius (μm), UME Radius
00	6	000	96E-10		4	00	19	16	275	00	00	5	00
0.0000	2.0000E-6	0.95000	-7.0890E-10	NaN	1.9082E-4	0.0000	3.0715E-19	3.7193E-16	0.14274	0.0000	0.0000	4.6114E-5	1.0000
0.0000	2.0000E-6	0.97000	-7.0885E-10	NaN	1.9080E-4	0.0000	2.9642E-19	3.7190E-16	0.14273	0.0000	0.0000	4.6110E-5	1.0000
0.0000	2.0000E-6	0.99000	-7.0879E-10	NaN	1.9079E-4	0.0000	2.8589E-19	3.7187E-16	0.14272	0.0000	0.0000	4.6107E-5	1.0000
0.0000	2.4000E-6	0.0000	-7.1527E-9	NaN	2.2700E-4	0.0000	-8.9760E-30	-6.8528E-30	0.22700	0.0000	0.0000	5.1223E-19	1.0000
0.0000	2.4000E-6	0.0050000	-8.4135E-10	NaN	2.1747E-4	0.0000	6.1744E-16	3.0175E-16	0.18066	0.0000	0.0000	3.9191E-5	1.0000
0.0000	2.4000E-6	0.010000	-8.0249E-10	NaN	2.1140E-4	0.0000	3.5974E-16	3.2591E-16	0.17208	0.0000	0.0000	3.7333E-5	1.0000
0.0000	2.4000E-6	0.015000	-7.8294E-10	NaN	2.0758E-4	0.0000	2.3205E-16	3.2902E-16	0.16782	0.0000	0.0000	3.6406E-5	1.0000
0.0000	2.4000E-6	0.020000	-7.7059E-10	NaN	2.0498E-4	0.0000	1.6080E-16	3.2910E-16	0.16514	0.0000	0.0000	3.5823E-5	1.0000
0.0000	2.4000E-6	0.025000	-7.6172E-10	NaN	2.0308E-4	0.0000	1.1479E-16	3.2902E-16	0.16322	0.0000	0.0000	3.5406E-5	1.0000

k0Sub (m/s)	d (m)	Time (s)	-(tds2.ntflux_Oxi)*n*F (A), UME	Surface ConcOxi (M), OxiMem	Surface ConcOxi (M), OxiNuc	Substrate Kinetics (m/s), k0Sub probe	Normal total flux (mol/s), UME OCR membrane	Normal total flux (mol/s), UME OCR nucleus	Concentration (mol/m^3), Point: (5.5109E-16, 2.5)	k0Sub*Oxi (mol/s), Mem Flux	k0Sub*Oxi (mol/s), Nuc Flux	Normal total flux (mol/(m^2*s)), Point: (5.5109E-16, 2.5)	WElectrode radius (µm), UME Radius
0.0000	2.4000E-6	0.030000	-7.5525E-10	NaN	2.0166E-4	0.0000	8.4246E-17	3.2866E-16	0.16182	0.0000	0.0000	3.5101E-5	1.0000
0.0000	2.4000E-6	0.035000	-7.5039E-10	NaN	2.0057E-4	0.0000	6.3528E-17	3.2818E-16	0.16077	0.0000	0.0000	3.4873E-5	1.0000
0.0000	2.4000E-6	0.040000	-7.4639E-10	NaN	1.9966E-4	0.0000	4.6961E-17	3.2774E-16	0.15990	0.0000	0.0000	3.4685E-5	1.0000
0.0000	2.4000E-6	0.045000	-7.4348E-10	NaN	1.9899E-4	0.0000	3.6467E-17	3.2727E-16	0.15928	0.0000	0.0000	3.4548E-5	1.0000
0.0000	2.4000E-6	0.050000	-7.4097E-10	NaN	1.9840E-4	0.0000	2.7679E-17	3.2684E-16	0.15873	0.0000	0.0000	3.4431E-5	1.0000
0.0000	2.4000E-6	0.055000	-7.3910E-10	NaN	1.9795E-4	0.0000	2.2236E-17	3.2642E-16	0.15833	0.0000	0.0000	3.4343E-5	1.0000
0.0000	2.4000E-6	0.060000	-7.3780E-10	NaN	1.9762E-4	0.0000	1.9576E-17	3.2602E-16	0.15805	0.0000	0.0000	3.4282E-5	1.0000
0.0000	2.4000E-6	0.065000	-7.3651E-10	NaN	1.9731E-4	0.0000	1.6500E-17	3.2567E-16	0.15777	0.0000	0.0000	3.4222E-5	1.0000
0.0000	2.4000E-6	0.070000	-7.3553E-10	NaN	1.9706E-4	0.0000	1.4704E-17	3.2535E-16	0.15756	0.0000	0.0000	3.4176E-5	1.0000
0.0000	2.4000E-6	0.075000	-7.3471E-	NaN	1.9685E-4	0.0000	1.3466E-17	3.2506E-16	0.15739	0.0000	0.0000	3.4138E-5	1.0000

k0Sub (m/s)	d (m)	Time (s)	-(tds2.ntflux_Oxi)*n*F (A), UME	Surface ConcOxi (M), Oxi Mem	Surface ConcOxi (M), OxiNuc	Substrate Kinetics (m/s), k0Sub probe	Normal total flux (mol/s), UME OCR membrane	Normal total flux (mol/s), UME OCR nucleus	Concentration (mol/m^3), Point: (5.5109E-16, 2.5)	k0Sub*Oxi (mol/s), Mem Flux	k0Sub*Oxi (mol/s), Nuc Flux	Normal total flux (mol/(m^2*s)), Point: (5.5109E-16, 2.5)	WElectrode radius (µm), UME Radius
			10										
0.0000	2.4000E-6	0.080000	-7.3393E-10	NaN	1.9665E-4	0.0000	1.2170E-17	3.2480E-16	0.15722	0.0000	0.0000	3.4102E-5	1.0000
0.0000	2.4000E-6	0.085000	-7.3329E-10	NaN	1.9649E-4	0.0000	1.1286E-17	3.2458E-16	0.15708	0.0000	0.0000	3.4072E-5	1.0000
0.0000	2.4000E-6	0.090000	-7.3266E-10	NaN	1.9633E-4	0.0000	1.0356E-17	3.2436E-16	0.15695	0.0000	0.0000	3.4043E-5	1.0000
0.0000	2.4000E-6	0.095000	-7.3206E-10	NaN	1.9618E-4	0.0000	9.4379E-18	3.2416E-16	0.15682	0.0000	0.0000	3.4015E-5	1.0000
0.0000	2.4000E-6	0.10000	-7.3152E-10	NaN	1.9604E-4	0.0000	8.6106E-18	3.2397E-16	0.15670	0.0000	0.0000	3.3989E-5	1.0000
0.0000	2.4000E-6	0.10500	-7.3102E-10	NaN	1.9591E-4	0.0000	7.8736E-18	3.2380E-16	0.15659	0.0000	0.0000	3.3966E-5	1.0000
0.0000	2.4000E-6	0.11000	-7.3057E-10	NaN	1.9580E-4	0.0000	7.2270E-18	3.2365E-16	0.15650	0.0000	0.0000	3.3945E-5	1.0000
0.0000	2.4000E-6	0.11500	-7.3022E-10	NaN	1.9571E-4	0.0000	6.7955E-18	3.2353E-16	0.15642	0.0000	0.0000	3.3929E-5	1.0000
0.0000	2.4000E-6	0.12000	-7.2999E-10	NaN	1.9565E-4	0.0000	6.5759E-18	3.2344E-16	0.15637	0.0000	0.0000	3.3918E-5	1.0000
0.00	2.4000E-	0.12	-7.29	NaN	1.9559E-	0.00	6.3563E-	3.2335E-	0.15	0.00	0.00	3.3908E-	1.00

k0Sub (m/s)	d (m)	Time (s)	-(tds2.ntflux_Oxi)*n*F (A), UME	Surface ConcOxi (M), OxiMem	Surface ConcOxi (M), OxiNuc	Substrate Kinetics (m/s), k0Sub probe	Normal total flux (mol/s), UME OCR membrane	Normal total flux (mol/s), UME OCR nucleus	Concentration (mol/m^3), Point: (5.5109E-16, 2.5)	k0Sub*Oxi (mol/s), MemFlux	k0Sub*Oxi (mol/s), NucFlux	Normal total flux (mol/(m^2*s)), Point: (5.5109E-16, 2.5)	WElectrode radius (µm), UME Radius
00	6	500	76E-10		4	00	18	16	632	00	00	5	00
0.0000	2.4000E-6	0.13000	-7.2953E-10	NaN	1.9553E-4	0.0000	6.1367E-18	3.2327E-16	0.15627	0.0000	0.0000	3.3897E-5	1.0000
0.0000	2.4000E-6	0.13500	-7.2930E-10	NaN	1.9547E-4	0.0000	5.9171E-18	3.2318E-16	0.15623	0.0000	0.0000	3.3886E-5	1.0000
0.0000	2.4000E-6	0.14000	-7.2907E-10	NaN	1.9541E-4	0.0000	5.6975E-18	3.2309E-16	0.15618	0.0000	0.0000	3.3875E-5	1.0000
0.0000	2.4000E-6	0.14500	-7.2884E-10	NaN	1.9535E-4	0.0000	5.4779E-18	3.2301E-16	0.15613	0.0000	0.0000	3.3865E-5	1.0000
0.0000	2.4000E-6	0.15000	-7.2861E-10	NaN	1.9529E-4	0.0000	5.2583E-18	3.2292E-16	0.15608	0.0000	0.0000	3.3854E-5	1.0000
0.0000	2.4000E-6	0.15500	-7.2838E-10	NaN	1.9523E-4	0.0000	5.0387E-18	3.2283E-16	0.15603	0.0000	0.0000	3.3843E-5	1.0000
0.0000	2.4000E-6	0.16000	-7.2815E-10	NaN	1.9517E-4	0.0000	4.8191E-18	3.2275E-16	0.15598	0.0000	0.0000	3.3832E-5	1.0000
0.0000	2.4000E-6	0.16500	-7.2792E-10	NaN	1.9511E-4	0.0000	4.5995E-18	3.2266E-16	0.15593	0.0000	0.0000	3.3822E-5	1.0000
0.0000	2.4000E-6	0.17000	-7.2773E-10	NaN	1.9506E-4	0.0000	4.4328E-18	3.2259E-16	0.15589	0.0000	0.0000	3.3813E-5	1.0000

k0Sub (m/s)	d (m)	Time (s)	-(tds2.ntflux_Oxi)*n*F (A), UME	Surface ConcOxi (M), Oxi Mem	Surface ConcOxi (M), OxiNuc	Substrate Kinetics (m/s), k0Sub probe	Normal total flux (mol/s), UME OCR membrane	Normal total flux (mol/s), UME OCR nucleus	Concentration (mol/m^3), Point: (5.5109E-16, 2.5)	k0Sub*Oxi (mol/s), Mem Flux	k0Sub*Oxi (mol/s), Nuc Flux	Normal total flux (mol/(m^2*s)), Point: (5.5109E-16, 2.5)	WElectrode radius (µm), UME Radius
0.0000	2.4000E-6	0.17500	-7.2757E-10	NaN	1.9502E-4	0.0000	4.3051E-18	3.2253E-16	0.15585	0.0000	0.0000	3.3806E-5	1.0000
0.0000	2.4000E-6	0.18000	-7.2742E-10	NaN	1.9498E-4	0.0000	4.1773E-18	3.2246E-16	0.15582	0.0000	0.0000	3.3798E-5	1.0000
0.0000	2.4000E-6	0.18500	-7.2726E-10	NaN	1.9494E-4	0.0000	4.0495E-18	3.2240E-16	0.15579	0.0000	0.0000	3.3791E-5	1.0000
0.0000	2.4000E-6	0.19000	-7.2710E-10	NaN	1.9490E-4	0.0000	3.9218E-18	3.2234E-16	0.15575	0.0000	0.0000	3.3784E-5	1.0000
0.0000	2.4000E-6	0.19500	-7.2695E-10	NaN	1.9486E-4	0.0000	3.7940E-18	3.2228E-16	0.15572	0.0000	0.0000	3.3777E-5	1.0000
0.0000	2.4000E-6	0.20000	-7.2679E-10	NaN	1.9482E-4	0.0000	3.6663E-18	3.2222E-16	0.15569	0.0000	0.0000	3.3769E-5	1.0000
0.0000	2.4000E-6	0.20500	-7.2664E-10	NaN	1.9478E-4	0.0000	3.5385E-18	3.2216E-16	0.15565	0.0000	0.0000	3.3762E-5	1.0000
0.0000	2.4000E-6	0.21000	-7.2648E-10	NaN	1.9474E-4	0.0000	3.4107E-18	3.2210E-16	0.15562	0.0000	0.0000	3.3755E-5	1.0000
0.0000	2.4000E-6	0.21500	-7.2633E-10	NaN	1.9470E-4	0.0000	3.2830E-18	3.2204E-16	0.15559	0.0000	0.0000	3.3747E-5	1.0000
0.0000	2.4000E-6	0.22000	-7.2617E-	NaN	1.9465E-4	0.0000	3.1552E-18	3.2198E-16	0.15555	0.0000	0.0000	3.3740E-5	1.0000

k0Sub (m/s)	d (m)	Time (s)	-(tds2.ntflux_Oxi)*n*F (A), UME	Surface ConcOxi (M), Oxi Mem	Surface ConcOxi (M), OxiNuc	Substrate Kinetics (m/s), k0Sub probe	Normal total flux (mol/s), UME OCR membrane	Normal total flux (mol/s), UME OCR nucleus	Concentration (mol/m^3), Point: (5.5109E-16, 2.5)	k0Sub*Oxi (mol/s), Mem Flux	k0Sub*Oxi (mol/s), Nuc Flux	Normal total flux (mol/(m^2*s)), Point: (5.5109E-16, 2.5)	WElectrode radius (µm), UME Radius
			10										
0.0000	2.4000E-6	0.22500	-7.2605E-10	NaN	1.9462E-4	0.0000	3.0754E-18	3.2194E-16	0.15553	0.0000	0.0000	3.3735E-5	1.0000
0.0000	2.4000E-6	0.23000	-7.2596E-10	NaN	1.9460E-4	0.0000	3.0132E-18	3.2190E-16	0.15551	0.0000	0.0000	3.3730E-5	1.0000
0.0000	2.4000E-6	0.23500	-7.2586E-10	NaN	1.9457E-4	0.0000	2.9511E-18	3.2186E-16	0.15549	0.0000	0.0000	3.3726E-5	1.0000
0.0000	2.4000E-6	0.24000	-7.2577E-10	NaN	1.9455E-4	0.0000	2.8890E-18	3.2182E-16	0.15547	0.0000	0.0000	3.3722E-5	1.0000
0.0000	2.4000E-6	0.24500	-7.2567E-10	NaN	1.9453E-4	0.0000	2.8269E-18	3.2179E-16	0.15545	0.0000	0.0000	3.3717E-5	1.0000
0.0000	2.4000E-6	0.25000	-7.2558E-10	NaN	1.9450E-4	0.0000	2.7648E-18	3.2175E-16	0.15543	0.0000	0.0000	3.3713E-5	1.0000
0.0000	2.4000E-6	0.25000	-7.2558E-10	NaN	1.9450E-4	0.0000	2.7648E-18	3.2175E-16	0.15543	0.0000	0.0000	3.3713E-5	1.0000
0.0000	2.4000E-6	0.27000	-7.2520E-10	NaN	1.9440E-4	0.0000	2.5163E-18	3.2160E-16	0.15535	0.0000	0.0000	3.3695E-5	1.0000
0.0000	2.4000E-6	0.29000	-7.2482E-10	NaN	1.9430E-4	0.0000	2.2678E-18	3.2145E-16	0.15526	0.0000	0.0000	3.3678E-5	1.0000
0.00	2.4000E-	0.31	-7.24	NaN	1.9420E-	0.00	2.0193E-	3.2130E-	0.15	0.00	0.00	3.3660E-	1.00

k0Sub (m/s)	d (m)	Time (s)	-(tds2.ntflux_Oxi)*n*F (A), UME	Surface ConcOxi (M), Oxi Mem	Surface ConcOxi (M), OxiNuc	Substrate Kinetics (m/s), k0Sub probe	Normal total flux (mol/s), UME OCR membrane	Normal total flux (mol/s), UME OCR nucleus	Concentration (mol/m^3), Point: (5.5109E-16, 2.5)	k0Sub*Oxi (mol/s), Mem Flux	k0Sub*Oxi (mol/s), Nuc Flux	Normal total flux (mol/(m^2*s)), Point: (5.5109E-16, 2.5)	WElectrode radius (µm), UME Radius
00	6	000	45E-10		4	00	18	16	518	00	00	5	00
0.0000	2.4000E-6	0.33000	-7.2413E-10	NaN	1.9412E-4	0.0000	1.8289E-18	3.2117E-16	0.15512	0.0000	0.0000	3.3645E-5	1.0000
0.0000	2.4000E-6	0.35000	-7.2388E-10	NaN	1.9405E-4	0.0000	1.6999E-18	3.2107E-16	0.15506	0.0000	0.0000	3.3634E-5	1.0000
0.0000	2.4000E-6	0.37000	-7.2363E-10	NaN	1.9399E-4	0.0000	1.5709E-18	3.2097E-16	0.15501	0.0000	0.0000	3.3622E-5	1.0000
0.0000	2.4000E-6	0.39000	-7.2338E-10	NaN	1.9392E-4	0.0000	1.4418E-18	3.2086E-16	0.15496	0.0000	0.0000	3.3611E-5	1.0000
0.0000	2.4000E-6	0.41000	-7.2313E-10	NaN	1.9386E-4	0.0000	1.3128E-18	3.2076E-16	0.15490	0.0000	0.0000	3.3599E-5	1.0000
0.0000	2.4000E-6	0.43000	-7.2292E-10	NaN	1.9380E-4	0.0000	1.2141E-18	3.2068E-16	0.15486	0.0000	0.0000	3.3589E-5	1.0000
0.0000	2.4000E-6	0.45000	-7.2275E-10	NaN	1.9376E-4	0.0000	1.1410E-18	3.2060E-16	0.15482	0.0000	0.0000	3.3581E-5	1.0000
0.0000	2.4000E-6	0.47000	-7.2257E-10	NaN	1.9371E-4	0.0000	1.0679E-18	3.2053E-16	0.15478	0.0000	0.0000	3.3573E-5	1.0000
0.0000	2.4000E-6	0.49000	-7.2239E-10	NaN	1.9366E-4	0.0000	9.9475E-19	3.2046E-16	0.15474	0.0000	0.0000	3.3565E-5	1.0000

k0Sub (m/s)	d (m)	Time (s)	-(tds2.ntflux_Oxi)*n*F (A), UME	Surface ConcOxi (M), Oxi Mem	Surface ConcOxi (M), OxiNuc	Substrate Kinetics (m/s), k0Sub probe	Normal total flux (mol/s), UME OCR membrane	Normal total flux (mol/s), UME OCR nucleus	Concentration (mol/m^3), Point: (5.5109E-16, 2.5)	k0Sub*Oxi (mol/s), Mem Flux	k0Sub*Oxi (mol/s), Nuc Flux	Normal total flux (mol/(m^2*s)), Point: (5.5109E-16, 2.5)	WElectrode radius (µm), UME Radius
0.0000	2.4000E-6	0.51000	-7.2222E-10	NaN	1.9362E-4	0.0000	9.2164E-19	3.2038E-16	0.15471	0.0000	0.0000	3.3556E-5	1.0000
0.0000	2.4000E-6	0.53000	-7.2207E-10	NaN	1.9358E-4	0.0000	8.6545E-19	3.2032E-16	0.15467	0.0000	0.0000	3.3549E-5	1.0000
0.0000	2.4000E-6	0.55000	-7.2194E-10	NaN	1.9354E-4	0.0000	8.2057E-19	3.2027E-16	0.15465	0.0000	0.0000	3.3543E-5	1.0000
0.0000	2.4000E-6	0.57000	-7.2180E-10	NaN	1.9351E-4	0.0000	7.7569E-19	3.2021E-16	0.15462	0.0000	0.0000	3.3537E-5	1.0000
0.0000	2.4000E-6	0.59000	-7.2167E-10	NaN	1.9347E-4	0.0000	7.3082E-19	3.2015E-16	0.15459	0.0000	0.0000	3.3531E-5	1.0000
0.0000	2.4000E-6	0.61000	-7.2154E-10	NaN	1.9344E-4	0.0000	6.8594E-19	3.2010E-16	0.15456	0.0000	0.0000	3.3525E-5	1.0000
0.0000	2.4000E-6	0.63000	-7.2142E-10	NaN	1.9341E-4	0.0000	6.5029E-19	3.2005E-16	0.15454	0.0000	0.0000	3.3519E-5	1.0000
0.0000	2.4000E-6	0.65000	-7.2132E-10	NaN	1.9338E-4	0.0000	6.1945E-19	3.2001E-16	0.15451	0.0000	0.0000	3.3515E-5	1.0000
0.0000	2.4000E-6	0.67000	-7.2121E-10	NaN	1.9335E-4	0.0000	5.8861E-19	3.1996E-16	0.15449	0.0000	0.0000	3.3510E-5	1.0000
0.0000	2.4000E-6	0.69000	-7.2111E-	NaN	1.9332E-4	0.0000	5.5777E-19	3.1992E-16	0.15447	0.0000	0.0000	3.3505E-5	1.0000

k0Sub (m/s)	d (m)	Time (s)	-(tds2.ntflux_Oxi)*n*F (A), UME	Surface ConcOxi (M), OxiMem	Surface ConcOxi (M), OxiNuc	Substrate Kinetics (m/s), k0Sub probe	Normal total flux (mol/s), UME OCR membrane	Normal total flux (mol/s), UME OCR nucleus	Concentration (mol/m^3), Point: (5.5109E-16, 2.5)	k0Sub*Oxi (mol/s), MemFlux	k0Sub*Oxi (mol/s), NucFlux	Normal total flux (mol/(m^2*s)), Point: (5.5109E-16, 2.5)	WElectrode radius (µm), UME Radius
			10										
0.0000	2.4000E-6	0.71000	-7.2100E-10	NaN	1.9329E-4	0.0000	5.2693E-19	3.1987E-16	0.15445	0.0000	0.0000	3.3500E-5	1.0000
0.0000	2.4000E-6	0.73000	-7.2091E-10	NaN	1.9327E-4	0.0000	5.0325E-19	3.1983E-16	0.15443	0.0000	0.0000	3.3496E-5	1.0000
0.0000	2.4000E-6	0.75000	-7.2083E-10	NaN	1.9325E-4	0.0000	4.8243E-19	3.1980E-16	0.15441	0.0000	0.0000	3.3492E-5	1.0000
0.0000	2.4000E-6	0.77000	-7.2074E-10	NaN	1.9322E-4	0.0000	4.6161E-19	3.1976E-16	0.15439	0.0000	0.0000	3.3488E-5	1.0000
0.0000	2.4000E-6	0.79000	-7.2066E-10	NaN	1.9320E-4	0.0000	4.4080E-19	3.1972E-16	0.15437	0.0000	0.0000	3.3484E-5	1.0000
0.0000	2.4000E-6	0.81000	-7.2057E-10	NaN	1.9318E-4	0.0000	4.1998E-19	3.1969E-16	0.15435	0.0000	0.0000	3.3480E-5	1.0000
0.0000	2.4000E-6	0.83000	-7.2050E-10	NaN	1.9316E-4	0.0000	4.0169E-19	3.1965E-16	0.15434	0.0000	0.0000	3.3476E-5	1.0000
0.0000	2.4000E-6	0.85000	-7.2042E-10	NaN	1.9314E-4	0.0000	3.8516E-19	3.1962E-16	0.15432	0.0000	0.0000	3.3473E-5	1.0000
0.0000	2.4000E-6	0.87000	-7.2035E-10	NaN	1.9312E-4	0.0000	3.6978E-19	3.1959E-16	0.15431	0.0000	0.0000	3.3469E-5	1.0000
0.00	2.4000E-	0.89	-7.20	NaN	1.9310E-	0.00	3.5555E-	3.1956E-	0.15	0.00	0.00	3.3466E-	1.00

k0Sub (m/s)	d (m)	Time (s)	-(tds2.ntflux_Oxi)*n*F (A), UME	Surface ConcOxi (M), Oxi Mem	Surface ConcOxi (M), OxiNuc	Substrate Kinetics (m/s), k0Sub probe	Normal total flux (mol/s), UME OCR membrane	Normal total flux (mol/s), UME OCR nucleus	Concentration (mol/m^3), Point: (5.5109E-16, 2.5)	k0Sub*Oxi (mol/s), Mem Flux	k0Sub*Oxi (mol/s), Nuc Flux	Normal total flux (mol/(m^2*s)), Point: (5.5109E-16, 2.5)	WEIectrode radius (µm), UME Radius
00	6	000	28E-10		4	00	19	16	429	00	00	5	00
0.0000	2.4000E-6	0.91000	-7.2021E-10	NaN	1.9308E-4	0.0000	3.4246E-19	3.1953E-16	0.15428	0.0000	0.0000	3.3463E-5	1.0000
0.0000	2.4000E-6	0.93000	-7.2015E-10	NaN	1.9307E-4	0.0000	3.2968E-19	3.1951E-16	0.15426	0.0000	0.0000	3.3460E-5	1.0000
0.0000	2.4000E-6	0.95000	-7.2009E-10	NaN	1.9305E-4	0.0000	3.1750E-19	3.1948E-16	0.15425	0.0000	0.0000	3.3457E-5	1.0000
0.0000	2.4000E-6	0.97000	-7.2003E-10	NaN	1.9303E-4	0.0000	3.0609E-19	3.1945E-16	0.15424	0.0000	0.0000	3.3454E-5	1.0000
0.0000	2.4000E-6	0.99000	-7.1997E-10	NaN	1.9302E-4	0.0000	2.9546E-19	3.1943E-16	0.15422	0.0000	0.0000	3.3452E-5	1.0000
0.0000	3.0000E-6	0.0000	-7.1631E-9	NaN	2.2700E-4	0.0000	-6.7830E-29	-2.2405E-30	0.22700	0.0000	0.0000	0.0000	1.0000
0.0000	3.0000E-6	0.0050000	-8.4775E-10	NaN	2.1955E-4	0.0000	5.5543E-16	2.2942E-16	0.19384	0.0000	0.0000	2.4976E-5	1.0000
0.0000	3.0000E-6	0.010000	-8.1284E-10	NaN	2.1405E-4	0.0000	3.3482E-16	2.5466E-16	0.18559	0.0000	0.0000	2.3927E-5	1.0000
0.0000	3.0000E-6	0.015000	-7.9497E-10	NaN	2.1050E-4	0.0000	2.1783E-16	2.5861E-16	0.18144	0.0000	0.0000	2.3385E-5	1.0000

k0Sub (m/s)	d (m)	Time (s)	-(tds2.ntflux_Oxi)*n*F (A), UME	Surface ConcOxi (M), Oxi Mem	Surface ConcOxi (M), OxiNuc	Substrate Kinetics (m/s), k0Sub probe	Normal total flux (mol/s), UME OCR membrane	Normal total flux (mol/s), UME OCR nucleus	Concentration (mol/m^3), Point: (5.5109E-16, 2.5)	k0Sub*Oxi (mol/s), Mem Flux	k0Sub*Oxi (mol/s), Nuc Flux	Normal total flux (mol/(m^2*s)), Point: (5.5109E-16, 2.5)	WElectrode radius (µm), UME Radius
0.0000	3.0000E-6	0.020000	-7.8304E-10	NaN	2.0803E-4	0.0000	1.5172E-16	2.6045E-16	0.17867	0.0000	0.0000	2.3030E-5	1.0000
0.0000	3.0000E-6	0.025000	-7.7463E-10	NaN	2.0621E-4	0.0000	1.1026E-16	2.6105E-16	0.17673	0.0000	0.0000	2.2781E-5	1.0000
0.0000	3.0000E-6	0.030000	-7.6826E-10	NaN	2.0480E-4	0.0000	8.0889E-17	2.6131E-16	0.17526	0.0000	0.0000	2.2591E-5	1.0000
0.0000	3.0000E-6	0.035000	-7.6345E-10	NaN	2.0372E-4	0.0000	6.0804E-17	2.6128E-16	0.17415	0.0000	0.0000	2.2448E-5	1.0000
0.0000	3.0000E-6	0.040000	-7.6001E-10	NaN	2.0292E-4	0.0000	4.8523E-17	2.6102E-16	0.17336	0.0000	0.0000	2.2347E-5	1.0000
0.0000	3.0000E-6	0.045000	-7.5721E-10	NaN	2.0226E-4	0.0000	3.9254E-17	2.6075E-16	0.17271	0.0000	0.0000	2.2264E-5	1.0000
0.0000	3.0000E-6	0.050000	-7.5511E-10	NaN	2.0175E-4	0.0000	3.3326E-17	2.6045E-16	0.17223	0.0000	0.0000	2.2201E-5	1.0000
0.0000	3.0000E-6	0.055000	-7.5310E-10	NaN	2.0127E-4	0.0000	2.7352E-17	2.6019E-16	0.17177	0.0000	0.0000	2.2142E-5	1.0000
0.0000	3.0000E-6	0.060000	-7.5139E-10	NaN	2.0085E-4	0.0000	2.2530E-17	2.5996E-16	0.17137	0.0000	0.0000	2.2091E-5	1.0000
0.0000	3.0000E-6	0.065000	-7.4998E-	NaN	2.0051E-4	0.0000	1.8861E-17	2.5975E-16	0.17105	0.0000	0.0000	2.2049E-5	1.0000

k0Sub (m/s)	d (m)	Time (s)	-(tds2.ntflux_Oxi)*n*F (A), UME	Surface Con cOxi (M), Oxi Mem	Surface Con cOxi (M), OxiNuc	Substrate Kinetics (m/s), k0Sub probe	Normal total flux (mol/s), UME OCR membrane	Normal total flux (mol/s), UME OCR nucleus	Concentration (mol/m^3), Point: (5.5109E-16, 2.5)	k0Sub*Oxi (mol/s), Mem Flux	k0Sub*Oxi (mol/s), Nuc Flux	Normal total flux (mol/(m^2*s)), Point: (5.5109E-16, 2.5)	WElectrode radius (µm), UME Radius
			10										
0.0000	3.0000E-6	0.070000	-7.4896E-10	NaN	2.0026E-4	0.0000	1.6622E-17	2.5956E-16	0.17082	0.0000	0.0000	2.2019E-5	1.0000
0.0000	3.0000E-6	0.075000	-7.4828E-10	NaN	2.0009E-4	0.0000	1.5577E-17	2.5939E-16	0.17066	0.0000	0.0000	2.1999E-5	1.0000
0.0000	3.0000E-6	0.080000	-7.4760E-10	NaN	1.9992E-4	0.0000	1.4533E-17	2.5923E-16	0.17051	0.0000	0.0000	2.1979E-5	1.0000
0.0000	3.0000E-6	0.085000	-7.4693E-10	NaN	1.9974E-4	0.0000	1.3489E-17	2.5907E-16	0.17035	0.0000	0.0000	2.1959E-5	1.0000
0.0000	3.0000E-6	0.090000	-7.4638E-10	NaN	1.9961E-4	0.0000	1.2670E-17	2.5894E-16	0.17023	0.0000	0.0000	2.1943E-5	1.0000
0.0000	3.0000E-6	0.095000	-7.4595E-10	NaN	1.9949E-4	0.0000	1.2060E-17	2.5883E-16	0.17013	0.0000	0.0000	2.1930E-5	1.0000
0.0000	3.0000E-6	0.10000	-7.4551E-10	NaN	1.9938E-4	0.0000	1.1450E-17	2.5872E-16	0.17003	0.0000	0.0000	2.1918E-5	1.0000
0.0000	3.0000E-6	0.10500	-7.4508E-10	NaN	1.9927E-4	0.0000	1.0840E-17	2.5861E-16	0.16993	0.0000	0.0000	2.1905E-5	1.0000
0.0000	3.0000E-6	0.11000	-7.4465E-10	NaN	1.9916E-4	0.0000	1.0230E-17	2.5851E-16	0.16983	0.0000	0.0000	2.1892E-5	1.0000
0.00	3.0000E-	0.11	-7.44	NaN	1.9905E-	0.00	9.6197E-	2.5840E-	0.16	0.00	0.00	2.1879E-	1.00

k0Sub (m/s)	d (m)	Time (s)	-(tds2.ntflux_Oxi)*n*F (A), UME	Surface ConcOxi (M), Oxi Mem	Surface ConcOxi (M), OxiNuc	Substrate Kinetics (m/s), k0Sub probe	Normal total flux (mol/s), UME OCR membrane	Normal total flux (mol/s), UME OCR nucleus	Concentration (mol/m^3), Point: (5.5109E-16, 2.5)	k0Sub*Oxi (mol/s), Mem Flux	k0Sub*Oxi (mol/s), Nuc Flux	Normal total flux (mol/(m^2*s)), Point: (5.5109E-16, 2.5)	WElectrode radius (μm), UME Radius
00	6	500	21E-10		4	00	18	16	973	00	00	5	00
0.0000	3.0000E-6	0.12000	-7.4378E-10	NaN	1.9894E-4	0.0000	9.0097E-18	2.5829E-16	0.16963	0.0000	0.0000	2.1866E-5	1.0000
0.0000	3.0000E-6	0.12500	-7.4337E-10	NaN	1.9884E-4	0.0000	8.4211E-18	2.5819E-16	0.16954	0.0000	0.0000	2.1854E-5	1.0000
0.0000	3.0000E-6	0.13000	-7.4313E-10	NaN	1.9878E-4	0.0000	8.1574E-18	2.5813E-16	0.16948	0.0000	0.0000	2.1847E-5	1.0000
0.0000	3.0000E-6	0.13500	-7.4290E-10	NaN	1.9872E-4	0.0000	7.8938E-18	2.5806E-16	0.16943	0.0000	0.0000	2.1840E-5	1.0000
0.0000	3.0000E-6	0.14000	-7.4267E-10	NaN	1.9866E-4	0.0000	7.6301E-18	2.5800E-16	0.16938	0.0000	0.0000	2.1834E-5	1.0000
0.0000	3.0000E-6	0.14500	-7.4244E-10	NaN	1.9860E-4	0.0000	7.3665E-18	2.5794E-16	0.16932	0.0000	0.0000	2.1827E-5	1.0000
0.0000	3.0000E-6	0.15000	-7.4220E-10	NaN	1.9854E-4	0.0000	7.1028E-18	2.5788E-16	0.16927	0.0000	0.0000	2.1820E-5	1.0000
0.0000	3.0000E-6	0.15500	-7.4197E-10	NaN	1.9848E-4	0.0000	6.8392E-18	2.5782E-16	0.16922	0.0000	0.0000	2.1813E-5	1.0000
0.0000	3.0000E-6	0.16000	-7.4174E-10	NaN	1.9842E-4	0.0000	6.5755E-18	2.5775E-16	0.16917	0.0000	0.0000	2.1806E-5	1.0000

k0Sub (m/s)	d (m)	Time (s)	-(tds2.ntflux_Oxi)*n*F (A), UME	Surface ConcOxi (M), OxiMem	Surface ConcOxi (M), OxiNuc	Substrate Kinetics (m/s), k0Sub probe	Normal total flux (mol/s), UME OCR membrane	Normal total flux (mol/s), UME OCR nucleus	Concentration (mol/m^3), Point: (5.5109E-16, 2.5)	k0Sub* Oxi (mol/s), Mem Flux	k0Sub* Oxi (mol/s), Nuc Flux	Normal total flux (mol/(m^2*s)), Point: (5.5109E-16, 2.5)	WElectrode radius (µm), UME Radius
0.0000	3.0000E-6	0.16500	-7.4151E-10	NaN	1.9836E-4	0.0000	6.3119E-18	2.5769E-16	0.16911	0.0000	0.0000	2.1799E-5	1.0000
0.0000	3.0000E-6	0.17000	-7.4128E-10	NaN	1.9830E-4	0.0000	6.0482E-18	2.5763E-16	0.16906	0.0000	0.0000	2.1793E-5	1.0000
0.0000	3.0000E-6	0.17500	-7.4104E-10	NaN	1.9824E-4	0.0000	5.7846E-18	2.5757E-16	0.16901	0.0000	0.0000	2.1786E-5	1.0000
0.0000	3.0000E-6	0.18000	-7.4081E-10	NaN	1.9818E-4	0.0000	5.5209E-18	2.5751E-16	0.16895	0.0000	0.0000	2.1779E-5	1.0000
0.0000	3.0000E-6	0.18500	-7.4058E-10	NaN	1.9812E-4	0.0000	5.2573E-18	2.5745E-16	0.16890	0.0000	0.0000	2.1772E-5	1.0000
0.0000	3.0000E-6	0.19000	-7.4035E-10	NaN	1.9806E-4	0.0000	4.9937E-18	2.5738E-16	0.16885	0.0000	0.0000	2.1765E-5	1.0000
0.0000	3.0000E-6	0.19500	-7.4012E-10	NaN	1.9800E-4	0.0000	4.7300E-18	2.5732E-16	0.16879	0.0000	0.0000	2.1758E-5	1.0000
0.0000	3.0000E-6	0.20000	-7.3990E-10	NaN	1.9795E-4	0.0000	4.4874E-18	2.5726E-16	0.16874	0.0000	0.0000	2.1752E-5	1.0000
0.0000	3.0000E-6	0.20500	-7.3976E-10	NaN	1.9791E-4	0.0000	4.3674E-18	2.5722E-16	0.16871	0.0000	0.0000	2.1748E-5	1.0000
0.0000	3.0000E-6	0.21000	-7.3963E-	NaN	1.9787E-4	0.0000	4.2474E-18	2.5718E-16	0.16868	0.0000	0.0000	2.1744E-5	1.0000

k0Sub (m/s)	d (m)	Time (s)	-(tds2.ntflux_Oxi)*n*F (A), UME	Surface ConcOxi (M), OxiMem	Surface ConcOxi (M), OxiNuc	Substrate Kinetics (m/s), k0Sub probe	Normal total flux (mol/s), UME OCR membrane	Normal total flux (mol/s), UME OCR nucleus	Concentration (mol/m^3), Point: (5.5109E-16, 2.5)	k0Sub*Oxi (mol/s), Mem Flux	k0Sub*Oxi (mol/s), Nuc Flux	Normal total flux (mol/(m^2*s)), Point: (5.5109E-16, 2.5)	WElectrode radius (µm), UME Radius
			10										
0.0000	3.0000E-6	0.21500	-7.3949E-10	NaN	1.9784E-4	0.0000	4.1273E-18	2.5715E-16	0.16865	0.0000	0.0000	2.1740E-5	1.0000
0.0000	3.0000E-6	0.22000	-7.3935E-10	NaN	1.9780E-4	0.0000	4.0073E-18	2.5711E-16	0.16862	0.0000	0.0000	2.1736E-5	1.0000
0.0000	3.0000E-6	0.22500	-7.3921E-10	NaN	1.9777E-4	0.0000	3.8872E-18	2.5707E-16	0.16859	0.0000	0.0000	2.1732E-5	1.0000
0.0000	3.0000E-6	0.23000	-7.3907E-10	NaN	1.9773E-4	0.0000	3.7672E-18	2.5703E-16	0.16856	0.0000	0.0000	2.1728E-5	1.0000
0.0000	3.0000E-6	0.23500	-7.3894E-10	NaN	1.9770E-4	0.0000	3.6471E-18	2.5699E-16	0.16852	0.0000	0.0000	2.1724E-5	1.0000
0.0000	3.0000E-6	0.24000	-7.3880E-10	NaN	1.9766E-4	0.0000	3.5271E-18	2.5695E-16	0.16849	0.0000	0.0000	2.1719E-5	1.0000
0.0000	3.0000E-6	0.24500	-7.3866E-10	NaN	1.9762E-4	0.0000	3.4071E-18	2.5691E-16	0.16846	0.0000	0.0000	2.1715E-5	1.0000
0.0000	3.0000E-6	0.25000	-7.3852E-10	NaN	1.9759E-4	0.0000	3.2870E-18	2.5687E-16	0.16843	0.0000	0.0000	2.1711E-5	1.0000
0.0000	3.0000E-6	0.25000	-7.3852E-10	NaN	1.9759E-4	0.0000	3.2870E-18	2.5687E-16	0.16843	0.0000	0.0000	2.1711E-5	1.0000
0.00	3.0000E-	0.27	-7.37	NaN	1.9744E-	0.00	2.8068E-	2.5671E-	0.16	0.00	0.00	2.1695E-	1.00

k0Sub (m/s)	d (m)	Time (s)	-(tds2.ntflux_Oxi)*n*F (A), UME	Surface ConcOxi (M), OxiMem	Surface ConcOxi (M), OxiNuc	Substrate Kinetics (m/s), k0Sub probe	Normal total flux (mol/s), UME OCR membrane	Normal total flux (mol/s), UME OCR nucleus	Concentration (mol/m^3), Point: (5.5109E-16, 2.5)	k0Sub*Oxi (mol/s), Mem Flux	k0Sub*Oxi (mol/s), Nuc Flux	Normal total flux (mol/(m^2*s)), Point: (5.5109E-16, 2.5)	WEIectrode radius (μm), UME Radius
00	6	000	97E-10		4	00	18	16	830	00	00	5	00
0.0000	3.0000E-6	0.29000	-7.3760E-10	NaN	1.9735E-4	0.0000	2.5411E-18	2.5660E-16	0.16822	0.0000	0.0000	2.1684E-5	1.0000
0.0000	3.0000E-6	0.31000	-7.3726E-10	NaN	1.9726E-4	0.0000	2.3267E-18	2.5650E-16	0.16814	0.0000	0.0000	2.1674E-5	1.0000
0.0000	3.0000E-6	0.33000	-7.3693E-10	NaN	1.9717E-4	0.0000	2.1124E-18	2.5640E-16	0.16807	0.0000	0.0000	2.1665E-5	1.0000
0.0000	3.0000E-6	0.35000	-7.3660E-10	NaN	1.9709E-4	0.0000	1.8981E-18	2.5630E-16	0.16799	0.0000	0.0000	2.1655E-5	1.0000
0.0000	3.0000E-6	0.37000	-7.3626E-10	NaN	1.9700E-4	0.0000	1.6838E-18	2.5620E-16	0.16791	0.0000	0.0000	2.1645E-5	1.0000
0.0000	3.0000E-6	0.39000	-7.3603E-10	NaN	1.9694E-4	0.0000	1.5595E-18	2.5612E-16	0.16786	0.0000	0.0000	2.1638E-5	1.0000
0.0000	3.0000E-6	0.41000	-7.3581E-10	NaN	1.9688E-4	0.0000	1.4509E-18	2.5605E-16	0.16781	0.0000	0.0000	2.1631E-5	1.0000
0.0000	3.0000E-6	0.43000	-7.3558E-10	NaN	1.9682E-4	0.0000	1.3422E-18	2.5598E-16	0.16776	0.0000	0.0000	2.1625E-5	1.0000
0.0000	3.0000E-6	0.45000	-7.3536E-10	NaN	1.9676E-4	0.0000	1.2335E-18	2.5591E-16	0.16771	0.0000	0.0000	2.1618E-5	1.0000

k0Sub (m/s)	d (m)	Time (s)	-(tds2.ntflux_Oxi)*n*F (A), UME	Surface ConcOxi (M), OxiMem	Surface ConcOxi (M), OxiNuc	Substrate Kinetics (m/s), k0Sub probe	Normal total flux (mol/s), UME OCR membrane	Normal total flux (mol/s), UME OCR nucleus	Concentration (mol/m^3), Point: (5.5109E-16, 2.5)	k0Sub*Oxi (mol/s), Mem Flux	k0Sub*Oxi (mol/s), Nuc Flux	Normal total flux (mol/(m^2*s)), Point: (5.5109E-16, 2.5)	WElectrode radius (µm), UME Radius
0.0000	3.0000E-6	0.47000	-7.3514E-10	NaN	1.9670E-4	0.0000	1.1249E-18	2.5584E-16	0.16766	0.0000	0.0000	2.1612E-5	1.0000
0.0000	3.0000E-6	0.49000	-7.3497E-10	NaN	1.9666E-4	0.0000	1.0582E-18	2.5579E-16	0.16762	0.0000	0.0000	2.1607E-5	1.0000
0.0000	3.0000E-6	0.51000	-7.3481E-10	NaN	1.9662E-4	0.0000	9.9630E-19	2.5574E-16	0.16758	0.0000	0.0000	2.1602E-5	1.0000
0.0000	3.0000E-6	0.53000	-7.3465E-10	NaN	1.9657E-4	0.0000	9.3439E-19	2.5569E-16	0.16755	0.0000	0.0000	2.1597E-5	1.0000
0.0000	3.0000E-6	0.55000	-7.3449E-10	NaN	1.9653E-4	0.0000	8.7249E-19	2.5564E-16	0.16751	0.0000	0.0000	2.1593E-5	1.0000
0.0000	3.0000E-6	0.57000	-7.3433E-10	NaN	1.9649E-4	0.0000	8.1059E-19	2.5559E-16	0.16747	0.0000	0.0000	2.1588E-5	1.0000
0.0000	3.0000E-6	0.59000	-7.3421E-10	NaN	1.9646E-4	0.0000	7.6868E-19	2.5554E-16	0.16744	0.0000	0.0000	2.1584E-5	1.0000
0.0000	3.0000E-6	0.61000	-7.3408E-10	NaN	1.9642E-4	0.0000	7.2800E-19	2.5550E-16	0.16742	0.0000	0.0000	2.1581E-5	1.0000
0.0000	3.0000E-6	0.63000	-7.3395E-10	NaN	1.9639E-4	0.0000	6.8732E-19	2.5546E-16	0.16739	0.0000	0.0000	2.1577E-5	1.0000
0.0000	3.0000E-6	0.65000	-7.3383E-	NaN	1.9636E-4	0.0000	6.4664E-19	2.5542E-16	0.16736	0.0000	0.0000	2.1573E-5	1.0000

k0Sub (m/s)	d (m)	Time (s)	-(tds2.ntflux_Oxi)*n*F (A), UME	Surface ConcOxi (M), OxiMem	Surface ConcOxi (M), OxiNuc	Substrate Kinetics (m/s), k0Sub probe	Normal total flux (mol/s), UME OCR membrane	Normal total flux (mol/s), UME OCR nucleus	Concentration (mol/m^3), Point: (5.5109E-16, 2.5)	k0Sub*Oxi (mol/s), Mem Flux	k0Sub*Oxi (mol/s), Nuc Flux	Normal total flux (mol/(m^2*s)), Point: (5.5109E-16, 2.5)	WEIectrode radius (µm), UME Radius
			10										
0.0000	3.0000E-6	0.67000	-7.3370E-10	NaN	1.9632E-4	0.0000	6.0579E-19	2.5538E-16	0.16733	0.0000	0.0000	2.1570E-5	1.0000
0.0000	3.0000E-6	0.69000	-7.3361E-10	NaN	1.9630E-4	0.0000	5.7956E-19	2.5535E-16	0.16731	0.0000	0.0000	2.1567E-5	1.0000
0.0000	3.0000E-6	0.71000	-7.3351E-10	NaN	1.9627E-4	0.0000	5.5333E-19	2.5532E-16	0.16728	0.0000	0.0000	2.1564E-5	1.0000
0.0000	3.0000E-6	0.73000	-7.3341E-10	NaN	1.9625E-4	0.0000	5.2710E-19	2.5528E-16	0.16726	0.0000	0.0000	2.1561E-5	1.0000
0.0000	3.0000E-6	0.75000	-7.3331E-10	NaN	1.9622E-4	0.0000	5.0087E-19	2.5525E-16	0.16724	0.0000	0.0000	2.1558E-5	1.0000
0.0000	3.0000E-6	0.77000	-7.3322E-10	NaN	1.9619E-4	0.0000	4.7477E-19	2.5522E-16	0.16722	0.0000	0.0000	2.1555E-5	1.0000
0.0000	3.0000E-6	0.79000	-7.3313E-10	NaN	1.9617E-4	0.0000	4.5332E-19	2.5519E-16	0.16720	0.0000	0.0000	2.1553E-5	1.0000
0.0000	3.0000E-6	0.81000	-7.3304E-10	NaN	1.9615E-4	0.0000	4.3346E-19	2.5516E-16	0.16718	0.0000	0.0000	2.1550E-5	1.0000
0.0000	3.0000E-6	0.83000	-7.3296E-10	NaN	1.9613E-4	0.0000	4.1518E-19	2.5514E-16	0.16716	0.0000	0.0000	2.1548E-5	1.0000
0.00	3.0000E-	0.85	-7.32	NaN	1.9611E-	0.00	3.9850E-	2.5511E-	0.16	0.00	0.00	2.1545E-	1.00

k0Sub (m/s)	d (m)	Time (s)	-(tds2.ntflux_Oxi)*n*F (A), UME	Surface ConcOxi (M), OxiMem	Surface ConcOxi (M), OxiNuc	Substrate Kinetics (m/s), k0Sub probe	Normal total flux (mol/s), UME OCR membrane	Normal total flux (mol/s), UME OCR nucleus	Concentration (mol/m^3), Point: (5.5109E-16, 2.5)	k0Sub*Oxi (mol/s), Mem Flux	k0Sub*Oxi (mol/s), Nuc Flux	Normal total flux (mol/(m^2*s)), Point: (5.5109E-16, 2.5)	WEIectrode radius (µm), UME Radius
00	6	000	89E-10		4	00	19	16	714	00	00	5	00
0.0000	3.0000E-6	0.87000	-7.3281E-10	NaN	1.9609E-4	0.0000	3.8325E-19	2.5509E-16	0.16713	0.0000	0.0000	2.1543E-5	1.0000
0.0000	3.0000E-6	0.89000	-7.3274E-10	NaN	1.9607E-4	0.0000	3.6794E-19	2.5506E-16	0.16711	0.0000	0.0000	2.1541E-5	1.0000
0.0000	3.0000E-6	0.91000	-7.3267E-10	NaN	1.9605E-4	0.0000	3.5362E-19	2.5504E-16	0.16709	0.0000	0.0000	2.1539E-5	1.0000
0.0000	3.0000E-6	0.93000	-7.3260E-10	NaN	1.9603E-4	0.0000	3.4030E-19	2.5501E-16	0.16708	0.0000	0.0000	2.1537E-5	1.0000
0.0000	3.0000E-6	0.95000	-7.3253E-10	NaN	1.9601E-4	0.0000	3.2798E-19	2.5499E-16	0.16706	0.0000	0.0000	2.1535E-5	1.0000
0.0000	3.0000E-6	0.97000	-7.3247E-10	NaN	1.9600E-4	0.0000	3.1655E-19	2.5497E-16	0.16705	0.0000	0.0000	2.1533E-5	1.0000
0.0000	3.0000E-6	0.99000	-7.3241E-10	NaN	1.9598E-4	0.0000	3.0525E-19	2.5495E-16	0.16703	0.0000	0.0000	2.1531E-5	1.0000
0.0000	4.0000E-6	0.0000	-7.1535E-9	NaN	2.2700E-4	0.0000	-1.4101E-29	-9.2201E-30	0.22700	0.0000	0.0000	2.5611E-19	1.0000
0.0000	4.0000E-6	0.0050000	-8.5359E-10	NaN	2.2214E-4	0.0000	4.4664E-16	1.4254E-16	0.20750	0.0000	0.0000	1.3336E-5	1.0000

k0Sub (m/s)	d (m)	Time (s)	-(tds2.ntflux_Oxi)*n*F (A), UME	Surface ConcOxi (M), OxiMem	Surface ConcOxi (M), OxiNuc	Substrate Kinetics (m/s), k0Sub probe	Normal total flux (mol/s), UME OCR membrane	Normal total flux (mol/s), UME OCR nucleus	Concentration (mol/m^3), Point: (5.5109E-16, 2.5)	k0Sub*Oxi (mol/s), Mem Flux	k0Sub*Oxi (mol/s), Nuc Flux	Normal total flux (mol/(m^2*s)), Point: (5.5109E-16, 2.5)	WElectrode radius (µm), UME Radius
0.0000	4.0000E-6	0.010000	-8.2275E-10	NaN	2.1746E-4	0.0000	2.9633E-16	1.7068E-16	0.19988	0.0000	0.0000	1.2986E-5	1.0000
0.0000	4.0000E-6	0.015000	-8.0848E-10	NaN	2.1454E-4	0.0000	2.0678E-16	1.7517E-16	0.19631	0.0000	0.0000	1.2743E-5	1.0000
0.0000	4.0000E-6	0.020000	-7.9936E-10	NaN	2.1258E-4	0.0000	1.5800E-16	1.7708E-16	0.19404	0.0000	0.0000	1.2593E-5	1.0000
0.0000	4.0000E-6	0.025000	-7.9220E-10	NaN	2.1102E-4	0.0000	1.2313E-16	1.7841E-16	0.19227	0.0000	0.0000	1.2479E-5	1.0000
0.0000	4.0000E-6	0.030000	-7.8691E-10	NaN	2.0984E-4	0.0000	9.9800E-17	1.7918E-16	0.19097	0.0000	0.0000	1.2395E-5	1.0000
0.0000	4.0000E-6	0.035000	-7.8288E-10	NaN	2.0893E-4	0.0000	8.3244E-17	1.7964E-16	0.18997	0.0000	0.0000	1.2331E-5	1.0000
0.0000	4.0000E-6	0.040000	-7.7968E-10	NaN	2.0819E-4	0.0000	7.0967E-17	1.7990E-16	0.18918	0.0000	0.0000	1.2280E-5	1.0000
0.0000	4.0000E-6	0.045000	-7.7647E-10	NaN	2.0746E-4	0.0000	5.8690E-17	1.8017E-16	0.18839	0.0000	0.0000	1.2229E-5	1.0000
0.0000	4.0000E-6	0.050000	-7.7422E-10	NaN	2.0693E-4	0.0000	5.1007E-17	1.8026E-16	0.18784	0.0000	0.0000	1.2193E-5	1.0000
0.0000	4.0000E-6	0.055000	-7.7213E-	NaN	2.0644E-4	0.0000	4.4074E-17	1.8033E-16	0.18733	0.0000	0.0000	1.2160E-5	1.0000

k0Sub (m/s)	d (m)	Time (s)	-(tds2.ntflux_Oxi)*n*F (A), UME	Surface ConcOxi (M), Oxi Mem	Surface ConcOxi (M), OxiNuc	Substrate Kinetics (m/s), k0Sub probe	Normal total flux (mol/s), UME OCR membrane	Normal total flux (mol/s), UME OCR nucleus	Concentration (mol/m^3), Point: (5.5109E-16, 2.5)	k0Sub*Oxi (mol/s), Mem Flux	k0Sub*Oxi (mol/s), Nuc Flux	Normal total flux (mol/(m^2*s)), Point: (5.5109E-16, 2.5)	WElectrode radius (µm), UME Radius
			10										
0.0000	4.0000E-6	0.060000	-7.7015E-10	NaN	2.0597E-4	0.0000	3.7612E-17	1.8038E-16	0.18684	0.0000	0.0000	1.2129E-5	1.0000
0.0000	4.0000E-6	0.065000	-7.6901E-10	NaN	2.0569E-4	0.0000	3.4533E-17	1.8035E-16	0.18656	0.0000	0.0000	1.2111E-5	1.0000
0.0000	4.0000E-6	0.070000	-7.6787E-10	NaN	2.0542E-4	0.0000	3.1454E-17	1.8032E-16	0.18628	0.0000	0.0000	1.2093E-5	1.0000
0.0000	4.0000E-6	0.075000	-7.6673E-10	NaN	2.0514E-4	0.0000	2.8376E-17	1.8029E-16	0.18600	0.0000	0.0000	1.2074E-5	1.0000
0.0000	4.0000E-6	0.080000	-7.6559E-10	NaN	2.0486E-4	0.0000	2.5297E-17	1.8026E-16	0.18572	0.0000	0.0000	1.2056E-5	1.0000
0.0000	4.0000E-6	0.085000	-7.6445E-10	NaN	2.0459E-4	0.0000	2.2219E-17	1.8023E-16	0.18545	0.0000	0.0000	1.2038E-5	1.0000
0.0000	4.0000E-6	0.090000	-7.6359E-10	NaN	2.0438E-4	0.0000	2.0168E-17	1.8018E-16	0.18524	0.0000	0.0000	1.2025E-5	1.0000
0.0000	4.0000E-6	0.095000	-7.6291E-10	NaN	2.0421E-4	0.0000	1.8685E-17	1.8013E-16	0.18507	0.0000	0.0000	1.2014E-5	1.0000
0.0000	4.0000E-6	0.10000	-7.6222E-10	NaN	2.0404E-4	0.0000	1.7202E-17	1.8008E-16	0.18490	0.0000	0.0000	1.2003E-5	1.0000
0.00	4.0000E-	0.10	-7.61	NaN	2.0387E-	0.00	1.5719E-	1.8003E-	0.18	0.00	0.00	1.1992E-	1.00

k0Sub (m/s)	d (m)	Time (s)	-(tds2.ntflux_Oxi)*n*F (A), UME	Surface ConcOxi (M), OxiMem	Surface ConcOxi (M), OxiNuc	Substrate Kinetics (m/s), k0Sub probe	Normal total flux (mol/s), UME OCR membrane	Normal total flux (mol/s), UME OCR nucleus	Concentration (mol/m^3), Point: (5.5109E-16, 2.5)	k0Sub*Oxi (mol/s), Mem Flux	k0Sub*Oxi (mol/s), Nuc Flux	Normal total flux (mol/(m^2*s)), Point: (5.5109E-16, 2.5)	WElectrode radius (µm), UME Radius
00	6	500	54E-10		4	00	17	16	473	00	00	5	00
0.0000	4.0000E-6	0.11000	-7.6086E-10	NaN	2.0370E-4	0.0000	1.4236E-17	1.7998E-16	0.18457	0.0000	0.0000	1.1981E-5	1.0000
0.0000	4.0000E-6	0.11500	-7.6022E-10	NaN	2.0354E-4	0.0000	1.2908E-17	1.7993E-16	0.18441	0.0000	0.0000	1.1971E-5	1.0000
0.0000	4.0000E-6	0.12000	-7.5986E-10	NaN	2.0345E-4	0.0000	1.2342E-17	1.7989E-16	0.18432	0.0000	0.0000	1.1966E-5	1.0000
0.0000	4.0000E-6	0.12500	-7.5951E-10	NaN	2.0336E-4	0.0000	1.1776E-17	1.7985E-16	0.18424	0.0000	0.0000	1.1960E-5	1.0000
0.0000	4.0000E-6	0.13000	-7.5915E-10	NaN	2.0327E-4	0.0000	1.1210E-17	1.7980E-16	0.18415	0.0000	0.0000	1.1954E-5	1.0000
0.0000	4.0000E-6	0.13500	-7.5880E-10	NaN	2.0318E-4	0.0000	1.0645E-17	1.7976E-16	0.18406	0.0000	0.0000	1.1949E-5	1.0000
0.0000	4.0000E-6	0.14000	-7.5844E-10	NaN	2.0309E-4	0.0000	1.0079E-17	1.7972E-16	0.18398	0.0000	0.0000	1.1943E-5	1.0000
0.0000	4.0000E-6	0.14500	-7.5809E-10	NaN	2.0300E-4	0.0000	9.5127E-18	1.7967E-16	0.18389	0.0000	0.0000	1.1937E-5	1.0000
0.0000	4.0000E-6	0.15000	-7.5773E-10	NaN	2.0291E-4	0.0000	8.9467E-18	1.7963E-16	0.18380	0.0000	0.0000	1.1932E-5	1.0000

k0Sub (m/s)	d (m)	Time (s)	-(tds2.ntflux_Oxi)*n*F (A), UME	Surface ConcOxi (M), Oxi Mem	Surface ConcOxi (M), OxiNuc	Substrate Kinetics (m/s), k0Sub probe	Normal total flux (mol/s), UME OCR membrane	Normal total flux (mol/s), UME OCR nucleus	Concentration (mol/m^3), Point: (5.5109E-16, 2.5)	k0Sub*Oxi (mol/s), Mem Flux	k0Sub*Oxi (mol/s), Nuc Flux	Normal total flux (mol/(m^2*s)), Point: (5.5109E-16, 2.5)	WElectrode radius (μm), UME Radius
0.0000	4.0000E-6	0.15500	-7.5738E-10	NaN	2.0282E-4	0.0000	8.3808E-18	1.7959E-16	0.18372	0.0000	0.0000	1.1926E-5	1.0000
0.0000	4.0000E-6	0.16000	-7.5702E-10	NaN	2.0273E-4	0.0000	7.8148E-18	1.7955E-16	0.18363	0.0000	0.0000	1.1921E-5	1.0000
0.0000	4.0000E-6	0.16500	-7.5667E-10	NaN	2.0264E-4	0.0000	7.2489E-18	1.7950E-16	0.18355	0.0000	0.0000	1.1915E-5	1.0000
0.0000	4.0000E-6	0.17000	-7.5634E-10	NaN	2.0256E-4	0.0000	6.7520E-18	1.7946E-16	0.18346	0.0000	0.0000	1.1910E-5	1.0000
0.0000	4.0000E-6	0.17500	-7.5613E-10	NaN	2.0250E-4	0.0000	6.5064E-18	1.7943E-16	0.18341	0.0000	0.0000	1.1906E-5	1.0000
0.0000	4.0000E-6	0.18000	-7.5592E-10	NaN	2.0245E-4	0.0000	6.2608E-18	1.7940E-16	0.18336	0.0000	0.0000	1.1903E-5	1.0000
0.0000	4.0000E-6	0.18500	-7.5571E-10	NaN	2.0239E-4	0.0000	6.0152E-18	1.7936E-16	0.18331	0.0000	0.0000	1.1900E-5	1.0000
0.0000	4.0000E-6	0.19000	-7.5550E-10	NaN	2.0234E-4	0.0000	5.7696E-18	1.7933E-16	0.18326	0.0000	0.0000	1.1896E-5	1.0000
0.0000	4.0000E-6	0.19500	-7.5529E-10	NaN	2.0229E-4	0.0000	5.5240E-18	1.7930E-16	0.18321	0.0000	0.0000	1.1893E-5	1.0000
0.0000	4.0000E-6	0.20000	-7.5508E-	NaN	2.0223E-4	0.0000	5.2784E-18	1.7927E-16	0.18316	0.0000	0.0000	1.1890E-5	1.0000

k0Sub (m/s)	d (m)	Time (s)	-(tds2.ntflux_Oxi)*n*F (A), UME	Surface ConcOxi (M), OxiMem	Surface ConcOxi (M), OxiNuc	Substrate Kinetics (m/s), k0Sub probe	Normal total flux (mol/s), UME OCR membrane	Normal total flux (mol/s), UME OCR nucleus	Concentration (mol/m^3), Point: (5.5109E-16, 2.5)	k0Sub*Oxi (mol/s), Mem Flux	k0Sub*Oxi (mol/s), Nuc Flux	Normal total flux (mol/(m^2*s)), Point: (5.5109E-16, 2.5)	WEIectrode radius (µm), UME Radius
			10										
0.0000	4.0000E-6	0.20500	-7.5487E-10	NaN	2.0218E-4	0.0000	5.0329E-18	1.7924E-16	0.18311	0.0000	0.0000	1.1887E-5	1.0000
0.0000	4.0000E-6	0.21000	-7.5466E-10	NaN	2.0212E-4	0.0000	4.7873E-18	1.7920E-16	0.18306	0.0000	0.0000	1.1883E-5	1.0000
0.0000	4.0000E-6	0.21500	-7.5445E-10	NaN	2.0207E-4	0.0000	4.5417E-18	1.7917E-16	0.18301	0.0000	0.0000	1.1880E-5	1.0000
0.0000	4.0000E-6	0.22000	-7.5424E-10	NaN	2.0202E-4	0.0000	4.2961E-18	1.7914E-16	0.18295	0.0000	0.0000	1.1877E-5	1.0000
0.0000	4.0000E-6	0.22500	-7.5405E-10	NaN	2.0197E-4	0.0000	4.0913E-18	1.7911E-16	0.18291	0.0000	0.0000	1.1874E-5	1.0000
0.0000	4.0000E-6	0.23000	-7.5394E-10	NaN	2.0194E-4	0.0000	3.9978E-18	1.7909E-16	0.18288	0.0000	0.0000	1.1872E-5	1.0000
0.0000	4.0000E-6	0.23500	-7.5382E-10	NaN	2.0191E-4	0.0000	3.9043E-18	1.7907E-16	0.18285	0.0000	0.0000	1.1870E-5	1.0000
0.0000	4.0000E-6	0.24000	-7.5371E-10	NaN	2.0188E-4	0.0000	3.8108E-18	1.7905E-16	0.18282	0.0000	0.0000	1.1868E-5	1.0000
0.0000	4.0000E-6	0.24500	-7.5359E-10	NaN	2.0185E-4	0.0000	3.7173E-18	1.7903E-16	0.18280	0.0000	0.0000	1.1866E-5	1.0000
0.00	4.0000E-	0.25	-7.53	NaN	2.0182E-	0.00	3.6238E-	1.7900E-	0.18	0.00	0.00	1.1865E-	1.00

k0Sub (m/s)	d (m)	Time (s)	-(tds2.ntflux_Oxi)*n*F (A), UME	Surface ConcOxi (M), Oxi Mem	Surface ConcOxi (M), OxiNuc	Substrate Kinetics (m/s), k0Sub probe	Normal total flux (mol/s), UME OCR membrane	Normal total flux (mol/s), UME OCR nucleus	Concentration (mol/m^3), Point: (5.5109E-16, 2.5)	k0Sub*Oxi (mol/s), Mem Flux	k0Sub*Oxi (mol/s), Nuc Flux	Normal total flux (mol/(m^2*s)), Point: (5.5109E-16, 2.5)	WElectrode radius (µm), UME Radius
00	6	000	48E-10		4	00	18	16	277	00	00	5	00
0.0000	4.0000E-6	0.25000	-7.5348E-10	NaN	2.0182E-4	0.0000	3.6238E-18	1.7900E-16	0.18277	0.0000	0.0000	1.1865E-5	1.0000
0.0000	4.0000E-6	0.27000	-7.5302E-10	NaN	2.0170E-4	0.0000	3.2498E-18	1.7892E-16	0.18266	0.0000	0.0000	1.1857E-5	1.0000
0.0000	4.0000E-6	0.29000	-7.5256E-10	NaN	2.0158E-4	0.0000	2.8757E-18	1.7884E-16	0.18254	0.0000	0.0000	1.1850E-5	1.0000
0.0000	4.0000E-6	0.31000	-7.5210E-10	NaN	2.0146E-4	0.0000	2.5017E-18	1.7876E-16	0.18243	0.0000	0.0000	1.1843E-5	1.0000
0.0000	4.0000E-6	0.33000	-7.5170E-10	NaN	2.0135E-4	0.0000	2.2044E-18	1.7868E-16	0.18234	0.0000	0.0000	1.1836E-5	1.0000
0.0000	4.0000E-6	0.35000	-7.5141E-10	NaN	2.0128E-4	0.0000	2.0365E-18	1.7862E-16	0.18227	0.0000	0.0000	1.1832E-5	1.0000
0.0000	4.0000E-6	0.37000	-7.5113E-10	NaN	2.0120E-4	0.0000	1.8686E-18	1.7857E-16	0.18220	0.0000	0.0000	1.1827E-5	1.0000
0.0000	4.0000E-6	0.39000	-7.5084E-10	NaN	2.0113E-4	0.0000	1.7007E-18	1.7851E-16	0.18213	0.0000	0.0000	1.1823E-5	1.0000
0.0000	4.0000E-6	0.41000	-7.5055E-10	NaN	2.0105E-4	0.0000	1.5328E-18	1.7845E-16	0.18206	0.0000	0.0000	1.1818E-5	1.0000

k0Sub (m/s)	d (m)	Time (s)	-(tds2.ntflux_Oxi)*n*F (A), UME	Surface ConcOxi (M), OxiMem	Surface ConcOxi (M), OxiNuc	Substrate Kinetics (m/s), k0Sub probe	Normal total flux (mol/s), UME OCR membrane	Normal total flux (mol/s), UME OCR nucleus	Concentration (mol/m^3), Point: (5.5109E-16, 2.5)	k0Sub*Oxi (mol/s), Mem Flux	k0Sub*Oxi (mol/s), Nuc Flux	Normal total flux (mol/(m^2*s)), Point: (5.5109E-16, 2.5)	WElectrode radius (μm), UME Radius
0.0000	4.0000E-6	0.43000	-7.5030E-10	NaN	2.0099E-4	0.0000	1.3990E-18	1.7840E-16	0.18200	0.0000	0.0000	1.1814E-5	1.0000
0.0000	4.0000E-6	0.45000	-7.5010E-10	NaN	2.0093E-4	0.0000	1.3106E-18	1.7836E-16	0.18195	0.0000	0.0000	1.1811E-5	1.0000
0.0000	4.0000E-6	0.47000	-7.4990E-10	NaN	2.0088E-4	0.0000	1.2222E-18	1.7832E-16	0.18190	0.0000	0.0000	1.1808E-5	1.0000
0.0000	4.0000E-6	0.49000	-7.4971E-10	NaN	2.0083E-4	0.0000	1.1338E-18	1.7828E-16	0.18185	0.0000	0.0000	1.1805E-5	1.0000
0.0000	4.0000E-6	0.51000	-7.4951E-10	NaN	2.0078E-4	0.0000	1.0454E-18	1.7824E-16	0.18180	0.0000	0.0000	1.1802E-5	1.0000
0.0000	4.0000E-6	0.53000	-7.4933E-10	NaN	2.0073E-4	0.0000	9.7459E-19	1.7820E-16	0.18176	0.0000	0.0000	1.1799E-5	1.0000
0.0000	4.0000E-6	0.55000	-7.4919E-10	NaN	2.0069E-4	0.0000	9.2240E-19	1.7817E-16	0.18172	0.0000	0.0000	1.1797E-5	1.0000
0.0000	4.0000E-6	0.57000	-7.4904E-10	NaN	2.0065E-4	0.0000	8.7021E-19	1.7814E-16	0.18169	0.0000	0.0000	1.1794E-5	1.0000
0.0000	4.0000E-6	0.59000	-7.4889E-10	NaN	2.0061E-4	0.0000	8.1801E-19	1.7811E-16	0.18165	0.0000	0.0000	1.1792E-5	1.0000
0.0000	4.0000E-6	0.61000	-7.4875E-	NaN	2.0058E-4	0.0000	7.6582E-19	1.7808E-16	0.18162	0.0000	0.0000	1.1790E-5	1.0000

k0Sub (m/s)	d (m)	Time (s)	-(tds2.ntflux_Oxi)*n*F (A), UME	Surface Con cOxi (M), Oxi Mem	Surface Con cOxi (M), OxiNuc	Substrate Kinetics (m/s), k0Sub probe	Normal total flux (mol/s), UME OCR membrane	Normal total flux (mol/s), UME OCR nucleus	Concentration (mol/m^3), Point: (5.5109E-16, 2.5)	k0Sub*Oxi (mol/s), Mem Flux	k0Sub*Oxi (mol/s), Nuc Flux	Normal total flux (mol/(m^2*s)), Point: (5.5109E-16, 2.5)	WElectrode radius (µm), UME Radius
			10										
0.0000	4.0000E-6	0.63000	-7.4862E-10	NaN	2.0054E-4	0.0000	7.2290E-19	1.7805E-16	0.18159	0.0000	0.0000	1.1788E-5	1.0000
0.0000	4.0000E-6	0.65000	-7.4850E-10	NaN	2.0051E-4	0.0000	6.8773E-19	1.7802E-16	0.18156	0.0000	0.0000	1.1786E-5	1.0000
0.0000	4.0000E-6	0.67000	-7.4838E-10	NaN	2.0048E-4	0.0000	6.5256E-19	1.7800E-16	0.18153	0.0000	0.0000	1.1784E-5	1.0000
0.0000	4.0000E-6	0.69000	-7.4827E-10	NaN	2.0045E-4	0.0000	6.1739E-19	1.7797E-16	0.18150	0.0000	0.0000	1.1782E-5	1.0000
0.0000	4.0000E-6	0.71000	-7.4815E-10	NaN	2.0042E-4	0.0000	5.8222E-19	1.7795E-16	0.18147	0.0000	0.0000	1.1780E-5	1.0000
0.0000	4.0000E-6	0.73000	-7.4805E-10	NaN	2.0039E-4	0.0000	5.5417E-19	1.7793E-16	0.18145	0.0000	0.0000	1.1779E-5	1.0000
0.0000	4.0000E-6	0.75000	-7.4796E-10	NaN	2.0037E-4	0.0000	5.3086E-19	1.7791E-16	0.18143	0.0000	0.0000	1.1777E-5	1.0000
0.0000	4.0000E-6	0.77000	-7.4786E-10	NaN	2.0034E-4	0.0000	5.0754E-19	1.7789E-16	0.18140	0.0000	0.0000	1.1776E-5	1.0000
0.0000	4.0000E-6	0.79000	-7.4777E-10	NaN	2.0032E-4	0.0000	4.8423E-19	1.7787E-16	0.18138	0.0000	0.0000	1.1774E-5	1.0000
0.00	4.0000E-	0.81	-7.47	NaN	2.0029E-	0.00	4.6092E-	1.7784E-	0.18	0.00	0.00	1.1773E-	1.00

k0Sub (m/s)	d (m)	Time (s)	-(tds2.ntflux_Oxi)*n*F (A), UME	Surface ConcOxi (M), OxiMem	Surface ConcOxi (M), OxiNuc	Substrate Kinetics (m/s), k0Sub probe	Normal total flux (mol/s), UME OCR membrane	Normal total flux (mol/s), UME OCR nucleus	Concentration (mol/m^3), Point: (5.5109E-16, 2.5)	k0Sub*Oxi (mol/s), Mem Flux	k0Sub*Oxi (mol/s), Nuc Flux	Normal total flux (mol/(m^2*s)), Point: (5.5109E-16, 2.5)	WElectrode radius (µm), UME Radius
00	6	000	68E-10		4	00	19	16	136	00	00	5	00
0.0000	4.0000E-6	0.83000	-7.4759E-10	NaN	2.0027E-4	0.0000	4.4005E-19	1.7783E-16	0.18134	0.0000	0.0000	1.1772E-5	1.0000
0.0000	4.0000E-6	0.85000	-7.4751E-10	NaN	2.0025E-4	0.0000	4.2157E-19	1.7781E-16	0.18132	0.0000	0.0000	1.1770E-5	1.0000
0.0000	4.0000E-6	0.87000	-7.4743E-10	NaN	2.0023E-4	0.0000	4.0441E-19	1.7779E-16	0.18130	0.0000	0.0000	1.1769E-5	1.0000
0.0000	4.0000E-6	0.89000	-7.4736E-10	NaN	2.0021E-4	0.0000	3.8859E-19	1.7777E-16	0.18128	0.0000	0.0000	1.1768E-5	1.0000
0.0000	4.0000E-6	0.91000	-7.4728E-10	NaN	2.0019E-4	0.0000	3.7409E-19	1.7776E-16	0.18126	0.0000	0.0000	1.1767E-5	1.0000
0.0000	4.0000E-6	0.93000	-7.4721E-10	NaN	2.0017E-4	0.0000	3.6005E-19	1.7774E-16	0.18125	0.0000	0.0000	1.1766E-5	1.0000
0.0000	4.0000E-6	0.95000	-7.4714E-10	NaN	2.0015E-4	0.0000	3.4654E-19	1.7773E-16	0.18123	0.0000	0.0000	1.1765E-5	1.0000
0.0000	4.0000E-6	0.97000	-7.4708E-10	NaN	2.0013E-4	0.0000	3.3390E-19	1.7771E-16	0.18121	0.0000	0.0000	1.1764E-5	1.0000
0.0000	4.0000E-6	0.99000	-7.4701E-10	NaN	2.0011E-4	0.0000	3.2213E-19	1.7770E-16	0.18120	0.0000	0.0000	1.1762E-5	1.0000

k0Sub (m/s)	d (m)	Time (s)	-(tds2.ntflux_Oxi)*n*F (A), UME	Surface ConcOxi (M), Oxi Mem	Surface ConcOxi (M), OxiNuc	Substrate Kinetics (m/s), k0Sub probe	Normal total flux (mol/s), UME OCR membrane	Normal total flux (mol/s), UME OCR nucleus	Concentration (mol/m^3), Point: (5.5109E-16, 2.5)	k0Sub*Oxi (mol/s), Mem Flux	k0Sub*Oxi (mol/s), Nuc Flux	Normal total flux (mol/(m^2*s)), Point: (5.5109E-16, 2.5)	WElectrode radius (µm), UME Radius
0.0000	5.0000E-6	0.0000	-7.1537E-9	NaN	2.2700E-4	0.0000	-1.0245E-28	-3.3494E-30	0.22700	0.0000	0.0000	-2.5611E-19	1.0000
0.0000	5.0000E-6	0.005000 0	-8.5517E-10	NaN	2.2391E-4	0.0000	3.4646E-16	8.9134E-17	0.21536	0.0000	0.0000	7.6786E-6	1.0000
0.0000	5.0000E-6	0.010000	-8.2729E-10	NaN	2.2001E-4	0.0000	2.6326E-16	1.1720E-16	0.20868	0.0000	0.0000	7.7460E-6	1.0000
0.0000	5.0000E-6	0.015000	-8.1473E-10	NaN	2.1741E-4	0.0000	1.8646E-16	1.2232E-16	0.20536	0.0000	0.0000	7.6108E-6	1.0000
0.0000	5.0000E-6	0.020000	-8.0662E-10	NaN	2.1561E-4	0.0000	1.4326E-16	1.2450E-16	0.20324	0.0000	0.0000	7.5260E-6	1.0000
0.0000	5.0000E-6	0.025000	-8.0025E-10	NaN	2.1419E-4	0.0000	1.1301E-16	1.2602E-16	0.20159	0.0000	0.0000	7.4644E-6	1.0000
0.0000	5.0000E-6	0.030000	-7.9542E-10	NaN	2.1309E-4	0.0000	9.2326E-17	1.2698E-16	0.20034	0.0000	0.0000	7.4187E-6	1.0000
0.0000	5.0000E-6	0.035000	-7.9167E-10	NaN	2.1223E-4	0.0000	7.7399E-17	1.2761E-16	0.19938	0.0000	0.0000	7.3832E-6	1.0000
0.0000	5.0000E-6	0.040000	-7.8871E-10	NaN	2.1154E-4	0.0000	6.6572E-17	1.2800E-16	0.19862	0.0000	0.0000	7.3554E-6	1.0000
0.0000	5.0000E-6	0.045000	-7.8576E-	NaN	2.1084E-4	0.0000	5.5746E-17	1.2838E-16	0.19786	0.0000	0.0000	7.3275E-6	1.0000

k0Sub (m/s)	d (m)	Time (s)	-(tds2.ntflux_Oxi)*n*F (A), UME	Surface ConcOxi (M), OxiMem	Surface ConcOxi (M), OxiNuc	Substrate Kinetics (m/s), k0Sub probe	Normal total flux (mol/s), UME OCR membrane	Normal total flux (mol/s), UME OCR nucleus	Concentration (mol/m^3), Point: (5.5109E-16, 2.5)	k0Sub*Oxi (mol/s), Mem Flux	k0Sub*Oxi (mol/s), Nuc Flux	Normal total flux (mol/(m^2*s)), Point: (5.5109E-16, 2.5)	WElectrode radius (µm), UME Radius
			10										
0.0000	5.0000E-6	0.050000	-7.8358E-10	NaN	2.1032E-4	0.0000	4.8567E-17	1.2860E-16	0.19730	0.0000	0.0000	7.3068E-6	1.0000
0.0000	5.0000E-6	0.055000	-7.8162E-10	NaN	2.0985E-4	0.0000	4.2344E-17	1.2876E-16	0.19680	0.0000	0.0000	7.2883E-6	1.0000
0.0000	5.0000E-6	0.060000	-7.7968E-10	NaN	2.0939E-4	0.0000	3.6242E-17	1.2891E-16	0.19630	0.0000	0.0000	7.2699E-6	1.0000
0.0000	5.0000E-6	0.065000	-7.7858E-10	NaN	2.0912E-4	0.0000	3.3406E-17	1.2894E-16	0.19602	0.0000	0.0000	7.2595E-6	1.0000
0.0000	5.0000E-6	0.070000	-7.7748E-10	NaN	2.0885E-4	0.0000	3.0570E-17	1.2897E-16	0.19574	0.0000	0.0000	7.2490E-6	1.0000
0.0000	5.0000E-6	0.075000	-7.7638E-10	NaN	2.0857E-4	0.0000	2.7735E-17	1.2900E-16	0.19546	0.0000	0.0000	7.2386E-6	1.0000
0.0000	5.0000E-6	0.080000	-7.7528E-10	NaN	2.0830E-4	0.0000	2.4899E-17	1.2903E-16	0.19518	0.0000	0.0000	7.2282E-6	1.0000
0.0000	5.0000E-6	0.085000	-7.7418E-10	NaN	2.0803E-4	0.0000	2.2063E-17	1.2906E-16	0.19490	0.0000	0.0000	7.2177E-6	1.0000
0.0000	5.0000E-6	0.090000	-7.7329E-10	NaN	2.0781E-4	0.0000	1.9972E-17	1.2907E-16	0.19467	0.0000	0.0000	7.2093E-6	1.0000
0.00	5.0000E-	0.09	-7.72	NaN	2.0764E-	0.00	1.8570E-	1.2906E-	0.19	0.00	0.00	7.2029E-	1.00

k0Sub (m/s)	d (m)	Time (s)	-(tds2.ntflux_Oxi)*n*F (A), UME	Surface ConcOxi (M), OxiMem	Surface ConcOxi (M), OxiNuc	Substrate Kinetics (m/s), k0Sub probe	Normal total flux (mol/s), UME OCR membrane	Normal total flux (mol/s), UME OCR nucleus	Concentration (mol/m^3), Point: (5.5109E-16, 2.5)	k0Sub*Oxi (mol/s), Mem Flux	k0Sub*Oxi (mol/s), Nuc Flux	Normal total flux (mol/(m^2*s)), Point: (5.5109E-16, 2.5)	WElectrode radius (µm), UME Radius
00	6	5000	62E-10		4	00	17	16	450	00	00	6	00
0.0000	5.0000E-6	0.10000	-7.7195E-10	NaN	2.0747E-4	0.0000	1.7168E-17	1.2905E-16	0.19433	0.0000	0.0000	7.1965E-6	1.0000
0.0000	5.0000E-6	0.10500	-7.7127E-10	NaN	2.0731E-4	0.0000	1.5766E-17	1.2904E-16	0.19416	0.0000	0.0000	7.1901E-6	1.0000
0.0000	5.0000E-6	0.11000	-7.7060E-10	NaN	2.0714E-4	0.0000	1.4364E-17	1.2903E-16	0.19399	0.0000	0.0000	7.1838E-6	1.0000
0.0000	5.0000E-6	0.11500	-7.6992E-10	NaN	2.0697E-4	0.0000	1.2962E-17	1.2902E-16	0.19382	0.0000	0.0000	7.1774E-6	1.0000
0.0000	5.0000E-6	0.12000	-7.6956E-10	NaN	2.0687E-4	0.0000	1.2416E-17	1.2900E-16	0.19372	0.0000	0.0000	7.1739E-6	1.0000
0.0000	5.0000E-6	0.12500	-7.6920E-10	NaN	2.0678E-4	0.0000	1.1866E-17	1.2897E-16	0.19363	0.0000	0.0000	7.1706E-6	1.0000
0.0000	5.0000E-6	0.13000	-7.6885E-10	NaN	2.0669E-4	0.0000	1.1316E-17	1.2895E-16	0.19354	0.0000	0.0000	7.1672E-6	1.0000
0.0000	5.0000E-6	0.13500	-7.6849E-10	NaN	2.0660E-4	0.0000	1.0766E-17	1.2893E-16	0.19345	0.0000	0.0000	7.1638E-6	1.0000
0.0000	5.0000E-6	0.14000	-7.6814E-10	NaN	2.0651E-4	0.0000	1.0216E-17	1.2891E-16	0.19336	0.0000	0.0000	7.1605E-6	1.0000

k0Sub (m/s)	d (m)	Time (s)	-(tds2.ntflux_Oxi)*n*F (A), UME	Surface ConcOxi (M), Oxi Mem	Surface ConcOxi (M), OxiNuc	Substrate Kinetics (m/s), k0Sub probe	Normal total flux (mol/s), UME OCR membrane	Normal total flux (mol/s), UME OCR nucleus	Concentration (mol/m^3), Point: (5.5109E-16, 2.5)	k0Sub*Oxi (mol/s), Mem Flux	k0Sub*Oxi (mol/s), Nuc Flux	Normal total flux (mol/(m^2*s)), Point: (5.5109E-16, 2.5)	WElectrode radius (µm), UME Radius
0.0000	5.0000E-6	0.14500	-7.6778E-10	NaN	2.0642E-4	0.0000	9.6654E-18	1.2889E-16	0.19327	0.0000	0.0000	7.1571E-6	1.0000
0.0000	5.0000E-6	0.15000	-7.6742E-10	NaN	2.0633E-4	0.0000	9.1152E-18	1.2887E-16	0.19318	0.0000	0.0000	7.1537E-6	1.0000
0.0000	5.0000E-6	0.15500	-7.6707E-10	NaN	2.0624E-4	0.0000	8.5649E-18	1.2885E-16	0.19309	0.0000	0.0000	7.1504E-6	1.0000
0.0000	5.0000E-6	0.16000	-7.6671E-10	NaN	2.0614E-4	0.0000	8.0147E-18	1.2883E-16	0.19300	0.0000	0.0000	7.1470E-6	1.0000
0.0000	5.0000E-6	0.16500	-7.6636E-10	NaN	2.0605E-4	0.0000	7.4645E-18	1.2881E-16	0.19291	0.0000	0.0000	7.1436E-6	1.0000
0.0000	5.0000E-6	0.17000	-7.6600E-10	NaN	2.0596E-4	0.0000	6.9143E-18	1.2879E-16	0.19282	0.0000	0.0000	7.1403E-6	1.0000
0.0000	5.0000E-6	0.17500	-7.6578E-10	NaN	2.0590E-4	0.0000	6.6619E-18	1.2877E-16	0.19276	0.0000	0.0000	7.1382E-6	1.0000
0.0000	5.0000E-6	0.18000	-7.6557E-10	NaN	2.0585E-4	0.0000	6.4177E-18	1.2875E-16	0.19271	0.0000	0.0000	7.1362E-6	1.0000
0.0000	5.0000E-6	0.18500	-7.6535E-10	NaN	2.0579E-4	0.0000	6.1735E-18	1.2873E-16	0.19266	0.0000	0.0000	7.1342E-6	1.0000
0.0000	5.0000E-6	0.19000	-7.6514E-	NaN	2.0574E-4	0.0000	5.9293E-18	1.2871E-16	0.19260	0.0000	0.0000	7.1322E-6	1.0000

k0Sub (m/s)	d (m)	Time (s)	-(tds2.ntflux_Oxi)*n*F (A), UME	Surface ConcOxi (M), Oxi Mem	Surface ConcOxi (M), OxiNuc	Substrate Kinetics (m/s), k0Sub probe	Normal total flux (mol/s), UME OCR membrane	Normal total flux (mol/s), UME OCR nucleus	Concentration (mol/m^3), Point: (5.5109E-16, 2.5)	k0Sub*Oxi (mol/s), Mem Flux	k0Sub*Oxi (mol/s), Nuc Flux	Normal total flux (mol/(m^2*s)), Point: (5.5109E-16, 2.5)	WElectrode radius (µm), UME Radius
			10										
0.0000	5.0000E-6	0.19500	-7.6493E-10	NaN	2.0568E-4	0.0000	5.6851E-18	1.2869E-16	0.19255	0.0000	0.0000	7.1302E-6	1.0000
0.0000	5.0000E-6	0.20000	-7.6471E-10	NaN	2.0563E-4	0.0000	5.4409E-18	1.2867E-16	0.19249	0.0000	0.0000	7.1282E-6	1.0000
0.0000	5.0000E-6	0.20500	-7.6450E-10	NaN	2.0557E-4	0.0000	5.1968E-18	1.2865E-16	0.19244	0.0000	0.0000	7.1261E-6	1.0000
0.0000	5.0000E-6	0.21000	-7.6429E-10	NaN	2.0552E-4	0.0000	4.9526E-18	1.2863E-16	0.19239	0.0000	0.0000	7.1241E-6	1.0000
0.0000	5.0000E-6	0.21500	-7.6407E-10	NaN	2.0546E-4	0.0000	4.7084E-18	1.2862E-16	0.19233	0.0000	0.0000	7.1221E-6	1.0000
0.0000	5.0000E-6	0.22000	-7.6386E-10	NaN	2.0541E-4	0.0000	4.4642E-18	1.2860E-16	0.19228	0.0000	0.0000	7.1201E-6	1.0000
0.0000	5.0000E-6	0.22500	-7.6365E-10	NaN	2.0535E-4	0.0000	4.2200E-18	1.2858E-16	0.19223	0.0000	0.0000	7.1181E-6	1.0000
0.0000	5.0000E-6	0.23000	-7.6352E-10	NaN	2.0532E-4	0.0000	4.1173E-18	1.2856E-16	0.19219	0.0000	0.0000	7.1169E-6	1.0000
0.0000	5.0000E-6	0.23500	-7.6340E-10	NaN	2.0529E-4	0.0000	4.0225E-18	1.2855E-16	0.19216	0.0000	0.0000	7.1158E-6	1.0000
0.00	5.0000E-	0.24	-7.63	NaN	2.0526E-	0.00	3.9277E-	1.2854E-	0.19	0.00	0.00	7.1147E-	1.00

k0Sub (m/s)	d (m)	Time (s)	-(tds2.ntflux_Oxi)*n*F (A), UME	Surface ConcOxi (M), OxiMem	Surface ConcOxi (M), OxiNuc	Substrate Kinetics (m/s), k0Sub probe	Normal total flux (mol/s), UME OCR membrane	Normal total flux (mol/s), UME OCR nucleus	Concentration (mol/m^3), Point: (5.5109E-16, 2.5)	k0Sub*Oxi (mol/s), Mem Flux	k0Sub*Oxi (mol/s), Nuc Flux	Normal total flux (mol/(m^2*s)), Point: (5.5109E-16, 2.5)	WElectrode radius (µm), UME Radius
00	6	000	29E-10		4	00	18	16	213	00	00	6	00
0.0000	5.0000E-6	0.24500	-7.6317E-10	NaN	2.0523E-4	0.0000	3.8330E-18	1.2852E-16	0.19210	0.0000	0.0000	7.1136E-6	1.0000
0.0000	5.0000E-6	0.25000	-7.6305E-10	NaN	2.0520E-4	0.0000	3.7382E-18	1.2851E-16	0.19207	0.0000	0.0000	7.1125E-6	1.0000
0.0000	5.0000E-6	0.25000	-7.6305E-10	NaN	2.0520E-4	0.0000	3.7382E-18	1.2851E-16	0.19207	0.0000	0.0000	7.1125E-6	1.0000
0.0000	5.0000E-6	0.27000	-7.6258E-10	NaN	2.0507E-4	0.0000	3.3591E-18	1.2846E-16	0.19196	0.0000	0.0000	7.1081E-6	1.0000
0.0000	5.0000E-6	0.29000	-7.6211E-10	NaN	2.0495E-4	0.0000	2.9800E-18	1.2840E-16	0.19184	0.0000	0.0000	7.1037E-6	1.0000
0.0000	5.0000E-6	0.31000	-7.6164E-10	NaN	2.0482E-4	0.0000	2.6010E-18	1.2835E-16	0.19172	0.0000	0.0000	7.0992E-6	1.0000
0.0000	5.0000E-6	0.33000	-7.6122E-10	NaN	2.0471E-4	0.0000	2.2821E-18	1.2830E-16	0.19161	0.0000	0.0000	7.0953E-6	1.0000
0.0000	5.0000E-6	0.35000	-7.6092E-10	NaN	2.0464E-4	0.0000	2.1100E-18	1.2826E-16	0.19154	0.0000	0.0000	7.0925E-6	1.0000
0.0000	5.0000E-6	0.37000	-7.6063E-10	NaN	2.0456E-4	0.0000	1.9379E-18	1.2822E-16	0.19146	0.0000	0.0000	7.0897E-6	1.0000

k0Sub (m/s)	d (m)	Time (s)	-(tds2.ntflux_Oxi)*n*F (A), UME	Surface ConcOxi (M), OxiMem	Surface ConcOxi (M), OxiNuc	Substrate Kinetics (m/s), k0Sub probe	Normal total flux (mol/s), UME OCR membrane	Normal total flux (mol/s), UME OCR nucleus	Concentration (mol/m^3), Point: (5.5109E-16, 2.5)	k0Sub*Oxi (mol/s), Mem Flux	k0Sub*Oxi (mol/s), Nuc Flux	Normal total flux (mol/(m^2*s)), Point: (5.5109E-16, 2.5)	WElectrode radius (µm), UME Radius
0.0000	5.0000E-6	0.39000	-7.6034E-10	NaN	2.0448E-4	0.0000	1.7659E-18	1.2819E-16	0.19139	0.0000	0.0000	7.0870E-6	1.0000
0.0000	5.0000E-6	0.41000	-7.6004E-10	NaN	2.0440E-4	0.0000	1.5938E-18	1.2815E-16	0.19131	0.0000	0.0000	7.0842E-6	1.0000
0.0000	5.0000E-6	0.43000	-7.5978E-10	NaN	2.0433E-4	0.0000	1.4498E-18	1.2811E-16	0.19125	0.0000	0.0000	7.0817E-6	1.0000
0.0000	5.0000E-6	0.45000	-7.5957E-10	NaN	2.0428E-4	0.0000	1.3588E-18	1.2808E-16	0.19120	0.0000	0.0000	7.0798E-6	1.0000
0.0000	5.0000E-6	0.47000	-7.5937E-10	NaN	2.0422E-4	0.0000	1.2678E-18	1.2806E-16	0.19114	0.0000	0.0000	7.0779E-6	1.0000
0.0000	5.0000E-6	0.49000	-7.5917E-10	NaN	2.0417E-4	0.0000	1.1768E-18	1.2803E-16	0.19109	0.0000	0.0000	7.0760E-6	1.0000
0.0000	5.0000E-6	0.51000	-7.5896E-10	NaN	2.0412E-4	0.0000	1.0858E-18	1.2800E-16	0.19104	0.0000	0.0000	7.0741E-6	1.0000
0.0000	5.0000E-6	0.53000	-7.5878E-10	NaN	2.0407E-4	0.0000	1.0097E-18	1.2797E-16	0.19099	0.0000	0.0000	7.0724E-6	1.0000
0.0000	5.0000E-6	0.55000	-7.5863E-10	NaN	2.0403E-4	0.0000	9.5590E-19	1.2795E-16	0.19096	0.0000	0.0000	7.0710E-6	1.0000
0.0000	5.0000E-6	0.57000	-7.5848E-	NaN	2.0399E-4	0.0000	9.0208E-19	1.2793E-16	0.19092	0.0000	0.0000	7.0696E-6	1.0000

k0Sub (m/s)	d (m)	Time (s)	-(tds2.ntflux_Oxi)*n*F (A), UME	Surface ConcOxi (M), Oxi Mem	Surface ConcOxi (M), OxiNuc	Substrate Kinetics (m/s), k0Sub probe	Normal total flux (mol/s), UME OCR membrane	Normal total flux (mol/s), UME OCR nucleus	Concentration (mol/m^3), Point: (5.5109E-16, 2.5)	k0Sub*Oxi (mol/s), Mem Flux	k0Sub*Oxi (mol/s), Nuc Flux	Normal total flux (mol/(m^2*s)), Point: (5.5109E-16, 2.5)	WElectrode radius (µm), UME Radius
			10										
0.0000	5.0000E-6	0.59000	-7.5833E-10	NaN	2.0395E-4	0.0000	8.4827E-19	1.2791E-16	0.19088	0.0000	0.0000	7.0682E-6	1.0000
0.0000	5.0000E-6	0.61000	-7.5818E-10	NaN	2.0391E-4	0.0000	7.9446E-19	1.2789E-16	0.19084	0.0000	0.0000	7.0668E-6	1.0000
0.0000	5.0000E-6	0.63000	-7.5804E-10	NaN	2.0387E-4	0.0000	7.4867E-19	1.2787E-16	0.19081	0.0000	0.0000	7.0655E-6	1.0000
0.0000	5.0000E-6	0.65000	-7.5792E-10	NaN	2.0384E-4	0.0000	7.1240E-19	1.2785E-16	0.19078	0.0000	0.0000	7.0644E-6	1.0000
0.0000	5.0000E-6	0.67000	-7.5780E-10	NaN	2.0381E-4	0.0000	6.7612E-19	1.2783E-16	0.19075	0.0000	0.0000	7.0633E-6	1.0000
0.0000	5.0000E-6	0.69000	-7.5768E-10	NaN	2.0378E-4	0.0000	6.3984E-19	1.2781E-16	0.19072	0.0000	0.0000	7.0621E-6	1.0000
0.0000	5.0000E-6	0.71000	-7.5756E-10	NaN	2.0374E-4	0.0000	6.0356E-19	1.2779E-16	0.19069	0.0000	0.0000	7.0610E-6	1.0000
0.0000	5.0000E-6	0.73000	-7.5745E-10	NaN	2.0372E-4	0.0000	5.7355E-19	1.2778E-16	0.19066	0.0000	0.0000	7.0600E-6	1.0000
0.0000	5.0000E-6	0.75000	-7.5736E-10	NaN	2.0369E-4	0.0000	5.4950E-19	1.2776E-16	0.19064	0.0000	0.0000	7.0591E-6	1.0000
0.00	5.0000E-	0.77	-7.57	NaN	2.0366E-	0.00	5.2545E-	1.2775E-	0.19	0.00	0.00	7.0582E-	1.00

k0Sub (m/s)	d (m)	Time (s)	-(tds2.ntflux_Oxi)*n*F (A), UME	Surface ConcOxi (M), Oxi Mem	Surface ConcOxi (M), OxiNuc	Substrate Kinetics (m/s), k0Sub probe	Normal total flux (mol/s), UME OCR membrane	Normal total flux (mol/s), UME OCR nucleus	Concentration (mol/m^3), Point: (5.5109E-16, 2.5)	k0Sub*Oxi (mol/s), Mem Flux	k0Sub*Oxi (mol/s), Nuc Flux	Normal total flux (mol/(m^2*s)), Point: (5.5109E-16, 2.5)	WElectrode radius (μm), UME Radius
00	6	000	26E-10		4	00	19	16	061	00	00	6	00
0.0000	5.0000E-6	0.79000	-7.5717E-10	NaN	2.0364E-4	0.0000	5.0140E-19	1.2774E-16	0.19059	0.0000	0.0000	7.0574E-6	1.0000
0.0000	5.0000E-6	0.81000	-7.5708E-10	NaN	2.0361E-4	0.0000	4.7734E-19	1.2772E-16	0.19057	0.0000	0.0000	7.0565E-6	1.0000
0.0000	5.0000E-6	0.83000	-7.5698E-10	NaN	2.0359E-4	0.0000	4.5544E-19	1.2771E-16	0.19054	0.0000	0.0000	7.0556E-6	1.0000
0.0000	5.0000E-6	0.85000	-7.5690E-10	NaN	2.0357E-4	0.0000	4.3624E-19	1.2769E-16	0.19052	0.0000	0.0000	7.0548E-6	1.0000
0.0000	5.0000E-6	0.87000	-7.5682E-10	NaN	2.0355E-4	0.0000	4.1842E-19	1.2768E-16	0.19050	0.0000	0.0000	7.0541E-6	1.0000
0.0000	5.0000E-6	0.89000	-7.5674E-10	NaN	2.0352E-4	0.0000	4.0197E-19	1.2767E-16	0.19048	0.0000	0.0000	7.0533E-6	1.0000
0.0000	5.0000E-6	0.91000	-7.5667E-10	NaN	2.0350E-4	0.0000	3.8689E-19	1.2766E-16	0.19046	0.0000	0.0000	7.0526E-6	1.0000
0.0000	5.0000E-6	0.93000	-7.5659E-10	NaN	2.0349E-4	0.0000	3.7241E-19	1.2765E-16	0.19044	0.0000	0.0000	7.0520E-6	1.0000
0.0000	5.0000E-6	0.95000	-7.5652E-10	NaN	2.0347E-4	0.0000	3.5839E-19	1.2764E-16	0.19043	0.0000	0.0000	7.0513E-6	1.0000

k0Sub (m/s)	d (m)	Time (s)	-(tds2.ntflux_Oxi)*n*F (A), UME	Surface ConcOxi (M), OxiMem	Surface ConcOxi (M), OxiNuc	Substrate Kinetics (m/s), k0Sub probe	Normal total flux (mol/s), UME OCR membrane	Normal total flux (mol/s), UME OCR nucleus	Concentration (mol/m^3), Point: (5.5109E-16, 2.5)	k0Sub*Oxi (mol/s), Mem Flux	k0Sub*Oxi (mol/s), Nuc Flux	Normal total flux (mol/(m^2*s)), Point: (5.5109E-16, 2.5)	WEIectrode radius (μm), UME Radius
0.0000	5.0000E-6	0.97000	-7.5645E-10	NaN	2.0345E-4	0.0000	3.4527E-19	1.2762E-16	0.19041	0.0000	0.0000	7.0507E-6	1.0000
0.0000	5.0000E-6	0.99000	-7.5639E-10	NaN	2.0343E-4	0.0000	3.3305E-19	1.2761E-16	0.19039	0.0000	0.0000	7.0501E-6	1.0000
0.0000	6.0000E-6	0.0000	-7.1535E-9	NaN	2.2700E-4	0.0000	2.8894E-30	-1.4724E-29	0.22700	0.0000	0.0000	0.0000	1.0000
0.0000	6.0000E-6	0.0050000	-8.5561E-10	NaN	2.2509E-4	0.0000	2.5552E-16	5.4742E-17	0.22009	0.0000	0.0000	4.5478E-6	1.0000
0.0000	6.0000E-6	0.010000	-8.2932E-10	NaN	2.2194E-4	0.0000	2.2970E-16	8.1378E-17	0.21443	0.0000	0.0000	4.8963E-6	1.0000
0.0000	6.0000E-6	0.015000	-8.1810E-10	NaN	2.1962E-4	0.0000	1.6807E-16	8.7033E-17	0.21140	0.0000	0.0000	4.8308E-6	1.0000
0.0000	6.0000E-6	0.020000	-8.1085E-10	NaN	2.1798E-4	0.0000	1.3016E-16	8.9392E-17	0.20943	0.0000	0.0000	4.7793E-6	1.0000
0.0000	6.0000E-6	0.025000	-8.0514E-10	NaN	2.1668E-4	0.0000	1.0375E-16	9.0989E-17	0.20790	0.0000	0.0000	4.7435E-6	1.0000
0.0000	6.0000E-6	0.030000	-8.0075E-10	NaN	2.1566E-4	0.0000	8.5373E-17	9.2022E-17	0.20673	0.0000	0.0000	4.7167E-6	1.0000
0.0000	6.0000E-6	0.035000	-7.9729E-	NaN	2.1485E-4	0.0000	7.1954E-17	9.2723E-17	0.20581	0.0000	0.0000	4.6957E-6	1.0000

k0Sub (m/s)	d (m)	Time (s)	-(tds2.ntflux_Oxi)*n*F (A), UME	Surface Con cOxi (M), Oxi Mem	Surface Con cOxi (M), OxiNuc	Substrate Kinetics (m/s), k0Sub probe	Normal total flux (mol/s), UME OCR membrane	Normal total flux (mol/s), UME OCR nucleus	Concentration (mol/m^3), Point: (5.5109E-16, 2.5)	k0Sub*Oxi (mol/s), Mem Flux	k0Sub*Oxi (mol/s), Nuc Flux	Normal total flux (mol/(m^2*s)), Point: (5.5109E-16, 2.5)	WElectrode radius (µm), UME Radius
			10										
0.0000	6.0000E-6	0.040000	-7.9457E-10	NaN	2.1420E-4	0.0000	6.2303E-17	9.3170E-17	0.20509	0.0000	0.0000	4.6791E-6	1.0000
0.0000	6.0000E-6	0.045000	-7.9185E-10	NaN	2.1354E-4	0.0000	5.2652E-17	9.3616E-17	0.20437	0.0000	0.0000	4.6625E-6	1.0000
0.0000	6.0000E-6	0.050000	-7.8979E-10	NaN	2.1304E-4	0.0000	4.6033E-17	9.3887E-17	0.20382	0.0000	0.0000	4.6498E-6	1.0000
0.0000	6.0000E-6	0.055000	-7.8795E-10	NaN	2.1259E-4	0.0000	4.0403E-17	9.4096E-17	0.20334	0.0000	0.0000	4.6385E-6	1.0000
0.0000	6.0000E-6	0.060000	-7.8611E-10	NaN	2.1214E-4	0.0000	3.4774E-17	9.4306E-17	0.20285	0.0000	0.0000	4.6272E-6	1.0000
0.0000	6.0000E-6	0.065000	-7.8504E-10	NaN	2.1187E-4	0.0000	3.2109E-17	9.4374E-17	0.20257	0.0000	0.0000	4.6206E-6	1.0000
0.0000	6.0000E-6	0.070000	-7.8398E-10	NaN	2.1161E-4	0.0000	2.9488E-17	9.4438E-17	0.20229	0.0000	0.0000	4.6141E-6	1.0000
0.0000	6.0000E-6	0.075000	-7.8293E-10	NaN	2.1135E-4	0.0000	2.6867E-17	9.4503E-17	0.20201	0.0000	0.0000	4.6076E-6	1.0000
0.0000	6.0000E-6	0.080000	-7.8188E-10	NaN	2.1108E-4	0.0000	2.4245E-17	9.4567E-17	0.20174	0.0000	0.0000	4.6012E-6	1.0000
0.00	6.0000E-	0.08	-7.80	NaN	2.1082E-	0.00	2.1624E-	9.4631E-	0.20	0.00	0.00	4.5947E-	1.00

k0Sub (m/s)	d (m)	Time (s)	-(tds2.ntflux_Oxi)*n*F (A), UME	Surface Con cOxi (M), Oxi Mem	Surface Con cOxi (M), OxiNuc	Substrate Kinetics (m/s), k0Sub probe	Normal total flux (mol/s), UME OCR membrane	Normal total flux (mol/s), UME OCR nucleus	Concentration (mol/m^3), Point: (5.5109E-16, 2.5)	k0Sub* Oxi (mol/s), Mem Flux	k0Sub* Oxi (mol/s), Nuc Flux	Normal total flux (mol/(m^2*s)), Point: (5.5109E-16, 2.5)	WElectrode radius (µm), UME Radius
00	6	5000	82E-10		4	00	17	17	146	00	00	6	00
0.0000	6.0000E-6	0.090000	-7.7994E-10	NaN	2.1060E-4	0.0000	1.9582E-17	9.4673E-17	0.20123	0.0000	0.0000	4.5892E-6	1.0000
0.0000	6.0000E-6	0.095000	-7.7928E-10	NaN	2.1043E-4	0.0000	1.8257E-17	9.4687E-17	0.20105	0.0000	0.0000	4.5852E-6	1.0000
0.0000	6.0000E-6	0.10000	-7.7862E-10	NaN	2.1026E-4	0.0000	1.6933E-17	9.4700E-17	0.20088	0.0000	0.0000	4.5811E-6	1.0000
0.0000	6.0000E-6	0.10500	-7.7796E-10	NaN	2.1009E-4	0.0000	1.5609E-17	9.4714E-17	0.20071	0.0000	0.0000	4.5771E-6	1.0000
0.0000	6.0000E-6	0.11000	-7.7731E-10	NaN	2.0993E-4	0.0000	1.4285E-17	9.4727E-17	0.20054	0.0000	0.0000	4.5731E-6	1.0000
0.0000	6.0000E-6	0.11500	-7.7665E-10	NaN	2.0976E-4	0.0000	1.2961E-17	9.4741E-17	0.20036	0.0000	0.0000	4.5690E-6	1.0000
0.0000	6.0000E-6	0.12000	-7.7626E-10	NaN	2.0966E-4	0.0000	1.2356E-17	9.4736E-17	0.20026	0.0000	0.0000	4.5667E-6	1.0000
0.0000	6.0000E-6	0.12500	-7.7591E-10	NaN	2.0957E-4	0.0000	1.1824E-17	9.4729E-17	0.20017	0.0000	0.0000	4.5645E-6	1.0000
0.0000	6.0000E-6	0.13000	-7.7555E-10	NaN	2.0947E-4	0.0000	1.1291E-17	9.4723E-17	0.20008	0.0000	0.0000	4.5623E-6	1.0000

k0Sub (m/s)	d (m)	Time (s)	-(tds2.ntflux_Oxi)*n*F (A), UME	Surface ConcOxi (M), OxiMem	Surface ConcOxi (M), OxiNuc	Substrate Kinetics (m/s), k0Sub probe	Normal total flux (mol/s), UME OCR membrane	Normal total flux (mol/s), UME OCR nucleus	Concentration (mol/m^3), Point: (5.5109E-16, 2.5)	k0Sub*Oxi (mol/s), Mem Flux	k0Sub*Oxi (mol/s), Nuc Flux	Normal total flux (mol/(m^2*s)), Point: (5.5109E-16, 2.5)	WElectrode radius (µm), UME Radius
0.0000	6.0000E-6	0.13500	-7.7520E-10	NaN	2.0938E-4	0.0000	1.0758E-17	9.4716E-17	0.19999	0.0000	0.0000	4.5602E-6	1.0000
0.0000	6.0000E-6	0.14000	-7.7484E-10	NaN	2.0929E-4	0.0000	1.0225E-17	9.4709E-17	0.19989	0.0000	0.0000	4.5580E-6	1.0000
0.0000	6.0000E-6	0.14500	-7.7449E-10	NaN	2.0920E-4	0.0000	9.6927E-18	9.4702E-17	0.19980	0.0000	0.0000	4.5559E-6	1.0000
0.0000	6.0000E-6	0.15000	-7.7414E-10	NaN	2.0911E-4	0.0000	9.1600E-18	9.4696E-17	0.19971	0.0000	0.0000	4.5537E-6	1.0000
0.0000	6.0000E-6	0.15500	-7.7378E-10	NaN	2.0902E-4	0.0000	8.6273E-18	9.4689E-17	0.19962	0.0000	0.0000	4.5515E-6	1.0000
0.0000	6.0000E-6	0.16000	-7.7343E-10	NaN	2.0892E-4	0.0000	8.0946E-18	9.4682E-17	0.19952	0.0000	0.0000	4.5494E-6	1.0000
0.0000	6.0000E-6	0.16500	-7.7307E-10	NaN	2.0883E-4	0.0000	7.5619E-18	9.4675E-17	0.19943	0.0000	0.0000	4.5472E-6	1.0000
0.0000	6.0000E-6	0.17000	-7.7272E-10	NaN	2.0874E-4	0.0000	7.0292E-18	9.4668E-17	0.19934	0.0000	0.0000	4.5451E-6	1.0000
0.0000	6.0000E-6	0.17500	-7.7248E-10	NaN	2.0868E-4	0.0000	6.7387E-18	9.4659E-17	0.19928	0.0000	0.0000	4.5436E-6	1.0000
0.0000	6.0000E-6	0.18000	-7.7226E-	NaN	2.0862E-4	0.0000	6.4975E-18	9.4649E-17	0.19922	0.0000	0.0000	4.5423E-6	1.0000

k0Sub (m/s)	d (m)	Time (s)	-(tds2.ntflux_Oxi)*n*F (A), UME	Surface ConcOxi (M), OxiMem	Surface ConcOxi (M), OxiNuc	Substrate Kinetics (m/s), k0Sub probe	Normal total flux (mol/s), UME OCR membrane	Normal total flux (mol/s), UME OCR nucleus	Concentration (mol/m^3), Point: (5.5109E-16, 2.5)	k0Sub*Oxi (mol/s), Mem Flux	k0Sub*Oxi (mol/s), Nuc Flux	Normal total flux (mol/(m^2*s)), Point: (5.5109E-16, 2.5)	WElectrode radius (μm), UME Radius
			10										
0.0000	6.0000E-6	0.18500	-7.7205E-10	NaN	2.0857E-4	0.0000	6.2562E-18	9.4639E-17	0.19916	0.0000	0.0000	4.5410E-6	1.0000
0.0000	6.0000E-6	0.19000	-7.7184E-10	NaN	2.0851E-4	0.0000	6.0149E-18	9.4629E-17	0.19911	0.0000	0.0000	4.5397E-6	1.0000
0.0000	6.0000E-6	0.19500	-7.7162E-10	NaN	2.0845E-4	0.0000	5.7736E-18	9.4619E-17	0.19905	0.0000	0.0000	4.5384E-6	1.0000
0.0000	6.0000E-6	0.20000	-7.7141E-10	NaN	2.0840E-4	0.0000	5.5323E-18	9.4608E-17	0.19900	0.0000	0.0000	4.5371E-6	1.0000
0.0000	6.0000E-6	0.20500	-7.7120E-10	NaN	2.0834E-4	0.0000	5.2910E-18	9.4598E-17	0.19894	0.0000	0.0000	4.5358E-6	1.0000
0.0000	6.0000E-6	0.21000	-7.7098E-10	NaN	2.0829E-4	0.0000	5.0497E-18	9.4588E-17	0.19889	0.0000	0.0000	4.5345E-6	1.0000
0.0000	6.0000E-6	0.21500	-7.7077E-10	NaN	2.0823E-4	0.0000	4.8085E-18	9.4578E-17	0.19883	0.0000	0.0000	4.5332E-6	1.0000
0.0000	6.0000E-6	0.22000	-7.7055E-10	NaN	2.0817E-4	0.0000	4.5672E-18	9.4568E-17	0.19877	0.0000	0.0000	4.5320E-6	1.0000
0.0000	6.0000E-6	0.22500	-7.7034E-10	NaN	2.0812E-4	0.0000	4.3259E-18	9.4558E-17	0.19872	0.0000	0.0000	4.5307E-6	1.0000
0.00	6.0000E-	0.23	-7.70	NaN	2.0808E-	0.00	4.1953E-	9.4549E-	0.19	0.00	0.00	4.5298E-	1.00

k0Sub (m/s)	d (m)	Time (s)	-(tds2.ntflux_Oxi)*n*F (A), UME	Surface Concentration cOxi (M), Oxi Mem	Surface Concentration cOxi (M), OxiNuc	Substrate Kinetics (m/s), k0Sub probe	Normal total flux (mol/s), UME OCR membrane	Normal total flux (mol/s), UME OCR nucleus	Concentration (mol/m^3), Point: (5.5109E-16, 2.5)	k0Sub*Oxi (mol/s), Mem Flux	k0Sub*Oxi (mol/s), Nuc Flux	Normal total flux (mol/(m^2*s)), Point: (5.5109E-16, 2.5)	WEIectrode radius (µm), UME Radius
00	6	000	19E-10		4	00	18	17	868	00	00	6	00
0.0000	6.0000E-6	0.23500	-7.7007E-10	NaN	2.0805E-4	0.0000	4.0999E-18	9.4541E-17	0.19865	0.0000	0.0000	4.5291E-6	1.0000
0.0000	6.0000E-6	0.24000	-7.6996E-10	NaN	2.0802E-4	0.0000	4.0046E-18	9.4532E-17	0.19862	0.0000	0.0000	4.5283E-6	1.0000
0.0000	6.0000E-6	0.24500	-7.6984E-10	NaN	2.0798E-4	0.0000	3.9093E-18	9.4524E-17	0.19859	0.0000	0.0000	4.5276E-6	1.0000
0.0000	6.0000E-6	0.25000	-7.6972E-10	NaN	2.0795E-4	0.0000	3.8140E-18	9.4515E-17	0.19856	0.0000	0.0000	4.5269E-6	1.0000
0.0000	6.0000E-6	0.25000	-7.6972E-10	NaN	2.0795E-4	0.0000	3.8140E-18	9.4515E-17	0.19856	0.0000	0.0000	4.5269E-6	1.0000
0.0000	6.0000E-6	0.27000	-7.6924E-10	NaN	2.0783E-4	0.0000	3.4327E-18	9.4482E-17	0.19843	0.0000	0.0000	4.5241E-6	1.0000
0.0000	6.0000E-6	0.29000	-7.6876E-10	NaN	2.0770E-4	0.0000	3.0514E-18	9.4448E-17	0.19831	0.0000	0.0000	4.5212E-6	1.0000
0.0000	6.0000E-6	0.31000	-7.6829E-10	NaN	2.0757E-4	0.0000	2.6702E-18	9.4415E-17	0.19819	0.0000	0.0000	4.5183E-6	1.0000
0.0000	6.0000E-6	0.33000	-7.6785E-10	NaN	2.0746E-4	0.0000	2.3390E-18	9.4383E-17	0.19807	0.0000	0.0000	4.5157E-6	1.0000

k0Sub (m/s)	d (m)	Time (s)	-(tds2.ntflux_Oxi)*n*F (A), UME	Surface ConcOxi (M), OxiMem	Surface ConcOxi (M), OxiNuc	Substrate Kinetics (m/s), k0Sub probe	Normal total flux (mol/s), UME OCR membrane	Normal total flux (mol/s), UME OCR nucleus	Concentration (mol/m^3), Point: (5.5109E-16, 2.5)	k0Sub*Oxi (mol/s), MemFlux	k0Sub*Oxi (mol/s), NucFlux	Normal total flux (mol/(m^2*s)), Point: (5.5109E-16, 2.5)	WElectrode radius (μm), UME Radius
0.0000	6.0000E-6	0.35000	-7.6755E-10	NaN	2.0738E-4	0.0000	2.1641E-18	9.4357E-17	0.19800	0.0000	0.0000	4.5139E-6	1.0000
0.0000	6.0000E-6	0.37000	-7.6725E-10	NaN	2.0730E-4	0.0000	1.9891E-18	9.4332E-17	0.19792	0.0000	0.0000	4.5121E-6	1.0000
0.0000	6.0000E-6	0.39000	-7.6695E-10	NaN	2.0722E-4	0.0000	1.8142E-18	9.4306E-17	0.19784	0.0000	0.0000	4.5103E-6	1.0000
0.0000	6.0000E-6	0.41000	-7.6665E-10	NaN	2.0714E-4	0.0000	1.6392E-18	9.4281E-17	0.19776	0.0000	0.0000	4.5085E-6	1.0000
0.0000	6.0000E-6	0.43000	-7.6638E-10	NaN	2.0707E-4	0.0000	1.4887E-18	9.4257E-17	0.19769	0.0000	0.0000	4.5069E-6	1.0000
0.0000	6.0000E-6	0.45000	-7.6617E-10	NaN	2.0701E-4	0.0000	1.3957E-18	9.4237E-17	0.19764	0.0000	0.0000	4.5057E-6	1.0000
0.0000	6.0000E-6	0.47000	-7.6596E-10	NaN	2.0696E-4	0.0000	1.3027E-18	9.4218E-17	0.19758	0.0000	0.0000	4.5044E-6	1.0000
0.0000	6.0000E-6	0.49000	-7.6576E-10	NaN	2.0690E-4	0.0000	1.2097E-18	9.4198E-17	0.19753	0.0000	0.0000	4.5032E-6	1.0000
0.0000	6.0000E-6	0.51000	-7.6555E-10	NaN	2.0685E-4	0.0000	1.1167E-18	9.4179E-17	0.19748	0.0000	0.0000	4.5020E-6	1.0000
0.0000	6.0000E-6	0.53000	-7.6536E-	NaN	2.0680E-4	0.0000	1.0371E-18	9.4161E-17	0.19743	0.0000	0.0000	4.5008E-6	1.0000

k0Sub (m/s)	d (m)	Time (s)	-(tds2.ntflux_Oxi)*n*F (A), UME	Surface Con cOxi (M), Oxi Mem	Surface Con cOxi (M), OxiNuc	Substrate Kinetics (m/s), k0Sub probe	Normal total flux (mol/s), UME OCR membrane	Normal total flux (mol/s), UME OCR nucleus	Concentration (mol/m^3), Point: (5.5109E-16, 2.5)	k0Sub*Oxi (mol/s), Mem Flux	k0Sub*Oxi (mol/s), Nuc Flux	Normal total flux (mol/(m^2*s)), Point: (5.5109E-16, 2.5)	WElectrode radius (µm), UME Radius
			10										
0.0000	6.0000E-6	0.55000	-7.6521E-10	NaN	2.0675E-4	0.0000	9.8200E-19	9.4145E-17	0.19739	0.0000	0.0000	4.4999E-6	1.0000
0.0000	6.0000E-6	0.57000	-7.6505E-10	NaN	2.0671E-4	0.0000	9.2690E-19	9.4130E-17	0.19735	0.0000	0.0000	4.4990E-6	1.0000
0.0000	6.0000E-6	0.59000	-7.6490E-10	NaN	2.0667E-4	0.0000	8.7179E-19	9.4115E-17	0.19731	0.0000	0.0000	4.4981E-6	1.0000
0.0000	6.0000E-6	0.61000	-7.6475E-10	NaN	2.0663E-4	0.0000	8.1669E-19	9.4099E-17	0.19727	0.0000	0.0000	4.4972E-6	1.0000
0.0000	6.0000E-6	0.63000	-7.6461E-10	NaN	2.0659E-4	0.0000	7.6892E-19	9.4085E-17	0.19723	0.0000	0.0000	4.4964E-6	1.0000
0.0000	6.0000E-6	0.65000	-7.6449E-10	NaN	2.0656E-4	0.0000	7.3174E-19	9.4072E-17	0.19720	0.0000	0.0000	4.4956E-6	1.0000
0.0000	6.0000E-6	0.67000	-7.6436E-10	NaN	2.0653E-4	0.0000	6.9457E-19	9.4060E-17	0.19717	0.0000	0.0000	4.4949E-6	1.0000
0.0000	6.0000E-6	0.69000	-7.6424E-10	NaN	2.0650E-4	0.0000	6.5739E-19	9.4047E-17	0.19714	0.0000	0.0000	4.4942E-6	1.0000
0.0000	6.0000E-6	0.71000	-7.6412E-10	NaN	2.0646E-4	0.0000	6.2022E-19	9.4034E-17	0.19711	0.0000	0.0000	4.4935E-6	1.0000
0.00	6.0000E-	0.73	-7.64	NaN	2.0643E-	0.00	5.8884E-	9.4023E-	0.19	0.00	0.00	4.4928E-	1.00

k0Sub (m/s)	d (m)	Time (s)	-(tds2.ntflux_Oxi)*n*F (A), UME	Surface ConcOxi (M), OxiMem	Surface ConcOxi (M), OxiNuc	Substrate Kinetics (m/s), k0Sub probe	Normal total flux (mol/s), UME OCR membrane	Normal total flux (mol/s), UME OCR nucleus	Concentration (mol/m^3), Point: (5.5109E-16, 2.5)	k0Sub*Oxi (mol/s), Mem Flux	k0Sub*Oxi (mol/s), Nuc Flux	Normal total flux (mol/(m^2*s)), Point: (5.5109E-16, 2.5)	WEIectrode radius (µm), UME Radius
00	6	000	01E-10		4	00	19	17	708	00	00	6	00
0.0000	6.0000E-6	0.75000	-7.6391E-10	NaN	2.0641E-4	0.0000	5.6419E-19	9.4012E-17	0.19705	0.0000	0.0000	4.4922E-6	1.0000
0.0000	6.0000E-6	0.77000	-7.6382E-10	NaN	2.0638E-4	0.0000	5.3953E-19	9.4002E-17	0.19703	0.0000	0.0000	4.4917E-6	1.0000
0.0000	6.0000E-6	0.79000	-7.6372E-10	NaN	2.0636E-4	0.0000	5.1487E-19	9.3992E-17	0.19700	0.0000	0.0000	4.4911E-6	1.0000
0.0000	6.0000E-6	0.81000	-7.6362E-10	NaN	2.0633E-4	0.0000	4.9021E-19	9.3981E-17	0.19698	0.0000	0.0000	4.4905E-6	1.0000
0.0000	6.0000E-6	0.83000	-7.6353E-10	NaN	2.0630E-4	0.0000	4.6754E-19	9.3971E-17	0.19695	0.0000	0.0000	4.4900E-6	1.0000
0.0000	6.0000E-6	0.85000	-7.6344E-10	NaN	2.0628E-4	0.0000	4.4780E-19	9.3962E-17	0.19693	0.0000	0.0000	4.4895E-6	1.0000
0.0000	6.0000E-6	0.87000	-7.6336E-10	NaN	2.0626E-4	0.0000	4.2945E-19	9.3953E-17	0.19691	0.0000	0.0000	4.4890E-6	1.0000
0.0000	6.0000E-6	0.89000	-7.6328E-10	NaN	2.0624E-4	0.0000	4.1252E-19	9.3944E-17	0.19689	0.0000	0.0000	4.4885E-6	1.0000
0.0000	6.0000E-6	0.91000	-7.6321E-10	NaN	2.0622E-4	0.0000	3.9699E-19	9.3936E-17	0.19687	0.0000	0.0000	4.4881E-6	1.0000

k0Sub (m/s)	d (m)	Time (s)	-(tds2.ntflux_Oxi)*n*F (A), UME	Surface ConcOxi (M), OxiMem	Surface ConcOxi (M), OxiNuc	Substrate Kinetics (m/s), k0Sub probe	Normal total flux (mol/s), UME OCR membrane	Normal total flux (mol/s), UME OCR nucleus	Concentration (mol/m^3), Point: (5.5109E-16, 2.5)	k0Sub*Oxi (mol/s), MemFlux	k0Sub*Oxi (mol/s), NucFlux	Normal total flux (mol/(m^2*s)), Point: (5.5109E-16, 2.5)	WEIectrode radius (µm), UME Radius
0.0000	6.0000E-6	0.93000	-7.6313E-10	NaN	2.0620E-4	0.0000	3.8213E-19	9.3928E-17	0.19685	0.0000	0.0000	4.4876E-6	1.0000
0.0000	6.0000E-6	0.95000	-7.6306E-10	NaN	2.0618E-4	0.0000	3.6772E-19	9.3920E-17	0.19683	0.0000	0.0000	4.4872E-6	1.0000
0.0000	6.0000E-6	0.97000	-7.6299E-10	NaN	2.0616E-4	0.0000	3.5422E-19	9.3912E-17	0.19681	0.0000	0.0000	4.4868E-6	1.0000
0.0000	6.0000E-6	0.99000	-7.6292E-10	NaN	2.0614E-4	0.0000	3.4165E-19	9.3905E-17	0.19680	0.0000	0.0000	4.4864E-6	1.0000
0.0000	8.0000E-6	0.0000	-7.1631E-9	NaN	2.2700E-4	0.0000	2.4937E-29	-8.1952E-30	0.22700	0.0000	0.0000	-5.1223E-19	1.0000
0.0000	8.0000E-6	0.0050000	-8.5502E-10	NaN	2.2634E-4	0.0000	1.1825E-16	1.8663E-17	0.22471	0.0000	0.0000	1.6814E-6	1.0000
0.0000	8.0000E-6	0.010000	-8.3181E-10	NaN	2.2446E-4	0.0000	1.5320E-16	3.7562E-17	0.22118	0.0000	0.0000	2.2344E-6	1.0000
0.0000	8.0000E-6	0.015000	-8.2206E-10	NaN	2.2278E-4	0.0000	1.2866E-16	4.3864E-17	0.21884	0.0000	0.0000	2.2950E-6	1.0000
0.0000	8.0000E-6	0.020000	-8.1570E-10	NaN	2.2149E-4	0.0000	1.0712E-16	4.6902E-17	0.21720	0.0000	0.0000	2.3079E-6	1.0000
0.0000	8.0000E-6	0.025000	-8.1088E-	NaN	2.2043E-4	0.0000	8.9655E-17	4.8743E-17	0.21592	0.0000	0.0000	2.3069E-6	1.0000

k0Sub (m/s)	d (m)	Time (s)	-(tds2.ntflux_Oxi)*n*F (A), UME	Surface ConcOxi (M), OxiMem	Surface ConcOxi (M), OxiNuc	Substrate Kinetics (m/s), k0Sub probe	Normal total flux (mol/s), UME OCR membrane	Normal total flux (mol/s), UME OCR nucleus	Concentration (mol/m^3), Point: (5.5109E-16, 2.5)	k0Sub*Oxi (mol/s), MemFlux	k0Sub*Oxi (mol/s), NucFlux	Normal total flux (mol/(m^2*s)), Point: (5.5109E-16, 2.5)	WElectrode radius (μm), UME Radius
			10										
0.0000	8.0000E-6	0.030000	-8.0694E-10	NaN	2.1953E-4	0.0000	7.5389E-17	5.0060E-17	0.21485	0.0000	0.0000	2.3027E-6	1.0000
0.0000	8.0000E-6	0.035000	-8.0368E-10	NaN	2.1876E-4	0.0000	6.3752E-17	5.0973E-17	0.21396	0.0000	0.0000	2.2959E-6	1.0000
0.0000	8.0000E-6	0.040000	-8.0131E-10	NaN	2.1818E-4	0.0000	5.5757E-17	5.1519E-17	0.21330	0.0000	0.0000	2.2898E-6	1.0000
0.0000	8.0000E-6	0.045000	-7.9938E-10	NaN	2.1770E-4	0.0000	4.9747E-17	5.1879E-17	0.21277	0.0000	0.0000	2.2840E-6	1.0000
0.0000	8.0000E-6	0.050000	-7.9746E-10	NaN	2.1722E-4	0.0000	4.3736E-17	5.2239E-17	0.21224	0.0000	0.0000	2.2782E-6	1.0000
0.0000	8.0000E-6	0.055000	-7.9553E-10	NaN	2.1674E-4	0.0000	3.7726E-17	5.2599E-17	0.21170	0.0000	0.0000	2.2723E-6	1.0000
0.0000	8.0000E-6	0.060000	-7.9420E-10	NaN	2.1640E-4	0.0000	3.4026E-17	5.2783E-17	0.21134	0.0000	0.0000	2.2680E-6	1.0000
0.0000	8.0000E-6	0.065000	-7.9296E-10	NaN	2.1608E-4	0.0000	3.0754E-17	5.2939E-17	0.21099	0.0000	0.0000	2.2638E-6	1.0000
0.0000	8.0000E-6	0.070000	-7.9172E-10	NaN	2.1576E-4	0.0000	2.7483E-17	5.3095E-17	0.21065	0.0000	0.0000	2.2597E-6	1.0000
0.00	8.0000E-	0.07	-7.90	NaN	2.1545E-	0.00	2.4281E-	5.3245E-	0.21	0.00	0.00	2.2557E-	1.00

k0Sub (m/s)	d (m)	Time (s)	-(tds2.ntflux_Oxi)*n*F (A), UME	Surface ConcOxi (M), Oxi Mem	Surface ConcOxi (M), OxiNuc	Substrate Kinetics (m/s), k0Sub probe	Normal total flux (mol/s), UME OCR membrane	Normal total flux (mol/s), UME OCR nucleus	Concentration (mol/m^3), Point: (5.5109E-16, 2.5)	k0Sub*Oxi (mol/s), Mem Flux	k0Sub*Oxi (mol/s), Nuc Flux	Normal total flux (mol/(m^2*s)), Point: (5.5109E-16, 2.5)	WElectrode radius (µm), UME Radius
00	6	5000	51E-10		4	00	17	17	032	00	00	6	00
0.0000	8.0000E-6	0.080000	-7.8983E-10	NaN	2.1527E-4	0.0000	2.2869E-17	5.3295E-17	0.21013	0.0000	0.0000	2.2534E-6	1.0000
0.0000	8.0000E-6	0.085000	-7.8914E-10	NaN	2.1509E-4	0.0000	2.1457E-17	5.3344E-17	0.20994	0.0000	0.0000	2.2511E-6	1.0000
0.0000	8.0000E-6	0.090000	-7.8845E-10	NaN	2.1491E-4	0.0000	2.0045E-17	5.3394E-17	0.20975	0.0000	0.0000	2.2488E-6	1.0000
0.0000	8.0000E-6	0.095000	-7.8777E-10	NaN	2.1473E-4	0.0000	1.8633E-17	5.3444E-17	0.20956	0.0000	0.0000	2.2465E-6	1.0000
0.0000	8.0000E-6	0.10000	-7.8708E-10	NaN	2.1455E-4	0.0000	1.7221E-17	5.3493E-17	0.20938	0.0000	0.0000	2.2442E-6	1.0000
0.0000	8.0000E-6	0.10500	-7.8640E-10	NaN	2.1437E-4	0.0000	1.5809E-17	5.3543E-17	0.20919	0.0000	0.0000	2.2419E-6	1.0000
0.0000	8.0000E-6	0.11000	-7.8571E-10	NaN	2.1419E-4	0.0000	1.4397E-17	5.3593E-17	0.20900	0.0000	0.0000	2.2395E-6	1.0000
0.0000	8.0000E-6	0.11500	-7.8515E-10	NaN	2.1405E-4	0.0000	1.3301E-17	5.3626E-17	0.20884	0.0000	0.0000	2.2377E-6	1.0000
0.0000	8.0000E-6	0.12000	-7.8472E-10	NaN	2.1393E-4	0.0000	1.2627E-17	5.3641E-17	0.20873	0.0000	0.0000	2.2363E-6	1.0000

k0Sub (m/s)	d (m)	Time (s)	-(tds2.ntflux_Oxi)*n*F (A), UME	Surface ConcOxi (M), OxiMem	Surface ConcOxi (M), OxiNuc	Substrate Kinetics (m/s), k0Sub probe	Normal total flux (mol/s), UME OCR membrane	Normal total flux (mol/s), UME OCR nucleus	Concentration (mol/m^3), Point: (5.5109E-16, 2.5)	k0Sub*Oxi (mol/s), Mem Flux	k0Sub*Oxi (mol/s), Nuc Flux	Normal total flux (mol/(m^2*s)), Point: (5.5109E-16, 2.5)	WElectrode radius (µm), UME Radius
0.0000	8.0000E-6	0.12500	-7.8430E-10	NaN	2.1382E-4	0.0000	1.1953E-17	5.3657E-17	0.20861	0.0000	0.0000	2.2349E-6	1.0000
0.0000	8.0000E-6	0.13000	-7.8388E-10	NaN	2.1371E-4	0.0000	1.1279E-17	5.3672E-17	0.20850	0.0000	0.0000	2.2335E-6	1.0000
0.0000	8.0000E-6	0.13500	-7.8346E-10	NaN	2.1360E-4	0.0000	1.0605E-17	5.3687E-17	0.20838	0.0000	0.0000	2.2321E-6	1.0000
0.0000	8.0000E-6	0.14000	-7.8304E-10	NaN	2.1349E-4	0.0000	9.9306E-18	5.3702E-17	0.20827	0.0000	0.0000	2.2307E-6	1.0000
0.0000	8.0000E-6	0.14500	-7.8262E-10	NaN	2.1338E-4	0.0000	9.2564E-18	5.3717E-17	0.20815	0.0000	0.0000	2.2293E-6	1.0000
0.0000	8.0000E-6	0.15000	-7.8220E-10	NaN	2.1327E-4	0.0000	8.5822E-18	5.3732E-17	0.20804	0.0000	0.0000	2.2279E-6	1.0000
0.0000	8.0000E-6	0.15500	-7.8195E-10	NaN	2.1320E-4	0.0000	8.2463E-18	5.3735E-17	0.20797	0.0000	0.0000	2.2271E-6	1.0000
0.0000	8.0000E-6	0.16000	-7.8172E-10	NaN	2.1314E-4	0.0000	7.9864E-18	5.3736E-17	0.20791	0.0000	0.0000	2.2264E-6	1.0000
0.0000	8.0000E-6	0.16500	-7.8150E-10	NaN	2.1308E-4	0.0000	7.7265E-18	5.3737E-17	0.20785	0.0000	0.0000	2.2257E-6	1.0000
0.0000	8.0000E-6	0.17000	-7.8128E-	NaN	2.1302E-4	0.0000	7.4667E-18	5.3738E-17	0.20779	0.0000	0.0000	2.2250E-6	1.0000

k0Sub (m/s)	d (m)	Time (s)	-(tds2.ntflux_Oxi)*n*F (A), UME	Surface ConcOxi (M), Oxi Mem	Surface ConcOxi (M), OxiNuc	Substrate Kinetics (m/s), k0Sub probe	Normal total flux (mol/s), UME OCR membrane	Normal total flux (mol/s), UME OCR nucleus	Concentration (mol/m^3), Point: (5.5109E-16, 2.5)	k0Sub* Oxi (mol/s), Mem Flux	k0Sub* Oxi (mol/s), Nuc Flux	Normal total flux (mol/(m^2*s)), Point: (5.5109E-16, 2.5)	WElectrode radius (µm), UME Radius
			10										
0.0000	8.0000E-6	0.17500	-7.8105E-10	NaN	2.1296E-4	0.0000	7.2068E-18	5.3739E-17	0.20773	0.0000	0.0000	2.2242E-6	1.0000
0.0000	8.0000E-6	0.18000	-7.8083E-10	NaN	2.1290E-4	0.0000	6.9469E-18	5.3739E-17	0.20767	0.0000	0.0000	2.2235E-6	1.0000
0.0000	8.0000E-6	0.18500	-7.8060E-10	NaN	2.1284E-4	0.0000	6.6871E-18	5.3740E-17	0.20761	0.0000	0.0000	2.2228E-6	1.0000
0.0000	8.0000E-6	0.19000	-7.8038E-10	NaN	2.1278E-4	0.0000	6.4272E-18	5.3741E-17	0.20755	0.0000	0.0000	2.2221E-6	1.0000
0.0000	8.0000E-6	0.19500	-7.8015E-10	NaN	2.1272E-4	0.0000	6.1673E-18	5.3742E-17	0.20748	0.0000	0.0000	2.2214E-6	1.0000
0.0000	8.0000E-6	0.20000	-7.7993E-10	NaN	2.1266E-4	0.0000	5.9074E-18	5.3743E-17	0.20742	0.0000	0.0000	2.2207E-6	1.0000
0.0000	8.0000E-6	0.20500	-7.7970E-10	NaN	2.1260E-4	0.0000	5.6476E-18	5.3744E-17	0.20736	0.0000	0.0000	2.2199E-6	1.0000
0.0000	8.0000E-6	0.21000	-7.7948E-10	NaN	2.1254E-4	0.0000	5.3877E-18	5.3745E-17	0.20730	0.0000	0.0000	2.2192E-6	1.0000
0.0000	8.0000E-6	0.21500	-7.7926E-10	NaN	2.1248E-4	0.0000	5.1278E-18	5.3746E-17	0.20724	0.0000	0.0000	2.2185E-6	1.0000
0.00	8.0000E-	0.22	-7.79	NaN	2.1242E-	0.00	4.8680E-	5.3746E-	0.20	0.00	0.00	2.2178E-	1.00

k0Sub (m/s)	d (m)	Time (s)	-(tds2.ntflux_Oxi)*n*F (A), UME	Surface ConcOxi (M), Oxi Mem	Surface ConcOxi (M), OxiNuc	Substrate Kinetics (m/s), k0Sub probe	Normal total flux (mol/s), UME OCR membrane	Normal total flux (mol/s), UME OCR nucleus	Concentration (mol/m^3), Point: (5.5109E-16, 2.5)	k0Sub*Oxi (mol/s), Mem Flux	k0Sub*Oxi (mol/s), Nuc Flux	Normal total flux (mol/(m^2*s)), Point: (5.5109E-16, 2.5)	WElectrode radius (μm), UME Radius
00	6	000	03E-10		4	00	18	17	718	00	00	6	00
0.0000	8.0000E-6	0.22500	-7.7881E-10	NaN	2.1236E-4	0.0000	4.6081E-18	5.3747E-17	0.20712	0.0000	0.0000	2.2171E-6	1.0000
0.0000	8.0000E-6	0.23000	-7.7864E-10	NaN	2.1231E-4	0.0000	4.4351E-18	5.3746E-17	0.20708	0.0000	0.0000	2.2165E-6	1.0000
0.0000	8.0000E-6	0.23500	-7.7851E-10	NaN	2.1228E-4	0.0000	4.3203E-18	5.3744E-17	0.20704	0.0000	0.0000	2.2161E-6	1.0000
0.0000	8.0000E-6	0.24000	-7.7837E-10	NaN	2.1224E-4	0.0000	4.2055E-18	5.3742E-17	0.20700	0.0000	0.0000	2.2157E-6	1.0000
0.0000	8.0000E-6	0.24500	-7.7823E-10	NaN	2.1220E-4	0.0000	4.0907E-18	5.3739E-17	0.20697	0.0000	0.0000	2.2153E-6	1.0000
0.0000	8.0000E-6	0.25000	-7.7810E-10	NaN	2.1217E-4	0.0000	3.9759E-18	5.3737E-17	0.20693	0.0000	0.0000	2.2148E-6	1.0000
0.0000	8.0000E-6	0.25000	-7.7810E-10	NaN	2.1217E-4	0.0000	3.9759E-18	5.3737E-17	0.20693	0.0000	0.0000	2.2148E-6	1.0000
0.0000	8.0000E-6	0.27000	-7.7756E-10	NaN	2.1202E-4	0.0000	3.5166E-18	5.3728E-17	0.20678	0.0000	0.0000	2.2132E-6	1.0000
0.0000	8.0000E-6	0.29000	-7.7701E-10	NaN	2.1188E-4	0.0000	3.0574E-18	5.3718E-17	0.20664	0.0000	0.0000	2.2115E-6	1.0000

k0Sub (m/s)	d (m)	Time (s)	-(tds2.ntflux_Oxi)*n*F (A), UME	Surface ConcOxi (M), Oxi Mem	Surface ConcOxi (M), OxiNuc	Substrate Kinetics (m/s), k0Sub probe	Normal total flux (mol/s), UME OCR membrane	Normal total flux (mol/s), UME OCR nucleus	Concentration (mol/m^3), Point: (5.5109E-16, 2.5)	k0Sub*Oxi (mol/s), Mem Flux	k0Sub*Oxi (mol/s), Nuc Flux	Normal total flux (mol/(m^2*s)), Point: (5.5109E-16, 2.5)	WElectrode radius (µm), UME Radius
0.0000	8.0000E-6	0.31000	-7.7655E-10	NaN	2.1175E-4	0.0000	2.6840E-18	5.3708E-17	0.20651	0.0000	0.0000	2.2100E-6	1.0000
0.0000	8.0000E-6	0.33000	-7.7621E-10	NaN	2.1166E-4	0.0000	2.4746E-18	5.3698E-17	0.20642	0.0000	0.0000	2.2090E-6	1.0000
0.0000	8.0000E-6	0.35000	-7.7587E-10	NaN	2.1157E-4	0.0000	2.2652E-18	5.3687E-17	0.20633	0.0000	0.0000	2.2080E-6	1.0000
0.0000	8.0000E-6	0.37000	-7.7554E-10	NaN	2.1148E-4	0.0000	2.0559E-18	5.3676E-17	0.20624	0.0000	0.0000	2.2069E-6	1.0000
0.0000	8.0000E-6	0.39000	-7.7520E-10	NaN	2.1139E-4	0.0000	1.8465E-18	5.3666E-17	0.20615	0.0000	0.0000	2.2059E-6	1.0000
0.0000	8.0000E-6	0.41000	-7.7491E-10	NaN	2.1131E-4	0.0000	1.6761E-18	5.3656E-17	0.20607	0.0000	0.0000	2.2050E-6	1.0000
0.0000	8.0000E-6	0.43000	-7.7468E-10	NaN	2.1125E-4	0.0000	1.5671E-18	5.3646E-17	0.20601	0.0000	0.0000	2.2043E-6	1.0000
0.0000	8.0000E-6	0.45000	-7.7445E-10	NaN	2.1118E-4	0.0000	1.4580E-18	5.3637E-17	0.20595	0.0000	0.0000	2.2036E-6	1.0000
0.0000	8.0000E-6	0.47000	-7.7422E-10	NaN	2.1112E-4	0.0000	1.3490E-18	5.3627E-17	0.20589	0.0000	0.0000	2.2030E-6	1.0000
0.0000	8.0000E-6	0.49000	-7.7399E-	NaN	2.1106E-4	0.0000	1.2400E-18	5.3618E-17	0.20582	0.0000	0.0000	2.2023E-6	1.0000

k0Sub (m/s)	d (m)	Time (s)	-(tds2.ntflux_Oxi)*n*F (A), UME	Surface ConcOxi (M), Oxi Mem	Surface ConcOxi (M), OxiNuc	Substrate Kinetics (m/s), k0Sub probe	Normal total flux (mol/s), UME OCR membrane	Normal total flux (mol/s), UME OCR nucleus	Concentration (mol/m^3), Point: (5.5109E-16, 2.5)	k0Sub*Oxi (mol/s), Mem Flux	k0Sub*Oxi (mol/s), Nuc Flux	Normal total flux (mol/(m^2*s)), Point: (5.5109E-16, 2.5)	WElectrode radius (µm), UME Radius
			10										
0.0000	8.0000E-6	0.51000	-7.7379E-10	NaN	2.1100E-4	0.0000	1.1507E-18	5.3609E-17	0.20577	0.0000	0.0000	2.2017E-6	1.0000
0.0000	8.0000E-6	0.53000	-7.7362E-10	NaN	2.1096E-4	0.0000	1.0873E-18	5.3601E-17	0.20573	0.0000	0.0000	2.2012E-6	1.0000
0.0000	8.0000E-6	0.55000	-7.7346E-10	NaN	2.1091E-4	0.0000	1.0239E-18	5.3594E-17	0.20568	0.0000	0.0000	2.2007E-6	1.0000
0.0000	8.0000E-6	0.57000	-7.7329E-10	NaN	2.1087E-4	0.0000	9.6042E-19	5.3586E-17	0.20564	0.0000	0.0000	2.2002E-6	1.0000
0.0000	8.0000E-6	0.59000	-7.7312E-10	NaN	2.1082E-4	0.0000	8.9699E-19	5.3578E-17	0.20559	0.0000	0.0000	2.1997E-6	1.0000
0.0000	8.0000E-6	0.61000	-7.7297E-10	NaN	2.1078E-4	0.0000	8.4362E-19	5.3571E-17	0.20555	0.0000	0.0000	2.1992E-6	1.0000
0.0000	8.0000E-6	0.63000	-7.7284E-10	NaN	2.1075E-4	0.0000	8.0137E-19	5.3564E-17	0.20552	0.0000	0.0000	2.1988E-6	1.0000
0.0000	8.0000E-6	0.65000	-7.7271E-10	NaN	2.1071E-4	0.0000	7.5912E-19	5.3557E-17	0.20548	0.0000	0.0000	2.1984E-6	1.0000
0.0000	8.0000E-6	0.67000	-7.7258E-10	NaN	2.1068E-4	0.0000	7.1687E-19	5.3551E-17	0.20545	0.0000	0.0000	2.1981E-6	1.0000
0.00	8.0000E-	0.69	-7.72	NaN	2.1064E-	0.00	6.7462E-	5.3544E-	0.20	0.00	0.00	2.1977E-	1.00

k0Sub (m/s)	d (m)	Time (s)	-(tds2.ntflux_Oxi)*n*F (A), UME	Surface ConcOxi (M), OxiMem	Surface ConcOxi (M), OxiNuc	Substrate Kinetics (m/s), k0Sub probe	Normal total flux (mol/s), UME OCR membrane	Normal total flux (mol/s), UME OCR nucleus	Concentration (mol/m^3), Point: (5.5109E-16, 2.5)	k0Sub*Oxi (mol/s), Mem Flux	k0Sub*Oxi (mol/s), Nuc Flux	Normal total flux (mol/(m^2*s)), Point: (5.5109E-16, 2.5)	WElectrode radius (μm), UME Radius
00	6	000	44E-10		4	00	19	17	541	00	00	6	00
0.0000	8.0000E-6	0.71000	-7.7233E-10	NaN	2.1061E-4	0.0000	6.3996E-19	5.3538E-17	0.20538	0.0000	0.0000	2.1973E-6	1.0000
0.0000	8.0000E-6	0.73000	-7.7222E-10	NaN	2.1058E-4	0.0000	6.1233E-19	5.3532E-17	0.20535	0.0000	0.0000	2.1970E-6	1.0000
0.0000	8.0000E-6	0.75000	-7.7212E-10	NaN	2.1055E-4	0.0000	5.8470E-19	5.3527E-17	0.20532	0.0000	0.0000	2.1967E-6	1.0000
0.0000	8.0000E-6	0.77000	-7.7202E-10	NaN	2.1052E-4	0.0000	5.5706E-19	5.3521E-17	0.20530	0.0000	0.0000	2.1964E-6	1.0000
0.0000	8.0000E-6	0.79000	-7.7191E-10	NaN	2.1049E-4	0.0000	5.2943E-19	5.3515E-17	0.20527	0.0000	0.0000	2.1961E-6	1.0000
0.0000	8.0000E-6	0.81000	-7.7181E-10	NaN	2.1047E-4	0.0000	5.0432E-19	5.3510E-17	0.20524	0.0000	0.0000	2.1958E-6	1.0000
0.0000	8.0000E-6	0.83000	-7.7172E-10	NaN	2.1044E-4	0.0000	4.8244E-19	5.3505E-17	0.20522	0.0000	0.0000	2.1955E-6	1.0000
0.0000	8.0000E-6	0.85000	-7.7163E-10	NaN	2.1042E-4	0.0000	4.6219E-19	5.3500E-17	0.20519	0.0000	0.0000	2.1953E-6	1.0000
0.0000	8.0000E-6	0.87000	-7.7155E-10	NaN	2.1040E-4	0.0000	4.4356E-19	5.3495E-17	0.20517	0.0000	0.0000	2.1950E-6	1.0000

k0Sub (m/s)	d (m)	Time (s)	-(tds2.ntflux_Oxi)*n*F (A), UME	Surface ConcOxi (M), OxiMem	Surface ConcOxi (M), OxiNuc	Substrate Kinetics (m/s), k0Sub probe	Normal total flux (mol/s), UME OCR membrane	Normal total flux (mol/s), UME OCR nucleus	Concentration (mol/m^3), Point: (5.5109E-16, 2.5)	k0Sub*Oxi (mol/s), Mem Flux	k0Sub*Oxi (mol/s), Nuc Flux	Normal total flux (mol/(m^2*s)), Point: (5.5109E-16, 2.5)	WElectrode radius (µm), UME Radius
0.0000	8.0000E-6	0.89000	-7.7147E-10	NaN	2.1037E-4	0.0000	4.2656E-19	5.3491E-17	0.20515	0.0000	0.0000	2.1948E-6	1.0000
0.0000	8.0000E-6	0.91000	-7.7139E-10	NaN	2.1035E-4	0.0000	4.1015E-19	5.3486E-17	0.20513	0.0000	0.0000	2.1946E-6	1.0000
0.0000	8.0000E-6	0.93000	-7.7131E-10	NaN	2.1033E-4	0.0000	3.9431E-19	5.3482E-17	0.20511	0.0000	0.0000	2.1943E-6	1.0000
0.0000	8.0000E-6	0.95000	-7.7124E-10	NaN	2.1031E-4	0.0000	3.7951E-19	5.3477E-17	0.20509	0.0000	0.0000	2.1941E-6	1.0000
0.0000	8.0000E-6	0.97000	-7.7117E-10	NaN	2.1029E-4	0.0000	3.6576E-19	5.3473E-17	0.20507	0.0000	0.0000	2.1939E-6	1.0000
0.0000	8.0000E-6	0.99000	-7.7110E-10	NaN	2.1027E-4	0.0000	3.5306E-19	5.3469E-17	0.20505	0.0000	0.0000	2.1937E-6	1.0000
0.0000	1.2000E-5	0.0000	-7.1532E-9	NaN	2.2700E-4	0.0000	-5.0847E-30	-1.4058E-29	0.22700	0.0000	0.0000	-2.5611E-19	1.0000
0.0000	1.2000E-5	0.0050000	-8.5576E-10	NaN	2.2694E-4	0.0000	1.4960E-17	1.5953E-18	0.22681	0.0000	0.0000	1.7224E-7	1.0000
0.0000	1.2000E-5	0.010000	-8.3076E-10	NaN	2.2645E-4	0.0000	5.4413E-17	8.1176E-18	0.22580	0.0000	0.0000	5.4033E-7	1.0000
0.0000	1.2000E-5	0.015000	-8.2168E-	NaN	2.2565E-4	0.0000	6.4247E-17	1.2697E-17	0.22459	0.0000	0.0000	6.8612E-7	1.0000

k0Sub (m/s)	d (m)	Time (s)	-(tds2.ntflux_Oxi)*n*F (A), UME	Surface Con cOxi (M), Oxi Mem	Surface Con cOxi (M), OxiNuc	Substrate Kinetics (m/s), k0Sub probe	Normal total flux (mol/s), UME OCR membrane	Normal total flux (mol/s), UME OCR nucleus	Concentration (mol/m^3), Point: (5.5109E-16, 2.5)	k0Sub*Oxi (mol/s), Mem Flux	k0Sub*Oxi (mol/s), Nuc Flux	Normal total flux (mol/(m^2*s)), Point: (5.5109E-16, 2.5)	WEIectrode radius (µm), UME Radius
			10										
0.0000	1.2000E-5	0.020000	-8.1647E-10	NaN	2.2487E-4	0.0000	6.0872E-17	1.5120E-17	0.22356	0.0000	0.0000	7.3824E-7	1.0000
0.0000	1.2000E-5	0.025000	-8.1260E-10	NaN	2.2417E-4	0.0000	5.4990E-17	1.6642E-17	0.22270	0.0000	0.0000	7.6308E-7	1.0000
0.0000	1.2000E-5	0.030000	-8.1010E-10	NaN	2.2363E-4	0.0000	4.9780E-17	1.7433E-17	0.22207	0.0000	0.0000	7.7135E-7	1.0000
0.0000	1.2000E-5	0.035000	-8.0781E-10	NaN	2.2311E-4	0.0000	4.4612E-17	1.8114E-17	0.22147	0.0000	0.0000	7.7712E-7	1.0000
0.0000	1.2000E-5	0.040000	-8.0557E-10	NaN	2.2261E-4	0.0000	3.9599E-17	1.8767E-17	0.22089	0.0000	0.0000	7.8245E-7	1.0000
0.0000	1.2000E-5	0.045000	-8.0406E-10	NaN	2.2222E-4	0.0000	3.5876E-17	1.9100E-17	0.22046	0.0000	0.0000	7.8276E-7	1.0000
0.0000	1.2000E-5	0.050000	-8.0255E-10	NaN	2.2184E-4	0.0000	3.2154E-17	1.9433E-17	0.22004	0.0000	0.0000	7.8306E-7	1.0000
0.0000	1.2000E-5	0.055000	-8.0122E-10	NaN	2.2150E-4	0.0000	2.8979E-17	1.9707E-17	0.21966	0.0000	0.0000	7.8298E-7	1.0000
0.0000	1.2000E-5	0.060000	-8.0033E-10	NaN	2.2126E-4	0.0000	2.6987E-17	1.9837E-17	0.21940	0.0000	0.0000	7.8197E-7	1.0000
0.00	1.2000E-	0.06	-7.99	NaN	2.2101E-	0.00	2.4995E-	1.9967E-	0.21	0.00	0.00	7.8095E-	1.00

k0Sub (m/s)	d (m)	Time (s)	-(tds2.ntflux_Oxi)*n*F (A), UME	Surface ConcOxi (M), Oxi Mem	Surface ConcOxi (M), OxiNuc	Substrate Kinetics (m/s), k0Sub probe	Normal total flux (mol/s), UME OCR membrane	Normal total flux (mol/s), UME OCR nucleus	Concentration (mol/m^3), Point: (5.5109E-16, 2.5)	k0Sub*Oxi (mol/s), Mem Flux	k0Sub*Oxi (mol/s), Nuc Flux	Normal total flux (mol/(m^2*s)), Point: (5.5109E-16, 2.5)	WElectrode radius (µm), UME Radius
00	5	5000	44E-10		4	00	17	17	914	00	00	7	00
0.0000	1.2000E-5	0.070000	-7.9855E-10	NaN	2.2077E-4	0.0000	2.3003E-17	2.0097E-17	0.21888	0.0000	0.0000	7.7994E-7	1.0000
0.0000	1.2000E-5	0.075000	-7.9766E-10	NaN	2.2053E-4	0.0000	2.1012E-17	2.0226E-17	0.21862	0.0000	0.0000	7.7893E-7	1.0000
0.0000	1.2000E-5	0.080000	-7.9677E-10	NaN	2.2029E-4	0.0000	1.9020E-17	2.0356E-17	0.21836	0.0000	0.0000	7.7791E-7	1.0000
0.0000	1.2000E-5	0.085000	-7.9611E-10	NaN	2.2011E-4	0.0000	1.7691E-17	2.0434E-17	0.21817	0.0000	0.0000	7.7696E-7	1.0000
0.0000	1.2000E-5	0.090000	-7.9553E-10	NaN	2.1995E-4	0.0000	1.6593E-17	2.0491E-17	0.21800	0.0000	0.0000	7.7604E-7	1.0000
0.0000	1.2000E-5	0.095000	-7.9495E-10	NaN	2.1978E-4	0.0000	1.5495E-17	2.0548E-17	0.21783	0.0000	0.0000	7.7512E-7	1.0000
0.0000	1.2000E-5	0.10000	-7.9437E-10	NaN	2.1962E-4	0.0000	1.4396E-17	2.0605E-17	0.21765	0.0000	0.0000	7.7420E-7	1.0000
0.0000	1.2000E-5	0.10500	-7.9379E-10	NaN	2.1946E-4	0.0000	1.3298E-17	2.0662E-17	0.21748	0.0000	0.0000	7.7328E-7	1.0000
0.0000	1.2000E-5	0.11000	-7.9325E-10	NaN	2.1931E-4	0.0000	1.2307E-17	2.0713E-17	0.21733	0.0000	0.0000	7.7241E-7	1.0000

k0Sub (m/s)	d (m)	Time (s)	-(tds2.ntflux_Oxi)*n*F (A), UME	Surface ConcOxi (M), OxiMem	Surface ConcOxi (M), OxiNuc	Substrate Kinetics (m/s), k0Sub probe	Normal total flux (mol/s), UME OCR membrane	Normal total flux (mol/s), UME OCR nucleus	Concentration (mol/m^3), Point: (5.5109E-16, 2.5)	k0Sub*Oxi (mol/s), Mem Flux	k0Sub*Oxi (mol/s), Nuc Flux	Normal total flux (mol/(m^2*s)), Point: (5.5109E-16, 2.5)	WElectrode radius (μm), UME Radius
0.0000	1.2000E-5	0.11500	-7.9292E-10	NaN	2.1922E-4	0.0000	1.1828E-17	2.0733E-17	0.21723	0.0000	0.0000	7.7187E-7	1.0000
0.0000	1.2000E-5	0.12000	-7.9258E-10	NaN	2.1912E-4	0.0000	1.1350E-17	2.0752E-17	0.21713	0.0000	0.0000	7.7133E-7	1.0000
0.0000	1.2000E-5	0.12500	-7.9225E-10	NaN	2.1903E-4	0.0000	1.0872E-17	2.0772E-17	0.21704	0.0000	0.0000	7.7079E-7	1.0000
0.0000	1.2000E-5	0.13000	-7.9192E-10	NaN	2.1894E-4	0.0000	1.0393E-17	2.0792E-17	0.21694	0.0000	0.0000	7.7025E-7	1.0000
0.0000	1.2000E-5	0.13500	-7.9159E-10	NaN	2.1884E-4	0.0000	9.9152E-18	2.0811E-17	0.21684	0.0000	0.0000	7.6971E-7	1.0000
0.0000	1.2000E-5	0.14000	-7.9126E-10	NaN	2.1875E-4	0.0000	9.4369E-18	2.0831E-17	0.21675	0.0000	0.0000	7.6917E-7	1.0000
0.0000	1.2000E-5	0.14500	-7.9093E-10	NaN	2.1866E-4	0.0000	8.9586E-18	2.0850E-17	0.21665	0.0000	0.0000	7.6863E-7	1.0000
0.0000	1.2000E-5	0.15000	-7.9060E-10	NaN	2.1857E-4	0.0000	8.4803E-18	2.0870E-17	0.21656	0.0000	0.0000	7.6809E-7	1.0000
0.0000	1.2000E-5	0.15500	-7.9027E-10	NaN	2.1847E-4	0.0000	8.0020E-18	2.0890E-17	0.21646	0.0000	0.0000	7.6755E-7	1.0000
0.0000	1.2000E-5	0.16000	-7.8994E-	NaN	2.1838E-4	0.0000	7.5237E-18	2.0909E-17	0.21636	0.0000	0.0000	7.6701E-7	1.0000

k0Sub (m/s)	d (m)	Time (s)	-(tds2.ntflux_Oxi)*n*F (A), UME	Surface Con cOxi (M), Oxi Mem	Surface Con cOxi (M), OxiNuc	Substrate Kinetics (m/s), k0Sub probe	Normal total flux (mol/s), UME OCR membrane	Normal total flux (mol/s), UME OCR nucleus	Concentration (mol/m^3), Point: (5.5109E-16, 2.5)	k0Sub*Oxi (mol/s), Mem Flux	k0Sub*Oxi (mol/s), Nuc Flux	Normal total flux (mol/(m^2*s)), Point: (5.5109E-16, 2.5)	WElectrode radius (µm), UME Radius
			10										
0.0000	1.2000E-5	0.16500	-7.8961E-10	NaN	2.1829E-4	0.0000	7.0454E-18	2.0929E-17	0.21627	0.0000	0.0000	7.6647E-7	1.0000
0.0000	1.2000E-5	0.17000	-7.8940E-10	NaN	2.1823E-4	0.0000	6.8215E-18	2.0936E-17	0.21621	0.0000	0.0000	7.6615E-7	1.0000
0.0000	1.2000E-5	0.17500	-7.8919E-10	NaN	2.1817E-4	0.0000	6.5904E-18	2.0943E-17	0.21615	0.0000	0.0000	7.6582E-7	1.0000
0.0000	1.2000E-5	0.18000	-7.8898E-10	NaN	2.1811E-4	0.0000	6.3592E-18	2.0951E-17	0.21609	0.0000	0.0000	7.6550E-7	1.0000
0.0000	1.2000E-5	0.18500	-7.8877E-10	NaN	2.1805E-4	0.0000	6.1281E-18	2.0958E-17	0.21603	0.0000	0.0000	7.6518E-7	1.0000
0.0000	1.2000E-5	0.19000	-7.8856E-10	NaN	2.1799E-4	0.0000	5.8969E-18	2.0965E-17	0.21597	0.0000	0.0000	7.6485E-7	1.0000
0.0000	1.2000E-5	0.19500	-7.8835E-10	NaN	2.1793E-4	0.0000	5.6658E-18	2.0973E-17	0.21591	0.0000	0.0000	7.6453E-7	1.0000
0.0000	1.2000E-5	0.20000	-7.8814E-10	NaN	2.1787E-4	0.0000	5.4346E-18	2.0980E-17	0.21585	0.0000	0.0000	7.6421E-7	1.0000
0.0000	1.2000E-5	0.20500	-7.8793E-10	NaN	2.1782E-4	0.0000	5.2035E-18	2.0987E-17	0.21579	0.0000	0.0000	7.6388E-7	1.0000
0.00	1.2000E-	0.21	-7.87	NaN	2.1776E-	0.00	4.9723E-	2.0995E-	0.21	0.00	0.00	7.6356E-	1.00

k0Sub (m/s)	d (m)	Time (s)	-(tds2.ntflux_Oxi)*n*F (A), UME	Surface ConcOxi (M), OxiMem	Surface ConcOxi (M), OxiNuc	Substrate Kinetics (m/s), k0Sub probe	Normal total flux (mol/s), UME OCR membrane	Normal total flux (mol/s), UME OCR nucleus	Concentration (mol/m^3), Point: (5.5109E-16, 2.5)	k0Sub*Oxi (mol/s), Mem Flux	k0Sub*Oxi (mol/s), Nuc Flux	Normal total flux (mol/(m^2*s)), Point: (5.5109E-16, 2.5)	WEIectrode radius (µm), UME Radius
00	5	000	72E-10		4	00	18	17	573	00	00	7	00
0.0000	1.2000E-5	0.21500	-7.8752E-10	NaN	2.1770E-4	0.0000	4.7412E-18	2.1002E-17	0.21566	0.0000	0.0000	7.6324E-7	1.0000
0.0000	1.2000E-5	0.22000	-7.8731E-10	NaN	2.1764E-4	0.0000	4.5100E-18	2.1009E-17	0.21560	0.0000	0.0000	7.6291E-7	1.0000
0.0000	1.2000E-5	0.22500	-7.8717E-10	NaN	2.1760E-4	0.0000	4.3986E-18	2.1012E-17	0.21557	0.0000	0.0000	7.6272E-7	1.0000
0.0000	1.2000E-5	0.23000	-7.8705E-10	NaN	2.1757E-4	0.0000	4.3017E-18	2.1014E-17	0.21553	0.0000	0.0000	7.6254E-7	1.0000
0.0000	1.2000E-5	0.23500	-7.8693E-10	NaN	2.1753E-4	0.0000	4.2049E-18	2.1016E-17	0.21550	0.0000	0.0000	7.6237E-7	1.0000
0.0000	1.2000E-5	0.24000	-7.8681E-10	NaN	2.1750E-4	0.0000	4.1080E-18	2.1018E-17	0.21546	0.0000	0.0000	7.6220E-7	1.0000
0.0000	1.2000E-5	0.24500	-7.8669E-10	NaN	2.1747E-4	0.0000	4.0111E-18	2.1020E-17	0.21543	0.0000	0.0000	7.6203E-7	1.0000
0.0000	1.2000E-5	0.25000	-7.8657E-10	NaN	2.1743E-4	0.0000	3.9142E-18	2.1022E-17	0.21539	0.0000	0.0000	7.6186E-7	1.0000
0.0000	1.2000E-5	0.25000	-7.8657E-10	NaN	2.1743E-4	0.0000	3.9142E-18	2.1022E-17	0.21539	0.0000	0.0000	7.6186E-7	1.0000

k0Sub (m/s)	d (m)	Time (s)	-(tds2.ntflux_Oxi)*n*F (A), UME	Surface ConcOxi (M), OxiMem	Surface ConcOxi (M), OxiNuc	Substrate Kinetics (m/s), k0Sub probe	Normal total flux (mol/s), UME OCR membrane	Normal total flux (mol/s), UME OCR nucleus	Concentration (mol/m^3), Point: (5.5109E-16, 2.5)	k0Sub*Oxi (mol/s), Mem Flux	k0Sub*Oxi (mol/s), Nuc Flux	Normal total flux (mol/(m^2*s)), Point: (5.5109E-16, 2.5)	WElectrode radius (μm), UME Radius
0.0000	1.2000E-5	0.27000	-7.8608E-10	NaN	2.1730E-4	0.0000	3.5268E-18	2.1029E-17	0.21526	0.0000	0.0000	7.6117E-7	1.0000
0.0000	1.2000E-5	0.29000	-7.8560E-10	NaN	2.1716E-4	0.0000	3.1393E-18	2.1037E-17	0.21512	0.0000	0.0000	7.6049E-7	1.0000
0.0000	1.2000E-5	0.31000	-7.8511E-10	NaN	2.1702E-4	0.0000	2.7518E-18	2.1045E-17	0.21498	0.0000	0.0000	7.5980E-7	1.0000
0.0000	1.2000E-5	0.33000	-7.8472E-10	NaN	2.1691E-4	0.0000	2.4710E-18	2.1049E-17	0.21487	0.0000	0.0000	7.5926E-7	1.0000
0.0000	1.2000E-5	0.35000	-7.8441E-10	NaN	2.1683E-4	0.0000	2.2859E-18	2.1051E-17	0.21478	0.0000	0.0000	7.5885E-7	1.0000
0.0000	1.2000E-5	0.37000	-7.8410E-10	NaN	2.1674E-4	0.0000	2.1008E-18	2.1052E-17	0.21469	0.0000	0.0000	7.5844E-7	1.0000
0.0000	1.2000E-5	0.39000	-7.8379E-10	NaN	2.1665E-4	0.0000	1.9156E-18	2.1053E-17	0.21461	0.0000	0.0000	7.5803E-7	1.0000
0.0000	1.2000E-5	0.41000	-7.8348E-10	NaN	2.1657E-4	0.0000	1.7305E-18	2.1055E-17	0.21452	0.0000	0.0000	7.5762E-7	1.0000
0.0000	1.2000E-5	0.43000	-7.8322E-10	NaN	2.1649E-4	0.0000	1.5946E-18	2.1055E-17	0.21445	0.0000	0.0000	7.5729E-7	1.0000
0.0000	1.2000E-5	0.45000	-7.8300E-	NaN	2.1643E-4	0.0000	1.4941E-18	2.1054E-17	0.21439	0.0000	0.0000	7.5702E-7	1.0000

k0Sub (m/s)	d (m)	Time (s)	-(tds2.ntflux_Oxi)*n*F (A), UME	Surface ConcOxi (M), OxiMem	Surface ConcOxi (M), OxiNuc	Substrate Kinetics (m/s), k0Sub probe	Normal total flux (mol/s), UME OCR membrane	Normal total flux (mol/s), UME OCR nucleus	Concentration (mol/m^3), Point: (5.5109E-16, 2.5)	k0Sub*Oxi (mol/s), MemFlux	k0Sub*Oxi (mol/s), NucFlux	Normal total flux (mol/(m^2*s)), Point: (5.5109E-16, 2.5)	WEIectrode radius (μm), UME Radius
			10										
0.0000	1.2000E-5	0.47000	-7.8278E-10	NaN	2.1637E-4	0.0000	1.3935E-18	2.1053E-17	0.21433	0.0000	0.0000	7.5675E-7	1.0000
0.0000	1.2000E-5	0.49000	-7.8257E-10	NaN	2.1631E-4	0.0000	1.2930E-18	2.1053E-17	0.21427	0.0000	0.0000	7.5648E-7	1.0000
0.0000	1.2000E-5	0.51000	-7.8235E-10	NaN	2.1625E-4	0.0000	1.1924E-18	2.1052E-17	0.21420	0.0000	0.0000	7.5621E-7	1.0000
0.0000	1.2000E-5	0.53000	-7.8217E-10	NaN	2.1620E-4	0.0000	1.1176E-18	2.1051E-17	0.21415	0.0000	0.0000	7.5599E-7	1.0000
0.0000	1.2000E-5	0.55000	-7.8201E-10	NaN	2.1616E-4	0.0000	1.0574E-18	2.1050E-17	0.21411	0.0000	0.0000	7.5580E-7	1.0000
0.0000	1.2000E-5	0.57000	-7.8185E-10	NaN	2.1611E-4	0.0000	9.9718E-19	2.1048E-17	0.21406	0.0000	0.0000	7.5561E-7	1.0000
0.0000	1.2000E-5	0.59000	-7.8168E-10	NaN	2.1607E-4	0.0000	9.3696E-19	2.1047E-17	0.21402	0.0000	0.0000	7.5542E-7	1.0000
0.0000	1.2000E-5	0.61000	-7.8152E-10	NaN	2.1602E-4	0.0000	8.7674E-19	2.1046E-17	0.21397	0.0000	0.0000	7.5522E-7	1.0000
0.0000	1.2000E-5	0.63000	-7.8138E-10	NaN	2.1598E-4	0.0000	8.3003E-19	2.1044E-17	0.21394	0.0000	0.0000	7.5506E-7	1.0000
0.00	1.2000E-	0.65	-7.81	NaN	2.1595E-	0.00	7.8924E-	2.1043E-	0.21	0.00	0.00	7.5491E-	1.00

k0Sub (m/s)	d (m)	Time (s)	-(tds2.ntflux_Oxi)*n*F (A), UME	Surface ConcOxi (M), Oxi Mem	Surface ConcOxi (M), OxiNuc	Substrate Kinetics (m/s), k0Sub probe	Normal total flux (mol/s), UME OCR membrane	Normal total flux (mol/s), UME OCR nucleus	Concentration (mol/m^3), Point: (5.5109E-16, 2.5)	k0Sub*Oxi (mol/s), Mem Flux	k0Sub*Oxi (mol/s), Nuc Flux	Normal total flux (mol/(m^2*s)), Point: (5.5109E-16, 2.5)	WEIectrode radius (µm), UME Radius
00	5	000	26E-10		4	00	19	17	390	00	00	7	00
0.0000	1.2000E-5	0.67000	-7.8113E-10	NaN	2.1591E-4	0.0000	7.4844E-19	2.1042E-17	0.21386	0.0000	0.0000	7.5476E-7	1.0000
0.0000	1.2000E-5	0.69000	-7.8100E-10	NaN	2.1588E-4	0.0000	7.0765E-19	2.1040E-17	0.21383	0.0000	0.0000	7.5461E-7	1.0000
0.0000	1.2000E-5	0.71000	-7.8087E-10	NaN	2.1584E-4	0.0000	6.6686E-19	2.1039E-17	0.21379	0.0000	0.0000	7.5447E-7	1.0000
0.0000	1.2000E-5	0.73000	-7.8076E-10	NaN	2.1581E-4	0.0000	6.3632E-19	2.1037E-17	0.21376	0.0000	0.0000	7.5434E-7	1.0000
0.0000	1.2000E-5	0.75000	-7.8066E-10	NaN	2.1578E-4	0.0000	6.0919E-19	2.1036E-17	0.21373	0.0000	0.0000	7.5423E-7	1.0000
0.0000	1.2000E-5	0.77000	-7.8056E-10	NaN	2.1575E-4	0.0000	5.8206E-19	2.1035E-17	0.21371	0.0000	0.0000	7.5411E-7	1.0000
0.0000	1.2000E-5	0.79000	-7.8045E-10	NaN	2.1573E-4	0.0000	5.5493E-19	2.1033E-17	0.21368	0.0000	0.0000	7.5399E-7	1.0000
0.0000	1.2000E-5	0.81000	-7.8035E-10	NaN	2.1570E-4	0.0000	5.2780E-19	2.1032E-17	0.21365	0.0000	0.0000	7.5388E-7	1.0000
0.0000	1.2000E-5	0.83000	-7.8026E-10	NaN	2.1567E-4	0.0000	5.0425E-19	2.1030E-17	0.21362	0.0000	0.0000	7.5377E-7	1.0000

k0Sub (m/s)	d (m)	Time (s)	-(tds2.ntflux_Oxi)*n*F (A), UME	Surface ConcOxi (M), OxiMem	Surface ConcOxi (M), OxiNuc	Substrate Kinetics (m/s), k0Sub probe	Normal total flux (mol/s), UME OCR membrane	Normal total flux (mol/s), UME OCR nucleus	Concentration (mol/m^3), Point: (5.5109E-16, 2.5)	k0Sub*Oxi (mol/s), Mem Flux	k0Sub*Oxi (mol/s), Nuc Flux	Normal total flux (mol/(m^2*s)), Point: (5.5109E-16, 2.5)	WElectrode radius (µm), UME Radius
0.0000	1.2000E-5	0.85000	-7.8017E-10	NaN	2.1565E-4	0.0000	4.8295E-19	2.1029E-17	0.21360	0.0000	0.0000	7.5367E-7	1.0000
0.0000	1.2000E-5	0.87000	-7.8008E-10	NaN	2.1562E-4	0.0000	4.6319E-19	2.1028E-17	0.21357	0.0000	0.0000	7.5358E-7	1.0000
0.0000	1.2000E-5	0.89000	-7.8000E-10	NaN	2.1560E-4	0.0000	4.4498E-19	2.1026E-17	0.21355	0.0000	0.0000	7.5349E-7	1.0000
0.0000	1.2000E-5	0.91000	-7.7992E-10	NaN	2.1558E-4	0.0000	4.2831E-19	2.1025E-17	0.21353	0.0000	0.0000	7.5340E-7	1.0000
0.0000	1.2000E-5	0.93000	-7.7984E-10	NaN	2.1555E-4	0.0000	4.1192E-19	2.1024E-17	0.21351	0.0000	0.0000	7.5331E-7	1.0000
0.0000	1.2000E-5	0.95000	-7.7976E-10	NaN	2.1553E-4	0.0000	3.9635E-19	2.1023E-17	0.21349	0.0000	0.0000	7.5323E-7	1.0000
0.0000	1.2000E-5	0.97000	-7.7969E-10	NaN	2.1551E-4	0.0000	3.8180E-19	2.1021E-17	0.21347	0.0000	0.0000	7.5315E-7	1.0000
0.0000	1.2000E-5	0.99000	-7.7962E-10	NaN	2.1549E-4	0.0000	3.6828E-19	2.1020E-17	0.21345	0.0000	0.0000	7.5308E-7	1.0000
0.0000	1.5000E-5	0.0000	-7.1540E-9	NaN	2.2700E-4	0.0000	4.4771E-30	-6.7638E-30	0.22700	0.0000	0.0000	-5.1223E-19	1.0000
0.0000	1.5000E-5	0.0050000	-8.5579E-	NaN	2.2699E-4	0.0000	2.2177E-18	1.9817E-19	0.22698	0.0000	0.0000	2.3463E-8	1.0000

k0Sub (m/s)	d (m)	Time (s)	-(tds2.ntflux_Oxi)*n*F (A), UME	Surface ConcOxi (M), OxiMem	Surface ConcOxi (M), OxiNuc	Substrate Kinetics (m/s), k0Sub probe	Normal total flux (mol/s), UME OCR membrane	Normal total flux (mol/s), UME OCR nucleus	Concentration (mol/m^3), Point: (5.5109E-16, 2.5)	k0Sub*Oxi (mol/s), Mem Flux	k0Sub*Oxi (mol/s), Nuc Flux	Normal total flux (mol/(m^2*s)), Point: (5.5109E-16, 2.5)	WElectrode radius (µm), UME Radius
			10										
0.0000	1.5000E-5	0.010000	-8.3080E-10	NaN	2.2685E-4	0.0000	1.8457E-17	2.2245E-18	0.22667	0.0000	0.0000	1.6289E-7	1.0000
0.0000	1.5000E-5	0.015000	-8.2173E-10	NaN	2.2647E-4	0.0000	3.1314E-17	4.8042E-18	0.22609	0.0000	0.0000	2.7847E-7	1.0000
0.0000	1.5000E-5	0.020000	-8.1660E-10	NaN	2.2602E-4	0.0000	3.5283E-17	6.5866E-18	0.22547	0.0000	0.0000	3.3883E-7	1.0000
0.0000	1.5000E-5	0.025000	-8.1294E-10	NaN	2.2556E-4	0.0000	3.5239E-17	7.7942E-18	0.22490	0.0000	0.0000	3.7216E-7	1.0000
0.0000	1.5000E-5	0.030000	-8.1058E-10	NaN	2.2517E-4	0.0000	3.3515E-17	8.4857E-18	0.22443	0.0000	0.0000	3.8714E-7	1.0000
0.0000	1.5000E-5	0.035000	-8.0850E-10	NaN	2.2479E-4	0.0000	3.1425E-17	9.0731E-18	0.22399	0.0000	0.0000	3.9842E-7	1.0000
0.0000	1.5000E-5	0.040000	-8.0643E-10	NaN	2.2441E-4	0.0000	2.9355E-17	9.6537E-18	0.22355	0.0000	0.0000	4.0951E-7	1.0000
0.0000	1.5000E-5	0.045000	-8.0510E-10	NaN	2.2410E-4	0.0000	2.7225E-17	9.9558E-18	0.22321	0.0000	0.0000	4.1293E-7	1.0000
0.0000	1.5000E-5	0.050000	-8.0377E-10	NaN	2.2380E-4	0.0000	2.5094E-17	1.0258E-17	0.22287	0.0000	0.0000	4.1635E-7	1.0000
0.00	1.5000E-	0.05	-8.02	NaN	2.2352E-	0.00	2.3153E-	1.0521E-	0.22	0.00	0.00	4.1908E-	1.00

k0Sub (m/s)	d (m)	Time (s)	-(tds2.ntflux_Oxi)*n*F (A), UME	Surface ConcOxi (M), OxiMem	Surface ConcOxi (M), OxiNuc	Substrate Kinetics (m/s), k0Sub probe	Normal total flux (mol/s), UME OCR membrane	Normal total flux (mol/s), UME OCR nucleus	Concentration (mol/m^3), Point: (5.5109E-16, 2.5)	k0Sub*Oxi (mol/s), Mem Flux	k0Sub*Oxi (mol/s), Nuc Flux	Normal total flux (mol/(m^2*s)), Point: (5.5109E-16, 2.5)	WEIectrode radius (µm), UME Radius
00	5	5000	56E-10		4	00	17	17	256	00	00	7	00
0.0000	1.5000E-5	0.060000	-8.0180E-10	NaN	2.2331E-4	0.0000	2.1767E-17	1.0643E-17	0.22234	0.0000	0.0000	4.1931E-7	1.0000
0.0000	1.5000E-5	0.065000	-8.0103E-10	NaN	2.2311E-4	0.0000	2.0380E-17	1.0764E-17	0.22212	0.0000	0.0000	4.1955E-7	1.0000
0.0000	1.5000E-5	0.070000	-8.0027E-10	NaN	2.2290E-4	0.0000	1.8994E-17	1.0886E-17	0.22190	0.0000	0.0000	4.1978E-7	1.0000
0.0000	1.5000E-5	0.075000	-7.9950E-10	NaN	2.2270E-4	0.0000	1.7608E-17	1.1008E-17	0.22168	0.0000	0.0000	4.2002E-7	1.0000
0.0000	1.5000E-5	0.080000	-7.9874E-10	NaN	2.2249E-4	0.0000	1.6221E-17	1.1130E-17	0.22146	0.0000	0.0000	4.2025E-7	1.0000
0.0000	1.5000E-5	0.085000	-7.9813E-10	NaN	2.2233E-4	0.0000	1.5176E-17	1.1211E-17	0.22128	0.0000	0.0000	4.2013E-7	1.0000
0.0000	1.5000E-5	0.090000	-7.9763E-10	NaN	2.2218E-4	0.0000	1.4328E-17	1.1266E-17	0.22113	0.0000	0.0000	4.1976E-7	1.0000
0.0000	1.5000E-5	0.095000	-7.9713E-10	NaN	2.2204E-4	0.0000	1.3480E-17	1.1321E-17	0.22098	0.0000	0.0000	4.1939E-7	1.0000
0.0000	1.5000E-5	0.10000	-7.9663E-10	NaN	2.2190E-4	0.0000	1.2631E-17	1.1376E-17	0.22083	0.0000	0.0000	4.1902E-7	1.0000

k0Sub (m/s)	d (m)	Time (s)	-(tds2.ntflux_Oxi)*n*F (A), UME	Surface ConcOxi (M), OxiMem	Surface ConcOxi (M), OxiNuc	Substrate Kinetics (m/s), k0Sub probe	Normal total flux (mol/s), UME OCR membrane	Normal total flux (mol/s), UME OCR nucleus	Concentration (mol/m^3), Point: (5.5109E-16, 2.5)	k0Sub*Oxi (mol/s), Mem Flux	k0Sub*Oxi (mol/s), Nuc Flux	Normal total flux (mol/(m^2*s)), Point: (5.5109E-16, 2.5)	WElectrode radius (μm), UME Radius
0.0000	1.5000E-5	0.10500	-7.9613E-10	NaN	2.2176E-4	0.0000	1.1783E-17	1.1430E-17	0.22068	0.0000	0.0000	4.1865E-7	1.0000
0.0000	1.5000E-5	0.11000	-7.9563E-10	NaN	2.2161E-4	0.0000	1.0935E-17	1.1485E-17	0.22053	0.0000	0.0000	4.1828E-7	1.0000
0.0000	1.5000E-5	0.11500	-7.9534E-10	NaN	2.2153E-4	0.0000	1.0547E-17	1.1506E-17	0.22044	0.0000	0.0000	4.1795E-7	1.0000
0.0000	1.5000E-5	0.12000	-7.9504E-10	NaN	2.2144E-4	0.0000	1.0150E-17	1.1526E-17	0.22035	0.0000	0.0000	4.1763E-7	1.0000
0.0000	1.5000E-5	0.12500	-7.9475E-10	NaN	2.2136E-4	0.0000	9.7519E-18	1.1546E-17	0.22026	0.0000	0.0000	4.1731E-7	1.0000
0.0000	1.5000E-5	0.13000	-7.9446E-10	NaN	2.2127E-4	0.0000	9.3543E-18	1.1565E-17	0.22017	0.0000	0.0000	4.1698E-7	1.0000
0.0000	1.5000E-5	0.13500	-7.9417E-10	NaN	2.2119E-4	0.0000	8.9566E-18	1.1585E-17	0.22008	0.0000	0.0000	4.1666E-7	1.0000
0.0000	1.5000E-5	0.14000	-7.9387E-10	NaN	2.2110E-4	0.0000	8.5590E-18	1.1605E-17	0.21999	0.0000	0.0000	4.1634E-7	1.0000
0.0000	1.5000E-5	0.14500	-7.9358E-10	NaN	2.2102E-4	0.0000	8.1614E-18	1.1625E-17	0.21991	0.0000	0.0000	4.1601E-7	1.0000
0.0000	1.5000E-5	0.15000	-7.9329E-	NaN	2.2093E-4	0.0000	7.7638E-18	1.1645E-17	0.21982	0.0000	0.0000	4.1569E-7	1.0000

k0Sub (m/s)	d (m)	Time (s)	-(tds2.ntflux_Oxi)*n*F (A), UME	Surface ConcOxi (M), Oxi Mem	Surface ConcOxi (M), OxiNuc	Substrate Kinetics (m/s), k0Sub probe	Normal total flux (mol/s), UME OCR membrane	Normal total flux (mol/s), UME OCR nucleus	Concentration (mol/m^3), Point: (5.5109E-16, 2.5)	k0Sub*Oxi (mol/s), Mem Flux	k0Sub*Oxi (mol/s), Nuc Flux	Normal total flux (mol/(m^2*s)), Point: (5.5109E-16, 2.5)	WElectrode radius (µm), UME Radius
			10										
0.0000	1.5000E-5	0.15500	-7.9299E-10	NaN	2.2085E-4	0.0000	7.3662E-18	1.1665E-17	0.21973	0.0000	0.0000	4.1536E-7	1.0000
0.0000	1.5000E-5	0.16000	-7.9270E-10	NaN	2.2076E-4	0.0000	6.9686E-18	1.1685E-17	0.21964	0.0000	0.0000	4.1504E-7	1.0000
0.0000	1.5000E-5	0.16500	-7.9241E-10	NaN	2.2068E-4	0.0000	6.5709E-18	1.1705E-17	0.21955	0.0000	0.0000	4.1472E-7	1.0000
0.0000	1.5000E-5	0.17000	-7.9219E-10	NaN	2.2061E-4	0.0000	6.3298E-18	1.1716E-17	0.21949	0.0000	0.0000	4.1448E-7	1.0000
0.0000	1.5000E-5	0.17500	-7.9200E-10	NaN	2.2056E-4	0.0000	6.1281E-18	1.1724E-17	0.21943	0.0000	0.0000	4.1426E-7	1.0000
0.0000	1.5000E-5	0.18000	-7.9181E-10	NaN	2.2050E-4	0.0000	5.9264E-18	1.1732E-17	0.21938	0.0000	0.0000	4.1405E-7	1.0000
0.0000	1.5000E-5	0.18500	-7.9162E-10	NaN	2.2045E-4	0.0000	5.7248E-18	1.1741E-17	0.21932	0.0000	0.0000	4.1383E-7	1.0000
0.0000	1.5000E-5	0.19000	-7.9143E-10	NaN	2.2039E-4	0.0000	5.5231E-18	1.1749E-17	0.21926	0.0000	0.0000	4.1362E-7	1.0000
0.0000	1.5000E-5	0.19500	-7.9124E-10	NaN	2.2034E-4	0.0000	5.3214E-18	1.1757E-17	0.21920	0.0000	0.0000	4.1340E-7	1.0000
0.00	1.5000E-	0.20	-7.91	NaN	2.2028E-	0.00	5.1198E-	1.1765E-	0.21	0.00	0.00	4.1319E-	1.00

k0Sub (m/s)	d (m)	Time (s)	-(tds2.ntflux_Oxi)*n*F (A), UME	Surface ConcOxi (M), Oxi Mem	Surface ConcOxi (M), OxiNuc	Substrate Kinetics (m/s), k0Sub probe	Normal total flux (mol/s), UME OCR membrane	Normal total flux (mol/s), UME OCR nucleus	Concentration (mol/m^3), Point: (5.5109E-16, 2.5)	k0Sub*Oxi (mol/s), Mem Flux	k0Sub*Oxi (mol/s), Nuc Flux	Normal total flux (mol/(m^2*s)), Point: (5.5109E-16, 2.5)	WElectrode radius (µm), UME Radius
00	5	000	05E-10		4	00	18	17	915	00	00	7	00
0.0000	1.5000E-5	0.20500	-7.9086E-10	NaN	2.2023E-4	0.0000	4.9181E-18	1.1773E-17	0.21909	0.0000	0.0000	4.1297E-7	1.0000
0.0000	1.5000E-5	0.21000	-7.9068E-10	NaN	2.2017E-4	0.0000	4.7164E-18	1.1782E-17	0.21903	0.0000	0.0000	4.1276E-7	1.0000
0.0000	1.5000E-5	0.21500	-7.9049E-10	NaN	2.2011E-4	0.0000	4.5148E-18	1.1790E-17	0.21898	0.0000	0.0000	4.1254E-7	1.0000
0.0000	1.5000E-5	0.22000	-7.9030E-10	NaN	2.2006E-4	0.0000	4.3131E-18	1.1798E-17	0.21892	0.0000	0.0000	4.1233E-7	1.0000
0.0000	1.5000E-5	0.22500	-7.9015E-10	NaN	2.2002E-4	0.0000	4.1774E-18	1.1804E-17	0.21888	0.0000	0.0000	4.1216E-7	1.0000
0.0000	1.5000E-5	0.23000	-7.9003E-10	NaN	2.1998E-4	0.0000	4.0892E-18	1.1806E-17	0.21884	0.0000	0.0000	4.1205E-7	1.0000
0.0000	1.5000E-5	0.23500	-7.8992E-10	NaN	2.1995E-4	0.0000	4.0011E-18	1.1809E-17	0.21881	0.0000	0.0000	4.1193E-7	1.0000
0.0000	1.5000E-5	0.24000	-7.8981E-10	NaN	2.1992E-4	0.0000	3.9129E-18	1.1812E-17	0.21878	0.0000	0.0000	4.1182E-7	1.0000
0.0000	1.5000E-5	0.24500	-7.8969E-10	NaN	2.1988E-4	0.0000	3.8248E-18	1.1815E-17	0.21874	0.0000	0.0000	4.1170E-7	1.0000

k0Sub (m/s)	d (m)	Time (s)	-(tds2.ntflux_Oxi)*n*F (A), UME	Surface ConcOxi (M), OxiMem	Surface ConcOxi (M), OxiNuc	Substrate Kinetics (m/s), k0Sub probe	Normal total flux (mol/s), UME OCR membrane	Normal total flux (mol/s), UME OCR nucleus	Concentration (mol/m^3), Point: (5.5109E-16, 2.5)	k0Sub* Oxi (mol/s), Mem Flux	k0Sub* Oxi (mol/s), Nuc Flux	Normal total flux (mol/(m^2*s)), Point: (5.5109E-16, 2.5)	WElectrode radius (µm), UME Radius
0.0000	1.5000E-5	0.25000	-7.8958E-10	NaN	2.1985E-4	0.0000	3.7366E-18	1.1818E-17	0.21871	0.0000	0.0000	4.1158E-7	1.0000
0.0000	1.5000E-5	0.25000	-7.8958E-10	NaN	2.1985E-4	0.0000	3.7366E-18	1.1818E-17	0.21871	0.0000	0.0000	4.1158E-7	1.0000
0.0000	1.5000E-5	0.27000	-7.8913E-10	NaN	2.1972E-4	0.0000	3.3840E-18	1.1829E-17	0.21858	0.0000	0.0000	4.1112E-7	1.0000
0.0000	1.5000E-5	0.29000	-7.8867E-10	NaN	2.1959E-4	0.0000	3.0313E-18	1.1840E-17	0.21844	0.0000	0.0000	4.1066E-7	1.0000
0.0000	1.5000E-5	0.31000	-7.8822E-10	NaN	2.1946E-4	0.0000	2.6787E-18	1.1851E-17	0.21831	0.0000	0.0000	4.1019E-7	1.0000
0.0000	1.5000E-5	0.33000	-7.8784E-10	NaN	2.1935E-4	0.0000	2.4064E-18	1.1860E-17	0.21820	0.0000	0.0000	4.0981E-7	1.0000
0.0000	1.5000E-5	0.35000	-7.8754E-10	NaN	2.1926E-4	0.0000	2.2325E-18	1.1864E-17	0.21811	0.0000	0.0000	4.0953E-7	1.0000
0.0000	1.5000E-5	0.37000	-7.8724E-10	NaN	2.1918E-4	0.0000	2.0586E-18	1.1868E-17	0.21803	0.0000	0.0000	4.0926E-7	1.0000
0.0000	1.5000E-5	0.39000	-7.8695E-10	NaN	2.1909E-4	0.0000	1.8846E-18	1.1872E-17	0.21794	0.0000	0.0000	4.0898E-7	1.0000
0.0000	1.5000E-5	0.41000	-7.8665E-	NaN	2.1901E-4	0.0000	1.7107E-18	1.1876E-17	0.21785	0.0000	0.0000	4.0871E-7	1.0000

k0Sub (m/s)	d (m)	Time (s)	-(tds2.ntflux_Oxi)*n*F (A), UME	Surface ConcOxi (M), Oxi Mem	Surface ConcOxi (M), OxiNuc	Substrate Kinetics (m/s), k0Sub probe	Normal total flux (mol/s), UME OCR membrane	Normal total flux (mol/s), UME OCR nucleus	Concentration (mol/m^3), Point: (5.5109E-16, 2.5)	k0Sub*Oxi (mol/s), Mem Flux	k0Sub*Oxi (mol/s), Nuc Flux	Normal total flux (mol/(m^2*s)), Point: (5.5109E-16, 2.5)	WElectrode radius (µm), UME Radius
			10										
0.0000	1.5000E-5	0.43000	-7.8639E-10	NaN	2.1893E-4	0.0000	1.5759E-18	1.1879E-17	0.21778	0.0000	0.0000	4.0848E-7	1.0000
0.0000	1.5000E-5	0.45000	-7.8618E-10	NaN	2.1887E-4	0.0000	1.4793E-18	1.1880E-17	0.21772	0.0000	0.0000	4.0830E-7	1.0000
0.0000	1.5000E-5	0.47000	-7.8597E-10	NaN	2.1881E-4	0.0000	1.3828E-18	1.1882E-17	0.21766	0.0000	0.0000	4.0813E-7	1.0000
0.0000	1.5000E-5	0.49000	-7.8576E-10	NaN	2.1875E-4	0.0000	1.2863E-18	1.1883E-17	0.21760	0.0000	0.0000	4.0795E-7	1.0000
0.0000	1.5000E-5	0.51000	-7.8555E-10	NaN	2.1869E-4	0.0000	1.1897E-18	1.1885E-17	0.21754	0.0000	0.0000	4.0777E-7	1.0000
0.0000	1.5000E-5	0.53000	-7.8537E-10	NaN	2.1864E-4	0.0000	1.1145E-18	1.1886E-17	0.21749	0.0000	0.0000	4.0762E-7	1.0000
0.0000	1.5000E-5	0.55000	-7.8521E-10	NaN	2.1860E-4	0.0000	1.0558E-18	1.1886E-17	0.21744	0.0000	0.0000	4.0750E-7	1.0000
0.0000	1.5000E-5	0.57000	-7.8505E-10	NaN	2.1855E-4	0.0000	9.9712E-19	1.1886E-17	0.21740	0.0000	0.0000	4.0737E-7	1.0000
0.0000	1.5000E-5	0.59000	-7.8490E-10	NaN	2.1851E-4	0.0000	9.3845E-19	1.1887E-17	0.21735	0.0000	0.0000	4.0725E-7	1.0000
0.00	1.5000E-	0.61	-7.84	NaN	2.1846E-	0.00	8.7979E-	1.1887E-	0.21	0.00	0.00	4.0712E-	1.00

k0Sub (m/s)	d (m)	Time (s)	-(tds2.ntflux_Oxi)*n*F (A), UME	Surface ConcOxi (M), OxiMem	Surface ConcOxi (M), OxiNuc	Substrate Kinetics (m/s), k0Sub probe	Normal total flux (mol/s), UME OCR membrane	Normal total flux (mol/s), UME OCR nucleus	Concentration (mol/m^3), Point: (5.5109E-16, 2.5)	k0Sub*Oxi (mol/s), Mem Flux	k0Sub*Oxi (mol/s), Nuc Flux	Normal total flux (mol/(m^2*s)), Point: (5.5109E-16, 2.5)	WEIectrode radius (µm), UME Radius
00	5	000	74E-10		4	00	19	17	731	00	00	7	00
0.0000	1.5000E-5	0.63000	-7.8460E-10	NaN	2.1842E-4	0.0000	8.3260E-19	1.1887E-17	0.21727	0.0000	0.0000	4.0701E-7	1.0000
0.0000	1.5000E-5	0.65000	-7.8447E-10	NaN	2.1839E-4	0.0000	7.9250E-19	1.1887E-17	0.21723	0.0000	0.0000	4.0692E-7	1.0000
0.0000	1.5000E-5	0.67000	-7.8435E-10	NaN	2.1835E-4	0.0000	7.5241E-19	1.1887E-17	0.21719	0.0000	0.0000	4.0682E-7	1.0000
0.0000	1.5000E-5	0.69000	-7.8422E-10	NaN	2.1832E-4	0.0000	7.1232E-19	1.1887E-17	0.21716	0.0000	0.0000	4.0673E-7	1.0000
0.0000	1.5000E-5	0.71000	-7.8410E-10	NaN	2.1828E-4	0.0000	6.7223E-19	1.1887E-17	0.21712	0.0000	0.0000	4.0663E-7	1.0000
0.0000	1.5000E-5	0.73000	-7.8399E-10	NaN	2.1825E-4	0.0000	6.4102E-19	1.1887E-17	0.21709	0.0000	0.0000	4.0655E-7	1.0000
0.0000	1.5000E-5	0.75000	-7.8388E-10	NaN	2.1822E-4	0.0000	6.1412E-19	1.1887E-17	0.21706	0.0000	0.0000	4.0648E-7	1.0000
0.0000	1.5000E-5	0.77000	-7.8378E-10	NaN	2.1819E-4	0.0000	5.8722E-19	1.1887E-17	0.21703	0.0000	0.0000	4.0641E-7	1.0000
0.0000	1.5000E-5	0.79000	-7.8368E-10	NaN	2.1816E-4	0.0000	5.6032E-19	1.1886E-17	0.21701	0.0000	0.0000	4.0634E-7	1.0000

k0Sub (m/s)	d (m)	Time (s)	-(tds2.ntflux_Oxi)*n*F (A), UME	Surface ConcOxi (M), OxiMem	Surface ConcOxi (M), OxiNuc	Substrate Kinetics (m/s), k0Sub probe	Normal total flux (mol/s), UME OCR membrane	Normal total flux (mol/s), UME OCR nucleus	Concentration (mol/m^3), Point: (5.5109E-16, 2.5)	k0Sub*Oxi (mol/s), Mem Flux	k0Sub*Oxi (mol/s), Nuc Flux	Normal total flux (mol/(m^2*s)), Point: (5.5109E-16, 2.5)	WElectrode radius (µm), UME Radius
0.0000	1.5000E-5	0.81000	-7.8358E-10	NaN	2.1813E-4	0.0000	5.3342E-19	1.1886E-17	0.21698	0.0000	0.0000	4.0626E-7	1.0000
0.0000	1.5000E-5	0.83000	-7.8349E-10	NaN	2.1811E-4	0.0000	5.0962E-19	1.1886E-17	0.21695	0.0000	0.0000	4.0620E-7	1.0000
0.0000	1.5000E-5	0.85000	-7.8340E-10	NaN	2.1808E-4	0.0000	4.8828E-19	1.1885E-17	0.21692	0.0000	0.0000	4.0613E-7	1.0000
0.0000	1.5000E-5	0.87000	-7.8331E-10	NaN	2.1806E-4	0.0000	4.6844E-19	1.1885E-17	0.21690	0.0000	0.0000	4.0608E-7	1.0000
0.0000	1.5000E-5	0.89000	-7.8323E-10	NaN	2.1803E-4	0.0000	4.5011E-19	1.1884E-17	0.21688	0.0000	0.0000	4.0602E-7	1.0000
0.0000	1.5000E-5	0.91000	-7.8315E-10	NaN	2.1801E-4	0.0000	4.3327E-19	1.1884E-17	0.21685	0.0000	0.0000	4.0596E-7	1.0000
0.0000	1.5000E-5	0.93000	-7.8307E-10	NaN	2.1799E-4	0.0000	4.1688E-19	1.1884E-17	0.21683	0.0000	0.0000	4.0591E-7	1.0000
0.0000	1.5000E-5	0.95000	-7.8300E-10	NaN	2.1797E-4	0.0000	4.0121E-19	1.1883E-17	0.21681	0.0000	0.0000	4.0586E-7	1.0000
0.0000	1.5000E-5	0.97000	-7.8292E-10	NaN	2.1795E-4	0.0000	3.8656E-19	1.1883E-17	0.21679	0.0000	0.0000	4.0581E-7	1.0000
0.0000	1.5000E-5	0.99000	-7.8285E-	NaN	2.1793E-4	0.0000	3.7291E-19	1.1882E-17	0.21677	0.0000	0.0000	4.0577E-7	1.0000

k0Sub (m/s)	d (m)	Time (s)	-(tds2.ntflux_Oxi)*n*F (A), UME	Surface ConcOxi (M), OxiMem	Surface ConcOxi (M), OxiNuc	Substrate Kinetics (m/s), k0Sub probe	Normal total flux (mol/s), UME OCR membrane	Normal total flux (mol/s), UME OCR nucleus	Concentration (mol/m^3), Point: (5.5109E-16, 2.5)	k0Sub*Oxi (mol/s), MemFlux	k0Sub*Oxi (mol/s), NucFlux	Normal total flux (mol/(m^2*s)), Point: (5.5109E-16, 2.5)	WElectrode radius (µm), UME Radius
			10										
1.5100E-9	5.0000E-7	0.0000	-7.1493E-9	NaN	2.2700E-4	1.5100E-9	3.5276E-18	-1.1292E-20	0.22697	1.0088E-19	2.0267E-20	9.5099E-7	1.0000
1.5100E-9	5.0000E-7	0.005000 0	-7.4819E-10	NaN	2.0657E-4	1.5100E-9	8.6904E-16	7.0679E-16	0.062312	9.3684E-20	1.6560E-20	2.2781E-4	1.0000
1.5100E-9	5.0000E-7	0.010000	-7.0196E-10	NaN	1.9858E-4	1.5100E-9	4.5892E-16	7.0444E-16	0.058401	9.0327E-20	1.5652E-20	2.1349E-4	1.0000
1.5100E-9	5.0000E-7	0.015000	-6.7909E-10	NaN	1.9390E-4	1.5100E-9	2.7181E-16	6.9541E-16	0.056479	8.8303E-20	1.5179E-20	2.0645E-4	1.0000
1.5100E-9	5.0000E-7	0.020000	-6.6597E-10	NaN	1.9100E-4	1.5100E-9	1.7637E-16	6.8827E-16	0.055380	8.7035E-20	1.4903E-20	2.0243E-4	1.0000
1.5100E-9	5.0000E-7	0.025000	-6.5729E-10	NaN	1.8904E-4	1.5100E-9	1.1875E-16	6.8325E-16	0.054653	8.6172E-20	1.4719E-20	1.9977E-4	1.0000
1.5100E-9	5.0000E-7	0.030000	-6.5123E-10	NaN	1.8765E-4	1.5100E-9	8.1402E-17	6.7959E-16	0.054146	8.5558E-20	1.4590E-20	1.9791E-4	1.0000
1.5100E-9	5.0000E-7	0.035000	-6.4706E-10	NaN	1.8667E-4	1.5100E-9	5.8321E-17	6.7687E-16	0.053797	8.5122E-20	1.4501E-20	1.9664E-4	1.0000
1.5100E-9	5.0000E-7	0.040000	-6.4375E-10	NaN	1.8588E-4	1.5100E-9	4.0836E-17	6.7462E-16	0.053520	8.4770E-20	1.4430E-20	1.9563E-4	1.0000
1.5100E-9	5.0000E-7	0.04	-6.41	NaN	1.8526E-	1.5100E-	2.8566E-	6.7283E-	0.05	8.4496E-	1.4376E-	1.9486E-	1.00

k0Sub (m/s)	d (m)	Time (s)	-(tds2.ntflux_Oxi)*n*F (A), UME	Surface ConcOxi (M), OxiMem	Surface ConcOxi (M), OxiNuc	Substrate Kinetics (m/s), k0Sub probe	Normal total flux (mol/s), UME OCR membrane	Normal total flux (mol/s), UME OCR nucleus	Concentration (mol/m^3), Point: (5.5109E-16, 2.5)	k0Sub*Oxi (mol/s), Mem Flux	k0Sub*Oxi (mol/s), Nuc Flux	Normal total flux (mol/(m^2*s)), Point: (5.5109E-16, 2.5)	WElectrode radius (µm), UME Radius
9	7	5000	23E-10		4	9	17	16	3309	20	20	4	00
1.5100E-9	5.0000E-7	0.050000	-6.3967E-10	NaN	1.8486E-4	1.5100E-9	2.2598E-17	6.7158E-16	0.053179	8.4318E-20	1.4342E-20	1.9438E-4	1.0000
1.5100E-9	5.0000E-7	0.055000	-6.3881E-10	NaN	1.8463E-4	1.5100E-9	2.0527E-17	6.7080E-16	0.053107	8.4214E-20	1.4323E-20	1.9412E-4	1.0000
1.5100E-9	5.0000E-7	0.060000	-6.3795E-10	NaN	1.8440E-4	1.5100E-9	1.8457E-17	6.7002E-16	0.053036	8.4109E-20	1.4304E-20	1.9386E-4	1.0000
1.5100E-9	5.0000E-7	0.065000	-6.3720E-10	NaN	1.8420E-4	1.5100E-9	1.6719E-17	6.6934E-16	0.052973	8.4019E-20	1.4288E-20	1.9363E-4	1.0000
1.5100E-9	5.0000E-7	0.070000	-6.3655E-10	NaN	1.8403E-4	1.5100E-9	1.5238E-17	6.6875E-16	0.052920	8.3940E-20	1.4273E-20	1.9343E-4	1.0000
1.5100E-9	5.0000E-7	0.075000	-6.3591E-10	NaN	1.8385E-4	1.5100E-9	1.3757E-17	6.6816E-16	0.052866	8.3861E-20	1.4259E-20	1.9324E-4	1.0000
1.5100E-9	5.0000E-7	0.080000	-6.3545E-10	NaN	1.8373E-4	1.5100E-9	1.2810E-17	6.6774E-16	0.052828	8.3805E-20	1.4249E-20	1.9310E-4	1.0000
1.5100E-9	5.0000E-7	0.085000	-6.3503E-10	NaN	1.8361E-4	1.5100E-9	1.1936E-17	6.6735E-16	0.052793	8.3754E-20	1.4240E-20	1.9297E-4	1.0000
1.5100E-9	5.0000E-7	0.090000	-6.3461E-10	NaN	1.8350E-4	1.5100E-9	1.1062E-17	6.6696E-16	0.052758	8.3702E-20	1.4230E-20	1.9284E-4	1.0000

k0Sub (m/s)	d (m)	Time (s)	-(tds2.ntflux_Oxi)*n*F (A), UME	Surface ConcOxi (M), OxiMem	Surface ConcOxi (M), OxiNuc	Substrate Kinetics (m/s), k0Sub probe	Normal total flux (mol/s), UME OCR membrane	Normal total flux (mol/s), UME OCR nucleus	Concentration (mol/m^3), Point: (5.5109E-16, 2.5)	k0Sub*Oxi (mol/s), Mem Flux	k0Sub*Oxi (mol/s), Nuc Flux	Normal total flux (mol/(m^2*s)), Point: (5.5109E-16, 2.5)	WElectrode radius (µm), UME Radius
1.5100E-9	5.0000E-7	0.095000	-6.3419E-10	NaN	1.8338E-4	1.5100E-9	1.0188E-17	6.6657E-16	0.052722	8.3650E-20	1.4221E-20	1.9271E-4	1.0000
1.5100E-9	5.0000E-7	0.10000	-6.3376E-10	NaN	1.8327E-4	1.5100E-9	9.3134E-18	6.6618E-16	0.052687	8.3598E-20	1.4212E-20	1.9258E-4	1.0000
1.5100E-9	5.0000E-7	0.10500	-6.3343E-10	NaN	1.8318E-4	1.5100E-9	8.6955E-18	6.6587E-16	0.052660	8.3557E-20	1.4204E-20	1.9248E-4	1.0000
1.5100E-9	5.0000E-7	0.11000	-6.3320E-10	NaN	1.8312E-4	1.5100E-9	8.3210E-18	6.6565E-16	0.052640	8.3528E-20	1.4199E-20	1.9241E-4	1.0000
1.5100E-9	5.0000E-7	0.11500	-6.3297E-10	NaN	1.8305E-4	1.5100E-9	7.9464E-18	6.6543E-16	0.052621	8.3500E-20	1.4194E-20	1.9234E-4	1.0000
1.5100E-9	5.0000E-7	0.12000	-6.3274E-10	NaN	1.8299E-4	1.5100E-9	7.5719E-18	6.6521E-16	0.052602	8.3471E-20	1.4189E-20	1.9227E-4	1.0000
1.5100E-9	5.0000E-7	0.12500	-6.3251E-10	NaN	1.8292E-4	1.5100E-9	7.1973E-18	6.6499E-16	0.052583	8.3442E-20	1.4184E-20	1.9220E-4	1.0000
1.5100E-9	5.0000E-7	0.13000	-6.3228E-10	NaN	1.8286E-4	1.5100E-9	6.8228E-18	6.6477E-16	0.052563	8.3413E-20	1.4179E-20	1.9213E-4	1.0000
1.5100E-9	5.0000E-7	0.13500	-6.3205E-10	NaN	1.8280E-4	1.5100E-9	6.4483E-18	6.6455E-16	0.052544	8.3384E-20	1.4174E-20	1.9206E-4	1.0000
1.5100E-9	5.0000E-7	0.14000	-6.3181E-	NaN	1.8273E-4	1.5100E-9	6.0737E-18	6.6433E-16	0.052525	8.3355E-20	1.4168E-20	1.9199E-4	1.0000

k0Sub (m/s)	d (m)	Time (s)	-(tds2.ntflux_Oxi)*n*F (A), UME	Surface Con cOxi (M), Oxi Mem	Surface Con cOxi (M), OxiNuc	Substrate Kinetics (m/s), k0Sub probe	Normal total flux (mol/s), UME OCR membrane	Normal total flux (mol/s), UME OCR nucleus	Concentration (mol/m^3), Point: (5.5109E-16, 2.5)	k0Sub*Oxi (mol/s), Mem Flux	k0Sub*Oxi (mol/s), Nuc Flux	Normal total flux (mol/(m^2*s)), Point: (5.5109E-16, 2.5)	WElectrode radius (µm), UME Radius
			10										
1.5100E-9	5.0000E-7	0.14500	-6.3158E-10	NaN	1.8267E-4	1.5100E-9	5.6992E-18	6.6411E-16	0.052506	8.3327E-20	1.4163E-20	1.9192E-4	1.0000
1.5100E-9	5.0000E-7	0.15000	-6.3135E-10	NaN	1.8261E-4	1.5100E-9	5.3247E-18	6.6389E-16	0.052486	8.3298E-20	1.4158E-20	1.9185E-4	1.0000
1.5100E-9	5.0000E-7	0.15500	-6.3112E-10	NaN	1.8254E-4	1.5100E-9	4.9501E-18	6.6367E-16	0.052467	8.3269E-20	1.4153E-20	1.9178E-4	1.0000
1.5100E-9	5.0000E-7	0.16000	-6.3098E-10	NaN	1.8250E-4	1.5100E-9	4.7706E-18	6.6353E-16	0.052455	8.3251E-20	1.4150E-20	1.9173E-4	1.0000
1.5100E-9	5.0000E-7	0.16500	-6.3086E-10	NaN	1.8247E-4	1.5100E-9	4.6446E-18	6.6342E-16	0.052446	8.3236E-20	1.4147E-20	1.9170E-4	1.0000
1.5100E-9	5.0000E-7	0.17000	-6.3075E-10	NaN	1.8244E-4	1.5100E-9	4.5186E-18	6.6331E-16	0.052436	8.3222E-20	1.4145E-20	1.9166E-4	1.0000
1.5100E-9	5.0000E-7	0.17500	-6.3064E-10	NaN	1.8241E-4	1.5100E-9	4.3925E-18	6.6320E-16	0.052427	8.3208E-20	1.4142E-20	1.9163E-4	1.0000
1.5100E-9	5.0000E-7	0.18000	-6.3053E-10	NaN	1.8238E-4	1.5100E-9	4.2665E-18	6.6309E-16	0.052418	8.3193E-20	1.4140E-20	1.9160E-4	1.0000
1.5100E-9	5.0000E-7	0.18500	-6.3041E-10	NaN	1.8235E-4	1.5100E-9	4.1405E-18	6.6298E-16	0.052408	8.3179E-20	1.4137E-20	1.9156E-4	1.0000
1.5100E-	5.0000E-	0.19	-6.30	NaN	1.8231E-	1.5100E-	4.0145E-	6.6286E-	0.05	8.3165E-	1.4135E-	1.9153E-	1.00

k0Sub (m/s)	d (m)	Time (s)	-(tds2.ntflux_Oxi)*n*F (A), UME	Surface ConcOxi (M), Oxi Mem	Surface ConcOxi (M), OxiNuc	Substrate Kinetics (m/s), k0Sub probe	Normal total flux (mol/s), UME OCR membrane	Normal total flux (mol/s), UME OCR nucleus	Concentration (mol/m^3), Point: (5.5109E-16, 2.5)	k0Sub*Oxi (mol/s), Mem Flux	k0Sub*Oxi (mol/s), Nuc Flux	Normal total flux (mol/(m^2*s)), Point: (5.5109E-16, 2.5)	WElectrode radius (µm), UME Radius
9	7	000	30E-10		4	9	18	16	2399	20	20	4	00
1.5100E-9	5.0000E-7	0.19500	-6.3019E-10	NaN	1.8228E-4	1.5100E-9	3.8885E-18	6.6275E-16	0.052389	8.3150E-20	1.4132E-20	1.9149E-4	1.0000
1.5100E-9	5.0000E-7	0.20000	-6.3007E-10	NaN	1.8225E-4	1.5100E-9	3.7625E-18	6.6264E-16	0.052380	8.3136E-20	1.4130E-20	1.9146E-4	1.0000
1.5100E-9	5.0000E-7	0.20500	-6.2996E-10	NaN	1.8222E-4	1.5100E-9	3.6365E-18	6.6253E-16	0.052371	8.3122E-20	1.4127E-20	1.9142E-4	1.0000
1.5100E-9	5.0000E-7	0.21000	-6.2985E-10	NaN	1.8219E-4	1.5100E-9	3.5105E-18	6.6242E-16	0.052361	8.3107E-20	1.4125E-20	1.9139E-4	1.0000
1.5100E-9	5.0000E-7	0.21500	-6.2973E-10	NaN	1.8216E-4	1.5100E-9	3.3845E-18	6.6231E-16	0.052352	8.3093E-20	1.4122E-20	1.9136E-4	1.0000
1.5100E-9	5.0000E-7	0.22000	-6.2962E-10	NaN	1.8212E-4	1.5100E-9	3.2585E-18	6.6220E-16	0.052342	8.3079E-20	1.4120E-20	1.9132E-4	1.0000
1.5100E-9	5.0000E-7	0.22500	-6.2951E-10	NaN	1.8209E-4	1.5100E-9	3.1325E-18	6.6209E-16	0.052333	8.3064E-20	1.4117E-20	1.9129E-4	1.0000
1.5100E-9	5.0000E-7	0.23000	-6.2940E-10	NaN	1.8206E-4	1.5100E-9	3.0065E-18	6.6198E-16	0.052324	8.3050E-20	1.4115E-20	1.9125E-4	1.0000
1.5100E-9	5.0000E-7	0.23500	-6.2928E-10	NaN	1.8203E-4	1.5100E-9	2.8805E-18	6.6186E-16	0.052314	8.3035E-20	1.4112E-20	1.9122E-4	1.0000

k0Sub (m/s)	d (m)	Time (s)	-(tds2.ntflux_Oxi)*n*F (A), UME	Surface Con cOxi (M), Oxi Mem	Surface Con cOxi (M), OxiNuc	Substrate Kinetics (m/s), k0Sub probe	Normal total flux (mol/s), UME OCR membrane	Normal total flux (mol/s), UME OCR nucleus	Concentration (mol/m^3), Point: (5.5109E-16, 2.5)	k0Sub* Oxi (mol/s), Mem Flux	k0Sub* Oxi (mol/s), Nuc Flux	Normal total flux (mol/(m^2*s)), Point: (5.5109E-16, 2.5)	WEIectrode radius (µm), UME Radius
1.5100E-9	5.0000E-7	0.24000	-6.2917E-10	NaN	1.8200E-4	1.5100E-9	2.7545E-18	6.6175E-16	0.052305	8.3021E-20	1.4110E-20	1.9118E-4	1.0000
1.5100E-9	5.0000E-7	0.24500	-6.2906E-10	NaN	1.8197E-4	1.5100E-9	2.6285E-18	6.6164E-16	0.052295	8.3007E-20	1.4107E-20	1.9115E-4	1.0000
1.5100E-9	5.0000E-7	0.25000	-6.2894E-10	NaN	1.8193E-4	1.5100E-9	2.5025E-18	6.6153E-16	0.052286	8.2992E-20	1.4105E-20	1.9112E-4	1.0000
1.5100E-9	5.0000E-7	0.25000	-6.2894E-10	NaN	1.8193E-4	1.5100E-9	2.5025E-18	6.6153E-16	0.052286	8.2992E-20	1.4105E-20	1.9112E-4	1.0000
1.5100E-9	5.0000E-7	0.27000	-6.2863E-10	NaN	1.8185E-4	1.5100E-9	2.2268E-18	6.6122E-16	0.052260	8.2953E-20	1.4098E-20	1.9102E-4	1.0000
1.5100E-9	5.0000E-7	0.29000	-6.2837E-10	NaN	1.8177E-4	1.5100E-9	2.0271E-18	6.6096E-16	0.052239	8.2919E-20	1.4092E-20	1.9094E-4	1.0000
1.5100E-9	5.0000E-7	0.31000	-6.2811E-10	NaN	1.8170E-4	1.5100E-9	1.8274E-18	6.6070E-16	0.052217	8.2886E-20	1.4086E-20	1.9086E-4	1.0000
1.5100E-9	5.0000E-7	0.33000	-6.2786E-10	NaN	1.8163E-4	1.5100E-9	1.6277E-18	6.6044E-16	0.052195	8.2853E-20	1.4080E-20	1.9078E-4	1.0000
1.5100E-9	5.0000E-7	0.35000	-6.2760E-10	NaN	1.8155E-4	1.5100E-9	1.4280E-18	6.6018E-16	0.052174	8.2819E-20	1.4075E-20	1.9070E-4	1.0000
1.5100E-9	5.0000E-7	0.37000	-6.2741E-	NaN	1.8150E-4	1.5100E-9	1.3119E-18	6.5999E-16	0.052158	8.2795E-20	1.4070E-20	1.9065E-4	1.0000

k0Sub (m/s)	d (m)	Time (s)	-(tds2.ntflux_Oxi)*n*F (A), UME	Surface Con cOxi (M), Oxi Mem	Surface Con cOxi (M), OxiNuc	Substrate Kinetics (m/s), k0Sub probe	Normal total flux (mol/s), UME OCR membrane	Normal total flux (mol/s), UME OCR nucleus	Concentration (mol/m^3), Point: (5.5109E-16, 2.5)	k0Sub*Oxi (mol/s), Mem Flux	k0Sub*Oxi (mol/s), Nuc Flux	Normal total flux (mol/(m^2*s)), Point: (5.5109E-16, 2.5)	WElectrode radius (µm), UME Radius
			10										
1.5100E-9	5.0000E-7	0.39000	-6.2724E-10	NaN	1.8145E-4	1.5100E-9	1.2159E-18	6.5981E-16	0.052144	8.2773E-20	1.4067E-20	1.9060E-4	1.0000
1.5100E-9	5.0000E-7	0.41000	-6.2707E-10	NaN	1.8140E-4	1.5100E-9	1.1198E-18	6.5964E-16	0.052130	8.2751E-20	1.4063E-20	1.9054E-4	1.0000
1.5100E-9	5.0000E-7	0.43000	-6.2690E-10	NaN	1.8136E-4	1.5100E-9	1.0238E-18	6.5947E-16	0.052116	8.2729E-20	1.4059E-20	1.9049E-4	1.0000
1.5100E-9	5.0000E-7	0.45000	-6.2673E-10	NaN	1.8131E-4	1.5100E-9	9.2772E-19	6.5930E-16	0.052102	8.2707E-20	1.4055E-20	1.9044E-4	1.0000
1.5100E-9	5.0000E-7	0.47000	-6.2660E-10	NaN	1.8127E-4	1.5100E-9	8.6867E-19	6.5916E-16	0.052091	8.2690E-20	1.4052E-20	1.9040E-4	1.0000
1.5100E-9	5.0000E-7	0.49000	-6.2648E-10	NaN	1.8124E-4	1.5100E-9	8.1542E-19	6.5904E-16	0.052081	8.2675E-20	1.4050E-20	1.9037E-4	1.0000
1.5100E-9	5.0000E-7	0.51000	-6.2636E-10	NaN	1.8120E-4	1.5100E-9	7.6216E-19	6.5892E-16	0.052071	8.2659E-20	1.4047E-20	1.9033E-4	1.0000
1.5100E-9	5.0000E-7	0.53000	-6.2624E-10	NaN	1.8117E-4	1.5100E-9	7.0891E-19	6.5879E-16	0.052061	8.2643E-20	1.4044E-20	1.9029E-4	1.0000
1.5100E-9	5.0000E-7	0.55000	-6.2612E-10	NaN	1.8113E-4	1.5100E-9	6.5565E-19	6.5867E-16	0.052051	8.2628E-20	1.4042E-20	1.9026E-4	1.0000
1.5100E-	5.0000E-	0.57	-6.26	NaN	1.8110E-	1.5100E-	6.1970E-	6.5857E-	0.05	8.2615E-	1.4039E-	1.9023E-	1.00

k0Sub (m/s)	d (m)	Time (s)	-(tds2.ntflux_Oxi)*n*F (A), UME	Surface ConcOxi (M), OxiMem	Surface ConcOxi (M), OxiNuc	Substrate Kinetics (m/s), k0Sub probe	Normal total flux (mol/s), UME OCR membrane	Normal total flux (mol/s), UME OCR nucleus	Concentration (mol/m^3), Point: (5.5109E-16, 2.5)	k0Sub*Oxi (mol/s), Mem Flux	k0Sub*Oxi (mol/s), Nuc Flux	Normal total flux (mol/(m^2*s)), Point: (5.5109E-16, 2.5)	WElectrode radius (µm), UME Radius
9	7	000	02E-10		4	9	19	16	2043	20	20	4	00
1.5100E-9	5.0000E-7	0.59000	-6.2593E-10	NaN	1.8108E-4	1.5100E-9	5.8520E-19	6.5847E-16	0.052035	8.2603E-20	1.4037E-20	1.9020E-4	1.0000
1.5100E-9	5.0000E-7	0.61000	-6.2583E-10	NaN	1.8105E-4	1.5100E-9	5.5070E-19	6.5838E-16	0.052027	8.2590E-20	1.4035E-20	1.9017E-4	1.0000
1.5100E-9	5.0000E-7	0.63000	-6.2574E-10	NaN	1.8102E-4	1.5100E-9	5.1620E-19	6.5828E-16	0.052019	8.2578E-20	1.4033E-20	1.9014E-4	1.0000
1.5100E-9	5.0000E-7	0.65000	-6.2565E-10	NaN	1.8100E-4	1.5100E-9	4.8170E-19	6.5818E-16	0.052012	8.2566E-20	1.4031E-20	1.9011E-4	1.0000
1.5100E-9	5.0000E-7	0.67000	-6.2557E-10	NaN	1.8098E-4	1.5100E-9	4.5950E-19	6.5811E-16	0.052005	8.2556E-20	1.4029E-20	1.9009E-4	1.0000
1.5100E-9	5.0000E-7	0.69000	-6.2550E-10	NaN	1.8096E-4	1.5100E-9	4.3756E-19	6.5803E-16	0.051999	8.2547E-20	1.4028E-20	1.9007E-4	1.0000
1.5100E-9	5.0000E-7	0.71000	-6.2543E-10	NaN	1.8093E-4	1.5100E-9	4.1563E-19	6.5796E-16	0.051993	8.2537E-20	1.4026E-20	1.9005E-4	1.0000
1.5100E-9	5.0000E-7	0.73000	-6.2535E-10	NaN	1.8091E-4	1.5100E-9	3.9370E-19	6.5788E-16	0.051987	8.2528E-20	1.4024E-20	1.9002E-4	1.0000
1.5100E-9	5.0000E-7	0.75000	-6.2528E-10	NaN	1.8089E-4	1.5100E-9	3.7176E-19	6.5780E-16	0.051981	8.2518E-20	1.4023E-20	1.9000E-4	1.0000

k0Sub (m/s)	d (m)	Time (s)	-(tds2.ntflux_Oxi)*n*F (A), UME	Surface ConcOxi (M), OxiMem	Surface ConcOxi (M), OxiNuc	Substrate Kinetics (m/s), k0Sub probe	Normal total flux (mol/s), UME OCR membrane	Normal total flux (mol/s), UME OCR nucleus	Concentration (mol/m^3), Point: (5.5109E-16, 2.5)	k0Sub*Oxi (mol/s), Mem Flux	k0Sub*Oxi (mol/s), Nuc Flux	Normal total flux (mol/(m^2*s)), Point: (5.5109E-16, 2.5)	WElectrode radius (µm), UME Radius
1.5100E-9	5.0000E-7	0.77000	-6.2521E-10	NaN	1.8087E-4	1.5100E-9	3.5405E-19	6.5774E-16	0.051976	8.2510E-20	1.4021E-20	1.8998E-4	1.0000
1.5100E-9	5.0000E-7	0.79000	-6.2515E-10	NaN	1.8086E-4	1.5100E-9	3.3759E-19	6.5767E-16	0.051971	8.2501E-20	1.4020E-20	1.8996E-4	1.0000
1.5100E-9	5.0000E-7	0.81000	-6.2509E-10	NaN	1.8084E-4	1.5100E-9	3.2249E-19	6.5761E-16	0.051966	8.2494E-20	1.4019E-20	1.8994E-4	1.0000
1.5100E-9	5.0000E-7	0.83000	-6.2503E-10	NaN	1.8082E-4	1.5100E-9	3.0875E-19	6.5755E-16	0.051961	8.2486E-20	1.4017E-20	1.8993E-4	1.0000
1.5100E-9	5.0000E-7	0.85000	-6.2498E-10	NaN	1.8081E-4	1.5100E-9	2.9629E-19	6.5749E-16	0.051956	8.2479E-20	1.4016E-20	1.8991E-4	1.0000
1.5100E-9	5.0000E-7	0.87000	-6.2492E-10	NaN	1.8079E-4	1.5100E-9	2.8369E-19	6.5744E-16	0.051952	8.2472E-20	1.4015E-20	1.8989E-4	1.0000
1.5100E-9	5.0000E-7	0.89000	-6.2487E-10	NaN	1.8078E-4	1.5100E-9	2.7192E-19	6.5738E-16	0.051947	8.2465E-20	1.4014E-20	1.8988E-4	1.0000
1.5100E-9	5.0000E-7	0.91000	-6.2482E-10	NaN	1.8076E-4	1.5100E-9	2.6099E-19	6.5733E-16	0.051943	8.2458E-20	1.4013E-20	1.8986E-4	1.0000
1.5100E-9	5.0000E-7	0.93000	-6.2477E-10	NaN	1.8075E-4	1.5100E-9	2.5089E-19	6.5728E-16	0.051939	8.2452E-20	1.4012E-20	1.8985E-4	1.0000
1.5100E-9	5.0000E-7	0.95000	-6.2473E-	NaN	1.8073E-4	1.5100E-9	2.4155E-19	6.5723E-16	0.051935	8.2446E-20	1.4010E-20	1.8983E-4	1.0000

k0Sub (m/s)	d (m)	Time (s)	-(tds2.ntflux_Oxi)*n*F (A), UME	Surface ConcOxi (M), Oxi Mem	Surface ConcOxi (M), OxiNuc	Substrate Kinetics (m/s), k0Sub probe	Normal total flux (mol/s), UME OCR membrane	Normal total flux (mol/s), UME OCR nucleus	Concentration (mol/m^3), Point: (5.5109E-16, 2.5)	k0Sub* Oxi (mol/s), Mem Flux	k0Sub* Oxi (mol/s), Nuc Flux	Normal total flux (mol/(m^2*s)), Point: (5.5109E-16, 2.5)	WElectrode radius (µm), UME Radius
			10										
1.5100E-9	5.0000E-7	0.97000	-6.2468E-10	NaN	1.8072E-4	1.5100E-9	2.2330E-19	6.5718E-16	0.051932	8.2440E-20	1.4009E-20	1.8982E-4	1.0000
1.5100E-9	5.0000E-7	0.99000	-6.2464E-10	NaN	1.8071E-4	1.5100E-9	2.2361E-19	6.5714E-16	0.051928	8.2434E-20	1.4008E-20	1.8981E-4	1.0000
1.5100E-9	1.0000E-6	0.0000	-7.1537E-9	NaN	2.2700E-4	1.5100E-9	-2.0193E-20	-1.1923E-20	0.22700	1.0088E-19	2.0267E-20	-1.1900E-10	1.0000
1.5100E-9	1.0000E-6	0.0050000	-7.9198E-10	NaN	2.1025E-4	1.5100E-9	8.0009E-16	5.6569E-16	0.11268	9.4920E-20	1.7288E-20	1.4396E-4	1.0000
1.5100E-9	1.0000E-6	0.010000	-7.4672E-10	NaN	2.0278E-4	1.5100E-9	4.3921E-16	5.7401E-16	0.10612	9.1802E-20	1.6417E-20	1.3552E-4	1.0000
1.5100E-9	1.0000E-6	0.015000	-7.2418E-10	NaN	1.9831E-4	1.5100E-9	2.7178E-16	5.6979E-16	0.10288	8.9877E-20	1.5957E-20	1.3137E-4	1.0000
1.5100E-9	1.0000E-6	0.020000	-7.1001E-10	NaN	1.9536E-4	1.5100E-9	1.7727E-16	5.6599E-16	0.10085	8.8595E-20	1.5665E-20	1.2877E-4	1.0000
1.5100E-9	1.0000E-6	0.025000	-7.0045E-10	NaN	1.9331E-4	1.5100E-9	1.1870E-16	5.6287E-16	0.099479	8.7698E-20	1.5467E-20	1.2702E-4	1.0000
1.5100E-9	1.0000E-6	0.030000	-6.9382E-10	NaN	1.9183E-4	1.5100E-9	8.1509E-17	5.6033E-16	0.098529	8.7052E-20	1.5328E-20	1.2580E-4	1.0000
1.5100E-	1.0000E-	0.03	-6.89	NaN	1.9082E-	1.5100E-	6.0087E-	5.5831E-	0.09	8.6603E-	1.5235E-	1.2499E-	1.00

k0Sub (m/s)	d (m)	Time (s)	-(tds2.ntflux_Oxi)*n*F (A), UME	Surface ConcOxi (M), OxiMem	Surface ConcOxi (M), OxiNuc	Substrate Kinetics (m/s), k0Sub probe	Normal total flux (mol/s), UME OCR membrane	Normal total flux (mol/s), UME OCR nucleus	Concentration (mol/m^3), Point: (5.5109E-16, 2.5)	k0Sub*Oxi (mol/s), Mem Flux	k0Sub*Oxi (mol/s), Nuc Flux	Normal total flux (mol/(m^2*s)), Point: (5.5109E-16, 2.5)	WElectrode radius (µm), UME Radius
9	6	5000	41E-10		4	9	17	16	7899	20	20	4	00
1.5100E-9	1.0000E-6	0.040000	-6.8627E-10	NaN	1.9007E-4	1.5100E-9	4.6559E-17	5.5670E-16	0.097450	8.6272E-20	1.5168E-20	1.2442E-4	1.0000
1.5100E-9	1.0000E-6	0.045000	-6.8386E-10	NaN	1.8949E-4	1.5100E-9	3.7125E-17	5.5541E-16	0.097107	8.6015E-20	1.5117E-20	1.2398E-4	1.0000
1.5100E-9	1.0000E-6	0.050000	-6.8182E-10	NaN	1.8900E-4	1.5100E-9	2.9397E-17	5.5429E-16	0.096816	8.5795E-20	1.5073E-20	1.2361E-4	1.0000
1.5100E-9	1.0000E-6	0.055000	-6.8015E-10	NaN	1.8859E-4	1.5100E-9	2.3476E-17	5.5334E-16	0.096577	8.5613E-20	1.5037E-20	1.2330E-4	1.0000
1.5100E-9	1.0000E-6	0.060000	-6.7884E-10	NaN	1.8827E-4	1.5100E-9	1.9283E-17	5.5257E-16	0.096390	8.5467E-20	1.5009E-20	1.2306E-4	1.0000
1.5100E-9	1.0000E-6	0.065000	-6.7768E-10	NaN	1.8798E-4	1.5100E-9	1.5811E-17	5.5187E-16	0.096225	8.5338E-20	1.4984E-20	1.2285E-4	1.0000
1.5100E-9	1.0000E-6	0.070000	-6.7679E-10	NaN	1.8775E-4	1.5100E-9	1.3592E-17	5.5129E-16	0.096099	8.5237E-20	1.4965E-20	1.2269E-4	1.0000
1.5100E-9	1.0000E-6	0.075000	-6.7603E-10	NaN	1.8756E-4	1.5100E-9	1.1877E-17	5.5079E-16	0.095990	8.5148E-20	1.4948E-20	1.2255E-4	1.0000
1.5100E-9	1.0000E-6	0.080000	-6.7526E-10	NaN	1.8736E-4	1.5100E-9	9.9888E-18	5.5029E-16	0.095881	8.5061E-20	1.4932E-20	1.2241E-4	1.0000

k0Sub (m/s)	d (m)	Time (s)	-(tds2.ntflux_Oxi)*n*F (A), UME	Surface ConcOxi (M), Oxi Mem	Surface ConcOxi (M), OxiNuc	Substrate Kinetics (m/s), k0Sub probe	Normal total flux (mol/s), UME OCR membrane	Normal total flux (mol/s), UME OCR nucleus	Concentration (mol/m^3), Point: (5.5109E-16, 2.5)	k0Sub*Oxi (mol/s), Mem Flux	k0Sub*Oxi (mol/s), Nuc Flux	Normal total flux (mol/(m^2*s)), Point: (5.5109E-16, 2.5)	WElectrode radius (µm), UME Radius
1.5100E-9	1.0000E-6	0.085000	-6.7459E-10	NaN	1.8719E-4	1.5100E-9	8.4341E-18	5.4984E-16	0.095785	8.4983E-20	1.4917E-20	1.2229E-4	1.0000
1.5100E-9	1.0000E-6	0.090000	-6.7400E-10	NaN	1.8704E-4	1.5100E-9	7.2122E-18	5.4945E-16	0.095701	8.4915E-20	1.4904E-20	1.2218E-4	1.0000
1.5100E-9	1.0000E-6	0.095000	-6.7351E-10	NaN	1.8691E-4	1.5100E-9	6.3233E-18	5.4911E-16	0.095631	8.4857E-20	1.4893E-20	1.2209E-4	1.0000
1.5100E-9	1.0000E-6	0.10000	-6.7312E-10	NaN	1.8680E-4	1.5100E-9	5.7935E-18	5.4882E-16	0.095576	8.4810E-20	1.4885E-20	1.2202E-4	1.0000
1.5100E-9	1.0000E-6	0.10500	-6.7291E-10	NaN	1.8675E-4	1.5100E-9	5.6395E-18	5.4866E-16	0.095546	8.4785E-20	1.4880E-20	1.2199E-4	1.0000
1.5100E-9	1.0000E-6	0.11000	-6.7271E-10	NaN	1.8669E-4	1.5100E-9	5.4855E-18	5.4850E-16	0.095517	8.4759E-20	1.4876E-20	1.2195E-4	1.0000
1.5100E-9	1.0000E-6	0.11500	-6.7250E-10	NaN	1.8663E-4	1.5100E-9	5.3315E-18	5.4834E-16	0.095487	8.4734E-20	1.4871E-20	1.2191E-4	1.0000
1.5100E-9	1.0000E-6	0.12000	-6.7229E-10	NaN	1.8658E-4	1.5100E-9	5.1775E-18	5.4818E-16	0.095458	8.4708E-20	1.4867E-20	1.2187E-4	1.0000
1.5100E-9	1.0000E-6	0.12500	-6.7209E-10	NaN	1.8652E-4	1.5100E-9	5.0235E-18	5.4802E-16	0.095429	8.4683E-20	1.4862E-20	1.2184E-4	1.0000
1.5100E-9	1.0000E-6	0.13000	-6.7188E-	NaN	1.8647E-4	1.5100E-9	4.8695E-18	5.4786E-16	0.095399	8.4657E-20	1.4858E-20	1.2180E-4	1.0000

k0Sub (m/s)	d (m)	Time (s)	-(tds2.ntflux_Oxi)*n*F (A), UME	Surface ConcOxi (M), OxiMem	Surface ConcOxi (M), OxiNuc	Substrate Kinetics (m/s), k0Sub probe	Normal total flux (mol/s), UME OCR membrane	Normal total flux (mol/s), UME OCR nucleus	Concentration (mol/m^3), Point: (5.5109E-16, 2.5)	k0Sub*Oxi (mol/s), Mem Flux	k0Sub*Oxi (mol/s), Nuc Flux	Normal total flux (mol/(m^2*s)), Point: (5.5109E-16, 2.5)	WElectrode radius (µm), UME Radius
			10										
1.5100E-9	1.0000E-6	0.13500	-6.7167E-10	NaN	1.8641E-4	1.5100E-9	4.7155E-18	5.4770E-16	0.095370	8.4632E-20	1.4853E-20	1.2176E-4	1.0000
1.5100E-9	1.0000E-6	0.14000	-6.7146E-10	NaN	1.8635E-4	1.5100E-9	4.5615E-18	5.4754E-16	0.095340	8.4606E-20	1.4848E-20	1.2172E-4	1.0000
1.5100E-9	1.0000E-6	0.14500	-6.7126E-10	NaN	1.8630E-4	1.5100E-9	4.4075E-18	5.4738E-16	0.095311	8.4581E-20	1.4844E-20	1.2169E-4	1.0000
1.5100E-9	1.0000E-6	0.15000	-6.7105E-10	NaN	1.8624E-4	1.5100E-9	4.2535E-18	5.4722E-16	0.095282	8.4556E-20	1.4839E-20	1.2165E-4	1.0000
1.5100E-9	1.0000E-6	0.15500	-6.7086E-10	NaN	1.8619E-4	1.5100E-9	4.1127E-18	5.4707E-16	0.095254	8.4532E-20	1.4835E-20	1.2161E-4	1.0000
1.5100E-9	1.0000E-6	0.16000	-6.7072E-10	NaN	1.8615E-4	1.5100E-9	4.0013E-18	5.4696E-16	0.095234	8.4515E-20	1.4832E-20	1.2159E-4	1.0000
1.5100E-9	1.0000E-6	0.16500	-6.7057E-10	NaN	1.8611E-4	1.5100E-9	3.8899E-18	5.4685E-16	0.095214	8.4497E-20	1.4829E-20	1.2156E-4	1.0000
1.5100E-9	1.0000E-6	0.17000	-6.7043E-10	NaN	1.8607E-4	1.5100E-9	3.7785E-18	5.4675E-16	0.095194	8.4480E-20	1.4826E-20	1.2154E-4	1.0000
1.5100E-9	1.0000E-6	0.17500	-6.7029E-10	NaN	1.8604E-4	1.5100E-9	3.6671E-18	5.4664E-16	0.095174	8.4463E-20	1.4823E-20	1.2151E-4	1.0000
1.5100E-	1.0000E-	0.18	-6.70	NaN	1.8600E-	1.5100E-	3.5557E-	5.4653E-	0.09	8.4445E-	1.4820E-	1.2149E-	1.00

k0Sub (m/s)	d (m)	Time (s)	-(tds2.ntflux_Oxi)*n*F (A), UME	Surface Con cOxi (M), Oxi Mem	Surface Con cOxi (M), OxiNuc	Substrate Kinetics (m/s), k0Sub probe	Normal total flux (mol/s), UME OCR membrane	Normal total flux (mol/s), UME OCR nucleus	Concentration (mol/m^3), Point: (5.5109E-16, 2.5)	k0Sub*Oxi (mol/s), Mem Flux	k0Sub*Oxi (mol/s), Nuc Flux	Normal total flux (mol/(m^2*s)), Point: (5.5109E-16, 2.5)	WEIectrode radius (µm), UME Radius
9	6	000	15E-10		4	9	18	16	5154	20	20	4	00
1.5100E-9	1.0000E-6	0.18500	-6.7001E-10	NaN	1.8596E-4	1.5100E-9	3.4443E-18	5.4642E-16	0.095134	8.4428E-20	1.4817E-20	1.2146E-4	1.0000
1.5100E-9	1.0000E-6	0.19000	-6.6987E-10	NaN	1.8592E-4	1.5100E-9	3.3329E-18	5.4631E-16	0.095114	8.4411E-20	1.4813E-20	1.2143E-4	1.0000
1.5100E-9	1.0000E-6	0.19500	-6.6973E-10	NaN	1.8588E-4	1.5100E-9	3.2215E-18	5.4621E-16	0.095094	8.4394E-20	1.4810E-20	1.2141E-4	1.0000
1.5100E-9	1.0000E-6	0.20000	-6.6959E-10	NaN	1.8584E-4	1.5100E-9	3.1102E-18	5.4610E-16	0.095074	8.4376E-20	1.4807E-20	1.2138E-4	1.0000
1.5100E-9	1.0000E-6	0.20500	-6.6945E-10	NaN	1.8581E-4	1.5100E-9	2.9988E-18	5.4599E-16	0.095054	8.4359E-20	1.4804E-20	1.2136E-4	1.0000
1.5100E-9	1.0000E-6	0.21000	-6.6932E-10	NaN	1.8577E-4	1.5100E-9	2.9087E-18	5.4590E-16	0.095036	8.4344E-20	1.4801E-20	1.2134E-4	1.0000
1.5100E-9	1.0000E-6	0.21500	-6.6924E-10	NaN	1.8575E-4	1.5100E-9	2.8492E-18	5.4583E-16	0.095024	8.4334E-20	1.4800E-20	1.2132E-4	1.0000
1.5100E-9	1.0000E-6	0.22000	-6.6915E-10	NaN	1.8573E-4	1.5100E-9	2.7897E-18	5.4577E-16	0.095012	8.4323E-20	1.4798E-20	1.2130E-4	1.0000
1.5100E-9	1.0000E-6	0.22500	-6.6907E-10	NaN	1.8570E-4	1.5100E-9	2.7302E-18	5.4570E-16	0.095000	8.4313E-20	1.4796E-20	1.2129E-4	1.0000

k0Sub (m/s)	d (m)	Time (s)	-(tds2.ntflux_Oxi)*n*F (A), UME	Surface ConcOxi (M), Oxi Mem	Surface ConcOxi (M), OxiNuc	Substrate Kinetics (m/s), k0Sub probe	Normal total flux (mol/s), UME OCR membrane	Normal total flux (mol/s), UME OCR nucleus	Concentration (mol/m^3), Point: (5.5109E-16, 2.5)	k0Sub*Oxi (mol/s), Mem Flux	k0Sub*Oxi (mol/s), Nuc Flux	Normal total flux (mol/(m^2*s)), Point: (5.5109E-16, 2.5)	WElectrode radius (μm), UME Radius
1.5100E-9	1.0000E-6	0.23000	-6.6898E-10	NaN	1.8568E-4	1.5100E-9	2.6707E-18	5.4563E-16	0.094988	8.4302E-20	1.4794E-20	1.2127E-4	1.0000
1.5100E-9	1.0000E-6	0.23500	-6.6890E-10	NaN	1.8566E-4	1.5100E-9	2.6112E-18	5.4557E-16	0.094976	8.4292E-20	1.4792E-20	1.2126E-4	1.0000
1.5100E-9	1.0000E-6	0.24000	-6.6881E-10	NaN	1.8564E-4	1.5100E-9	2.5517E-18	5.4550E-16	0.094964	8.4281E-20	1.4790E-20	1.2124E-4	1.0000
1.5100E-9	1.0000E-6	0.24500	-6.6873E-10	NaN	1.8561E-4	1.5100E-9	2.4922E-18	5.4544E-16	0.094952	8.4271E-20	1.4788E-20	1.2123E-4	1.0000
1.5100E-9	1.0000E-6	0.25000	-6.6864E-10	NaN	1.8559E-4	1.5100E-9	2.4327E-18	5.4537E-16	0.094940	8.4261E-20	1.4786E-20	1.2121E-4	1.0000
1.5100E-9	1.0000E-6	0.25000	-6.6864E-10	NaN	1.8559E-4	1.5100E-9	2.4327E-18	5.4537E-16	0.094940	8.4261E-20	1.4786E-20	1.2121E-4	1.0000
1.5100E-9	1.0000E-6	0.27000	-6.6830E-10	NaN	1.8550E-4	1.5100E-9	2.1947E-18	5.4511E-16	0.094891	8.4219E-20	1.4779E-20	1.2115E-4	1.0000
1.5100E-9	1.0000E-6	0.29000	-6.6796E-10	NaN	1.8540E-4	1.5100E-9	1.9567E-18	5.4485E-16	0.094843	8.4177E-20	1.4772E-20	1.2109E-4	1.0000
1.5100E-9	1.0000E-6	0.31000	-6.6764E-10	NaN	1.8532E-4	1.5100E-9	1.7361E-18	5.4460E-16	0.094797	8.4137E-20	1.4764E-20	1.2103E-4	1.0000
1.5100E-9	1.0000E-6	0.33000	-6.6742E-	NaN	1.8526E-4	1.5100E-9	1.6121E-18	5.4442E-16	0.094766	8.4110E-20	1.4760E-20	1.2099E-4	1.0000

k0Sub (m/s)	d (m)	Time (s)	-(tds2.ntflux_Oxi)*n*F (A), UME	Surface ConcOxi (M), OxiMem	Surface ConcOxi (M), OxiNuc	Substrate Kinetics (m/s), k0Sub probe	Normal total flux (mol/s), UME OCR membrane	Normal total flux (mol/s), UME OCR nucleus	Concentration (mol/m^3), Point: (5.5109E-16, 2.5)	k0Sub*Oxi (mol/s), Mem Flux	k0Sub*Oxi (mol/s), Nuc Flux	Normal total flux (mol/(m^2*s)), Point: (5.5109E-16, 2.5)	WElectrode radius (µm), UME Radius
			10										
1.5100E-9	1.0000E-6	0.35000	-6.6720E-10	NaN	1.8520E-4	1.5100E-9	1.4881E-18	5.4425E-16	0.094734	8.4083E-20	1.4755E-20	1.2095E-4	1.0000
1.5100E-9	1.0000E-6	0.37000	-6.6698E-10	NaN	1.8514E-4	1.5100E-9	1.3641E-18	5.4408E-16	0.094703	8.4055E-20	1.4750E-20	1.2091E-4	1.0000
1.5100E-9	1.0000E-6	0.39000	-6.6676E-10	NaN	1.8508E-4	1.5100E-9	1.2401E-18	5.4391E-16	0.094671	8.4028E-20	1.4745E-20	1.2087E-4	1.0000
1.5100E-9	1.0000E-6	0.41000	-6.6655E-10	NaN	1.8502E-4	1.5100E-9	1.1274E-18	5.4374E-16	0.094642	8.4002E-20	1.4740E-20	1.2083E-4	1.0000
1.5100E-9	1.0000E-6	0.43000	-6.6639E-10	NaN	1.8498E-4	1.5100E-9	1.0584E-18	5.4362E-16	0.094620	8.3983E-20	1.4737E-20	1.2080E-4	1.0000
1.5100E-9	1.0000E-6	0.45000	-6.6624E-10	NaN	1.8493E-4	1.5100E-9	9.8932E-19	5.4350E-16	0.094598	8.3964E-20	1.4734E-20	1.2078E-4	1.0000
1.5100E-9	1.0000E-6	0.47000	-6.6608E-10	NaN	1.8489E-4	1.5100E-9	9.2026E-19	5.4338E-16	0.094576	8.3945E-20	1.4730E-20	1.2075E-4	1.0000
1.5100E-9	1.0000E-6	0.49000	-6.6593E-10	NaN	1.8485E-4	1.5100E-9	8.5120E-19	5.4326E-16	0.094554	8.3925E-20	1.4727E-20	1.2072E-4	1.0000
1.5100E-9	1.0000E-6	0.51000	-6.6578E-10	NaN	1.8481E-4	1.5100E-9	7.8923E-19	5.4314E-16	0.094533	8.3907E-20	1.4723E-20	1.2069E-4	1.0000
1.5100E-	1.0000E-	0.53	-6.65	NaN	1.8478E-	1.5100E-	7.4746E-	5.4305E-	0.09	8.3893E-	1.4721E-	1.2067E-	1.00

k0Sub (m/s)	d (m)	Time (s)	-(tds2.ntflux_Oxi)*n*F (A), UME	Surface ConcOxi (M), Oxi Mem	Surface ConcOxi (M), OxiNuc	Substrate Kinetics (m/s), k0Sub probe	Normal total flux (mol/s), UME OCR membrane	Normal total flux (mol/s), UME OCR nucleus	Concentration (mol/m^3), Point: (5.5109E-16, 2.5)	k0Sub*Oxi (mol/s), Mem Flux	k0Sub*Oxi (mol/s), Nuc Flux	Normal total flux (mol/(m^2*s)), Point: (5.5109E-16, 2.5)	WEIectrode radius (µm), UME Radius
9	6	000	67E-10		4	9	19	16	4517	20	20	4	00
1.5100E-9	1.0000E-6	0.55000	-6.6555E-10	NaN	1.8475E-4	1.5100E-9	7.0568E-19	5.4296E-16	0.094501	8.3879E-20	1.4718E-20	1.2065E-4	1.0000
1.5100E-9	1.0000E-6	0.57000	-6.6544E-10	NaN	1.8471E-4	1.5100E-9	6.6390E-19	5.4287E-16	0.094484	8.3864E-20	1.4716E-20	1.2063E-4	1.0000
1.5100E-9	1.0000E-6	0.59000	-6.6532E-10	NaN	1.8468E-4	1.5100E-9	6.2212E-19	5.4278E-16	0.094468	8.3850E-20	1.4713E-20	1.2061E-4	1.0000
1.5100E-9	1.0000E-6	0.61000	-6.6522E-10	NaN	1.8465E-4	1.5100E-9	5.8454E-19	5.4269E-16	0.094453	8.3837E-20	1.4711E-20	1.2059E-4	1.0000
1.5100E-9	1.0000E-6	0.63000	-6.6512E-10	NaN	1.8463E-4	1.5100E-9	5.5608E-19	5.4262E-16	0.094439	8.3825E-20	1.4709E-20	1.2057E-4	1.0000
1.5100E-9	1.0000E-6	0.65000	-6.6503E-10	NaN	1.8460E-4	1.5100E-9	5.2762E-19	5.4255E-16	0.094426	8.3814E-20	1.4707E-20	1.2056E-4	1.0000
1.5100E-9	1.0000E-6	0.67000	-6.6494E-10	NaN	1.8458E-4	1.5100E-9	4.9916E-19	5.4247E-16	0.094413	8.3802E-20	1.4705E-20	1.2054E-4	1.0000
1.5100E-9	1.0000E-6	0.69000	-6.6485E-10	NaN	1.8455E-4	1.5100E-9	4.7071E-19	5.4240E-16	0.094400	8.3791E-20	1.4703E-20	1.2052E-4	1.0000
1.5100E-9	1.0000E-6	0.71000	-6.6476E-10	NaN	1.8453E-4	1.5100E-9	4.4573E-19	5.4233E-16	0.094388	8.3780E-20	1.4701E-20	1.2051E-4	1.0000

k0Sub (m/s)	d (m)	Time (s)	-(tds2.ntflux_Oxi)*n*F (A), UME	Surface ConcOxi (M), Oxi Mem	Surface ConcOxi (M), OxiNuc	Substrate Kinetics (m/s), k0Sub probe	Normal total flux (mol/s), UME OCR membrane	Normal total flux (mol/s), UME OCR nucleus	Concentration (mol/m^3), Point: (5.5109E-16, 2.5)	k0Sub*Oxi (mol/s), Mem Flux	k0Sub*Oxi (mol/s), Nuc Flux	Normal total flux (mol/(m^2*s)), Point: (5.5109E-16, 2.5)	WElectrode radius (µm), UME Radius
1.5100E-9	1.0000E-6	0.73000	-6.6469E-10	NaN	1.8451E-4	1.5100E-9	4.2669E-19	5.4227E-16	0.094378	8.3771E-20	1.4699E-20	1.2049E-4	1.0000
1.5100E-9	1.0000E-6	0.75000	-6.6462E-10	NaN	1.8449E-4	1.5100E-9	4.0765E-19	5.4221E-16	0.094368	8.3762E-20	1.4698E-20	1.2048E-4	1.0000
1.5100E-9	1.0000E-6	0.77000	-6.6454E-10	NaN	1.8447E-4	1.5100E-9	3.8861E-19	5.4216E-16	0.094357	8.3753E-20	1.4696E-20	1.2047E-4	1.0000
1.5100E-9	1.0000E-6	0.79000	-6.6447E-10	NaN	1.8445E-4	1.5100E-9	3.6957E-19	5.4210E-16	0.094347	8.3744E-20	1.4695E-20	1.2045E-4	1.0000
1.5100E-9	1.0000E-6	0.81000	-6.6440E-10	NaN	1.8443E-4	1.5100E-9	3.5174E-19	5.4204E-16	0.094337	8.3735E-20	1.4693E-20	1.2044E-4	1.0000
1.5100E-9	1.0000E-6	0.83000	-6.6434E-10	NaN	1.8441E-4	1.5100E-9	3.3633E-19	5.4199E-16	0.094328	8.3727E-20	1.4692E-20	1.2043E-4	1.0000
1.5100E-9	1.0000E-6	0.85000	-6.6427E-10	NaN	1.8439E-4	1.5100E-9	3.2199E-19	5.4194E-16	0.094319	8.3719E-20	1.4690E-20	1.2042E-4	1.0000
1.5100E-9	1.0000E-6	0.87000	-6.6421E-10	NaN	1.8438E-4	1.5100E-9	3.0871E-19	5.4189E-16	0.094310	8.3711E-20	1.4689E-20	1.2041E-4	1.0000
1.5100E-9	1.0000E-6	0.89000	-6.6415E-10	NaN	1.8436E-4	1.5100E-9	2.9651E-19	5.4184E-16	0.094302	8.3704E-20	1.4688E-20	1.2040E-4	1.0000
1.5100E-9	1.0000E-6	0.91000	-6.6410E-	NaN	1.8435E-4	1.5100E-9	2.8494E-19	5.4180E-16	0.094294	8.3697E-20	1.4686E-20	1.2039E-4	1.0000

k0Sub (m/s)	d (m)	Time (s)	-(tds2.ntflux_Oxi)*n*F (A), UME	Surface Con cOxi (M), Oxi Mem	Surface Con cOxi (M), OxiNuc	Substrate Kinetics (m/s), k0Sub probe	Normal total flux (mol/s), UME OCR membrane	Normal total flux (mol/s), UME OCR nucleus	Concentration (mol/m^3), Point: (5.5109E-16, 2.5)	k0Sub*Oxi (mol/s), Mem Flux	k0Sub*Oxi (mol/s), Nuc Flux	Normal total flux (mol/(m^2*s)), Point: (5.5109E-16, 2.5)	WElectrode radius (µm), UME Radius
			10										
1.5100E-9	1.0000E-6	0.93000	-6.6404E-10	NaN	1.8433E-4	1.5100E-9	2.7365E-19	5.4175E-16	0.094286	8.3690E-20	1.4685E-20	1.2038E-4	1.0000
1.5100E-9	1.0000E-6	0.95000	-6.6399E-10	NaN	1.8432E-4	1.5100E-9	2.6308E-19	5.4171E-16	0.094278	8.3684E-20	1.4684E-20	1.2037E-4	1.0000
1.5100E-9	1.0000E-6	0.97000	-6.6394E-10	NaN	1.8430E-4	1.5100E-9	2.5322E-19	5.4167E-16	0.094271	8.3677E-20	1.4683E-20	1.2036E-4	1.0000
1.5100E-9	1.0000E-6	0.99000	-6.6389E-10	NaN	1.8429E-4	1.5100E-9	2.4408E-19	5.4163E-16	0.094264	8.3671E-20	1.4682E-20	1.2035E-4	1.0000
1.5100E-9	1.5000E-6	0.0000	-7.1533E-9	NaN	2.2700E-4	1.5100E-9	-2.0267E-20	-1.2323E-20	0.22700	1.0088E-19	2.0267E-20	-1.6335E-10	1.0000
1.5100E-9	1.5000E-6	0.005000	-8.1796E-10	NaN	2.1331E-4	1.5100E-9	7.3028E-16	4.5102E-16	0.14619	9.5958E-20	1.7883E-20	8.6780E-5	1.0000
1.5100E-9	1.5000E-6	0.010000	-7.7421E-10	NaN	2.0632E-4	1.5100E-9	4.0758E-16	4.6761E-16	0.13820	9.3064E-20	1.7046E-20	8.1990E-5	1.0000
1.5100E-9	1.5000E-6	0.015000	-7.5228E-10	NaN	2.0205E-4	1.5100E-9	2.5501E-16	4.6721E-16	0.13423	9.1235E-20	1.6599E-20	7.9624E-5	1.0000
1.5100E-9	1.5000E-6	0.020000	-7.3906E-10	NaN	1.9927E-4	1.5100E-9	1.7347E-16	4.6490E-16	0.13185	9.0024E-20	1.6324E-20	7.8206E-5	1.0000
1.5100E-	1.5000E-	0.02	-7.29	NaN	1.9730E-	1.5100E-	1.2255E-	4.6298E-	0.13	8.9164E-	1.6133E-	7.7228E-	1.00

k0Sub (m/s)	d (m)	Time (s)	-(tds2.ntflux_Oxi)*n*F (A), UME	Surface ConcOxi (M), OxiMem	Surface ConcOxi (M), OxiNuc	Substrate Kinetics (m/s), k0Sub probe	Normal total flux (mol/s), UME OCR membrane	Normal total flux (mol/s), UME OCR nucleus	Concentration (mol/m^3), Point: (5.5109E-16, 2.5)	k0Sub*Oxi (mol/s), Mem Flux	k0Sub*Oxi (mol/s), Nuc Flux	Normal total flux (mol/(m^2*s)), Point: (5.5109E-16, 2.5)	WEIectrode radius (µm), UME Radius
9	6	5000	94E-10		4	9	16	16	021	20	20	5	00
1.5100E-9	1.5000E-6	0.030000	-7.2332E-10	NaN	1.9585E-4	1.5100E-9	8.8282E-17	4.6144E-16	0.12902	8.8529E-20	1.5994E-20	7.6519E-5	1.0000
1.5100E-9	1.5000E-6	0.035000	-7.1857E-10	NaN	1.9478E-4	1.5100E-9	6.6072E-17	4.6014E-16	0.12817	8.8060E-20	1.5894E-20	7.6011E-5	1.0000
1.5100E-9	1.5000E-6	0.040000	-7.1469E-10	NaN	1.9390E-4	1.5100E-9	4.8664E-17	4.5899E-16	0.12747	8.7672E-20	1.5812E-20	7.5596E-5	1.0000
1.5100E-9	1.5000E-6	0.045000	-7.1167E-10	NaN	1.9320E-4	1.5100E-9	3.6079E-17	4.5801E-16	0.12692	8.7364E-20	1.5748E-20	7.5273E-5	1.0000
1.5100E-9	1.5000E-6	0.050000	-7.0945E-10	NaN	1.9268E-4	1.5100E-9	2.7987E-17	4.5718E-16	0.12653	8.7131E-20	1.5700E-20	7.5036E-5	1.0000
1.5100E-9	1.5000E-6	0.055000	-7.0753E-10	NaN	1.9222E-4	1.5100E-9	2.1280E-17	4.5644E-16	0.12618	8.6929E-20	1.5659E-20	7.4831E-5	1.0000
1.5100E-9	1.5000E-6	0.060000	-7.0618E-10	NaN	1.9189E-4	1.5100E-9	1.7594E-17	4.5582E-16	0.12594	8.6780E-20	1.5630E-20	7.4687E-5	1.0000
1.5100E-9	1.5000E-6	0.065000	-7.0512E-10	NaN	1.9162E-4	1.5100E-9	1.5300E-17	4.5529E-16	0.12575	8.6661E-20	1.5607E-20	7.4575E-5	1.0000
1.5100E-9	1.5000E-6	0.070000	-7.0415E-10	NaN	1.9138E-4	1.5100E-9	1.3090E-17	4.5481E-16	0.12558	8.6551E-20	1.5586E-20	7.4472E-5	1.0000

k0Sub (m/s)	d (m)	Time (s)	-(tds2.ntflux_Oxi)*n*F (A), UME	Surface ConcOxi (M), OxiMem	Surface ConcOxi (M), OxiNuc	Substrate Kinetics (m/s), k0Sub probe	Normal total flux (mol/s), UME OCR membrane	Normal total flux (mol/s), UME OCR nucleus	Concentration (mol/m^3), Point: (5.5109E-16, 2.5)	k0Sub*Oxi (mol/s), Mem Flux	k0Sub*Oxi (mol/s), Nuc Flux	Normal total flux (mol/(m^2*s)), Point: (5.5109E-16, 2.5)	WElectrode radius (µm), UME Radius
1.5100E-9	1.5000E-6	0.075000	-7.0342E-10	NaN	1.9119E-4	1.5100E-9	1.1979E-17	4.5441E-16	0.12545	8.6467E-20	1.5570E-20	7.4395E-5	1.0000
1.5100E-9	1.5000E-6	0.080000	-7.0269E-10	NaN	1.9100E-4	1.5100E-9	1.0654E-17	4.5402E-16	0.12532	8.6383E-20	1.5554E-20	7.4317E-5	1.0000
1.5100E-9	1.5000E-6	0.085000	-7.0200E-10	NaN	1.9082E-4	1.5100E-9	9.3313E-18	4.5366E-16	0.12519	8.6304E-20	1.5539E-20	7.4244E-5	1.0000
1.5100E-9	1.5000E-6	0.090000	-7.0138E-10	NaN	1.9067E-4	1.5100E-9	8.2067E-18	4.5333E-16	0.12508	8.6233E-20	1.5525E-20	7.4178E-5	1.0000
1.5100E-9	1.5000E-6	0.095000	-7.0083E-10	NaN	1.9053E-4	1.5100E-9	7.2807E-18	4.5304E-16	0.12498	8.6170E-20	1.5513E-20	7.4120E-5	1.0000
1.5100E-9	1.5000E-6	0.10000	-7.0036E-10	NaN	1.9040E-4	1.5100E-9	6.5531E-18	4.5279E-16	0.12490	8.6115E-20	1.5503E-20	7.4070E-5	1.0000
1.5100E-9	1.5000E-6	0.10500	-7.0003E-10	NaN	1.9032E-4	1.5100E-9	6.1558E-18	4.5260E-16	0.12484	8.6076E-20	1.5496E-20	7.4035E-5	1.0000
1.5100E-9	1.5000E-6	0.11000	-6.9981E-10	NaN	1.9026E-4	1.5100E-9	5.9831E-18	4.5247E-16	0.12480	8.6049E-20	1.5491E-20	7.4011E-5	1.0000
1.5100E-9	1.5000E-6	0.11500	-6.9958E-10	NaN	1.9020E-4	1.5100E-9	5.8104E-18	4.5233E-16	0.12476	8.6023E-20	1.5486E-20	7.3988E-5	1.0000
1.5100E-9	1.5000E-6	0.12000	-6.9936E-	NaN	1.9014E-4	1.5100E-9	5.6378E-18	4.5220E-16	0.12472	8.5996E-20	1.5481E-20	7.3964E-5	1.0000

k0Sub (m/s)	d (m)	Time (s)	-(tds2.ntflux_Oxi)*n*F (A), UME	Surface ConcOxi (M), OxiMem	Surface ConcOxi (M), OxiNuc	Substrate Kinetics (m/s), k0Sub probe	Normal total flux (mol/s), UME OCR membrane	Normal total flux (mol/s), UME OCR nucleus	Concentration (mol/m^3), Point: (5.5109E-16, 2.5)	k0Sub*Oxi (mol/s), Mem Flux	k0Sub*Oxi (mol/s), Nuc Flux	Normal total flux (mol/(m^2*s)), Point: (5.5109E-16, 2.5)	WElectrode radius (µm), UME Radius
			10										
1.5100E-9	1.5000E-6	0.12500	-6.9914E-10	NaN	1.9008E-4	1.5100E-9	5.4651E-18	4.5207E-16	0.12468	8.5970E-20	1.5476E-20	7.3941E-5	1.0000
1.5100E-9	1.5000E-6	0.13000	-6.9892E-10	NaN	1.9002E-4	1.5100E-9	5.2924E-18	4.5194E-16	0.12464	8.5943E-20	1.5471E-20	7.3917E-5	1.0000
1.5100E-9	1.5000E-6	0.13500	-6.9870E-10	NaN	1.8996E-4	1.5100E-9	5.1198E-18	4.5180E-16	0.12460	8.5917E-20	1.5467E-20	7.3894E-5	1.0000
1.5100E-9	1.5000E-6	0.14000	-6.9848E-10	NaN	1.8991E-4	1.5100E-9	4.9471E-18	4.5167E-16	0.12456	8.5890E-20	1.5462E-20	7.3870E-5	1.0000
1.5100E-9	1.5000E-6	0.14500	-6.9825E-10	NaN	1.8985E-4	1.5100E-9	4.7744E-18	4.5154E-16	0.12452	8.5864E-20	1.5457E-20	7.3847E-5	1.0000
1.5100E-9	1.5000E-6	0.15000	-6.9803E-10	NaN	1.8979E-4	1.5100E-9	4.6018E-18	4.5140E-16	0.12448	8.5837E-20	1.5452E-20	7.3823E-5	1.0000
1.5100E-9	1.5000E-6	0.15500	-6.9781E-10	NaN	1.8973E-4	1.5100E-9	4.4291E-18	4.5127E-16	0.12444	8.5811E-20	1.5447E-20	7.3800E-5	1.0000
1.5100E-9	1.5000E-6	0.16000	-6.9763E-10	NaN	1.8968E-4	1.5100E-9	4.2916E-18	4.5117E-16	0.12441	8.5790E-20	1.5443E-20	7.3781E-5	1.0000
1.5100E-9	1.5000E-6	0.16500	-6.9748E-10	NaN	1.8964E-4	1.5100E-9	4.1715E-18	4.5107E-16	0.12439	8.5772E-20	1.5440E-20	7.3765E-5	1.0000
1.5100E-9	1.5000E-	0.17	-6.97	NaN	1.8960E-	1.5100E-	4.0513E-	4.5098E-	0.12	8.5753E-	1.5437E-	7.3749E-	1.00

k0Sub (m/s)	d (m)	Time (s)	-(tds2.ntflux_Oxi)*n*F (A), UME	Surface ConcOxi (M), Oxi Mem	Surface ConcOxi (M), OxiNuc	Substrate Kinetics (m/s), k0Sub probe	Normal total flux (mol/s), UME OCR membrane	Normal total flux (mol/s), UME OCR nucleus	Concentration (mol/m^3), Point: (5.5109E-16, 2.5)	k0Sub* Oxi (mol/s), Mem Flux	k0Sub* Oxi (mol/s), Nuc Flux	Normal total flux (mol/(m^2*s)), Point: (5.5109E-16, 2.5)	WElectrode radius (µm), UME Radius
9	6	000	33E-10		4	9	18	16	436	20	20	5	00
1.5100E-9	1.5000E-6	0.17500	-6.9718E-10	NaN	1.8956E-4	1.5100E-9	3.9312E-18	4.5089E-16	0.12433	8.5735E-20	1.5433E-20	7.3733E-5	1.0000
1.5100E-9	1.5000E-6	0.18000	-6.9703E-10	NaN	1.8952E-4	1.5100E-9	3.8110E-18	4.5080E-16	0.12430	8.5717E-20	1.5430E-20	7.3717E-5	1.0000
1.5100E-9	1.5000E-6	0.18500	-6.9687E-10	NaN	1.8948E-4	1.5100E-9	3.6909E-18	4.5071E-16	0.12428	8.5699E-20	1.5427E-20	7.3701E-5	1.0000
1.5100E-9	1.5000E-6	0.19000	-6.9672E-10	NaN	1.8944E-4	1.5100E-9	3.5708E-18	4.5062E-16	0.12425	8.5681E-20	1.5423E-20	7.3685E-5	1.0000
1.5100E-9	1.5000E-6	0.19500	-6.9657E-10	NaN	1.8940E-4	1.5100E-9	3.4506E-18	4.5053E-16	0.12422	8.5663E-20	1.5420E-20	7.3669E-5	1.0000
1.5100E-9	1.5000E-6	0.20000	-6.9642E-10	NaN	1.8936E-4	1.5100E-9	3.3305E-18	4.5044E-16	0.12420	8.5645E-20	1.5417E-20	7.3653E-5	1.0000
1.5100E-9	1.5000E-6	0.20500	-6.9627E-10	NaN	1.8932E-4	1.5100E-9	3.2104E-18	4.5035E-16	0.12417	8.5627E-20	1.5413E-20	7.3637E-5	1.0000
1.5100E-9	1.5000E-6	0.21000	-6.9612E-10	NaN	1.8928E-4	1.5100E-9	3.0902E-18	4.5026E-16	0.12414	8.5609E-20	1.5410E-20	7.3620E-5	1.0000
1.5100E-9	1.5000E-6	0.21500	-6.9602E-10	NaN	1.8925E-4	1.5100E-9	3.0198E-18	4.5020E-16	0.12412	8.5597E-20	1.5408E-20	7.3610E-5	1.0000

k0Sub (m/s)	d (m)	Time (s)	-(tds2.ntflux_Oxi)*n*F (A), UME	Surface ConcOxi (M), OxiMem	Surface ConcOxi (M), OxiNuc	Substrate Kinetics (m/s), k0Sub probe	Normal total flux (mol/s), UME OCR membrane	Normal total flux (mol/s), UME OCR nucleus	Concentration (mol/m^3), Point: (5.5109E-16, 2.5)	k0Sub*Oxi (mol/s), Mem Flux	k0Sub*Oxi (mol/s), Nuc Flux	Normal total flux (mol/(m^2*s)), Point: (5.5109E-16, 2.5)	WElectrode radius (µm), UME Radius
1.5100E-9	1.5000E-6	0.22000	-6.9592E-10	NaN	1.8923E-4	1.5100E-9	2.9575E-18	4.5015E-16	0.12411	8.5586E-20	1.5406E-20	7.3600E-5	1.0000
1.5100E-9	1.5000E-6	0.22500	-6.9583E-10	NaN	1.8920E-4	1.5100E-9	2.8953E-18	4.5009E-16	0.12409	8.5575E-20	1.5404E-20	7.3590E-5	1.0000
1.5100E-9	1.5000E-6	0.23000	-6.9574E-10	NaN	1.8918E-4	1.5100E-9	2.8330E-18	4.5004E-16	0.12408	8.5564E-20	1.5402E-20	7.3581E-5	1.0000
1.5100E-9	1.5000E-6	0.23500	-6.9565E-10	NaN	1.8916E-4	1.5100E-9	2.7708E-18	4.4998E-16	0.12406	8.5553E-20	1.5400E-20	7.3571E-5	1.0000
1.5100E-9	1.5000E-6	0.24000	-6.9556E-10	NaN	1.8913E-4	1.5100E-9	2.7085E-18	4.4993E-16	0.12404	8.5542E-20	1.5398E-20	7.3561E-5	1.0000
1.5100E-9	1.5000E-6	0.24500	-6.9547E-10	NaN	1.8911E-4	1.5100E-9	2.6463E-18	4.4987E-16	0.12403	8.5531E-20	1.5396E-20	7.3552E-5	1.0000
1.5100E-9	1.5000E-6	0.25000	-6.9538E-10	NaN	1.8908E-4	1.5100E-9	2.5840E-18	4.4982E-16	0.12401	8.5520E-20	1.5394E-20	7.3542E-5	1.0000
1.5100E-9	1.5000E-6	0.25000	-6.9538E-10	NaN	1.8908E-4	1.5100E-9	2.5840E-18	4.4982E-16	0.12401	8.5520E-20	1.5394E-20	7.3542E-5	1.0000
1.5100E-9	1.5000E-6	0.27000	-6.9501E-10	NaN	1.8899E-4	1.5100E-9	2.3350E-18	4.4960E-16	0.12395	8.5476E-20	1.5385E-20	7.3503E-5	1.0000
1.5100E-9	1.5000E-6	0.29000	-6.9464E-	NaN	1.8889E-4	1.5100E-9	2.0860E-18	4.4938E-16	0.12388	8.5432E-20	1.5377E-20	7.3465E-5	1.0000

k0Sub (m/s)	d (m)	Time (s)	-(tds2.ntflux_Oxi)*n*F (A), UME	Surface ConcOxi (M), Oxi Mem	Surface ConcOxi (M), OxiNuc	Substrate Kinetics (m/s), k0Sub probe	Normal total flux (mol/s), UME OCR membrane	Normal total flux (mol/s), UME OCR nucleus	Concentration (mol/m^3), Point: (5.5109E-16, 2.5)	k0Sub*Oxi (mol/s), Mem Flux	k0Sub*Oxi (mol/s), Nuc Flux	Normal total flux (mol/(m^2*s)), Point: (5.5109E-16, 2.5)	WElectrode radius (µm), UME Radius
			10										
1.5100E-9	1.5000E-6	0.31000	-6.9428E-10	NaN	1.8879E-4	1.5100E-9	1.8387E-18	4.4916E-16	0.12381	8.5389E-20	1.5369E-20	7.3426E-5	1.0000
1.5100E-9	1.5000E-6	0.33000	-6.9404E-10	NaN	1.8873E-4	1.5100E-9	1.7093E-18	4.4901E-16	0.12377	8.5360E-20	1.5364E-20	7.3401E-5	1.0000
1.5100E-9	1.5000E-6	0.35000	-6.9380E-10	NaN	1.8867E-4	1.5100E-9	1.5799E-18	4.4887E-16	0.12373	8.5331E-20	1.5359E-20	7.3376E-5	1.0000
1.5100E-9	1.5000E-6	0.37000	-6.9357E-10	NaN	1.8860E-4	1.5100E-9	1.4505E-18	4.4872E-16	0.12369	8.5303E-20	1.5354E-20	7.3350E-5	1.0000
1.5100E-9	1.5000E-6	0.39000	-6.9333E-10	NaN	1.8854E-4	1.5100E-9	1.3211E-18	4.4858E-16	0.12364	8.5274E-20	1.5348E-20	7.3325E-5	1.0000
1.5100E-9	1.5000E-6	0.41000	-6.9310E-10	NaN	1.8848E-4	1.5100E-9	1.1955E-18	4.4843E-16	0.12360	8.5246E-20	1.5343E-20	7.3300E-5	1.0000
1.5100E-9	1.5000E-6	0.43000	-6.9293E-10	NaN	1.8843E-4	1.5100E-9	1.1232E-18	4.4833E-16	0.12357	8.5226E-20	1.5340E-20	7.3283E-5	1.0000
1.5100E-9	1.5000E-6	0.45000	-6.9276E-10	NaN	1.8839E-4	1.5100E-9	1.0509E-18	4.4823E-16	0.12354	8.5205E-20	1.5336E-20	7.3265E-5	1.0000
1.5100E-9	1.5000E-6	0.47000	-6.9259E-10	NaN	1.8834E-4	1.5100E-9	9.7856E-19	4.4812E-16	0.12351	8.5185E-20	1.5332E-20	7.3247E-5	1.0000
1.5100E-	1.5000E-	0.49	-6.92	NaN	1.8830E-	1.5100E-	9.0624E-	4.4802E-	0.12	8.5165E-	1.5329E-	7.3230E-	1.00

k0Sub (m/s)	d (m)	Time (s)	-(tds2.ntflux_Oxi)*n*F (A), UME	Surface ConcOxi (M), Oxi Mem	Surface ConcOxi (M), OxiNuc	Substrate Kinetics (m/s), k0Sub probe	Normal total flux (mol/s), UME OCR membrane	Normal total flux (mol/s), UME OCR nucleus	Concentration (mol/m^3), Point: (5.5109E-16, 2.5)	k0Sub* Oxi (mol/s), Mem Flux	k0Sub* Oxi (mol/s), Nuc Flux	Normal total flux (mol/(m^2*s)), Point: (5.5109E-16, 2.5)	WElectrode radius (µm), UME Radius
9	6	000	43E-10		4	9	19	16	348	20	20	5	00
1.5100E-9	1.5000E-6	0.51000	-6.9227E-10	NaN	1.8825E-4	1.5100E-9	8.3731E-19	4.4792E-16	0.12346	8.5145E-20	1.5325E-20	7.3213E-5	1.0000
1.5100E-9	1.5000E-6	0.53000	-6.9214E-10	NaN	1.8822E-4	1.5100E-9	7.9345E-19	4.4784E-16	0.12343	8.5130E-20	1.5322E-20	7.3200E-5	1.0000
1.5100E-9	1.5000E-6	0.55000	-6.9202E-10	NaN	1.8819E-4	1.5100E-9	7.4959E-19	4.4777E-16	0.12341	8.5115E-20	1.5320E-20	7.3186E-5	1.0000
1.5100E-9	1.5000E-6	0.57000	-6.9189E-10	NaN	1.8815E-4	1.5100E-9	7.0574E-19	4.4769E-16	0.12339	8.5100E-20	1.5317E-20	7.3173E-5	1.0000
1.5100E-9	1.5000E-6	0.59000	-6.9177E-10	NaN	1.8812E-4	1.5100E-9	6.6188E-19	4.4761E-16	0.12337	8.5085E-20	1.5314E-20	7.3160E-5	1.0000
1.5100E-9	1.5000E-6	0.61000	-6.9165E-10	NaN	1.8809E-4	1.5100E-9	6.2041E-19	4.4754E-16	0.12335	8.5071E-20	1.5311E-20	7.3147E-5	1.0000
1.5100E-9	1.5000E-6	0.63000	-6.9155E-10	NaN	1.8806E-4	1.5100E-9	5.9050E-19	4.4747E-16	0.12333	8.5058E-20	1.5309E-20	7.3137E-5	1.0000
1.5100E-9	1.5000E-6	0.65000	-6.9145E-10	NaN	1.8803E-4	1.5100E-9	5.6059E-19	4.4741E-16	0.12331	8.5046E-20	1.5307E-20	7.3126E-5	1.0000
1.5100E-9	1.5000E-6	0.67000	-6.9135E-10	NaN	1.8801E-4	1.5100E-9	5.3068E-19	4.4735E-16	0.12329	8.5034E-20	1.5305E-20	7.3116E-5	1.0000

k0Sub (m/s)	d (m)	Time (s)	-(tds2.ntflux_Oxi)*n*F (A), UME	Surface Con cOxi (M), Oxi Mem	Surface Con cOxi (M), OxiNuc	Substrate Kinetics (m/s), k0Sub probe	Normal total flux (mol/s), UME OCR membrane	Normal total flux (mol/s), UME OCR nucleus	Concentration (mol/m^3), Point: (5.5109E-16, 2.5)	k0Sub*Oxi (mol/s), Mem Flux	k0Sub*Oxi (mol/s), Nuc Flux	Normal total flux (mol/(m^2*s)), Point: (5.5109E-16, 2.5)	WElectrode radius (µm), UME Radius
1.5100E-9	1.5000E-6	0.69000	-6.9125E-10	NaN	1.8798E-4	1.5100E-9	5.0076E-19	4.4729E-16	0.12327	8.5022E-20	1.5303E-20	7.3105E-5	1.0000
1.5100E-9	1.5000E-6	0.71000	-6.9116E-10	NaN	1.8796E-4	1.5100E-9	4.7306E-19	4.4723E-16	0.12326	8.5011E-20	1.5301E-20	7.3095E-5	1.0000
1.5100E-9	1.5000E-6	0.73000	-6.9108E-10	NaN	1.8793E-4	1.5100E-9	4.5303E-19	4.4718E-16	0.12324	8.5001E-20	1.5299E-20	7.3087E-5	1.0000
1.5100E-9	1.5000E-6	0.75000	-6.9100E-10	NaN	1.8791E-4	1.5100E-9	4.3299E-19	4.4713E-16	0.12323	8.4992E-20	1.5297E-20	7.3079E-5	1.0000
1.5100E-9	1.5000E-6	0.77000	-6.9092E-10	NaN	1.8789E-4	1.5100E-9	4.1296E-19	4.4708E-16	0.12322	8.4982E-20	1.5295E-20	7.3070E-5	1.0000
1.5100E-9	1.5000E-6	0.79000	-6.9084E-10	NaN	1.8787E-4	1.5100E-9	3.9292E-19	4.4703E-16	0.12320	8.4972E-20	1.5294E-20	7.3062E-5	1.0000
1.5100E-9	1.5000E-6	0.81000	-6.9077E-10	NaN	1.8785E-4	1.5100E-9	3.7369E-19	4.4698E-16	0.12319	8.4963E-20	1.5292E-20	7.3054E-5	1.0000
1.5100E-9	1.5000E-6	0.83000	-6.9070E-10	NaN	1.8783E-4	1.5100E-9	3.5730E-19	4.4694E-16	0.12317	8.4954E-20	1.5290E-20	7.3046E-5	1.0000
1.5100E-9	1.5000E-6	0.85000	-6.9063E-10	NaN	1.8781E-4	1.5100E-9	3.4203E-19	4.4689E-16	0.12316	8.4946E-20	1.5289E-20	7.3039E-5	1.0000
1.5100E-9	1.5000E-6	0.87000	-6.9056E-	NaN	1.8779E-4	1.5100E-9	3.2789E-19	4.4685E-16	0.12315	8.4938E-20	1.5287E-20	7.3032E-5	1.0000

k0Sub (m/s)	d (m)	Time (s)	-(tds2.ntflux_Oxi)*n*F (A), UME	Surface ConcOxi (M), OxiMem	Surface ConcOxi (M), OxiNuc	Substrate Kinetics (m/s), k0Sub probe	Normal total flux (mol/s), UME OCR membrane	Normal total flux (mol/s), UME OCR nucleus	Concentration (mol/m^3), Point: (5.5109E-16, 2.5)	k0Sub*Oxi (mol/s), Mem Flux	k0Sub*Oxi (mol/s), Nuc Flux	Normal total flux (mol/(m^2*s)), Point: (5.5109E-16, 2.5)	WElectrode radius (µm), UME Radius
			10										
1.5100E-9	1.5000E-6	0.89000	-6.9050E-10	NaN	1.8778E-4	1.5100E-9	3.1486E-19	4.4681E-16	0.12314	8.4930E-20	1.5286E-20	7.3026E-5	1.0000
1.5100E-9	1.5000E-6	0.91000	-6.9044E-10	NaN	1.8776E-4	1.5100E-9	3.0265E-19	4.4677E-16	0.12313	8.4923E-20	1.5285E-20	7.3019E-5	1.0000
1.5100E-9	1.5000E-6	0.93000	-6.9038E-10	NaN	1.8774E-4	1.5100E-9	2.9065E-19	4.4673E-16	0.12312	8.4916E-20	1.5283E-20	7.3013E-5	1.0000
1.5100E-9	1.5000E-6	0.95000	-6.9032E-10	NaN	1.8773E-4	1.5100E-9	2.7940E-19	4.4670E-16	0.12311	8.4909E-20	1.5282E-20	7.3007E-5	1.0000
1.5100E-9	1.5000E-6	0.97000	-6.9027E-10	NaN	1.8771E-4	1.5100E-9	2.6890E-19	4.4666E-16	0.12310	8.4902E-20	1.5281E-20	7.3001E-5	1.0000
1.5100E-9	1.5000E-6	0.99000	-6.9021E-10	NaN	1.8770E-4	1.5100E-9	2.5915E-19	4.4663E-16	0.12309	8.4895E-20	1.5280E-20	7.2995E-5	1.0000
1.5100E-9	2.0000E-6	0.0000	-7.1541E-9	NaN	2.2700E-4	1.5100E-9	-2.0267E-20	-1.2562E-20	0.22700	1.0088E-19	2.0267E-20	-1.8100E-10	1.0000
1.5100E-9	2.0000E-6	0.005000	-8.3271E-10	NaN	2.1578E-4	1.5100E-9	6.6764E-16	3.6490E-16	0.16804	9.6811E-20	1.8350E-20	5.4329E-5	1.0000
1.5100E-9	2.0000E-6	0.010000	-7.9304E-10	NaN	2.0938E-4	1.5100E-9	3.8336E-16	3.8300E-16	0.15985	9.4162E-20	1.7579E-20	5.1661E-5	1.0000
1.5100E-9	2.0000E-	0.01	-7.72	NaN	2.0537E-	1.5100E-	2.4602E-	3.8494E-	0.15	9.2450E-	1.7156E-	5.0290E-	1.00

k0Sub (m/s)	d (m)	Time (s)	-(tds2.ntflux_Oxi)*n*F (A), UME	Surface Con cOxi (M), Oxi Mem	Surface Con cOxi (M), OxiNuc	Substrate Kinetics (m/s), k0Sub probe	Normal total flux (mol/s), UME OCR membrane	Normal total flux (mol/s), UME OCR nucleus	Concentration (mol/m^3), Point: (5.5109E-16, 2.5)	k0Sub* Oxi (mol/s), Mem Flux	k0Sub* Oxi (mol/s), Nuc Flux	Normal total flux (mol/(m^2*s)), Point: (5.5109E-16, 2.5)	WElectrode radius (μm), UME Radius
9	6	5000	41E-10		4	9	16	16	563	20	20	5	00
1.5100E-9	2.0000E-6	0.020000	-7.5904E-10	NaN	2.0263E-4	1.5100E-9	1.6719E-16	3.8496E-16	0.15290	9.1266E-20	1.6878E-20	4.9405E-5	1.0000
1.5100E-9	2.0000E-6	0.025000	-7.4975E-10	NaN	2.0066E-4	1.5100E-9	1.1702E-16	3.8438E-16	0.15101	9.0410E-20	1.6683E-20	4.8791E-5	1.0000
1.5100E-9	2.0000E-6	0.030000	-7.4316E-10	NaN	1.9922E-4	1.5100E-9	8.4866E-17	3.8356E-16	0.14967	8.9780E-20	1.6543E-20	4.8357E-5	1.0000
1.5100E-9	2.0000E-6	0.035000	-7.3850E-10	NaN	1.9817E-4	1.5100E-9	6.4680E-17	3.8267E-16	0.14872	8.9316E-20	1.6444E-20	4.8050E-5	1.0000
1.5100E-9	2.0000E-6	0.040000	-7.3497E-10	NaN	1.9735E-4	1.5100E-9	5.0820E-17	3.8187E-16	0.14801	8.8956E-20	1.6368E-20	4.7818E-5	1.0000
1.5100E-9	2.0000E-6	0.045000	-7.3192E-10	NaN	1.9665E-4	1.5100E-9	3.9067E-17	3.8118E-16	0.14739	8.8646E-20	1.6302E-20	4.7618E-5	1.0000
1.5100E-9	2.0000E-6	0.050000	-7.2958E-10	NaN	1.9610E-4	1.5100E-9	3.0873E-17	3.8058E-16	0.14692	8.8403E-20	1.6252E-20	4.7464E-5	1.0000
1.5100E-9	2.0000E-6	0.055000	-7.2765E-10	NaN	1.9564E-4	1.5100E-9	2.4578E-17	3.8005E-16	0.14652	8.8200E-20	1.6210E-20	4.7337E-5	1.0000
1.5100E-9	2.0000E-6	0.060000	-7.2609E-10	NaN	1.9526E-4	1.5100E-9	2.0031E-17	3.7956E-16	0.14621	8.8033E-20	1.6176E-20	4.7235E-5	1.0000

k0Sub (m/s)	d (m)	Time (s)	-(tds2.ntflux_Oxi)*n*F (A), UME	Surface Conc cOxi (M), Oxi Mem	Surface Conc cOxi (M), OxiNuc	Substrate Kinetics (m/s), k0Sub probe	Normal total flux (mol/s), UME OCR membrane	Normal total flux (mol/s), UME OCR nucleus	Concentration (mol/m^3), Point: (5.5109E-16, 2.5)	k0Sub*Oxi (mol/s), Mem Flux	k0Sub*Oxi (mol/s), Nuc Flux	Normal total flux (mol/(m^2*s)), Point: (5.5109E-16, 2.5)	WElectrode radius (µm), UME Radius
1.5100E-9	2.0000E-6	0.065000	-7.2498E-10	NaN	1.9499E-4	1.5100E-9	1.7653E-17	3.7915E-16	0.14598	8.7910E-20	1.6152E-20	4.7163E-5	1.0000
1.5100E-9	2.0000E-6	0.070000	-7.2376E-10	NaN	1.9469E-4	1.5100E-9	1.4429E-17	3.7875E-16	0.14574	8.7778E-20	1.6126E-20	4.7083E-5	1.0000
1.5100E-9	2.0000E-6	0.075000	-7.2270E-10	NaN	1.9443E-4	1.5100E-9	1.1756E-17	3.7839E-16	0.14552	8.7661E-20	1.6103E-20	4.7013E-5	1.0000
1.5100E-9	2.0000E-6	0.080000	-7.2178E-10	NaN	1.9420E-4	1.5100E-9	9.6361E-18	3.7806E-16	0.14534	8.7560E-20	1.6083E-20	4.6953E-5	1.0000
1.5100E-9	2.0000E-6	0.085000	-7.2101E-10	NaN	1.9401E-4	1.5100E-9	8.0681E-18	3.7777E-16	0.14518	8.7475E-20	1.6066E-20	4.6903E-5	1.0000
1.5100E-9	2.0000E-6	0.090000	-7.2040E-10	NaN	1.9385E-4	1.5100E-9	7.0523E-18	3.7752E-16	0.14506	8.7405E-20	1.6052E-20	4.6863E-5	1.0000
1.5100E-9	2.0000E-6	0.095000	-7.2001E-10	NaN	1.9375E-4	1.5100E-9	6.7313E-18	3.7733E-16	0.14498	8.7359E-20	1.6044E-20	4.6837E-5	1.0000
1.5100E-9	2.0000E-6	0.10000	-7.1970E-10	NaN	1.9367E-4	1.5100E-9	6.5932E-18	3.7718E-16	0.14492	8.7322E-20	1.6037E-20	4.6817E-5	1.0000
1.5100E-9	2.0000E-6	0.10500	-7.1940E-10	NaN	1.9359E-4	1.5100E-9	6.4551E-18	3.7702E-16	0.14486	8.7286E-20	1.6030E-20	4.6797E-5	1.0000
1.5100E-9	2.0000E-6	0.11000	-7.1909E-	NaN	1.9351E-4	1.5100E-9	6.3170E-18	3.7687E-16	0.14479	8.7249E-20	1.6023E-20	4.6777E-5	1.0000

k0Sub (m/s)	d (m)	Time (s)	-(tds2.ntflux_Oxi)*n*F (A), UME	Surface ConcOxi (M), Oxi Mem	Surface ConcOxi (M), OxiNuc	Substrate Kinetics (m/s), k0Sub probe	Normal total flux (mol/s), UME OCR membrane	Normal total flux (mol/s), UME OCR nucleus	Concentration (mol/m^3), Point: (5.5109E-16, 2.5)	k0Sub*Oxi (mol/s), Mem Flux	k0Sub*Oxi (mol/s), Nuc Flux	Normal total flux (mol/(m^2*s)), Point: (5.5109E-16, 2.5)	WElectrode radius (µm), UME Radius
			10										
1.5100E-9	2.0000E-6	0.11500	-7.1878E-10	NaN	1.9342E-4	1.5100E-9	6.1789E-18	3.7671E-16	0.14473	8.7212E-20	1.6016E-20	4.6758E-5	1.0000
1.5100E-9	2.0000E-6	0.12000	-7.1849E-10	NaN	1.9335E-4	1.5100E-9	6.0431E-18	3.7657E-16	0.14467	8.7178E-20	1.6010E-20	4.6739E-5	1.0000
1.5100E-9	2.0000E-6	0.12500	-7.1828E-10	NaN	1.9329E-4	1.5100E-9	5.8756E-18	3.7647E-16	0.14463	8.7153E-20	1.6005E-20	4.6725E-5	1.0000
1.5100E-9	2.0000E-6	0.13000	-7.1807E-10	NaN	1.9324E-4	1.5100E-9	5.7081E-18	3.7637E-16	0.14459	8.7128E-20	1.6001E-20	4.6711E-5	1.0000
1.5100E-9	2.0000E-6	0.13500	-7.1786E-10	NaN	1.9318E-4	1.5100E-9	5.5405E-18	3.7627E-16	0.14455	8.7103E-20	1.5996E-20	4.6697E-5	1.0000
1.5100E-9	2.0000E-6	0.14000	-7.1765E-10	NaN	1.9312E-4	1.5100E-9	5.3730E-18	3.7616E-16	0.14450	8.7078E-20	1.5991E-20	4.6683E-5	1.0000
1.5100E-9	2.0000E-6	0.14500	-7.1743E-10	NaN	1.9307E-4	1.5100E-9	5.2055E-18	3.7606E-16	0.14446	8.7053E-20	1.5987E-20	4.6670E-5	1.0000
1.5100E-9	2.0000E-6	0.15000	-7.1722E-10	NaN	1.9301E-4	1.5100E-9	5.0379E-18	3.7596E-16	0.14442	8.7028E-20	1.5982E-20	4.6656E-5	1.0000
1.5100E-9	2.0000E-6	0.15500	-7.1701E-10	NaN	1.9296E-4	1.5100E-9	4.8704E-18	3.7586E-16	0.14438	8.7003E-20	1.5977E-20	4.6642E-5	1.0000
1.5100E-	2.0000E-	0.16	-7.16	NaN	1.9290E-	1.5100E-	4.7029E-	3.7576E-	0.14	8.6978E-	1.5972E-	4.6628E-	1.00

k0Sub (m/s)	d (m)	Time (s)	-(tds2.ntflux_Oxi)*n*F (A), UME	Surface ConcOxi (M), Oxi Mem	Surface ConcOxi (M), OxiNuc	Substrate Kinetics (m/s), k0Sub probe	Normal total flux (mol/s), UME OCR membrane	Normal total flux (mol/s), UME OCR nucleus	Concentration (mol/m^3), Point: (5.5109E-16, 2.5)	k0Sub*Oxi (mol/s), Mem Flux	k0Sub*Oxi (mol/s), Nuc Flux	Normal total flux (mol/(m^2*s)), Point: (5.5109E-16, 2.5)	WElectrode radius (µm), UME Radius
9	6	000	80E-10		4	9	18	16	433	20	20	5	00
1.5100E-9	2.0000E-6	0.16500	-7.1659E-10	NaN	1.9285E-4	1.5100E-9	4.5353E-18	3.7566E-16	0.14429	8.6953E-20	1.5968E-20	4.6614E-5	1.0000
1.5100E-9	2.0000E-6	0.17000	-7.1637E-10	NaN	1.9279E-4	1.5100E-9	4.3678E-18	3.7556E-16	0.14425	8.6928E-20	1.5963E-20	4.6601E-5	1.0000
1.5100E-9	2.0000E-6	0.17500	-7.1620E-10	NaN	1.9274E-4	1.5100E-9	4.2365E-18	3.7548E-16	0.14421	8.6907E-20	1.5959E-20	4.6589E-5	1.0000
1.5100E-9	2.0000E-6	0.18000	-7.1607E-10	NaN	1.9271E-4	1.5100E-9	4.1433E-18	3.7542E-16	0.14419	8.6892E-20	1.5956E-20	4.6581E-5	1.0000
1.5100E-9	2.0000E-6	0.18500	-7.1595E-10	NaN	1.9268E-4	1.5100E-9	4.0501E-18	3.7536E-16	0.14416	8.6878E-20	1.5954E-20	4.6573E-5	1.0000
1.5100E-9	2.0000E-6	0.19000	-7.1583E-10	NaN	1.9265E-4	1.5100E-9	3.9569E-18	3.7530E-16	0.14414	8.6863E-20	1.5951E-20	4.6565E-5	1.0000
1.5100E-9	2.0000E-6	0.19500	-7.1571E-10	NaN	1.9262E-4	1.5100E-9	3.8637E-18	3.7525E-16	0.14411	8.6849E-20	1.5948E-20	4.6557E-5	1.0000
1.5100E-9	2.0000E-6	0.20000	-7.1558E-10	NaN	1.9258E-4	1.5100E-9	3.7705E-18	3.7519E-16	0.14409	8.6835E-20	1.5946E-20	4.6549E-5	1.0000
1.5100E-9	2.0000E-6	0.20500	-7.1546E-10	NaN	1.9255E-4	1.5100E-9	3.6772E-18	3.7513E-16	0.14406	8.6820E-20	1.5943E-20	4.6541E-5	1.0000

k0Sub (m/s)	d (m)	Time (s)	-(tds2.ntflux_Oxi)*n*F (A), UME	Surface ConcOxi (M), Oxi Mem	Surface ConcOxi (M), OxiNuc	Substrate Kinetics (m/s), k0Sub probe	Normal total flux (mol/s), UME OCR membrane	Normal total flux (mol/s), UME OCR nucleus	Concentration (mol/m^3), Point: (5.5109E-16, 2.5)	k0Sub*Oxi (mol/s), Mem Flux	k0Sub*Oxi (mol/s), Nuc Flux	Normal total flux (mol/(m^2*s)), Point: (5.5109E-16, 2.5)	WElectrode radius (µm), UME Radius
1.5100E-9	2.0000E-6	0.21000	-7.1534E-10	NaN	1.9252E-4	1.5100E-9	3.5840E-18	3.7507E-16	0.14404	8.6806E-20	1.5940E-20	4.6533E-5	1.0000
1.5100E-9	2.0000E-6	0.21500	-7.1522E-10	NaN	1.9249E-4	1.5100E-9	3.4908E-18	3.7501E-16	0.14401	8.6791E-20	1.5937E-20	4.6525E-5	1.0000
1.5100E-9	2.0000E-6	0.22000	-7.1510E-10	NaN	1.9246E-4	1.5100E-9	3.3976E-18	3.7496E-16	0.14399	8.6777E-20	1.5935E-20	4.6517E-5	1.0000
1.5100E-9	2.0000E-6	0.22500	-7.1497E-10	NaN	1.9242E-4	1.5100E-9	3.3044E-18	3.7490E-16	0.14396	8.6763E-20	1.5932E-20	4.6509E-5	1.0000
1.5100E-9	2.0000E-6	0.23000	-7.1485E-10	NaN	1.9239E-4	1.5100E-9	3.2112E-18	3.7484E-16	0.14394	8.6748E-20	1.5929E-20	4.6501E-5	1.0000
1.5100E-9	2.0000E-6	0.23500	-7.1473E-10	NaN	1.9236E-4	1.5100E-9	3.1180E-18	3.7478E-16	0.14392	8.6734E-20	1.5927E-20	4.6493E-5	1.0000
1.5100E-9	2.0000E-6	0.24000	-7.1461E-10	NaN	1.9233E-4	1.5100E-9	3.0248E-18	3.7473E-16	0.14389	8.6719E-20	1.5924E-20	4.6485E-5	1.0000
1.5100E-9	2.0000E-6	0.24500	-7.1448E-10	NaN	1.9230E-4	1.5100E-9	2.9316E-18	3.7467E-16	0.14387	8.6705E-20	1.5921E-20	4.6477E-5	1.0000
1.5100E-9	2.0000E-6	0.25000	-7.1436E-10	NaN	1.9226E-4	1.5100E-9	2.8384E-18	3.7461E-16	0.14384	8.6691E-20	1.5918E-20	4.6469E-5	1.0000
1.5100E-9	2.0000E-6	0.25000	-7.1436E-	NaN	1.9226E-4	1.5100E-9	2.8384E-18	3.7461E-16	0.14384	8.6691E-20	1.5918E-20	4.6469E-5	1.0000

k0Sub (m/s)	d (m)	Time (s)	-(tds2.ntflux_Oxi)*n*F (A), UME	Surface ConcOxi (M), OxiMem	Surface ConcOxi (M), OxiNuc	Substrate Kinetics (m/s), k0Sub probe	Normal total flux (mol/s), UME OCR membrane	Normal total flux (mol/s), UME OCR nucleus	Concentration (mol/m^3), Point: (5.5109E-16, 2.5)	k0Sub*Oxi (mol/s), Mem Flux	k0Sub*Oxi (mol/s), Nuc Flux	Normal total flux (mol/(m^2*s)), Point: (5.5109E-16, 2.5)	WElectrode radius (µm), UME Radius
			10										
1.5100E-9	2.0000E-6	0.27000	-7.1387E-10	NaN	1.9213E-4	1.5100E-9	2.4656E-18	3.7438E-16	0.14374	8.6633E-20	1.5908E-20	4.6438E-5	1.0000
1.5100E-9	2.0000E-6	0.29000	-7.1355E-10	NaN	1.9205E-4	1.5100E-9	2.2636E-18	3.7422E-16	0.14368	8.6595E-20	1.5901E-20	4.6417E-5	1.0000
1.5100E-9	2.0000E-6	0.31000	-7.1325E-10	NaN	1.9197E-4	1.5100E-9	2.0771E-18	3.7408E-16	0.14362	8.6559E-20	1.5894E-20	4.6397E-5	1.0000
1.5100E-9	2.0000E-6	0.33000	-7.1294E-10	NaN	1.9189E-4	1.5100E-9	1.8905E-18	3.7393E-16	0.14356	8.6523E-20	1.5887E-20	4.6377E-5	1.0000
1.5100E-9	2.0000E-6	0.35000	-7.1264E-10	NaN	1.9181E-4	1.5100E-9	1.7040E-18	3.7378E-16	0.14349	8.6487E-20	1.5880E-20	4.6357E-5	1.0000
1.5100E-9	2.0000E-6	0.37000	-7.1234E-10	NaN	1.9173E-4	1.5100E-9	1.5174E-18	3.7364E-16	0.14343	8.6451E-20	1.5874E-20	4.6338E-5	1.0000
1.5100E-9	2.0000E-6	0.39000	-7.1213E-10	NaN	1.9167E-4	1.5100E-9	1.4161E-18	3.7353E-16	0.14339	8.6426E-20	1.5869E-20	4.6324E-5	1.0000
1.5100E-9	2.0000E-6	0.41000	-7.1192E-10	NaN	1.9162E-4	1.5100E-9	1.3174E-18	3.7343E-16	0.14335	8.6402E-20	1.5864E-20	4.6311E-5	1.0000
1.5100E-9	2.0000E-6	0.43000	-7.1172E-10	NaN	1.9157E-4	1.5100E-9	1.2187E-18	3.7333E-16	0.14331	8.6377E-20	1.5860E-20	4.6297E-5	1.0000
1.5100E-	2.0000E-	0.45	-7.11	NaN	1.9151E-	1.5100E-	1.1200E-	3.7323E-	0.14	8.6353E-	1.5855E-	4.6284E-	1.00

k0Sub (m/s)	d (m)	Time (s)	-(tds2.ntflux_Oxi)*n*F (A), UME	Surface ConcOxi (M), Oxi Mem	Surface ConcOxi (M), OxiNuc	Substrate Kinetics (m/s), k0Sub probe	Normal total flux (mol/s), UME OCR membrane	Normal total flux (mol/s), UME OCR nucleus	Concentration (mol/m^3), Point: (5.5109E-16, 2.5)	k0Sub*Oxi (mol/s), Mem Flux	k0Sub*Oxi (mol/s), Nuc Flux	Normal total flux (mol/(m^2*s)), Point: (5.5109E-16, 2.5)	WElectrode radius (µm), UME Radius
9	6	000	51E-10		4	9	18	16	327	20	20	5	00
1.5100E-9	2.0000E-6	0.47000	-7.1131E-10	NaN	1.9146E-4	1.5100E-9	1.0213E-18	3.7313E-16	0.14323	8.6328E-20	1.5851E-20	4.6271E-5	1.0000
1.5100E-9	2.0000E-6	0.49000	-7.1116E-10	NaN	1.9142E-4	1.5100E-9	9.6513E-19	3.7306E-16	0.14320	8.6311E-20	1.5847E-20	4.6261E-5	1.0000
1.5100E-9	2.0000E-6	0.51000	-7.1101E-10	NaN	1.9138E-4	1.5100E-9	9.0792E-19	3.7298E-16	0.14317	8.6293E-20	1.5844E-20	4.6251E-5	1.0000
1.5100E-9	2.0000E-6	0.53000	-7.1086E-10	NaN	1.9134E-4	1.5100E-9	8.5071E-19	3.7291E-16	0.14314	8.6275E-20	1.5841E-20	4.6242E-5	1.0000
1.5100E-9	2.0000E-6	0.55000	-7.1072E-10	NaN	1.9130E-4	1.5100E-9	7.9350E-19	3.7284E-16	0.14311	8.6257E-20	1.5838E-20	4.6232E-5	1.0000
1.5100E-9	2.0000E-6	0.57000	-7.1057E-10	NaN	1.9126E-4	1.5100E-9	7.3786E-19	3.7276E-16	0.14308	8.6240E-20	1.5834E-20	4.6222E-5	1.0000
1.5100E-9	2.0000E-6	0.59000	-7.1045E-10	NaN	1.9123E-4	1.5100E-9	6.9998E-19	3.7270E-16	0.14305	8.6226E-20	1.5832E-20	4.6215E-5	1.0000
1.5100E-9	2.0000E-6	0.61000	-7.1034E-10	NaN	1.9120E-4	1.5100E-9	6.6210E-19	3.7265E-16	0.14303	8.6212E-20	1.5829E-20	4.6207E-5	1.0000
1.5100E-9	2.0000E-6	0.63000	-7.1022E-10	NaN	1.9117E-4	1.5100E-9	6.2422E-19	3.7259E-16	0.14301	8.6198E-20	1.5827E-20	4.6200E-5	1.0000

k0Sub (m/s)	d (m)	Time (s)	-(tds2.ntflux_Oxi)*n*F (A), UME	Surface ConcOxi (M), Oxi Mem	Surface ConcOxi (M), OxiNuc	Substrate Kinetics (m/s), k0Sub probe	Normal total flux (mol/s), UME OCR membrane	Normal total flux (mol/s), UME OCR nucleus	Concentration (mol/m^3), Point: (5.5109E-16, 2.5)	k0Sub* Oxi (mol/s), Mem Flux	k0Sub* Oxi (mol/s), Nuc Flux	Normal total flux (mol/(m^2*s)), Point: (5.5109E-16, 2.5)	WElectrode radius (µm), UME Radius
1.5100E-9	2.0000E-6	0.65000	-7.1010E-10	NaN	1.9114E-4	1.5100E-9	5.8634E-19	3.7253E-16	0.14298	8.6184E-20	1.5824E-20	4.6192E-5	1.0000
1.5100E-9	2.0000E-6	0.67000	-7.0999E-10	NaN	1.9111E-4	1.5100E-9	5.5027E-19	3.7247E-16	0.14296	8.6170E-20	1.5821E-20	4.6185E-5	1.0000
1.5100E-9	2.0000E-6	0.69000	-7.0990E-10	NaN	1.9108E-4	1.5100E-9	5.2568E-19	3.7243E-16	0.14294	8.6159E-20	1.5819E-20	4.6179E-5	1.0000
1.5100E-9	2.0000E-6	0.71000	-7.0981E-10	NaN	1.9106E-4	1.5100E-9	5.0109E-19	3.7238E-16	0.14292	8.6148E-20	1.5817E-20	4.6173E-5	1.0000
1.5100E-9	2.0000E-6	0.73000	-7.0972E-10	NaN	1.9103E-4	1.5100E-9	4.7650E-19	3.7233E-16	0.14290	8.6137E-20	1.5815E-20	4.6167E-5	1.0000
1.5100E-9	2.0000E-6	0.75000	-7.0962E-10	NaN	1.9101E-4	1.5100E-9	4.5191E-19	3.7229E-16	0.14289	8.6127E-20	1.5813E-20	4.6161E-5	1.0000
1.5100E-9	2.0000E-6	0.77000	-7.0953E-10	NaN	1.9099E-4	1.5100E-9	4.2804E-19	3.7224E-16	0.14287	8.6116E-20	1.5811E-20	4.6155E-5	1.0000
1.5100E-9	2.0000E-6	0.79000	-7.0945E-10	NaN	1.9096E-4	1.5100E-9	4.0811E-19	3.7220E-16	0.14285	8.6106E-20	1.5810E-20	4.6150E-5	1.0000
1.5100E-9	2.0000E-6	0.81000	-7.0938E-10	NaN	1.9094E-4	1.5100E-9	3.8965E-19	3.7216E-16	0.14284	8.6097E-20	1.5808E-20	4.6145E-5	1.0000
1.5100E-9	2.0000E-6	0.83000	-7.0930E-	NaN	1.9092E-4	1.5100E-9	3.7267E-19	3.7212E-16	0.14282	8.6087E-20	1.5806E-20	4.6140E-5	1.0000

k0Sub (m/s)	d (m)	Time (s)	-(tds2.ntflux_Oxi)*n*F (A), UME	Surface ConcOxi (M), OxiMem	Surface ConcOxi (M), OxiNuc	Substrate Kinetics (m/s), k0Sub probe	Normal total flux (mol/s), UME OCR membrane	Normal total flux (mol/s), UME OCR nucleus	Concentration (mol/m^3), Point: (5.5109E-16, 2.5)	k0Sub*Oxi (mol/s), Mem Flux	k0Sub*Oxi (mol/s), Nuc Flux	Normal total flux (mol/(m^2*s)), Point: (5.5109E-16, 2.5)	WElectrode radius (µm), UME Radius
			10										
1.5100E-9	2.0000E-6	0.85000	-7.0923E-10	NaN	1.9090E-4	1.5100E-9	3.5716E-19	3.7208E-16	0.14281	8.6079E-20	1.5805E-20	4.6135E-5	1.0000
1.5100E-9	2.0000E-6	0.87000	-7.0916E-10	NaN	1.9088E-4	1.5100E-9	3.4281E-19	3.7205E-16	0.14279	8.6070E-20	1.5803E-20	4.6131E-5	1.0000
1.5100E-9	2.0000E-6	0.89000	-7.0909E-10	NaN	1.9087E-4	1.5100E-9	3.2854E-19	3.7201E-16	0.14278	8.6062E-20	1.5801E-20	4.6126E-5	1.0000
1.5100E-9	2.0000E-6	0.91000	-7.0903E-10	NaN	1.9085E-4	1.5100E-9	3.1520E-19	3.7198E-16	0.14277	8.6054E-20	1.5800E-20	4.6122E-5	1.0000
1.5100E-9	2.0000E-6	0.93000	-7.0896E-10	NaN	1.9083E-4	1.5100E-9	3.0281E-19	3.7195E-16	0.14275	8.6047E-20	1.5799E-20	4.6118E-5	1.0000
1.5100E-9	2.0000E-6	0.95000	-7.0890E-10	NaN	1.9082E-4	1.5100E-9	2.9135E-19	3.7192E-16	0.14274	8.6040E-20	1.5797E-20	4.6114E-5	1.0000
1.5100E-9	2.0000E-6	0.97000	-7.0884E-10	NaN	1.9080E-4	1.5100E-9	2.8062E-19	3.7189E-16	0.14273	8.6033E-20	1.5796E-20	4.6110E-5	1.0000
1.5100E-9	2.0000E-6	0.99000	-7.0879E-10	NaN	1.9079E-4	1.5100E-9	2.7009E-19	3.7186E-16	0.14272	8.6026E-20	1.5795E-20	4.6106E-5	1.0000
1.5100E-9	2.4000E-6	0.0000	-7.1527E-9	NaN	2.2700E-4	1.5100E-9	-2.0267E-20	-1.2710E-20	0.22700	1.0088E-19	2.0267E-20	-1.8988E-10	1.0000
1.5100E-	2.4000E-	0.005000	-8.41	NaN	2.1747E-	1.5100E-	6.1742E-	3.0174E-	0.18	9.7393E-	1.8670E-	3.9191E-	1.00

k0Sub (m/s)	d (m)	Time (s)	-(tds2.ntflux_Oxi)*n*F (A), UME	Surface ConcOxi (M), OxiMem	Surface ConcOxi (M), OxiNuc	Substrate Kinetics (m/s), k0Sub probe	Normal total flux (mol/s), UME OCR membrane	Normal total flux (mol/s), UME OCR nucleus	Concentration (mol/m^3), Point: (5.5109E-16, 2.5)	k0Sub*Oxi (mol/s), Mem Flux	k0Sub*Oxi (mol/s), Nuc Flux	Normal total flux (mol/(m^2*s)), Point: (5.5109E-16, 2.5)	WElectrode radius (µm), UME Radius
9	6	0	34E-10		4	9	16	16	066	20	20	5	00
1.5100E-9	2.4000E-6	0.010000	-8.0248E-10	NaN	2.1140E-4	1.5100E-9	3.5972E-16	3.2589E-16	0.17208	9.4898E-20	1.7922E-20	3.7333E-5	1.0000
1.5100E-9	2.4000E-6	0.015000	-7.8293E-10	NaN	2.0758E-4	1.5100E-9	2.3203E-16	3.2901E-16	0.16782	9.3269E-20	1.7516E-20	3.6406E-5	1.0000
1.5100E-9	2.4000E-6	0.020000	-7.7059E-10	NaN	2.0498E-4	1.5100E-9	1.6079E-16	3.2909E-16	0.16514	9.2143E-20	1.7254E-20	3.5823E-5	1.0000
1.5100E-9	2.4000E-6	0.025000	-7.6172E-10	NaN	2.0308E-4	1.5100E-9	1.1478E-16	3.2901E-16	0.16322	9.1317E-20	1.7066E-20	3.5405E-5	1.0000
1.5100E-9	2.4000E-6	0.030000	-7.5524E-10	NaN	2.0166E-4	1.5100E-9	8.4229E-17	3.2864E-16	0.16182	9.0696E-20	1.6927E-20	3.5101E-5	1.0000
1.5100E-9	2.4000E-6	0.035000	-7.5039E-10	NaN	2.0057E-4	1.5100E-9	6.3511E-17	3.2817E-16	0.16077	9.0218E-20	1.6823E-20	3.4873E-5	1.0000
1.5100E-9	2.4000E-6	0.040000	-7.4638E-10	NaN	1.9966E-4	1.5100E-9	4.6944E-17	3.2772E-16	0.15990	8.9820E-20	1.6737E-20	3.4684E-5	1.0000
1.5100E-9	2.4000E-6	0.045000	-7.4348E-10	NaN	1.9899E-4	1.5100E-9	3.6450E-17	3.2726E-16	0.15927	8.9522E-20	1.6674E-20	3.4548E-5	1.0000
1.5100E-9	2.4000E-6	0.050000	-7.4096E-10	NaN	1.9840E-4	1.5100E-9	2.7662E-17	3.2683E-16	0.15873	8.9263E-20	1.6620E-20	3.4430E-5	1.0000

k0Sub (m/s)	d (m)	Time (s)	-(tds2.ntflux_Oxi)*n*F (A), UME	Surface ConcOxi (M), Oxi Mem	Surface ConcOxi (M), OxiNuc	Substrate Kinetics (m/s), k0Sub probe	Normal total flux (mol/s), UME OCR membrane	Normal total flux (mol/s), UME OCR nucleus	Concentration (mol/m^3), Point: (5.5109E-16, 2.5)	k0Sub*Oxi (mol/s), Mem Flux	k0Sub*Oxi (mol/s), Nuc Flux	Normal total flux (mol/(m^2*s)), Point: (5.5109E-16, 2.5)	WElectrode radius (µm), UME Radius
1.5100E-9	2.4000E-6	0.055000	-7.3910E-10	NaN	1.9795E-4	1.5100E-9	2.2219E-17	3.2641E-16	0.15833	8.9065E-20	1.6579E-20	3.4343E-5	1.0000
1.5100E-9	2.4000E-6	0.060000	-7.3780E-10	NaN	1.9762E-4	1.5100E-9	1.9559E-17	3.2600E-16	0.15805	8.8920E-20	1.6550E-20	3.4282E-5	1.0000
1.5100E-9	2.4000E-6	0.065000	-7.3651E-10	NaN	1.9731E-4	1.5100E-9	1.6483E-17	3.2565E-16	0.15777	8.8779E-20	1.6522E-20	3.4222E-5	1.0000
1.5100E-9	2.4000E-6	0.070000	-7.3553E-10	NaN	1.9706E-4	1.5100E-9	1.4688E-17	3.2534E-16	0.15756	8.8669E-20	1.6500E-20	3.4176E-5	1.0000
1.5100E-9	2.4000E-6	0.075000	-7.3471E-10	NaN	1.9685E-4	1.5100E-9	1.3449E-17	3.2505E-16	0.15739	8.8575E-20	1.6482E-20	3.4138E-5	1.0000
1.5100E-9	2.4000E-6	0.080000	-7.3393E-10	NaN	1.9665E-4	1.5100E-9	1.2153E-17	3.2479E-16	0.15722	8.8487E-20	1.6465E-20	3.4102E-5	1.0000
1.5100E-9	2.4000E-6	0.085000	-7.3329E-10	NaN	1.9649E-4	1.5100E-9	1.1269E-17	3.2457E-16	0.15708	8.8414E-20	1.6450E-20	3.4072E-5	1.0000
1.5100E-9	2.4000E-6	0.090000	-7.3266E-10	NaN	1.9633E-4	1.5100E-9	1.0339E-17	3.2435E-16	0.15695	8.8342E-20	1.6437E-20	3.4043E-5	1.0000
1.5100E-9	2.4000E-6	0.095000	-7.3206E-10	NaN	1.9618E-4	1.5100E-9	9.4214E-18	3.2415E-16	0.15682	8.8275E-20	1.6423E-20	3.4015E-5	1.0000
1.5100E-9	2.4000E-6	0.10000	-7.3151E-	NaN	1.9604E-4	1.5100E-9	8.5941E-18	3.2396E-16	0.15670	8.8212E-20	1.6411E-20	3.3989E-5	1.0000

k0Sub (m/s)	d (m)	Time (s)	-(tds2.ntflux_Oxi)*n*F (A), UME	Surface ConcOxi (M), OxiMem	Surface ConcOxi (M), OxiNuc	Substrate Kinetics (m/s), k0Sub probe	Normal total flux (mol/s), UME OCR membrane	Normal total flux (mol/s), UME OCR nucleus	Concentration (mol/m^3), Point: (5.5109E-16, 2.5)	k0Sub*Oxi (mol/s), Mem Flux	k0Sub*Oxi (mol/s), Nuc Flux	Normal total flux (mol/(m^2*s)), Point: (5.5109E-16, 2.5)	WElectrode radius (µm), UME Radius
			10										
1.5100E-9	2.4000E-6	0.10500	-7.3101E-10	NaN	1.9591E-4	1.5100E-9	7.8572E-18	3.2379E-16	0.15659	8.8155E-20	1.6400E-20	3.3966E-5	1.0000
1.5100E-9	2.4000E-6	0.11000	-7.3057E-10	NaN	1.9580E-4	1.5100E-9	7.2105E-18	3.2364E-16	0.15650	8.8104E-20	1.6390E-20	3.3945E-5	1.0000
1.5100E-9	2.4000E-6	0.11500	-7.3022E-10	NaN	1.9571E-4	1.5100E-9	6.7790E-18	3.2352E-16	0.15642	8.8065E-20	1.6383E-20	3.3929E-5	1.0000
1.5100E-9	2.4000E-6	0.12000	-7.2999E-10	NaN	1.9565E-4	1.5100E-9	6.5594E-18	3.2343E-16	0.15637	8.8038E-20	1.6377E-20	3.3918E-5	1.0000
1.5100E-9	2.4000E-6	0.12500	-7.2976E-10	NaN	1.9559E-4	1.5100E-9	6.3398E-18	3.2334E-16	0.15632	8.8011E-20	1.6372E-20	3.3907E-5	1.0000
1.5100E-9	2.4000E-6	0.13000	-7.2953E-10	NaN	1.9553E-4	1.5100E-9	6.1203E-18	3.2326E-16	0.15627	8.7985E-20	1.6367E-20	3.3897E-5	1.0000
1.5100E-9	2.4000E-6	0.13500	-7.2930E-10	NaN	1.9547E-4	1.5100E-9	5.9007E-18	3.2317E-16	0.15623	8.7958E-20	1.6362E-20	3.3886E-5	1.0000
1.5100E-9	2.4000E-6	0.14000	-7.2907E-10	NaN	1.9541E-4	1.5100E-9	5.6811E-18	3.2308E-16	0.15618	8.7931E-20	1.6357E-20	3.3875E-5	1.0000
1.5100E-9	2.4000E-6	0.14500	-7.2884E-10	NaN	1.9535E-4	1.5100E-9	5.4615E-18	3.2300E-16	0.15613	8.7904E-20	1.6352E-20	3.3864E-5	1.0000
1.5100E-	2.4000E-	0.15	-7.28	NaN	1.9529E-	1.5100E-	5.2419E-	3.2291E-	0.15	8.7878E-	1.6347E-	3.3854E-	1.00

k0Sub (m/s)	d (m)	Time (s)	-(tds2.ntflux_Oxi)*n*F (A), UME	Surface ConcOxi (M), Oxi Mem	Surface ConcOxi (M), OxiNuc	Substrate Kinetics (m/s), k0Sub probe	Normal total flux (mol/s), UME OCR membrane	Normal total flux (mol/s), UME OCR nucleus	Concentration (mol/m^3), Point: (5.5109E-16, 2.5)	k0Sub*Oxi (mol/s), Mem Flux	k0Sub*Oxi (mol/s), Nuc Flux	Normal total flux (mol/(m^2*s)), Point: (5.5109E-16, 2.5)	WElectrode radius (µm), UME Radius
9	6	000	61E-10		4	9	18	16	608	20	20	5	00
1.5100E-9	2.4000E-6	0.15500	-7.2838E-10	NaN	1.9523E-4	1.5100E-9	5.0223E-18	3.2282E-16	0.15603	8.7851E-20	1.6342E-20	3.3843E-5	1.0000
1.5100E-9	2.4000E-6	0.16000	-7.2815E-10	NaN	1.9517E-4	1.5100E-9	4.8027E-18	3.2274E-16	0.15598	8.7824E-20	1.6336E-20	3.3832E-5	1.0000
1.5100E-9	2.4000E-6	0.16500	-7.2792E-10	NaN	1.9511E-4	1.5100E-9	4.5831E-18	3.2265E-16	0.15593	8.7797E-20	1.6331E-20	3.3822E-5	1.0000
1.5100E-9	2.4000E-6	0.17000	-7.2773E-10	NaN	1.9506E-4	1.5100E-9	4.4165E-18	3.2257E-16	0.15589	8.7775E-20	1.6327E-20	3.3813E-5	1.0000
1.5100E-9	2.4000E-6	0.17500	-7.2757E-10	NaN	1.9502E-4	1.5100E-9	4.2887E-18	3.2251E-16	0.15585	8.7757E-20	1.6324E-20	3.3805E-5	1.0000
1.5100E-9	2.4000E-6	0.18000	-7.2741E-10	NaN	1.9498E-4	1.5100E-9	4.1610E-18	3.2245E-16	0.15582	8.7739E-20	1.6320E-20	3.3798E-5	1.0000
1.5100E-9	2.4000E-6	0.18500	-7.2726E-10	NaN	1.9494E-4	1.5100E-9	4.0332E-18	3.2239E-16	0.15579	8.7720E-20	1.6317E-20	3.3791E-5	1.0000
1.5100E-9	2.4000E-6	0.19000	-7.2710E-10	NaN	1.9490E-4	1.5100E-9	3.9054E-18	3.2233E-16	0.15575	8.7702E-20	1.6313E-20	3.3784E-5	1.0000
1.5100E-9	2.4000E-6	0.19500	-7.2695E-10	NaN	1.9486E-4	1.5100E-9	3.7777E-18	3.2227E-16	0.15572	8.7684E-20	1.6310E-20	3.3776E-5	1.0000

k0Sub (m/s)	d (m)	Time (s)	-(tds2.ntflux_Oxi)*n*F (A), UME	Surface ConcOxi (M), Oxi Mem	Surface ConcOxi (M), OxiNuc	Substrate Kinetics (m/s), k0Sub probe	Normal total flux (mol/s), UME OCR membrane	Normal total flux (mol/s), UME OCR nucleus	Concentration (mol/m^3), Point: (5.5109E-16, 2.5)	k0Sub*Oxi (mol/s), Mem Flux	k0Sub*Oxi (mol/s), Nuc Flux	Normal total flux (mol/(m^2*s)), Point: (5.5109E-16, 2.5)	WElectrode radius (µm), UME Radius
1.5100E-9	2.4000E-6	0.20000	-7.2679E-10	NaN	1.9482E-4	1.5100E-9	3.6499E-18	3.2221E-16	0.15569	8.7666E-20	1.6306E-20	3.3769E-5	1.0000
1.5100E-9	2.4000E-6	0.20500	-7.2664E-10	NaN	1.9478E-4	1.5100E-9	3.5222E-18	3.2215E-16	0.15565	8.7648E-20	1.6303E-20	3.3762E-5	1.0000
1.5100E-9	2.4000E-6	0.21000	-7.2648E-10	NaN	1.9474E-4	1.5100E-9	3.3944E-18	3.2209E-16	0.15562	8.7629E-20	1.6299E-20	3.3755E-5	1.0000
1.5100E-9	2.4000E-6	0.21500	-7.2632E-10	NaN	1.9470E-4	1.5100E-9	3.2667E-18	3.2203E-16	0.15559	8.7611E-20	1.6296E-20	3.3747E-5	1.0000
1.5100E-9	2.4000E-6	0.22000	-7.2617E-10	NaN	1.9465E-4	1.5100E-9	3.1389E-18	3.2197E-16	0.15555	8.7593E-20	1.6292E-20	3.3740E-5	1.0000
1.5100E-9	2.4000E-6	0.22500	-7.2605E-10	NaN	1.9462E-4	1.5100E-9	3.0590E-18	3.2193E-16	0.15553	8.7579E-20	1.6290E-20	3.3735E-5	1.0000
1.5100E-9	2.4000E-6	0.23000	-7.2596E-10	NaN	1.9460E-4	1.5100E-9	2.9969E-18	3.2189E-16	0.15551	8.7568E-20	1.6288E-20	3.3730E-5	1.0000
1.5100E-9	2.4000E-6	0.23500	-7.2586E-10	NaN	1.9457E-4	1.5100E-9	2.9348E-18	3.2185E-16	0.15549	8.7557E-20	1.6286E-20	3.3726E-5	1.0000
1.5100E-9	2.4000E-6	0.24000	-7.2577E-10	NaN	1.9455E-4	1.5100E-9	2.8727E-18	3.2181E-16	0.15547	8.7546E-20	1.6283E-20	3.3721E-5	1.0000
1.5100E-9	2.4000E-6	0.24500	-7.2567E-	NaN	1.9452E-4	1.5100E-9	2.8106E-18	3.2178E-16	0.15545	8.7535E-20	1.6281E-20	3.3717E-5	1.0000

k0Sub (m/s)	d (m)	Time (s)	-(tds2.ntflux_Oxi)*n*F (A), UME	Surface ConcOxi (M), Oxi Mem	Surface ConcOxi (M), OxiNuc	Substrate Kinetics (m/s), k0Sub probe	Normal total flux (mol/s), UME OCR membrane	Normal total flux (mol/s), UME OCR nucleus	Concentration (mol/m^3), Point: (5.5109E-16, 2.5)	k0Sub*Oxi (mol/s), Mem Flux	k0Sub*Oxi (mol/s), Nuc Flux	Normal total flux (mol/(m^2*s)), Point: (5.5109E-16, 2.5)	WElectrode radius (µm), UME Radius
			10										
1.5100E-9	2.4000E-6	0.25000	-7.2558E-10	NaN	1.9450E-4	1.5100E-9	2.7485E-18	3.2174E-16	0.15543	8.7524E-20	1.6279E-20	3.3713E-5	1.0000
1.5100E-9	2.4000E-6	0.25000	-7.2558E-10	NaN	1.9450E-4	1.5100E-9	2.7485E-18	3.2174E-16	0.15543	8.7524E-20	1.6279E-20	3.3713E-5	1.0000
1.5100E-9	2.4000E-6	0.27000	-7.2520E-10	NaN	1.9440E-4	1.5100E-9	2.5000E-18	3.2159E-16	0.15535	8.7479E-20	1.6271E-20	3.3695E-5	1.0000
1.5100E-9	2.4000E-6	0.29000	-7.2482E-10	NaN	1.9430E-4	1.5100E-9	2.2515E-18	3.2144E-16	0.15526	8.7435E-20	1.6262E-20	3.3677E-5	1.0000
1.5100E-9	2.4000E-6	0.31000	-7.2445E-10	NaN	1.9420E-4	1.5100E-9	2.0030E-18	3.2129E-16	0.15518	8.7390E-20	1.6254E-20	3.3660E-5	1.0000
1.5100E-9	2.4000E-6	0.33000	-7.2413E-10	NaN	1.9412E-4	1.5100E-9	1.8127E-18	3.2116E-16	0.15512	8.7353E-20	1.6247E-20	3.3645E-5	1.0000
1.5100E-9	2.4000E-6	0.35000	-7.2388E-10	NaN	1.9405E-4	1.5100E-9	1.6837E-18	3.2106E-16	0.15506	8.7323E-20	1.6241E-20	3.3633E-5	1.0000
1.5100E-9	2.4000E-6	0.37000	-7.2363E-10	NaN	1.9399E-4	1.5100E-9	1.5546E-18	3.2096E-16	0.15501	8.7294E-20	1.6236E-20	3.3622E-5	1.0000
1.5100E-9	2.4000E-6	0.39000	-7.2338E-10	NaN	1.9392E-4	1.5100E-9	1.4256E-18	3.2085E-16	0.15496	8.7265E-20	1.6230E-20	3.3610E-5	1.0000
1.5100E-	2.4000E-	0.41	-7.23	NaN	1.9386E-	1.5100E-	1.2966E-	3.2075E-	0.15	8.7235E-	1.6225E-	3.3599E-	1.00

k0Sub (m/s)	d (m)	Time (s)	-(tds2.ntflux_Oxi)*n*F (A), UME	Surface ConcOxi (M), Oxi Mem	Surface ConcOxi (M), OxiNuc	Substrate Kinetics (m/s), k0Sub probe	Normal total flux (mol/s), UME OCR membrane	Normal total flux (mol/s), UME OCR nucleus	Concentration (mol/m^3), Point: (5.5109E-16, 2.5)	k0Sub*Oxi (mol/s), Mem Flux	k0Sub*Oxi (mol/s), Nuc Flux	Normal total flux (mol/(m^2*s)), Point: (5.5109E-16, 2.5)	WEIectrode radius (μm), UME Radius
9	6	000	13E-10		4	9	18	16	490	20	20	5	00
1.5100E-9	2.4000E-6	0.43000	-7.2292E-10	NaN	1.9380E-4	1.5100E-9	1.1979E-18	3.2067E-16	0.15486	8.7210E-20	1.6220E-20	3.3589E-5	1.0000
1.5100E-9	2.4000E-6	0.45000	-7.2274E-10	NaN	1.9375E-4	1.5100E-9	1.1248E-18	3.2059E-16	0.15482	8.7189E-20	1.6216E-20	3.3581E-5	1.0000
1.5100E-9	2.4000E-6	0.47000	-7.2257E-10	NaN	1.9371E-4	1.5100E-9	1.0516E-18	3.2052E-16	0.15478	8.7168E-20	1.6212E-20	3.3573E-5	1.0000
1.5100E-9	2.4000E-6	0.49000	-7.2239E-10	NaN	1.9366E-4	1.5100E-9	9.7854E-19	3.2045E-16	0.15474	8.7147E-20	1.6208E-20	3.3564E-5	1.0000
1.5100E-9	2.4000E-6	0.51000	-7.2222E-10	NaN	1.9362E-4	1.5100E-9	9.0543E-19	3.2037E-16	0.15471	8.7127E-20	1.6204E-20	3.3556E-5	1.0000
1.5100E-9	2.4000E-6	0.53000	-7.2207E-10	NaN	1.9358E-4	1.5100E-9	8.4924E-19	3.2031E-16	0.15467	8.7109E-20	1.6201E-20	3.3549E-5	1.0000
1.5100E-9	2.4000E-6	0.55000	-7.2193E-10	NaN	1.9354E-4	1.5100E-9	8.0437E-19	3.2025E-16	0.15464	8.7093E-20	1.6198E-20	3.3543E-5	1.0000
1.5100E-9	2.4000E-6	0.57000	-7.2180E-10	NaN	1.9351E-4	1.5100E-9	7.5949E-19	3.2020E-16	0.15462	8.7077E-20	1.6195E-20	3.3537E-5	1.0000
1.5100E-9	2.4000E-6	0.59000	-7.2167E-10	NaN	1.9347E-4	1.5100E-9	7.1462E-19	3.2014E-16	0.15459	8.7062E-20	1.6192E-20	3.3531E-5	1.0000

k0Sub (m/s)	d (m)	Time (s)	-(tds2.ntflux_Oxi)*n*F (A), UME	Surface Con cOxi (M), Oxi Mem	Surface Con cOxi (M), OxiNuc	Substrate Kinetics (m/s), k0Sub probe	Normal total flux (mol/s), UME OCR membrane	Normal total flux (mol/s), UME OCR nucleus	Concentration (mol/m^3), Point: (5.5109E-16, 2.5)	k0Sub*Oxi (mol/s), Mem Flux	k0Sub*Oxi (mol/s), Nuc Flux	Normal total flux (mol/(m^2*s)), Point: (5.5109E-16, 2.5)	WEIectrode radius (µm), UME Radius
1.5100E-9	2.4000E-6	0.61000	-7.2154E-10	NaN	1.9344E-4	1.5100E-9	6.6975E-19	3.2009E-16	0.15456	8.7046E-20	1.6189E-20	3.3525E-5	1.0000
1.5100E-9	2.4000E-6	0.63000	-7.2142E-10	NaN	1.9340E-4	1.5100E-9	6.3410E-19	3.2004E-16	0.15454	8.7032E-20	1.6186E-20	3.3519E-5	1.0000
1.5100E-9	2.4000E-6	0.65000	-7.2132E-10	NaN	1.9338E-4	1.5100E-9	6.0326E-19	3.1999E-16	0.15451	8.7020E-20	1.6184E-20	3.3514E-5	1.0000
1.5100E-9	2.4000E-6	0.67000	-7.2121E-10	NaN	1.9335E-4	1.5100E-9	5.7243E-19	3.1995E-16	0.15449	8.7007E-20	1.6182E-20	3.3509E-5	1.0000
1.5100E-9	2.4000E-6	0.69000	-7.2111E-10	NaN	1.9332E-4	1.5100E-9	5.4159E-19	3.1991E-16	0.15447	8.6994E-20	1.6179E-20	3.3505E-5	1.0000
1.5100E-9	2.4000E-6	0.71000	-7.2100E-10	NaN	1.9329E-4	1.5100E-9	5.1075E-19	3.1986E-16	0.15445	8.6982E-20	1.6177E-20	3.3500E-5	1.0000
1.5100E-9	2.4000E-6	0.73000	-7.2091E-10	NaN	1.9327E-4	1.5100E-9	4.8707E-19	3.1982E-16	0.15443	8.6971E-20	1.6175E-20	3.3495E-5	1.0000
1.5100E-9	2.4000E-6	0.75000	-7.2083E-10	NaN	1.9325E-4	1.5100E-9	4.6625E-19	3.1979E-16	0.15441	8.6961E-20	1.6173E-20	3.3491E-5	1.0000
1.5100E-9	2.4000E-6	0.77000	-7.2074E-10	NaN	1.9322E-4	1.5100E-9	4.4544E-19	3.1975E-16	0.15439	8.6951E-20	1.6171E-20	3.3488E-5	1.0000
1.5100E-9	2.4000E-6	0.79000	-7.2066E-	NaN	1.9320E-4	1.5100E-9	4.2463E-19	3.1971E-16	0.15437	8.6941E-20	1.6169E-20	3.3484E-5	1.0000

k0Sub (m/s)	d (m)	Time (s)	-(tds2.ntflux_Oxi)*n*F (A), UME	Surface ConcOxi (M), Oxi Mem	Surface ConcOxi (M), OxiNuc	Substrate Kinetics (m/s), k0Sub probe	Normal total flux (mol/s), UME OCR membrane	Normal total flux (mol/s), UME OCR nucleus	Concentration (mol/m^3), Point: (5.5109E-16, 2.5)	k0Sub*Oxi (mol/s), Mem Flux	k0Sub*Oxi (mol/s), Nuc Flux	Normal total flux (mol/(m^2*s)), Point: (5.5109E-16, 2.5)	WElectrode radius (μm), UME Radius
			10										
1.5100E-9	2.4000E-6	0.81000	-7.2057E-10	NaN	1.9318E-4	1.5100E-9	4.0381E-19	3.1968E-16	0.15435	8.6931E-20	1.6167E-20	3.3480E-5	1.0000
1.5100E-9	2.4000E-6	0.83000	-7.2049E-10	NaN	1.9316E-4	1.5100E-9	3.8552E-19	3.1964E-16	0.15434	8.6921E-20	1.6166E-20	3.3476E-5	1.0000
1.5100E-9	2.4000E-6	0.85000	-7.2042E-10	NaN	1.9314E-4	1.5100E-9	3.6900E-19	3.1961E-16	0.15432	8.6912E-20	1.6164E-20	3.3473E-5	1.0000
1.5100E-9	2.4000E-6	0.87000	-7.2035E-10	NaN	1.9312E-4	1.5100E-9	3.5362E-19	3.1958E-16	0.15430	8.6904E-20	1.6162E-20	3.3469E-5	1.0000
1.5100E-9	2.4000E-6	0.89000	-7.2028E-10	NaN	1.9310E-4	1.5100E-9	3.3939E-19	3.1955E-16	0.15429	8.6895E-20	1.6161E-20	3.3466E-5	1.0000
1.5100E-9	2.4000E-6	0.91000	-7.2021E-10	NaN	1.9308E-4	1.5100E-9	3.2630E-19	3.1952E-16	0.15428	8.6887E-20	1.6159E-20	3.3463E-5	1.0000
1.5100E-9	2.4000E-6	0.93000	-7.2015E-10	NaN	1.9307E-4	1.5100E-9	3.1352E-19	3.1950E-16	0.15426	8.6880E-20	1.6158E-20	3.3460E-5	1.0000
1.5100E-9	2.4000E-6	0.95000	-7.2008E-10	NaN	1.9305E-4	1.5100E-9	3.0134E-19	3.1947E-16	0.15425	8.6872E-20	1.6156E-20	3.3457E-5	1.0000
1.5100E-9	2.4000E-6	0.97000	-7.2002E-10	NaN	1.9303E-4	1.5100E-9	2.8993E-19	3.1944E-16	0.15424	8.6865E-20	1.6155E-20	3.3454E-5	1.0000
1.5100E-	2.4000E-	0.99	-7.19	NaN	1.9302E-	1.5100E-	2.7930E-	3.1942E-	0.15	8.6858E-	1.6154E-	3.3452E-	1.00

k0Sub (m/s)	d (m)	Time (s)	-(tds2.ntflux_Oxi)*n*F (A), UME	Surface ConcOxi (M), Oxi Mem	Surface ConcOxi (M), OxiNuc	Substrate Kinetics (m/s), k0Sub probe	Normal total flux (mol/s), UME OCR membrane	Normal total flux (mol/s), UME OCR nucleus	Concentration (mol/m^3), Point: (5.5109E-16, 2.5)	k0Sub*Oxi (mol/s), Mem Flux	k0Sub*Oxi (mol/s), Nuc Flux	Normal total flux (mol/(m^2*s)), Point: (5.5109E-16, 2.5)	WEIectrode radius (µm), UME Radius
9	6	000	97E-10		4	9	19	16	422	20	20	5	00
1.5100E-9	3.0000E-6	0.0000	-7.1631E-9	NaN	2.2700E-4	1.5100E-9	-2.0267E-20	-1.2904E-20	0.22700	1.0088E-19	2.0267E-20	-2.0069E-10	1.0000
1.5100E-9	3.0000E-6	0.0050000	-8.4775E-10	NaN	2.1955E-4	1.5100E-9	5.5541E-16	2.2941E-16	0.19384	9.8124E-20	1.9048E-20	2.4975E-5	1.0000
1.5100E-9	3.0000E-6	0.010000	-8.1284E-10	NaN	2.1404E-4	1.5100E-9	3.3480E-16	2.5464E-16	0.18559	9.5869E-20	1.8364E-20	2.3926E-5	1.0000
1.5100E-9	3.0000E-6	0.015000	-7.9497E-10	NaN	2.1050E-4	1.5100E-9	2.1781E-16	2.5860E-16	0.18144	9.4358E-20	1.7985E-20	2.3385E-5	1.0000
1.5100E-9	3.0000E-6	0.020000	-7.8304E-10	NaN	2.0803E-4	1.5100E-9	1.5170E-16	2.6044E-16	0.17867	9.3290E-20	1.7731E-20	2.3030E-5	1.0000
1.5100E-9	3.0000E-6	0.025000	-7.7463E-10	NaN	2.0621E-4	1.5100E-9	1.1025E-16	2.6104E-16	0.17673	9.2500E-20	1.7550E-20	2.2780E-5	1.0000
1.5100E-9	3.0000E-6	0.030000	-7.6826E-10	NaN	2.0480E-4	1.5100E-9	8.0872E-17	2.6130E-16	0.17526	9.1889E-20	1.7412E-20	2.2591E-5	1.0000
1.5100E-9	3.0000E-6	0.035000	-7.6345E-10	NaN	2.0372E-4	1.5100E-9	6.0786E-17	2.6127E-16	0.17415	9.1415E-20	1.7307E-20	2.2448E-5	1.0000
1.5100E-9	3.0000E-6	0.040000	-7.6001E-10	NaN	2.0292E-4	1.5100E-9	4.8506E-17	2.6101E-16	0.17336	9.1062E-20	1.7232E-20	2.2346E-5	1.0000

k0Sub (m/s)	d (m)	Time (s)	-(tds2.ntflux_Oxi)*n*F (A), UME	Surface Con cOxi (M), Oxi Mem	Surface Con cOxi (M), OxiNuc	Substrate Kinetics (m/s), k0Sub probe	Normal total flux (mol/s), UME OCR membrane	Normal total flux (mol/s), UME OCR nucleus	Concentration (mol/m^3), Point: (5.5109E-16, 2.5)	k0Sub* Oxi (mol/s), Mem Flux	k0Sub* Oxi (mol/s), Nuc Flux	Normal total flux (mol/(m^2*s)), Point: (5.5109E-16, 2.5)	WElectrode radius (µm), UME Radius
1.5100E-9	3.0000E-6	0.045000	-7.5721E-10	NaN	2.0226E-4	1.5100E-9	3.9236E-17	2.6074E-16	0.17271	9.0771E-20	1.7170E-20	2.2263E-5	1.0000
1.5100E-9	3.0000E-6	0.050000	-7.5511E-10	NaN	2.0175E-4	1.5100E-9	3.3309E-17	2.6043E-16	0.17223	9.0546E-20	1.7124E-20	2.2201E-5	1.0000
1.5100E-9	3.0000E-6	0.055000	-7.5310E-10	NaN	2.0127E-4	1.5100E-9	2.7335E-17	2.6018E-16	0.17177	9.0334E-20	1.7079E-20	2.2142E-5	1.0000
1.5100E-9	3.0000E-6	0.060000	-7.5139E-10	NaN	2.0085E-4	1.5100E-9	2.2513E-17	2.5995E-16	0.17137	9.0152E-20	1.7041E-20	2.2091E-5	1.0000
1.5100E-9	3.0000E-6	0.065000	-7.4998E-10	NaN	2.0051E-4	1.5100E-9	1.8844E-17	2.5974E-16	0.17105	9.0000E-20	1.7010E-20	2.2049E-5	1.0000
1.5100E-9	3.0000E-6	0.070000	-7.4896E-10	NaN	2.0026E-4	1.5100E-9	1.6604E-17	2.5955E-16	0.17082	8.9889E-20	1.6988E-20	2.2019E-5	1.0000
1.5100E-9	3.0000E-6	0.075000	-7.4828E-10	NaN	2.0009E-4	1.5100E-9	1.5560E-17	2.5938E-16	0.17066	8.9812E-20	1.6972E-20	2.1999E-5	1.0000
1.5100E-9	3.0000E-6	0.080000	-7.4760E-10	NaN	1.9992E-4	1.5100E-9	1.4516E-17	2.5922E-16	0.17051	8.9735E-20	1.6957E-20	2.1979E-5	1.0000
1.5100E-9	3.0000E-6	0.085000	-7.4692E-10	NaN	1.9974E-4	1.5100E-9	1.3472E-17	2.5906E-16	0.17035	8.9659E-20	1.6942E-20	2.1959E-5	1.0000
1.5100E-9	3.0000E-6	0.090000	-7.4638E-	NaN	1.9961E-4	1.5100E-9	1.2653E-17	2.5892E-16	0.17023	8.9597E-20	1.6930E-20	2.1943E-5	1.0000

k0Sub (m/s)	d (m)	Time (s)	-(tds2.ntflux_Oxi)*n*F (A), UME	Surface ConcOxi (M), OxiMem	Surface ConcOxi (M), OxiNuc	Substrate Kinetics (m/s), k0Sub probe	Normal total flux (mol/s), UME OCR membrane	Normal total flux (mol/s), UME OCR nucleus	Concentration (mol/m^3), Point: (5.5109E-16, 2.5)	k0Sub*Oxi (mol/s), Mem Flux	k0Sub*Oxi (mol/s), Nuc Flux	Normal total flux (mol/(m^2*s)), Point: (5.5109E-16, 2.5)	WElectrode radius (µm), UME Radius
			10										
1.5100E-9	3.0000E-6	0.095000	-7.4595E-10	NaN	1.9949E-4	1.5100E-9	1.2043E-17	2.5882E-16	0.17013	8.9548E-20	1.6920E-20	2.1930E-5	1.0000
1.5100E-9	3.0000E-6	0.10000	-7.4551E-10	NaN	1.9938E-4	1.5100E-9	1.1433E-17	2.5871E-16	0.17003	8.9499E-20	1.6910E-20	2.1917E-5	1.0000
1.5100E-9	3.0000E-6	0.10500	-7.4508E-10	NaN	1.9927E-4	1.5100E-9	1.0823E-17	2.5860E-16	0.16993	8.9449E-20	1.6901E-20	2.1905E-5	1.0000
1.5100E-9	3.0000E-6	0.11000	-7.4465E-10	NaN	1.9916E-4	1.5100E-9	1.0213E-17	2.5849E-16	0.16983	8.9400E-20	1.6891E-20	2.1892E-5	1.0000
1.5100E-9	3.0000E-6	0.11500	-7.4421E-10	NaN	1.9905E-4	1.5100E-9	9.6028E-18	2.5839E-16	0.16973	8.9351E-20	1.6881E-20	2.1879E-5	1.0000
1.5100E-9	3.0000E-6	0.12000	-7.4378E-10	NaN	1.9894E-4	1.5100E-9	8.9928E-18	2.5828E-16	0.16963	8.9301E-20	1.6871E-20	2.1866E-5	1.0000
1.5100E-9	3.0000E-6	0.12500	-7.4336E-10	NaN	1.9884E-4	1.5100E-9	8.4042E-18	2.5818E-16	0.16954	8.9254E-20	1.6862E-20	2.1854E-5	1.0000
1.5100E-9	3.0000E-6	0.13000	-7.4313E-10	NaN	1.9878E-4	1.5100E-9	8.1405E-18	2.5811E-16	0.16948	8.9228E-20	1.6857E-20	2.1847E-5	1.0000
1.5100E-9	3.0000E-6	0.13500	-7.4290E-10	NaN	1.9872E-4	1.5100E-9	7.8769E-18	2.5805E-16	0.16943	8.9201E-20	1.6852E-20	2.1840E-5	1.0000
1.5100E-9	3.0000E-	0.14	-7.42	NaN	1.9866E-	1.5100E-	7.6132E-	2.5799E-	0.16	8.9175E-	1.6846E-	2.1833E-	1.00

k0Sub (m/s)	d (m)	Time (s)	-(tds2.ntflux_Oxi)*n*F (A), UME	Surface ConcOxi (M), Oxi Mem	Surface ConcOxi (M), OxiNuc	Substrate Kinetics (m/s), k0Sub probe	Normal total flux (mol/s), UME OCR membrane	Normal total flux (mol/s), UME OCR nucleus	Concentration (mol/m^3), Point: (5.5109E-16, 2.5)	k0Sub*Oxi (mol/s), Mem Flux	k0Sub*Oxi (mol/s), Nuc Flux	Normal total flux (mol/(m^2*s)), Point: (5.5109E-16, 2.5)	WElectrode radius (µm), UME Radius
9	6	000	67E-10		4	9	18	16	938	20	20	5	00
1.5100E-9	3.0000E-6	0.14500	-7.4244E-10	NaN	1.9860E-4	1.5100E-9	7.3496E-18	2.5793E-16	0.16932	8.9148E-20	1.6841E-20	2.1827E-5	1.0000
1.5100E-9	3.0000E-6	0.15000	-7.4220E-10	NaN	1.9854E-4	1.5100E-9	7.0860E-18	2.5787E-16	0.16927	8.9121E-20	1.6836E-20	2.1820E-5	1.0000
1.5100E-9	3.0000E-6	0.15500	-7.4197E-10	NaN	1.9848E-4	1.5100E-9	6.8223E-18	2.5781E-16	0.16922	8.9095E-20	1.6831E-20	2.1813E-5	1.0000
1.5100E-9	3.0000E-6	0.16000	-7.4174E-10	NaN	1.9842E-4	1.5100E-9	6.5587E-18	2.5774E-16	0.16916	8.9068E-20	1.6826E-20	2.1806E-5	1.0000
1.5100E-9	3.0000E-6	0.16500	-7.4151E-10	NaN	1.9836E-4	1.5100E-9	6.2950E-18	2.5768E-16	0.16911	8.9041E-20	1.6820E-20	2.1799E-5	1.0000
1.5100E-9	3.0000E-6	0.17000	-7.4127E-10	NaN	1.9830E-4	1.5100E-9	6.0314E-18	2.5762E-16	0.16906	8.9015E-20	1.6815E-20	2.1792E-5	1.0000
1.5100E-9	3.0000E-6	0.17500	-7.4104E-10	NaN	1.9824E-4	1.5100E-9	5.7678E-18	2.5756E-16	0.16901	8.8988E-20	1.6810E-20	2.1785E-5	1.0000
1.5100E-9	3.0000E-6	0.18000	-7.4081E-10	NaN	1.9818E-4	1.5100E-9	5.5041E-18	2.5750E-16	0.16895	8.8961E-20	1.6805E-20	2.1779E-5	1.0000
1.5100E-9	3.0000E-6	0.18500	-7.4058E-10	NaN	1.9812E-4	1.5100E-9	5.2405E-18	2.5743E-16	0.16890	8.8935E-20	1.6800E-20	2.1772E-5	1.0000

k0Sub (m/s)	d (m)	Time (s)	-(tds2.ntflux_Oxi)*n*F (A), UME	Surface ConcOxi (M), OxiMem	Surface ConcOxi (M), OxiNuc	Substrate Kinetics (m/s), k0Sub probe	Normal total flux (mol/s), UME OCR membrane	Normal total flux (mol/s), UME OCR nucleus	Concentration (mol/m^3), Point: (5.5109E-16, 2.5)	k0Sub*Oxi (mol/s), Mem Flux	k0Sub*Oxi (mol/s), Nuc Flux	Normal total flux (mol/(m^2*s)), Point: (5.5109E-16, 2.5)	WElectrode radius (μm), UME Radius
1.5100E-9	3.0000E-6	0.19000	-7.4035E-10	NaN	1.9806E-4	1.5100E-9	4.9768E-18	2.5737E-16	0.16885	8.8908E-20	1.6794E-20	2.1765E-5	1.0000
1.5100E-9	3.0000E-6	0.19500	-7.4011E-10	NaN	1.9800E-4	1.5100E-9	4.7132E-18	2.5731E-16	0.16879	8.8881E-20	1.6789E-20	2.1758E-5	1.0000
1.5100E-9	3.0000E-6	0.20000	-7.3990E-10	NaN	1.9795E-4	1.5100E-9	4.4706E-18	2.5725E-16	0.16874	8.8857E-20	1.6784E-20	2.1752E-5	1.0000
1.5100E-9	3.0000E-6	0.20500	-7.3976E-10	NaN	1.9791E-4	1.5100E-9	4.3506E-18	2.5721E-16	0.16871	8.8841E-20	1.6781E-20	2.1748E-5	1.0000
1.5100E-9	3.0000E-6	0.21000	-7.3962E-10	NaN	1.9787E-4	1.5100E-9	4.2306E-18	2.5717E-16	0.16868	8.8825E-20	1.6778E-20	2.1744E-5	1.0000
1.5100E-9	3.0000E-6	0.21500	-7.3949E-10	NaN	1.9784E-4	1.5100E-9	4.1105E-18	2.5713E-16	0.16865	8.8809E-20	1.6775E-20	2.1740E-5	1.0000
1.5100E-9	3.0000E-6	0.22000	-7.3935E-10	NaN	1.9780E-4	1.5100E-9	3.9905E-18	2.5709E-16	0.16862	8.8793E-20	1.6772E-20	2.1736E-5	1.0000
1.5100E-9	3.0000E-6	0.22500	-7.3921E-10	NaN	1.9777E-4	1.5100E-9	3.8704E-18	2.5706E-16	0.16859	8.8777E-20	1.6769E-20	2.1731E-5	1.0000
1.5100E-9	3.0000E-6	0.23000	-7.3907E-10	NaN	1.9773E-4	1.5100E-9	3.7504E-18	2.5702E-16	0.16856	8.8761E-20	1.6766E-20	2.1727E-5	1.0000
1.5100E-9	3.0000E-6	0.23500	-7.3893E-	NaN	1.9769E-4	1.5100E-9	3.6304E-18	2.5698E-16	0.16852	8.8745E-20	1.6763E-20	2.1723E-5	1.0000

k0Sub (m/s)	d (m)	Time (s)	-(tds2.ntflux_Oxi)*n*F (A), UME	Surface ConcOxi (M), OxiMem	Surface ConcOxi (M), OxiNuc	Substrate Kinetics (m/s), k0Sub probe	Normal total flux (mol/s), UME OCR membrane	Normal total flux (mol/s), UME OCR nucleus	Concentration (mol/m^3), Point: (5.5109E-16, 2.5)	k0Sub*Oxi (mol/s), Mem Flux	k0Sub*Oxi (mol/s), Nuc Flux	Normal total flux (mol/(m^2*s)), Point: (5.5109E-16, 2.5)	WElectrode radius (µm), UME Radius
			10										
1.5100E-9	3.0000E-6	0.24000	-7.3880E-10	NaN	1.9766E-4	1.5100E-9	3.5103E-18	2.5694E-16	0.16849	8.8729E-20	1.6759E-20	2.1719E-5	1.0000
1.5100E-9	3.0000E-6	0.24500	-7.3866E-10	NaN	1.9762E-4	1.5100E-9	3.3903E-18	2.5690E-16	0.16846	8.8713E-20	1.6756E-20	2.1715E-5	1.0000
1.5100E-9	3.0000E-6	0.25000	-7.3852E-10	NaN	1.9759E-4	1.5100E-9	3.2703E-18	2.5686E-16	0.16843	8.8697E-20	1.6753E-20	2.1711E-5	1.0000
1.5100E-9	3.0000E-6	0.25000	-7.3852E-10	NaN	1.9759E-4	1.5100E-9	3.2703E-18	2.5686E-16	0.16843	8.8697E-20	1.6753E-20	2.1711E-5	1.0000
1.5100E-9	3.0000E-6	0.27000	-7.3797E-10	NaN	1.9744E-4	1.5100E-9	2.7901E-18	2.5670E-16	0.16830	8.8633E-20	1.6741E-20	2.1695E-5	1.0000
1.5100E-9	3.0000E-6	0.29000	-7.3760E-10	NaN	1.9735E-4	1.5100E-9	2.5243E-18	2.5659E-16	0.16822	8.8589E-20	1.6732E-20	2.1684E-5	1.0000
1.5100E-9	3.0000E-6	0.31000	-7.3726E-10	NaN	1.9726E-4	1.5100E-9	2.3100E-18	2.5649E-16	0.16814	8.8550E-20	1.6725E-20	2.1674E-5	1.0000
1.5100E-9	3.0000E-6	0.33000	-7.3693E-10	NaN	1.9717E-4	1.5100E-9	2.0957E-18	2.5639E-16	0.16807	8.8511E-20	1.6717E-20	2.1664E-5	1.0000
1.5100E-9	3.0000E-6	0.35000	-7.3660E-10	NaN	1.9709E-4	1.5100E-9	1.8814E-18	2.5629E-16	0.16799	8.8472E-20	1.6710E-20	2.1654E-5	1.0000
1.5100E-	3.0000E-	0.37	-7.36	NaN	1.9700E-	1.5100E-	1.6670E-	2.5619E-	0.16	8.8433E-	1.6702E-	2.1645E-	1.00

k0Sub (m/s)	d (m)	Time (s)	-(tds2.ntflux_Oxi)*n*F (A), UME	Surface ConcOxi (M), OxiMem	Surface ConcOxi (M), OxiNuc	Substrate Kinetics (m/s), k0Sub probe	Normal total flux (mol/s), UME OCR membrane	Normal total flux (mol/s), UME OCR nucleus	Concentration (mol/m^3), Point: (5.5109E-16, 2.5)	k0Sub*Oxi (mol/s), Mem Flux	k0Sub*Oxi (mol/s), Nuc Flux	Normal total flux (mol/(m^2*s)), Point: (5.5109E-16, 2.5)	WElectrode radius (µm), UME Radius
9	6	000	26E-10		4	9	18	16	791	20	20	5	00
1.5100E-9	3.0000E-6	0.39000	-7.3603E-10	NaN	1.9694E-4	1.5100E-9	1.5428E-18	2.5611E-16	0.16786	8.8405E-20	1.6697E-20	2.1638E-5	1.0000
1.5100E-9	3.0000E-6	0.41000	-7.3580E-10	NaN	1.9688E-4	1.5100E-9	1.4342E-18	2.5604E-16	0.16781	8.8379E-20	1.6692E-20	2.1631E-5	1.0000
1.5100E-9	3.0000E-6	0.43000	-7.3558E-10	NaN	1.9682E-4	1.5100E-9	1.3255E-18	2.5597E-16	0.16776	8.8353E-20	1.6687E-20	2.1625E-5	1.0000
1.5100E-9	3.0000E-6	0.45000	-7.3536E-10	NaN	1.9676E-4	1.5100E-9	1.2169E-18	2.5590E-16	0.16771	8.8327E-20	1.6682E-20	2.1618E-5	1.0000
1.5100E-9	3.0000E-6	0.47000	-7.3514E-10	NaN	1.9670E-4	1.5100E-9	1.1082E-18	2.5583E-16	0.16766	8.8300E-20	1.6677E-20	2.1612E-5	1.0000
1.5100E-9	3.0000E-6	0.49000	-7.3497E-10	NaN	1.9666E-4	1.5100E-9	1.0415E-18	2.5578E-16	0.16762	8.8281E-20	1.6673E-20	2.1607E-5	1.0000
1.5100E-9	3.0000E-6	0.51000	-7.3481E-10	NaN	1.9662E-4	1.5100E-9	9.7962E-19	2.5573E-16	0.16758	8.8262E-20	1.6669E-20	2.1602E-5	1.0000
1.5100E-9	3.0000E-6	0.53000	-7.3465E-10	NaN	1.9657E-4	1.5100E-9	9.1772E-19	2.5568E-16	0.16755	8.8243E-20	1.6666E-20	2.1597E-5	1.0000
1.5100E-9	3.0000E-6	0.55000	-7.3449E-10	NaN	1.9653E-4	1.5100E-9	8.5582E-19	2.5563E-16	0.16751	8.8224E-20	1.6662E-20	2.1593E-5	1.0000

k0Sub (m/s)	d (m)	Time (s)	-(tds2.ntflux_Oxi)*n*F (A), UME	Surface ConcOxi (M), OxiMem	Surface ConcOxi (M), OxiNuc	Substrate Kinetics (m/s), k0Sub probe	Normal total flux (mol/s), UME OCR membrane	Normal total flux (mol/s), UME OCR nucleus	Concentration (mol/m^3), Point: (5.5109E-16, 2.5)	k0Sub*Oxi (mol/s), Mem Flux	k0Sub*Oxi (mol/s), Nuc Flux	Normal total flux (mol/(m^2*s)), Point: (5.5109E-16, 2.5)	WEIectrode radius (µm), UME Radius
1.5100E-9	3.0000E-6	0.57000	-7.3433E-10	NaN	1.9649E-4	1.5100E-9	7.9392E-19	2.5557E-16	0.16747	8.8205E-20	1.6659E-20	2.1588E-5	1.0000
1.5100E-9	3.0000E-6	0.59000	-7.3420E-10	NaN	1.9646E-4	1.5100E-9	7.5202E-19	2.5553E-16	0.16744	8.8190E-20	1.6656E-20	2.1584E-5	1.0000
1.5100E-9	3.0000E-6	0.61000	-7.3408E-10	NaN	1.9642E-4	1.5100E-9	7.1134E-19	2.5549E-16	0.16741	8.8175E-20	1.6653E-20	2.1580E-5	1.0000
1.5100E-9	3.0000E-6	0.63000	-7.3395E-10	NaN	1.9639E-4	1.5100E-9	6.7067E-19	2.5545E-16	0.16739	8.8160E-20	1.6650E-20	2.1577E-5	1.0000
1.5100E-9	3.0000E-6	0.65000	-7.3383E-10	NaN	1.9636E-4	1.5100E-9	6.2999E-19	2.5541E-16	0.16736	8.8145E-20	1.6647E-20	2.1573E-5	1.0000
1.5100E-9	3.0000E-6	0.67000	-7.3370E-10	NaN	1.9632E-4	1.5100E-9	5.8914E-19	2.5537E-16	0.16733	8.8131E-20	1.6644E-20	2.1569E-5	1.0000
1.5100E-9	3.0000E-6	0.69000	-7.3361E-10	NaN	1.9630E-4	1.5100E-9	5.6291E-19	2.5534E-16	0.16731	8.8119E-20	1.6642E-20	2.1566E-5	1.0000
1.5100E-9	3.0000E-6	0.71000	-7.3351E-10	NaN	1.9627E-4	1.5100E-9	5.3668E-19	2.5531E-16	0.16728	8.8107E-20	1.6640E-20	2.1564E-5	1.0000
1.5100E-9	3.0000E-6	0.73000	-7.3341E-10	NaN	1.9624E-4	1.5100E-9	5.1046E-19	2.5527E-16	0.16726	8.8096E-20	1.6638E-20	2.1561E-5	1.0000
1.5100E-9	3.0000E-6	0.75000	-7.3331E-	NaN	1.9622E-4	1.5100E-9	4.8423E-19	2.5524E-16	0.16724	8.8084E-20	1.6636E-20	2.1558E-5	1.0000

k0Sub (m/s)	d (m)	Time (s)	-(tds2.ntflux_Oxi)*n*F (A), UME	Surface ConcOxi (M), OxiMem	Surface ConcOxi (M), OxiNuc	Substrate Kinetics (m/s), k0Sub probe	Normal total flux (mol/s), UME OCR membrane	Normal total flux (mol/s), UME OCR nucleus	Concentration (mol/m^3), Point: (5.5109E-16, 2.5)	k0Sub*Oxi (mol/s), Mem Flux	k0Sub*Oxi (mol/s), Nuc Flux	Normal total flux (mol/(m^2*s)), Point: (5.5109E-16, 2.5)	WElectrode radius (μm), UME Radius
			10										
1.5100E-9	3.0000E-6	0.77000	-7.3321E-10	NaN	1.9619E-4	1.5100E-9	4.5813E-19	2.5521E-16	0.16722	8.8072E-20	1.6633E-20	2.1555E-5	1.0000
1.5100E-9	3.0000E-6	0.79000	-7.3313E-10	NaN	1.9617E-4	1.5100E-9	4.3669E-19	2.5518E-16	0.16720	8.8062E-20	1.6631E-20	2.1552E-5	1.0000
1.5100E-9	3.0000E-6	0.81000	-7.3304E-10	NaN	1.9615E-4	1.5100E-9	4.1683E-19	2.5515E-16	0.16718	8.8052E-20	1.6629E-20	2.1550E-5	1.0000
1.5100E-9	3.0000E-6	0.83000	-7.3296E-10	NaN	1.9613E-4	1.5100E-9	3.9855E-19	2.5512E-16	0.16716	8.8042E-20	1.6628E-20	2.1548E-5	1.0000
1.5100E-9	3.0000E-6	0.85000	-7.3288E-10	NaN	1.9611E-4	1.5100E-9	3.8187E-19	2.5510E-16	0.16714	8.8033E-20	1.6626E-20	2.1545E-5	1.0000
1.5100E-9	3.0000E-6	0.87000	-7.3281E-10	NaN	1.9609E-4	1.5100E-9	3.6663E-19	2.5507E-16	0.16713	8.8024E-20	1.6624E-20	2.1543E-5	1.0000
1.5100E-9	3.0000E-6	0.89000	-7.3274E-10	NaN	1.9607E-4	1.5100E-9	3.5131E-19	2.5505E-16	0.16711	8.8015E-20	1.6623E-20	2.1541E-5	1.0000
1.5100E-9	3.0000E-6	0.91000	-7.3267E-10	NaN	1.9605E-4	1.5100E-9	3.3700E-19	2.5503E-16	0.16709	8.8007E-20	1.6621E-20	2.1539E-5	1.0000
1.5100E-9	3.0000E-6	0.93000	-7.3260E-10	NaN	1.9603E-4	1.5100E-9	3.2368E-19	2.5500E-16	0.16708	8.7999E-20	1.6619E-20	2.1537E-5	1.0000
1.5100E-	3.0000E-	0.95	-7.32	NaN	1.9601E-	1.5100E-	3.1136E-	2.5498E-	0.16	8.7991E-	1.6618E-	2.1535E-	1.00

k0Sub (m/s)	d (m)	Time (s)	-(tds2.ntflux_Oxi)*n*F (A), UME	Surface ConcOxi (M), OxiMem	Surface ConcOxi (M), OxiNuc	Substrate Kinetics (m/s), k0Sub probe	Normal total flux (mol/s), UME OCR membrane	Normal total flux (mol/s), UME OCR nucleus	Concentration (mol/m^3), Point: (5.5109E-16, 2.5)	k0Sub*Oxi (mol/s), Mem Flux	k0Sub*Oxi (mol/s), Nuc Flux	Normal total flux (mol/(m^2*s)), Point: (5.5109E-16, 2.5)	WElectrode radius (µm), UME Radius
9	6	000	53E-10		4	9	19	16	706	20	20	5	00
1.5100E-9	3.0000E-6	0.97000	-7.3247E-10	NaN	1.9600E-4	1.5100E-9	2.9993E-19	2.5496E-16	0.16705	8.7984E-20	1.6617E-20	2.1533E-5	1.0000
1.5100E-9	3.0000E-6	0.99000	-7.3241E-10	NaN	1.9598E-4	1.5100E-9	2.8863E-19	2.5494E-16	0.16703	8.7977E-20	1.6615E-20	2.1531E-5	1.0000
1.5100E-9	4.0000E-6	0.0000	-7.1535E-9	NaN	2.2700E-4	1.5100E-9	-2.0267E-20	-1.3063E-20	0.22700	1.0088E-19	2.0267E-20	-2.1094E-10	1.0000
1.5100E-9	4.0000E-6	0.0050000	-8.5359E-10	NaN	2.2214E-4	1.5100E-9	4.4662E-16	1.4253E-16	0.20750	9.9050E-20	1.9503E-20	1.3335E-5	1.0000
1.5100E-9	4.0000E-6	0.010000	-8.2275E-10	NaN	2.1746E-4	1.5100E-9	2.9631E-16	1.7066E-16	0.19988	9.7142E-20	1.8912E-20	1.2986E-5	1.0000
1.5100E-9	4.0000E-6	0.015000	-8.0848E-10	NaN	2.1454E-4	1.5100E-9	2.0676E-16	1.7516E-16	0.19631	9.5899E-20	1.8600E-20	1.2742E-5	1.0000
1.5100E-9	4.0000E-6	0.020000	-7.9936E-10	NaN	2.1258E-4	1.5100E-9	1.5798E-16	1.7707E-16	0.19404	9.5053E-20	1.8399E-20	1.2593E-5	1.0000
1.5100E-9	4.0000E-6	0.025000	-7.9220E-10	NaN	2.1102E-4	1.5100E-9	1.2311E-16	1.7840E-16	0.19227	9.4378E-20	1.8242E-20	1.2478E-5	1.0000
1.5100E-9	4.0000E-6	0.030000	-7.8691E-10	NaN	2.0984E-4	1.5100E-9	9.9782E-17	1.7917E-16	0.19096	9.3866E-20	1.8125E-20	1.2395E-5	1.0000

k0Sub (m/s)	d (m)	Time (s)	-(tds2.ntflux_Oxi)*n*F (A), UME	Surface ConcOxi (M), OxiMem	Surface ConcOxi (M), OxiNuc	Substrate Kinetics (m/s), k0Sub probe	Normal total flux (mol/s), UME OCR membrane	Normal total flux (mol/s), UME OCR nucleus	Concentration (mol/m^3), Point: (5.5109E-16, 2.5)	k0Sub*Oxi (mol/s), Mem Flux	k0Sub*Oxi (mol/s), Nuc Flux	Normal total flux (mol/(m^2*s)), Point: (5.5109E-16, 2.5)	WEIectrode radius (µm), UME Radius
1.5100E-9	4.0000E-6	0.035000	-7.8288E-10	NaN	2.0893E-4	1.5100E-9	8.3226E-17	1.7963E-16	0.18997	9.3468E-20	1.8035E-20	1.2331E-5	1.0000
1.5100E-9	4.0000E-6	0.040000	-7.7967E-10	NaN	2.0819E-4	1.5100E-9	7.0949E-17	1.7989E-16	0.18918	9.3147E-20	1.7964E-20	1.2280E-5	1.0000
1.5100E-9	4.0000E-6	0.045000	-7.7647E-10	NaN	2.0746E-4	1.5100E-9	5.8672E-17	1.8016E-16	0.18839	9.2825E-20	1.7893E-20	1.2229E-5	1.0000
1.5100E-9	4.0000E-6	0.050000	-7.7421E-10	NaN	2.0693E-4	1.5100E-9	5.0989E-17	1.8025E-16	0.18784	9.2593E-20	1.7842E-20	1.2193E-5	1.0000
1.5100E-9	4.0000E-6	0.055000	-7.7213E-10	NaN	2.0644E-4	1.5100E-9	4.4056E-17	1.8032E-16	0.18733	9.2378E-20	1.7795E-20	1.2160E-5	1.0000
1.5100E-9	4.0000E-6	0.060000	-7.7015E-10	NaN	2.0597E-4	1.5100E-9	3.7594E-17	1.8037E-16	0.18684	9.2172E-20	1.7751E-20	1.2129E-5	1.0000
1.5100E-9	4.0000E-6	0.065000	-7.6901E-10	NaN	2.0569E-4	1.5100E-9	3.4515E-17	1.8034E-16	0.18656	9.2051E-20	1.7725E-20	1.2110E-5	1.0000
1.5100E-9	4.0000E-6	0.070000	-7.6787E-10	NaN	2.0542E-4	1.5100E-9	3.1437E-17	1.8031E-16	0.18628	9.1929E-20	1.7700E-20	1.2092E-5	1.0000
1.5100E-9	4.0000E-6	0.075000	-7.6673E-10	NaN	2.0514E-4	1.5100E-9	2.8358E-17	1.8028E-16	0.18600	9.1807E-20	1.7674E-20	1.2074E-5	1.0000
1.5100E-9	4.0000E-6	0.080000	-7.6559E-	NaN	2.0486E-4	1.5100E-9	2.5280E-17	1.8025E-16	0.18572	9.1685E-20	1.7648E-20	1.2056E-5	1.0000

k0Sub (m/s)	d (m)	Time (s)	-(tds2.ntflux_Oxi)*n*F (A), UME	Surface ConcOxi (M), OxiMem	Surface ConcOxi (M), OxiNuc	Substrate Kinetics (m/s), k0Sub probe	Normal total flux (mol/s), UME OCR membrane	Normal total flux (mol/s), UME OCR nucleus	Concentration (mol/m^3), Point: (5.5109E-16, 2.5)	k0Sub*Oxi (mol/s), Mem Flux	k0Sub*Oxi (mol/s), Nuc Flux	Normal total flux (mol/(m^2*s)), Point: (5.5109E-16, 2.5)	WElectrode radius (μm), UME Radius
			10										
1.5100E-9	4.0000E-6	0.085000	-7.6445E-10	NaN	2.0459E-4	1.5100E-9	2.2201E-17	1.8021E-16	0.18544	9.1564E-20	1.7623E-20	1.2038E-5	1.0000
1.5100E-9	4.0000E-6	0.090000	-7.6359E-10	NaN	2.0438E-4	1.5100E-9	2.0151E-17	1.8017E-16	0.18524	9.1471E-20	1.7603E-20	1.2024E-5	1.0000
1.5100E-9	4.0000E-6	0.095000	-7.6291E-10	NaN	2.0421E-4	1.5100E-9	1.8668E-17	1.8012E-16	0.18507	9.1396E-20	1.7588E-20	1.2014E-5	1.0000
1.5100E-9	4.0000E-6	0.10000	-7.6222E-10	NaN	2.0404E-4	1.5100E-9	1.7184E-17	1.8007E-16	0.18490	9.1321E-20	1.7572E-20	1.2003E-5	1.0000
1.5100E-9	4.0000E-6	0.10500	-7.6154E-10	NaN	2.0387E-4	1.5100E-9	1.5701E-17	1.8002E-16	0.18473	9.1246E-20	1.7557E-20	1.1992E-5	1.0000
1.5100E-9	4.0000E-6	0.11000	-7.6085E-10	NaN	2.0370E-4	1.5100E-9	1.4218E-17	1.7997E-16	0.18457	9.1171E-20	1.7541E-20	1.1981E-5	1.0000
1.5100E-9	4.0000E-6	0.11500	-7.6022E-10	NaN	2.0354E-4	1.5100E-9	1.2891E-17	1.7992E-16	0.18441	9.1101E-20	1.7527E-20	1.1971E-5	1.0000
1.5100E-9	4.0000E-6	0.12000	-7.5986E-10	NaN	2.0345E-4	1.5100E-9	1.2325E-17	1.7988E-16	0.18432	9.1061E-20	1.7519E-20	1.1965E-5	1.0000
1.5100E-9	4.0000E-6	0.12500	-7.5951E-10	NaN	2.0336E-4	1.5100E-9	1.1759E-17	1.7983E-16	0.18424	9.1021E-20	1.7511E-20	1.1960E-5	1.0000
1.5100E-	4.0000E-	0.13	-7.59	NaN	2.0327E-	1.5100E-	1.1193E-	1.7979E-	0.18	9.0981E-	1.7502E-	1.1954E-	1.00

k0Sub (m/s)	d (m)	Time (s)	-(tds2.ntflux_Oxi)*n*F (A), UME	Surface ConcOxi (M), Oxi Mem	Surface ConcOxi (M), OxiNuc	Substrate Kinetics (m/s), k0Sub probe	Normal total flux (mol/s), UME OCR membrane	Normal total flux (mol/s), UME OCR nucleus	Concentration (mol/m^3), Point: (5.5109E-16, 2.5)	k0Sub*Oxi (mol/s), Mem Flux	k0Sub*Oxi (mol/s), Nuc Flux	Normal total flux (mol/(m^2*s)), Point: (5.5109E-16, 2.5)	WElectrode radius (μm), UME Radius
9	6	000	15E-10		4	9	17	16	415	20	20	5	00
1.5100E-9	4.0000E-6	0.13500	-7.5880E-10	NaN	2.0318E-4	1.5100E-9	1.0627E-17	1.7975E-16	0.18406	9.0941E-20	1.7494E-20	1.1949E-5	1.0000
1.5100E-9	4.0000E-6	0.14000	-7.5844E-10	NaN	2.0309E-4	1.5100E-9	1.0061E-17	1.7971E-16	0.18398	9.0901E-20	1.7486E-20	1.1943E-5	1.0000
1.5100E-9	4.0000E-6	0.14500	-7.5809E-10	NaN	2.0300E-4	1.5100E-9	9.4952E-18	1.7966E-16	0.18389	9.0861E-20	1.7478E-20	1.1937E-5	1.0000
1.5100E-9	4.0000E-6	0.15000	-7.5773E-10	NaN	2.0291E-4	1.5100E-9	8.9292E-18	1.7962E-16	0.18380	9.0821E-20	1.7470E-20	1.1932E-5	1.0000
1.5100E-9	4.0000E-6	0.15500	-7.5738E-10	NaN	2.0282E-4	1.5100E-9	8.3633E-18	1.7958E-16	0.18372	9.0781E-20	1.7462E-20	1.1926E-5	1.0000
1.5100E-9	4.0000E-6	0.16000	-7.5702E-10	NaN	2.0273E-4	1.5100E-9	7.7974E-18	1.7953E-16	0.18363	9.0741E-20	1.7454E-20	1.1920E-5	1.0000
1.5100E-9	4.0000E-6	0.16500	-7.5667E-10	NaN	2.0264E-4	1.5100E-9	7.2314E-18	1.7949E-16	0.18354	9.0702E-20	1.7446E-20	1.1915E-5	1.0000
1.5100E-9	4.0000E-6	0.17000	-7.5634E-10	NaN	2.0256E-4	1.5100E-9	6.7345E-18	1.7945E-16	0.18346	9.0664E-20	1.7438E-20	1.1910E-5	1.0000
1.5100E-9	4.0000E-6	0.17500	-7.5613E-10	NaN	2.0250E-4	1.5100E-9	6.4889E-18	1.7942E-16	0.18341	9.0640E-20	1.7434E-20	1.1906E-5	1.0000

k0Sub (m/s)	d (m)	Time (s)	-(tds2.ntflux_Oxi)*n*F (A), UME	Surface ConcOxi (M), Oxi Mem	Surface ConcOxi (M), OxiNuc	Substrate Kinetics (m/s), k0Sub probe	Normal total flux (mol/s), UME OCR membrane	Normal total flux (mol/s), UME OCR nucleus	Concentration (mol/m^3), Point: (5.5109E-16, 2.5)	k0Sub*Oxi (mol/s), Mem Flux	k0Sub*Oxi (mol/s), Nuc Flux	Normal total flux (mol/(m^2*s)), Point: (5.5109E-16, 2.5)	WEIectrode radius (μm), UME Radius
1.5100E-9	4.0000E-6	0.18000	-7.5592E-10	NaN	2.0245E-4	1.5100E-9	6.2434E-18	1.7939E-16	0.18336	9.0616E-20	1.7429E-20	1.1903E-5	1.0000
1.5100E-9	4.0000E-6	0.18500	-7.5571E-10	NaN	2.0239E-4	1.5100E-9	5.9978E-18	1.7935E-16	0.18331	9.0592E-20	1.7424E-20	1.1900E-5	1.0000
1.5100E-9	4.0000E-6	0.19000	-7.5550E-10	NaN	2.0234E-4	1.5100E-9	5.7522E-18	1.7932E-16	0.18326	9.0568E-20	1.7419E-20	1.1896E-5	1.0000
1.5100E-9	4.0000E-6	0.19500	-7.5529E-10	NaN	2.0229E-4	1.5100E-9	5.5066E-18	1.7929E-16	0.18321	9.0544E-20	1.7414E-20	1.1893E-5	1.0000
1.5100E-9	4.0000E-6	0.20000	-7.5508E-10	NaN	2.0223E-4	1.5100E-9	5.2610E-18	1.7926E-16	0.18316	9.0520E-20	1.7410E-20	1.1890E-5	1.0000
1.5100E-9	4.0000E-6	0.20500	-7.5487E-10	NaN	2.0218E-4	1.5100E-9	5.0154E-18	1.7922E-16	0.18311	9.0496E-20	1.7405E-20	1.1886E-5	1.0000
1.5100E-9	4.0000E-6	0.21000	-7.5466E-10	NaN	2.0212E-4	1.5100E-9	4.7698E-18	1.7919E-16	0.18306	9.0472E-20	1.7400E-20	1.1883E-5	1.0000
1.5100E-9	4.0000E-6	0.21500	-7.5445E-10	NaN	2.0207E-4	1.5100E-9	4.5243E-18	1.7916E-16	0.18300	9.0448E-20	1.7395E-20	1.1880E-5	1.0000
1.5100E-9	4.0000E-6	0.22000	-7.5424E-10	NaN	2.0202E-4	1.5100E-9	4.2787E-18	1.7913E-16	0.18295	9.0424E-20	1.7390E-20	1.1876E-5	1.0000
1.5100E-9	4.0000E-6	0.22500	-7.5405E-	NaN	2.0197E-4	1.5100E-9	4.0739E-18	1.7910E-16	0.18291	9.0402E-20	1.7386E-20	1.1873E-5	1.0000

k0Sub (m/s)	d (m)	Time (s)	-(tds2.ntflux_Oxi)*n*F (A), UME	Surface Con cOxi (M), Oxi Mem	Surface Con cOxi (M), OxiNuc	Substrate Kinetics (m/s), k0Sub probe	Normal total flux (mol/s), UME OCR membrane	Normal total flux (mol/s), UME OCR nucleus	Concentration (mol/m^3), Point: (5.5109E-16, 2.5)	k0Sub*Oxi (mol/s), Mem Flux	k0Sub*Oxi (mol/s), Nuc Flux	Normal total flux (mol/(m^2*s)), Point: (5.5109E-16, 2.5)	WElectrode radius (µm), UME Radius
			10										
1.5100E-9	4.0000E-6	0.23000	-7.5394E-10	NaN	2.0194E-4	1.5100E-9	3.9804E-18	1.7908E-16	0.18288	9.0389E-20	1.7383E-20	1.1872E-5	1.0000
1.5100E-9	4.0000E-6	0.23500	-7.5382E-10	NaN	2.0191E-4	1.5100E-9	3.8869E-18	1.7906E-16	0.18285	9.0376E-20	1.7381E-20	1.1870E-5	1.0000
1.5100E-9	4.0000E-6	0.24000	-7.5371E-10	NaN	2.0188E-4	1.5100E-9	3.7934E-18	1.7903E-16	0.18282	9.0362E-20	1.7378E-20	1.1868E-5	1.0000
1.5100E-9	4.0000E-6	0.24500	-7.5359E-10	NaN	2.0185E-4	1.5100E-9	3.6999E-18	1.7901E-16	0.18280	9.0349E-20	1.7376E-20	1.1866E-5	1.0000
1.5100E-9	4.0000E-6	0.25000	-7.5348E-10	NaN	2.0182E-4	1.5100E-9	3.6064E-18	1.7899E-16	0.18277	9.0335E-20	1.7373E-20	1.1864E-5	1.0000
1.5100E-9	4.0000E-6	0.25000	-7.5348E-10	NaN	2.0182E-4	1.5100E-9	3.6064E-18	1.7899E-16	0.18277	9.0335E-20	1.7373E-20	1.1864E-5	1.0000
1.5100E-9	4.0000E-6	0.27000	-7.5302E-10	NaN	2.0170E-4	1.5100E-9	3.2324E-18	1.7891E-16	0.18266	9.0282E-20	1.7362E-20	1.1857E-5	1.0000
1.5100E-9	4.0000E-6	0.29000	-7.5256E-10	NaN	2.0158E-4	1.5100E-9	2.8584E-18	1.7883E-16	0.18254	9.0228E-20	1.7352E-20	1.1850E-5	1.0000
1.5100E-9	4.0000E-6	0.31000	-7.5210E-10	NaN	2.0146E-4	1.5100E-9	2.4843E-18	1.7874E-16	0.18243	9.0175E-20	1.7341E-20	1.1843E-5	1.0000
1.5100E-	4.0000E-	0.33	-7.51	NaN	2.0135E-	1.5100E-	2.1871E-	1.7867E-	0.18	9.0129E-	1.7332E-	1.1836E-	1.00

k0Sub (m/s)	d (m)	Time (s)	-(tds2.ntflux_Oxi)*n*F (A), UME	Surface ConcOxi (M), Oxi Mem	Surface ConcOxi (M), OxiNuc	Substrate Kinetics (m/s), k0Sub probe	Normal total flux (mol/s), UME OCR membrane	Normal total flux (mol/s), UME OCR nucleus	Concentration (mol/m^3), Point: (5.5109E-16, 2.5)	k0Sub*Oxi (mol/s), Mem Flux	k0Sub*Oxi (mol/s), Nuc Flux	Normal total flux (mol/(m^2*s)), Point: (5.5109E-16, 2.5)	WElectrode radius (μm), UME Radius
9	6	000	70E-10		4	9	18	16	233	20	20	5	00
1.5100E-9	4.0000E-6	0.35000	-7.5141E-10	NaN	2.0128E-4	1.5100E-9	2.0192E-18	1.7861E-16	0.18227	9.0095E-20	1.7326E-20	1.1832E-5	1.0000
1.5100E-9	4.0000E-6	0.37000	-7.5112E-10	NaN	2.0120E-4	1.5100E-9	1.8513E-18	1.7856E-16	0.18220	9.0061E-20	1.7319E-20	1.1827E-5	1.0000
1.5100E-9	4.0000E-6	0.39000	-7.5084E-10	NaN	2.0113E-4	1.5100E-9	1.6834E-18	1.7850E-16	0.18213	9.0028E-20	1.7312E-20	1.1823E-5	1.0000
1.5100E-9	4.0000E-6	0.41000	-7.5055E-10	NaN	2.0105E-4	1.5100E-9	1.5155E-18	1.7844E-16	0.18206	8.9994E-20	1.7306E-20	1.1818E-5	1.0000
1.5100E-9	4.0000E-6	0.43000	-7.5030E-10	NaN	2.0099E-4	1.5100E-9	1.3817E-18	1.7839E-16	0.18199	8.9964E-20	1.7300E-20	1.1814E-5	1.0000
1.5100E-9	4.0000E-6	0.45000	-7.5010E-10	NaN	2.0093E-4	1.5100E-9	1.2933E-18	1.7835E-16	0.18195	8.9941E-20	1.7295E-20	1.1811E-5	1.0000
1.5100E-9	4.0000E-6	0.47000	-7.4990E-10	NaN	2.0088E-4	1.5100E-9	1.2049E-18	1.7831E-16	0.18190	8.9918E-20	1.7291E-20	1.1808E-5	1.0000
1.5100E-9	4.0000E-6	0.49000	-7.4971E-10	NaN	2.0083E-4	1.5100E-9	1.1165E-18	1.7827E-16	0.18185	8.9894E-20	1.7286E-20	1.1805E-5	1.0000
1.5100E-9	4.0000E-6	0.51000	-7.4951E-10	NaN	2.0078E-4	1.5100E-9	1.0281E-18	1.7823E-16	0.18180	8.9871E-20	1.7282E-20	1.1802E-5	1.0000

k0Sub (m/s)	d (m)	Time (s)	-(tds2.ntflux_Oxi)*n*F (A), UME	Surface ConcOxi (M), Oxi Mem	Surface ConcOxi (M), OxiNuc	Substrate Kinetics (m/s), k0Sub probe	Normal total flux (mol/s), UME OCR membrane	Normal total flux (mol/s), UME OCR nucleus	Concentration (mol/m^3), Point: (5.5109E-16, 2.5)	k0Sub*Oxi (mol/s), Mem Flux	k0Sub*Oxi (mol/s), Nuc Flux	Normal total flux (mol/(m^2*s)), Point: (5.5109E-16, 2.5)	WElectrode radius (μm), UME Radius
1.5100E-9	4.0000E-6	0.53000	-7.4933E-10	NaN	2.0073E-4	1.5100E-9	9.5731E-19	1.7819E-16	0.18176	8.9850E-20	1.7278E-20	1.1799E-5	1.0000
1.5100E-9	4.0000E-6	0.55000	-7.4919E-10	NaN	2.0069E-4	1.5100E-9	9.0512E-19	1.7816E-16	0.18172	8.9833E-20	1.7274E-20	1.1797E-5	1.0000
1.5100E-9	4.0000E-6	0.57000	-7.4904E-10	NaN	2.0065E-4	1.5100E-9	8.5293E-19	1.7813E-16	0.18169	8.9816E-20	1.7271E-20	1.1794E-5	1.0000
1.5100E-9	4.0000E-6	0.59000	-7.4889E-10	NaN	2.0061E-4	1.5100E-9	8.0074E-19	1.7810E-16	0.18165	8.9798E-20	1.7268E-20	1.1792E-5	1.0000
1.5100E-9	4.0000E-6	0.61000	-7.4875E-10	NaN	2.0058E-4	1.5100E-9	7.4855E-19	1.7807E-16	0.18162	8.9781E-20	1.7264E-20	1.1790E-5	1.0000
1.5100E-9	4.0000E-6	0.63000	-7.4862E-10	NaN	2.0054E-4	1.5100E-9	7.0563E-19	1.7804E-16	0.18159	8.9766E-20	1.7261E-20	1.1788E-5	1.0000
1.5100E-9	4.0000E-6	0.65000	-7.4850E-10	NaN	2.0051E-4	1.5100E-9	6.7047E-19	1.7801E-16	0.18156	8.9752E-20	1.7259E-20	1.1786E-5	1.0000
1.5100E-9	4.0000E-6	0.67000	-7.4838E-10	NaN	2.0048E-4	1.5100E-9	6.3530E-19	1.7799E-16	0.18153	8.9738E-20	1.7256E-20	1.1784E-5	1.0000
1.5100E-9	4.0000E-6	0.69000	-7.4827E-10	NaN	2.0045E-4	1.5100E-9	6.0013E-19	1.7796E-16	0.18150	8.9724E-20	1.7253E-20	1.1782E-5	1.0000
1.5100E-9	4.0000E-6	0.71000	-7.4815E-	NaN	2.0042E-4	1.5100E-9	5.6496E-19	1.7794E-16	0.18147	8.9710E-20	1.7251E-20	1.1780E-5	1.0000

k0Sub (m/s)	d (m)	Time (s)	-(tds2.ntflux_Oxi)*n*F (A), UME	Surface ConcOxi (M), OxiMem	Surface ConcOxi (M), OxiNuc	Substrate Kinetics (m/s), k0Sub probe	Normal total flux (mol/s), UME OCR membrane	Normal total flux (mol/s), UME OCR nucleus	Concentration (mol/m^3), Point: (5.5109E-16, 2.5)	k0Sub*Oxi (mol/s), Mem Flux	k0Sub*Oxi (mol/s), Nuc Flux	Normal total flux (mol/(m^2*s)), Point: (5.5109E-16, 2.5)	WElectrode radius (µm), UME Radius
			10										
1.5100E-9	4.0000E-6	0.73000	-7.4805E-10	NaN	2.0039E-4	1.5100E-9	5.3692E-19	1.7792E-16	0.18145	8.9698E-20	1.7248E-20	1.1779E-5	1.0000
1.5100E-9	4.0000E-6	0.75000	-7.4796E-10	NaN	2.0037E-4	1.5100E-9	5.1361E-19	1.7789E-16	0.18143	8.9687E-20	1.7246E-20	1.1777E-5	1.0000
1.5100E-9	4.0000E-6	0.77000	-7.4786E-10	NaN	2.0034E-4	1.5100E-9	4.9030E-19	1.7787E-16	0.18140	8.9676E-20	1.7244E-20	1.1776E-5	1.0000
1.5100E-9	4.0000E-6	0.79000	-7.4777E-10	NaN	2.0032E-4	1.5100E-9	4.6699E-19	1.7785E-16	0.18138	8.9665E-20	1.7242E-20	1.1774E-5	1.0000
1.5100E-9	4.0000E-6	0.81000	-7.4768E-10	NaN	2.0029E-4	1.5100E-9	4.4367E-19	1.7783E-16	0.18136	8.9654E-20	1.7240E-20	1.1773E-5	1.0000
1.5100E-9	4.0000E-6	0.83000	-7.4759E-10	NaN	2.0027E-4	1.5100E-9	4.2281E-19	1.7781E-16	0.18134	8.9644E-20	1.7238E-20	1.1771E-5	1.0000
1.5100E-9	4.0000E-6	0.85000	-7.4751E-10	NaN	2.0025E-4	1.5100E-9	4.0433E-19	1.7780E-16	0.18132	8.9634E-20	1.7236E-20	1.1770E-5	1.0000
1.5100E-9	4.0000E-6	0.87000	-7.4743E-10	NaN	2.0023E-4	1.5100E-9	3.8717E-19	1.7778E-16	0.18130	8.9625E-20	1.7234E-20	1.1769E-5	1.0000
1.5100E-9	4.0000E-6	0.89000	-7.4736E-10	NaN	2.0021E-4	1.5100E-9	3.7135E-19	1.7776E-16	0.18128	8.9616E-20	1.7232E-20	1.1768E-5	1.0000
1.5100E-	4.0000E-	0.91	-7.47	NaN	2.0019E-	1.5100E-	3.5686E-	1.7775E-	0.18	8.9607E-	1.7231E-	1.1767E-	1.00

k0Sub (m/s)	d (m)	Time (s)	-(tds2.ntflux_Oxi)*n*F (A), UME	Surface ConcOxi (M), Oxi Mem	Surface ConcOxi (M), OxiNuc	Substrate Kinetics (m/s), k0Sub probe	Normal total flux (mol/s), UME OCR membrane	Normal total flux (mol/s), UME OCR nucleus	Concentration (mol/m^3), Point: (5.5109E-16, 2.5)	k0Sub*Oxi (mol/s), Mem Flux	k0Sub*Oxi (mol/s), Nuc Flux	Normal total flux (mol/(m^2*s)), Point: (5.5109E-16, 2.5)	WElectrode radius (µm), UME Radius
9	6	000	28E-10		4	9	19	16	126	20	20	5	00
1.5100E-9	4.0000E-6	0.93000	-7.4721E-10	NaN	2.0017E-4	1.5100E-9	3.4282E-19	1.7773E-16	0.18125	8.9599E-20	1.7229E-20	1.1765E-5	1.0000
1.5100E-9	4.0000E-6	0.95000	-7.4714E-10	NaN	2.0015E-4	1.5100E-9	3.2931E-19	1.7771E-16	0.18123	8.9591E-20	1.7227E-20	1.1764E-5	1.0000
1.5100E-9	4.0000E-6	0.97000	-7.4708E-10	NaN	2.0013E-4	1.5100E-9	3.1667E-19	1.7770E-16	0.18121	8.9583E-20	1.7226E-20	1.1763E-5	1.0000
1.5100E-9	4.0000E-6	0.99000	-7.4701E-10	NaN	2.0011E-4	1.5100E-9	3.0491E-19	1.7768E-16	0.18120	8.9575E-20	1.7224E-20	1.1762E-5	1.0000
1.5100E-9	5.0000E-6	0.0000	-7.1537E-9	NaN	2.2700E-4	1.5100E-9	-2.0266E-20	-1.3133E-20	0.22700	1.0088E-19	2.0266E-20	-2.1786E-10	1.0000
1.5100E-9	5.0000E-6	0.0050000	-8.5517E-10	NaN	2.2391E-4	1.5100E-9	3.4644E-16	8.9121E-17	0.21536	9.9699E-20	1.9799E-20	7.6784E-6	1.0000
1.5100E-9	5.0000E-6	0.010000	-8.2729E-10	NaN	2.2001E-4	1.5100E-9	2.6324E-16	1.1719E-16	0.20867	9.8114E-20	1.9304E-20	7.7458E-6	1.0000
1.5100E-9	5.0000E-6	0.015000	-8.1473E-10	NaN	2.1741E-4	1.5100E-9	1.8645E-16	1.2231E-16	0.20536	9.7005E-20	1.9022E-20	7.6105E-6	1.0000
1.5100E-9	5.0000E-6	0.020000	-8.0662E-10	NaN	2.1561E-4	1.5100E-9	1.4324E-16	1.2449E-16	0.20324	9.6234E-20	1.8837E-20	7.5258E-6	1.0000

k0Sub (m/s)	d (m)	Time (s)	-(tds2.ntflux_Oxi)*n*F (A), UME	Surface ConcOxi (M), OxiMem	Surface ConcOxi (M), OxiNuc	Substrate Kinetics (m/s), k0Sub probe	Normal total flux (mol/s), UME OCR membrane	Normal total flux (mol/s), UME OCR nucleus	Concentration (mol/m^3), Point: (5.5109E-16, 2.5)	k0Sub*Oxi (mol/s), Mem Flux	k0Sub*Oxi (mol/s), Nuc Flux	Normal total flux (mol/(m^2*s)), Point: (5.5109E-16, 2.5)	WElectrode radius (μm), UME Radius
1.5100E-9	5.0000E-6	0.025000	-8.0025E-10	NaN	2.1419E-4	1.5100E-9	1.1299E-16	1.2601E-16	0.20159	9.5619E-20	1.8694E-20	7.4642E-6	1.0000
1.5100E-9	5.0000E-6	0.030000	-7.9542E-10	NaN	2.1309E-4	1.5100E-9	9.2307E-17	1.2697E-16	0.20034	9.5142E-20	1.8584E-20	7.4185E-6	1.0000
1.5100E-9	5.0000E-6	0.035000	-7.9167E-10	NaN	2.1223E-4	1.5100E-9	7.7380E-17	1.2759E-16	0.19938	9.4765E-20	1.8499E-20	7.3830E-6	1.0000
1.5100E-9	5.0000E-6	0.040000	-7.8871E-10	NaN	2.1154E-4	1.5100E-9	6.6554E-17	1.2798E-16	0.19862	9.4463E-20	1.8432E-20	7.3551E-6	1.0000
1.5100E-9	5.0000E-6	0.045000	-7.8576E-10	NaN	2.1084E-4	1.5100E-9	5.5727E-17	1.2837E-16	0.19786	9.4160E-20	1.8365E-20	7.3273E-6	1.0000
1.5100E-9	5.0000E-6	0.050000	-7.8358E-10	NaN	2.1032E-4	1.5100E-9	4.8549E-17	1.2858E-16	0.19730	9.3933E-20	1.8315E-20	7.3066E-6	1.0000
1.5100E-9	5.0000E-6	0.055000	-7.8162E-10	NaN	2.0985E-4	1.5100E-9	4.2326E-17	1.2874E-16	0.19680	9.3727E-20	1.8270E-20	7.2881E-6	1.0000
1.5100E-9	5.0000E-6	0.060000	-7.7968E-10	NaN	2.0939E-4	1.5100E-9	3.6224E-17	1.2890E-16	0.19630	9.3522E-20	1.8226E-20	7.2697E-6	1.0000
1.5100E-9	5.0000E-6	0.065000	-7.7858E-10	NaN	2.0912E-4	1.5100E-9	3.3388E-17	1.2893E-16	0.19602	9.3403E-20	1.8200E-20	7.2593E-6	1.0000
1.5100E-9	5.0000E-6	0.070000	-7.7748E-	NaN	2.0885E-4	1.5100E-9	3.0552E-17	1.2896E-16	0.19574	9.3284E-20	1.8175E-20	7.2488E-6	1.0000

k0Sub (m/s)	d (m)	Time (s)	-(tds2.ntflux_Oxi)*n*F (A), UME	Surface ConcOxi (M), OxiMem	Surface ConcOxi (M), OxiNuc	Substrate Kinetics (m/s), k0Sub probe	Normal total flux (mol/s), UME OCR membrane	Normal total flux (mol/s), UME OCR nucleus	Concentration (mol/m^3), Point: (5.5109E-16, 2.5)	k0Sub*Oxi (mol/s), Mem Flux	k0Sub*Oxi (mol/s), Nuc Flux	Normal total flux (mol/(m^2*s)), Point: (5.5109E-16, 2.5)	WElectrode radius (µm), UME Radius
			10										
1.5100E-9	5.0000E-6	0.075000	-7.7638E-10	NaN	2.0857E-4	1.5100E-9	2.7716E-17	1.2899E-16	0.19546	9.3164E-20	1.8150E-20	7.2384E-6	1.0000
1.5100E-9	5.0000E-6	0.080000	-7.7528E-10	NaN	2.0830E-4	1.5100E-9	2.4881E-17	1.2902E-16	0.19518	9.3045E-20	1.8124E-20	7.2280E-6	1.0000
1.5100E-9	5.0000E-6	0.085000	-7.7418E-10	NaN	2.0803E-4	1.5100E-9	2.2045E-17	1.2905E-16	0.19490	9.2926E-20	1.8099E-20	7.2175E-6	1.0000
1.5100E-9	5.0000E-6	0.090000	-7.7329E-10	NaN	2.0781E-4	1.5100E-9	1.9954E-17	1.2906E-16	0.19467	9.2829E-20	1.8079E-20	7.2091E-6	1.0000
1.5100E-9	5.0000E-6	0.095000	-7.7262E-10	NaN	2.0764E-4	1.5100E-9	1.8552E-17	1.2905E-16	0.19450	9.2754E-20	1.8063E-20	7.2027E-6	1.0000
1.5100E-9	5.0000E-6	0.10000	-7.7195E-10	NaN	2.0747E-4	1.5100E-9	1.7150E-17	1.2904E-16	0.19433	9.2679E-20	1.8047E-20	7.1963E-6	1.0000
1.5100E-9	5.0000E-6	0.10500	-7.7127E-10	NaN	2.0730E-4	1.5100E-9	1.5748E-17	1.2903E-16	0.19416	9.2605E-20	1.8032E-20	7.1899E-6	1.0000
1.5100E-9	5.0000E-6	0.11000	-7.7060E-10	NaN	2.0714E-4	1.5100E-9	1.4346E-17	1.2902E-16	0.19399	9.2530E-20	1.8016E-20	7.1836E-6	1.0000
1.5100E-9	5.0000E-6	0.11500	-7.6992E-10	NaN	2.0697E-4	1.5100E-9	1.2944E-17	1.2900E-16	0.19382	9.2455E-20	1.8001E-20	7.1772E-6	1.0000
1.5100E-	5.0000E-	0.12	-7.69	NaN	2.0687E-	1.5100E-	1.2398E-	1.2898E-	0.19	9.2414E-	1.7992E-	7.1737E-	1.00

k0Sub (m/s)	d (m)	Time (s)	-(tds2.ntflux_Oxi)*n*F (A), UME	Surface ConcOxi (M), Oxi Mem	Surface ConcOxi (M), OxiNuc	Substrate Kinetics (m/s), k0Sub probe	Normal total flux (mol/s), UME OCR membrane	Normal total flux (mol/s), UME OCR nucleus	Concentration (mol/m^3), Point: (5.5109E-16, 2.5)	k0Sub*Oxi (mol/s), Mem Flux	k0Sub*Oxi (mol/s), Nuc Flux	Normal total flux (mol/(m^2*s)), Point: (5.5109E-16, 2.5)	WElectrode radius (μm), UME Radius
9	6	000	56E-10		4	9	17	16	372	20	20	6	00
1.5100E-9	5.0000E-6	0.12500	-7.6920E-10	NaN	2.0678E-4	1.5100E-9	1.1848E-17	1.2896E-16	0.19363	9.2374E-20	1.7984E-20	7.1704E-6	1.0000
1.5100E-9	5.0000E-6	0.13000	-7.6885E-10	NaN	2.0669E-4	1.5100E-9	1.1298E-17	1.2894E-16	0.19354	9.2333E-20	1.7976E-20	7.1670E-6	1.0000
1.5100E-9	5.0000E-6	0.13500	-7.6849E-10	NaN	2.0660E-4	1.5100E-9	1.0748E-17	1.2892E-16	0.19345	9.2293E-20	1.7967E-20	7.1636E-6	1.0000
1.5100E-9	5.0000E-6	0.14000	-7.6814E-10	NaN	2.0651E-4	1.5100E-9	1.0198E-17	1.2890E-16	0.19336	9.2252E-20	1.7959E-20	7.1603E-6	1.0000
1.5100E-9	5.0000E-6	0.14500	-7.6778E-10	NaN	2.0642E-4	1.5100E-9	9.6474E-18	1.2888E-16	0.19327	9.2212E-20	1.7951E-20	7.1569E-6	1.0000
1.5100E-9	5.0000E-6	0.15000	-7.6742E-10	NaN	2.0633E-4	1.5100E-9	9.0972E-18	1.2886E-16	0.19318	9.2172E-20	1.7943E-20	7.1535E-6	1.0000
1.5100E-9	5.0000E-6	0.15500	-7.6707E-10	NaN	2.0624E-4	1.5100E-9	8.5470E-18	1.2884E-16	0.19309	9.2131E-20	1.7934E-20	7.1502E-6	1.0000
1.5100E-9	5.0000E-6	0.16000	-7.6671E-10	NaN	2.0614E-4	1.5100E-9	7.9968E-18	1.2882E-16	0.19300	9.2091E-20	1.7926E-20	7.1468E-6	1.0000
1.5100E-9	5.0000E-6	0.16500	-7.6635E-10	NaN	2.0605E-4	1.5100E-9	7.4466E-18	1.2880E-16	0.19291	9.2050E-20	1.7918E-20	7.1434E-6	1.0000

k0Sub (m/s)	d (m)	Time (s)	-(tds2.ntflux_Oxi)*n*F (A), UME	Surface ConcOxi (M), OxiMem	Surface ConcOxi (M), OxiNuc	Substrate Kinetics (m/s), k0Sub probe	Normal total flux (mol/s), UME OCR membrane	Normal total flux (mol/s), UME OCR nucleus	Concentration (mol/m^3), Point: (5.5109E-16, 2.5)	k0Sub*Oxi (mol/s), Mem Flux	k0Sub*Oxi (mol/s), Nuc Flux	Normal total flux (mol/(m^2*s)), Point: (5.5109E-16, 2.5)	WEIectrode radius (µm), UME Radius
1.5100E-9	5.0000E-6	0.17000	-7.6600E-10	NaN	2.0596E-4	1.5100E-9	6.8964E-18	1.2877E-16	0.19282	9.2010E-20	1.7910E-20	7.1401E-6	1.0000
1.5100E-9	5.0000E-6	0.17500	-7.6578E-10	NaN	2.0590E-4	1.5100E-9	6.6439E-18	1.2876E-16	0.19276	9.1984E-20	1.7905E-20	7.1380E-6	1.0000
1.5100E-9	5.0000E-6	0.18000	-7.6556E-10	NaN	2.0585E-4	1.5100E-9	6.3997E-18	1.2874E-16	0.19271	9.1960E-20	1.7900E-20	7.1360E-6	1.0000
1.5100E-9	5.0000E-6	0.18500	-7.6535E-10	NaN	2.0579E-4	1.5100E-9	6.1556E-18	1.2872E-16	0.19266	9.1935E-20	1.7895E-20	7.1340E-6	1.0000
1.5100E-9	5.0000E-6	0.19000	-7.6514E-10	NaN	2.0574E-4	1.5100E-9	5.9114E-18	1.2870E-16	0.19260	9.1911E-20	1.7890E-20	7.1320E-6	1.0000
1.5100E-9	5.0000E-6	0.19500	-7.6493E-10	NaN	2.0568E-4	1.5100E-9	5.6672E-18	1.2868E-16	0.19255	9.1886E-20	1.7885E-20	7.1300E-6	1.0000
1.5100E-9	5.0000E-6	0.20000	-7.6471E-10	NaN	2.0563E-4	1.5100E-9	5.4230E-18	1.2866E-16	0.19249	9.1862E-20	1.7880E-20	7.1280E-6	1.0000
1.5100E-9	5.0000E-6	0.20500	-7.6450E-10	NaN	2.0557E-4	1.5100E-9	5.1789E-18	1.2864E-16	0.19244	9.1837E-20	1.7875E-20	7.1259E-6	1.0000
1.5100E-9	5.0000E-6	0.21000	-7.6429E-10	NaN	2.0552E-4	1.5100E-9	4.9347E-18	1.2862E-16	0.19239	9.1813E-20	1.7870E-20	7.1239E-6	1.0000
1.5100E-9	5.0000E-6	0.21500	-7.6407E-	NaN	2.0546E-4	1.5100E-9	4.6905E-18	1.2860E-16	0.19233	9.1788E-20	1.7865E-20	7.1219E-6	1.0000

k0Sub (m/s)	d (m)	Time (s)	-(tds2.ntflux_Oxi)*n*F (A), UME	Surface ConcOxi (M), Oxi Mem	Surface ConcOxi (M), OxiNuc	Substrate Kinetics (m/s), k0Sub probe	Normal total flux (mol/s), UME OCR membrane	Normal total flux (mol/s), UME OCR nucleus	Concentration (mol/m^3), Point: (5.5109E-16, 2.5)	k0Sub*Oxi (mol/s), Mem Flux	k0Sub*Oxi (mol/s), Nuc Flux	Normal total flux (mol/(m^2*s)), Point: (5.5109E-16, 2.5)	WElectrode radius (µm), UME Radius
			10										
1.5100E-9	5.0000E-6	0.22000	-7.6386E-10	NaN	2.0541E-4	1.5100E-9	4.4463E-18	1.2858E-16	0.19228	9.1764E-20	1.7860E-20	7.1199E-6	1.0000
1.5100E-9	5.0000E-6	0.22500	-7.6365E-10	NaN	2.0535E-4	1.5100E-9	4.2022E-18	1.2857E-16	0.19222	9.1739E-20	1.7855E-20	7.1179E-6	1.0000
1.5100E-9	5.0000E-6	0.23000	-7.6352E-10	NaN	2.0532E-4	1.5100E-9	4.0994E-18	1.2855E-16	0.19219	9.1724E-20	1.7852E-20	7.1167E-6	1.0000
1.5100E-9	5.0000E-6	0.23500	-7.6340E-10	NaN	2.0529E-4	1.5100E-9	4.0046E-18	1.2854E-16	0.19216	9.1710E-20	1.7849E-20	7.1156E-6	1.0000
1.5100E-9	5.0000E-6	0.24000	-7.6329E-10	NaN	2.0526E-4	1.5100E-9	3.9099E-18	1.2853E-16	0.19213	9.1697E-20	1.7847E-20	7.1145E-6	1.0000
1.5100E-9	5.0000E-6	0.24500	-7.6317E-10	NaN	2.0523E-4	1.5100E-9	3.8151E-18	1.2851E-16	0.19210	9.1683E-20	1.7844E-20	7.1134E-6	1.0000
1.5100E-9	5.0000E-6	0.25000	-7.6305E-10	NaN	2.0519E-4	1.5100E-9	3.7203E-18	1.2850E-16	0.19207	9.1669E-20	1.7841E-20	7.1123E-6	1.0000
1.5100E-9	5.0000E-6	0.25000	-7.6305E-10	NaN	2.0519E-4	1.5100E-9	3.7203E-18	1.2850E-16	0.19207	9.1669E-20	1.7841E-20	7.1123E-6	1.0000
1.5100E-9	5.0000E-6	0.27000	-7.6258E-10	NaN	2.0507E-4	1.5100E-9	3.3413E-18	1.2844E-16	0.19195	9.1614E-20	1.7830E-20	7.1079E-6	1.0000
1.5100E-9	5.0000E-	0.29	-7.62	NaN	2.0495E-	1.5100E-	2.9622E-	1.2839E-	0.19	9.1559E-	1.7819E-	7.1035E-	1.00

k0Sub (m/s)	d (m)	Time (s)	-(tds2.ntflux_Oxi)*n*F (A), UME	Surface ConcOxi (M), Oxi Mem	Surface ConcOxi (M), OxiNuc	Substrate Kinetics (m/s), k0Sub probe	Normal total flux (mol/s), UME OCR membrane	Normal total flux (mol/s), UME OCR nucleus	Concentration (mol/m^3), Point: (5.5109E-16, 2.5)	k0Sub*Oxi (mol/s), Mem Flux	k0Sub*Oxi (mol/s), Nuc Flux	Normal total flux (mol/(m^2*s)), Point: (5.5109E-16, 2.5)	WElectrode radius (µm), UME Radius
9	6	000	11E-10		4	9	18	16	184	20	20	6	00
1.5100E-9	5.0000E-6	0.31000	-7.6164E-10	NaN	2.0482E-4	1.5100E-9	2.5832E-18	1.2834E-16	0.19172	9.1505E-20	1.7808E-20	7.0990E-6	1.0000
1.5100E-9	5.0000E-6	0.33000	-7.6122E-10	NaN	2.0471E-4	1.5100E-9	2.2643E-18	1.2829E-16	0.19161	9.1455E-20	1.7798E-20	7.0951E-6	1.0000
1.5100E-9	5.0000E-6	0.35000	-7.6092E-10	NaN	2.0464E-4	1.5100E-9	2.0922E-18	1.2825E-16	0.19154	9.1420E-20	1.7792E-20	7.0923E-6	1.0000
1.5100E-9	5.0000E-6	0.37000	-7.6063E-10	NaN	2.0456E-4	1.5100E-9	1.9201E-18	1.2821E-16	0.19146	9.1386E-20	1.7785E-20	7.0896E-6	1.0000
1.5100E-9	5.0000E-6	0.39000	-7.6033E-10	NaN	2.0448E-4	1.5100E-9	1.7481E-18	1.2817E-16	0.19139	9.1351E-20	1.7778E-20	7.0868E-6	1.0000
1.5100E-9	5.0000E-6	0.41000	-7.6004E-10	NaN	2.0440E-4	1.5100E-9	1.5760E-18	1.2814E-16	0.19131	9.1316E-20	1.7771E-20	7.0840E-6	1.0000
1.5100E-9	5.0000E-6	0.43000	-7.5977E-10	NaN	2.0433E-4	1.5100E-9	1.4320E-18	1.2810E-16	0.19125	9.1285E-20	1.7765E-20	7.0815E-6	1.0000
1.5100E-9	5.0000E-6	0.45000	-7.5957E-10	NaN	2.0428E-4	1.5100E-9	1.3410E-18	1.2807E-16	0.19119	9.1261E-20	1.7760E-20	7.0796E-6	1.0000
1.5100E-9	5.0000E-6	0.47000	-7.5937E-10	NaN	2.0422E-4	1.5100E-9	1.2500E-18	1.2804E-16	0.19114	9.1237E-20	1.7755E-20	7.0777E-6	1.0000

k0Sub (m/s)	d (m)	Time (s)	-(tds2.ntflux_Oxi)*n*F (A), UME	Surface ConcOxi (M), OxiMem	Surface ConcOxi (M), OxiNuc	Substrate Kinetics (m/s), k0Sub probe	Normal total flux (mol/s), UME OCR membrane	Normal total flux (mol/s), UME OCR nucleus	Concentration (mol/m^3), Point: (5.5109E-16, 2.5)	k0Sub*Oxi (mol/s), Mem Flux	k0Sub*Oxi (mol/s), Nuc Flux	Normal total flux (mol/(m^2*s)), Point: (5.5109E-16, 2.5)	WElectrode radius (µm), UME Radius
1.5100E-9	5.0000E-6	0.49000	-7.5916E-10	NaN	2.0417E-4	1.5100E-9	1.1590E-18	1.2802E-16	0.19109	9.1213E-20	1.7751E-20	7.0758E-6	1.0000
1.5100E-9	5.0000E-6	0.51000	-7.5896E-10	NaN	2.0412E-4	1.5100E-9	1.0680E-18	1.2799E-16	0.19104	9.1189E-20	1.7746E-20	7.0739E-6	1.0000
1.5100E-9	5.0000E-6	0.53000	-7.5878E-10	NaN	2.0407E-4	1.5100E-9	9.9196E-19	1.2796E-16	0.19099	9.1167E-20	1.7742E-20	7.0722E-6	1.0000
1.5100E-9	5.0000E-6	0.55000	-7.5863E-10	NaN	2.0403E-4	1.5100E-9	9.3815E-19	1.2794E-16	0.19096	9.1149E-20	1.7738E-20	7.0708E-6	1.0000
1.5100E-9	5.0000E-6	0.57000	-7.5848E-10	NaN	2.0399E-4	1.5100E-9	8.8435E-19	1.2792E-16	0.19092	9.1132E-20	1.7735E-20	7.0694E-6	1.0000
1.5100E-9	5.0000E-6	0.59000	-7.5833E-10	NaN	2.0395E-4	1.5100E-9	8.3054E-19	1.2790E-16	0.19088	9.1114E-20	1.7731E-20	7.0680E-6	1.0000
1.5100E-9	5.0000E-6	0.61000	-7.5818E-10	NaN	2.0391E-4	1.5100E-9	7.7673E-19	1.2787E-16	0.19084	9.1096E-20	1.7728E-20	7.0666E-6	1.0000
1.5100E-9	5.0000E-6	0.63000	-7.5804E-10	NaN	2.0387E-4	1.5100E-9	7.3095E-19	1.2785E-16	0.19081	9.1080E-20	1.7724E-20	7.0653E-6	1.0000
1.5100E-9	5.0000E-6	0.65000	-7.5792E-10	NaN	2.0384E-4	1.5100E-9	6.9467E-19	1.2784E-16	0.19078	9.1065E-20	1.7722E-20	7.0642E-6	1.0000
1.5100E-9	5.0000E-6	0.67000	-7.5780E-	NaN	2.0381E-4	1.5100E-9	6.5840E-19	1.2782E-16	0.19075	9.1051E-20	1.7719E-20	7.0631E-6	1.0000

k0Sub (m/s)	d (m)	Time (s)	-(tds2.ntflux_Oxi)*n*F (A), UME	Surface ConcOxi (M), OxiMem	Surface ConcOxi (M), OxiNuc	Substrate Kinetics (m/s), k0Sub probe	Normal total flux (mol/s), UME OCR membrane	Normal total flux (mol/s), UME OCR nucleus	Concentration (mol/m^3), Point: (5.5109E-16, 2.5)	k0Sub*Oxi (mol/s), Mem Flux	k0Sub*Oxi (mol/s), Nuc Flux	Normal total flux (mol/(m^2*s)), Point: (5.5109E-16, 2.5)	WElectrode radius (µm), UME Radius
			10										
1.5100E-9	5.0000E-6	0.69000	-7.5768E-10	NaN	2.0378E-4	1.5100E-9	6.2212E-19	1.2780E-16	0.19072	9.1037E-20	1.7716E-20	7.0619E-6	1.0000
1.5100E-9	5.0000E-6	0.71000	-7.5756E-10	NaN	2.0374E-4	1.5100E-9	5.8585E-19	1.2778E-16	0.19069	9.1023E-20	1.7713E-20	7.0608E-6	1.0000
1.5100E-9	5.0000E-6	0.73000	-7.5745E-10	NaN	2.0371E-4	1.5100E-9	5.5584E-19	1.2777E-16	0.19066	9.1010E-20	1.7711E-20	7.0598E-6	1.0000
1.5100E-9	5.0000E-6	0.75000	-7.5736E-10	NaN	2.0369E-4	1.5100E-9	5.3179E-19	1.2775E-16	0.19064	9.0999E-20	1.7708E-20	7.0589E-6	1.0000
1.5100E-9	5.0000E-6	0.77000	-7.5726E-10	NaN	2.0366E-4	1.5100E-9	5.0774E-19	1.2774E-16	0.19061	9.0987E-20	1.7706E-20	7.0580E-6	1.0000
1.5100E-9	5.0000E-6	0.79000	-7.5717E-10	NaN	2.0364E-4	1.5100E-9	4.8369E-19	1.2772E-16	0.19059	9.0976E-20	1.7704E-20	7.0572E-6	1.0000
1.5100E-9	5.0000E-6	0.81000	-7.5707E-10	NaN	2.0361E-4	1.5100E-9	4.5964E-19	1.2771E-16	0.19056	9.0965E-20	1.7702E-20	7.0563E-6	1.0000
1.5100E-9	5.0000E-6	0.83000	-7.5698E-10	NaN	2.0359E-4	1.5100E-9	4.3773E-19	1.2770E-16	0.19054	9.0954E-20	1.7700E-20	7.0554E-6	1.0000
1.5100E-9	5.0000E-6	0.85000	-7.5690E-10	NaN	2.0357E-4	1.5100E-9	4.1854E-19	1.2768E-16	0.19052	9.0944E-20	1.7698E-20	7.0546E-6	1.0000
1.5100E-	5.0000E-	0.87	-7.56	NaN	2.0355E-	1.5100E-	4.0072E-	1.2767E-	0.19	9.0934E-	1.7696E-	7.0539E-	1.00

k0Sub (m/s)	d (m)	Time (s)	-(tds2.ntflux_Oxi)*n*F (A), UME	Surface ConcOxi (M), OxiMem	Surface ConcOxi (M), OxiNuc	Substrate Kinetics (m/s), k0Sub probe	Normal total flux (mol/s), UME OCR membrane	Normal total flux (mol/s), UME OCR nucleus	Concentration (mol/m^3), Point: (5.5109E-16, 2.5)	k0Sub*Oxi (mol/s), Mem Flux	k0Sub*Oxi (mol/s), Nuc Flux	Normal total flux (mol/(m^2*s)), Point: (5.5109E-16, 2.5)	WEIectrode radius (μm), UME Radius
9	6	000	82E-10		4	9	19	16	050	20	20	6	00
1.5100E-9	5.0000E-6	0.89000	-7.5674E-10	NaN	2.0352E-4	1.5100E-9	3.8428E-19	1.2766E-16	0.19048	9.0925E-20	1.7694E-20	7.0532E-6	1.0000
1.5100E-9	5.0000E-6	0.91000	-7.5667E-10	NaN	2.0350E-4	1.5100E-9	3.6920E-19	1.2765E-16	0.19046	9.0916E-20	1.7692E-20	7.0525E-6	1.0000
1.5100E-9	5.0000E-6	0.93000	-7.5659E-10	NaN	2.0348E-4	1.5100E-9	3.5471E-19	1.2763E-16	0.19044	9.0907E-20	1.7691E-20	7.0518E-6	1.0000
1.5100E-9	5.0000E-6	0.95000	-7.5652E-10	NaN	2.0347E-4	1.5100E-9	3.4070E-19	1.2762E-16	0.19043	9.0899E-20	1.7689E-20	7.0511E-6	1.0000
1.5100E-9	5.0000E-6	0.97000	-7.5645E-10	NaN	2.0345E-4	1.5100E-9	3.2758E-19	1.2761E-16	0.19041	9.0891E-20	1.7687E-20	7.0505E-6	1.0000
1.5100E-9	5.0000E-6	0.99000	-7.5639E-10	NaN	2.0343E-4	1.5100E-9	3.1537E-19	1.2760E-16	0.19039	9.0883E-20	1.7686E-20	7.0499E-6	1.0000
1.5100E-9	6.0000E-6	0.0000	-7.1535E-9	NaN	2.2700E-4	1.5100E-9	-2.0266E-20	-1.3187E-20	0.22700	1.0088E-19	2.0266E-20	-2.2422E-10	1.0000
1.5100E-9	6.0000E-6	0.0050000	-8.5561E-10	NaN	2.2509E-4	1.5100E-9	2.5550E-16	5.4729E-17	0.22009	1.0014E-19	1.9988E-20	4.5476E-6	1.0000
1.5100E-9	6.0000E-6	0.010000	-8.2932E-10	NaN	2.2194E-4	1.5100E-9	2.2968E-16	8.1365E-17	0.21443	9.8857E-20	1.9587E-20	4.8961E-6	1.0000

k0Sub (m/s)	d (m)	Time (s)	-(tds2.ntflux_Oxi)*n*F (A), UME	Surface ConcOxi (M), OxiMem	Surface ConcOxi (M), OxiNuc	Substrate Kinetics (m/s), k0Sub probe	Normal total flux (mol/s), UME OCR membrane	Normal total flux (mol/s), UME OCR nucleus	Concentration (mol/m^3), Point: (5.5109E-16, 2.5)	k0Sub*Oxi (mol/s), Mem Flux	k0Sub*Oxi (mol/s), Nuc Flux	Normal total flux (mol/(m^2*s)), Point: (5.5109E-16, 2.5)	WElectrode radius (µm), UME Radius
1.5100E-9	6.0000E-6	0.015000	-8.1810E-10	NaN	2.1962E-4	1.5100E-9	1.6805E-16	8.7021E-17	0.21140	9.7874E-20	1.9334E-20	4.8306E-6	1.0000
1.5100E-9	6.0000E-6	0.020000	-8.1084E-10	NaN	2.1798E-4	1.5100E-9	1.3014E-16	8.9379E-17	0.20943	9.7170E-20	1.9165E-20	4.7790E-6	1.0000
1.5100E-9	6.0000E-6	0.025000	-8.0514E-10	NaN	2.1668E-4	1.5100E-9	1.0373E-16	9.0977E-17	0.20790	9.6608E-20	1.9033E-20	4.7433E-6	1.0000
1.5100E-9	6.0000E-6	0.030000	-8.0075E-10	NaN	2.1566E-4	1.5100E-9	8.5353E-17	9.2010E-17	0.20673	9.6164E-20	1.8932E-20	4.7165E-6	1.0000
1.5100E-9	6.0000E-6	0.035000	-7.9729E-10	NaN	2.1485E-4	1.5100E-9	7.1935E-17	9.2711E-17	0.20581	9.5809E-20	1.8851E-20	4.6955E-6	1.0000
1.5100E-9	6.0000E-6	0.040000	-7.9457E-10	NaN	2.1420E-4	1.5100E-9	6.2284E-17	9.3157E-17	0.20509	9.5525E-20	1.8788E-20	4.6789E-6	1.0000
1.5100E-9	6.0000E-6	0.045000	-7.9185E-10	NaN	2.1354E-4	1.5100E-9	5.2633E-17	9.3604E-17	0.20437	9.5241E-20	1.8725E-20	4.6623E-6	1.0000
1.5100E-9	6.0000E-6	0.050000	-7.8978E-10	NaN	2.1304E-4	1.5100E-9	4.6014E-17	9.3874E-17	0.20382	9.5021E-20	1.8676E-20	4.6496E-6	1.0000
1.5100E-9	6.0000E-6	0.055000	-7.8795E-10	NaN	2.1259E-4	1.5100E-9	4.0385E-17	9.4084E-17	0.20334	9.4824E-20	1.8634E-20	4.6383E-6	1.0000
1.5100E-9	6.0000E-6	0.060000	-7.8611E-	NaN	2.1214E-4	1.5100E-9	3.4756E-17	9.4294E-17	0.20285	9.4627E-20	1.8591E-20	4.6270E-6	1.0000

k0Sub (m/s)	d (m)	Time (s)	-(tds2.ntflux_Oxi)*n*F (A), UME	Surface ConcOxi (M), Oxi Mem	Surface ConcOxi (M), OxiNuc	Substrate Kinetics (m/s), k0Sub probe	Normal total flux (mol/s), UME OCR membrane	Normal total flux (mol/s), UME OCR nucleus	Concentration (mol/m^3), Point: (5.5109E-16, 2.5)	k0Sub*Oxi (mol/s), Mem Flux	k0Sub*Oxi (mol/s), Nuc Flux	Normal total flux (mol/(m^2*s)), Point: (5.5109E-16, 2.5)	WElectrode radius (µm), UME Radius
			10										
1.5100E-9	6.0000E-6	0.065000	-7.8504E-10	NaN	2.1187E-4	1.5100E-9	3.2090E-17	9.4362E-17	0.20257	9.4509E-20	1.8565E-20	4.6204E-6	1.0000
1.5100E-9	6.0000E-6	0.070000	-7.8398E-10	NaN	2.1161E-4	1.5100E-9	2.9469E-17	9.4426E-17	0.20229	9.4393E-20	1.8541E-20	4.6139E-6	1.0000
1.5100E-9	6.0000E-6	0.075000	-7.8293E-10	NaN	2.1135E-4	1.5100E-9	2.6848E-17	9.4490E-17	0.20201	9.4276E-20	1.8516E-20	4.6074E-6	1.0000
1.5100E-9	6.0000E-6	0.080000	-7.8188E-10	NaN	2.1108E-4	1.5100E-9	2.4227E-17	9.4555E-17	0.20174	9.4160E-20	1.8491E-20	4.6009E-6	1.0000
1.5100E-9	6.0000E-6	0.085000	-7.8082E-10	NaN	2.1082E-4	1.5100E-9	2.1606E-17	9.4619E-17	0.20146	9.4044E-20	1.8467E-20	4.5945E-6	1.0000
1.5100E-9	6.0000E-6	0.090000	-7.7994E-10	NaN	2.1060E-4	1.5100E-9	1.9563E-17	9.4661E-17	0.20123	9.3946E-20	1.8446E-20	4.5890E-6	1.0000
1.5100E-9	6.0000E-6	0.095000	-7.7928E-10	NaN	2.1043E-4	1.5100E-9	1.8239E-17	9.4675E-17	0.20105	9.3872E-20	1.8430E-20	4.5850E-6	1.0000
1.5100E-9	6.0000E-6	0.10000	-7.7862E-10	NaN	2.1026E-4	1.5100E-9	1.6915E-17	9.4688E-17	0.20088	9.3798E-20	1.8415E-20	4.5809E-6	1.0000
1.5100E-9	6.0000E-6	0.10500	-7.7796E-10	NaN	2.1009E-4	1.5100E-9	1.5591E-17	9.4702E-17	0.20071	9.3724E-20	1.8399E-20	4.5769E-6	1.0000
1.5100E-	6.0000E-	0.11	-7.77	NaN	2.0993E-	1.5100E-	1.4266E-	9.4715E-	0.20	9.3650E-	1.8384E-	4.5729E-	1.00

k0Sub (m/s)	d (m)	Time (s)	-(tds2.ntflux_Oxi)*n*F (A), UME	Surface Conc cOxi (M), OxiMem	Surface Conc cOxi (M), OxiNuc	Substrate Kinetics (m/s), k0Sub probe	Normal total flux (mol/s), UME OCR membrane	Normal total flux (mol/s), UME OCR nucleus	Concentration (mol/m^3), Point: (5.5109E-16, 2.5)	k0Sub*Oxi (mol/s), Mem Flux	k0Sub*Oxi (mol/s), Nuc Flux	Normal total flux (mol/(m^2*s)), Point: (5.5109E-16, 2.5)	WElectrode radius (µm), UME Radius
9	6	000	31E-10		4	9	17	17	054	20	20	6	00
1.5100E-9	6.0000E-6	0.11500	-7.7665E-10	NaN	2.0976E-4	1.5100E-9	1.2942E-17	9.4729E-17	0.20036	9.3576E-20	1.8368E-20	4.5688E-6	1.0000
1.5100E-9	6.0000E-6	0.12000	-7.7626E-10	NaN	2.0966E-4	1.5100E-9	1.2338E-17	9.4724E-17	0.20026	9.3532E-20	1.8359E-20	4.5664E-6	1.0000
1.5100E-9	6.0000E-6	0.12500	-7.7590E-10	NaN	2.0957E-4	1.5100E-9	1.1805E-17	9.4717E-17	0.20017	9.3491E-20	1.8351E-20	4.5643E-6	1.0000
1.5100E-9	6.0000E-6	0.13000	-7.7555E-10	NaN	2.0947E-4	1.5100E-9	1.1272E-17	9.4711E-17	0.20008	9.3451E-20	1.8343E-20	4.5621E-6	1.0000
1.5100E-9	6.0000E-6	0.13500	-7.7520E-10	NaN	2.0938E-4	1.5100E-9	1.0740E-17	9.4704E-17	0.19999	9.3410E-20	1.8334E-20	4.5600E-6	1.0000
1.5100E-9	6.0000E-6	0.14000	-7.7484E-10	NaN	2.0929E-4	1.5100E-9	1.0207E-17	9.4697E-17	0.19989	9.3369E-20	1.8326E-20	4.5578E-6	1.0000
1.5100E-9	6.0000E-6	0.14500	-7.7449E-10	NaN	2.0920E-4	1.5100E-9	9.6744E-18	9.4690E-17	0.19980	9.3329E-20	1.8318E-20	4.5557E-6	1.0000
1.5100E-9	6.0000E-6	0.15000	-7.7414E-10	NaN	2.0911E-4	1.5100E-9	9.1417E-18	9.4683E-17	0.19971	9.3288E-20	1.8309E-20	4.5535E-6	1.0000
1.5100E-9	6.0000E-6	0.15500	-7.7378E-10	NaN	2.0902E-4	1.5100E-9	8.6090E-18	9.4677E-17	0.19962	9.3248E-20	1.8301E-20	4.5513E-6	1.0000

k0Sub (m/s)	d (m)	Time (s)	-(tds2.ntflux_Oxi)*n*F (A), UME	Surface ConcOxi (M), OxiMem	Surface ConcOxi (M), OxiNuc	Substrate Kinetics (m/s), k0Sub probe	Normal total flux (mol/s), UME OCR membrane	Normal total flux (mol/s), UME OCR nucleus	Concentration (mol/m^3), Point: (5.5109E-16, 2.5)	k0Sub*Oxi (mol/s), Mem Flux	k0Sub*Oxi (mol/s), Nuc Flux	Normal total flux (mol/(m^2*s)), Point: (5.5109E-16, 2.5)	WEIectrode radius (µm), UME Radius
1.5100E-9	6.0000E-6	0.16000	-7.7343E-10	NaN	2.0892E-4	1.5100E-9	8.0763E-18	9.4670E-17	0.19952	9.3207E-20	1.8292E-20	4.5492E-6	1.0000
1.5100E-9	6.0000E-6	0.16500	-7.7307E-10	NaN	2.0883E-4	1.5100E-9	7.5436E-18	9.4663E-17	0.19943	9.3166E-20	1.8284E-20	4.5470E-6	1.0000
1.5100E-9	6.0000E-6	0.17000	-7.7272E-10	NaN	2.0874E-4	1.5100E-9	7.0109E-18	9.4656E-17	0.19934	9.3126E-20	1.8276E-20	4.5449E-6	1.0000
1.5100E-9	6.0000E-6	0.17500	-7.7248E-10	NaN	2.0868E-4	1.5100E-9	6.7204E-18	9.4647E-17	0.19928	9.3098E-20	1.8270E-20	4.5434E-6	1.0000
1.5100E-9	6.0000E-6	0.18000	-7.7226E-10	NaN	2.0862E-4	1.5100E-9	6.4792E-18	9.4637E-17	0.19922	9.3073E-20	1.8265E-20	4.5421E-6	1.0000
1.5100E-9	6.0000E-6	0.18500	-7.7205E-10	NaN	2.0857E-4	1.5100E-9	6.2379E-18	9.4627E-17	0.19916	9.3048E-20	1.8260E-20	4.5408E-6	1.0000
1.5100E-9	6.0000E-6	0.19000	-7.7184E-10	NaN	2.0851E-4	1.5100E-9	5.9966E-18	9.4617E-17	0.19911	9.3023E-20	1.8255E-20	4.5395E-6	1.0000
1.5100E-9	6.0000E-6	0.19500	-7.7162E-10	NaN	2.0845E-4	1.5100E-9	5.7553E-18	9.4607E-17	0.19905	9.2998E-20	1.8250E-20	4.5382E-6	1.0000
1.5100E-9	6.0000E-6	0.20000	-7.7141E-10	NaN	2.0840E-4	1.5100E-9	5.5141E-18	9.4596E-17	0.19900	9.2974E-20	1.8245E-20	4.5369E-6	1.0000
1.5100E-9	6.0000E-6	0.20500	-7.7119E-	NaN	2.0834E-4	1.5100E-9	5.2728E-18	9.4586E-17	0.19894	9.2949E-20	1.8240E-20	4.5356E-6	1.0000

k0Sub (m/s)	d (m)	Time (s)	-(tds2.ntflux_Oxi)*n*F (A), UME	Surface ConcOxi (M), OxiMem	Surface ConcOxi (M), OxiNuc	Substrate Kinetics (m/s), k0Sub probe	Normal total flux (mol/s), UME OCR membrane	Normal total flux (mol/s), UME OCR nucleus	Concentration (mol/m^3), Point: (5.5109E-16, 2.5)	k0Sub* Oxi (mol/s), Mem Flux	k0Sub* Oxi (mol/s), Nuc Flux	Normal total flux (mol/(m^2*s)), Point: (5.5109E-16, 2.5)	WElectrode radius (µm), UME Radius
			10										
1.5100E-9	6.0000E-6	0.21000	-7.7098E-10	NaN	2.0828E-4	1.5100E-9	5.0315E-18	9.4576E-17	0.19889	9.2924E-20	1.8235E-20	4.5343E-6	1.0000
1.5100E-9	6.0000E-6	0.21500	-7.7077E-10	NaN	2.0823E-4	1.5100E-9	4.7902E-18	9.4566E-17	0.19883	9.2899E-20	1.8230E-20	4.5330E-6	1.0000
1.5100E-9	6.0000E-6	0.22000	-7.7055E-10	NaN	2.0817E-4	1.5100E-9	4.5489E-18	9.4556E-17	0.19877	9.2874E-20	1.8225E-20	4.5318E-6	1.0000
1.5100E-9	6.0000E-6	0.22500	-7.7034E-10	NaN	2.0812E-4	1.5100E-9	4.3077E-18	9.4546E-17	0.19872	9.2849E-20	1.8220E-20	4.5305E-6	1.0000
1.5100E-9	6.0000E-6	0.23000	-7.7019E-10	NaN	2.0808E-4	1.5100E-9	4.1770E-18	9.4537E-17	0.19868	9.2832E-20	1.8216E-20	4.5296E-6	1.0000
1.5100E-9	6.0000E-6	0.23500	-7.7007E-10	NaN	2.0805E-4	1.5100E-9	4.0817E-18	9.4529E-17	0.19865	9.2818E-20	1.8213E-20	4.5289E-6	1.0000
1.5100E-9	6.0000E-6	0.24000	-7.6995E-10	NaN	2.0802E-4	1.5100E-9	3.9864E-18	9.4520E-17	0.19862	9.2804E-20	1.8211E-20	4.5281E-6	1.0000
1.5100E-9	6.0000E-6	0.24500	-7.6984E-10	NaN	2.0798E-4	1.5100E-9	3.8911E-18	9.4512E-17	0.19859	9.2790E-20	1.8208E-20	4.5274E-6	1.0000
1.5100E-9	6.0000E-6	0.25000	-7.6972E-10	NaN	2.0795E-4	1.5100E-9	3.7958E-18	9.4503E-17	0.19856	9.2776E-20	1.8205E-20	4.5267E-6	1.0000
1.5100E-9	6.0000E-	0.25	-7.69	NaN	2.0795E-	1.5100E-	3.7958E-	9.4503E-	0.19	9.2776E-	1.8205E-	4.5267E-	1.00

k0Sub (m/s)	d (m)	Time (s)	-(tds2.ntflux_Oxi)*n*F (A), UME	Surface Conc cOxi (M), Oxi Mem	Surface Conc cOxi (M), OxiNuc	Substrate Kinetics (m/s), k0Sub probe	Normal total flux (mol/s), UME OCR membrane	Normal total flux (mol/s), UME OCR nucleus	Concentration (mol/m^3), Point: (5.5109E-16, 2.5)	k0Sub* Oxi (mol/s), Mem Flux	k0Sub* Oxi (mol/s), Nuc Flux	Normal total flux (mol/(m^2*s)), Point: (5.5109E-16, 2.5)	WElectrode radius (μm), UME Radius
9	6	000	72E-10		4	9	18	17	856	20	20	6	00
1.5100E-9	6.0000E-6	0.27000	-7.6924E-10	NaN	2.0783E-4	1.5100E-9	3.4145E-18	9.4470E-17	0.19843	9.2720E-20	1.8194E-20	4.5238E-6	1.0000
1.5100E-9	6.0000E-6	0.29000	-7.6876E-10	NaN	2.0770E-4	1.5100E-9	3.0332E-18	9.4436E-17	0.19831	9.2664E-20	1.8182E-20	4.5210E-6	1.0000
1.5100E-9	6.0000E-6	0.31000	-7.6829E-10	NaN	2.0757E-4	1.5100E-9	2.6520E-18	9.4403E-17	0.19819	9.2608E-20	1.8171E-20	4.5181E-6	1.0000
1.5100E-9	6.0000E-6	0.33000	-7.6785E-10	NaN	2.0746E-4	1.5100E-9	2.3209E-18	9.4371E-17	0.19807	9.2557E-20	1.8161E-20	4.5155E-6	1.0000
1.5100E-9	6.0000E-6	0.35000	-7.6755E-10	NaN	2.0738E-4	1.5100E-9	2.1459E-18	9.4346E-17	0.19800	9.2521E-20	1.8154E-20	4.5137E-6	1.0000
1.5100E-9	6.0000E-6	0.37000	-7.6725E-10	NaN	2.0730E-4	1.5100E-9	1.9710E-18	9.4320E-17	0.19792	9.2486E-20	1.8147E-20	4.5119E-6	1.0000
1.5100E-9	6.0000E-6	0.39000	-7.6695E-10	NaN	2.0722E-4	1.5100E-9	1.7960E-18	9.4294E-17	0.19784	9.2450E-20	1.8140E-20	4.5101E-6	1.0000
1.5100E-9	6.0000E-6	0.41000	-7.6665E-10	NaN	2.0714E-4	1.5100E-9	1.6211E-18	9.4269E-17	0.19776	9.2415E-20	1.8133E-20	4.5083E-6	1.0000
1.5100E-9	6.0000E-6	0.43000	-7.6638E-10	NaN	2.0707E-4	1.5100E-9	1.4705E-18	9.4245E-17	0.19769	9.2382E-20	1.8126E-20	4.5067E-6	1.0000

k0Sub (m/s)	d (m)	Time (s)	-(tds2.ntflux_Oxi)*n*F (A), UME	Surface ConcOxi (M), Oxi Mem	Surface ConcOxi (M), OxiNuc	Substrate Kinetics (m/s), k0Sub probe	Normal total flux (mol/s), UME OCR membrane	Normal total flux (mol/s), UME OCR nucleus	Concentration (mol/m^3), Point: (5.5109E-16, 2.5)	k0Sub*Oxi (mol/s), Mem Flux	k0Sub*Oxi (mol/s), Nuc Flux	Normal total flux (mol/(m^2*s)), Point: (5.5109E-16, 2.5)	WEIectrode radius (µm), UME Radius
1.5100E-9	6.0000E-6	0.45000	-7.6617E-10	NaN	2.0701E-4	1.5100E-9	1.3776E-18	9.4225E-17	0.19764	9.2358E-20	1.8121E-20	4.5055E-6	1.0000
1.5100E-9	6.0000E-6	0.47000	-7.6596E-10	NaN	2.0696E-4	1.5100E-9	1.2846E-18	9.4206E-17	0.19758	9.2333E-20	1.8116E-20	4.5042E-6	1.0000
1.5100E-9	6.0000E-6	0.49000	-7.6576E-10	NaN	2.0690E-4	1.5100E-9	1.1916E-18	9.4186E-17	0.19753	9.2309E-20	1.8111E-20	4.5030E-6	1.0000
1.5100E-9	6.0000E-6	0.51000	-7.6555E-10	NaN	2.0685E-4	1.5100E-9	1.0986E-18	9.4167E-17	0.19748	9.2284E-20	1.8107E-20	4.5018E-6	1.0000
1.5100E-9	6.0000E-6	0.53000	-7.6536E-10	NaN	2.0679E-4	1.5100E-9	1.0190E-18	9.4149E-17	0.19743	9.2261E-20	1.8102E-20	4.5006E-6	1.0000
1.5100E-9	6.0000E-6	0.55000	-7.6521E-10	NaN	2.0675E-4	1.5100E-9	9.6390E-19	9.4133E-17	0.19739	9.2243E-20	1.8098E-20	4.4997E-6	1.0000
1.5100E-9	6.0000E-6	0.57000	-7.6505E-10	NaN	2.0671E-4	1.5100E-9	9.0880E-19	9.4118E-17	0.19735	9.2225E-20	1.8095E-20	4.4988E-6	1.0000
1.5100E-9	6.0000E-6	0.59000	-7.6490E-10	NaN	2.0667E-4	1.5100E-9	8.5370E-19	9.4103E-17	0.19731	9.2207E-20	1.8091E-20	4.4979E-6	1.0000
1.5100E-9	6.0000E-6	0.61000	-7.6475E-10	NaN	2.0663E-4	1.5100E-9	7.9860E-19	9.4087E-17	0.19727	9.2188E-20	1.8088E-20	4.4970E-6	1.0000
1.5100E-9	6.0000E-6	0.63000	-7.6461E-	NaN	2.0659E-4	1.5100E-9	7.5083E-19	9.4073E-17	0.19723	9.2171E-20	1.8084E-20	4.4962E-6	1.0000

k0Sub (m/s)	d (m)	Time (s)	-(tds2.ntflux_Oxi)*n*F (A), UME	Surface ConcOxi (M), OxiMem	Surface ConcOxi (M), OxiNuc	Substrate Kinetics (m/s), k0Sub probe	Normal total flux (mol/s), UME OCR membrane	Normal total flux (mol/s), UME OCR nucleus	Concentration (mol/m^3), Point: (5.5109E-16, 2.5)	k0Sub*Oxi (mol/s), Mem Flux	k0Sub*Oxi (mol/s), Nuc Flux	Normal total flux (mol/(m^2*s)), Point: (5.5109E-16, 2.5)	WElectrode radius (µm), UME Radius
			10										
1.5100E-9	6.0000E-6	0.65000	-7.6448E-10	NaN	2.0656E-4	1.5100E-9	7.1366E-19	9.4060E-17	0.19720	9.2157E-20	1.8081E-20	4.4954E-6	1.0000
1.5100E-9	6.0000E-6	0.67000	-7.6436E-10	NaN	2.0653E-4	1.5100E-9	6.7649E-19	9.4048E-17	0.19717	9.2142E-20	1.8078E-20	4.4947E-6	1.0000
1.5100E-9	6.0000E-6	0.69000	-7.6424E-10	NaN	2.0650E-4	1.5100E-9	6.3932E-19	9.4035E-17	0.19714	9.2128E-20	1.8076E-20	4.4940E-6	1.0000
1.5100E-9	6.0000E-6	0.71000	-7.6412E-10	NaN	2.0646E-4	1.5100E-9	6.0215E-19	9.4023E-17	0.19711	9.2113E-20	1.8073E-20	4.4933E-6	1.0000
1.5100E-9	6.0000E-6	0.73000	-7.6401E-10	NaN	2.0643E-4	1.5100E-9	5.7077E-19	9.4011E-17	0.19708	9.2100E-20	1.8070E-20	4.4926E-6	1.0000
1.5100E-9	6.0000E-6	0.75000	-7.6391E-10	NaN	2.0641E-4	1.5100E-9	5.4612E-19	9.4001E-17	0.19705	9.2088E-20	1.8068E-20	4.4920E-6	1.0000
1.5100E-9	6.0000E-6	0.77000	-7.6382E-10	NaN	2.0638E-4	1.5100E-9	5.2146E-19	9.3990E-17	0.19703	9.2077E-20	1.8066E-20	4.4915E-6	1.0000
1.5100E-9	6.0000E-6	0.79000	-7.6372E-10	NaN	2.0635E-4	1.5100E-9	4.9681E-19	9.3980E-17	0.19700	9.2065E-20	1.8063E-20	4.4909E-6	1.0000
1.5100E-9	6.0000E-6	0.81000	-7.6362E-10	NaN	2.0633E-4	1.5100E-9	4.7215E-19	9.3970E-17	0.19698	9.2054E-20	1.8061E-20	4.4903E-6	1.0000
1.5100E-	6.0000E-	0.83	-7.63	NaN	2.0630E-	1.5100E-	4.4948E-	9.3960E-	0.19	9.2043E-	1.8059E-	4.4898E-	1.00

k0Sub (m/s)	d (m)	Time (s)	-(tds2.ntflux_Oxi)*n*F (A), UME	Surface ConcOxi (M), Oxi Mem	Surface ConcOxi (M), OxiNuc	Substrate Kinetics (m/s), k0Sub probe	Normal total flux (mol/s), UME OCR membrane	Normal total flux (mol/s), UME OCR nucleus	Concentration (mol/m^3), Point: (5.5109E-16, 2.5)	k0Sub*Oxi (mol/s), Mem Flux	k0Sub*Oxi (mol/s), Nuc Flux	Normal total flux (mol/(m^2*s)), Point: (5.5109E-16, 2.5)	WElectrode radius (µm), UME Radius
9	6	000	53E-10		4	9	19	17	695	20	20	6	00
1.5100E-9	6.0000E-6	0.85000	-7.6344E-10	NaN	2.0628E-4	1.5100E-9	4.2974E-19	9.3950E-17	0.19693	9.2032E-20	1.8057E-20	4.4893E-6	1.0000
1.5100E-9	6.0000E-6	0.87000	-7.6336E-10	NaN	2.0626E-4	1.5100E-9	4.1140E-19	9.3941E-17	0.19691	9.2022E-20	1.8055E-20	4.4888E-6	1.0000
1.5100E-9	6.0000E-6	0.89000	-7.6328E-10	NaN	2.0624E-4	1.5100E-9	3.9446E-19	9.3933E-17	0.19689	9.2013E-20	1.8053E-20	4.4883E-6	1.0000
1.5100E-9	6.0000E-6	0.91000	-7.6320E-10	NaN	2.0622E-4	1.5100E-9	3.7893E-19	9.3924E-17	0.19687	9.2004E-20	1.8051E-20	4.4879E-6	1.0000
1.5100E-9	6.0000E-6	0.93000	-7.6313E-10	NaN	2.0620E-4	1.5100E-9	3.6408E-19	9.3916E-17	0.19685	9.1995E-20	1.8049E-20	4.4874E-6	1.0000
1.5100E-9	6.0000E-6	0.95000	-7.6306E-10	NaN	2.0618E-4	1.5100E-9	3.4967E-19	9.3908E-17	0.19683	9.1986E-20	1.8048E-20	4.4870E-6	1.0000
1.5100E-9	6.0000E-6	0.97000	-7.6299E-10	NaN	2.0616E-4	1.5100E-9	3.3617E-19	9.3900E-17	0.19681	9.1978E-20	1.8046E-20	4.4866E-6	1.0000
1.5100E-9	6.0000E-6	0.99000	-7.6292E-10	NaN	2.0614E-4	1.5100E-9	3.2361E-19	9.3893E-17	0.19680	9.1970E-20	1.8044E-20	4.4862E-6	1.0000
1.5100E-9	8.0000E-6	0.0000	-7.1631E-9	NaN	2.2700E-4	1.5100E-9	-2.0266E-20	-1.3256E-20	0.22700	1.0088E-19	2.0266E-20	-2.2911E-10	1.0000

k0Sub (m/s)	d (m)	Time (s)	-(tds2.ntflux_Oxi)*n*F (A), UME	Surface ConcOxi (M), OxiMem	Surface ConcOxi (M), OxiNuc	Substrate Kinetics (m/s), k0Sub probe	Normal total flux (mol/s), UME OCR membrane	Normal total flux (mol/s), UME OCR nucleus	Concentration (mol/m^3), Point: (5.5109E-16, 2.5)	k0Sub*Oxi (mol/s), MemFlux	k0Sub*Oxi (mol/s), NucFlux	Normal total flux (mol/(m^2*s)), Point: (5.5109E-16, 2.5)	WEIectrode radius (µm), UME Radius
1.5100E-9	8.0000E-6	0.005000	-8.5501E-10	NaN	2.2634E-4	1.5100E-9	1.1823E-16	1.8650E-17	0.22471	1.0062E-19	2.0177E-20	1.6812E-6	1.0000
1.5100E-9	8.0000E-6	0.010000	-8.3181E-10	NaN	2.2446E-4	1.5100E-9	1.5318E-16	3.7549E-17	0.22118	9.9847E-20	1.9943E-20	2.2342E-6	1.0000
1.5100E-9	8.0000E-6	0.015000	-8.2206E-10	NaN	2.2278E-4	1.5100E-9	1.2864E-16	4.3851E-17	0.21884	9.9136E-20	1.9757E-20	2.2948E-6	1.0000
1.5100E-9	8.0000E-6	0.020000	-8.1570E-10	NaN	2.2149E-4	1.5100E-9	1.0710E-16	4.6889E-17	0.21720	9.8585E-20	1.9622E-20	2.3077E-6	1.0000
1.5100E-9	8.0000E-6	0.025000	-8.1088E-10	NaN	2.2043E-4	1.5100E-9	8.9636E-17	4.8731E-17	0.21592	9.8128E-20	1.9514E-20	2.3067E-6	1.0000
1.5100E-9	8.0000E-6	0.030000	-8.0694E-10	NaN	2.1953E-4	1.5100E-9	7.5369E-17	5.0047E-17	0.21485	9.7740E-20	1.9423E-20	2.3024E-6	1.0000
1.5100E-9	8.0000E-6	0.035000	-8.0368E-10	NaN	2.1876E-4	1.5100E-9	6.3733E-17	5.0961E-17	0.21396	9.7403E-20	1.9347E-20	2.2957E-6	1.0000
1.5100E-9	8.0000E-6	0.040000	-8.0131E-10	NaN	2.1818E-4	1.5100E-9	5.5738E-17	5.1506E-17	0.21330	9.7151E-20	1.9290E-20	2.2896E-6	1.0000
1.5100E-9	8.0000E-6	0.045000	-7.9938E-10	NaN	2.1770E-4	1.5100E-9	4.9727E-17	5.1866E-17	0.21277	9.6941E-20	1.9243E-20	2.2838E-6	1.0000
1.5100E-9	8.0000E-6	0.050000	-7.9745E-	NaN	2.1722E-4	1.5100E-9	4.3717E-17	5.2226E-17	0.21224	9.6730E-20	1.9197E-20	2.2779E-6	1.0000

k0Sub (m/s)	d (m)	Time (s)	-(tds2.ntflux_Oxi)*n*F (A), UME	Surface ConcOxi (M), OxiMem	Surface ConcOxi (M), OxiNuc	Substrate Kinetics (m/s), k0Sub probe	Normal total flux (mol/s), UME OCR membrane	Normal total flux (mol/s), UME OCR nucleus	Concentration (mol/m^3), Point: (5.5109E-16, 2.5)	k0Sub*Oxi (mol/s), MemFlux	k0Sub*Oxi (mol/s), NucFlux	Normal total flux (mol/(m^2*s)), Point: (5.5109E-16, 2.5)	WElectrode radius (µm), UME Radius
			10										
1.5100E-9	8.0000E-6	0.055000	-7.9553E-10	NaN	2.1674E-4	1.5100E-9	3.7706E-17	5.2587E-17	0.21170	9.6520E-20	1.9150E-20	2.2721E-6	1.0000
1.5100E-9	8.0000E-6	0.060000	-7.9420E-10	NaN	2.1640E-4	1.5100E-9	3.4007E-17	5.2770E-17	0.21134	9.6371E-20	1.9118E-20	2.2678E-6	1.0000
1.5100E-9	8.0000E-6	0.065000	-7.9296E-10	NaN	2.1608E-4	1.5100E-9	3.0735E-17	5.2927E-17	0.21099	9.6231E-20	1.9088E-20	2.2636E-6	1.0000
1.5100E-9	8.0000E-6	0.070000	-7.9172E-10	NaN	2.1576E-4	1.5100E-9	2.7464E-17	5.3083E-17	0.21065	9.6091E-20	1.9058E-20	2.2595E-6	1.0000
1.5100E-9	8.0000E-6	0.075000	-7.9051E-10	NaN	2.1545E-4	1.5100E-9	2.4262E-17	5.3232E-17	0.21032	9.5955E-20	1.9029E-20	2.2555E-6	1.0000
1.5100E-9	8.0000E-6	0.080000	-7.8983E-10	NaN	2.1527E-4	1.5100E-9	2.2850E-17	5.3282E-17	0.21013	9.5876E-20	1.9012E-20	2.2532E-6	1.0000
1.5100E-9	8.0000E-6	0.085000	-7.8914E-10	NaN	2.1509E-4	1.5100E-9	2.1438E-17	5.3332E-17	0.20994	9.5797E-20	1.8995E-20	2.2509E-6	1.0000
1.5100E-9	8.0000E-6	0.090000	-7.8845E-10	NaN	2.1491E-4	1.5100E-9	2.0026E-17	5.3381E-17	0.20975	9.5718E-20	1.8978E-20	2.2486E-6	1.0000
1.5100E-9	8.0000E-6	0.095000	-7.8777E-10	NaN	2.1473E-4	1.5100E-9	1.8614E-17	5.3431E-17	0.20956	9.5638E-20	1.8962E-20	2.2462E-6	1.0000
1.5100E-	8.0000E-	0.10	-7.87	NaN	2.1455E-	1.5100E-	1.7202E-	5.3481E-	0.20	9.5559E-	1.8945E-	2.2439E-	1.00

k0Sub (m/s)	d (m)	Time (s)	-(tds2.ntflux_Oxi)*n*F (A), UME	Surface ConcOxi (M), Oxi Mem	Surface ConcOxi (M), OxiNuc	Substrate Kinetics (m/s), k0Sub probe	Normal total flux (mol/s), UME OCR membrane	Normal total flux (mol/s), UME OCR nucleus	Concentration (mol/m^3), Point: (5.5109E-16, 2.5)	k0Sub*Oxi (mol/s), Mem Flux	k0Sub*Oxi (mol/s), Nuc Flux	Normal total flux (mol/(m^2*s)), Point: (5.5109E-16, 2.5)	WElectrode radius (µm), UME Radius
9	6	000	08E-10		4	9	17	17	937	20	20	6	00
1.5100E-9	8.0000E-6	0.10500	-7.8639E-10	NaN	2.1437E-4	1.5100E-9	1.5790E-17	5.3530E-17	0.20919	9.5480E-20	1.8928E-20	2.2416E-6	1.0000
1.5100E-9	8.0000E-6	0.11000	-7.8571E-10	NaN	2.1419E-4	1.5100E-9	1.4378E-17	5.3580E-17	0.20900	9.5401E-20	1.8912E-20	2.2393E-6	1.0000
1.5100E-9	8.0000E-6	0.11500	-7.8514E-10	NaN	2.1404E-4	1.5100E-9	1.3282E-17	5.3614E-17	0.20884	9.5335E-20	1.8898E-20	2.2374E-6	1.0000
1.5100E-9	8.0000E-6	0.12000	-7.8472E-10	NaN	2.1393E-4	1.5100E-9	1.2608E-17	5.3629E-17	0.20873	9.5286E-20	1.8888E-20	2.2361E-6	1.0000
1.5100E-9	8.0000E-6	0.12500	-7.8430E-10	NaN	2.1382E-4	1.5100E-9	1.1934E-17	5.3644E-17	0.20861	9.5237E-20	1.8877E-20	2.2347E-6	1.0000
1.5100E-9	8.0000E-6	0.13000	-7.8388E-10	NaN	2.1371E-4	1.5100E-9	1.1260E-17	5.3659E-17	0.20850	9.5188E-20	1.8867E-20	2.2333E-6	1.0000
1.5100E-9	8.0000E-6	0.13500	-7.8346E-10	NaN	2.1360E-4	1.5100E-9	1.0586E-17	5.3674E-17	0.20838	9.5139E-20	1.8857E-20	2.2319E-6	1.0000
1.5100E-9	8.0000E-6	0.14000	-7.8304E-10	NaN	2.1349E-4	1.5100E-9	9.9117E-18	5.3689E-17	0.20827	9.5089E-20	1.8847E-20	2.2305E-6	1.0000
1.5100E-9	8.0000E-6	0.14500	-7.8262E-10	NaN	2.1338E-4	1.5100E-9	9.2375E-18	5.3705E-17	0.20815	9.5040E-20	1.8836E-20	2.2291E-6	1.0000

k0Sub (m/s)	d (m)	Time (s)	-(tds2.ntflux_Oxi)*n*F (A), UME	Surface ConcOxi (M), OxiMem	Surface ConcOxi (M), OxiNuc	Substrate Kinetics (m/s), k0Sub probe	Normal total flux (mol/s), UME OCR membrane	Normal total flux (mol/s), UME OCR nucleus	Concentration (mol/m^3), Point: (5.5109E-16, 2.5)	k0Sub*Oxi (mol/s), Mem Flux	k0Sub*Oxi (mol/s), Nuc Flux	Normal total flux (mol/(m^2*s)), Point: (5.5109E-16, 2.5)	WElectrode radius (µm), UME Radius
1.5100E-9	8.0000E-6	0.15000	-7.8220E-10	NaN	2.1326E-4	1.5100E-9	8.5634E-18	5.3720E-17	0.20804	9.4991E-20	1.8826E-20	2.2277E-6	1.0000
1.5100E-9	8.0000E-6	0.15500	-7.8195E-10	NaN	2.1320E-4	1.5100E-9	8.2274E-18	5.3723E-17	0.20797	9.4961E-20	1.8820E-20	2.2269E-6	1.0000
1.5100E-9	8.0000E-6	0.16000	-7.8172E-10	NaN	2.1314E-4	1.5100E-9	7.9676E-18	5.3724E-17	0.20791	9.4934E-20	1.8815E-20	2.2262E-6	1.0000
1.5100E-9	8.0000E-6	0.16500	-7.8150E-10	NaN	2.1308E-4	1.5100E-9	7.7077E-18	5.3724E-17	0.20785	9.4908E-20	1.8809E-20	2.2255E-6	1.0000
1.5100E-9	8.0000E-6	0.17000	-7.8127E-10	NaN	2.1302E-4	1.5100E-9	7.4478E-18	5.3725E-17	0.20779	9.4881E-20	1.8804E-20	2.2248E-6	1.0000
1.5100E-9	8.0000E-6	0.17500	-7.8105E-10	NaN	2.1296E-4	1.5100E-9	7.1880E-18	5.3726E-17	0.20773	9.4855E-20	1.8798E-20	2.2240E-6	1.0000
1.5100E-9	8.0000E-6	0.18000	-7.8083E-10	NaN	2.1290E-4	1.5100E-9	6.9281E-18	5.3727E-17	0.20767	9.4828E-20	1.8793E-20	2.2233E-6	1.0000
1.5100E-9	8.0000E-6	0.18500	-7.8060E-10	NaN	2.1284E-4	1.5100E-9	6.6683E-18	5.3728E-17	0.20761	9.4802E-20	1.8787E-20	2.2226E-6	1.0000
1.5100E-9	8.0000E-6	0.19000	-7.8038E-10	NaN	2.1278E-4	1.5100E-9	6.4084E-18	5.3729E-17	0.20755	9.4775E-20	1.8782E-20	2.2219E-6	1.0000
1.5100E-9	8.0000E-6	0.19500	-7.8015E-	NaN	2.1272E-4	1.5100E-9	6.1485E-18	5.3730E-17	0.20748	9.4748E-20	1.8777E-20	2.2212E-6	1.0000

k0Sub (m/s)	d (m)	Time (s)	-(tds2.ntflux_Oxi)*n*F (A), UME	Surface Con cOxi (M), Oxi Mem	Surface Con cOxi (M), OxiNuc	Substrate Kinetics (m/s), k0Sub probe	Normal total flux (mol/s), UME OCR membrane	Normal total flux (mol/s), UME OCR nucleus	Concentration (mol/m^3), Point: (5.5109E-16, 2.5)	k0Sub*Oxi (mol/s), Mem Flux	k0Sub*Oxi (mol/s), Nuc Flux	Normal total flux (mol/(m^2*s)), Point: (5.5109E-16, 2.5)	WElectrode radius (µm), UME Radius
			10										
1.5100E-9	8.0000E-6	0.20000	-7.7993E-10	NaN	2.1266E-4	1.5100E-9	5.8887E-18	5.3731E-17	0.20742	9.4722E-20	1.8771E-20	2.2204E-6	1.0000
1.5100E-9	8.0000E-6	0.20500	-7.7970E-10	NaN	2.1260E-4	1.5100E-9	5.6288E-18	5.3732E-17	0.20736	9.4695E-20	1.8766E-20	2.2197E-6	1.0000
1.5100E-9	8.0000E-6	0.21000	-7.7948E-10	NaN	2.1254E-4	1.5100E-9	5.3689E-18	5.3732E-17	0.20730	9.4669E-20	1.8760E-20	2.2190E-6	1.0000
1.5100E-9	8.0000E-6	0.21500	-7.7926E-10	NaN	2.1248E-4	1.5100E-9	5.1091E-18	5.3733E-17	0.20724	9.4642E-20	1.8755E-20	2.2183E-6	1.0000
1.5100E-9	8.0000E-6	0.22000	-7.7903E-10	NaN	2.1242E-4	1.5100E-9	4.8492E-18	5.3734E-17	0.20718	9.4615E-20	1.8749E-20	2.2176E-6	1.0000
1.5100E-9	8.0000E-6	0.22500	-7.7881E-10	NaN	2.1236E-4	1.5100E-9	4.5893E-18	5.3735E-17	0.20712	9.4589E-20	1.8744E-20	2.2168E-6	1.0000
1.5100E-9	8.0000E-6	0.23000	-7.7864E-10	NaN	2.1231E-4	1.5100E-9	4.4164E-18	5.3734E-17	0.20707	9.4569E-20	1.8740E-20	2.2163E-6	1.0000
1.5100E-9	8.0000E-6	0.23500	-7.7850E-10	NaN	2.1228E-4	1.5100E-9	4.3016E-18	5.3732E-17	0.20704	9.4553E-20	1.8737E-20	2.2159E-6	1.0000
1.5100E-9	8.0000E-6	0.24000	-7.7837E-10	NaN	2.1224E-4	1.5100E-9	4.1868E-18	5.3729E-17	0.20700	9.4537E-20	1.8733E-20	2.2155E-6	1.0000
1.5100E-	8.0000E-	0.24	-7.78	NaN	2.1220E-	1.5100E-	4.0719E-	5.3727E-	0.20	9.4521E-	1.8730E-	2.2151E-	1.00

k0Sub (m/s)	d (m)	Time (s)	-(tds2.ntflux_Oxi)*n*F (A), UME	Surface ConcOxi (M), OxiMem	Surface ConcOxi (M), OxiNuc	Substrate Kinetics (m/s), k0Sub probe	Normal total flux (mol/s), UME OCR membrane	Normal total flux (mol/s), UME OCR nucleus	Concentration (mol/m^3), Point: (5.5109E-16, 2.5)	k0Sub*Oxi (mol/s), Mem Flux	k0Sub*Oxi (mol/s), Nuc Flux	Normal total flux (mol/(m^2*s)), Point: (5.5109E-16, 2.5)	WElectrode radius (µm), UME Radius
9	6	500	23E-10		4	9	18	17	697	20	20	6	00
1.5100E-9	8.0000E-6	0.25000	-7.7810E-10	NaN	2.1217E-4	1.5100E-9	3.9571E-18	5.3725E-17	0.20693	9.4504E-20	1.8727E-20	2.2146E-6	1.0000
1.5100E-9	8.0000E-6	0.25000	-7.7810E-10	NaN	2.1217E-4	1.5100E-9	3.9571E-18	5.3725E-17	0.20693	9.4504E-20	1.8727E-20	2.2146E-6	1.0000
1.5100E-9	8.0000E-6	0.27000	-7.7756E-10	NaN	2.1202E-4	1.5100E-9	3.4979E-18	5.3715E-17	0.20678	9.4440E-20	1.8714E-20	2.2129E-6	1.0000
1.5100E-9	8.0000E-6	0.29000	-7.7701E-10	NaN	2.1188E-4	1.5100E-9	3.0387E-18	5.3706E-17	0.20664	9.4375E-20	1.8700E-20	2.2113E-6	1.0000
1.5100E-9	8.0000E-6	0.31000	-7.7655E-10	NaN	2.1175E-4	1.5100E-9	2.6653E-18	5.3696E-17	0.20651	9.4319E-20	1.8689E-20	2.2098E-6	1.0000
1.5100E-9	8.0000E-6	0.33000	-7.7621E-10	NaN	2.1166E-4	1.5100E-9	2.4559E-18	5.3685E-17	0.20642	9.4279E-20	1.8681E-20	2.2088E-6	1.0000
1.5100E-9	8.0000E-6	0.35000	-7.7587E-10	NaN	2.1157E-4	1.5100E-9	2.2466E-18	5.3675E-17	0.20633	9.4238E-20	1.8673E-20	2.2077E-6	1.0000
1.5100E-9	8.0000E-6	0.37000	-7.7554E-10	NaN	2.1148E-4	1.5100E-9	2.0372E-18	5.3664E-17	0.20624	9.4198E-20	1.8665E-20	2.2067E-6	1.0000
1.5100E-9	8.0000E-6	0.39000	-7.7520E-10	NaN	2.1139E-4	1.5100E-9	1.8278E-18	5.3653E-17	0.20615	9.4158E-20	1.8657E-20	2.2057E-6	1.0000

k0Sub (m/s)	d (m)	Time (s)	-(tds2.ntflux_Oxi)*n*F (A), UME	Surface ConcOxi (M), OxiMem	Surface ConcOxi (M), OxiNuc	Substrate Kinetics (m/s), k0Sub probe	Normal total flux (mol/s), UME OCR membrane	Normal total flux (mol/s), UME OCR nucleus	Concentration (mol/m^3), Point: (5.5109E-16, 2.5)	k0Sub*Oxi (mol/s), Mem Flux	k0Sub*Oxi (mol/s), Nuc Flux	Normal total flux (mol/(m^2*s)), Point: (5.5109E-16, 2.5)	WElectrode radius (µm), UME Radius
1.5100E-9	8.0000E-6	0.41000	-7.7491E-10	NaN	2.1131E-4	1.5100E-9	1.6574E-18	5.3643E-17	0.20607	9.4123E-20	1.8649E-20	2.2048E-6	1.0000
1.5100E-9	8.0000E-6	0.43000	-7.7468E-10	NaN	2.1125E-4	1.5100E-9	1.5484E-18	5.3634E-17	0.20601	9.4095E-20	1.8644E-20	2.2041E-6	1.0000
1.5100E-9	8.0000E-6	0.45000	-7.7445E-10	NaN	2.1118E-4	1.5100E-9	1.4394E-18	5.3624E-17	0.20595	9.4067E-20	1.8638E-20	2.2034E-6	1.0000
1.5100E-9	8.0000E-6	0.47000	-7.7422E-10	NaN	2.1112E-4	1.5100E-9	1.3304E-18	5.3615E-17	0.20589	9.4040E-20	1.8633E-20	2.2027E-6	1.0000
1.5100E-9	8.0000E-6	0.49000	-7.7399E-10	NaN	2.1106E-4	1.5100E-9	1.2214E-18	5.3606E-17	0.20582	9.4012E-20	1.8627E-20	2.2020E-6	1.0000
1.5100E-9	8.0000E-6	0.51000	-7.7379E-10	NaN	2.1100E-4	1.5100E-9	1.1321E-18	5.3597E-17	0.20577	9.3988E-20	1.8622E-20	2.2014E-6	1.0000
1.5100E-9	8.0000E-6	0.53000	-7.7362E-10	NaN	2.1096E-4	1.5100E-9	1.0687E-18	5.3589E-17	0.20573	9.3968E-20	1.8618E-20	2.2010E-6	1.0000
1.5100E-9	8.0000E-6	0.55000	-7.7346E-10	NaN	2.1091E-4	1.5100E-9	1.0052E-18	5.3581E-17	0.20568	9.3948E-20	1.8614E-20	2.2005E-6	1.0000
1.5100E-9	8.0000E-6	0.57000	-7.7329E-10	NaN	2.1087E-4	1.5100E-9	9.4181E-19	5.3573E-17	0.20564	9.3928E-20	1.8610E-20	2.2000E-6	1.0000
1.5100E-9	8.0000E-6	0.59000	-7.7312E-	NaN	2.1082E-4	1.5100E-9	8.7838E-19	5.3566E-17	0.20559	9.3907E-20	1.8606E-20	2.1995E-6	1.0000

k0Sub (m/s)	d (m)	Time (s)	-(tds2.ntflux_Oxi)*n*F (A), UME	Surface ConcOxi (M), OxiMem	Surface ConcOxi (M), OxiNuc	Substrate Kinetics (m/s), k0Sub probe	Normal total flux (mol/s), UME OCR membrane	Normal total flux (mol/s), UME OCR nucleus	Concentration (mol/m^3), Point: (5.5109E-16, 2.5)	k0Sub*Oxi (mol/s), Mem Flux	k0Sub*Oxi (mol/s), Nuc Flux	Normal total flux (mol/(m^2*s)), Point: (5.5109E-16, 2.5)	WElectrode radius (µm), UME Radius
			10										
1.5100E-9	8.0000E-6	0.61000	-7.7297E-10	NaN	2.1078E-4	1.5100E-9	8.2501E-19	5.3558E-17	0.20555	9.3889E-20	1.8603E-20	2.1990E-6	1.0000
1.5100E-9	8.0000E-6	0.63000	-7.7284E-10	NaN	2.1075E-4	1.5100E-9	7.8277E-19	5.3552E-17	0.20552	9.3873E-20	1.8600E-20	2.1986E-6	1.0000
1.5100E-9	8.0000E-6	0.65000	-7.7271E-10	NaN	2.1071E-4	1.5100E-9	7.4052E-19	5.3545E-17	0.20548	9.3858E-20	1.8596E-20	2.1982E-6	1.0000
1.5100E-9	8.0000E-6	0.67000	-7.7258E-10	NaN	2.1067E-4	1.5100E-9	6.9827E-19	5.3538E-17	0.20545	9.3842E-20	1.8593E-20	2.1978E-6	1.0000
1.5100E-9	8.0000E-6	0.69000	-7.7244E-10	NaN	2.1064E-4	1.5100E-9	6.5603E-19	5.3532E-17	0.20541	9.3826E-20	1.8590E-20	2.1975E-6	1.0000
1.5100E-9	8.0000E-6	0.71000	-7.7233E-10	NaN	2.1061E-4	1.5100E-9	6.2137E-19	5.3526E-17	0.20538	9.3812E-20	1.8587E-20	2.1971E-6	1.0000
1.5100E-9	8.0000E-6	0.73000	-7.7222E-10	NaN	2.1058E-4	1.5100E-9	5.9374E-19	5.3520E-17	0.20535	9.3799E-20	1.8585E-20	2.1968E-6	1.0000
1.5100E-9	8.0000E-6	0.75000	-7.7212E-10	NaN	2.1055E-4	1.5100E-9	5.6611E-19	5.3514E-17	0.20532	9.3786E-20	1.8582E-20	2.1965E-6	1.0000
1.5100E-9	8.0000E-6	0.77000	-7.7202E-10	NaN	2.1052E-4	1.5100E-9	5.3848E-19	5.3509E-17	0.20530	9.3774E-20	1.8580E-20	2.1962E-6	1.0000
1.5100E-	8.0000E-	0.79	-7.71	NaN	2.1049E-	1.5100E-	5.1085E-	5.3503E-	0.20	9.3761E-	1.8577E-	2.1959E-	1.00

k0Sub (m/s)	d (m)	Time (s)	-(tds2.ntflux_Oxi)*n*F (A), UME	Surface ConcOxi (M), Oxi Mem	Surface ConcOxi (M), OxiNuc	Substrate Kinetics (m/s), k0Sub probe	Normal total flux (mol/s), UME OCR membrane	Normal total flux (mol/s), UME OCR nucleus	Concentration (mol/m^3), Point: (5.5109E-16, 2.5)	k0Sub*Oxi (mol/s), Mem Flux	k0Sub*Oxi (mol/s), Nuc Flux	Normal total flux (mol/(m^2*s)), Point: (5.5109E-16, 2.5)	WElectrode radius (µm), UME Radius
9	6	000	91E-10		4	9	19	17	527	20	20	6	00
1.5100E-9	8.0000E-6	0.81000	-7.7181E-10	NaN	2.1047E-4	1.5100E-9	4.8575E-19	5.3498E-17	0.20524	9.3749E-20	1.8575E-20	2.1956E-6	1.0000
1.5100E-9	8.0000E-6	0.83000	-7.7172E-10	NaN	2.1044E-4	1.5100E-9	4.6387E-19	5.3493E-17	0.20522	9.3738E-20	1.8573E-20	2.1953E-6	1.0000
1.5100E-9	8.0000E-6	0.85000	-7.7163E-10	NaN	2.1042E-4	1.5100E-9	4.4362E-19	5.3488E-17	0.20519	9.3728E-20	1.8570E-20	2.1951E-6	1.0000
1.5100E-9	8.0000E-6	0.87000	-7.7155E-10	NaN	2.1040E-4	1.5100E-9	4.2499E-19	5.3483E-17	0.20517	9.3717E-20	1.8568E-20	2.1948E-6	1.0000
1.5100E-9	8.0000E-6	0.89000	-7.7147E-10	NaN	2.1037E-4	1.5100E-9	4.0799E-19	5.3478E-17	0.20515	9.3708E-20	1.8566E-20	2.1946E-6	1.0000
1.5100E-9	8.0000E-6	0.91000	-7.7139E-10	NaN	2.1035E-4	1.5100E-9	3.9159E-19	5.3474E-17	0.20513	9.3698E-20	1.8565E-20	2.1944E-6	1.0000
1.5100E-9	8.0000E-6	0.93000	-7.7131E-10	NaN	2.1033E-4	1.5100E-9	3.7574E-19	5.3470E-17	0.20511	9.3689E-20	1.8563E-20	2.1941E-6	1.0000
1.5100E-9	8.0000E-6	0.95000	-7.7124E-10	NaN	2.1031E-4	1.5100E-9	3.6095E-19	5.3465E-17	0.20509	9.3680E-20	1.8561E-20	2.1939E-6	1.0000
1.5100E-9	8.0000E-6	0.97000	-7.7117E-10	NaN	2.1029E-4	1.5100E-9	3.4720E-19	5.3461E-17	0.20507	9.3671E-20	1.8559E-20	2.1937E-6	1.0000

k0Sub (m/s)	d (m)	Time (s)	-(tds2.ntflux_Oxi)*n*F (A), UME	Surface ConcOxi (M), OxiMem	Surface ConcOxi (M), OxiNuc	Substrate Kinetics (m/s), k0Sub probe	Normal total flux (mol/s), UME OCR membrane	Normal total flux (mol/s), UME OCR nucleus	Concentration (mol/m^3), Point: (5.5109E-16, 2.5)	k0Sub*Oxi (mol/s), Mem Flux	k0Sub*Oxi (mol/s), Nuc Flux	Normal total flux (mol/(m^2*s)), Point: (5.5109E-16, 2.5)	WElectrode radius (µm), UME Radius
1.5100E-9	8.0000E-6	0.99000	-7.7110E-10	NaN	2.1027E-4	1.5100E-9	3.3450E-19	5.3457E-17	0.20505	9.3663E-20	1.8558E-20	2.1935E-6	1.0000
1.5100E-9	1.2000E-5	0.0000	-7.1532E-9	NaN	2.2700E-4	1.5100E-9	-2.0266E-20	-1.3324E-20	0.22700	1.0088E-19	2.0266E-20	-2.3241E-10	1.0000
1.5100E-9	1.2000E-5	0.0050000	-8.5576E-10	NaN	2.2694E-4	1.5100E-9	1.4940E-17	1.5820E-18	0.22681	1.0086E-19	2.0259E-20	1.7201E-7	1.0000
1.5100E-9	1.2000E-5	0.010000	-8.3076E-10	NaN	2.2645E-4	1.5100E-9	5.4393E-17	8.1044E-18	0.22580	1.0065E-19	2.0201E-20	5.4010E-7	1.0000
1.5100E-9	1.2000E-5	0.015000	-8.2168E-10	NaN	2.2565E-4	1.5100E-9	6.4227E-17	1.2684E-17	0.22459	1.0031E-19	2.0112E-20	6.8589E-7	1.0000
1.5100E-9	1.2000E-5	0.020000	-8.1647E-10	NaN	2.2487E-4	1.5100E-9	6.0852E-17	1.5107E-17	0.22356	9.9980E-20	2.0031E-20	7.3801E-7	1.0000
1.5100E-9	1.2000E-5	0.025000	-8.1260E-10	NaN	2.2417E-4	1.5100E-9	5.4970E-17	1.6628E-17	0.22270	9.9677E-20	1.9959E-20	7.6285E-7	1.0000
1.5100E-9	1.2000E-5	0.030000	-8.1010E-10	NaN	2.2363E-4	1.5100E-9	4.9760E-17	1.7420E-17	0.22207	9.9442E-20	1.9906E-20	7.7113E-7	1.0000
1.5100E-9	1.2000E-5	0.035000	-8.0781E-10	NaN	2.2311E-4	1.5100E-9	4.4592E-17	1.8101E-17	0.22147	9.9217E-20	1.9855E-20	7.7690E-7	1.0000
1.5100E-9	1.2000E-5	0.040000	-8.0557E-	NaN	2.2261E-4	1.5100E-9	3.9579E-17	1.8754E-17	0.22089	9.8997E-20	1.9805E-20	7.8223E-7	1.0000

k0Sub (m/s)	d (m)	Time (s)	-(tds2.ntflux_Oxi)*n*F (A), UME	Surface ConcOxi (M), Oxi Mem	Surface ConcOxi (M), OxiNuc	Substrate Kinetics (m/s), k0Sub probe	Normal total flux (mol/s), UME OCR membrane	Normal total flux (mol/s), UME OCR nucleus	Concentration (mol/m^3), Point: (5.5109E-16, 2.5)	k0Sub*Oxi (mol/s), Mem Flux	k0Sub*Oxi (mol/s), Nuc Flux	Normal total flux (mol/(m^2*s)), Point: (5.5109E-16, 2.5)	WElectrode radius (µm), UME Radius
			10										
1.5100E-9	1.2000E-5	0.045000	-8.0406E-10	NaN	2.2222E-4	1.5100E-9	3.5857E-17	1.9087E-17	0.22046	9.8829E-20	1.9769E-20	7.8253E-7	1.0000
1.5100E-9	1.2000E-5	0.050000	-8.0255E-10	NaN	2.2184E-4	1.5100E-9	3.2134E-17	1.9420E-17	0.22004	9.8662E-20	1.9732E-20	7.8284E-7	1.0000
1.5100E-9	1.2000E-5	0.055000	-8.0122E-10	NaN	2.2150E-4	1.5100E-9	2.8959E-17	1.9694E-17	0.21966	9.8512E-20	1.9699E-20	7.8276E-7	1.0000
1.5100E-9	1.2000E-5	0.060000	-8.0033E-10	NaN	2.2126E-4	1.5100E-9	2.6967E-17	1.9824E-17	0.21940	9.8406E-20	1.9676E-20	7.8174E-7	1.0000
1.5100E-9	1.2000E-5	0.065000	-7.9944E-10	NaN	2.2101E-4	1.5100E-9	2.4976E-17	1.9954E-17	0.21914	9.8300E-20	1.9653E-20	7.8073E-7	1.0000
1.5100E-9	1.2000E-5	0.070000	-7.9855E-10	NaN	2.2077E-4	1.5100E-9	2.2984E-17	2.0084E-17	0.21888	9.8194E-20	1.9631E-20	7.7971E-7	1.0000
1.5100E-9	1.2000E-5	0.075000	-7.9766E-10	NaN	2.2053E-4	1.5100E-9	2.0992E-17	2.0214E-17	0.21862	9.8088E-20	1.9608E-20	7.7870E-7	1.0000
1.5100E-9	1.2000E-5	0.080000	-7.9677E-10	NaN	2.2029E-4	1.5100E-9	1.9000E-17	2.0343E-17	0.21836	9.7982E-20	1.9585E-20	7.7769E-7	1.0000
1.5100E-9	1.2000E-5	0.085000	-7.9611E-10	NaN	2.2011E-4	1.5100E-9	1.7672E-17	2.0421E-17	0.21816	9.7901E-20	1.9568E-20	7.7674E-7	1.0000
1.5100E-	1.2000E-	0.09	-7.95	NaN	2.1994E-	1.5100E-	1.6573E-	2.0478E-	0.21	9.7829E-	1.9553E-	7.7582E-	1.00

k0Sub (m/s)	d (m)	Time (s)	-(tds2.ntflux_Oxi)*n*F (A), UME	Surface ConcOxi (M), OxiMem	Surface ConcOxi (M), OxiNuc	Substrate Kinetics (m/s), k0Sub probe	Normal total flux (mol/s), UME OCR membrane	Normal total flux (mol/s), UME OCR nucleus	Concentration (mol/m^3), Point: (5.5109E-16, 2.5)	k0Sub*Oxi (mol/s), Mem Flux	k0Sub*Oxi (mol/s), Nuc Flux	Normal total flux (mol/(m^2*s)), Point: (5.5109E-16, 2.5)	WElectrode radius (μm), UME Radius
9	5	0000	53E-10		4	9	17	17	799	20	20	7	00
1.5100E-9	1.2000E-5	0.095000	-7.9495E-10	NaN	2.1978E-4	1.5100E-9	1.5475E-17	2.0535E-17	0.21782	9.7758E-20	1.9538E-20	7.7490E-7	1.0000
1.5100E-9	1.2000E-5	0.10000	-7.9437E-10	NaN	2.1962E-4	1.5100E-9	1.4377E-17	2.0592E-17	0.21765	9.7687E-20	1.9523E-20	7.7397E-7	1.0000
1.5100E-9	1.2000E-5	0.10500	-7.9379E-10	NaN	2.1946E-4	1.5100E-9	1.3278E-17	2.0649E-17	0.21748	9.7616E-20	1.9508E-20	7.7305E-7	1.0000
1.5100E-9	1.2000E-5	0.11000	-7.9325E-10	NaN	2.1931E-4	1.5100E-9	1.2287E-17	2.0701E-17	0.21732	9.7549E-20	1.9494E-20	7.7219E-7	1.0000
1.5100E-9	1.2000E-5	0.11500	-7.9291E-10	NaN	2.1922E-4	1.5100E-9	1.1809E-17	2.0720E-17	0.21723	9.7508E-20	1.9485E-20	7.7165E-7	1.0000
1.5100E-9	1.2000E-5	0.12000	-7.9258E-10	NaN	2.1912E-4	1.5100E-9	1.1331E-17	2.0740E-17	0.21713	9.7467E-20	1.9477E-20	7.7111E-7	1.0000
1.5100E-9	1.2000E-5	0.12500	-7.9225E-10	NaN	2.1903E-4	1.5100E-9	1.0852E-17	2.0759E-17	0.21704	9.7426E-20	1.9468E-20	7.7057E-7	1.0000
1.5100E-9	1.2000E-5	0.13000	-7.9192E-10	NaN	2.1894E-4	1.5100E-9	1.0374E-17	2.0779E-17	0.21694	9.7385E-20	1.9460E-20	7.7003E-7	1.0000
1.5100E-9	1.2000E-5	0.13500	-7.9159E-10	NaN	2.1884E-4	1.5100E-9	9.8957E-18	2.0798E-17	0.21684	9.7344E-20	1.9451E-20	7.6949E-7	1.0000

k0Sub (m/s)	d (m)	Time (s)	-(tds2.ntflux_Oxi)*n*F (A), UME	Surface ConcOxi (M), OxiMem	Surface ConcOxi (M), OxiNuc	Substrate Kinetics (m/s), k0Sub probe	Normal total flux (mol/s), UME OCR membrane	Normal total flux (mol/s), UME OCR nucleus	Concentration (mol/m^3), Point: (5.5109E-16, 2.5)	k0Sub* Oxi (mol/s), Mem Flux	k0Sub* Oxi (mol/s), Nuc Flux	Normal total flux (mol/(m^2*s)), Point: (5.5109E-16, 2.5)	WElectrode radius (µm), UME Radius
1.5100E-9	1.2000E-5	0.14000	-7.9126E-10	NaN	2.1875E-4	1.5100E-9	9.4174E-18	2.0818E-17	0.21675	9.7303E-20	1.9443E-20	7.6895E-7	1.0000
1.5100E-9	1.2000E-5	0.14500	-7.9093E-10	NaN	2.1866E-4	1.5100E-9	8.9392E-18	2.0838E-17	0.21665	9.7261E-20	1.9434E-20	7.6841E-7	1.0000
1.5100E-9	1.2000E-5	0.15000	-7.9060E-10	NaN	2.1857E-4	1.5100E-9	8.4609E-18	2.0857E-17	0.21656	9.7220E-20	1.9426E-20	7.6787E-7	1.0000
1.5100E-9	1.2000E-5	0.15500	-7.9027E-10	NaN	2.1847E-4	1.5100E-9	7.9826E-18	2.0877E-17	0.21646	9.7179E-20	1.9417E-20	7.6733E-7	1.0000
1.5100E-9	1.2000E-5	0.16000	-7.8994E-10	NaN	2.1838E-4	1.5100E-9	7.5043E-18	2.0896E-17	0.21636	9.7138E-20	1.9409E-20	7.6679E-7	1.0000
1.5100E-9	1.2000E-5	0.16500	-7.8961E-10	NaN	2.1829E-4	1.5100E-9	7.0260E-18	2.0916E-17	0.21627	9.7097E-20	1.9400E-20	7.6625E-7	1.0000
1.5100E-9	1.2000E-5	0.17000	-7.8940E-10	NaN	2.1823E-4	1.5100E-9	6.8021E-18	2.0923E-17	0.21621	9.7071E-20	1.9395E-20	7.6593E-7	1.0000
1.5100E-9	1.2000E-5	0.17500	-7.8919E-10	NaN	2.1817E-4	1.5100E-9	6.5710E-18	2.0931E-17	0.21615	9.7045E-20	1.9389E-20	7.6560E-7	1.0000
1.5100E-9	1.2000E-5	0.18000	-7.8898E-10	NaN	2.1811E-4	1.5100E-9	6.3398E-18	2.0938E-17	0.21609	9.7019E-20	1.9384E-20	7.6528E-7	1.0000
1.5100E-9	1.2000E-5	0.18500	-7.8877E-	NaN	2.1805E-4	1.5100E-9	6.1087E-18	2.0945E-17	0.21603	9.6993E-20	1.9379E-20	7.6496E-7	1.0000

k0Sub (m/s)	d (m)	Time (s)	-(tds2.ntflux_Oxi)*n*F (A), UME	Surface ConcOxi (M), OxiMem	Surface ConcOxi (M), OxiNuc	Substrate Kinetics (m/s), k0Sub probe	Normal total flux (mol/s), UME OCR membrane	Normal total flux (mol/s), UME OCR nucleus	Concentration (mol/m^3), Point: (5.5109E-16, 2.5)	k0Sub*Oxi (mol/s), Mem Flux	k0Sub*Oxi (mol/s), Nuc Flux	Normal total flux (mol/(m^2*s)), Point: (5.5109E-16, 2.5)	WElectrode radius (μm), UME Radius
			10										
1.5100E-9	1.2000E-5	0.19000	-7.8856E-10	NaN	2.1799E-4	1.5100E-9	5.8775E-18	2.0953E-17	0.21597	9.6967E-20	1.9373E-20	7.6463E-7	1.0000
1.5100E-9	1.2000E-5	0.19500	-7.8835E-10	NaN	2.1793E-4	1.5100E-9	5.6464E-18	2.0960E-17	0.21591	9.6941E-20	1.9368E-20	7.6431E-7	1.0000
1.5100E-9	1.2000E-5	0.20000	-7.8814E-10	NaN	2.1787E-4	1.5100E-9	5.4152E-18	2.0967E-17	0.21585	9.6915E-20	1.9363E-20	7.6399E-7	1.0000
1.5100E-9	1.2000E-5	0.20500	-7.8793E-10	NaN	2.1782E-4	1.5100E-9	5.1841E-18	2.0975E-17	0.21578	9.6889E-20	1.9357E-20	7.6366E-7	1.0000
1.5100E-9	1.2000E-5	0.21000	-7.8772E-10	NaN	2.1776E-4	1.5100E-9	4.9530E-18	2.0982E-17	0.21572	9.6863E-20	1.9352E-20	7.6334E-7	1.0000
1.5100E-9	1.2000E-5	0.21500	-7.8751E-10	NaN	2.1770E-4	1.5100E-9	4.7218E-18	2.0989E-17	0.21566	9.6837E-20	1.9347E-20	7.6301E-7	1.0000
1.5100E-9	1.2000E-5	0.22000	-7.8731E-10	NaN	2.1764E-4	1.5100E-9	4.4907E-18	2.0996E-17	0.21560	9.6811E-20	1.9341E-20	7.6269E-7	1.0000
1.5100E-9	1.2000E-5	0.22500	-7.8717E-10	NaN	2.1760E-4	1.5100E-9	4.3792E-18	2.0999E-17	0.21557	9.6794E-20	1.9338E-20	7.6249E-7	1.0000
1.5100E-9	1.2000E-5	0.23000	-7.8705E-10	NaN	2.1757E-4	1.5100E-9	4.2824E-18	2.1001E-17	0.21553	9.6779E-20	1.9335E-20	7.6232E-7	1.0000
1.5100E-9	1.2000E-	0.23	-7.86	NaN	2.1753E-	1.5100E-	4.1855E-	2.1003E-	0.21	9.6764E-	1.9332E-	7.6215E-	1.00

k0Sub (m/s)	d (m)	Time (s)	-(tds2.ntflux_Oxi)*n*F (A), UME	Surface ConcOxi (M), OxiMem	Surface ConcOxi (M), OxiNuc	Substrate Kinetics (m/s), k0Sub probe	Normal total flux (mol/s), UME OCR membrane	Normal total flux (mol/s), UME OCR nucleus	Concentration (mol/m^3), Point: (5.5109E-16, 2.5)	k0Sub*Oxi (mol/s), Mem Flux	k0Sub*Oxi (mol/s), Nuc Flux	Normal total flux (mol/(m^2*s)), Point: (5.5109E-16, 2.5)	WElectrode radius (µm), UME Radius
9	5	500	93E-10		4	9	18	17	550	20	20	7	00
1.5100E-9	1.2000E-5	0.24000	-7.8681E-10	NaN	2.1750E-4	1.5100E-9	4.0886E-18	2.1005E-17	0.21546	9.6749E-20	1.9329E-20	7.6198E-7	1.0000
1.5100E-9	1.2000E-5	0.24500	-7.8669E-10	NaN	2.1747E-4	1.5100E-9	3.9918E-18	2.1007E-17	0.21543	9.6734E-20	1.9325E-20	7.6181E-7	1.0000
1.5100E-9	1.2000E-5	0.25000	-7.8657E-10	NaN	2.1743E-4	1.5100E-9	3.8949E-18	2.1009E-17	0.21539	9.6719E-20	1.9322E-20	7.6164E-7	1.0000
1.5100E-9	1.2000E-5	0.25000	-7.8657E-10	NaN	2.1743E-4	1.5100E-9	3.8949E-18	2.1009E-17	0.21539	9.6719E-20	1.9322E-20	7.6164E-7	1.0000
1.5100E-9	1.2000E-5	0.27000	-7.8608E-10	NaN	2.1730E-4	1.5100E-9	3.5075E-18	2.1017E-17	0.21526	9.6659E-20	1.9310E-20	7.6095E-7	1.0000
1.5100E-9	1.2000E-5	0.29000	-7.8560E-10	NaN	2.1716E-4	1.5100E-9	3.1200E-18	2.1024E-17	0.21512	9.6598E-20	1.9298E-20	7.6027E-7	1.0000
1.5100E-9	1.2000E-5	0.31000	-7.8511E-10	NaN	2.1702E-4	1.5100E-9	2.7325E-18	2.1032E-17	0.21498	9.6538E-20	1.9286E-20	7.5958E-7	1.0000
1.5100E-9	1.2000E-5	0.33000	-7.8472E-10	NaN	2.1691E-4	1.5100E-9	2.4518E-18	2.1037E-17	0.21487	9.6489E-20	1.9276E-20	7.5904E-7	1.0000
1.5100E-9	1.2000E-5	0.35000	-7.8441E-10	NaN	2.1683E-4	1.5100E-9	2.2666E-18	2.1038E-17	0.21478	9.6451E-20	1.9268E-20	7.5863E-7	1.0000

k0Sub (m/s)	d (m)	Time (s)	-(tds2.ntflux_Oxi)*n*F (A), UME	Surface ConcOxi (M), OxiMem	Surface ConcOxi (M), OxiNuc	Substrate Kinetics (m/s), k0Sub probe	Normal total flux (mol/s), UME OCR membrane	Normal total flux (mol/s), UME OCR nucleus	Concentration (mol/m^3), Point: (5.5109E-16, 2.5)	k0Sub*Oxi (mol/s), Mem Flux	k0Sub*Oxi (mol/s), Nuc Flux	Normal total flux (mol/(m^2*s)), Point: (5.5109E-16, 2.5)	WElectrode radius (µm), UME Radius
1.5100E-9	1.2000E-5	0.37000	-7.8410E-10	NaN	2.1674E-4	1.5100E-9	2.0815E-18	2.1039E-17	0.21469	9.6412E-20	1.9260E-20	7.5822E-7	1.0000
1.5100E-9	1.2000E-5	0.39000	-7.8379E-10	NaN	2.1665E-4	1.5100E-9	1.8964E-18	2.1041E-17	0.21461	9.6374E-20	1.9252E-20	7.5781E-7	1.0000
1.5100E-9	1.2000E-5	0.41000	-7.8348E-10	NaN	2.1657E-4	1.5100E-9	1.7112E-18	2.1042E-17	0.21452	9.6335E-20	1.9244E-20	7.5740E-7	1.0000
1.5100E-9	1.2000E-5	0.43000	-7.8322E-10	NaN	2.1649E-4	1.5100E-9	1.5754E-18	2.1042E-17	0.21445	9.6303E-20	1.9238E-20	7.5707E-7	1.0000
1.5100E-9	1.2000E-5	0.45000	-7.8300E-10	NaN	2.1643E-4	1.5100E-9	1.4748E-18	2.1041E-17	0.21439	9.6276E-20	1.9232E-20	7.5680E-7	1.0000
1.5100E-9	1.2000E-5	0.47000	-7.8278E-10	NaN	2.1637E-4	1.5100E-9	1.3743E-18	2.1041E-17	0.21433	9.6249E-20	1.9227E-20	7.5653E-7	1.0000
1.5100E-9	1.2000E-5	0.49000	-7.8257E-10	NaN	2.1631E-4	1.5100E-9	1.2737E-18	2.1040E-17	0.21426	9.6223E-20	1.9222E-20	7.5626E-7	1.0000
1.5100E-9	1.2000E-5	0.51000	-7.8235E-10	NaN	2.1625E-4	1.5100E-9	1.1732E-18	2.1039E-17	0.21420	9.6196E-20	1.9216E-20	7.5599E-7	1.0000
1.5100E-9	1.2000E-5	0.53000	-7.8217E-10	NaN	2.1620E-4	1.5100E-9	1.0984E-18	2.1038E-17	0.21415	9.6173E-20	1.9212E-20	7.5577E-7	1.0000
1.5100E-9	1.2000E-5	0.55000	-7.8201E-	NaN	2.1616E-4	1.5100E-9	1.0382E-18	2.1037E-17	0.21411	9.6153E-20	1.9208E-20	7.5558E-7	1.0000

k0Sub (m/s)	d (m)	Time (s)	-(tds2.ntflux_Oxi)*n*F (A), UME	Surface ConcOxi (M), OxiMem	Surface ConcOxi (M), OxiNuc	Substrate Kinetics (m/s), k0Sub probe	Normal total flux (mol/s), UME OCR membrane	Normal total flux (mol/s), UME OCR nucleus	Concentration (mol/m^3), Point: (5.5109E-16, 2.5)	k0Sub*Oxi (mol/s), Mem Flux	k0Sub*Oxi (mol/s), Nuc Flux	Normal total flux (mol/(m^2*s)), Point: (5.5109E-16, 2.5)	WElectrode radius (µm), UME Radius
			10										
1.5100E-9	1.2000E-5	0.57000	-7.8184E-10	NaN	2.1611E-4	1.5100E-9	9.7797E-19	2.1036E-17	0.21406	9.6133E-20	1.9204E-20	7.5539E-7	1.0000
1.5100E-9	1.2000E-5	0.59000	-7.8168E-10	NaN	2.1607E-4	1.5100E-9	9.1775E-19	2.1034E-17	0.21402	9.6113E-20	1.9200E-20	7.5520E-7	1.0000
1.5100E-9	1.2000E-5	0.61000	-7.8152E-10	NaN	2.1602E-4	1.5100E-9	8.5754E-19	2.1033E-17	0.21397	9.6093E-20	1.9196E-20	7.5500E-7	1.0000
1.5100E-9	1.2000E-5	0.63000	-7.8138E-10	NaN	2.1598E-4	1.5100E-9	8.1083E-19	2.1032E-17	0.21393	9.6076E-20	1.9192E-20	7.5484E-7	1.0000
1.5100E-9	1.2000E-5	0.65000	-7.8125E-10	NaN	2.1595E-4	1.5100E-9	7.7005E-19	2.1030E-17	0.21390	9.6060E-20	1.9189E-20	7.5469E-7	1.0000
1.5100E-9	1.2000E-5	0.67000	-7.8113E-10	NaN	2.1591E-4	1.5100E-9	7.2926E-19	2.1029E-17	0.21386	9.6044E-20	1.9186E-20	7.5454E-7	1.0000
1.5100E-9	1.2000E-5	0.69000	-7.8100E-10	NaN	2.1588E-4	1.5100E-9	6.8847E-19	2.1028E-17	0.21383	9.6029E-20	1.9182E-20	7.5439E-7	1.0000
1.5100E-9	1.2000E-5	0.71000	-7.8087E-10	NaN	2.1584E-4	1.5100E-9	6.4768E-19	2.1026E-17	0.21379	9.6013E-20	1.9179E-20	7.5425E-7	1.0000
1.5100E-9	1.2000E-5	0.73000	-7.8076E-10	NaN	2.1581E-4	1.5100E-9	6.1715E-19	2.1025E-17	0.21376	9.5999E-20	1.9177E-20	7.5412E-7	1.0000
1.5100E-	1.2000E-	0.75	-7.80	NaN	2.1578E-	1.5100E-	5.9002E-	2.1023E-	0.21	9.5986E-	1.9174E-	7.5401E-	1.00

k0Sub (m/s)	d (m)	Time (s)	-(tds2.ntflux_Oxi)*n*F (A), UME	Surface ConcOxi (M), OxiMem	Surface ConcOxi (M), OxiNuc	Substrate Kinetics (m/s), k0Sub probe	Normal total flux (mol/s), UME OCR membrane	Normal total flux (mol/s), UME OCR nucleus	Concentration (mol/m^3), Point: (5.5109E-16, 2.5)	k0Sub*Oxi (mol/s), Mem Flux	k0Sub*Oxi (mol/s), Nuc Flux	Normal total flux (mol/(m^2*s)), Point: (5.5109E-16, 2.5)	WEIectrode radius (μm), UME Radius
9	5	000	66E-10		4	9	19	17	373	20	20	7	00
1.5100E-9	1.2000E-5	0.77000	-7.8056E-10	NaN	2.1575E-4	1.5100E-9	5.6289E-19	2.1022E-17	0.21370	9.5974E-20	1.9172E-20	7.5389E-7	1.0000
1.5100E-9	1.2000E-5	0.79000	-7.8045E-10	NaN	2.1572E-4	1.5100E-9	5.3576E-19	2.1021E-17	0.21368	9.5961E-20	1.9169E-20	7.5378E-7	1.0000
1.5100E-9	1.2000E-5	0.81000	-7.8035E-10	NaN	2.1570E-4	1.5100E-9	5.0864E-19	2.1019E-17	0.21365	9.5949E-20	1.9166E-20	7.5366E-7	1.0000
1.5100E-9	1.2000E-5	0.83000	-7.8026E-10	NaN	2.1567E-4	1.5100E-9	4.8508E-19	2.1018E-17	0.21362	9.5937E-20	1.9164E-20	7.5355E-7	1.0000
1.5100E-9	1.2000E-5	0.85000	-7.8017E-10	NaN	2.1565E-4	1.5100E-9	4.6379E-19	2.1017E-17	0.21360	9.5926E-20	1.9162E-20	7.5345E-7	1.0000
1.5100E-9	1.2000E-5	0.87000	-7.8008E-10	NaN	2.1562E-4	1.5100E-9	4.4403E-19	2.1015E-17	0.21357	9.5915E-20	1.9160E-20	7.5336E-7	1.0000
1.5100E-9	1.2000E-5	0.89000	-7.8000E-10	NaN	2.1560E-4	1.5100E-9	4.2582E-19	2.1014E-17	0.21355	9.5905E-20	1.9158E-20	7.5327E-7	1.0000
1.5100E-9	1.2000E-5	0.91000	-7.7992E-10	NaN	2.1558E-4	1.5100E-9	4.0915E-19	2.1013E-17	0.21353	9.5895E-20	1.9156E-20	7.5318E-7	1.0000
1.5100E-9	1.2000E-5	0.93000	-7.7984E-10	NaN	2.1555E-4	1.5100E-9	3.9277E-19	2.1011E-17	0.21351	9.5886E-20	1.9154E-20	7.5309E-7	1.0000

k0Sub (m/s)	d (m)	Time (s)	-(tds2.ntflux_Oxi)*n*F (A), UME	Surface ConcOxi (M), OxiMem	Surface ConcOxi (M), OxiNuc	Substrate Kinetics (m/s), k0Sub probe	Normal total flux (mol/s), UME OCR membrane	Normal total flux (mol/s), UME OCR nucleus	Concentration (mol/m^3), Point: (5.5109E-16, 2.5)	k0Sub*Oxi (mol/s), Mem Flux	k0Sub*Oxi (mol/s), Nuc Flux	Normal total flux (mol/(m^2*s)), Point: (5.5109E-16, 2.5)	WElectrode radius (µm), UME Radius
1.5100E-9	1.2000E-5	0.95000	-7.7976E-10	NaN	2.1553E-4	1.5100E-9	3.7720E-19	2.1010E-17	0.21349	9.5876E-20	1.9152E-20	7.5301E-7	1.0000
1.5100E-9	1.2000E-5	0.97000	-7.7969E-10	NaN	2.1551E-4	1.5100E-9	3.6265E-19	2.1009E-17	0.21347	9.5867E-20	1.9150E-20	7.5293E-7	1.0000
1.5100E-9	1.2000E-5	0.99000	-7.7962E-10	NaN	2.1549E-4	1.5100E-9	3.4913E-19	2.1008E-17	0.21345	9.5859E-20	1.9148E-20	7.5286E-7	1.0000
1.5100E-9	1.5000E-5	0.0000	-7.1540E-9	NaN	2.2700E-4	1.5100E-9	-2.0266E-20	-1.3335E-20	0.22700	1.0088E-19	2.0266E-20	-2.3285E-10	1.0000
1.5100E-9	1.5000E-5	0.000500000	-8.5579E-10	NaN	2.2699E-4	1.5100E-9	2.1974E-18	1.8483E-19	0.22698	1.0088E-19	2.0266E-20	2.3230E-8	1.0000
1.5100E-9	1.5000E-5	0.010000	-8.3079E-10	NaN	2.2685E-4	1.5100E-9	1.8436E-17	2.2112E-18	0.22667	1.0082E-19	2.0249E-20	1.6266E-7	1.0000
1.5100E-9	1.5000E-5	0.015000	-8.2172E-10	NaN	2.2647E-4	1.5100E-9	3.1294E-17	4.7909E-18	0.22609	1.0066E-19	2.0208E-20	2.7824E-7	1.0000
1.5100E-9	1.5000E-5	0.020000	-8.1660E-10	NaN	2.2602E-4	1.5100E-9	3.5263E-17	6.5733E-18	0.22547	1.0046E-19	2.0160E-20	3.3860E-7	1.0000
1.5100E-9	1.5000E-5	0.025000	-8.1294E-10	NaN	2.2556E-4	1.5100E-9	3.5219E-17	7.7809E-18	0.22490	1.0026E-19	2.0114E-20	3.7193E-7	1.0000
1.5100E-9	1.5000E-5	0.030000	-8.1058E-	NaN	2.2517E-4	1.5100E-9	3.3495E-17	8.4725E-18	0.22443	1.0009E-19	2.0075E-20	3.8691E-7	1.0000

k0Sub (m/s)	d (m)	Time (s)	-(tds2.ntflux_Oxi)*n*F (A), UME	Surface ConcOxi (M), OxiMem	Surface ConcOxi (M), OxiNuc	Substrate Kinetics (m/s), k0Sub probe	Normal total flux (mol/s), UME OCR membrane	Normal total flux (mol/s), UME OCR nucleus	Concentration (mol/m^3), Point: (5.5109E-16, 2.5)	k0Sub*Oxi (mol/s), Mem Flux	k0Sub*Oxi (mol/s), Nuc Flux	Normal total flux (mol/(m^2*s)), Point: (5.5109E-16, 2.5)	WElectrode radius (µm), UME Radius
			10										
1.5100E-9	1.5000E-5	0.035000	-8.0850E-10	NaN	2.2479E-4	1.5100E-9	3.1404E-17	9.0599E-18	0.22399	9.9928E-20	2.0038E-20	3.9819E-7	1.0000
1.5100E-9	1.5000E-5	0.040000	-8.0643E-10	NaN	2.2441E-4	1.5100E-9	2.9335E-17	9.6405E-18	0.22355	9.9763E-20	2.0001E-20	4.0928E-7	1.0000
1.5100E-9	1.5000E-5	0.045000	-8.0510E-10	NaN	2.2410E-4	1.5100E-9	2.7205E-17	9.9426E-18	0.22321	9.9630E-20	1.9972E-20	4.1270E-7	1.0000
1.5100E-9	1.5000E-5	0.050000	-8.0377E-10	NaN	2.2380E-4	1.5100E-9	2.5074E-17	1.0245E-17	0.22287	9.9497E-20	1.9943E-20	4.1612E-7	1.0000
1.5100E-9	1.5000E-5	0.055000	-8.0256E-10	NaN	2.2352E-4	1.5100E-9	2.3133E-17	1.0508E-17	0.22256	9.9373E-20	1.9916E-20	4.1885E-7	1.0000
1.5100E-9	1.5000E-5	0.060000	-8.0180E-10	NaN	2.2331E-4	1.5100E-9	2.1747E-17	1.0630E-17	0.22234	9.9283E-20	1.9896E-20	4.1908E-7	1.0000
1.5100E-9	1.5000E-5	0.065000	-8.0103E-10	NaN	2.2311E-4	1.5100E-9	2.0361E-17	1.0751E-17	0.22212	9.9193E-20	1.9877E-20	4.1932E-7	1.0000
1.5100E-9	1.5000E-5	0.070000	-8.0027E-10	NaN	2.2290E-4	1.5100E-9	1.8974E-17	1.0873E-17	0.22190	9.9103E-20	1.9858E-20	4.1956E-7	1.0000
1.5100E-9	1.5000E-5	0.075000	-7.9950E-10	NaN	2.2270E-4	1.5100E-9	1.7588E-17	1.0995E-17	0.22168	9.9013E-20	1.9839E-20	4.1979E-7	1.0000
1.5100E-	1.5000E-	0.08	-7.98	NaN	2.2249E-	1.5100E-	1.6201E-	1.1117E-	0.22	9.8923E-	1.9820E-	4.2003E-	1.00

k0Sub (m/s)	d (m)	Time (s)	-(tds2.ntflux_Oxi)*n*F (A), UME	Surface ConcOxi (M), Oxi Mem	Surface ConcOxi (M), OxiNuc	Substrate Kinetics (m/s), k0Sub probe	Normal total flux (mol/s), UME OCR membrane	Normal total flux (mol/s), UME OCR nucleus	Concentration (mol/m^3), Point: (5.5109E-16, 2.5)	k0Sub* Oxi (mol/s), Mem Flux	k0Sub* Oxi (mol/s), Nuc Flux	Normal total flux (mol/(m^2*s)), Point: (5.5109E-16, 2.5)	WElectrode radius (µm), UME Radius
9	5	0000	74E-10		4	9	17	17	145	20	20	7	00
1.5100E-9	1.5000E-5	0.085000	-7.9813E-10	NaN	2.2233E-4	1.5100E-9	1.5156E-17	1.1198E-17	0.22128	9.8850E-20	1.9804E-20	4.1990E-7	1.0000
1.5100E-9	1.5000E-5	0.090000	-7.9763E-10	NaN	2.2218E-4	1.5100E-9	1.4308E-17	1.1253E-17	0.22113	9.8787E-20	1.9791E-20	4.1953E-7	1.0000
1.5100E-9	1.5000E-5	0.095000	-7.9713E-10	NaN	2.2204E-4	1.5100E-9	1.3460E-17	1.1308E-17	0.22098	9.8724E-20	1.9777E-20	4.1916E-7	1.0000
1.5100E-9	1.5000E-5	0.10000	-7.9663E-10	NaN	2.2190E-4	1.5100E-9	1.2611E-17	1.1363E-17	0.22083	9.8661E-20	1.9764E-20	4.1879E-7	1.0000
1.5100E-9	1.5000E-5	0.10500	-7.9613E-10	NaN	2.2176E-4	1.5100E-9	1.1763E-17	1.1417E-17	0.22068	9.8598E-20	1.9751E-20	4.1842E-7	1.0000
1.5100E-9	1.5000E-5	0.11000	-7.9563E-10	NaN	2.2161E-4	1.5100E-9	1.0915E-17	1.1472E-17	0.22053	9.8536E-20	1.9738E-20	4.1805E-7	1.0000
1.5100E-9	1.5000E-5	0.11500	-7.9533E-10	NaN	2.2153E-4	1.5100E-9	1.0527E-17	1.1493E-17	0.22044	9.8497E-20	1.9730E-20	4.1773E-7	1.0000
1.5100E-9	1.5000E-5	0.12000	-7.9504E-10	NaN	2.2144E-4	1.5100E-9	1.0130E-17	1.1513E-17	0.22035	9.8460E-20	1.9722E-20	4.1740E-7	1.0000
1.5100E-9	1.5000E-5	0.12500	-7.9475E-10	NaN	2.2136E-4	1.5100E-9	9.7322E-18	1.1533E-17	0.22026	9.8422E-20	1.9714E-20	4.1708E-7	1.0000

k0Sub (m/s)	d (m)	Time (s)	-(tds2.ntflux_Oxi)*n*F (A), UME	Surface ConcOxi (M), OxiMem	Surface ConcOxi (M), OxiNuc	Substrate Kinetics (m/s), k0Sub probe	Normal total flux (mol/s), UME OCR membrane	Normal total flux (mol/s), UME OCR nucleus	Concentration (mol/m^3), Point: (5.5109E-16, 2.5)	k0Sub*Oxi (mol/s), Mem Flux	k0Sub*Oxi (mol/s), Nuc Flux	Normal total flux (mol/(m^2*s)), Point: (5.5109E-16, 2.5)	WElectrode radius (µm), UME Radius
1.5100E-9	1.5000E-5	0.13000	-7.9446E-10	NaN	2.2127E-4	1.5100E-9	9.3345E-18	1.1552E-17	0.22017	9.8384E-20	1.9706E-20	4.1676E-7	1.0000
1.5100E-9	1.5000E-5	0.13500	-7.9416E-10	NaN	2.2119E-4	1.5100E-9	8.9369E-18	1.1572E-17	0.22008	9.8347E-20	1.9699E-20	4.1643E-7	1.0000
1.5100E-9	1.5000E-5	0.14000	-7.9387E-10	NaN	2.2110E-4	1.5100E-9	8.5393E-18	1.1592E-17	0.21999	9.8309E-20	1.9691E-20	4.1611E-7	1.0000
1.5100E-9	1.5000E-5	0.14500	-7.9358E-10	NaN	2.2102E-4	1.5100E-9	8.1417E-18	1.1612E-17	0.21991	9.8271E-20	1.9683E-20	4.1579E-7	1.0000
1.5100E-9	1.5000E-5	0.15000	-7.9329E-10	NaN	2.2093E-4	1.5100E-9	7.7441E-18	1.1632E-17	0.21982	9.8234E-20	1.9675E-20	4.1546E-7	1.0000
1.5100E-9	1.5000E-5	0.15500	-7.9299E-10	NaN	2.2085E-4	1.5100E-9	7.3465E-18	1.1652E-17	0.21973	9.8196E-20	1.9667E-20	4.1514E-7	1.0000
1.5100E-9	1.5000E-5	0.16000	-7.9270E-10	NaN	2.2076E-4	1.5100E-9	6.9489E-18	1.1672E-17	0.21964	9.8158E-20	1.9659E-20	4.1481E-7	1.0000
1.5100E-9	1.5000E-5	0.16500	-7.9241E-10	NaN	2.2068E-4	1.5100E-9	6.5513E-18	1.1692E-17	0.21955	9.8121E-20	1.9652E-20	4.1449E-7	1.0000
1.5100E-9	1.5000E-5	0.17000	-7.9219E-10	NaN	2.2061E-4	1.5100E-9	6.3101E-18	1.1703E-17	0.21949	9.8093E-20	1.9646E-20	4.1425E-7	1.0000
1.5100E-9	1.5000E-5	0.17500	-7.9200E-	NaN	2.2056E-4	1.5100E-9	6.1084E-18	1.1711E-17	0.21943	9.8068E-20	1.9641E-20	4.1403E-7	1.0000

k0Sub (m/s)	d (m)	Time (s)	-(tds2.ntflux_Oxi)*n*F (A), UME	Surface ConcOxi (M), OxiMem	Surface ConcOxi (M), OxiNuc	Substrate Kinetics (m/s), k0Sub probe	Normal total flux (mol/s), UME OCR membrane	Normal total flux (mol/s), UME OCR nucleus	Concentration (mol/m^3), Point: (5.5109E-16, 2.5)	k0Sub*Oxi (mol/s), Mem Flux	k0Sub*Oxi (mol/s), Nuc Flux	Normal total flux (mol/(m^2*s)), Point: (5.5109E-16, 2.5)	WElectrode radius (µm), UME Radius
			10										
1.5100E-9	1.5000E-5	0.18000	-7.9181E-10	NaN	2.2050E-4	1.5100E-9	5.9068E-18	1.1719E-17	0.21937	9.8044E-20	1.9636E-20	4.1382E-7	1.0000
1.5100E-9	1.5000E-5	0.18500	-7.9162E-10	NaN	2.2045E-4	1.5100E-9	5.7051E-18	1.1728E-17	0.21932	9.8019E-20	1.9631E-20	4.1361E-7	1.0000
1.5100E-9	1.5000E-5	0.19000	-7.9143E-10	NaN	2.2039E-4	1.5100E-9	5.5035E-18	1.1736E-17	0.21926	9.7995E-20	1.9626E-20	4.1339E-7	1.0000
1.5100E-9	1.5000E-5	0.19500	-7.9124E-10	NaN	2.2034E-4	1.5100E-9	5.3018E-18	1.1744E-17	0.21920	9.7970E-20	1.9621E-20	4.1318E-7	1.0000
1.5100E-9	1.5000E-5	0.20000	-7.9105E-10	NaN	2.2028E-4	1.5100E-9	5.1001E-18	1.1752E-17	0.21915	9.7946E-20	1.9616E-20	4.1296E-7	1.0000
1.5100E-9	1.5000E-5	0.20500	-7.9086E-10	NaN	2.2022E-4	1.5100E-9	4.8985E-18	1.1761E-17	0.21909	9.7921E-20	1.9611E-20	4.1275E-7	1.0000
1.5100E-9	1.5000E-5	0.21000	-7.9067E-10	NaN	2.2017E-4	1.5100E-9	4.6968E-18	1.1769E-17	0.21903	9.7897E-20	1.9606E-20	4.1253E-7	1.0000
1.5100E-9	1.5000E-5	0.21500	-7.9048E-10	NaN	2.2011E-4	1.5100E-9	4.4951E-18	1.1777E-17	0.21898	9.7872E-20	1.9601E-20	4.1232E-7	1.0000
1.5100E-9	1.5000E-5	0.22000	-7.9029E-10	NaN	2.2006E-4	1.5100E-9	4.2935E-18	1.1785E-17	0.21892	9.7848E-20	1.9596E-20	4.1210E-7	1.0000
1.5100E-	1.5000E-	0.22	-7.90	NaN	2.2002E-	1.5100E-	4.1578E-	1.1791E-	0.21	9.7829E-	1.9592E-	4.1194E-	1.00

k0Sub (m/s)	d (m)	Time (s)	-(tds2.ntflux_Oxi)*n*F (A), UME	Surface ConcOxi (M), OxiMem	Surface ConcOxi (M), OxiNuc	Substrate Kinetics (m/s), k0Sub probe	Normal total flux (mol/s), UME OCR membrane	Normal total flux (mol/s), UME OCR nucleus	Concentration (mol/m^3), Point: (5.5109E-16, 2.5)	k0Sub*Oxi (mol/s), MemFlux	k0Sub*Oxi (mol/s), NucFlux	Normal total flux (mol/(m^2*s)), Point: (5.5109E-16, 2.5)	WEIectrode radius (μm), UME Radius
9	5	500	14E-10		4	9	18	17	888	20	20	7	00
1.5100E-9	1.5000E-5	0.23000	-7.9003E-10	NaN	2.1998E-4	1.5100E-9	4.0696E-18	1.1793E-17	0.21884	9.7814E-20	1.9589E-20	4.1182E-7	1.0000
1.5100E-9	1.5000E-5	0.23500	-7.8992E-10	NaN	2.1995E-4	1.5100E-9	3.9815E-18	1.1796E-17	0.21881	9.7799E-20	1.9586E-20	4.1171E-7	1.0000
1.5100E-9	1.5000E-5	0.24000	-7.8981E-10	NaN	2.1992E-4	1.5100E-9	3.8933E-18	1.1799E-17	0.21878	9.7785E-20	1.9583E-20	4.1159E-7	1.0000
1.5100E-9	1.5000E-5	0.24500	-7.8969E-10	NaN	2.1988E-4	1.5100E-9	3.8052E-18	1.1802E-17	0.21874	9.7770E-20	1.9580E-20	4.1148E-7	1.0000
1.5100E-9	1.5000E-5	0.25000	-7.8958E-10	NaN	2.1985E-4	1.5100E-9	3.7170E-18	1.1805E-17	0.21871	9.7756E-20	1.9577E-20	4.1136E-7	1.0000
1.5100E-9	1.5000E-5	0.25000	-7.8958E-10	NaN	2.1985E-4	1.5100E-9	3.7170E-18	1.1805E-17	0.21871	9.7756E-20	1.9577E-20	4.1136E-7	1.0000
1.5100E-9	1.5000E-5	0.27000	-7.8913E-10	NaN	2.1972E-4	1.5100E-9	3.3644E-18	1.1816E-17	0.21858	9.7698E-20	1.9565E-20	4.1089E-7	1.0000
1.5100E-9	1.5000E-5	0.29000	-7.8867E-10	NaN	2.1959E-4	1.5100E-9	3.0118E-18	1.1827E-17	0.21844	9.7640E-20	1.9553E-20	4.1043E-7	1.0000
1.5100E-9	1.5000E-5	0.31000	-7.8822E-10	NaN	2.1946E-4	1.5100E-9	2.6591E-18	1.1839E-17	0.21831	9.7582E-20	1.9541E-20	4.0997E-7	1.0000

k0Sub (m/s)	d (m)	Time (s)	-(tds2.ntflux_Oxi)*n*F (A), UME	Surface ConcOxi (M), OxiMem	Surface ConcOxi (M), OxiNuc	Substrate Kinetics (m/s), k0Sub probe	Normal total flux (mol/s), UME OCR membrane	Normal total flux (mol/s), UME OCR nucleus	Concentration (mol/m^3), Point: (5.5109E-16, 2.5)	k0Sub*Oxi (mol/s), Mem Flux	k0Sub*Oxi (mol/s), Nuc Flux	Normal total flux (mol/(m^2*s)), Point: (5.5109E-16, 2.5)	WElectrode radius (µm), UME Radius
1.5100E-9	1.5000E-5	0.33000	-7.8784E-10	NaN	2.1935E-4	1.5100E-9	2.3869E-18	1.1847E-17	0.21820	9.7532E-20	1.9531E-20	4.0958E-7	1.0000
1.5100E-9	1.5000E-5	0.35000	-7.8754E-10	NaN	2.1926E-4	1.5100E-9	2.2130E-18	1.1851E-17	0.21811	9.7495E-20	1.9523E-20	4.0931E-7	1.0000
1.5100E-9	1.5000E-5	0.37000	-7.8724E-10	NaN	2.1918E-4	1.5100E-9	2.0390E-18	1.1855E-17	0.21803	9.7457E-20	1.9516E-20	4.0903E-7	1.0000
1.5100E-9	1.5000E-5	0.39000	-7.8694E-10	NaN	2.1909E-4	1.5100E-9	1.8651E-18	1.1859E-17	0.21794	9.7419E-20	1.9508E-20	4.0876E-7	1.0000
1.5100E-9	1.5000E-5	0.41000	-7.8665E-10	NaN	2.1901E-4	1.5100E-9	1.6912E-18	1.1863E-17	0.21785	9.7381E-20	1.9500E-20	4.0849E-7	1.0000
1.5100E-9	1.5000E-5	0.43000	-7.8639E-10	NaN	2.1893E-4	1.5100E-9	1.5564E-18	1.1866E-17	0.21778	9.7349E-20	1.9494E-20	4.0826E-7	1.0000
1.5100E-9	1.5000E-5	0.45000	-7.8618E-10	NaN	2.1887E-4	1.5100E-9	1.4598E-18	1.1868E-17	0.21772	9.7322E-20	1.9488E-20	4.0808E-7	1.0000
1.5100E-9	1.5000E-5	0.47000	-7.8597E-10	NaN	2.1881E-4	1.5100E-9	1.3633E-18	1.1869E-17	0.21766	9.7295E-20	1.9483E-20	4.0790E-7	1.0000
1.5100E-9	1.5000E-5	0.49000	-7.8576E-10	NaN	2.1875E-4	1.5100E-9	1.2668E-18	1.1870E-17	0.21760	9.7269E-20	1.9478E-20	4.0772E-7	1.0000
1.5100E-9	1.5000E-5	0.51000	-7.8555E-	NaN	2.1869E-4	1.5100E-9	1.1703E-18	1.1872E-17	0.21754	9.7242E-20	1.9472E-20	4.0755E-7	1.0000

k0Sub (m/s)	d (m)	Time (s)	-(tds2.ntflux_Oxi)*n*F (A), UME	Surface ConcOxi (M), OxiMem	Surface ConcOxi (M), OxiNuc	Substrate Kinetics (m/s), k0Sub probe	Normal total flux (mol/s), UME OCR membrane	Normal total flux (mol/s), UME OCR nucleus	Concentration (mol/m^3), Point: (5.5109E-16, 2.5)	k0Sub*Oxi (mol/s), Mem Flux	k0Sub*Oxi (mol/s), Nuc Flux	Normal total flux (mol/(m^2*s)), Point: (5.5109E-16, 2.5)	WElectrode radius (µm), UME Radius
			10										
1.5100E-9	1.5000E-5	0.53000	-7.8537E-10	NaN	2.1864E-4	1.5100E-9	1.0950E-18	1.1873E-17	0.21749	9.7219E-20	1.9468E-20	4.0740E-7	1.0000
1.5100E-9	1.5000E-5	0.55000	-7.8521E-10	NaN	2.1860E-4	1.5100E-9	1.0363E-18	1.1873E-17	0.21744	9.7199E-20	1.9464E-20	4.0727E-7	1.0000
1.5100E-9	1.5000E-5	0.57000	-7.8505E-10	NaN	2.1855E-4	1.5100E-9	9.7766E-19	1.1874E-17	0.21740	9.7179E-20	1.9460E-20	4.0715E-7	1.0000
1.5100E-9	1.5000E-5	0.59000	-7.8490E-10	NaN	2.1851E-4	1.5100E-9	9.1900E-19	1.1874E-17	0.21735	9.7159E-20	1.9456E-20	4.0702E-7	1.0000
1.5100E-9	1.5000E-5	0.61000	-7.8474E-10	NaN	2.1846E-4	1.5100E-9	8.6033E-19	1.1874E-17	0.21731	9.7140E-20	1.9452E-20	4.0690E-7	1.0000
1.5100E-9	1.5000E-5	0.63000	-7.8460E-10	NaN	2.1842E-4	1.5100E-9	8.1315E-19	1.1875E-17	0.21727	9.7122E-20	1.9448E-20	4.0679E-7	1.0000
1.5100E-9	1.5000E-5	0.65000	-7.8447E-10	NaN	2.1839E-4	1.5100E-9	7.7306E-19	1.1875E-17	0.21723	9.7106E-20	1.9445E-20	4.0670E-7	1.0000
1.5100E-9	1.5000E-5	0.67000	-7.8435E-10	NaN	2.1835E-4	1.5100E-9	7.3297E-19	1.1875E-17	0.21719	9.7090E-20	1.9442E-20	4.0660E-7	1.0000
1.5100E-9	1.5000E-5	0.69000	-7.8422E-10	NaN	2.1832E-4	1.5100E-9	6.9288E-19	1.1874E-17	0.21716	9.7074E-20	1.9438E-20	4.0651E-7	1.0000
1.5100E-	1.5000E-	0.71	-7.84	NaN	2.1828E-	1.5100E-	6.5279E-	1.1874E-	0.21	9.7058E-	1.9435E-	4.0641E-	1.00

k0Sub (m/s)	d (m)	Time (s)	-(tds2.ntflux_Oxi)*n*F (A), UME	Surface ConcOxi (M), Oxi Mem	Surface ConcOxi (M), OxiNuc	Substrate Kinetics (m/s), k0Sub probe	Normal total flux (mol/s), UME OCR membrane	Normal total flux (mol/s), UME OCR nucleus	Concentration (mol/m^3), Point: (5.5109E-16, 2.5)	k0Sub*Oxi (mol/s), Mem Flux	k0Sub*Oxi (mol/s), Nuc Flux	Normal total flux (mol/(m^2*s)), Point: (5.5109E-16, 2.5)	WElectrode radius (μm), UME Radius
9	5	000	10E-10		4	9	19	17	712	20	20	7	00
1.5100E-9	1.5000E-5	0.73000	-7.8399E-10	NaN	2.1825E-4	1.5100E-9	6.2158E-19	1.1874E-17	0.21709	9.7045E-20	1.9432E-20	4.0633E-7	1.0000
1.5100E-9	1.5000E-5	0.75000	-7.8388E-10	NaN	2.1822E-4	1.5100E-9	5.9468E-19	1.1874E-17	0.21706	9.7032E-20	1.9430E-20	4.0626E-7	1.0000
1.5100E-9	1.5000E-5	0.77000	-7.8378E-10	NaN	2.1819E-4	1.5100E-9	5.6779E-19	1.1874E-17	0.21703	9.7019E-20	1.9427E-20	4.0619E-7	1.0000
1.5100E-9	1.5000E-5	0.79000	-7.8368E-10	NaN	2.1816E-4	1.5100E-9	5.4089E-19	1.1873E-17	0.21701	9.7007E-20	1.9425E-20	4.0611E-7	1.0000
1.5100E-9	1.5000E-5	0.81000	-7.8358E-10	NaN	2.1813E-4	1.5100E-9	5.1399E-19	1.1873E-17	0.21698	9.6994E-20	1.9422E-20	4.0604E-7	1.0000
1.5100E-9	1.5000E-5	0.83000	-7.8349E-10	NaN	2.1811E-4	1.5100E-9	4.9020E-19	1.1873E-17	0.21695	9.6982E-20	1.9420E-20	4.0597E-7	1.0000
1.5100E-9	1.5000E-5	0.85000	-7.8340E-10	NaN	2.1808E-4	1.5100E-9	4.6886E-19	1.1872E-17	0.21692	9.6971E-20	1.9417E-20	4.0591E-7	1.0000
1.5100E-9	1.5000E-5	0.87000	-7.8331E-10	NaN	2.1806E-4	1.5100E-9	4.4903E-19	1.1872E-17	0.21690	9.6960E-20	1.9415E-20	4.0585E-7	1.0000
1.5100E-9	1.5000E-5	0.89000	-7.8323E-10	NaN	2.1803E-4	1.5100E-9	4.3069E-19	1.1872E-17	0.21688	9.6950E-20	1.9413E-20	4.0580E-7	1.0000

k0Sub (m/s)	d (m)	Time (s)	-(tds2.ntflux_Oxi)*n*F (A), UME	Surface ConcOxi (M), Oxi Mem	Surface ConcOxi (M), OxiNuc	Substrate Kinetics (m/s), k0Sub probe	Normal total flux (mol/s), UME OCR membrane	Normal total flux (mol/s), UME OCR nucleus	Concentration (mol/m^3), Point: (5.5109E-16, 2.5)	k0Sub*Oxi (mol/s), Mem Flux	k0Sub*Oxi (mol/s), Nuc Flux	Normal total flux (mol/(m^2*s)), Point: (5.5109E-16, 2.5)	WEIectrode radius (µm), UME Radius
1.5100E-9	1.5000E-5	0.91000	-7.8315E-10	NaN	2.1801E-4	1.5100E-9	4.1386E-19	1.1871E-17	0.21685	9.6940E-20	1.9411E-20	4.0574E-7	1.0000
1.5100E-9	1.5000E-5	0.93000	-7.8307E-10	NaN	2.1799E-4	1.5100E-9	3.9747E-19	1.1871E-17	0.21683	9.6930E-20	1.9409E-20	4.0569E-7	1.0000
1.5100E-9	1.5000E-5	0.95000	-7.8300E-10	NaN	2.1797E-4	1.5100E-9	3.8180E-19	1.1870E-17	0.21681	9.6921E-20	1.9407E-20	4.0564E-7	1.0000
1.5100E-9	1.5000E-5	0.97000	-7.8292E-10	NaN	2.1795E-4	1.5100E-9	3.6715E-19	1.1870E-17	0.21679	9.6911E-20	1.9406E-20	4.0559E-7	1.0000
1.5100E-9	1.5000E-5	0.99000	-7.8285E-10	NaN	2.1793E-4	1.5100E-9	3.5350E-19	1.1870E-17	0.21677	9.6903E-20	1.9404E-20	4.0554E-7	1.0000
1.5100E-8	5.0000E-7	0.0000	-7.1490E-9	NaN	2.2699E-4	1.5100E-8	3.3451E-18	-1.1291E-19	0.22696	1.0088E-18	2.0266E-19	9.5015E-7	1.0000
1.5100E-8	5.0000E-7	0.0050000	-7.4816E-10	NaN	2.0656E-4	1.5100E-8	8.6885E-16	7.0669E-16	0.062310	9.3682E-19	1.6560E-19	2.2780E-4	1.0000
1.5100E-8	5.0000E-7	0.010000	-7.0194E-10	NaN	1.9857E-4	1.5100E-8	4.5875E-16	7.0434E-16	0.058400	9.0325E-19	1.5651E-19	2.1348E-4	1.0000
1.5100E-8	5.0000E-7	0.015000	-6.7907E-10	NaN	1.9390E-4	1.5100E-8	2.7166E-16	6.9532E-16	0.056478	8.8302E-19	1.5179E-19	2.0645E-4	1.0000
1.5100E-8	5.0000E-7	0.020000	-6.6595E-	NaN	1.9100E-4	1.5100E-8	1.7623E-16	6.8817E-16	0.055378	8.7034E-19	1.4902E-19	2.0242E-4	1.0000

k0Sub (m/s)	d (m)	Time (s)	-(tds2.ntflux_Oxi)*n*F (A), UME	Surface ConcOxi (M), OxiMem	Surface ConcOxi (M), OxiNuc	Substrate Kinetics (m/s), k0Sub probe	Normal total flux (mol/s), UME OCR membrane	Normal total flux (mol/s), UME OCR nucleus	Concentration (mol/m^3), Point: (5.5109E-16, 2.5)	k0Sub*Oxi (mol/s), Mem Flux	k0Sub*Oxi (mol/s), Nuc Flux	Normal total flux (mol/(m^2*s)), Point: (5.5109E-16, 2.5)	WElectrode radius (µm), UME Radius
			10										
1.5100E-8	5.0000E-7	0.025000	-6.5727E-10	NaN	1.8904E-4	1.5100E-8	1.1861E-16	6.8315E-16	0.054651	8.6171E-19	1.4718E-19	1.9977E-4	1.0000
1.5100E-8	5.0000E-7	0.030000	-6.5121E-10	NaN	1.8765E-4	1.5100E-8	8.1265E-17	6.7950E-16	0.054144	8.5557E-19	1.4590E-19	1.9791E-4	1.0000
1.5100E-8	5.0000E-7	0.035000	-6.4705E-10	NaN	1.8666E-4	1.5100E-8	5.8185E-17	6.7677E-16	0.053796	8.5121E-19	1.4501E-19	1.9663E-4	1.0000
1.5100E-8	5.0000E-7	0.040000	-6.4374E-10	NaN	1.8587E-4	1.5100E-8	4.0703E-17	6.7453E-16	0.053519	8.4769E-19	1.4430E-19	1.9562E-4	1.0000
1.5100E-8	5.0000E-7	0.045000	-6.4121E-10	NaN	1.8526E-4	1.5100E-8	2.8435E-17	6.7273E-16	0.053308	8.4495E-19	1.4376E-19	1.9485E-4	1.0000
1.5100E-8	5.0000E-7	0.050000	-6.3965E-10	NaN	1.8486E-4	1.5100E-8	2.2467E-17	6.7149E-16	0.053177	8.4317E-19	1.4342E-19	1.9437E-4	1.0000
1.5100E-8	5.0000E-7	0.055000	-6.3879E-10	NaN	1.8463E-4	1.5100E-8	2.0396E-17	6.7071E-16	0.053106	8.4213E-19	1.4323E-19	1.9411E-4	1.0000
1.5100E-8	5.0000E-7	0.060000	-6.3793E-10	NaN	1.8440E-4	1.5100E-8	1.8326E-17	6.6993E-16	0.053034	8.4108E-19	1.4304E-19	1.9385E-4	1.0000
1.5100E-8	5.0000E-7	0.065000	-6.3718E-10	NaN	1.8420E-4	1.5100E-8	1.6589E-17	6.6925E-16	0.052972	8.4018E-19	1.4287E-19	1.9362E-4	1.0000
1.5100E-	5.0000E-	0.07	-6.36	NaN	1.8402E-	1.5100E-	1.5108E-	6.6866E-	0.05	8.3939E-	1.4273E-	1.9343E-	1.00

k0Sub (m/s)	d (m)	Time (s)	-(tds2.ntflux_Oxi)*n*F (A), UME	Surface ConcOxi (M), OxiMem	Surface ConcOxi (M), OxiNuc	Substrate Kinetics (m/s), k0Sub probe	Normal total flux (mol/s), UME OCR membrane	Normal total flux (mol/s), UME OCR nucleus	Concentration (mol/m^3), Point: (5.5109E-16, 2.5)	k0Sub*Oxi (mol/s), Mem Flux	k0Sub*Oxi (mol/s), Nuc Flux	Normal total flux (mol/(m^2*s)), Point: (5.5109E-16, 2.5)	WElectrode radius (μm), UME Radius
8	7	0000	54E-10		4	8	17	16	2918	19	19	4	00
1.5100E-8	5.0000E-7	0.075000	-6.3589E-10	NaN	1.8385E-4	1.5100E-8	1.3627E-17	6.6807E-16	0.052865	8.3860E-19	1.4259E-19	1.9323E-4	1.0000
1.5100E-8	5.0000E-7	0.080000	-6.3544E-10	NaN	1.8373E-4	1.5100E-8	1.2681E-17	6.6765E-16	0.052826	8.3804E-19	1.4249E-19	1.9309E-4	1.0000
1.5100E-8	5.0000E-7	0.085000	-6.3501E-10	NaN	1.8361E-4	1.5100E-8	1.1807E-17	6.6726E-16	0.052791	8.3753E-19	1.4239E-19	1.9296E-4	1.0000
1.5100E-8	5.0000E-7	0.090000	-6.3459E-10	NaN	1.8350E-4	1.5100E-8	1.0933E-17	6.6687E-16	0.052756	8.3701E-19	1.4230E-19	1.9283E-4	1.0000
1.5100E-8	5.0000E-7	0.095000	-6.3417E-10	NaN	1.8338E-4	1.5100E-8	1.0059E-17	6.6648E-16	0.052721	8.3649E-19	1.4221E-19	1.9271E-4	1.0000
1.5100E-8	5.0000E-7	0.10000	-6.3375E-10	NaN	1.8327E-4	1.5100E-8	9.1848E-18	6.6609E-16	0.052686	8.3597E-19	1.4211E-19	1.9258E-4	1.0000
1.5100E-8	5.0000E-7	0.10500	-6.3342E-10	NaN	1.8318E-4	1.5100E-8	8.5669E-18	6.6578E-16	0.052658	8.3556E-19	1.4204E-19	1.9248E-4	1.0000
1.5100E-8	5.0000E-7	0.11000	-6.3319E-10	NaN	1.8311E-4	1.5100E-8	8.1924E-18	6.6556E-16	0.052639	8.3527E-19	1.4199E-19	1.9241E-4	1.0000
1.5100E-8	5.0000E-7	0.11500	-6.3295E-10	NaN	1.8305E-4	1.5100E-8	7.8180E-18	6.6534E-16	0.052620	8.3499E-19	1.4194E-19	1.9233E-4	1.0000

k0Sub (m/s)	d (m)	Time (s)	-(tds2.ntflux_Oxi)*n*F (A), UME	Surface ConcOxi (M), OxiMem	Surface ConcOxi (M), OxiNuc	Substrate Kinetics (m/s), k0Sub probe	Normal total flux (mol/s), UME OCR membrane	Normal total flux (mol/s), UME OCR nucleus	Concentration (mol/m^3), Point: (5.5109E-16, 2.5)	k0Sub*Oxi (mol/s), Mem Flux	k0Sub*Oxi (mol/s), Nuc Flux	Normal total flux (mol/(m^2*s)), Point: (5.5109E-16, 2.5)	WEIectrode radius (µm), UME Radius
1.5100E-8	5.0000E-7	0.12000	-6.3272E-10	NaN	1.8299E-4	1.5100E-8	7.4435E-18	6.6512E-16	0.052600	8.3470E-19	1.4189E-19	1.9226E-4	1.0000
1.5100E-8	5.0000E-7	0.12500	-6.3249E-10	NaN	1.8292E-4	1.5100E-8	7.0691E-18	6.6490E-16	0.052581	8.3441E-19	1.4183E-19	1.9219E-4	1.0000
1.5100E-8	5.0000E-7	0.13000	-6.3226E-10	NaN	1.8286E-4	1.5100E-8	6.6946E-18	6.6468E-16	0.052562	8.3412E-19	1.4178E-19	1.9212E-4	1.0000
1.5100E-8	5.0000E-7	0.13500	-6.3203E-10	NaN	1.8280E-4	1.5100E-8	6.3202E-18	6.6446E-16	0.052543	8.3383E-19	1.4173E-19	1.9205E-4	1.0000
1.5100E-8	5.0000E-7	0.14000	-6.3180E-10	NaN	1.8273E-4	1.5100E-8	5.9458E-18	6.6424E-16	0.052524	8.3354E-19	1.4168E-19	1.9198E-4	1.0000
1.5100E-8	5.0000E-7	0.14500	-6.3157E-10	NaN	1.8267E-4	1.5100E-8	5.5713E-18	6.6402E-16	0.052504	8.3326E-19	1.4163E-19	1.9191E-4	1.0000
1.5100E-8	5.0000E-7	0.15000	-6.3134E-10	NaN	1.8260E-4	1.5100E-8	5.1969E-18	6.6380E-16	0.052485	8.3297E-19	1.4158E-19	1.9184E-4	1.0000
1.5100E-8	5.0000E-7	0.15500	-6.3111E-10	NaN	1.8254E-4	1.5100E-8	4.8224E-18	6.6358E-16	0.052466	8.3268E-19	1.4153E-19	1.9177E-4	1.0000
1.5100E-8	5.0000E-7	0.16000	-6.3096E-10	NaN	1.8250E-4	1.5100E-8	4.6428E-18	6.6344E-16	0.052454	8.3250E-19	1.4150E-19	1.9173E-4	1.0000
1.5100E-8	5.0000E-7	0.16500	-6.3085E-	NaN	1.8247E-4	1.5100E-8	4.5168E-18	6.6333E-16	0.052444	8.3235E-19	1.4147E-19	1.9169E-4	1.0000

k0Sub (m/s)	d (m)	Time (s)	-(tds2.ntflux_Oxi)*n*F (A), UME	Surface ConcOxi (M), OxiMem	Surface ConcOxi (M), OxiNuc	Substrate Kinetics (m/s), k0Sub probe	Normal total flux (mol/s), UME OCR membrane	Normal total flux (mol/s), UME OCR nucleus	Concentration (mol/m^3), Point: (5.5109E-16, 2.5)	k0Sub*Oxi (mol/s), Mem Flux	k0Sub*Oxi (mol/s), Nuc Flux	Normal total flux (mol/(m^2*s)), Point: (5.5109E-16, 2.5)	WElectrode radius (µm), UME Radius
			10										
1.5100E-8	5.0000E-7	0.17000	-6.3074E-10	NaN	1.8244E-4	1.5100E-8	4.3909E-18	6.6322E-16	0.052435	8.3221E-19	1.4145E-19	1.9166E-4	1.0000
1.5100E-8	5.0000E-7	0.17500	-6.3062E-10	NaN	1.8241E-4	1.5100E-8	4.2649E-18	6.6311E-16	0.052426	8.3207E-19	1.4142E-19	1.9163E-4	1.0000
1.5100E-8	5.0000E-7	0.18000	-6.3051E-10	NaN	1.8237E-4	1.5100E-8	4.1389E-18	6.6300E-16	0.052416	8.3192E-19	1.4140E-19	1.9159E-4	1.0000
1.5100E-8	5.0000E-7	0.18500	-6.3040E-10	NaN	1.8234E-4	1.5100E-8	4.0130E-18	6.6289E-16	0.052407	8.3178E-19	1.4137E-19	1.9156E-4	1.0000
1.5100E-8	5.0000E-7	0.19000	-6.3028E-10	NaN	1.8231E-4	1.5100E-8	3.8870E-18	6.6277E-16	0.052397	8.3164E-19	1.4134E-19	1.9152E-4	1.0000
1.5100E-8	5.0000E-7	0.19500	-6.3017E-10	NaN	1.8228E-4	1.5100E-8	3.7610E-18	6.6266E-16	0.052388	8.3149E-19	1.4132E-19	1.9149E-4	1.0000
1.5100E-8	5.0000E-7	0.20000	-6.3006E-10	NaN	1.8225E-4	1.5100E-8	3.6351E-18	6.6255E-16	0.052379	8.3135E-19	1.4129E-19	1.9145E-4	1.0000
1.5100E-8	5.0000E-7	0.20500	-6.2995E-10	NaN	1.8222E-4	1.5100E-8	3.5091E-18	6.6244E-16	0.052369	8.3121E-19	1.4127E-19	1.9142E-4	1.0000
1.5100E-8	5.0000E-7	0.21000	-6.2983E-10	NaN	1.8218E-4	1.5100E-8	3.3831E-18	6.6233E-16	0.052360	8.3106E-19	1.4124E-19	1.9138E-4	1.0000
1.5100E-	5.0000E-	0.21	-6.29	NaN	1.8215E-	1.5100E-	3.2572E-	6.6222E-	0.05	8.3092E-	1.4122E-	1.9135E-	1.00

k0Sub (m/s)	d (m)	Time (s)	-(tds2.ntflux_Oxi)*n*F (A), UME	Surface ConcOxi (M), Oxi Mem	Surface ConcOxi (M), OxiNuc	Substrate Kinetics (m/s), k0Sub probe	Normal total flux (mol/s), UME OCR membrane	Normal total flux (mol/s), UME OCR nucleus	Concentration (mol/m^3), Point: (5.5109E-16, 2.5)	k0Sub*Oxi (mol/s), Mem Flux	k0Sub*Oxi (mol/s), Nuc Flux	Normal total flux (mol/(m^2*s)), Point: (5.5109E-16, 2.5)	WElectrode radius (μm), UME Radius
8	7	500	72E-10		4	8	18	16	2350	19	19	4	00
1.5100E-8	5.0000E-7	0.22000	-6.2961E-10	NaN	1.8212E-4	1.5100E-8	3.1312E-18	6.6211E-16	0.052341	8.3078E-19	1.4119E-19	1.9132E-4	1.0000
1.5100E-8	5.0000E-7	0.22500	-6.2949E-10	NaN	1.8209E-4	1.5100E-8	3.0052E-18	6.6200E-16	0.052332	8.3063E-19	1.4117E-19	1.9128E-4	1.0000
1.5100E-8	5.0000E-7	0.23000	-6.2938E-10	NaN	1.8206E-4	1.5100E-8	2.8792E-18	6.6189E-16	0.052322	8.3049E-19	1.4114E-19	1.9125E-4	1.0000
1.5100E-8	5.0000E-7	0.23500	-6.2927E-10	NaN	1.8203E-4	1.5100E-8	2.7533E-18	6.6178E-16	0.052313	8.3035E-19	1.4112E-19	1.9121E-4	1.0000
1.5100E-8	5.0000E-7	0.24000	-6.2915E-10	NaN	1.8200E-4	1.5100E-8	2.6273E-18	6.6166E-16	0.052303	8.3020E-19	1.4109E-19	1.9118E-4	1.0000
1.5100E-8	5.0000E-7	0.24500	-6.2904E-10	NaN	1.8196E-4	1.5100E-8	2.5013E-18	6.6155E-16	0.052294	8.3006E-19	1.4107E-19	1.9114E-4	1.0000
1.5100E-8	5.0000E-7	0.25000	-6.2893E-10	NaN	1.8193E-4	1.5100E-8	2.3754E-18	6.6144E-16	0.052285	8.2992E-19	1.4104E-19	1.9111E-4	1.0000
1.5100E-8	5.0000E-7	0.25000	-6.2893E-10	NaN	1.8193E-4	1.5100E-8	2.3754E-18	6.6144E-16	0.052285	8.2992E-19	1.4104E-19	1.9111E-4	1.0000
1.5100E-8	5.0000E-7	0.27000	-6.2862E-10	NaN	1.8184E-4	1.5100E-8	2.0998E-18	6.6113E-16	0.052259	8.2952E-19	1.4097E-19	1.9102E-4	1.0000

k0Sub (m/s)	d (m)	Time (s)	-(tds2.ntflux_Oxi)*n*F (A), UME	Surface ConcOxi (M), Oxi Mem	Surface ConcOxi (M), OxiNuc	Substrate Kinetics (m/s), k0Sub probe	Normal total flux (mol/s), UME OCR membrane	Normal total flux (mol/s), UME OCR nucleus	Concentration (mol/m^3), Point: (5.5109E-16, 2.5)	k0Sub* Oxi (mol/s), Mem Flux	k0Sub* Oxi (mol/s), Nuc Flux	Normal total flux (mol/(m^2*s)), Point: (5.5109E-16, 2.5)	WElectrode radius (µm), UME Radius
1.5100E-8	5.0000E-7	0.29000	-6.2836E-10	NaN	1.8177E-4	1.5100E-8	1.9001E-18	6.6087E-16	0.052237	8.2918E-19	1.4092E-19	1.9094E-4	1.0000
1.5100E-8	5.0000E-7	0.31000	-6.2810E-10	NaN	1.8170E-4	1.5100E-8	1.7005E-18	6.6061E-16	0.052216	8.2885E-19	1.4086E-19	1.9086E-4	1.0000
1.5100E-8	5.0000E-7	0.33000	-6.2784E-10	NaN	1.8162E-4	1.5100E-8	1.5008E-18	6.6035E-16	0.052194	8.2852E-19	1.4080E-19	1.9078E-4	1.0000
1.5100E-8	5.0000E-7	0.35000	-6.2758E-10	NaN	1.8155E-4	1.5100E-8	1.3012E-18	6.6009E-16	0.052172	8.2818E-19	1.4074E-19	1.9070E-4	1.0000
1.5100E-8	5.0000E-7	0.37000	-6.2739E-10	NaN	1.8150E-4	1.5100E-8	1.1852E-18	6.5990E-16	0.052157	8.2794E-19	1.4070E-19	1.9064E-4	1.0000
1.5100E-8	5.0000E-7	0.39000	-6.2722E-10	NaN	1.8145E-4	1.5100E-8	1.0892E-18	6.5973E-16	0.052143	8.2772E-19	1.4066E-19	1.9059E-4	1.0000
1.5100E-8	5.0000E-7	0.41000	-6.2705E-10	NaN	1.8140E-4	1.5100E-8	9.9317E-19	6.5955E-16	0.052129	8.2750E-19	1.4062E-19	1.9054E-4	1.0000
1.5100E-8	5.0000E-7	0.43000	-6.2688E-10	NaN	1.8135E-4	1.5100E-8	8.9717E-19	6.5938E-16	0.052114	8.2728E-19	1.4059E-19	1.9049E-4	1.0000
1.5100E-8	5.0000E-7	0.45000	-6.2671E-10	NaN	1.8130E-4	1.5100E-8	8.0116E-19	6.5921E-16	0.052100	8.2706E-19	1.4055E-19	1.9044E-4	1.0000
1.5100E-8	5.0000E-7	0.47000	-6.2658E-	NaN	1.8127E-4	1.5100E-8	7.4214E-19	6.5908E-16	0.052090	8.2689E-19	1.4052E-19	1.9040E-4	1.0000

k0Sub (m/s)	d (m)	Time (s)	-(tds2.ntflux_Oxi)*n*F (A), UME	Surface ConcOxi (M), OxiMem	Surface ConcOxi (M), OxiNuc	Substrate Kinetics (m/s), k0Sub probe	Normal total flux (mol/s), UME OCR membrane	Normal total flux (mol/s), UME OCR nucleus	Concentration (mol/m^3), Point: (5.5109E-16, 2.5)	k0Sub*Oxi (mol/s), Mem Flux	k0Sub*Oxi (mol/s), Nuc Flux	Normal total flux (mol/(m^2*s)), Point: (5.5109E-16, 2.5)	WElectrode radius (µm), UME Radius
			10										
1.5100E-8	5.0000E-7	0.49000	-6.2646E-10	NaN	1.8123E-4	1.5100E-8	6.8891E-19	6.5895E-16	0.052080	8.2674E-19	1.4049E-19	1.9036E-4	1.0000
1.5100E-8	5.0000E-7	0.51000	-6.2634E-10	NaN	1.8120E-4	1.5100E-8	6.3569E-19	6.5883E-16	0.052070	8.2658E-19	1.4047E-19	1.9032E-4	1.0000
1.5100E-8	5.0000E-7	0.53000	-6.2622E-10	NaN	1.8116E-4	1.5100E-8	5.8246E-19	6.5870E-16	0.052060	8.2642E-19	1.4044E-19	1.9029E-4	1.0000
1.5100E-8	5.0000E-7	0.55000	-6.2610E-10	NaN	1.8113E-4	1.5100E-8	5.2923E-19	6.5858E-16	0.052050	8.2627E-19	1.4041E-19	1.9025E-4	1.0000
1.5100E-8	5.0000E-7	0.57000	-6.2601E-10	NaN	1.8110E-4	1.5100E-8	4.9330E-19	6.5848E-16	0.052041	8.2614E-19	1.4039E-19	1.9022E-4	1.0000
1.5100E-8	5.0000E-7	0.59000	-6.2591E-10	NaN	1.8108E-4	1.5100E-8	4.5882E-19	6.5838E-16	0.052034	8.2602E-19	1.4037E-19	1.9019E-4	1.0000
1.5100E-8	5.0000E-7	0.61000	-6.2582E-10	NaN	1.8105E-4	1.5100E-8	4.2434E-19	6.5829E-16	0.052026	8.2590E-19	1.4035E-19	1.9016E-4	1.0000
1.5100E-8	5.0000E-7	0.63000	-6.2572E-10	NaN	1.8102E-4	1.5100E-8	3.8986E-19	6.5819E-16	0.052018	8.2577E-19	1.4033E-19	1.9014E-4	1.0000
1.5100E-8	5.0000E-7	0.65000	-6.2563E-10	NaN	1.8099E-4	1.5100E-8	3.5538E-19	6.5809E-16	0.052010	8.2565E-19	1.4031E-19	1.9011E-4	1.0000
1.5100E-	5.0000E-	0.67	-6.25	NaN	1.8097E-	1.5100E-	3.3320E-	6.5802E-	0.05	8.2555E-	1.4029E-	1.9008E-	1.00

k0Sub (m/s)	d (m)	Time (s)	-(tds2.ntflux_Oxi)*n*F (A), UME	Surface ConcOxi (M), Oxi Mem	Surface ConcOxi (M), OxiNuc	Substrate Kinetics (m/s), k0Sub probe	Normal total flux (mol/s), UME OCR membrane	Normal total flux (mol/s), UME OCR nucleus	Concentration (mol/m^3), Point: (5.5109E-16, 2.5)	k0Sub* Oxi (mol/s), Mem Flux	k0Sub* Oxi (mol/s), Nuc Flux	Normal total flux (mol/(m^2*s)), Point: (5.5109E-16, 2.5)	WElectrode radius (µm), UME Radius
8	7	000	56E-10		4	8	19	16	2004	19	19	4	00
1.5100E-8	5.0000E-7	0.69000	-6.2548E-10	NaN	1.8095E-4	1.5100E-8	3.1128E-19	6.5794E-16	0.051998	8.2546E-19	1.4027E-19	1.9006E-4	1.0000
1.5100E-8	5.0000E-7	0.71000	-6.2541E-10	NaN	1.8093E-4	1.5100E-8	2.8937E-19	6.5787E-16	0.051992	8.2536E-19	1.4026E-19	1.9004E-4	1.0000
1.5100E-8	5.0000E-7	0.73000	-6.2534E-10	NaN	1.8091E-4	1.5100E-8	2.6745E-19	6.5779E-16	0.051986	8.2527E-19	1.4024E-19	1.9002E-4	1.0000
1.5100E-8	5.0000E-7	0.75000	-6.2526E-10	NaN	1.8089E-4	1.5100E-8	2.4553E-19	6.5772E-16	0.051980	8.2517E-19	1.4023E-19	1.9000E-4	1.0000
1.5100E-8	5.0000E-7	0.77000	-6.2520E-10	NaN	1.8087E-4	1.5100E-8	2.2784E-19	6.5765E-16	0.051974	8.2509E-19	1.4021E-19	1.8998E-4	1.0000
1.5100E-8	5.0000E-7	0.79000	-6.2514E-10	NaN	1.8085E-4	1.5100E-8	2.1139E-19	6.5758E-16	0.051969	8.2501E-19	1.4020E-19	1.8996E-4	1.0000
1.5100E-8	5.0000E-7	0.81000	-6.2508E-10	NaN	1.8084E-4	1.5100E-8	1.9630E-19	6.5752E-16	0.051964	8.2493E-19	1.4018E-19	1.8994E-4	1.0000
1.5100E-8	5.0000E-7	0.83000	-6.2502E-10	NaN	1.8082E-4	1.5100E-8	1.8257E-19	6.5746E-16	0.051959	8.2485E-19	1.4017E-19	1.8992E-4	1.0000
1.5100E-8	5.0000E-7	0.85000	-6.2496E-10	NaN	1.8080E-4	1.5100E-8	1.7013E-19	6.5740E-16	0.051955	8.2478E-19	1.4016E-19	1.8990E-4	1.0000

k0Sub (m/s)	d (m)	Time (s)	-(tds2.ntflux_Oxi)*n*F (A), UME	Surface ConcOxi (M), OxiMem	Surface ConcOxi (M), OxiNuc	Substrate Kinetics (m/s), k0Sub probe	Normal total flux (mol/s), UME OCR membrane	Normal total flux (mol/s), UME OCR nucleus	Concentration (mol/m^3), Point: (5.5109E-16, 2.5)	k0Sub*Oxi (mol/s), Mem Flux	k0Sub*Oxi (mol/s), Nuc Flux	Normal total flux (mol/(m^2*s)), Point: (5.5109E-16, 2.5)	WElectrode radius (µm), UME Radius
1.5100E-8	5.0000E-7	0.87000	-6.2491E-10	NaN	1.8079E-4	1.5100E-8	1.5754E-19	6.5735E-16	0.051950	8.2471E-19	1.4015E-19	1.8989E-4	1.0000
1.5100E-8	5.0000E-7	0.89000	-6.2486E-10	NaN	1.8077E-4	1.5100E-8	1.4578E-19	6.5729E-16	0.051946	8.2464E-19	1.4013E-19	1.8987E-4	1.0000
1.5100E-8	5.0000E-7	0.91000	-6.2481E-10	NaN	1.8076E-4	1.5100E-8	1.3486E-19	6.5724E-16	0.051942	8.2457E-19	1.4012E-19	1.8986E-4	1.0000
1.5100E-8	5.0000E-7	0.93000	-6.2476E-10	NaN	1.8074E-4	1.5100E-8	1.2477E-19	6.5719E-16	0.051938	8.2451E-19	1.4011E-19	1.8984E-4	1.0000
1.5100E-8	5.0000E-7	0.95000	-6.2471E-10	NaN	1.8073E-4	1.5100E-8	1.1544E-19	6.5714E-16	0.051934	8.2445E-19	1.4010E-19	1.8983E-4	1.0000
1.5100E-8	5.0000E-7	0.97000	-6.2467E-10	NaN	1.8072E-4	1.5100E-8	1.0620E-19	6.5710E-16	0.051930	8.2439E-19	1.4009E-19	1.8981E-4	1.0000
1.5100E-8	5.0000E-7	0.99000	-6.2462E-10	NaN	1.8071E-4	1.5100E-8	9.7517E-20	6.5705E-16	0.051927	8.2433E-19	1.4008E-19	1.8980E-4	1.0000
1.5100E-8	1.0000E-6	0.0000	-7.1535E-9	NaN	2.2700E-4	1.5100E-8	-2.0259E-19	-1.1923E-19	0.22699	1.0088E-18	2.0266E-19	-1.3373E-9	1.0000
1.5100E-8	1.0000E-6	0.0050000	-7.9196E-10	NaN	2.1025E-4	1.5100E-8	7.9989E-16	5.6558E-16	0.11268	9.4918E-19	1.7288E-19	1.4395E-4	1.0000
1.5100E-8	1.0000E-6	0.010000	-7.4670E-	NaN	2.0277E-4	1.5100E-8	4.3904E-16	5.7391E-16	0.10612	9.1801E-19	1.6416E-19	1.3552E-4	1.0000

k0Sub (m/s)	d (m)	Time (s)	-(tds2.ntflux_Oxi)*n*F (A), UME	Surface ConcOxi (M), OxiMem	Surface ConcOxi (M), OxiNuc	Substrate Kinetics (m/s), k0Sub probe	Normal total flux (mol/s), UME OCR membrane	Normal total flux (mol/s), UME OCR nucleus	Concentration (mol/m^3), Point: (5.5109E-16, 2.5)	k0Sub*Oxi (mol/s), Mem Flux	k0Sub*Oxi (mol/s), Nuc Flux	Normal total flux (mol/(m^2*s)), Point: (5.5109E-16, 2.5)	WElectrode radius (µm), UME Radius
			10										
1.5100E-8	1.0000E-6	0.015000	-7.2416E-10	NaN	1.9830E-4	1.5100E-8	2.7163E-16	5.6969E-16	0.10288	8.9876E-19	1.5957E-19	1.3137E-4	1.0000
1.5100E-8	1.0000E-6	0.020000	-7.0999E-10	NaN	1.9536E-4	1.5100E-8	1.7711E-16	5.6590E-16	0.10084	8.8594E-19	1.5665E-19	1.2876E-4	1.0000
1.5100E-8	1.0000E-6	0.025000	-7.0044E-10	NaN	1.9330E-4	1.5100E-8	1.1855E-16	5.6278E-16	0.099476	8.7697E-19	1.5467E-19	1.2701E-4	1.0000
1.5100E-8	1.0000E-6	0.030000	-6.9380E-10	NaN	1.9183E-4	1.5100E-8	8.1365E-17	5.6024E-16	0.098526	8.7051E-19	1.5328E-19	1.2580E-4	1.0000
1.5100E-8	1.0000E-6	0.035000	-6.8940E-10	NaN	1.9082E-4	1.5100E-8	5.9945E-17	5.5821E-16	0.097896	8.6602E-19	1.5234E-19	1.2499E-4	1.0000
1.5100E-8	1.0000E-6	0.040000	-6.8625E-10	NaN	1.9007E-4	1.5100E-8	4.6419E-17	5.5661E-16	0.097448	8.6271E-19	1.5168E-19	1.2442E-4	1.0000
1.5100E-8	1.0000E-6	0.045000	-6.8385E-10	NaN	1.8949E-4	1.5100E-8	3.6986E-17	5.5532E-16	0.097105	8.6014E-19	1.5116E-19	1.2398E-4	1.0000
1.5100E-8	1.0000E-6	0.050000	-6.8181E-10	NaN	1.8900E-4	1.5100E-8	2.9259E-17	5.5419E-16	0.096813	8.5794E-19	1.5073E-19	1.2361E-4	1.0000
1.5100E-8	1.0000E-6	0.055000	-6.8014E-10	NaN	1.8859E-4	1.5100E-8	2.3339E-17	5.5325E-16	0.096575	8.5612E-19	1.5037E-19	1.2330E-4	1.0000
1.5100E-8	1.0000E-	0.06	-6.78	NaN	1.8826E-	1.5100E-	1.9147E-	5.5247E-	0.09	8.5466E-	1.5008E-	1.2306E-	1.00

k0Sub (m/s)	d (m)	Time (s)	-(tds2.ntflux_Oxi)*n*F (A), UME	Surface ConcOxi (M), OxiMem	Surface ConcOxi (M), OxiNuc	Substrate Kinetics (m/s), k0Sub probe	Normal total flux (mol/s), UME OCR membrane	Normal total flux (mol/s), UME OCR nucleus	Concentration (mol/m^3), Point: (5.5109E-16, 2.5)	k0Sub*Oxi (mol/s), Mem Flux	k0Sub*Oxi (mol/s), Nuc Flux	Normal total flux (mol/(m^2*s)), Point: (5.5109E-16, 2.5)	WEIectrode radius (µm), UME Radius
8	6	0000	82E-10		4	8	17	16	6387	19	19	4	00
1.5100E-8	1.0000E-6	0.065000	-6.7767E-10	NaN	1.8798E-4	1.5100E-8	1.5675E-17	5.5177E-16	0.096223	8.5337E-19	1.4984E-19	1.2285E-4	1.0000
1.5100E-8	1.0000E-6	0.070000	-6.7678E-10	NaN	1.8775E-4	1.5100E-8	1.3456E-17	5.5120E-16	0.096096	8.5236E-19	1.4964E-19	1.2269E-4	1.0000
1.5100E-8	1.0000E-6	0.075000	-6.7602E-10	NaN	1.8755E-4	1.5100E-8	1.1741E-17	5.5069E-16	0.095988	8.5147E-19	1.4948E-19	1.2255E-4	1.0000
1.5100E-8	1.0000E-6	0.080000	-6.7525E-10	NaN	1.8736E-4	1.5100E-8	9.8538E-18	5.5020E-16	0.095879	8.5060E-19	1.4931E-19	1.2241E-4	1.0000
1.5100E-8	1.0000E-6	0.085000	-6.7457E-10	NaN	1.8718E-4	1.5100E-8	8.2993E-18	5.4975E-16	0.095782	8.4982E-19	1.4917E-19	1.2229E-4	1.0000
1.5100E-8	1.0000E-6	0.090000	-6.7399E-10	NaN	1.8703E-4	1.5100E-8	7.0777E-18	5.4936E-16	0.095699	8.4914E-19	1.4904E-19	1.2218E-4	1.0000
1.5100E-8	1.0000E-6	0.095000	-6.7350E-10	NaN	1.8690E-4	1.5100E-8	6.1889E-18	5.4901E-16	0.095629	8.4856E-19	1.4893E-19	1.2209E-4	1.0000
1.5100E-8	1.0000E-6	0.10000	-6.7310E-10	NaN	1.8680E-4	1.5100E-8	5.6591E-18	5.4873E-16	0.095573	8.4809E-19	1.4885E-19	1.2202E-4	1.0000
1.5100E-8	1.0000E-6	0.10500	-6.7290E-10	NaN	1.8674E-4	1.5100E-8	5.5051E-18	5.4857E-16	0.095544	8.4784E-19	1.4880E-19	1.2198E-4	1.0000

k0Sub (m/s)	d (m)	Time (s)	-(tds2.ntflux_Oxi)*n*F (A), UME	Surface ConcOxi (M), OxiMem	Surface ConcOxi (M), OxiNuc	Substrate Kinetics (m/s), k0Sub probe	Normal total flux (mol/s), UME OCR membrane	Normal total flux (mol/s), UME OCR nucleus	Concentration (mol/m^3), Point: (5.5109E-16, 2.5)	k0Sub*Oxi (mol/s), Mem Flux	k0Sub*Oxi (mol/s), Nuc Flux	Normal total flux (mol/(m^2*s)), Point: (5.5109E-16, 2.5)	WEIectrode radius (µm), UME Radius
1.5100E-8	1.0000E-6	0.11000	-6.7269E-10	NaN	1.8669E-4	1.5100E-8	5.3512E-18	5.4841E-16	0.095514	8.4758E-19	1.4875E-19	1.2195E-4	1.0000
1.5100E-8	1.0000E-6	0.11500	-6.7248E-10	NaN	1.8663E-4	1.5100E-8	5.1972E-18	5.4825E-16	0.095485	8.4733E-19	1.4871E-19	1.2191E-4	1.0000
1.5100E-8	1.0000E-6	0.12000	-6.7228E-10	NaN	1.8658E-4	1.5100E-8	5.0433E-18	5.4809E-16	0.095456	8.4707E-19	1.4866E-19	1.2187E-4	1.0000
1.5100E-8	1.0000E-6	0.12500	-6.7207E-10	NaN	1.8652E-4	1.5100E-8	4.8893E-18	5.4793E-16	0.095426	8.4682E-19	1.4862E-19	1.2183E-4	1.0000
1.5100E-8	1.0000E-6	0.13000	-6.7186E-10	NaN	1.8646E-4	1.5100E-8	4.7354E-18	5.4777E-16	0.095397	8.4656E-19	1.4857E-19	1.2179E-4	1.0000
1.5100E-8	1.0000E-6	0.13500	-6.7166E-10	NaN	1.8641E-4	1.5100E-8	4.5815E-18	5.4761E-16	0.095367	8.4631E-19	1.4853E-19	1.2176E-4	1.0000
1.5100E-8	1.0000E-6	0.14000	-6.7145E-10	NaN	1.8635E-4	1.5100E-8	4.4275E-18	5.4745E-16	0.095338	8.4606E-19	1.4848E-19	1.2172E-4	1.0000
1.5100E-8	1.0000E-6	0.14500	-6.7124E-10	NaN	1.8629E-4	1.5100E-8	4.2736E-18	5.4729E-16	0.095309	8.4580E-19	1.4844E-19	1.2168E-4	1.0000
1.5100E-8	1.0000E-6	0.15000	-6.7104E-10	NaN	1.8624E-4	1.5100E-8	4.1196E-18	5.4713E-16	0.095279	8.4555E-19	1.4839E-19	1.2164E-4	1.0000
1.5100E-8	1.0000E-6	0.15500	-6.7084E-	NaN	1.8619E-4	1.5100E-8	3.9789E-18	5.4698E-16	0.095252	8.4531E-19	1.4835E-19	1.2161E-4	1.0000

k0Sub (m/s)	d (m)	Time (s)	-(tds2.ntflux_Oxi)*n*F (A), UME	Surface ConcOxi (M), Oxi Mem	Surface ConcOxi (M), OxiNuc	Substrate Kinetics (m/s), k0Sub probe	Normal total flux (mol/s), UME OCR membrane	Normal total flux (mol/s), UME OCR nucleus	Concentration (mol/m^3), Point: (5.5109E-16, 2.5)	k0Sub*Oxi (mol/s), Mem Flux	k0Sub*Oxi (mol/s), Nuc Flux	Normal total flux (mol/(m^2*s)), Point: (5.5109E-16, 2.5)	WElectrode radius (μm), UME Radius
			10										
1.5100E-8	1.0000E-6	0.16000	-6.7070E-10	NaN	1.8615E-4	1.5100E-8	3.8675E-18	5.4687E-16	0.095232	8.4514E-19	1.4832E-19	1.2158E-4	1.0000
1.5100E-8	1.0000E-6	0.16500	-6.7056E-10	NaN	1.8611E-4	1.5100E-8	3.7562E-18	5.4676E-16	0.095212	8.4496E-19	1.4829E-19	1.2156E-4	1.0000
1.5100E-8	1.0000E-6	0.17000	-6.7042E-10	NaN	1.8607E-4	1.5100E-8	3.6448E-18	5.4665E-16	0.095192	8.4479E-19	1.4825E-19	1.2153E-4	1.0000
1.5100E-8	1.0000E-6	0.17500	-6.7028E-10	NaN	1.8603E-4	1.5100E-8	3.5335E-18	5.4655E-16	0.095172	8.4462E-19	1.4822E-19	1.2151E-4	1.0000
1.5100E-8	1.0000E-6	0.18000	-6.7014E-10	NaN	1.8600E-4	1.5100E-8	3.4221E-18	5.4644E-16	0.095152	8.4444E-19	1.4819E-19	1.2148E-4	1.0000
1.5100E-8	1.0000E-6	0.18500	-6.7000E-10	NaN	1.8596E-4	1.5100E-8	3.3108E-18	5.4633E-16	0.095132	8.4427E-19	1.4816E-19	1.2146E-4	1.0000
1.5100E-8	1.0000E-6	0.19000	-6.6986E-10	NaN	1.8592E-4	1.5100E-8	3.1994E-18	5.4622E-16	0.095112	8.4410E-19	1.4813E-19	1.2143E-4	1.0000
1.5100E-8	1.0000E-6	0.19500	-6.6971E-10	NaN	1.8588E-4	1.5100E-8	3.0880E-18	5.4611E-16	0.095091	8.4393E-19	1.4810E-19	1.2140E-4	1.0000
1.5100E-8	1.0000E-6	0.20000	-6.6957E-10	NaN	1.8584E-4	1.5100E-8	2.9767E-18	5.4601E-16	0.095071	8.4375E-19	1.4807E-19	1.2138E-4	1.0000
1.5100E-	1.0000E-	0.20	-6.69	NaN	1.8580E-	1.5100E-	2.8653E-	5.4590E-	0.09	8.4358E-	1.4804E-	1.2135E-	1.00

k0Sub (m/s)	d (m)	Time (s)	-(tds2.ntflux_Oxi)*n*F (A), UME	Surface Conc cOxi (M), Oxi Mem	Surface Conc cOxi (M), OxiNuc	Substrate Kinetics (m/s), k0Sub probe	Normal total flux (mol/s), UME OCR membrane	Normal total flux (mol/s), UME OCR nucleus	Concentration (mol/m^3), Point: (5.5109E-16, 2.5)	k0Sub*Oxi (mol/s), Mem Flux	k0Sub*Oxi (mol/s), Nuc Flux	Normal total flux (mol/(m^2*s)), Point: (5.5109E-16, 2.5)	WElectrode radius (µm), UME Radius
8	6	500	43E-10		4	8	18	16	5051	19	19	4	00
1.5100E-8	1.0000E-6	0.21000	-6.6931E-10	NaN	1.8577E-4	1.5100E-8	2.7753E-18	5.4580E-16	0.095034	8.4343E-19	1.4801E-19	1.2133E-4	1.0000
1.5100E-8	1.0000E-6	0.21500	-6.6923E-10	NaN	1.8575E-4	1.5100E-8	2.7158E-18	5.4574E-16	0.095022	8.4333E-19	1.4799E-19	1.2132E-4	1.0000
1.5100E-8	1.0000E-6	0.22000	-6.6914E-10	NaN	1.8572E-4	1.5100E-8	2.6563E-18	5.4567E-16	0.095010	8.4322E-19	1.4797E-19	1.2130E-4	1.0000
1.5100E-8	1.0000E-6	0.22500	-6.6906E-10	NaN	1.8570E-4	1.5100E-8	2.5969E-18	5.4561E-16	0.094998	8.4312E-19	1.4795E-19	1.2129E-4	1.0000
1.5100E-8	1.0000E-6	0.23000	-6.6897E-10	NaN	1.8568E-4	1.5100E-8	2.5374E-18	5.4554E-16	0.094986	8.4301E-19	1.4794E-19	1.2127E-4	1.0000
1.5100E-8	1.0000E-6	0.23500	-6.6889E-10	NaN	1.8566E-4	1.5100E-8	2.4779E-18	5.4548E-16	0.094974	8.4291E-19	1.4792E-19	1.2125E-4	1.0000
1.5100E-8	1.0000E-6	0.24000	-6.6880E-10	NaN	1.8563E-4	1.5100E-8	2.4184E-18	5.4541E-16	0.094962	8.4280E-19	1.4790E-19	1.2124E-4	1.0000
1.5100E-8	1.0000E-6	0.24500	-6.6872E-10	NaN	1.8561E-4	1.5100E-8	2.3589E-18	5.4535E-16	0.094949	8.4270E-19	1.4788E-19	1.2122E-4	1.0000
1.5100E-8	1.0000E-6	0.25000	-6.6863E-10	NaN	1.8559E-4	1.5100E-8	2.2995E-18	5.4528E-16	0.094937	8.4260E-19	1.4786E-19	1.2121E-4	1.0000

k0Sub (m/s)	d (m)	Time (s)	-(tds2.ntflux_Oxi)*n*F (A), UME	Surface ConcOxi (M), Oxi Mem	Surface ConcOxi (M), OxiNuc	Substrate Kinetics (m/s), k0Sub probe	Normal total flux (mol/s), UME OCR membrane	Normal total flux (mol/s), UME OCR nucleus	Concentration (mol/m^3), Point: (5.5109E-16, 2.5)	k0Sub*Oxi (mol/s), Mem Flux	k0Sub*Oxi (mol/s), Nuc Flux	Normal total flux (mol/(m^2*s)), Point: (5.5109E-16, 2.5)	WEIectrode radius (μm), UME Radius
1.5100E-8	1.0000E-6	0.25000	-6.6863E-10	NaN	1.8559E-4	1.5100E-8	2.2995E-18	5.4528E-16	0.094937	8.4260E-19	1.4786E-19	1.2121E-4	1.0000
1.5100E-8	1.0000E-6	0.27000	-6.6829E-10	NaN	1.8549E-4	1.5100E-8	2.0616E-18	5.4502E-16	0.094889	8.4218E-19	1.4779E-19	1.2115E-4	1.0000
1.5100E-8	1.0000E-6	0.29000	-6.6795E-10	NaN	1.8540E-4	1.5100E-8	1.8236E-18	5.4476E-16	0.094841	8.4176E-19	1.4771E-19	1.2108E-4	1.0000
1.5100E-8	1.0000E-6	0.31000	-6.6763E-10	NaN	1.8531E-4	1.5100E-8	1.6031E-18	5.4451E-16	0.094795	8.4136E-19	1.4764E-19	1.2103E-4	1.0000
1.5100E-8	1.0000E-6	0.33000	-6.6741E-10	NaN	1.8525E-4	1.5100E-8	1.4791E-18	5.4433E-16	0.094763	8.4109E-19	1.4759E-19	1.2099E-4	1.0000
1.5100E-8	1.0000E-6	0.35000	-6.6718E-10	NaN	1.8519E-4	1.5100E-8	1.3552E-18	5.4416E-16	0.094732	8.4082E-19	1.4754E-19	1.2095E-4	1.0000
1.5100E-8	1.0000E-6	0.37000	-6.6696E-10	NaN	1.8513E-4	1.5100E-8	1.2313E-18	5.4399E-16	0.094701	8.4054E-19	1.4749E-19	1.2091E-4	1.0000
1.5100E-8	1.0000E-6	0.39000	-6.6674E-10	NaN	1.8507E-4	1.5100E-8	1.1074E-18	5.4382E-16	0.094669	8.4027E-19	1.4745E-19	1.2087E-4	1.0000
1.5100E-8	1.0000E-6	0.41000	-6.6653E-10	NaN	1.8502E-4	1.5100E-8	9.9470E-19	5.4365E-16	0.094640	8.4001E-19	1.4740E-19	1.2083E-4	1.0000
1.5100E-8	1.0000E-6	0.43000	-6.6638E-	NaN	1.8497E-4	1.5100E-8	9.2567E-19	5.4353E-16	0.094618	8.3982E-19	1.4737E-19	1.2080E-4	1.0000

k0Sub (m/s)	d (m)	Time (s)	-(tds2.ntflux_Oxi)*n*F (A), UME	Surface ConcOxi (M), Oxi Mem	Surface ConcOxi (M), OxiNuc	Substrate Kinetics (m/s), k0Sub probe	Normal total flux (mol/s), UME OCR membrane	Normal total flux (mol/s), UME OCR nucleus	Concentration (mol/m^3), Point: (5.5109E-16, 2.5)	k0Sub*Oxi (mol/s), Mem Flux	k0Sub*Oxi (mol/s), Nuc Flux	Normal total flux (mol/(m^2*s)), Point: (5.5109E-16, 2.5)	WElectrode radius (µm), UME Radius
			10										
1.5100E-8	1.0000E-6	0.45000	-6.6622E-10	NaN	1.8493E-4	1.5100E-8	8.5665E-19	5.4341E-16	0.094596	8.3963E-19	1.4733E-19	1.2077E-4	1.0000
1.5100E-8	1.0000E-6	0.47000	-6.6607E-10	NaN	1.8489E-4	1.5100E-8	7.8763E-19	5.4329E-16	0.094574	8.3944E-19	1.4730E-19	1.2074E-4	1.0000
1.5100E-8	1.0000E-6	0.49000	-6.6592E-10	NaN	1.8485E-4	1.5100E-8	7.1861E-19	5.4317E-16	0.094552	8.3925E-19	1.4726E-19	1.2072E-4	1.0000
1.5100E-8	1.0000E-6	0.51000	-6.6577E-10	NaN	1.8481E-4	1.5100E-8	6.5667E-19	5.4305E-16	0.094531	8.3906E-19	1.4723E-19	1.2069E-4	1.0000
1.5100E-8	1.0000E-6	0.53000	-6.6565E-10	NaN	1.8478E-4	1.5100E-8	6.1492E-19	5.4296E-16	0.094515	8.3892E-19	1.4721E-19	1.2067E-4	1.0000
1.5100E-8	1.0000E-6	0.55000	-6.6554E-10	NaN	1.8474E-4	1.5100E-8	5.7317E-19	5.4287E-16	0.094498	8.3878E-19	1.4718E-19	1.2065E-4	1.0000
1.5100E-8	1.0000E-6	0.57000	-6.6542E-10	NaN	1.8471E-4	1.5100E-8	5.3142E-19	5.4278E-16	0.094482	8.3864E-19	1.4716E-19	1.2063E-4	1.0000
1.5100E-8	1.0000E-6	0.59000	-6.6531E-10	NaN	1.8468E-4	1.5100E-8	4.8966E-19	5.4269E-16	0.094466	8.3849E-19	1.4713E-19	1.2061E-4	1.0000
1.5100E-8	1.0000E-6	0.61000	-6.6520E-10	NaN	1.8465E-4	1.5100E-8	4.5211E-19	5.4260E-16	0.094450	8.3836E-19	1.4711E-19	1.2059E-4	1.0000
1.5100E-	1.0000E-	0.63	-6.65	NaN	1.8463E-	1.5100E-	4.2367E-	5.4253E-	0.09	8.3824E-	1.4709E-	1.2057E-	1.00

k0Sub (m/s)	d (m)	Time (s)	-(tds2.ntflux_Oxi)*n*F (A), UME	Surface Conc cOxi (M), Oxi Mem	Surface Conc cOxi (M), OxiNuc	Substrate Kinetics (m/s), k0Sub probe	Normal total flux (mol/s), UME OCR membrane	Normal total flux (mol/s), UME OCR nucleus	Concentration (mol/m^3), Point: (5.5109E-16, 2.5)	k0Sub*Oxi (mol/s), Mem Flux	k0Sub*Oxi (mol/s), Nuc Flux	Normal total flux (mol/(m^2*s)), Point: (5.5109E-16, 2.5)	WElectrode radius (µm), UME Radius
8	6	000	11E-10		4	8	19	16	4437	19	19	4	00
1.5100E-8	1.0000E-6	0.65000	-6.6502E-10	NaN	1.8460E-4	1.5100E-8	3.9523E-19	5.4245E-16	0.094424	8.3813E-19	1.4707E-19	1.2055E-4	1.0000
1.5100E-8	1.0000E-6	0.67000	-6.6493E-10	NaN	1.8458E-4	1.5100E-8	3.6679E-19	5.4238E-16	0.094411	8.3801E-19	1.4705E-19	1.2054E-4	1.0000
1.5100E-8	1.0000E-6	0.69000	-6.6483E-10	NaN	1.8455E-4	1.5100E-8	3.3835E-19	5.4231E-16	0.094398	8.3790E-19	1.4703E-19	1.2052E-4	1.0000
1.5100E-8	1.0000E-6	0.71000	-6.6475E-10	NaN	1.8453E-4	1.5100E-8	3.1339E-19	5.4224E-16	0.094386	8.3779E-19	1.4701E-19	1.2050E-4	1.0000
1.5100E-8	1.0000E-6	0.73000	-6.6468E-10	NaN	1.8451E-4	1.5100E-8	2.9437E-19	5.4218E-16	0.094376	8.3770E-19	1.4699E-19	1.2049E-4	1.0000
1.5100E-8	1.0000E-6	0.75000	-6.6460E-10	NaN	1.8449E-4	1.5100E-8	2.7534E-19	5.4212E-16	0.094365	8.3761E-19	1.4697E-19	1.2048E-4	1.0000
1.5100E-8	1.0000E-6	0.77000	-6.6453E-10	NaN	1.8447E-4	1.5100E-8	2.5632E-19	5.4207E-16	0.094355	8.3752E-19	1.4696E-19	1.2046E-4	1.0000
1.5100E-8	1.0000E-6	0.79000	-6.6446E-10	NaN	1.8445E-4	1.5100E-8	2.3729E-19	5.4201E-16	0.094345	8.3743E-19	1.4694E-19	1.2045E-4	1.0000
1.5100E-8	1.0000E-6	0.81000	-6.6439E-10	NaN	1.8443E-4	1.5100E-8	2.1948E-19	5.4195E-16	0.094335	8.3734E-19	1.4693E-19	1.2044E-4	1.0000

k0Sub (m/s)	d (m)	Time (s)	-(tds2.ntflux_Oxi)*n*F (A), UME	Surface Con cOxi (M), Oxi Mem	Surface Con cOxi (M), OxiNuc	Substrate Kinetics (m/s), k0Sub probe	Normal total flux (mol/s), UME OCR membrane	Normal total flux (mol/s), UME OCR nucleus	Concentration (mol/m^3), Point: (5.5109E-16, 2.5)	k0Sub* Oxi (mol/s), Mem Flux	k0Sub* Oxi (mol/s), Nuc Flux	Normal total flux (mol/(m^2*s)), Point: (5.5109E-16, 2.5)	WElectrode radius (µm), UME Radius
1.5100E-8	1.0000E-6	0.83000	-6.6432E-10	NaN	1.8441E-4	1.5100E-8	2.0409E-19	5.4190E-16	0.094325	8.3726E-19	1.4691E-19	1.2043E-4	1.0000
1.5100E-8	1.0000E-6	0.85000	-6.6426E-10	NaN	1.8439E-4	1.5100E-8	1.8976E-19	5.4185E-16	0.094316	8.3718E-19	1.4690E-19	1.2042E-4	1.0000
1.5100E-8	1.0000E-6	0.87000	-6.6420E-10	NaN	1.8437E-4	1.5100E-8	1.7650E-19	5.4180E-16	0.094308	8.3711E-19	1.4688E-19	1.2040E-4	1.0000
1.5100E-8	1.0000E-6	0.89000	-6.6414E-10	NaN	1.8436E-4	1.5100E-8	1.6430E-19	5.4175E-16	0.094300	8.3703E-19	1.4687E-19	1.2039E-4	1.0000
1.5100E-8	1.0000E-6	0.91000	-6.6408E-10	NaN	1.8434E-4	1.5100E-8	1.5274E-19	5.4171E-16	0.094292	8.3696E-19	1.4686E-19	1.2038E-4	1.0000
1.5100E-8	1.0000E-6	0.93000	-6.6403E-10	NaN	1.8433E-4	1.5100E-8	1.4147E-19	5.4166E-16	0.094284	8.3689E-19	1.4685E-19	1.2037E-4	1.0000
1.5100E-8	1.0000E-6	0.95000	-6.6398E-10	NaN	1.8431E-4	1.5100E-8	1.3091E-19	5.4162E-16	0.094276	8.3683E-19	1.4684E-19	1.2036E-4	1.0000
1.5100E-8	1.0000E-6	0.97000	-6.6392E-10	NaN	1.8430E-4	1.5100E-8	1.2106E-19	5.4158E-16	0.094269	8.3676E-19	1.4682E-19	1.2035E-4	1.0000
1.5100E-8	1.0000E-6	0.99000	-6.6388E-10	NaN	1.8429E-4	1.5100E-8	1.1193E-19	5.4154E-16	0.094262	8.3670E-19	1.4681E-19	1.2035E-4	1.0000
1.5100E-8	1.5000E-6	0.0000	-7.1532E-	NaN	2.2700E-4	1.5100E-8	-2.0267E-	-1.2323E-	0.22699	1.0088E-18	2.0267E-19	-1.6335E-	1.0000

k0Sub (m/s)	d (m)	Time (s)	-(tds2.ntflux_Oxi)*n*F (A), UME	Surface ConcOxi (M), OxiMem	Surface ConcOxi (M), OxiNuc	Substrate Kinetics (m/s), k0Sub probe	Normal total flux (mol/s), UME OCR membrane	Normal total flux (mol/s), UME OCR nucleus	Concentration (mol/m^3), Point: (5.5109E-16, 2.5)	k0Sub*Oxi (mol/s), Mem Flux	k0Sub*Oxi (mol/s), Nuc Flux	Normal total flux (mol/(m^2*s)), Point: (5.5109E-16, 2.5)	WElectrode radius (μm), UME Radius
			9				19	19				9	
1.5100E-8	1.5000E-6	0.005000 0	-8.1794E-10	NaN	2.1330E-4	1.5100E-8	7.3009E-16	4.5091E-16	0.14619	9.5956E-19	1.7883E-19	8.6777E-5	1.0000
1.5100E-8	1.5000E-6	0.010000	-7.7419E-10	NaN	2.0631E-4	1.5100E-8	4.0741E-16	4.6750E-16	0.13819	9.3063E-19	1.7046E-19	8.1986E-5	1.0000
1.5100E-8	1.5000E-6	0.015000	-7.5226E-10	NaN	2.0205E-4	1.5100E-8	2.5485E-16	4.6711E-16	0.13423	9.1234E-19	1.6599E-19	7.9621E-5	1.0000
1.5100E-8	1.5000E-6	0.020000	-7.3904E-10	NaN	1.9926E-4	1.5100E-8	1.7331E-16	4.6480E-16	0.13185	9.0023E-19	1.6324E-19	7.8203E-5	1.0000
1.5100E-8	1.5000E-6	0.025000	-7.2993E-10	NaN	1.9729E-4	1.5100E-8	1.2240E-16	4.6288E-16	0.13021	8.9163E-19	1.6133E-19	7.7225E-5	1.0000
1.5100E-8	1.5000E-6	0.030000	-7.2330E-10	NaN	1.9584E-4	1.5100E-8	8.8133E-17	4.6135E-16	0.12902	8.8528E-19	1.5994E-19	7.6516E-5	1.0000
1.5100E-8	1.5000E-6	0.035000	-7.1856E-10	NaN	1.9478E-4	1.5100E-8	6.5925E-17	4.6004E-16	0.12816	8.8059E-19	1.5894E-19	7.6008E-5	1.0000
1.5100E-8	1.5000E-6	0.040000	-7.1468E-10	NaN	1.9390E-4	1.5100E-8	4.8519E-17	4.5890E-16	0.12746	8.7671E-19	1.5812E-19	7.5593E-5	1.0000
1.5100E-8	1.5000E-6	0.045000	-7.1165E-10	NaN	1.9320E-4	1.5100E-8	3.5935E-17	4.5791E-16	0.12692	8.7363E-19	1.5747E-19	7.5270E-5	1.0000
1.5100E-	1.5000E-	0.05	-7.09	NaN	1.9267E-	1.5100E-	2.7844E-	4.5708E-	0.12	8.7130E-	1.5700E-	7.5033E-	1.00

k0Sub (m/s)	d (m)	Time (s)	-(tds2.ntflux_Oxi)*n*F (A), UME	Surface ConcOxi (M), Oxi Mem	Surface ConcOxi (M), OxiNuc	Substrate Kinetics (m/s), k0Sub probe	Normal total flux (mol/s), UME OCR membrane	Normal total flux (mol/s), UME OCR nucleus	Concentration (mol/m^3), Point: (5.5109E-16, 2.5)	k0Sub* Oxi (mol/s), Mem Flux	k0Sub* Oxi (mol/s), Nuc Flux	Normal total flux (mol/(m^2*s)), Point: (5.5109E-16, 2.5)	WElectrode radius (µm), UME Radius
8	6	0000	43E-10		4	8	17	16	652	19	19	5	00
1.5100E-8	1.5000E-6	0.055000	-7.0752E-10	NaN	1.9222E-4	1.5100E-8	2.1138E-17	4.5634E-16	0.12618	8.6928E-19	1.5659E-19	7.4829E-5	1.0000
1.5100E-8	1.5000E-6	0.060000	-7.0616E-10	NaN	1.9188E-4	1.5100E-8	1.7452E-17	4.5572E-16	0.12594	8.6779E-19	1.5630E-19	7.4685E-5	1.0000
1.5100E-8	1.5000E-6	0.065000	-7.0511E-10	NaN	1.9162E-4	1.5100E-8	1.5158E-17	4.5519E-16	0.12575	8.6660E-19	1.5607E-19	7.4573E-5	1.0000
1.5100E-8	1.5000E-6	0.070000	-7.0413E-10	NaN	1.9137E-4	1.5100E-8	1.2949E-17	4.5471E-16	0.12557	8.6550E-19	1.5585E-19	7.4469E-5	1.0000
1.5100E-8	1.5000E-6	0.075000	-7.0341E-10	NaN	1.9119E-4	1.5100E-8	1.1838E-17	4.5431E-16	0.12544	8.6466E-19	1.5570E-19	7.4392E-5	1.0000
1.5100E-8	1.5000E-6	0.080000	-7.0268E-10	NaN	1.9100E-4	1.5100E-8	1.0514E-17	4.5393E-16	0.12531	8.6382E-19	1.5554E-19	7.4315E-5	1.0000
1.5100E-8	1.5000E-6	0.085000	-7.0198E-10	NaN	1.9082E-4	1.5100E-8	9.1909E-18	4.5357E-16	0.12519	8.6303E-19	1.5539E-19	7.4241E-5	1.0000
1.5100E-8	1.5000E-6	0.090000	-7.0136E-10	NaN	1.9066E-4	1.5100E-8	8.0665E-18	4.5324E-16	0.12508	8.6232E-19	1.5525E-19	7.4175E-5	1.0000
1.5100E-8	1.5000E-6	0.095000	-7.0082E-10	NaN	1.9052E-4	1.5100E-8	7.1406E-18	4.5295E-16	0.12498	8.6169E-19	1.5513E-19	7.4117E-5	1.0000

k0Sub (m/s)	d (m)	Time (s)	-(tds2.ntflux_Oxi)*n*F (A), UME	Surface ConcOxi (M), Oxi Mem	Surface ConcOxi (M), OxiNuc	Substrate Kinetics (m/s), k0Sub probe	Normal total flux (mol/s), UME OCR membrane	Normal total flux (mol/s), UME OCR nucleus	Concentration (mol/m^3), Point: (5.5109E-16, 2.5)	k0Sub*Oxi (mol/s), Mem Flux	k0Sub*Oxi (mol/s), Nuc Flux	Normal total flux (mol/(m^2*s)), Point: (5.5109E-16, 2.5)	WEIectrode radius (µm), UME Radius
1.5100E-8	1.5000E-6	0.10000	-7.0035E-10	NaN	1.9040E-4	1.5100E-8	6.4132E-18	4.5269E-16	0.12490	8.6114E-19	1.5503E-19	7.4067E-5	1.0000
1.5100E-8	1.5000E-6	0.10500	-7.0001E-10	NaN	1.9031E-4	1.5100E-8	6.0159E-18	4.5250E-16	0.12484	8.6075E-19	1.5496E-19	7.4032E-5	1.0000
1.5100E-8	1.5000E-6	0.11000	-6.9979E-10	NaN	1.9026E-4	1.5100E-8	5.8433E-18	4.5237E-16	0.12480	8.6049E-19	1.5491E-19	7.4009E-5	1.0000
1.5100E-8	1.5000E-6	0.11500	-6.9957E-10	NaN	1.9020E-4	1.5100E-8	5.6707E-18	4.5224E-16	0.12476	8.6022E-19	1.5486E-19	7.3985E-5	1.0000
1.5100E-8	1.5000E-6	0.12000	-6.9935E-10	NaN	1.9014E-4	1.5100E-8	5.4981E-18	4.5211E-16	0.12472	8.5996E-19	1.5481E-19	7.3962E-5	1.0000
1.5100E-8	1.5000E-6	0.12500	-6.9913E-10	NaN	1.9008E-4	1.5100E-8	5.3254E-18	4.5197E-16	0.12468	8.5969E-19	1.5476E-19	7.3938E-5	1.0000
1.5100E-8	1.5000E-6	0.13000	-6.9890E-10	NaN	1.9002E-4	1.5100E-8	5.1528E-18	4.5184E-16	0.12464	8.5942E-19	1.5471E-19	7.3915E-5	1.0000
1.5100E-8	1.5000E-6	0.13500	-6.9868E-10	NaN	1.8996E-4	1.5100E-8	4.9802E-18	4.5171E-16	0.12460	8.5916E-19	1.5466E-19	7.3891E-5	1.0000
1.5100E-8	1.5000E-6	0.14000	-6.9846E-10	NaN	1.8990E-4	1.5100E-8	4.8076E-18	4.5158E-16	0.12456	8.5889E-19	1.5461E-19	7.3868E-5	1.0000
1.5100E-8	1.5000E-6	0.14500	-6.9824E-	NaN	1.8984E-4	1.5100E-8	4.6350E-18	4.5144E-16	0.12452	8.5863E-19	1.5457E-19	7.3844E-5	1.0000

k0Sub (m/s)	d (m)	Time (s)	-(tds2.ntflux_Oxi)*n*F (A), UME	Surface ConcOxi (M), OxiMem	Surface ConcOxi (M), OxiNuc	Substrate Kinetics (m/s), k0Sub probe	Normal total flux (mol/s), UME OCR membrane	Normal total flux (mol/s), UME OCR nucleus	Concentration (mol/m^3), Point: (5.5109E-16, 2.5)	k0Sub*Oxi (mol/s), Mem Flux	k0Sub*Oxi (mol/s), Nuc Flux	Normal total flux (mol/(m^2*s)), Point: (5.5109E-16, 2.5)	WElectrode radius (µm), UME Radius
			10										
1.5100E-8	1.5000E-6	0.15000	-6.9802E-10	NaN	1.8979E-4	1.5100E-8	4.4624E-18	4.5131E-16	0.12448	8.5836E-19	1.5452E-19	7.3821E-5	1.0000
1.5100E-8	1.5000E-6	0.15500	-6.9780E-10	NaN	1.8973E-4	1.5100E-8	4.2898E-18	4.5118E-16	0.12444	8.5810E-19	1.5447E-19	7.3797E-5	1.0000
1.5100E-8	1.5000E-6	0.16000	-6.9762E-10	NaN	1.8968E-4	1.5100E-8	4.1523E-18	4.5107E-16	0.12441	8.5789E-19	1.5443E-19	7.3778E-5	1.0000
1.5100E-8	1.5000E-6	0.16500	-6.9747E-10	NaN	1.8964E-4	1.5100E-8	4.0322E-18	4.5098E-16	0.12438	8.5771E-19	1.5439E-19	7.3762E-5	1.0000
1.5100E-8	1.5000E-6	0.17000	-6.9732E-10	NaN	1.8960E-4	1.5100E-8	3.9121E-18	4.5089E-16	0.12436	8.5753E-19	1.5436E-19	7.3746E-5	1.0000
1.5100E-8	1.5000E-6	0.17500	-6.9716E-10	NaN	1.8956E-4	1.5100E-8	3.7920E-18	4.5080E-16	0.12433	8.5734E-19	1.5433E-19	7.3730E-5	1.0000
1.5100E-8	1.5000E-6	0.18000	-6.9701E-10	NaN	1.8952E-4	1.5100E-8	3.6719E-18	4.5071E-16	0.12430	8.5716E-19	1.5429E-19	7.3714E-5	1.0000
1.5100E-8	1.5000E-6	0.18500	-6.9686E-10	NaN	1.8948E-4	1.5100E-8	3.5518E-18	4.5062E-16	0.12428	8.5698E-19	1.5426E-19	7.3698E-5	1.0000
1.5100E-8	1.5000E-6	0.19000	-6.9671E-10	NaN	1.8944E-4	1.5100E-8	3.4317E-18	4.5053E-16	0.12425	8.5680E-19	1.5423E-19	7.3682E-5	1.0000
1.5100E-	1.5000E-	0.19	-6.96	NaN	1.8940E-	1.5100E-	3.3117E-	4.5044E-	0.12	8.5662E-	1.5420E-	7.3666E-	1.00

k0Sub (m/s)	d (m)	Time (s)	-(tds2.ntflux_Oxi)*n*F (A), UME	Surface ConcOxi (M), Oxi Mem	Surface ConcOxi (M), OxiNuc	Substrate Kinetics (m/s), k0Sub probe	Normal total flux (mol/s), UME OCR membrane	Normal total flux (mol/s), UME OCR nucleus	Concentration (mol/m^3), Point: (5.5109E-16, 2.5)	k0Sub* Oxi (mol/s), Mem Flux	k0Sub* Oxi (mol/s), Nuc Flux	Normal total flux (mol/(m^2*s)), Point: (5.5109E-16, 2.5)	WEIectrode radius (μm), UME Radius
8	6	500	56E-10		4	8	18	16	422	19	19	5	00
1.5100E-8	1.5000E-6	0.20000	-6.9641E-10	NaN	1.8936E-4	1.5100E-8	3.1916E-18	4.5035E-16	0.12419	8.5644E-19	1.5416E-19	7.3650E-5	1.0000
1.5100E-8	1.5000E-6	0.20500	-6.9625E-10	NaN	1.8932E-4	1.5100E-8	3.0715E-18	4.5026E-16	0.12417	8.5626E-19	1.5413E-19	7.3634E-5	1.0000
1.5100E-8	1.5000E-6	0.21000	-6.9610E-10	NaN	1.8928E-4	1.5100E-8	2.9514E-18	4.5017E-16	0.12414	8.5608E-19	1.5410E-19	7.3618E-5	1.0000
1.5100E-8	1.5000E-6	0.21500	-6.9600E-10	NaN	1.8925E-4	1.5100E-8	2.8809E-18	4.5011E-16	0.12412	8.5596E-19	1.5407E-19	7.3607E-5	1.0000
1.5100E-8	1.5000E-6	0.22000	-6.9591E-10	NaN	1.8923E-4	1.5100E-8	2.8187E-18	4.5005E-16	0.12411	8.5585E-19	1.5405E-19	7.3598E-5	1.0000
1.5100E-8	1.5000E-6	0.22500	-6.9582E-10	NaN	1.8920E-4	1.5100E-8	2.7565E-18	4.5000E-16	0.12409	8.5574E-19	1.5403E-19	7.3588E-5	1.0000
1.5100E-8	1.5000E-6	0.23000	-6.9573E-10	NaN	1.8918E-4	1.5100E-8	2.6942E-18	4.4994E-16	0.12407	8.5563E-19	1.5401E-19	7.3578E-5	1.0000
1.5100E-8	1.5000E-6	0.23500	-6.9564E-10	NaN	1.8915E-4	1.5100E-8	2.6320E-18	4.4989E-16	0.12406	8.5552E-19	1.5399E-19	7.3568E-5	1.0000
1.5100E-8	1.5000E-6	0.24000	-6.9554E-10	NaN	1.8913E-4	1.5100E-8	2.5698E-18	4.4983E-16	0.12404	8.5541E-19	1.5397E-19	7.3559E-5	1.0000

k0Sub (m/s)	d (m)	Time (s)	-(tds2.ntflux_Oxi)*n*F (A), UME	Surface ConcOxi (M), Oxi Mem	Surface ConcOxi (M), OxiNuc	Substrate Kinetics (m/s), k0Sub probe	Normal total flux (mol/s), UME OCR membrane	Normal total flux (mol/s), UME OCR nucleus	Concentration (mol/m^3), Point: (5.5109E-16, 2.5)	k0Sub*Oxi (mol/s), Mem Flux	k0Sub*Oxi (mol/s), Nuc Flux	Normal total flux (mol/(m^2*s)), Point: (5.5109E-16, 2.5)	WElectrode radius (μm), UME Radius
1.5100E-8	1.5000E-6	0.24500	-6.9545E-10	NaN	1.8911E-4	1.5100E-8	2.5075E-18	4.4978E-16	0.12402	8.5530E-19	1.5395E-19	7.3549E-5	1.0000
1.5100E-8	1.5000E-6	0.25000	-6.9536E-10	NaN	1.8908E-4	1.5100E-8	2.4453E-18	4.4972E-16	0.12401	8.5519E-19	1.5393E-19	7.3539E-5	1.0000
1.5100E-8	1.5000E-6	0.25000	-6.9536E-10	NaN	1.8908E-4	1.5100E-8	2.4453E-18	4.4972E-16	0.12401	8.5519E-19	1.5393E-19	7.3539E-5	1.0000
1.5100E-8	1.5000E-6	0.27000	-6.9500E-10	NaN	1.8898E-4	1.5100E-8	2.1964E-18	4.4950E-16	0.12394	8.5475E-19	1.5385E-19	7.3501E-5	1.0000
1.5100E-8	1.5000E-6	0.29000	-6.9463E-10	NaN	1.8889E-4	1.5100E-8	1.9474E-18	4.4928E-16	0.12388	8.5431E-19	1.5377E-19	7.3462E-5	1.0000
1.5100E-8	1.5000E-6	0.31000	-6.9427E-10	NaN	1.8879E-4	1.5100E-8	1.7003E-18	4.4906E-16	0.12381	8.5388E-19	1.5369E-19	7.3423E-5	1.0000
1.5100E-8	1.5000E-6	0.33000	-6.9403E-10	NaN	1.8873E-4	1.5100E-8	1.5709E-18	4.4892E-16	0.12377	8.5359E-19	1.5364E-19	7.3398E-5	1.0000
1.5100E-8	1.5000E-6	0.35000	-6.9379E-10	NaN	1.8866E-4	1.5100E-8	1.4416E-18	4.4877E-16	0.12373	8.5330E-19	1.5359E-19	7.3373E-5	1.0000
1.5100E-8	1.5000E-6	0.37000	-6.9355E-10	NaN	1.8860E-4	1.5100E-8	1.3122E-18	4.4863E-16	0.12368	8.5302E-19	1.5353E-19	7.3348E-5	1.0000
1.5100E-8	1.5000E-6	0.39000	-6.9332E-	NaN	1.8854E-4	1.5100E-8	1.1829E-18	4.4848E-16	0.12364	8.5273E-19	1.5348E-19	7.3323E-5	1.0000

k0Sub (m/s)	d (m)	Time (s)	-(tds2.ntflux_Oxi)*n*F (A), UME	Surface ConcOxi (M), OxiMem	Surface ConcOxi (M), OxiNuc	Substrate Kinetics (m/s), k0Sub probe	Normal total flux (mol/s), UME OCR membrane	Normal total flux (mol/s), UME OCR nucleus	Concentration (mol/m^3), Point: (5.5109E-16, 2.5)	k0Sub*Oxi (mol/s), Mem Flux	k0Sub*Oxi (mol/s), Nuc Flux	Normal total flux (mol/(m^2*s)), Point: (5.5109E-16, 2.5)	WElectrode radius (µm), UME Radius
			10										
1.5100E-8	1.5000E-6	0.41000	-6.9308E-10	NaN	1.8847E-4	1.5100E-8	1.0573E-18	4.4834E-16	0.12360	8.5245E-19	1.5343E-19	7.3298E-5	1.0000
1.5100E-8	1.5000E-6	0.43000	-6.9291E-10	NaN	1.8843E-4	1.5100E-8	9.8507E-19	4.4824E-16	0.12357	8.5225E-19	1.5339E-19	7.3280E-5	1.0000
1.5100E-8	1.5000E-6	0.45000	-6.9275E-10	NaN	1.8838E-4	1.5100E-8	9.1279E-19	4.4813E-16	0.12354	8.5204E-19	1.5336E-19	7.3263E-5	1.0000
1.5100E-8	1.5000E-6	0.47000	-6.9258E-10	NaN	1.8834E-4	1.5100E-8	8.4051E-19	4.4803E-16	0.12351	8.5184E-19	1.5332E-19	7.3245E-5	1.0000
1.5100E-8	1.5000E-6	0.49000	-6.9241E-10	NaN	1.8829E-4	1.5100E-8	7.6823E-19	4.4793E-16	0.12348	8.5164E-19	1.5328E-19	7.3227E-5	1.0000
1.5100E-8	1.5000E-6	0.51000	-6.9225E-10	NaN	1.8825E-4	1.5100E-8	6.9934E-19	4.4783E-16	0.12345	8.5144E-19	1.5325E-19	7.3210E-5	1.0000
1.5100E-8	1.5000E-6	0.53000	-6.9213E-10	NaN	1.8822E-4	1.5100E-8	6.5551E-19	4.4775E-16	0.12343	8.5129E-19	1.5322E-19	7.3197E-5	1.0000
1.5100E-8	1.5000E-6	0.55000	-6.9200E-10	NaN	1.8818E-4	1.5100E-8	6.1168E-19	4.4767E-16	0.12341	8.5114E-19	1.5319E-19	7.3184E-5	1.0000
1.5100E-8	1.5000E-6	0.57000	-6.9188E-10	NaN	1.8815E-4	1.5100E-8	5.6785E-19	4.4759E-16	0.12339	8.5099E-19	1.5316E-19	7.3171E-5	1.0000
1.5100E-	1.5000E-	0.59	-6.91	NaN	1.8812E-	1.5100E-	5.2402E-	4.4752E-	0.12	8.5084E-	1.5314E-	7.3158E-	1.00

k0Sub (m/s)	d (m)	Time (s)	-(tds2.ntflux_Oxi)*n*F (A), UME	Surface ConcOxi (M), OxiMem	Surface ConcOxi (M), OxiNuc	Substrate Kinetics (m/s), k0Sub probe	Normal total flux (mol/s), UME OCR membrane	Normal total flux (mol/s), UME OCR nucleus	Concentration (mol/m^3), Point: (5.5109E-16, 2.5)	k0Sub*Oxi (mol/s), Mem Flux	k0Sub*Oxi (mol/s), Nuc Flux	Normal total flux (mol/(m^2*s)), Point: (5.5109E-16, 2.5)	WEIectrode radius (µm), UME Radius
8	6	000	76E-10		4	8	19	16	336	19	19	5	00
[truncated: 353,786 more chars]
